# Alternative splicing of helicase-like transcription factor (Hltf): Intron retention-dependent activation of immune tolerance at the feto-maternal interface

**DOI:** 10.1371/journal.pone.0200211

**Published:** 2018-07-05

**Authors:** Gurvinder Kaur, Rebecca A. Helmer, Lisa A. Smith, Raul Martinez-Zaguilan, Jannette M. Dufour, Beverly S. Chilton

**Affiliations:** 1 Department of Cell Biology & Biochemistry, Texas Tech University Health Sciences Center, Lubbock, Texas, United States of America; 2 Department of Pathology, Texas Tech University Health Sciences Center, Lubbock, Texas, United States of America; 3 Department of Cell Physiology & Molecular Biophysics, Texas Tech University Health Sciences Center, Lubbock, Texas, United States of America; Academic Medical Centre, University of Amsterdam, NETHERLANDS

## Abstract

*Hltf* is regulated by intron retention, and global *Hltf*-deletion causes perinatal lethality from hypoglycemia. In heart, full-length Hltf is a transcriptional regulator of *Hif-1α* that controls transport systems. Thus, we tested the hypothesis that *Hltf* deletion from placenta caused or exacerbated neonatal hypoglycemia via *Hif-1α* regulation of nutrient transporters. RNA-seq data analyses identified significant changes in transcript expression and alternative splicing (AS) in E18.5 placentome. iPathwayGuide was used for gene ontology (GO) analysis of biological processes, molecular functions and cellular components. Elim pruning algorithm identified hierarchical relationships. The methylome was interrogated by Methyl-MiniSeq Epiquest analysis. GO analysis identified gene enrichment within biological processes. Protein expression was visualized with immunohistochemistry. Although two Hltf mRNA isoforms are quantifiable in most murine tissues, only the truncated Hltf isoform is expressed in placenta. The responsible intron retention event occurs in the absence of DNA methylation. iPathwayGuide analysis identified 157 target genes of 11,538 total genes with measured expression. These were obtained using a threshold of 0.05 for statistical significance (p-value) and a long fold change of expression with absolute value of at least 0.6. *Hltf* deletion altered transcription of trophoblast lineage-specific genes, and increased transcription of the *Cxcr7* (p = 0.004) gene whose protein product is a co-receptor for human and simian immunodeficiency viruses. Concomitant increased Cxcr7 protein was identified with immunolabeling. *Hltf* deletion had no effect on transcription or site-specific methylation patterns of *Hif-1α*, the major glucose transporters, or System A amino acid transporters. There was no measureable evidence of uteroplacental dysfunction or fetal compromise. iPathGuide analysis revealed Hltf suppresses cytolysis (10/21 genes; p-value 1.900e-12; p-value correction: Elim pruning; GO:019835) including the perforin-granzyme pathway in uterine natural killer cells. Our findings 1) prove the truncated Hltf protein isoform is a transcription factor, 2) establish a functional link between AS of Hltf and immunosuppression at the feto-maternal interface, 3) correlate intron retention with the absence of DNA methylation, and 4) underscore the importance of differential splicing analysis to identify Hltf’s functional diversity.

## Introduction

Pregnancy and malignancy share molecular mechanisms [1–3] in order to cope with environmental stress, alter the metabolomics of the microenvironment to promote growth and invasion, and achieve immune tolerance. One critical mechanism for the expansion of protein diversity in response to environmental challenges is alternative splicing (AS) [4] especially intron retention [5]. The association of AS with human placenta abnormalities and disorders of pregnancy has been validated in mouse models [6]. AS is also responsible for tumor heterogeneity [7, 8] leading to the functional success of the malignancy including chemo-resistance [9].

A second critical mechanism, which is an adaptive response to genotoxic stress, is ploidy [10]. Trophoblast stem cells of the placenta exit the mitotic cell cycle to become endoreplicating trophoblast giant cells [11]. These polyploid cells lack functional DNA damage response mechanisms [12, 13]. In the human pregnancy complication of preeclampsia, cytotrophoblasts display selective resistance to DNA damage, i.e. DNA damage is localized to the maternal side not the fetal side of the placenta [14]. Polyploidy is also a hallmark of human cancers where endoreplication improves cellular adaptation [12] and drug resistance [13].

A third critical mechanism, which is the adaptive transcriptional response to hypoxic stress − a shared component of placenta and cancer − is mediated by the HIF pathway [15]. All mouse knockout models for HIF subunits (Hif-1α, Hif-2α, or Hif-β) are embryonic lethal with abnormal placenta developmental. HIF activation leads to increased transcription of genes associated with many processes including metabolism. In response to uteroplacental hypoxia, HIF-1 upregulates glucose (GLUT or *SLC2A* gene family) transporters in invasive trophoblast [16]. HIF-1-mediated transcription promotes proliferation in tumor cells through the induction of rate limiting enzymes in the glycolytic pathway and increased expression of glucose transporters [17]. HIF-1 induction promotes angiogenesis and metastasis.

A fourth critical mechanism is the adaptive response to the immune system. Like invading tumor cells [18, 19], fetal trophoblasts encounter host cells including immune cells [20] such as macrophages, dendritic cells, NK cells and T cells. Uterine NK cells and their putative counterpart, a subset of NK cells that infiltrate the tumor microenvironment, have tolerogenic functions [21–23]. NK and uNK cells, are able to inhibit HIV-1 infection [24, 25] and they express proangiogenic activity in their respective microenvironments, i.e. either HIV-associated malignancies or placentae [26].

Alternatively spliced tumor suppressor genes like *p53* encode proteins that promote genomic stability and homeostasis in placenta [27]. Human helicase-like transcription factor (*HLTF*)–a tumor suppressor in colorectal cancer–is regulated by AS [28]. In mice, post-transcriptional RNA processing yields a full-length message isoform and a 3´-truncated message isoform. The structural domain organization of the full-length Hltf proteins in humans [29–31] and mice [32, 33] is the same. C-terminally truncated Hltf proteins encoded by the truncated message isoform retain all of the domain structure except for the DNA repair domain.

The ratio of full-length to truncated Hltf mRNA isoforms varies in murine tissues [32, 33]. In heart, where Hltf is a direct transcriptional regulator of Hif-1α, this function is attributed to the full length isoform because its ratio to the truncated isoform is 26:1 [33]. This more abundant (dominant) full-length isoform is credited with regulation of the G2/M transition of the cell cycle. Although the full-length HLTF message predominates in most tissues, there are three reported exceptions that favor synthesis of a truncated isoform. One occurs in rabbit endometrium in response to estrogen-regulation [34, 35], and the other two steroid-independent events affect the human gene in the progression of head and neck squamous cell carcinoma [36] and cervical adenocarcinoma [37]. Here we report a fourth example, normal mouse placenta, an immune privileged organ that throughout its temporary existence assumes the functions of the liver, kidneys, gastrointestinal tract, lungs and endocrine glands [38].

## Materials and methods

All studies were conducted in accord with the NIH Guidelines for the Care and Use of Laboratory Animals, as reviewed and approved by the Animal Care and Use Committee at TTUHSC [NIH Assurance of Compliance A3056-01; USDA Certification 74-R-0050, Customer 1481]. TTUHSC's IACUC specifically approved this study. All efforts were made to minimize pain and suffering. Term pregnant females received an IP injection of a Ketamine/Xylazine cocktail at 100 microliters per 20 g body weight. The cocktail contained 87.5 mg/kg Ketamine and 12.5 mg/kg Xylazine. Following surgical removal of unborn pups and their placenta, previously pregnant females were euthanized by drug overdose followed by cervical dislocation.

### Reagents and kits

Sigma-Aldrich was the source of anti-HLTF (HPA 015284 to human HLTF aa 164–300). Abcam was the source of anti-HLTF (ab183042 to human HLTF aa 950-C-terminus), anti-protocadherin gamma (pan)—C-terminal (ab187186) and anti-perforin (ab180773) antibodies. Novus Biologicals was the source of anti-Cxcr7 (MAB42273). DBA lectin (L6533), diaminobenzidine (D12384), and Harris Modified Hematoxylin (HHS16) were purchased from Sigma-Aldrich for use with Streptavidin, peroxidase conjugate (189733) from MilliporeSigma. Biotinylated goat-anti-mouse (BA-9200) and goat anti-rabbit (BA-1000) IgG antibodies and the ABC-enzyme complex were purchased from Vector Laboratories, Inc. DNeasy Blood & Tissue Kit (69506) was purchased from Qiagen for isolation of genomic DNA from tail biopsies. SequalPrep™ Long PCR Kit with dNTPs (A10498) was purchased from ThermoFisher Scientific. PCR primers were synthesized by Midland Certified Reagent Company. OmniPur agarose (2120) was purchased from Calbiochem division of EMD4Biosciences, and MetaPhor® agarose (50181) was purchased from Lonza Rockland, Inc. Promega was the source of agarose gel markers (G171A, G173A, and G176A). ZR Genomic DNA-Tissue MidiPreps (D3110) were purchased from Zmyo Research for use in conjunction with Agilent DNA Chips (5067–1522) and DNA 12000 reagents on the Agilent 2100 bioanalyzer. All protocols are accessible in protocols.io (doi: dx.doi.org/10.17504/protocols.io.pnidmce).

### Hltf null mice

Global Hltf null mice were developed in collaboration with genOway (Lyon, France) as previously described [32] and backcrossed into the C57BL/6J genomic background for 10 generations [33]. These mice were used throughout the study. For a limited comparison study, the Hltf deletion was bred into the recombinase activating gene 2 (Rag2)/common gamma (IL2rg) double knockout background, i.e. mice lacking lymphocytes (NK-, T- B-; alymphoid). Briefly, stock female [*Hltf*^*+/+*^*/Rag2*^*-/-*^*/Il2rg*^*-/-*^] mice from Taconic (4111-F) were crossed with our male [*Hltf*^*-/-*^*/Rag2*^*+/+*^*/Il2*^*+/y*^*]* mice to produce the F1 generation in which all males had the genotype *Hltf*^*+/-*^*/Rag2*^*+/-*^*/Il2*^*-/y*^. These mice were intercrossed with stock female mice [*Hltf*^*+/+*^*/Rag2*^*-/-*^*/Il2rg*^*-/-*^] to produce F2 mice fixed for the Il2rg null allele (homozygous for all offspring). Select F2 mice [*Hltf*^*+/-*^*/Rag*^*-/-*^*/Il2rg*^*-/-*^ x *Hltf*^*+/-*^*/Rag*^*-/-*^*/Il2rg*^*-/y*^] homozygous for Rag2^-/-^ and heterozygous for Hltf^+/-^ were intercrossed to yield F3 triple knockout [*Hltf*^*-/-*^*/Rag2*^*-/-*^*/Il2rg*^*-/y*^ or *Hltf*^*-/-*^*/Rag2*^*-/-*^*/Il2rg*^*-/-*^] and control [*Hltf*^*+/+*^*/Rag2*^*-/-*^*/Il2rg*^*-/-*^or *Hltf*^*+/+*^*/Rag2*^*-/-*^*/Il2rg*^*-/y*^] mice. Triple knockout and control mice are immune compromised as they completely lack T cells, B cells, and natural killer cells [39]. These mice are bred and maintained in sentinel-monitored, bioBubble™-husbandry conditions in the Laboratory Animal Resource Center (LARC) at Texas Tech University Health Sciences Center (TTUHSC).

All studies were conducted in accord with the NIH Guidelines for the Care and Use of Laboratory Animals, as reviewed and approved by the Animal Care and Use Committee at TTUHSC [NIH Assurance of Compliance A3056-01; USDA Certification 74-R-0050, Customer 1481]. TTUHSC's IACUC specifically approved this study. All efforts were made to minimize pain and suffering. Term pregnant females received an IP injection of a Ketamine/Xylazine cocktail at 100 microliters per 20 g body weight. The cocktail contained 87.5 mg/kg Ketamine and 12.5 mg/kg Xylazine. Following surgical removal of unborn pups and their placenta, previously pregnant females were euthanized by drug overdose followed by cervical dislocation.

### Genotyping

At weaning, PCR screening reactions were used to authenticate the *Hltf* null vs wild type genotype [32, 33]. The Rag2 genotyping PCR protocol was adapted from bkeelab.bsd.uchicago.edu/Rag2.pdf. Sexing of fetal mice associated with each placenta was achieved by inspection of genital tubercles, i.e. male newborn mice have a pigmented spot over the scrotum [40], and validated by PCR analysis of the sex-determining region of the Y chromosome (Sry) and myogenin (Myog) of the X chromosome in DNA isolated from fetal tail biopsies [41]. PCR primer sequences are provided in Table 1.

**Table 1:** Primers for gender identification and genotyping.

	Primer Sequence
Hltf—forward	5´-GTTAGGAGTGTTCTGCGTTCTAGGACTGATG-3´
Hltf—reverse	5´-GGGGGAGTAGAAAACTAGACGTACTGAC-3´
RagA	5´-GGGAGGACACTCACTTGCCAGTA-3´
RagB	5´-AGTCAGGAGTCTCCATCTCACTGA -3´
RagNeo	5´-CGGCCGGAGAACCTGCGTGCAA-3´
Myog—forward	5´-TTACGTCCATCGTGGACAGC-3´
Myog—reverse	5´-TGGGCTGGGTGTTAGTCTTA-3´
Sry—forward	5´-TCATGAGACTGCCAACCACAG-3´
Sry—reverse	5´-CATGACCACCACCACCACCAA-3´

### Microscopy

For histological evaluation, each uterine horn was cut between implantation sites. Placentae were peeled away from their attachment to the decidua leaving a residual mesometrial myometrium landmark, emersion-fixed in formalin-based fixatives, cut mid-sagittally and paraffin embedded cut-face down. A mid-sagittal plane is the preferred orientation for visualization of the lymphocyte-rich mural micro domain otherwise known as the mesometrial lymphoid aggregate of pregnancy [21, 42] characterized by an abundance of immune competent uNK cells. Tissue blocks were serially sectioned (4 μm). Two sections were placed on each slide and deparaffinized before staining. Beginning with the first slide, sections on every fifth slide were stained with hematoxylin and eosin (H&E) and evaluated by light microscopy. Sections on alternate slides were stained for amylase-resistant periodic acid Schiff (PAS) positive granules in uNK cells, Dolichos biflorus (DBA) lectin reaction to a glycoconjugate containing N-acetyl D-galatosamine terminal sugar moiety in the plasma membrane and granules of uNK cells, or immunostained (Hltf, protocadherin gamma, Cxcr7, perforin1). The placement of two tissue sections per slide facilitated the use of one section for positive staining, and the companion section for negative (minus primary antibody) control staining. Primary antibodies were used at the following concentrations Hltf (1:100 for HPA015284; 1:50 for ab183042), protocadherin gamma (1:5), Cxcr7 (1:10) and perforin1 (1:25). The secondary antibody was either biotinylated goat anti-mouse or goat anti-rabbit (1:200) depending upon the species in which the primary antibody was generated.

Uterine NK cells were counted (double-blind by RAH and BSC) in duplicate cross-sections from the middle of three different placentae from immunocompetent wild type and Hltf null mice. The fetal-to-placental-weight ratio was calculated from 14 control and 19 null values. Litter size was calculated from 37 null, 31 control, and 26 triple null values. All statistical comparisons were made with GraphPad Prism v7.02 (significance, p<0.05).

### Placentome (RNA-seq)

Pregnancy was determined by the presence of a vaginal plug, i.e. 0.5 day post coitum/embryonic day (dpc/E), obvious presence of embryos after day 14 of gestation, and nest building behavior. Placenta were removed at term (E18.5). Individual samples [1 term placenta/sample x 5 biological replicates for test and control littermate female mice = 10 total samples] were flash frozen and sent to Otogenetics Corp. (Norcross, GA) for RNA-seq assays as previously described [32, 33]. Paired-end 100 nucleotide reads were aligned (mapped, averaged 53.68%) to genomic assembly mm9 (S1 Table) and analyzed using the platform provided by DNAnexus, Inc. (Mountain View, CA) to generate an unbiased gene expression analysis report of RNA-seq; AS analysis of Hltf; mutation/RNA-editing analysis and parallel comparison of expression profiles between null and control samples. The power in detecting AS was dramatically increased by paired-end sequencing relative to single-end sequencing. FPKM (fragments per kilobase of transcript per million mapped reads) were mapped against mm9 with Tophat (V1.3.3) to obtain .bam mapping files that were input into Cufflinks for transcript assembly. Cuffdiff (V 1.3.0), part of the Cufflinks package, uses the alignment reads for rigorous statistical comparison of two conditions (null, control) and five replicates for each condition. Data were imported into iPathwayGuide (Advaita Corporation 2017) a next-gen pathway analysis tool. Standard enrichment parameters (0.6, p<0.05) were used. iPathwayGuide sets a default minimum threshold log fold change (logFC) of 0.6 for inclusion. All RNA-seq data in this publication are accessible through NCBI's Gene Expression Omnibus (GEO) Series accession number GSE114146 (https://www.ncbi.nlm.nih.gov/geo/query/acc.cgi?acc=GSE114146).

### Methyl-MiniSeq™ Epiquest analysis

Individual samples [1 term placenta/sample x 3 biological replicates for null and control mice = 6 total samples] were flash frozen prior to DNA isolation and purity assessment using the Agilent Bioanalyzer. Genomic DNA was sent to Zymo Research (Irvine, CA) for Full Service Methyl-MiniSeq Epiquest analysis. Genomic DNA was subjected to DNA fragmentation, endo modification, adaptor addition, bisulfite conversion, limited amplification, and Illumina HiSeq2000 sequencing. Paired-end reads were aligned (mapped) to genomic assembly mm10 (S2 Table). Nine data sets were generated for each sample: one each for CpG islands, gene promoters, and gene bodies (according to USCS browser GCRm38/mm10 assembly annotations) in CpG, CHG, and CHH contexts.

Samples were grouped based on similarity for the top 100 differentially methylated CpG, CHG, and CHH sites covered in the assay. These genes were subjected to gene ontology analysis with DAVID (Database for Annotation, Visualization and Integrated Discovery) v6.7 to analyze gene enrichment within biological processes. DAVID analysis is agnostic to the direction of methylation change. It included sites that were significantly different for either hypo- or hyper-methylation. Methy-MiniSeq data as discussed in this publication have been deposited in the same GEO Series accession number GSE114146 as the RNA-seq data.

## Results

### Transcriptomic profiling of the E18.5 term placenta

**Fig 1:**
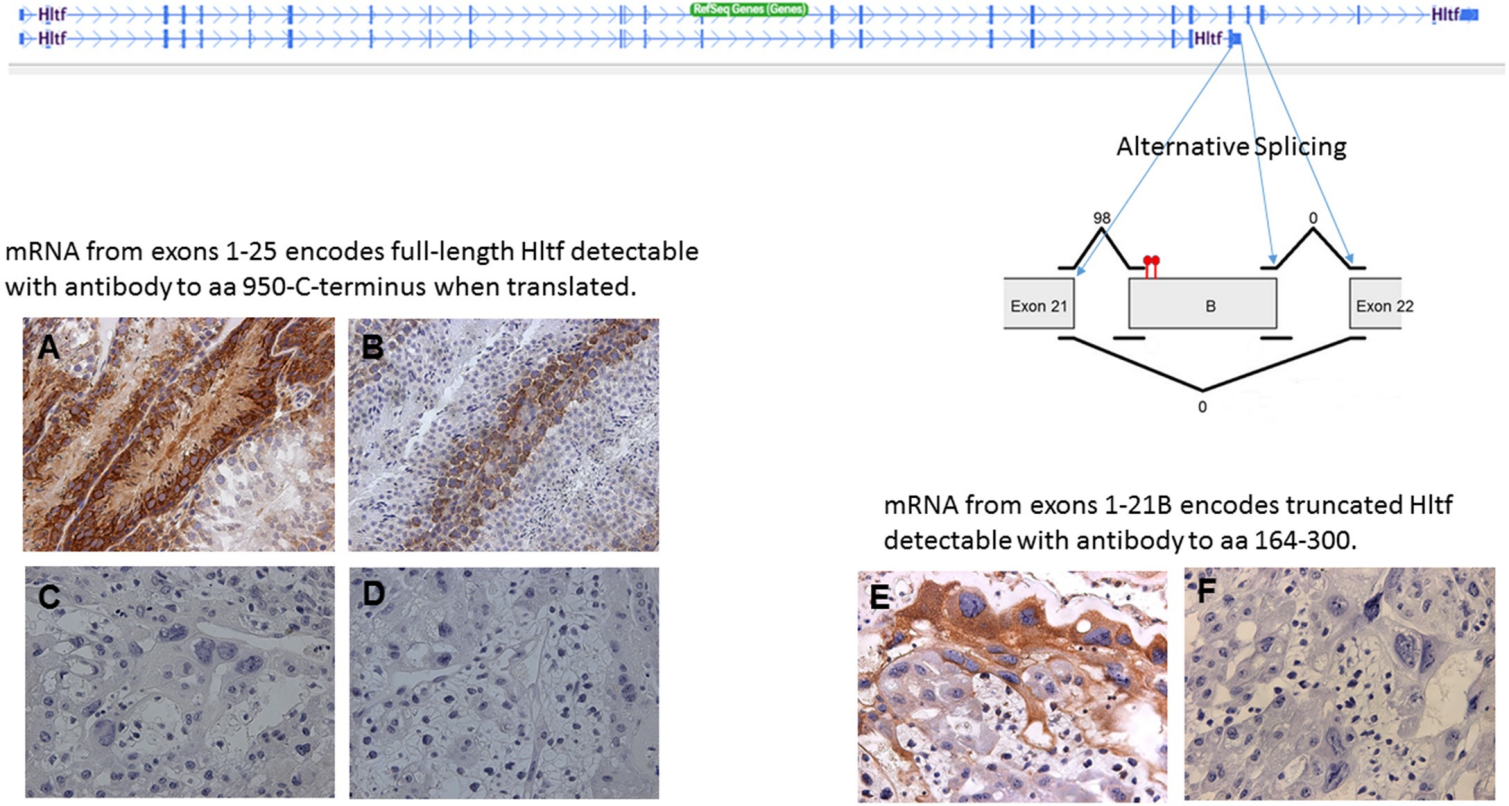
Isoform discovery and alternative expression of Hltf. A diagram illustrating the intron retention event at exon 21 resulting in the formation of exon 21B. Cufflinks assembled the five control transcripts and estimated their abundance. Then cuffdiff tested for differential Hltf isoform expression based on exon and junction read counts. Exon 21 was joined (98 total reads) with intron B to form exon 21B; however, neither exon 21 nor intron B were joined (0 reads) to exon 22. The diagram shows the tandem stop codons (red) in exon 21B that terminate protein translation. Positive immunolabeling of the C-terminus of Hltf in secondary spermatocytes in seminiferous tubules of rat (A) and mouse (B) testis compared with the absence of immunolabeling to the C-terminus of Hltf in placenta (C; negative control D) confirms no full-length Hltf protein is expressed in any cell types of the placenta. Alternatively, immunolabeling with an antibody to amino acids (aa) 164–300 that are common to all known Hltf protein variants confirms the abundance of truncated Hltf protein in placenta (E; negative control F). 40x magnification.

Hltf is alternatively spliced. RNA processing yields a full-length message isoform (4955-bp; exons 1–25) and a 3´-truncated isoform (3059-bp; exons 1–21 with exon 21 extended via an intron retention event) in mouse brain [32] and heart [33]. DNAnexus AS analysis quantified the usage of each exon and each possible splice junction for Hltf in RNA-seq samples from immunocompetent control placentas. As shown in Fig 1, the truncated splice variant is exclusively expressed in term placenta (Fig 1).

In this study, **157** differentially expressed genes were identified out of a total of **11538** genes with measured expression (S3 Table) in immune competent control vs null placenta. These data were analyzed in the context of pathways obtained from the Kyoto Encyclopedia of Genes and Genomes (KEGG) database (Release 81.0+/01-20, Jan 17), gene ontologies from the Gene Ontology Consortium database (2016-Sep26), and diseases from the KEGG database (Release 81.0+/01-20, Jan 17). The top biological process is cytolysis (GO:0019835; p = 1.900e-12), serine-type endopeptidase activity is the top molecular function (GO:0004175; p = 8.700e-7), and extracellular space (GO:0005615; 1.700e-9) is the top cellular component.

### Regulation at the feto-maternal interface

**Fig 2:**
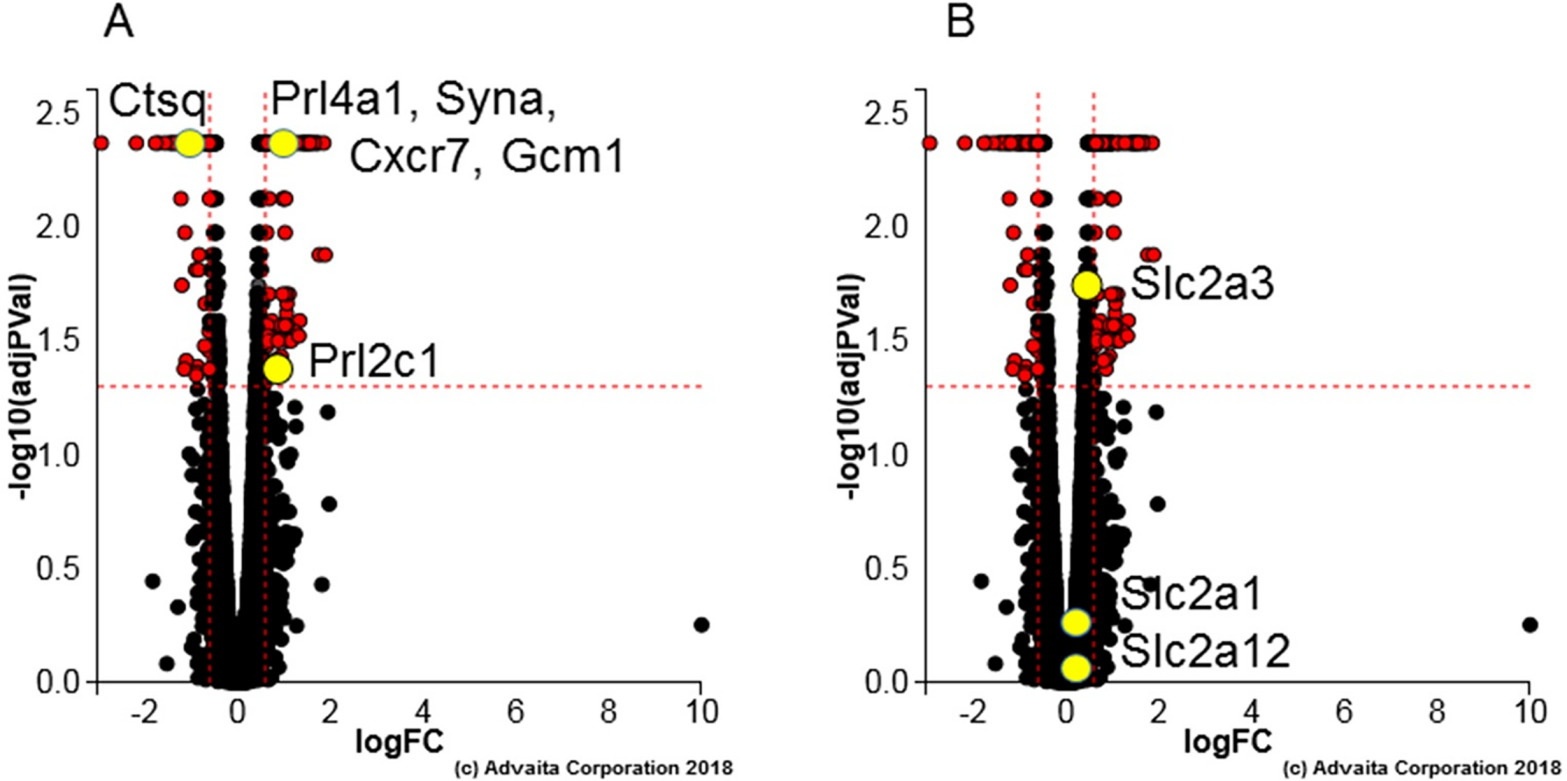
**Volcano plots:** Differential expression (DE) of (A) trophoblast lineage markers [*Prl2c1*, *Ctsq*, *Prl4a1*, *Syna*, *Cxcr7* and *Gcm1*] and (B) glucose regulators [*Slc2a1* (Glut 1), *Slc2a3* (Glut 3), and *Slc2a12* (Glut 12)] is represented in terms of their measured expression change (x-axis) and the significance of the change (y-axis). *Prl4a1*, *Gcm1*, *Syna*, and *Cxcr7* are represented by a single yellow dot because they share the same change in gene expression (p = 0.004) compared to their matched controls. Significance is represented in terms of the negative log (base 10) of the p-value, so that more significant genes are plotted higher on the y-axis. The dotted lines represent the thresholds used to select the DE genes: LogFC = ***0*.*6*** for expression change and ***0*.*05*** for significance (p-value shown in terms of the negative log (base 10) value).

**Fig 3:**
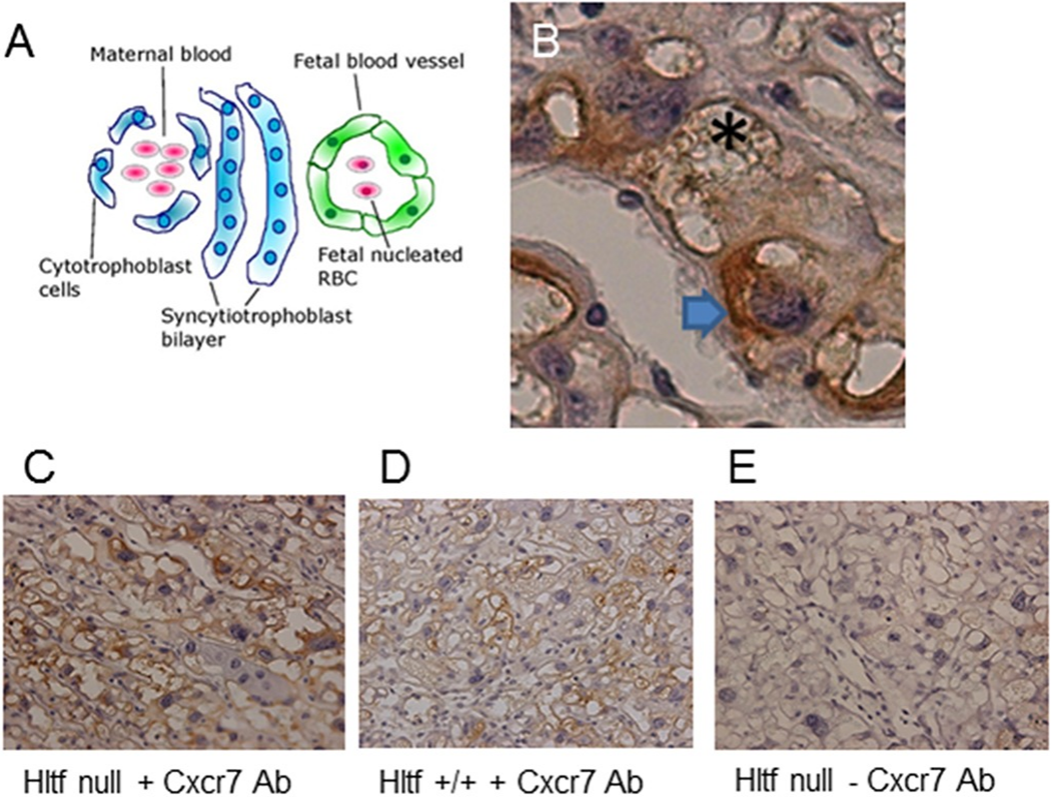
Trilaminar trophoblast structure of the feto-maternal interface. The diagrammatic representation (A) is provided as a guide to understanding Cxcr7 protein localization on the maternal side (arrow) of Hltf null trophoblast (B) with fetal capillary (asterisk). Increased Cxcr7 protein in Hltf null (C) compared to control (D) placenta. Minus primary antibody (E) in Hltf null placenta shows negligible immunostaining. 40x magnification.

As shown in Fig 2A, Hltf deletion revealed the role of Hltf in trophoblast-specific gene transcription. Hltf deletion caused up-regulation of invasive trophoblast giant cell markers (*Prl4a1*, *Prl2c1*), increased transcriptional availability of the syncytiotrophoblast cell markers glial cell missing-1 (*Gcm1*) and Syncytin-A (*Syna*), but decreased transcription of *Ctsq* (sinusoidal trophoblast giant cell marker). Of the three placenta layer-specific markers, *Syna* is a gene of retroviral origin [43]. Importantly, then, transcriptional availability of *Cxcr7* (Fig 2B), a putative co-receptor for HIV-1, HIV-2 and simian immunodeficiency virus (SIV) strains [44], was increased (p = 0.004) concomitant with increased Cxcr7 protein in Hltf null trophoblasts at the feto-maternal interface (Fig 3).

**Fig 4:**
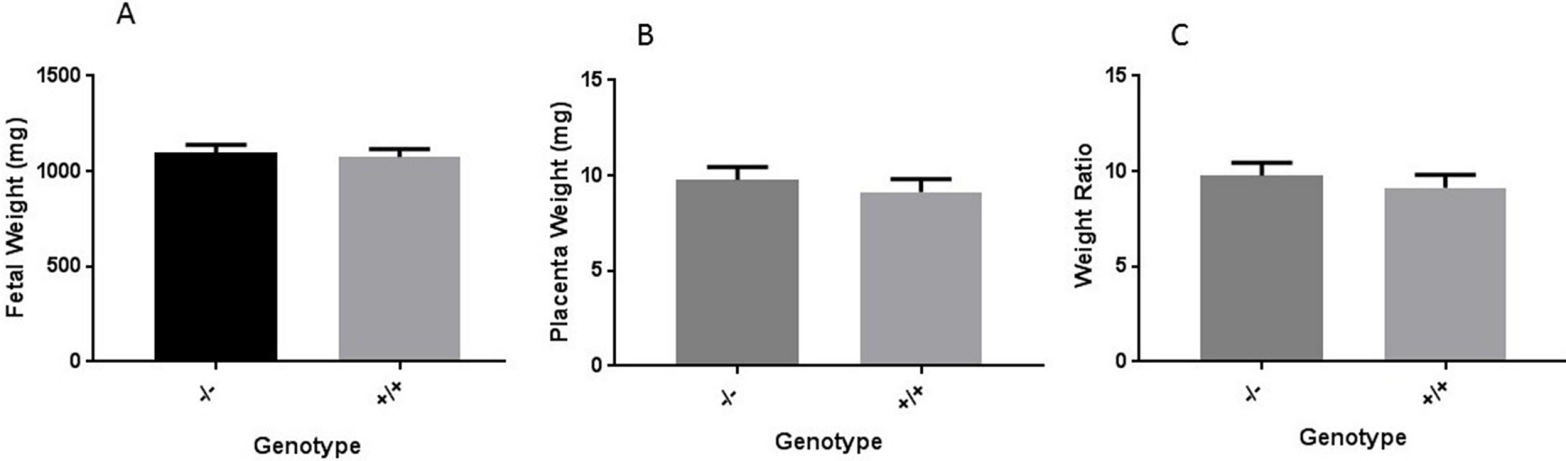
The effect of Hltf deletion on the fetal-to-placental-weight ratio. Fetal weight (A), placental weight (B) and fetal/placental weight ratios (C) were unaffected by *Hltf* deletion. Statistics for *Hltf* -/- animals were: fetal weight (n = 18), placental weight (n = 28) and ratio data (n = 18 paired values). Statistics for *Hltf* +/+ animals were: fetal weight (n = 19), placental weight (n = 26), and ratio data (n = 19 paired values). Values in each category were compared with Student’s *t*-test (significance, p<0.05). The histograms show data are not different (p>0.05).

As shown in Fig 2B, iPathway analysis revealed a negligible effect of *Hltf* deletion on the expression of the facilitated glucose transporter *Slc2a1*, and the insulin-sensitive glucose transporter *Slc2a12*. However, the effect of Hltf deletion on the expression change (0.453) for the constitutive glucose transporter *Slc2a3* with p = 0.018 initially seemed problematic. Although the p value was significant, the expression change did not meet the default minimum threshold log fold change (logFC) of 0.6 for inclusion. Additional analyses showed there were no accompanying differences in System A amino acid transporters (S3 Table). Most importantly there were no measurable differences the classical hallmarks [45, 46] for adverse pregnancy outcomes, i.e. placental weight or shape, fetal/placental weight ratios, or newborn weights (Fig 4). The finding of fetal normalcy supports our conclusion that neonatal lethal phenotype is not caused by changes in placental nutrient transport.

### Hltf represses transcription of genes in the innate immune system

**Fig 5:**
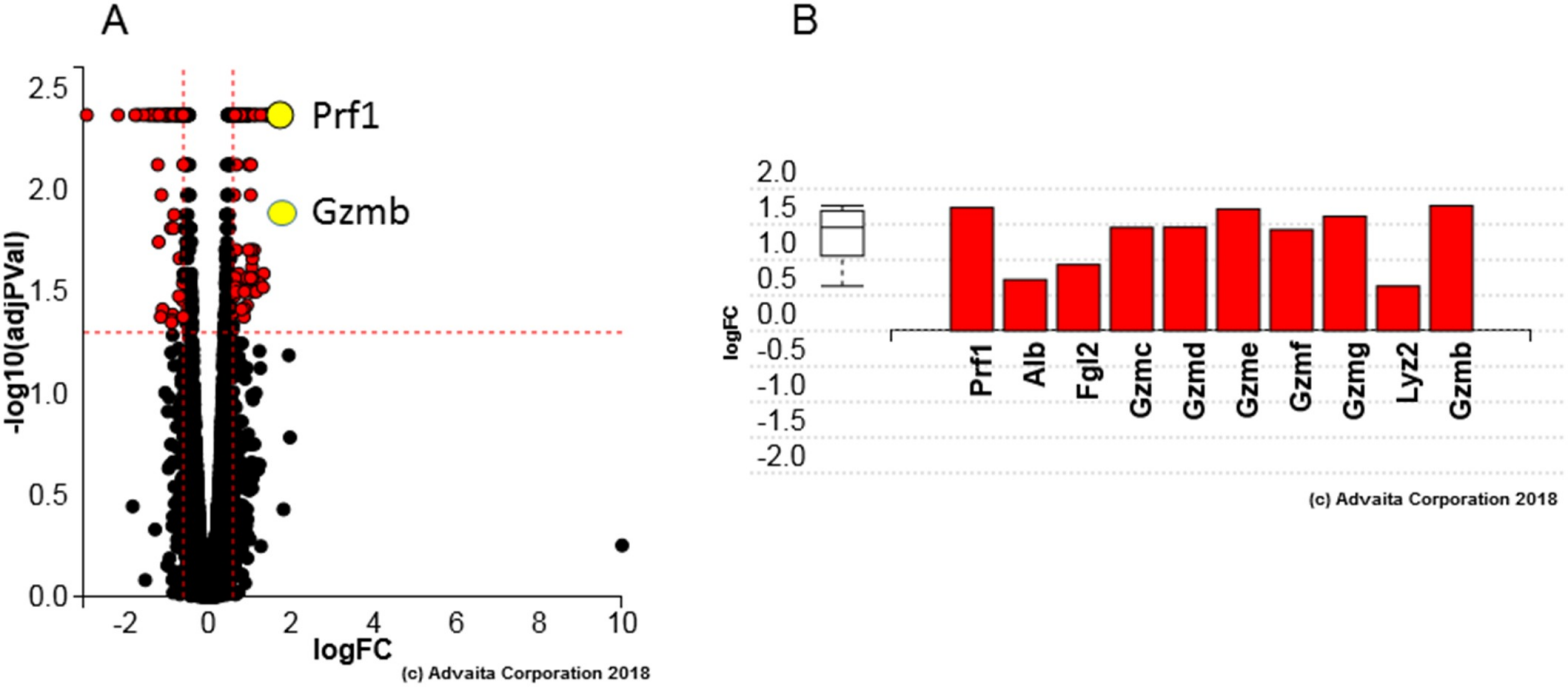
**Gene measured composite volcano and expression bar plots:** A. Volcano plot highlighting increased expression of perforin 1 *(Prf1*) and granzyme B (*Gzmb*) when *Hltf* is deleted. B. All the differentially increased genes [perforin1 (*Prf1*), albumin (*Alb*), fibrinogen-like protein 2 (*Fgl2*), granzymes (*Gzmb-Grzmg*) and lysozyme 2 (*Lyz2*)] that are annotated to cytolysis are ranked based on the significance of their measured logFC. The box and whisker plot on the left summarizes the distribution of all the differentially increased genes that are annotated to this GO:0019835 term. The box represents the 1st quartile, the median and the 3rd quartile.

Immune competent uNK cells peak at gestational day (gd) 10–12, and begin to decline on or about gd15 [47]. As late as gd18.5 Hltf is a transcriptional repressor of the cytolytic perforin-granzyme pathway (10/21 genes; p-value 1.900e-12; p-value correction: Elim pruining) in uterine natural killer (uNK) cells (Fig 5).

**Fig 6:**
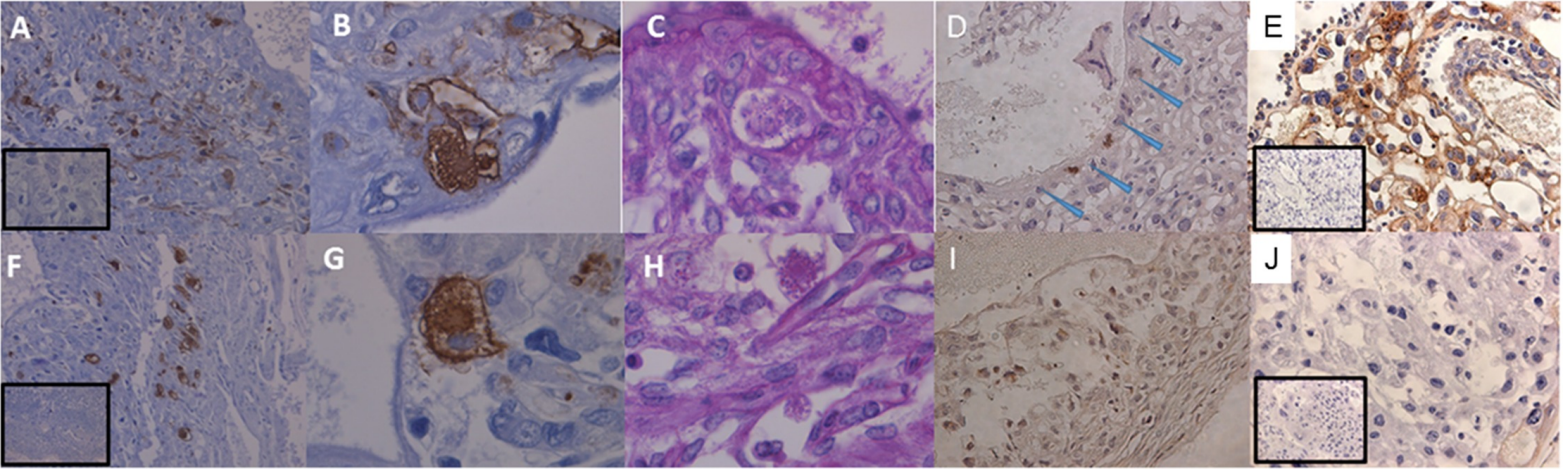
Photomicrographs of histological sections of mouse placenta on gd18.5. Sections from wild type (A-E) and Hltf null (F-J) placentae show widespread DBA-lectin reactivity for uNK cells at 10 X magnification (A, F). Comparisons at 40 X magnification of DBA-lectin reactivity for uNK cells (B, G) and amylase-resistant PAS positive uNK cells (C, H) show abundant cytoplasmic granules contained within the cellular membranes. Immunolabeling (20 x magnification) for perforin is negligible in uNK cells from wild type (D; blue arrows) compared with Hltf null (I). Immunolabeling (20 x magnification) for Hltf shows both nuclear and cytoplasmic staining throughout the wild type (E) placenta compared to the complete absence of Hltf protein in the null placenta (J). All insets are negative (minus primary antibody) controls.

Quantification of uNK cell populations on gd18.5 showed there was no effect of Hltf deletion on uNK cell numbers per section (controls 197.8 ± 23.09 vs null 155.3 ± 24.04, p = 0.2494). This finding is consistent with the fact that there was no change in the transcriptional availability (S3 Table) of stromal cell genes (*Cxcl10*, *Cx3cl1*, and *Ccl2*) that promote migration of peripheral uNK cells. As shown in Fig 6, DBA lectin was used to identify both granule-rich and granule-depleted Hltf-positive uNK cells due to its specificity for glycoconjugate containing N-acetyl D-galactosamine terminal sugar moiety in the plasma membrane and granules [48]. Amylase-resistant PAS staining was used to identify both DBA- and DBA+ cells [49]. As shown in Fig 5, when Hltf is silenced, transcription of the genes for perforin, a pore forming cytolytic protein, and serine proteases (granzymes B-G) is increased along with increased transcription of other members of the innate immune system: lysozyme (*Lyz2*), albumin (*Alb*) and fibrinogen-like protein 2 (*Fgl2*). Increased immunolabeling for perforin (Fig 6) is commensurate with increased perforin gene transcription (RNA-seq). However, silencing Hltf did not trigger cytosolic release as there was negligible evidence of degranulation in uNK cells in either Hltf null or control placentae. These data confirm the immunoprotective role of Hltf in placenta.

Uterine NK cells are the dominant lymphocytes with a role in angiogenesis during decidualization [20, 21]; however, there was no increase in the transcriptional availability (S3 Table) of angiogenic growth factors (*Vegf-C*, placental growth factor, angiopoietin-1 and -2, or *Tgfβ1*). Moreover, breeding the *Hltf* null deletion into the *Rag2/Il2*-null background, which is genetically deficient for NK/uNK cells, allowed us to evaluate the effects of *Hltf* silencing on the placenta in the absence of uNK cells. Litter sizes for *Hltf* null, *Hltf*+/+, as well as immunocompromised mice with and without Hltf deletion [*Hltf*^*-/-*^*/Rag2*^*-/-*^*/Il2rg*^*-/-*^ and *Hltf*^*+/+*^*/Rag2*^*-/-*^*/Il2rg*^*-/-*^] was not different with gestation averaging 18.5–19 days and the number of live born pups averaging 6–8.

### Methyl-MiniSeq Epiquest analysis

**Fig 7:**
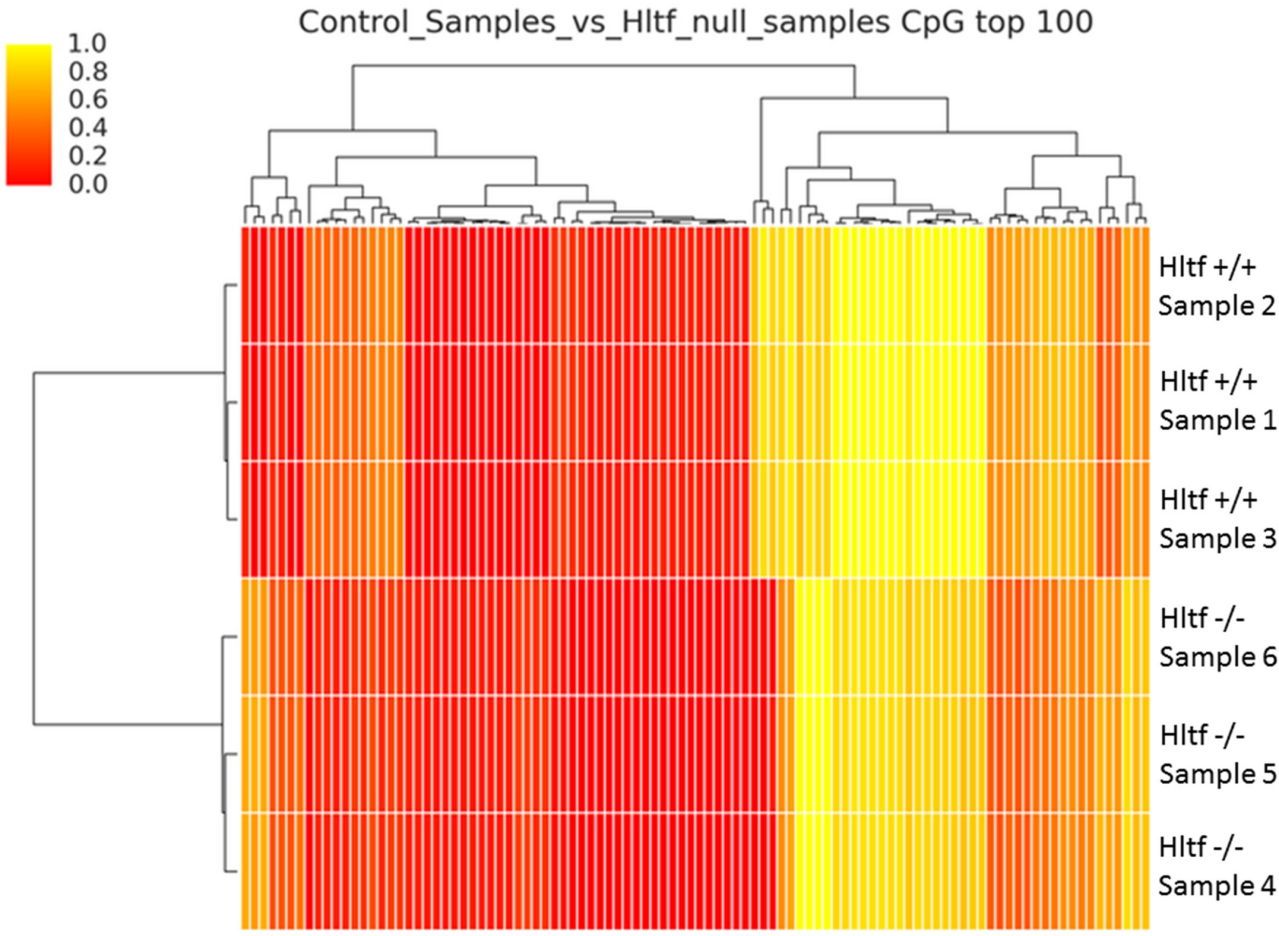
Heat map. Hierarchical clustering of control (1–3) vs *Hltf* null (4–6) samples grouped according to similarity for the top 100 differentially methylated CpG sites covered by EpiQuest Genome-wide DNA methylation analysis. The color gradation from yellow (high) through orange (intermediate) to red (low) indicates the levels of DNA methylation.

**Fig 8:**
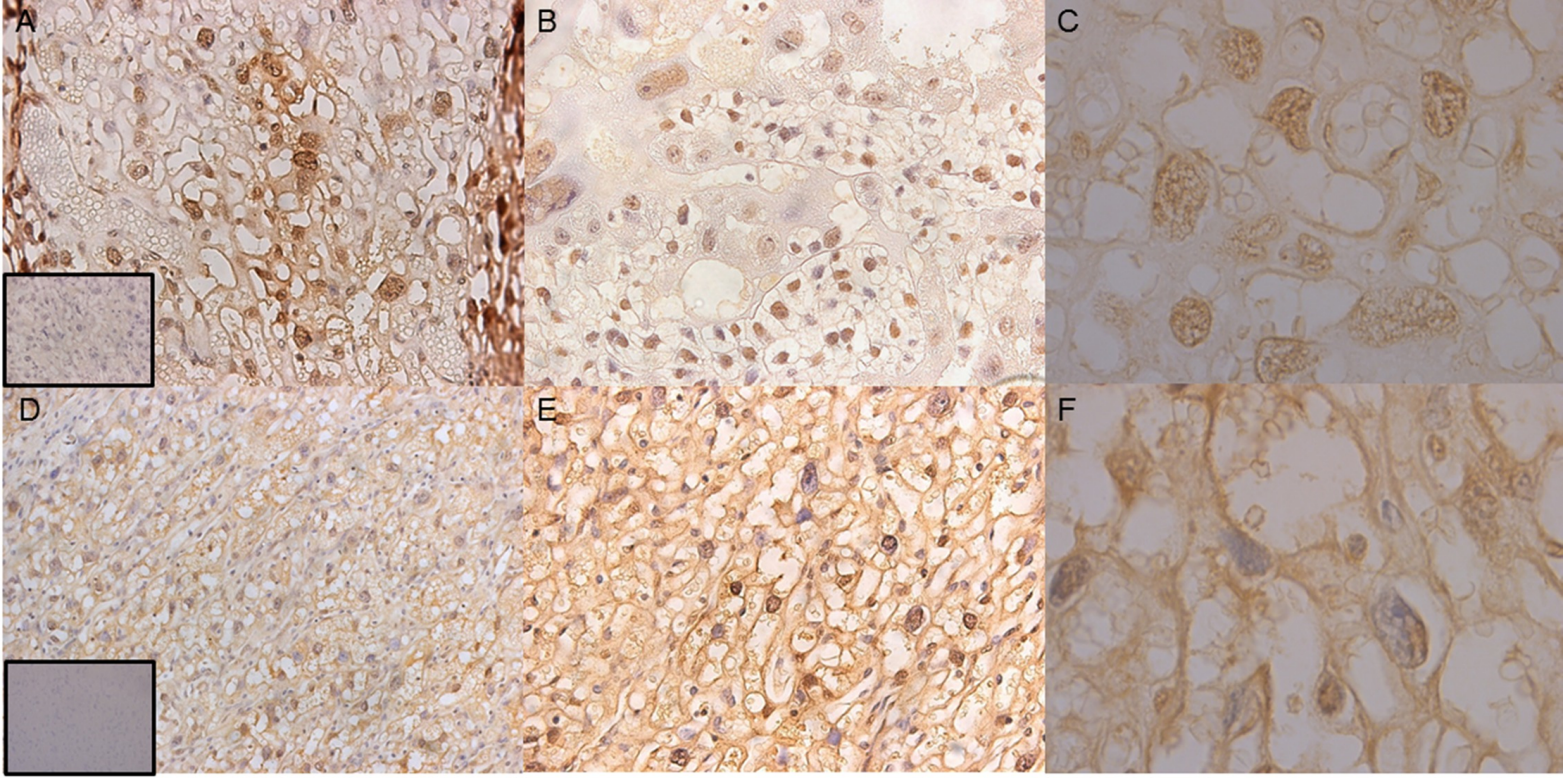
Nuclear localization of protocadherin gamma in trophoblasts. Proteolytic processing by γ-secretase precedes nuclear localization of the C-terminal domains common to all γ-protocadherin adhesion proteins. Immunolabeling with an antibody to the C-terminal domain of all γ-protocadherin proteins shows extensive nuclear localization of the proteolytic fragment in wild type control (A-C) compared with staining on the inner, cytoplasmic, side of the cell membrane, diffuse cytoplasmic stain and generally negligible nuclear staining in *Hltf* null (D-F) placenta. Magnifications are A, D 20X, B, E 40X, C, F 100X oil emersion.

Genome-wide methylation profiling at single-base resolution was performed to determine whether DNA methylation accompanied intron retention to form exon 21B, whether deleting *Hltf* altered DNA methylation patterns, and whether differentially methylated regions (DMRs) coincided with changes in transcriptional regulation of Hltf target genes. EpiQuest sequencing from Zymo Research was used to investigated changes in all three genome-wide cytosine methylation contexts, i.e. GpG, CHG and CHH, where H is A,T, or C but not G. The average depth of CpG coverage was 13-17x, and the bisulfate conversion rates were 99% for all the samples (S2 Table). As shown in S1 Fig, the intron retention event responsible for the intron-retaining transcripts occurs in the absence of any cytosine methylation. The heat map with hierarchical clustering (Fig 7), showed altered patterns of CpG DNA methylation occurred in response to *Hltf* deletion. DAVID analysis in which GO terms for CpG sites with a count of at least 9 genes and an enrichment score greater than 5 revealed protocadherin gamma isoforms A1-8 and B1, 2, 4 and 5 were most dramatically affected, i.e. 156-fold enrichment (S4 Table). Surprisingly, these changes in site-specific DNA methylation were unaccompanied by altered transcription or differential splicing. However, as shown in Fig 8, nuclear localization of the C-terminal domains common to all protocadherin gamma proteins [50, 51] is evident in cells from sections of wild type control labyrinth, and negligible in cells of the labyrinth of *Hltf* null placenta. This effect of *Hltf* deletion occurs in the absence of any change in the transcriptional availability (S3 Table) of either of the two major components (MMPs, γ-secretase) responsible for this proteolytic processing [51].

## Discussion

AS increases proteomic diversity; however, for most genes there is a paucity of experimental validation of isoform-specific function [52]. Mice [32, 33] and rabbits [34, 35] have two HLTF splice variants, a full-length message isoform and a 3´-truncation isoform that results from an intron retention event. As shown in Fig 1, the retained intron contains in-frame tandem stop codons, which distinguishes it from an exitron (exonic intron) that is characterized by the absence of stop codons [53, 54]. Wong et al [55] recently reported intron retention is mediated by DNA methylation. Our findings are consistent with their conclusions, i.e. the absence of DNA methylation near the splice junctions of Hltf promotes intron retention. However, intron-retaining transcripts frequently contain premature termination codons that cause their removal by nonsense-mediated decay (NMD) thereby reducing gene expression and cellular differentiation. In contrast, AS of Hltf in placenta is a model in which gene expression and cellular differentiation are mediated by functional Hltf protein from intron-retained transcripts that are not lost to NMD. This is an important distinction because in our model no full-length transcripts are produced.

We are the first to identify the mouse placenta as an *in vivo* model in which to study the function of the Hltf 3´-truncation isoform that encodes a protein capable of transcriptional regulation but is theoretically incapable of DNA damage repair. In this context, Hltf promotes immune homeostasis through suppression of the innate immune system at the fetal-maternal interface and may have contributed to the evolution of the mammalian placenta. Certainly, the absence of DNA repair would support a permissive environment for endoreplication. Moreover, the absence of adequate DNA damage repair mechanisms necessary to placenta development helps explain the defective repair response in a variety of human scenarios such as smoking-exposed placentae [14].

*HLTF* is a tumor suppressor silenced by promoter hypermethylation [28]. Although the reason for epigenetic *HLTF* silencing is unknown, it is assumed the elimination of HLTF’s DNA damage repair function benefits tumor progression. Recent studies showed HLTF is targeted for ubiquitination by viral protein R (Vpr) expressing immunodeficiency virus (HIV-1) [56, 57]. Surprisingly, HLTF deficiency did not trigger DNA-damage checkpoint. Perhaps it is the loss of HLTF as a transcription factor that is of paramount importance rather than its role in DNA damage repair.

Uteroplacental blood flow and the expression of specific transporter genes mediate nutritional effects on fetal growth [58, 59]. Molecular mechanisms controlling placental transporters are incompletely understood; however, altered nutrient transport causes pathophysiological fetal growth including intrauterine growth restriction (IUGR) and large for gestational age (LGA, macrosomia). Placental size and weight are important indicators of nutrient transport [45, 46]. The fetal to placenta weight ratio is an indicator of nutrient transporter efficiency because fetal and placental weight in humans and mice are positively correlated near term. As the primary energy substrate for growth of fetus and placenta, maternal glucose is supplied from the maternal circulation. Altered expression of glucose transporters causes fetal hypoglycemia [58, 59]. Because *Hltf* null mice become hypoglycemic after birth, it was important to test the effects of *Hltf* deletion on transcriptional availability of nutrient transporters in placenta. The negligible effect of *Hltf* deletion on the expression of glucose transports as well as System A amino acid transporters, also known to modulate fetal under- or overgrowth [60], in placenta allows us to eliminate the effects of *Hltf* deletion on these nutrient transporters on the perinatal lethal phenotype.

Clustered protocadherins consist of three gene families designated *Pcdhα*, *Pcdhβ*, and *Pcdh*γ [50, 51].The mouse protocadherin-γ gene cluster is part of a tandem array on chromosome 18. Each transcript contains the 5’-most variable exon–encoding the entire extracellular component consisting of 6 cadherin ectodomains, a single-pass transmembrane domain, and a proximal cytoplasmic domain—followed by three consistent exons that encode an additional 125 amino acid C-terminal domain common to all 22 members of the cluster. Proteolytic processing of protocadherin-γ proteins requires the sequential release of the ectodomain by sheddases such as α-secretase (matrix metaloprotinase), followed by γ-secretase intramembrane proteolysis (unknown cleavage site) to release the C-terminal fragment (CTF) into the cytosol [51]. The CTF is translocated to the nucleus where it is purported to regulate gene transcription. However, because the placenta has no nerves, and much of what is known about protocadherins is in neurodevelopment [61], we can only report that Hltf plays an as yet undetermined role in the translocation of the CTF to the nucleus of non-neuronal cells.

## Conclusions

Hltf is an alternatively spliced transcription factor [32, 33]. Heretofore it was unknown that the C-terminal truncated Hltf protein resulting from an intron-retention event is expressed solely in the murine placenta where it regulates transcription of trophoblast lineage-specific genes (S3 Table) and limits the cytotoxic function of uterine natural killer (uNK) cells in close proximity to invading trophoblast cells (S3 Table). Moreover, Hltf deletion alters the epigenetic landscape of the placenta (S4 Table). Having eliminated the placenta in a supporting role for the Hltf null perinatal lethal phenotype, the next step is to quantify Hltf deletion effects in a pancreatic beta cell-specific knockout mouse model.

## Supporting information

S1 Fig Screen shot. Superimposition of all the Hltf control sequences (1,2 and 3) for cytosine methylation contexts, i.e. GpG, CHG and CHH, on the GCRm38/mm10 assembly for Hltf shows there is no site-specific cytosine-methylation affiliated with the targeted intron retention event resulting in Exon21B transcripts.
(PDF)

S1 Table Sample quality control and RNA-seq outcome. Agilent 2100 Expert software assigns an RNA integrity number (RIN) to the entire electrophoretic tract of the total RNA isolated from each of the ten placenta samples. The goal is to limit biased degradation of representative RNA species as a result of rRNA depletion. High RIN scores (7–10) and a narrow distribution of scores (1–1.5) coincides with the fact that the total bases, total reads, and total mapped reads are comparable for control and Hltf null samples.
(PDF)

S2 Table Statistics of the mapping of methylation profiles of control (+/+) and Hltf null (-/-) placentae. The Methyl-MiniSeq platform is based on an expanded RRBS (Reduced Representation Bisulfite Sequencing) protocol. In this genome-wide pipeline, a CpG-enriched fraction is used to represent the methylation signature of the whole genome. DNA methylation occurs predominantly in a CpG context, and these CpG di-nucleotides are more abundant in select regions of the genome.
(PDF)

S3 Table Met-analysis gene summay identified 157 differentially expressed genes. Cuffdiff, a component of Cufflinks, uses RPKM values to calculate changes in gene expression. Cuffdiff data were input into iPathwayGuide. Met-analysis calculated log fold change (logFC) and an adjusted p-value (adjpv) for each comparison. OtA5088-5092 and OtA5093-5097 are designations for five sets of control and Hltf null data, respectively.
(PDF)

S4 Table DAVID functional annotation analysis. This is the first study to evaluate epigenetic changes due to Hltf deletion. DNA methylation changes in CpG islands in mouse placenta are region-specific, and indicate Hltf is important in the maintenance of the epigenetic landscape.
(PDF)
