# Supplementary material for: Alternative splicing of helicase-like transcription factor (Hltf): Intron retention-dependent activation of immune tolerance at the feto-maternal interface
Source: PLoS One. 2018 Jul 5;13(7):e0200211. doi: 10.1371/journal.pone.0200211 (PMC6033450; doi:10.1371/journal.pone.0200211)
Supplement: S1 Fig — Superimposition of all the Hltf control sequences (1,2 and 3) for cytosine methylation contexts, i.e. GpG, CHG and CHH, on the GCRm38/mm10 assembly for Hltf shows there is no site-specific cytosine-methylation affiliated with the targeted intron retention event resulting in Exon21B transcripts. (PDF) [file pone.0200211.s001.pdf]

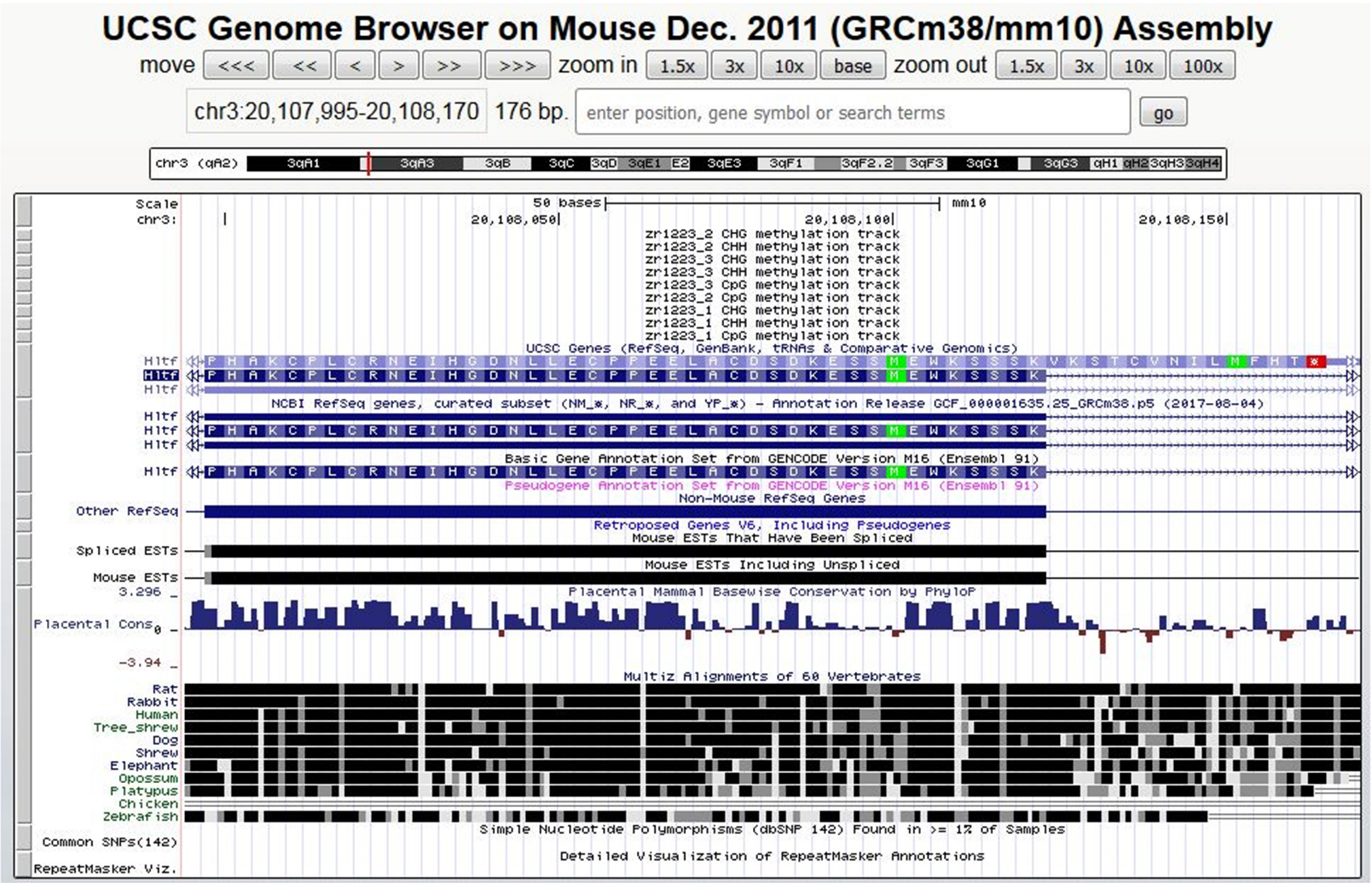

UCSC Genome Browser on Mouse Dec. 2011 (GRCm38/mm10) Assembly
move <<< << < > >> >>> zoom in 1.5x 3x 10x base zoom out 1.5x 3x 10x 100x
chr3:20,107,995-20,108,170 176 bp.
enter position, gene symbol or search terms
go
chr3 (qA2)
Scale
50 bases
mm10
chr3:
20,108,050
20,108,100
20,108,150
zr1223_2 CHG methylation track
zr1223_2 CHH methylation track
zr1223_3 CHG methylation track
zr1223_3 CHH methylation track
zr1223_3 CpG methylation track
zr1223_2 CpG methylation track
zr1223_1 CHG methylation track
zr1223_1 CHH methylation track
zr1223_1 CpG methylation track
UCSC Genes (RefSeq, GenBank, tRNAs & Comparative Genomics)
Hltf
NCBI RefSeq genes, curated subset (NM_*, NR_*, and YP_*) - Annotation Release GCF_000001635.25_GRCm38.p5 (2017-08-04)
Basic Gene Annotation Set from GENCODE Version M16 (Ensembl 91)
Pseudogene Annotation Set from GENCODE Version M16 (Ensembl 91)
Non-Mouse RefSeq Genes
Other RefSeq
Retroposed Genes V6, Including Pseudogenes
Mouse ESTs That Have Been Spliced
Spliced ESTs
Mouse ESTs Including Unspliced
Mouse ESTs
3.296
Placental Mammal Basewise Conservation by PhyloP
Placental Cons
0
-3.94
Multiz Alignments of 60 Vertebrates
Rat
Rabbit
Human
Tree_shrew
Dog
Shrew
Elephant
Opossum
Platypus
Chicken
Zebrafish
Simple Nucleotide Polymorphisms (dbSNP 142) Found in >= 1% of Samples
Common SNPs(142)
Detailed Visualization of RepeatMasker Annotations
RepeatMasker Viz.
