# Supplementary material for: Alternative splicing of helicase-like transcription factor (Hltf): Intron retention-dependent activation of immune tolerance at the feto-maternal interface
Source: PLoS One. 2018 Jul 5;13(7):e0200211. doi: 10.1371/journal.pone.0200211 (PMC6033450; doi:10.1371/journal.pone.0200211)
Supplement: S1 Table — Agilent 2100 Expert software assigns an RNA integrity number (RIN) to the entire electrophoretic tract of the total RNA isolated from each of the ten placenta samples. The goal is to limit biased degradation of representative RNA species as a result of rRNA depletion. High RIN scores (7–10) and a narrow distribution of scores (1–1.5) coincides with the fact that the total bases, total reads, and total mapped reads are comparable for control and Hltf null samples. (PDF) [file pone.0200211.s002.pdf]

S1 Table Sample quality control and RNA-seq outcome.

Sample ID	OD260/280	RIN	Total Bases	Total Reads	Mapped Reads
1-Control	2.13	7.4	3,389,172,000	33,891,720	58.08%
2-Control	2.11	7.6	4,010,136,800	40,101,368	50.29%
3-Control	2.10	8.0	4,156,136,800	41,561,368	51.83%
4-Control	2.13	8.2	3,129,473,400	31,294,734	62.31%
5-Control	2.09	7.1	3,422,688,400	34,226,884	58.97%
6-Null	2.11	8.1	3,322,239,000	33,222,390	56.45%
7-Null	2.10	8.9	3,652,915,400	36,529,154	47.91%
8-Null	2.11	8.4	3,291,146,000	32,911,460	52.59%
9-Null	2.12	8.3	4,017,065,800	40,170,658	54.39%
10-Null	2.11	8.3	3,196,424,400	31,964,244	43.99%

An RNA integrity number (RIN) from an Agilent Bioanalyzer within the range of 7-10 indicates high RNA sample quality.
