# Supplementary material for: Alternative splicing of helicase-like transcription factor (Hltf): Intron retention-dependent activation of immune tolerance at the feto-maternal interface
Source: PLoS One. 2018 Jul 5;13(7):e0200211. doi: 10.1371/journal.pone.0200211 (PMC6033450; doi:10.1371/journal.pone.0200211)
Supplement: S2 Table — The Methyl-MiniSeq platform is based on an expanded RRBS (Reduced Representation Bisulfite Sequencing) protocol. In this genome-wide pipeline, a CpG-enriched fraction is used to represent the methylation signature of the whole genome. DNA methylation occurs predominantly in a CpG context, and these CpG di-nucleotides are more abundant in select regions of the genome. (PDF) [file pone.0200211.s003.pdf]

S2 Table Statistics of the mapping of methylation profiles of control (+/+) and Hltf null (-/-) placentae					
Sample ID	Total Read Number (read pairs)	Mapping Efficiency	Unique CpGs	Avg CpG Coverage	Bisulfite Conversion Rate
Zr1223_1 Hltf +/+	41,800,153	57%	4,488,110	16X	99%
Zr1223_2 Hltf +/+	40,741,707	56%	4,454,312	16X	99%
Zr1223_3 Hltf +/+	39,530,119	55%	4,453,769	16X	99%
Zr1223_4 Hltf -/-	40,456,588	55%	4,462,078	16X	99%
Zr1223_5 Hltf -/-	35,411,784	57%	4,335,448	13X	99%
Zr1223_6 Hltf -/-	42,954,559	56%	4,442,555	17X	99%
