# Supplementary material for: Alternative splicing of helicase-like transcription factor (Hltf): Intron retention-dependent activation of immune tolerance at the feto-maternal interface
Source: PLoS One. 2018 Jul 5;13(7):e0200211. doi: 10.1371/journal.pone.0200211 (PMC6033450; doi:10.1371/journal.pone.0200211)
Supplement: S4 Table — This is the first study to evaluate epigenetic changes due to Hltf deletion. DNA methylation changes in CpG islands in mouse placenta are region-specific, and indicate Hltf is important in the maintenance of the epigenetic landscape. (PDF) [file pone.0200211.s005.pdf]

S4 Table DAVID functional annotation analysis.

Annotation Cluster 1 | Enrichment Score: 5.023844105978458

Category	Term	Count	%	PValue
INTERPRO	IPR015492:Protocadherin gamma	9	10.84337	1.43E-16
INTERPRO	IPR013164:Cadherin, N-terminal	9	10.84337	1.93E-11
INTERPRO	IPR002126:Cadherin	10	12.04819	2.99E-10
GOTERM_BP_FAT	GO:0007156~homophilic cell adhesion	10	12.04819	1.03E-09
SMART	SM00112:CA	10	12.04819	2.21E-09
GOTERM_BP_FAT	GO:0016337~cell-cell adhesion	12	14.45783	2.57E-09
GOTERM_BP_FAT	GO:0007155~cell adhesion	14	16.86747	3.25E-07
GOTERM_BP_FAT	GO:0022610~biological adhesion	14	16.86747	3.32E-07
SP_PIR_KEYWORDS	cell adhesion	12	14.45783	4.42E-07
SP_PIR_KEYWORDS	calcium	14	16.86747	8.50E-06
GOTERM_MF_FAT	GO:0005509~calcium ion binding	14	16.86747	6.27E-05
GOTERM_CC_FAT	GO:0005624~membrane fraction	11	13.25301	6.52E-05
GOTERM_CC_FAT	GO:0005626~insoluble fraction	11	13.25301	8.71E-05
GOTERM_CC_FAT	GO:0000267~cell fraction	11	13.25301	2.35E-04
SP_PIR_KEYWORDS	cell membrane	18	21.68675	5.20E-04
GOTERM_CC_FAT	GO:0005886~plasma membrane	23	27.71084	0.004033
SP_PIR_KEYWORDS	membrane	28	33.73494	0.176361
UP_SEQ_FEATURE	topological domain:Cytoplasmic	13	15.66265	0.411365
SP_PIR_KEYWORDS	transmembrane	24	28.91566	0.413024
GOTERM_CC_FAT	GO:0016021~integral to membrane	26	31.3253	0.564287
GOTERM_CC_FAT	GO:0031224~intrinsic to membrane	26	31.3253	0.65801
UP_SEQ_FEATURE	transmembrane region	15	18.07229	0.777217

Genes
PCDHGB1, PCDHGA8, PCDHGA7, PCDHGA6, PCDHGA5, PCDHGA4, PCDHGB2, PCDHGA3, PCDHGB5, PCDHGA2, PCD
PCDHGB1, PCDHGA8, PCDHGA7, PCDHGA6, PCDHGA5, PCDHGA4, PCDHGB2, PCDHGA3, PCDHGB5, PCDHGA2, PCD
PCDHGA8, PCDHGA7, PCDHGA6, PCDHGA5, PCDHGA4, PCDHGB2, PCDHGA3, PCDHGA2, PCDHGB5, PCDHGB4, PCD
PCDHGA8, PCDHGA7, PCDHGA6, PCDHGA5, PCDHGA4, PCDHGB2, PCDHGA3, PCDHGA2, PCDHGB5, PCDHGB4, PCD
PCDHGA8, PCDHGA7, PCDHGA6, PCDHGA5, PCDHGA4, PCDHGB2, PCDHGA3, PCDHGA2, PCDHGB5, PCDHGB4, PCD
PCDHGA8, PCDHGA7, PCDHGA6, PCDHGA5, PCDHGA4, PCDHGB2, PCDHGA3, PCDHGA2, PCDHGB5, CTNNA3, PCDH
PPARD, PCDHGA8, PCDHGA7, PCDHGA6, PCDHGA5, PCDHGA4, PCDHGB2, PCDHGA3, CTNNA3, PCDHGA2, PCDHG
PPARD, PCDHGA8, PCDHGA7, PCDHGA6, PCDHGA5, PCDHGA4, PCDHGB2, PCDHGA3, CTNNA3, PCDHGA2, PCDHG
PCDHGA8, PCDHGA7, PCDHGA6, PCDHGA5, PCDHGA4, PCDHGB2, PCDHGA3, PCDHGA2, PCDHGB5, CTNNA3, PCDH
SCUBE3, PCDHGA8, PCDHGA7, PCDHGA6, PCDHGA5, PCDHGA4, PCDHGB2, PCDHGA3, PCDHGA2, PCDHGB5, PCDH
SCUBE3, PCDHGA8, PCDHGA7, PCDHGA6, PCDHGA5, PCDHGA4, PCDHGB2, PCDHGA3, PCDHGA2, PCDHGB5, PCDH
PCDHGA8, PCDHGA7, PCDHGA6, PCDHGA5, PCDHGA4, PCDHGB2, PCDHGA3, PCDHGA2, PCDHGB5, PCDHGB4, PCD
PCDHGA8, PCDHGA7, PCDHGA6, PCDHGA5, PCDHGA4, PCDHGB2, PCDHGA3, PCDHGA2, PCDHGB5, PCDHGB4, PCD
PCDHGA8, PCDHGA7, PCDHGA6, PCDHGA5, PCDHGA4, PCDHGB2, PCDHGA3, PCDHGA2, PCDHGB5, PCDHGB4, PCD
COL23A1, DLGAP2, GRIP1, PCDHGA8, PCDHGA7, PCDHGA6, SEZ6L, PCDHGA5, GNG12, PCDHGA4, IL17RD, PCDHG
GRIP1, PCDHGA8, PCDHGA7, PCDHGA6, GNG12, PCDHGA5, PCDHGA4, PCDHGA3, IL17RD, PCDHGA2, PCDHGA1, C
NDST1, FAM69A, GRIP1, PCDHGA8, PCDHGA7, PCDHGA6, PCDHGA5, GNG12, PCDHGA4, AMN, IL17RD, PCDHGA3,
CDH22, SCAMP3, COL23A1, CLGN, NDST1, ATP1A3, SEZ6L, SLC38A1, MPL, AMN, IL17RD, SORCS2, SLC9A1
NDST1, FAM69A, PCDHGA8, PCDHGA7, PCDHGA6, PCDHGA5, AMN, PCDHGA4, PCDHGA3, IL17RD, SORCS2, PCDH
NDST1, FAM69A, PCDHGA8, PCDHGA7, PCDHGA6, PCDHGA5, PCDHGA4, AMN, PCDHGA3, IL17RD, SORCS2, PCDH
NDST1, FAM69A, PCDHGA8, PCDHGA7, PCDHGA6, PCDHGA5, PCDHGA4, AMN, PCDHGA3, IL17RD, SORCS2, PCDH
SCAMP3, COL23A1, NDST1, FAM69A, ATP1A3, SEZ6L, GM7120, AMN, IL17RD, SORCS2, CDH22, CLGN, SLC38A1, M

List Total	Pop Hits	Pop Total	Fold Enrich	Bonferroni	Benjamini	FDR
73	14	17763	156.4256	2.28E-14	2.28E-14	1.44E-13
73	49	17763	44.69304	3.95E-09	1.98E-09	2.43E-08
73	102	17763	23.85576	6.13E-08	2.04E-08	3.76E-07
57	117	13588	20.37487	6.27E-07	6.27E-07	1.52E-06
49	102	9131	18.26931	1.13E-07	1.13E-07	2.14E-06
57	236	13588	12.12132	1.57E-06	7.85E-07	3.81E-06
57	561	13588	5.949026	1.98E-04	6.62E-05	4.82E-04
57	562	13588	5.93844	2.03E-04	5.06E-05	4.91E-04
75	380	17854	7.517474	6.10E-05	6.10E-05	5.20E-04
75	731	17854	4.559161	0.001173	5.87E-04	0.010008
60	840	13288	3.691111	0.011594	0.002911	0.077618
56	510	12504	4.815966	0.008638	0.008638	0.07624
56	528	12504	4.651786	0.011523	0.005778	0.101838
56	596	12504	4.121045	0.030807	0.010376	0.27472
75	1713	17854	2.501436	0.069298	0.023654	0.610603
56	2906	12504	1.76723	0.415783	0.125734	4.615411
75	5507	17854	1.210367	1	0.852291	89.80729
64	2780	16021	1.170599	1	1	99.93034
75	5237	17854	1.090945	1	0.964631	99.81083
56	5709	12504	1.016891	1	0.994813	99.99396
56	5914	12504	0.981642	1	0.998476	99.99964
64	4113	16021	0.91294	1	1	100
