# Supplementary material for: Alternative splicing of helicase-like transcription factor (Hltf): Intron retention-dependent activation of immune tolerance at the feto-maternal interface
Source: PLoS One. 2018 Jul 5;13(7):e0200211. doi: 10.1371/journal.pone.0200211 (PMC6033450; doi:10.1371/journal.pone.0200211)
Supplement: S3 Table — Cuffdiff, a component of Cufflinks, uses RPKM values to calculate changes in gene expression. Cuffdiff data were input into iPathwayGuide. Met-analysis calculated log fold change (logFC) and an adjusted p-value (adjpv) for each comparison. OtA5088-5092 and OtA5093-5097 are designations for five sets of control and Hltf null data, respectively. (PDF) [file pone.0200211.s004.pdf]

S3 Table Differential gene expression analysis.

gene	locus	Controls	Hltf nulls
A2m	chr6:121636172-121679238	OtA5088-OtA5092	OtA5093-OtA5097
Abcd4	chr12:84602530-84617466	OtA5088-OtA5092	OtA5093-OtA5097
Acer2	chr4:86874413-86920881	OtA5088-OtA5092	OtA5093-OtA5097
Aco1	chr4:40143264-40199009	OtA5088-OtA5092	OtA5093-OtA5097
Adam8	chr7:139978940-139992488	OtA5088-OtA5092	OtA5093-OtA5097
Adamts2	chr11:50602084-50804049	OtA5088-OtA5092	OtA5093-OtA5097
Aebp1	chr11:5861865-5878256	OtA5088-OtA5092	OtA5093-OtA5097
Alb	chr5:90460888-90476603	OtA5088-OtA5092	OtA5093-OtA5097
Aldh1b1	chr4:45799021-45804608	OtA5088-OtA5092	OtA5093-OtA5097
Aldob	chr4:49535994-49549483	OtA5088-OtA5092	OtA5093-OtA5097
Aplnr	chr2:85136359-85139923	OtA5088-OtA5092	OtA5093-OtA5097
Arhgef10l	chr4:140514484-140665905	OtA5088-OtA5092	OtA5093-OtA5097
Atrnl1	chr19:57611033-58133340	OtA5088-OtA5092	OtA5093-OtA5097
Atrx	chrX:105797614-105929372	OtA5088-OtA5092	OtA5093-OtA5097
Bend4	chr5:67392146-67427799	OtA5088-OtA5092	OtA5093-OtA5097
Bmper	chr9:23223075-23485202	OtA5088-OtA5092	OtA5093-OtA5097
Cd320	chr17:33843090-33849774	OtA5088-OtA5092	OtA5093-OtA5097
Cds1	chr5:101765129-101823852	OtA5088-OtA5092	OtA5093-OtA5097
Ceacam-ps1	chr7:16649029-16657478	OtA5088-OtA5092	OtA5093-OtA5097
Ceacam15	chr7:16671330-16675705	OtA5088-OtA5092	OtA5093-OtA5097
Cidec	chr6:113424635-113435755	OtA5088-OtA5092	OtA5093-OtA5097
Clca1	chr3:144730315-144760739	OtA5088-OtA5092	OtA5093-OtA5097
Cldn11	chr3:31149919-31164326	OtA5088-OtA5092	OtA5093-OtA5097
Col11a1	chr3:114030539-114220326	OtA5088-OtA5092	OtA5093-OtA5097
Cpm	chr10:117629499-117687352	OtA5088-OtA5092	OtA5093-OtA5097
Cts6	chr13:61195146-61203392	OtA5088-OtA5092	OtA5093-OtA5097
Ctsq	chr13:61035037-61040597	OtA5088-OtA5092	OtA5093-OtA5097
Ctsr	chr13:61159214-61164188	OtA5088-OtA5092	OtA5093-OtA5097
Ctss	chr3:95526785-95556403	OtA5088-OtA5092	OtA5093-OtA5097
Cxcl14	chr13:56288642-56296551	OtA5088-OtA5092	OtA5093-OtA5097
Cxcr7	chr1:90204002-90216288	OtA5088-OtA5092	OtA5093-OtA5097
Ddx3y	chrY:1260714-1286613	OtA5088-OtA5092	OtA5093-OtA5097
Dgkk	chrX:6873483-6948363	OtA5088-OtA5092	OtA5093-OtA5097
Dock11	chrX:35888831-36076562	OtA5088-OtA5092	OtA5093-OtA5097
Doxl2	chr6:48975142-48978745	OtA5088-OtA5092	OtA5093-OtA5097
Efr3b	chr12:3962553-4038915	OtA5088-OtA5092	OtA5093-OtA5097
Eml2	chr7:19181169-19206482	OtA5088-OtA5092	OtA5093-OtA5097
Entpd3	chr9:120539817-120568326	OtA5088-OtA5092	OtA5093-OtA5097
Fam167a	chr14:63436393-63465502	OtA5088-OtA5092	OtA5093-OtA5097
Fam178a	chr19:44931118-44984550	OtA5088-OtA5092	OtA5093-OtA5097
Fam78a	chr2:32066884-32083705	OtA5088-OtA5092	OtA5093-OtA5097
Fga	chr3:83026152-83033617	OtA5088-OtA5092	OtA5093-OtA5097
Fgb	chr3:83042304-83049790	OtA5088-OtA5092	OtA5093-OtA5097
Fgl2	chr5:21292960-21424677	OtA5088-OtA5092	OtA5093-OtA5097
Fnd3c2	chrX:106235245-106255372	OtA5088-OtA5092	OtA5093-OtA5097

Folr1	chr7:101858330-101870788	OtA5088-OtA5092	OtA5093-OtA5097
Foxk1	chr5:142401496-142462015	OtA5088-OtA5092	OtA5093-OtA5097
Fras1	chr5:96373954-96784728	OtA5088-OtA5092	OtA5093-OtA5097
Gcm1	chr9:78051957-78065624	OtA5088-OtA5092	OtA5093-OtA5097
Gpnmb	chr6:49036517-49058182	OtA5088-OtA5092	OtA5093-OtA5097
Gusb	chr5:129989020-130002828	OtA5088-OtA5092	OtA5093-OtA5097
Gzmc	chr14:56231400-56234656	OtA5088-OtA5092	OtA5093-OtA5097
Gzmd	chr14:56129567-56132593	OtA5088-OtA5092	OtA5093-OtA5097
Gzme	chr14:56117618-56120625	OtA5088-OtA5092	OtA5093-OtA5097
Gzmf	chr14:56205262-56211407	OtA5088-OtA5092	OtA5093-OtA5097
Gzmg	chr14:56156580-56159579	OtA5088-OtA5092	OtA5093-OtA5097
Hbb-y	chr7:103851753-103853207	OtA5088-OtA5092	OtA5093-OtA5097
Hipk2	chr6:38697839-38876190	OtA5088-OtA5092	OtA5093-OtA5097
Hist1h2ac	chr13:23681466-23683959	OtA5088-OtA5092	OtA5093-OtA5097
Hist1h2bm	chr13:21722097-21722478	OtA5088-OtA5092	OtA5093-OtA5097
Hist1h3c	chr13:23745041-23745521	OtA5088-OtA5092	OtA5093-OtA5097
Hist1h4m,Hist1h4n	chr13:21811745-21812150	OtA5088-OtA5092	OtA5093-OtA5097
Hivep1	chr13:42052020-42185026	OtA5088-OtA5092	OtA5093-OtA5097
Hltf	chr3:20057810-20118490	OtA5088-OtA5092	OtA5093-OtA5097
Hsd3b1	chr3:98852196-98859794	OtA5088-OtA5092	OtA5093-OtA5097
Igfbp3	chr11:7206090-7213923	OtA5088-OtA5092	OtA5093-OtA5097
Il1r2	chr1:40076211-40125226	OtA5088-OtA5092	OtA5093-OtA5097
Itm2a	chrX:107397194-107403360	OtA5088-OtA5092	OtA5093-OtA5097
Kcnk6	chr7:29221927-29232522	OtA5088-OtA5092	OtA5093-OtA5097
Kcnq1ot1	chr7:143107253-143427042	OtA5088-OtA5092	OtA5093-OtA5097
Klhl30	chr1:91351072-91362404	OtA5088-OtA5092	OtA5093-OtA5097
Krt6a	chr15:101689927-101694305	OtA5088-OtA5092	OtA5093-OtA5097
Lbp	chr2:158306492-158332852	OtA5088-OtA5092	OtA5093-OtA5097
Lipe	chr7:25379526-25566417	OtA5088-OtA5092	OtA5093-OtA5097
Lpin1	chr12:16535668-16589770	OtA5088-OtA5092	OtA5093-OtA5097
Mgp	chr6:136872434-136875805	OtA5088-OtA5092	OtA5093-OtA5097
Mrvi1	chr7:110868266-110982095	OtA5088-OtA5092	OtA5093-OtA5097
Nomo1	chr7:46033695-46084212	OtA5088-OtA5092	OtA5093-OtA5097
Nppb	chr4:147985788-147987205	OtA5088-OtA5092	OtA5093-OtA5097
Npr3	chr15:11839896-11905674	OtA5088-OtA5092	OtA5093-OtA5097
Olfml3	chr3:103735393-103738001	OtA5088-OtA5092	OtA5093-OtA5097
Pcsk6	chr7:65862135-66050386	OtA5088-OtA5092	OtA5093-OtA5097
Pik3r1	chr13:101680761-101768217	OtA5088-OtA5092	OtA5093-OtA5097
Plat	chr8:22757743-22782848	OtA5088-OtA5092	OtA5093-OtA5097
Plbd1	chr6:136612070-136661893	OtA5088-OtA5092	OtA5093-OtA5097
Plcg2	chr8:117498290-117635142	OtA5088-OtA5092	OtA5093-OtA5097
Polr3g	chr13:81673836-81711013	OtA5088-OtA5092	OtA5093-OtA5097
Postn	chr3:54361106-54391041	OtA5088-OtA5092	OtA5093-OtA5097
Prdx6	chr1:161240111-161251166	OtA5088-OtA5092	OtA5093-OtA5097
Prex2	chr1:10993464-11303682	OtA5088-OtA5092	OtA5093-OtA5097
Prf1	chr10:61297835-61304263	OtA5088-OtA5092	OtA5093-OtA5097
Prl4a1	chr13:28016222-28023546	OtA5088-OtA5092	OtA5093-OtA5097

Prl5a1	chr13:28142483-28151595	OtA5088-OtA5092	OtA5093-OtA5097
Scn5a	chr9:119483407-119579016	OtA5088-OtA5092	OtA5093-OtA5097
Sema4b	chr7:80186840-80226524	OtA5088-OtA5092	OtA5093-OtA5097
Sgk2	chr2:162987480-163014127	OtA5088-OtA5092	OtA5093-OtA5097
Sh3tc1	chr5:35697179-35729276	OtA5088-OtA5092	OtA5093-OtA5097
Sh3tc2	chr18:61953074-62015719	OtA5088-OtA5092	OtA5093-OtA5097
Skil	chr3:31095060-31122577	OtA5088-OtA5092	OtA5093-OtA5097
Slc22a18	chr7:143473754-143499332	OtA5088-OtA5092	OtA5093-OtA5097
Slc23a3	chr1:75125541-75133890	OtA5088-OtA5092	OtA5093-OtA5097
Slc26a7	chr4:14505196-14621778	OtA5088-OtA5092	OtA5093-OtA5097
Slc35f2	chr9:53771534-53818161	OtA5088-OtA5092	OtA5093-OtA5097
Slc39a5	chr10:128395930-128401224	OtA5088-OtA5092	OtA5093-OtA5097
Slc4a1	chr11:102348819-102365281	OtA5088-OtA5092	OtA5093-OtA5097
Slc52a3	chr2:151996510-152009258	OtA5088-OtA5092	OtA5093-OtA5097
Slc6a13	chr6:121300295-121337718	OtA5088-OtA5092	OtA5093-OtA5097
Slc7a5	chr8:121881145-121907686	OtA5088-OtA5092	OtA5093-OtA5097
Snora74a	chr18:35553409-35558316	OtA5088-OtA5092	OtA5093-OtA5097
Sox11	chr12:27334267-27342718	OtA5088-OtA5092	OtA5093-OtA5097
Spp1	chr5:104435110-104441053	OtA5088-OtA5092	OtA5093-OtA5097
Syna	chr5:134557253-134560171	OtA5088-OtA5092	OtA5093-OtA5097
Syp	chrX:7638579-7653256	OtA5088-OtA5092	OtA5093-OtA5097
Tcf20	chr15:82808625-82912134	OtA5088-OtA5092	OtA5093-OtA5097
Tfrc	chr16:32608895-32632794	OtA5088-OtA5092	OtA5093-OtA5097
Timp3	chr10:86048745-86498896	OtA5088-OtA5092	OtA5093-OtA5097
Tmem100	chr11:90030347-90036505	OtA5088-OtA5092	OtA5093-OtA5097
Tph1	chr7:46647130-46672537	OtA5088-OtA5092	OtA5093-OtA5097
Tppp	chr13:74009418-74035753	OtA5088-OtA5092	OtA5093-OtA5097
Trpv2	chr11:62574485-62600305	OtA5088-OtA5092	OtA5093-OtA5097
Tst	chr15:78399555-78405859	OtA5088-OtA5092	OtA5093-OtA5097
Tubb4a	chr17:57080065-57087782	OtA5088-OtA5092	OtA5093-OtA5097
Ubtd1	chr19:41981762-42034641	OtA5088-OtA5092	OtA5093-OtA5097
Unc13d	chr11:116062095-116077961	OtA5088-OtA5092	OtA5093-OtA5097
Vdr	chr15:97854426-97908296	OtA5088-OtA5092	OtA5093-OtA5097
Vnn1	chr10:23894687-23905343	OtA5088-OtA5092	OtA5093-OtA5097
Xist	chrX:103431516-103484957	OtA5088-OtA5092	OtA5093-OtA5097
Zdbf2	chr1:63273268-63314575	OtA5088-OtA5092	OtA5093-OtA5097
Car4	chr11:84957753-84966054	OtA5088-OtA5092	OtA5093-OtA5097
Cdc34	chr10:79682194-79688398	OtA5088-OtA5092	OtA5093-OtA5097
Col6a3	chr1:90766859-90843971	OtA5088-OtA5092	OtA5093-OtA5097
Emr1	chr17:57358685-57483529	OtA5088-OtA5092	OtA5093-OtA5097
Entpd2	chr2:25395873-25401323	OtA5088-OtA5092	OtA5093-OtA5097
Fmr1	chrX:68678554-68717961	OtA5088-OtA5092	OtA5093-OtA5097
Hapln4	chr8:70083528-70090862	OtA5088-OtA5092	OtA5093-OtA5097
Hist1h1b	chr13:21779882-21780554	OtA5088-OtA5092	OtA5093-OtA5097
Homer2	chr7:81600480-81706925	OtA5088-OtA5092	OtA5093-OtA5097
Ly6g6c	chr17:35067324-35070048	OtA5088-OtA5092	OtA5093-OtA5097
Maoa	chrX:16619697-16687812	OtA5088-OtA5092	OtA5093-OtA5097

Matn2	chr15:34306680-34436240	OtA5088-OtA5092	OtA5093-OtA5097
Nktr	chr9:121719180-121759943	OtA5088-OtA5092	OtA5093-OtA5097
Papln	chr12:83763633-83792384	OtA5088-OtA5092	OtA5093-OtA5097
Plvap	chr8:71497752-71511769	OtA5088-OtA5092	OtA5093-OtA5097
Podxl	chr6:31519492-31563937	OtA5088-OtA5092	OtA5093-OtA5097
Slco4a1	chr2:180460977-180474853	OtA5088-OtA5092	OtA5093-OtA5097
Stard8	chrX:99042580-99074728	OtA5088-OtA5092	OtA5093-OtA5097
Tspan18	chr2:93201759-93334487	OtA5088-OtA5092	OtA5093-OtA5097
Gdf10	chr14:33923586-33935282	OtA5088-OtA5092	OtA5093-OtA5097
Lrrn4	chr2:132868515-132880862	OtA5088-OtA5092	OtA5093-OtA5097
Ltf	chr9:111019291-111042766	OtA5088-OtA5092	OtA5093-OtA5097
Lyz2	chr10:117277540-117282272	OtA5088-OtA5092	OtA5093-OtA5097
Mt1	chr8:94179088-94180327	OtA5088-OtA5092	OtA5093-OtA5097
Phf3	chr1:30802341-30863256	OtA5088-OtA5092	OtA5093-OtA5097
Prdm2	chr4:143107390-143212709	OtA5088-OtA5092	OtA5093-OtA5097
Scnn1b	chr7:121865110-121918516	OtA5088-OtA5092	OtA5093-OtA5097
Snap91	chr9:86765935-86880372	OtA5088-OtA5092	OtA5093-OtA5097
Sos1	chr17:80393751-80480453	OtA5088-OtA5092	OtA5093-OtA5097
Bach2	chr4:32417434-32586108	OtA5088-OtA5092	OtA5093-OtA5097
Fmo1	chr1:162829560-162866548	OtA5088-OtA5092	OtA5093-OtA5097
Gcgr	chr11:120530726-120538984	OtA5088-OtA5092	OtA5093-OtA5097
Gzmb	chr14:56258857-56262260	OtA5088-OtA5092	OtA5093-OtA5097
Htr1d	chr4:136423523-136444402	OtA5088-OtA5092	OtA5093-OtA5097
Lrp8	chr4:107802258-107876840	OtA5088-OtA5092	OtA5093-OtA5097
Plxna2	chr1:194619828-194816868	OtA5088-OtA5092	OtA5093-OtA5097
Slc45a3	chr1:131962966-131982964	OtA5088-OtA5092	OtA5093-OtA5097
Tex13	chrX:140808306-140813433	OtA5088-OtA5092	OtA5093-OtA5097
Ccnjl	chr11:43528783-43586997	OtA5088-OtA5092	OtA5093-OtA5097
Golgb1	chr16:36880518-36933085	OtA5088-OtA5092	OtA5093-OtA5097
Gpbp1	chr13:111425679-111490041	OtA5088-OtA5092	OtA5093-OtA5097
Lepr	chr4:101717406-101815354	OtA5088-OtA5092	OtA5093-OtA5097
Mtss1l	chr8:110721483-110741400	OtA5088-OtA5092	OtA5093-OtA5097
Myh10	chr11:68691914-68816624	OtA5088-OtA5092	OtA5093-OtA5097
Senp3	chr11:69673109-69682084	OtA5088-OtA5092	OtA5093-OtA5097
Sftpd	chr14:41172211-41185198	OtA5088-OtA5092	OtA5093-OtA5097
Slc12a7	chr13:73763696-73816742	OtA5088-OtA5092	OtA5093-OtA5097
Slc30a3	chr5:31086105-31093527	OtA5088-OtA5092	OtA5093-OtA5097
Svep1	chr4:58042795-58206596	OtA5088-OtA5092	OtA5093-OtA5097
Tro	chrX:150645302-150657436	OtA5088-OtA5092	OtA5093-OtA5097
Arhgap21	chr2:20847918-20967721	OtA5088-OtA5092	OtA5093-OtA5097
Arhgap29	chr3:121953325-122016153	OtA5088-OtA5092	OtA5093-OtA5097
Scn9a	chr2:66440879-66634952	OtA5088-OtA5092	OtA5093-OtA5097
Slc2a3	chr6:122727808-122742745	OtA5088-OtA5092	OtA5093-OtA5097
Uty	chrY:1097143-1245738	OtA5088-OtA5092	OtA5093-OtA5097
Car2	chr3:14886425-14900770	OtA5088-OtA5092	OtA5093-OtA5097
Cep350	chr1:155844963-155973255	OtA5088-OtA5092	OtA5093-OtA5097
Fam83g	chr11:61672781-61720799	OtA5088-OtA5092	OtA5093-OtA5097

Gm7694	chr1:170298192-170306332	OtA5088-OtA5092	OtA5093-OtA5097
Mpeg1	chr19:12460778-12465285	OtA5088-OtA5092	OtA5093-OtA5097
Mum1l1	chrX:139210043-139238335	OtA5088-OtA5092	OtA5093-OtA5097
Nid2	chr14:19751256-19823823	OtA5088-OtA5092	OtA5093-OtA5097
Oas3	chr5:120753097-120777659	OtA5088-OtA5092	OtA5093-OtA5097
Pepd	chr7:34912406-35044708	OtA5088-OtA5092	OtA5093-OtA5097
Pex5l	chr3:32949630-33143082	OtA5088-OtA5092	OtA5093-OtA5097
Sema4d	chr13:51701247-51793644	OtA5088-OtA5092	OtA5093-OtA5097
Smpd1	chr7:105554359-105558389	OtA5088-OtA5092	OtA5093-OtA5097
Wnt10b	chr15:98771751-98778150	OtA5088-OtA5092	OtA5093-OtA5097
Adam23	chr1:63445903-63596515	OtA5088-OtA5092	OtA5093-OtA5097
Ctsj	chr13:61000277-61005911	OtA5088-OtA5092	OtA5093-OtA5097
Foxp1	chr6:98925341-99435345	OtA5088-OtA5092	OtA5093-OtA5097
Padi2	chr4:140906359-140957902	OtA5088-OtA5092	OtA5093-OtA5097
Penk	chr4:4133535-4138445	OtA5088-OtA5092	OtA5093-OtA5097
Psd3	chr8:67689081-67974574	OtA5088-OtA5092	OtA5093-OtA5097
Ptger3	chr3:157566891-157644758	OtA5088-OtA5092	OtA5093-OtA5097
Snx8	chr5:140340302-140389247	OtA5088-OtA5092	OtA5093-OtA5097
Aim1l	chr4:134068451-134092504	OtA5088-OtA5092	OtA5093-OtA5097
Hba-x	chr11:32276599-32278115	OtA5088-OtA5092	OtA5093-OtA5097
Stab1	chr14:31134852-31168641	OtA5088-OtA5092	OtA5093-OtA5097
Tgfbi	chr13:56609602-56639339	OtA5088-OtA5092	OtA5093-OtA5097
Baz2b	chr2:59899362-60125740	OtA5088-OtA5092	OtA5093-OtA5097
Glp1r	chr17:30901866-30936510	OtA5088-OtA5092	OtA5093-OtA5097
Meg3	chr12:109540995-109571729	OtA5088-OtA5092	OtA5093-OtA5097
Mll1	chr9:44803354-44881274	OtA5088-OtA5092	OtA5093-OtA5097
Mmp12	chr9:7347373-7360461	OtA5088-OtA5092	OtA5093-OtA5097
Pde4b	chr4:102254741-102607262	OtA5088-OtA5092	OtA5093-OtA5097
Sez6l	chr5:112419150-112577198	OtA5088-OtA5092	OtA5093-OtA5097
Braf	chr6:39603236-39725463	OtA5088-OtA5092	OtA5093-OtA5097
Chd7	chr4:8690920-8866809	OtA5088-OtA5092	OtA5093-OtA5097
Cthrc1	chr15:39076931-39087119	OtA5088-OtA5092	OtA5093-OtA5097
Cttnbp2nl	chr3:105001914-105053146	OtA5088-OtA5092	OtA5093-OtA5097
Itgb2	chr10:77530347-77565674	OtA5088-OtA5092	OtA5093-OtA5097
Myo1f	chr17:33555706-33607764	OtA5088-OtA5092	OtA5093-OtA5097
Phf21a	chr2:92184181-92364666	OtA5088-OtA5092	OtA5093-OtA5097
Prl3d1	chr13:27094188-27100260	OtA5088-OtA5092	OtA5093-OtA5097
Slc2a2	chr3:28697902-28728360	OtA5088-OtA5092	OtA5093-OtA5097
Smpdl3b	chr4:132732965-132757171	OtA5088-OtA5092	OtA5093-OtA5097
Spon1	chr7:113765997-114043375	OtA5088-OtA5092	OtA5093-OtA5097
Arhgef16	chr4:154278469-154299895	OtA5088-OtA5092	OtA5093-OtA5097
Col5a3	chr9:20770049-20815034	OtA5088-OtA5092	OtA5093-OtA5097
Eea1	chr10:95940662-96045518	OtA5088-OtA5092	OtA5093-OtA5097
Ephx2	chr14:66084376-66124500	OtA5088-OtA5092	OtA5093-OtA5097
Fabp5	chr3:10012605-10016610	OtA5088-OtA5092	OtA5093-OtA5097
Igdcc3	chr9:65141188-65185870	OtA5088-OtA5092	OtA5093-OtA5097
Slco2b1	chr7:99657803-99711340	OtA5088-OtA5092	OtA5093-OtA5097

Abhd3	chr18:10644410-10706696	OtA5088-OtA5092	OtA5093-OtA5097
Cdk19	chr10:40349307-40483818	OtA5088-OtA5092	OtA5093-OtA5097
Hist1h2bh	chr13:23542971-23543352	OtA5088-OtA5092	OtA5093-OtA5097
Mkln1	chr6:31398827-31509477	OtA5088-OtA5092	OtA5093-OtA5097
Rdx	chr9:52002060-52088738	OtA5088-OtA5092	OtA5093-OtA5097
S100a16	chr3:90541222-90543151	OtA5088-OtA5092	OtA5093-OtA5097
Selenbp1	chr3:94933082-94944758	OtA5088-OtA5092	OtA5093-OtA5097
Hist1h2ak	chr13:21753434-21753827	OtA5088-OtA5092	OtA5093-OtA5097
Kirrel3	chr9:34488730-35036307	OtA5088-OtA5092	OtA5093-OtA5097
Nfat5	chr8:107293469-107379517	OtA5088-OtA5092	OtA5093-OtA5097
Nnt	chr13:119334316-119409138	OtA5088-OtA5092	OtA5093-OtA5097
Slc29a4	chr5:142702100-142722490	OtA5088-OtA5092	OtA5093-OtA5097
Slc9a2	chr1:40681711-40768885	OtA5088-OtA5092	OtA5093-OtA5097
Cmtm7	chr9:114756835-114781993	OtA5088-OtA5092	OtA5093-OtA5097
Fndc3c1	chrX:106420042-106485229	OtA5088-OtA5092	OtA5093-OtA5097
Maob	chrX:16709280-16817366	OtA5088-OtA5092	OtA5093-OtA5097
Rock2	chr12:16894977-16988274	OtA5088-OtA5092	OtA5093-OtA5097
Tmem59l	chr8:70483866-70487358	OtA5088-OtA5092	OtA5093-OtA5097
Ccnd1	chr7:144929930-144939925	OtA5088-OtA5092	OtA5093-OtA5097
Lpin2	chr17:71183977-71249818	OtA5088-OtA5092	OtA5093-OtA5097
Slc39a10	chr1:46807543-46853509	OtA5088-OtA5092	OtA5093-OtA5097
Ube2h	chr6:30211289-30304539	OtA5088-OtA5092	OtA5093-OtA5097
Tcf23	chr5:30968676-30977018	OtA5088-OtA5092	OtA5093-OtA5097
Zfhx3	chr8:108714643-108961636	OtA5088-OtA5092	OtA5093-OtA5097
Diap2	chrX:129749741-130465833	OtA5088-OtA5092	OtA5093-OtA5097
Hist1h2bb	chr13:23746733-23747201	OtA5088-OtA5092	OtA5093-OtA5097
Klk15	chr7:43933770-43939590	OtA5088-OtA5092	OtA5093-OtA5097
Phgdh	chr3:98313170-98339969	OtA5088-OtA5092	OtA5093-OtA5097
Sorl1	chr9:41968488-42124289	OtA5088-OtA5092	OtA5093-OtA5097
Arap2	chr5:62602445-62766177	OtA5088-OtA5092	OtA5093-OtA5097
Col4a5	chrX:141475418-141689235	OtA5088-OtA5092	OtA5093-OtA5097
Cyp39a1	chr17:43667424-43751431	OtA5088-OtA5092	OtA5093-OtA5097
Ddx46	chr13:55635026-55681256	OtA5088-OtA5092	OtA5093-OtA5097
Gm5424	chr2:31470269-31520670	OtA5088-OtA5092	OtA5093-OtA5097
Id2	chr12:25093798-25096092	OtA5088-OtA5092	OtA5093-OtA5097
Itga5	chr15:103344285-103366748	OtA5088-OtA5092	OtA5093-OtA5097
Pkp4	chr2:59160849-59355205	OtA5088-OtA5092	OtA5093-OtA5097
Rian	chr12:109603944-109661711	OtA5088-OtA5092	OtA5093-OtA5097
Snora78	chr17:24719530-24719965	OtA5088-OtA5092	OtA5093-OtA5097
Stab2	chr10:86841209-87007942	OtA5088-OtA5092	OtA5093-OtA5097
Tfeb	chr17:47737036-47792416	OtA5088-OtA5092	OtA5093-OtA5097
Zfp609	chr9:65691582-65827564	OtA5088-OtA5092	OtA5093-OtA5097
C4b	chr17:34728380-34743897	OtA5088-OtA5092	OtA5093-OtA5097
Gcap14	chr14:36874935-36968764	OtA5088-OtA5092	OtA5093-OtA5097
Gclc	chr9:77754534-77794489	OtA5088-OtA5092	OtA5093-OtA5097
Hpn	chr7:31098724-31115290	OtA5088-OtA5092	OtA5093-OtA5097
Nrcam	chr12:44328884-44601846	OtA5088-OtA5092	OtA5093-OtA5097

Prl7b1	chr13:27601818-27610582	OtA5088-OtA5092	OtA5093-OtA5097
Prrg4	chr2:104830740-104849850	OtA5088-OtA5092	OtA5093-OtA5097
Dtx1	chr5:120680263-120711669	OtA5088-OtA5092	OtA5093-OtA5097
Rgs4	chr1:169741476-169747642	OtA5088-OtA5092	OtA5093-OtA5097
Sh3bgrl2	chr9:83548337-83600291	OtA5088-OtA5092	OtA5093-OtA5097
Usp6nl	chr2:6322756-6443820	OtA5088-OtA5092	OtA5093-OtA5097
Zcchc11	chr4:108459425-108559415	OtA5088-OtA5092	OtA5093-OtA5097
Adcy1	chr11:7063488-7178505	OtA5088-OtA5092	OtA5093-OtA5097
Cldn5	chr16:18776846-18778262	OtA5088-OtA5092	OtA5093-OtA5097
Eif4ebp3	chr18:36664059-36666324	OtA5088-OtA5092	OtA5093-OtA5097
Prl2c1	chr13:27849401-27857708	OtA5088-OtA5092	OtA5093-OtA5097
Serping1	chr2:84765359-84775429	OtA5088-OtA5092	OtA5093-OtA5097
Slc16a3	chr11:120948483-120959000	OtA5088-OtA5092	OtA5093-OtA5097
Dst	chr1:34011824-34308652	OtA5088-OtA5092	OtA5093-OtA5097
Ktn1	chr14:47663755-47736564	OtA5088-OtA5092	OtA5093-OtA5097
Osta	chr16:32475577-32487879	OtA5088-OtA5092	OtA5093-OtA5097
Pla1a	chr16:38396116-38433145	OtA5088-OtA5092	OtA5093-OtA5097
Gm7120	chr13:119487256-119610459	OtA5088-OtA5092	OtA5093-OtA5097
Mll3	chr5:25271793-25498783	OtA5088-OtA5092	OtA5093-OtA5097
Zfp462	chr4:54947944-55083563	OtA5088-OtA5092	OtA5093-OtA5097
Abcb8	chr5:24394155-24409947	OtA5088-OtA5092	OtA5093-OtA5097
Aldh1a3	chr7:66390892-66427477	OtA5088-OtA5092	OtA5093-OtA5097
BC021891	chr8:125910449-125947439	OtA5088-OtA5092	OtA5093-OtA5097
Fam83f	chr15:80671846-80700425	OtA5088-OtA5092	OtA5093-OtA5097
Lonrf3	chrX:36328408-36366856	OtA5088-OtA5092	OtA5093-OtA5097
St8sia5	chr18:77185846-77255450	OtA5088-OtA5092	OtA5093-OtA5097
Akt3	chr1:177022114-177248767	OtA5088-OtA5092	OtA5093-OtA5097
Apoa1bp	chr3:88056522-88058495	OtA5088-OtA5092	OtA5093-OtA5097
Prrg3	chrX:71963020-71972728	OtA5088-OtA5092	OtA5093-OtA5097
Stk38l	chr6:146724929-146778814	OtA5088-OtA5092	OtA5093-OtA5097
Fam84b	chr15:60818995-60831400	OtA5088-OtA5092	OtA5093-OtA5097
Mical1	chr10:41476313-41487030	OtA5088-OtA5092	OtA5093-OtA5097
Mtch1	chr17:29332075-29347904	OtA5088-OtA5092	OtA5093-OtA5097
Smcr7	chr11:60728397-60732951	OtA5088-OtA5092	OtA5093-OtA5097
Fam101a	chr5:125003474-125012547	OtA5088-OtA5092	OtA5093-OtA5097
Fgfrl1	chr5:108694228-108706950	OtA5088-OtA5092	OtA5093-OtA5097
Phactr2	chr10:13207716-13474396	OtA5088-OtA5092	OtA5093-OtA5097
Sfrs18	chr4:21846326-21876475	OtA5088-OtA5092	OtA5093-OtA5097
Cpxm1	chr2:130390774-130397629	OtA5088-OtA5092	OtA5093-OtA5097
Fkbp5	chr17:28399094-28486112	OtA5088-OtA5092	OtA5093-OtA5097
Nyx	chrX:13467671-13489313	OtA5088-OtA5092	OtA5093-OtA5097
Sirpa	chr2:129592838-129632228	OtA5088-OtA5092	OtA5093-OtA5097
Slc1a1	chr19:28835165-28913960	OtA5088-OtA5092	OtA5093-OtA5097
Vamp8	chr6:72385220-72390667	OtA5088-OtA5092	OtA5093-OtA5097
Gpr56	chr8:94977108-95014208	OtA5088-OtA5092	OtA5093-OtA5097
Psd4	chr2:24385396-24408729	OtA5088-OtA5092	OtA5093-OtA5097
Rtl1	chr12:109590168-109595403	OtA5088-OtA5092	OtA5093-OtA5097

Ttr	chr18:20665249-20674326	OtA5088-OtA5092	OtA5093-OtA5097
Ppp1r3g	chr13:35967905-35970388	OtA5088-OtA5092	OtA5093-OtA5097
Scaf11	chr15:96411697-96460843	OtA5088-OtA5092	OtA5093-OtA5097
Acnat1	chr4:49443531-49451109	OtA5088-OtA5092	OtA5093-OtA5097
Erv3	chr2:131853677-131859747	OtA5088-OtA5092	OtA5093-OtA5097
Il4ra	chr7:125552281-125579474	OtA5088-OtA5092	OtA5093-OtA5097
Slc5a5	chr8:70882888-70892757	OtA5088-OtA5092	OtA5093-OtA5097
Spen	chr4:141444672-141538597	OtA5088-OtA5092	OtA5093-OtA5097
Atp5o	chr16:91925222-91931630	OtA5088-OtA5092	OtA5093-OtA5097
Hist1h1a	chr13:23763667-23764414	OtA5088-OtA5092	OtA5093-OtA5097
Pdzk1	chr3:96829825-96870926	OtA5088-OtA5092	OtA5093-OtA5097
S1pr2	chr9:20965951-20976793	OtA5088-OtA5092	OtA5093-OtA5097
Acss1	chr2:150618110-150668932	OtA5088-OtA5092	OtA5093-OtA5097
Cramp1l	chr17:24961225-25015230	OtA5088-OtA5092	OtA5093-OtA5097
Slc25a25	chr2:32414486-32455476	OtA5088-OtA5092	OtA5093-OtA5097
Slc29a2	chr19:5024005-5031972	OtA5088-OtA5092	OtA5093-OtA5097
Slc4a2	chr5:24425231-24440947	OtA5088-OtA5092	OtA5093-OtA5097
Susd2	chr10:75636618-75644008	OtA5088-OtA5092	OtA5093-OtA5097
Apc	chr18:34207774-34322190	OtA5088-OtA5092	OtA5093-OtA5097
Brd3	chr2:27445580-27475673	OtA5088-OtA5092	OtA5093-OtA5097
Itpka	chr2:119742336-119751253	OtA5088-OtA5092	OtA5093-OtA5097
Mxi1	chr19:53310505-53375810	OtA5088-OtA5092	OtA5093-OtA5097
Rps6ka6	chrX:111388191-111537945	OtA5088-OtA5092	OtA5093-OtA5097
Capn11	chr17:45630203-45659309	OtA5088-OtA5092	OtA5093-OtA5097
Fndc5	chr4:129137059-129144593	OtA5088-OtA5092	OtA5093-OtA5097
Pam	chr1:97821093-98095632	OtA5088-OtA5092	OtA5093-OtA5097
Gbp5	chr3:142496933-142522344	OtA5088-OtA5092	OtA5093-OtA5097
Myl4	chr11:104550662-104587219	OtA5088-OtA5092	OtA5093-OtA5097
Slc22a4	chr11:53983125-54028090	OtA5088-OtA5092	OtA5093-OtA5097
Gjb2	chr14:57098601-57104702	OtA5088-OtA5092	OtA5093-OtA5097
Mgat3	chr15:80173720-80215519	OtA5088-OtA5092	OtA5093-OtA5097
Cebpb	chr2:167688914-167690418	OtA5088-OtA5092	OtA5093-OtA5097
Chpf2	chr5:24586749-24592486	OtA5088-OtA5092	OtA5093-OtA5097
Fnbp1l	chr3:122538718-122619667	OtA5088-OtA5092	OtA5093-OtA5097
Hist1h2bg	chr13:23571399-23572014	OtA5088-OtA5092	OtA5093-OtA5097
Idh2	chr7:80094846-80115350	OtA5088-OtA5092	OtA5093-OtA5097
Kif13a	chr13:46749087-46929718	OtA5088-OtA5092	OtA5093-OtA5097
Lpl	chr8:68880554-68906932	OtA5088-OtA5092	OtA5093-OtA5097
Rock1	chr18:10064400-10181792	OtA5088-OtA5092	OtA5093-OtA5097
Igfbp1	chr11:7197786-7202546	OtA5088-OtA5092	OtA5093-OtA5097
Klk10	chr7:43781053-43785410	OtA5088-OtA5092	OtA5093-OtA5097
Ncoa2	chr1:13139158-13374083	OtA5088-OtA5092	OtA5093-OtA5097
Odz4	chr7:96210636-96908554	OtA5088-OtA5092	OtA5093-OtA5097
Slc16a1	chr3:104638667-104658462	OtA5088-OtA5092	OtA5093-OtA5097
Tmem159	chr7:120102425-120120986	OtA5088-OtA5092	OtA5093-OtA5097
Arfgef1	chr1:10137506-10232670	OtA5088-OtA5092	OtA5093-OtA5097
Plin2	chr4:86656564-86670059	OtA5088-OtA5092	OtA5093-OtA5097

Srgn	chr10:62494427-62507755	OtA5088-OtA5092	OtA5093-OtA5097
Wfdc2	chr2:164562715-164568506	OtA5088-OtA5092	OtA5093-OtA5097
Rsf1	chr7:97579895-97692782	OtA5088-OtA5092	OtA5093-OtA5097
Scel	chr14:103513340-103613346	OtA5088-OtA5092	OtA5093-OtA5097
Zranb2	chr3:157534396-157548339	OtA5088-OtA5092	OtA5093-OtA5097
Mgll	chr6:88724411-88828360	OtA5088-OtA5092	OtA5093-OtA5097
Sec62	chr3:30792875-30821263	OtA5088-OtA5092	OtA5093-OtA5097
Slc41a2	chr10:83231138-83337817	OtA5088-OtA5092	OtA5093-OtA5097
Smc5	chr19:23206440-23273897	OtA5088-OtA5092	OtA5093-OtA5097
Tex264	chr9:106658745-106685948	OtA5088-OtA5092	OtA5093-OtA5097
Abca5	chr11:110269368-110337716	OtA5088-OtA5092	OtA5093-OtA5097
Atp2b1	chr10:98915151-99026143	OtA5088-OtA5092	OtA5093-OtA5097
Coro1a	chr7:126699773-126704754	OtA5088-OtA5092	OtA5093-OtA5097
Serpina1a	chr12:103853294-103863619	OtA5088-OtA5092	OtA5093-OtA5097
Utrn	chr10:12382187-12861735	OtA5088-OtA5092	OtA5093-OtA5097
Acvrl1	chr15:101128536-101145336	OtA5088-OtA5092	OtA5093-OtA5097
Gja4	chr4:127311420-127313978	OtA5088-OtA5092	OtA5093-OtA5097
Gpr146	chr5:139359738-139460534	OtA5088-OtA5092	OtA5093-OtA5097
Htra3	chr5:35652032-35679782	OtA5088-OtA5092	OtA5093-OtA5097
Cyth4	chr15:78597046-78622019	OtA5088-OtA5092	OtA5093-OtA5097
Hhipl1	chr12:108306269-108328300	OtA5088-OtA5092	OtA5093-OtA5097
Bscl2	chr19:8836928-8848683	OtA5088-OtA5092	OtA5093-OtA5097
Maml2	chr9:13619988-13709533	OtA5088-OtA5092	OtA5093-OtA5097
Serinc5	chr13:92611137-92711946	OtA5088-OtA5092	OtA5093-OtA5097
Stra6	chr9:58129087-58153997	OtA5088-OtA5092	OtA5093-OtA5097
Pcp4l1	chr1:171173263-171196268	OtA5088-OtA5092	OtA5093-OtA5097
Slc39a14	chr14:70303466-70351424	OtA5088-OtA5092	OtA5093-OtA5097
Fcgr1	chr3:96282908-96293969	OtA5088-OtA5092	OtA5093-OtA5097
Hmox1	chr8:75093617-75100593	OtA5088-OtA5092	OtA5093-OtA5097
Ms4a7	chr19:11321405-11336116	OtA5088-OtA5092	OtA5093-OtA5097
Zfp871	chr17:32765496-32788287	OtA5088-OtA5092	OtA5093-OtA5097
Gm608	chr16:44173396-44227466	OtA5088-OtA5092	OtA5093-OtA5097
Faah	chr4:115996655-116017902	OtA5088-OtA5092	OtA5093-OtA5097
Hist1h4b	chr13:23757020-23757332	OtA5088-OtA5092	OtA5093-OtA5097
Sox9	chr11:112782209-112787757	OtA5088-OtA5092	OtA5093-OtA5097
Zfp652	chr11:95749066-95764713	OtA5088-OtA5092	OtA5093-OtA5097
Arhgap5	chr12:52516076-52567851	OtA5088-OtA5092	OtA5093-OtA5097
Muc13	chr16:33794036-33819927	OtA5088-OtA5092	OtA5093-OtA5097
Steap4	chr5:7960471-7982213	OtA5088-OtA5092	OtA5093-OtA5097
Adsl	chr15:80948518-80969373	OtA5088-OtA5092	OtA5093-OtA5097
Pcyox1	chr6:86386005-86397150	OtA5088-OtA5092	OtA5093-OtA5097
Ccbe1	chr18:66056855-66291838	OtA5088-OtA5092	OtA5093-OtA5097
Cdkn1c	chr7:143458338-143461050	OtA5088-OtA5092	OtA5093-OtA5097
Gfra3	chr18:34689902-34720387	OtA5088-OtA5092	OtA5093-OtA5097
N6amt2	chr14:57549597-57571569	OtA5088-OtA5092	OtA5093-OtA5097
Ptprf	chr4:118208212-118291397	OtA5088-OtA5092	OtA5093-OtA5097
Blnk	chr19:40928926-40994535	OtA5088-OtA5092	OtA5093-OtA5097

Col23a1	chr11:51289919-51583925	OtA5088-OtA5092	OtA5093-OtA5097
Igf2	chr7:142650767-142670356	OtA5088-OtA5092	OtA5093-OtA5097
Slc11a2	chr15:100387899-100423055	OtA5088-OtA5092	OtA5093-OtA5097
Atp5g3	chr2:73908449-73911294	OtA5088-OtA5092	OtA5093-OtA5097
Mdm4	chr1:132989844-133025348	OtA5088-OtA5092	OtA5093-OtA5097
Appl1	chr14:26918987-26970551	OtA5088-OtA5092	OtA5093-OtA5097
Car5b	chrX:163976821-164028010	OtA5088-OtA5092	OtA5093-OtA5097
Endou	chr15:97711018-97731405	OtA5088-OtA5092	OtA5093-OtA5097
Abhd16a	chr17:35089290-35102987	OtA5088-OtA5092	OtA5093-OtA5097
Acot2	chr12:83987860-83993875	OtA5088-OtA5092	OtA5093-OtA5097
Cep110	chr2:35109491-35178822	OtA5088-OtA5092	OtA5093-OtA5097
Jmjd1c	chr10:67127257-67256326	OtA5088-OtA5092	OtA5093-OtA5097
Serpinb9g	chr13:33484789-33496000	OtA5088-OtA5092	OtA5093-OtA5097
Rrbp1	chr2:143947394-144011263	OtA5088-OtA5092	OtA5093-OtA5097
Tnrc6b	chr15:80711312-80941086	OtA5088-OtA5092	OtA5093-OtA5097
Dll1	chr17:15367353-15375823	OtA5088-OtA5092	OtA5093-OtA5097
BC032203	chr17:46798115-46831413	OtA5088-OtA5092	OtA5093-OtA5097
Klf13	chr7:63886350-63938915	OtA5088-OtA5092	OtA5093-OtA5097
Pigs	chr11:78328421-78342776	OtA5088-OtA5092	OtA5093-OtA5097
Prl7a2	chr13:27658583-27668036	OtA5088-OtA5092	OtA5093-OtA5097
Mrgprg	chr7:143763709-143766993	OtA5088-OtA5092	OtA5093-OtA5097
Paqr7	chr4:134496977-134508810	OtA5088-OtA5092	OtA5093-OtA5097
Zmym6	chr4:127077382-127124294	OtA5088-OtA5092	OtA5093-OtA5097
Abcc1	chr16:14361557-14474878	OtA5088-OtA5092	OtA5093-OtA5097
Ankrd50	chr3:38449260-38484816	OtA5088-OtA5092	OtA5093-OtA5097
Qser1	chr2:104754796-104816696	OtA5088-OtA5092	OtA5093-OtA5097
Rab32	chr10:10545038-10558207	OtA5088-OtA5092	OtA5093-OtA5097
Casp8ap2	chr4:32615472-32653265	OtA5088-OtA5092	OtA5093-OtA5097
Nipbl	chr15:8291224-8444463	OtA5088-OtA5092	OtA5093-OtA5097
Olr1	chr6:129485246-129507165	OtA5088-OtA5092	OtA5093-OtA5097
Rbm25	chr12:83632233-83683123	OtA5088-OtA5092	OtA5093-OtA5097
Dock5	chr14:67751927-67933572	OtA5088-OtA5092	OtA5093-OtA5097
Gm12824	chr4:114406723-114615098	OtA5088-OtA5092	OtA5093-OtA5097
Kcnk5	chr14:20140057-20181782	OtA5088-OtA5092	OtA5093-OtA5097
Ace2	chrX:164139341-164188418	OtA5088-OtA5092	OtA5093-OtA5097
Prdx2	chr8:84969647-84974313	OtA5088-OtA5092	OtA5093-OtA5097
Serpina5	chr12:104101112-104106137	OtA5088-OtA5092	OtA5093-OtA5097
Zfp869	chr8:69705136-69716902	OtA5088-OtA5092	OtA5093-OtA5097
Cebpa	chr7:35119292-35121931	OtA5088-OtA5092	OtA5093-OtA5097
Emilin2	chr17:71252175-71310965	OtA5088-OtA5092	OtA5093-OtA5097
Lilrb4	chr10:51490974-51496611	OtA5088-OtA5092	OtA5093-OtA5097
Pde10a	chr17:8801744-8986648	OtA5088-OtA5092	OtA5093-OtA5097
Fgd5	chr6:91987109-92076005	OtA5088-OtA5092	OtA5093-OtA5097
Fam193b	chr13:55539315-55571120	OtA5088-OtA5092	OtA5093-OtA5097
Apba1	chr19:23758875-23949597	OtA5088-OtA5092	OtA5093-OtA5097
Gbp7	chr3:142530337-142550083	OtA5088-OtA5092	OtA5093-OtA5097
Gypa	chr8:80494044-80510785	OtA5088-OtA5092	OtA5093-OtA5097

Magi3	chr3:104013264-104220406	OtA5088-OtA5092	OtA5093-OtA5097
Atf3	chr1:191170296-191183333	OtA5088-OtA5092	OtA5093-OtA5097
Hpse	chr5:100679485-100719683	OtA5088-OtA5092	OtA5093-OtA5097
Kank3	chr17:33810522-33822914	OtA5088-OtA5092	OtA5093-OtA5097
Zmiz1	chr14:25459184-25666747	OtA5088-OtA5092	OtA5093-OtA5097
Gramd1b	chr9:40297906-40455764	OtA5088-OtA5092	OtA5093-OtA5097
Ovol2	chr2:144305175-144332080	OtA5088-OtA5092	OtA5093-OtA5097
Stox1	chr10:62659421-62726099	OtA5088-OtA5092	OtA5093-OtA5097
Adamts15	chr9:30899154-30922452	OtA5088-OtA5092	OtA5093-OtA5097
Jub	chr14:54567468-54577661	OtA5088-OtA5092	OtA5093-OtA5097
Mllt4	chr17:13716435-13905794	OtA5088-OtA5092	OtA5093-OtA5097
Mogs	chr6:83115505-83118898	OtA5088-OtA5092	OtA5093-OtA5097
Ppm1e	chr11:87226905-87358994	OtA5088-OtA5092	OtA5093-OtA5097
Acvr1	chr2:58446437-58566828	OtA5088-OtA5092	OtA5093-OtA5097
Bmp8a	chr4:123312649-123343252	OtA5088-OtA5092	OtA5093-OtA5097
Ptk2b	chr14:66153256-66281052	OtA5088-OtA5092	OtA5093-OtA5097
Slc6a4	chr11:76998596-77032343	OtA5088-OtA5092	OtA5093-OtA5097
Sod3	chr5:52363803-52369738	OtA5088-OtA5092	OtA5093-OtA5097
Klk7	chr7:43811443-43816359	OtA5088-OtA5092	OtA5093-OtA5097
Cish	chr9:107296688-107301961	OtA5088-OtA5092	OtA5093-OtA5097
Tab2	chr10:7905647-7956123	OtA5088-OtA5092	OtA5093-OtA5097
Crtc3	chr7:80586631-80688877	OtA5088-OtA5092	OtA5093-OtA5097
Mthfd1	chr12:76255231-76319820	OtA5088-OtA5092	OtA5093-OtA5097
Jhdm1d	chr6:39118472-39206773	OtA5088-OtA5092	OtA5093-OtA5097
Thoc2	chrX:41794993-41911901	OtA5088-OtA5092	OtA5093-OtA5097
Setbp1	chr18:78750377-79109391	OtA5088-OtA5092	OtA5093-OtA5097
Ccrn4l	chr3:51224446-51251654	OtA5088-OtA5092	OtA5093-OtA5097
Edn1	chr13:42301269-42307989	OtA5088-OtA5092	OtA5093-OtA5097
Cobl	chr11:12236675-12464960	OtA5088-OtA5092	OtA5093-OtA5097
Prl8a6	chr13:27432852-27438597	OtA5088-OtA5092	OtA5093-OtA5097
Rnmtl1	chr11:76243735-76250622	OtA5088-OtA5092	OtA5093-OtA5097
Arid2	chr15:96287521-96405463	OtA5088-OtA5092	OtA5093-OtA5097
Cdcp1	chr9:123172284-123216038	OtA5088-OtA5092	OtA5093-OtA5097
Prrc2c	chr1:162671784-162740556	OtA5088-OtA5092	OtA5093-OtA5097
Gpatch8	chr11:102475915-102556158	OtA5088-OtA5092	OtA5093-OtA5097
Ablim1	chr19:57033263-57216032	OtA5088-OtA5092	OtA5093-OtA5097
Col5a2	chr1:45374330-45503282	OtA5088-OtA5092	OtA5093-OtA5097
Mier1	chr4:103114464-103165749	OtA5088-OtA5092	OtA5093-OtA5097
Adap1	chr5:139271875-139325464	OtA5088-OtA5092	OtA5093-OtA5097
Exph5	chr9:53301669-53381158	OtA5088-OtA5092	OtA5093-OtA5097
Glyctk	chr9:106152859-106158138	OtA5088-OtA5092	OtA5093-OtA5097
Tagap1	chr17:6954964-6961156	OtA5088-OtA5092	OtA5093-OtA5097
Fos	chr12:85473900-85477270	OtA5088-OtA5092	OtA5093-OtA5097
Naga	chr15:82329531-82338826	OtA5088-OtA5092	OtA5093-OtA5097
Hrk	chr5:118169763-118189478	OtA5088-OtA5092	OtA5093-OtA5097
Pla2g4d	chr2:120265868-120289069	OtA5088-OtA5092	OtA5093-OtA5097
Acot9	chrX:155262499-155297507	OtA5088-OtA5092	OtA5093-OtA5097

Pstpip1	chr9:56089975-56128890	OtA5088-OtA5092	OtA5093-OtA5097
Islr	chr9:58156263-58159221	OtA5088-OtA5092	OtA5093-OtA5097
C1qc	chr4:136889801-136892914	OtA5088-OtA5092	OtA5093-OtA5097
Dcn	chr10:97479499-97518162	OtA5088-OtA5092	OtA5093-OtA5097
Dlg3	chrX:100767722-100818410	OtA5088-OtA5092	OtA5093-OtA5097
Fgg	chr3:83007895-83015049	OtA5088-OtA5092	OtA5093-OtA5097
Igsf8	chr1:172312406-172319835	OtA5088-OtA5092	OtA5093-OtA5097
Esco1	chr18:10566511-10610352	OtA5088-OtA5092	OtA5093-OtA5097
Mrpl28	chr17:26123502-26126613	OtA5088-OtA5092	OtA5093-OtA5097
Serpina1e	chr12:103946931-103956897	OtA5088-OtA5092	OtA5093-OtA5097
Armcx4	chrX:134686518-134697772	OtA5088-OtA5092	OtA5093-OtA5097
Heg1	chr16:33684465-33768195	OtA5088-OtA5092	OtA5093-OtA5097
Tmem132e	chr11:82388899-82446327	OtA5088-OtA5092	OtA5093-OtA5097
Fnbp4	chr2:90745369-90781020	OtA5088-OtA5092	OtA5093-OtA5097
Zmym5	chr14:56790584-56811716	OtA5088-OtA5092	OtA5093-OtA5097
Aqp3	chr4:41092723-41098183	OtA5088-OtA5092	OtA5093-OtA5097
Slc5a6	chr5:31036035-31054623	OtA5088-OtA5092	OtA5093-OtA5097
Tmem14c	chr13:41016249-41022582	OtA5088-OtA5092	OtA5093-OtA5097
Ptpru	chr4:131768456-131838278	OtA5088-OtA5092	OtA5093-OtA5097
Ndrg1	chr15:66929317-66969641	OtA5088-OtA5092	OtA5093-OtA5097
Rab15	chr12:76797962-76822524	OtA5088-OtA5092	OtA5093-OtA5097
Slc41a1	chr1:131828011-131848864	OtA5088-OtA5092	OtA5093-OtA5097
Tnrc6a	chr7:123123884-123195296	OtA5088-OtA5092	OtA5093-OtA5097
Id1	chr2:152736273-152737410	OtA5088-OtA5092	OtA5093-OtA5097
Cpt2	chr4:107903981-107923589	OtA5088-OtA5092	OtA5093-OtA5097
Lpin3	chr2:160880669-160906000	OtA5088-OtA5092	OtA5093-OtA5097
Smad6	chr9:63953075-64022059	OtA5088-OtA5092	OtA5093-OtA5097
Fcer1g	chr1:171229571-171234349	OtA5088-OtA5092	OtA5093-OtA5097
Plxnb1	chr9:109095435-109119915	OtA5088-OtA5092	OtA5093-OtA5097
Suv39h1	chrX:8061170-8074760	OtA5088-OtA5092	OtA5093-OtA5097
Taf4a	chr2:179912145-179976646	OtA5088-OtA5092	OtA5093-OtA5097
Elf3	chr1:135253573-135258472	OtA5088-OtA5092	OtA5093-OtA5097
Kifc1	chr17:33875665-33890633	OtA5088-OtA5092	OtA5093-OtA5097
Gtf2h3	chr5:124579147-124597680	OtA5088-OtA5092	OtA5093-OtA5097
Abcd1	chrX:73716596-73738287	OtA5088-OtA5092	OtA5093-OtA5097
Akap12	chr10:4266328-4359471	OtA5088-OtA5092	OtA5093-OtA5097
Bmpr1a	chr14:34411067-34502546	OtA5088-OtA5092	OtA5093-OtA5097
Cbx5	chr15:103191545-103239816	OtA5088-OtA5092	OtA5093-OtA5097
Kcnb1	chr2:167095968-167188818	OtA5088-OtA5092	OtA5093-OtA5097
Mxra8	chr4:155839679-155844102	OtA5088-OtA5092	OtA5093-OtA5097
Pld4	chr12:112760654-112768986	OtA5088-OtA5092	OtA5093-OtA5097
Gpx1	chr9:108339079-108340344	OtA5088-OtA5092	OtA5093-OtA5097
Ubp1	chr9:113930933-114026751	OtA5088-OtA5092	OtA5093-OtA5097
Agrn	chr4:156165289-156197488	OtA5088-OtA5092	OtA5093-OtA5097
Ddrgk1	chr2:130654082-130664645	OtA5088-OtA5092	OtA5093-OtA5097
Kdm6b	chr11:69398517-69413675	OtA5088-OtA5092	OtA5093-OtA5097
Stx6	chr1:155158702-155203517	OtA5088-OtA5092	OtA5093-OtA5097

Pak6	chr2:118663576-118698020	OtA5088-OtA5092	OtA5093-OtA5097
Ccdc41	chr10:94688789-94790336	OtA5088-OtA5092	OtA5093-OtA5097
Hist1h4f	chr13:23551331-23551643	OtA5088-OtA5092	OtA5093-OtA5097
Pcm1	chr8:41239758-41334087	OtA5088-OtA5092	OtA5093-OtA5097
Ptprb	chr10:116301373-116389538	OtA5088-OtA5092	OtA5093-OtA5097
Tbc1d10a	chr11:4186832-4215505	OtA5088-OtA5092	OtA5093-OtA5097
N4bp2	chr5:65763520-65826784	OtA5088-OtA5092	OtA5093-OtA5097
Pdgfa	chr5:138976971-138994953	OtA5088-OtA5092	OtA5093-OtA5097
Tead1	chr7:112679319-112906805	OtA5088-OtA5092	OtA5093-OtA5097
Cdc42bpa	chr1:179961088-180196020	OtA5088-OtA5092	OtA5093-OtA5097
Prl3a1	chr13:27259488-27276660	OtA5088-OtA5092	OtA5093-OtA5097
Dbf4	chr5:8396968-8422716	OtA5088-OtA5092	OtA5093-OtA5097
Ing1	chr8:11556065-11563250	OtA5088-OtA5092	OtA5093-OtA5097
Arl6ip5	chr6:97210791-97233315	OtA5088-OtA5092	OtA5093-OtA5097
Bmf	chr2:118528756-118549678	OtA5088-OtA5092	OtA5093-OtA5097
Lcn2	chr2:32384636-32387739	OtA5088-OtA5092	OtA5093-OtA5097
Myst3	chr8:22859538-22943262	OtA5088-OtA5092	OtA5093-OtA5097
Usp34	chr11:23306894-23497946	OtA5088-OtA5092	OtA5093-OtA5097
Ampd3	chr7:110772603-110812395	OtA5088-OtA5092	OtA5093-OtA5097
Smc6	chr12:11265885-11319785	OtA5088-OtA5092	OtA5093-OtA5097
Nos1ap	chr1:170317495-170589849	OtA5088-OtA5092	OtA5093-OtA5097
Coch	chr12:51593340-51605773	OtA5088-OtA5092	OtA5093-OtA5097
Bok	chr1:93685693-93695762	OtA5088-OtA5092	OtA5093-OtA5097
Tsen34	chr7:3693609-3701035	OtA5088-OtA5092	OtA5093-OtA5097
Ap1m2	chr9:21295456-21312333	OtA5088-OtA5092	OtA5093-OtA5097
Hapln3	chr7:79117026-79131018	OtA5088-OtA5092	OtA5093-OtA5097
Lamb2	chr9:108479861-108490530	OtA5088-OtA5092	OtA5093-OtA5097
Zfp91	chr19:12763527-12796123	OtA5088-OtA5092	OtA5093-OtA5097
Acat1	chr9:53580521-53610350	OtA5088-OtA5092	OtA5093-OtA5097
Cercam	chr2:29869493-29882840	OtA5088-OtA5092	OtA5093-OtA5097
Efnb2	chr8:8617438-8660773	OtA5088-OtA5092	OtA5093-OtA5097
Mll2	chr15:98831668-98871205	OtA5088-OtA5092	OtA5093-OtA5097
Prkd3	chr17:78949404-79020816	OtA5088-OtA5092	OtA5093-OtA5097
Pigg	chr5:108312924-108348779	OtA5088-OtA5092	OtA5093-OtA5097
Ubac2	chr14:121878605-122021035	OtA5088-OtA5092	OtA5093-OtA5097
Neurog2	chr3:127633144-127635631	OtA5088-OtA5092	OtA5093-OtA5097
Gulo	chr14:65986786-66009210	OtA5088-OtA5092	OtA5093-OtA5097
Mfi2	chr16:31878809-31899019	OtA5088-OtA5092	OtA5093-OtA5097
Aim1	chr10:43950306-44004846	OtA5088-OtA5092	OtA5093-OtA5097
Neat1	chr19:5842301-5845478	OtA5088-OtA5092	OtA5093-OtA5097
Acot1	chr12:84009501-84017669	OtA5088-OtA5092	OtA5093-OtA5097
Luc7l2	chr6:38551443-38609470	OtA5088-OtA5092	OtA5093-OtA5097
Snx13	chr12:35047188-35147477	OtA5088-OtA5092	OtA5093-OtA5097
Gch1	chr14:47153894-47189402	OtA5088-OtA5092	OtA5093-OtA5097
Prss35	chr9:86743632-86757506	OtA5088-OtA5092	OtA5093-OtA5097
Bap1	chr14:31251488-31259929	OtA5088-OtA5092	OtA5093-OtA5097
Rap1gap2	chr11:74383482-74590158	OtA5088-OtA5092	OtA5093-OtA5097

Fbrs	chr7:127485220-127491513	OtA5088-OtA5092	OtA5093-OtA5097
Klhdc10	chr6:30401908-30455174	OtA5088-OtA5092	OtA5093-OtA5097
Nck1	chr9:100495002-100546053	OtA5088-OtA5092	OtA5093-OtA5097
Samhd1	chr2:157097528-157135222	OtA5088-OtA5092	OtA5093-OtA5097
Sorbs3	chr14:70180467-70207637	OtA5088-OtA5092	OtA5093-OtA5097
Dusp6	chr10:99263230-99267489	OtA5088-OtA5092	OtA5093-OtA5097
Mysm1	chr4:94942040-94979100	OtA5088-OtA5092	OtA5093-OtA5097
Psmb2	chr4:126677642-126709715	OtA5088-OtA5092	OtA5093-OtA5097
Mll5	chr5:23434428-23616571	OtA5088-OtA5092	OtA5093-OtA5097
Mov10	chr3:104794833-104818563	OtA5088-OtA5092	OtA5093-OtA5097
Mcoln3	chr3:146121790-146140646	OtA5088-OtA5092	OtA5093-OtA5097
Dlx3	chr11:95120116-95125291	OtA5088-OtA5092	OtA5093-OtA5097
Smpd3	chr8:106252547-106337988	OtA5088-OtA5092	OtA5093-OtA5097
Add2	chr6:86078083-86119555	OtA5088-OtA5092	OtA5093-OtA5097
Klk8	chr7:43797576-43803822	OtA5088-OtA5092	OtA5093-OtA5097
Irak4	chr15:94543659-94568316	OtA5088-OtA5092	OtA5093-OtA5097
Trem2	chr17:48346400-48352273	OtA5088-OtA5092	OtA5093-OtA5097
Cfb	chr17:34856373-34862514	OtA5088-OtA5092	OtA5093-OtA5097
Mrc2	chr11:105292645-105351145	OtA5088-OtA5092	OtA5093-OtA5097
Rfx2	chr17:56775896-56831008	OtA5088-OtA5092	OtA5093-OtA5097
Lipg	chr18:74939321-74961263	OtA5088-OtA5092	OtA5093-OtA5097
Macf1	chr4:123349632-123684360	OtA5088-OtA5092	OtA5093-OtA5097
Arhgap18	chr10:26772511-26918648	OtA5088-OtA5092	OtA5093-OtA5097
Asb10	chr5:24532696-24540448	OtA5088-OtA5092	OtA5093-OtA5097
Hist1h4d	chr13:23581601-23581913	OtA5088-OtA5092	OtA5093-OtA5097
Cecr2	chr6:120666420-120771191	OtA5088-OtA5092	OtA5093-OtA5097
Ctsg	chr14:56099880-56102574	OtA5088-OtA5092	OtA5093-OtA5097
Pgam1	chr19:41911870-41918665	OtA5088-OtA5092	OtA5093-OtA5097
11-Sep	chr5:93093456-93174958	OtA5088-OtA5092	OtA5093-OtA5097
Shb	chr4:45423275-45530828	OtA5088-OtA5092	OtA5093-OtA5097
Abcc9	chr6:142587861-142702274	OtA5088-OtA5092	OtA5093-OtA5097
Med13	chr11:86265714-86357525	OtA5088-OtA5092	OtA5093-OtA5097
Fgd6	chr10:94036000-94145339	OtA5088-OtA5092	OtA5093-OtA5097
Irf2	chr8:46739744-46847458	OtA5088-OtA5092	OtA5093-OtA5097
Adh1	chr3:138277644-138290691	OtA5088-OtA5092	OtA5093-OtA5097
Sec22c	chr9:121680044-121705029	OtA5088-OtA5092	OtA5093-OtA5097
Eif4e3	chr6:99625136-99666771	OtA5088-OtA5092	OtA5093-OtA5097
Pxmp4	chr2:154587043-154603673	OtA5088-OtA5092	OtA5093-OtA5097
Tlr2	chr3:83836271-83841608	OtA5088-OtA5092	OtA5093-OtA5097
Pgr	chr9:8899832-8968611	OtA5088-OtA5092	OtA5093-OtA5097
Ralgps2	chr1:156804165-156939626	OtA5088-OtA5092	OtA5093-OtA5097
Calm2	chr17:87433400-87446935	OtA5088-OtA5092	OtA5093-OtA5097
Nup210	chr6:91013066-91116826	OtA5088-OtA5092	OtA5093-OtA5097
Zmynd11	chr13:9684835-9765314	OtA5088-OtA5092	OtA5093-OtA5097
Pcolce	chr5:137605106-137611404	OtA5088-OtA5092	OtA5093-OtA5097
Pth1r	chr9:110722084-110747145	OtA5088-OtA5092	OtA5093-OtA5097
Plcb4	chr2:135741829-136013068	OtA5088-OtA5092	OtA5093-OtA5097

Mapk9	chr11:49846750-49886421	OtA5088-OtA5092	OtA5093-OtA5097
Slk	chr19:47580018-47645246	OtA5088-OtA5092	OtA5093-OtA5097
Aida	chr1:183297059-183324501	OtA5088-OtA5092	OtA5093-OtA5097
Nes	chr3:87971092-87980451	OtA5088-OtA5092	OtA5093-OtA5097
Sdk1	chr5:141241533-142213791	OtA5088-OtA5092	OtA5093-OtA5097
Ascc3	chr10:50592668-50851202	OtA5088-OtA5092	OtA5093-OtA5097
Kcnk16	chr14:20262755-20269162	OtA5088-OtA5092	OtA5093-OtA5097
Trim62	chr4:128884139-128911326	OtA5088-OtA5092	OtA5093-OtA5097
F7	chr8:13026033-13035809	OtA5088-OtA5092	OtA5093-OtA5097
Krt15	chr11:100131758-100135949	OtA5088-OtA5092	OtA5093-OtA5097
Tmem200c	chr17:68837135-68843138	OtA5088-OtA5092	OtA5093-OtA5097
Ankrd11	chr8:122883821-123042284	OtA5088-OtA5092	OtA5093-OtA5097
Crb3	chr17:57062276-57065914	OtA5088-OtA5092	OtA5093-OtA5097
Ap2a2	chr7:141562179-141633011	OtA5088-OtA5092	OtA5093-OtA5097
Pea15a	chr1:172196728-172206781	OtA5088-OtA5092	OtA5093-OtA5097
Hk1	chr10:62268854-62379908	OtA5088-OtA5092	OtA5093-OtA5097
Mmp23	chr4:155650654-155653384	OtA5088-OtA5092	OtA5093-OtA5097
Shoc2	chr19:53892230-54033278	OtA5088-OtA5092	OtA5093-OtA5097
Zmat4	chr8:23669660-24063105	OtA5088-OtA5092	OtA5093-OtA5097
Hbb-b1,Hbb-b2	chr7:103812523-103813923	OtA5088-OtA5092	OtA5093-OtA5097
Krt14	chr11:100203161-100207510	OtA5088-OtA5092	OtA5093-OtA5097
Ly6c1	chr15:75044017-75048837	OtA5088-OtA5092	OtA5093-OtA5097
Id3	chr4:136143821-136145392	OtA5088-OtA5092	OtA5093-OtA5097
Slc29a1	chr17:45585199-45599603	OtA5088-OtA5092	OtA5093-OtA5097
Zbtb8b	chr4:129425764-129440818	OtA5088-OtA5092	OtA5093-OtA5097
Il17rc	chr6:113471454-113483163	OtA5088-OtA5092	OtA5093-OtA5097
Slc9a3	chr13:74121514-74166064	OtA5088-OtA5092	OtA5093-OtA5097
Arid1a	chr4:133679007-133753611	OtA5088-OtA5092	OtA5093-OtA5097
Tpt1	chr14:75845255-75848303	OtA5088-OtA5092	OtA5093-OtA5097
Ghrh	chr2:157329495-157346655	OtA5088-OtA5092	OtA5093-OtA5097
Zfp664	chr5:124862704-124888630	OtA5088-OtA5092	OtA5093-OtA5097
Fut4	chr9:14748458-14752122	OtA5088-OtA5092	OtA5093-OtA5097
Vps13a	chr19:16615365-16780933	OtA5088-OtA5092	OtA5093-OtA5097
Acacb	chr5:114165517-114250758	OtA5088-OtA5092	OtA5093-OtA5097
Sult1e1	chr5:87575967-87591611	OtA5088-OtA5092	OtA5093-OtA5097
Fcho2	chr13:98723405-98815449	OtA5088-OtA5092	OtA5093-OtA5097
Setd2	chr9:110532596-110618633	OtA5088-OtA5092	OtA5093-OtA5097
Elk4	chr1:132007604-132025684	OtA5088-OtA5092	OtA5093-OtA5097
Pde7a	chr3:19225917-19311322	OtA5088-OtA5092	OtA5093-OtA5097
Ly6c2	chr15:75108160-75111949	OtA5088-OtA5092	OtA5093-OtA5097
Col1a2	chr6:4505696-4541543	OtA5088-OtA5092	OtA5093-OtA5097
Pnrc1	chr4:33245422-33248787	OtA5088-OtA5092	OtA5093-OtA5097
Dusp2	chr2:127336158-127338377	OtA5088-OtA5092	OtA5093-OtA5097
Vwf	chr6:125552962-125686677	OtA5088-OtA5092	OtA5093-OtA5097
Ralgapa1	chr12:55602898-55821167	OtA5088-OtA5092	OtA5093-OtA5097
Bri3bp	chr5:125441567-125460885	OtA5088-OtA5092	OtA5093-OtA5097
Smarcad1	chr6:65042666-65116049	OtA5088-OtA5092	OtA5093-OtA5097

Tnfsf9	chr17:57092022-57107757	OtA5088-OtA5092	OtA5093-OtA5097
Zmat1	chrX:134971372-135009209	OtA5088-OtA5092	OtA5093-OtA5097
Snx7	chr3:117781496-117868936	OtA5088-OtA5092	OtA5093-OtA5097
Prps2	chrX:167346332-167382699	OtA5088-OtA5092	OtA5093-OtA5097
Serpinf1	chr11:75410028-75422623	OtA5088-OtA5092	OtA5093-OtA5097
Galnt6	chr15:100689914-100729376	OtA5088-OtA5092	OtA5093-OtA5097
Mtf1	chr4:124802548-124850730	OtA5088-OtA5092	OtA5093-OtA5097
Col1a1	chr11:94936269-94951856	OtA5088-OtA5092	OtA5093-OtA5097
Bcam	chr7:19756137-19770532	OtA5088-OtA5092	OtA5093-OtA5097
Usp20	chr2:30982278-31022655	OtA5088-OtA5092	OtA5093-OtA5097
C230081A13Rik	chr9:56201128-56418050	OtA5088-OtA5092	OtA5093-OtA5097
Dhtkd1	chr2:5898059-5942792	OtA5088-OtA5092	OtA5093-OtA5097
Kdm5c	chrX:152233229-152274354	OtA5088-OtA5092	OtA5093-OtA5097
C1qa	chr4:136895915-136898844	OtA5088-OtA5092	OtA5093-OtA5097
Bod1l	chr5:41787539-41844315	OtA5088-OtA5092	OtA5093-OtA5097
Scd1	chr19:44394449-44407709	OtA5088-OtA5092	OtA5093-OtA5097
Psmb5	chr14:54614119-54617995	OtA5088-OtA5092	OtA5093-OtA5097
Zfp644	chr5:106616740-106696830	OtA5088-OtA5092	OtA5093-OtA5097
Nphp3	chr9:104002543-104043811	OtA5088-OtA5092	OtA5093-OtA5097
Eif5b	chr1:37998009-38129662	OtA5088-OtA5092	OtA5093-OtA5097
Nov	chr15:54745927-54753761	OtA5088-OtA5092	OtA5093-OtA5097
Hoga1	chr19:42045850-42070939	OtA5088-OtA5092	OtA5093-OtA5097
Fam20b	chr1:156678570-156718910	OtA5088-OtA5092	OtA5093-OtA5097
Myom1	chr17:71019556-71126856	OtA5088-OtA5092	OtA5093-OtA5097
Fmnl1	chr11:103171137-103198900	OtA5088-OtA5092	OtA5093-OtA5097
Trpm6	chr19:18749982-18892511	OtA5088-OtA5092	OtA5093-OtA5097
Ash1l	chr3:88965811-89079375	OtA5088-OtA5092	OtA5093-OtA5097
Ech1	chr7:28825337-28832239	OtA5088-OtA5092	OtA5093-OtA5097
Gpr133	chr5:129096749-129204599	OtA5088-OtA5092	OtA5093-OtA5097
Hist1h3f	chr13:23544051-23545054	OtA5088-OtA5092	OtA5093-OtA5097
Nupr1	chr7:126623245-126625470	OtA5088-OtA5092	OtA5093-OtA5097
Lgr4	chr2:109917646-110014257	OtA5088-OtA5092	OtA5093-OtA5097
Crtap	chr9:114368323-114390712	OtA5088-OtA5092	OtA5093-OtA5097
Mt2	chr8:94172617-94173567	OtA5088-OtA5092	OtA5093-OtA5097
Ap2m1	chr16:20535503-20544053	OtA5088-OtA5092	OtA5093-OtA5097
Dmd	chrX:82948869-85205050	OtA5088-OtA5092	OtA5093-OtA5097
Galnt12	chr4:47091952-47123042	OtA5088-OtA5092	OtA5093-OtA5097
Hist1h3e	chr13:23561954-23562365	OtA5088-OtA5092	OtA5093-OtA5097
Ipo4	chr14:55625628-55635678	OtA5088-OtA5092	OtA5093-OtA5097
Naa50	chr16:44139808-44163364	OtA5088-OtA5092	OtA5093-OtA5097
Slc25a20	chr9:108662097-108684641	OtA5088-OtA5092	OtA5093-OtA5097
Slc27a3	chr3:90385232-90389927	OtA5088-OtA5092	OtA5093-OtA5097
Itgam	chr7:128062639-128118491	OtA5088-OtA5092	OtA5093-OtA5097
Trim44	chr2:102300118-102400900	OtA5088-OtA5092	OtA5093-OtA5097
Cd83	chr13:43785106-43803132	OtA5088-OtA5092	OtA5093-OtA5097
Gmppb	chr9:108049289-108079375	OtA5088-OtA5092	OtA5093-OtA5097
Lgals9	chr11:78962978-78984924	OtA5088-OtA5092	OtA5093-OtA5097

Agt	chr8:124556586-124569707	OtA5088-OtA5092	OtA5093-OtA5097
Cebpg	chr7:35046421-35056566	OtA5088-OtA5092	OtA5093-OtA5097
Il34	chr8:110741828-110805903	OtA5088-OtA5092	OtA5093-OtA5097
Chd6	chr2:160946977-161109056	OtA5088-OtA5092	OtA5093-OtA5097
BC013712	chr4:132782356-132796364	OtA5088-OtA5092	OtA5093-OtA5097
Pak2	chr16:32017862-32079273	OtA5088-OtA5092	OtA5093-OtA5097
Akap9	chr5:3928185-4080204	OtA5088-OtA5092	OtA5093-OtA5097
Ankrd35	chr3:96670130-96691034	OtA5088-OtA5092	OtA5093-OtA5097
Ap1b1	chr11:4938755-5042794	OtA5088-OtA5092	OtA5093-OtA5097
Podn	chr4:108014792-108032090	OtA5088-OtA5092	OtA5093-OtA5097
Rab7l1	chr1:131867276-131872889	OtA5088-OtA5092	OtA5093-OtA5097
Uba1	chrX:20658420-20683179	OtA5088-OtA5092	OtA5093-OtA5097
Unc119	chr11:78343521-78349156	OtA5088-OtA5092	OtA5093-OtA5097
Irf2bp2	chr8:126588295-126593436	OtA5088-OtA5092	OtA5093-OtA5097
Atp6v0d1	chr8:105524469-105566040	OtA5088-OtA5092	OtA5093-OtA5097
Ceacam3	chr7:17150399-17163231	OtA5088-OtA5092	OtA5093-OtA5097
Pla2g7	chr17:43568450-43612201	OtA5088-OtA5092	OtA5093-OtA5097
Actg2	chr6:83512908-83536251	OtA5088-OtA5092	OtA5093-OtA5097
Chid1	chr7:141475235-141539857	OtA5088-OtA5092	OtA5093-OtA5097
Ccdc6	chr10:70097120-70193200	OtA5088-OtA5092	OtA5093-OtA5097
Tmed4	chr11:6270713-6274837	OtA5088-OtA5092	OtA5093-OtA5097
Gab2	chr7:97081750-97308951	OtA5088-OtA5092	OtA5093-OtA5097
Cd109	chr9:78615545-78716260	OtA5088-OtA5092	OtA5093-OtA5097
Hck	chr2:153108467-153151441	OtA5088-OtA5092	OtA5093-OtA5097
Obsl1	chr1:75485824-75506452	OtA5088-OtA5092	OtA5093-OtA5097
Trim33	chr3:103279292-103358768	OtA5088-OtA5092	OtA5093-OtA5097
Tmem38b	chr4:53826044-53862018	OtA5088-OtA5092	OtA5093-OtA5097
Wdr81	chr11:75440942-75454717	OtA5088-OtA5092	OtA5093-OtA5097
Atn1	chr6:124742543-124756487	OtA5088-OtA5092	OtA5093-OtA5097
Hist1h2ab	chr13:23751087-23751593	OtA5088-OtA5092	OtA5093-OtA5097
Nppc	chr1:86666292-86670573	OtA5088-OtA5092	OtA5093-OtA5097
Pkp3	chr7:141078228-141090510	OtA5088-OtA5092	OtA5093-OtA5097
Ppil4	chr10:7792893-7822563	OtA5088-OtA5092	OtA5093-OtA5097
Rasa4	chr5:136083915-136111860	OtA5088-OtA5092	OtA5093-OtA5097
Slmap	chr14:26413174-26533740	OtA5088-OtA5092	OtA5093-OtA5097
Cd63	chr10:128908918-128912818	OtA5088-OtA5092	OtA5093-OtA5097
Pcnp	chr16:56015507-56029717	OtA5088-OtA5092	OtA5093-OtA5097
Pigr	chr1:130826683-130852249	OtA5088-OtA5092	OtA5093-OtA5097
Tfam	chr10:71225476-71238044	OtA5088-OtA5092	OtA5093-OtA5097
Pdlim4	chr11:54054927-54069017	OtA5088-OtA5092	OtA5093-OtA5097
Cd52	chr4:134093537-134095073	OtA5088-OtA5092	OtA5093-OtA5097
Col3a1	chr1:45311537-45349706	OtA5088-OtA5092	OtA5093-OtA5097
Lnpep	chr17:17527722-17624489	OtA5088-OtA5092	OtA5093-OtA5097
Hey1	chr3:8663358-8667038	OtA5088-OtA5092	OtA5093-OtA5097
Fhit	chr14:9550093-11162032	OtA5088-OtA5092	OtA5093-OtA5097
Lrp2	chr2:69424334-69586067	OtA5088-OtA5092	OtA5093-OtA5097
Mall	chr2:127704389-127729897	OtA5088-OtA5092	OtA5093-OtA5097

Ncl	chr1:86344718-86359455	OtA5088-OtA5092	OtA5093-OtA5097
Ubn2	chr6:38433924-38512763	OtA5088-OtA5092	OtA5093-OtA5097
Tmem106a	chr11:101582241-101591785	OtA5088-OtA5092	OtA5093-OtA5097
Zfp280d	chr9:72274898-72363771	OtA5088-OtA5092	OtA5093-OtA5097
Plekhm3	chr1:64789120-64956824	OtA5088-OtA5092	OtA5093-OtA5097
Cdh1	chr8:106603367-106670246	OtA5088-OtA5092	OtA5093-OtA5097
Dock7	chr4:98936664-99120915	OtA5088-OtA5092	OtA5093-OtA5097
Prr5	chr15:84680997-84703673	OtA5088-OtA5092	OtA5093-OtA5097
Bmpr2	chr1:59764636-59870859	OtA5088-OtA5092	OtA5093-OtA5097
C8g	chr2:25498655-25500506	OtA5088-OtA5092	OtA5093-OtA5097
Lsr	chr7:30957769-30973469	OtA5088-OtA5092	OtA5093-OtA5097
Taldo1	chr7:141392159-141402976	OtA5088-OtA5092	OtA5093-OtA5097
Tfcp2l1	chr1:118627944-118685168	OtA5088-OtA5092	OtA5093-OtA5097
Prr5l	chr2:101714284-101838980	OtA5088-OtA5092	OtA5093-OtA5097
Trpm7	chr2:126791557-126876261	OtA5088-OtA5092	OtA5093-OtA5097
Maml3	chr3:51687612-52105006	OtA5088-OtA5092	OtA5093-OtA5097
Oxsr1	chr9:119238432-119322427	OtA5088-OtA5092	OtA5093-OtA5097
Alg3	chr16:20605457-20610749	OtA5088-OtA5092	OtA5093-OtA5097
Arl4d	chr11:101665540-101667832	OtA5088-OtA5092	OtA5093-OtA5097
Eln	chr5:134702595-134747254	OtA5088-OtA5092	OtA5093-OtA5097
Hprt	chrX:52988077-53021660	OtA5088-OtA5092	OtA5093-OtA5097
Myo18b	chr5:112688875-112896362	OtA5088-OtA5092	OtA5093-OtA5097
Ntrk2	chr13:58806595-59129967	OtA5088-OtA5092	OtA5093-OtA5097
Saa3	chr7:46711997-46715676	OtA5088-OtA5092	OtA5093-OtA5097
Slc5a1	chr5:33104218-33162699	OtA5088-OtA5092	OtA5093-OtA5097
Tcerg1	chr18:42511486-42575785	OtA5088-OtA5092	OtA5093-OtA5097
Tcf7l2	chr19:55741809-55933655	OtA5088-OtA5092	OtA5093-OtA5097
Nucks1	chr1:131910457-131936321	OtA5088-OtA5092	OtA5093-OtA5097
Pard6b	chr2:168081003-168101203	OtA5088-OtA5092	OtA5093-OtA5097
Atg7	chr6:114643096-114860614	OtA5088-OtA5092	OtA5093-OtA5097
C1qb	chr4:136880144-136886177	OtA5088-OtA5092	OtA5093-OtA5097
Cryaa	chr17:31677944-31681724	OtA5088-OtA5092	OtA5093-OtA5097
Hbegf	chr18:36504926-36515805	OtA5088-OtA5092	OtA5093-OtA5097
Myo10	chr15:25622549-25813671	OtA5088-OtA5092	OtA5093-OtA5097
Ppp1r12a	chr10:108162399-108277575	OtA5088-OtA5092	OtA5093-OtA5097
Scaf4	chr16:90229143-90284425	OtA5088-OtA5092	OtA5093-OtA5097
Acat2	chr17:12943041-12960725	OtA5088-OtA5092	OtA5093-OtA5097
Asl	chr5:130011502-130024331	OtA5088-OtA5092	OtA5093-OtA5097
Gart	chr16:91621396-91646972	OtA5088-OtA5092	OtA5093-OtA5097
Gps1	chr11:120784271-120789102	OtA5088-OtA5092	OtA5093-OtA5097
Cldn2	chrX:139800807-139811388	OtA5088-OtA5092	OtA5093-OtA5097
Epdr1	chr13:19591707-19619830	OtA5088-OtA5092	OtA5093-OtA5097
Mgat4a	chr1:37439339-37536259	OtA5088-OtA5092	OtA5093-OtA5097
Nostrin	chr2:69135799-69189329	OtA5088-OtA5092	OtA5093-OtA5097
Cops2	chr2:125830303-125859018	OtA5088-OtA5092	OtA5093-OtA5097
Pkn2	chr3:142790901-142882004	OtA5088-OtA5092	OtA5093-OtA5097
Fbxo30	chr10:11281329-11297969	OtA5088-OtA5092	OtA5093-OtA5097

Tmem150a	chr6:72355482-72362381	OtA5088-OtA5092	OtA5093-OtA5097
St3gal4	chr9:35046578-35175987	OtA5088-OtA5092	OtA5093-OtA5097
Stard13	chr5:151037514-151190193	OtA5088-OtA5092	OtA5093-OtA5097
Wsb2	chr5:117357304-117378589	OtA5088-OtA5092	OtA5093-OtA5097
Kdm3a	chr6:71588971-71632905	OtA5088-OtA5092	OtA5093-OtA5097
Clcn5	chrX:7158411-7319358	OtA5088-OtA5092	OtA5093-OtA5097
Med13l	chr5:118560718-118765437	OtA5088-OtA5092	OtA5093-OtA5097
Hist1h4h	chr13:23531043-23531521	OtA5088-OtA5092	OtA5093-OtA5097
Nrm	chr17:35861317-35865400	OtA5088-OtA5092	OtA5093-OtA5097
Gatm	chr2:122594472-122611277	OtA5088-OtA5092	OtA5093-OtA5097
Drosha	chr15:12824814-12935291	OtA5088-OtA5092	OtA5093-OtA5097
Mtmr4	chr11:87592216-87616296	OtA5088-OtA5092	OtA5093-OtA5097
Reep1	chr6:71707680-71810705	OtA5088-OtA5092	OtA5093-OtA5097
Zfp451	chr1:33761752-33814427	OtA5088-OtA5092	OtA5093-OtA5097
Pacsin3	chr2:91256812-91264679	OtA5088-OtA5092	OtA5093-OtA5097
Stx1b	chr7:127806843-127824531	OtA5088-OtA5092	OtA5093-OtA5097
Plau	chr14:20836661-20843388	OtA5088-OtA5092	OtA5093-OtA5097
Bcl9	chr3:97204627-97216507	OtA5088-OtA5092	OtA5093-OtA5097
Yrdc	chr4:124850758-124862171	OtA5088-OtA5092	OtA5093-OtA5097
Zfp866	chr8:69761323-69774911	OtA5088-OtA5092	OtA5093-OtA5097
Mab21l3	chr3:101813075-101836223	OtA5088-OtA5092	OtA5093-OtA5097
Rlf	chr4:121145372-121188534	OtA5088-OtA5092	OtA5093-OtA5097
Mboat1	chr13:30136489-30246694	OtA5088-OtA5092	OtA5093-OtA5097
B4galt6	chr18:20684598-20746404	OtA5088-OtA5092	OtA5093-OtA5097
Zbtb32	chr7:30589680-30592942	OtA5088-OtA5092	OtA5093-OtA5097
Hist1h2af	chr13:23533910-23534303	OtA5088-OtA5092	OtA5093-OtA5097
Ctsz	chr2:174415803-174438992	OtA5088-OtA5092	OtA5093-OtA5097
Pik3ca	chr3:32436150-32468486	OtA5088-OtA5092	OtA5093-OtA5097
Myo1g	chr11:6506547-6520958	OtA5088-OtA5092	OtA5093-OtA5097
Plcd1	chr9:119071527-119093496	OtA5088-OtA5092	OtA5093-OtA5097
Gabpb2	chr3:95181765-95217942	OtA5088-OtA5092	OtA5093-OtA5097
Xrn1	chr9:95954759-96053029	OtA5088-OtA5092	OtA5093-OtA5097
Psma5	chr3:108256925-108279952	OtA5088-OtA5092	OtA5093-OtA5097
Zfp395	chr14:65358675-65398930	OtA5088-OtA5092	OtA5093-OtA5097
Klf2	chr8:72319061-72321654	OtA5088-OtA5092	OtA5093-OtA5097
Rnf216	chr5:142990892-143113020	OtA5088-OtA5092	OtA5093-OtA5097
Adam10	chr9:70679000-70780229	OtA5088-OtA5092	OtA5093-OtA5097
Chchd10	chr10:75935572-75937734	OtA5088-OtA5092	OtA5093-OtA5097
Plekhg6	chr6:125362650-125380504	OtA5088-OtA5092	OtA5093-OtA5097
Rere	chr4:150281915-150621966	OtA5088-OtA5092	OtA5093-OtA5097
Tmem62	chr2:120977061-121007842	OtA5088-OtA5092	OtA5093-OtA5097
Tug1	chr11:3639793-3648808	OtA5088-OtA5092	OtA5093-OtA5097
Chd1	chr17:15704966-15772612	OtA5088-OtA5092	OtA5093-OtA5097
Map4k3	chr17:80580511-80728093	OtA5088-OtA5092	OtA5093-OtA5097
Mst1r	chr9:107906888-107920383	OtA5088-OtA5092	OtA5093-OtA5097
Papola	chr12:105784701-105838943	OtA5088-OtA5092	OtA5093-OtA5097
Pgd	chr4:149149984-149166707	OtA5088-OtA5092	OtA5093-OtA5097

Ralgapa2	chr2:146241298-146512004	OtA5088-OtA5092	OtA5093-OtA5097
Vdac2	chr14:21831560-21848910	OtA5088-OtA5092	OtA5093-OtA5097
Vmp1	chr11:86583864-86683822	OtA5088-OtA5092	OtA5093-OtA5097
Gcc2	chr10:58255525-58305592	OtA5088-OtA5092	OtA5093-OtA5097
Ly86	chr13:37345344-37419036	OtA5088-OtA5092	OtA5093-OtA5097
Mid2	chrX:140678027-140767715	OtA5088-OtA5092	OtA5093-OtA5097
Otud4	chr8:79639675-79677755	OtA5088-OtA5092	OtA5093-OtA5097
Rbm6	chr9:107773558-107872819	OtA5088-OtA5092	OtA5093-OtA5097
Akap11	chr14:78492245-78536860	OtA5088-OtA5092	OtA5093-OtA5097
Tmf1	chr6:97151949-97179124	OtA5088-OtA5092	OtA5093-OtA5097
Cobll1	chr2:65088338-65238626	OtA5088-OtA5092	OtA5093-OtA5097
Rcor1	chr12:111039797-111113386	OtA5088-OtA5092	OtA5093-OtA5097
Hist1h4k	chr13:21750193-21750505	OtA5088-OtA5092	OtA5093-OtA5097
Pklr	chr3:89136141-89146594	OtA5088-OtA5092	OtA5093-OtA5097
Hspa12a	chr19:58795750-58860984	OtA5088-OtA5092	OtA5093-OtA5097
Enpp7	chr11:118988187-118992841	OtA5088-OtA5092	OtA5093-OtA5097
Eif3i	chr4:129591973-129600648	OtA5088-OtA5092	OtA5093-OtA5097
Zfr	chr15:12117850-12185449	OtA5088-OtA5092	OtA5093-OtA5097
Dcun1d1	chr3:35892104-35932966	OtA5088-OtA5092	OtA5093-OtA5097
Ptk2	chr15:73205104-73423191	OtA5088-OtA5092	OtA5093-OtA5097
Rell1	chr5:63908897-63968897	OtA5088-OtA5092	OtA5093-OtA5097
Dhrs9	chr2:69380461-69403086	OtA5088-OtA5092	OtA5093-OtA5097
Rnf222	chr11:68888552-68895015	OtA5088-OtA5092	OtA5093-OtA5097
Tcea3	chr4:136247956-136274899	OtA5088-OtA5092	OtA5093-OtA5097
Cldn23	chr8:35824708-35826559	OtA5088-OtA5092	OtA5093-OtA5097
Mogat2	chr7:99219083-99238611	OtA5088-OtA5092	OtA5093-OtA5097
Ppp1r9a	chr6:4903319-5165661	OtA5088-OtA5092	OtA5093-OtA5097
Arl15	chr13:113794507-114157461	OtA5088-OtA5092	OtA5093-OtA5097
Ctsk	chr3:95499285-95509362	OtA5088-OtA5092	OtA5093-OtA5097
Itch	chr2:155133480-155226855	OtA5088-OtA5092	OtA5093-OtA5097
Sox7	chr14:63943705-63950732	OtA5088-OtA5092	OtA5093-OtA5097
Zfp608	chr18:54888044-54990180	OtA5088-OtA5092	OtA5093-OtA5097
Smg6	chr11:74925871-75164448	OtA5088-OtA5092	OtA5093-OtA5097
Adarb1	chr10:77290726-77418273	OtA5088-OtA5092	OtA5093-OtA5097
Capn1	chr19:5988544-6018459	OtA5088-OtA5092	OtA5093-OtA5097
Ak7	chr12:105705981-105782447	OtA5088-OtA5092	OtA5093-OtA5097
Bst1	chr5:43818892-43843468	OtA5088-OtA5092	OtA5093-OtA5097
Prdm6	chr18:53464545-53575857	OtA5088-OtA5092	OtA5093-OtA5097
Rab6b	chr9:103112073-103185270	OtA5088-OtA5092	OtA5093-OtA5097
Smc3	chr19:53600395-53645831	OtA5088-OtA5092	OtA5093-OtA5097
Bdp1	chr13:100017993-100104070	OtA5088-OtA5092	OtA5093-OtA5097
Slc39a1	chr3:90248191-90253612	OtA5088-OtA5092	OtA5093-OtA5097
Hist1h2bf	chr13:23573809-23574190	OtA5088-OtA5092	OtA5093-OtA5097
Ppfibp1	chr6:146888493-147032023	OtA5088-OtA5092	OtA5093-OtA5097
Ptprn	chr1:75247040-75264208	OtA5088-OtA5092	OtA5093-OtA5097
Nrbp2	chr15:76085593-76090013	OtA5088-OtA5092	OtA5093-OtA5097
Pde5a	chr3:122729157-122859374	OtA5088-OtA5092	OtA5093-OtA5097

Rasef	chr4:73714578-73790602	OtA5088-OtA5092	OtA5093-OtA5097
Rnf38	chr4:44126211-44168283	OtA5088-OtA5092	OtA5093-OtA5097
Pak1ip1	chr13:41001009-41013033	OtA5088-OtA5092	OtA5093-OtA5097
Dusp5	chr19:53529317-53541322	OtA5088-OtA5092	OtA5093-OtA5097
Itpk1	chr12:102568582-102704869	OtA5088-OtA5092	OtA5093-OtA5097
Col15a1	chr4:47208011-47313165	OtA5088-OtA5092	OtA5093-OtA5097
Adcy9	chr16:4287544-4419587	OtA5088-OtA5092	OtA5093-OtA5097
Azi1	chr11:120064429-120086827	OtA5088-OtA5092	OtA5093-OtA5097
Fnbp1	chr2:31026205-31142008	OtA5088-OtA5092	OtA5093-OtA5097
Fermt3	chr19:6996130-7019469	OtA5088-OtA5092	OtA5093-OtA5097
Sfrp5	chr19:42197970-42202252	OtA5088-OtA5092	OtA5093-OtA5097
Ctsd	chr7:142375915-142387870	OtA5088-OtA5092	OtA5093-OtA5097
Nhsl2	chrX:101849384-102092055	OtA5088-OtA5092	OtA5093-OtA5097
Igf2bp1	chr11:95957163-96005944	OtA5088-OtA5092	OtA5093-OtA5097
Sbno1	chr5:124368701-124425914	OtA5088-OtA5092	OtA5093-OtA5097
Mosc1	chr1:184786766-184811313	OtA5088-OtA5092	OtA5093-OtA5097
Psg22	chr7:18718089-18727248	OtA5088-OtA5092	OtA5093-OtA5097
Cox5a	chr9:57521231-57532426	OtA5088-OtA5092	OtA5093-OtA5097
D4Bwg0951e	chr4:80910685-80954301	OtA5088-OtA5092	OtA5093-OtA5097
Fas	chr19:34290658-34327770	OtA5088-OtA5092	OtA5093-OtA5097
Micall1	chr15:79108982-79141251	OtA5088-OtA5092	OtA5093-OtA5097
Phc1	chr6:122317730-122337015	OtA5088-OtA5092	OtA5093-OtA5097
Pygb	chr2:150786795-150831748	OtA5088-OtA5092	OtA5093-OtA5097
Vps18	chr2:119288741-119298453	OtA5088-OtA5092	OtA5093-OtA5097
Srsf4	chr4:131873638-131901725	OtA5088-OtA5092	OtA5093-OtA5097
Ankrd17	chr5:90227165-90366185	OtA5088-OtA5092	OtA5093-OtA5097
Arg1	chr10:24915206-24927470	OtA5088-OtA5092	OtA5093-OtA5097
Cmbl	chr15:31568911-31590119	OtA5088-OtA5092	OtA5093-OtA5097
Gcnt1	chr19:17326140-17372844	OtA5088-OtA5092	OtA5093-OtA5097
Nckap1l	chr15:103453824-103498800	OtA5088-OtA5092	OtA5093-OtA5097
Ppp1ca	chr19:4192173-4201603	OtA5088-OtA5092	OtA5093-OtA5097
Ppp2r1a	chr17:20945453-20965905	OtA5088-OtA5092	OtA5093-OtA5097
Zswim1	chr2:164822685-164826867	OtA5088-OtA5092	OtA5093-OtA5097
Amt	chr9:108296921-108301597	OtA5088-OtA5092	OtA5093-OtA5097
Enpp3	chr10:24773813-24836195	OtA5088-OtA5092	OtA5093-OtA5097
Pmepa1	chr2:173224464-173276533	OtA5088-OtA5092	OtA5093-OtA5097
Atp5a1	chr18:77773767-77782868	OtA5088-OtA5092	OtA5093-OtA5097
Lrp10	chr14:54464146-54470291	OtA5088-OtA5092	OtA5093-OtA5097
Sesn2	chr4:132492806-132510456	OtA5088-OtA5092	OtA5093-OtA5097
Snta1	chr2:154376313-154408084	OtA5088-OtA5092	OtA5093-OtA5097
Fam76b	chr9:13827726-13846522	OtA5088-OtA5092	OtA5093-OtA5097
Pik3c2a	chr7:116337275-116443458	OtA5088-OtA5092	OtA5093-OtA5097
Angpt4	chr2:151911331-151944654	OtA5088-OtA5092	OtA5093-OtA5097
Dnajc27	chr12:4082573-4110612	OtA5088-OtA5092	OtA5093-OtA5097
Appl2	chr10:83600033-83648664	OtA5088-OtA5092	OtA5093-OtA5097
Gxylt1	chr15:93239741-93275084	OtA5088-OtA5092	OtA5093-OtA5097
Glis2	chr16:4594712-4615957	OtA5088-OtA5092	OtA5093-OtA5097

Klk6	chr7:43824543-43832027	OtA5088-OtA5092	OtA5093-OtA5097
Zfp187	chr13:21442174-21453727	OtA5088-OtA5092	OtA5093-OtA5097
Ceacam18	chr7:43634738-43649294	OtA5088-OtA5092	OtA5093-OtA5097
Ptp4a2	chr4:129820478-129850003	OtA5088-OtA5092	OtA5093-OtA5097
Qk	chr17:10206470-10319361	OtA5088-OtA5092	OtA5093-OtA5097
Adamtsl1	chr4:86053914-86428382	OtA5088-OtA5092	OtA5093-OtA5097
Acadl	chr1:66830852-66863267	OtA5088-OtA5092	OtA5093-OtA5097
Atp1a3	chr7:24978168-25005895	OtA5088-OtA5092	OtA5093-OtA5097
Man2b1	chr8:85083268-85098739	OtA5088-OtA5092	OtA5093-OtA5097
Prmt2	chr10:76207221-76237865	OtA5088-OtA5092	OtA5093-OtA5097
Prkcz	chr4:155260119-155361391	OtA5088-OtA5092	OtA5093-OtA5097
Tmem177	chr1:119907898-119913168	OtA5088-OtA5092	OtA5093-OtA5097
Ap2s1	chr7:16738443-16749290	OtA5088-OtA5092	OtA5093-OtA5097
Rac2	chr15:78559168-78572783	OtA5088-OtA5092	OtA5093-OtA5097
Frat2	chr19:41845975-41848132	OtA5088-OtA5092	OtA5093-OtA5097
Frmpd1	chr4:45184905-45285936	OtA5088-OtA5092	OtA5093-OtA5097
Srek1	chr13:103741614-103764582	OtA5088-OtA5092	OtA5093-OtA5097
Xk	chrX:9272783-9313245	OtA5088-OtA5092	OtA5093-OtA5097
Zbtb7c	chr18:75820177-76148564	OtA5088-OtA5092	OtA5093-OtA5097
Zfp62	chr11:49203499-49218816	OtA5088-OtA5092	OtA5093-OtA5097
Nceh1	chr3:27183003-27244911	OtA5088-OtA5092	OtA5093-OtA5097
Sbf2	chr7:110308012-110614920	OtA5088-OtA5092	OtA5093-OtA5097
Flnc	chr6:29433152-29461888	OtA5088-OtA5092	OtA5093-OtA5097
Eri1	chr8:35465264-35495533	OtA5088-OtA5092	OtA5093-OtA5097
Ate1	chr7:130391493-130519961	OtA5088-OtA5092	OtA5093-OtA5097
D17Wsu92e	chr17:27751234-27820542	OtA5088-OtA5092	OtA5093-OtA5097
R3hdm2	chr10:127390310-127499384	OtA5088-OtA5092	OtA5093-OtA5097
Satb2	chr1:56793980-56971334	OtA5088-OtA5092	OtA5093-OtA5097
Nrbp1	chr5:31240917-31251562	OtA5088-OtA5092	OtA5093-OtA5097
Ptgr1	chr4:58965589-58987078	OtA5088-OtA5092	OtA5093-OtA5097
Stk25	chr1:93512103-93635727	OtA5088-OtA5092	OtA5093-OtA5097
Tmco4	chr4:138972904-139059171	OtA5088-OtA5092	OtA5093-OtA5097
Npat	chr9:53537046-53575627	OtA5088-OtA5092	OtA5093-OtA5097
Snapc3	chr4:83417743-83453340	OtA5088-OtA5092	OtA5093-OtA5097
Cog2	chr8:124520766-124552007	OtA5088-OtA5092	OtA5093-OtA5097
Dnmt3l	chr10:78030030-78063615	OtA5088-OtA5092	OtA5093-OtA5097
Etl4	chr2:20289912-20810535	OtA5088-OtA5092	OtA5093-OtA5097
Gbgt1	chr2:28496890-28505415	OtA5088-OtA5092	OtA5093-OtA5097
Hdac6	chrX:7930121-7947889	OtA5088-OtA5092	OtA5093-OtA5097
Mpp5	chr12:78748946-78840713	OtA5088-OtA5092	OtA5093-OtA5097
Siglec1	chr2:131069219-131086765	OtA5088-OtA5092	OtA5093-OtA5097
Slc11a1	chr1:74375202-74386051	OtA5088-OtA5092	OtA5093-OtA5097
Srsf11	chr3:158010492-158036639	OtA5088-OtA5092	OtA5093-OtA5097
Tmem229b	chr12:78961794-79007277	OtA5088-OtA5092	OtA5093-OtA5097
Ttc7	chr17:87282885-87381770	OtA5088-OtA5092	OtA5093-OtA5097
Baz1b	chr5:135187322-135246129	OtA5088-OtA5092	OtA5093-OtA5097
Mier3	chr13:111686177-111718594	OtA5088-OtA5092	OtA5093-OtA5097

Pnpla7	chr2:24976032-25054072	OtA5088-OtA5092	OtA5093-OtA5097
Ccrl2	chr9:111054834-111057270	OtA5088-OtA5092	OtA5093-OtA5097
Slc39a11	chr11:113244854-113565815	OtA5088-OtA5092	OtA5093-OtA5097
Nccrp1	chr7:28543595-28547254	OtA5088-OtA5092	OtA5093-OtA5097
Zcchc6	chr13:59769965-59823147	OtA5088-OtA5092	OtA5093-OtA5097
Arrdc3	chr13:80883421-80896043	OtA5088-OtA5092	OtA5093-OtA5097
Echs1	chr7:140105722-140116423	OtA5088-OtA5092	OtA5093-OtA5097
Ncor2	chr5:125017152-125179214	OtA5088-OtA5092	OtA5093-OtA5097
Fzd1	chr5:4753838-4758216	OtA5088-OtA5092	OtA5093-OtA5097
Rassf5	chr1:131176409-131245178	OtA5088-OtA5092	OtA5093-OtA5097
Ccdc117	chr11:5528887-5542217	OtA5088-OtA5092	OtA5093-OtA5097
Dcun1d3	chr7:119853162-119895745	OtA5088-OtA5092	OtA5093-OtA5097
Phc3	chr3:30899294-30969415	OtA5088-OtA5092	OtA5093-OtA5097
Rev3l	chr10:39732159-39875205	OtA5088-OtA5092	OtA5093-OtA5097
Dnmt3b	chr2:153649453-153687730	OtA5088-OtA5092	OtA5093-OtA5097
Slc16a9	chr10:70245275-70285951	OtA5088-OtA5092	OtA5093-OtA5097
Figf	chrX:164373547-164402647	OtA5088-OtA5092	OtA5093-OtA5097
Neu1	chr17:34931252-34937297	OtA5088-OtA5092	OtA5093-OtA5097
Pabpc1	chr15:36595657-36608973	OtA5088-OtA5092	OtA5093-OtA5097
Yipf1	chr4:107314362-107359823	OtA5088-OtA5092	OtA5093-OtA5097
Gbp6	chr5:105270701-105293699	OtA5088-OtA5092	OtA5093-OtA5097
Zdhhc12	chr2:30078765-30093635	OtA5088-OtA5092	OtA5093-OtA5097
Pag1	chr3:9687481-9833679	OtA5088-OtA5092	OtA5093-OtA5097
Shisa3	chr5:67607882-67613987	OtA5088-OtA5092	OtA5093-OtA5097
Abca2	chr2:25428673-25448539	OtA5088-OtA5092	OtA5093-OtA5097
Rasip1	chr7:45627536-45639092	OtA5088-OtA5092	OtA5093-OtA5097
Dlk1	chr12:109452822-109463336	OtA5088-OtA5092	OtA5093-OtA5097
Trub2	chr2:29776110-29787671	OtA5088-OtA5092	OtA5093-OtA5097
Hist1h4c	chr13:23698137-23698449	OtA5088-OtA5092	OtA5093-OtA5097
Tmem158	chr9:123259056-123260789	OtA5088-OtA5092	OtA5093-OtA5097
Wdr86	chr5:24712268-24730680	OtA5088-OtA5092	OtA5093-OtA5097
Dnajc8	chr4:132535558-132553742	OtA5088-OtA5092	OtA5093-OtA5097
Thnsl2	chr6:71128397-71144376	OtA5088-OtA5092	OtA5093-OtA5097
Inha	chr1:75507076-75510354	OtA5088-OtA5092	OtA5093-OtA5097
Apln	chrX:48025145-48034852	OtA5088-OtA5092	OtA5093-OtA5097
Eif2c2	chr15:73101624-73184947	OtA5088-OtA5092	OtA5093-OtA5097
Hist1h2ae	chr13:23570516-23571220	OtA5088-OtA5092	OtA5093-OtA5097
Snap47	chr11:59407149-59449956	OtA5088-OtA5092	OtA5093-OtA5097
Rgs17	chr10:5825663-5922400	OtA5088-OtA5092	OtA5093-OtA5097
Spata2l	chr8:123232257-123254175	OtA5088-OtA5092	OtA5093-OtA5097
Atp8b1	chr18:64528978-64661000	OtA5088-OtA5092	OtA5093-OtA5097
Galntl2	chr14:32029102-32058326	OtA5088-OtA5092	OtA5093-OtA5097
Zbtb16	chr9:48654296-48835945	OtA5088-OtA5092	OtA5093-OtA5097
Tmem170b	chr13:41606215-41641357	OtA5088-OtA5092	OtA5093-OtA5097
Cdk16	chrX:20688492-20699877	OtA5088-OtA5092	OtA5093-OtA5097
Tmed3	chr9:89699202-89705043	OtA5088-OtA5092	OtA5093-OtA5097
Dennd2a	chr6:39462377-39557834	OtA5088-OtA5092	OtA5093-OtA5097

Esx1	chrX:137115396-137120326	OtA5088-OtA5092	OtA5093-OtA5097
Flot2	chr11:78037940-78060432	OtA5088-OtA5092	OtA5093-OtA5097
C1qtnf2	chr11:43474305-43491525	OtA5088-OtA5092	OtA5093-OtA5097
Tubb4b	chr2:25218744-25224702	OtA5088-OtA5092	OtA5093-OtA5097
Wipf3	chr6:54452882-54503768	OtA5088-OtA5092	OtA5093-OtA5097
Phip	chr9:82866158-82975489	OtA5088-OtA5092	OtA5093-OtA5097
Sult2b1	chr7:45729982-45759555	OtA5088-OtA5092	OtA5093-OtA5097
Ttbk2	chr2:120732816-120850584	OtA5088-OtA5092	OtA5093-OtA5097
Hdgf	chr3:87906320-87916132	OtA5088-OtA5092	OtA5093-OtA5097
Slc7a7	chr14:54360840-54417702	OtA5088-OtA5092	OtA5093-OtA5097
Tubb2b	chr13:34127007-34130354	OtA5088-OtA5092	OtA5093-OtA5097
Prrg2	chr7:45053606-45061652	OtA5088-OtA5092	OtA5093-OtA5097
Ctdspl	chr9:118926535-119044119	OtA5088-OtA5092	OtA5093-OtA5097
Creb1	chr1:64532803-64604548	OtA5088-OtA5092	OtA5093-OtA5097
Itfg3	chr17:26212691-26244242	OtA5088-OtA5092	OtA5093-OtA5097
Fam65c	chr2:167980785-168010593	OtA5088-OtA5092	OtA5093-OtA5097
Frem1	chr4:82897919-83052167	OtA5088-OtA5092	OtA5093-OtA5097
Plxna4	chr6:32144556-32588192	OtA5088-OtA5092	OtA5093-OtA5097
Lgals3	chr14:47373859-47386167	OtA5088-OtA5092	OtA5093-OtA5097
Muc1	chr3:89229051-89233369	OtA5088-OtA5092	OtA5093-OtA5097
Hnrpdl	chr5:100033578-100039222	OtA5088-OtA5092	OtA5093-OtA5097
Nkain1	chr4:130530130-130574036	OtA5088-OtA5092	OtA5093-OtA5097
Itga2	chr13:114835911-114932041	OtA5088-OtA5092	OtA5093-OtA5097
Usp33	chr3:152346477-152393614	OtA5088-OtA5092	OtA5093-OtA5097
Zscan21	chr5:138116904-138134259	OtA5088-OtA5092	OtA5093-OtA5097
Adcy3	chr12:4133396-4240123	OtA5088-OtA5092	OtA5093-OtA5097
Lsm14a	chr7:34344719-34389540	OtA5088-OtA5092	OtA5093-OtA5097
Rp2h	chrX:20364607-20400349	OtA5088-OtA5092	OtA5093-OtA5097
Tuba1c	chr15:99029890-99038105	OtA5088-OtA5092	OtA5093-OtA5097
B3gnt7	chr1:86303220-86307305	OtA5088-OtA5092	OtA5093-OtA5097
Cdh3	chr8:106510890-106556908	OtA5088-OtA5092	OtA5093-OtA5097
Gpr157	chr4:150087502-150105927	OtA5088-OtA5092	OtA5093-OtA5097
Ireb2	chr9:54863754-54912534	OtA5088-OtA5092	OtA5093-OtA5097
Zfp704	chr3:9427009-9610085	OtA5088-OtA5092	OtA5093-OtA5097
Crym	chr7:120186383-120201988	OtA5088-OtA5092	OtA5093-OtA5097
Bcl9l	chr9:44499135-44510412	OtA5088-OtA5092	OtA5093-OtA5097
Dnm1l	chr16:16312232-16359030	OtA5088-OtA5092	OtA5093-OtA5097
Selplg	chr5:113817797-113830501	OtA5088-OtA5092	OtA5093-OtA5097
Jagn1	chr6:113442516-113448229	OtA5088-OtA5092	OtA5093-OtA5097
Slc25a42	chr8:70184339-70212281	OtA5088-OtA5092	OtA5093-OtA5097
Gjb1	chrX:101377336-101385624	OtA5088-OtA5092	OtA5093-OtA5097
Pls3	chrX:75785653-75875170	OtA5088-OtA5092	OtA5093-OtA5097
Abp1	chr6:48895254-48909187	OtA5088-OtA5092	OtA5093-OtA5097
Cldn7	chr11:69964778-69967886	OtA5088-OtA5092	OtA5093-OtA5097
Hist1h2bj	chr13:22043229-22043610	OtA5088-OtA5092	OtA5093-OtA5097
Cxcr2	chr1:74153993-74161246	OtA5088-OtA5092	OtA5093-OtA5097
Mcf2l	chr8:12915892-13020509	OtA5088-OtA5092	OtA5093-OtA5097

Acads	chr5:115110298-115119313	OtA5088-OtA5092	OtA5093-OtA5097
Pcf11	chr7:92643711-92669912	OtA5088-OtA5092	OtA5093-OtA5097
Serpinf2	chr11:75431735-75439501	OtA5088-OtA5092	OtA5093-OtA5097
Spry4	chr18:38586264-38601268	OtA5088-OtA5092	OtA5093-OtA5097
Zc3h7a	chr16:11136593-11176393	OtA5088-OtA5092	OtA5093-OtA5097
Slc7a2	chr8:40862366-40922070	OtA5088-OtA5092	OtA5093-OtA5097
Vim	chr2:13574310-13582826	OtA5088-OtA5092	OtA5093-OtA5097
Hivep2	chr10:13966378-14151378	OtA5088-OtA5092	OtA5093-OtA5097
Fam193a	chr5:34369932-34486458	OtA5088-OtA5092	OtA5093-OtA5097
Atmin	chr8:116943392-116960445	OtA5088-OtA5092	OtA5093-OtA5097
Slco5a1	chr1:12866549-12991135	OtA5088-OtA5092	OtA5093-OtA5097
Ncf2	chr1:152807889-152836990	OtA5088-OtA5092	OtA5093-OtA5097
Slc45a4	chr15:73580290-73624744	OtA5088-OtA5092	OtA5093-OtA5097
Fbxo3	chr2:104027798-104063237	OtA5088-OtA5092	OtA5093-OtA5097
Mapk8ip3	chr17:24896499-24936977	OtA5088-OtA5092	OtA5093-OtA5097
Usp47	chr7:112023505-112111386	OtA5088-OtA5092	OtA5093-OtA5097
Hist1h3h	chr13:21717627-21718115	OtA5088-OtA5092	OtA5093-OtA5097
Hook3	chr8:26021420-26119224	OtA5088-OtA5092	OtA5093-OtA5097
Gpbp1l1	chr4:116557726-116600266	OtA5088-OtA5092	OtA5093-OtA5097
Cpeb4	chr11:31872210-31931577	OtA5088-OtA5092	OtA5093-OtA5097
Grasp	chr15:101221190-101232756	OtA5088-OtA5092	OtA5093-OtA5097
Grik5	chr7:25009850-25072369	OtA5088-OtA5092	OtA5093-OtA5097
Ncoa6	chr2:155390655-155440783	OtA5088-OtA5092	OtA5093-OtA5097
Lhx2	chr2:38351307-38369728	OtA5088-OtA5092	OtA5093-OtA5097
Dennd2c	chr3:103127555-103169733	OtA5088-OtA5092	OtA5093-OtA5097
Wnk4	chr11:101260566-101277409	OtA5088-OtA5092	OtA5093-OtA5097
Tyk2	chr9:21104067-21131275	OtA5088-OtA5092	OtA5093-OtA5097
Gcn1l1	chr5:115565262-115622654	OtA5088-OtA5092	OtA5093-OtA5097
Fam118a	chr15:85037061-85062830	OtA5088-OtA5092	OtA5093-OtA5097
Nr4a1	chr15:101266845-101274794	OtA5088-OtA5092	OtA5093-OtA5097
Asxl2	chr12:3426856-3506849	OtA5088-OtA5092	OtA5093-OtA5097
Cgn	chr3:94760069-94786515	OtA5088-OtA5092	OtA5093-OtA5097
Chtf8	chr8:106883862-106893593	OtA5088-OtA5092	OtA5093-OtA5097
Ddost	chr4:138304737-138312611	OtA5088-OtA5092	OtA5093-OtA5097
Foxq1	chr13:31558169-31560974	OtA5088-OtA5092	OtA5093-OtA5097
Gltscr1	chr7:15971261-15999495	OtA5088-OtA5092	OtA5093-OtA5097
Hoxa10	chr6:52231196-52240854	OtA5088-OtA5092	OtA5093-OtA5097
Polk	chr13:96480688-96542485	OtA5088-OtA5092	OtA5093-OtA5097
Syt11	chr3:88744700-88772599	OtA5088-OtA5092	OtA5093-OtA5097
Slc30a5	chr13:100802647-100833427	OtA5088-OtA5092	OtA5093-OtA5097
Atp6v0e2	chr6:48537568-48541800	OtA5088-OtA5092	OtA5093-OtA5097
Cited4	chr4:120666562-120667820	OtA5088-OtA5092	OtA5093-OtA5097
Gigyf2	chr1:87326997-87450810	OtA5088-OtA5092	OtA5093-OtA5097
Prdx5	chr19:6906818-6909645	OtA5088-OtA5092	OtA5093-OtA5097
Dqx1	chr6:83057843-83067219	OtA5088-OtA5092	OtA5093-OtA5097
Fuca1	chr4:135920725-135940300	OtA5088-OtA5092	OtA5093-OtA5097
Galc	chr12:98202299-98259337	OtA5088-OtA5092	OtA5093-OtA5097

Pi4kb	chr3:94974730-95015238	OtA5088-OtA5092	OtA5093-OtA5097
Tec	chr5:72755722-72868448	OtA5088-OtA5092	OtA5093-OtA5097
Socs2	chr10:95411489-95416857	OtA5088-OtA5092	OtA5093-OtA5097
Dus1l	chr11:120789201-120796395	OtA5088-OtA5092	OtA5093-OtA5097
Fbxo27	chr7:28692848-28699337	OtA5088-OtA5092	OtA5093-OtA5097
Nemf	chr12:69311542-69357176	OtA5088-OtA5092	OtA5093-OtA5097
Tuba4a	chr1:75210828-75219253	OtA5088-OtA5092	OtA5093-OtA5097
Fam133b	chr5:3543832-3570546	OtA5088-OtA5092	OtA5093-OtA5097
Dok2	chr14:70774380-70778494	OtA5088-OtA5092	OtA5093-OtA5097
Nubpl	chr12:52097745-52310959	OtA5088-OtA5092	OtA5093-OtA5097
Tnrc6c	chr11:117654288-117763441	OtA5088-OtA5092	OtA5093-OtA5097
Gemin4	chr11:76210570-76217572	OtA5088-OtA5092	OtA5093-OtA5097
Igdcc4	chr9:65101494-65137943	OtA5088-OtA5092	OtA5093-OtA5097
Tm9sf1	chr14:55635965-55643806	OtA5088-OtA5092	OtA5093-OtA5097
Adam15	chr3:89339639-89350010	OtA5088-OtA5092	OtA5093-OtA5097
Atrn	chr2:130906495-131030326	OtA5088-OtA5092	OtA5093-OtA5097
Colec12	chr18:9707647-9877995	OtA5088-OtA5092	OtA5093-OtA5097
Etohi1	chr2:178023283-178035859	OtA5088-OtA5092	OtA5093-OtA5097
Il17rd	chr14:27039000-27107286	OtA5088-OtA5092	OtA5093-OtA5097
Plk1s1	chr2:146855888-146970089	OtA5088-OtA5092	OtA5093-OtA5097
Slc30a6	chr17:74395607-74424229	OtA5088-OtA5092	OtA5093-OtA5097
Inmt	chr6:55170626-55174990	OtA5088-OtA5092	OtA5093-OtA5097
Slfn5	chr11:82911252-82964850	OtA5088-OtA5092	OtA5093-OtA5097
Calm3	chr7:16915378-16924032	OtA5088-OtA5092	OtA5093-OtA5097
Osbpl6	chr2:76406537-76600647	OtA5088-OtA5092	OtA5093-OtA5097
Zeb1	chr18:5491500-5775468	OtA5088-OtA5092	OtA5093-OtA5097
Efna4	chr3:89333392-89338028	OtA5088-OtA5092	OtA5093-OtA5097
Parp1	chr1:180568974-180601254	OtA5088-OtA5092	OtA5093-OtA5097
Slc39a8	chr3:135825490-135888572	OtA5088-OtA5092	OtA5093-OtA5097
C3ar1	chr6:122847139-122856157	OtA5088-OtA5092	OtA5093-OtA5097
Acad11	chr9:104063702-104127646	OtA5088-OtA5092	OtA5093-OtA5097
Sec63	chr10:42761495-42832514	OtA5088-OtA5092	OtA5093-OtA5097
Whsc1l1	chr8:25602305-25719667	OtA5088-OtA5092	OtA5093-OtA5097
Cul3	chr1:80266817-80340430	OtA5088-OtA5092	OtA5093-OtA5097
Cwc22	chr2:77895652-77946353	OtA5088-OtA5092	OtA5093-OtA5097
Nav2	chr7:48959072-49610088	OtA5088-OtA5092	OtA5093-OtA5097
Stmn1	chr4:134468319-134473843	OtA5088-OtA5092	OtA5093-OtA5097
Galnt11	chr5:25222892-25265918	OtA5088-OtA5092	OtA5093-OtA5097
Pwp2	chr10:78170909-78185149	OtA5088-OtA5092	OtA5093-OtA5097
Dapk1	chr13:60601946-60763186	OtA5088-OtA5092	OtA5093-OtA5097
Zfp710	chr7:80024813-80092751	OtA5088-OtA5092	OtA5093-OtA5097
Ada	chr2:163726583-163750177	OtA5088-OtA5092	OtA5093-OtA5097
Atxn7	chr14:14012490-14107301	OtA5088-OtA5092	OtA5093-OtA5097
Dmxl1	chr18:49832996-49965473	OtA5088-OtA5092	OtA5093-OtA5097
Eif2b5	chr16:20498816-20509325	OtA5088-OtA5092	OtA5093-OtA5097
Gsn	chr2:35256358-35307902	OtA5088-OtA5092	OtA5093-OtA5097
Herc1	chr9:66350449-66514593	OtA5088-OtA5092	OtA5093-OtA5097

Iah1	chr12:21316391-21373591	OtA5088-OtA5092	OtA5093-OtA5097
Jmjd5	chr7:125444619-125462268	OtA5088-OtA5092	OtA5093-OtA5097
Lpar1	chr4:58435251-58553491	OtA5088-OtA5092	OtA5093-OtA5097
Mn1	chr5:111418165-111457025	OtA5088-OtA5092	OtA5093-OtA5097
Plekhg1	chr10:3740376-3967302	OtA5088-OtA5092	OtA5093-OtA5097
Dnajc16	chr4:141761997-141790644	OtA5088-OtA5092	OtA5093-OtA5097
Cks1b	chr3:89415471-89418291	OtA5088-OtA5092	OtA5093-OtA5097
Cpn1	chr19:43956307-43986520	OtA5088-OtA5092	OtA5093-OtA5097
Fam129b	chr2:32876133-32961255	OtA5088-OtA5092	OtA5093-OtA5097
Mdn1	chr4:32657118-32775217	OtA5088-OtA5092	OtA5093-OtA5097
Mtap1b	chr13:99421463-99516602	OtA5088-OtA5092	OtA5093-OtA5097
Tmcc1	chr6:116018617-116193374	OtA5088-OtA5092	OtA5093-OtA5097
Dynlt1b	chr17:6430111-6436295	OtA5088-OtA5092	OtA5093-OtA5097
Lrp6	chr6:134446477-134566913	OtA5088-OtA5092	OtA5093-OtA5097
Loxl4	chr19:42592278-42612806	OtA5088-OtA5092	OtA5093-OtA5097
Stat2	chr10:128270575-128292849	OtA5088-OtA5092	OtA5093-OtA5097
Slc38a10	chr11:120103950-120151351	OtA5088-OtA5092	OtA5093-OtA5097
Casd1	chr6:4601065-4643381	OtA5088-OtA5092	OtA5093-OtA5097
Stard5	chr7:83632016-83642328	OtA5088-OtA5092	OtA5093-OtA5097
Llgl2	chr11:115824057-115855780	OtA5088-OtA5092	OtA5093-OtA5097
Mettl7a1	chr15:100304816-100314348	OtA5088-OtA5092	OtA5093-OtA5097
Lmcd1	chr6:112273757-112330423	OtA5088-OtA5092	OtA5093-OtA5097
Shroom4	chrX:6400144-6633717	OtA5088-OtA5092	OtA5093-OtA5097
R3hdm1	chr1:128103305-128237735	OtA5088-OtA5092	OtA5093-OtA5097
Ghr	chr15:3317759-3583230	OtA5088-OtA5092	OtA5093-OtA5097
Pvrl2	chr7:19716643-19749573	OtA5088-OtA5092	OtA5093-OtA5097
Rbm39	chr2:156147240-156180240	OtA5088-OtA5092	OtA5093-OtA5097
Specc1	chr11:62077096-62223013	OtA5088-OtA5092	OtA5093-OtA5097
Adck1	chr12:88360800-88461718	OtA5088-OtA5092	OtA5093-OtA5097
Irx4	chr13:73260496-73269620	OtA5088-OtA5092	OtA5093-OtA5097
P4ha3	chr7:100285519-100319699	OtA5088-OtA5092	OtA5093-OtA5097
Sall1	chr8:89027242-89044162	OtA5088-OtA5092	OtA5093-OtA5097
4-Sep	chr11:87581130-87590539	OtA5088-OtA5092	OtA5093-OtA5097
Tmem181b-ps	chr17:6439001-6450994	OtA5088-OtA5092	OtA5093-OtA5097
Fam168b	chr1:34813217-34843050	OtA5088-OtA5092	OtA5093-OtA5097
Nfib	chr4:82290172-82505308	OtA5088-OtA5092	OtA5093-OtA5097
Gm15800	chr5:121220218-121368577	OtA5088-OtA5092	OtA5093-OtA5097
Ftsjd2	chr17:29660600-29717017	OtA5088-OtA5092	OtA5093-OtA5097
Adss	chr1:177763177-177796509	OtA5088-OtA5092	OtA5093-OtA5097
Nufip2	chr11:77686138-77717966	OtA5088-OtA5092	OtA5093-OtA5097
Osbp2	chr11:3703730-3863903	OtA5088-OtA5092	OtA5093-OtA5097
Tgfbr1	chr4:47353304-47414924	OtA5088-OtA5092	OtA5093-OtA5097
Tmem39a	chr16:38558697-38592162	OtA5088-OtA5092	OtA5093-OtA5097
Tnfrsf21	chr17:43016554-43089188	OtA5088-OtA5092	OtA5093-OtA5097
Vtn	chr11:78499119-78502325	OtA5088-OtA5092	OtA5093-OtA5097
Zcchc8	chr5:123698301-123721044	OtA5088-OtA5092	OtA5093-OtA5097
Gjb4	chr4:127351085-127354081	OtA5088-OtA5092	OtA5093-OtA5097

Mocos	chr18:24653690-24701556	OtA5088-OtA5092	OtA5093-OtA5097
Pla2r1	chr2:60417542-60553308	OtA5088-OtA5092	OtA5093-OtA5097
Ly75	chr2:60293759-60383231	OtA5088-OtA5092	OtA5093-OtA5097
Gm13889	chr2:93955809-93957100	OtA5088-OtA5092	OtA5093-OtA5097
Srpk1	chr17:28589591-28622454	OtA5088-OtA5092	OtA5093-OtA5097
Pigt	chr2:164497524-164508301	OtA5088-OtA5092	OtA5093-OtA5097
Hn1l	chr17:24942469-24960623	OtA5088-OtA5092	OtA5093-OtA5097
Ankrd23	chr1:36530533-36535729	OtA5088-OtA5092	OtA5093-OtA5097
Tnxb	chr17:34670534-34719815	OtA5088-OtA5092	OtA5093-OtA5097
Luzp1	chr4:136469760-136549318	OtA5088-OtA5092	OtA5093-OtA5097
Lhx6	chr2:36081952-36105408	OtA5088-OtA5092	OtA5093-OtA5097
Tpr	chr1:150361310-150466165	OtA5088-OtA5092	OtA5093-OtA5097
Tor2a	chr2:32757233-32775383	OtA5088-OtA5092	OtA5093-OtA5097
Twsg1	chr17:65923064-65951187	OtA5088-OtA5092	OtA5093-OtA5097
Adprh	chr16:38445398-38452689	OtA5088-OtA5092	OtA5093-OtA5097
BC033916	chr17:33905134-33906675	OtA5088-OtA5092	OtA5093-OtA5097
Rnd3	chr2:51130438-51149111	OtA5088-OtA5092	OtA5093-OtA5097
Slc25a3	chr10:91116577-91123963	OtA5088-OtA5092	OtA5093-OtA5097
Gys1	chr7:45434838-45456617	OtA5088-OtA5092	OtA5093-OtA5097
Bend6	chr1:33852051-33907621	OtA5088-OtA5092	OtA5093-OtA5097
Fkbp6	chr5:135291703-135349991	OtA5088-OtA5092	OtA5093-OtA5097
Prelid1	chr13:55322054-55329730	OtA5088-OtA5092	OtA5093-OtA5097
Gas1	chr13:60174404-60177535	OtA5088-OtA5092	OtA5093-OtA5097
Acaa2	chr18:74779211-74806207	OtA5088-OtA5092	OtA5093-OtA5097
Asap1	chr15:64088538-64382919	OtA5088-OtA5092	OtA5093-OtA5097
Cdc5l	chr17:45391887-45433707	OtA5088-OtA5092	OtA5093-OtA5097
Clec2h	chr6:128662384-128677374	OtA5088-OtA5092	OtA5093-OtA5097
Klhl21	chr4:152008890-152017677	OtA5088-OtA5092	OtA5093-OtA5097
Ptprn2	chr12:116485719-117337012	OtA5088-OtA5092	OtA5093-OtA5097
Rasgef1b	chr5:99217419-99252927	OtA5088-OtA5092	OtA5093-OtA5097
Srrm1	chr4:135320483-135353214	OtA5088-OtA5092	OtA5093-OtA5097
Stag2	chrX:42149411-42277175	OtA5088-OtA5092	OtA5093-OtA5097
Lepre1	chr4:119232914-119248977	OtA5088-OtA5092	OtA5093-OtA5097
Pgm2l1	chr7:100227606-100278872	OtA5088-OtA5092	OtA5093-OtA5097
Eif2ak4	chr2:118388616-118475234	OtA5088-OtA5092	OtA5093-OtA5097
Brms1l	chr12:55836365-55869735	OtA5088-OtA5092	OtA5093-OtA5097
Mustn1	chr14:30879256-30881610	OtA5088-OtA5092	OtA5093-OtA5097
Csde1	chr3:103020545-103058189	OtA5088-OtA5092	OtA5093-OtA5097
Arid4b	chr13:14063791-14199603	OtA5088-OtA5092	OtA5093-OtA5097
Clec1a	chr6:129426683-129452000	OtA5088-OtA5092	OtA5093-OtA5097
Enoph1	chr5:100039993-100068765	OtA5088-OtA5092	OtA5093-OtA5097
Jag1	chr2:137081450-137116520	OtA5088-OtA5092	OtA5093-OtA5097
Myl7	chr11:5896636-5898782	OtA5088-OtA5092	OtA5093-OtA5097
Pvrl1	chr9:43744575-43807461	OtA5088-OtA5092	OtA5093-OtA5097
Slc48a1	chr15:97784364-97844502	OtA5088-OtA5092	OtA5093-OtA5097
Zcchc14	chr8:121598702-121651933	OtA5088-OtA5092	OtA5093-OtA5097
Slc27a1	chr8:71568926-71586708	OtA5088-OtA5092	OtA5093-OtA5097

Steap3	chr1:120226415-120271082	OtA5088-OtA5092	OtA5093-OtA5097
Tep1	chr14:50824060-50870554	OtA5088-OtA5092	OtA5093-OtA5097
Prpf4b	chr13:34875493-34902878	OtA5088-OtA5092	OtA5093-OtA5097
Ankrd12	chr17:65967500-66077046	OtA5088-OtA5092	OtA5093-OtA5097
Ddit4	chr10:59949674-59951770	OtA5088-OtA5092	OtA5093-OtA5097
Tjp1	chr7:65296164-65371244	OtA5088-OtA5092	OtA5093-OtA5097
Atp6v0c	chr17:24163864-24169429	OtA5088-OtA5092	OtA5093-OtA5097
Atxn1	chr13:45549755-45964991	OtA5088-OtA5092	OtA5093-OtA5097
Crebbp	chr16:4084047-4213404	OtA5088-OtA5092	OtA5093-OtA5097
Hebp1	chr6:135137518-135168215	OtA5088-OtA5092	OtA5093-OtA5097
Hipk1	chr3:103739814-103791275	OtA5088-OtA5092	OtA5093-OtA5097
Hist1h2bl	chr13:21715762-21716143	OtA5088-OtA5092	OtA5093-OtA5097
Map2k6	chr11:110399121-110513637	OtA5088-OtA5092	OtA5093-OtA5097
Pcgf3	chr5:108461331-108503099	OtA5088-OtA5092	OtA5093-OtA5097
Rin2	chr2:145786115-145887616	OtA5088-OtA5092	OtA5093-OtA5097
Ttc39a	chr4:109407099-109444745	OtA5088-OtA5092	OtA5093-OtA5097
Ube2q2	chr9:55149368-55207529	OtA5088-OtA5092	OtA5093-OtA5097
Apoa2	chr1:171225053-171226379	OtA5088-OtA5092	OtA5093-OtA5097
Irak1	chrX:74013913-74023936	OtA5088-OtA5092	OtA5093-OtA5097
Rabgap1l	chr1:160219173-160792938	OtA5088-OtA5092	OtA5093-OtA5097
Osbpl7	chr11:97050819-97068904	OtA5088-OtA5092	OtA5093-OtA5097
Cnot6l	chr5:96075737-96161990	OtA5088-OtA5092	OtA5093-OtA5097
Dpep1	chr8:123186234-123201813	OtA5088-OtA5092	OtA5093-OtA5097
Ramp1	chr1:91179821-91225196	OtA5088-OtA5092	OtA5093-OtA5097
Ept1	chr5:30232617-30272430	OtA5088-OtA5092	OtA5093-OtA5097
Itih2	chr2:10094590-10130683	OtA5088-OtA5092	OtA5093-OtA5097
Zbtb11	chr16:55973267-56008912	OtA5088-OtA5092	OtA5093-OtA5097
Cfp	chrX:20925534-20931524	OtA5088-OtA5092	OtA5093-OtA5097
Chd9	chr8:90828834-91054508	OtA5088-OtA5092	OtA5093-OtA5097
Fbxo32	chr15:58175878-58214892	OtA5088-OtA5092	OtA5093-OtA5097
Gpi1	chr7:34201326-34230305	OtA5088-OtA5092	OtA5093-OtA5097
Pkm2	chr9:59656367-59679375	OtA5088-OtA5092	OtA5093-OtA5097
Rad54l2	chr9:106688079-106789213	OtA5088-OtA5092	OtA5093-OtA5097
Calcrl	chr2:84330626-84425266	OtA5088-OtA5092	OtA5093-OtA5097
Fam135a	chr1:24010757-24100341	OtA5088-OtA5092	OtA5093-OtA5097
Tmprss2	chr16:97564681-97611195	OtA5088-OtA5092	OtA5093-OtA5097
Eif2d	chr1:131153206-131173471	OtA5088-OtA5092	OtA5093-OtA5097
Fgfr1op2	chr6:146577910-146599198	OtA5088-OtA5092	OtA5093-OtA5097
Patl1	chr19:11912398-11945096	OtA5088-OtA5092	OtA5093-OtA5097
Per3	chr4:151003654-151044622	OtA5088-OtA5092	OtA5093-OtA5097
Pigc	chr1:161942088-161979636	OtA5088-OtA5092	OtA5093-OtA5097
Plxnd1	chr6:115954810-115995005	OtA5088-OtA5092	OtA5093-OtA5097
Zfp292	chr4:34803109-34882948	OtA5088-OtA5092	OtA5093-OtA5097
Fam53b	chr7:132712083-132813196	OtA5088-OtA5092	OtA5093-OtA5097
Cd59a	chr2:104095800-104115351	OtA5088-OtA5092	OtA5093-OtA5097
Rhof	chr5:123118179-123132629	OtA5088-OtA5092	OtA5093-OtA5097
Tmem120b	chr5:123076274-123117445	OtA5088-OtA5092	OtA5093-OtA5097

Trip6	chr5:137309898-137314241	OtA5088-OtA5092	OtA5093-OtA5097
Nucb2	chr7:116504373-116540588	OtA5088-OtA5092	OtA5093-OtA5097
Ttc3	chr16:94358762-94469221	OtA5088-OtA5092	OtA5093-OtA5097
Aspscr1	chr11:120672972-120709446	OtA5088-OtA5092	OtA5093-OtA5097
Plb1	chr5:32232707-32364356	OtA5088-OtA5092	OtA5093-OtA5097
Rnh1	chr7:141160325-141172851	OtA5088-OtA5092	OtA5093-OtA5097
Slc23a2	chr2:132052495-132145108	OtA5088-OtA5092	OtA5093-OtA5097
Trip12	chr1:84721188-84839304	OtA5088-OtA5092	OtA5093-OtA5097
Setd5	chr6:113077638-113153424	OtA5088-OtA5092	OtA5093-OtA5097
Slc22a23	chr13:34179157-34345182	OtA5088-OtA5092	OtA5093-OtA5097
Gaa	chr11:119267966-119285698	OtA5088-OtA5092	OtA5093-OtA5097
Ppp4r2	chr6:100833637-100868717	OtA5088-OtA5092	OtA5093-OtA5097
Fblim1	chr4:141576061-141606052	OtA5088-OtA5092	OtA5093-OtA5097
Zfp362	chr4:128773084-128806112	OtA5088-OtA5092	OtA5093-OtA5097
Naglu	chr11:101070093-101077671	OtA5088-OtA5092	OtA5093-OtA5097
Npy	chr6:49822728-49829505	OtA5088-OtA5092	OtA5093-OtA5097
Rnu12	chr15:83149644-83149794	OtA5088-OtA5092	OtA5093-OtA5097
Atp6v0d2	chr4:19876837-19922566	OtA5088-OtA5092	OtA5093-OtA5097
Lrp12	chr15:39870602-39943757	OtA5088-OtA5092	OtA5093-OtA5097
Mif	chr10:75859352-75860250	OtA5088-OtA5092	OtA5093-OtA5097
Sox17	chr1:4490927-4496413	OtA5088-OtA5092	OtA5093-OtA5097
Ttf1	chr2:29060262-29087650	OtA5088-OtA5092	OtA5093-OtA5097
Ehhadh	chr16:21761284-21787834	OtA5088-OtA5092	OtA5093-OtA5097
Tram2	chr1:21001377-21079225	OtA5088-OtA5092	OtA5093-OtA5097
Zfp318	chr17:46383765-46420918	OtA5088-OtA5092	OtA5093-OtA5097
Kdm5b	chr1:134560177-134632878	OtA5088-OtA5092	OtA5093-OtA5097
Slc22a3	chr17:12419973-12507704	OtA5088-OtA5092	OtA5093-OtA5097
Sdhd	chr9:50596339-50603849	OtA5088-OtA5092	OtA5093-OtA5097
Enpep	chr3:129269176-129332749	OtA5088-OtA5092	OtA5093-OtA5097
Inhbb	chr1:119415464-119422248	OtA5088-OtA5092	OtA5093-OtA5097
Ppp1r7	chr1:93343644-93367618	OtA5088-OtA5092	OtA5093-OtA5097
Luc7l3	chr11:94291138-94321911	OtA5088-OtA5092	OtA5093-OtA5097
Abcg2	chr6:58596671-58692451	OtA5088-OtA5092	OtA5093-OtA5097
Hic1	chr11:75164564-75170255	OtA5088-OtA5092	OtA5093-OtA5097
Psg18	chr7:18345807-18355007	OtA5088-OtA5092	OtA5093-OtA5097
Aff3	chr1:38177326-38664955	OtA5088-OtA5092	OtA5093-OtA5097
Pawr	chr10:108332188-108414391	OtA5088-OtA5092	OtA5093-OtA5097
Psca	chr15:74714838-74717065	OtA5088-OtA5092	OtA5093-OtA5097
Rhox9	chrX:37899096-37901770	OtA5088-OtA5092	OtA5093-OtA5097
Grn	chr11:102430321-102436809	OtA5088-OtA5092	OtA5093-OtA5097
Nlrc5	chr8:94472762-94527272	OtA5088-OtA5092	OtA5093-OtA5097
Ddhd1	chr14:45593173-45658143	OtA5088-OtA5092	OtA5093-OtA5097
L3mbtl2	chr15:81663935-81697287	OtA5088-OtA5092	OtA5093-OtA5097
Ciapin1	chr8:94819817-94838340	OtA5088-OtA5092	OtA5093-OtA5097
Cdk13	chr13:17715961-17805097	OtA5088-OtA5092	OtA5093-OtA5097
Hmbs	chr9:44336347-44344228	OtA5088-OtA5092	OtA5093-OtA5097
Prmt5	chr14:54507181-54517470	OtA5088-OtA5092	OtA5093-OtA5097

Slfn2	chr11:83065111-83070678	OtA5088-OtA5092	OtA5093-OtA5097
Metap2	chr10:93858489-93891202	OtA5088-OtA5092	OtA5093-OtA5097
Crabp1	chr9:54764807-54773109	OtA5088-OtA5092	OtA5093-OtA5097
Rangap1	chr15:81704247-81729919	OtA5088-OtA5092	OtA5093-OtA5097
Abhd12	chr2:150832514-150904731	OtA5088-OtA5092	OtA5093-OtA5097
Tmem102	chr11:69803594-69806038	OtA5088-OtA5092	OtA5093-OtA5097
Sfr1	chr19:47731755-47735588	OtA5088-OtA5092	OtA5093-OtA5097
Pou2f1	chr1:165788680-166002634	OtA5088-OtA5092	OtA5093-OtA5097
Parp14	chr16:35832877-35871382	OtA5088-OtA5092	OtA5093-OtA5097
Ganab	chr19:8898110-8916665	OtA5088-OtA5092	OtA5093-OtA5097
Tex19.1	chr11:121146142-121148313	OtA5088-OtA5092	OtA5093-OtA5097
Itga7	chr10:128933812-128960988	OtA5088-OtA5092	OtA5093-OtA5097
Cbx7	chr15:79915806-79932646	OtA5088-OtA5092	OtA5093-OtA5097
Kras	chr6:145216698-145250231	OtA5088-OtA5092	OtA5093-OtA5097
Mast4	chr13:102732488-103334492	OtA5088-OtA5092	OtA5093-OtA5097
Mecp2	chrX:74026823-74085636	OtA5088-OtA5092	OtA5093-OtA5097
Kitl	chr10:100015823-100100412	OtA5088-OtA5092	OtA5093-OtA5097
Ell2	chr13:75707483-75772358	OtA5088-OtA5092	OtA5093-OtA5097
Hist2h2bb	chr3:96269699-96270192	OtA5088-OtA5092	OtA5093-OtA5097
Napa	chr7:16098642-16117975	OtA5088-OtA5092	OtA5093-OtA5097
Osgep	chr14:50915373-50924893	OtA5088-OtA5092	OtA5093-OtA5097
Sema6d	chr2:124610295-124667770	OtA5088-OtA5092	OtA5093-OtA5097
Herpud1	chr8:94386499-94395358	OtA5088-OtA5092	OtA5093-OtA5097
Ctcf	chr8:105636537-105682922	OtA5088-OtA5092	OtA5093-OtA5097
Lrrc42	chr4:107233513-107253533	OtA5088-OtA5092	OtA5093-OtA5097
Pank3	chr11:35769494-35791285	OtA5088-OtA5092	OtA5093-OtA5097
Perp	chr10:18845070-18857072	OtA5088-OtA5092	OtA5093-OtA5097
Rmnd5a	chr6:71388633-71440637	OtA5088-OtA5092	OtA5093-OtA5097
Sh3d19	chr3:86084433-86130521	OtA5088-OtA5092	OtA5093-OtA5097
Farp1	chr14:121035573-121283726	OtA5088-OtA5092	OtA5093-OtA5097
Atp5b	chr10:128083306-128090388	OtA5088-OtA5092	OtA5093-OtA5097
Gnaq	chr19:16132830-16387453	OtA5088-OtA5092	OtA5093-OtA5097
Grrp1	chr4:134251109-134254106	OtA5088-OtA5092	OtA5093-OtA5097
Hexa	chr9:59539666-59565105	OtA5088-OtA5092	OtA5093-OtA5097
Plekha2	chr8:25039143-25101811	OtA5088-OtA5092	OtA5093-OtA5097
Rasd2	chr8:75213943-75224113	OtA5088-OtA5092	OtA5093-OtA5097
Stim2	chr5:53998522-54121057	OtA5088-OtA5092	OtA5093-OtA5097
Abtb2	chr2:103566309-103718423	OtA5088-OtA5092	OtA5093-OtA5097
Atp6ap1	chrX:74297096-74304721	OtA5088-OtA5092	OtA5093-OtA5097
Dgkz	chr2:91932821-91963563	OtA5088-OtA5092	OtA5093-OtA5097
Dhx40	chr11:86768848-86807660	OtA5088-OtA5092	OtA5093-OtA5097
Fam105a	chr15:27655070-27681542	OtA5088-OtA5092	OtA5093-OtA5097
Fem1c	chr18:46504605-46525971	OtA5088-OtA5092	OtA5093-OtA5097
Got1	chr19:43499752-43524605	OtA5088-OtA5092	OtA5093-OtA5097
Gpaa1	chr15:76331293-76334899	OtA5088-OtA5092	OtA5093-OtA5097
Lass6	chr2:68861556-69111290	OtA5088-OtA5092	OtA5093-OtA5097
Lrrc41	chr4:116075464-116123690	OtA5088-OtA5092	OtA5093-OtA5097

Mcm5	chr8:75109527-75128439	OtA5088-OtA5092	OtA5093-OtA5097
Ovol1	chr19:5549136-5560575	OtA5088-OtA5092	OtA5093-OtA5097
Ppa1	chr10:61648620-61674165	OtA5088-OtA5092	OtA5093-OtA5097
Rfx7	chr9:72532239-72622949	OtA5088-OtA5092	OtA5093-OtA5097
Srpx	chrX:10037976-10117661	OtA5088-OtA5092	OtA5093-OtA5097
Zranb1	chr7:132949621-132983951	OtA5088-OtA5092	OtA5093-OtA5097
Emp1	chr6:135362930-135383173	OtA5088-OtA5092	OtA5093-OtA5097
Shroom1	chr11:53457204-53467755	OtA5088-OtA5092	OtA5093-OtA5097
Slc38a7	chr8:95835922-95853491	OtA5088-OtA5092	OtA5093-OtA5097
Snx17	chr5:31193303-31198900	OtA5088-OtA5092	OtA5093-OtA5097
Spsb4	chr9:96943481-97018355	OtA5088-OtA5092	OtA5093-OtA5097
Cspp1	chr1:10038217-10136768	OtA5088-OtA5092	OtA5093-OtA5097
Dpp3	chr19:4907228-4928287	OtA5088-OtA5092	OtA5093-OtA5097
Epn3	chr11:94489598-94499974	OtA5088-OtA5092	OtA5093-OtA5097
H2-K2	chr17:33974658-33978791	OtA5088-OtA5092	OtA5093-OtA5097
Lmnb1	chr18:56707812-56753424	OtA5088-OtA5092	OtA5093-OtA5097
Ppp1cc	chr5:122158278-122175269	OtA5088-OtA5092	OtA5093-OtA5097
Rpp38	chr2:3328948-3332628	OtA5088-OtA5092	OtA5093-OtA5097
Plekho2	chr9:65554383-65580034	OtA5088-OtA5092	OtA5093-OtA5097
Synj2	chr17:5941279-6079739	OtA5088-OtA5092	OtA5093-OtA5097
Gpr126	chr10:14402584-14545036	OtA5088-OtA5092	OtA5093-OtA5097
C130074G19Rik	chr1:184871925-184883036	OtA5088-OtA5092	OtA5093-OtA5097
Tspan13	chr12:36014554-36042478	OtA5088-OtA5092	OtA5093-OtA5097
Slit3	chr11:35121455-35708507	OtA5088-OtA5092	OtA5093-OtA5097
St3gal1	chr15:67102874-67176882	OtA5088-OtA5092	OtA5093-OtA5097
Mybl2	chr2:163054634-163084687	OtA5088-OtA5092	OtA5093-OtA5097
Cad	chr5:31054810-31078479	OtA5088-OtA5092	OtA5093-OtA5097
Des	chr1:75360343-75367715	OtA5088-OtA5092	OtA5093-OtA5097
Mga	chr2:119897227-119969581	OtA5088-OtA5092	OtA5093-OtA5097
Ahcyl1	chr3:107663119-107696548	OtA5088-OtA5092	OtA5093-OtA5097
BC046404	chr11:62604883-62648523	OtA5088-OtA5092	OtA5093-OtA5097
Cdc73	chr1:143607498-143702684	OtA5088-OtA5092	OtA5093-OtA5097
Cdipt	chr7:126976348-126980501	OtA5088-OtA5092	OtA5093-OtA5097
Dao	chr5:114003825-114025460	OtA5088-OtA5092	OtA5093-OtA5097
Esf1	chr2:140119880-140170558	OtA5088-OtA5092	OtA5093-OtA5097
Fam49b	chr15:63929093-64060448	OtA5088-OtA5092	OtA5093-OtA5097
Fbxo25	chr8:13907805-13940521	OtA5088-OtA5092	OtA5093-OtA5097
Pcsk5	chr19:17432831-17837632	OtA5088-OtA5092	OtA5093-OtA5097
Rps13	chr7:116331506-116334190	OtA5088-OtA5092	OtA5093-OtA5097
Sgpp2	chr1:78310345-78420286	OtA5088-OtA5092	OtA5093-OtA5097
Prdm10	chr9:31315106-31378543	OtA5088-OtA5092	OtA5093-OtA5097
Iqcb1	chr16:36792883-36874826	OtA5088-OtA5092	OtA5093-OtA5097
Slc44a1	chr4:53440412-53622478	OtA5088-OtA5092	OtA5093-OtA5097
Fads2	chr19:10064163-10101503	OtA5088-OtA5092	OtA5093-OtA5097
Pqlc3	chr12:16988647-17000118	OtA5088-OtA5092	OtA5093-OtA5097
Dgat2	chr7:99153662-99182713	OtA5088-OtA5092	OtA5093-OtA5097
Zfp935	chr13:62453015-62466812	OtA5088-OtA5092	OtA5093-OtA5097

Pmp22	chr11:63131509-63159546	OtA5088-OtA5092	OtA5093-OtA5097
Naprt1	chr15:75890963-75894481	OtA5088-OtA5092	OtA5093-OtA5097
Ralbp1	chr17:65848427-65885755	OtA5088-OtA5092	OtA5093-OtA5097
Trak2	chr1:58900449-58973482	OtA5088-OtA5092	OtA5093-OtA5097
Mlycd	chr8:119394891-119411088	OtA5088-OtA5092	OtA5093-OtA5097
Mfsd12	chr10:81357569-81365820	OtA5088-OtA5092	OtA5093-OtA5097
Whsc1	chr5:33843111-33897966	OtA5088-OtA5092	OtA5093-OtA5097
Cpz	chr5:35502217-35525626	OtA5088-OtA5092	OtA5093-OtA5097
Dna2	chr10:62947028-62974188	OtA5088-OtA5092	OtA5093-OtA5097
Foxf2	chr13:31625815-31631406	OtA5088-OtA5092	OtA5093-OtA5097
Glipr1	chr10:111972694-111997264	OtA5088-OtA5092	OtA5093-OtA5097
Mgat2	chr12:69184157-69186773	OtA5088-OtA5092	OtA5093-OtA5097
Ppp5c	chr7:17004640-17027914	OtA5088-OtA5092	OtA5093-OtA5097
Psmd8	chr7:29174186-29180673	OtA5088-OtA5092	OtA5093-OtA5097
Zfp229	chr17:21733723-21748969	OtA5088-OtA5092	OtA5093-OtA5097
Dusp7	chr9:106368631-106375723	OtA5088-OtA5092	OtA5093-OtA5097
Epb4.1l4b	chr4:57061725-57143156	OtA5088-OtA5092	OtA5093-OtA5097
Fam110a	chr2:151969397-151980219	OtA5088-OtA5092	OtA5093-OtA5097
Fam57a	chr11:76202055-76208257	OtA5088-OtA5092	OtA5093-OtA5097
Lins	chr7:66689888-66717256	OtA5088-OtA5092	OtA5093-OtA5097
Tsen54	chr11:115814738-115823102	OtA5088-OtA5092	OtA5093-OtA5097
Smg5	chr3:88336259-88362337	OtA5088-OtA5092	OtA5093-OtA5097
Klf4	chr4:55527136-55532475	OtA5088-OtA5092	OtA5093-OtA5097
Smndc1	chr19:53379213-53390573	OtA5088-OtA5092	OtA5093-OtA5097
Camsap1	chr2:25926837-25983282	OtA5088-OtA5092	OtA5093-OtA5097
Gm12669	chr7:137437647-137468594	OtA5088-OtA5092	OtA5093-OtA5097
Sdc4	chr2:164424246-164443188	OtA5088-OtA5092	OtA5093-OtA5097
Pitpnm2	chr5:124118689-124216418	OtA5088-OtA5092	OtA5093-OtA5097
P4ha2	chr11:54100923-54131667	OtA5088-OtA5092	OtA5093-OtA5097
Zfp386	chr12:116047723-116060594	OtA5088-OtA5092	OtA5093-OtA5097
Nfyb	chr10:82748699-82764141	OtA5088-OtA5092	OtA5093-OtA5097
Jmjd6	chr11:116837431-116843449	OtA5088-OtA5092	OtA5093-OtA5097
Tbcd	chr11:121451948-121673151	OtA5088-OtA5092	OtA5093-OtA5097
Ehd1	chr19:6276895-6300096	OtA5088-OtA5092	OtA5093-OtA5097
Fcgr3	chr1:171051168-171059403	OtA5088-OtA5092	OtA5093-OtA5097
Itpr2	chr6:146111479-146501912	OtA5088-OtA5092	OtA5093-OtA5097
Nr3c1	chr18:39410544-39487245	OtA5088-OtA5092	OtA5093-OtA5097
B4galnt1	chr10:127165155-127172340	OtA5088-OtA5092	OtA5093-OtA5097
Dscr3	chr16:94497723-94526629	OtA5088-OtA5092	OtA5093-OtA5097
Gm13363	chr1:30940302-30949755	OtA5088-OtA5092	OtA5093-OtA5097
Jmy	chr13:93430096-93499808	OtA5088-OtA5092	OtA5093-OtA5097
Parp10	chr15:76232994-76243440	OtA5088-OtA5092	OtA5093-OtA5097
Sh3kbp1	chrX:159627407-159975917	OtA5088-OtA5092	OtA5093-OtA5097
Smarca2	chr19:26605159-26823907	OtA5088-OtA5092	OtA5093-OtA5097
Spry1	chr3:37639949-37644599	OtA5088-OtA5092	OtA5093-OtA5097
Taok1	chr11:77529161-77607815	OtA5088-OtA5092	OtA5093-OtA5097
Ubxn7	chr16:32332251-32393747	OtA5088-OtA5092	OtA5093-OtA5097

Ywhae	chr11:75732886-75765841	OtA5088-OtA5092	OtA5093-OtA5097
Pla2g4f	chr2:120299956-120314165	OtA5088-OtA5092	OtA5093-OtA5097
Idh3g	chrX:73778962-73786897	OtA5088-OtA5092	OtA5093-OtA5097
Nr2f6	chr8:71374118-71381952	OtA5088-OtA5092	OtA5093-OtA5097
Gmds	chr13:31819585-32338544	OtA5088-OtA5092	OtA5093-OtA5097
Ccdc51	chr9:109082495-109093363	OtA5088-OtA5092	OtA5093-OtA5097
Trex1	chr9:109057931-109059723	OtA5088-OtA5092	OtA5093-OtA5097
G6pc3	chr11:102189698-102194081	OtA5088-OtA5092	OtA5093-OtA5097
Hlcs	chr16:94129305-94287856	OtA5088-OtA5092	OtA5093-OtA5097
Kcnk1	chr8:125995101-126030688	OtA5088-OtA5092	OtA5093-OtA5097
Scnn1a	chr6:125321339-125344942	OtA5088-OtA5092	OtA5093-OtA5097
Slc35d1	chr4:103171717-103214884	OtA5088-OtA5092	OtA5093-OtA5097
Eftud2	chr11:102838471-102880975	OtA5088-OtA5092	OtA5093-OtA5097
Tmem147	chr7:30727700-30729534	OtA5088-OtA5092	OtA5093-OtA5097
Foxn3	chr12:99195093-99450074	OtA5088-OtA5092	OtA5093-OtA5097
Cpd	chr11:76777207-76847008	OtA5088-OtA5092	OtA5093-OtA5097
Csf1	chr3:107741047-107760469	OtA5088-OtA5092	OtA5093-OtA5097
Dak	chr19:10592196-10604258	OtA5088-OtA5092	OtA5093-OtA5097
Dync2h1	chr9:6928502-7177046	OtA5088-OtA5092	OtA5093-OtA5097
Ino80	chr2:119373041-119477629	OtA5088-OtA5092	OtA5093-OtA5097
Pstk	chr7:131371145-131387838	OtA5088-OtA5092	OtA5093-OtA5097
Sap130	chr18:31634382-31723061	OtA5088-OtA5092	OtA5093-OtA5097
Set	chr2:30061995-30072577	OtA5088-OtA5092	OtA5093-OtA5097
Taf15	chr11:83473107-83506740	OtA5088-OtA5092	OtA5093-OtA5097
Trap1a	chrX:139240225-139357738	OtA5088-OtA5092	OtA5093-OtA5097
Zfp334	chr2:165377435-165388259	OtA5088-OtA5092	OtA5093-OtA5097
Rerg	chr6:137054824-137170496	OtA5088-OtA5092	OtA5093-OtA5097
Shroom3	chr5:92683434-92965759	OtA5088-OtA5092	OtA5093-OtA5097
Terf2	chr8:107069401-107096545	OtA5088-OtA5092	OtA5093-OtA5097
Srgap1	chr10:121780990-122047315	OtA5088-OtA5092	OtA5093-OtA5097
Dock9	chr14:121542038-121797734	OtA5088-OtA5092	OtA5093-OtA5097
Ltv1	chr10:13178637-13193137	OtA5088-OtA5092	OtA5093-OtA5097
Pemt	chr11:59970616-60036839	OtA5088-OtA5092	OtA5093-OtA5097
Slc25a33	chr4:149744035-149774267	OtA5088-OtA5092	OtA5093-OtA5097
Sardh	chr2:27188392-27247303	OtA5088-OtA5092	OtA5093-OtA5097
Cubn	chr2:13276337-13491876	OtA5088-OtA5092	OtA5093-OtA5097
Tnfaip8	chr18:49979426-50093229	OtA5088-OtA5092	OtA5093-OtA5097
Auts2	chr5:131437681-132542343	OtA5088-OtA5092	OtA5093-OtA5097
Cyp51	chr5:4080673-4104697	OtA5088-OtA5092	OtA5093-OtA5097
Fam199x	chrX:137049593-137082501	OtA5088-OtA5092	OtA5093-OtA5097
H2-Ab1	chr17:34263226-34269418	OtA5088-OtA5092	OtA5093-OtA5097
Hmgcl	chr4:135946452-135962617	OtA5088-OtA5092	OtA5093-OtA5097
Ppip5k2	chr1:97706045-97770092	OtA5088-OtA5092	OtA5093-OtA5097
Psmc4	chr7:28041701-28050092	OtA5088-OtA5092	OtA5093-OtA5097
Stag1	chr9:100643622-100958544	OtA5088-OtA5092	OtA5093-OtA5097
Star	chr8:25808512-25815982	OtA5088-OtA5092	OtA5093-OtA5097
Taf7l	chrX:134460115-134476490	OtA5088-OtA5092	OtA5093-OtA5097

Zbtb34	chr2:33406107-33431324	OtA5088-OtA5092	OtA5093-OtA5097
Dock8	chr19:24999528-25202432	OtA5088-OtA5092	OtA5093-OtA5097
Hist2h3b	chr3:96268693-96269104	OtA5088-OtA5092	OtA5093-OtA5097
N4bp1	chr8:86841138-86885258	OtA5088-OtA5092	OtA5093-OtA5097
Prkacb	chr3:146729578-146812946	OtA5088-OtA5092	OtA5093-OtA5097
Rasl11a	chr5:146845070-146847726	OtA5088-OtA5092	OtA5093-OtA5097
Ubap2l	chr3:89998759-90052515	OtA5088-OtA5092	OtA5093-OtA5097
Zfp366	chr13:99184822-99247032	OtA5088-OtA5092	OtA5093-OtA5097
Oxr1	chr15:41447481-41861047	OtA5088-OtA5092	OtA5093-OtA5097
Cast	chr13:74694285-74807921	OtA5088-OtA5092	OtA5093-OtA5097
Cpe	chr8:64592550-64693040	OtA5088-OtA5092	OtA5093-OtA5097
Man1a	chr10:53906032-54075796	OtA5088-OtA5092	OtA5093-OtA5097
Zfp217	chr2:170108642-170142675	OtA5088-OtA5092	OtA5093-OtA5097
Xlr3a	chrX:73086292-73097095	OtA5088-OtA5092	OtA5093-OtA5097
Srrm2	chr17:23803186-23829109	OtA5088-OtA5092	OtA5093-OtA5097
Purb	chr11:6467598-6476076	OtA5088-OtA5092	OtA5093-OtA5097
Pde9a	chr17:31386233-31476309	OtA5088-OtA5092	OtA5093-OtA5097
Dmtf1	chr5:9100736-9161776	OtA5088-OtA5092	OtA5093-OtA5097
Jak2	chr19:29251827-29313080	OtA5088-OtA5092	OtA5093-OtA5097
Lmf1	chr17:25579173-25662826	OtA5088-OtA5092	OtA5093-OtA5097
Rprd2	chr3:95759872-95818953	OtA5088-OtA5092	OtA5093-OtA5097
Atp2a3	chr11:72961168-72993043	OtA5088-OtA5092	OtA5093-OtA5097
D5Ertd579e	chr5:36600488-36695969	OtA5088-OtA5092	OtA5093-OtA5097
Fam126b	chr1:58522805-58586333	OtA5088-OtA5092	OtA5093-OtA5097
Gc	chr5:89417510-89457898	OtA5088-OtA5092	OtA5093-OtA5097
Lpcat3	chr6:124663103-124712178	OtA5088-OtA5092	OtA5093-OtA5097
Pcmtd1	chr1:7088919-7173628	OtA5088-OtA5092	OtA5093-OtA5097
Rtkn	chr6:83135807-83152579	OtA5088-OtA5092	OtA5093-OtA5097
Strn	chr17:78653963-78736560	OtA5088-OtA5092	OtA5093-OtA5097
Rhox12	chrX:38104061-38110907	OtA5088-OtA5092	OtA5093-OtA5097
Hs3st1	chr5:39613934-39644631	OtA5088-OtA5092	OtA5093-OtA5097
Oplah	chr15:76294433-76307245	OtA5088-OtA5092	OtA5093-OtA5097
Cbll1	chr12:31484828-31499616	OtA5088-OtA5092	OtA5093-OtA5097
Mal	chr2:127633225-127656695	OtA5088-OtA5092	OtA5093-OtA5097
Sec16b	chr1:157506795-157568424	OtA5088-OtA5092	OtA5093-OtA5097
Serpina10	chr12:103616674-103631405	OtA5088-OtA5092	OtA5093-OtA5097
Capg	chr6:72544438-72562980	OtA5088-OtA5092	OtA5093-OtA5097
Ubl4	chrX:74365717-74368548	OtA5088-OtA5092	OtA5093-OtA5097
C5ar1	chr7:16246742-16259540	OtA5088-OtA5092	OtA5093-OtA5097
Nmd3	chr3:69722054-69749046	OtA5088-OtA5092	OtA5093-OtA5097
Tmprss6	chr15:78439666-78468634	OtA5088-OtA5092	OtA5093-OtA5097
Mbd5	chr2:48949507-49317069	OtA5088-OtA5092	OtA5093-OtA5097
Plch2	chr4:154983114-155010984	OtA5088-OtA5092	OtA5093-OtA5097
Rapgef5	chr12:117516478-117756978	OtA5088-OtA5092	OtA5093-OtA5097
Zfc3h1	chr10:115384958-115432771	OtA5088-OtA5092	OtA5093-OtA5097
Katnal1	chr5:148871583-148928647	OtA5088-OtA5092	OtA5093-OtA5097
Hmgcr	chr13:96648961-96670936	OtA5088-OtA5092	OtA5093-OtA5097

Fbln2	chr6:91212763-91272540	OtA5088-OtA5092	OtA5093-OtA5097
Arid5b	chr10:68095592-68278726	OtA5088-OtA5092	OtA5093-OtA5097
Tap1	chr17:34187555-34197225	OtA5088-OtA5092	OtA5093-OtA5097
Lama5	chr2:180176372-180225859	OtA5088-OtA5092	OtA5093-OtA5097
Pomgnt1	chr4:116150517-116159844	OtA5088-OtA5092	OtA5093-OtA5097
Vkorc1	chr7:127893062-127895617	OtA5088-OtA5092	OtA5093-OtA5097
Ilvbl	chr10:78574499-78584502	OtA5088-OtA5092	OtA5093-OtA5097
Tmem218	chr9:37208332-37223211	OtA5088-OtA5092	OtA5093-OtA5097
Asap2	chr12:21111755-21286237	OtA5088-OtA5092	OtA5093-OtA5097
Epn1	chr7:5080234-5098178	OtA5088-OtA5092	OtA5093-OtA5097
Jarid2	chr13:44730773-44921643	OtA5088-OtA5092	OtA5093-OtA5097
Plekha5	chr6:140424098-140594906	OtA5088-OtA5092	OtA5093-OtA5097
Dnaja1	chr4:40722467-40757885	OtA5088-OtA5092	OtA5093-OtA5097
Map4k5	chr12:69746847-69987002	OtA5088-OtA5092	OtA5093-OtA5097
Mical3	chr6:121007239-121081609	OtA5088-OtA5092	OtA5093-OtA5097
Pde4d	chr13:108654176-109955969	OtA5088-OtA5092	OtA5093-OtA5097
Npnt	chr3:132882695-132950291	OtA5088-OtA5092	OtA5093-OtA5097
Impdh1	chr6:29200439-29212271	OtA5088-OtA5092	OtA5093-OtA5097
Mrpl3	chr9:105053267-105077476	OtA5088-OtA5092	OtA5093-OtA5097
Zfp281	chr1:136624900-136630391	OtA5088-OtA5092	OtA5093-OtA5097
Atr	chr9:95855549-95951644	OtA5088-OtA5092	OtA5093-OtA5097
Cacna1g	chr11:94408390-94474198	OtA5088-OtA5092	OtA5093-OtA5097
Eng	chr2:32646594-32694175	OtA5088-OtA5092	OtA5093-OtA5097
Psmb3	chr11:97703433-97713500	OtA5088-OtA5092	OtA5093-OtA5097
Smyd5	chr6:85431975-85446429	OtA5088-OtA5092	OtA5093-OtA5097
Klhl29	chr12:5077465-5375682	OtA5088-OtA5092	OtA5093-OtA5097
Unc5b	chr10:60762594-60831581	OtA5088-OtA5092	OtA5093-OtA5097
Ceacam12	chr7:18065928-18077986	OtA5088-OtA5092	OtA5093-OtA5097
Plek	chr11:16971205-17008718	OtA5088-OtA5092	OtA5093-OtA5097
Pabpn1	chr14:54894142-54898927	OtA5088-OtA5092	OtA5093-OtA5097
Slc9a3r2	chr17:24639281-24650305	OtA5088-OtA5092	OtA5093-OtA5097
Emilin1	chr5:30913785-30921273	OtA5088-OtA5092	OtA5093-OtA5097
Fuca2	chr10:13501027-13517275	OtA5088-OtA5092	OtA5093-OtA5097
Grhl2	chr15:37233035-37363568	OtA5088-OtA5092	OtA5093-OtA5097
Mfap3	chr11:57518664-57533817	OtA5088-OtA5092	OtA5093-OtA5097
Mfge8	chr7:79133767-79149060	OtA5088-OtA5092	OtA5093-OtA5097
Prkcd	chr14:30595353-30626208	OtA5088-OtA5092	OtA5093-OtA5097
Safb	chr17:56584981-56606294	OtA5088-OtA5092	OtA5093-OtA5097
Sephs2	chr7:127271878-127274059	OtA5088-OtA5092	OtA5093-OtA5097
Ext2	chr2:93695630-93822568	OtA5088-OtA5092	OtA5093-OtA5097
Atp2c2	chr8:119700008-119757718	OtA5088-OtA5092	OtA5093-OtA5097
Ccdc50	chr16:27388976-27452218	OtA5088-OtA5092	OtA5093-OtA5097
Aldh3a2	chr11:61244754-61267127	OtA5088-OtA5092	OtA5093-OtA5097
Adora1	chr1:134193447-134235431	OtA5088-OtA5092	OtA5093-OtA5097
Morc3	chr16:93832120-93876073	OtA5088-OtA5092	OtA5093-OtA5097
Uck1	chr2:32236682-32260105	OtA5088-OtA5092	OtA5093-OtA5097
Ndufb8	chr19:44550253-44555415	OtA5088-OtA5092	OtA5093-OtA5097

Psg19	chr7:18786301-18798510	OtA5088-OtA5092	OtA5093-OtA5097
Alad	chr4:62509169-62520063	OtA5088-OtA5092	OtA5093-OtA5097
Pip5k1a	chr3:95059595-95106858	OtA5088-OtA5092	OtA5093-OtA5097
Prss8	chr7:127912516-127930113	OtA5088-OtA5092	OtA5093-OtA5097
Nsd1	chr13:55209781-55318325	OtA5088-OtA5092	OtA5093-OtA5097
Ckb	chr12:111669354-111672338	OtA5088-OtA5092	OtA5093-OtA5097
Gnai1	chr5:18265134-18360413	OtA5088-OtA5092	OtA5093-OtA5097
Hand1	chr11:57828712-57832147	OtA5088-OtA5092	OtA5093-OtA5097
Mia3	chr1:183326235-183369565	OtA5088-OtA5092	OtA5093-OtA5097
Rgs16	chr1:153740352-153745468	OtA5088-OtA5092	OtA5093-OtA5097
Sc5d	chr9:42254176-42264300	OtA5088-OtA5092	OtA5093-OtA5097
Srgap3	chr6:112717971-112947266	OtA5088-OtA5092	OtA5093-OtA5097
Gatad2b	chr3:90341653-90358120	OtA5088-OtA5092	OtA5093-OtA5097
Supt16h	chr14:52110902-52197239	OtA5088-OtA5092	OtA5093-OtA5097
Arhgef12	chr9:42963841-43105718	OtA5088-OtA5092	OtA5093-OtA5097
Hmgb1	chr5:149047226-149053037	OtA5088-OtA5092	OtA5093-OtA5097
Adamts9	chr6:92772698-92901441	OtA5088-OtA5092	OtA5093-OtA5097
Slc2a13	chr15:91267690-91573261	OtA5088-OtA5092	OtA5093-OtA5097
Sdf2	chr11:78245745-78255496	OtA5088-OtA5092	OtA5093-OtA5097
Cenpa	chr5:30666901-30674826	OtA5088-OtA5092	OtA5093-OtA5097
Atp5g2	chr15:102662867-102671047	OtA5088-OtA5092	OtA5093-OtA5097
Fam117b	chr1:59913005-59985346	OtA5088-OtA5092	OtA5093-OtA5097
Acsm1	chr7:119617827-119662515	OtA5088-OtA5092	OtA5093-OtA5097
Atg4b	chr1:93755032-93789529	OtA5088-OtA5092	OtA5093-OtA5097
Ppp2r2a	chr14:67014055-67072471	OtA5088-OtA5092	OtA5093-OtA5097
Suz12	chr11:79993105-80034123	OtA5088-OtA5092	OtA5093-OtA5097
Ms4a6c	chr19:11469367-11482196	OtA5088-OtA5092	OtA5093-OtA5097
Atf7ip	chr6:136518850-136607379	OtA5088-OtA5092	OtA5093-OtA5097
Irx2	chr13:72628977-72634194	OtA5088-OtA5092	OtA5093-OtA5097
Tmem110	chr14:30825593-30877210	OtA5088-OtA5092	OtA5093-OtA5097
H1f0	chr15:79028211-79030500	OtA5088-OtA5092	OtA5093-OtA5097
Bcl6b	chr11:70224126-70229798	OtA5088-OtA5092	OtA5093-OtA5097
Ccdc55	chr11:77044291-77078435	OtA5088-OtA5092	OtA5093-OtA5097
Fasn	chr11:120805957-120824547	OtA5088-OtA5092	OtA5093-OtA5097
Gata3	chr2:9857077-9878600	OtA5088-OtA5092	OtA5093-OtA5097
Lmtk2	chr5:144100435-144188204	OtA5088-OtA5092	OtA5093-OtA5097
Cd36	chr5:17781689-17888959	OtA5088-OtA5092	OtA5093-OtA5097
Trappc5	chr8:3676476-3680921	OtA5088-OtA5092	OtA5093-OtA5097
Clec14a	chr12:58264719-58269258	OtA5088-OtA5092	OtA5093-OtA5097
Trim32	chr4:65380802-66404483	OtA5088-OtA5092	OtA5093-OtA5097
Bdkrb2	chr12:105563171-105593071	OtA5088-OtA5092	OtA5093-OtA5097
Chpf	chr1:75474568-75479471	OtA5088-OtA5092	OtA5093-OtA5097
Pdia5	chr16:35397311-35490873	OtA5088-OtA5092	OtA5093-OtA5097
Slc22a2	chr17:12584188-12628488	OtA5088-OtA5092	OtA5093-OtA5097
Emilin3	chr2:160906437-160912328	OtA5088-OtA5092	OtA5093-OtA5097
Bclaf1	chr10:20312468-20342501	OtA5088-OtA5092	OtA5093-OtA5097
Itih5	chr2:10153542-10256529	OtA5088-OtA5092	OtA5093-OtA5097

Vars	chr17:35000906-35016329	OtA5088-OtA5092	OtA5093-OtA5097
Kif2a	chr13:106960584-107022114	OtA5088-OtA5092	OtA5093-OtA5097
Lphn2	chr3:148815585-148954635	OtA5088-OtA5092	OtA5093-OtA5097
Nup205	chr6:35177615-35247598	OtA5088-OtA5092	OtA5093-OtA5097
Sacm1l	chr9:123529881-123592598	OtA5088-OtA5092	OtA5093-OtA5097
Slc25a40	chr5:8422849-8454793	OtA5088-OtA5092	OtA5093-OtA5097
Syde1	chr10:78584502-78591964	OtA5088-OtA5092	OtA5093-OtA5097
Tmem189	chr2:167643224-167661544	OtA5088-OtA5092	OtA5093-OtA5097
Zfp330	chr8:82763619-82774126	OtA5088-OtA5092	OtA5093-OtA5097
C330019G07Rik	chr5:32789206-32854230	OtA5088-OtA5092	OtA5093-OtA5097
Cep57	chr9:13807787-13827107	OtA5088-OtA5092	OtA5093-OtA5097
Cyp24a1	chr2:170482964-170519070	OtA5088-OtA5092	OtA5093-OtA5097
Ezh2	chr6:47530273-47595030	OtA5088-OtA5092	OtA5093-OtA5097
Gm9846,Rps27	chr3:90212666-90213648	OtA5088-OtA5092	OtA5093-OtA5097
Prx	chr7:27499323-27520041	OtA5088-OtA5092	OtA5093-OtA5097
Rreb1	chr13:37826037-37952005	OtA5088-OtA5092	OtA5093-OtA5097
Upk1a	chr7:30603091-30612734	OtA5088-OtA5092	OtA5093-OtA5097
Prr15l	chr11:96929323-96935647	OtA5088-OtA5092	OtA5093-OtA5097
Nmt2	chr2:3284287-3326369	OtA5088-OtA5092	OtA5093-OtA5097
Smoc2	chr17:14279505-14404790	OtA5088-OtA5092	OtA5093-OtA5097
Aip	chr19:4114445-4125827	OtA5088-OtA5092	OtA5093-OtA5097
Pign	chr1:105518421-105663676	OtA5088-OtA5092	OtA5093-OtA5097
Bcorl1	chrX:48341357-48406728	OtA5088-OtA5092	OtA5093-OtA5097
Bms1	chr6:118383380-118419417	OtA5088-OtA5092	OtA5093-OtA5097
Dek	chr13:47043498-47106221	OtA5088-OtA5092	OtA5093-OtA5097
Ndufa7	chr17:33824613-33838313	OtA5088-OtA5092	OtA5093-OtA5097
Anxa11	chr14:25842154-25886804	OtA5088-OtA5092	OtA5093-OtA5097
Gpr116	chr17:43389465-43459557	OtA5088-OtA5092	OtA5093-OtA5097
Slc19a1	chr10:77032738-77050432	OtA5088-OtA5092	OtA5093-OtA5097
Zfyve26	chr12:79232346-79296282	OtA5088-OtA5092	OtA5093-OtA5097
Cnot6	chr11:49674705-49712710	OtA5088-OtA5092	OtA5093-OtA5097
Ctsc	chr7:88278092-88310875	OtA5088-OtA5092	OtA5093-OtA5097
Fam195a	chr17:25863697-25868738	OtA5088-OtA5092	OtA5093-OtA5097
Kif5a	chr10:127225694-127263363	OtA5088-OtA5092	OtA5093-OtA5097
Ocel1	chr8:71371297-71373689	OtA5088-OtA5092	OtA5093-OtA5097
Pde3a	chr6:141249268-141499351	OtA5088-OtA5092	OtA5093-OtA5097
Phospho1	chr11:95824499-95845731	OtA5088-OtA5092	OtA5093-OtA5097
Trim24	chr6:37870810-37966289	OtA5088-OtA5092	OtA5093-OtA5097
Zfp667	chr7:6286579-6307883	OtA5088-OtA5092	OtA5093-OtA5097
Arhgap28	chr17:67842707-68004108	OtA5088-OtA5092	OtA5093-OtA5097
Golm1	chr13:59634995-59675784	OtA5088-OtA5092	OtA5093-OtA5097
Med20	chr17:47611595-47624418	OtA5088-OtA5092	OtA5093-OtA5097
Slc16a10	chr10:40033534-40142254	OtA5088-OtA5092	OtA5093-OtA5097
Ylpm1	chr12:84996320-85070515	OtA5088-OtA5092	OtA5093-OtA5097
Susd1	chr4:59314682-59438633	OtA5088-OtA5092	OtA5093-OtA5097
Ranbp2	chr10:58446851-58494154	OtA5088-OtA5092	OtA5093-OtA5097
Acad10	chr5:121621028-121660510	OtA5088-OtA5092	OtA5093-OtA5097

Ascl2	chr7:142966821-142969264	OtA5088-OtA5092	OtA5093-OtA5097
B3gat3	chr19:8920392-8927236	OtA5088-OtA5092	OtA5093-OtA5097
Birc7	chr2:180929022-180934010	OtA5088-OtA5092	OtA5093-OtA5097
Cd248	chr19:5068077-5070639	OtA5088-OtA5092	OtA5093-OtA5097
Rps6ka2	chr17:7170114-7303316	OtA5088-OtA5092	OtA5093-OtA5097
Sin3b	chr8:72723287-72758203	OtA5088-OtA5092	OtA5093-OtA5097
Rnf169	chr7:99920253-99980458	OtA5088-OtA5092	OtA5093-OtA5097
Rpa2	chr4:132768359-132778746	OtA5088-OtA5092	OtA5093-OtA5097
Igfbp5	chr1:72858064-72874865	OtA5088-OtA5092	OtA5093-OtA5097
Mmp15	chr8:95352336-95374293	OtA5088-OtA5092	OtA5093-OtA5097
Nfatc3	chr8:106059602-106130537	OtA5088-OtA5092	OtA5093-OtA5097
Hmbox1	chr14:64822217-64949847	OtA5088-OtA5092	OtA5093-OtA5097
Sfpi1	chr2:91096796-91115756	OtA5088-OtA5092	OtA5093-OtA5097
Mynn	chr3:30602086-30618494	OtA5088-OtA5092	OtA5093-OtA5097
Gng11	chr6:4003986-4008446	OtA5088-OtA5092	OtA5093-OtA5097
Aldh18a1	chr19:40550256-40588463	OtA5088-OtA5092	OtA5093-OtA5097
Bex1	chrX:136213971-136215513	OtA5088-OtA5092	OtA5093-OtA5097
Ccdc9	chr7:16274041-16286795	OtA5088-OtA5092	OtA5093-OtA5097
Efcab4a	chr7:141461093-141466602	OtA5088-OtA5092	OtA5093-OtA5097
Gatsl3	chr11:4218250-4222409	OtA5088-OtA5092	OtA5093-OtA5097
Oat	chr7:132557474-132576398	OtA5088-OtA5092	OtA5093-OtA5097
Adamtsl4	chr3:95676203-95687858	OtA5088-OtA5092	OtA5093-OtA5097
Phf19	chr2:34893754-34913976	OtA5088-OtA5092	OtA5093-OtA5097
Ncoa1	chr12:4247361-4477182	OtA5088-OtA5092	OtA5093-OtA5097
Prkcdbp	chr7:105480615-105482197	OtA5088-OtA5092	OtA5093-OtA5097
Sdf2l1	chr16:17130137-17132383	OtA5088-OtA5092	OtA5093-OtA5097
Lemd2	chr17:27189599-27204438	OtA5088-OtA5092	OtA5093-OtA5097
Creld2	chr15:88819645-88826681	OtA5088-OtA5092	OtA5093-OtA5097
Plekhh2	chr17:84511894-84622142	OtA5088-OtA5092	OtA5093-OtA5097
Rai14	chr15:10568977-10714631	OtA5088-OtA5092	OtA5093-OtA5097
Sec13	chr6:113728051-113740681	OtA5088-OtA5092	OtA5093-OtA5097
Brp44l	chr17:8283812-8297661	OtA5088-OtA5092	OtA5093-OtA5097
Rcan1	chr16:92391952-92466146	OtA5088-OtA5092	OtA5093-OtA5097
Ptafr	chr4:132564066-132580866	OtA5088-OtA5092	OtA5093-OtA5097
Ncoa7	chr10:30645581-30803107	OtA5088-OtA5092	OtA5093-OtA5097
BC056474	chr8:85080962-85082339	OtA5088-OtA5092	OtA5093-OtA5097
Nle1	chr11:82900767-82908395	OtA5088-OtA5092	OtA5093-OtA5097
Derl1	chr15:57869501-57892418	OtA5088-OtA5092	OtA5093-OtA5097
Cdk12	chr11:98203304-98253540	OtA5088-OtA5092	OtA5093-OtA5097
Aph1a	chr3:95893995-95904639	OtA5088-OtA5092	OtA5093-OtA5097
Dcaf11	chr14:55560028-55570065	OtA5088-OtA5092	OtA5093-OtA5097
Grk5	chr19:60889748-61092555	OtA5088-OtA5092	OtA5093-OtA5097
Tyrobp	chr7:30413787-30417579	OtA5088-OtA5092	OtA5093-OtA5097
Brd4	chr17:32196273-32284123	OtA5088-OtA5092	OtA5093-OtA5097
Mrps33	chr6:39801806-39810936	OtA5088-OtA5092	OtA5093-OtA5097
Arhgap42	chr9:8994952-9260885	OtA5088-OtA5092	OtA5093-OtA5097
Cdc42ep2	chr19:5917555-5924816	OtA5088-OtA5092	OtA5093-OtA5097

Ddah2	chr17:35059034-35062099	OtA5088-OtA5092	OtA5093-OtA5097
Usp32	chr11:84984487-85139955	OtA5088-OtA5092	OtA5093-OtA5097
Creld1	chr6:113483568-113493338	OtA5088-OtA5092	OtA5093-OtA5097
Mcm3	chr1:20803013-20820213	OtA5088-OtA5092	OtA5093-OtA5097
Ndufa2	chr18:36735069-36744548	OtA5088-OtA5092	OtA5093-OtA5097
Tmsb4x	chrX:167207093-167209218	OtA5088-OtA5092	OtA5093-OtA5097
Tom1l1	chr11:90638183-90687601	OtA5088-OtA5092	OtA5093-OtA5097
BC027231	chr16:44724300-44737284	OtA5088-OtA5092	OtA5093-OtA5097
Dcun1d4	chr5:73481054-73560794	OtA5088-OtA5092	OtA5093-OtA5097
Smarcc1	chr9:110132023-110240178	OtA5088-OtA5092	OtA5093-OtA5097
Erh	chr12:80634022-80643861	OtA5088-OtA5092	OtA5093-OtA5097
Slc26a2	chr18:61196853-61211596	OtA5088-OtA5092	OtA5093-OtA5097
Pafah2	chr4:134396359-134427412	OtA5088-OtA5092	OtA5093-OtA5097
Vldlr	chr19:27217019-27254231	OtA5088-OtA5092	OtA5093-OtA5097
Oit3	chr10:59422959-59441779	OtA5088-OtA5092	OtA5093-OtA5097
Rhob	chr12:8497758-8499985	OtA5088-OtA5092	OtA5093-OtA5097
Lox	chr18:52517237-52529708	OtA5088-OtA5092	OtA5093-OtA5097
Mfap5	chr6:122513675-122529287	OtA5088-OtA5092	OtA5093-OtA5097
Tom1	chr8:75033685-75070121	OtA5088-OtA5092	OtA5093-OtA5097
Vps52	chr17:33955881-33966488	OtA5088-OtA5092	OtA5093-OtA5097
Entpd1	chr19:40659793-40741602	OtA5088-OtA5092	OtA5093-OtA5097
Cd68	chr11:69664370-69666062	OtA5088-OtA5092	OtA5093-OtA5097
Gnptab	chr10:88379411-88447329	OtA5088-OtA5092	OtA5093-OtA5097
Rmnd5b	chr11:51619772-51635896	OtA5088-OtA5092	OtA5093-OtA5097
Gpn3	chr5:122372507-122382769	OtA5088-OtA5092	OtA5093-OtA5097
Aldh2	chr5:121567686-121593824	OtA5088-OtA5092	OtA5093-OtA5097
Csnk1g1	chr9:65909009-66045014	OtA5088-OtA5092	OtA5093-OtA5097
D14Abb1e	chr14:27428846-27508460	OtA5088-OtA5092	OtA5093-OtA5097
Pdcd5	chr7:35641984-35647482	OtA5088-OtA5092	OtA5093-OtA5097
Smad5	chr13:56703009-56742378	OtA5088-OtA5092	OtA5093-OtA5097
Ccdc82	chr9:13246978-13292353	OtA5088-OtA5092	OtA5093-OtA5097
Smg1	chr7:118131311-118243637	OtA5088-OtA5092	OtA5093-OtA5097
Cyp4f18	chr8:71988481-72009626	OtA5088-OtA5092	OtA5093-OtA5097
Cdca3	chr6:124830175-124833701	OtA5088-OtA5092	OtA5093-OtA5097
Diexf	chr1:193104402-193130251	OtA5088-OtA5092	OtA5093-OtA5097
Arhgef5	chr6:43265643-43289320	OtA5088-OtA5092	OtA5093-OtA5097
Hoxd10	chr2:74691947-74695102	OtA5088-OtA5092	OtA5093-OtA5097
Tmem184a	chr5:139804951-139814283	OtA5088-OtA5092	OtA5093-OtA5097
Agfg1	chr1:82839482-82896275	OtA5088-OtA5092	OtA5093-OtA5097
Sdcbp	chr4:6365679-6396122	OtA5088-OtA5092	OtA5093-OtA5097
Meis2	chr2:115861263-116065058	OtA5088-OtA5092	OtA5093-OtA5097
Emid1	chr11:5106265-5152222	OtA5088-OtA5092	OtA5093-OtA5097
Kng1	chr16:23058299-23082078	OtA5088-OtA5092	OtA5093-OtA5097
Tbx2	chr11:85832614-85841948	OtA5088-OtA5092	OtA5093-OtA5097
Timm22	chr11:76407166-76416312	OtA5088-OtA5092	OtA5093-OtA5097
Zfp408	chr2:91643687-91649791	OtA5088-OtA5092	OtA5093-OtA5097
Btbd7	chr12:102784647-102878406	OtA5088-OtA5092	OtA5093-OtA5097

Cep250	chr2:155956557-155998900	OtA5088-OtA5092	OtA5093-OtA5097
Foxj2	chr6:122820183-122845366	OtA5088-OtA5092	OtA5093-OtA5097
Mrto4	chr4:139347444-139352298	OtA5088-OtA5092	OtA5093-OtA5097
Rbpj	chr5:53555778-53657445	OtA5088-OtA5092	OtA5093-OtA5097
Tmem57	chr4:134802759-134853345	OtA5088-OtA5092	OtA5093-OtA5097
Hnrnpd	chr5:99955935-99978938	OtA5088-OtA5092	OtA5093-OtA5097
Rif1	chr2:52072836-52122381	OtA5088-OtA5092	OtA5093-OtA5097
Ttc4	chr4:106661807-106678944	OtA5088-OtA5092	OtA5093-OtA5097
Ehd4	chr2:120089486-120154575	OtA5088-OtA5092	OtA5093-OtA5097
Capn2	chr1:182467258-182517483	OtA5088-OtA5092	OtA5093-OtA5097
Baz2a	chr10:128092782-128129303	OtA5088-OtA5092	OtA5093-OtA5097
Cntfr	chr4:41657497-41697091	OtA5088-OtA5092	OtA5093-OtA5097
Ngfrap1	chrX:136270252-136271978	OtA5088-OtA5092	OtA5093-OtA5097
Pafah1b1	chr11:74673948-74724384	OtA5088-OtA5092	OtA5093-OtA5097
Pnrc2	chr4:135870925-135873846	OtA5088-OtA5092	OtA5093-OtA5097
Stox2	chr8:47180047-47352348	OtA5088-OtA5092	OtA5093-OtA5097
Tpm4	chr8:72135291-72153129	OtA5088-OtA5092	OtA5093-OtA5097
Cryab	chr9:50752757-50756633	OtA5088-OtA5092	OtA5093-OtA5097
Rhpn2	chr7:35334236-35396767	OtA5088-OtA5092	OtA5093-OtA5097
Rrm2	chr12:24708253-24714146	OtA5088-OtA5092	OtA5093-OtA5097
Ctif	chr18:75431220-75697696	OtA5088-OtA5092	OtA5093-OtA5097
Rod1	chr4:59471867-59549364	OtA5088-OtA5092	OtA5093-OtA5097
C2cd2	chr16:97855208-97922633	OtA5088-OtA5092	OtA5093-OtA5097
Aga	chr8:53511701-53523422	OtA5088-OtA5092	OtA5093-OtA5097
Bloc1s3	chr7:19505803-19508331	OtA5088-OtA5092	OtA5093-OtA5097
Dlc1	chr8:36567738-36952442	OtA5088-OtA5092	OtA5093-OtA5097
Fam117a	chr11:95337017-95381872	OtA5088-OtA5092	OtA5093-OtA5097
Itpr3	chr17:27057303-27122223	OtA5088-OtA5092	OtA5093-OtA5097
Morc2a	chr11:3649493-3690372	OtA5088-OtA5092	OtA5093-OtA5097
Slc34a2	chr5:53049352-53071663	OtA5088-OtA5092	OtA5093-OtA5097
Tab3	chrX:85574021-85634469	OtA5088-OtA5092	OtA5093-OtA5097
Hand2	chr8:57320982-57324517	OtA5088-OtA5092	OtA5093-OtA5097
Hif3a	chr7:17030992-17062427	OtA5088-OtA5092	OtA5093-OtA5097
Itga3	chr11:95044481-95076714	OtA5088-OtA5092	OtA5093-OtA5097
Lhfpl2	chr13:94057795-94195409	OtA5088-OtA5092	OtA5093-OtA5097
Lrrc17	chr5:21483846-21645605	OtA5088-OtA5092	OtA5093-OtA5097
Sumf1	chr6:108107020-108185583	OtA5088-OtA5092	OtA5093-OtA5097
Suv420h1	chr19:3767420-3818303	OtA5088-OtA5092	OtA5093-OtA5097
Uqcrc1	chr9:108936647-108949641	OtA5088-OtA5092	OtA5093-OtA5097
Smad7	chr18:75367364-75395934	OtA5088-OtA5092	OtA5093-OtA5097
Agfg2	chr5:137650853-137684693	OtA5088-OtA5092	OtA5093-OtA5097
Ceacam14	chr7:17812681-17815627	OtA5088-OtA5092	OtA5093-OtA5097
Sos2	chr12:69583760-69681852	OtA5088-OtA5092	OtA5093-OtA5097
Rcsd1	chr1:165648944-165708094	OtA5088-OtA5092	OtA5093-OtA5097
Ccnyl1	chr1:64691344-64725642	OtA5088-OtA5092	OtA5093-OtA5097
Epha2	chr4:141301220-141329384	OtA5088-OtA5092	OtA5093-OtA5097
Mid1ip1	chrX:10717364-10719702	OtA5088-OtA5092	OtA5093-OtA5097

Nosip	chr7:45062428-45078503	OtA5088-OtA5092	OtA5093-OtA5097
Snrk	chr9:122117265-122169702	OtA5088-OtA5092	OtA5093-OtA5097
Top1	chr2:160645896-160722763	OtA5088-OtA5092	OtA5093-OtA5097
S1pr3	chr13:51408617-51422797	OtA5088-OtA5092	OtA5093-OtA5097
Hsd11b1	chr1:193221640-193264045	OtA5088-OtA5092	OtA5093-OtA5097
Cyc1	chr15:76343522-76345934	OtA5088-OtA5092	OtA5093-OtA5097
Flt3	chr5:147330741-147400489	OtA5088-OtA5092	OtA5093-OtA5097
Tgs1	chr4:3574878-3616623	OtA5088-OtA5092	OtA5093-OtA5097
Golim4	chr3:75876383-75956831	OtA5088-OtA5092	OtA5093-OtA5097
Lta4h	chr10:93453395-93484896	OtA5088-OtA5092	OtA5093-OtA5097
Afap1l1	chr18:61730261-61786662	OtA5088-OtA5092	OtA5093-OtA5097
Tigd2	chr6:59208869-59212033	OtA5088-OtA5092	OtA5093-OtA5097
Ggcx	chr6:72414332-72430707	OtA5088-OtA5092	OtA5093-OtA5097
Havcr2	chr11:46454930-46481254	OtA5088-OtA5092	OtA5093-OtA5097
Limd1	chr9:123478700-123521552	OtA5088-OtA5092	OtA5093-OtA5097
Rptor	chr11:119602994-119899591	OtA5088-OtA5092	OtA5093-OtA5097
Tgfbr2	chr9:116087694-116175363	OtA5088-OtA5092	OtA5093-OtA5097
Tmem53	chr4:117251988-117268580	OtA5088-OtA5092	OtA5093-OtA5097
Man2b2	chr5:36806812-36830649	OtA5088-OtA5092	OtA5093-OtA5097
Scly	chr1:91298337-91321080	OtA5088-OtA5092	OtA5093-OtA5097
Fmnl2	chr2:52857867-53134202	OtA5088-OtA5092	OtA5093-OtA5097
Cltb	chr13:54592938-54611272	OtA5088-OtA5092	OtA5093-OtA5097
Upf2	chr2:5951468-6056703	OtA5088-OtA5092	OtA5093-OtA5097
Fam135b	chr15:71445677-71727838	OtA5088-OtA5092	OtA5093-OtA5097
C030046E11Rik	chr19:29522281-29605921	OtA5088-OtA5092	OtA5093-OtA5097
Gata2	chr6:88198663-88207032	OtA5088-OtA5092	OtA5093-OtA5097
Lyve1	chr7:110850606-110862953	OtA5088-OtA5092	OtA5093-OtA5097
Myl12b	chr17:70973962-70990516	OtA5088-OtA5092	OtA5093-OtA5097
Cep170	chr1:176733652-176807124	OtA5088-OtA5092	OtA5093-OtA5097
Icam1	chr9:21015959-21028796	OtA5088-OtA5092	OtA5093-OtA5097
Pfkfb3	chr2:11471430-11553929	OtA5088-OtA5092	OtA5093-OtA5097
Tmem185b	chr1:119526153-119528983	OtA5088-OtA5092	OtA5093-OtA5097
Gpcpd1	chr2:132529082-132578248	OtA5088-OtA5092	OtA5093-OtA5097
Slc25a29	chr12:108825877-108835876	OtA5088-OtA5092	OtA5093-OtA5097
Fam175b	chr7:132859224-132885114	OtA5088-OtA5092	OtA5093-OtA5097
Sf3b1	chr1:54985169-55027478	OtA5088-OtA5092	OtA5093-OtA5097
Mylk3	chr8:85324928-85365324	OtA5088-OtA5092	OtA5093-OtA5097
Tbc1d20	chr2:152293871-152312590	OtA5088-OtA5092	OtA5093-OtA5097
Hgsnat	chr8:25944458-25976744	OtA5088-OtA5092	OtA5093-OtA5097
Psenen	chr7:30561865-30563184	OtA5088-OtA5092	OtA5093-OtA5097
Xdh	chr17:73883907-73950182	OtA5088-OtA5092	OtA5093-OtA5097
Mtap7d1	chr4:126232167-126256319	OtA5088-OtA5092	OtA5093-OtA5097
Tuba1b	chr15:98931430-98934390	OtA5088-OtA5092	OtA5093-OtA5097
Bzw1	chr1:58393135-58406548	OtA5088-OtA5092	OtA5093-OtA5097
Mmp14	chr14:54431603-54441258	OtA5088-OtA5092	OtA5093-OtA5097
Dysf	chr6:84008589-84211042	OtA5088-OtA5092	OtA5093-OtA5097
Pitpnb	chr5:111330762-111388359	OtA5088-OtA5092	OtA5093-OtA5097

Ube2g1	chr11:72607260-72686481	OtA5088-OtA5092	OtA5093-OtA5097
Vmac	chr17:56713931-56717699	OtA5088-OtA5092	OtA5093-OtA5097
Sfswap	chr5:129501230-129571384	OtA5088-OtA5092	OtA5093-OtA5097
Krt25	chr11:99315843-99322941	OtA5088-OtA5092	OtA5093-OtA5097
Mlst8	chr17:24473549-24479078	OtA5088-OtA5092	OtA5093-OtA5097
Tbrg3	chr15:82808625-82912134	OtA5088-OtA5092	OtA5093-OtA5097
Lipa	chr19:34492315-34527474	OtA5088-OtA5092	OtA5093-OtA5097
Dnajc1	chr2:18055236-18392830	OtA5088-OtA5092	OtA5093-OtA5097
Myom3	chr4:135759714-135815564	OtA5088-OtA5092	OtA5093-OtA5097
Pdap1	chr5:145128769-145140089	OtA5088-OtA5092	OtA5093-OtA5097
Vcan	chr13:89655309-89742512	OtA5088-OtA5092	OtA5093-OtA5097
Lbh	chr17:72918304-72941946	OtA5088-OtA5092	OtA5093-OtA5097
Tubd1	chr11:86544990-86567360	OtA5088-OtA5092	OtA5093-OtA5097
Degs1	chr1:182275769-182282759	OtA5088-OtA5092	OtA5093-OtA5097
Tle4	chr19:14448071-14597983	OtA5088-OtA5092	OtA5093-OtA5097
Ypel2	chr11:86936424-86993762	OtA5088-OtA5092	OtA5093-OtA5097
Ass1	chr2:31470269-31520670	OtA5088-OtA5092	OtA5093-OtA5097
Sh3glb1	chr3:144688843-144720323	OtA5088-OtA5092	OtA5093-OtA5097
Cask	chrX:13517081-13846541	OtA5088-OtA5092	OtA5093-OtA5097
Gsc	chr12:104471208-104473236	OtA5088-OtA5092	OtA5093-OtA5097
Irf7	chr7:141263182-141266424	OtA5088-OtA5092	OtA5093-OtA5097
Thumpd1	chr7:119715093-119720765	OtA5088-OtA5092	OtA5093-OtA5097
Tbl1xr1	chr3:22076651-22216594	OtA5088-OtA5092	OtA5093-OtA5097
Nqo1	chr8:107388224-107403205	OtA5088-OtA5092	OtA5093-OtA5097
BC004004	chr17:29268787-29302887	OtA5088-OtA5092	OtA5093-OtA5097
Cyp17a1	chr19:46667164-46673000	OtA5088-OtA5092	OtA5093-OtA5097
C530028O21Rik	chr6:124996719-125003097	OtA5088-OtA5092	OtA5093-OtA5097
Lmnb2	chr10:80901362-80918245	OtA5088-OtA5092	OtA5093-OtA5097
Sec22b	chr3:97901226-97922318	OtA5088-OtA5092	OtA5093-OtA5097
Tyro3	chr2:119799513-119818103	OtA5088-OtA5092	OtA5093-OtA5097
Bhlha15	chr5:144190285-144194441	OtA5088-OtA5092	OtA5093-OtA5097
Disp2	chr2:118779718-118795175	OtA5088-OtA5092	OtA5093-OtA5097
Mrpl47	chr3:32727496-32736755	OtA5088-OtA5092	OtA5093-OtA5097
Rdh11	chr12:79175550-79191819	OtA5088-OtA5092	OtA5093-OtA5097
Slc29a3	chr10:60712071-60752782	OtA5088-OtA5092	OtA5093-OtA5097
Triml1	chr8:43129806-43141486	OtA5088-OtA5092	OtA5093-OtA5097
Cops3	chr11:59810079-59839767	OtA5088-OtA5092	OtA5093-OtA5097
Lpp	chr16:24392555-24992578	OtA5088-OtA5092	OtA5093-OtA5097
Cops4	chr5:100518308-100547802	OtA5088-OtA5092	OtA5093-OtA5097
D730048J04Rik	chr17:43534173-43543639	OtA5088-OtA5092	OtA5093-OtA5097
Hist1h3d	chr13:23575801-23576212	OtA5088-OtA5092	OtA5093-OtA5097
Psg29	chr7:17203476-17215756	OtA5088-OtA5092	OtA5093-OtA5097
Pex5	chr6:124396815-124415067	OtA5088-OtA5092	OtA5093-OtA5097
Ltbp2	chr12:84783211-84879755	OtA5088-OtA5092	OtA5093-OtA5097
Ccdc124	chr8:70868226-70873490	OtA5088-OtA5092	OtA5093-OtA5097
Hist1h3g	chr13:23535417-23535909	OtA5088-OtA5092	OtA5093-OtA5097
Ttyh3	chr5:140620577-140649031	OtA5088-OtA5092	OtA5093-OtA5097

Adck4	chr7:27233012-27257949	OtA5088-OtA5092	OtA5093-OtA5097
Commd9	chr2:101886261-101901639	OtA5088-OtA5092	OtA5093-OtA5097
Def6	chr17:28207777-28228608	OtA5088-OtA5092	OtA5093-OtA5097
Ext1	chr15:53068260-53346183	OtA5088-OtA5092	OtA5093-OtA5097
Fut11	chr14:20694967-20700197	OtA5088-OtA5092	OtA5093-OtA5097
Narfl	chr17:25773775-25785333	OtA5088-OtA5092	OtA5093-OtA5097
Npas2	chr1:39194271-39363240	OtA5088-OtA5092	OtA5093-OtA5097
Phlda1	chr10:111506285-111508649	OtA5088-OtA5092	OtA5093-OtA5097
Gba	chr3:89202927-89208681	OtA5088-OtA5092	OtA5093-OtA5097
Zc3h12a	chr4:125118413-125127881	OtA5088-OtA5092	OtA5093-OtA5097
Dse	chr10:34151392-34207551	OtA5088-OtA5092	OtA5093-OtA5097
Bambi	chr18:3507956-3516404	OtA5088-OtA5092	OtA5093-OtA5097
Sfrp4	chr13:19623174-19632823	OtA5088-OtA5092	OtA5093-OtA5097
Tsta3	chr15:75924682-75929730	OtA5088-OtA5092	OtA5093-OtA5097
Pmpcb	chr5:21737159-21757152	OtA5088-OtA5092	OtA5093-OtA5097
Slc25a44	chr3:88410495-88425139	OtA5088-OtA5092	OtA5093-OtA5097
Ift74	chr4:94614519-94693233	OtA5088-OtA5092	OtA5093-OtA5097
Aftph	chr11:20685084-20741548	OtA5088-OtA5092	OtA5093-OtA5097
Hsd3b7	chr7:127800608-127803802	OtA5088-OtA5092	OtA5093-OtA5097
Clic1	chr17:35050242-35058719	OtA5088-OtA5092	OtA5093-OtA5097
Cp	chr3:19957053-20035310	OtA5088-OtA5092	OtA5093-OtA5097
Fubp1	chr3:152210457-152236830	OtA5088-OtA5092	OtA5093-OtA5097
Glce	chr9:62057248-62070606	OtA5088-OtA5092	OtA5093-OtA5097
Glt25d1	chr8:71611023-71624911	OtA5088-OtA5092	OtA5093-OtA5097
Hebp2	chr10:18540122-18546076	OtA5088-OtA5092	OtA5093-OtA5097
Hyou1	chr9:44379489-44392369	OtA5088-OtA5092	OtA5093-OtA5097
Ihh	chr1:74945317-74951651	OtA5088-OtA5092	OtA5093-OtA5097
Kcnq4	chr4:120697472-120747176	OtA5088-OtA5092	OtA5093-OtA5097
Sh2d4a	chr8:68276527-68347704	OtA5088-OtA5092	OtA5093-OtA5097
Trib3	chr2:152337424-152344060	OtA5088-OtA5092	OtA5093-OtA5097
Usp5	chr6:124815018-124829447	OtA5088-OtA5092	OtA5093-OtA5097
Def8	chr8:123442955-123463899	OtA5088-OtA5092	OtA5093-OtA5097
Ankrd28	chr14:31700014-31830415	OtA5088-OtA5092	OtA5093-OtA5097
Ppbp	chr5:90768517-90770060	OtA5088-OtA5092	OtA5093-OtA5097
Flii	chr11:60699689-60727221	OtA5088-OtA5092	OtA5093-OtA5097
Plin3	chr17:56278961-56290511	OtA5088-OtA5092	OtA5093-OtA5097
Batf3	chr1:191098413-191108943	OtA5088-OtA5092	OtA5093-OtA5097
Golga4	chr9:118506317-118582519	OtA5088-OtA5092	OtA5093-OtA5097
Ppapdc3	chr2:32095650-32110820	OtA5088-OtA5092	OtA5093-OtA5097
Tmem129	chr5:33653215-33657832	OtA5088-OtA5092	OtA5093-OtA5097
Gpr89	chr3:96871065-96905298	OtA5088-OtA5092	OtA5093-OtA5097
Dchs1	chr7:105752988-105787550	OtA5088-OtA5092	OtA5093-OtA5097
Tgfa	chr6:86195250-86275449	OtA5088-OtA5092	OtA5093-OtA5097
Galnt2	chr8:124231393-124345723	OtA5088-OtA5092	OtA5093-OtA5097
Gas7	chr11:67532997-67688992	OtA5088-OtA5092	OtA5093-OtA5097
Tmem5	chr10:122081259-122097102	OtA5088-OtA5092	OtA5093-OtA5097
Sbk1	chr7:126272618-126294999	OtA5088-OtA5092	OtA5093-OtA5097

Fam122b	chrX:53243414-53269805	OtA5088-OtA5092	OtA5093-OtA5097
Gimap1	chr6:48739046-48743795	OtA5088-OtA5092	OtA5093-OtA5097
Cbfa2t3	chr8:122625135-122699109	OtA5088-OtA5092	OtA5093-OtA5097
Il15	chr8:82331623-82402586	OtA5088-OtA5092	OtA5093-OtA5097
Crlf1	chr8:70493155-70504081	OtA5088-OtA5092	OtA5093-OtA5097
Lmo2	chr2:103957994-103981878	OtA5088-OtA5092	OtA5093-OtA5097
Orc2	chr1:58462770-58504919	OtA5088-OtA5092	OtA5093-OtA5097
Psmb4	chr3:94884323-94886958	OtA5088-OtA5092	OtA5093-OtA5097
Lamb1	chr12:31265293-31329639	OtA5088-OtA5092	OtA5093-OtA5097
Cth	chr3:157894247-157925063	OtA5088-OtA5092	OtA5093-OtA5097
Dolk	chr2:30284228-30286354	OtA5088-OtA5092	OtA5093-OtA5097
Esrp1	chr4:11332037-11386783	OtA5088-OtA5092	OtA5093-OtA5097
Hist1h2bk	chr13:22035886-22036267	OtA5088-OtA5092	OtA5093-OtA5097
Lmbr1	chr5:29229801-29378390	OtA5088-OtA5092	OtA5093-OtA5097
Osbpl5	chr7:143688761-143741963	OtA5088-OtA5092	OtA5093-OtA5097
Slc4a7	chr14:14703024-14799943	OtA5088-OtA5092	OtA5093-OtA5097
Tanc2	chr11:105589985-105929303	OtA5088-OtA5092	OtA5093-OtA5097
Tspan8	chr10:115817283-115849893	OtA5088-OtA5092	OtA5093-OtA5097
Polr3e	chr7:120917743-120947432	OtA5088-OtA5092	OtA5093-OtA5097
Psap	chr10:60277627-60302600	OtA5088-OtA5092	OtA5093-OtA5097
Gga2	chr7:121986721-122021198	OtA5088-OtA5092	OtA5093-OtA5097
Tysnd1	chr10:61695513-61702773	OtA5088-OtA5092	OtA5093-OtA5097
Ell	chr8:70539674-70592858	OtA5088-OtA5092	OtA5093-OtA5097
Snx11	chr11:96767548-96777555	OtA5088-OtA5092	OtA5093-OtA5097
Mgst2	chr3:51661192-51682675	OtA5088-OtA5092	OtA5093-OtA5097
Idh3b	chr2:130274411-130284451	OtA5088-OtA5092	OtA5093-OtA5097
Bptf	chr11:107033080-107131922	OtA5088-OtA5092	OtA5093-OtA5097
Slpi	chr2:164354069-164356507	OtA5088-OtA5092	OtA5093-OtA5097
Akr7a5	chr4:139310743-139318786	OtA5088-OtA5092	OtA5093-OtA5097
Mblac1	chr5:138194313-138195621	OtA5088-OtA5092	OtA5093-OtA5097
Npepl1	chr2:174110350-174122702	OtA5088-OtA5092	OtA5093-OtA5097
Pkdcc	chr17:83215282-83225069	OtA5088-OtA5092	OtA5093-OtA5097
Stk3	chr15:34875498-35155806	OtA5088-OtA5092	OtA5093-OtA5097
Ly6e	chr15:74955050-74959905	OtA5088-OtA5092	OtA5093-OtA5097
Tcf7l1	chr6:72626379-72788956	OtA5088-OtA5092	OtA5093-OtA5097
Asb1	chr1:91540564-91559590	OtA5088-OtA5092	OtA5093-OtA5097
Mycbp2	chr14:103113410-103346800	OtA5088-OtA5092	OtA5093-OtA5097
Arhgap25	chr6:87459384-87533235	OtA5088-OtA5092	OtA5093-OtA5097
Cd244	chr1:171559192-171585316	OtA5088-OtA5092	OtA5093-OtA5097
Dimt1	chr13:106947128-106960224	OtA5088-OtA5092	OtA5093-OtA5097
Fhod3	chr18:24709444-25168877	OtA5088-OtA5092	OtA5093-OtA5097
Gli3	chr13:15463722-15730025	OtA5088-OtA5092	OtA5093-OtA5097
Ick	chr9:78109191-78172109	OtA5088-OtA5092	OtA5093-OtA5097
Mtmr2	chr9:13749180-13806481	OtA5088-OtA5092	OtA5093-OtA5097
Nr1d1	chr11:98767934-98775242	OtA5088-OtA5092	OtA5093-OtA5097
Serpina1c	chr12:103894925-103904950	OtA5088-OtA5092	OtA5093-OtA5097
Slc25a38	chr9:120110398-120124319	OtA5088-OtA5092	OtA5093-OtA5097

Spock1	chr13:57421194-57908332	OtA5088-OtA5092	OtA5093-OtA5097
Tmcc3	chr10:94514856-94590954	OtA5088-OtA5092	OtA5093-OtA5097
Lcor	chr19:41549638-41559781	OtA5088-OtA5092	OtA5093-OtA5097
Trim35	chr14:66295328-66311424	OtA5088-OtA5092	OtA5093-OtA5097
Nme2	chr11:93949814-93956007	OtA5088-OtA5092	OtA5093-OtA5097
Gpr153	chr4:152274361-152285337	OtA5088-OtA5092	OtA5093-OtA5097
Rgma	chr7:73313805-73419899	OtA5088-OtA5092	OtA5093-OtA5097
Fah	chr7:84585158-84605942	OtA5088-OtA5092	OtA5093-OtA5097
Fen1	chr19:10199138-10203943	OtA5088-OtA5092	OtA5093-OtA5097
Scarb1	chr5:125277086-125341094	OtA5088-OtA5092	OtA5093-OtA5097
Tpm3	chr3:90072650-90100902	OtA5088-OtA5092	OtA5093-OtA5097
Amn	chr12:111271110-111276426	OtA5088-OtA5092	OtA5093-OtA5097
Dkk1	chr19:30545884-30549496	OtA5088-OtA5092	OtA5093-OtA5097
Lsm5	chr6:56701062-56704699	OtA5088-OtA5092	OtA5093-OtA5097
Senp1	chr15:98038743-98132447	OtA5088-OtA5092	OtA5093-OtA5097
Orc4	chr2:48814108-48949267	OtA5088-OtA5092	OtA5093-OtA5097
Krt13	chr11:100117595-100121495	OtA5088-OtA5092	OtA5093-OtA5097
C2cd3	chr7:100372232-100470159	OtA5088-OtA5092	OtA5093-OtA5097
Ddi2	chr4:141683562-141723419	OtA5088-OtA5092	OtA5093-OtA5097
Ston2	chr12:91633008-91786436	OtA5088-OtA5092	OtA5093-OtA5097
Synj2bp	chr12:81492193-81532907	OtA5088-OtA5092	OtA5093-OtA5097
Gtf2f1	chr17:57003401-57011288	OtA5088-OtA5092	OtA5093-OtA5097
Mttp	chr3:138089854-138143388	OtA5088-OtA5092	OtA5093-OtA5097
Pop7	chr5:137501438-137502429	OtA5088-OtA5092	OtA5093-OtA5097
Cox5b	chr1:36691486-36693388	OtA5088-OtA5092	OtA5093-OtA5097
Arid3b	chr9:57790504-57834234	OtA5088-OtA5092	OtA5093-OtA5097
Ankrd49	chr9:14780197-14782964	OtA5088-OtA5092	OtA5093-OtA5097
Cck	chr9:121489823-121495689	OtA5088-OtA5092	OtA5093-OtA5097
Secisbp2l	chr2:125736985-125782870	OtA5088-OtA5092	OtA5093-OtA5097
Rc3h1	chr1:160906410-160974976	OtA5088-OtA5092	OtA5093-OtA5097
Apobr	chr7:126585007-126589092	OtA5088-OtA5092	OtA5093-OtA5097
Bahcc1	chr11:120232946-120292297	OtA5088-OtA5092	OtA5093-OtA5097
Hoxa11	chr6:52242105-52249769	OtA5088-OtA5092	OtA5093-OtA5097
Pex7	chr10:19860089-19907674	OtA5088-OtA5092	OtA5093-OtA5097
Slc2a1	chr4:119108744-119137329	OtA5088-OtA5092	OtA5093-OtA5097
Wasf1	chr10:40883533-40938569	OtA5088-OtA5092	OtA5093-OtA5097
Gm2382	chr9:88688604-88719798	OtA5088-OtA5092	OtA5093-OtA5097
Phactr1	chr13:42680622-43138512	OtA5088-OtA5092	OtA5093-OtA5097
Tpcn1	chr5:120534156-120588613	OtA5088-OtA5092	OtA5093-OtA5097
Dok1	chr6:83030935-83033471	OtA5088-OtA5092	OtA5093-OtA5097
Hs1bp3	chr12:8313432-8343824	OtA5088-OtA5092	OtA5093-OtA5097
Rpl31	chr1:39367850-39478747	OtA5088-OtA5092	OtA5093-OtA5097
Sltm	chr9:70542777-70592232	OtA5088-OtA5092	OtA5093-OtA5097
Wdr61	chr9:54717154-54734528	OtA5088-OtA5092	OtA5093-OtA5097
Chst13	chr6:90308350-90325185	OtA5088-OtA5092	OtA5093-OtA5097
Col16a1	chr4:130047839-130099277	OtA5088-OtA5092	OtA5093-OtA5097
Gon4l	chr3:88835230-88913950	OtA5088-OtA5092	OtA5093-OtA5097

Lpar5	chr6:125067919-125082472	OtA5088-OtA5092	OtA5093-OtA5097
Slc15a4	chr5:127595665-127617392	OtA5088-OtA5092	OtA5093-OtA5097
Slu7	chr11:43433730-43447981	OtA5088-OtA5092	OtA5093-OtA5097
Hspa5	chr2:34772089-34776529	OtA5088-OtA5092	OtA5093-OtA5097
Mpp7	chr18:7347961-7626863	OtA5088-OtA5092	OtA5093-OtA5097
Zfp37	chr4:62189542-62208546	OtA5088-OtA5092	OtA5093-OtA5097
Ndst2	chr14:20723729-20734562	OtA5088-OtA5092	OtA5093-OtA5097
Cdk2ap2	chr19:4097350-4099017	OtA5088-OtA5092	OtA5093-OtA5097
Dppa5a	chr9:78367053-78368199	OtA5088-OtA5092	OtA5093-OtA5097
Gm20300	chr10:30603194-30606634	OtA5088-OtA5092	OtA5093-OtA5097
Hspb8	chr5:116408494-116422864	OtA5088-OtA5092	OtA5093-OtA5097
Setx	chr2:29124991-29182471	OtA5088-OtA5092	OtA5093-OtA5097
Cdc25a	chr9:109875578-109893890	OtA5088-OtA5092	OtA5093-OtA5097
Lass5	chr15:99735591-99772515	OtA5088-OtA5092	OtA5093-OtA5097
Ddx56	chr11:6257544-6267729	OtA5088-OtA5092	OtA5093-OtA5097
Dynll1	chr5:115297109-115300990	OtA5088-OtA5092	OtA5093-OtA5097
Fastk	chr5:24441039-24445235	OtA5088-OtA5092	OtA5093-OtA5097
Glcci1	chr6:8509595-8597548	OtA5088-OtA5092	OtA5093-OtA5097
Gm6644	chr6:34303929-34317489	OtA5088-OtA5092	OtA5093-OtA5097
Igfbp2	chr1:72824479-72852471	OtA5088-OtA5092	OtA5093-OtA5097
Naa16	chr14:79334506-79390668	OtA5088-OtA5092	OtA5093-OtA5097
Ppp2r5e	chr12:75450880-75596200	OtA5088-OtA5092	OtA5093-OtA5097
Tmem106b	chr6:13069758-13089269	OtA5088-OtA5092	OtA5093-OtA5097
Zc3h11a	chr1:133619870-133661380	OtA5088-OtA5092	OtA5093-OtA5097
Zfp143	chr7:110061701-110095392	OtA5088-OtA5092	OtA5093-OtA5097
Zfp467	chr6:48427691-48445825	OtA5088-OtA5092	OtA5093-OtA5097
Cul4b	chrX:38531620-38576196	OtA5088-OtA5092	OtA5093-OtA5097
Hist1h2be	chr13:23583669-23622558	OtA5088-OtA5092	OtA5093-OtA5097
Shmt1	chr11:60788896-60811265	OtA5088-OtA5092	OtA5093-OtA5097
Denr	chr5:123907274-123928832	OtA5088-OtA5092	OtA5093-OtA5097
Ulk2	chr11:61775597-61855092	OtA5088-OtA5092	OtA5093-OtA5097
Prkd2	chr7:16842901-16870461	OtA5088-OtA5092	OtA5093-OtA5097
Wdr5	chr2:27515146-27536538	OtA5088-OtA5092	OtA5093-OtA5097
Kap	chr6:133849854-133853667	OtA5088-OtA5092	OtA5093-OtA5097
Hist1h2ai	chr13:21716421-21716814	OtA5088-OtA5092	OtA5093-OtA5097
Ssh2	chr11:77216424-77460219	OtA5088-OtA5092	OtA5093-OtA5097
Kdm5a	chr6:120324321-120444574	OtA5088-OtA5092	OtA5093-OtA5097
Fbxl21	chr13:56522507-56537786	OtA5088-OtA5092	OtA5093-OtA5097
Psrc1	chr3:108383803-108388231	OtA5088-OtA5092	OtA5093-OtA5097
Spry2	chr14:105891946-105896819	OtA5088-OtA5092	OtA5093-OtA5097
Cog4	chr8:110847023-110882234	OtA5088-OtA5092	OtA5093-OtA5097
Smarca5	chr8:80699942-80739459	OtA5088-OtA5092	OtA5093-OtA5097
Ncapd2	chr6:125168006-125191586	OtA5088-OtA5092	OtA5093-OtA5097
Rpl22	chr4:152325877-152334071	OtA5088-OtA5092	OtA5093-OtA5097
Cacnb3	chr15:98632219-98644530	OtA5088-OtA5092	OtA5093-OtA5097
Fam159a	chr4:108367776-108383349	OtA5088-OtA5092	OtA5093-OtA5097
Stk38	chr17:28970884-29007937	OtA5088-OtA5092	OtA5093-OtA5097

Lsm10	chr4:126096652-126098584	OtA5088-OtA5092	OtA5093-OtA5097
Ppfia4	chr1:134296782-134332928	OtA5088-OtA5092	OtA5093-OtA5097
Smg7	chr1:152836994-152902646	OtA5088-OtA5092	OtA5093-OtA5097
Tgm2	chr2:158116404-158146392	OtA5088-OtA5092	OtA5093-OtA5097
Alox5ap	chr5:149265057-149288047	OtA5088-OtA5092	OtA5093-OtA5097
Col12a1	chr9:79598990-79718678	OtA5088-OtA5092	OtA5093-OtA5097
E2f7	chr10:110745464-110787384	OtA5088-OtA5092	OtA5093-OtA5097
Kif5c	chr2:49619313-49774778	OtA5088-OtA5092	OtA5093-OtA5097
Pcnt	chr10:76351253-76442912	OtA5088-OtA5092	OtA5093-OtA5097
Pla2g6	chr15:79286227-79328371	OtA5088-OtA5092	OtA5093-OtA5097
Rab11fip1	chr8:27138772-27174646	OtA5088-OtA5092	OtA5093-OtA5097
Fbxw5	chr2:25500777-25505470	OtA5088-OtA5092	OtA5093-OtA5097
Npl	chr1:153503015-153549714	OtA5088-OtA5092	OtA5093-OtA5097
Zfp397	chr18:23954687-23964670	OtA5088-OtA5092	OtA5093-OtA5097
Laptm5	chr4:130913333-130936148	OtA5088-OtA5092	OtA5093-OtA5097
Stac2	chr11:98036623-98053462	OtA5088-OtA5092	OtA5093-OtA5097
Kdm2b	chr5:122870674-122989092	OtA5088-OtA5092	OtA5093-OtA5097
Nhedc2	chr3:135307699-135342767	OtA5088-OtA5092	OtA5093-OtA5097
Nop58	chr1:59685005-59711510	OtA5088-OtA5092	OtA5093-OtA5097
Rps6ka4	chr19:6829083-6840601	OtA5088-OtA5092	OtA5093-OtA5097
Mfsd7a	chr5:108441053-108448891	OtA5088-OtA5092	OtA5093-OtA5097
Xrcc4	chr13:89848913-90089608	OtA5088-OtA5092	OtA5093-OtA5097
Asap3	chr4:136206364-136246573	OtA5088-OtA5092	OtA5093-OtA5097
Plxna1	chr6:89316313-89362613	OtA5088-OtA5092	OtA5093-OtA5097
Uso1	chr5:92137937-92202795	OtA5088-OtA5092	OtA5093-OtA5097
Ptges2	chr2:32395889-32402740	OtA5088-OtA5092	OtA5093-OtA5097
Inpp1	chr1:52789419-52817688	OtA5088-OtA5092	OtA5093-OtA5097
Specc1l	chr10:75212072-75312400	OtA5088-OtA5092	OtA5093-OtA5097
Golt1a	chr1:133309822-133323026	OtA5088-OtA5092	OtA5093-OtA5097
Ssr2	chr3:88579670-88588413	OtA5088-OtA5092	OtA5093-OtA5097
Cdv3	chr9:103353101-103365780	OtA5088-OtA5092	OtA5093-OtA5097
Klf3	chr5:64803522-64830129	OtA5088-OtA5092	OtA5093-OtA5097
Nxt1	chr2:148672614-148676026	OtA5088-OtA5092	OtA5093-OtA5097
Slc30a2	chr4:134343045-134354484	OtA5088-OtA5092	OtA5093-OtA5097
Rprd1b	chr2:158028793-158091797	OtA5088-OtA5092	OtA5093-OtA5097
Atf7	chr15:102536643-102625421	OtA5088-OtA5092	OtA5093-OtA5097
Pfn1	chr11:70651846-70654650	OtA5088-OtA5092	OtA5093-OtA5097
Iigp1	chr18:60376028-60392629	OtA5088-OtA5092	OtA5093-OtA5097
Lrrc33	chr16:32142824-32165476	OtA5088-OtA5092	OtA5093-OtA5097
Cav1	chr6:17306334-17341328	OtA5088-OtA5092	OtA5093-OtA5097
Rbm33	chr5:28317188-28419242	OtA5088-OtA5092	OtA5093-OtA5097
Rnf19a	chr15:36239933-36283147	OtA5088-OtA5092	OtA5093-OtA5097
Ccdc56	chr11:101277969-101278948	OtA5088-OtA5092	OtA5093-OtA5097
Ccnt2	chr1:127774163-127804837	OtA5088-OtA5092	OtA5093-OtA5097
Gcfc1	chr16:90936096-91044379	OtA5088-OtA5092	OtA5093-OtA5097
Igfals	chr17:24878769-24882008	OtA5088-OtA5092	OtA5093-OtA5097
Proz	chr8:13060907-13075006	OtA5088-OtA5092	OtA5093-OtA5097

Anxa5	chr3:36448923-36475887	OtA5088-OtA5092	OtA5093-OtA5097
Prune	chr3:95253673-95282076	OtA5088-OtA5092	OtA5093-OtA5097
Poldip2	chr11:78512295-78522736	OtA5088-OtA5092	OtA5093-OtA5097
Ms4a6d	chr19:11586605-11604804	OtA5088-OtA5092	OtA5093-OtA5097
Eml3	chr19:8929693-8941582	OtA5088-OtA5092	OtA5093-OtA5097
Mest	chr6:30733505-30748466	OtA5088-OtA5092	OtA5093-OtA5097
Arhgef15	chr11:68943153-68956858	OtA5088-OtA5092	OtA5093-OtA5097
Ptpn3	chr4:57190840-57301837	OtA5088-OtA5092	OtA5093-OtA5097
Xiap	chrX:42067835-42109664	OtA5088-OtA5092	OtA5093-OtA5097
Bet1l	chr7:140853383-140856383	OtA5088-OtA5092	OtA5093-OtA5097
Rps11	chr7:45122387-45124389	OtA5088-OtA5092	OtA5093-OtA5097
Cpsf1	chr15:76576358-76607591	OtA5088-OtA5092	OtA5093-OtA5097
Spint1	chr2:119237359-119249513	OtA5088-OtA5092	OtA5093-OtA5097
Rpl10a	chr17:28328470-28331033	OtA5088-OtA5092	OtA5093-OtA5097
Eif3f	chr7:108934414-108941942	OtA5088-OtA5092	OtA5093-OtA5097
Ankrd16	chr2:11777752-11790323	OtA5088-OtA5092	OtA5093-OtA5097
Ctage5	chr12:59129745-59190220	OtA5088-OtA5092	OtA5093-OtA5097
Bcor	chrX:12036737-12160355	OtA5088-OtA5092	OtA5093-OtA5097
Cuzd1	chr7:131308553-131322292	OtA5088-OtA5092	OtA5093-OtA5097
Nrk	chrX:138914429-139009090	OtA5088-OtA5092	OtA5093-OtA5097
Trim46	chr3:89234176-89245897	OtA5088-OtA5092	OtA5093-OtA5097
Sgce	chr6:4674349-4747204	OtA5088-OtA5092	OtA5093-OtA5097
Tmem79	chr3:88328652-88334433	OtA5088-OtA5092	OtA5093-OtA5097
Ripply1	chrX:139779680-139782353	OtA5088-OtA5092	OtA5093-OtA5097
Atp9a	chr2:168634437-168741800	OtA5088-OtA5092	OtA5093-OtA5097
Nudt6	chr3:37404983-37419590	OtA5088-OtA5092	OtA5093-OtA5097
Slit1	chr19:41600256-41743856	OtA5088-OtA5092	OtA5093-OtA5097
Kif5b	chr18:6201004-6241524	OtA5088-OtA5092	OtA5093-OtA5097
Sqrdl	chr2:122765358-122809551	OtA5088-OtA5092	OtA5093-OtA5097
Trp53i11	chr2:93187583-93201757	OtA5088-OtA5092	OtA5093-OtA5097
Adamts3	chr5:89673840-89883334	OtA5088-OtA5092	OtA5093-OtA5097
Aqp1	chr6:55336298-55348555	OtA5088-OtA5092	OtA5093-OtA5097
Atxn7l1	chr12:33302514-33368277	OtA5088-OtA5092	OtA5093-OtA5097
BC006779	chr2:181227614-181242027	OtA5088-OtA5092	OtA5093-OtA5097
BC031353	chr9:74953052-75032468	OtA5088-OtA5092	OtA5093-OtA5097
Bmp7	chr2:172869686-172940293	OtA5088-OtA5092	OtA5093-OtA5097
Cited1	chrX:102247439-102251769	OtA5088-OtA5092	OtA5093-OtA5097
Clk4	chr11:51263170-51281764	OtA5088-OtA5092	OtA5093-OtA5097
Kif3c	chr12:3365131-3426747	OtA5088-OtA5092	OtA5093-OtA5097
Mgrn1	chr16:4886099-4941020	OtA5088-OtA5092	OtA5093-OtA5097
Nol7	chr13:43398375-43480973	OtA5088-OtA5092	OtA5093-OtA5097
Polr3f	chr2:144527744-144541779	OtA5088-OtA5092	OtA5093-OtA5097
Rabep1	chr11:70844762-70969973	OtA5088-OtA5092	OtA5093-OtA5097
Renbp	chrX:73922120-73930850	OtA5088-OtA5092	OtA5093-OtA5097
Sdhb	chr4:140961270-140979192	OtA5088-OtA5092	OtA5093-OtA5097
Slc25a36	chr9:97077010-97111041	OtA5088-OtA5092	OtA5093-OtA5097
Slc25a4	chr8:46207340-46211009	OtA5088-OtA5092	OtA5093-OtA5097

Taf1a	chr1:183388884-183410206	OtA5088-OtA5092	OtA5093-OtA5097
Thbs2	chr17:14665499-14694262	OtA5088-OtA5092	OtA5093-OtA5097
Trap1	chr16:4037144-4077810	OtA5088-OtA5092	OtA5093-OtA5097
Tsc1	chr2:28641242-28691167	OtA5088-OtA5092	OtA5093-OtA5097
Uimc1	chr13:55027879-55100295	OtA5088-OtA5092	OtA5093-OtA5097
Slc30a4	chr2:122681238-122702663	OtA5088-OtA5092	OtA5093-OtA5097
Vax2	chr6:83692805-83738304	OtA5088-OtA5092	OtA5093-OtA5097
Slx1b	chr7:126688926-126695783	OtA5088-OtA5092	OtA5093-OtA5097
Zfp623	chr15:75940951-75949400	OtA5088-OtA5092	OtA5093-OtA5097
Clns1a	chr7:97696656-97720793	OtA5088-OtA5092	OtA5093-OtA5097
Lrp1	chr10:127538157-127621148	OtA5088-OtA5092	OtA5093-OtA5097
Vac14	chr8:110618637-110720398	OtA5088-OtA5092	OtA5093-OtA5097
Sytl1	chr4:133253089-133263087	OtA5088-OtA5092	OtA5093-OtA5097
Slc35e1	chr8:72477994-72492614	OtA5088-OtA5092	OtA5093-OtA5097
Gbp4	chr5:105115766-105139586	OtA5088-OtA5092	OtA5093-OtA5097
Tulp4	chr17:6106829-6240637	OtA5088-OtA5092	OtA5093-OtA5097
Mtap4	chr9:109931773-110083954	OtA5088-OtA5092	OtA5093-OtA5097
Naa15	chr3:51416015-51475985	OtA5088-OtA5092	OtA5093-OtA5097
Nif3l1	chr1:58447632-58462276	OtA5088-OtA5092	OtA5093-OtA5097
Socs3	chr11:117966086-117969366	OtA5088-OtA5092	OtA5093-OtA5097
Arpc4	chr6:113378112-113414571	OtA5088-OtA5092	OtA5093-OtA5097
Chmp2b	chr16:65539132-65562697	OtA5088-OtA5092	OtA5093-OtA5097
Slc39a13	chr2:91061790-91070221	OtA5088-OtA5092	OtA5093-OtA5097
Lum	chr10:97565500-97572703	OtA5088-OtA5092	OtA5093-OtA5097
Fabp3	chr4:130308777-130315463	OtA5088-OtA5092	OtA5093-OtA5097
Hmgxb3	chr18:61131276-61177050	OtA5088-OtA5092	OtA5093-OtA5097
Rlim	chrX:103957166-103981284	OtA5088-OtA5092	OtA5093-OtA5097
Chrd	chr16:20724453-20742384	OtA5088-OtA5092	OtA5093-OtA5097
Klc1	chr12:111758848-111813841	OtA5088-OtA5092	OtA5093-OtA5097
Irf6	chr1:193153111-193172035	OtA5088-OtA5092	OtA5093-OtA5097
Enpp5	chr17:44078845-44086561	OtA5088-OtA5092	OtA5093-OtA5097
Fam189a2	chr19:23972749-24031019	OtA5088-OtA5092	OtA5093-OtA5097
Mllt10	chr2:18055236-18392830	OtA5088-OtA5092	OtA5093-OtA5097
Taf13	chr3:108571698-108582068	OtA5088-OtA5092	OtA5093-OtA5097
Ergic1	chr17:26561511-26656933	OtA5088-OtA5092	OtA5093-OtA5097
Arhgap1	chr2:91650117-91672320	OtA5088-OtA5092	OtA5093-OtA5097
Kank4	chr4:98754891-98817537	OtA5088-OtA5092	OtA5093-OtA5097
Magel2	chr7:62376978-62381640	OtA5088-OtA5092	OtA5093-OtA5097
Gabarapl2	chr8:111940702-111952915	OtA5088-OtA5092	OtA5093-OtA5097
Rhox6	chrX:37827054-37829857	OtA5088-OtA5092	OtA5093-OtA5097
Zim1	chr7:6675442-6696432	OtA5088-OtA5092	OtA5093-OtA5097
Tubb2a	chr13:34074279-34078008	OtA5088-OtA5092	OtA5093-OtA5097
Snora70	chrX:74270815-74273135	OtA5088-OtA5092	OtA5093-OtA5097
Gm98	chr19:10208270-10240748	OtA5088-OtA5092	OtA5093-OtA5097
Gpr115	chr17:42656890-42692284	OtA5088-OtA5092	OtA5093-OtA5097
H2afx	chr9:44334714-44336073	OtA5088-OtA5092	OtA5093-OtA5097
Bckdha	chr7:25629851-25658761	OtA5088-OtA5092	OtA5093-OtA5097

Zfp238	chr1:177444660-177450764	OtA5088-OtA5092	OtA5093-OtA5097
Lmo7	chr14:101729927-101934693	OtA5088-OtA5092	OtA5093-OtA5097
Hist1h2bn	chr13:21754122-21754503	OtA5088-OtA5092	OtA5093-OtA5097
Rb1cc1	chr1:6214661-6276104	OtA5088-OtA5092	OtA5093-OtA5097
Zmiz2	chr11:6389363-6406162	OtA5088-OtA5092	OtA5093-OtA5097
Dync1i2	chr2:71211705-71263302	OtA5088-OtA5092	OtA5093-OtA5097
Snrnp70	chr7:45376453-45395647	OtA5088-OtA5092	OtA5093-OtA5097
Alg9	chr9:50775224-50843639	OtA5088-OtA5092	OtA5093-OtA5097
Atf2	chr2:73816521-73892628	OtA5088-OtA5092	OtA5093-OtA5097
C330007P06Rik	chrX:36848543-36864246	OtA5088-OtA5092	OtA5093-OtA5097
Dab2	chr15:6299788-6440709	OtA5088-OtA5092	OtA5093-OtA5097
Hnrnpu	chr1:178328299-178337784	OtA5088-OtA5092	OtA5093-OtA5097
Ino80d	chr1:63047800-63114267	OtA5088-OtA5092	OtA5093-OtA5097
Klf8	chrX:153238044-153396134	OtA5088-OtA5092	OtA5093-OtA5097
Letm1	chr5:33741351-33782704	OtA5088-OtA5092	OtA5093-OtA5097
Mapre1	chr2:153741286-153773314	OtA5088-OtA5092	OtA5093-OtA5097
Mmadhc	chr2:50279880-50296677	OtA5088-OtA5092	OtA5093-OtA5097
Mxra7	chr11:116803399-116828046	OtA5088-OtA5092	OtA5093-OtA5097
Nudt8	chr19:4000579-4002102	OtA5088-OtA5092	OtA5093-OtA5097
Pelp1	chr11:70392880-70410031	OtA5088-OtA5092	OtA5093-OtA5097
Pole4	chr6:82646711-82652865	OtA5088-OtA5092	OtA5093-OtA5097
Ppm1h	chr10:122678761-122945793	OtA5088-OtA5092	OtA5093-OtA5097
Ppp1r12b	chr1:134765942-134955940	OtA5088-OtA5092	OtA5093-OtA5097
Ppp6r3	chr19:3454928-3575749	OtA5088-OtA5092	OtA5093-OtA5097
Senp2	chr16:22009483-22049269	OtA5088-OtA5092	OtA5093-OtA5097
Serp2	chr14:76532811-76556889	OtA5088-OtA5092	OtA5093-OtA5097
Snx9	chr17:5841379-5930711	OtA5088-OtA5092	OtA5093-OtA5097
Upk1b	chr16:38773183-38800203	OtA5088-OtA5092	OtA5093-OtA5097
Aars	chr8:111033966-111057599	OtA5088-OtA5092	OtA5093-OtA5097
F11	chr8:45241169-45262031	OtA5088-OtA5092	OtA5093-OtA5097
Dact2	chr17:14195229-14203831	OtA5088-OtA5092	OtA5093-OtA5097
Fgfr1op	chr17:8165517-8196470	OtA5088-OtA5092	OtA5093-OtA5097
Dynlt3	chrX:9654269-9662983	OtA5088-OtA5092	OtA5093-OtA5097
Rin3	chr12:102283073-102390854	OtA5088-OtA5092	OtA5093-OtA5097
Gpr137b	chr13:13357619-13393624	OtA5088-OtA5092	OtA5093-OtA5097
Ankhd1	chr18:36560602-36658908	OtA5088-OtA5092	OtA5093-OtA5097
Dhx30	chr9:110084318-110117616	OtA5088-OtA5092	OtA5093-OtA5097
H2-Q2	chr17:35342332-35345722	OtA5088-OtA5092	OtA5093-OtA5097
Scarna13	chr12:105030616-105032279	OtA5088-OtA5092	OtA5093-OtA5097
Eif3l	chr15:79075222-79094400	OtA5088-OtA5092	OtA5093-OtA5097
Asxl1	chr2:153346138-153404007	OtA5088-OtA5092	OtA5093-OtA5097
Traf4	chr11:78158422-78165550	OtA5088-OtA5092	OtA5093-OtA5097
Anxa3	chr5:96793384-96845968	OtA5088-OtA5092	OtA5093-OtA5097
Ddx5	chr11:106780355-106788494	OtA5088-OtA5092	OtA5093-OtA5097
Mapkapk2	chr1:131053703-131097543	OtA5088-OtA5092	OtA5093-OtA5097
Nfatc1	chr18:80606204-80713071	OtA5088-OtA5092	OtA5093-OtA5097
Pno1	chr11:17203199-17211589	OtA5088-OtA5092	OtA5093-OtA5097

Fnta	chr8:25998721-26015601	OtA5088-OtA5092	OtA5093-OtA5097
Akt2	chr7:27591559-27639453	OtA5088-OtA5092	OtA5093-OtA5097
Corin	chr5:72300024-72504540	OtA5088-OtA5092	OtA5093-OtA5097
Aldoa	chr7:126795233-126800447	OtA5088-OtA5092	OtA5093-OtA5097
Gimap6	chr6:48701582-48708244	OtA5088-OtA5092	OtA5093-OtA5097
Pla2g4e	chr2:120166411-120245335	OtA5088-OtA5092	OtA5093-OtA5097
Leng8	chr7:4137055-4148173	OtA5088-OtA5092	OtA5093-OtA5097
Fam125a	chr8:71542929-71548026	OtA5088-OtA5092	OtA5093-OtA5097
Golga2	chr2:32288252-32307921	OtA5088-OtA5092	OtA5093-OtA5097
Mbtd1	chr11:93886218-93946984	OtA5088-OtA5092	OtA5093-OtA5097
Ncln	chr10:81486458-81496363	OtA5088-OtA5092	OtA5093-OtA5097
Panx1	chr9:15005784-15045478	OtA5088-OtA5092	OtA5093-OtA5097
Psg17	chr7:18813936-18821591	OtA5088-OtA5092	OtA5093-OtA5097
Pgam5	chr5:110259134-110269899	OtA5088-OtA5092	OtA5093-OtA5097
Ric8	chr7:140857396-140882309	OtA5088-OtA5092	OtA5093-OtA5097
Strn3	chr12:51608540-51738939	OtA5088-OtA5092	OtA5093-OtA5097
Larp4	chr15:99970073-100016358	OtA5088-OtA5092	OtA5093-OtA5097
Pglyrp1	chr7:18884689-18890438	OtA5088-OtA5092	OtA5093-OtA5097
Bmp10	chr6:87429001-87434512	OtA5088-OtA5092	OtA5093-OtA5097
Degs2	chr12:108410654-108702306	OtA5088-OtA5092	OtA5093-OtA5097
Phkg1	chr5:129846989-129879083	OtA5088-OtA5092	OtA5093-OtA5097
Rtn4rl1	chr11:75193992-75267762	OtA5088-OtA5092	OtA5093-OtA5097
Tpst2	chr5:112276706-112315356	OtA5088-OtA5092	OtA5093-OtA5097
Tspan12	chr6:21771394-21852515	OtA5088-OtA5092	OtA5093-OtA5097
Zfyve21	chr12:111814169-111828388	OtA5088-OtA5092	OtA5093-OtA5097
Wapal	chr14:34673927-34746217	OtA5088-OtA5092	OtA5093-OtA5097
Raf1	chr6:115601937-115676635	OtA5088-OtA5092	OtA5093-OtA5097
Cks2	chr13:51645231-51650662	OtA5088-OtA5092	OtA5093-OtA5097
Arap1	chr7:101348068-101412586	OtA5088-OtA5092	OtA5093-OtA5097
BC018507	chr13:70588688-70637634	OtA5088-OtA5092	OtA5093-OtA5097
Fkbp8	chr8:70527742-70535328	OtA5088-OtA5092	OtA5093-OtA5097
Mfn2	chr4:147873594-147904819	OtA5088-OtA5092	OtA5093-OtA5097
Pde4a	chr9:21165713-21213248	OtA5088-OtA5092	OtA5093-OtA5097
Pphln1	chr15:93398349-93491912	OtA5088-OtA5092	OtA5093-OtA5097
Smarcc2	chr10:128459235-128490174	OtA5088-OtA5092	OtA5093-OtA5097
Lass2	chr3:95315251-95357202	OtA5088-OtA5092	OtA5093-OtA5097
Gpc1	chr1:92831685-92860196	OtA5088-OtA5092	OtA5093-OtA5097
Gm11974	chr11:6525590-6528760	OtA5088-OtA5092	OtA5093-OtA5097
Trank1	chr9:111311738-111395775	OtA5088-OtA5092	OtA5093-OtA5097
Rc3h2	chr2:37370070-37422903	OtA5088-OtA5092	OtA5093-OtA5097
Hadhb	chr5:30155321-30184583	OtA5088-OtA5092	OtA5093-OtA5097
Orai3	chr7:127769814-127775150	OtA5088-OtA5092	OtA5093-OtA5097
Ranbp10	chr8:105768307-105827350	OtA5088-OtA5092	OtA5093-OtA5097
Slc25a37	chr14:69241850-69285103	OtA5088-OtA5092	OtA5093-OtA5097
Pim1	chr17:29491044-29495459	OtA5088-OtA5092	OtA5093-OtA5097
Cox8a	chr19:7215157-7217616	OtA5088-OtA5092	OtA5093-OtA5097
Mapkbp1	chr2:119972698-120027403	OtA5088-OtA5092	OtA5093-OtA5097

Rsrc2	chr5:123728429-123749412	OtA5088-OtA5092	OtA5093-OtA5097
Bag6	chr17:35135177-35147322	OtA5088-OtA5092	OtA5093-OtA5097
Rcor2	chr19:7269763-7275225	OtA5088-OtA5092	OtA5093-OtA5097
Rad50	chr11:53649518-53707319	OtA5088-OtA5092	OtA5093-OtA5097
Shpk	chr11:73199481-73224506	OtA5088-OtA5092	OtA5093-OtA5097
Loh12cr1	chr6:134639511-134711184	OtA5088-OtA5092	OtA5093-OtA5097
Hist1h3i	chr13:21782914-21783397	OtA5088-OtA5092	OtA5093-OtA5097
Plekha4	chr7:45526329-45554229	OtA5088-OtA5092	OtA5093-OtA5097
Clybl	chr14:122181693-122451146	OtA5088-OtA5092	OtA5093-OtA5097
Nup37	chr10:88146991-88178395	OtA5088-OtA5092	OtA5093-OtA5097
Csrnp2	chr15:100479569-100495239	OtA5088-OtA5092	OtA5093-OtA5097
Plac8	chr5:100553732-100572205	OtA5088-OtA5092	OtA5093-OtA5097
Tmem43	chr6:91473750-91488458	OtA5088-OtA5092	OtA5093-OtA5097
S100a6	chr3:90612893-90614414	OtA5088-OtA5092	OtA5093-OtA5097
Kctd12b	chrX:153685153-153696280	OtA5088-OtA5092	OtA5093-OtA5097
Aff2	chrX:69360330-69868037	OtA5088-OtA5092	OtA5093-OtA5097
Atp8b2	chr3:89939480-89963508	OtA5088-OtA5092	OtA5093-OtA5097
Atxn10	chr15:85336380-85463836	OtA5088-OtA5092	OtA5093-OtA5097
Dopey2	chr16:93711906-93810588	OtA5088-OtA5092	OtA5093-OtA5097
Fam3c	chr6:22306521-22356081	OtA5088-OtA5092	OtA5093-OtA5097
Galntl4	chr7:111471660-111779977	OtA5088-OtA5092	OtA5093-OtA5097
Gata1	chrX:7959259-7967815	OtA5088-OtA5092	OtA5093-OtA5097
Hdac5	chr11:102195746-102230172	OtA5088-OtA5092	OtA5093-OtA5097
Mtmr12	chr15:12205093-12272240	OtA5088-OtA5092	OtA5093-OtA5097
Ppp2r1b	chr9:50856934-51009073	OtA5088-OtA5092	OtA5093-OtA5097
Stx7	chr10:24149316-24188959	OtA5088-OtA5092	OtA5093-OtA5097
Tlk1	chr2:70712407-70825480	OtA5088-OtA5092	OtA5093-OtA5097
Tmem111	chr6:113514886-113531638	OtA5088-OtA5092	OtA5093-OtA5097
Foxs1	chr2:152931897-152933208	OtA5088-OtA5092	OtA5093-OtA5097
Impdh2	chr9:108560500-108565566	OtA5088-OtA5092	OtA5093-OtA5097
Ndufs2	chr1:171234859-171247112	OtA5088-OtA5092	OtA5093-OtA5097
Cgnl1	chr9:71626508-71771602	OtA5088-OtA5092	OtA5093-OtA5097
Letmd1	chr15:100469033-100479252	OtA5088-OtA5092	OtA5093-OtA5097
Baz1a	chr12:54892988-54986336	OtA5088-OtA5092	OtA5093-OtA5097
Tppp3	chr8:105467491-105471422	OtA5088-OtA5092	OtA5093-OtA5097
Dbi	chr1:120113279-120121096	OtA5088-OtA5092	OtA5093-OtA5097
Hsph1	chr5:149616844-149636315	OtA5088-OtA5092	OtA5093-OtA5097
Slc10a3	chrX:74369218-74373349	OtA5088-OtA5092	OtA5093-OtA5097
Tmem128	chr5:38260374-38269618	OtA5088-OtA5092	OtA5093-OtA5097
Birc3	chr9:7848700-7873170	OtA5088-OtA5092	OtA5093-OtA5097
Sipa1l1	chr12:82170015-82451784	OtA5088-OtA5092	OtA5093-OtA5097
Prkce	chr17:86167784-86657919	OtA5088-OtA5092	OtA5093-OtA5097
Nsun6	chr2:14995130-15054872	OtA5088-OtA5092	OtA5093-OtA5097
Pdgfb	chr15:79995875-80014808	OtA5088-OtA5092	OtA5093-OtA5097
Cbx2	chr11:119023028-119031270	OtA5088-OtA5092	OtA5093-OtA5097
Fxyd2	chr9:45399708-45410278	OtA5088-OtA5092	OtA5093-OtA5097
Slc6a8	chrX:73673132-73682500	OtA5088-OtA5092	OtA5093-OtA5097

Trip11	chr12:101837371-101913171	OtA5088-OtA5092	OtA5093-OtA5097
Crls1	chr2:132846665-132866768	OtA5088-OtA5092	OtA5093-OtA5097
Itpkc	chr7:27207169-27228597	OtA5088-OtA5092	OtA5093-OtA5097
Mef2a	chr7:67233826-67372858	OtA5088-OtA5092	OtA5093-OtA5097
Trmt2b	chrX:134222954-134276984	OtA5088-OtA5092	OtA5093-OtA5097
Slc39a7	chr17:34028265-34031690	OtA5088-OtA5092	OtA5093-OtA5097
Mphosph8	chr14:56668247-56701000	OtA5088-OtA5092	OtA5093-OtA5097
Acox2	chr14:8225510-8259019	OtA5088-OtA5092	OtA5093-OtA5097
Iqsec2	chrX:152144267-152225236	OtA5088-OtA5092	OtA5093-OtA5097
Atp13a2	chr4:140986872-141007701	OtA5088-OtA5092	OtA5093-OtA5097
Dock1	chr7:134670686-135173647	OtA5088-OtA5092	OtA5093-OtA5097
Ddr1	chr17:35681566-35704139	OtA5088-OtA5092	OtA5093-OtA5097
Etnk2	chr1:133363571-133380319	OtA5088-OtA5092	OtA5093-OtA5097
Ndufb5	chr3:32737062-32751559	OtA5088-OtA5092	OtA5093-OtA5097
Nxf7	chrX:135579786-135593855	OtA5088-OtA5092	OtA5093-OtA5097
Nip7	chr8:107056876-107060928	OtA5088-OtA5092	OtA5093-OtA5097
Cldn3	chr5:134986213-135001350	OtA5088-OtA5092	OtA5093-OtA5097
Inpp5a	chr7:139389108-139582797	OtA5088-OtA5092	OtA5093-OtA5097
Flcn	chr11:59791750-59809773	OtA5088-OtA5092	OtA5093-OtA5097
Gcdh	chr8:84872110-84893921	OtA5088-OtA5092	OtA5093-OtA5097
Chm	chrX:113040591-113185515	OtA5088-OtA5092	OtA5093-OtA5097
Fcrls	chr3:87250964-87263524	OtA5088-OtA5092	OtA5093-OtA5097
Ggt5	chr10:75589380-75616968	OtA5088-OtA5092	OtA5093-OtA5097
Mafg	chr11:120628350-120633547	OtA5088-OtA5092	OtA5093-OtA5097
Mkl2	chr16:13256480-13417529	OtA5088-OtA5092	OtA5093-OtA5097
Pds5b	chr5:150673826-150810669	OtA5088-OtA5092	OtA5093-OtA5097
Prpf39	chr12:65036333-65073938	OtA5088-OtA5092	OtA5093-OtA5097
Relb	chr7:19606221-19629438	OtA5088-OtA5092	OtA5093-OtA5097
Safb2	chr17:56562941-56584583	OtA5088-OtA5092	OtA5093-OtA5097
Serpina1b	chr12:103728155-103738189	OtA5088-OtA5092	OtA5093-OtA5097
Slc26a1	chr5:108669105-108684558	OtA5088-OtA5092	OtA5093-OtA5097
Tmem154	chr3:84666191-84704575	OtA5088-OtA5092	OtA5093-OtA5097
Tpcn2	chr7:145253922-145283927	OtA5088-OtA5092	OtA5093-OtA5097
Ubap2	chr4:41194314-41275135	OtA5088-OtA5092	OtA5093-OtA5097
Pfkfb2	chr1:130689042-130729253	OtA5088-OtA5092	OtA5093-OtA5097
Fig4	chr10:41188171-41303241	OtA5088-OtA5092	OtA5093-OtA5097
Hnrnpl	chr7:28810889-28822266	OtA5088-OtA5092	OtA5093-OtA5097
Nbl1	chr4:139082291-139092970	OtA5088-OtA5092	OtA5093-OtA5097
Pex1	chr5:3571715-3637101	OtA5088-OtA5092	OtA5093-OtA5097
Ppm1b	chr17:84917181-85023992	OtA5088-OtA5092	OtA5093-OtA5097
Pld1	chr3:27938679-28133362	OtA5088-OtA5092	OtA5093-OtA5097
Endod1	chr9:14353989-14381242	OtA5088-OtA5092	OtA5093-OtA5097
Dynll2	chr11:87979524-87998298	OtA5088-OtA5092	OtA5093-OtA5097
Srgap2	chr1:131285250-131527361	OtA5088-OtA5092	OtA5093-OtA5097
Havcr1	chr11:46740222-46779578	OtA5088-OtA5092	OtA5093-OtA5097
Stard4	chr18:33201420-33213816	OtA5088-OtA5092	OtA5093-OtA5097
Ebf4	chr2:130295938-130370481	OtA5088-OtA5092	OtA5093-OtA5097

Ppp1r13l	chr7:19361215-19378533	OtA5088-OtA5092	OtA5093-OtA5097
Sin3a	chr9:57072039-57128368	OtA5088-OtA5092	OtA5093-OtA5097
Brd7	chr8:88332310-88362191	OtA5088-OtA5092	OtA5093-OtA5097
Ncrna00085	chr17:17830974-17839071	OtA5088-OtA5092	OtA5093-OtA5097
Ppip5k1	chr2:121289601-121351013	OtA5088-OtA5092	OtA5093-OtA5097
Ptges3	chr10:128058981-128077254	OtA5088-OtA5092	OtA5093-OtA5097
Serhl	chr15:83100204-83122801	OtA5088-OtA5092	OtA5093-OtA5097
Slc25a11	chr11:70644026-70647039	OtA5088-OtA5092	OtA5093-OtA5097
Spice1	chr16:44347400-44388492	OtA5088-OtA5092	OtA5093-OtA5097
Tkt	chr14:30549148-30574722	OtA5088-OtA5092	OtA5093-OtA5097
Usp27x	chrX:7372590-7375830	OtA5088-OtA5092	OtA5093-OtA5097
Alyref2	chr1:171503477-171504750	OtA5088-OtA5092	OtA5093-OtA5097
Rpl38	chr11:114344525-114757886	OtA5088-OtA5092	OtA5093-OtA5097
Rprd1a	chr18:24484961-24530204	OtA5088-OtA5092	OtA5093-OtA5097
Samm50	chr15:84192232-84214303	OtA5088-OtA5092	OtA5093-OtA5097
Zfml	chr6:83914352-83986871	OtA5088-OtA5092	OtA5093-OtA5097
Gm648	chrX:56543873-56549606	OtA5088-OtA5092	OtA5093-OtA5097
Ccnl2	chr4:155812488-155824543	OtA5088-OtA5092	OtA5093-OtA5097
Cdc40	chr10:40831621-40883143	OtA5088-OtA5092	OtA5093-OtA5097
Vps54	chr11:21238888-21321133	OtA5088-OtA5092	OtA5093-OtA5097
Ephb6	chr6:41605481-41620507	OtA5088-OtA5092	OtA5093-OtA5097
Pnkp	chr7:44857180-44862929	OtA5088-OtA5092	OtA5093-OtA5097
Ddah1	chr3:145758691-145894277	OtA5088-OtA5092	OtA5093-OtA5097
Hps4	chr5:112343094-112378424	OtA5088-OtA5092	OtA5093-OtA5097
Ints5	chr19:8892986-8897890	OtA5088-OtA5092	OtA5093-OtA5097
Osgin2	chr4:15997120-16013877	OtA5088-OtA5092	OtA5093-OtA5097
Rarres1	chr3:67478885-67515523	OtA5088-OtA5092	OtA5093-OtA5097
Tfdp2	chr9:96196274-96323646	OtA5088-OtA5092	OtA5093-OtA5097
Zfp964	chr8:69654555-69664453	OtA5088-OtA5092	OtA5093-OtA5097
Pnp2	chr14:50956140-50964749	OtA5088-OtA5092	OtA5093-OtA5097
Decr1	chr4:15917239-15945507	OtA5088-OtA5092	OtA5093-OtA5097
Lims2	chr18:31931506-31958619	OtA5088-OtA5092	OtA5093-OtA5097
Limch1	chr5:66745839-67057159	OtA5088-OtA5092	OtA5093-OtA5097
Pibf1	chr14:99076633-99254493	OtA5088-OtA5092	OtA5093-OtA5097
D10Bwg1379e	chr10:18588010-18743758	OtA5088-OtA5092	OtA5093-OtA5097
Ptpn12	chr5:20986644-21055797	OtA5088-OtA5092	OtA5093-OtA5097
Slc25a32	chr15:39094190-39112716	OtA5088-OtA5092	OtA5093-OtA5097
Kifc3	chr8:95081200-95142540	OtA5088-OtA5092	OtA5093-OtA5097
Relt	chr7:100845847-100863413	OtA5088-OtA5092	OtA5093-OtA5097
Yif1a	chr19:5088537-5098418	OtA5088-OtA5092	OtA5093-OtA5097
Ddb1	chr19:10605624-10629821	OtA5088-OtA5092	OtA5093-OtA5097
Dnase1l3	chr14:7965189-7994182	OtA5088-OtA5092	OtA5093-OtA5097
Gbf1	chr19:46152557-46286510	OtA5088-OtA5092	OtA5093-OtA5097
Gprc5b	chr7:118842217-118995211	OtA5088-OtA5092	OtA5093-OtA5097
Laptm4a	chr12:8921306-8938741	OtA5088-OtA5092	OtA5093-OtA5097
Setd1a	chr7:127777388-127800119	OtA5088-OtA5092	OtA5093-OtA5097
Tgif2	chr2:156840076-156862945	OtA5088-OtA5092	OtA5093-OtA5097

Tmem181a	chr17:6270474-6305783	OtA5088-OtA5092	OtA5093-OtA5097
Trib2	chr12:15791726-15816785	OtA5088-OtA5092	OtA5093-OtA5097
Cenpt	chr8:105844677-105852008	OtA5088-OtA5092	OtA5093-OtA5097
Csnk2b	chr17:35116194-35121447	OtA5088-OtA5092	OtA5093-OtA5097
Esam	chr9:37528088-37538319	OtA5088-OtA5092	OtA5093-OtA5097
Fam76a	chr4:132899212-132922551	OtA5088-OtA5092	OtA5093-OtA5097
Mgst3	chr1:167372383-167393797	OtA5088-OtA5092	OtA5093-OtA5097
Mrpl10	chr11:97041585-97049213	OtA5088-OtA5092	OtA5093-OtA5097
Nhs	chrX:161836267-162159790	OtA5088-OtA5092	OtA5093-OtA5097
Pcdh12	chr18:38267091-38284401	OtA5088-OtA5092	OtA5093-OtA5097
Pin1	chr9:20652129-20666584	OtA5088-OtA5092	OtA5093-OtA5097
Zfp36	chr7:28376783-28379228	OtA5088-OtA5092	OtA5093-OtA5097
Aif1l	chr2:31950302-31973442	OtA5088-OtA5092	OtA5093-OtA5097
Egln3	chr12:54178980-54203874	OtA5088-OtA5092	OtA5093-OtA5097
Il10ra	chr9:45253838-45269146	OtA5088-OtA5092	OtA5093-OtA5097
Kif3a	chr11:53567378-53604244	OtA5088-OtA5092	OtA5093-OtA5097
Pcmtd2	chr2:181837902-181857450	OtA5088-OtA5092	OtA5093-OtA5097
Pou3f1	chr4:124657645-124660655	OtA5088-OtA5092	OtA5093-OtA5097
Prodh	chr16:18071725-18089190	OtA5088-OtA5092	OtA5093-OtA5097
Rasa1	chr13:85214698-85289486	OtA5088-OtA5092	OtA5093-OtA5097
Stat6	chr10:127642985-127666703	OtA5088-OtA5092	OtA5093-OtA5097
Zzz3	chr3:152396009-152681432	OtA5088-OtA5092	OtA5093-OtA5097
Ammecr1	chrX:142853473-142966728	OtA5088-OtA5092	OtA5093-OtA5097
Bst2	chr8:71534261-71537437	OtA5088-OtA5092	OtA5093-OtA5097
Dera	chr6:137754576-137837872	OtA5088-OtA5092	OtA5093-OtA5097
Maea	chr5:33335571-33373294	OtA5088-OtA5092	OtA5093-OtA5097
Map2k2	chr10:81105946-81124697	OtA5088-OtA5092	OtA5093-OtA5097
Ndufc1	chr3:51405478-51408955	OtA5088-OtA5092	OtA5093-OtA5097
Spg21	chr9:65460936-65488470	OtA5088-OtA5092	OtA5093-OtA5097
Arl13b	chr16:62793688-62847040	OtA5088-OtA5092	OtA5093-OtA5097
Lrrc20	chr10:61475832-61582228	OtA5088-OtA5092	OtA5093-OtA5097
Rpl36a	chrX:134585653-134588062	OtA5088-OtA5092	OtA5093-OtA5097
l7Rn6	chr7:89918688-89941185	OtA5088-OtA5092	OtA5093-OtA5097
Cdk11b	chr4:155624868-155649932	OtA5088-OtA5092	OtA5093-OtA5097
Arhgap27	chr11:103331483-103363692	OtA5088-OtA5092	OtA5093-OtA5097
Islr2	chr9:58196296-58208808	OtA5088-OtA5092	OtA5093-OtA5097
Xab2	chr8:3610089-3621296	OtA5088-OtA5092	OtA5093-OtA5097
Zfp131	chr13:119765186-119790805	OtA5088-OtA5092	OtA5093-OtA5097
Wdr5b	chr16:36041189-36042974	OtA5088-OtA5092	OtA5093-OtA5097
Pdcl3	chr1:38987813-38997236	OtA5088-OtA5092	OtA5093-OtA5097
Lrch3	chr16:32914099-33056186	OtA5088-OtA5092	OtA5093-OtA5097
Chsy1	chr7:66109514-66173798	OtA5088-OtA5092	OtA5093-OtA5097
Ivns1abp	chr1:151344497-151364445	OtA5088-OtA5092	OtA5093-OtA5097
Tjap1	chr17:46257850-46283026	OtA5088-OtA5092	OtA5093-OtA5097
Angptl2	chr2:33136972-33371478	OtA5088-OtA5092	OtA5093-OtA5097
Atl3	chr19:7494039-7538609	OtA5088-OtA5092	OtA5093-OtA5097
Ppm1d	chr11:85311253-85347071	OtA5088-OtA5092	OtA5093-OtA5097

Slc25a12	chr2:71274294-71367554	OtA5088-OtA5092	OtA5093-OtA5097
Ankle2	chr5:110231003-110256651	OtA5088-OtA5092	OtA5093-OtA5097
Igf2bp3	chr6:49085217-49214954	OtA5088-OtA5092	OtA5093-OtA5097
Nfic	chr10:81396190-81427173	OtA5088-OtA5092	OtA5093-OtA5097
A4galt	chr15:83226721-83251774	OtA5088-OtA5092	OtA5093-OtA5097
Ermap	chr4:119175456-119190011	OtA5088-OtA5092	OtA5093-OtA5097
Fst	chr13:114452261-114458730	OtA5088-OtA5092	OtA5093-OtA5097
Gtf2a1	chr12:91555261-91590487	OtA5088-OtA5092	OtA5093-OtA5097
Hccs	chrX:169311530-169320343	OtA5088-OtA5092	OtA5093-OtA5097
Il13ra1	chrX:36112138-36171261	OtA5088-OtA5092	OtA5093-OtA5097
Ptpn13	chr5:103425191-103598361	OtA5088-OtA5092	OtA5093-OtA5097
Srsf5	chr12:80945503-80950507	OtA5088-OtA5092	OtA5093-OtA5097
Tceb1	chr1:16642764-16656865	OtA5088-OtA5092	OtA5093-OtA5097
Pcdh1	chr18:38196693-38209762	OtA5088-OtA5092	OtA5093-OtA5097
Atp6ap2	chrX:12587758-12617051	OtA5088-OtA5092	OtA5093-OtA5097
Ccdc93	chr1:121431066-121506460	OtA5088-OtA5092	OtA5093-OtA5097
Cd38	chr5:43868826-43912374	OtA5088-OtA5092	OtA5093-OtA5097
Cmtm8	chr9:114789344-114844152	OtA5088-OtA5092	OtA5093-OtA5097
Csrp2	chr10:110920175-110939514	OtA5088-OtA5092	OtA5093-OtA5097
Cyba	chr8:122424770-122432940	OtA5088-OtA5092	OtA5093-OtA5097
Emp3	chr7:45918022-45921426	OtA5088-OtA5092	OtA5093-OtA5097
Map4k4	chr1:39900912-40026310	OtA5088-OtA5092	OtA5093-OtA5097
Nit2	chr16:57156664-57167332	OtA5088-OtA5092	OtA5093-OtA5097
Pds5a	chr5:65615259-65697856	OtA5088-OtA5092	OtA5093-OtA5097
Rcn2	chr9:56041844-56059083	OtA5088-OtA5092	OtA5093-OtA5097
Rfesd	chr13:76001534-76018712	OtA5088-OtA5092	OtA5093-OtA5097
Rnf39	chr17:36943050-36947986	OtA5088-OtA5092	OtA5093-OtA5097
Rxrb	chr17:34031811-34038403	OtA5088-OtA5092	OtA5093-OtA5097
Stk39	chr2:68210446-68471981	OtA5088-OtA5092	OtA5093-OtA5097
Tcea1	chr1:4857693-4897909	OtA5088-OtA5092	OtA5093-OtA5097
Trim10	chr17:36869573-36877833	OtA5088-OtA5092	OtA5093-OtA5097
Utp11l	chr4:124678763-124693554	OtA5088-OtA5092	OtA5093-OtA5097
Apeh	chr9:108085413-108094480	OtA5088-OtA5092	OtA5093-OtA5097
Arglu1	chr8:8666575-8690537	OtA5088-OtA5092	OtA5093-OtA5097
Atoh8	chr6:72206176-72235577	OtA5088-OtA5092	OtA5093-OtA5097
C1galt1	chr6:7845223-7872042	OtA5088-OtA5092	OtA5093-OtA5097
C1qtnf3	chr15:10952331-10980162	OtA5088-OtA5092	OtA5093-OtA5097
Slc44a2	chr9:21320718-21355028	OtA5088-OtA5092	OtA5093-OtA5097
Tmem120a	chr5:135735489-135744172	OtA5088-OtA5092	OtA5093-OtA5097
Zfp597	chr16:3861543-3872374	OtA5088-OtA5092	OtA5093-OtA5097
Rnf103	chr6:71493893-71510881	OtA5088-OtA5092	OtA5093-OtA5097
Gjb3	chr4:127325234-127330836	OtA5088-OtA5092	OtA5093-OtA5097
Sox13	chr1:133382299-133424212	OtA5088-OtA5092	OtA5093-OtA5097
Nfkb2	chr19:46304736-46327156	OtA5088-OtA5092	OtA5093-OtA5097
Cdadc1	chr14:59559387-59597959	OtA5088-OtA5092	OtA5093-OtA5097
Fbln1	chr15:85206007-85286294	OtA5088-OtA5092	OtA5093-OtA5097
Mcm2	chr6:88883473-88898780	OtA5088-OtA5092	OtA5093-OtA5097

Tbc1d8b	chrX:139684995-139754218	OtA5088-OtA5092	OtA5093-OtA5097
St3gal6	chr16:58470540-58523312	OtA5088-OtA5092	OtA5093-OtA5097
Dsel	chr1:111858701-111864918	OtA5088-OtA5092	OtA5093-OtA5097
Pdlim3	chr8:45885484-45919546	OtA5088-OtA5092	OtA5093-OtA5097
Ank3	chr10:69533707-70027436	OtA5088-OtA5092	OtA5093-OtA5097
Lgals3bp	chr11:118392751-118401931	OtA5088-OtA5092	OtA5093-OtA5097
Dpp8	chr9:65032457-65082651	OtA5088-OtA5092	OtA5093-OtA5097
Ubxn1	chr19:8871558-8875656	OtA5088-OtA5092	OtA5093-OtA5097
Fat4	chr3:38886939-39011983	OtA5088-OtA5092	OtA5093-OtA5097
Rasa2	chr9:96539299-96631503	OtA5088-OtA5092	OtA5093-OtA5097
Sec24a	chr11:51692262-51756834	OtA5088-OtA5092	OtA5093-OtA5097
Cln5	chr14:103070215-103077630	OtA5088-OtA5092	OtA5093-OtA5097
Elovl6	chr3:129532385-129638493	OtA5088-OtA5092	OtA5093-OtA5097
Ern1	chr11:106397619-106487796	OtA5088-OtA5092	OtA5093-OtA5097
Fam195b	chr11:120542887-120549727	OtA5088-OtA5092	OtA5093-OtA5097
Fuk	chr8:110882462-110902459	OtA5088-OtA5092	OtA5093-OtA5097
Gja1	chr10:56377299-56390419	OtA5088-OtA5092	OtA5093-OtA5097
Neurl1a	chr19:47178819-47259441	OtA5088-OtA5092	OtA5093-OtA5097
Pbrm1	chr14:31019137-31121592	OtA5088-OtA5092	OtA5093-OtA5097
Pnn	chr12:59066918-59074017	OtA5088-OtA5092	OtA5093-OtA5097
Zfp146	chr7:30161267-30169727	OtA5088-OtA5092	OtA5093-OtA5097
Arhgap17	chr7:123279148-123369915	OtA5088-OtA5092	OtA5093-OtA5097
Ampd2	chr3:108074061-108086627	OtA5088-OtA5092	OtA5093-OtA5097
Pnpo	chr11:96937815-96944019	OtA5088-OtA5092	OtA5093-OtA5097
Tbc1d25	chrX:8154471-8176181	OtA5088-OtA5092	OtA5093-OtA5097
Nrip1	chr16:76290861-76373049	OtA5088-OtA5092	OtA5093-OtA5097
Vps8	chr16:21423117-21644680	OtA5088-OtA5092	OtA5093-OtA5097
Cdk4	chr10:127063602-127067282	OtA5088-OtA5092	OtA5093-OtA5097
Wnk1	chr6:119923968-120038655	OtA5088-OtA5092	OtA5093-OtA5097
Phf20	chr2:156196646-156309953	OtA5088-OtA5092	OtA5093-OtA5097
Acvr2b	chr9:119402500-119433506	OtA5088-OtA5092	OtA5093-OtA5097
Map3k2	chr18:32163088-32236751	OtA5088-OtA5092	OtA5093-OtA5097
Coro2a	chr4:46536936-46601929	OtA5088-OtA5092	OtA5093-OtA5097
Ms4a10	chr19:10962294-10974670	OtA5088-OtA5092	OtA5093-OtA5097
Bbx	chr16:50191843-50432389	OtA5088-OtA5092	OtA5093-OtA5097
Cct3	chr3:88297115-88321767	OtA5088-OtA5092	OtA5093-OtA5097
Slc46a3	chr5:147878440-147894802	OtA5088-OtA5092	OtA5093-OtA5097
Cplx1	chr5:108518553-108550027	OtA5088-OtA5092	OtA5093-OtA5097
Rbl1	chr2:157145893-157204534	OtA5088-OtA5092	OtA5093-OtA5097
Thy1	chr9:44043383-44048579	OtA5088-OtA5092	OtA5093-OtA5097
Ube2e2	chr14:18573576-18893627	OtA5088-OtA5092	OtA5093-OtA5097
Aspg	chr12:112106682-112127573	OtA5088-OtA5092	OtA5093-OtA5097
Atp5c1	chr2:10056030-10080510	OtA5088-OtA5092	OtA5093-OtA5097
Dtwd1	chr2:126152140-126165277	OtA5088-OtA5092	OtA5093-OtA5097
Dusp18	chr11:3895239-3901296	OtA5088-OtA5092	OtA5093-OtA5097
Fam82a1	chr17:79614899-79682152	OtA5088-OtA5092	OtA5093-OtA5097
Mtfmt	chr9:65435781-65453054	OtA5088-OtA5092	OtA5093-OtA5097

Rbmx	chrX:57383347-57393036	OtA5088-OtA5092	OtA5093-OtA5097
Rnf135	chr11:80183871-80199753	OtA5088-OtA5092	OtA5093-OtA5097
Sirt1	chr10:63319004-63339035	OtA5088-OtA5092	OtA5093-OtA5097
Tnfaip8l1	chr17:56162490-56173955	OtA5088-OtA5092	OtA5093-OtA5097
Aplp1	chr7:30434979-30445582	OtA5088-OtA5092	OtA5093-OtA5097
Fignl2	chr15:101050193-101054399	OtA5088-OtA5092	OtA5093-OtA5097
Myo1b	chr1:51749765-51915974	OtA5088-OtA5092	OtA5093-OtA5097
Zfp74	chr7:29932790-29951893	OtA5088-OtA5092	OtA5093-OtA5097
Angpt2	chr8:18595172-18803188	OtA5088-OtA5092	OtA5093-OtA5097
Plg	chr17:12378608-12419384	OtA5088-OtA5092	OtA5093-OtA5097
Atad2b	chr12:4917352-5047410	OtA5088-OtA5092	OtA5093-OtA5097
Ibtk	chr9:85687359-85749334	OtA5088-OtA5092	OtA5093-OtA5097
Mrps21	chr3:95862651-95870619	OtA5088-OtA5092	OtA5093-OtA5097
Pcbp3	chr10:76761853-76961947	OtA5088-OtA5092	OtA5093-OtA5097
Senp6	chr9:80066902-80144780	OtA5088-OtA5092	OtA5093-OtA5097
Pde4dip	chr3:97689828-97888707	OtA5088-OtA5092	OtA5093-OtA5097
Dnpep	chr1:75308564-75317637	OtA5088-OtA5092	OtA5093-OtA5097
E2f2	chr4:136172393-136196056	OtA5088-OtA5092	OtA5093-OtA5097
Kdm2a	chr19:4316146-4397077	OtA5088-OtA5092	OtA5093-OtA5097
Adcyap1r1	chr6:55451979-55501455	OtA5088-OtA5092	OtA5093-OtA5097
Gucy1b3	chr3:82032003-82074711	OtA5088-OtA5092	OtA5093-OtA5097
Fam73b	chr2:30364232-30385519	OtA5088-OtA5092	OtA5093-OtA5097
Gnpda1	chr18:38327536-38338993	OtA5088-OtA5092	OtA5093-OtA5097
Nvl	chr1:181093422-181144157	OtA5088-OtA5092	OtA5093-OtA5097
Sowahd	chrX:37048844-37050418	OtA5088-OtA5092	OtA5093-OtA5097
Dhx32	chr7:133709332-133776803	OtA5088-OtA5092	OtA5093-OtA5097
Tmem87b	chr2:128818302-128854261	OtA5088-OtA5092	OtA5093-OtA5097
Adk	chr14:21052573-21448569	OtA5088-OtA5092	OtA5093-OtA5097
Lap3	chr5:45493373-45512691	OtA5088-OtA5092	OtA5093-OtA5097
Txnrd3	chr6:89643987-89675529	OtA5088-OtA5092	OtA5093-OtA5097
Wdr1	chr5:38526812-38561595	OtA5088-OtA5092	OtA5093-OtA5097
Snx10	chr6:51523902-51590670	OtA5088-OtA5092	OtA5093-OtA5097
Tra2b	chr16:22245740-22265929	OtA5088-OtA5092	OtA5093-OtA5097
Vegfb	chr19:6982471-6987651	OtA5088-OtA5092	OtA5093-OtA5097
Igf2bp2	chr16:22059008-22163299	OtA5088-OtA5092	OtA5093-OtA5097
Cmtm3	chr8:104340593-104347672	OtA5088-OtA5092	OtA5093-OtA5097
Pdlim2	chr14:70164217-70177672	OtA5088-OtA5092	OtA5093-OtA5097
Ptdss2	chr7:141131285-141156154	OtA5088-OtA5092	OtA5093-OtA5097
Ttyh2	chr11:114344525-114757886	OtA5088-OtA5092	OtA5093-OtA5097
Ddx3x	chrX:13281021-13293983	OtA5088-OtA5092	OtA5093-OtA5097
Fbxl20	chr11:98082553-98149616	OtA5088-OtA5092	OtA5093-OtA5097
Scmh1	chr4:120405280-120530199	OtA5088-OtA5092	OtA5093-OtA5097
Spns1	chr7:126370059-126377934	OtA5088-OtA5092	OtA5093-OtA5097
Stk35	chr2:129798368-129832285	OtA5088-OtA5092	OtA5093-OtA5097
Prom1	chr5:43993621-44101736	OtA5088-OtA5092	OtA5093-OtA5097
Hist1h2bc	chr13:23684198-23692480	OtA5088-OtA5092	OtA5093-OtA5097
Nop14	chr5:34638535-34660148	OtA5088-OtA5092	OtA5093-OtA5097

Tanc1	chr2:59612043-59846149	OtA5088-OtA5092	OtA5093-OtA5097
Apobec1	chr6:122577791-122602444	OtA5088-OtA5092	OtA5093-OtA5097
Hoxd4	chr2:74710043-74748271	OtA5088-OtA5092	OtA5093-OtA5097
Igf1r	chr7:67952256-68233667	OtA5088-OtA5092	OtA5093-OtA5097
Isg20	chr7:78913764-78920396	OtA5088-OtA5092	OtA5093-OtA5097
Krba1	chr6:48395585-48419855	OtA5088-OtA5092	OtA5093-OtA5097
Mapk1ip1l	chr14:47298313-47323091	OtA5088-OtA5092	OtA5093-OtA5097
Ptk7	chr17:46564450-46629504	OtA5088-OtA5092	OtA5093-OtA5097
Usp7	chr16:8688721-8738342	OtA5088-OtA5092	OtA5093-OtA5097
Zfpm1	chr8:122282140-122337247	OtA5088-OtA5092	OtA5093-OtA5097
Dbnl	chr11:5788482-5800980	OtA5088-OtA5092	OtA5093-OtA5097
D10Jhu81e	chr10:78162066-78169768	OtA5088-OtA5092	OtA5093-OtA5097
Myo9a	chr9:59751173-59928866	OtA5088-OtA5092	OtA5093-OtA5097
Gchfr	chr2:119167787-119172389	OtA5088-OtA5092	OtA5093-OtA5097
Ikzf4	chr10:128632414-128645993	OtA5088-OtA5092	OtA5093-OtA5097
Gfpt1	chr6:87042845-87092207	OtA5088-OtA5092	OtA5093-OtA5097
Dennd1a	chr2:37776248-38287384	OtA5088-OtA5092	OtA5093-OtA5097
Odc1	chr12:17544872-17551502	OtA5088-OtA5092	OtA5093-OtA5097
Plekhm1	chr11:103365091-103412664	OtA5088-OtA5092	OtA5093-OtA5097
Scaf8	chr17:3114971-3198859	OtA5088-OtA5092	OtA5093-OtA5097
Uqcrc2	chr7:120635188-120670343	OtA5088-OtA5092	OtA5093-OtA5097
Cnst	chr1:179546528-179627473	OtA5088-OtA5092	OtA5093-OtA5097
Cetn2	chrX:72913564-72918344	OtA5088-OtA5092	OtA5093-OtA5097
Fbxw7	chr3:84815576-84979198	OtA5088-OtA5092	OtA5093-OtA5097
Gmeb1	chr4:132221024-132261549	OtA5088-OtA5092	OtA5093-OtA5097
Htra1	chr7:130936202-130985658	OtA5088-OtA5092	OtA5093-OtA5097
Rpap3	chr15:97675104-97705822	OtA5088-OtA5092	OtA5093-OtA5097
Snca	chr6:60731572-60829855	OtA5088-OtA5092	OtA5093-OtA5097
Wdr45l	chr11:121327202-121354447	OtA5088-OtA5092	OtA5093-OtA5097
Arhgap39	chr15:76723984-76818170	OtA5088-OtA5092	OtA5093-OtA5097
Atp7a	chrX:106027275-106128160	OtA5088-OtA5092	OtA5093-OtA5097
Axl	chr7:25756499-25788733	OtA5088-OtA5092	OtA5093-OtA5097
Fbxo22	chr9:55208934-55283625	OtA5088-OtA5092	OtA5093-OtA5097
Gpr156	chr16:37916495-38007529	OtA5088-OtA5092	OtA5093-OtA5097
Prmt1	chr7:44976754-44986420	OtA5088-OtA5092	OtA5093-OtA5097
Sbf1	chr15:89288235-89315311	OtA5088-OtA5092	OtA5093-OtA5097
Socs7	chr11:97362550-97398542	OtA5088-OtA5092	OtA5093-OtA5097
Tek	chr4:94739299-94874939	OtA5088-OtA5092	OtA5093-OtA5097
Ttc14	chr3:33800194-33844310	OtA5088-OtA5092	OtA5093-OtA5097
Zbtb9	chr17:26973178-26976203	OtA5088-OtA5092	OtA5093-OtA5097
Aldh6a1	chr12:84409067-84450950	OtA5088-OtA5092	OtA5093-OtA5097
Ankrd6	chr4:32804034-32950826	OtA5088-OtA5092	OtA5093-OtA5097
Chuk	chr19:44073333-44107477	OtA5088-OtA5092	OtA5093-OtA5097
Cox16	chr12:81470703-81485110	OtA5088-OtA5092	OtA5093-OtA5097
Deptor	chr15:55099916-55254202	OtA5088-OtA5092	OtA5093-OtA5097
Eltd1	chr3:151437881-151545081	OtA5088-OtA5092	OtA5093-OtA5097
Fxr1	chr3:34020078-34081326	OtA5088-OtA5092	OtA5093-OtA5097

Jag2	chr12:112908589-112929495	OtA5088-OtA5092	OtA5093-OtA5097
Mtrr	chr13:68560779-68582121	OtA5088-OtA5092	OtA5093-OtA5097
Ncdn	chr4:126743749-126753429	OtA5088-OtA5092	OtA5093-OtA5097
Ndrg3	chr2:156927341-156992111	OtA5088-OtA5092	OtA5093-OtA5097
Ofd1	chrX:166390032-166440704	OtA5088-OtA5092	OtA5093-OtA5097
Rnf130	chr11:50025330-50104733	OtA5088-OtA5092	OtA5093-OtA5097
Rpf1	chr3:146506345-146521423	OtA5088-OtA5092	OtA5093-OtA5097
Sort1	chr3:108284131-108361511	OtA5088-OtA5092	OtA5093-OtA5097
Tcf12	chr9:71844251-72111819	OtA5088-OtA5092	OtA5093-OtA5097
Vsig10	chr5:117319265-117355006	OtA5088-OtA5092	OtA5093-OtA5097
Cyp2s1	chr7:25802475-25816530	OtA5088-OtA5092	OtA5093-OtA5097
Wdr85	chr2:24962421-24975098	OtA5088-OtA5092	OtA5093-OtA5097
Amz1	chr5:140724126-140753312	OtA5088-OtA5092	OtA5093-OtA5097
Cyp21a1	chr17:34801347-34804426	OtA5088-OtA5092	OtA5093-OtA5097
Elf2	chr3:51255664-51325987	OtA5088-OtA5092	OtA5093-OtA5097
Gdap2	chr3:100162517-100206981	OtA5088-OtA5092	OtA5093-OtA5097
Hpca	chr4:129105529-129121740	OtA5088-OtA5092	OtA5093-OtA5097
Reep3	chr10:67009188-67096988	OtA5088-OtA5092	OtA5093-OtA5097
Zfp948	chr17:21567045-21588682	OtA5088-OtA5092	OtA5093-OtA5097
Ddx47	chr6:135011611-135023776	OtA5088-OtA5092	OtA5093-OtA5097
Pccb	chr9:100982037-101034875	OtA5088-OtA5092	OtA5093-OtA5097
Ankrd13d	chr19:4270179-4283137	OtA5088-OtA5092	OtA5093-OtA5097
Crocc	chr4:141016636-141060545	OtA5088-OtA5092	OtA5093-OtA5097
Med25	chr7:44879385-44892366	OtA5088-OtA5092	OtA5093-OtA5097
Arhgap12	chr18:6024449-6136098	OtA5088-OtA5092	OtA5093-OtA5097
Bdh1	chr16:31422296-31458901	OtA5088-OtA5092	OtA5093-OtA5097
Cyb5	chr18:84851413-84879863	OtA5088-OtA5092	OtA5093-OtA5097
Dohh	chr10:81384427-81388352	OtA5088-OtA5092	OtA5093-OtA5097
Fbxl12	chr9:20637786-20646789	OtA5088-OtA5092	OtA5093-OtA5097
Fmnl3	chr15:99317222-99370466	OtA5088-OtA5092	OtA5093-OtA5097
Hlx	chr1:184727144-184732493	OtA5088-OtA5092	OtA5093-OtA5097
Klhl25	chr7:75848337-75874130	OtA5088-OtA5092	OtA5093-OtA5097
Rilpl2	chr5:124463264-124478235	OtA5088-OtA5092	OtA5093-OtA5097
Soat1	chr1:156428107-156474328	OtA5088-OtA5092	OtA5093-OtA5097
Tapbp	chr17:33919477-33929290	OtA5088-OtA5092	OtA5093-OtA5097
Txndc15	chr13:55714649-55726228	OtA5088-OtA5092	OtA5093-OtA5097
Zkscan14	chr5:145194945-145201882	OtA5088-OtA5092	OtA5093-OtA5097
Vrk3	chr7:44748628-44777514	OtA5088-OtA5092	OtA5093-OtA5097
Fbp2	chr13:62836884-62858370	OtA5088-OtA5092	OtA5093-OtA5097
Spats2	chr15:99126844-99212466	OtA5088-OtA5092	OtA5093-OtA5097
Actr10	chr12:70937856-70964717	OtA5088-OtA5092	OtA5093-OtA5097
Adamts7	chr9:90162977-90200102	OtA5088-OtA5092	OtA5093-OtA5097
Ezh1	chr11:101191114-101226450	OtA5088-OtA5092	OtA5093-OtA5097
Icosl	chr10:78069367-78079525	OtA5088-OtA5092	OtA5093-OtA5097
Ncor1	chr11:62281472-62457332	OtA5088-OtA5092	OtA5093-OtA5097
S100a8	chr3:90669070-90670034	OtA5088-OtA5092	OtA5093-OtA5097
Ttc13	chr8:124671326-124721975	OtA5088-OtA5092	OtA5093-OtA5097

Xrcc1	chr7:24547149-24573438	OtA5088-OtA5092	OtA5093-OtA5097
Dync2li1	chr17:84626498-84655564	OtA5088-OtA5092	OtA5093-OtA5097
Man1b1	chr2:25332742-25352213	OtA5088-OtA5092	OtA5093-OtA5097
Wdr55	chr18:36760238-36766214	OtA5088-OtA5092	OtA5093-OtA5097
Elk3	chr10:93247415-93311159	OtA5088-OtA5092	OtA5093-OtA5097
Pfas	chr11:68985700-69008460	OtA5088-OtA5092	OtA5093-OtA5097
Rpl7	chr1:16101295-16104433	OtA5088-OtA5092	OtA5093-OtA5097
Spast	chr17:74338986-74391113	OtA5088-OtA5092	OtA5093-OtA5097
Coasy	chr11:101082624-101086619	OtA5088-OtA5092	OtA5093-OtA5097
Cpt1a	chr19:3323300-3385733	OtA5088-OtA5092	OtA5093-OtA5097
Tbc1d22b	chr17:29549801-29606808	OtA5088-OtA5092	OtA5093-OtA5097
Coro1c	chr5:113842438-113908706	OtA5088-OtA5092	OtA5093-OtA5097
Rasl11b	chr5:74195325-74199477	OtA5088-OtA5092	OtA5093-OtA5097
Snrpg	chr6:86371539-86378902	OtA5088-OtA5092	OtA5093-OtA5097
Tacc3	chr5:33658146-33672197	OtA5088-OtA5092	OtA5093-OtA5097
Idh3a	chr9:54586510-54604662	OtA5088-OtA5092	OtA5093-OtA5097
Magi1	chr6:93675454-94283904	OtA5088-OtA5092	OtA5093-OtA5097
Man1c1	chr4:134561689-134704290	OtA5088-OtA5092	OtA5093-OtA5097
Pax8	chr2:24420559-24475566	OtA5088-OtA5092	OtA5093-OtA5097
Tmc6	chr11:117765984-117780683	OtA5088-OtA5092	OtA5093-OtA5097
Ccdc97	chr7:25711116-25719053	OtA5088-OtA5092	OtA5093-OtA5097
Dhx36	chr3:62468641-62506988	OtA5088-OtA5092	OtA5093-OtA5097
Dpcr1	chr17:35635754-35643695	OtA5088-OtA5092	OtA5093-OtA5097
Foxred1	chr9:35204220-35210925	OtA5088-OtA5092	OtA5093-OtA5097
Gucy1a3	chr3:82092426-82145877	OtA5088-OtA5092	OtA5093-OtA5097
Papd4	chr13:93147399-93192283	OtA5088-OtA5092	OtA5093-OtA5097
Ranbp6	chr19:29808107-29812974	OtA5088-OtA5092	OtA5093-OtA5097
Rec8	chr14:55618165-55625395	OtA5088-OtA5092	OtA5093-OtA5097
Sh3pxd2a	chr19:47260173-47464411	OtA5088-OtA5092	OtA5093-OtA5097
Spata20	chr11:94478903-94485310	OtA5088-OtA5092	OtA5093-OtA5097
Stra13	chr11:120710940-120713767	OtA5088-OtA5092	OtA5093-OtA5097
Upf1	chr8:70315774-70353273	OtA5088-OtA5092	OtA5093-OtA5097
Wdr62	chr7:30240137-30280421	OtA5088-OtA5092	OtA5093-OtA5097
Ankrd46	chr15:36477667-36496791	OtA5088-OtA5092	OtA5093-OtA5097
Acap3	chr4:155891874-155907251	OtA5088-OtA5092	OtA5093-OtA5097
Brf2	chr8:27123831-27128632	OtA5088-OtA5092	OtA5093-OtA5097
Cstb	chr10:78425669-78427622	OtA5088-OtA5092	OtA5093-OtA5097
Daam2	chr17:49456021-49564337	OtA5088-OtA5092	OtA5093-OtA5097
Hsd17b11	chr5:103989764-104021796	OtA5088-OtA5092	OtA5093-OtA5097
Lnp	chr2:74514836-74578948	OtA5088-OtA5092	OtA5093-OtA5097
Masp1	chr16:23451784-23520590	OtA5088-OtA5092	OtA5093-OtA5097
Npb	chr11:120608476-120609100	OtA5088-OtA5092	OtA5093-OtA5097
Polg	chr7:79392337-79466273	OtA5088-OtA5092	OtA5093-OtA5097
Scpep1	chr11:88905927-88955442	OtA5088-OtA5092	OtA5093-OtA5097
Sgsh	chr11:119314786-119355510	OtA5088-OtA5092	OtA5093-OtA5097
Slc3a2	chr19:8706881-8723369	OtA5088-OtA5092	OtA5093-OtA5097
Sp1	chr15:102406315-102436404	OtA5088-OtA5092	OtA5093-OtA5097

Tuba8	chr6:121210769-121226097	OtA5088-OtA5092	OtA5093-OtA5097
Ube2c	chr2:164769928-164772902	OtA5088-OtA5092	OtA5093-OtA5097
Gde1	chr7:118688557-118705738	OtA5088-OtA5092	OtA5093-OtA5097
Aen	chr7:78895926-78908833	OtA5088-OtA5092	OtA5093-OtA5097
Gcnt2	chr13:40859767-40960892	OtA5088-OtA5092	OtA5093-OtA5097
Aldh16a1	chr7:45141839-45160064	OtA5088-OtA5092	OtA5093-OtA5097
Doc2g	chr19:4003384-4007005	OtA5088-OtA5092	OtA5093-OtA5097
Erbb2ip	chr13:103818785-103920514	OtA5088-OtA5092	OtA5093-OtA5097
Hmgn2	chr4:133964738-133967991	OtA5088-OtA5092	OtA5093-OtA5097
Hvcn1	chr5:122209736-122264460	OtA5088-OtA5092	OtA5093-OtA5097
Igfbp7	chr5:77310483-77408045	OtA5088-OtA5092	OtA5093-OtA5097
Jmjd4	chr11:59450044-59458567	OtA5088-OtA5092	OtA5093-OtA5097
Mknk2	chr10:80665329-80671976	OtA5088-OtA5092	OtA5093-OtA5097
Ndfip1	chr18:38418974-38464406	OtA5088-OtA5092	OtA5093-OtA5097
Nudt4	chr10:95547006-95564167	OtA5088-OtA5092	OtA5093-OtA5097
Pdk1	chr2:71873271-71903858	OtA5088-OtA5092	OtA5093-OtA5097
Pon2	chr6:5264623-5298373	OtA5088-OtA5092	OtA5093-OtA5097
Rufy4	chr1:74125540-74148230	OtA5088-OtA5092	OtA5093-OtA5097
Shprh	chr10:11149429-11215273	OtA5088-OtA5092	OtA5093-OtA5097
St6galnac4	chr2:32587489-32620804	OtA5088-OtA5092	OtA5093-OtA5097
Tradd	chr8:105258574-105264594	OtA5088-OtA5092	OtA5093-OtA5097
Ube2k	chr5:65537260-65598989	OtA5088-OtA5092	OtA5093-OtA5097
Zbtb45	chr7:13005665-13009800	OtA5088-OtA5092	OtA5093-OtA5097
Foxj3	chr4:119539687-119629119	OtA5088-OtA5092	OtA5093-OtA5097
Mtch2	chr2:90847154-90866634	OtA5088-OtA5092	OtA5093-OtA5097
Gpr108	chr17:57234914-57247641	OtA5088-OtA5092	OtA5093-OtA5097
Abcc5	chr16:20331303-20426394	OtA5088-OtA5092	OtA5093-OtA5097
Asprv1	chr6:86628173-86629704	OtA5088-OtA5092	OtA5093-OtA5097
Cd276	chr9:58524299-58540940	OtA5088-OtA5092	OtA5093-OtA5097
Myo9b	chr8:71272713-71360712	OtA5088-OtA5092	OtA5093-OtA5097
Pppde2	chr15:81992522-82016140	OtA5088-OtA5092	OtA5093-OtA5097
Tirap	chr9:35184390-35200291	OtA5088-OtA5092	OtA5093-OtA5097
Ubr2	chr17:46928290-47010532	OtA5088-OtA5092	OtA5093-OtA5097
Akirin1	chr4:123735194-123750299	OtA5088-OtA5092	OtA5093-OtA5097
D0H4S114	chr18:33437018-33464029	OtA5088-OtA5092	OtA5093-OtA5097
Etfb	chr7:43444142-43457782	OtA5088-OtA5092	OtA5093-OtA5097
Htatsf1	chrX:57053569-57067182	OtA5088-OtA5092	OtA5093-OtA5097
Mrps5	chr2:127587425-127603986	OtA5088-OtA5092	OtA5093-OtA5097
Ndrg2	chr14:51905270-51913488	OtA5088-OtA5092	OtA5093-OtA5097
Nup153	chr13:46679901-46727849	OtA5088-OtA5092	OtA5093-OtA5097
Ppp1r14b	chr19:6975047-6977324	OtA5088-OtA5092	OtA5093-OtA5097
Slc28a3	chr13:58553307-58610877	OtA5088-OtA5092	OtA5093-OtA5097
Slc35b2	chr17:45564151-45567669	OtA5088-OtA5092	OtA5093-OtA5097
Trio	chr15:27730648-28025848	OtA5088-OtA5092	OtA5093-OtA5097
Usp36	chr11:118259652-118290244	OtA5088-OtA5092	OtA5093-OtA5097
Yes1	chr5:32611170-32687066	OtA5088-OtA5092	OtA5093-OtA5097
Cdkal1	chr13:29325305-29855588	OtA5088-OtA5092	OtA5093-OtA5097

Ier3	chr17:35821712-35822911	OtA5088-OtA5092	OtA5093-OtA5097
Rnf215	chr11:4135159-4160293	OtA5088-OtA5092	OtA5093-OtA5097
Celsr1	chr15:85898757-86033777	OtA5088-OtA5092	OtA5093-OtA5097
Eif6	chr2:155819836-155826925	OtA5088-OtA5092	OtA5093-OtA5097
Rdh13	chr7:4425664-4445657	OtA5088-OtA5092	OtA5093-OtA5097
Wdr60	chr12:116207049-116263025	OtA5088-OtA5092	OtA5093-OtA5097
Adck2	chr6:39573875-39588769	OtA5088-OtA5092	OtA5093-OtA5097
Ccdc126	chr6:49319352-49341581	OtA5088-OtA5092	OtA5093-OtA5097
Taok3	chr5:117120128-117275098	OtA5088-OtA5092	OtA5093-OtA5097
Dcaf12l1	chrX:44786566-44790197	OtA5088-OtA5092	OtA5093-OtA5097
Fam83h	chr15:76001091-76014336	OtA5088-OtA5092	OtA5093-OtA5097
Gnpat	chr8:124863032-124890057	OtA5088-OtA5092	OtA5093-OtA5097
Rnd2	chr11:101468337-101471306	OtA5088-OtA5092	OtA5093-OtA5097
Trp53inp1	chr4:11156440-11174377	OtA5088-OtA5092	OtA5093-OtA5097
Csk	chr9:57626645-57645180	OtA5088-OtA5092	OtA5093-OtA5097
Heatr7a	chr15:76380512-76477269	OtA5088-OtA5092	OtA5093-OtA5097
Sfxn1	chr13:54071844-54108345	OtA5088-OtA5092	OtA5093-OtA5097
Cd300lg	chr11:102041510-102055617	OtA5088-OtA5092	OtA5093-OtA5097
Eif2s3x	chrX:94188708-94212651	OtA5088-OtA5092	OtA5093-OtA5097
Gng12	chr6:66896396-67021361	OtA5088-OtA5092	OtA5093-OtA5097
Mtus1	chr8:40990911-41133726	OtA5088-OtA5092	OtA5093-OtA5097
Prap1	chr7:140093395-140097203	OtA5088-OtA5092	OtA5093-OtA5097
Daxx	chr17:33909444-33915590	OtA5088-OtA5092	OtA5093-OtA5097
Nfe2	chr15:103248217-103255416	OtA5088-OtA5092	OtA5093-OtA5097
Abhd4	chr14:54254128-54269169	OtA5088-OtA5092	OtA5093-OtA5097
Ikbkb	chr8:22659204-22706583	OtA5088-OtA5092	OtA5093-OtA5097
Mdfic	chr6:15720660-15802169	OtA5088-OtA5092	OtA5093-OtA5097
Mfsd5	chr15:102279455-102281744	OtA5088-OtA5092	OtA5093-OtA5097
Plscr1	chr9:92250193-92272561	OtA5088-OtA5092	OtA5093-OtA5097
Elac1	chr18:73735037-73754479	OtA5088-OtA5092	OtA5093-OtA5097
Smap1	chr1:23761925-23922289	OtA5088-OtA5092	OtA5093-OtA5097
Gpr27	chr6:99692678-99693818	OtA5088-OtA5092	OtA5093-OtA5097
Gda	chr19:21391306-21472661	OtA5088-OtA5092	OtA5093-OtA5097
Npm1	chr11:33152497-33163206	OtA5088-OtA5092	OtA5093-OtA5097
Serpinb6b	chr13:32965512-32979037	OtA5088-OtA5092	OtA5093-OtA5097
Sucla2	chr14:73552785-73596142	OtA5088-OtA5092	OtA5093-OtA5097
Ahcy	chr2:155059311-155074497	OtA5088-OtA5092	OtA5093-OtA5097
Apoo-ps	chrX:94367109-94417092	OtA5088-OtA5092	OtA5093-OtA5097
Bckdhb	chr9:83948780-84124240	OtA5088-OtA5092	OtA5093-OtA5097
Irf5	chr6:29526624-29537320	OtA5088-OtA5092	OtA5093-OtA5097
Kcne4	chr1:78816948-78820025	OtA5088-OtA5092	OtA5093-OtA5097
Lgals2	chr15:78842646-78855529	OtA5088-OtA5092	OtA5093-OtA5097
Nf2	chr11:4765844-4849544	OtA5088-OtA5092	OtA5093-OtA5097
Nsun4	chr4:116031769-116053876	OtA5088-OtA5092	OtA5093-OtA5097
Phf14	chr6:11925880-12081198	OtA5088-OtA5092	OtA5093-OtA5097
Uchl3	chr14:101653966-101696125	OtA5088-OtA5092	OtA5093-OtA5097
BC016423	chr13:3538074-3611108	OtA5088-OtA5092	OtA5093-OtA5097

Armc5	chr7:128234450-128245100	OtA5088-OtA5092	OtA5093-OtA5097
Tlr4	chr4:66827810-66842796	OtA5088-OtA5092	OtA5093-OtA5097
Cachd1	chr4:100776678-101003748	OtA5088-OtA5092	OtA5093-OtA5097
Ilk	chr7:105731729-105744338	OtA5088-OtA5092	OtA5093-OtA5097
Pamr1	chr2:102550020-102643040	OtA5088-OtA5092	OtA5093-OtA5097
Nkd1	chr8:88521343-88594887	OtA5088-OtA5092	OtA5093-OtA5097
Aldh9a1	chr1:167349990-167368530	OtA5088-OtA5092	OtA5093-OtA5097
Mcat	chr15:83546796-83555711	OtA5088-OtA5092	OtA5093-OtA5097
Plaur	chr7:24462499-24475873	OtA5088-OtA5092	OtA5093-OtA5097
Rap1a	chr3:105728281-105801336	OtA5088-OtA5092	OtA5093-OtA5097
Hectd3	chr4:116995347-117005277	OtA5088-OtA5092	OtA5093-OtA5097
Tmem214	chr5:30869646-30877467	OtA5088-OtA5092	OtA5093-OtA5097
Ly6a	chr15:74994876-74997888	OtA5088-OtA5092	OtA5093-OtA5097
Nubp1	chr16:10411937-10447350	OtA5088-OtA5092	OtA5093-OtA5097
Eef1g	chr19:8967040-8978180	OtA5088-OtA5092	OtA5093-OtA5097
Napsa	chr7:44572444-44586846	OtA5088-OtA5092	OtA5093-OtA5097
Acaa1a	chr9:119341293-119350295	OtA5088-OtA5092	OtA5093-OtA5097
Aimp2	chr5:143871861-143909839	OtA5088-OtA5092	OtA5093-OtA5097
Numa1	chr7:101969842-102014959	OtA5088-OtA5092	OtA5093-OtA5097
Sars	chr3:108424863-108445259	OtA5088-OtA5092	OtA5093-OtA5097
Tgfb1	chr7:25687001-25704996	OtA5088-OtA5092	OtA5093-OtA5097
Tln2	chr9:67217084-67559703	OtA5088-OtA5092	OtA5093-OtA5097
Agpat2	chr2:26593586-26604210	OtA5088-OtA5092	OtA5093-OtA5097
Ubxn6	chr17:56068252-56074989	OtA5088-OtA5092	OtA5093-OtA5097
Tlk2	chr11:105181529-105283956	OtA5088-OtA5092	OtA5093-OtA5097
Svil	chr18:5046588-5119293	OtA5088-OtA5092	OtA5093-OtA5097
Sphk1	chr11:116531910-116536675	OtA5088-OtA5092	OtA5093-OtA5097
Ambp	chr4:63143278-63154142	OtA5088-OtA5092	OtA5093-OtA5097
Rwdd1	chr10:33996554-34019616	OtA5088-OtA5092	OtA5093-OtA5097
Golga7b	chr19:42247577-42270348	OtA5088-OtA5092	OtA5093-OtA5097
Hnrnph1	chr11:50377718-50386528	OtA5088-OtA5092	OtA5093-OtA5097
7-Sep	chr9:25252438-25308571	OtA5088-OtA5092	OtA5093-OtA5097
Rsbn1	chr3:103914119-104007491	OtA5088-OtA5092	OtA5093-OtA5097
Arf6	chr12:69372149-69375980	OtA5088-OtA5092	OtA5093-OtA5097
Cpsf4	chr5:145167223-145182040	OtA5088-OtA5092	OtA5093-OtA5097
Avil	chr10:127000708-127020994	OtA5088-OtA5092	OtA5093-OtA5097
Cep57l1	chr10:41718839-41809868	OtA5088-OtA5092	OtA5093-OtA5097
Jak3	chr8:71676382-71690577	OtA5088-OtA5092	OtA5093-OtA5097
Klf11	chr12:24651370-24662782	OtA5088-OtA5092	OtA5093-OtA5097
Akt1	chr12:112653820-112674276	OtA5088-OtA5092	OtA5093-OtA5097
Fam134b	chr15:25843263-25973687	OtA5088-OtA5092	OtA5093-OtA5097
Usp8	chr2:126707327-126759314	OtA5088-OtA5092	OtA5093-OtA5097
Anp32a	chr9:62341342-62378805	OtA5088-OtA5092	OtA5093-OtA5097
Psmd1	chr1:86064618-86139295	OtA5088-OtA5092	OtA5093-OtA5097
Abcb1b	chr5:8798146-8866314	OtA5088-OtA5092	OtA5093-OtA5097
Adamts5	chr16:85858156-85901125	OtA5088-OtA5092	OtA5093-OtA5097
Bak1	chr17:27018034-27036378	OtA5088-OtA5092	OtA5093-OtA5097

Clcn7	chr17:25133393-25162099	OtA5088-OtA5092	OtA5093-OtA5097
Eif2ak2	chr17:78852549-78882572	OtA5088-OtA5092	OtA5093-OtA5097
Eps8l2	chr7:141339001-141365110	OtA5088-OtA5092	OtA5093-OtA5097
Gbp2	chr3:142620662-142638008	OtA5088-OtA5092	OtA5093-OtA5097
Gfm1	chr3:67430114-67475068	OtA5088-OtA5092	OtA5093-OtA5097
Ggnbp2	chr11:84832360-84870738	OtA5088-OtA5092	OtA5093-OtA5097
Gk5	chr9:96119428-96182953	OtA5088-OtA5092	OtA5093-OtA5097
H2-DMa	chr17:34135670-34138535	OtA5088-OtA5092	OtA5093-OtA5097
Macrod1	chr19:7056767-7198062	OtA5088-OtA5092	OtA5093-OtA5097
Mxd1	chr6:86647044-86669159	OtA5088-OtA5092	OtA5093-OtA5097
Ncbp2	chr16:31948545-31958472	OtA5088-OtA5092	OtA5093-OtA5097
Ubl3	chr5:148504630-148552788	OtA5088-OtA5092	OtA5093-OtA5097
Xlr4a	chrX:73074344-73082507	OtA5088-OtA5092	OtA5093-OtA5097
Bivm	chr1:44119967-44144771	OtA5088-OtA5092	OtA5093-OtA5097
Diras2	chr13:52504374-52530836	OtA5088-OtA5092	OtA5093-OtA5097
Vcp	chr4:42979963-43000507	OtA5088-OtA5092	OtA5093-OtA5097
Pcbd1	chr10:61089342-61094329	OtA5088-OtA5092	OtA5093-OtA5097
Phf20l1	chr15:66577571-66645255	OtA5088-OtA5092	OtA5093-OtA5097
Rpl23	chr11:97777525-97782439	OtA5088-OtA5092	OtA5093-OtA5097
Cd9	chr6:125460265-125494755	OtA5088-OtA5092	OtA5093-OtA5097
Peli2	chr14:48120868-48260883	OtA5088-OtA5092	OtA5093-OtA5097
Serinc3	chr2:163623272-163645800	OtA5088-OtA5092	OtA5093-OtA5097
Dock4	chr12:40446052-40846488	OtA5088-OtA5092	OtA5093-OtA5097
Gjc1	chr11:102799477-102819686	OtA5088-OtA5092	OtA5093-OtA5097
Gpm6b	chrX:166238942-166389033	OtA5088-OtA5092	OtA5093-OtA5097
Mpped2	chr2:106693458-106868360	OtA5088-OtA5092	OtA5093-OtA5097
Zc3hav1l	chr6:38287393-38299259	OtA5088-OtA5092	OtA5093-OtA5097
Atp6v1a	chr16:44085403-44139019	OtA5088-OtA5092	OtA5093-OtA5097
Rnf217	chr10:31501886-31609725	OtA5088-OtA5092	OtA5093-OtA5097
Dhx9	chr1:153455757-153487660	OtA5088-OtA5092	OtA5093-OtA5097
Gprin3	chr6:59352460-59426290	OtA5088-OtA5092	OtA5093-OtA5097
Gpr50	chrX:71663766-71668089	OtA5088-OtA5092	OtA5093-OtA5097
Taf1c	chr8:119575234-119605240	OtA5088-OtA5092	OtA5093-OtA5097
Dirc2	chr16:35694902-35769356	OtA5088-OtA5092	OtA5093-OtA5097
Trim45	chr3:100922492-100936925	OtA5088-OtA5092	OtA5093-OtA5097
Adnp2	chr18:80127134-80151482	OtA5088-OtA5092	OtA5093-OtA5097
Arfip2	chr7:105634200-105640416	OtA5088-OtA5092	OtA5093-OtA5097
Atp1a1	chr3:101576218-101604707	OtA5088-OtA5092	OtA5093-OtA5097
Atp6v0b	chr4:117884329-117887329	OtA5088-OtA5092	OtA5093-OtA5097
Card14	chr11:119314786-119355510	OtA5088-OtA5092	OtA5093-OtA5097
Cnnm2	chr19:46761608-46878580	OtA5088-OtA5092	OtA5093-OtA5097
Cyth1	chr11:118164165-118248592	OtA5088-OtA5092	OtA5093-OtA5097
Dhps	chr8:85071756-85080746	OtA5088-OtA5092	OtA5093-OtA5097
Dnmbp	chr19:43847966-43912392	OtA5088-OtA5092	OtA5093-OtA5097
Fbxw9	chr8:85060118-85067120	OtA5088-OtA5092	OtA5093-OtA5097
Flna	chrX:74223460-74246534	OtA5088-OtA5092	OtA5093-OtA5097
Gpx3	chr11:54902853-54910382	OtA5088-OtA5092	OtA5093-OtA5097

Mpzl2	chr9:45042343-45054043	OtA5088-OtA5092	OtA5093-OtA5097
Neurl1b	chr17:26414964-26446342	OtA5088-OtA5092	OtA5093-OtA5097
Nhlrc2	chr19:56548260-56598846	OtA5088-OtA5092	OtA5093-OtA5097
Osr2	chr15:35296111-35303305	OtA5088-OtA5092	OtA5093-OtA5097
Paip2	chr18:35598666-35627227	OtA5088-OtA5092	OtA5093-OtA5097
Pex14	chr4:148960534-149099812	OtA5088-OtA5092	OtA5093-OtA5097
Pfdn2	chr1:171345698-171358170	OtA5088-OtA5092	OtA5093-OtA5097
Ptpre	chr7:135537823-135686294	OtA5088-OtA5092	OtA5093-OtA5097
Rapgef6	chr11:54522844-54699286	OtA5088-OtA5092	OtA5093-OtA5097
Rnf145	chr11:44518963-44565520	OtA5088-OtA5092	OtA5093-OtA5097
Sfi1	chr11:3124020-3193463	OtA5088-OtA5092	OtA5093-OtA5097
Shkbp1	chr7:27342132-27356008	OtA5088-OtA5092	OtA5093-OtA5097
Tnfrsf11b	chr15:54250618-54278484	OtA5088-OtA5092	OtA5093-OtA5097
Tnnt2	chr1:135836385-135852261	OtA5088-OtA5092	OtA5093-OtA5097
Trim28	chr7:13024151-13031032	OtA5088-OtA5092	OtA5093-OtA5097
Zcchc7	chr4:44756558-44932214	OtA5088-OtA5092	OtA5093-OtA5097
Ednra	chr8:77663028-77724452	OtA5088-OtA5092	OtA5093-OtA5097
Samd5	chr10:9627258-9675208	OtA5088-OtA5092	OtA5093-OtA5097
Acsl4	chrX:142317992-142390535	OtA5088-OtA5092	OtA5093-OtA5097
Agtrap	chr4:148077060-148088064	OtA5088-OtA5092	OtA5093-OtA5097
Akr1b7	chr6:34412361-34423137	OtA5088-OtA5092	OtA5093-OtA5097
Bop1	chr15:76380512-76477269	OtA5088-OtA5092	OtA5093-OtA5097
Dcaf10	chr4:45342100-45379722	OtA5088-OtA5092	OtA5093-OtA5097
Dusp16	chr6:134715472-134792628	OtA5088-OtA5092	OtA5093-OtA5097
Far1	chr7:113513861-113570458	OtA5088-OtA5092	OtA5093-OtA5097
Gstm1	chr3:108012249-108017973	OtA5088-OtA5092	OtA5093-OtA5097
Isca1	chr13:59755414-59769789	OtA5088-OtA5092	OtA5093-OtA5097
Rapgef2	chr3:79062528-79145875	OtA5088-OtA5092	OtA5093-OtA5097
Tsc22d2	chr3:58415688-58466787	OtA5088-OtA5092	OtA5093-OtA5097
Tshz3	chr7:36698117-36773457	OtA5088-OtA5092	OtA5093-OtA5097
Wipi2	chr5:142629583-142669370	OtA5088-OtA5092	OtA5093-OtA5097
Zfp827	chr8:79028436-79193766	OtA5088-OtA5092	OtA5093-OtA5097
Ly6f	chr15:75268420-75272234	OtA5088-OtA5092	OtA5093-OtA5097
Cxx1c	chrX:53607921-53609134	OtA5088-OtA5092	OtA5093-OtA5097
Mrps18a	chr17:46111003-46128908	OtA5088-OtA5092	OtA5093-OtA5097
Ncaph2	chr15:89355721-89377037	OtA5088-OtA5092	OtA5093-OtA5097
Ncoa4	chr14:32159891-32179363	OtA5088-OtA5092	OtA5093-OtA5097
Nol10	chr12:17348492-17430095	OtA5088-OtA5092	OtA5093-OtA5097
Pa2g4	chr10:128557765-128565934	OtA5088-OtA5092	OtA5093-OtA5097
Prkch	chr12:73585040-73778184	OtA5088-OtA5092	OtA5093-OtA5097
Stim1	chr7:102267823-102436855	OtA5088-OtA5092	OtA5093-OtA5097
Tceanc	chrX:166499814-166510478	OtA5088-OtA5092	OtA5093-OtA5097
Tcta	chr9:108302957-108305951	OtA5088-OtA5092	OtA5093-OtA5097
Zfp655	chr5:145231714-145247306	OtA5088-OtA5092	OtA5093-OtA5097
Cpeb1	chr7:81347025-81454758	OtA5088-OtA5092	OtA5093-OtA5097
Hmgcs2	chr3:98280430-98310738	OtA5088-OtA5092	OtA5093-OtA5097
Wbp11	chr6:136813653-136828216	OtA5088-OtA5092	OtA5093-OtA5097

Ndufa8	chr2:36036333-36049292	OtA5088-OtA5092	OtA5093-OtA5097
Asb8	chr15:98134639-98165578	OtA5088-OtA5092	OtA5093-OtA5097
Dars	chr1:128363706-128417416	OtA5088-OtA5092	OtA5093-OtA5097
Dhcr24	chr4:106561037-106589113	OtA5088-OtA5092	OtA5093-OtA5097
Gm17066	chr14:105106727-105177327	OtA5088-OtA5092	OtA5093-OtA5097
Lnx1	chr5:74535481-74702903	OtA5088-OtA5092	OtA5093-OtA5097
Acta2	chr19:34241090-34255336	OtA5088-OtA5092	OtA5093-OtA5097
Ipo9	chr1:135382314-135430491	OtA5088-OtA5092	OtA5093-OtA5097
Arid4a	chr12:71015966-71099351	OtA5088-OtA5092	OtA5093-OtA5097
Zfp574	chr7:25077204-25083492	OtA5088-OtA5092	OtA5093-OtA5097
B230208H17Rik	chr2:25583017-25608446	OtA5088-OtA5092	OtA5093-OtA5097
Dnajc12	chr10:63382442-63408840	OtA5088-OtA5092	OtA5093-OtA5097
Edem2	chr2:155701672-155729475	OtA5088-OtA5092	OtA5093-OtA5097
Eif3g	chr9:20894348-20898590	OtA5088-OtA5092	OtA5093-OtA5097
Rnf128	chrX:139563339-139673145	OtA5088-OtA5092	OtA5093-OtA5097
Wtap	chr17:12966798-12992539	OtA5088-OtA5092	OtA5093-OtA5097
Dhx16	chr17:35879777-35892668	OtA5088-OtA5092	OtA5093-OtA5097
Gbp3	chr3:142560051-142573212	OtA5088-OtA5092	OtA5093-OtA5097
Mta2	chr19:8941919-8952300	OtA5088-OtA5092	OtA5093-OtA5097
Slc20a2	chr8:22462613-22569616	OtA5088-OtA5092	OtA5093-OtA5097
Ssna1	chr2:25271038-25272418	OtA5088-OtA5092	OtA5093-OtA5097
Zfp764	chr7:127403667-127406822	OtA5088-OtA5092	OtA5093-OtA5097
Chrdl2	chr7:100006403-100034726	OtA5088-OtA5092	OtA5093-OtA5097
Srm	chr4:148591512-148594619	OtA5088-OtA5092	OtA5093-OtA5097
Ttc28	chr5:110879802-111289779	OtA5088-OtA5092	OtA5093-OtA5097
Msl2	chr9:101100569-101104799	OtA5088-OtA5092	OtA5093-OtA5097
Rpa1	chr11:75300258-75348383	OtA5088-OtA5092	OtA5093-OtA5097
Cmpk2	chr12:26469214-26479837	OtA5088-OtA5092	OtA5093-OtA5097
Hacl1	chr14:31607225-31640965	OtA5088-OtA5092	OtA5093-OtA5097
Casp3	chr8:46617448-46638708	OtA5088-OtA5092	OtA5093-OtA5097
Mark1	chr1:184896423-184999549	OtA5088-OtA5092	OtA5093-OtA5097
Psmd14	chr2:61711693-61800376	OtA5088-OtA5092	OtA5093-OtA5097
Picalm	chr7:90130231-90209447	OtA5088-OtA5092	OtA5093-OtA5097
Rbck1	chr2:152316333-152332639	OtA5088-OtA5092	OtA5093-OtA5097
Tnfrsf9	chr4:150920154-150946102	OtA5088-OtA5092	OtA5093-OtA5097
Tpp1	chr7:105744846-105752207	OtA5088-OtA5092	OtA5093-OtA5097
Zfp654	chr16:64780346-64786321	OtA5088-OtA5092	OtA5093-OtA5097
N4bp2l2	chr5:150635972-150665612	OtA5088-OtA5092	OtA5093-OtA5097
Shisa4	chr1:135371455-135375063	OtA5088-OtA5092	OtA5093-OtA5097
Ppic	chr18:53406340-53418007	OtA5088-OtA5092	OtA5093-OtA5097
Gsk3b	chr16:38085063-38246079	OtA5088-OtA5092	OtA5093-OtA5097
Carhsp1	chr16:8658586-8672153	OtA5088-OtA5092	OtA5093-OtA5097
Kcnj12	chr11:61065803-61071131	OtA5088-OtA5092	OtA5093-OtA5097
Zbp1	chr2:173206611-173218922	OtA5088-OtA5092	OtA5093-OtA5097
Acsbg1	chr9:54604996-54661885	OtA5088-OtA5092	OtA5093-OtA5097
Ak8	chr2:28700160-28813165	OtA5088-OtA5092	OtA5093-OtA5097
Adrb1	chr19:56722371-56724862	OtA5088-OtA5092	OtA5093-OtA5097

Aifm3	chr16:17489690-17507482	OtA5088-OtA5092	OtA5093-OtA5097
Anxa2	chr9:69453682-69491785	OtA5088-OtA5092	OtA5093-OtA5097
Bex4	chrX:136139044-136140437	OtA5088-OtA5092	OtA5093-OtA5097
Rnaseh1	chr12:28649601-28659591	OtA5088-OtA5092	OtA5093-OtA5097
Scarb2	chr5:92443872-92505608	OtA5088-OtA5092	OtA5093-OtA5097
Tagap	chr17:7925999-7934897	OtA5088-OtA5092	OtA5093-OtA5097
Trabd	chr15:89076063-89087075	OtA5088-OtA5092	OtA5093-OtA5097
Dyrk1a	chr16:94570205-94695519	OtA5088-OtA5092	OtA5093-OtA5097
Adar	chr3:89715021-89764632	OtA5088-OtA5092	OtA5093-OtA5097
H19	chr7:142575531-142578146	OtA5088-OtA5092	OtA5093-OtA5097
Hnrnpul2	chr19:8819400-8834142	OtA5088-OtA5092	OtA5093-OtA5097
Hsd11b2	chr8:105518745-105523988	OtA5088-OtA5092	OtA5093-OtA5097
Mob3c	chr4:115828091-115836183	OtA5088-OtA5092	OtA5093-OtA5097
Usp49	chr17:47630689-47684067	OtA5088-OtA5092	OtA5093-OtA5097
Dact3	chr7:16875316-16887301	OtA5088-OtA5092	OtA5093-OtA5097
Ndufb10	chr17:24722066-24724388	OtA5088-OtA5092	OtA5093-OtA5097
Acad8	chr9:26974138-26999549	OtA5088-OtA5092	OtA5093-OtA5097
B3galt4	chr17:33949911-33951488	OtA5088-OtA5092	OtA5093-OtA5097
Pofut1	chr2:153241531-153270249	OtA5088-OtA5092	OtA5093-OtA5097
Ppp1r37	chr7:19530966-19562398	OtA5088-OtA5092	OtA5093-OtA5097
Urgcp	chr11:5713416-5784710	OtA5088-OtA5092	OtA5093-OtA5097
Zbtb44	chr9:31030643-31075885	OtA5088-OtA5092	OtA5093-OtA5097
Zfp942	chr17:21926962-21962464	OtA5088-OtA5092	OtA5093-OtA5097
Cebpd	chr16:15887285-16146833	OtA5088-OtA5092	OtA5093-OtA5097
Ddx21	chr10:62580246-62602298	OtA5088-OtA5092	OtA5093-OtA5097
Kdsr	chr1:106720409-106759742	OtA5088-OtA5092	OtA5093-OtA5097
Ifih1	chr2:62595792-62646255	OtA5088-OtA5092	OtA5093-OtA5097
Smchd1	chr17:71344492-71475343	OtA5088-OtA5092	OtA5093-OtA5097
Znrf1	chr8:111536639-111626030	OtA5088-OtA5092	OtA5093-OtA5097
Creb3l3	chr10:81084332-81098872	OtA5088-OtA5092	OtA5093-OtA5097
Eif2c1	chr4:126435012-126468421	OtA5088-OtA5092	OtA5093-OtA5097
Tnf	chr17:35199390-35201996	OtA5088-OtA5092	OtA5093-OtA5097
Wdr6	chr9:108569891-108578670	OtA5088-OtA5092	OtA5093-OtA5097
E430025E21Rik	chr15:59331996-59374152	OtA5088-OtA5092	OtA5093-OtA5097
Errfi1	chr4:150855090-150868880	OtA5088-OtA5092	OtA5093-OtA5097
Sarnp	chr10:128821770-128877638	OtA5088-OtA5092	OtA5093-OtA5097
Lrrc32	chr7:98494221-98501830	OtA5088-OtA5092	OtA5093-OtA5097
Slc25a13	chr6:6041217-6217173	OtA5088-OtA5092	OtA5093-OtA5097
Fbxw17	chr13:50417876-50433769	OtA5088-OtA5092	OtA5093-OtA5097
Serpinb6c	chr13:33879815-33905708	OtA5088-OtA5092	OtA5093-OtA5097
Wac	chr18:7868857-7929027	OtA5088-OtA5092	OtA5093-OtA5097
Engase	chr11:118476959-118489198	OtA5088-OtA5092	OtA5093-OtA5097
Pgc	chr17:47726841-47734478	OtA5088-OtA5092	OtA5093-OtA5097
Psmc3	chr2:91054015-91059438	OtA5088-OtA5092	OtA5093-OtA5097
Sfmbt1	chr14:30714848-30822721	OtA5088-OtA5092	OtA5093-OtA5097
Tdrd3	chr14:87416582-87545508	OtA5088-OtA5092	OtA5093-OtA5097
C430048L16Rik	chr4:12089369-12096271	OtA5088-OtA5092	OtA5093-OtA5097

Rufy1	chr11:50389302-50431111	OtA5088-OtA5092	OtA5093-OtA5097
Mrpl14	chr17:45686371-45698495	OtA5088-OtA5092	OtA5093-OtA5097
Strada	chr11:106162973-106193603	OtA5088-OtA5092	OtA5093-OtA5097
Vegfa	chr17:46016992-46032377	OtA5088-OtA5092	OtA5093-OtA5097
Nt5dc3	chr10:86779004-86838389	OtA5088-OtA5092	OtA5093-OtA5097
Ap2a1	chr7:44896078-44929490	OtA5088-OtA5092	OtA5093-OtA5097
Col6a2	chr10:76595755-76623404	OtA5088-OtA5092	OtA5093-OtA5097
Exosc2	chr2:31670736-31681307	OtA5088-OtA5092	OtA5093-OtA5097
Fbxo11	chr17:87975049-88065285	OtA5088-OtA5092	OtA5093-OtA5097
Fhod1	chr8:105329159-105347970	OtA5088-OtA5092	OtA5093-OtA5097
Gltp	chr5:114669499-114690935	OtA5088-OtA5092	OtA5093-OtA5097
Greb1	chr12:16670614-16800886	OtA5088-OtA5092	OtA5093-OtA5097
Ier2	chr8:84661330-84662852	OtA5088-OtA5092	OtA5093-OtA5097
Lats1	chr10:7681208-7716461	OtA5088-OtA5092	OtA5093-OtA5097
Med27	chr2:29346835-29524788	OtA5088-OtA5092	OtA5093-OtA5097
Nek7	chr1:138484713-138619696	OtA5088-OtA5092	OtA5093-OtA5097
Nudt12	chr17:59001400-59013322	OtA5088-OtA5092	OtA5093-OtA5097
Rab5b	chr10:128677182-128696268	OtA5088-OtA5092	OtA5093-OtA5097
Smarcd1	chr15:99702286-99713995	OtA5088-OtA5092	OtA5093-OtA5097
Tmem194	chr10:127677064-127701047	OtA5088-OtA5092	OtA5093-OtA5097
Hspa1a	chr17:34969358-34972156	OtA5088-OtA5092	OtA5093-OtA5097
Urod	chr4:116990216-116994375	OtA5088-OtA5092	OtA5093-OtA5097
Asb6	chr2:30823097-30828300	OtA5088-OtA5092	OtA5093-OtA5097
Herc4	chr10:63243796-63317881	OtA5088-OtA5092	OtA5093-OtA5097
Mcrs1	chr15:99224975-99262041	OtA5088-OtA5092	OtA5093-OtA5097
Prcc	chr3:87858902-87885562	OtA5088-OtA5092	OtA5093-OtA5097
Rnf157	chr11:116336344-116413032	OtA5088-OtA5092	OtA5093-OtA5097
Slc12a4	chr8:105943589-105966115	OtA5088-OtA5092	OtA5093-OtA5097
Stk17b	chr1:53755511-53785215	OtA5088-OtA5092	OtA5093-OtA5097
Lrrc47	chr4:154011802-154026044	OtA5088-OtA5092	OtA5093-OtA5097
Atp11b	chr3:35754137-35856276	OtA5088-OtA5092	OtA5093-OtA5097
Atxn1l	chr8:109726450-109737739	OtA5088-OtA5092	OtA5093-OtA5097
Zfyve9	chr4:108639258-108781904	OtA5088-OtA5092	OtA5093-OtA5097
C130022K22Rik	chr6:91878052-91899848	OtA5088-OtA5092	OtA5093-OtA5097
Alkbh8	chr9:3335230-3385846	OtA5088-OtA5092	OtA5093-OtA5097
Atg9b	chr5:24364818-24392143	OtA5088-OtA5092	OtA5093-OtA5097
Cars	chr7:143557229-143600090	OtA5088-OtA5092	OtA5093-OtA5097
Phldb3	chr7:24611327-24629297	OtA5088-OtA5092	OtA5093-OtA5097
BC016579	chr16:45626847-45654118	OtA5088-OtA5092	OtA5093-OtA5097
Cfh	chr1:140085854-140183411	OtA5088-OtA5092	OtA5093-OtA5097
Dusp23	chr1:172630768-172632974	OtA5088-OtA5092	OtA5093-OtA5097
Gkn1	chr6:87345652-87350915	OtA5088-OtA5092	OtA5093-OtA5097
Ccr1l1	chr9:123976948-123978408	OtA5088-OtA5092	OtA5093-OtA5097
Plxnb2	chr15:89155548-89180788	OtA5088-OtA5092	OtA5093-OtA5097
Nod1	chr6:54923941-54972612	OtA5088-OtA5092	OtA5093-OtA5097
Tspan7	chrX:10485115-10596604	OtA5088-OtA5092	OtA5093-OtA5097
Agpat3	chr10:78271562-78351700	OtA5088-OtA5092	OtA5093-OtA5097

Akap7	chr10:25169089-25299163	OtA5088-OtA5092	OtA5093-OtA5097
H2-Aa	chr17:34282750-34287771	OtA5088-OtA5092	OtA5093-OtA5097
Tjp2	chr19:24094501-24225026	OtA5088-OtA5092	OtA5093-OtA5097
Tprn	chr2:25262597-25269886	OtA5088-OtA5092	OtA5093-OtA5097
Amotl1	chr9:14541966-14615000	OtA5088-OtA5092	OtA5093-OtA5097
Eif4g3	chr4:137993455-138207079	OtA5088-OtA5092	OtA5093-OtA5097
Gprc5a	chr6:135065661-135084708	OtA5088-OtA5092	OtA5093-OtA5097
Hnrnpm	chr17:33646232-33685458	OtA5088-OtA5092	OtA5093-OtA5097
Gapdh	chr6:125161851-125165583	OtA5088-OtA5092	OtA5093-OtA5097
Hps6	chr19:46003477-46006173	OtA5088-OtA5092	OtA5093-OtA5097
Mdc1	chr17:35841497-35859670	OtA5088-OtA5092	OtA5093-OtA5097
Psma2	chr13:14613241-14625673	OtA5088-OtA5092	OtA5093-OtA5097
Scap	chr9:110333292-110384946	OtA5088-OtA5092	OtA5093-OtA5097
6-Sep	chrX:36910833-36989695	OtA5088-OtA5092	OtA5093-OtA5097
Zdhhc3	chr9:123072309-123113205	OtA5088-OtA5092	OtA5093-OtA5097
Fam168a	chr7:100706701-100841630	OtA5088-OtA5092	OtA5093-OtA5097
Stk11ip	chr1:75521528-75537335	OtA5088-OtA5092	OtA5093-OtA5097
Tspan1	chr4:116161880-116167598	OtA5088-OtA5092	OtA5093-OtA5097
Mettl13	chr1:162533671-162548345	OtA5088-OtA5092	OtA5093-OtA5097
Ndor1	chr2:25244812-25255414	OtA5088-OtA5092	OtA5093-OtA5097
Sigirr	chr7:141091174-141100546	OtA5088-OtA5092	OtA5093-OtA5097
Hnf4a	chr2:163547187-163572907	OtA5088-OtA5092	OtA5093-OtA5097
Pold3	chr7:100082112-100121500	OtA5088-OtA5092	OtA5093-OtA5097
Trim27	chr13:21179930-21194723	OtA5088-OtA5092	OtA5093-OtA5097
Zmym2	chr14:56887794-56962579	OtA5088-OtA5092	OtA5093-OtA5097
Ola1	chr2:73092800-73214447	OtA5088-OtA5092	OtA5093-OtA5097
Rce1	chr19:4622550-4625617	OtA5088-OtA5092	OtA5093-OtA5097
Creg1	chr1:165763779-165775304	OtA5088-OtA5092	OtA5093-OtA5097
Eps15	chr4:109280274-109387816	OtA5088-OtA5092	OtA5093-OtA5097
Rcan3	chr4:135412308-135433805	OtA5088-OtA5092	OtA5093-OtA5097
Brwd1	chr16:95992091-96082428	OtA5088-OtA5092	OtA5093-OtA5097
Fdx1	chr9:51943306-51963533	OtA5088-OtA5092	OtA5093-OtA5097
Nmb	chr7:80902226-80905071	OtA5088-OtA5092	OtA5093-OtA5097
Retsat	chr6:72598627-72607488	OtA5088-OtA5092	OtA5093-OtA5097
Ssbp4	chr8:70597489-70608314	OtA5088-OtA5092	OtA5093-OtA5097
Arhgap6	chrX:168795098-169304435	OtA5088-OtA5092	OtA5093-OtA5097
Capza2	chr6:17637097-17666536	OtA5088-OtA5092	OtA5093-OtA5097
Efhd2	chr4:141858141-141874920	OtA5088-OtA5092	OtA5093-OtA5097
Atp6v0a2	chr5:124712207-124722146	OtA5088-OtA5092	OtA5093-OtA5097
C1qtnf5	chr9:44101769-44109187	OtA5088-OtA5092	OtA5093-OtA5097
Cct5	chr15:31590883-31601804	OtA5088-OtA5092	OtA5093-OtA5097
Abcc4	chr14:118482691-118706219	OtA5088-OtA5092	OtA5093-OtA5097
Coro1b	chr19:4148662-4154035	OtA5088-OtA5092	OtA5093-OtA5097
Dnajc10	chr2:80315465-80354055	OtA5088-OtA5092	OtA5093-OtA5097
Hnrnph2	chrX:134601285-134607054	OtA5088-OtA5092	OtA5093-OtA5097
Ikbkap	chr4:56749679-56809605	OtA5088-OtA5092	OtA5093-OtA5097
Rab23	chr1:33719895-33742564	OtA5088-OtA5092	OtA5093-OtA5097

Tnfaip2	chr12:111442660-111455018	OtA5088-OtA5092	OtA5093-OtA5097
Txndc16	chr14:45134447-45219394	OtA5088-OtA5092	OtA5093-OtA5097
Wdr77	chr3:105942862-105969760	OtA5088-OtA5092	OtA5093-OtA5097
Vps33b	chr7:80269654-80291579	OtA5088-OtA5092	OtA5093-OtA5097
Btf3l4	chr4:108814294-108833584	OtA5088-OtA5092	OtA5093-OtA5097
Scaper	chr9:55549882-55938115	OtA5088-OtA5092	OtA5093-OtA5097
Adra2b	chr2:127363285-127367221	OtA5088-OtA5092	OtA5093-OtA5097
Anxa7	chr14:20455260-20480133	OtA5088-OtA5092	OtA5093-OtA5097
Bin1	chr18:32359056-32435740	OtA5088-OtA5092	OtA5093-OtA5097
Bmp4	chr14:46379523-46390599	OtA5088-OtA5092	OtA5093-OtA5097
Bmyc	chr2:25706878-25707719	OtA5088-OtA5092	OtA5093-OtA5097
Crat	chr2:30392415-30415748	OtA5088-OtA5092	OtA5093-OtA5097
Fancl	chr11:26387083-26593920	OtA5088-OtA5092	OtA5093-OtA5097
Hdac3	chr18:37936970-37954988	OtA5088-OtA5092	OtA5093-OtA5097
Lrrc16a	chr13:24012483-24280790	OtA5088-OtA5092	OtA5093-OtA5097
Mphosph10	chr7:64376540-64392236	OtA5088-OtA5092	OtA5093-OtA5097
Naaa	chr5:92257659-92278181	OtA5088-OtA5092	OtA5093-OtA5097
Nrn1l	chr8:105893569-105895015	OtA5088-OtA5092	OtA5093-OtA5097
Ptpn11	chr5:121130532-121191397	OtA5088-OtA5092	OtA5093-OtA5097
2-Sep	chr1:93478992-93509732	OtA5088-OtA5092	OtA5093-OtA5097
Serpinb9b	chr13:33027413-33040558	OtA5088-OtA5092	OtA5093-OtA5097
Tmem97	chr11:78541816-78550735	OtA5088-OtA5092	OtA5093-OtA5097
Yif1b	chr7:29238322-29248467	OtA5088-OtA5092	OtA5093-OtA5097
Zfp423	chr8:87661809-87959595	OtA5088-OtA5092	OtA5093-OtA5097
Ptpn14	chr1:189728267-189876693	OtA5088-OtA5092	OtA5093-OtA5097
Rbm42	chr7:30640994-30650228	OtA5088-OtA5092	OtA5093-OtA5097
Sumf2	chr5:129846989-129879083	OtA5088-OtA5092	OtA5093-OtA5097
Plekhg4	chr8:105375380-105413487	OtA5088-OtA5092	OtA5093-OtA5097
Prpf40a	chr2:53138475-53191187	OtA5088-OtA5092	OtA5093-OtA5097
Zmpste24	chr4:121059237-121098243	OtA5088-OtA5092	OtA5093-OtA5097
Prl2b1	chr13:27383344-27390846	OtA5088-OtA5092	OtA5093-OtA5097
Actr1b	chr1:36699201-36709925	OtA5088-OtA5092	OtA5093-OtA5097
Col13a1	chr10:61838497-61979108	OtA5088-OtA5092	OtA5093-OtA5097
Rasd1	chr11:59948213-59964942	OtA5088-OtA5092	OtA5093-OtA5097
Slc18a2	chr19:59260877-59296012	OtA5088-OtA5092	OtA5093-OtA5097
Slc35f5	chr1:125561015-125595684	OtA5088-OtA5092	OtA5093-OtA5097
Fbxl17	chr17:63045951-63500580	OtA5088-OtA5092	OtA5093-OtA5097
Fanca	chr8:123254194-123318576	OtA5088-OtA5092	OtA5093-OtA5097
Mrps15	chr4:126046927-126055536	OtA5088-OtA5092	OtA5093-OtA5097
Fem1a	chr17:56256792-56263608	OtA5088-OtA5092	OtA5093-OtA5097
Tbx20	chr9:24720811-24774303	OtA5088-OtA5092	OtA5093-OtA5097
Thada	chr17:84190055-84466208	OtA5088-OtA5092	OtA5093-OtA5097
Paics	chr5:76913248-76967505	OtA5088-OtA5092	OtA5093-OtA5097
Dgcr2	chr16:17840355-17891728	OtA5088-OtA5092	OtA5093-OtA5097
Sec61a2	chr2:5870986-5895353	OtA5088-OtA5092	OtA5093-OtA5097
Bhlhb9	chrX:135885850-135891081	OtA5088-OtA5092	OtA5093-OtA5097
Rbm27	chr18:42275352-42341540	OtA5088-OtA5092	OtA5093-OtA5097

Fam114a1	chr5:64970074-65041901	OtA5088-OtA5092	OtA5093-OtA5097
Aldh1a2	chr9:71215788-71296243	OtA5088-OtA5092	OtA5093-OtA5097
Klf6	chr13:5861488-5870393	OtA5088-OtA5092	OtA5093-OtA5097
Leprel4	chr11:100408749-100414819	OtA5088-OtA5092	OtA5093-OtA5097
B230312C02Rik	chr2:180370857-180401802	OtA5088-OtA5092	OtA5093-OtA5097
Gpatch2	chr1:187215510-187351429	OtA5088-OtA5092	OtA5093-OtA5097
Matk	chr10:81257544-81262981	OtA5088-OtA5092	OtA5093-OtA5097
Med23	chr10:24869985-24913529	OtA5088-OtA5092	OtA5093-OtA5097
Mettl7b	chr10:128933812-128960988	OtA5088-OtA5092	OtA5093-OtA5097
Mpp1	chrX:75095853-75130949	OtA5088-OtA5092	OtA5093-OtA5097
Pola2	chr19:5940542-5964206	OtA5088-OtA5092	OtA5093-OtA5097
Rpl39	chrX:37082519-37085184	OtA5088-OtA5092	OtA5093-OtA5097
Zbtb3	chr19:8802495-8804854	OtA5088-OtA5092	OtA5093-OtA5097
Ctsa	chr2:164830729-164857708	OtA5088-OtA5092	OtA5093-OtA5097
D19Ertd386e	chr19:42518804-42592256	OtA5088-OtA5092	OtA5093-OtA5097
Ints3	chr3:90391379-90433636	OtA5088-OtA5092	OtA5093-OtA5097
Unc93b1	chr19:3935185-3949340	OtA5088-OtA5092	OtA5093-OtA5097
Cnpy2	chr10:128322458-128327187	OtA5088-OtA5092	OtA5093-OtA5097
Ikzf5	chr7:131388648-131410478	OtA5088-OtA5092	OtA5093-OtA5097
Klhl12	chr1:134455554-134490873	OtA5088-OtA5092	OtA5093-OtA5097
Mtmr9	chr14:63523609-63543953	OtA5088-OtA5092	OtA5093-OtA5097
Nln	chr13:104023438-104109614	OtA5088-OtA5092	OtA5093-OtA5097
Oaf	chr9:43221277-43239816	OtA5088-OtA5092	OtA5093-OtA5097
Rbms3	chr9:116572746-117629913	OtA5088-OtA5092	OtA5093-OtA5097
Smurf2	chr11:106820063-106920715	OtA5088-OtA5092	OtA5093-OtA5097
Timp2	chr11:118301060-118355411	OtA5088-OtA5092	OtA5093-OtA5097
Tinagl1	chr4:130165599-130175122	OtA5088-OtA5092	OtA5093-OtA5097
Tyw1	chr5:130255618-130341567	OtA5088-OtA5092	OtA5093-OtA5097
Zcchc2	chr1:105990405-106034079	OtA5088-OtA5092	OtA5093-OtA5097
Slc16a6	chr11:109440077-109573329	OtA5088-OtA5092	OtA5093-OtA5097
Abcc2	chr19:43782307-43838218	OtA5088-OtA5092	OtA5093-OtA5097
Klc4	chr17:46630630-46645144	OtA5088-OtA5092	OtA5093-OtA5097
Mrpl43	chr19:45005013-45006442	OtA5088-OtA5092	OtA5093-OtA5097
Bspry	chr4:62480066-62497298	OtA5088-OtA5092	OtA5093-OtA5097
Impa2	chr18:67289222-67318841	OtA5088-OtA5092	OtA5093-OtA5097
P4hb	chr11:120560303-120572936	OtA5088-OtA5092	OtA5093-OtA5097
Eif4a2	chr16:23107478-23127730	OtA5088-OtA5092	OtA5093-OtA5097
Gne	chr4:44034074-44084177	OtA5088-OtA5092	OtA5093-OtA5097
Gtf3c4	chr2:28822299-28840360	OtA5088-OtA5092	OtA5093-OtA5097
Nt5c2	chr19:46886830-47024346	OtA5088-OtA5092	OtA5093-OtA5097
Agl	chr3:116739998-116808166	OtA5088-OtA5092	OtA5093-OtA5097
Atp5s	chr12:69724961-69744658	OtA5088-OtA5092	OtA5093-OtA5097
Tor3a	chr1:156653616-156674339	OtA5088-OtA5092	OtA5093-OtA5097
Esr1	chr10:4612045-5003761	OtA5088-OtA5092	OtA5093-OtA5097
Rhox10	chrX:38066474-38071688	OtA5088-OtA5092	OtA5093-OtA5097
Zfp507	chr7:35772345-35838074	OtA5088-OtA5092	OtA5093-OtA5097
Atad1	chr19:32672562-32712298	OtA5088-OtA5092	OtA5093-OtA5097

Golga1	chr2:39016502-39065406	OtA5088-OtA5092	OtA5093-OtA5097
Spag9	chr11:93996090-94126082	OtA5088-OtA5092	OtA5093-OtA5097
Ano1	chr7:144588548-144738592	OtA5088-OtA5092	OtA5093-OtA5097
Calr	chr8:84842087-84846931	OtA5088-OtA5092	OtA5093-OtA5097
Dut	chr2:125247247-125259049	OtA5088-OtA5092	OtA5093-OtA5097
Mbnl2	chr14:120275668-120431698	OtA5088-OtA5092	OtA5093-OtA5097
Mtor	chr4:148448581-148557685	OtA5088-OtA5092	OtA5093-OtA5097
Rnf149	chr1:39551295-39577347	OtA5088-OtA5092	OtA5093-OtA5097
Uqcrfs1	chr13:30540311-30545316	OtA5088-OtA5092	OtA5093-OtA5097
Akr1b8	chr6:34354163-34368454	OtA5088-OtA5092	OtA5093-OtA5097
Rpa3	chr6:8255935-8259141	OtA5088-OtA5092	OtA5093-OtA5097
Zcrb1	chr15:93386112-93398290	OtA5088-OtA5092	OtA5093-OtA5097
Atrip	chr9:109059746-109074124	OtA5088-OtA5092	OtA5093-OtA5097
Layn	chr9:51056779-51077094	OtA5088-OtA5092	OtA5093-OtA5097
Vamp2	chr11:69088527-69092381	OtA5088-OtA5092	OtA5093-OtA5097
Cops7a	chr6:124958410-124965529	OtA5088-OtA5092	OtA5093-OtA5097
Ifi47	chr11:49037659-49135387	OtA5088-OtA5092	OtA5093-OtA5097
Oas1a	chr5:120896256-120907525	OtA5088-OtA5092	OtA5093-OtA5097
Wasf2	chr4:133130632-133198330	OtA5088-OtA5092	OtA5093-OtA5097
Procr	chr2:155751216-155755478	OtA5088-OtA5092	OtA5093-OtA5097
Tmem192	chr8:64947184-64969037	OtA5088-OtA5092	OtA5093-OtA5097
Larp7	chr3:127536713-127553349	OtA5088-OtA5092	OtA5093-OtA5097
Vps13d	chr4:144975136-145190592	OtA5088-OtA5092	OtA5093-OtA5097
Zdhhc5	chr2:84678401-84715164	OtA5088-OtA5092	OtA5093-OtA5097
Prpf40b	chr15:99295408-99317007	OtA5088-OtA5092	OtA5093-OtA5097
Ercc5	chr1:44147743-44181260	OtA5088-OtA5092	OtA5093-OtA5097
Papolg	chr11:23862645-23895270	OtA5088-OtA5092	OtA5093-OtA5097
Psmd13	chr7:140882393-140898642	OtA5088-OtA5092	OtA5093-OtA5097
Cpt1b	chr15:89416404-89429927	OtA5088-OtA5092	OtA5093-OtA5097
Phf8	chrX:151520671-151633857	OtA5088-OtA5092	OtA5093-OtA5097
Nampt	chr12:32820334-32853369	OtA5088-OtA5092	OtA5093-OtA5097
Sec23b	chr2:144556228-144590753	OtA5088-OtA5092	OtA5093-OtA5097
Uba6	chr5:86110729-86172743	OtA5088-OtA5092	OtA5093-OtA5097
Cd40	chr2:165055635-165071654	OtA5088-OtA5092	OtA5093-OtA5097
D7Ertd143e	chr7:3218783-3221015	OtA5088-OtA5092	OtA5093-OtA5097
Gm9781	chr2:122028582-122118386	OtA5088-OtA5092	OtA5093-OtA5097
Hk2	chr6:82725026-82774454	OtA5088-OtA5092	OtA5093-OtA5097
Hnf1b	chr11:83850868-83905815	OtA5088-OtA5092	OtA5093-OtA5097
Ifi44	chr3:151730922-151749959	OtA5088-OtA5092	OtA5093-OtA5097
Lama4	chr10:38965514-39110188	OtA5088-OtA5092	OtA5093-OtA5097
Riok2	chr17:17374331-17394899	OtA5088-OtA5092	OtA5093-OtA5097
Rps14	chr18:60774595-60778546	OtA5088-OtA5092	OtA5093-OtA5097
St6gal1	chr16:23224739-23360350	OtA5088-OtA5092	OtA5093-OtA5097
Pex12	chr11:83291698-83298977	OtA5088-OtA5092	OtA5093-OtA5097
Prmt7	chr8:106211053-106251694	OtA5088-OtA5092	OtA5093-OtA5097
Cdk14	chr5:4803384-5380251	OtA5088-OtA5092	OtA5093-OtA5097
Ndufv2	chr17:66078794-66101491	OtA5088-OtA5092	OtA5093-OtA5097

Srsf9	chr5:115327176-115333080	OtA5088-OtA5092	OtA5093-OtA5097
Dhrs7	chr12:72650352-72664828	OtA5088-OtA5092	OtA5093-OtA5097
Foxf1a	chr8:121084473-121086879	OtA5088-OtA5092	OtA5093-OtA5097
Fundc1	chrX:17556568-17572297	OtA5088-OtA5092	OtA5093-OtA5097
Rabep2	chr7:126428766-126463073	OtA5088-OtA5092	OtA5093-OtA5097
Ebag9	chr15:44619640-44641026	OtA5088-OtA5092	OtA5093-OtA5097
Setd8	chr5:124439929-124462307	OtA5088-OtA5092	OtA5093-OtA5097
Gm13375	chr2:20968873-20970348	OtA5088-OtA5092	OtA5093-OtA5097
Gpr39	chr1:125676995-125912214	OtA5088-OtA5092	OtA5093-OtA5097
Hspd1	chr1:55077833-55087932	OtA5088-OtA5092	OtA5093-OtA5097
Nmnat1	chr4:149468786-149485142	OtA5088-OtA5092	OtA5093-OtA5097
Ptgis	chr2:167203195-167240537	OtA5088-OtA5092	OtA5093-OtA5097
Asb13	chr13:3634075-3651779	OtA5088-OtA5092	OtA5093-OtA5097
Ehd2	chr7:15948986-15967535	OtA5088-OtA5092	OtA5093-OtA5097
Grlf1	chr7:16494472-16614993	OtA5088-OtA5092	OtA5093-OtA5097
Loxl2	chr14:69609475-69695834	OtA5088-OtA5092	OtA5093-OtA5097
Msh2	chr17:87672556-87723713	OtA5088-OtA5092	OtA5093-OtA5097
Ndufa10	chr1:92439718-92473758	OtA5088-OtA5092	OtA5093-OtA5097
Ndufv3	chr17:31520114-31531325	OtA5088-OtA5092	OtA5093-OtA5097
Nudc	chr4:133532541-133546027	OtA5088-OtA5092	OtA5093-OtA5097
Rnf146	chr10:29313165-29362442	OtA5088-OtA5092	OtA5093-OtA5097
Skap2	chr6:51859164-52012549	OtA5088-OtA5092	OtA5093-OtA5097
Slc27a2	chr2:126553023-126588243	OtA5088-OtA5092	OtA5093-OtA5097
Smc1a	chrX:152016427-152061973	OtA5088-OtA5092	OtA5093-OtA5097
Synj1	chr16:90936096-91044379	OtA5088-OtA5092	OtA5093-OtA5097
Rdh10	chr1:16105881-16132550	OtA5088-OtA5092	OtA5093-OtA5097
Golga7	chr8:23241325-23257080	OtA5088-OtA5092	OtA5093-OtA5097
Nedd4	chr9:72662346-72749848	OtA5088-OtA5092	OtA5093-OtA5097
Shc2	chr10:79617937-79637918	OtA5088-OtA5092	OtA5093-OtA5097
Zc3h15	chr2:83644577-83664616	OtA5088-OtA5092	OtA5093-OtA5097
Tbc1d4	chr14:101442359-101609191	OtA5088-OtA5092	OtA5093-OtA5097
Cbfa2t2	chr2:154436483-154539356	OtA5088-OtA5092	OtA5093-OtA5097
Cry1	chr10:85131699-85185054	OtA5088-OtA5092	OtA5093-OtA5097
Gle1	chr2:29935408-29959432	OtA5088-OtA5092	OtA5093-OtA5097
Ssu72	chr4:155704814-155733873	OtA5088-OtA5092	OtA5093-OtA5097
Tnc	chr4:63959784-64047015	OtA5088-OtA5092	OtA5093-OtA5097
Col18a1	chr10:77052178-77166530	OtA5088-OtA5092	OtA5093-OtA5097
Lancl1	chr1:67000516-67038872	OtA5088-OtA5092	OtA5093-OtA5097
Pdia4	chr6:47796140-47813512	OtA5088-OtA5092	OtA5093-OtA5097
Atox1	chr11:55446642-55461138	OtA5088-OtA5092	OtA5093-OtA5097
D19Bwg1357e	chr19:27388701-27429820	OtA5088-OtA5092	OtA5093-OtA5097
Hap1	chr11:100347326-100356141	OtA5088-OtA5092	OtA5093-OtA5097
Myl9	chr2:156775463-156781657	OtA5088-OtA5092	OtA5093-OtA5097
F2rl1	chr13:95511729-95525240	OtA5088-OtA5092	OtA5093-OtA5097
Hspa2	chr12:76404175-76406936	OtA5088-OtA5092	OtA5093-OtA5097
Atxn2l	chr7:126491707-126503302	OtA5088-OtA5092	OtA5093-OtA5097
Ckap5	chr2:91546321-91620665	OtA5088-OtA5092	OtA5093-OtA5097

Pcgf6	chr19:47033618-47050845	OtA5088-OtA5092	OtA5093-OtA5097
Ppp2r4	chr2:30416049-30447807	OtA5088-OtA5092	OtA5093-OtA5097
Hist1h4j	chr13:21735095-21735407	OtA5088-OtA5092	OtA5093-OtA5097
Hist2h2ac	chr3:96220454-96220844	OtA5088-OtA5092	OtA5093-OtA5097
Mirg	chr12:109734980-109749457	OtA5088-OtA5092	OtA5093-OtA5097
Smcr7l	chr15:80234079-80253371	OtA5088-OtA5092	OtA5093-OtA5097
Btg1	chr10:96617000-96622811	OtA5088-OtA5092	OtA5093-OtA5097
Mfap1a	chr2:121492680-121506656	OtA5088-OtA5092	OtA5093-OtA5097
Ankmy2	chr12:36157123-36197291	OtA5088-OtA5092	OtA5093-OtA5097
Synrg	chr11:83964427-84042079	OtA5088-OtA5092	OtA5093-OtA5097
Aptx	chr4:40682077-40703187	OtA5088-OtA5092	OtA5093-OtA5097
Bub3	chr7:131560390-131571898	OtA5088-OtA5092	OtA5093-OtA5097
Fam188a	chr2:12347263-12419460	OtA5088-OtA5092	OtA5093-OtA5097
Cabin1	chr10:75646109-75764357	OtA5088-OtA5092	OtA5093-OtA5097
Sox5	chr6:143828424-144209568	OtA5088-OtA5092	OtA5093-OtA5097
Wasl	chr6:24613809-24664995	OtA5088-OtA5092	OtA5093-OtA5097
Wdr70	chr15:7873054-8099209	OtA5088-OtA5092	OtA5093-OtA5097
Clk1	chr1:58411987-58424088	OtA5088-OtA5092	OtA5093-OtA5097
Irf9	chr14:55603984-55610030	OtA5088-OtA5092	OtA5093-OtA5097
Atg2b	chr12:105613539-105685241	OtA5088-OtA5092	OtA5093-OtA5097
BC017647	chr11:78094672-78157339	OtA5088-OtA5092	OtA5093-OtA5097
BC031181	chr18:75005899-75009933	OtA5088-OtA5092	OtA5093-OtA5097
Bex2	chrX:136066564-136068236	OtA5088-OtA5092	OtA5093-OtA5097
Brcc3	chrX:75416627-75455701	OtA5088-OtA5092	OtA5093-OtA5097
Clpp	chr17:56990263-56996371	OtA5088-OtA5092	OtA5093-OtA5097
Edem3	chr1:151755373-151822328	OtA5088-OtA5092	OtA5093-OtA5097
Erg	chr16:95360203-95530365	OtA5088-OtA5092	OtA5093-OtA5097
Fmod	chr1:134037514-134048277	OtA5088-OtA5092	OtA5093-OtA5097
Gmip	chr8:69808686-69821870	OtA5088-OtA5092	OtA5093-OtA5097
Ikbip	chr10:91083038-91102613	OtA5088-OtA5092	OtA5093-OtA5097
Pik3c2b	chr1:133046011-133108688	OtA5088-OtA5092	OtA5093-OtA5097
Plekha6	chr1:133246096-133303435	OtA5088-OtA5092	OtA5093-OtA5097
Ptpn18	chr1:34459745-34473779	OtA5088-OtA5092	OtA5093-OtA5097
Rcc1	chr4:132331918-132345750	OtA5088-OtA5092	OtA5093-OtA5097
Rcc2	chr4:140701472-140723220	OtA5088-OtA5092	OtA5093-OtA5097
Tmem109	chr19:10870660-10881743	OtA5088-OtA5092	OtA5093-OtA5097
Txn1	chr4:57943372-57956411	OtA5088-OtA5092	OtA5093-OtA5097
Letm2	chr8:25578489-25597487	OtA5088-OtA5092	OtA5093-OtA5097
Hmg20a	chr9:56418845-56496936	OtA5088-OtA5092	OtA5093-OtA5097
Mfsd11	chr11:116854014-116875637	OtA5088-OtA5092	OtA5093-OtA5097
Rnf115	chr3:96727610-96791155	OtA5088-OtA5092	OtA5093-OtA5097
Adrb2	chr18:62177815-62179959	OtA5088-OtA5092	OtA5093-OtA5097
Arl5a	chr2:52397950-52424874	OtA5088-OtA5092	OtA5093-OtA5097
Enho	chr4:41569793-41640302	OtA5088-OtA5092	OtA5093-OtA5097
Capn5	chr7:98121564-98184799	OtA5088-OtA5092	OtA5093-OtA5097
Cnn3	chr3:121426540-121458205	OtA5088-OtA5092	OtA5093-OtA5097
Ap3b2	chr7:81460398-81493925	OtA5088-OtA5092	OtA5093-OtA5097

Med12l	chr3:59006977-59344256	OtA5088-OtA5092	OtA5093-OtA5097
Pecr	chr1:72259172-72284314	OtA5088-OtA5092	OtA5093-OtA5097
Phactr4	chr4:132355924-132422446	OtA5088-OtA5092	OtA5093-OtA5097
Scfd2	chr5:74204815-74531749	OtA5088-OtA5092	OtA5093-OtA5097
Sox4	chr13:28950729-28953682	OtA5088-OtA5092	OtA5093-OtA5097
Strap	chr6:137735081-137751930	OtA5088-OtA5092	OtA5093-OtA5097
Trove2	chr1:143750790-143777051	OtA5088-OtA5092	OtA5093-OtA5097
Med17	chr9:15260350-15279867	OtA5088-OtA5092	OtA5093-OtA5097
Rab14	chr2:35180204-35201120	OtA5088-OtA5092	OtA5093-OtA5097
Gbp9	chr5:105078393-105110292	OtA5088-OtA5092	OtA5093-OtA5097
Icam4	chr9:21029372-21030531	OtA5088-OtA5092	OtA5093-OtA5097
Carkd	chr8:11497505-11513286	OtA5088-OtA5092	OtA5093-OtA5097
Htatip2	chr7:49759105-49773999	OtA5088-OtA5092	OtA5093-OtA5097
Kcnn4	chr7:24370262-24385212	OtA5088-OtA5092	OtA5093-OtA5097
Prosc	chr8:27042554-27056132	OtA5088-OtA5092	OtA5093-OtA5097
Socs5	chr17:87107678-87137588	OtA5088-OtA5092	OtA5093-OtA5097
Uggt1	chr1:36140027-36244302	OtA5088-OtA5092	OtA5093-OtA5097
Ttc1	chr11:43730005-43747973	OtA5088-OtA5092	OtA5093-OtA5097
Arl4c	chr1:88698225-88702191	OtA5088-OtA5092	OtA5093-OtA5097
Clrn3	chr7:135511455-135528654	OtA5088-OtA5092	OtA5093-OtA5097
Ildr2	chr1:166254138-166316832	OtA5088-OtA5092	OtA5093-OtA5097
Lzts1	chr8:69135502-69140953	OtA5088-OtA5092	OtA5093-OtA5097
Rnf121	chr7:102019871-102065132	OtA5088-OtA5092	OtA5093-OtA5097
Zbtb41	chr1:139422382-139453007	OtA5088-OtA5092	OtA5093-OtA5097
Ndufv1	chr19:4007504-4012725	OtA5088-OtA5092	OtA5093-OtA5097
Abhd10	chr16:45730396-45742906	OtA5088-OtA5092	OtA5093-OtA5097
Kdm4b	chr17:56326049-56402873	OtA5088-OtA5092	OtA5093-OtA5097
Zfp326	chr5:105876567-105915820	OtA5088-OtA5092	OtA5093-OtA5097
Ap1m1	chr8:72240131-72257379	OtA5088-OtA5092	OtA5093-OtA5097
B4galt7	chr13:55600110-55609954	OtA5088-OtA5092	OtA5093-OtA5097
BC024479	chr9:37489320-37524150	OtA5088-OtA5092	OtA5093-OtA5097
Galns	chr8:122578236-122611487	OtA5088-OtA5092	OtA5093-OtA5097
Mpdz	chr4:81278499-81442805	OtA5088-OtA5092	OtA5093-OtA5097
Ralgps1	chr2:33136972-33371478	OtA5088-OtA5092	OtA5093-OtA5097
Agtr1a	chr13:30336355-30382867	OtA5088-OtA5092	OtA5093-OtA5097
Alg12	chr15:88805242-88819318	OtA5088-OtA5092	OtA5093-OtA5097
Aurka	chr2:172356195-172370506	OtA5088-OtA5092	OtA5093-OtA5097
Flot1	chr17:35823356-35832787	OtA5088-OtA5092	OtA5093-OtA5097
Kpna2	chr11:106988628-106999525	OtA5088-OtA5092	OtA5093-OtA5097
Ube2l3	chr16:17152014-17201492	OtA5088-OtA5092	OtA5093-OtA5097
Acox3	chr5:35581226-35613801	OtA5088-OtA5092	OtA5093-OtA5097
Dapk3	chr10:81183006-81193197	OtA5088-OtA5092	OtA5093-OtA5097
Ogdh	chr11:6291596-6359094	OtA5088-OtA5092	OtA5093-OtA5097
Rab11fip4	chr11:79591211-79694012	OtA5088-OtA5092	OtA5093-OtA5097
Poli	chr18:70508679-70530321	OtA5088-OtA5092	OtA5093-OtA5097
Lmf2	chr15:89351003-89355659	OtA5088-OtA5092	OtA5093-OtA5097
Slc13a4	chr6:35267952-35308126	OtA5088-OtA5092	OtA5093-OtA5097

Trappc6a	chr7:19508728-19516145	OtA5088-OtA5092	OtA5093-OtA5097
Actr1a	chr19:46376813-46395735	OtA5088-OtA5092	OtA5093-OtA5097
Dnajc22	chr15:99099483-99104707	OtA5088-OtA5092	OtA5093-OtA5097
Itga9	chr9:118606708-118901003	OtA5088-OtA5092	OtA5093-OtA5097
Pef1	chr4:130107555-130128134	OtA5088-OtA5092	OtA5093-OtA5097
Slc12a2	chr18:57878677-57946821	OtA5088-OtA5092	OtA5093-OtA5097
Tmed9	chr13:55593134-55597694	OtA5088-OtA5092	OtA5093-OtA5097
Zfyve27	chr19:42170566-42194592	OtA5088-OtA5092	OtA5093-OtA5097
Bcl2	chr1:106538178-106714290	OtA5088-OtA5092	OtA5093-OtA5097
Ebp	chrX:8185330-8193512	OtA5088-OtA5092	OtA5093-OtA5097
Metrnl	chr11:121702426-121717389	OtA5088-OtA5092	OtA5093-OtA5097
Porcn	chrX:8193849-8206525	OtA5088-OtA5092	OtA5093-OtA5097
Fggy	chr4:95557506-95926939	OtA5088-OtA5092	OtA5093-OtA5097
Gata6	chr18:11052509-11085635	OtA5088-OtA5092	OtA5093-OtA5097
Kctd11	chr11:69878263-69880985	OtA5088-OtA5092	OtA5093-OtA5097
Padi1	chr4:140810992-140845778	OtA5088-OtA5092	OtA5093-OtA5097
Pdzd3	chr9:44247311-44251464	OtA5088-OtA5092	OtA5093-OtA5097
Tbc1d5	chr17:50733126-51613053	OtA5088-OtA5092	OtA5093-OtA5097
Bmp8b	chr4:123105164-123126091	OtA5088-OtA5092	OtA5093-OtA5097
Slc30a1	chr1:191894071-191913247	OtA5088-OtA5092	OtA5093-OtA5097
Gpc6	chr14:116925314-117979529	OtA5088-OtA5092	OtA5093-OtA5097
Ypel1	chr16:17070310-17111252	OtA5088-OtA5092	OtA5093-OtA5097
Rab3b	chr4:108879069-108972077	OtA5088-OtA5092	OtA5093-OtA5097
Slc12a9	chr5:137314557-137333582	OtA5088-OtA5092	OtA5093-OtA5097
Scrn1	chr6:54508815-54566382	OtA5088-OtA5092	OtA5093-OtA5097
Ndnl2	chr7:64756095-65156528	OtA5088-OtA5092	OtA5093-OtA5097
Arsa	chr15:89472475-89477424	OtA5088-OtA5092	OtA5093-OtA5097
Lonp1	chr17:56614297-56626903	OtA5088-OtA5092	OtA5093-OtA5097
Rictor	chr15:6708380-6800400	OtA5088-OtA5092	OtA5093-OtA5097
Zbed6	chr1:133619870-133661380	OtA5088-OtA5092	OtA5093-OtA5097
Ddx54	chr5:120613129-120634235	OtA5088-OtA5092	OtA5093-OtA5097
Mlh1	chr9:111228227-111271608	OtA5088-OtA5092	OtA5093-OtA5097
Cct7	chr6:85446784-85468477	OtA5088-OtA5092	OtA5093-OtA5097
Nol6	chr4:41114426-41124339	OtA5088-OtA5092	OtA5093-OtA5097
Tet3	chr6:83362373-83441678	OtA5088-OtA5092	OtA5093-OtA5097
Gpr137b-ps	chr13:12614064-12650395	OtA5088-OtA5092	OtA5093-OtA5097
Mapk8	chr14:33377898-33447158	OtA5088-OtA5092	OtA5093-OtA5097
Pgm3	chr9:86467153-86571842	OtA5088-OtA5092	OtA5093-OtA5097
Lgals4	chr7:28833793-28841703	OtA5088-OtA5092	OtA5093-OtA5097
Oas1c	chr5:120800198-120812514	OtA5088-OtA5092	OtA5093-OtA5097
Tdp2	chr13:24831658-24842153	OtA5088-OtA5092	OtA5093-OtA5097
Akap10	chr11:61871306-61930226	OtA5088-OtA5092	OtA5093-OtA5097
Sorbs2	chr8:45507787-45827906	OtA5088-OtA5092	OtA5093-OtA5097
Cbfb	chr8:105170673-105217988	OtA5088-OtA5092	OtA5093-OtA5097
Cbx3	chr6:51470615-51483704	OtA5088-OtA5092	OtA5093-OtA5097
Cdk18	chr1:132113546-132139685	OtA5088-OtA5092	OtA5093-OtA5097
Eml1	chr12:108410654-108702306	OtA5088-OtA5092	OtA5093-OtA5097

Fam100b	chr11:116434093-116439077	OtA5088-OtA5092	OtA5093-OtA5097
Fam40b	chr6:29917012-29959680	OtA5088-OtA5092	OtA5093-OtA5097
Habp4	chr13:64161865-64186537	OtA5088-OtA5092	OtA5093-OtA5097
Mcee	chr7:64392770-64412119	OtA5088-OtA5092	OtA5093-OtA5097
Mvp	chr7:126986859-127014594	OtA5088-OtA5092	OtA5093-OtA5097
Nub1	chr5:24685814-24710555	OtA5088-OtA5092	OtA5093-OtA5097
Nudt14	chr12:112934732-112942118	OtA5088-OtA5092	OtA5093-OtA5097
Popdc2	chr16:38362172-38378216	OtA5088-OtA5092	OtA5093-OtA5097
Prl8a8	chr13:27507071-27513213	OtA5088-OtA5092	OtA5093-OtA5097
Psmd3	chr11:98682553-98695978	OtA5088-OtA5092	OtA5093-OtA5097
Ptpn21	chr12:98676740-98737405	OtA5088-OtA5092	OtA5093-OtA5097
Slc15a3	chr19:10842543-10869779	OtA5088-OtA5092	OtA5093-OtA5097
Snx19	chr9:30427328-30466726	OtA5088-OtA5092	OtA5093-OtA5097
Tbl3	chr17:24700652-24707653	OtA5088-OtA5092	OtA5093-OtA5097
Zfyve1	chr12:83546940-83597147	OtA5088-OtA5092	OtA5093-OtA5097
Cnot1	chr8:95719450-95821728	OtA5088-OtA5092	OtA5093-OtA5097
Hax1	chr3:89995456-89998686	OtA5088-OtA5092	OtA5093-OtA5097
Iqce	chr5:140663504-140702378	OtA5088-OtA5092	OtA5093-OtA5097
D330028D13Rik	chr6:49367738-49389904	OtA5088-OtA5092	OtA5093-OtA5097
Gnai3	chr3:108107298-108146114	OtA5088-OtA5092	OtA5093-OtA5097
Ftsj3	chr11:106249143-106255802	OtA5088-OtA5092	OtA5093-OtA5097
Psme4	chr11:30771774-30880361	OtA5088-OtA5092	OtA5093-OtA5097
Rabgef1	chr5:130171818-130214337	OtA5088-OtA5092	OtA5093-OtA5097
Zfp213	chr17:23556766-23564226	OtA5088-OtA5092	OtA5093-OtA5097
Csf1r	chr18:61105571-61131139	OtA5088-OtA5092	OtA5093-OtA5097
Snord15b	chr7:99477896-99483709	OtA5088-OtA5092	OtA5093-OtA5097
Tspan17	chr13:54789404-54796775	OtA5088-OtA5092	OtA5093-OtA5097
Tfe3	chrX:7762660-7775202	OtA5088-OtA5092	OtA5093-OtA5097
Ddx50	chr10:62616022-62651198	OtA5088-OtA5092	OtA5093-OtA5097
Dph3	chr14:32080561-32085665	OtA5088-OtA5092	OtA5093-OtA5097
Mms19	chr19:41933471-41981136	OtA5088-OtA5092	OtA5093-OtA5097
Plcl2	chr17:50509546-50688494	OtA5088-OtA5092	OtA5093-OtA5097
Pomt2	chr12:87106865-87164723	OtA5088-OtA5092	OtA5093-OtA5097
Sync	chr4:129287620-129335370	OtA5088-OtA5092	OtA5093-OtA5097
Ccdc59	chr10:105841478-105847510	OtA5088-OtA5092	OtA5093-OtA5097
Impact	chr18:12972251-12992948	OtA5088-OtA5092	OtA5093-OtA5097
Irak2	chr6:113638466-113695027	OtA5088-OtA5092	OtA5093-OtA5097
Mpzl3	chr9:45055187-45074888	OtA5088-OtA5092	OtA5093-OtA5097
Pisd	chr5:32736313-32785626	OtA5088-OtA5092	OtA5093-OtA5097
Zfp513	chr5:31198980-31202303	OtA5088-OtA5092	OtA5093-OtA5097
Serpinb9f	chr13:33324076-33335366	OtA5088-OtA5092	OtA5093-OtA5097
Cryl1	chr14:57275033-57398483	OtA5088-OtA5092	OtA5093-OtA5097
P2ry2	chr7:100996571-101011991	OtA5088-OtA5092	OtA5093-OtA5097
Rai12	chr11:69968225-69990600	OtA5088-OtA5092	OtA5093-OtA5097
Cpped1	chr16:11803720-11930594	OtA5088-OtA5092	OtA5093-OtA5097
Mul1	chr4:138434671-138442265	OtA5088-OtA5092	OtA5093-OtA5097
Atg4d	chr9:21265298-21287969	OtA5088-OtA5092	OtA5093-OtA5097

Amfr	chr8:93971587-94012640	OtA5088-OtA5092	OtA5093-OtA5097
Manba	chr3:135485610-135571403	OtA5088-OtA5092	OtA5093-OtA5097
Mlxip	chr5:123394818-123457931	OtA5088-OtA5092	OtA5093-OtA5097
Dus3l	chr17:56764750-56770093	OtA5088-OtA5092	OtA5093-OtA5097
Gtf2ird1	chr5:134357660-134456716	OtA5088-OtA5092	OtA5093-OtA5097
Aktip	chr8:91070093-91135496	OtA5088-OtA5092	OtA5093-OtA5097
Ddx42	chr11:106216925-106249140	OtA5088-OtA5092	OtA5093-OtA5097
Fnip2	chr3:79345375-79567679	OtA5088-OtA5092	OtA5093-OtA5097
Gatad2a	chr8:69907068-69996379	OtA5088-OtA5092	OtA5093-OtA5097
Gnpda2	chr5:69575001-69592285	OtA5088-OtA5092	OtA5093-OtA5097
Hilpda	chr6:29272487-29275449	OtA5088-OtA5092	OtA5093-OtA5097
Pcgf1	chr6:83078389-83080855	OtA5088-OtA5092	OtA5093-OtA5097
Pid1	chr1:84036292-84284645	OtA5088-OtA5092	OtA5093-OtA5097
Slc27a4	chr2:29802679-29817522	OtA5088-OtA5092	OtA5093-OtA5097
Gtf2a2	chr9:70012549-70022847	OtA5088-OtA5092	OtA5093-OtA5097
Ubb	chr11:62551503-62553212	OtA5088-OtA5092	OtA5093-OtA5097
Efemp2	chr19:5474689-5481854	OtA5088-OtA5092	OtA5093-OtA5097
Rbm3	chrX:8138974-8147963	OtA5088-OtA5092	OtA5093-OtA5097
Zbtb6	chr2:37425499-37430919	OtA5088-OtA5092	OtA5093-OtA5097
Commd7	chr2:153616929-153632781	OtA5088-OtA5092	OtA5093-OtA5097
Hexb	chr13:97137936-97198357	OtA5088-OtA5092	OtA5093-OtA5097
Rab3il1	chr19:10018227-10035586	OtA5088-OtA5092	OtA5093-OtA5097
Lats2	chr14:57689661-57746123	OtA5088-OtA5092	OtA5093-OtA5097
Rpl8	chr15:76904070-76906318	OtA5088-OtA5092	OtA5093-OtA5097
Sall4	chr2:168748331-168767201	OtA5088-OtA5092	OtA5093-OtA5097
Il17rb	chr14:29996167-30008896	OtA5088-OtA5092	OtA5093-OtA5097
Hist1h3b	chr13:23752379-23752790	OtA5088-OtA5092	OtA5093-OtA5097
Cant1	chr11:118406288-118419052	OtA5088-OtA5092	OtA5093-OtA5097
Cog3	chr14:75702350-75754493	OtA5088-OtA5092	OtA5093-OtA5097
Csda	chr6:131364857-131388450	OtA5088-OtA5092	OtA5093-OtA5097
Ebna1bp2	chr4:118620798-118631915	OtA5088-OtA5092	OtA5093-OtA5097
Hs6st1	chr1:36068399-36106446	OtA5088-OtA5092	OtA5093-OtA5097
Ier5	chr1:155096366-155099636	OtA5088-OtA5092	OtA5093-OtA5097
Kbtbd4	chr2:90904785-90910560	OtA5088-OtA5092	OtA5093-OtA5097
Nkiras2	chr11:100622954-100650693	OtA5088-OtA5092	OtA5093-OtA5097
Pitx1	chr13:55825053-55831425	OtA5088-OtA5092	OtA5093-OtA5097
Pkig	chr2:163658385-163726158	OtA5088-OtA5092	OtA5093-OtA5097
Ppif	chr14:25694169-25701282	OtA5088-OtA5092	OtA5093-OtA5097
Psmb8	chr17:34198194-34201454	OtA5088-OtA5092	OtA5093-OtA5097
Sap18	chr14:57798188-57804980	OtA5088-OtA5092	OtA5093-OtA5097
She	chr3:89831369-89858846	OtA5088-OtA5092	OtA5093-OtA5097
Suclg1	chr6:73248504-73276907	OtA5088-OtA5092	OtA5093-OtA5097
Trpm4	chr7:45303154-45333780	OtA5088-OtA5092	OtA5093-OtA5097
Wipf1	chr2:73429616-73529410	OtA5088-OtA5092	OtA5093-OtA5097
Psip1	chr4:83455680-83486390	OtA5088-OtA5092	OtA5093-OtA5097
Tmem176a	chr6:48841482-48845364	OtA5088-OtA5092	OtA5093-OtA5097
Fez1	chr9:36843658-36878640	OtA5088-OtA5092	OtA5093-OtA5097

Trp53bp1	chr2:121171205-121271386	OtA5088-OtA5092	OtA5093-OtA5097
Rbmxl1	chr8:78505268-78508928	OtA5088-OtA5092	OtA5093-OtA5097
Clptm1l	chr13:73604001-73620639	OtA5088-OtA5092	OtA5093-OtA5097
Eif5	chr12:111538100-111546753	OtA5088-OtA5092	OtA5093-OtA5097
Fkbp11	chr15:98724367-98728198	OtA5088-OtA5092	OtA5093-OtA5097
Hist1h2an	chr13:21786825-21787218	OtA5088-OtA5092	OtA5093-OtA5097
Pex19	chr1:172126754-172136497	OtA5088-OtA5092	OtA5093-OtA5097
Fibp	chr19:5460606-5465052	OtA5088-OtA5092	OtA5093-OtA5097
Litaf	chr16:10959272-10993121	OtA5088-OtA5092	OtA5093-OtA5097
Agk	chr6:40325477-40396762	OtA5088-OtA5092	OtA5093-OtA5097
Atp6v1f	chr6:29467782-29470509	OtA5088-OtA5092	OtA5093-OtA5097
Ceacam11	chr7:17972166-17978556	OtA5088-OtA5092	OtA5093-OtA5097
Ciz1	chr2:32363009-32378313	OtA5088-OtA5092	OtA5093-OtA5097
Ercc2	chr7:19382038-19399856	OtA5088-OtA5092	OtA5093-OtA5097
Shank3	chr15:89499856-89560260	OtA5088-OtA5092	OtA5093-OtA5097
Spty2d1	chr7:46990395-47008414	OtA5088-OtA5092	OtA5093-OtA5097
Sumo1	chr1:59639433-59670834	OtA5088-OtA5092	OtA5093-OtA5097
Ythdc1	chr5:86804489-86836657	OtA5088-OtA5092	OtA5093-OtA5097
Hist2h3c1	chr3:96246684-96248509	OtA5088-OtA5092	OtA5093-OtA5097
Uhrf1bp1l	chr10:89744990-89819869	OtA5088-OtA5092	OtA5093-OtA5097
Vangl2	chr1:172004370-172027268	OtA5088-OtA5092	OtA5093-OtA5097
Ostm1	chr10:42678915-42702462	OtA5088-OtA5092	OtA5093-OtA5097
Zfp180	chr7:24081943-24107215	OtA5088-OtA5092	OtA5093-OtA5097
Atp6v0e	chr17:26676395-26699646	OtA5088-OtA5092	OtA5093-OtA5097
Kpna3	chr14:61365185-61439947	OtA5088-OtA5092	OtA5093-OtA5097
Prph	chr15:99055173-99058978	OtA5088-OtA5092	OtA5093-OtA5097
Sh3bp1	chr15:78899785-78912052	OtA5088-OtA5092	OtA5093-OtA5097
Mex3b	chr7:82867332-82871576	OtA5088-OtA5092	OtA5093-OtA5097
Spink3	chr18:43728068-43737237	OtA5088-OtA5092	OtA5093-OtA5097
Tiparp	chr3:65527484-65555518	OtA5088-OtA5092	OtA5093-OtA5097
Tmem86a	chr7:47050639-47054776	OtA5088-OtA5092	OtA5093-OtA5097
Ttc38	chr15:85832303-85858822	OtA5088-OtA5092	OtA5093-OtA5097
Ttc7b	chr12:100276608-100520822	OtA5088-OtA5092	OtA5093-OtA5097
Ube2a	chrX:36874365-36884220	OtA5088-OtA5092	OtA5093-OtA5097
Ubxn4	chr1:128244180-128279377	OtA5088-OtA5092	OtA5093-OtA5097
Gsdmd	chr15:75862338-75867404	OtA5088-OtA5092	OtA5093-OtA5097
Naa30	chr14:49172226-49191031	OtA5088-OtA5092	OtA5093-OtA5097
Nos3	chr5:24364818-24392143	OtA5088-OtA5092	OtA5093-OtA5097
Pkn3	chr2:30078765-30093635	OtA5088-OtA5092	OtA5093-OtA5097
Il3ra	chr14:14346494-14355490	OtA5088-OtA5092	OtA5093-OtA5097
Ppp1r3d	chr2:178411206-178414472	OtA5088-OtA5092	OtA5093-OtA5097
Adam19	chr11:46055986-46147342	OtA5088-OtA5092	OtA5093-OtA5097
Aff4	chr11:53350766-53421830	OtA5088-OtA5092	OtA5093-OtA5097
Chrna4	chr2:181022310-181039177	OtA5088-OtA5092	OtA5093-OtA5097
Crim1	chr17:78200247-78376592	OtA5088-OtA5092	OtA5093-OtA5097
Ecd	chr14:20319858-20348121	OtA5088-OtA5092	OtA5093-OtA5097
Ldlrap1	chr4:134745411-134768004	OtA5088-OtA5092	OtA5093-OtA5097

Mfn1	chr3:32529481-32579225	OtA5088-OtA5092	OtA5093-OtA5097
Nr2c2	chr6:92091417-92184023	OtA5088-OtA5092	OtA5093-OtA5097
Nr6a1	chr2:38723373-38926461	OtA5088-OtA5092	OtA5093-OtA5097
Ipp	chr4:116507548-116538235	OtA5088-OtA5092	OtA5093-OtA5097
Ctdnep1	chr11:69968225-69990600	OtA5088-OtA5092	OtA5093-OtA5097
Phka2	chrX:160502165-160598878	OtA5088-OtA5092	OtA5093-OtA5097
Mrpl54	chr10:81264721-81266926	OtA5088-OtA5092	OtA5093-OtA5097
Serpinb9e	chr13:33249609-33260846	OtA5088-OtA5092	OtA5093-OtA5097
Tm2d2	chr8:25017210-25023260	OtA5088-OtA5092	OtA5093-OtA5097
Pvr	chr7:19903577-19921143	OtA5088-OtA5092	OtA5093-OtA5097
Mapre3	chr5:30814755-30866106	OtA5088-OtA5092	OtA5093-OtA5097
Camta2	chr11:70669462-70688105	OtA5088-OtA5092	OtA5093-OtA5097
Eif4e	chr3:138526190-138557599	OtA5088-OtA5092	OtA5093-OtA5097
Mat2b	chr11:40679313-40695203	OtA5088-OtA5092	OtA5093-OtA5097
Tax1bp1	chr6:52713728-52766779	OtA5088-OtA5092	OtA5093-OtA5097
Abca4	chr3:122044459-122180061	OtA5088-OtA5092	OtA5093-OtA5097
Ap1g2	chr14:55094783-55106593	OtA5088-OtA5092	OtA5093-OtA5097
Chmp6	chr11:119913809-119919552	OtA5088-OtA5092	OtA5093-OtA5097
Ears2	chr7:122038820-122067063	OtA5088-OtA5092	OtA5093-OtA5097
Eif2b2	chr12:85219512-85226628	OtA5088-OtA5092	OtA5093-OtA5097
Emp2	chr16:10281748-10313968	OtA5088-OtA5092	OtA5093-OtA5097
Gpr137	chr19:6938069-6951893	OtA5088-OtA5092	OtA5093-OtA5097
Grem2	chr1:174833784-174921819	OtA5088-OtA5092	OtA5093-OtA5097
Grwd1	chr7:45825222-45830789	OtA5088-OtA5092	OtA5093-OtA5097
Hint2	chr4:43654226-43656445	OtA5088-OtA5092	OtA5093-OtA5097
Mlec	chr5:115142980-115158176	OtA5088-OtA5092	OtA5093-OtA5097
Parp3	chr9:106470352-106476651	OtA5088-OtA5092	OtA5093-OtA5097
Pbld1	chr10:63061616-63077536	OtA5088-OtA5092	OtA5093-OtA5097
Ptpla	chr2:14026830-14056035	OtA5088-OtA5092	OtA5093-OtA5097
Rnf125	chr18:20944624-20983848	OtA5088-OtA5092	OtA5093-OtA5097
Sh3bp4	chr1:89070461-89153793	OtA5088-OtA5092	OtA5093-OtA5097
Slc4a8	chr15:100761746-100823971	OtA5088-OtA5092	OtA5093-OtA5097
Sptlc1	chr13:53332747-53377361	OtA5088-OtA5092	OtA5093-OtA5097
Tgif1	chr17:70844204-70853532	OtA5088-OtA5092	OtA5093-OtA5097
Tnpo2	chr8:85036914-85057582	OtA5088-OtA5092	OtA5093-OtA5097
Ubiad1	chr4:148434496-148444751	OtA5088-OtA5092	OtA5093-OtA5097
Upf3b	chrX:37091833-37110322	OtA5088-OtA5092	OtA5093-OtA5097
Usp31	chr7:121642020-121707253	OtA5088-OtA5092	OtA5093-OtA5097
Wls	chr3:159839694-159935175	OtA5088-OtA5092	OtA5093-OtA5097
Xylt1	chr7:117380978-117667630	OtA5088-OtA5092	OtA5093-OtA5097
Arfgap3	chr15:83299739-83350247	OtA5088-OtA5092	OtA5093-OtA5097
Klhl15	chrX:94234929-94273515	OtA5088-OtA5092	OtA5093-OtA5097
Myd88	chr9:119335987-119340040	OtA5088-OtA5092	OtA5093-OtA5097
P2rx4	chr5:122707556-122729042	OtA5088-OtA5092	OtA5093-OtA5097
Pdhx	chr2:103021056-103073513	OtA5088-OtA5092	OtA5093-OtA5097
Psmd2	chr16:20651651-20663414	OtA5088-OtA5092	OtA5093-OtA5097
Tuft1	chr3:94612758-94658806	OtA5088-OtA5092	OtA5093-OtA5097

Atf6b	chr17:34647179-34655072	OtA5088-OtA5092	OtA5093-OtA5097
Sele	chr1:164048233-164057677	OtA5088-OtA5092	OtA5093-OtA5097
Apol9a	chr15:77403788-77411080	OtA5088-OtA5092	OtA5093-OtA5097
Arrdc1	chr2:24925351-24935281	OtA5088-OtA5092	OtA5093-OtA5097
Copz2	chr11:96849875-96861202	OtA5088-OtA5092	OtA5093-OtA5097
Epb4.1	chr4:131923426-132075313	OtA5088-OtA5092	OtA5093-OtA5097
Fam165b	chr16:92301302-92313041	OtA5088-OtA5092	OtA5093-OtA5097
Maml1	chr11:50255634-50292336	OtA5088-OtA5092	OtA5093-OtA5097
Plekha7	chr7:116124117-116189841	OtA5088-OtA5092	OtA5093-OtA5097
Psat1	chr19:15905122-15925059	OtA5088-OtA5092	OtA5093-OtA5097
Ran	chr5:129020155-129024321	OtA5088-OtA5092	OtA5093-OtA5097
Rassf3	chr10:121410350-121476250	OtA5088-OtA5092	OtA5093-OtA5097
Skiv2l2	chr13:112867779-112927380	OtA5088-OtA5092	OtA5093-OtA5097
Slc28a2	chr2:122426476-122461130	OtA5088-OtA5092	OtA5093-OtA5097
Ssbp1	chr6:40471414-40481823	OtA5088-OtA5092	OtA5093-OtA5097
Gkn2	chr6:87373364-87379494	OtA5088-OtA5092	OtA5093-OtA5097
Fam123b	chrX:95420313-95444840	OtA5088-OtA5092	OtA5093-OtA5097
Afap1l2	chr19:56912353-57008575	OtA5088-OtA5092	OtA5093-OtA5097
Ctnnb1	chr9:120933399-120960507	OtA5088-OtA5092	OtA5093-OtA5097
Ifngr2	chr16:91547093-91564007	OtA5088-OtA5092	OtA5093-OtA5097
Pdrg1	chr2:153008889-153015383	OtA5088-OtA5092	OtA5093-OtA5097
Usp38	chr8:80980732-81014906	OtA5088-OtA5092	OtA5093-OtA5097
Eef1e1	chr13:38645690-38659028	OtA5088-OtA5092	OtA5093-OtA5097
Leprel2	chr6:124841094-124857687	OtA5088-OtA5092	OtA5093-OtA5097
Cyb5r4	chr9:87022028-87077774	OtA5088-OtA5092	OtA5093-OtA5097
Cd97	chr8:83715176-83741311	OtA5088-OtA5092	OtA5093-OtA5097
Cltc	chr11:86694652-86757492	OtA5088-OtA5092	OtA5093-OtA5097
Sp100	chr1:85650049-85709448	OtA5088-OtA5092	OtA5093-OtA5097
Aph1b	chr9:66775486-66795423	OtA5088-OtA5092	OtA5093-OtA5097
Arhgap31	chr16:38598342-38713035	OtA5088-OtA5092	OtA5093-OtA5097
Cgref1	chr5:30933142-30945480	OtA5088-OtA5092	OtA5093-OtA5097
Cul7	chr17:46650337-46664364	OtA5088-OtA5092	OtA5093-OtA5097
D630045J12Rik	chr6:38048482-38254009	OtA5088-OtA5092	OtA5093-OtA5097
Foxa2	chr2:148042877-148046969	OtA5088-OtA5092	OtA5093-OtA5097
Mxd4	chr5:34176579-34187710	OtA5088-OtA5092	OtA5093-OtA5097
Arpc1b	chr5:145114255-145128186	OtA5088-OtA5092	OtA5093-OtA5097
Entpd7	chr19:43689688-43753000	OtA5088-OtA5092	OtA5093-OtA5097
Rps2	chr17:24720062-24721927	OtA5088-OtA5092	OtA5093-OtA5097
Pgls	chr8:71592183-71596267	OtA5088-OtA5092	OtA5093-OtA5097
Uba3	chr6:97183815-97205642	OtA5088-OtA5092	OtA5093-OtA5097
Rps19-ps3	chr4:147821776-147822202	OtA5088-OtA5092	OtA5093-OtA5097
Adam12	chr7:133883198-134225097	OtA5088-OtA5092	OtA5093-OtA5097
Lman2	chr13:55343832-55362783	OtA5088-OtA5092	OtA5093-OtA5097
Pdlim5	chr3:142239584-142395696	OtA5088-OtA5092	OtA5093-OtA5097
Rabgap1	chr2:37443284-37566437	OtA5088-OtA5092	OtA5093-OtA5097
Acbd3	chr1:180726042-180754204	OtA5088-OtA5092	OtA5093-OtA5097
Vprbp	chr9:106821975-106880992	OtA5088-OtA5092	OtA5093-OtA5097

Ahr	chr12:35497978-35534989	OtA5088-OtA5092	OtA5093-OtA5097
Birc2	chr9:7818225-7835255	OtA5088-OtA5092	OtA5093-OtA5097
Ndufb2	chr6:39592582-39599471	OtA5088-OtA5092	OtA5093-OtA5097
Wdr91	chr6:34880425-34910831	OtA5088-OtA5092	OtA5093-OtA5097
Adrm1	chr2:180171587-180176283	OtA5088-OtA5092	OtA5093-OtA5097
Cyld	chr8:88697027-88751945	OtA5088-OtA5092	OtA5093-OtA5097
Ndufa5	chr6:24518665-24527687	OtA5088-OtA5092	OtA5093-OtA5097
Pisd-ps1	chr11:3124020-3193463	OtA5088-OtA5092	OtA5093-OtA5097
Arhgef18	chr8:3393007-3456600	OtA5088-OtA5092	OtA5093-OtA5097
Aqp5	chr15:99591027-99594829	OtA5088-OtA5092	OtA5093-OtA5097
Ccdc23	chr4:119195309-119201298	OtA5088-OtA5092	OtA5093-OtA5097
Dnajc18	chr18:35671104-35703144	OtA5088-OtA5092	OtA5093-OtA5097
Flrt2	chr12:95692225-95785213	OtA5088-OtA5092	OtA5093-OtA5097
Gmnn	chr13:24751844-24761937	OtA5088-OtA5092	OtA5093-OtA5097
Hdlbp	chr1:93405939-93478808	OtA5088-OtA5092	OtA5093-OtA5097
Imp4	chr1:34439828-34445747	OtA5088-OtA5092	OtA5093-OtA5097
Mdk	chr2:91922188-91932282	OtA5088-OtA5092	OtA5093-OtA5097
Mesdc1	chr7:83880494-83884341	OtA5088-OtA5092	OtA5093-OtA5097
Mpv17l2	chr8:70754678-70760921	OtA5088-OtA5092	OtA5093-OtA5097
Plbd2	chr5:120476546-120503623	OtA5088-OtA5092	OtA5093-OtA5097
Rab25	chr3:88542028-88548279	OtA5088-OtA5092	OtA5093-OtA5097
Tmem143	chr7:45897068-45917413	OtA5088-OtA5092	OtA5093-OtA5097
Tmem30a	chr9:79768940-79793430	OtA5088-OtA5092	OtA5093-OtA5097
Unc119b	chr5:115122565-115134975	OtA5088-OtA5092	OtA5093-OtA5097
Usp4	chr9:108347830-108392529	OtA5088-OtA5092	OtA5093-OtA5097
Maf	chr8:115703252-115706894	OtA5088-OtA5092	OtA5093-OtA5097
Slc37a4	chr9:44398175-44402965	OtA5088-OtA5092	OtA5093-OtA5097
Grina	chr15:76246806-76249904	OtA5088-OtA5092	OtA5093-OtA5097
Sptlc2	chr12:87307888-87388230	OtA5088-OtA5092	OtA5093-OtA5097
B3galt6	chr4:155989465-155992678	OtA5088-OtA5092	OtA5093-OtA5097
Fbln5	chr12:101746564-101819055	OtA5088-OtA5092	OtA5093-OtA5097
Med11	chr11:70451930-70453726	OtA5088-OtA5092	OtA5093-OtA5097
Nagpa	chr16:5195279-5204012	OtA5088-OtA5092	OtA5093-OtA5097
Ppp4c	chr7:126785867-126792471	OtA5088-OtA5092	OtA5093-OtA5097
Serpina3n	chr12:104406707-104414329	OtA5088-OtA5092	OtA5093-OtA5097
Arcn1	chr9:44742143-44767808	OtA5088-OtA5092	OtA5093-OtA5097
Ttc15	chr12:28690627-28750453	OtA5088-OtA5092	OtA5093-OtA5097
C1qbp	chr11:70970199-70983026	OtA5088-OtA5092	OtA5093-OtA5097
Frmd6	chr12:70825513-70902234	OtA5088-OtA5092	OtA5093-OtA5097
Got2	chr8:95864136-95888365	OtA5088-OtA5092	OtA5093-OtA5097
Gtpbp4	chr13:8972477-8996012	OtA5088-OtA5092	OtA5093-OtA5097
Ica1	chr6:8630526-8778484	OtA5088-OtA5092	OtA5093-OtA5097
Kctd12	chr14:102976580-102982637	OtA5088-OtA5092	OtA5093-OtA5097
Msl3l2	chr10:56014293-56228689	OtA5088-OtA5092	OtA5093-OtA5097
Noc4l	chr5:110648418-110653382	OtA5088-OtA5092	OtA5093-OtA5097
Rps27a	chr11:29545841-29578352	OtA5088-OtA5092	OtA5093-OtA5097
Sema3g	chr14:31217872-31229511	OtA5088-OtA5092	OtA5093-OtA5097

Spr	chr6:85133679-85137764	OtA5088-OtA5092	OtA5093-OtA5097
Tax1bp3	chr11:73177082-73182046	OtA5088-OtA5092	OtA5093-OtA5097
Tmem160	chr7:16452778-16455490	OtA5088-OtA5092	OtA5093-OtA5097
Wbp1	chr6:83119043-83121461	OtA5088-OtA5092	OtA5093-OtA5097
Tmem41a	chr16:21934326-21947552	OtA5088-OtA5092	OtA5093-OtA5097
Tomm20	chr8:126930663-126945921	OtA5088-OtA5092	OtA5093-OtA5097
Hcls1	chr16:36934982-36963214	OtA5088-OtA5092	OtA5093-OtA5097
Otud6b	chr4:14809504-14826587	OtA5088-OtA5092	OtA5093-OtA5097
Tmem67	chr4:12039355-12087957	OtA5088-OtA5092	OtA5093-OtA5097
Farsa	chr8:84856985-84869257	OtA5088-OtA5092	OtA5093-OtA5097
Ggta1	chr2:35400178-35461425	OtA5088-OtA5092	OtA5093-OtA5097
Psmc2	chr5:21785282-21803784	OtA5088-OtA5092	OtA5093-OtA5097
Ckap4	chr10:84526304-84533888	OtA5088-OtA5092	OtA5093-OtA5097
Clptm1	chr7:19631579-19665030	OtA5088-OtA5092	OtA5093-OtA5097
Stmn2	chr3:8509526-8561604	OtA5088-OtA5092	OtA5093-OtA5097
Tmem200b	chr4:131921770-131923140	OtA5088-OtA5092	OtA5093-OtA5097
Col14a1	chr15:55307749-55520803	OtA5088-OtA5092	OtA5093-OtA5097
Gripap1	chrX:7789994-7820563	OtA5088-OtA5092	OtA5093-OtA5097
Pskh1	chr8:105900473-105931802	OtA5088-OtA5092	OtA5093-OtA5097
Fxyd5	chr7:31032722-31042322	OtA5088-OtA5092	OtA5093-OtA5097
Cd34	chr1:194938820-194976959	OtA5088-OtA5092	OtA5093-OtA5097
Uqcr11	chr10:80402996-80406821	OtA5088-OtA5092	OtA5093-OtA5097
Cfdp1	chr8:111768472-111854310	OtA5088-OtA5092	OtA5093-OtA5097
Lrp4	chr2:91457530-91513901	OtA5088-OtA5092	OtA5093-OtA5097
Pfkfb1	chrX:150589920-150643860	OtA5088-OtA5092	OtA5093-OtA5097
Zfp874b	chr13:67471512-67484253	OtA5088-OtA5092	OtA5093-OtA5097
C330006K01Rik	chr5:142463930-142478715	OtA5088-OtA5092	OtA5093-OtA5097
Cat	chr2:103453903-103485153	OtA5088-OtA5092	OtA5093-OtA5097
Col20a1	chr2:180986534-181017540	OtA5088-OtA5092	OtA5093-OtA5097
Crip1	chr12:113152011-113153879	OtA5088-OtA5092	OtA5093-OtA5097
Dgat1	chr15:76502014-76511818	OtA5088-OtA5092	OtA5093-OtA5097
Dopey1	chr9:86467153-86571842	OtA5088-OtA5092	OtA5093-OtA5097
Mob1b	chr5:88720870-88758455	OtA5088-OtA5092	OtA5093-OtA5097
Pon3	chr6:5220851-5256233	OtA5088-OtA5092	OtA5093-OtA5097
Pus7	chr5:23740695-23783661	OtA5088-OtA5092	OtA5093-OtA5097
Rusc1	chr3:89083978-89093363	OtA5088-OtA5092	OtA5093-OtA5097
Sema4g	chr19:44989343-45003395	OtA5088-OtA5092	OtA5093-OtA5097
Slc7a8	chr14:54722214-54781886	OtA5088-OtA5092	OtA5093-OtA5097
Tmem127	chr2:127247974-127260764	OtA5088-OtA5092	OtA5093-OtA5097
Tmem131	chr1:36792188-36939527	OtA5088-OtA5092	OtA5093-OtA5097
Tmem87a	chr2:120355308-120504919	OtA5088-OtA5092	OtA5093-OtA5097
Tomm5	chr4:45105209-45108113	OtA5088-OtA5092	OtA5093-OtA5097
Uhmk1	chr1:170199255-170215393	OtA5088-OtA5092	OtA5093-OtA5097
Vti1a	chr19:55316350-55626561	OtA5088-OtA5092	OtA5093-OtA5097
Qpct	chr17:79051905-79090243	OtA5088-OtA5092	OtA5093-OtA5097
Tspan31	chr10:127067289-127070261	OtA5088-OtA5092	OtA5093-OtA5097
Ube3a	chr7:59228749-59306727	OtA5088-OtA5092	OtA5093-OtA5097

Ccs	chr19:4825365-4839322	OtA5088-OtA5092	OtA5093-OtA5097
Clasp1	chr1:118389057-118609462	OtA5088-OtA5092	OtA5093-OtA5097
Ypel3	chr7:126776974-126780514	OtA5088-OtA5092	OtA5093-OtA5097
Commd3	chr2:18672461-18676216	OtA5088-OtA5092	OtA5093-OtA5097
Pnpla6	chr8:3515383-3544267	OtA5088-OtA5092	OtA5093-OtA5097
Gnb5	chr9:75311394-75345671	OtA5088-OtA5092	OtA5093-OtA5097
Olfml1	chr7:107284774-107591365	OtA5088-OtA5092	OtA5093-OtA5097
Pnp	chr14:50944302-50953412	OtA5088-OtA5092	OtA5093-OtA5097
Nme6	chr9:109832793-109842961	OtA5088-OtA5092	OtA5093-OtA5097
Prkar2b	chr12:31958478-32061279	OtA5088-OtA5092	OtA5093-OtA5097
Rb1	chr14:73195501-73325791	OtA5088-OtA5092	OtA5093-OtA5097
Zeb2	chr2:44983633-45114084	OtA5088-OtA5092	OtA5093-OtA5097
Dnmt3a	chr12:3806979-3914443	OtA5088-OtA5092	OtA5093-OtA5097
Sh3rf1	chr8:61224170-61396072	OtA5088-OtA5092	OtA5093-OtA5097
Pdgfrb	chr18:61045149-61085067	OtA5088-OtA5092	OtA5093-OtA5097
Srebf2	chr15:82147268-82204960	OtA5088-OtA5092	OtA5093-OtA5097
Zfp428	chr7:24507086-24515682	OtA5088-OtA5092	OtA5093-OtA5097
Zfx	chrX:94074630-94123407	OtA5088-OtA5092	OtA5093-OtA5097
Copb1	chr7:114215558-114254680	OtA5088-OtA5092	OtA5093-OtA5097
Eps8l1	chr7:4464741-4479242	OtA5088-OtA5092	OtA5093-OtA5097
Iqgap2	chr13:95627176-95891922	OtA5088-OtA5092	OtA5093-OtA5097
Mtr	chr13:12186539-12258113	OtA5088-OtA5092	OtA5093-OtA5097
Pomc	chr12:3954950-3960618	OtA5088-OtA5092	OtA5093-OtA5097
Tspan2	chr3:102720230-102772310	OtA5088-OtA5092	OtA5093-OtA5097
Creb3l1	chr2:91982327-92024170	OtA5088-OtA5092	OtA5093-OtA5097
Fam176b	chr4:126148002-126149874	OtA5088-OtA5092	OtA5093-OtA5097
Aco2	chr15:81872462-81926213	OtA5088-OtA5092	OtA5093-OtA5097
Ptma	chr1:86526735-86530698	OtA5088-OtA5092	OtA5093-OtA5097
Camkk2	chr5:122731169-122779410	OtA5088-OtA5092	OtA5093-OtA5097
Foxn2	chr17:88440711-88490533	OtA5088-OtA5092	OtA5093-OtA5097
Ggh	chr4:20042051-20066111	OtA5088-OtA5092	OtA5093-OtA5097
Lad1	chr1:135818597-135833341	OtA5088-OtA5092	OtA5093-OtA5097
Rusc2	chr4:43381981-43429134	OtA5088-OtA5092	OtA5093-OtA5097
Lrig3	chr10:125966218-126015359	OtA5088-OtA5092	OtA5093-OtA5097
Crem	chr18:3266353-3366863	OtA5088-OtA5092	OtA5093-OtA5097
Cul2	chr18:3383224-3436700	OtA5088-OtA5092	OtA5093-OtA5097
Itpr1	chr6:108213095-108551116	OtA5088-OtA5092	OtA5093-OtA5097
Pabpc4	chr4:123282910-123298835	OtA5088-OtA5092	OtA5093-OtA5097
Fam113b	chr15:97244073-97385691	OtA5088-OtA5092	OtA5093-OtA5097
Cenpc1	chr5:86012024-86065583	OtA5088-OtA5092	OtA5093-OtA5097
Ergic3	chr2:156008124-156018279	OtA5088-OtA5092	OtA5093-OtA5097
Fry	chr5:150259929-150497753	OtA5088-OtA5092	OtA5093-OtA5097
Spsb3	chr17:24886673-24892147	OtA5088-OtA5092	OtA5093-OtA5097
Vps25	chr11:101253742-101259546	OtA5088-OtA5092	OtA5093-OtA5097
Ankrd44	chr1:54645339-54926387	OtA5088-OtA5092	OtA5093-OtA5097
Cdhr2	chr13:54701462-54736662	OtA5088-OtA5092	OtA5093-OtA5097
Cdx1	chr18:61018861-61036199	OtA5088-OtA5092	OtA5093-OtA5097

Gpatch3	chr4:133574744-133584242	OtA5088-OtA5092	OtA5093-OtA5097
Ift122	chr6:115853527-115926699	OtA5088-OtA5092	OtA5093-OtA5097
Med22	chr2:26905266-26910642	OtA5088-OtA5092	OtA5093-OtA5097
Psmb1	chr17:15475720-15498276	OtA5088-OtA5092	OtA5093-OtA5097
Purg	chr8:33386324-33417469	OtA5088-OtA5092	OtA5093-OtA5097
Rbm4b	chr19:4756524-4765941	OtA5088-OtA5092	OtA5093-OtA5097
Samd9l	chr6:3372257-3399571	OtA5088-OtA5092	OtA5093-OtA5097
Smad2	chr18:76241700-76311747	OtA5088-OtA5092	OtA5093-OtA5097
Snw1	chr12:87428077-87472299	OtA5088-OtA5092	OtA5093-OtA5097
Stx2	chr5:128984560-129008564	OtA5088-OtA5092	OtA5093-OtA5097
Thoc5	chr11:4895342-4928865	OtA5088-OtA5092	OtA5093-OtA5097
Tmub1	chr5:24445462-24447846	OtA5088-OtA5092	OtA5093-OtA5097
Sec24b	chr3:129983184-130060907	OtA5088-OtA5092	OtA5093-OtA5097
Ldlrad3	chr2:101950200-102186377	OtA5088-OtA5092	OtA5093-OtA5097
Alyref	chr11:120594515-120598365	OtA5088-OtA5092	OtA5093-OtA5097
Ciao1	chr2:127240937-127247816	OtA5088-OtA5092	OtA5093-OtA5097
Gpr125	chr5:49959950-50058996	OtA5088-OtA5092	OtA5093-OtA5097
Prkaca	chr8:83972977-83996445	OtA5088-OtA5092	OtA5093-OtA5097
Apaf1	chr10:90989310-91082743	OtA5088-OtA5092	OtA5093-OtA5097
Cda	chr4:138338527-138367955	OtA5088-OtA5092	OtA5093-OtA5097
Zc3h18	chr8:122376615-122417360	OtA5088-OtA5092	OtA5093-OtA5097
Rapgef4	chr2:71981214-72257474	OtA5088-OtA5092	OtA5093-OtA5097
Pdk4	chr6:5483350-5496278	OtA5088-OtA5092	OtA5093-OtA5097
Rpn2	chr2:157208549-157326318	OtA5088-OtA5092	OtA5093-OtA5097
P2ry1	chr3:61002794-61008979	OtA5088-OtA5092	OtA5093-OtA5097
Usp30	chr5:114100332-114122924	OtA5088-OtA5092	OtA5093-OtA5097
Zfp592	chr7:80993683-81045162	OtA5088-OtA5092	OtA5093-OtA5097
Brap	chr5:121660585-121687247	OtA5088-OtA5092	OtA5093-OtA5097
Rars	chr11:35808380-35834528	OtA5088-OtA5092	OtA5093-OtA5097
Abcb6	chr1:75171640-75180283	OtA5088-OtA5092	OtA5093-OtA5097
Rbm26	chr14:105106727-105177327	OtA5088-OtA5092	OtA5093-OtA5097
Vps26a	chr10:62454842-62486805	OtA5088-OtA5092	OtA5093-OtA5097
Zkscan3	chr13:21387003-21402755	OtA5088-OtA5092	OtA5093-OtA5097
Fam83d	chr2:158768098-158786637	OtA5088-OtA5092	OtA5093-OtA5097
Pdhb	chr14:8165990-8172992	OtA5088-OtA5092	OtA5093-OtA5097
Hspb7	chr4:141420778-141425310	OtA5088-OtA5092	OtA5093-OtA5097
Cox4i1	chr8:120668289-120674209	OtA5088-OtA5092	OtA5093-OtA5097
Trmt61a	chr12:111678104-111683902	OtA5088-OtA5092	OtA5093-OtA5097
Psg20	chr7:18674365-18685992	OtA5088-OtA5092	OtA5093-OtA5097
Rps4x	chrX:102184942-102188371	OtA5088-OtA5092	OtA5093-OtA5097
Sc4mol	chr8:64718144-64733578	OtA5088-OtA5092	OtA5093-OtA5097
Sgpp1	chr12:75714247-75735729	OtA5088-OtA5092	OtA5093-OtA5097
Rasgrf1	chr9:89909774-90026979	OtA5088-OtA5092	OtA5093-OtA5097
Zfp617	chr8:71922824-71934630	OtA5088-OtA5092	OtA5093-OtA5097
Cdc42ep1	chr15:78842646-78855529	OtA5088-OtA5092	OtA5093-OtA5097
Clip2	chr5:134489385-134552434	OtA5088-OtA5092	OtA5093-OtA5097
Nudcd2	chr11:40733641-40740046	OtA5088-OtA5092	OtA5093-OtA5097

Phf2	chr13:48801749-48870885	OtA5088-OtA5092	OtA5093-OtA5097
Wscd2	chr5:113550419-113589725	OtA5088-OtA5092	OtA5093-OtA5097
Stambpl1	chr19:34192269-34240328	OtA5088-OtA5092	OtA5093-OtA5097
Akap1	chr11:88830791-88864586	OtA5088-OtA5092	OtA5093-OtA5097
P2rx5	chr11:73160529-73172687	OtA5088-OtA5092	OtA5093-OtA5097
Rbm18	chr2:36116078-36190283	OtA5088-OtA5092	OtA5093-OtA5097
Fitm2	chr2:163468702-163473003	OtA5088-OtA5092	OtA5093-OtA5097
Dynlt1c,Dynlt1f	chr17:6646605-6655939	OtA5088-OtA5092	OtA5093-OtA5097
Limk1	chr5:134656038-134688590	OtA5088-OtA5092	OtA5093-OtA5097
Ndel1	chr11:68821445-68853131	OtA5088-OtA5092	OtA5093-OtA5097
Stxbp2	chr8:3631159-3643644	OtA5088-OtA5092	OtA5093-OtA5097
Sft2d2	chr1:165174340-165194433	OtA5088-OtA5092	OtA5093-OtA5097
Ttc39c	chr18:12643532-12737052	OtA5088-OtA5092	OtA5093-OtA5097
Abr	chr11:76416733-76577727	OtA5088-OtA5092	OtA5093-OtA5097
Eri3	chr4:117550493-117674281	OtA5088-OtA5092	OtA5093-OtA5097
Txlna	chr4:129626076-129641065	OtA5088-OtA5092	OtA5093-OtA5097
Maf1	chr15:76351293-76354378	OtA5088-OtA5092	OtA5093-OtA5097
Pip4k2b	chr11:97715156-97744704	OtA5088-OtA5092	OtA5093-OtA5097
Tial1	chr7:128439776-128461513	OtA5088-OtA5092	OtA5093-OtA5097
Edc3	chr9:57708568-57750162	OtA5088-OtA5092	OtA5093-OtA5097
Zfp637	chr6:117841241-117845956	OtA5088-OtA5092	OtA5093-OtA5097
Prl6a1	chr13:27312626-27319252	OtA5088-OtA5092	OtA5093-OtA5097
Aggf1	chr13:95350716-95375334	OtA5088-OtA5092	OtA5093-OtA5097
B3galtl	chr5:149678256-149762599	OtA5088-OtA5092	OtA5093-OtA5097
Col9a2	chr4:121039565-121055325	OtA5088-OtA5092	OtA5093-OtA5097
Rybp	chr6:100228564-100287358	OtA5088-OtA5092	OtA5093-OtA5097
Lnx2	chr5:147016654-147076572	OtA5088-OtA5092	OtA5093-OtA5097
Atp5e	chr2:174461074-174464101	OtA5088-OtA5092	OtA5093-OtA5097
Ndufab1	chr7:122088043-122101848	OtA5088-OtA5092	OtA5093-OtA5097
Pde1b	chr15:103503297-103530052	OtA5088-OtA5092	OtA5093-OtA5097
Bet1	chr6:4076903-4086927	OtA5088-OtA5092	OtA5093-OtA5097
Gm8883	chr1:71888111-71891204	OtA5088-OtA5092	OtA5093-OtA5097
Igsf9	chr1:172482212-172498877	OtA5088-OtA5092	OtA5093-OtA5097
Mmp7	chr9:7692110-7699416	OtA5088-OtA5092	OtA5093-OtA5097
Thsd1	chr8:22227302-22261332	OtA5088-OtA5092	OtA5093-OtA5097
Zfyve16	chr13:92487748-92530810	OtA5088-OtA5092	OtA5093-OtA5097
Mafk	chr5:139791535-139802652	OtA5088-OtA5092	OtA5093-OtA5097
Mccc2	chr13:99948531-100015639	OtA5088-OtA5092	OtA5093-OtA5097
Abl2	chr1:156558786-156649619	OtA5088-OtA5092	OtA5093-OtA5097
Actr2	chr11:20062303-20112951	OtA5088-OtA5092	OtA5093-OtA5097
Atp6v1b2	chr8:69037707-69113717	OtA5088-OtA5092	OtA5093-OtA5097
Cdc42bpb	chr12:111292971-111377718	OtA5088-OtA5092	OtA5093-OtA5097
Egfr	chr11:16752202-16913907	OtA5088-OtA5092	OtA5093-OtA5097
Fam96b	chr8:104639838-104641728	OtA5088-OtA5092	OtA5093-OtA5097
Fdxacb1	chr9:50768237-50772670	OtA5088-OtA5092	OtA5093-OtA5097
Fut8	chr12:77238103-77475996	OtA5088-OtA5092	OtA5093-OtA5097
Hspb1	chr5:135887918-135889563	OtA5088-OtA5092	OtA5093-OtA5097

Lsm12	chr11:102163488-102185256	OtA5088-OtA5092	OtA5093-OtA5097
Meis1	chr11:18880427-19018969	OtA5088-OtA5092	OtA5093-OtA5097
Ndufa4	chr6:11900372-11907446	OtA5088-OtA5092	OtA5093-OtA5097
Pars2	chr4:106651068-106655282	OtA5088-OtA5092	OtA5093-OtA5097
Rap1gap	chr4:137664725-137729861	OtA5088-OtA5092	OtA5093-OtA5097
Zfp36l2	chr17:84183927-84187947	OtA5088-OtA5092	OtA5093-OtA5097
Adc	chr4:128932145-128962417	OtA5088-OtA5092	OtA5093-OtA5097
Mapkapk3	chr9:107254926-107289877	OtA5088-OtA5092	OtA5093-OtA5097
St14	chr9:31088589-31131799	OtA5088-OtA5092	OtA5093-OtA5097
Bcar1	chr8:111710474-111743849	OtA5088-OtA5092	OtA5093-OtA5097
Ssr4	chrX:73787027-73790828	OtA5088-OtA5092	OtA5093-OtA5097
Zmynd8	chr2:165784151-165884751	OtA5088-OtA5092	OtA5093-OtA5097
Mvd	chr8:122433595-122443422	OtA5088-OtA5092	OtA5093-OtA5097
Fam173b	chr15:31602115-31617535	OtA5088-OtA5092	OtA5093-OtA5097
Gkap1	chr13:58233350-58274188	OtA5088-OtA5092	OtA5093-OtA5097
Terf2ip	chr8:112011358-112020528	OtA5088-OtA5092	OtA5093-OtA5097
Lrpprc	chr17:84705246-84790786	OtA5088-OtA5092	OtA5093-OtA5097
Lrrc8d	chr5:105699968-105815215	OtA5088-OtA5092	OtA5093-OtA5097
Bag1	chr4:40936397-40948294	OtA5088-OtA5092	OtA5093-OtA5097
Fam32a	chr8:72219729-72223775	OtA5088-OtA5092	OtA5093-OtA5097
Khdrbs1	chr4:129703168-129742303	OtA5088-OtA5092	OtA5093-OtA5097
Sympk	chr7:19024376-19054622	OtA5088-OtA5092	OtA5093-OtA5097
Ap4b1	chr3:103809516-103822025	OtA5088-OtA5092	OtA5093-OtA5097
Bphl	chr13:34037640-34074074	OtA5088-OtA5092	OtA5093-OtA5097
Ptp4a1	chr1:30940302-30949755	OtA5088-OtA5092	OtA5093-OtA5097
Chaf1b	chr16:93883900-93906106	OtA5088-OtA5092	OtA5093-OtA5097
Dars2	chr1:161040612-161070632	OtA5088-OtA5092	OtA5093-OtA5097
Kif16b	chr2:142618344-142901464	OtA5088-OtA5092	OtA5093-OtA5097
Scamp2	chr9:57560943-57588798	OtA5088-OtA5092	OtA5093-OtA5097
Cpne3	chr4:19519251-19570104	OtA5088-OtA5092	OtA5093-OtA5097
Srek1ip1	chr13:104817238-104838659	OtA5088-OtA5092	OtA5093-OtA5097
Wdr8	chr4:154142371-154156818	OtA5088-OtA5092	OtA5093-OtA5097
Wnt5a	chr14:28505472-28525515	OtA5088-OtA5092	OtA5093-OtA5097
Adamts1	chr16:85793827-85803115	OtA5088-OtA5092	OtA5093-OtA5097
Apol6	chr15:77015486-77057108	OtA5088-OtA5092	OtA5093-OtA5097
Armc7	chr11:115475676-115491814	OtA5088-OtA5092	OtA5093-OtA5097
BC057079	chr4:88094629-88411011	OtA5088-OtA5092	OtA5093-OtA5097
Bcs1l	chr1:74588360-74592443	OtA5088-OtA5092	OtA5093-OtA5097
Bola1	chr3:96196587-96197586	OtA5088-OtA5092	OtA5093-OtA5097
C230096C10Rik	chr4:139352592-139378730	OtA5088-OtA5092	OtA5093-OtA5097
C330006A16Rik	chr2:26136806-26140506	OtA5088-OtA5092	OtA5093-OtA5097
Ccdc47	chr11:106199355-106216367	OtA5088-OtA5092	OtA5093-OtA5097
Cd72	chr4:43442276-43454626	OtA5088-OtA5092	OtA5093-OtA5097
Cdkn2a	chr4:89274472-89294619	OtA5088-OtA5092	OtA5093-OtA5097
Cdyl	chr13:35659861-35874064	OtA5088-OtA5092	OtA5093-OtA5097
Clec16a	chr16:10545338-10744878	OtA5088-OtA5092	OtA5093-OtA5097
Clic6	chr16:92498146-92541241	OtA5088-OtA5092	OtA5093-OtA5097

Cog8	chr8:107048708-107056737	OtA5088-OtA5092	OtA5093-OtA5097
Cpsf3l	chr4:155869566-155891762	OtA5088-OtA5092	OtA5093-OtA5097
Crybg3	chr16:59471774-59555752	OtA5088-OtA5092	OtA5093-OtA5097
Ddx19b	chr8:111003185-111031751	OtA5088-OtA5092	OtA5093-OtA5097
Dis3l	chr9:64306755-64341257	OtA5088-OtA5092	OtA5093-OtA5097
Dnajc17	chr2:119172499-119217050	OtA5088-OtA5092	OtA5093-OtA5097
Ehbp1l1	chr19:5707373-5726317	OtA5088-OtA5092	OtA5093-OtA5097
Eid2	chr7:28267880-28269168	OtA5088-OtA5092	OtA5093-OtA5097
Eif4g2	chr7:111067984-111083030	OtA5088-OtA5092	OtA5093-OtA5097
Ermp1	chr19:29609882-29648420	OtA5088-OtA5092	OtA5093-OtA5097
Ets1	chr9:32696041-32757820	OtA5088-OtA5092	OtA5093-OtA5097
Fcgbp	chr7:28071235-28120864	OtA5088-OtA5092	OtA5093-OtA5097
Fxr2	chr11:69632970-69653297	OtA5088-OtA5092	OtA5093-OtA5097
Gadd45b	chr10:80930090-80932204	OtA5088-OtA5092	OtA5093-OtA5097
Gale	chr4:135965164-135968178	OtA5088-OtA5092	OtA5093-OtA5097
Gm16039	chr6:8259287-8428765	OtA5088-OtA5092	OtA5093-OtA5097
Gstm2	chr3:107981701-107986436	OtA5088-OtA5092	OtA5093-OtA5097
Il6st	chr13:112464069-112506860	OtA5088-OtA5092	OtA5093-OtA5097
Lrwd1	chr5:136123065-136136074	OtA5088-OtA5092	OtA5093-OtA5097
Mark2	chr19:7275395-7341844	OtA5088-OtA5092	OtA5093-OtA5097
Max	chr12:76937268-76962248	OtA5088-OtA5092	OtA5093-OtA5097
Mrps31	chr8:22411339-22429665	OtA5088-OtA5092	OtA5093-OtA5097
Mzt2	chr16:15848440-15863322	OtA5088-OtA5092	OtA5093-OtA5097
Napepld	chr5:21662902-21701345	OtA5088-OtA5092	OtA5093-OtA5097
Nob1	chr8:107412488-107425038	OtA5088-OtA5092	OtA5093-OtA5097
Phf12	chr11:77982815-78030535	OtA5088-OtA5092	OtA5093-OtA5097
Pja2	chr17:64281005-64331883	OtA5088-OtA5092	OtA5093-OtA5097
Pla2g12a	chr3:129878621-129895821	OtA5088-OtA5092	OtA5093-OtA5097
Polr2g	chr19:8793128-8798557	OtA5088-OtA5092	OtA5093-OtA5097
Ppp1r14a	chr7:29289319-29293390	OtA5088-OtA5092	OtA5093-OtA5097
Pros1	chr16:62854333-62929340	OtA5088-OtA5092	OtA5093-OtA5097
Pura	chr18:36281161-36288244	OtA5088-OtA5092	OtA5093-OtA5097
Pxdn	chr12:29938035-30017658	OtA5088-OtA5092	OtA5093-OtA5097
Pxmp2	chr5:110274285-110286168	OtA5088-OtA5092	OtA5093-OtA5097
Rasl12	chr9:65398487-65422773	OtA5088-OtA5092	OtA5093-OtA5097
Rbm17	chr2:11585438-11603199	OtA5088-OtA5092	OtA5093-OtA5097
Rpl28	chr7:4792964-4794547	OtA5088-OtA5092	OtA5093-OtA5097
Rps24	chr14:24490680-24496146	OtA5088-OtA5092	OtA5093-OtA5097
Rtn4	chr11:29692897-30026033	OtA5088-OtA5092	OtA5093-OtA5097
Samd14	chr11:95009878-95026087	OtA5088-OtA5092	OtA5093-OtA5097
Sbsn	chr7:30751470-30756134	OtA5088-OtA5092	OtA5093-OtA5097
Smarcal1	chr1:72583250-72636790	OtA5088-OtA5092	OtA5093-OtA5097
Smarcb1	chr10:75896768-75921614	OtA5088-OtA5092	OtA5093-OtA5097
Spred1	chr2:117121449-117179336	OtA5088-OtA5092	OtA5093-OtA5097
Stag3	chr5:138280508-138312393	OtA5088-OtA5092	OtA5093-OtA5097
Sulf1	chr1:12692429-12860372	OtA5088-OtA5092	OtA5093-OtA5097
Supt6h	chr11:78206748-78245703	OtA5088-OtA5092	OtA5093-OtA5097

Tapt1	chr5:44175161-44226606	OtA5088-OtA5092	OtA5093-OtA5097
Tbcel	chr9:42412316-42472226	OtA5088-OtA5092	OtA5093-OtA5097
Tlcd1	chr11:78178765-78180772	OtA5088-OtA5092	OtA5093-OtA5097
Tmx2	chr2:84671316-84678047	OtA5088-OtA5092	OtA5093-OtA5097
Tspo2	chr17:48448434-48451501	OtA5088-OtA5092	OtA5093-OtA5097
Ubl7	chr9:57910985-57929968	OtA5088-OtA5092	OtA5093-OtA5097
Vti1b	chr12:79130787-79172458	OtA5088-OtA5092	OtA5093-OtA5097
Wdr47	chr3:108591277-108645719	OtA5088-OtA5092	OtA5093-OtA5097
Zkscan6	chr11:65807243-65829239	OtA5088-OtA5092	OtA5093-OtA5097
Dlx4	chr11:95140446-95159047	OtA5088-OtA5092	OtA5093-OtA5097
Apobec3	chr15:79889531-79908429	OtA5088-OtA5092	OtA5093-OtA5097
Kif20a	chr18:34598614-34651736	OtA5088-OtA5092	OtA5093-OtA5097
Sys1	chr2:164460970-164465510	OtA5088-OtA5092	OtA5093-OtA5097
Pou6f1	chr15:100575317-100586365	OtA5088-OtA5092	OtA5093-OtA5097
Anxa4	chr6:86736839-86793584	OtA5088-OtA5092	OtA5093-OtA5097
Efs	chr14:54916542-54926788	OtA5088-OtA5092	OtA5093-OtA5097
C78339	chr13:46669521-46675773	OtA5088-OtA5092	OtA5093-OtA5097
Dnase1l1	chrX:74273216-74282333	OtA5088-OtA5092	OtA5093-OtA5097
Ggt1	chr10:75573592-75586182	OtA5088-OtA5092	OtA5093-OtA5097
Itpa	chr2:130667840-130681614	OtA5088-OtA5092	OtA5093-OtA5097
Prpf19	chr19:10895230-10909559	OtA5088-OtA5092	OtA5093-OtA5097
Sdad1	chr5:92284009-92310024	OtA5088-OtA5092	OtA5093-OtA5097
5-Sep	chr16:18620318-18629938	OtA5088-OtA5092	OtA5093-OtA5097
Tsn	chr1:118298517-118311132	OtA5088-OtA5092	OtA5093-OtA5097
Eif2b1	chr5:124570213-124579057	OtA5088-OtA5092	OtA5093-OtA5097
Mylk	chr16:34784949-35002434	OtA5088-OtA5092	OtA5093-OtA5097
Wbscr22	chr5:135052956-135065365	OtA5088-OtA5092	OtA5093-OtA5097
Ccdc86	chr19:10937159-10949266	OtA5088-OtA5092	OtA5093-OtA5097
Commd6	chr14:101633765-101640471	OtA5088-OtA5092	OtA5093-OtA5097
Cpeb2	chr5:43151685-43289724	OtA5088-OtA5092	OtA5093-OtA5097
Ece2	chr16:20611600-20645915	OtA5088-OtA5092	OtA5093-OtA5097
Gcat	chr15:79030873-79043558	OtA5088-OtA5092	OtA5093-OtA5097
Glb1	chr9:114401094-114474377	OtA5088-OtA5092	OtA5093-OtA5097
Mfsd9	chr1:40772039-40790657	OtA5088-OtA5092	OtA5093-OtA5097
Obfc2a	chr1:51469487-51478399	OtA5088-OtA5092	OtA5093-OtA5097
Pak1	chr7:97842938-97912381	OtA5088-OtA5092	OtA5093-OtA5097
Pgm2	chr4:99929450-99987294	OtA5088-OtA5092	OtA5093-OtA5097
Rfc1	chr5:65261851-65335639	OtA5088-OtA5092	OtA5093-OtA5097
Sbds	chr5:130245731-130255462	OtA5088-OtA5092	OtA5093-OtA5097
Syt8	chr7:142434976-142440398	OtA5088-OtA5092	OtA5093-OtA5097
Fbf1	chr11:116142284-116168178	OtA5088-OtA5092	OtA5093-OtA5097
Sema4a	chr3:88435961-88461182	OtA5088-OtA5092	OtA5093-OtA5097
Rps6kb1	chr11:86415843-86544807	OtA5088-OtA5092	OtA5093-OtA5097
Rnf138	chr18:21001340-21028223	OtA5088-OtA5092	OtA5093-OtA5097
Apoo	chrX:94367109-94417092	OtA5088-OtA5092	OtA5093-OtA5097
Ddx60	chr8:61928088-62037695	OtA5088-OtA5092	OtA5093-OtA5097
Usp45	chr4:21776269-21837872	OtA5088-OtA5092	OtA5093-OtA5097

Anapc7	chr5:122422443-122444911	OtA5088-OtA5092	OtA5093-OtA5097
Frrs1	chr3:116859566-116903750	OtA5088-OtA5092	OtA5093-OtA5097
Gpn2	chr4:133584372-133591735	OtA5088-OtA5092	OtA5093-OtA5097
Higd2a	chr13:54590230-54591147	OtA5088-OtA5092	OtA5093-OtA5097
Luc7l	chr17:26252909-26285506	OtA5088-OtA5092	OtA5093-OtA5097
Prpf18	chr2:4622166-4652086	OtA5088-OtA5092	OtA5093-OtA5097
Smurf1	chr5:144876494-144965830	OtA5088-OtA5092	OtA5093-OtA5097
Xrcc2	chr5:25689815-25705797	OtA5088-OtA5092	OtA5093-OtA5097
Agap1	chr1:89454810-89895282	OtA5088-OtA5092	OtA5093-OtA5097
Ccdc134	chr15:82127921-82142202	OtA5088-OtA5092	OtA5093-OtA5097
Ccdc68	chr18:69925558-69969484	OtA5088-OtA5092	OtA5093-OtA5097
Ccnl1	chr3:65946150-65958225	OtA5088-OtA5092	OtA5093-OtA5097
Dtymk	chr1:93792575-93801934	OtA5088-OtA5092	OtA5093-OtA5097
Htr2b	chr1:86064618-86139295	OtA5088-OtA5092	OtA5093-OtA5097
Imp3	chr9:56937499-56938398	OtA5088-OtA5092	OtA5093-OtA5097
Inpp5b	chr4:124741877-124801511	OtA5088-OtA5092	OtA5093-OtA5097
Lpar2	chr8:69822564-69831102	OtA5088-OtA5092	OtA5093-OtA5097
Lrrc25	chr8:70616843-70620850	OtA5088-OtA5092	OtA5093-OtA5097
Mfsd7c	chr12:85746538-85813585	OtA5088-OtA5092	OtA5093-OtA5097
Pecam1	chr11:106654217-106715281	OtA5088-OtA5092	OtA5093-OtA5097
Rab31	chr17:65651725-65772752	OtA5088-OtA5092	OtA5093-OtA5097
Rnf168	chr16:32277460-32301439	OtA5088-OtA5092	OtA5093-OtA5097
Rnf2	chr1:151469405-151500823	OtA5088-OtA5092	OtA5093-OtA5097
Rtn1	chr12:72211751-72408939	OtA5088-OtA5092	OtA5093-OtA5097
Sertad1	chr7:27486952-27490314	OtA5088-OtA5092	OtA5093-OtA5097
Sord	chr2:122234838-122265337	OtA5088-OtA5092	OtA5093-OtA5097
Tln1	chr4:43531512-43562583	OtA5088-OtA5092	OtA5093-OtA5097
Tmx1	chr12:70244534-70669399	OtA5088-OtA5092	OtA5093-OtA5097
Yars	chr4:129189794-129248443	OtA5088-OtA5092	OtA5093-OtA5097
Zfp422	chr6:116624015-116628977	OtA5088-OtA5092	OtA5093-OtA5097
Cobra1	chr2:25199711-25211489	OtA5088-OtA5092	OtA5093-OtA5097
Adnp	chr2:168180964-168207062	OtA5088-OtA5092	OtA5093-OtA5097
Stat3	chr11:100886809-100939511	OtA5088-OtA5092	OtA5093-OtA5097
Kpna1	chr16:35983362-36036162	OtA5088-OtA5092	OtA5093-OtA5097
Prkar2a	chr9:108692142-108749511	OtA5088-OtA5092	OtA5093-OtA5097
Rgs2	chr1:143999337-144004149	OtA5088-OtA5092	OtA5093-OtA5097
Decr2	chr17:26081210-26090164	OtA5088-OtA5092	OtA5093-OtA5097
Insl6	chr19:29321353-29325318	OtA5088-OtA5092	OtA5093-OtA5097
Kctd18	chr1:57955100-57970084	OtA5088-OtA5092	OtA5093-OtA5097
Lmna	chr3:88481148-88503332	OtA5088-OtA5092	OtA5093-OtA5097
Prlr	chr15:10177237-10349180	OtA5088-OtA5092	OtA5093-OtA5097
Top1mt	chr15:75657032-75678790	OtA5088-OtA5092	OtA5093-OtA5097
Nfxl1	chr5:72513303-72559645	OtA5088-OtA5092	OtA5093-OtA5097
Celf1	chr2:90940396-91019497	OtA5088-OtA5092	OtA5093-OtA5097
Kit	chr5:75574986-75656721	OtA5088-OtA5092	OtA5093-OtA5097
Scnn1g	chr7:121734506-121768477	OtA5088-OtA5092	OtA5093-OtA5097
Snx24	chr18:53245661-53390825	OtA5088-OtA5092	OtA5093-OtA5097

Fndc4	chr5:31292245-31295877	OtA5088-OtA5092	OtA5093-OtA5097
Sepsecs	chr5:52643406-52669701	OtA5088-OtA5092	OtA5093-OtA5097
Rab5a	chr17:53479233-53539451	OtA5088-OtA5092	OtA5093-OtA5097
Cxx1a	chrX:53642488-53643763	OtA5088-OtA5092	OtA5093-OtA5097
Rfc5	chr5:117379144-117389023	OtA5088-OtA5092	OtA5093-OtA5097
Trmt112	chr19:6909697-6921808	OtA5088-OtA5092	OtA5093-OtA5097
Dcaf6	chr1:165329500-165460463	OtA5088-OtA5092	OtA5093-OtA5097
Apex2	chrX:150571506-150588149	OtA5088-OtA5092	OtA5093-OtA5097
Dcun1d2	chr8:13255962-13322924	OtA5088-OtA5092	OtA5093-OtA5097
Flt4	chr11:49609678-49652739	OtA5088-OtA5092	OtA5093-OtA5097
Hist1h1d	chr13:23555031-23558008	OtA5088-OtA5092	OtA5093-OtA5097
Kbtbd11	chr8:15011024-15033332	OtA5088-OtA5092	OtA5093-OtA5097
Klf7	chr1:64035670-64121389	OtA5088-OtA5092	OtA5093-OtA5097
Mpv17	chr5:31140660-31154242	OtA5088-OtA5092	OtA5093-OtA5097
Plod1	chr4:147909752-147936776	OtA5088-OtA5092	OtA5093-OtA5097
Ppp1cb	chr5:32458969-32493712	OtA5088-OtA5092	OtA5093-OtA5097
Rhbdd2	chr5:135632653-135646376	OtA5088-OtA5092	OtA5093-OtA5097
Slc24a6	chr5:120511191-120534024	OtA5088-OtA5092	OtA5093-OtA5097
Unk	chr11:116030321-116061194	OtA5088-OtA5092	OtA5093-OtA5097
Wdtc1	chr4:133292465-133339315	OtA5088-OtA5092	OtA5093-OtA5097
Zfp13	chr17:23575851-23599487	OtA5088-OtA5092	OtA5093-OtA5097
Gpr44	chr19:10937159-10949266	OtA5088-OtA5092	OtA5093-OtA5097
Apoa1	chr9:46228629-46230466	OtA5088-OtA5092	OtA5093-OtA5097
Btg2	chr1:134074864-134079155	OtA5088-OtA5092	OtA5093-OtA5097
Tti1	chr2:157981802-158009360	OtA5088-OtA5092	OtA5093-OtA5097
Pdcd11	chr19:47090765-47131145	OtA5088-OtA5092	OtA5093-OtA5097
Tbrg1	chr9:37649181-37657312	OtA5088-OtA5092	OtA5093-OtA5097
Ndufb6	chr4:40270662-40279368	OtA5088-OtA5092	OtA5093-OtA5097
Mrps23	chr11:88204417-88211507	OtA5088-OtA5092	OtA5093-OtA5097
Nptxr	chr15:79786350-79834333	OtA5088-OtA5092	OtA5093-OtA5097
Zc3hc1	chr6:30366387-30391010	OtA5088-OtA5092	OtA5093-OtA5097
Bhlhe41	chr6:145862736-145865340	OtA5088-OtA5092	OtA5093-OtA5097
Actr6	chr10:89711972-89732295	OtA5088-OtA5092	OtA5093-OtA5097
Bfar	chr16:13671857-13703612	OtA5088-OtA5092	OtA5093-OtA5097
Ccm2	chr11:6546886-6596761	OtA5088-OtA5092	OtA5093-OtA5097
Ceacam1	chr7:25379526-25566417	OtA5088-OtA5092	OtA5093-OtA5097
Cmas	chr6:142756685-142775714	OtA5088-OtA5092	OtA5093-OtA5097
Dhrs3	chr4:144892826-144927645	OtA5088-OtA5092	OtA5093-OtA5097
Foxp4	chr17:47867132-47924632	OtA5088-OtA5092	OtA5093-OtA5097
Polr2m	chr9:71478436-71485983	OtA5088-OtA5092	OtA5093-OtA5097
Slc14a1	chr18:78100090-78142119	OtA5088-OtA5092	OtA5093-OtA5097
Uqcrq	chr11:53428947-53430831	OtA5088-OtA5092	OtA5093-OtA5097
Wipf2	chr11:98863597-98905578	OtA5088-OtA5092	OtA5093-OtA5097
Zc3h13	chr14:75004109-75388146	OtA5088-OtA5092	OtA5093-OtA5097
Aamp	chr1:74279839-74284738	OtA5088-OtA5092	OtA5093-OtA5097
Rpl3	chr15:80077780-80083406	OtA5088-OtA5092	OtA5093-OtA5097
Zdhhc17	chr10:110941779-111010066	OtA5088-OtA5092	OtA5093-OtA5097

Rpl11	chr4:136049947-136053371	OtA5088-OtA5092	OtA5093-OtA5097
Anpep	chr7:79821802-79842352	OtA5088-OtA5092	OtA5093-OtA5097
Thumpd3	chr6:113046326-113077244	OtA5088-OtA5092	OtA5093-OtA5097
Dcp2	chr18:44380499-44424969	OtA5088-OtA5092	OtA5093-OtA5097
Erdr1	chrY:90785441-90816465	OtA5088-OtA5092	OtA5093-OtA5097
Ppig	chr2:69723087-69754059	OtA5088-OtA5092	OtA5093-OtA5097
Tank	chr2:61578585-61654169	OtA5088-OtA5092	OtA5093-OtA5097
Zbtb8a	chr4:129353631-129378028	OtA5088-OtA5092	OtA5093-OtA5097
Gramd2	chr9:59707763-59716424	OtA5088-OtA5092	OtA5093-OtA5097
Git2	chr5:114727407-114773492	OtA5088-OtA5092	OtA5093-OtA5097
Ndufs8	chr19:3908869-3912717	OtA5088-OtA5092	OtA5093-OtA5097
Taf1b	chr12:24498580-24558571	OtA5088-OtA5092	OtA5093-OtA5097
Lrrfip1	chr1:90998726-91128944	OtA5088-OtA5092	OtA5093-OtA5097
Dhrs1	chr14:55739019-55745684	OtA5088-OtA5092	OtA5093-OtA5097
Dnajc2	chr5:21757276-21785165	OtA5088-OtA5092	OtA5093-OtA5097
Secisbp2	chr13:51651713-51683646	OtA5088-OtA5092	OtA5093-OtA5097
Tff1	chr17:31161395-31165053	OtA5088-OtA5092	OtA5093-OtA5097
Ctsm	chr13:61536443-61541839	OtA5088-OtA5092	OtA5093-OtA5097
Fech	chr18:64456549-64489066	OtA5088-OtA5092	OtA5093-OtA5097
Zcchc4	chr5:52783066-52819765	OtA5088-OtA5092	OtA5093-OtA5097
Axin1	chr17:26138685-26195811	OtA5088-OtA5092	OtA5093-OtA5097
Daam1	chr12:71831077-71992367	OtA5088-OtA5092	OtA5093-OtA5097
Shc1	chr3:89418550-89430029	OtA5088-OtA5092	OtA5093-OtA5097
Tada1	chr1:166379166-166409828	OtA5088-OtA5092	OtA5093-OtA5097
Apoc4	chr7:19678093-19681416	OtA5088-OtA5092	OtA5093-OtA5097
Fam13a	chr6:58933535-59024502	OtA5088-OtA5092	OtA5093-OtA5097
Acin1	chr14:54642160-54690742	OtA5088-OtA5092	OtA5093-OtA5097
Ap4e1	chr2:127008710-127069814	OtA5088-OtA5092	OtA5093-OtA5097
Cc2d1a	chr8:84132827-84147753	OtA5088-OtA5092	OtA5093-OtA5097
D19Wsu162e	chr19:46599105-46657389	OtA5088-OtA5092	OtA5093-OtA5097
Dad1	chr14:54235484-54253929	OtA5088-OtA5092	OtA5093-OtA5097
Gm962	chr19:5568073-5571261	OtA5088-OtA5092	OtA5093-OtA5097
Mdh1	chr11:21556691-21571934	OtA5088-OtA5092	OtA5093-OtA5097
Psme2	chr14:55587439-55591101	OtA5088-OtA5092	OtA5093-OtA5097
Ripk4	chr16:97741941-97763755	OtA5088-OtA5092	OtA5093-OtA5097
Rsbn1l	chr5:20893023-20956398	OtA5088-OtA5092	OtA5093-OtA5097
Slc2a12	chr10:22645010-22731447	OtA5088-OtA5092	OtA5093-OtA5097
Tle6	chr10:81590904-81600900	OtA5088-OtA5092	OtA5093-OtA5097
Copg	chr6:87887939-87913594	OtA5088-OtA5092	OtA5093-OtA5097
Pgpep1	chr8:70646435-70659738	OtA5088-OtA5092	OtA5093-OtA5097
Sntb1	chr15:55639153-55906949	OtA5088-OtA5092	OtA5093-OtA5097
Crtc2	chr3:90254280-90264125	OtA5088-OtA5092	OtA5093-OtA5097
Golga3	chr5:110176700-110223155	OtA5088-OtA5092	OtA5093-OtA5097
Nbeal1	chr1:60180598-60334705	OtA5088-OtA5092	OtA5093-OtA5097
Srsf3	chr17:29032659-29043372	OtA5088-OtA5092	OtA5093-OtA5097
Ssb	chr2:69861561-69885604	OtA5088-OtA5092	OtA5093-OtA5097
Wrnip1	chr13:32802029-32822610	OtA5088-OtA5092	OtA5093-OtA5097

Zfp142	chr1:74409383-74588028	OtA5088-OtA5092	OtA5093-OtA5097
Acot7	chr4:152178099-152271855	OtA5088-OtA5092	OtA5093-OtA5097
Atxn3	chr12:101918900-101958243	OtA5088-OtA5092	OtA5093-OtA5097
Cdkn2d	chr9:21288463-21291209	OtA5088-OtA5092	OtA5093-OtA5097
Ddx26b	chrX:56454838-56507843	OtA5088-OtA5092	OtA5093-OtA5097
Dr1	chr5:108268896-108280521	OtA5088-OtA5092	OtA5093-OtA5097
Gpr1	chr1:63182690-63214209	OtA5088-OtA5092	OtA5093-OtA5097
Kirrel	chr3:87078591-87174747	OtA5088-OtA5092	OtA5093-OtA5097
Ltc4s	chr11:50236471-50238471	OtA5088-OtA5092	OtA5093-OtA5097
Tbc1d22a	chr15:86214458-86498503	OtA5088-OtA5092	OtA5093-OtA5097
Gyltl1b	chr2:92365045-92371036	OtA5088-OtA5092	OtA5093-OtA5097
Cdkn2aip	chr8:47709343-47713931	OtA5088-OtA5092	OtA5093-OtA5097
Tpi1	chr6:124808940-124814296	OtA5088-OtA5092	OtA5093-OtA5097
Vars2	chr17:35655634-35667592	OtA5088-OtA5092	OtA5093-OtA5097
Ccdc104	chr11:29221535-29247272	OtA5088-OtA5092	OtA5093-OtA5097
Ogfr	chr2:180589406-180595837	OtA5088-OtA5092	OtA5093-OtA5097
Rps12	chr10:23785182-23787209	OtA5088-OtA5092	OtA5093-OtA5097
Coq2	chr5:100654725-100674256	OtA5088-OtA5092	OtA5093-OtA5097
Dcbld2	chr16:58408534-58469745	OtA5088-OtA5092	OtA5093-OtA5097
Dusp22	chr13:30660095-30711225	OtA5088-OtA5092	OtA5093-OtA5097
Exosc9	chr3:36552605-36571996	OtA5088-OtA5092	OtA5093-OtA5097
Glrx	chr13:75839885-75850151	OtA5088-OtA5092	OtA5093-OtA5097
Mettl14	chr3:123368294-123385990	OtA5088-OtA5092	OtA5093-OtA5097
Mtx3	chr13:92844786-92858230	OtA5088-OtA5092	OtA5093-OtA5097
Phlda2	chr7:143501547-143502524	OtA5088-OtA5092	OtA5093-OtA5097
Ppm1k	chr6:57506501-57535426	OtA5088-OtA5092	OtA5093-OtA5097
Psma7	chr2:180036375-180042402	OtA5088-OtA5092	OtA5093-OtA5097
Sharpin	chr15:76347039-76351110	OtA5088-OtA5092	OtA5093-OtA5097
Ssfa2	chr2:79635424-79672964	OtA5088-OtA5092	OtA5093-OtA5097
St8sia2	chr7:73939119-74013682	OtA5088-OtA5092	OtA5093-OtA5097
Tsg101	chr7:46889026-46919930	OtA5088-OtA5092	OtA5093-OtA5097
Zfp385a	chr15:103313894-103340086	OtA5088-OtA5092	OtA5093-OtA5097
Actg1	chr11:120345689-120348484	OtA5088-OtA5092	OtA5093-OtA5097
Epor	chr9:21958898-21963576	OtA5088-OtA5092	OtA5093-OtA5097
Rpl5	chr5:107900527-107987077	OtA5088-OtA5092	OtA5093-OtA5097
Slc25a1	chr16:17925210-17928219	OtA5088-OtA5092	OtA5093-OtA5097
Vps41	chr13:18717291-18866811	OtA5088-OtA5092	OtA5093-OtA5097
Xrcc5	chr1:72307420-72394953	OtA5088-OtA5092	OtA5093-OtA5097
Papd7	chr13:69497958-69533864	OtA5088-OtA5092	OtA5093-OtA5097
Aifm2	chr10:61715262-61783864	OtA5088-OtA5092	OtA5093-OtA5097
Dicer1	chr12:104687741-104751952	OtA5088-OtA5092	OtA5093-OtA5097
Faf2	chr13:54621783-54664063	OtA5088-OtA5092	OtA5093-OtA5097
Kdm3b	chr18:34777007-34839370	OtA5088-OtA5092	OtA5093-OtA5097
Hira	chr16:18876749-18970308	OtA5088-OtA5092	OtA5093-OtA5097
Cdo1	chr18:46713204-46728342	OtA5088-OtA5092	OtA5093-OtA5097
Fam53a	chr5:33600352-33629635	OtA5088-OtA5092	OtA5093-OtA5097
Klk4	chr7:43881171-43885804	OtA5088-OtA5092	OtA5093-OtA5097

Arhgef3	chr14:27238038-27403904	OtA5088-OtA5092	OtA5093-OtA5097
Snrpn	chr7:59982500-60140219	OtA5088-OtA5092	OtA5093-OtA5097
Clock	chr5:76183879-76469519	OtA5088-OtA5092	OtA5093-OtA5097
Csrp2bp	chr2:144368982-144407675	OtA5088-OtA5092	OtA5093-OtA5097
Cstf2t	chr19:30564486-31765033	OtA5088-OtA5092	OtA5093-OtA5097
Dhx57	chr17:80238303-80297476	OtA5088-OtA5092	OtA5093-OtA5097
Dusp3	chr11:101971143-101984791	OtA5088-OtA5092	OtA5093-OtA5097
Athl1	chr7:140941580-140947658	OtA5088-OtA5092	OtA5093-OtA5097
Ucp2	chr7:100493344-100502173	OtA5088-OtA5092	OtA5093-OtA5097
Ddx20	chr3:105678461-105687571	OtA5088-OtA5092	OtA5093-OtA5097
Skiv2l	chr17:34839225-34850204	OtA5088-OtA5092	OtA5093-OtA5097
Nme1	chr11:93958924-93968521	OtA5088-OtA5092	OtA5093-OtA5097
Atg14	chr14:47540892-47568434	OtA5088-OtA5092	OtA5093-OtA5097
Crct1	chr3:93014204-93015686	OtA5088-OtA5092	OtA5093-OtA5097
Gnao1	chr8:93810837-93969388	OtA5088-OtA5092	OtA5093-OtA5097
Spag1	chr15:36179529-36235177	OtA5088-OtA5092	OtA5093-OtA5097
Wwox	chr8:114439651-115352712	OtA5088-OtA5092	OtA5093-OtA5097
Agpat6	chr8:23172945-23208453	OtA5088-OtA5092	OtA5093-OtA5097
Exoc6b	chr6:84618485-85069513	OtA5088-OtA5092	OtA5093-OtA5097
Gal3st1	chr11:3983635-3999328	OtA5088-OtA5092	OtA5093-OtA5097
Lactb	chr9:66955392-66975484	OtA5088-OtA5092	OtA5093-OtA5097
Mybbp1a	chr11:72441377-72451550	OtA5088-OtA5092	OtA5093-OtA5097
Nfkbil1	chr17:35220174-35235815	OtA5088-OtA5092	OtA5093-OtA5097
Nubp2	chr17:24882610-24886350	OtA5088-OtA5092	OtA5093-OtA5097
Pdzd2	chr15:12357053-12592556	OtA5088-OtA5092	OtA5093-OtA5097
Spryd3	chr15:102116527-102136215	OtA5088-OtA5092	OtA5093-OtA5097
Vat1	chr11:101458747-101466199	OtA5088-OtA5092	OtA5093-OtA5097
H2-Q7,H2-Q9	chr17:35439154-35443773	OtA5088-OtA5092	OtA5093-OtA5097
Npm3	chr19:45747733-45749563	OtA5088-OtA5092	OtA5093-OtA5097
Rab22a	chr2:173659844-173702182	OtA5088-OtA5092	OtA5093-OtA5097
Raver1	chr9:21067513-21092008	OtA5088-OtA5092	OtA5093-OtA5097
Ube2g2	chr10:77622320-77645990	OtA5088-OtA5092	OtA5093-OtA5097
Fgd3	chr13:49263109-49309208	OtA5088-OtA5092	OtA5093-OtA5097
Mdm2	chr10:117688904-117710716	OtA5088-OtA5092	OtA5093-OtA5097
Bod1	chr11:31665149-31671862	OtA5088-OtA5092	OtA5093-OtA5097
Cyb5b	chr8:107150660-107187470	OtA5088-OtA5092	OtA5093-OtA5097
Dcaf12	chr4:41291299-41314901	OtA5088-OtA5092	OtA5093-OtA5097
Lypla2	chr4:135968224-135972594	OtA5088-OtA5092	OtA5093-OtA5097
Ppp1r3b	chr8:35375740-35388137	OtA5088-OtA5092	OtA5093-OtA5097
BC013529	chr10:7767946-7780917	OtA5088-OtA5092	OtA5093-OtA5097
Ehmt2	chr17:34898498-34914048	OtA5088-OtA5092	OtA5093-OtA5097
Lsmd1	chr11:69395790-69398234	OtA5088-OtA5092	OtA5093-OtA5097
Lyrm2	chr4:32800258-32802254	OtA5088-OtA5092	OtA5093-OtA5097
Mfsd10	chr5:34633646-34637114	OtA5088-OtA5092	OtA5093-OtA5097
Ndufa13	chr8:69894181-69903518	OtA5088-OtA5092	OtA5093-OtA5097
Thbs1	chr2:118111921-118127133	OtA5088-OtA5092	OtA5093-OtA5097
Tmem54	chr4:129105529-129121740	OtA5088-OtA5092	OtA5093-OtA5097

Rrad	chr8:104628065-104631321	OtA5088-OtA5092	OtA5093-OtA5097
Hdac7	chr15:97784364-97844502	OtA5088-OtA5092	OtA5093-OtA5097
Adipor1	chr1:134415466-134432341	OtA5088-OtA5092	OtA5093-OtA5097
Arl1	chr10:88731413-88743202	OtA5088-OtA5092	OtA5093-OtA5097
B4galt1	chr4:40804601-40853998	OtA5088-OtA5092	OtA5093-OtA5097
Copz1	chr15:103272917-103299864	OtA5088-OtA5092	OtA5093-OtA5097
Cyp27a1	chr1:74713573-74737890	OtA5088-OtA5092	OtA5093-OtA5097
Dlat	chr9:50634632-50659780	OtA5088-OtA5092	OtA5093-OtA5097
Erc1	chr6:119570795-119848150	OtA5088-OtA5092	OtA5093-OtA5097
Inpp5k	chr11:75631019-75648865	OtA5088-OtA5092	OtA5093-OtA5097
Kbtbd2	chr6:56777524-56797813	OtA5088-OtA5092	OtA5093-OtA5097
Peg13	chr15:72589619-73061204	OtA5088-OtA5092	OtA5093-OtA5097
Pikfyve	chr1:65186684-65278696	OtA5088-OtA5092	OtA5093-OtA5097
Rbms1	chr2:60751952-60963204	OtA5088-OtA5092	OtA5093-OtA5097
Scara5	chr14:65666402-65764826	OtA5088-OtA5092	OtA5093-OtA5097
Siae	chr9:37613846-37648318	OtA5088-OtA5092	OtA5093-OtA5097
Zfp84	chr7:29768551-29781419	OtA5088-OtA5092	OtA5093-OtA5097
Fbn2	chr18:58008622-58209926	OtA5088-OtA5092	OtA5093-OtA5097
Recql5	chr11:115892594-115933492	OtA5088-OtA5092	OtA5093-OtA5097
Tomm40	chr7:19701312-19715429	OtA5088-OtA5092	OtA5093-OtA5097
Spata21	chr4:141088344-141112759	OtA5088-OtA5092	OtA5093-OtA5097
Eif4b	chr15:102073772-102097173	OtA5088-OtA5092	OtA5093-OtA5097
Aig1	chr10:13652708-13868830	OtA5088-OtA5092	OtA5093-OtA5097
C3	chr17:57203972-57228136	OtA5088-OtA5092	OtA5093-OtA5097
Chd8	chr14:52198150-52237572	OtA5088-OtA5092	OtA5093-OtA5097
Cyp4f17	chr17:32506461-32528894	OtA5088-OtA5092	OtA5093-OtA5097
Irf1	chr11:53770013-53778374	OtA5088-OtA5092	OtA5093-OtA5097
Mif4gd	chr11:115607918-115612969	OtA5088-OtA5092	OtA5093-OtA5097
Rnpc3	chr3:113605066-113630149	OtA5088-OtA5092	OtA5093-OtA5097
Rpl7a	chr2:26910806-26913311	OtA5088-OtA5092	OtA5093-OtA5097
Socs1	chr16:10783808-10785536	OtA5088-OtA5092	OtA5093-OtA5097
Xpot	chr10:121587379-121626316	OtA5088-OtA5092	OtA5093-OtA5097
Lman2l	chr1:36423185-36445239	OtA5088-OtA5092	OtA5093-OtA5097
Palld	chr8:61515020-61902690	OtA5088-OtA5092	OtA5093-OtA5097
Fnip1	chr11:54438178-54518241	OtA5088-OtA5092	OtA5093-OtA5097
Sod2	chr17:13007838-13018119	OtA5088-OtA5092	OtA5093-OtA5097
Tmem139	chr6:42261969-42264555	OtA5088-OtA5092	OtA5093-OtA5097
Nol8	chr13:49653349-49679015	OtA5088-OtA5092	OtA5093-OtA5097
Msl1	chr11:98795768-98833807	OtA5088-OtA5092	OtA5093-OtA5097
Tbrg4	chr11:6615597-6626067	OtA5088-OtA5092	OtA5093-OtA5097
Ap1s1	chr5:137034993-137046060	OtA5088-OtA5092	OtA5093-OtA5097
Ezr	chr17:6738130-6782780	OtA5088-OtA5092	OtA5093-OtA5097
Grtp1	chr8:13176868-13200624	OtA5088-OtA5092	OtA5093-OtA5097
Hyal1	chr9:107576951-107580133	OtA5088-OtA5092	OtA5093-OtA5097
Idh1	chr1:65158615-65186479	OtA5088-OtA5092	OtA5093-OtA5097
Thrap3	chr4:126164082-126202710	OtA5088-OtA5092	OtA5093-OtA5097
Arl6ip6	chr2:53192083-53219221	OtA5088-OtA5092	OtA5093-OtA5097

Atp6v0a1	chr11:101009451-101063717	OtA5088-OtA5092	OtA5093-OtA5097
Ifnar2	chr16:91372782-91405587	OtA5088-OtA5092	OtA5093-OtA5097
Lfng	chr5:140607340-140615545	OtA5088-OtA5092	OtA5093-OtA5097
Mfsd6	chr1:52656304-52727318	OtA5088-OtA5092	OtA5093-OtA5097
Paqr8	chr1:20890621-20938756	OtA5088-OtA5092	OtA5093-OtA5097
Pfdn5	chr15:102326115-102331489	OtA5088-OtA5092	OtA5093-OtA5097
Rffl	chr11:82781134-82871210	OtA5088-OtA5092	OtA5093-OtA5097
Stx16	chr2:174077050-174099771	OtA5088-OtA5092	OtA5093-OtA5097
Supv3l1	chr10:62429377-62451353	OtA5088-OtA5092	OtA5093-OtA5097
Tbx4	chr11:85890062-85916097	OtA5088-OtA5092	OtA5093-OtA5097
Ttc33	chr15:5185559-5218332	OtA5088-OtA5092	OtA5093-OtA5097
Usp37	chr1:74409383-74588028	OtA5088-OtA5092	OtA5093-OtA5097
Tnip1	chr11:54910786-54962940	OtA5088-OtA5092	OtA5093-OtA5097
Mmp10	chr9:7502359-7510240	OtA5088-OtA5092	OtA5093-OtA5097
Actn1	chr12:80167541-80260371	OtA5088-OtA5092	OtA5093-OtA5097
Alg10b	chr15:90224310-90230554	OtA5088-OtA5092	OtA5093-OtA5097
B3gnt1	chr19:5038825-5041134	OtA5088-OtA5092	OtA5093-OtA5097
D6Wsu116e	chr6:116208032-116262671	OtA5088-OtA5092	OtA5093-OtA5097
Dnttip2	chr3:122274413-122285271	OtA5088-OtA5092	OtA5093-OtA5097
E2f1	chr2:154559638-154569852	OtA5088-OtA5092	OtA5093-OtA5097
Enpp2	chr15:54838897-54920146	OtA5088-OtA5092	OtA5093-OtA5097
Gigyf1	chr5:137518879-137527935	OtA5088-OtA5092	OtA5093-OtA5097
Gorasp2	chr2:70661508-70691725	OtA5088-OtA5092	OtA5093-OtA5097
Ilf3	chr9:21368002-21411720	OtA5088-OtA5092	OtA5093-OtA5097
Mapkapk5	chr5:121525050-121545892	OtA5088-OtA5092	OtA5093-OtA5097
Pcbp2	chr15:102470631-102517004	OtA5088-OtA5092	OtA5093-OtA5097
Phf16	chrX:20460526-20517896	OtA5088-OtA5092	OtA5093-OtA5097
Scarf1	chr11:75513540-75526580	OtA5088-OtA5092	OtA5093-OtA5097
Srxn1	chr2:152105729-152111353	OtA5088-OtA5092	OtA5093-OtA5097
St13	chr15:81365043-81399694	OtA5088-OtA5092	OtA5093-OtA5097
Ubash3b	chr9:41013640-41157494	OtA5088-OtA5092	OtA5093-OtA5097
Uevld	chr7:46923215-46958518	OtA5088-OtA5092	OtA5093-OtA5097
Usp35	chr7:97309379-97325964	OtA5088-OtA5092	OtA5093-OtA5097
Tmem59	chr4:107178629-107200996	OtA5088-OtA5092	OtA5093-OtA5097
Fcna	chr2:25624666-25627974	OtA5088-OtA5092	OtA5093-OtA5097
Wdr13	chrX:8123300-8132858	OtA5088-OtA5092	OtA5093-OtA5097
Exosc7	chr9:123113230-123136129	OtA5088-OtA5092	OtA5093-OtA5097
Parg	chr14:32201970-32297549	OtA5088-OtA5092	OtA5093-OtA5097
Tm9sf3	chr19:41210841-41264004	OtA5088-OtA5092	OtA5093-OtA5097
D9Ertd402e	chr9:122805546-122836332	OtA5088-OtA5092	OtA5093-OtA5097
Fut10	chr8:31187331-31261483	OtA5088-OtA5092	OtA5093-OtA5097
Lin54	chr5:100442034-100500639	OtA5088-OtA5092	OtA5093-OtA5097
Manea	chr4:26324505-26346652	OtA5088-OtA5092	OtA5093-OtA5097
Micall2	chr5:139706692-139736333	OtA5088-OtA5092	OtA5093-OtA5097
Mypop	chr7:18991244-19001766	OtA5088-OtA5092	OtA5093-OtA5097
Npepps	chr11:97205855-97280576	OtA5088-OtA5092	OtA5093-OtA5097
Phb	chr11:95666956-95680773	OtA5088-OtA5092	OtA5093-OtA5097

Pex10	chr4:155067029-155072406	OtA5088-OtA5092	OtA5093-OtA5097
Ero1lb	chr13:12565882-12609528	OtA5088-OtA5092	OtA5093-OtA5097
Yeats4	chr10:117215140-117224507	OtA5088-OtA5092	OtA5093-OtA5097
Entpd6	chr2:150749080-150771674	OtA5088-OtA5092	OtA5093-OtA5097
Trim68	chr7:102677579-102687326	OtA5088-OtA5092	OtA5093-OtA5097
Bysl	chr17:47505050-47611492	OtA5088-OtA5092	OtA5093-OtA5097
Cables2	chr2:180258538-180273465	OtA5088-OtA5092	OtA5093-OtA5097
Wfdc1	chr8:119666364-119688020	OtA5088-OtA5092	OtA5093-OtA5097
Qpctl	chr7:19140216-19149196	OtA5088-OtA5092	OtA5093-OtA5097
Vamp3	chr4:151047304-151057953	OtA5088-OtA5092	OtA5093-OtA5097
Cd74	chr18:60803848-60812652	OtA5088-OtA5092	OtA5093-OtA5097
Hist3h2a	chr11:58954684-58956674	OtA5088-OtA5092	OtA5093-OtA5097
Clec12b	chr6:129375512-129385874	OtA5088-OtA5092	OtA5093-OtA5097
Gmpr2	chr14:55672234-55678751	OtA5088-OtA5092	OtA5093-OtA5097
Znf512b	chr2:181569152-181592461	OtA5088-OtA5092	OtA5093-OtA5097
Tomm7	chr5:23787743-24161231	OtA5088-OtA5092	OtA5093-OtA5097
Tbc1d2b	chr9:90202048-90270769	OtA5088-OtA5092	OtA5093-OtA5097
Epn2	chr11:61517248-61579687	OtA5088-OtA5092	OtA5093-OtA5097
Gnl2	chr4:125030013-125065657	OtA5088-OtA5092	OtA5093-OtA5097
Ipo7	chr7:110018424-110055114	OtA5088-OtA5092	OtA5093-OtA5097
Mmp19	chr10:128790909-128804370	OtA5088-OtA5092	OtA5093-OtA5097
Rab1	chr11:20201601-20226856	OtA5088-OtA5092	OtA5093-OtA5097
Tardbp	chr4:148602543-148626996	OtA5088-OtA5092	OtA5093-OtA5097
Tpp2	chr1:43934006-44002971	OtA5088-OtA5092	OtA5093-OtA5097
Zfp518a	chr19:40894704-40917947	OtA5088-OtA5092	OtA5093-OtA5097
Zfp532	chr18:65580229-65689436	OtA5088-OtA5092	OtA5093-OtA5097
Cpsf7	chr19:10525243-10547735	OtA5088-OtA5092	OtA5093-OtA5097
Csgalnact1	chr8:68356780-68735146	OtA5088-OtA5092	OtA5093-OtA5097
Myo18a	chr11:77777264-77865980	OtA5088-OtA5092	OtA5093-OtA5097
Klf14	chr6:30956020-30958990	OtA5088-OtA5092	OtA5093-OtA5097
Upk3b	chr5:136038495-136044993	OtA5088-OtA5092	OtA5093-OtA5097
Coq5	chr5:115279701-115296972	OtA5088-OtA5092	OtA5093-OtA5097
Ddx39	chr8:83715176-83741311	OtA5088-OtA5092	OtA5093-OtA5097
Gm10664	chr8:65028416-65037336	OtA5088-OtA5092	OtA5093-OtA5097
Mrpl9	chr3:94443335-94448708	OtA5088-OtA5092	OtA5093-OtA5097
Fan1	chr7:64346757-64374095	OtA5088-OtA5092	OtA5093-OtA5097
Gpc2	chr5:138273659-138279937	OtA5088-OtA5092	OtA5093-OtA5097
Lonp2	chr8:86624042-86716638	OtA5088-OtA5092	OtA5093-OtA5097
Ppp2r3a	chr9:101104988-101251832	OtA5088-OtA5092	OtA5093-OtA5097
Rnf10	chr5:115241769-115272895	OtA5088-OtA5092	OtA5093-OtA5097
Oas1g	chr5:120876141-120887598	OtA5088-OtA5092	OtA5093-OtA5097
Drd4	chr7:141292005-141295001	OtA5088-OtA5092	OtA5093-OtA5097
Mrpl35	chr6:71812996-71823784	OtA5088-OtA5092	OtA5093-OtA5097
Wdr25	chr12:108894271-109028452	OtA5088-OtA5092	OtA5093-OtA5097
Adam17	chr12:21316391-21373591	OtA5088-OtA5092	OtA5093-OtA5097
Mdh2	chr5:135778648-135790386	OtA5088-OtA5092	OtA5093-OtA5097
Zbtb26	chr2:37432167-37443121	OtA5088-OtA5092	OtA5093-OtA5097

Zfp790	chr7:29816071-29832042	OtA5088-OtA5092	OtA5093-OtA5097
Coq10a	chr10:128363096-128370037	OtA5088-OtA5092	OtA5093-OtA5097
Gclm	chr3:122245591-122266648	OtA5088-OtA5092	OtA5093-OtA5097
Ammecr1l	chr18:31759823-31784083	OtA5088-OtA5092	OtA5093-OtA5097
Apoe	chr7:19696243-19699166	OtA5088-OtA5092	OtA5093-OtA5097
Cdk1	chr10:69336634-69352912	OtA5088-OtA5092	OtA5093-OtA5097
Cyp4f13	chr17:32924687-32947361	OtA5088-OtA5092	OtA5093-OtA5097
Dcaf13	chr15:39112873-39146855	OtA5088-OtA5092	OtA5093-OtA5097
Dlg4	chr11:70018604-70045531	OtA5088-OtA5092	OtA5093-OtA5097
Mrpl16	chr19:11770414-11774946	OtA5088-OtA5092	OtA5093-OtA5097
Pi4k2a	chr19:42090434-42122218	OtA5088-OtA5092	OtA5093-OtA5097
Plxdc2	chr2:16356321-16752115	OtA5088-OtA5092	OtA5093-OtA5097
Ufm1	chr3:53853375-53863807	OtA5088-OtA5092	OtA5093-OtA5097
Hmgb3	chrX:71555992-71560654	OtA5088-OtA5092	OtA5093-OtA5097
Hspe1	chr1:55088147-55091317	OtA5088-OtA5092	OtA5093-OtA5097
Krcc1	chr6:71272018-71285319	OtA5088-OtA5092	OtA5093-OtA5097
Polr2j	chr5:136116690-136122947	OtA5088-OtA5092	OtA5093-OtA5097
Tmem208	chr8:105326363-105329057	OtA5088-OtA5092	OtA5093-OtA5097
Hdac2	chr10:36974543-37001888	OtA5088-OtA5092	OtA5093-OtA5097
Tmem173	chr18:35733677-35740554	OtA5088-OtA5092	OtA5093-OtA5097
Hbb-b2	chr7:103812523-103813923	OtA5088-OtA5092	OtA5093-OtA5097
Ncf1	chr5:134220259-134229625	OtA5088-OtA5092	OtA5093-OtA5097
Fcgr4	chr1:171018925-171029761	OtA5088-OtA5092	OtA5093-OtA5097
Ndufa1	chrX:37187588-37191238	OtA5088-OtA5092	OtA5093-OtA5097
Tox2	chr2:163225453-163323102	OtA5088-OtA5092	OtA5093-OtA5097
Dhx33	chr11:70984090-71004432	OtA5088-OtA5092	OtA5093-OtA5097
Eed	chr7:89954653-89980976	OtA5088-OtA5092	OtA5093-OtA5097
Mef2d	chr3:88142394-88169167	OtA5088-OtA5092	OtA5093-OtA5097
Tspan15	chr10:62185395-62231218	OtA5088-OtA5092	OtA5093-OtA5097
Ino80b	chr6:83121827-83125029	OtA5088-OtA5092	OtA5093-OtA5097
Ttll5	chr12:85824949-86053760	OtA5088-OtA5092	OtA5093-OtA5097
Snhg12	chr4:132308677-132311024	OtA5088-OtA5092	OtA5093-OtA5097
Slc38a4	chr15:96994822-97055956	OtA5088-OtA5092	OtA5093-OtA5097
Tie1	chr4:118471190-118489849	OtA5088-OtA5092	OtA5093-OtA5097
Cul9	chr17:46500604-46546388	OtA5088-OtA5092	OtA5093-OtA5097
Dnajc13	chr9:104151596-104262930	OtA5088-OtA5092	OtA5093-OtA5097
Fam65a	chr8:105605228-105622218	OtA5088-OtA5092	OtA5093-OtA5097
Mettl15	chr2:109092299-109278290	OtA5088-OtA5092	OtA5093-OtA5097
Mri1	chr8:84250575-84257324	OtA5088-OtA5092	OtA5093-OtA5097
Prl3b1	chr13:27242429-27249740	OtA5088-OtA5092	OtA5093-OtA5097
Mlx	chr11:101087289-101095435	OtA5088-OtA5092	OtA5093-OtA5097
Mrpl36	chr13:73331008-73332178	OtA5088-OtA5092	OtA5093-OtA5097
Sgk3	chr1:9798129-9902568	OtA5088-OtA5092	OtA5093-OtA5097
Tmem60	chr5:20882452-20886870	OtA5088-OtA5092	OtA5093-OtA5097
Mettl8	chr2:70964561-71055606	OtA5088-OtA5092	OtA5093-OtA5097
Tmem165	chr5:76183879-76469519	OtA5088-OtA5092	OtA5093-OtA5097
Uba7	chr9:107975566-107984056	OtA5088-OtA5092	OtA5093-OtA5097

Ppp2cb	chr8:33599620-33641976	OtA5088-OtA5092	OtA5093-OtA5097
B2m	chr2:122120107-122186189	OtA5088-OtA5092	OtA5093-OtA5097
B3galnt1	chr3:69574185-69598861	OtA5088-OtA5092	OtA5093-OtA5097
Klhl24	chr16:20097553-20127744	OtA5088-OtA5092	OtA5093-OtA5097
Liph	chr16:21953817-21995542	OtA5088-OtA5092	OtA5093-OtA5097
Nop2	chr6:125131882-125144753	OtA5088-OtA5092	OtA5093-OtA5097
Notch4	chr17:34564294-34588543	OtA5088-OtA5092	OtA5093-OtA5097
Olfm1	chr2:28193092-28230736	OtA5088-OtA5092	OtA5093-OtA5097
Pla2g15	chr8:106150398-106164715	OtA5088-OtA5092	OtA5093-OtA5097
Scyl2	chr10:89640106-89686285	OtA5088-OtA5092	OtA5093-OtA5097
Sh2d3c	chr2:32721054-32755007	OtA5088-OtA5092	OtA5093-OtA5097
Tmem125	chr4:118540940-118543726	OtA5088-OtA5092	OtA5093-OtA5097
Tmem178	chr17:80944631-81001816	OtA5088-OtA5092	OtA5093-OtA5097
Tmem30b	chr12:73543113-73546395	OtA5088-OtA5092	OtA5093-OtA5097
Cbx1	chr11:96789135-96808640	OtA5088-OtA5092	OtA5093-OtA5097
Csad	chr15:102176997-102189043	OtA5088-OtA5092	OtA5093-OtA5097
Fhl2	chr1:43123073-43163961	OtA5088-OtA5092	OtA5093-OtA5097
Med15	chr16:17651220-17722930	OtA5088-OtA5092	OtA5093-OtA5097
Elmod3	chr6:72565921-72598413	OtA5088-OtA5092	OtA5093-OtA5097
Cryz	chr3:154576519-154623182	OtA5088-OtA5092	OtA5093-OtA5097
Deb1	chr9:121710388-121712921	OtA5088-OtA5092	OtA5093-OtA5097
Fam50a	chrX:74313032-74320149	OtA5088-OtA5092	OtA5093-OtA5097
Lcmt2	chr2:121137291-121140698	OtA5088-OtA5092	OtA5093-OtA5097
Zfp295	chr16:97947434-97962621	OtA5088-OtA5092	OtA5093-OtA5097
Chdh	chr14:30008999-30527056	OtA5088-OtA5092	OtA5093-OtA5097
Cops5	chr1:10024830-10037898	OtA5088-OtA5092	OtA5093-OtA5097
Fam48a	chr3:54693104-54716837	OtA5088-OtA5092	OtA5093-OtA5097
Fdx1l	chr9:21067513-21092008	OtA5088-OtA5092	OtA5093-OtA5097
Grpel1	chr5:36465184-36484285	OtA5088-OtA5092	OtA5093-OtA5097
Haus7	chrX:73437314-73459029	OtA5088-OtA5092	OtA5093-OtA5097
Lrrc58	chr16:37868399-37888857	OtA5088-OtA5092	OtA5093-OtA5097
Mex3c	chr18:73572704-73592578	OtA5088-OtA5092	OtA5093-OtA5097
Pkd2	chr5:104459456-104505819	OtA5088-OtA5092	OtA5093-OtA5097
Sart1	chr19:5377522-5390069	OtA5088-OtA5092	OtA5093-OtA5097
Scn1b	chr7:31116523-31126945	OtA5088-OtA5092	OtA5093-OtA5097
Tbc1d15	chr10:115197870-115251493	OtA5088-OtA5092	OtA5093-OtA5097
Trim26	chr17:36837140-36859380	OtA5088-OtA5092	OtA5093-OtA5097
Tusc1	chr4:93334147-93335511	OtA5088-OtA5092	OtA5093-OtA5097
Zfp316	chr5:143249694-143270022	OtA5088-OtA5092	OtA5093-OtA5097
Ank	chr15:27466676-27594907	OtA5088-OtA5092	OtA5093-OtA5097
Gm6793	chr2:75659258-75669407	OtA5088-OtA5092	OtA5093-OtA5097
Irx3	chr8:91798510-91801654	OtA5088-OtA5092	OtA5093-OtA5097
Mgat4b	chr11:50225334-50235103	OtA5088-OtA5092	OtA5093-OtA5097
Ppib	chr9:66060168-66069731	OtA5088-OtA5092	OtA5093-OtA5097
Triobp	chr15:78947723-79011041	OtA5088-OtA5092	OtA5093-OtA5097
Ubtd2	chr11:32455371-32518709	OtA5088-OtA5092	OtA5093-OtA5097
BC020535	chr2:153066001-153081735	OtA5088-OtA5092	OtA5093-OtA5097

Ehbp1	chr11:22005825-22286795	OtA5088-OtA5092	OtA5093-OtA5097
Birc6	chr17:74528294-74703773	OtA5088-OtA5092	OtA5093-OtA5097
Gpr180	chr14:118137126-118164232	OtA5088-OtA5092	OtA5093-OtA5097
Stk31	chr6:49395603-49469502	OtA5088-OtA5092	OtA5093-OtA5097
Bsdc1	chr4:129461678-129488432	OtA5088-OtA5092	OtA5093-OtA5097
Cep164	chr9:45766945-45828638	OtA5088-OtA5092	OtA5093-OtA5097
Pck2	chr14:55540265-55550017	OtA5088-OtA5092	OtA5093-OtA5097
Pla2g2e	chr4:138877941-138882814	OtA5088-OtA5092	OtA5093-OtA5097
Ublcp1	chr11:44454570-44470548	OtA5088-OtA5092	OtA5093-OtA5097
Heatr3	chr8:88137884-88171943	OtA5088-OtA5092	OtA5093-OtA5097
Prl2c2	chr13:12996123-13005330	OtA5088-OtA5092	OtA5093-OtA5097
Slc1a5	chr7:16781345-16798274	OtA5088-OtA5092	OtA5093-OtA5097
Tmem9	chr1:136008218-136035030	OtA5088-OtA5092	OtA5093-OtA5097
Ankrd52	chr10:128377123-128394006	OtA5088-OtA5092	OtA5093-OtA5097
Cnnm4	chr1:36471596-36508776	OtA5088-OtA5092	OtA5093-OtA5097
Col5a1	chr2:27886424-28039510	OtA5088-OtA5092	OtA5093-OtA5097
Dguok	chr6:83480213-83506969	OtA5088-OtA5092	OtA5093-OtA5097
Dip2c	chr13:9276524-9668926	OtA5088-OtA5092	OtA5093-OtA5097
Dtx3l	chr16:35926514-35972621	OtA5088-OtA5092	OtA5093-OtA5097
Ethe1	chr7:24583837-24608926	OtA5088-OtA5092	OtA5093-OtA5097
Fbrsl1	chr5:110361751-110448503	OtA5088-OtA5092	OtA5093-OtA5097
Hspg2	chr4:137468802-137570630	OtA5088-OtA5092	OtA5093-OtA5097
Ift88	chr14:57424070-57517936	OtA5088-OtA5092	OtA5093-OtA5097
Nadsyn1	chr7:143795593-143822841	OtA5088-OtA5092	OtA5093-OtA5097
Odf2l	chr3:145118588-145153915	OtA5088-OtA5092	OtA5093-OtA5097
Sash1	chr10:8722218-8886070	OtA5088-OtA5092	OtA5093-OtA5097
Snrpd1	chr18:10617795-10628230	OtA5088-OtA5092	OtA5093-OtA5097
Sp3	chr2:72936431-72989249	OtA5088-OtA5092	OtA5093-OtA5097
Sumo3	chr10:77606233-77618331	OtA5088-OtA5092	OtA5093-OtA5097
Tead4	chr6:128227142-128300813	OtA5088-OtA5092	OtA5093-OtA5097
Tmod3	chr9:75497783-75559657	OtA5088-OtA5092	OtA5093-OtA5097
Tut1	chr19:8953849-8966210	OtA5088-OtA5092	OtA5093-OtA5097
Usp25	chr16:77014068-77116780	OtA5088-OtA5092	OtA5093-OtA5097
Chst14	chr2:118926496-118928583	OtA5088-OtA5092	OtA5093-OtA5097
Klhl13	chrX:23219270-23365053	OtA5088-OtA5092	OtA5093-OtA5097
Nckap5l	chr15:99422033-99457748	OtA5088-OtA5092	OtA5093-OtA5097
Ttc37	chr13:76098733-76187983	OtA5088-OtA5092	OtA5093-OtA5097
Mtmr11	chr3:96161969-96171718	OtA5088-OtA5092	OtA5093-OtA5097
Cdk5r1	chr11:80477045-80481179	OtA5088-OtA5092	OtA5093-OtA5097
Ifrd2	chr9:107587717-107593038	OtA5088-OtA5092	OtA5093-OtA5097
S100a11	chr3:93520495-93526288	OtA5088-OtA5092	OtA5093-OtA5097
Tfip11	chr5:112326368-112343040	OtA5088-OtA5092	OtA5093-OtA5097
Acyp1	chr12:85272397-85280435	OtA5088-OtA5092	OtA5093-OtA5097
Cdk5	chr5:24418241-24423530	OtA5088-OtA5092	OtA5093-OtA5097
Clec2d	chr6:129180614-129186535	OtA5088-OtA5092	OtA5093-OtA5097
Htt	chr5:34761739-34912521	OtA5088-OtA5092	OtA5093-OtA5097
Rhbdd3	chr11:5099437-5106091	OtA5088-OtA5092	OtA5093-OtA5097

Sowahb	chr5:93041122-93045022	OtA5088-OtA5092	OtA5093-OtA5097
Fam110b	chr4:5644178-5799944	OtA5088-OtA5092	OtA5093-OtA5097
Pdzd8	chr19:59296083-59345780	OtA5088-OtA5092	OtA5093-OtA5097
Rbfox2	chr15:77078989-77307053	OtA5088-OtA5092	OtA5093-OtA5097
Tusc5	chr11:76679872-76698664	OtA5088-OtA5092	OtA5093-OtA5097
Eno1,Gm5506	chr4:150237196-150248873	OtA5088-OtA5092	OtA5093-OtA5097
Arhgef40	chr14:51984832-52020733	OtA5088-OtA5092	OtA5093-OtA5097
Ercc4	chr16:13109735-13152009	OtA5088-OtA5092	OtA5093-OtA5097
Rab27a	chr9:73044864-73097592	OtA5088-OtA5092	OtA5093-OtA5097
Sec14l2	chr11:4097039-4118729	OtA5088-OtA5092	OtA5093-OtA5097
Abi1	chr2:22895521-23040240	OtA5088-OtA5092	OtA5093-OtA5097
Ddx6	chr9:44604891-44640731	OtA5088-OtA5092	OtA5093-OtA5097
Mrps6	chr16:92058321-92112227	OtA5088-OtA5092	OtA5093-OtA5097
Mrps7	chr11:115604150-115607624	OtA5088-OtA5092	OtA5093-OtA5097
Nfatc4	chr14:55824794-55833943	OtA5088-OtA5092	OtA5093-OtA5097
Nr2f2	chr7:70351949-70411146	OtA5088-OtA5092	OtA5093-OtA5097
Osbpl1a	chr18:12755311-12941841	OtA5088-OtA5092	OtA5093-OtA5097
Rbp1	chr9:98422960-98446550	OtA5088-OtA5092	OtA5093-OtA5097
Rexo2	chr9:48468513-48480611	OtA5088-OtA5092	OtA5093-OtA5097
Rnf170	chr8:26119379-26143869	OtA5088-OtA5092	OtA5093-OtA5097
Sema3b	chr9:107598114-107609241	OtA5088-OtA5092	OtA5093-OtA5097
Traf6	chr2:101678439-101700974	OtA5088-OtA5092	OtA5093-OtA5097
Txndc5	chr13:38500274-38528460	OtA5088-OtA5092	OtA5093-OtA5097
Vps35	chr8:85260391-85299497	OtA5088-OtA5092	OtA5093-OtA5097
Zic3	chrX:58030627-58036630	OtA5088-OtA5092	OtA5093-OtA5097
Dclre1a	chr19:56529160-56548222	OtA5088-OtA5092	OtA5093-OtA5097
Dido1	chr2:180657963-180709999	OtA5088-OtA5092	OtA5093-OtA5097
Fam132a	chr4:155962311-155966629	OtA5088-OtA5092	OtA5093-OtA5097
Snurf	chr7:59982500-60140219	OtA5088-OtA5092	OtA5093-OtA5097
Acn9	chr6:6956017-7039220	OtA5088-OtA5092	OtA5093-OtA5097
Airn	chr17:12682405-12868143	OtA5088-OtA5092	OtA5093-OtA5097
Dennd5a	chr7:109893780-109960422	OtA5088-OtA5092	OtA5093-OtA5097
Eif3b	chr5:140419304-140443358	OtA5088-OtA5092	OtA5093-OtA5097
Fads3	chr19:10041547-10059671	OtA5088-OtA5092	OtA5093-OtA5097
Gas6	chr8:13465373-13494535	OtA5088-OtA5092	OtA5093-OtA5097
Rps27l	chr9:66946117-66949509	OtA5088-OtA5092	OtA5093-OtA5097
Rsad1	chr11:94539797-94549207	OtA5088-OtA5092	OtA5093-OtA5097
Slc9a6	chrX:56609834-56664230	OtA5088-OtA5092	OtA5093-OtA5097
Rabggta	chr14:55715876-55722176	OtA5088-OtA5092	OtA5093-OtA5097
Trpv6	chr6:41620618-41636405	OtA5088-OtA5092	OtA5093-OtA5097
Cog1	chr11:113649528-113684151	OtA5088-OtA5092	OtA5093-OtA5097
Acp2	chr2:91202911-91237066	OtA5088-OtA5092	OtA5093-OtA5097
Atic	chr1:71557155-71579403	OtA5088-OtA5092	OtA5093-OtA5097
Cela1	chr15:100674421-100687920	OtA5088-OtA5092	OtA5093-OtA5097
Cotl1	chr8:119809213-119840579	OtA5088-OtA5092	OtA5093-OtA5097
Dis3l2	chr1:86703803-87050097	OtA5088-OtA5092	OtA5093-OtA5097
Gmfb	chr14:46808148-46822242	OtA5088-OtA5092	OtA5093-OtA5097

Gps2	chr11:69914191-69916591	OtA5088-OtA5092	OtA5093-OtA5097
Heatr1	chr13:12395374-12438893	OtA5088-OtA5092	OtA5093-OtA5097
Hook2	chr8:84990594-85003364	OtA5088-OtA5092	OtA5093-OtA5097
Lxn	chr3:67430114-67475068	OtA5088-OtA5092	OtA5093-OtA5097
Nin	chr12:70011434-70111925	OtA5088-OtA5092	OtA5093-OtA5097
Oxa1l	chr14:54360840-54417702	OtA5088-OtA5092	OtA5093-OtA5097
Ppp1r18	chr17:35865594-35875596	OtA5088-OtA5092	OtA5093-OtA5097
Rars2	chr4:34614957-34660167	OtA5088-OtA5092	OtA5093-OtA5097
Tceb3	chr4:136003369-136021649	OtA5088-OtA5092	OtA5093-OtA5097
Usp40	chr1:87945120-88008551	OtA5088-OtA5092	OtA5093-OtA5097
Zbtb2	chr10:4367073-4388108	OtA5088-OtA5092	OtA5093-OtA5097
Zfp106	chr2:120506829-120563831	OtA5088-OtA5092	OtA5093-OtA5097
Zfp42	chr8:43295066-43307009	OtA5088-OtA5092	OtA5093-OtA5097
Cd82	chr2:93419111-93469874	OtA5088-OtA5092	OtA5093-OtA5097
Megf6	chr4:154170712-154275721	OtA5088-OtA5092	OtA5093-OtA5097
Hal	chr10:93488767-93516743	OtA5088-OtA5092	OtA5093-OtA5097
Psmg4	chr13:34162976-34178172	OtA5088-OtA5092	OtA5093-OtA5097
Rgmb	chr17:15806252-15826586	OtA5088-OtA5092	OtA5093-OtA5097
Adm	chr7:110627668-110629819	OtA5088-OtA5092	OtA5093-OtA5097
Bcap29	chr12:31595353-31634658	OtA5088-OtA5092	OtA5093-OtA5097
Eif2s2	chr2:154871409-154892906	OtA5088-OtA5092	OtA5093-OtA5097
Eif4a3	chr11:119288362-119300043	OtA5088-OtA5092	OtA5093-OtA5097
Enah	chr1:181904444-182019980	OtA5088-OtA5092	OtA5093-OtA5097
Fam125b	chr2:33729955-33887946	OtA5088-OtA5092	OtA5093-OtA5097
Fam98a	chr17:75537085-75551946	OtA5088-OtA5092	OtA5093-OtA5097
Hunk	chr16:90386396-90499553	OtA5088-OtA5092	OtA5093-OtA5097
Pitpnc1	chr11:107207891-107470720	OtA5088-OtA5092	OtA5093-OtA5097
Whrn	chr4:63414909-63495951	OtA5088-OtA5092	OtA5093-OtA5097
Pde3b	chr7:114415253-114537937	OtA5088-OtA5092	OtA5093-OtA5097
Cdk5rap3	chr11:96907785-96916481	OtA5088-OtA5092	OtA5093-OtA5097
Ecsit	chr9:22055459-22389206	OtA5088-OtA5092	OtA5093-OtA5097
Fam46a	chr9:85320438-85327150	OtA5088-OtA5092	OtA5093-OtA5097
Gramd3	chr18:56432131-56503792	OtA5088-OtA5092	OtA5093-OtA5097
Slc4a5	chr6:83237374-83304945	OtA5088-OtA5092	OtA5093-OtA5097
Tmem115	chr9:107533944-107538656	OtA5088-OtA5092	OtA5093-OtA5097
Vps11	chr9:44348104-44361670	OtA5088-OtA5092	OtA5093-OtA5097
Zfyve20	chr6:92186711-92214811	OtA5088-OtA5092	OtA5093-OtA5097
Alms1	chr6:85587530-85702751	OtA5088-OtA5092	OtA5093-OtA5097
Angel2	chr1:190925107-190946491	OtA5088-OtA5092	OtA5093-OtA5097
Sat1	chrX:155213138-155216409	OtA5088-OtA5092	OtA5093-OtA5097
Casc4	chr2:121866969-121936207	OtA5088-OtA5092	OtA5093-OtA5097
Wbp5	chrX:136245079-136247139	OtA5088-OtA5092	OtA5093-OtA5097
Nudt13	chr14:20294689-20317575	OtA5088-OtA5092	OtA5093-OtA5097
Adora2a	chr10:75316942-75334788	OtA5088-OtA5092	OtA5093-OtA5097
Dnajc11	chr4:151933719-151981959	OtA5088-OtA5092	OtA5093-OtA5097
Fam46c	chr3:100471535-100489192	OtA5088-OtA5092	OtA5093-OtA5097
Kctd3	chr1:188971097-189007840	OtA5088-OtA5092	OtA5093-OtA5097

Pqlc2	chr4:139298004-139310700	OtA5088-OtA5092	OtA5093-OtA5097
Rai2	chrX:161717035-161779494	OtA5088-OtA5092	OtA5093-OtA5097
Rcor3	chr1:192100461-192151025	OtA5088-OtA5092	OtA5093-OtA5097
Otub2	chr12:103376893-103406350	OtA5088-OtA5092	OtA5093-OtA5097
Malt1	chr18:65430996-65478792	OtA5088-OtA5092	OtA5093-OtA5097
Rab24	chr13:55319222-55321980	OtA5088-OtA5092	OtA5093-OtA5097
Dixdc1	chr9:50662752-50727984	OtA5088-OtA5092	OtA5093-OtA5097
Raph1	chr1:60483184-60566765	OtA5088-OtA5092	OtA5093-OtA5097
Atl2	chr17:79848391-79896123	OtA5088-OtA5092	OtA5093-OtA5097
Atp5g1	chr11:96072792-96075694	OtA5088-OtA5092	OtA5093-OtA5097
Bcl6	chr16:23965051-23988612	OtA5088-OtA5092	OtA5093-OtA5097
Cep192	chr18:67800106-67885170	OtA5088-OtA5092	OtA5093-OtA5097
Gnptg	chr17:25188399-25240116	OtA5088-OtA5092	OtA5093-OtA5097
Lsm2	chr17:34981853-34985893	OtA5088-OtA5092	OtA5093-OtA5097
Megf8	chr7:25317163-25365917	OtA5088-OtA5092	OtA5093-OtA5097
Pdcd2l	chr7:34184496-34196647	OtA5088-OtA5092	OtA5093-OtA5097
Phf15	chr11:51813455-51857481	OtA5088-OtA5092	OtA5093-OtA5097
Pink1	chr4:138313409-138326296	OtA5088-OtA5092	OtA5093-OtA5097
Slc25a19	chr11:115614180-115628295	OtA5088-OtA5092	OtA5093-OtA5097
Tnks	chr8:34829178-34965690	OtA5088-OtA5092	OtA5093-OtA5097
Vps4b	chr1:106770787-106796725	OtA5088-OtA5092	OtA5093-OtA5097
Zbed3	chr13:95325236-95337841	OtA5088-OtA5092	OtA5093-OtA5097
Zfyve19	chr2:119172499-119217050	OtA5088-OtA5092	OtA5093-OtA5097
Apoa4	chr9:46240843-46243458	OtA5088-OtA5092	OtA5093-OtA5097
Arsk	chr13:76060421-76098660	OtA5088-OtA5092	OtA5093-OtA5097
Bend7	chr2:4717830-4802146	OtA5088-OtA5092	OtA5093-OtA5097
Bnip3	chr7:138890835-138909506	OtA5088-OtA5092	OtA5093-OtA5097
Ccbp2	chr9:121898463-121911064	OtA5088-OtA5092	OtA5093-OtA5097
Cdkn1b	chr6:134920400-134925525	OtA5088-OtA5092	OtA5093-OtA5097
Csnk2a1	chr2:152226839-152281851	OtA5088-OtA5092	OtA5093-OtA5097
Echdc2	chr4:108165436-108179308	OtA5088-OtA5092	OtA5093-OtA5097
Efhc1	chr1:20951625-20990841	OtA5088-OtA5092	OtA5093-OtA5097
Eif2ak3	chr6:70844526-70905240	OtA5088-OtA5092	OtA5093-OtA5097
Ep300	chr15:81586213-81652077	OtA5088-OtA5092	OtA5093-OtA5097
Ficd	chr5:113735781-113740607	OtA5088-OtA5092	OtA5093-OtA5097
Gstp2	chr19:4040287-4042221	OtA5088-OtA5092	OtA5093-OtA5097
Guca2b	chr4:119656602-119658945	OtA5088-OtA5092	OtA5093-OtA5097
Ikbke	chr1:131254601-131279563	OtA5088-OtA5092	OtA5093-OtA5097
Krt19	chr11:100140810-100145919	OtA5088-OtA5092	OtA5093-OtA5097
Loxl3	chr6:83034223-83054571	OtA5088-OtA5092	OtA5093-OtA5097
Map2k3	chr11:60932056-60952803	OtA5088-OtA5092	OtA5093-OtA5097
Mmp17	chr5:129584213-129608211	OtA5088-OtA5092	OtA5093-OtA5097
Mospd1	chrX:53345020-53370492	OtA5088-OtA5092	OtA5093-OtA5097
Ndufa9	chr6:126821862-126849144	OtA5088-OtA5092	OtA5093-OtA5097
Nudt18	chr14:70577846-70582571	OtA5088-OtA5092	OtA5093-OtA5097
Pgrmc2	chr3:41066325-41083046	OtA5088-OtA5092	OtA5093-OtA5097
Plekhf1	chr7:38220653-38227994	OtA5088-OtA5092	OtA5093-OtA5097

Polr3d	chr14:70438747-70443471	OtA5088-OtA5092	OtA5093-OtA5097
Ppcdc	chr9:57412659-57440114	OtA5088-OtA5092	OtA5093-OtA5097
Prl2c5	chr13:13182781-13191840	OtA5088-OtA5092	OtA5093-OtA5097
Rgnef	chr13:97898594-98206165	OtA5088-OtA5092	OtA5093-OtA5097
Rnasek	chr11:70238122-70239852	OtA5088-OtA5092	OtA5093-OtA5097
9-Sep	chr11:117199660-117362325	OtA5088-OtA5092	OtA5093-OtA5097
Sfxn5	chr6:85213050-85333422	OtA5088-OtA5092	OtA5093-OtA5097
Sh3pxd2b	chr11:32347810-32428183	OtA5088-OtA5092	OtA5093-OtA5097
St6galnac6	chr2:32587489-32620804	OtA5088-OtA5092	OtA5093-OtA5097
Thsd7a	chr6:12311607-12749253	OtA5088-OtA5092	OtA5093-OtA5097
Tmem199	chr11:78507054-78512168	OtA5088-OtA5092	OtA5093-OtA5097
Usp16	chr16:87454984-87495869	OtA5088-OtA5092	OtA5093-OtA5097
Zfp800	chr6:28239930-28261601	OtA5088-OtA5092	OtA5093-OtA5097
Zfp90	chr8:106415338-106425889	OtA5088-OtA5092	OtA5093-OtA5097
Bcap31	chrX:73686182-73716175	OtA5088-OtA5092	OtA5093-OtA5097
Ccdc163	chr4:116708929-116715104	OtA5088-OtA5092	OtA5093-OtA5097
Cd44	chr2:102811141-102901665	OtA5088-OtA5092	OtA5093-OtA5097
Cep70	chr9:99243467-99300403	OtA5088-OtA5092	OtA5093-OtA5097
Fgf2	chr3:37348652-37404830	OtA5088-OtA5092	OtA5093-OtA5097
Fh1	chr1:175601377-175625635	OtA5088-OtA5092	OtA5093-OtA5097
Fkbp7	chr2:76663033-76673098	OtA5088-OtA5092	OtA5093-OtA5097
Grhl1	chr12:24572286-24617391	OtA5088-OtA5092	OtA5093-OtA5097
Lin37	chr7:30555440-30559646	OtA5088-OtA5092	OtA5093-OtA5097
Nasp	chr4:116601051-116627951	OtA5088-OtA5092	OtA5093-OtA5097
Nrtn	chr17:56751324-56757530	OtA5088-OtA5092	OtA5093-OtA5097
Nup107	chr10:117750642-117792705	OtA5088-OtA5092	OtA5093-OtA5097
Phkg2	chr7:127573347-127588595	OtA5088-OtA5092	OtA5093-OtA5097
Pwp1	chr10:85871830-85889103	OtA5088-OtA5092	OtA5093-OtA5097
Ralgapb	chr2:158409852-158499253	OtA5088-OtA5092	OtA5093-OtA5097
Rwdd2a	chr9:86571987-86574899	OtA5088-OtA5092	OtA5093-OtA5097
Sart3	chr5:113742445-113771649	OtA5088-OtA5092	OtA5093-OtA5097
Churc1	chr12:76765572-76783178	OtA5088-OtA5092	OtA5093-OtA5097
Helz	chr11:107547959-107686943	OtA5088-OtA5092	OtA5093-OtA5097
Hes1	chr16:30065356-30067796	OtA5088-OtA5092	OtA5093-OtA5097
Msln	chr17:25748612-25754327	OtA5088-OtA5092	OtA5093-OtA5097
Rrm2b	chr15:37923952-37961055	OtA5088-OtA5092	OtA5093-OtA5097
Thoc1	chr18:9958179-10030149	OtA5088-OtA5092	OtA5093-OtA5097
Hint1	chr11:54866437-54870496	OtA5088-OtA5092	OtA5093-OtA5097
Cys1	chr12:24665837-24681795	OtA5088-OtA5092	OtA5093-OtA5097
Opa1	chr16:29579333-29654602	OtA5088-OtA5092	OtA5093-OtA5097
Akap8l	chr17:32321423-32350577	OtA5088-OtA5092	OtA5093-OtA5097
Atg4c	chr4:99193933-99259787	OtA5088-OtA5092	OtA5093-OtA5097
Chchd8	chr7:100537099-100539812	OtA5088-OtA5092	OtA5093-OtA5097
Itgb4	chr11:115974724-116012719	OtA5088-OtA5092	OtA5093-OtA5097
Lancl2	chr6:57702454-57739449	OtA5088-OtA5092	OtA5093-OtA5097
Mterfd2	chr1:93299210-93305870	OtA5088-OtA5092	OtA5093-OtA5097
Rgs3	chr4:62559846-62703019	OtA5088-OtA5092	OtA5093-OtA5097

Rrp1	chr10:78400361-78413043	OtA5088-OtA5092	OtA5093-OtA5097
Tspan5	chr3:138742194-138904424	OtA5088-OtA5092	OtA5093-OtA5097
E030024N20Rik	chr19:16132830-16387453	OtA5088-OtA5092	OtA5093-OtA5097
Gpd1l	chr9:114899338-114933987	OtA5088-OtA5092	OtA5093-OtA5097
H2-T24	chr17:35994503-36038174	OtA5088-OtA5092	OtA5093-OtA5097
Rab4b	chr7:27168432-27178883	OtA5088-OtA5092	OtA5093-OtA5097
Rac1	chr5:143505480-143527993	OtA5088-OtA5092	OtA5093-OtA5097
Adh5	chr3:138443163-138455499	OtA5088-OtA5092	OtA5093-OtA5097
Cib1	chr7:80227159-80232616	OtA5088-OtA5092	OtA5093-OtA5097
Cxcl12	chr6:117168534-117181368	OtA5088-OtA5092	OtA5093-OtA5097
Ephb4	chr5:137350108-137374522	OtA5088-OtA5092	OtA5093-OtA5097
Fam103a1	chr7:81762952-81769490	OtA5088-OtA5092	OtA5093-OtA5097
Gm2a	chr11:55097984-55113028	OtA5088-OtA5092	OtA5093-OtA5097
Gm4980	chr7:99624066-99627103	OtA5088-OtA5092	OtA5093-OtA5097
H2afy	chr13:56073621-56135550	OtA5088-OtA5092	OtA5093-OtA5097
H47	chr7:66079648-66089405	OtA5088-OtA5092	OtA5093-OtA5097
Itga8	chr2:12106659-12312315	OtA5088-OtA5092	OtA5093-OtA5097
Lcat	chr8:105939550-105943402	OtA5088-OtA5092	OtA5093-OtA5097
Lgals8	chr13:12439414-12461738	OtA5088-OtA5092	OtA5093-OtA5097
Ltbp4	chr7:27305140-27337612	OtA5088-OtA5092	OtA5093-OtA5097
Marcksl1	chr4:129513580-129515981	OtA5088-OtA5092	OtA5093-OtA5097
Nup35	chr2:80638811-80660071	OtA5088-OtA5092	OtA5093-OtA5097
Polm	chr11:5827859-5838016	OtA5088-OtA5092	OtA5093-OtA5097
Ppm1f	chr16:16896468-16927375	OtA5088-OtA5092	OtA5093-OtA5097
Ptpra	chr2:130450277-130563749	OtA5088-OtA5092	OtA5093-OtA5097
Rrp9	chr9:106477308-106485415	OtA5088-OtA5092	OtA5093-OtA5097
Siva1	chr12:112644827-112649152	OtA5088-OtA5092	OtA5093-OtA5097
Slc9a8	chr2:167421720-167476998	OtA5088-OtA5092	OtA5093-OtA5097
Stxbp1	chr2:32787606-32847237	OtA5088-OtA5092	OtA5093-OtA5097
Thtpa	chr14:55094783-55106593	OtA5088-OtA5092	OtA5093-OtA5097
Tufm	chr7:126487354-126490731	OtA5088-OtA5092	OtA5093-OtA5097
Tulp1	chr17:28351518-28365143	OtA5088-OtA5092	OtA5093-OtA5097
Ube2d3	chr3:135438758-135467178	OtA5088-OtA5092	OtA5093-OtA5097
Ube2ql1	chr13:69702831-69739897	OtA5088-OtA5092	OtA5093-OtA5097
Ubn1	chr16:5050067-5086285	OtA5088-OtA5092	OtA5093-OtA5097
Wnk2	chr13:49036301-49148014	OtA5088-OtA5092	OtA5093-OtA5097
Zfp335	chr2:164891891-164911750	OtA5088-OtA5092	OtA5093-OtA5097
Cbx6	chr15:79786350-79834333	OtA5088-OtA5092	OtA5093-OtA5097
Basp1	chr15:25363276-25413764	OtA5088-OtA5092	OtA5093-OtA5097
Col4a3bp	chr13:96542734-96640167	OtA5088-OtA5092	OtA5093-OtA5097
Etfdh	chr3:79603787-79628767	OtA5088-OtA5092	OtA5093-OtA5097
Izumo4	chr10:80702692-80705382	OtA5088-OtA5092	OtA5093-OtA5097
Klb	chr5:65348410-65384003	OtA5088-OtA5092	OtA5093-OtA5097
Me2	chr18:73770039-73815392	OtA5088-OtA5092	OtA5093-OtA5097
Fhl1	chrX:56731786-56793346	OtA5088-OtA5092	OtA5093-OtA5097
Cs	chr10:128337831-128362479	OtA5088-OtA5092	OtA5093-OtA5097
Fbxo21	chr5:117976769-118010191	OtA5088-OtA5092	OtA5093-OtA5097

Herc3	chr6:58833699-58920396	OtA5088-OtA5092	OtA5093-OtA5097
7-Mar	chr2:60209935-60248385	OtA5088-OtA5092	OtA5093-OtA5097
Rpap1	chr2:119763958-119787537	OtA5088-OtA5092	OtA5093-OtA5097
Smad3	chr9:63646766-63757994	OtA5088-OtA5092	OtA5093-OtA5097
Lrch1	chr14:74754672-74947877	OtA5088-OtA5092	OtA5093-OtA5097
Stat1	chr1:52119437-52161865	OtA5088-OtA5092	OtA5093-OtA5097
Fkrp	chr7:16809266-16840931	OtA5088-OtA5092	OtA5093-OtA5097
Thap4	chr1:93705390-93754838	OtA5088-OtA5092	OtA5093-OtA5097
Tmem20	chr19:38395979-38405607	OtA5088-OtA5092	OtA5093-OtA5097
Rbm5	chr9:107740494-107771002	OtA5088-OtA5092	OtA5093-OtA5097
Map1lc3a	chr2:155276363-155278073	OtA5088-OtA5092	OtA5093-OtA5097
Actr8	chr14:29978336-29993221	OtA5088-OtA5092	OtA5093-OtA5097
Cir1	chr2:73283871-73312592	OtA5088-OtA5092	OtA5093-OtA5097
Dhrs11	chr11:84820727-84829003	OtA5088-OtA5092	OtA5093-OtA5097
Hmgb2	chr8:57511842-57515999	OtA5088-OtA5092	OtA5093-OtA5097
Kctd6	chr14:8214147-8223569	OtA5088-OtA5092	OtA5093-OtA5097
Pebp1	chr5:117282650-117287564	OtA5088-OtA5092	OtA5093-OtA5097
Snf8	chr11:96034916-96047405	OtA5088-OtA5092	OtA5093-OtA5097
Utp3	chr5:88554482-88556083	OtA5088-OtA5092	OtA5093-OtA5097
Zfp9	chr6:118461949-118479273	OtA5088-OtA5092	OtA5093-OtA5097
Akr1b3	chr6:34303929-34317489	OtA5088-OtA5092	OtA5093-OtA5097
Zufsp	chr10:33926935-33951212	OtA5088-OtA5092	OtA5093-OtA5097
Fam3a	chrX:74384719-74393139	OtA5088-OtA5092	OtA5093-OtA5097
Nde1	chr16:14163274-14192923	OtA5088-OtA5092	OtA5093-OtA5097
Tnfsf10	chr3:27317076-27339665	OtA5088-OtA5092	OtA5093-OtA5097
Lamb3	chr1:193301993-193343878	OtA5088-OtA5092	OtA5093-OtA5097
Mad2l1bp	chr17:46147384-46153551	OtA5088-OtA5092	OtA5093-OtA5097
Dapp1	chr3:137931005-137981549	OtA5088-OtA5092	OtA5093-OtA5097
Itih4	chr14:30886491-30901983	OtA5088-OtA5092	OtA5093-OtA5097
Srp14	chr2:118475842-118479696	OtA5088-OtA5092	OtA5093-OtA5097
L3mbtl3	chr10:26275451-26375185	OtA5088-OtA5092	OtA5093-OtA5097
Myef2	chr2:125068126-125123660	OtA5088-OtA5092	OtA5093-OtA5097
Plcb3	chr19:6953712-6969752	OtA5088-OtA5092	OtA5093-OtA5097
Sh2b1	chr7:126466993-126475130	OtA5088-OtA5092	OtA5093-OtA5097
Tbc1d7	chr13:43151741-43171501	OtA5088-OtA5092	OtA5093-OtA5097
Zfp236	chr18:82593596-82692734	OtA5088-OtA5092	OtA5093-OtA5097
Tgoln2	chr6:72608420-72617000	OtA5088-OtA5092	OtA5093-OtA5097
Lrrc28	chr7:67513409-67645236	OtA5088-OtA5092	OtA5093-OtA5097
Pitpna	chr11:75588107-75628778	OtA5088-OtA5092	OtA5093-OtA5097
LOC106740	chr17:14943183-14961260	OtA5088-OtA5092	OtA5093-OtA5097
Sar1a	chr10:61680320-61693297	OtA5088-OtA5092	OtA5093-OtA5097
Aldh7a1	chr18:56525735-56587712	OtA5088-OtA5092	OtA5093-OtA5097
Ddb2	chr2:91202911-91237066	OtA5088-OtA5092	OtA5093-OtA5097
Dyrk3	chr1:131128440-131138234	OtA5088-OtA5092	OtA5093-OtA5097
Pafah1b3	chr7:25295048-25297955	OtA5088-OtA5092	OtA5093-OtA5097
Hnrnpf	chr6:117900339-117925622	OtA5088-OtA5092	OtA5093-OtA5097
Ppp3cc	chr14:70217897-70289449	OtA5088-OtA5092	OtA5093-OtA5097

Spcs1	chr14:30999825-31019089	OtA5088-OtA5092	OtA5093-OtA5097
D8Ertd82e	chr8:36094827-36147787	OtA5088-OtA5092	OtA5093-OtA5097
Ptprj	chr2:90429755-90580647	OtA5088-OtA5092	OtA5093-OtA5097
Mtmr14	chr6:113237842-113281392	OtA5088-OtA5092	OtA5093-OtA5097
Prr12	chr7:45027706-45052881	OtA5088-OtA5092	OtA5093-OtA5097
Rfwd2	chr1:159232325-159347580	OtA5088-OtA5092	OtA5093-OtA5097
Arpc1a	chr5:145083868-145108756	OtA5088-OtA5092	OtA5093-OtA5097
B4galnt3	chr6:120203809-120294559	OtA5088-OtA5092	OtA5093-OtA5097
Rrn3	chr16:13780698-13814841	OtA5088-OtA5092	OtA5093-OtA5097
Sdc2	chr15:32920722-33034721	OtA5088-OtA5092	OtA5093-OtA5097
Snrpf	chr10:93540631-93605245	OtA5088-OtA5092	OtA5093-OtA5097
Bcl2l11	chr2:128126037-128162547	OtA5088-OtA5092	OtA5093-OtA5097
Drap1	chr19:5406873-5424916	OtA5088-OtA5092	OtA5093-OtA5097
F2	chr2:91625319-91636414	OtA5088-OtA5092	OtA5093-OtA5097
Galk1	chr11:115974724-116012719	OtA5088-OtA5092	OtA5093-OtA5097
Gfod1	chr13:43195518-43304172	OtA5088-OtA5092	OtA5093-OtA5097
Msl3	chrX:168654117-168673902	OtA5088-OtA5092	OtA5093-OtA5097
Ncoa5	chr2:165000356-165034779	OtA5088-OtA5092	OtA5093-OtA5097
Pdzrn3	chr6:101149606-101377897	OtA5088-OtA5092	OtA5093-OtA5097
Psen2	chr1:180227003-180256300	OtA5088-OtA5092	OtA5093-OtA5097
Psg25	chr7:18519701-18532227	OtA5088-OtA5092	OtA5093-OtA5097
Selenbp2	chr3:94693572-94704406	OtA5088-OtA5092	OtA5093-OtA5097
Slc22a15	chr3:101855770-101924453	OtA5088-OtA5092	OtA5093-OtA5097
Zfp407	chr18:84207701-84589504	OtA5088-OtA5092	OtA5093-OtA5097
Taf6l	chr19:8772521-8786417	OtA5088-OtA5092	OtA5093-OtA5097
Ddc	chr11:11814100-11898144	OtA5088-OtA5092	OtA5093-OtA5097
Fam69b	chr2:26628456-26636497	OtA5088-OtA5092	OtA5093-OtA5097
Gm16386	chr17:22779857-22817747	OtA5088-OtA5092	OtA5093-OtA5097
Gtl3	chr8:95420249-95434869	OtA5088-OtA5092	OtA5093-OtA5097
Metap1d	chr2:71453337-71525191	OtA5088-OtA5092	OtA5093-OtA5097
Pdcd6	chr13:74303120-74317326	OtA5088-OtA5092	OtA5093-OtA5097
Rnf123	chr9:108049289-108079375	OtA5088-OtA5092	OtA5093-OtA5097
Casp9	chr4:141793611-141826003	OtA5088-OtA5092	OtA5093-OtA5097
Esyt1	chr10:128510249-128525859	OtA5088-OtA5092	OtA5093-OtA5097
Myo1e	chr9:70207349-70400067	OtA5088-OtA5092	OtA5093-OtA5097
Bloc1s1	chr10:128919913-128923524	OtA5088-OtA5092	OtA5093-OtA5097
Cct6b	chr11:82719247-82764288	OtA5088-OtA5092	OtA5093-OtA5097
Cdh4	chr2:179442477-179899375	OtA5088-OtA5092	OtA5093-OtA5097
Commd2	chr3:57644348-57651684	OtA5088-OtA5092	OtA5093-OtA5097
Dap	chr15:31224384-31274338	OtA5088-OtA5092	OtA5093-OtA5097
Dlg5	chr14:24133952-24245920	OtA5088-OtA5092	OtA5093-OtA5097
Eif3d	chr15:77958997-77970824	OtA5088-OtA5092	OtA5093-OtA5097
Eps8l3	chr3:107877229-107898685	OtA5088-OtA5092	OtA5093-OtA5097
G3bp1	chr11:55469751-55500887	OtA5088-OtA5092	OtA5093-OtA5097
Hjurp	chr1:88227019-88277579	OtA5088-OtA5092	OtA5093-OtA5097
Il17re	chr6:113458483-113470751	OtA5088-OtA5092	OtA5093-OtA5097
Men1	chr19:6334978-6340894	OtA5088-OtA5092	OtA5093-OtA5097

Ndn	chr7:62348276-62349927	OtA5088-OtA5092	OtA5093-OtA5097
Pllp	chr8:94674894-94696242	OtA5088-OtA5092	OtA5093-OtA5097
Rabepk	chr2:34778665-34799912	OtA5088-OtA5092	OtA5093-OtA5097
Rnf34	chr5:122850187-122868945	OtA5088-OtA5092	OtA5093-OtA5097
Robo1	chr16:72663148-73046100	OtA5088-OtA5092	OtA5093-OtA5097
Rras	chr7:45018006-45021644	OtA5088-OtA5092	OtA5093-OtA5097
Siah2	chr3:58674948-58692388	OtA5088-OtA5092	OtA5093-OtA5097
Sms	chrX:157443953-157492046	OtA5088-OtA5092	OtA5093-OtA5097
Srp72	chr5:76974700-76999935	OtA5088-OtA5092	OtA5093-OtA5097
Zfp41	chr15:75616684-75625295	OtA5088-OtA5092	OtA5093-OtA5097
Ubox5	chr2:130589995-130630038	OtA5088-OtA5092	OtA5093-OtA5097
Wdr75	chr1:45795500-45823613	OtA5088-OtA5092	OtA5093-OtA5097
Gpn1	chr5:31494760-31511627	OtA5088-OtA5092	OtA5093-OtA5097
Golga5	chr12:102469133-102497907	OtA5088-OtA5092	OtA5093-OtA5097
Ifrd1	chr12:40203128-40223189	OtA5088-OtA5092	OtA5093-OtA5097
Lrpap1	chr5:35091505-35105697	OtA5088-OtA5092	OtA5093-OtA5097
Smyd3	chr1:178955038-179518003	OtA5088-OtA5092	OtA5093-OtA5097
Yod1	chr1:130689042-130729253	OtA5088-OtA5092	OtA5093-OtA5097
Zfp445	chr9:122848909-122866006	OtA5088-OtA5092	OtA5093-OtA5097
Gata4	chr14:63198914-63245260	OtA5088-OtA5092	OtA5093-OtA5097
Gosr1	chr11:76726601-76763555	OtA5088-OtA5092	OtA5093-OtA5097
Stt3a	chr9:36732412-36767578	OtA5088-OtA5092	OtA5093-OtA5097
Rnf41	chr10:128411615-128441441	OtA5088-OtA5092	OtA5093-OtA5097
E130012A19Rik	chr11:97627386-97629716	OtA5088-OtA5092	OtA5093-OtA5097
Pbx2	chr17:34592470-34597341	OtA5088-OtA5092	OtA5093-OtA5097
Arhgef25	chr10:127182520-127190054	OtA5088-OtA5092	OtA5093-OtA5097
Cnnm3	chr1:36511875-36528237	OtA5088-OtA5092	OtA5093-OtA5097
D330012F22Rik	chr7:80232892-80241420	OtA5088-OtA5092	OtA5093-OtA5097
Pkn1	chr8:83666639-83699179	OtA5088-OtA5092	OtA5093-OtA5097
Tmem45b	chr9:31426195-31464238	OtA5088-OtA5092	OtA5093-OtA5097
Lrrc8a	chr2:30237768-30263790	OtA5088-OtA5092	OtA5093-OtA5097
Coq9	chr8:94838416-94854895	OtA5088-OtA5092	OtA5093-OtA5097
Arhgef19	chr4:141242883-141257562	OtA5088-OtA5092	OtA5093-OtA5097
Baat1	chr5:140705022-140719334	OtA5088-OtA5092	OtA5093-OtA5097
Bcl7b	chr5:135168371-135181852	OtA5088-OtA5092	OtA5093-OtA5097
Fam107b	chr2:3713457-3782134	OtA5088-OtA5092	OtA5093-OtA5097
Pga5	chr19:10668956-10678071	OtA5088-OtA5092	OtA5093-OtA5097
Setdb1	chr3:95315251-95357202	OtA5088-OtA5092	OtA5093-OtA5097
Nudcd3	chr11:6105691-6200451	OtA5088-OtA5092	OtA5093-OtA5097
Sdr42e1	chr8:117661398-117671515	OtA5088-OtA5092	OtA5093-OtA5097
Srfbp1	chr18:52465692-52490738	OtA5088-OtA5092	OtA5093-OtA5097
Tgm1	chr14:55700008-55713492	OtA5088-OtA5092	OtA5093-OtA5097
Uros	chr7:133686329-133709295	OtA5088-OtA5092	OtA5093-OtA5097
Usp42	chr5:143710325-143732280	OtA5088-OtA5092	OtA5093-OtA5097
Axin2	chr11:108920348-108950781	OtA5088-OtA5092	OtA5093-OtA5097
Zfp35	chr18:23989633-24005371	OtA5088-OtA5092	OtA5093-OtA5097
Zfp26	chr9:20428317-20460160	OtA5088-OtA5092	OtA5093-OtA5097

D230037D09Rik	chr12:102739829-102757810	OtA5088-OtA5092	OtA5093-OtA5097
Selm	chr11:3514701-3517351	OtA5088-OtA5092	OtA5093-OtA5097
Tctn2	chr5:124598748-124627738	OtA5088-OtA5092	OtA5093-OtA5097
Ascc2	chr11:4637792-4683386	OtA5088-OtA5092	OtA5093-OtA5097
Kars	chr8:111993439-112011305	OtA5088-OtA5092	OtA5093-OtA5097
Zfp369	chr13:65278853-65297795	OtA5088-OtA5092	OtA5093-OtA5097
C85492	chr9:121981605-121996026	OtA5088-OtA5092	OtA5093-OtA5097
Ptpn9	chr9:56994967-57071966	OtA5088-OtA5092	OtA5093-OtA5097
Rfc2	chr5:134582689-134598328	OtA5088-OtA5092	OtA5093-OtA5097
Slc25a15	chr8:22375549-22398621	OtA5088-OtA5092	OtA5093-OtA5097
Slc4a11	chr2:130684107-130697519	OtA5088-OtA5092	OtA5093-OtA5097
Gak	chr5:108569413-108629739	OtA5088-OtA5092	OtA5093-OtA5097
Smn1	chr13:100123204-100137698	OtA5088-OtA5092	OtA5093-OtA5097
Ccdc66	chr14:27428846-27508460	OtA5088-OtA5092	OtA5093-OtA5097
Dmrtc1a	chrX:102903220-102908687	OtA5088-OtA5092	OtA5093-OtA5097
Sh3bp5l	chr11:58330717-58347728	OtA5088-OtA5092	OtA5093-OtA5097
Uckl1	chr2:181569152-181592461	OtA5088-OtA5092	OtA5093-OtA5097
Egfl7	chr2:26581055-26592682	OtA5088-OtA5092	OtA5093-OtA5097
Cadm4	chr7:24482022-24504533	OtA5088-OtA5092	OtA5093-OtA5097
Ccdc32	chr2:119017778-119029393	OtA5088-OtA5092	OtA5093-OtA5097
Cnot4	chr6:35022064-35133737	OtA5088-OtA5092	OtA5093-OtA5097
Diap1	chr18:37844824-37935411	OtA5088-OtA5092	OtA5093-OtA5097
Dusp11	chr6:85942268-85961667	OtA5088-OtA5092	OtA5093-OtA5097
Eif1	chr11:100319995-100322096	OtA5088-OtA5092	OtA5093-OtA5097
Il2rg	chrX:101264386-101268206	OtA5088-OtA5092	OtA5093-OtA5097
Itih1	chr14:30929179-30943289	OtA5088-OtA5092	OtA5093-OtA5097
Jak1	chr4:101069037-101265282	OtA5088-OtA5092	OtA5093-OtA5097
Ldb2	chr5:44472132-44799707	OtA5088-OtA5092	OtA5093-OtA5097
Pip4k2a	chr2:18842255-18998121	OtA5088-OtA5092	OtA5093-OtA5097
Rnf20	chr4:49632059-49656886	OtA5088-OtA5092	OtA5093-OtA5097
Serpinh1	chr7:99345374-99353239	OtA5088-OtA5092	OtA5093-OtA5097
Snap23	chr2:120567670-120600722	OtA5088-OtA5092	OtA5093-OtA5097
Sorbs1	chr19:40292039-40513812	OtA5088-OtA5092	OtA5093-OtA5097
Usp12	chr5:146734811-146794956	OtA5088-OtA5092	OtA5093-OtA5097
Chd2	chr7:73426651-73558395	OtA5088-OtA5092	OtA5093-OtA5097
Ippk	chr13:49421310-49462990	OtA5088-OtA5092	OtA5093-OtA5097
Med21	chr6:146642578-146650600	OtA5088-OtA5092	OtA5093-OtA5097
Rassf7	chr7:141215859-141218658	OtA5088-OtA5092	OtA5093-OtA5097
Dmap1	chr4:117674685-117682225	OtA5088-OtA5092	OtA5093-OtA5097
Ftsjd1	chr8:110217959-110224489	OtA5088-OtA5092	OtA5093-OtA5097
Mrpl2	chr17:46646247-46650132	OtA5088-OtA5092	OtA5093-OtA5097
Synpo2	chr3:123076518-123236149	OtA5088-OtA5092	OtA5093-OtA5097
Larp6	chr9:60713120-60738801	OtA5088-OtA5092	OtA5093-OtA5097
Rps19	chr7:24884713-24889802	OtA5088-OtA5092	OtA5093-OtA5097
Serinc1	chr10:57515774-57532529	OtA5088-OtA5092	OtA5093-OtA5097
Shmt2	chr10:127514938-127522444	OtA5088-OtA5092	OtA5093-OtA5097
Slc7a6	chr8:106168874-106198704	OtA5088-OtA5092	OtA5093-OtA5097

Tmbim1	chr1:74285033-74353692	OtA5088-OtA5092	OtA5093-OtA5097
Tmem2	chr19:21778341-21858327	OtA5088-OtA5092	OtA5093-OtA5097
Trim65	chr11:116124707-116131128	OtA5088-OtA5092	OtA5093-OtA5097
Tspyl1	chr10:34282189-34284885	OtA5088-OtA5092	OtA5093-OtA5097
Hras1	chr7:141189933-141194004	OtA5088-OtA5092	OtA5093-OtA5097
Syngr2	chr11:117809666-117814286	OtA5088-OtA5092	OtA5093-OtA5097
Dctpp1	chr7:127256958-127260667	OtA5088-OtA5092	OtA5093-OtA5097
B4galnt2	chr11:95863558-95914871	OtA5088-OtA5092	OtA5093-OtA5097
Extl3	chr14:65052058-65098106	OtA5088-OtA5092	OtA5093-OtA5097
Kctd13	chr7:126928878-126945609	OtA5088-OtA5092	OtA5093-OtA5097
Rpph1	chr14:50807446-50807771	OtA5088-OtA5092	OtA5093-OtA5097
Spsb1	chr4:149896283-149955006	OtA5088-OtA5092	OtA5093-OtA5097
Gramd1a	chr7:31130126-31151050	OtA5088-OtA5092	OtA5093-OtA5097
Nmnat2	chr1:152955100-153119261	OtA5088-OtA5092	OtA5093-OtA5097
Runx1	chr16:92601465-92826066	OtA5088-OtA5092	OtA5093-OtA5097
Prkab2	chr3:97658211-97673067	OtA5088-OtA5092	OtA5093-OtA5097
Rai1	chr11:60105012-60220604	OtA5088-OtA5092	OtA5093-OtA5097
Alkbh6	chr7:30308752-30314303	OtA5088-OtA5092	OtA5093-OtA5097
Fastkd3	chr13:68582247-68592279	OtA5088-OtA5092	OtA5093-OtA5097
Ube3b	chr5:114380606-114421166	OtA5088-OtA5092	OtA5093-OtA5097
Znhit2-ps	chr19:6061206-6062468	OtA5088-OtA5092	OtA5093-OtA5097
Afmid	chr11:117815518-117839908	OtA5088-OtA5092	OtA5093-OtA5097
Elovl5	chr9:77917364-77984519	OtA5088-OtA5092	OtA5093-OtA5097
Phospho2	chr2:69789736-69797168	OtA5088-OtA5092	OtA5093-OtA5097
Fam109a	chr5:121849027-121854599	OtA5088-OtA5092	OtA5093-OtA5097
Fam89b	chr19:5728086-5729666	OtA5088-OtA5092	OtA5093-OtA5097
Lsm3	chr6:91516034-91522620	OtA5088-OtA5092	OtA5093-OtA5097
Erlec1	chr11:30929783-30954131	OtA5088-OtA5092	OtA5093-OtA5097
Git1	chr11:77493411-77507774	OtA5088-OtA5092	OtA5093-OtA5097
Spred2	chr11:19924441-20022597	OtA5088-OtA5092	OtA5093-OtA5097
Vta1	chr10:14655332-14705489	OtA5088-OtA5092	OtA5093-OtA5097
Minpp1	chr19:32485768-32515370	OtA5088-OtA5092	OtA5093-OtA5097
Ptdss1	chr13:66912097-67032405	OtA5088-OtA5092	OtA5093-OtA5097
Rab35	chr5:115631986-115647158	OtA5088-OtA5092	OtA5093-OtA5097
Tomm22	chr15:79670867-79672862	OtA5088-OtA5092	OtA5093-OtA5097
Dusp4	chr8:34807609-34819894	OtA5088-OtA5092	OtA5093-OtA5097
Ecscr	chr18:35713088-35721491	OtA5088-OtA5092	OtA5093-OtA5097
Gm15772	chr11:68901565-68904534	OtA5088-OtA5092	OtA5093-OtA5097
Abhd6	chr14:8002901-8056555	OtA5088-OtA5092	OtA5093-OtA5097
Bach1	chr16:87698953-87733346	OtA5088-OtA5092	OtA5093-OtA5097
Cpsf3	chr12:21286296-21315056	OtA5088-OtA5092	OtA5093-OtA5097
C330005M16Rik	chr7:28607636-28631015	OtA5088-OtA5092	OtA5093-OtA5097
Ptgs2	chr1:150100123-150108012	OtA5088-OtA5092	OtA5093-OtA5097
Kdm6a	chrX:18162666-18279358	OtA5088-OtA5092	OtA5093-OtA5097
Fbxl3	chr14:103080238-103099509	OtA5088-OtA5092	OtA5093-OtA5097
Uspl1	chr5:149184559-149215434	OtA5088-OtA5092	OtA5093-OtA5097
Acy1	chr9:106432995-106438236	OtA5088-OtA5092	OtA5093-OtA5097

E2f3	chr13:29906574-29986063	OtA5088-OtA5092	OtA5093-OtA5097
Ercc6	chr14:32513520-32580989	OtA5088-OtA5092	OtA5093-OtA5097
Gtpbp1	chr15:79690895-79721479	OtA5088-OtA5092	OtA5093-OtA5097
H2-Eb1	chr17:34305866-34316674	OtA5088-OtA5092	OtA5093-OtA5097
Lrrc14	chr15:76710739-76715091	OtA5088-OtA5092	OtA5093-OtA5097
Otud7b	chr3:96080595-96161129	OtA5088-OtA5092	OtA5093-OtA5097
Pim3	chr15:88862193-88865726	OtA5088-OtA5092	OtA5093-OtA5097
Ppp3cb	chr14:20499313-20618354	OtA5088-OtA5092	OtA5093-OtA5097
Rnf208	chr2:25242928-25244261	OtA5088-OtA5092	OtA5093-OtA5097
Serpind1	chr16:17280350-17430826	OtA5088-OtA5092	OtA5093-OtA5097
Soat2	chr15:102150574-102163436	OtA5088-OtA5092	OtA5093-OtA5097
Sugp2	chr8:70220192-70263105	OtA5088-OtA5092	OtA5093-OtA5097
Tinag	chr9:76951697-77045781	OtA5088-OtA5092	OtA5093-OtA5097
Top2b	chr14:16365205-16430787	OtA5088-OtA5092	OtA5093-OtA5097
Trim14	chr4:46506846-46536144	OtA5088-OtA5092	OtA5093-OtA5097
Ywhab	chr2:163995196-164018587	OtA5088-OtA5092	OtA5093-OtA5097
Ctnnbl1	chr2:157737400-157891903	OtA5088-OtA5092	OtA5093-OtA5097
Wdr89	chr12:75630593-75669537	OtA5088-OtA5092	OtA5093-OtA5097
Snx14	chr9:88376746-88438951	OtA5088-OtA5092	OtA5093-OtA5097
Zfp828	chr8:13869640-13881639	OtA5088-OtA5092	OtA5093-OtA5097
Dand5	chr8:84815404-84832265	OtA5088-OtA5092	OtA5093-OtA5097
Ndufb9	chr15:58933809-58939489	OtA5088-OtA5092	OtA5093-OtA5097
Acadvl	chr11:70010191-70015407	OtA5088-OtA5092	OtA5093-OtA5097
B4galt5	chr2:167298444-167349178	OtA5088-OtA5092	OtA5093-OtA5097
Elp4	chr2:105700108-105904500	OtA5088-OtA5092	OtA5093-OtA5097
Nt5c3	chr6:56882401-56923932	OtA5088-OtA5092	OtA5093-OtA5097
Arrdc4	chr7:68736993-68749238	OtA5088-OtA5092	OtA5093-OtA5097
Apcdd1	chr18:62922326-62953195	OtA5088-OtA5092	OtA5093-OtA5097
Mtif2	chr11:29526456-29545255	OtA5088-OtA5092	OtA5093-OtA5097
Pparg	chr6:115361220-115490401	OtA5088-OtA5092	OtA5093-OtA5097
Kcnv2	chr19:27322618-27337179	OtA5088-OtA5092	OtA5093-OtA5097
Ttc8	chr12:98920573-98983238	OtA5088-OtA5092	OtA5093-OtA5097
Ip6k2	chr9:108795993-108806333	OtA5088-OtA5092	OtA5093-OtA5097
Acbd4	chr11:103101687-103112199	OtA5088-OtA5092	OtA5093-OtA5097
Fndc3a	chr14:72537952-72710003	OtA5088-OtA5092	OtA5093-OtA5097
Gm9992	chr17:7363711-7385305	OtA5088-OtA5092	OtA5093-OtA5097
Rassf10	chr7:112953961-112957458	OtA5088-OtA5092	OtA5093-OtA5097
Dtna	chr18:23415414-23659714	OtA5088-OtA5092	OtA5093-OtA5097
Slc22a5	chr11:53864541-53891703	OtA5088-OtA5092	OtA5093-OtA5097
Mfap1b	chr2:121461665-121474023	OtA5088-OtA5092	OtA5093-OtA5097
Galnt1	chr18:24205343-24286816	OtA5088-OtA5092	OtA5093-OtA5097
Htra2	chr6:83034223-83054571	OtA5088-OtA5092	OtA5093-OtA5097
Mex3d	chr10:80380354-80387651	OtA5088-OtA5092	OtA5093-OtA5097
Mtdh	chr15:34082718-34142385	OtA5088-OtA5092	OtA5093-OtA5097
Mto1	chr9:78448209-78474152	OtA5088-OtA5092	OtA5093-OtA5097
Rimklb	chr6:122453608-122486305	OtA5088-OtA5092	OtA5093-OtA5097
Slc25a46	chr18:31580167-31609902	OtA5088-OtA5092	OtA5093-OtA5097

Tap2	chr17:34204478-34216321	OtA5088-OtA5092	OtA5093-OtA5097
Utp14b	chr1:78657824-78707743	OtA5088-OtA5092	OtA5093-OtA5097
Wdr24	chr17:25823626-25828730	OtA5088-OtA5092	OtA5093-OtA5097
Zdhhc14	chr17:5492599-5753891	OtA5088-OtA5092	OtA5093-OtA5097
Zfp251	chr15:76852140-76871435	OtA5088-OtA5092	OtA5093-OtA5097
Vezf1	chr11:88068340-88084729	OtA5088-OtA5092	OtA5093-OtA5097
Commd4	chr9:57155040-57158299	OtA5088-OtA5092	OtA5093-OtA5097
Gm561	chr2:144594064-144595365	OtA5088-OtA5092	OtA5093-OtA5097
Rrp1b	chr17:32036161-32062862	OtA5088-OtA5092	OtA5093-OtA5097
Ypel5	chr17:72836703-72851195	OtA5088-OtA5092	OtA5093-OtA5097
Zfp777	chr6:48024187-48048114	OtA5088-OtA5092	OtA5093-OtA5097
Lsm4	chr8:70673230-70678752	OtA5088-OtA5092	OtA5093-OtA5097
Mansc1	chr6:134609206-134632488	OtA5088-OtA5092	OtA5093-OtA5097
Pin4	chrX:102119464-102127673	OtA5088-OtA5092	OtA5093-OtA5097
Plekhh1	chr12:79029162-79089670	OtA5088-OtA5092	OtA5093-OtA5097
Prl3c1	chr13:27196659-27203750	OtA5088-OtA5092	OtA5093-OtA5097
Rab4a	chr8:123805995-123835291	OtA5088-OtA5092	OtA5093-OtA5097
Atat1	chr17:35897597-35910068	OtA5088-OtA5092	OtA5093-OtA5097
Pet112l	chr3:85574128-85654470	OtA5088-OtA5092	OtA5093-OtA5097
Plac1	chrX:53070001-53114405	OtA5088-OtA5092	OtA5093-OtA5097
Ppp1r35	chr5:137778917-137780107	OtA5088-OtA5092	OtA5093-OtA5097
Taf11	chr17:27901127-27907724	OtA5088-OtA5092	OtA5093-OtA5097
Guk1	chr11:59183854-59191952	OtA5088-OtA5092	OtA5093-OtA5097
Pcdh18	chr3:49743295-49757316	OtA5088-OtA5092	OtA5093-OtA5097
Pltp	chr2:164830729-164857708	OtA5088-OtA5092	OtA5093-OtA5097
Rsph3a	chr17:7945613-7979556	OtA5088-OtA5092	OtA5093-OtA5097
Tesk2	chr4:116720954-116807559	OtA5088-OtA5092	OtA5093-OtA5097
Sigmar1	chr4:41738495-41741313	OtA5088-OtA5092	OtA5093-OtA5097
Tpd52l2	chr2:181497252-181516757	OtA5088-OtA5092	OtA5093-OtA5097
Wdr45	chrX:7722248-7728201	OtA5088-OtA5092	OtA5093-OtA5097
Alg2	chr4:47469832-47474367	OtA5088-OtA5092	OtA5093-OtA5097
Malat1	chr19:5795689-5802671	OtA5088-OtA5092	OtA5093-OtA5097
Mllt6	chr11:97663411-97685458	OtA5088-OtA5092	OtA5093-OtA5097
Rpl13a	chr7:45125562-45128745	OtA5088-OtA5092	OtA5093-OtA5097
Mbd6	chr10:127266261-127288771	OtA5088-OtA5092	OtA5093-OtA5097
Usp11	chrX:20703908-20720539	OtA5088-OtA5092	OtA5093-OtA5097
Timm10	chr2:84827020-84830213	OtA5088-OtA5092	OtA5093-OtA5097
Ctgf	chr10:24595441-24598682	OtA5088-OtA5092	OtA5093-OtA5097
Lipt1	chr1:37872205-37876298	OtA5088-OtA5092	OtA5093-OtA5097
Nup160	chr2:90677214-90736328	OtA5088-OtA5092	OtA5093-OtA5097
Sh3bp2	chr5:34525783-34563639	OtA5088-OtA5092	OtA5093-OtA5097
Wfs1	chr5:36966103-36988982	OtA5088-OtA5092	OtA5093-OtA5097
Mob2	chr7:142008552-142061034	OtA5088-OtA5092	OtA5093-OtA5097
Fam124a	chr14:62555736-62608485	OtA5088-OtA5092	OtA5093-OtA5097
Slc24a3	chr2:145242610-145641939	OtA5088-OtA5092	OtA5093-OtA5097
Tmem68	chr4:3549040-3574768	OtA5088-OtA5092	OtA5093-OtA5097
Tob2	chr15:81848269-81858326	OtA5088-OtA5092	OtA5093-OtA5097

Nrd1	chr4:109000804-109202272	OtA5088-OtA5092	OtA5093-OtA5097
Pias4	chr10:81153965-81167720	OtA5088-OtA5092	OtA5093-OtA5097
Snx18	chr13:113592178-113618564	OtA5088-OtA5092	OtA5093-OtA5097
Sema3e	chr5:14025275-14256689	OtA5088-OtA5092	OtA5093-OtA5097
Fads1	chr19:10182887-10196872	OtA5088-OtA5092	OtA5093-OtA5097
Arl3	chr19:46531108-46573085	OtA5088-OtA5092	OtA5093-OtA5097
Cnot8	chr11:58104152-58118594	OtA5088-OtA5092	OtA5093-OtA5097
Colec11	chr12:28594172-28623290	OtA5088-OtA5092	OtA5093-OtA5097
Gabbr1	chr17:37045965-37074305	OtA5088-OtA5092	OtA5093-OtA5097
Psmg2	chr18:67641598-67654162	OtA5088-OtA5092	OtA5093-OtA5097
Topors	chr4:40259605-40270221	OtA5088-OtA5092	OtA5093-OtA5097
Anapc11	chr11:120598420-120608198	OtA5088-OtA5092	OtA5093-OtA5097
Cnih4	chr1:181150930-181168994	OtA5088-OtA5092	OtA5093-OtA5097
Naa20	chr2:145903240-145916425	OtA5088-OtA5092	OtA5093-OtA5097
Phlda3	chr1:135766084-135769134	OtA5088-OtA5092	OtA5093-OtA5097
Mitf	chr6:97807057-98021358	OtA5088-OtA5092	OtA5093-OtA5097
Sri	chr5:8046079-8069245	OtA5088-OtA5092	OtA5093-OtA5097
C130039O16Rik	chr12:84149173-84218881	OtA5088-OtA5092	OtA5093-OtA5097
Zfp36l3	chrX:53772685-53776394	OtA5088-OtA5092	OtA5093-OtA5097
Papd5	chr8:88199212-88259722	OtA5088-OtA5092	OtA5093-OtA5097
Dgkd	chr1:87853286-87944489	OtA5088-OtA5092	OtA5093-OtA5097
Ugt1a7c	chr1:88055410-88220002	OtA5088-OtA5092	OtA5093-OtA5097
Usmg5	chr19:47067747-47090625	OtA5088-OtA5092	OtA5093-OtA5097
Arhgef10	chr8:14911716-15001085	OtA5088-OtA5092	OtA5093-OtA5097
Taz	chrX:74282696-74290151	OtA5088-OtA5092	OtA5093-OtA5097
Nudt3	chr17:27579390-27623452	OtA5088-OtA5092	OtA5093-OtA5097
Pum2	chr12:8674133-8752583	OtA5088-OtA5092	OtA5093-OtA5097
Tbc1d24	chr17:24175430-24209387	OtA5088-OtA5092	OtA5093-OtA5097
Nt5e	chr9:88327608-88372089	OtA5088-OtA5092	OtA5093-OtA5097
Zmat2	chr18:36793922-36799660	OtA5088-OtA5092	OtA5093-OtA5097
Znfx1	chr2:167035796-167065862	OtA5088-OtA5092	OtA5093-OtA5097
Atxn7l3b	chr10:112925427-112929026	OtA5088-OtA5092	OtA5093-OtA5097
Cblb	chr16:52031548-52208046	OtA5088-OtA5092	OtA5093-OtA5097
Cited2	chr10:17723227-17725674	OtA5088-OtA5092	OtA5093-OtA5097
Ctla2b	chr13:60895350-60897447	OtA5088-OtA5092	OtA5093-OtA5097
Hus1	chr11:8993141-9011137	OtA5088-OtA5092	OtA5093-OtA5097
Mtg1	chr7:140137563-140154659	OtA5088-OtA5092	OtA5093-OtA5097
Thg1l	chr11:45948153-45955494	OtA5088-OtA5092	OtA5093-OtA5097
Uvrag	chr7:98886742-99141144	OtA5088-OtA5092	OtA5093-OtA5097
Zfp346	chr13:55105308-55135071	OtA5088-OtA5092	OtA5093-OtA5097
C430049B03Rik	chrX:53053111-53057190	OtA5088-OtA5092	OtA5093-OtA5097
Cnksr3	chr10:7119061-7212237	OtA5088-OtA5092	OtA5093-OtA5097
Gtf2h4	chr17:35667738-35673682	OtA5088-OtA5092	OtA5093-OtA5097
Ift172	chr5:31253278-31291114	OtA5088-OtA5092	OtA5093-OtA5097
Naa25	chr5:121397981-121440113	OtA5088-OtA5092	OtA5093-OtA5097
Cyp4v3	chr8:45305801-45333196	OtA5088-OtA5092	OtA5093-OtA5097
Pias1	chr9:62880076-62980879	OtA5088-OtA5092	OtA5093-OtA5097

Plekha1	chr7:130865909-130913302	OtA5088-OtA5092	OtA5093-OtA5097
Etv3	chr3:87525577-87540156	OtA5088-OtA5092	OtA5093-OtA5097
Fam171a2	chr11:102436980-102447663	OtA5088-OtA5092	OtA5093-OtA5097
Fkbp2	chr19:6977738-6980461	OtA5088-OtA5092	OtA5093-OtA5097
Nt5c	chr11:115475676-115491814	OtA5088-OtA5092	OtA5093-OtA5097
Cdh23	chr10:60302749-60696490	OtA5088-OtA5092	OtA5093-OtA5097
Get4	chr5:139252323-139270050	OtA5088-OtA5092	OtA5093-OtA5097
Rnf6	chr5:146209193-146221457	OtA5088-OtA5092	OtA5093-OtA5097
Alg1	chr16:5233620-5255956	OtA5088-OtA5092	OtA5093-OtA5097
Armc1	chr3:19132143-19163065	OtA5088-OtA5092	OtA5093-OtA5097
Bpnt1	chr1:185332158-185357769	OtA5088-OtA5092	OtA5093-OtA5097
Btg3	chr16:78359859-78376785	OtA5088-OtA5092	OtA5093-OtA5097
Clip1	chr5:123579069-123684291	OtA5088-OtA5092	OtA5093-OtA5097
Dnajb14	chr3:137867674-137908931	OtA5088-OtA5092	OtA5093-OtA5097
Gm16515	chr11:60902245-60913792	OtA5088-OtA5092	OtA5093-OtA5097
Grcc10	chr6:124739183-124741079	OtA5088-OtA5092	OtA5093-OtA5097
Hmgn1	chr16:96121587-96127725	OtA5088-OtA5092	OtA5093-OtA5097
Hsdl1	chr8:119561977-119575200	OtA5088-OtA5092	OtA5093-OtA5097
Hsp90aa1	chr12:110691035-110696395	OtA5088-OtA5092	OtA5093-OtA5097
Igsf1	chrX:49782536-49797745	OtA5088-OtA5092	OtA5093-OtA5097
Itga2b	chr11:102453296-102469883	OtA5088-OtA5092	OtA5093-OtA5097
Kank1	chr19:25237201-25434496	OtA5088-OtA5092	OtA5093-OtA5097
Mgmt	chr7:136894610-137128188	OtA5088-OtA5092	OtA5093-OtA5097
Mrm1	chr11:84813060-84819515	OtA5088-OtA5092	OtA5093-OtA5097
Mtap7	chr10:20148919-20281590	OtA5088-OtA5092	OtA5093-OtA5097
Mylip	chr13:45389741-45411940	OtA5088-OtA5092	OtA5093-OtA5097
Nop10	chr2:112261968-112262898	OtA5088-OtA5092	OtA5093-OtA5097
Nptx1	chr11:119538718-119547820	OtA5088-OtA5092	OtA5093-OtA5097
Rhoc	chr3:104789033-104794459	OtA5088-OtA5092	OtA5093-OtA5097
Rraga	chr4:86575672-86577283	OtA5088-OtA5092	OtA5093-OtA5097
Snrnp48	chr13:38204938-38227663	OtA5088-OtA5092	OtA5093-OtA5097
Sntb2	chr8:106935749-107014192	OtA5088-OtA5092	OtA5093-OtA5097
Tchh	chr3:93442329-93449077	OtA5088-OtA5092	OtA5093-OtA5097
Tm9sf4	chr2:153161300-153210463	OtA5088-OtA5092	OtA5093-OtA5097
Trappc8	chr18:20817223-20896078	OtA5088-OtA5092	OtA5093-OtA5097
Ttll4	chr1:74661753-74697973	OtA5088-OtA5092	OtA5093-OtA5097
Ube2j1	chr4:33031424-33052364	OtA5088-OtA5092	OtA5093-OtA5097
Uchl1	chr5:66676120-66687234	OtA5088-OtA5092	OtA5093-OtA5097
Zfp646	chr7:127877700-127890970	OtA5088-OtA5092	OtA5093-OtA5097
Klhl28	chr12:64942439-64965536	OtA5088-OtA5092	OtA5093-OtA5097
Rad52	chr6:119902697-119922823	OtA5088-OtA5092	OtA5093-OtA5097
Slc6a2	chr8:92961046-93001667	OtA5088-OtA5092	OtA5093-OtA5097
Lat2	chr5:134600264-134615023	OtA5088-OtA5092	OtA5093-OtA5097
Ndufa3	chr7:3617372-3620161	OtA5088-OtA5092	OtA5093-OtA5097
Zbtb38	chr9:96685422-96752831	OtA5088-OtA5092	OtA5093-OtA5097
Ankrd13c	chr3:157947465-158006837	OtA5088-OtA5092	OtA5093-OtA5097
Dclre1b	chr3:103800604-103809387	OtA5088-OtA5092	OtA5093-OtA5097

Anxa8	chr14:34051129-34102754	OtA5088-OtA5092	OtA5093-OtA5097
Mapk1	chr16:16983381-17047453	OtA5088-OtA5092	OtA5093-OtA5097
Ppp1r3f	chrX:7558561-7605420	OtA5088-OtA5092	OtA5093-OtA5097
Senp5	chr16:31959669-32003287	OtA5088-OtA5092	OtA5093-OtA5097
Snapc2	chr8:4253101-4256220	OtA5088-OtA5092	OtA5093-OtA5097
Tex10	chr4:48279801-48473422	OtA5088-OtA5092	OtA5093-OtA5097
Zmym3	chrX:101404383-101420685	OtA5088-OtA5092	OtA5093-OtA5097
Rngtt	chr4:33310310-33502610	OtA5088-OtA5092	OtA5093-OtA5097
Cts3	chr13:61564629-61570127	OtA5088-OtA5092	OtA5093-OtA5097
Mrpl44	chr1:79776017-79781445	OtA5088-OtA5092	OtA5093-OtA5097
Ywhag	chr5:135908378-135934641	OtA5088-OtA5092	OtA5093-OtA5097
Gstt3	chr10:75774121-75781414	OtA5088-OtA5092	OtA5093-OtA5097
Gypc	chr18:32528319-32560034	OtA5088-OtA5092	OtA5093-OtA5097
Arsi	chr18:60912239-60917768	OtA5088-OtA5092	OtA5093-OtA5097
Blvrb	chr7:27447977-27465981	OtA5088-OtA5092	OtA5093-OtA5097
Idua	chr5:108669105-108684558	OtA5088-OtA5092	OtA5093-OtA5097
Clps	chr17:28558213-28560714	OtA5088-OtA5092	OtA5093-OtA5097
Nuak2	chr1:132316124-132333488	OtA5088-OtA5092	OtA5093-OtA5097
Tcf15	chr2:152143608-152149096	OtA5088-OtA5092	OtA5093-OtA5097
Vil1	chr1:74409383-74588028	OtA5088-OtA5092	OtA5093-OtA5097
Asf1b	chr8:83955693-83970195	OtA5088-OtA5092	OtA5093-OtA5097
Znrf3	chr11:5276328-5444847	OtA5088-OtA5092	OtA5093-OtA5097
Gm1821	chr14:46084026-46084957	OtA5088-OtA5092	OtA5093-OtA5097
Igbp1	chrX:100494290-100516125	OtA5088-OtA5092	OtA5093-OtA5097
Mrpl21	chr19:3283046-3292837	OtA5088-OtA5092	OtA5093-OtA5097
Pcna	chr2:132249285-132253180	OtA5088-OtA5092	OtA5093-OtA5097
Phf17	chr3:41555733-41616864	OtA5088-OtA5092	OtA5093-OtA5097
Syk	chr13:52583436-52648792	OtA5088-OtA5092	OtA5093-OtA5097
Txn2	chr15:77915050-77928994	OtA5088-OtA5092	OtA5093-OtA5097
Fam113a	chr2:130417683-130444269	OtA5088-OtA5092	OtA5093-OtA5097
Nnat	chr2:157556361-157566361	OtA5088-OtA5092	OtA5093-OtA5097
Ppp1r12c	chr7:4481520-4501680	OtA5088-OtA5092	OtA5093-OtA5097
Psma1	chr7:114264549-114276116	OtA5088-OtA5092	OtA5093-OtA5097
Rprml	chr11:103649508-103650580	OtA5088-OtA5092	OtA5093-OtA5097
Sorcs2	chr5:36017180-36398139	OtA5088-OtA5092	OtA5093-OtA5097
Etf1	chr18:34902784-34932003	OtA5088-OtA5092	OtA5093-OtA5097
Fzd6	chr15:39006279-39038190	OtA5088-OtA5092	OtA5093-OtA5097
Pcbp1	chr6:86524496-86526165	OtA5088-OtA5092	OtA5093-OtA5097
Psmb9	chr17:34182098-34187330	OtA5088-OtA5092	OtA5093-OtA5097
Sik1	chr17:31844249-31855792	OtA5088-OtA5092	OtA5093-OtA5097
Tada2b	chr5:36465184-36484285	OtA5088-OtA5092	OtA5093-OtA5097
Tomm40l	chr1:171213969-171222514	OtA5088-OtA5092	OtA5093-OtA5097
Psmc5	chr11:106256184-106263112	OtA5088-OtA5092	OtA5093-OtA5097
Lpgat1	chr1:191718023-191784257	OtA5088-OtA5092	OtA5093-OtA5097
Zbtb37	chr1:161017755-161034259	OtA5088-OtA5092	OtA5093-OtA5097
Ccnd3	chr17:47505050-47611492	OtA5088-OtA5092	OtA5093-OtA5097
Mcoln1	chr8:3500518-3515231	OtA5088-OtA5092	OtA5093-OtA5097

Cpsf6	chr10:117344667-117376973	OtA5088-OtA5092	OtA5093-OtA5097
Cox6b1	chr7:30616973-30626151	OtA5088-OtA5092	OtA5093-OtA5097
St6galnac2	chr11:116676704-116694660	OtA5088-OtA5092	OtA5093-OtA5097
Cnpy3	chr17:46735712-46752212	OtA5088-OtA5092	OtA5093-OtA5097
L2hgdh	chr12:69690435-69724874	OtA5088-OtA5092	OtA5093-OtA5097
Nipsnap3b	chr4:53011923-53022059	OtA5088-OtA5092	OtA5093-OtA5097
Pten	chr19:32757576-32826160	OtA5088-OtA5092	OtA5093-OtA5097
Apip	chr2:103073674-103092649	OtA5088-OtA5092	OtA5093-OtA5097
Arih2	chr9:108602942-108649380	OtA5088-OtA5092	OtA5093-OtA5097
Ccny	chr18:9314043-9450150	OtA5088-OtA5092	OtA5093-OtA5097
Cnih	chr14:46775567-46788357	OtA5088-OtA5092	OtA5093-OtA5097
Eci1	chr17:24426682-24439316	OtA5088-OtA5092	OtA5093-OtA5097
Fzd2	chr11:102604430-102608058	OtA5088-OtA5092	OtA5093-OtA5097
Grsf1	chr5:88659236-88676171	OtA5088-OtA5092	OtA5093-OtA5097
Hsd17b7	chr1:169949536-169969205	OtA5088-OtA5092	OtA5093-OtA5097
Lrrk1	chr7:66258746-66388341	OtA5088-OtA5092	OtA5093-OtA5097
Psph	chr5:129765557-129787253	OtA5088-OtA5092	OtA5093-OtA5097
Spryd4	chr10:128194634-128211794	OtA5088-OtA5092	OtA5093-OtA5097
Tef	chr15:81802672-81826863	OtA5088-OtA5092	OtA5093-OtA5097
Traf3	chr12:111166547-111267148	OtA5088-OtA5092	OtA5093-OtA5097
Ubac1	chr2:25996957-26021760	OtA5088-OtA5092	OtA5093-OtA5097
Ubqln2	chrX:153498231-153501558	OtA5088-OtA5092	OtA5093-OtA5097
Ufc1	chr1:171288563-171294982	OtA5088-OtA5092	OtA5093-OtA5097
Magoh	chr4:107879812-107887398	OtA5088-OtA5092	OtA5093-OtA5097
Psma6	chr12:55280813-55492647	OtA5088-OtA5092	OtA5093-OtA5097
Pan3	chr5:147430579-147548501	OtA5088-OtA5092	OtA5093-OtA5097
Zfp398	chr6:47835660-47868257	OtA5088-OtA5092	OtA5093-OtA5097
Xrn2	chr2:147013059-147077997	OtA5088-OtA5092	OtA5093-OtA5097
Adap2	chr11:80154161-80178827	OtA5088-OtA5092	OtA5093-OtA5097
Arhgdia	chr11:120577234-120581620	OtA5088-OtA5092	OtA5093-OtA5097
Capn8	chr1:182565006-182632352	OtA5088-OtA5092	OtA5093-OtA5097
Dhx35	chr2:158794819-158858214	OtA5088-OtA5092	OtA5093-OtA5097
G3bp2	chr5:92052145-92083735	OtA5088-OtA5092	OtA5093-OtA5097
Junb	chr8:84976908-84978748	OtA5088-OtA5092	OtA5093-OtA5097
Mob4	chr1:55131244-55154899	OtA5088-OtA5092	OtA5093-OtA5097
Pcdh7	chr5:57718020-58132240	OtA5088-OtA5092	OtA5093-OtA5097
Prkra	chr2:76629936-76647994	OtA5088-OtA5092	OtA5093-OtA5097
Prkrip1	chr5:136180356-136198954	OtA5088-OtA5092	OtA5093-OtA5097
Rbm34	chr8:126947172-126971079	OtA5088-OtA5092	OtA5093-OtA5097
Shroom2	chrX:152609508-152769461	OtA5088-OtA5092	OtA5093-OtA5097
Skp1a	chr11:52231994-52246858	OtA5088-OtA5092	OtA5093-OtA5097
Slc1a4	chr11:20302179-20332713	OtA5088-OtA5092	OtA5093-OtA5097
Slc44a4	chr17:34914465-34930436	OtA5088-OtA5092	OtA5093-OtA5097
Spata5	chr3:37420279-37579095	OtA5088-OtA5092	OtA5093-OtA5097
Sufu	chr19:46396917-46486660	OtA5088-OtA5092	OtA5093-OtA5097
Tubgcp5	chr7:55794147-55831447	OtA5088-OtA5092	OtA5093-OtA5097
Zbed4	chr15:88751710-88784516	OtA5088-OtA5092	OtA5093-OtA5097

Avpi1	chr19:42123274-42128993	OtA5088-OtA5092	OtA5093-OtA5097
Mrpl45	chr11:97315715-97329920	OtA5088-OtA5092	OtA5093-OtA5097
Parp9	chr16:35926514-35972621	OtA5088-OtA5092	OtA5093-OtA5097
Pctp	chr11:89983416-90002894	OtA5088-OtA5092	OtA5093-OtA5097
Smek2	chr11:29172906-29220797	OtA5088-OtA5092	OtA5093-OtA5097
Tnks2	chr19:36834231-36893477	OtA5088-OtA5092	OtA5093-OtA5097
Chmp1a	chr8:123204260-123212788	OtA5088-OtA5092	OtA5093-OtA5097
Ddx49	chr8:70282998-70302452	OtA5088-OtA5092	OtA5093-OtA5097
Arrb1	chr7:99535485-99606771	OtA5088-OtA5092	OtA5093-OtA5097
C1rl	chr6:124493112-124510643	OtA5088-OtA5092	OtA5093-OtA5097
C80913	chr7:37959991-38019552	OtA5088-OtA5092	OtA5093-OtA5097
Ccdc25	chr14:65837301-65866604	OtA5088-OtA5092	OtA5093-OtA5097
Cd3eap	chr7:19345070-19359483	OtA5088-OtA5092	OtA5093-OtA5097
Cdc27	chr11:104502525-104550620	OtA5088-OtA5092	OtA5093-OtA5097
Coq6	chr12:84361967-84373796	OtA5088-OtA5092	OtA5093-OtA5097
Gm1673	chr5:33983473-33985006	OtA5088-OtA5092	OtA5093-OtA5097
Habp2	chr19:56287937-56390035	OtA5088-OtA5092	OtA5093-OtA5097
Klhdc2	chr12:69296680-69310687	OtA5088-OtA5092	OtA5093-OtA5097
Mrps9	chr1:42851232-42905683	OtA5088-OtA5092	OtA5093-OtA5097
Ncstn	chr1:172066012-172122332	OtA5088-OtA5092	OtA5093-OtA5097
Nras	chr3:103058284-103067914	OtA5088-OtA5092	OtA5093-OtA5097
Plekhg5	chr4:152096718-152115400	OtA5088-OtA5092	OtA5093-OtA5097
Rfx5	chr3:94955014-94961561	OtA5088-OtA5092	OtA5093-OtA5097
Slc30a9	chr5:67306956-67356145	OtA5088-OtA5092	OtA5093-OtA5097
Slc38a2	chr15:96687391-96699698	OtA5088-OtA5092	OtA5093-OtA5097
Snrpb2	chr2:143063068-143072052	OtA5088-OtA5092	OtA5093-OtA5097
Tmem144	chr3:79813152-79842662	OtA5088-OtA5092	OtA5093-OtA5097
Vdac3	chr8:22577074-22593813	OtA5088-OtA5092	OtA5093-OtA5097
Vhl	chr6:113624020-113631633	OtA5088-OtA5092	OtA5093-OtA5097
Wbp7	chr7:30568854-30588726	OtA5088-OtA5092	OtA5093-OtA5097
Wt1	chr2:105126528-105173614	OtA5088-OtA5092	OtA5093-OtA5097
Nme4	chr17:26091744-26095470	OtA5088-OtA5092	OtA5093-OtA5097
Gnb4	chr3:32583527-32616535	OtA5088-OtA5092	OtA5093-OtA5097
Zyg11a	chr4:108181933-108217922	OtA5088-OtA5092	OtA5093-OtA5097
Arsb	chr13:93771678-93943016	OtA5088-OtA5092	OtA5093-OtA5097
Cdk17	chr10:93160875-93241342	OtA5088-OtA5092	OtA5093-OtA5097
Rpl29	chr9:106429538-106431567	OtA5088-OtA5092	OtA5093-OtA5097
Ccdc22	chrX:7558561-7605420	OtA5088-OtA5092	OtA5093-OtA5097
Tspan14	chr14:40906443-40966807	OtA5088-OtA5092	OtA5093-OtA5097
Araf	chrX:20848542-20920918	OtA5088-OtA5092	OtA5093-OtA5097
Txndc9	chr1:37983866-37997208	OtA5088-OtA5092	OtA5093-OtA5097
Ahdc1	chr4:133011505-133078110	OtA5088-OtA5092	OtA5093-OtA5097
Ager	chr17:34597861-34600932	OtA5088-OtA5092	OtA5093-OtA5097
Ccdc127	chr13:74350316-74365783	OtA5088-OtA5092	OtA5093-OtA5097
Cry2	chr2:92403646-92434069	OtA5088-OtA5092	OtA5093-OtA5097
Pdk3	chrX:93764615-93832095	OtA5088-OtA5092	OtA5093-OtA5097
Stard3nl	chr13:19357675-19395752	OtA5088-OtA5092	OtA5093-OtA5097

Laptm4b	chr15:34238025-34284295	OtA5088-OtA5092	OtA5093-OtA5097
Rtf1	chr2:119675067-119735407	OtA5088-OtA5092	OtA5093-OtA5097
Nup62cl	chrX:140007672-140062568	OtA5088-OtA5092	OtA5093-OtA5097
Ap1ar	chr3:127807264-127837492	OtA5088-OtA5092	OtA5093-OtA5097
Dsg2	chr18:20558115-20604526	OtA5088-OtA5092	OtA5093-OtA5097
Mtmr1	chrX:71364759-71419196	OtA5088-OtA5092	OtA5093-OtA5097
Hddc3	chr7:80343136-80346097	OtA5088-OtA5092	OtA5093-OtA5097
Msx2	chr13:53466880-53472780	OtA5088-OtA5092	OtA5093-OtA5097
Cr1l	chr1:195103787-195131570	OtA5088-OtA5092	OtA5093-OtA5097
Cyb5d1	chr11:69393611-69395346	OtA5088-OtA5092	OtA5093-OtA5097
Pacsin2	chr15:83375606-83464606	OtA5088-OtA5092	OtA5093-OtA5097
Rnf181	chr6:72355482-72362381	OtA5088-OtA5092	OtA5093-OtA5097
Cd2ap	chr17:42792950-42876424	OtA5088-OtA5092	OtA5093-OtA5097
Ero1l	chr14:45283086-45318572	OtA5088-OtA5092	OtA5093-OtA5097
Il28ra	chr4:135686456-135708180	OtA5088-OtA5092	OtA5093-OtA5097
Isg15	chr4:156199423-156200818	OtA5088-OtA5092	OtA5093-OtA5097
Maff	chr15:79347677-79359076	OtA5088-OtA5092	OtA5093-OtA5097
Abca1	chr4:53030788-53159895	OtA5088-OtA5092	OtA5093-OtA5097
Chaf1a	chr17:56040415-56068026	OtA5088-OtA5092	OtA5093-OtA5097
Dda1	chr8:71469193-71486067	OtA5088-OtA5092	OtA5093-OtA5097
Asb11	chrX:164438100-164459100	OtA5088-OtA5092	OtA5093-OtA5097
Neurl4	chr11:69901877-69913822	OtA5088-OtA5092	OtA5093-OtA5097
Taf3	chr2:9914551-10048609	OtA5088-OtA5092	OtA5093-OtA5097
Itga6	chr2:71787082-71856758	OtA5088-OtA5092	OtA5093-OtA5097
Unc5cl	chr17:48454900-48468684	OtA5088-OtA5092	OtA5093-OtA5097
Pip5k1c	chr10:81292971-81319974	OtA5088-OtA5092	OtA5093-OtA5097
Bsg	chr10:79697304-80369637	OtA5088-OtA5092	OtA5093-OtA5097
Cdk5rap1	chr2:154335385-154372719	OtA5088-OtA5092	OtA5093-OtA5097
Cnbp	chr6:87842614-87851106	OtA5088-OtA5092	OtA5093-OtA5097
Dmkn	chr7:30763755-30781066	OtA5088-OtA5092	OtA5093-OtA5097
Fam58b	chr11:78750505-78751729	OtA5088-OtA5092	OtA5093-OtA5097
Fryl	chr5:73020190-73256618	OtA5088-OtA5092	OtA5093-OtA5097
Hibch	chr1:52845045-52920860	OtA5088-OtA5092	OtA5093-OtA5097
Per1	chr11:69098955-69109957	OtA5088-OtA5092	OtA5093-OtA5097
Polr3c	chr3:96711894-96727439	OtA5088-OtA5092	OtA5093-OtA5097
Smyd2	chr1:189880491-189922288	OtA5088-OtA5092	OtA5093-OtA5097
Tbc1d14	chr5:36490603-36586226	OtA5088-OtA5092	OtA5093-OtA5097
Trappc10	chr10:78186725-78244642	OtA5088-OtA5092	OtA5093-OtA5097
Ank2	chr3:126921606-126998452	OtA5088-OtA5092	OtA5093-OtA5097
Dip2b	chr15:100038663-100219473	OtA5088-OtA5092	OtA5093-OtA5097
Pex6	chr17:46711462-46725541	OtA5088-OtA5092	OtA5093-OtA5097
Eno3	chr11:70657212-70662513	OtA5088-OtA5092	OtA5093-OtA5097
Trp53i13	chr11:77508098-77513273	OtA5088-OtA5092	OtA5093-OtA5097
Mrpl38	chr11:116131816-116138868	OtA5088-OtA5092	OtA5093-OtA5097
Cilp2	chr8:69880365-69887392	OtA5088-OtA5092	OtA5093-OtA5097
Smad1	chr8:79338397-79399468	OtA5088-OtA5092	OtA5093-OtA5097
Anapc1	chr2:128610082-128687395	OtA5088-OtA5092	OtA5093-OtA5097

Tdp1	chr12:99884514-99955216	OtA5088-OtA5092	OtA5093-OtA5097
BC037034	chr5:138259657-138272754	OtA5088-OtA5092	OtA5093-OtA5097
Cdh11	chr8:102632994-102785111	OtA5088-OtA5092	OtA5093-OtA5097
Ccnt1	chr15:98543210-98570864	OtA5088-OtA5092	OtA5093-OtA5097
Eif5a	chr11:69916711-69921958	OtA5088-OtA5092	OtA5093-OtA5097
Lrp3	chr7:35186384-35215345	OtA5088-OtA5092	OtA5093-OtA5097
Mrps28	chr3:8802145-8923857	OtA5088-OtA5092	OtA5093-OtA5097
Rnf114	chr2:167481135-167516166	OtA5088-OtA5092	OtA5093-OtA5097
Tomm70a	chr16:57121713-57154530	OtA5088-OtA5092	OtA5093-OtA5097
Spata6	chr4:111720009-111829140	OtA5088-OtA5092	OtA5093-OtA5097
Triap1	chr5:115341246-115343552	OtA5088-OtA5092	OtA5093-OtA5097
Urb2	chr8:124023472-124048504	OtA5088-OtA5092	OtA5093-OtA5097
Acox1	chr11:116171887-116215318	OtA5088-OtA5092	OtA5093-OtA5097
Nat10	chr2:103721258-103761250	OtA5088-OtA5092	OtA5093-OtA5097
Robo4	chr9:37402045-37414022	OtA5088-OtA5092	OtA5093-OtA5097
Angptl4	chr17:33774899-33781575	OtA5088-OtA5092	OtA5093-OtA5097
Fam60a	chr6:148921058-148946432	OtA5088-OtA5092	OtA5093-OtA5097
Il11ra1	chr4:41760442-41774176	OtA5088-OtA5092	OtA5093-OtA5097
Sar1b	chr11:51763662-51791953	OtA5088-OtA5092	OtA5093-OtA5097
Tjp3	chr10:81268171-81291267	OtA5088-OtA5092	OtA5093-OtA5097
Isca2	chr12:84773269-84775089	OtA5088-OtA5092	OtA5093-OtA5097
Tmem93	chr11:73175502-73177042	OtA5088-OtA5092	OtA5093-OtA5097
Met	chr6:17463956-17573980	OtA5088-OtA5092	OtA5093-OtA5097
Nrgn	chr9:37544492-37552745	OtA5088-OtA5092	OtA5093-OtA5097
Smtn	chr11:3517521-3539292	OtA5088-OtA5092	OtA5093-OtA5097
Adprhl2	chr4:126316350-126321703	OtA5088-OtA5092	OtA5093-OtA5097
Cyb5r1	chr1:134405989-134411738	OtA5088-OtA5092	OtA5093-OtA5097
Prelp	chr1:133910303-133921401	OtA5088-OtA5092	OtA5093-OtA5097
Zfp703	chr8:26977335-26981462	OtA5088-OtA5092	OtA5093-OtA5097
Cisd1	chr10:71330493-71344849	OtA5088-OtA5092	OtA5093-OtA5097
Slx4	chr16:3979122-4001680	OtA5088-OtA5092	OtA5093-OtA5097
Abhd2	chr7:79273265-79361601	OtA5088-OtA5092	OtA5093-OtA5097
Atp5f1	chr3:105942862-105969760	OtA5088-OtA5092	OtA5093-OtA5097
B230219D22Rik	chr13:55693123-55703500	OtA5088-OtA5092	OtA5093-OtA5097
Brpf1	chr6:113307196-113324704	OtA5088-OtA5092	OtA5093-OtA5097
Ccndbp1	chr2:121008407-121016912	OtA5088-OtA5092	OtA5093-OtA5097
Chst12	chr5:140505608-140525238	OtA5088-OtA5092	OtA5093-OtA5097
Ctxn1	chr8:4257645-4259274	OtA5088-OtA5092	OtA5093-OtA5097
Ftl1	chr7:45457943-45459886	OtA5088-OtA5092	OtA5093-OtA5097
Gdf15	chr8:70629393-70631635	OtA5088-OtA5092	OtA5093-OtA5097
Kcnj13	chr1:87326997-87450810	OtA5088-OtA5092	OtA5093-OtA5097
Larp4b	chr13:9093904-9173090	OtA5088-OtA5092	OtA5093-OtA5097
Lmo4	chr3:144188529-144205255	OtA5088-OtA5092	OtA5093-OtA5097
Man1a2	chr3:100562204-100685473	OtA5088-OtA5092	OtA5093-OtA5097
Poglut1	chr16:38525179-38550184	OtA5088-OtA5092	OtA5093-OtA5097
Rmnd1	chr10:4403168-4432352	OtA5088-OtA5092	OtA5093-OtA5097
Slc35a2	chrX:7884243-7894492	OtA5088-OtA5092	OtA5093-OtA5097

Slc39a6	chr18:24579880-24603817	OtA5088-OtA5092	OtA5093-OtA5097
Sp6	chr11:97013568-97024738	OtA5088-OtA5092	OtA5093-OtA5097
Tpk1	chr6:43345006-43666175	OtA5088-OtA5092	OtA5093-OtA5097
Higd1a	chr9:121848559-121858000	OtA5088-OtA5092	OtA5093-OtA5097
Rbbp6	chr7:122970563-123002572	OtA5088-OtA5092	OtA5093-OtA5097
Armcx1	chrX:134717937-134721912	OtA5088-OtA5092	OtA5093-OtA5097
Galntl1	chr12:80518989-80603896	OtA5088-OtA5092	OtA5093-OtA5097
Stat5a	chr11:100859350-100885169	OtA5088-OtA5092	OtA5093-OtA5097
Hsbp1	chr8:119344537-119348929	OtA5088-OtA5092	OtA5093-OtA5097
Pcgf2	chr11:97688822-97700497	OtA5088-OtA5092	OtA5093-OtA5097
Gstt2	chr10:75831844-75834881	OtA5088-OtA5092	OtA5093-OtA5097
Mamdc2	chr19:23302608-23448322	OtA5088-OtA5092	OtA5093-OtA5097
Atg5	chr10:44268357-44364291	OtA5088-OtA5092	OtA5093-OtA5097
Klhdc4	chr8:121796307-121829569	OtA5088-OtA5092	OtA5093-OtA5097
Reps1	chr10:18055939-18125155	OtA5088-OtA5092	OtA5093-OtA5097
Dnajc3	chr14:118937931-118981702	OtA5088-OtA5092	OtA5093-OtA5097
Stard10	chr7:101321318-101346312	OtA5088-OtA5092	OtA5093-OtA5097
Tprgl	chr4:154157484-154160684	OtA5088-OtA5092	OtA5093-OtA5097
Ints10	chr8:68793953-68827644	OtA5088-OtA5092	OtA5093-OtA5097
Nup85	chr11:115564443-115583924	OtA5088-OtA5092	OtA5093-OtA5097
Yipf4	chr17:74489492-74500277	OtA5088-OtA5092	OtA5093-OtA5097
Alas1	chr9:106233454-106247954	OtA5088-OtA5092	OtA5093-OtA5097
Txnl4a	chr18:80206797-80225851	OtA5088-OtA5092	OtA5093-OtA5097
Oaz2	chr9:65676547-65690300	OtA5088-OtA5092	OtA5093-OtA5097
Etfa	chr9:55454435-55512243	OtA5088-OtA5092	OtA5093-OtA5097
Rg9mtd3	chr4:45297167-45316131	OtA5088-OtA5092	OtA5093-OtA5097
Rpl13	chr8:123102349-123105242	OtA5088-OtA5092	OtA5093-OtA5097
Fam116a	chr14:26579549-26632099	OtA5088-OtA5092	OtA5093-OtA5097
Fam83e	chr7:45721219-45729492	OtA5088-OtA5092	OtA5093-OtA5097
Sdc1	chr12:8771395-8793687	OtA5088-OtA5092	OtA5093-OtA5097
Hmgxb4	chr8:74993702-75031972	OtA5088-OtA5092	OtA5093-OtA5097
Pex11b	chr3:96635356-96645381	OtA5088-OtA5092	OtA5093-OtA5097
Abcf3	chr16:20548602-20561603	OtA5088-OtA5092	OtA5093-OtA5097
Abcg1	chr17:31057693-31117981	OtA5088-OtA5092	OtA5093-OtA5097
Akap13	chr7:75455533-75754609	OtA5088-OtA5092	OtA5093-OtA5097
Akr1a1	chr4:116636509-116651674	OtA5088-OtA5092	OtA5093-OtA5097
Akt1s1	chr7:44849223-44855421	OtA5088-OtA5092	OtA5093-OtA5097
Alg8	chr7:97371616-97392158	OtA5088-OtA5092	OtA5093-OtA5097
Ankrd24	chr10:81628539-81647612	OtA5088-OtA5092	OtA5093-OtA5097
Api5	chr2:94411726-94438147	OtA5088-OtA5092	OtA5093-OtA5097
Bmi1	chr2:18677017-18686629	OtA5088-OtA5092	OtA5093-OtA5097
Bnip3l	chr14:66985239-67008877	OtA5088-OtA5092	OtA5093-OtA5097
Cbr3	chr16:93683218-93690991	OtA5088-OtA5092	OtA5093-OtA5097
Ccdc45	chr11:106789251-106818861	OtA5088-OtA5092	OtA5093-OtA5097
Cdk10	chr8:123224840-123232256	OtA5088-OtA5092	OtA5093-OtA5097
Cdk7	chr13:100669480-100730942	OtA5088-OtA5092	OtA5093-OtA5097
Cdkn2b	chr4:89306288-89311032	OtA5088-OtA5092	OtA5093-OtA5097

Cecr5	chr6:120509493-120531299	OtA5088-OtA5092	OtA5093-OtA5097
Cinp	chr12:110872609-110889145	OtA5088-OtA5092	OtA5093-OtA5097
Cnot2	chr10:116485160-116581511	OtA5088-OtA5092	OtA5093-OtA5097
Coq3	chr4:21879674-21912126	OtA5088-OtA5092	OtA5093-OtA5097
Cx3cr1	chr9:120048692-120068282	OtA5088-OtA5092	OtA5093-OtA5097
Cxcl10	chr5:92331840-92414627	OtA5088-OtA5092	OtA5093-OtA5097
Cyth3	chr5:143622446-143710248	OtA5088-OtA5092	OtA5093-OtA5097
Dnajc4	chr19:6987910-6992272	OtA5088-OtA5092	OtA5093-OtA5097
Dpagt1	chr9:44326844-44333600	OtA5088-OtA5092	OtA5093-OtA5097
Ear2	chr14:44102653-44103531	OtA5088-OtA5092	OtA5093-OtA5097
Eefsec	chr6:88257333-88446539	OtA5088-OtA5092	OtA5093-OtA5097
Elovl1	chr4:118428092-118437343	OtA5088-OtA5092	OtA5093-OtA5097
Erlin2	chr8:27023798-27039435	OtA5088-OtA5092	OtA5093-OtA5097
Ets2	chr16:95702406-95721049	OtA5088-OtA5092	OtA5093-OtA5097
Evpl	chr11:116220558-116238091	OtA5088-OtA5092	OtA5093-OtA5097
Fam192a	chr8:94574940-94601726	OtA5088-OtA5092	OtA5093-OtA5097
Fkbp1b	chr12:4833173-4841595	OtA5088-OtA5092	OtA5093-OtA5097
Galt	chr4:41755252-41759125	OtA5088-OtA5092	OtA5093-OtA5097
Gar1	chr3:129824911-129831396	OtA5088-OtA5092	OtA5093-OtA5097
Ghdc	chr11:100766331-100770957	OtA5088-OtA5092	OtA5093-OtA5097
Gja5	chr3:97032415-97053634	OtA5088-OtA5092	OtA5093-OtA5097
Gpr107	chr2:31152315-31216567	OtA5088-OtA5092	OtA5093-OtA5097
Gpr161	chr1:165295765-165321851	OtA5088-OtA5092	OtA5093-OtA5097
Hiatl1	chr13:65065029-65112982	OtA5088-OtA5092	OtA5093-OtA5097
Hnrnpab	chr11:51584756-51606881	OtA5088-OtA5092	OtA5093-OtA5097
Ift52	chr2:163017471-163046135	OtA5088-OtA5092	OtA5093-OtA5097
Inf2	chr12:112588783-112615557	OtA5088-OtA5092	OtA5093-OtA5097
Klhl11	chr11:100462611-100472782	OtA5088-OtA5092	OtA5093-OtA5097
Ksr1	chr11:79014800-79146354	OtA5088-OtA5092	OtA5093-OtA5097
Lrig2	chr3:104453982-104511856	OtA5088-OtA5092	OtA5093-OtA5097
6-Mar	chr15:31455898-31531037	OtA5088-OtA5092	OtA5093-OtA5097
Med18	chr4:132458728-132463921	OtA5088-OtA5092	OtA5093-OtA5097
Mrpl13	chr15:55534094-55557312	OtA5088-OtA5092	OtA5093-OtA5097
Mrps18b	chr17:35910384-35916369	OtA5088-OtA5092	OtA5093-OtA5097
Myeov2	chr1:92637144-92641985	OtA5088-OtA5092	OtA5093-OtA5097
Myl12a	chr17:70993792-71002533	OtA5088-OtA5092	OtA5093-OtA5097
Nae1	chr8:104446718-104624396	OtA5088-OtA5092	OtA5093-OtA5097
Nolc1	chr19:46075862-46085531	OtA5088-OtA5092	OtA5093-OtA5097
Nrarp	chr2:25180757-25183332	OtA5088-OtA5092	OtA5093-OtA5097
Oxsm	chr14:16238658-16311926	OtA5088-OtA5092	OtA5093-OtA5097
Pafah1b2	chr9:45965310-45984871	OtA5088-OtA5092	OtA5093-OtA5097
Pask	chr1:93309436-93342788	OtA5088-OtA5092	OtA5093-OtA5097
Phlpp2	chr8:109868602-109944671	OtA5088-OtA5092	OtA5093-OtA5097
Pisd-ps2	chr17:3064317-3084183	OtA5088-OtA5092	OtA5093-OtA5097
Pld3	chr7:27532017-27553112	OtA5088-OtA5092	OtA5093-OtA5097
Pmvk	chr3:89459117-89469009	OtA5088-OtA5092	OtA5093-OtA5097
Ppp3r1	chr11:17159297-17200380	OtA5088-OtA5092	OtA5093-OtA5097

Prl2a1	chr13:27801654-27808716	OtA5088-OtA5092	OtA5093-OtA5097
Prps1l3	chr12:57230411-57457241	OtA5088-OtA5092	OtA5093-OtA5097
Prss12	chr3:123446912-123506602	OtA5088-OtA5092	OtA5093-OtA5097
Prss23	chr7:89507784-89517586	OtA5088-OtA5092	OtA5093-OtA5097
Ptcd3	chr6:71880637-71908762	OtA5088-OtA5092	OtA5093-OtA5097
Pttg1	chr11:43420247-43426248	OtA5088-OtA5092	OtA5093-OtA5097
Rap2b	chr3:61364506-61368703	OtA5088-OtA5092	OtA5093-OtA5097
Rhoq	chr17:86963110-87025401	OtA5088-OtA5092	OtA5093-OtA5097
Riok1	chr13:38036988-38061433	OtA5088-OtA5092	OtA5093-OtA5097
Rpf2	chr10:40223245-40247039	OtA5088-OtA5092	OtA5093-OtA5097
Rps23	chr13:90923121-90924732	OtA5088-OtA5092	OtA5093-OtA5097
Scg5	chr2:113776312-113829091	OtA5088-OtA5092	OtA5093-OtA5097
Sec61a1	chr6:88503606-88518800	OtA5088-OtA5092	OtA5093-OtA5097
Sfmbt2	chr2:10370450-10595253	OtA5088-OtA5092	OtA5093-OtA5097
Sh3yl1	chr12:30911668-30960149	OtA5088-OtA5092	OtA5093-OtA5097
Ski	chr4:155154074-155222535	OtA5088-OtA5092	OtA5093-OtA5097
Slain2	chr5:72914316-72978828	OtA5088-OtA5092	OtA5093-OtA5097
Snrpe	chr1:133603870-133610280	OtA5088-OtA5092	OtA5093-OtA5097
Snx12	chrX:101097785-101222563	OtA5088-OtA5092	OtA5093-OtA5097
Sugp1	chr8:70042812-70071953	OtA5088-OtA5092	OtA5093-OtA5097
Tab1	chr15:80133153-80161702	OtA5088-OtA5092	OtA5093-OtA5097
Tdo2	chr3:81958413-81975728	OtA5088-OtA5092	OtA5093-OtA5097
Tiam2	chr17:3326572-3557713	OtA5088-OtA5092	OtA5093-OtA5097
Tmed7	chr18:46585927-46597535	OtA5088-OtA5092	OtA5093-OtA5097
Tmem39b	chr4:129676354-129696838	OtA5088-OtA5092	OtA5093-OtA5097
Tmppe	chr9:114401094-114474377	OtA5088-OtA5092	OtA5093-OtA5097
Trpc4ap	chr2:155611211-155692384	OtA5088-OtA5092	OtA5093-OtA5097
Usp39	chr6:72318675-72345175	OtA5088-OtA5092	OtA5093-OtA5097
Zc3hav1	chr6:38310496-38354603	OtA5088-OtA5092	OtA5093-OtA5097
Zdhhc21	chr4:82798737-82859661	OtA5088-OtA5092	OtA5093-OtA5097
Zfp27	chr7:29893336-29906104	OtA5088-OtA5092	OtA5093-OtA5097
Zfp579	chr7:4992851-4996101	OtA5088-OtA5092	OtA5093-OtA5097
Zfp956	chr6:47943174-47965299	OtA5088-OtA5092	OtA5093-OtA5097
Acly	chr11:100476351-100528000	OtA5088-OtA5092	OtA5093-OtA5097
Dtwd2	chr18:49696144-49755601	OtA5088-OtA5092	OtA5093-OtA5097
Gstz1	chr12:87106865-87164723	OtA5088-OtA5092	OtA5093-OtA5097
Tspan4	chr7:141475235-141539857	OtA5088-OtA5092	OtA5093-OtA5097
Actl6a	chr3:32708545-32726971	OtA5088-OtA5092	OtA5093-OtA5097
Hist1h2ag	chr13:22042476-22042949	OtA5088-OtA5092	OtA5093-OtA5097
Ogt	chrX:101640063-101684351	OtA5088-OtA5092	OtA5093-OtA5097
Dym	chr18:75018771-75286966	OtA5088-OtA5092	OtA5093-OtA5097
Matr3	chr18:35562157-35592045	OtA5088-OtA5092	OtA5093-OtA5097
Trip4	chr9:65828925-65908794	OtA5088-OtA5092	OtA5093-OtA5097
Mecr	chr4:131843470-131867787	OtA5088-OtA5092	OtA5093-OtA5097
Cox7a1	chr7:30184170-30186030	OtA5088-OtA5092	OtA5093-OtA5097
Frem2	chr3:53513937-53657355	OtA5088-OtA5092	OtA5093-OtA5097
Gprc5c	chr11:114851531-114872617	OtA5088-OtA5092	OtA5093-OtA5097

Mmgt2	chr11:62648663-62666359	OtA5088-OtA5092	OtA5093-OtA5097
Slc20a1	chr2:129198772-129211612	OtA5088-OtA5092	OtA5093-OtA5097
Caprin1	chr2:103762944-103797640	OtA5088-OtA5092	OtA5093-OtA5097
Cbl	chr9:44149261-44234046	OtA5088-OtA5092	OtA5093-OtA5097
Edn2	chr4:120161423-120167360	OtA5088-OtA5092	OtA5093-OtA5097
Fam134a	chr1:75142785-75147909	OtA5088-OtA5092	OtA5093-OtA5097
Ifi30	chr8:70762772-70766663	OtA5088-OtA5092	OtA5093-OtA5097
Ing3	chr6:21949614-21976037	OtA5088-OtA5092	OtA5093-OtA5097
Iqcc	chr4:129615616-129619093	OtA5088-OtA5092	OtA5093-OtA5097
Lamtor2	chr3:88549818-88552927	OtA5088-OtA5092	OtA5093-OtA5097
Lpar6	chr14:73195501-73325791	OtA5088-OtA5092	OtA5093-OtA5097
Mcam	chr9:44134657-44142726	OtA5088-OtA5092	OtA5093-OtA5097
Mmp11	chr10:75923223-75932463	OtA5088-OtA5092	OtA5093-OtA5097
Mrps14	chr1:160195259-160201186	OtA5088-OtA5092	OtA5093-OtA5097
Pyroxd1	chr6:142345696-142387087	OtA5088-OtA5092	OtA5093-OtA5097
Timeless	chr10:128232062-128252941	OtA5088-OtA5092	OtA5093-OtA5097
Tmem161b	chr13:84222295-84295966	OtA5088-OtA5092	OtA5093-OtA5097
Urb1	chr16:90751526-90810413	OtA5088-OtA5092	OtA5093-OtA5097
Yme1l1	chr2:23156504-23199260	OtA5088-OtA5092	OtA5093-OtA5097
Sun2	chr15:79724067-79742536	OtA5088-OtA5092	OtA5093-OtA5097
Snip1	chr4:125066692-125074043	OtA5088-OtA5092	OtA5093-OtA5097
Gtf3c6	chr10:40249202-40257665	OtA5088-OtA5092	OtA5093-OtA5097
Hopx	chr5:77086985-77115123	OtA5088-OtA5092	OtA5093-OtA5097
Lss	chr10:76531626-76562226	OtA5088-OtA5092	OtA5093-OtA5097
Prpf31	chr7:3629984-3642484	OtA5088-OtA5092	OtA5093-OtA5097
Aven	chr2:112492963-112631253	OtA5088-OtA5092	OtA5093-OtA5097
Galk2	chr2:125859217-126146251	OtA5088-OtA5092	OtA5093-OtA5097
Prmt3	chr7:49778357-49858265	OtA5088-OtA5092	OtA5093-OtA5097
Rabif	chr1:134494659-134507884	OtA5088-OtA5092	OtA5093-OtA5097
Wdr20a	chr12:110737948-110795028	OtA5088-OtA5092	OtA5093-OtA5097
Zfp862	chr6:48504338-48534832	OtA5088-OtA5092	OtA5093-OtA5097
Actr3	chr1:125392904-125435727	OtA5088-OtA5092	OtA5093-OtA5097
Dnase2a	chr8:84908623-84911461	OtA5088-OtA5092	OtA5093-OtA5097
Gcnt4	chr13:96924688-96950914	OtA5088-OtA5092	OtA5093-OtA5097
Gm12191	chr15:34440505-34443276	OtA5088-OtA5092	OtA5093-OtA5097
H2-Ke2	chr17:33938908-33940343	OtA5088-OtA5092	OtA5093-OtA5097
Mill2	chr7:18839965-18858653	OtA5088-OtA5092	OtA5093-OtA5097
Trmt5	chr12:73280409-73286711	OtA5088-OtA5092	OtA5093-OtA5097
Zfand2b	chr1:75168645-75171626	OtA5088-OtA5092	OtA5093-OtA5097
Dhcr7	chr7:143823166-143848410	OtA5088-OtA5092	OtA5093-OtA5097
Gadd45gip1	chr8:84832281-84840641	OtA5088-OtA5092	OtA5093-OtA5097
Rad23a	chr8:84832281-84840641	OtA5088-OtA5092	OtA5093-OtA5097
Gltpd1	chr4:155864722-155869440	OtA5088-OtA5092	OtA5093-OtA5097
Mfsd8	chr3:40818171-40846854	OtA5088-OtA5092	OtA5093-OtA5097
Trappc1	chr11:69323985-69325793	OtA5088-OtA5092	OtA5093-OtA5097
Eno2	chr6:124760054-124769509	OtA5088-OtA5092	OtA5093-OtA5097
Itsn1	chr16:91729370-91920579	OtA5088-OtA5092	OtA5093-OtA5097

Plk2	chr13:110395043-110400843	OtA5088-OtA5092	OtA5093-OtA5097
Scfd1	chr12:51377579-51450096	OtA5088-OtA5092	OtA5093-OtA5097
Snx5	chr2:144250123-144270902	OtA5088-OtA5092	OtA5093-OtA5097
Tnfrsf10b	chr14:69767471-69784411	OtA5088-OtA5092	OtA5093-OtA5097
Wdr48	chr9:119894894-119937558	OtA5088-OtA5092	OtA5093-OtA5097
Zfp202	chr9:40192315-40213604	OtA5088-OtA5092	OtA5093-OtA5097
Slc19a3	chr1:83012522-83038448	OtA5088-OtA5092	OtA5093-OtA5097
Arrdc2	chr8:70835137-70839720	OtA5088-OtA5092	OtA5093-OtA5097
Dynlt1a	chr17:6310544-6317474	OtA5088-OtA5092	OtA5093-OtA5097
Scrib	chr15:76047185-76069730	OtA5088-OtA5092	OtA5093-OtA5097
Snapin	chr3:90476200-90491013	OtA5088-OtA5092	OtA5093-OtA5097
Tlr12	chr4:128615445-128618619	OtA5088-OtA5092	OtA5093-OtA5097
Traf7	chr17:24488782-24527938	OtA5088-OtA5092	OtA5093-OtA5097
Cops8	chr1:90603424-90613341	OtA5088-OtA5092	OtA5093-OtA5097
Haus4	chr14:54541784-54554361	OtA5088-OtA5092	OtA5093-OtA5097
Acot11	chr4:106733914-106799831	OtA5088-OtA5092	OtA5093-OtA5097
Ambra1	chr2:91730137-91918849	OtA5088-OtA5092	OtA5093-OtA5097
Btbd1	chr7:81792073-81829431	OtA5088-OtA5092	OtA5093-OtA5097
Ccdc90b	chr7:92561148-92582294	OtA5088-OtA5092	OtA5093-OtA5097
Cd200	chr16:45382134-45409053	OtA5088-OtA5092	OtA5093-OtA5097
Chst7	chrX:20059569-20097520	OtA5088-OtA5092	OtA5093-OtA5097
Eps15l1	chr8:72340996-72421474	OtA5088-OtA5092	OtA5093-OtA5097
Hexdc	chr11:121177592-121229309	OtA5088-OtA5092	OtA5093-OtA5097
Hist2h2ab	chr3:96219915-96220308	OtA5088-OtA5092	OtA5093-OtA5097
Hsf1	chr15:76477444-76500972	OtA5088-OtA5092	OtA5093-OtA5097
Itgb5	chr16:33829664-33949338	OtA5088-OtA5092	OtA5093-OtA5097
Kdelr1	chr7:45872839-45883726	OtA5088-OtA5092	OtA5093-OtA5097
Lyst	chr13:13590408-13777440	OtA5088-OtA5092	OtA5093-OtA5097
Mob1a	chr6:83326038-83340949	OtA5088-OtA5092	OtA5093-OtA5097
Mpst	chr15:78406711-78414015	OtA5088-OtA5092	OtA5093-OtA5097
Nfkbie	chr17:45555715-45563168	OtA5088-OtA5092	OtA5093-OtA5097
Pcsk9	chr4:106442333-106464325	OtA5088-OtA5092	OtA5093-OtA5097
Polg2	chr11:106768203-106779537	OtA5088-OtA5092	OtA5093-OtA5097
Ppox	chr1:171276991-171281186	OtA5088-OtA5092	OtA5093-OtA5097
Ptprs	chr17:56412425-56476480	OtA5088-OtA5092	OtA5093-OtA5097
Rspry1	chr8:94601940-94660276	OtA5088-OtA5092	OtA5093-OtA5097
Snrpb	chr2:130171635-130179364	OtA5088-OtA5092	OtA5093-OtA5097
Sypl	chr12:32953944-32979502	OtA5088-OtA5092	OtA5093-OtA5097
Tasp1	chr2:139833479-140066785	OtA5088-OtA5092	OtA5093-OtA5097
Xlr3b	chrX:73192178-73202930	OtA5088-OtA5092	OtA5093-OtA5097
Xlr4b	chrX:73214364-73222453	OtA5088-OtA5092	OtA5093-OtA5097
Zmym4	chr4:126753554-126967923	OtA5088-OtA5092	OtA5093-OtA5097
Rbbp7	chrX:162407199-163352564	OtA5088-OtA5092	OtA5093-OtA5097
Tecpr1	chr5:144195346-144223578	OtA5088-OtA5092	OtA5093-OtA5097
Ddt	chr10:75771232-75773374	OtA5088-OtA5092	OtA5093-OtA5097
Slc6a12	chr6:121346696-121365773	OtA5088-OtA5092	OtA5093-OtA5097
Tctex1d2	chr16:32419701-32428892	OtA5088-OtA5092	OtA5093-OtA5097

Arnt2	chr7:84246278-84409959	OtA5088-OtA5092	OtA5093-OtA5097
Dvl2	chr11:70000625-70010109	OtA5088-OtA5092	OtA5093-OtA5097
Ppt2	chr17:34616661-34627097	OtA5088-OtA5092	OtA5093-OtA5097
Psg28	chr7:18422535-18432055	OtA5088-OtA5092	OtA5093-OtA5097
Rhoj	chr12:75308312-75401455	OtA5088-OtA5092	OtA5093-OtA5097
Srrt	chr5:137295703-137307674	OtA5088-OtA5092	OtA5093-OtA5097
Tmem161a	chr8:70172410-70183663	OtA5088-OtA5092	OtA5093-OtA5097
Tpra1	chr6:88902250-88912240	OtA5088-OtA5092	OtA5093-OtA5097
Zdhhc18	chr4:133606991-133633429	OtA5088-OtA5092	OtA5093-OtA5097
Rpl34,Rpl34-ps1	chr3:130726830-130730329	OtA5088-OtA5092	OtA5093-OtA5097
Unkl	chr17:25188399-25240116	OtA5088-OtA5092	OtA5093-OtA5097
Zfp672	chr11:58315113-58330339	OtA5088-OtA5092	OtA5093-OtA5097
Fam73a	chr3:152273459-152340407	OtA5088-OtA5092	OtA5093-OtA5097
Slc35b1	chr11:95384921-95391652	OtA5088-OtA5092	OtA5093-OtA5097
Slc38a6	chr12:73286847-73354045	OtA5088-OtA5092	OtA5093-OtA5097
Ddhd2	chr8:25725323-25754280	OtA5088-OtA5092	OtA5093-OtA5097
Frmd4a	chr2:4152862-4614043	OtA5088-OtA5092	OtA5093-OtA5097
Sec61g	chr11:16501637-16508484	OtA5088-OtA5092	OtA5093-OtA5097
Sqstm1	chr11:50200151-50210790	OtA5088-OtA5092	OtA5093-OtA5097
Vav2	chr2:27263634-27426825	OtA5088-OtA5092	OtA5093-OtA5097
Wsb1	chr11:79239381-79254695	OtA5088-OtA5092	OtA5093-OtA5097
M6pr	chr6:122309009-122317677	OtA5088-OtA5092	OtA5093-OtA5097
S100a1	chr3:90511033-90514330	OtA5088-OtA5092	OtA5093-OtA5097
Hmgcs1	chr13:119690461-119708077	OtA5088-OtA5092	OtA5093-OtA5097
Mapk8ip1	chr2:92383675-92401263	OtA5088-OtA5092	OtA5093-OtA5097
Ppp6r1	chr7:4631494-4658950	OtA5088-OtA5092	OtA5093-OtA5097
Slc39a3	chr10:81028539-81033912	OtA5088-OtA5092	OtA5093-OtA5097
Twf1	chr15:94577947-94589824	OtA5088-OtA5092	OtA5093-OtA5097
Ccnf	chr17:24223231-24251409	OtA5088-OtA5092	OtA5093-OtA5097
Mrpl20	chr4:155803617-155808829	OtA5088-OtA5092	OtA5093-OtA5097
Abce1	chr8:79683441-79711740	OtA5088-OtA5092	OtA5093-OtA5097
Bglap-rs1	chr3:88368616-88369720	OtA5088-OtA5092	OtA5093-OtA5097
Btbd6	chr12:112959861-113000621	OtA5088-OtA5092	OtA5093-OtA5097
Fktn	chr4:53714181-53763271	OtA5088-OtA5092	OtA5093-OtA5097
Mfsd7b	chr1:191005831-191029781	OtA5088-OtA5092	OtA5093-OtA5097
Pggt1b	chr18:46239948-46280850	OtA5088-OtA5092	OtA5093-OtA5097
Phka1	chrX:102513974-102644246	OtA5088-OtA5092	OtA5093-OtA5097
Tpbpb	chr13:60901294-60904847	OtA5088-OtA5092	OtA5093-OtA5097
Msn	chrX:96096044-96168553	OtA5088-OtA5092	OtA5093-OtA5097
Ppil3	chr1:58430992-58445486	OtA5088-OtA5092	OtA5093-OtA5097
Haus5	chr7:30653707-30664994	OtA5088-OtA5092	OtA5093-OtA5097
Spg20	chr3:55112107-55137332	OtA5088-OtA5092	OtA5093-OtA5097
Cdk5rap2	chr4:70223023-70410367	OtA5088-OtA5092	OtA5093-OtA5097
Mthfsd	chr8:121097556-121108379	OtA5088-OtA5092	OtA5093-OtA5097
Irf8	chr8:120736375-120756692	OtA5088-OtA5092	OtA5093-OtA5097
Hagh	chr17:24850489-24864450	OtA5088-OtA5092	OtA5093-OtA5097
Elp2	chr18:24603960-24638830	OtA5088-OtA5092	OtA5093-OtA5097

Tmem132a	chr19:10842543-10869779	OtA5088-OtA5092	OtA5093-OtA5097
Dedd2	chr7:25202839-25219859	OtA5088-OtA5092	OtA5093-OtA5097
Eif2c3	chr4:126340677-126429542	OtA5088-OtA5092	OtA5093-OtA5097
Sf3b3	chr8:110810491-110846803	OtA5088-OtA5092	OtA5093-OtA5097
Klf5	chr14:99298690-99313409	OtA5088-OtA5092	OtA5093-OtA5097
Vrk2	chr11:26387083-26593920	OtA5088-OtA5092	OtA5093-OtA5097
Inhba	chr13:16014474-16027211	OtA5088-OtA5092	OtA5093-OtA5097
Kat2a	chr11:100704745-100712467	OtA5088-OtA5092	OtA5093-OtA5097
Pias2	chr18:77065207-77155708	OtA5088-OtA5092	OtA5093-OtA5097
Ripply3	chr16:94328421-94336935	OtA5088-OtA5092	OtA5093-OtA5097
Xpr1	chr1:155275656-155417444	OtA5088-OtA5092	OtA5093-OtA5097
Aff1	chr5:103754161-103855322	OtA5088-OtA5092	OtA5093-OtA5097
Atp11a	chr8:12757015-12868728	OtA5088-OtA5092	OtA5093-OtA5097
Ppy	chr11:102099930-102101300	OtA5088-OtA5092	OtA5093-OtA5097
B3galnt2	chr13:13954673-14039600	OtA5088-OtA5092	OtA5093-OtA5097
Galm	chr17:80127470-80185032	OtA5088-OtA5092	OtA5093-OtA5097
Grk6	chr13:55445333-55460927	OtA5088-OtA5092	OtA5093-OtA5097
Tubb5	chr17:35833919-35838301	OtA5088-OtA5092	OtA5093-OtA5097
Atp13a1	chr8:69791162-69807748	OtA5088-OtA5092	OtA5093-OtA5097
Carf	chr1:60076867-60151034	OtA5088-OtA5092	OtA5093-OtA5097
Chmp4c	chr3:10366972-10391005	OtA5088-OtA5092	OtA5093-OtA5097
Crk	chr11:75679309-75706092	OtA5088-OtA5092	OtA5093-OtA5097
Dtx2	chr5:135994799-136032881	OtA5088-OtA5092	OtA5093-OtA5097
Fam175a	chr5:100804801-100820935	OtA5088-OtA5092	OtA5093-OtA5097
Ism1	chr2:139678177-139758581	OtA5088-OtA5092	OtA5093-OtA5097
Upf3a	chr8:13785614-13798537	OtA5088-OtA5092	OtA5093-OtA5097
Neil1	chr9:57143255-57147034	OtA5088-OtA5092	OtA5093-OtA5097
Trappc2l	chr8:122611625-122615591	OtA5088-OtA5092	OtA5093-OtA5097
Arhgef37	chr18:61493793-61536536	OtA5088-OtA5092	OtA5093-OtA5097
Acbd5	chr2:23068200-23114512	OtA5088-OtA5092	OtA5093-OtA5097
Map3k11	chr19:5689130-5702864	OtA5088-OtA5092	OtA5093-OtA5097
Wnt7b	chr15:85535436-85593708	OtA5088-OtA5092	OtA5093-OtA5097
Glrx2	chr1:143739348-143749678	OtA5088-OtA5092	OtA5093-OtA5097
Ift20	chr11:78536435-78541473	OtA5088-OtA5092	OtA5093-OtA5097
Trim12c	chr7:104338753-104353358	OtA5088-OtA5092	OtA5093-OtA5097
Cyb561	chr11:105933703-105944147	OtA5088-OtA5092	OtA5093-OtA5097
Card10	chr15:78775135-78803042	OtA5088-OtA5092	OtA5093-OtA5097
Mbd3	chr10:80392540-80399479	OtA5088-OtA5092	OtA5093-OtA5097
Arl10	chr13:54575012-54581128	OtA5088-OtA5092	OtA5093-OtA5097
Ephb3	chr16:21204794-21223304	OtA5088-OtA5092	OtA5093-OtA5097
Mrs2	chr13:24992482-25020379	OtA5088-OtA5092	OtA5093-OtA5097
Bax	chr7:45461694-45466898	OtA5088-OtA5092	OtA5093-OtA5097
Nans	chr4:46489328-46503438	OtA5088-OtA5092	OtA5093-OtA5097
Crnkl1	chr2:145917481-145934700	OtA5088-OtA5092	OtA5093-OtA5097
Hist2h3c2	chr3:96238107-96239127	OtA5088-OtA5092	OtA5093-OtA5097
Adamts10	chr17:33524195-33553782	OtA5088-OtA5092	OtA5093-OtA5097
Dck	chr5:88765012-88783277	OtA5088-OtA5092	OtA5093-OtA5097

Fbxl4	chr4:22357564-22434046	OtA5088-OtA5092	OtA5093-OtA5097
Gtf2i	chr5:134237834-134314746	OtA5088-OtA5092	OtA5093-OtA5097
Mmaa	chr8:79266423-79294956	OtA5088-OtA5092	OtA5093-OtA5097
Aasdh	chr5:76875934-76905514	OtA5088-OtA5092	OtA5093-OtA5097
D16H22S680E	chr16:18300824-18343932	OtA5088-OtA5092	OtA5093-OtA5097
Anxa1	chr19:20373433-20390671	OtA5088-OtA5092	OtA5093-OtA5097
Bola3	chr6:83349483-83358392	OtA5088-OtA5092	OtA5093-OtA5097
Brpf3	chr17:28801125-28838789	OtA5088-OtA5092	OtA5093-OtA5097
Cdk20	chr13:64432552-64439721	OtA5088-OtA5092	OtA5093-OtA5097
Creb5	chr6:53573373-53695832	OtA5088-OtA5092	OtA5093-OtA5097
Dgcr14	chr16:17900708-17911348	OtA5088-OtA5092	OtA5093-OtA5097
Fam49a	chr12:12262138-12392475	OtA5088-OtA5092	OtA5093-OtA5097
Fam59a	chr18:21127341-21300139	OtA5088-OtA5092	OtA5093-OtA5097
Fam96a	chr9:66126610-66138968	OtA5088-OtA5092	OtA5093-OtA5097
Fam98c	chr7:29134932-29156210	OtA5088-OtA5092	OtA5093-OtA5097
Keap1	chr9:21229729-21239332	OtA5088-OtA5092	OtA5093-OtA5097
Paox	chr7:140125684-140134334	OtA5088-OtA5092	OtA5093-OtA5097
Rab11fip3	chr17:25989035-26069177	OtA5088-OtA5092	OtA5093-OtA5097
Sgta	chr10:81044072-81060154	OtA5088-OtA5092	OtA5093-OtA5097
Syncrip	chr9:88449737-88482397	OtA5088-OtA5092	OtA5093-OtA5097
Syt13	chr2:92915100-92956051	OtA5088-OtA5092	OtA5093-OtA5097
Tceal1	chrX:136708064-136709866	OtA5088-OtA5092	OtA5093-OtA5097
Trim34a	chr7:104244456-104262236	OtA5088-OtA5092	OtA5093-OtA5097
Trip10	chr17:57249450-57263697	OtA5088-OtA5092	OtA5093-OtA5097
Trmt1	chr8:84689246-84699808	OtA5088-OtA5092	OtA5093-OtA5097
Zrsr2	chrX:163935442-163958666	OtA5088-OtA5092	OtA5093-OtA5097
Rgl2	chr17:33929893-33937687	OtA5088-OtA5092	OtA5093-OtA5097
Efnb1	chrX:99136129-99148991	OtA5088-OtA5092	OtA5093-OtA5097
Dffb	chr4:153964448-153975081	OtA5088-OtA5092	OtA5093-OtA5097
Mageh1	chrX:153036165-153037563	OtA5088-OtA5092	OtA5093-OtA5097
Ppp2r2c	chr5:36868569-36955078	OtA5088-OtA5092	OtA5093-OtA5097
Tbcb	chr7:30224130-30232029	OtA5088-OtA5092	OtA5093-OtA5097
Wwtr1	chr3:57455643-57575910	OtA5088-OtA5092	OtA5093-OtA5097
Pcnxl3	chr19:5664634-5688908	OtA5088-OtA5092	OtA5093-OtA5097
Rnf25	chr1:74593751-74601397	OtA5088-OtA5092	OtA5093-OtA5097
Sp2	chr11:96944145-96977688	OtA5088-OtA5092	OtA5093-OtA5097
Daglb	chr5:143464492-143504442	OtA5088-OtA5092	OtA5093-OtA5097
Gprasp1	chrX:135742691-135803468	OtA5088-OtA5092	OtA5093-OtA5097
Mcm4	chr16:15623896-15637400	OtA5088-OtA5092	OtA5093-OtA5097
Rara	chr11:98937695-98974942	OtA5088-OtA5092	OtA5093-OtA5097
Src	chr2:157424292-157471838	OtA5088-OtA5092	OtA5093-OtA5097
Wrn	chr8:33234372-33385527	OtA5088-OtA5092	OtA5093-OtA5097
Ddx39b	chr17:35241745-35253707	OtA5088-OtA5092	OtA5093-OtA5097
Dgcr8	chr16:18248882-18289168	OtA5088-OtA5092	OtA5093-OtA5097
Dnajb2	chr1:75236422-75245692	OtA5088-OtA5092	OtA5093-OtA5097
Etv6	chr6:134035706-134270147	OtA5088-OtA5092	OtA5093-OtA5097
Fam174a	chr1:95313627-95335284	OtA5088-OtA5092	OtA5093-OtA5097

Oasl2	chr5:114896933-114912245	OtA5088-OtA5092	OtA5093-OtA5097
Prpf6	chr2:181601318-181655661	OtA5088-OtA5092	OtA5093-OtA5097
Brd9	chr13:73937837-73960895	OtA5088-OtA5092	OtA5093-OtA5097
Trim16	chr11:62820252-62842948	OtA5088-OtA5092	OtA5093-OtA5097
Slc31a2	chr4:62286444-62298411	OtA5088-OtA5092	OtA5093-OtA5097
Dip2a	chr10:76263048-76345291	OtA5088-OtA5092	OtA5093-OtA5097
Manf	chr9:106887414-106891938	OtA5088-OtA5092	OtA5093-OtA5097
Zswim3	chr2:164805113-164822127	OtA5088-OtA5092	OtA5093-OtA5097
Eif2ak1	chr5:143871861-143909839	OtA5088-OtA5092	OtA5093-OtA5097
Exoc4	chr6:33249149-33972930	OtA5088-OtA5092	OtA5093-OtA5097
Grap	chr11:61653320-61672777	OtA5088-OtA5092	OtA5093-OtA5097
Kti12	chr4:108834677-108862119	OtA5088-OtA5092	OtA5093-OtA5097
Ripk2	chr4:16123374-16163498	OtA5088-OtA5092	OtA5093-OtA5097
Rps6	chr4:86854098-86857367	OtA5088-OtA5092	OtA5093-OtA5097
Trim23	chr13:104179097-104202048	OtA5088-OtA5092	OtA5093-OtA5097
Zhx2	chr15:57694666-57839832	OtA5088-OtA5092	OtA5093-OtA5097
Taf8	chr17:47488049-47502287	OtA5088-OtA5092	OtA5093-OtA5097
Asah2	chr19:31984650-32103140	OtA5088-OtA5092	OtA5093-OtA5097
Ccdc71	chr9:108447638-108465945	OtA5088-OtA5092	OtA5093-OtA5097
Man2a2	chr7:80349096-80371375	OtA5088-OtA5092	OtA5093-OtA5097
Vbp1	chrX:75514296-75534946	OtA5088-OtA5092	OtA5093-OtA5097
Avl9	chr6:56714904-56761911	OtA5088-OtA5092	OtA5093-OtA5097
Orc5	chr5:22486488-22550331	OtA5088-OtA5092	OtA5093-OtA5097
Tns1	chr1:73910230-74124447	OtA5088-OtA5092	OtA5093-OtA5097
Pramel1	chr4:143394440-143399819	OtA5088-OtA5092	OtA5093-OtA5097
Pxmp3	chr3:5560187-5576239	OtA5088-OtA5092	OtA5093-OtA5097
Wscd1	chr11:71750702-71789646	OtA5088-OtA5092	OtA5093-OtA5097
Cldn4	chr5:134945123-134946934	OtA5088-OtA5092	OtA5093-OtA5097
Hip1	chr5:135406518-135545122	OtA5088-OtA5092	OtA5093-OtA5097
Itgb1	chr8:128685653-128733579	OtA5088-OtA5092	OtA5093-OtA5097
Prim2	chr1:33453810-33669758	OtA5088-OtA5092	OtA5093-OtA5097
Gtf2ird2	chr5:134184037-134218143	OtA5088-OtA5092	OtA5093-OtA5097
Map3k7	chr4:31964106-32023466	OtA5088-OtA5092	OtA5093-OtA5097
Pou2f3	chr9:43123938-43205755	OtA5088-OtA5092	OtA5093-OtA5097
Ctu1	chr7:43672030-43678297	OtA5088-OtA5092	OtA5093-OtA5097
Ppia	chr11:6415869-6419810	OtA5088-OtA5092	OtA5093-OtA5097
Rit1	chr3:88716853-88731048	OtA5088-OtA5092	OtA5093-OtA5097
Trim6	chr7:104218794-104235152	OtA5088-OtA5092	OtA5093-OtA5097
Acot13	chr13:24817954-24831489	OtA5088-OtA5092	OtA5093-OtA5097
Aldh3b1	chr19:3913490-3929716	OtA5088-OtA5092	OtA5093-OtA5097
Crip2	chr12:113140235-113145506	OtA5088-OtA5092	OtA5093-OtA5097
Dclre1c	chr2:3424130-3474986	OtA5088-OtA5092	OtA5093-OtA5097
Dph1	chr11:75175942-75190483	OtA5088-OtA5092	OtA5093-OtA5097
Fgfbp3	chr19:36917549-36919599	OtA5088-OtA5092	OtA5093-OtA5097
Igsf11	chr16:38902344-39027157	OtA5088-OtA5092	OtA5093-OtA5097
Magee1	chrX:105120395-105123911	OtA5088-OtA5092	OtA5093-OtA5097
Nav1	chr1:135434579-135585355	OtA5088-OtA5092	OtA5093-OtA5097

Prep	chr10:45067213-45158995	OtA5088-OtA5092	OtA5093-OtA5097
Rab8b	chr9:66843663-66919705	OtA5088-OtA5092	OtA5093-OtA5097
Rbak	chr5:143172188-143180743	OtA5088-OtA5092	OtA5093-OtA5097
Rsad2	chr12:26442742-26456452	OtA5088-OtA5092	OtA5093-OtA5097
Socs4	chr14:47277142-47291591	OtA5088-OtA5092	OtA5093-OtA5097
Trerf1	chr17:47140941-47359458	OtA5088-OtA5092	OtA5093-OtA5097
Wdr26	chr1:181173225-181211978	OtA5088-OtA5092	OtA5093-OtA5097
Atp5j2	chr5:145183705-145191592	OtA5088-OtA5092	OtA5093-OtA5097
Irf2bp1	chr7:19004064-19006763	OtA5088-OtA5092	OtA5093-OtA5097
Mnat1	chr12:73123716-73273988	OtA5088-OtA5092	OtA5093-OtA5097
Zfp87	chr13:67515781-67526231	OtA5088-OtA5092	OtA5093-OtA5097
Znrf2	chr6:54816915-54890224	OtA5088-OtA5092	OtA5093-OtA5097
Narg2	chr9:69397997-69433074	OtA5088-OtA5092	OtA5093-OtA5097
Klhl36	chr8:119862304-119876989	OtA5088-OtA5092	OtA5093-OtA5097
Rasgrp3	chr17:75435904-75529053	OtA5088-OtA5092	OtA5093-OtA5097
Vsig2	chr9:37539254-37544205	OtA5088-OtA5092	OtA5093-OtA5097
Zmym1	chr4:127047093-127061132	OtA5088-OtA5092	OtA5093-OtA5097
Ankrd37	chr8:45996907-45999850	OtA5088-OtA5092	OtA5093-OtA5097
Naa38	chr6:18848634-18854052	OtA5088-OtA5092	OtA5093-OtA5097
Dpm1	chr2:168209047-168230379	OtA5088-OtA5092	OtA5093-OtA5097
9-Mar	chr10:127056049-127060184	OtA5088-OtA5092	OtA5093-OtA5097
Wbscr27	chr5:134932372-134942637	OtA5088-OtA5092	OtA5093-OtA5097
Cyp20a1	chr1:60343371-60387387	OtA5088-OtA5092	OtA5093-OtA5097
Dlg1	chr16:31663442-31873356	OtA5088-OtA5092	OtA5093-OtA5097
Hspb6	chr7:30553301-30555439	OtA5088-OtA5092	OtA5093-OtA5097
Mpdu1	chr11:69655036-69662642	OtA5088-OtA5092	OtA5093-OtA5097
Ppm1l	chr3:69316917-69555396	OtA5088-OtA5092	OtA5093-OtA5097
App	chr16:84954435-85173707	OtA5088-OtA5092	OtA5093-OtA5097
C2cd2l	chr9:44309236-44320282	OtA5088-OtA5092	OtA5093-OtA5097
Ccl27a	chr4:41760442-41774176	OtA5088-OtA5092	OtA5093-OtA5097
Dnajc5	chr2:181520504-181552249	OtA5088-OtA5092	OtA5093-OtA5097
Lamp2	chrX:38405063-38456455	OtA5088-OtA5092	OtA5093-OtA5097
Madd	chr2:91137359-91183047	OtA5088-OtA5092	OtA5093-OtA5097
Nup88	chr11:70844762-70969973	OtA5088-OtA5092	OtA5093-OtA5097
Pum1	chr4:130663358-130781565	OtA5088-OtA5092	OtA5093-OtA5097
Ubxn2a	chr12:4879031-4907520	OtA5088-OtA5092	OtA5093-OtA5097
Ccdc53	chr10:88201096-88246158	OtA5088-OtA5092	OtA5093-OtA5097
Ccl25	chr8:4325209-4360020	OtA5088-OtA5092	OtA5093-OtA5097
Cct6a	chr5:129787384-129794500	OtA5088-OtA5092	OtA5093-OtA5097
Dpp7	chr2:25352289-25356332	OtA5088-OtA5092	OtA5093-OtA5097
Exosc10	chr4:148558426-148582400	OtA5088-OtA5092	OtA5093-OtA5097
Glul	chr1:153899944-153909714	OtA5088-OtA5092	OtA5093-OtA5097
Gnb2l1	chr11:48800359-48806241	OtA5088-OtA5092	OtA5093-OtA5097
Lgals1	chr15:78926724-78930465	OtA5088-OtA5092	OtA5093-OtA5097
Ndufa11	chr17:56717761-56724248	OtA5088-OtA5092	OtA5093-OtA5097
Shq1	chr6:100573081-100671157	OtA5088-OtA5092	OtA5093-OtA5097
Thoc3	chr13:54458836-54468840	OtA5088-OtA5092	OtA5093-OtA5097

Ccdc8	chr7:16994587-16996645	OtA5088-OtA5092	OtA5093-OtA5097
Cryzl1	chr16:91688897-91728802	OtA5088-OtA5092	OtA5093-OtA5097
Leprot	chr4:101647782-101659358	OtA5088-OtA5092	OtA5093-OtA5097
Lpcat4	chr2:112239840-112247111	OtA5088-OtA5092	OtA5093-OtA5097
Pla2g16	chr19:7557458-7588545	OtA5088-OtA5092	OtA5093-OtA5097
Plekhm2	chr4:141625733-141664115	OtA5088-OtA5092	OtA5093-OtA5097
Sema3c	chr5:17574815-17730267	OtA5088-OtA5092	OtA5093-OtA5097
Sra1	chr18:36667186-36670311	OtA5088-OtA5092	OtA5093-OtA5097
Tmc5	chr7:118597296-118675085	OtA5088-OtA5092	OtA5093-OtA5097
Tmem201	chr4:149715383-149738036	OtA5088-OtA5092	OtA5093-OtA5097
Tmem222	chr4:133266044-133277790	OtA5088-OtA5092	OtA5093-OtA5097
Zbtb46	chr2:181390885-181459426	OtA5088-OtA5092	OtA5093-OtA5097
Zfp606	chr7:12478304-12496235	OtA5088-OtA5092	OtA5093-OtA5097
Fbxw8	chr5:118064980-118155458	OtA5088-OtA5092	OtA5093-OtA5097
Atpif1	chr4:132530554-132533659	OtA5088-OtA5092	OtA5093-OtA5097
Gstp1	chr19:4035410-4037912	OtA5088-OtA5092	OtA5093-OtA5097
Mfhas1	chr8:35582502-35679449	OtA5088-OtA5092	OtA5093-OtA5097
Mtss1	chr15:58941233-59082026	OtA5088-OtA5092	OtA5093-OtA5097
Pfkm	chr15:98038743-98132447	OtA5088-OtA5092	OtA5093-OtA5097
Susd3	chr13:49230830-49248706	OtA5088-OtA5092	OtA5093-OtA5097
Rufy3	chr5:88583573-88642696	OtA5088-OtA5092	OtA5093-OtA5097
Zfp715	chr7:43296522-43313261	OtA5088-OtA5092	OtA5093-OtA5097
Bcl2l13	chr6:120836229-120892842	OtA5088-OtA5092	OtA5093-OtA5097
Cyp4f16	chr17:32536628-32551797	OtA5088-OtA5092	OtA5093-OtA5097
Eprs	chr1:185363094-185428355	OtA5088-OtA5092	OtA5093-OtA5097
Il1rap	chr16:26581704-26725147	OtA5088-OtA5092	OtA5093-OtA5097
Lsm6	chr8:78804867-78821152	OtA5088-OtA5092	OtA5093-OtA5097
Rab3ip	chr10:116905783-116950380	OtA5088-OtA5092	OtA5093-OtA5097
Synpo	chr18:60593989-60624305	OtA5088-OtA5092	OtA5093-OtA5097
Vasp	chr7:19256929-19271854	OtA5088-OtA5092	OtA5093-OtA5097
Wdr41	chr13:94976347-95023314	OtA5088-OtA5092	OtA5093-OtA5097
Zfp410	chr12:84316858-84344439	OtA5088-OtA5092	OtA5093-OtA5097
Mitd1	chr1:37878889-37890411	OtA5088-OtA5092	OtA5093-OtA5097
Gca	chr2:62664326-62694109	OtA5088-OtA5092	OtA5093-OtA5097
Tmem8b	chr4:43668970-43692667	OtA5088-OtA5092	OtA5093-OtA5097
Rilp	chr11:75510093-75513166	OtA5088-OtA5092	OtA5093-OtA5097
Net1	chr13:3882560-3918220	OtA5088-OtA5092	OtA5093-OtA5097
Slc4a1ap	chr5:31526994-31554038	OtA5088-OtA5092	OtA5093-OtA5097
Asb7	chr7:66644566-66689561	OtA5088-OtA5092	OtA5093-OtA5097
Fxyd4	chr6:117933558-117937335	OtA5088-OtA5092	OtA5093-OtA5097
Hemk1	chr9:107327081-107338350	OtA5088-OtA5092	OtA5093-OtA5097
Lims1	chr10:58323465-58424691	OtA5088-OtA5092	OtA5093-OtA5097
P2rx1	chr11:72999144-73015197	OtA5088-OtA5092	OtA5093-OtA5097
Pde12	chr14:26664116-26669846	OtA5088-OtA5092	OtA5093-OtA5097
Xaf1	chr11:72301628-72313733	OtA5088-OtA5092	OtA5093-OtA5097
Apoc2	chr7:19671578-19676864	OtA5088-OtA5092	OtA5093-OtA5097
Btrc	chr19:45363733-45533343	OtA5088-OtA5092	OtA5093-OtA5097

Itfg1	chr8:85717556-85840949	OtA5088-OtA5092	OtA5093-OtA5097
Krtcap3	chr5:31251705-31253197	OtA5088-OtA5092	OtA5093-OtA5097
Osbpl3	chr6:50293326-50456170	OtA5088-OtA5092	OtA5093-OtA5097
Plekhg3	chr12:76533559-76579039	OtA5088-OtA5092	OtA5093-OtA5097
Rbm19	chr5:120116512-120198971	OtA5088-OtA5092	OtA5093-OtA5097
Stat5b	chr11:100780730-100850585	OtA5088-OtA5092	OtA5093-OtA5097
Tmem223	chr19:8770995-8772475	OtA5088-OtA5092	OtA5093-OtA5097
Bub1b	chr2:118598210-118641592	OtA5088-OtA5092	OtA5093-OtA5097
Ebf1	chr11:44618133-45005172	OtA5088-OtA5092	OtA5093-OtA5097
Hspbp1	chr7:4660520-4684963	OtA5088-OtA5092	OtA5093-OtA5097
Mycbpap	chr11:94501346-94521502	OtA5088-OtA5092	OtA5093-OtA5097
Pdcd4	chr19:53892230-54033278	OtA5088-OtA5092	OtA5093-OtA5097
Taf1d	chr9:15283336-15357788	OtA5088-OtA5092	OtA5093-OtA5097
Tfb1m	chr17:3326572-3557713	OtA5088-OtA5092	OtA5093-OtA5097
Pappa2	chr1:158711730-158957438	OtA5088-OtA5092	OtA5093-OtA5097
Srp68	chr11:116245165-116274217	OtA5088-OtA5092	OtA5093-OtA5097
Htra4	chr8:25024927-25038962	OtA5088-OtA5092	OtA5093-OtA5097
Znhit3	chr11:84876312-84916356	OtA5088-OtA5092	OtA5093-OtA5097
Hspbap1	chr16:35770385-35828462	OtA5088-OtA5092	OtA5093-OtA5097
Pf4	chr5:90772434-90773383	OtA5088-OtA5092	OtA5093-OtA5097
Pik3cd	chr4:149649167-149701629	OtA5088-OtA5092	OtA5093-OtA5097
Tars2	chr3:95739973-95754977	OtA5088-OtA5092	OtA5093-OtA5097
Taco1	chr11:106066106-106073612	OtA5088-OtA5092	OtA5093-OtA5097
Ccdc3	chr2:5137775-5230866	OtA5088-OtA5092	OtA5093-OtA5097
Med29	chr7:28385246-28392690	OtA5088-OtA5092	OtA5093-OtA5097
Fgd1	chrX:151047232-151096543	OtA5088-OtA5092	OtA5093-OtA5097
Aagab	chr9:63602654-63641889	OtA5088-OtA5092	OtA5093-OtA5097
Arhgef26	chr3:62338776-62462221	OtA5088-OtA5092	OtA5093-OtA5097
Capza1	chr3:104822784-104864505	OtA5088-OtA5092	OtA5093-OtA5097
Cd99l2	chrX:71420059-71492849	OtA5088-OtA5092	OtA5093-OtA5097
Gemin6	chr17:80224488-80228497	OtA5088-OtA5092	OtA5093-OtA5097
Invs	chr4:48279801-48473422	OtA5088-OtA5092	OtA5093-OtA5097
Loxl1	chr9:58287722-58313212	OtA5088-OtA5092	OtA5093-OtA5097
Pex26	chr6:121183666-121196185	OtA5088-OtA5092	OtA5093-OtA5097
Slc39a2	chr14:51893609-51896745	OtA5088-OtA5092	OtA5093-OtA5097
Snord22	chr19:8723486-8726326	OtA5088-OtA5092	OtA5093-OtA5097
Tmem64	chr4:15265819-15286753	OtA5088-OtA5092	OtA5093-OtA5097
Klhdc1	chr12:69241831-69283961	OtA5088-OtA5092	OtA5093-OtA5097
Mast2	chr4:116306761-116464183	OtA5088-OtA5092	OtA5093-OtA5097
Snx29	chr16:11322903-11755473	OtA5088-OtA5092	OtA5093-OtA5097
Tsc22d3	chrX:140539528-140600522	OtA5088-OtA5092	OtA5093-OtA5097
Zbtb17	chr4:141444672-141538597	OtA5088-OtA5092	OtA5093-OtA5097
Kidins220	chr12:24974931-25059697	OtA5088-OtA5092	OtA5093-OtA5097
Rab34	chr11:78188426-78192193	OtA5088-OtA5092	OtA5093-OtA5097
Dus2l	chr8:106011506-106053819	OtA5088-OtA5092	OtA5093-OtA5097
Pycr2	chr1:180904273-180908088	OtA5088-OtA5092	OtA5093-OtA5097
Ubc	chr5:125385964-125390017	OtA5088-OtA5092	OtA5093-OtA5097

Nutf2-ps1	chr8:105860633-105880401	OtA5088-OtA5092	OtA5093-OtA5097
Nudt7	chr8:114133578-114152305	OtA5088-OtA5092	OtA5093-OtA5097
Ttl	chr2:129065946-129096283	OtA5088-OtA5092	OtA5093-OtA5097
Clip3	chr7:30291752-30308367	OtA5088-OtA5092	OtA5093-OtA5097
Tmem119	chr5:113793728-113800352	OtA5088-OtA5092	OtA5093-OtA5097
Fam171a1	chr2:3118387-3227809	OtA5088-OtA5092	OtA5093-OtA5097
Atpbd4	chr2:114516417-114654928	OtA5088-OtA5092	OtA5093-OtA5097
Gm18756	chr7:14530653-14623066	OtA5088-OtA5092	OtA5093-OtA5097
Gucy1b2	chr14:62392669-62456289	OtA5088-OtA5092	OtA5093-OtA5097
Wipi1	chr11:109573520-109611389	OtA5088-OtA5092	OtA5093-OtA5097
Ankrd39	chr1:36538172-36547201	OtA5088-OtA5092	OtA5093-OtA5097
Fbxo46	chr7:19119858-19138261	OtA5088-OtA5092	OtA5093-OtA5097
Limk2	chr11:3202352-3479831	OtA5088-OtA5092	OtA5093-OtA5097
Mme	chr3:63295871-63382231	OtA5088-OtA5092	OtA5093-OtA5097
Pex13	chr11:23646478-23732876	OtA5088-OtA5092	OtA5093-OtA5097
Rbm38	chr2:173021901-173034731	OtA5088-OtA5092	OtA5093-OtA5097
Setdb2	chr14:59402010-59440877	OtA5088-OtA5092	OtA5093-OtA5097
Vps29	chr5:122354412-122363287	OtA5088-OtA5092	OtA5093-OtA5097
Alkbh3	chr2:93980033-94010730	OtA5088-OtA5092	OtA5093-OtA5097
BC024659	chr13:41249843-41276577	OtA5088-OtA5092	OtA5093-OtA5097
Cdk6	chr5:3344311-3522225	OtA5088-OtA5092	OtA5093-OtA5097
Ctbp2	chr7:132987010-133123483	OtA5088-OtA5092	OtA5093-OtA5097
Cxxc1	chr18:74216211-74221491	OtA5088-OtA5092	OtA5093-OtA5097
Dld	chr12:31331561-31351437	OtA5088-OtA5092	OtA5093-OtA5097
Dtd1	chr2:144599952-144768747	OtA5088-OtA5092	OtA5093-OtA5097
Eif2b3	chr4:117019407-117086852	OtA5088-OtA5092	OtA5093-OtA5097
Fam174b	chr7:73740306-73776919	OtA5088-OtA5092	OtA5093-OtA5097
Fbn1	chr2:125300593-125506438	OtA5088-OtA5092	OtA5093-OtA5097
Haus6	chr4:86581284-86612022	OtA5088-OtA5092	OtA5093-OtA5097
Helq	chr5:100762147-100798600	OtA5088-OtA5092	OtA5093-OtA5097
Josd1	chr15:79674249-79687872	OtA5088-OtA5092	OtA5093-OtA5097
Kif13b	chr14:64652530-64806296	OtA5088-OtA5092	OtA5093-OtA5097
Lhfp	chr3:53041546-53261679	OtA5088-OtA5092	OtA5093-OtA5097
Map3k1	chr13:111746434-111808983	OtA5088-OtA5092	OtA5093-OtA5097
Mmachc	chr4:116702433-116708385	OtA5088-OtA5092	OtA5093-OtA5097
Nudt2	chr4:41465147-41480926	OtA5088-OtA5092	OtA5093-OtA5097
Nxn	chr11:76257225-76399141	OtA5088-OtA5092	OtA5093-OtA5097
Pigx	chr16:32084415-32099727	OtA5088-OtA5092	OtA5093-OtA5097
Ppapdc1b	chr8:25720047-25724887	OtA5088-OtA5092	OtA5093-OtA5097
Psmd10	chrX:140948424-140956711	OtA5088-OtA5092	OtA5093-OtA5097
Rsph3b	chr17:6904715-6948356	OtA5088-OtA5092	OtA5093-OtA5097
Rsrc1	chr3:66985671-67358403	OtA5088-OtA5092	OtA5093-OtA5097
Spn	chr7:127133463-127137817	OtA5088-OtA5092	OtA5093-OtA5097
Suclg2	chr6:95474133-95718837	OtA5088-OtA5092	OtA5093-OtA5097
Syngr1	chr15:80091333-80119501	OtA5088-OtA5092	OtA5093-OtA5097
Tinf2	chr14:55679079-55681817	OtA5088-OtA5092	OtA5093-OtA5097
Tomm6	chr17:47686644-47691092	OtA5088-OtA5092	OtA5093-OtA5097

Xpo1	chr11:23256040-23297597	OtA5088-OtA5092	OtA5093-OtA5097
Tlcd2	chr11:75468078-75470644	OtA5088-OtA5092	OtA5093-OtA5097
Pard6g	chr18:80046894-80119640	OtA5088-OtA5092	OtA5093-OtA5097
Arl4a	chr12:40033290-40037987	OtA5088-OtA5092	OtA5093-OtA5097
Cstf2	chrX:134059348-134086821	OtA5088-OtA5092	OtA5093-OtA5097
Fam69a	chr5:107900527-107987077	OtA5088-OtA5092	OtA5093-OtA5097
Hoxa13	chr6:52258852-52260880	OtA5088-OtA5092	OtA5093-OtA5097
Rangrf	chr11:68968130-68973820	OtA5088-OtA5092	OtA5093-OtA5097
Sema5a	chr15:32244812-32696341	OtA5088-OtA5092	OtA5093-OtA5097
Smpdl3a	chr10:57794543-57811830	OtA5088-OtA5092	OtA5093-OtA5097
Snrpa1	chr7:66060335-66074587	OtA5088-OtA5092	OtA5093-OtA5097
Stk16	chr1:75210828-75219253	OtA5088-OtA5092	OtA5093-OtA5097
Tmem116	chr5:121452708-121495421	OtA5088-OtA5092	OtA5093-OtA5097
Ttc39b	chr4:83220300-83324189	OtA5088-OtA5092	OtA5093-OtA5097
Nr2c2ap	chr8:70130805-70139197	OtA5088-OtA5092	OtA5093-OtA5097
Wdr90	chr17:25838837-25861515	OtA5088-OtA5092	OtA5093-OtA5097
Bcr	chr10:75060895-75184923	OtA5088-OtA5092	OtA5093-OtA5097
Ctsl	chr13:64363213-64370306	OtA5088-OtA5092	OtA5093-OtA5097
Fam46b	chr4:133480132-133487940	OtA5088-OtA5092	OtA5093-OtA5097
Fxyd3	chr7:31059934-31076697	OtA5088-OtA5092	OtA5093-OtA5097
Iars	chr13:49682129-49734267	OtA5088-OtA5092	OtA5093-OtA5097
Limd2	chr11:106156255-106160142	OtA5088-OtA5092	OtA5093-OtA5097
Ndufaf2	chr13:108052588-108158625	OtA5088-OtA5092	OtA5093-OtA5097
Prdx4	chrX:155323919-155338454	OtA5088-OtA5092	OtA5093-OtA5097
Csrnp1	chr9:119971165-119984658	OtA5088-OtA5092	OtA5093-OtA5097
Gins3	chr8:95633558-95645059	OtA5088-OtA5092	OtA5093-OtA5097
Pkd1	chr17:24549949-24632629	OtA5088-OtA5092	OtA5093-OtA5097
Rwdd4a	chr8:47533644-47552837	OtA5088-OtA5092	OtA5093-OtA5097
Slc5a3	chr16:92058321-92112227	OtA5088-OtA5092	OtA5093-OtA5097
Trim63	chr4:134315119-134329629	OtA5088-OtA5092	OtA5093-OtA5097
Zfp668	chr7:127865358-127876823	OtA5088-OtA5092	OtA5093-OtA5097
Abcc10	chr17:46297420-46328023	OtA5088-OtA5092	OtA5093-OtA5097
Antxr2	chr5:97884687-98030962	OtA5088-OtA5092	OtA5093-OtA5097
Epas1	chr17:86753863-86833410	OtA5088-OtA5092	OtA5093-OtA5097
Epc2	chr2:49451485-49551609	OtA5088-OtA5092	OtA5093-OtA5097
Fam160a1	chr3:85660062-85746209	OtA5088-OtA5092	OtA5093-OtA5097
Gabarapl1	chr6:129532183-129542331	OtA5088-OtA5092	OtA5093-OtA5097
Gpr182	chr10:127749601-127751798	OtA5088-OtA5092	OtA5093-OtA5097
Ifit1	chr19:34640888-34650009	OtA5088-OtA5092	OtA5093-OtA5097
Pmel	chr10:128706257-128744056	OtA5088-OtA5092	OtA5093-OtA5097
Psmg3	chr5:139823593-139826843	OtA5088-OtA5092	OtA5093-OtA5097
Sh2b3	chr5:121817214-121836801	OtA5088-OtA5092	OtA5093-OtA5097
Smad4	chr18:73639012-73703741	OtA5088-OtA5092	OtA5093-OtA5097
Spg11	chr2:122028582-122118386	OtA5088-OtA5092	OtA5093-OtA5097
Ssr3	chr3:65379656-65392553	OtA5088-OtA5092	OtA5093-OtA5097
Tgfbr3	chr5:107106569-107289595	OtA5088-OtA5092	OtA5093-OtA5097
Trmt6	chr2:132804214-132816054	OtA5088-OtA5092	OtA5093-OtA5097

Wdfy2	chr14:62837689-62956886	OtA5088-OtA5092	OtA5093-OtA5097
Jrk	chr15:74702411-74710374	OtA5088-OtA5092	OtA5093-OtA5097
Chml	chr1:175662427-175692590	OtA5088-OtA5092	OtA5093-OtA5097
Fes	chr7:80377757-80387946	OtA5088-OtA5092	OtA5093-OtA5097
Mbnl3	chrX:51113493-51205832	OtA5088-OtA5092	OtA5093-OtA5097
Pfkl	chr10:77986948-78009796	OtA5088-OtA5092	OtA5093-OtA5097
Rnf113a1	chrX:37191342-37192465	OtA5088-OtA5092	OtA5093-OtA5097
Aak1	chr6:86849516-87003227	OtA5088-OtA5092	OtA5093-OtA5097
Agbl5	chr5:30888851-30907788	OtA5088-OtA5092	OtA5093-OtA5097
Cap1	chr4:122859227-122885881	OtA5088-OtA5092	OtA5093-OtA5097
Cirh1a	chr8:106893639-106923094	OtA5088-OtA5092	OtA5093-OtA5097
Dap3	chr3:88920802-88950282	OtA5088-OtA5092	OtA5093-OtA5097
Dnajc15	chr14:77826216-77874917	OtA5088-OtA5092	OtA5093-OtA5097
Efha1	chr14:57916279-57999262	OtA5088-OtA5092	OtA5093-OtA5097
Fbxo7	chr10:86021928-86048328	OtA5088-OtA5092	OtA5093-OtA5097
Fzd5	chr1:64730557-64737750	OtA5088-OtA5092	OtA5093-OtA5097
Gdpd5	chr7:99381548-99460984	OtA5088-OtA5092	OtA5093-OtA5097
Gm7334	chr16:78359859-78376785	OtA5088-OtA5092	OtA5093-OtA5097
H2-M3	chr17:37270233-37274485	OtA5088-OtA5092	OtA5093-OtA5097
Hectd1	chr12:51743721-51829536	OtA5088-OtA5092	OtA5093-OtA5097
Khk	chr5:30921894-30931246	OtA5088-OtA5092	OtA5093-OtA5097
Lmod1	chr1:135324812-135368065	OtA5088-OtA5092	OtA5093-OtA5097
2-Mar	chr17:33685691-33718677	OtA5088-OtA5092	OtA5093-OtA5097
Nisch	chr14:31170927-31206826	OtA5088-OtA5092	OtA5093-OtA5097
Nufip1	chr14:76110890-76137379	OtA5088-OtA5092	OtA5093-OtA5097
Obfc2b	chr10:128401394-128409796	OtA5088-OtA5092	OtA5093-OtA5097
Ptbp2	chr3:119718741-119783388	OtA5088-OtA5092	OtA5093-OtA5097
Ptov1	chr7:44863067-44869788	OtA5088-OtA5092	OtA5093-OtA5097
Rab12	chr17:66494511-66519670	OtA5088-OtA5092	OtA5093-OtA5097
Rpl37	chr15:5116612-5119140	OtA5088-OtA5092	OtA5093-OtA5097
Snapc4	chr2:26362764-26380653	OtA5088-OtA5092	OtA5093-OtA5097
Sox15	chr11:69655036-69662642	OtA5088-OtA5092	OtA5093-OtA5097
Stx17	chr4:48124918-48186506	OtA5088-OtA5092	OtA5093-OtA5097
Timm9	chr12:71111427-71136675	OtA5088-OtA5092	OtA5093-OtA5097
Vps72	chr3:95111041-95123051	OtA5088-OtA5092	OtA5093-OtA5097
Zdhhc24	chr19:4878667-4885397	OtA5088-OtA5092	OtA5093-OtA5097
Arap3	chr18:37972622-37998969	OtA5088-OtA5092	OtA5093-OtA5097
Atp5sl	chr7:25619492-25625550	OtA5088-OtA5092	OtA5093-OtA5097
Crabp2	chr3:87948692-87953372	OtA5088-OtA5092	OtA5093-OtA5097
Cyth2	chr7:45806636-45814316	OtA5088-OtA5092	OtA5093-OtA5097
Dvl3	chr16:20517063-20532187	OtA5088-OtA5092	OtA5093-OtA5097
Fam18b	chr11:62879489-62895184	OtA5088-OtA5092	OtA5093-OtA5097
Kcnj8	chr6:142564938-142571356	OtA5088-OtA5092	OtA5093-OtA5097
Larp1b	chr3:40950630-40977793	OtA5088-OtA5092	OtA5093-OtA5097
Ltbp3	chr19:5740903-5771401	OtA5088-OtA5092	OtA5093-OtA5097
Necap2	chr4:141066511-141078345	OtA5088-OtA5092	OtA5093-OtA5097
Nupl2	chr5:24164962-24184008	OtA5088-OtA5092	OtA5093-OtA5097

Ostf1	chr19:18580363-18631813	OtA5088-OtA5092	OtA5093-OtA5097
Pmaip1	chr18:66458603-66465558	OtA5088-OtA5092	OtA5093-OtA5097
Sec24d	chr3:123267495-123365636	OtA5088-OtA5092	OtA5093-OtA5097
Sumo2	chr11:115523108-115536230	OtA5088-OtA5092	OtA5093-OtA5097
Usp18	chr6:121245905-121270917	OtA5088-OtA5092	OtA5093-OtA5097
Tada3	chr6:113366656-113377520	OtA5088-OtA5092	OtA5093-OtA5097
Cables1	chr18:11821087-11945627	OtA5088-OtA5092	OtA5093-OtA5097
Park7	chr4:150897132-150909921	OtA5088-OtA5092	OtA5093-OtA5097
Sgsm2	chr11:74849263-74897080	OtA5088-OtA5092	OtA5093-OtA5097
Snx3	chr10:42502053-42535369	OtA5088-OtA5092	OtA5093-OtA5097
Aldoc	chr11:78324197-78326760	OtA5088-OtA5092	OtA5093-OtA5097
BC051226	chr17:33908181-33909178	OtA5088-OtA5092	OtA5093-OtA5097
Gpr97	chr8:95017691-95045249	OtA5088-OtA5092	OtA5093-OtA5097
Lamc3	chr2:31887280-31946535	OtA5088-OtA5092	OtA5093-OtA5097
Lrrfip2	chr9:111118110-111225668	OtA5088-OtA5092	OtA5093-OtA5097
Phc2	chr4:128654701-128752881	OtA5088-OtA5092	OtA5093-OtA5097
Sdha	chr13:74322254-74350240	OtA5088-OtA5092	OtA5093-OtA5097
Sidt2	chr9:45937874-45955249	OtA5088-OtA5092	OtA5093-OtA5097
Zfp148	chr16:33380774-33503903	OtA5088-OtA5092	OtA5093-OtA5097
Fa2h	chr8:111345137-111393821	OtA5088-OtA5092	OtA5093-OtA5097
Tmem167b	chr3:108556424-108562466	OtA5088-OtA5092	OtA5093-OtA5097
Zkscan1	chr5:138085083-138107822	OtA5088-OtA5092	OtA5093-OtA5097
Add3	chr19:53140444-53247399	OtA5088-OtA5092	OtA5093-OtA5097
Nelf	chr2:25054378-25062881	OtA5088-OtA5092	OtA5093-OtA5097
Zscan20	chr4:128583538-128610098	OtA5088-OtA5092	OtA5093-OtA5097
Npdc1	chr2:25403082-25409494	OtA5088-OtA5092	OtA5093-OtA5097
Cdc25b	chr2:131127411-131604824	OtA5088-OtA5092	OtA5093-OtA5097
Dpysl2	chr14:66802863-66868600	OtA5088-OtA5092	OtA5093-OtA5097
Nus1	chr10:52417546-52440192	OtA5088-OtA5092	OtA5093-OtA5097
Slc38a9	chr13:112660765-112738752	OtA5088-OtA5092	OtA5093-OtA5097
Chst11	chr10:82985496-83195891	OtA5088-OtA5092	OtA5093-OtA5097
Csrp1	chr1:135729196-135752229	OtA5088-OtA5092	OtA5093-OtA5097
H2-Q8	chr17:35394098-35395632	OtA5088-OtA5092	OtA5093-OtA5097
Iws1	chr18:32067733-32104331	OtA5088-OtA5092	OtA5093-OtA5097
Ppap2b	chr4:105157346-105232767	OtA5088-OtA5092	OtA5093-OtA5097
Pus1	chr5:110773666-110780615	OtA5088-OtA5092	OtA5093-OtA5097
Sf3a1	chr11:4160353-4182541	OtA5088-OtA5092	OtA5093-OtA5097
Srpk2	chr5:23434428-23616571	OtA5088-OtA5092	OtA5093-OtA5097
Syce2	chr8:84872110-84893921	OtA5088-OtA5092	OtA5093-OtA5097
Atp13a3	chr16:30312423-31275697	OtA5088-OtA5092	OtA5093-OtA5097
Eif3k	chr7:28971372-28981814	OtA5088-OtA5092	OtA5093-OtA5097
Kdm1b	chr13:47043498-47106221	OtA5088-OtA5092	OtA5093-OtA5097
Slc43a2	chr11:75531693-75577572	OtA5088-OtA5092	OtA5093-OtA5097
Vps4a	chr8:107031325-107045756	OtA5088-OtA5092	OtA5093-OtA5097
Zfp446	chr7:12977847-12985716	OtA5088-OtA5092	OtA5093-OtA5097
Dcaf7	chr11:106036871-106059323	OtA5088-OtA5092	OtA5093-OtA5097
Eif3c	chr7:126546910-126566366	OtA5088-OtA5092	OtA5093-OtA5097

Gnb2	chr5:137528128-137533229	OtA5088-OtA5092	OtA5093-OtA5097
Nedd4l	chr18:64887755-65217826	OtA5088-OtA5092	OtA5093-OtA5097
Vash1	chr12:86678699-86695681	OtA5088-OtA5092	OtA5093-OtA5097
Oasl1	chr5:114923239-114937911	OtA5088-OtA5092	OtA5093-OtA5097
Cmc1	chr9:118064526-118150196	OtA5088-OtA5092	OtA5093-OtA5097
Eif2c4	chr4:126489542-126533458	OtA5088-OtA5092	OtA5093-OtA5097
Lonrf1	chr8:36216063-36249516	OtA5088-OtA5092	OtA5093-OtA5097
Map4k2	chr19:6341249-6353527	OtA5088-OtA5092	OtA5093-OtA5097
Polr3a	chr14:24448693-24487046	OtA5088-OtA5092	OtA5093-OtA5097
Pygl	chr12:70190814-70227683	OtA5088-OtA5092	OtA5093-OtA5097
Rnf139	chr15:58889228-58933730	OtA5088-OtA5092	OtA5093-OtA5097
Rpn1	chr6:88084472-88105304	OtA5088-OtA5092	OtA5093-OtA5097
Timm13	chr10:80879815-80900969	OtA5088-OtA5092	OtA5093-OtA5097
Serpinb9d	chr13:33192958-33203980	OtA5088-OtA5092	OtA5093-OtA5097
Snhg3	chr4:132351932-132353686	OtA5088-OtA5092	OtA5093-OtA5097
Tusc2	chr9:107563254-107566108	OtA5088-OtA5092	OtA5093-OtA5097
Efna1	chr3:89271729-89280951	OtA5088-OtA5092	OtA5093-OtA5097
Hic2	chr16:17233586-17263430	OtA5088-OtA5092	OtA5093-OtA5097
Mgat5	chr1:127204985-127482972	OtA5088-OtA5092	OtA5093-OtA5097
Tmcc2	chr1:132356314-132391281	OtA5088-OtA5092	OtA5093-OtA5097
Ablim2	chr5:35757879-35884979	OtA5088-OtA5092	OtA5093-OtA5097
Cul1	chr6:47454323-47526139	OtA5088-OtA5092	OtA5093-OtA5097
G630090E17Rik	chr10:39946911-39986646	OtA5088-OtA5092	OtA5093-OtA5097
Hoxd8	chr2:74705488-74707307	OtA5088-OtA5092	OtA5093-OtA5097
Ramp2	chr11:101246333-101248250	OtA5088-OtA5092	OtA5093-OtA5097
Rpl27a	chr7:109519194-109522369	OtA5088-OtA5092	OtA5093-OtA5097
Slc4a3	chr1:75546265-75559431	OtA5088-OtA5092	OtA5093-OtA5097
Chchd1	chr14:20702011-20704425	OtA5088-OtA5092	OtA5093-OtA5097
Hist2h4	chr3:96262969-96263317	OtA5088-OtA5092	OtA5093-OtA5097
Inca1	chr11:70688360-70700155	OtA5088-OtA5092	OtA5093-OtA5097
Tmem55b	chr14:50924948-50930849	OtA5088-OtA5092	OtA5093-OtA5097
Vps37a	chr8:40511782-40634792	OtA5088-OtA5092	OtA5093-OtA5097
Zfp593	chr4:134243305-134245873	OtA5088-OtA5092	OtA5093-OtA5097
B3gnt2	chr11:22834738-22860336	OtA5088-OtA5092	OtA5093-OtA5097
Ctdsp2	chr10:126978716-126999975	OtA5088-OtA5092	OtA5093-OtA5097
Lcmt1	chr7:123377981-123430358	OtA5088-OtA5092	OtA5093-OtA5097
Plekho1	chr3:95988835-95995839	OtA5088-OtA5092	OtA5093-OtA5097
Tra2a	chr6:49243920-49264052	OtA5088-OtA5092	OtA5093-OtA5097
BC096441	chr11:69682576-69696098	OtA5088-OtA5092	OtA5093-OtA5097
Cep78	chr19:15955772-15984989	OtA5088-OtA5092	OtA5093-OtA5097
Gas2l1	chr11:5054130-5065327	OtA5088-OtA5092	OtA5093-OtA5097
Mpp6	chr6:50110240-50198598	OtA5088-OtA5092	OtA5093-OtA5097
Mrgpre	chr7:143778362-143784500	OtA5088-OtA5092	OtA5093-OtA5097
Ppih	chr4:119300009-119320523	OtA5088-OtA5092	OtA5093-OtA5097
Rps18	chr17:33951998-33955641	OtA5088-OtA5092	OtA5093-OtA5097
Sub1	chr15:11981338-11996007	OtA5088-OtA5092	OtA5093-OtA5097
Tbc1d19	chr5:53809626-53904380	OtA5088-OtA5092	OtA5093-OtA5097

Zfp629	chr7:127607034-127614433	OtA5088-OtA5092	OtA5093-OtA5097
Armc8	chr9:99456242-99568899	OtA5088-OtA5092	OtA5093-OtA5097
Ndufb4	chr16:37647601-37654368	OtA5088-OtA5092	OtA5093-OtA5097
Dhx58	chr11:100694883-100704271	OtA5088-OtA5092	OtA5093-OtA5097
Srd5a1	chr13:69573448-69611463	OtA5088-OtA5092	OtA5093-OtA5097
Cd164	chr10:41519499-41531042	OtA5088-OtA5092	OtA5093-OtA5097
Mdfi	chr17:47815331-47834691	OtA5088-OtA5092	OtA5093-OtA5097
Zfp523	chr17:28177417-28205886	OtA5088-OtA5092	OtA5093-OtA5097
Rpl35a	chr16:33056452-33060188	OtA5088-OtA5092	OtA5093-OtA5097
Med28	chr5:45520228-45529284	OtA5088-OtA5092	OtA5093-OtA5097
Mllt1	chr17:56892610-56935388	OtA5088-OtA5092	OtA5093-OtA5097
Pik3r4	chr9:105642994-105687655	OtA5088-OtA5092	OtA5093-OtA5097
Tubg1	chr11:101120130-101126419	OtA5088-OtA5092	OtA5093-OtA5097
Oraov1	chr7:144915193-144921137	OtA5088-OtA5092	OtA5093-OtA5097
Trappc4	chr9:44403758-44407548	OtA5088-OtA5092	OtA5093-OtA5097
Dkk3	chr7:112116018-112159057	OtA5088-OtA5092	OtA5093-OtA5097
Pard6a	chr8:105701153-105703494	OtA5088-OtA5092	OtA5093-OtA5097
Wwp1	chr4:19609672-19708993	OtA5088-OtA5092	OtA5093-OtA5097
Slc25a47	chr12:108851128-108856815	OtA5088-OtA5092	OtA5093-OtA5097
Nicn1	chr9:108290456-108296496	OtA5088-OtA5092	OtA5093-OtA5097
Trib1	chr15:59648653-59657099	OtA5088-OtA5092	OtA5093-OtA5097
Aldh4a1	chr4:139622893-139649691	OtA5088-OtA5092	OtA5093-OtA5097
Azi2	chr9:118040498-118063907	OtA5088-OtA5092	OtA5093-OtA5097
B630005N14Rik	chr6:13625674-13677966	OtA5088-OtA5092	OtA5093-OtA5097
Btaf1	chr19:36926078-37014057	OtA5088-OtA5092	OtA5093-OtA5097
Casz1	chr4:148804391-148954892	OtA5088-OtA5092	OtA5093-OtA5097
Cisd3	chr11:97685951-97688625	OtA5088-OtA5092	OtA5093-OtA5097
Cldn1	chr16:26356645-26371839	OtA5088-OtA5092	OtA5093-OtA5097
Clu	chr14:65968482-65981545	OtA5088-OtA5092	OtA5093-OtA5097
Cma1	chr14:55941450-55944661	OtA5088-OtA5092	OtA5093-OtA5097
Copa	chr1:172066012-172122332	OtA5088-OtA5092	OtA5093-OtA5097
Cttn	chr7:143948404-144470935	OtA5088-OtA5092	OtA5093-OtA5097
Derl3	chr10:75893397-75895941	OtA5088-OtA5092	OtA5093-OtA5097
Dpf3	chr12:83269197-83487708	OtA5088-OtA5092	OtA5093-OtA5097
Eef1a1	chr9:78478453-78481724	OtA5088-OtA5092	OtA5093-OtA5097
Elk1	chrX:20933394-20950608	OtA5088-OtA5092	OtA5093-OtA5097
Erap1	chr13:74639871-74691875	OtA5088-OtA5092	OtA5093-OtA5097
Ewsr1	chr11:5069687-5099077	OtA5088-OtA5092	OtA5093-OtA5097
Fbxo18	chr2:11742572-11777527	OtA5088-OtA5092	OtA5093-OtA5097
Gm1943	chr8:109339799-109340908	OtA5088-OtA5092	OtA5093-OtA5097
Larp1	chr11:58009063-58062032	OtA5088-OtA5092	OtA5093-OtA5097
Lipt2	chr7:100159276-100160931	OtA5088-OtA5092	OtA5093-OtA5097
Lmbr1l	chr15:98903920-98918098	OtA5088-OtA5092	OtA5093-OtA5097
Lmbrd1	chr1:24678543-24766301	OtA5088-OtA5092	OtA5093-OtA5097
Mmd	chr11:90249475-90278573	OtA5088-OtA5092	OtA5093-OtA5097
Mrpl37	chr4:107055873-107066866	OtA5088-OtA5092	OtA5093-OtA5097
Mrps16	chr14:20391230-20393555	OtA5088-OtA5092	OtA5093-OtA5097

Nbr1	chr11:101552106-101581951	OtA5088-OtA5092	OtA5093-OtA5097
Ncrna00086	chrX:56374585-56378470	OtA5088-OtA5092	OtA5093-OtA5097
Nek6	chr2:38511696-38587490	OtA5088-OtA5092	OtA5093-OtA5097
Ostc	chr3:130695916-130709444	OtA5088-OtA5092	OtA5093-OtA5097
Pde6d	chr1:86543014-86582501	OtA5088-OtA5092	OtA5093-OtA5097
Peg3	chr7:6705959-6730419	OtA5088-OtA5092	OtA5093-OtA5097
Pias3	chr3:96696374-96708560	OtA5088-OtA5092	OtA5093-OtA5097
Plekha3	chr2:76675314-76697335	OtA5088-OtA5092	OtA5093-OtA5097
Pnpla2	chr7:141455187-141460743	OtA5088-OtA5092	OtA5093-OtA5097
Qprt	chr7:127107769-127122029	OtA5088-OtA5092	OtA5093-OtA5097
Rab28	chr5:41624975-41708155	OtA5088-OtA5092	OtA5093-OtA5097
Recql	chr6:142345696-142387087	OtA5088-OtA5092	OtA5093-OtA5097
Rin1	chr19:5050807-5057071	OtA5088-OtA5092	OtA5093-OtA5097
Rmrp	chr4:43492784-43493059	OtA5088-OtA5092	OtA5093-OtA5097
Snai1	chr2:167538226-167542811	OtA5088-OtA5092	OtA5093-OtA5097
Supt5h	chr7:28314895-28338719	OtA5088-OtA5092	OtA5093-OtA5097
Tk1	chr11:117815518-117839908	OtA5088-OtA5092	OtA5093-OtA5097
Zfp747	chr7:127372536-127376050	OtA5088-OtA5092	OtA5093-OtA5097
Brf1	chr12:112959861-113000621	OtA5088-OtA5092	OtA5093-OtA5097
Insig2	chr1:121304352-121332580	OtA5088-OtA5092	OtA5093-OtA5097
Lpar3	chr3:146220960-146286214	OtA5088-OtA5092	OtA5093-OtA5097
Ntn4	chr10:93641048-93745972	OtA5088-OtA5092	OtA5093-OtA5097
Zfp830	chr11:82764344-82767622	OtA5088-OtA5092	OtA5093-OtA5097
Myo1c	chr11:75651508-75674634	OtA5088-OtA5092	OtA5093-OtA5097
Pdlim7	chr13:55497486-55513439	OtA5088-OtA5092	OtA5093-OtA5097
Zfp385c	chr11:100622954-100650693	OtA5088-OtA5092	OtA5093-OtA5097
Mocs2	chr13:114818236-114829420	OtA5088-OtA5092	OtA5093-OtA5097
Ctns	chr11:73183132-73199019	OtA5088-OtA5092	OtA5093-OtA5097
Deaf1	chr7:141297175-141338783	OtA5088-OtA5092	OtA5093-OtA5097
Eef2k	chr7:120842854-120907219	OtA5088-OtA5092	OtA5093-OtA5097
Mapk6	chr9:75386900-75410014	OtA5088-OtA5092	OtA5093-OtA5097
Rev1	chr1:37998009-38129662	OtA5088-OtA5092	OtA5093-OtA5097
Sirt2	chr7:28766751-28788665	OtA5088-OtA5092	OtA5093-OtA5097
Ankrd42	chr7:92584182-92637142	OtA5088-OtA5092	OtA5093-OtA5097
Aplf	chr6:87628428-87672168	OtA5088-OtA5092	OtA5093-OtA5097
Cdc20	chr4:118428092-118437343	OtA5088-OtA5092	OtA5093-OtA5097
Cluap1	chr16:3909008-3941147	OtA5088-OtA5092	OtA5093-OtA5097
Fam102b	chr3:108970996-109027607	OtA5088-OtA5092	OtA5093-OtA5097
Fez2	chr17:78377884-78418103	OtA5088-OtA5092	OtA5093-OtA5097
Gm9855	chr10:82629837-82690650	OtA5088-OtA5092	OtA5093-OtA5097
Iffo2	chr4:139530547-139620382	OtA5088-OtA5092	OtA5093-OtA5097
Mtcp1	chrX:75404846-75416543	OtA5088-OtA5092	OtA5093-OtA5097
Pom121	chr5:135369952-135394546	OtA5088-OtA5092	OtA5093-OtA5097
Ppap2c	chr10:79526428-79533787	OtA5088-OtA5092	OtA5093-OtA5097
Pygo2	chr3:89430836-89435128	OtA5088-OtA5092	OtA5093-OtA5097
Zfp82	chr7:30056033-30072823	OtA5088-OtA5092	OtA5093-OtA5097
Tmc8	chr11:117782296-117793137	OtA5088-OtA5092	OtA5093-OtA5097

Rragd	chr4:32982997-33022180	OtA5088-OtA5092	OtA5093-OtA5097
Acy3	chr19:3986660-3990005	OtA5088-OtA5092	OtA5093-OtA5097
Apbb2	chr5:66298724-66618817	OtA5088-OtA5092	OtA5093-OtA5097
Asna1	chr8:85017930-85025278	OtA5088-OtA5092	OtA5093-OtA5097
Camk1	chr6:113326975-113343922	OtA5088-OtA5092	OtA5093-OtA5097
Ccdc64	chr5:115649285-115731559	OtA5088-OtA5092	OtA5093-OtA5097
Chst2	chr9:95400925-95407270	OtA5088-OtA5092	OtA5093-OtA5097
Commd5	chr15:76899940-76901297	OtA5088-OtA5092	OtA5093-OtA5097
Dhrs13	chr11:78032312-78037864	OtA5088-OtA5092	OtA5093-OtA5097
Eid2b	chr7:28277705-28280129	OtA5088-OtA5092	OtA5093-OtA5097
Furin	chr7:80389193-80405431	OtA5088-OtA5092	OtA5093-OtA5097
Gabrd	chr4:155384978-155398069	OtA5088-OtA5092	OtA5093-OtA5097
Gabrp	chr11:33550780-33578957	OtA5088-OtA5092	OtA5093-OtA5097
Miip	chr4:147860777-147868719	OtA5088-OtA5092	OtA5093-OtA5097
Pigp	chr16:94358762-94469221	OtA5088-OtA5092	OtA5093-OtA5097
Prrc1	chr18:57354732-57392719	OtA5088-OtA5092	OtA5093-OtA5097
Sat2	chr11:69622108-69623869	OtA5088-OtA5092	OtA5093-OtA5097
Slc40a1	chr1:45908069-45925594	OtA5088-OtA5092	OtA5093-OtA5097
Usp53	chr3:122933600-122984447	OtA5088-OtA5092	OtA5093-OtA5097
Zhx1	chr15:58047002-58076489	OtA5088-OtA5092	OtA5093-OtA5097
Abcf2	chr5:24565340-24577467	OtA5088-OtA5092	OtA5093-OtA5097
Pptc7	chr5:122284397-122324281	OtA5088-OtA5092	OtA5093-OtA5097
Ptplad2	chr4:88412929-88438926	OtA5088-OtA5092	OtA5093-OtA5097
Aprt	chr8:122574636-122576907	OtA5088-OtA5092	OtA5093-OtA5097
Fam105b	chr15:27605920-27630693	OtA5088-OtA5092	OtA5093-OtA5097
Sgms2	chr3:131318985-131344923	OtA5088-OtA5092	OtA5093-OtA5097
Dcakd	chr11:102994055-103017147	OtA5088-OtA5092	OtA5093-OtA5097
Fert2	chr17:63896017-64139496	OtA5088-OtA5092	OtA5093-OtA5097
Mfsd2a	chr4:122946850-122961188	OtA5088-OtA5092	OtA5093-OtA5097
Pdzk1ip1	chr4:115088707-115093894	OtA5088-OtA5092	OtA5093-OtA5097
Cetn3	chr13:81783291-81797157	OtA5088-OtA5092	OtA5093-OtA5097
Slc25a48	chr13:56438354-56472363	OtA5088-OtA5092	OtA5093-OtA5097
Tmem175	chr5:108629809-108660769	OtA5088-OtA5092	OtA5093-OtA5097
Ahsa2	chr11:23306894-23497946	OtA5088-OtA5092	OtA5093-OtA5097
Atp1b3	chr9:96332674-96364299	OtA5088-OtA5092	OtA5093-OtA5097
Bcl2l1	chr2:152780667-152831682	OtA5088-OtA5092	OtA5093-OtA5097
Btd	chr14:31641056-31668197	OtA5088-OtA5092	OtA5093-OtA5097
Cwc27	chr13:104631326-104816953	OtA5088-OtA5092	OtA5093-OtA5097
Dph2	chr4:117888642-117892003	OtA5088-OtA5092	OtA5093-OtA5097
Gtpbp5	chr2:180070592-180085902	OtA5088-OtA5092	OtA5093-OtA5097
Lmbrd2	chr15:9140569-9197450	OtA5088-OtA5092	OtA5093-OtA5097
Lypla1	chr1:4807892-4846735	OtA5088-OtA5092	OtA5093-OtA5097
Med4	chr14:73510048-73518545	OtA5088-OtA5092	OtA5093-OtA5097
Mesdc2	chr7:83891999-83901532	OtA5088-OtA5092	OtA5093-OtA5097
Ncapd3	chr9:27030174-27095315	OtA5088-OtA5092	OtA5093-OtA5097
Papss2	chr19:32595714-32667187	OtA5088-OtA5092	OtA5093-OtA5097
Pex3	chr10:13523841-13563376	OtA5088-OtA5092	OtA5093-OtA5097

Rnf31	chr14:55591789-55603671	OtA5088-OtA5092	OtA5093-OtA5097
Rsl24d1	chr9:73113468-73123333	OtA5088-OtA5092	OtA5093-OtA5097
Slc25a30	chr14:75761998-75787037	OtA5088-OtA5092	OtA5093-OtA5097
Amd2	chr10:40287457-40302188	OtA5088-OtA5092	OtA5093-OtA5097
Atp6v1e1	chr6:120795243-120822685	OtA5088-OtA5092	OtA5093-OtA5097
Cog7	chr7:121922838-121981693	OtA5088-OtA5092	OtA5093-OtA5097
Dync1li2	chr8:104417673-104443047	OtA5088-OtA5092	OtA5093-OtA5097
Fyttd1	chr16:32877783-32908963	OtA5088-OtA5092	OtA5093-OtA5097
Ivd	chr2:118861999-118881357	OtA5088-OtA5092	OtA5093-OtA5097
Srsf2	chr11:116849896-116853094	OtA5088-OtA5092	OtA5093-OtA5097
Taf5	chr19:47067747-47090625	OtA5088-OtA5092	OtA5093-OtA5097
Wdr12	chr1:60076867-60151034	OtA5088-OtA5092	OtA5093-OtA5097
Rbpms	chr8:33782643-33929841	OtA5088-OtA5092	OtA5093-OtA5097
Flad1	chr3:89402672-89411863	OtA5088-OtA5092	OtA5093-OtA5097
Twf2	chr9:106203107-106215387	OtA5088-OtA5092	OtA5093-OtA5097
Gtf3c2	chr5:31156005-31180144	OtA5088-OtA5092	OtA5093-OtA5097
BC024978	chr7:27187005-27204320	OtA5088-OtA5092	OtA5093-OtA5097
Ccar1	chr10:62743927-62792368	OtA5088-OtA5092	OtA5093-OtA5097
Dpy19l3	chr7:35685499-35754454	OtA5088-OtA5092	OtA5093-OtA5097
Aoc2	chr11:101325062-101329694	OtA5088-OtA5092	OtA5093-OtA5097
Gemin7	chr7:19564948-19573343	OtA5088-OtA5092	OtA5093-OtA5097
Psmd9	chr5:123228189-123250125	OtA5088-OtA5092	OtA5093-OtA5097
Gmeb2	chr2:181251450-181287966	OtA5088-OtA5092	OtA5093-OtA5097
Dennd1b	chr1:138963708-139176042	OtA5088-OtA5092	OtA5093-OtA5097
Dennd5b	chr6:148988068-149101680	OtA5088-OtA5092	OtA5093-OtA5097
Dvl1	chr4:155847411-155863353	OtA5088-OtA5092	OtA5093-OtA5097
Il17ra	chr6:120463196-120483727	OtA5088-OtA5092	OtA5093-OtA5097
Morn2	chr17:80238303-80297476	OtA5088-OtA5092	OtA5093-OtA5097
Mthfd2	chr6:83305703-83317604	OtA5088-OtA5092	OtA5093-OtA5097
Ptplad1	chr9:64986982-65021717	OtA5088-OtA5092	OtA5093-OtA5097
Rnf167	chr11:70647588-70651414	OtA5088-OtA5092	OtA5093-OtA5097
Rras2	chr7:114046781-114117781	OtA5088-OtA5092	OtA5093-OtA5097
Tdrd7	chr4:45987461-46034759	OtA5088-OtA5092	OtA5093-OtA5097
Tspo	chr15:83563572-83574203	OtA5088-OtA5092	OtA5093-OtA5097
E130308A19Rik	chr4:59626220-59754303	OtA5088-OtA5092	OtA5093-OtA5097
Preb	chr5:30950065-30960361	OtA5088-OtA5092	OtA5093-OtA5097
Vamp5	chr6:72368048-72380468	OtA5088-OtA5092	OtA5093-OtA5097
Pcdhga11	chr18:36930284-37841872	OtA5088-OtA5092	OtA5093-OtA5097
St7	chr6:17749215-17943022	OtA5088-OtA5092	OtA5093-OtA5097
Sdhc	chr1:171129156-171150603	OtA5088-OtA5092	OtA5093-OtA5097
Slc25a10	chr11:120491836-120501161	OtA5088-OtA5092	OtA5093-OtA5097
Kcnh1	chr1:192190871-192510158	OtA5088-OtA5092	OtA5093-OtA5097
Tubb1	chr2:174450694-174457882	OtA5088-OtA5092	OtA5093-OtA5097
Cep120	chr18:53648150-53744547	OtA5088-OtA5092	OtA5093-OtA5097
Ift27	chr15:78159462-78174108	OtA5088-OtA5092	OtA5093-OtA5097
Marveld3	chr8:109947908-109962205	OtA5088-OtA5092	OtA5093-OtA5097
Srr	chr11:74898079-74925798	OtA5088-OtA5092	OtA5093-OtA5097

Tmem205	chr9:21921008-21935872	OtA5088-OtA5092	OtA5093-OtA5097
Rp9	chr9:22448311-22468356	OtA5088-OtA5092	OtA5093-OtA5097
Adamts4	chr1:171250421-171259922	OtA5088-OtA5092	OtA5093-OtA5097
Apom	chr17:35128996-35131752	OtA5088-OtA5092	OtA5093-OtA5097
Bin3	chr14:70100144-70153791	OtA5088-OtA5092	OtA5093-OtA5097
Camk2d	chr3:126596972-126845054	OtA5088-OtA5092	OtA5093-OtA5097
Cpsf4l	chr11:113698170-113710017	OtA5088-OtA5092	OtA5093-OtA5097
Erbb2	chr11:98412483-98438988	OtA5088-OtA5092	OtA5093-OtA5097
Fam35a	chr14:34237034-34310503	OtA5088-OtA5092	OtA5093-OtA5097
Fam38a	chr8:122476142-122551329	OtA5088-OtA5092	OtA5093-OtA5097
Gfod2	chr8:105716112-105758607	OtA5088-OtA5092	OtA5093-OtA5097
Gm10532	chr18:75431220-75697696	OtA5088-OtA5092	OtA5093-OtA5097
Gtf2h2	chr13:100468576-100492609	OtA5088-OtA5092	OtA5093-OtA5097
Ints1	chr5:139751281-139775678	OtA5088-OtA5092	OtA5093-OtA5097
Pik3r2	chr8:70768180-70776712	OtA5088-OtA5092	OtA5093-OtA5097
Pknox2	chr9:36890982-37147314	OtA5088-OtA5092	OtA5093-OtA5097
Ppil1	chr17:29250834-29263971	OtA5088-OtA5092	OtA5093-OtA5097
Ptrh1	chr2:32775820-32784405	OtA5088-OtA5092	OtA5093-OtA5097
Tfpt	chr7:3620323-3629884	OtA5088-OtA5092	OtA5093-OtA5097
Tshz2	chr2:169633645-169888504	OtA5088-OtA5092	OtA5093-OtA5097
Zbtb40	chr4:136979731-137048695	OtA5088-OtA5092	OtA5093-OtA5097
Zbtb43	chr2:33450287-33468532	OtA5088-OtA5092	OtA5093-OtA5097
Prkx	chrX:77762029-77795960	OtA5088-OtA5092	OtA5093-OtA5097
Uqcrh	chr4:116066964-116075070	OtA5088-OtA5092	OtA5093-OtA5097
Gltscr2	chr7:15937835-15946108	OtA5088-OtA5092	OtA5093-OtA5097
Exoc1	chr5:76529322-76570285	OtA5088-OtA5092	OtA5093-OtA5097
Zfp498	chr5:145283342-145291469	OtA5088-OtA5092	OtA5093-OtA5097
Arntl	chr7:113207464-113314126	OtA5088-OtA5092	OtA5093-OtA5097
Cdkn1a	chr17:29090985-29100722	OtA5088-OtA5092	OtA5093-OtA5097
Gtf3c1	chr7:125640953-125707688	OtA5088-OtA5092	OtA5093-OtA5097
Ik	chr18:36744655-36757639	OtA5088-OtA5092	OtA5093-OtA5097
Zfp526	chr7:25221450-25227505	OtA5088-OtA5092	OtA5093-OtA5097
Agpat9	chr5:100846228-100899102	OtA5088-OtA5092	OtA5093-OtA5097
Pgap2	chr7:102223073-102237751	OtA5088-OtA5092	OtA5093-OtA5097
Stap2	chr17:55997077-56005568	OtA5088-OtA5092	OtA5093-OtA5097
Trim21	chr7:102557921-102565469	OtA5088-OtA5092	OtA5093-OtA5097
Wdyhv1	chr15:58141435-58158654	OtA5088-OtA5092	OtA5093-OtA5097
Ccdc85b	chr19:5447697-5457563	OtA5088-OtA5092	OtA5093-OtA5097
Creb3	chr4:43562633-43587487	OtA5088-OtA5092	OtA5093-OtA5097
Gtpbp3	chr8:71488102-71493400	OtA5088-OtA5092	OtA5093-OtA5097
Plscr3	chr11:69846371-69858730	OtA5088-OtA5092	OtA5093-OtA5097
Rapgef1	chr2:29619719-29740363	OtA5088-OtA5092	OtA5093-OtA5097
Rfxap	chr3:54803114-54807791	OtA5088-OtA5092	OtA5093-OtA5097
Rrs1	chr1:9545407-9547455	OtA5088-OtA5092	OtA5093-OtA5097
Tsfm	chr10:127022331-127030814	OtA5088-OtA5092	OtA5093-OtA5097
Bend3	chr10:43479139-43515417	OtA5088-OtA5092	OtA5093-OtA5097
Snhg6	chr1:9942024-9944118	OtA5088-OtA5092	OtA5093-OtA5097

Ndufb3	chr1:58586598-58595948	OtA5088-OtA5092	OtA5093-OtA5097
Paxip1	chr5:27740664-27791339	OtA5088-OtA5092	OtA5093-OtA5097
Ankrd33b	chr15:31291478-31367759	OtA5088-OtA5092	OtA5093-OtA5097
Crebl2	chr6:134830198-134857883	OtA5088-OtA5092	OtA5093-OtA5097
Fyco1	chr9:123789509-123851899	OtA5088-OtA5092	OtA5093-OtA5097
Ptp4a3	chr15:73723144-73758766	OtA5088-OtA5092	OtA5093-OtA5097
Tarbp2	chr15:102518191-102523676	OtA5088-OtA5092	OtA5093-OtA5097
Zfand1	chr3:10339955-10351301	OtA5088-OtA5092	OtA5093-OtA5097
Nf1	chr11:79339891-79581609	OtA5088-OtA5092	OtA5093-OtA5097
Apbb1	chr7:105558464-105581653	OtA5088-OtA5092	OtA5093-OtA5097
Ccdc102a	chr8:94902868-94918098	OtA5088-OtA5092	OtA5093-OtA5097
B9d1	chr11:61505171-61512927	OtA5088-OtA5092	OtA5093-OtA5097
Lamc1	chr1:153218921-153332786	OtA5088-OtA5092	OtA5093-OtA5097
Tbc1d1	chr5:64160210-64351486	OtA5088-OtA5092	OtA5093-OtA5097
Tmx3	chr18:90510153-90543267	OtA5088-OtA5092	OtA5093-OtA5097
Vps53	chr11:76046225-76179630	OtA5088-OtA5092	OtA5093-OtA5097
Tns3	chr11:8431651-8664535	OtA5088-OtA5092	OtA5093-OtA5097
Mrpl4	chr9:21002736-21008837	OtA5088-OtA5092	OtA5093-OtA5097
Clint1	chr11:45851963-45910625	OtA5088-OtA5092	OtA5093-OtA5097
Pacs2	chr12:113014507-113074401	OtA5088-OtA5092	OtA5093-OtA5097
Hkdc1	chr10:62383136-62422457	OtA5088-OtA5092	OtA5093-OtA5097
Aatk	chr11:120007315-120047145	OtA5088-OtA5092	OtA5093-OtA5097
Arhgef7	chr8:11728104-11835217	OtA5088-OtA5092	OtA5093-OtA5097
Armc9	chr1:86154779-86278284	OtA5088-OtA5092	OtA5093-OtA5097
B3gnt5	chr16:19760233-19772753	OtA5088-OtA5092	OtA5093-OtA5097
B930041F14Rik	chr4:155694341-155696483	OtA5088-OtA5092	OtA5093-OtA5097
Cdh5	chr8:104101624-104144502	OtA5088-OtA5092	OtA5093-OtA5097
Chchd3	chr6:32792226-33060152	OtA5088-OtA5092	OtA5093-OtA5097
Csnk1a1	chr18:61555581-61588299	OtA5088-OtA5092	OtA5093-OtA5097
E030010A14Rik	chr19:24674007-24861842	OtA5088-OtA5092	OtA5093-OtA5097
Eepd1	chr9:25481596-25604110	OtA5088-OtA5092	OtA5093-OtA5097
Fmr1nb	chrX:68761838-68804560	OtA5088-OtA5092	OtA5093-OtA5097
Fntb	chr12:76837466-76921412	OtA5088-OtA5092	OtA5093-OtA5097
Fzr1	chr10:81366878-81378370	OtA5088-OtA5092	OtA5093-OtA5097
Gnb1	chr4:155491360-155559269	OtA5088-OtA5092	OtA5093-OtA5097
Hcfc1r1	chr17:23660522-23677449	OtA5088-OtA5092	OtA5093-OtA5097
Irgm1	chr11:48865248-48871346	OtA5088-OtA5092	OtA5093-OtA5097
Klhdc5	chr6:147091074-147112778	OtA5088-OtA5092	OtA5093-OtA5097
Klhdc8b	chr9:108447638-108465945	OtA5088-OtA5092	OtA5093-OtA5097
Krt80	chr15:101349570-101370125	OtA5088-OtA5092	OtA5093-OtA5097
Leng9	chr7:4148182-4149872	OtA5088-OtA5092	OtA5093-OtA5097
Lin7c	chr2:109890877-109900936	OtA5088-OtA5092	OtA5093-OtA5097
Mpzl1	chr1:165592180-165634541	OtA5088-OtA5092	OtA5093-OtA5097
Mrfap1	chr5:36794866-36796754	OtA5088-OtA5092	OtA5093-OtA5097
Ndufb7	chr8:83566757-83571623	OtA5088-OtA5092	OtA5093-OtA5097
Nrp2	chr1:62703316-62818692	OtA5088-OtA5092	OtA5093-OtA5097
Ogg1	chr6:113326975-113343922	OtA5088-OtA5092	OtA5093-OtA5097

Ranbp3	chr17:56673224-56711769	OtA5088-OtA5092	OtA5093-OtA5097
Rnaset2b	chr17:8128590-8147832	OtA5088-OtA5092	OtA5093-OtA5097
S100a14	chr3:90526848-90528835	OtA5088-OtA5092	OtA5093-OtA5097
Scaf1	chr7:45002949-45016249	OtA5088-OtA5092	OtA5093-OtA5097
Slc26a11	chr11:119355556-119381076	OtA5088-OtA5092	OtA5093-OtA5097
Sparcl1	chr5:104079108-104114088	OtA5088-OtA5092	OtA5093-OtA5097
Tonsl	chr15:76626236-76639929	OtA5088-OtA5092	OtA5093-OtA5097
Trrap	chr5:144768791-144859773	OtA5088-OtA5092	OtA5093-OtA5097
Ubr5	chr15:37967327-38078853	OtA5088-OtA5092	OtA5093-OtA5097
Usp15	chr10:123113243-123196923	OtA5088-OtA5092	OtA5093-OtA5097
Uxs1	chr1:43750230-43827708	OtA5088-OtA5092	OtA5093-OtA5097
Vcam1	chr3:116110019-116129688	OtA5088-OtA5092	OtA5093-OtA5097
Zbtb1	chr12:76370265-76388747	OtA5088-OtA5092	OtA5093-OtA5097
Zdhhc20	chr14:57832701-57890262	OtA5088-OtA5092	OtA5093-OtA5097
Dbn1	chr13:55473427-55488076	OtA5088-OtA5092	OtA5093-OtA5097
Zbtb39	chr10:127739537-127747339	OtA5088-OtA5092	OtA5093-OtA5097
Pogz	chr3:94837566-94883567	OtA5088-OtA5092	OtA5093-OtA5097
Ptgfrn	chr3:101040235-101110166	OtA5088-OtA5092	OtA5093-OtA5097
Fbxl18	chr5:142871788-142895238	OtA5088-OtA5092	OtA5093-OtA5097
Ryk	chr9:102834919-102908307	OtA5088-OtA5092	OtA5093-OtA5097
Ahcyl2	chr6:29768442-29912310	OtA5088-OtA5092	OtA5093-OtA5097
Calm4	chr13:3837756-3838671	OtA5088-OtA5092	OtA5093-OtA5097
Dnajb3	chr1:88055410-88220002	OtA5088-OtA5092	OtA5093-OtA5097
Fbxo6	chr4:148145715-148152135	OtA5088-OtA5092	OtA5093-OtA5097
Hist1h2bp	chr13:21787487-21789213	OtA5088-OtA5092	OtA5093-OtA5097
Ier3ip1	chr18:76930026-76941614	OtA5088-OtA5092	OtA5093-OtA5097
Mtmr6	chr14:60265204-60302371	OtA5088-OtA5092	OtA5093-OtA5097
Tpbpa	chr13:60938691-60941935	OtA5088-OtA5092	OtA5093-OtA5097
Urm1	chr2:29827388-29844996	OtA5088-OtA5092	OtA5093-OtA5097
Zdhhc8	chr16:18220752-18235136	OtA5088-OtA5092	OtA5093-OtA5097
Zfp235	chr7:24134162-24143241	OtA5088-OtA5092	OtA5093-OtA5097
Gimap4	chr6:48684577-48692062	OtA5088-OtA5092	OtA5093-OtA5097
Akap2	chr4:57845247-57896984	OtA5088-OtA5092	OtA5093-OtA5097
Azin1	chr15:38487429-38519266	OtA5088-OtA5092	OtA5093-OtA5097
Esd	chr14:74732344-74750443	OtA5088-OtA5092	OtA5093-OtA5097
Mbp	chr18:82475122-82585637	OtA5088-OtA5092	OtA5093-OtA5097
Tollip	chr7:141881584-141902406	OtA5088-OtA5092	OtA5093-OtA5097
Asph	chr4:9269316-9669162	OtA5088-OtA5092	OtA5093-OtA5097
Atp2c1	chr9:105411361-105521257	OtA5088-OtA5092	OtA5093-OtA5097
Ccdc42	chr11:68587036-68597951	OtA5088-OtA5092	OtA5093-OtA5097
Ccdc94	chr17:55959186-55967951	OtA5088-OtA5092	OtA5093-OtA5097
Cox7a2	chr9:79755240-79759853	OtA5088-OtA5092	OtA5093-OtA5097
Heatr5a	chr12:51875872-51971321	OtA5088-OtA5092	OtA5093-OtA5097
Mbd1	chr18:74268287-74282684	OtA5088-OtA5092	OtA5093-OtA5097
Mff	chr1:82724917-82752389	OtA5088-OtA5092	OtA5093-OtA5097
Nudt17	chr3:96696374-96708560	OtA5088-OtA5092	OtA5093-OtA5097
Psg-ps1	chr7:17672312-17682060	OtA5088-OtA5092	OtA5093-OtA5097

S100a13	chr3:90514434-90524581	OtA5088-OtA5092	OtA5093-OtA5097
Tmem104	chr11:115187486-115247025	OtA5088-OtA5092	OtA5093-OtA5097
Vgll3	chr16:65815632-65863066	OtA5088-OtA5092	OtA5093-OtA5097
Arhgap24	chr5:102481390-102897937	OtA5088-OtA5092	OtA5093-OtA5097
Aste1	chr9:105395391-105405756	OtA5088-OtA5092	OtA5093-OtA5097
Banp	chr8:121950532-122029258	OtA5088-OtA5092	OtA5093-OtA5097
Canx	chr11:50293956-50325673	OtA5088-OtA5092	OtA5093-OtA5097
Cul5	chr9:53614581-53667507	OtA5088-OtA5092	OtA5093-OtA5097
Dbndd2	chr2:164486139-164493323	OtA5088-OtA5092	OtA5093-OtA5097
E330020D12Rik	chr1:153404185-153414233	OtA5088-OtA5092	OtA5093-OtA5097
Eef1b2	chr1:63176830-63180486	OtA5088-OtA5092	OtA5093-OtA5097
Mcph1	chr8:18595172-18803188	OtA5088-OtA5092	OtA5093-OtA5097
Nsdhl	chrX:72918520-72958528	OtA5088-OtA5092	OtA5093-OtA5097
Spopl	chr2:23510053-23572104	OtA5088-OtA5092	OtA5093-OtA5097
Tnnc1	chr14:31208311-31211711	OtA5088-OtA5092	OtA5093-OtA5097
Zfp868	chr8:69610653-69625548	OtA5088-OtA5092	OtA5093-OtA5097
Atp6v1h	chr1:5083172-5162549	OtA5088-OtA5092	OtA5093-OtA5097
Mcart1	chr4:45395923-45408766	OtA5088-OtA5092	OtA5093-OtA5097
Mrpl42	chr10:95480805-95501927	OtA5088-OtA5092	OtA5093-OtA5097
Rft1	chr14:30654374-30691313	OtA5088-OtA5092	OtA5093-OtA5097
Tmem85	chr2:112266313-112368027	OtA5088-OtA5092	OtA5093-OtA5097
Zmat5	chr11:4704677-4737666	OtA5088-OtA5092	OtA5093-OtA5097
Atg12	chr18:46732416-46741579	OtA5088-OtA5092	OtA5093-OtA5097
Atp10a	chr7:58658201-58829426	OtA5088-OtA5092	OtA5093-OtA5097
Nr1h3	chr2:91184060-91195116	OtA5088-OtA5092	OtA5093-OtA5097
Spire2	chr8:123332712-123369518	OtA5088-OtA5092	OtA5093-OtA5097
Clpb	chr7:101663767-101790168	OtA5088-OtA5092	OtA5093-OtA5097
Cbx8	chr11:119038435-119040913	OtA5088-OtA5092	OtA5093-OtA5097
Cyfip2	chr11:46193848-46312859	OtA5088-OtA5092	OtA5093-OtA5097
D230025D16Rik	chr8:105225187-105255151	OtA5088-OtA5092	OtA5093-OtA5097
Dnaja4	chr9:54699558-54716317	OtA5088-OtA5092	OtA5093-OtA5097
Epha1	chr6:42358486-42380558	OtA5088-OtA5092	OtA5093-OtA5097
Gm16516	chr11:60920940-60931867	OtA5088-OtA5092	OtA5093-OtA5097
Hat1	chr2:71389259-71441622	OtA5088-OtA5092	OtA5093-OtA5097
Hcn2	chr10:79697304-80369637	OtA5088-OtA5092	OtA5093-OtA5097
Heatr6	chr11:83753636-83783754	OtA5088-OtA5092	OtA5093-OtA5097
Hist1h4n	chr13:21811745-21812150	OtA5088-OtA5092	OtA5093-OtA5097
Hsp90ab1	chr17:45567777-45573261	OtA5088-OtA5092	OtA5093-OtA5097
Myo7a	chr7:98051053-98119493	OtA5088-OtA5092	OtA5093-OtA5097
Opa3	chr7:19228388-19246817	OtA5088-OtA5092	OtA5093-OtA5097
Oxct1	chr15:4020110-4155344	OtA5088-OtA5092	OtA5093-OtA5097
Pdik1l	chr4:134275004-134287846	OtA5088-OtA5092	OtA5093-OtA5097
Prkag2	chr5:24862734-25100642	OtA5088-OtA5092	OtA5093-OtA5097
Ptgr2	chr12:84285295-84315832	OtA5088-OtA5092	OtA5093-OtA5097
Rpl30	chr15:34440505-34443276	OtA5088-OtA5092	OtA5093-OtA5097
Six5	chr7:19094543-19098345	OtA5088-OtA5092	OtA5093-OtA5097
Snx33	chr9:56917199-56928371	OtA5088-OtA5092	OtA5093-OtA5097

Strn4	chr7:16809266-16840931	OtA5088-OtA5092	OtA5093-OtA5097
Tmem183a	chr1:134346096-134361999	OtA5088-OtA5092	OtA5093-OtA5097
Tnip2	chr5:34496095-34513979	OtA5088-OtA5092	OtA5093-OtA5097
Ubap1	chr4:41348995-41389766	OtA5088-OtA5092	OtA5093-OtA5097
Vgll1	chrX:57088105-57105446	OtA5088-OtA5092	OtA5093-OtA5097
Wdr7	chr18:63708694-63989759	OtA5088-OtA5092	OtA5093-OtA5097
Neu3	chr7:99811438-99828417	OtA5088-OtA5092	OtA5093-OtA5097
Ctsb	chr14:63122461-63177793	OtA5088-OtA5092	OtA5093-OtA5097
Gpank1	chr17:35121495-35124815	OtA5088-OtA5092	OtA5093-OtA5097
Gstk1	chr6:42245934-42250441	OtA5088-OtA5092	OtA5093-OtA5097
Ubqln1	chr13:58176155-58215653	OtA5088-OtA5092	OtA5093-OtA5097
Rnf144a	chr12:26306797-26415256	OtA5088-OtA5092	OtA5093-OtA5097
E2f4	chr8:105297662-105305370	OtA5088-OtA5092	OtA5093-OtA5097
Hnrnpa1	chr15:103240431-103244955	OtA5088-OtA5092	OtA5093-OtA5097
Kptn	chr7:16119875-16127516	OtA5088-OtA5092	OtA5093-OtA5097
Ifnar1	chr16:91485214-91510522	OtA5088-OtA5092	OtA5093-OtA5097
Eif3a	chr19:60761115-60790693	OtA5088-OtA5092	OtA5093-OtA5097
Polr1c	chr17:46243919-46248045	OtA5088-OtA5092	OtA5093-OtA5097
Exoc5	chr14:49012143-49087723	OtA5088-OtA5092	OtA5093-OtA5097
Rpl22l1	chr3:28805510-28807415	OtA5088-OtA5092	OtA5093-OtA5097
Slc12a6	chr2:112266313-112368027	OtA5088-OtA5092	OtA5093-OtA5097
Mocs1	chr17:49428363-49455430	OtA5088-OtA5092	OtA5093-OtA5097
Saal1	chr7:46686107-46710651	OtA5088-OtA5092	OtA5093-OtA5097
Ap1g1	chr8:109778639-109864208	OtA5088-OtA5092	OtA5093-OtA5097
Cd14	chr18:36725066-36726654	OtA5088-OtA5092	OtA5093-OtA5097
Rcbtb2	chr14:73142509-73184054	OtA5088-OtA5092	OtA5093-OtA5097
Rftn1	chr17:49993306-50190497	OtA5088-OtA5092	OtA5093-OtA5097
Sh2b2	chr5:136218148-136244903	OtA5088-OtA5092	OtA5093-OtA5097
Ngdn	chr14:55015453-55024137	OtA5088-OtA5092	OtA5093-OtA5097
Adamts14	chr10:61197111-61273438	OtA5088-OtA5092	OtA5093-OtA5097
Unc50	chr1:37430171-37439124	OtA5088-OtA5092	OtA5093-OtA5097
Clcc1	chr3:108653912-108722299	OtA5088-OtA5092	OtA5093-OtA5097
Bcat1	chr6:144993834-145076157	OtA5088-OtA5092	OtA5093-OtA5097
Cnot10	chr9:114585873-114640200	OtA5088-OtA5092	OtA5093-OtA5097
Mrpl51	chr6:125192199-125194392	OtA5088-OtA5092	OtA5093-OtA5097
Ninj1	chr13:49187546-49196251	OtA5088-OtA5092	OtA5093-OtA5097
Nxf1	chr19:8757102-8770910	OtA5088-OtA5092	OtA5093-OtA5097
Ppl	chr16:5086290-5132481	OtA5088-OtA5092	OtA5093-OtA5097
Ufsp2	chr8:45975538-45996859	OtA5088-OtA5092	OtA5093-OtA5097
Zfp276	chr8:123254194-123318576	OtA5088-OtA5092	OtA5093-OtA5097
Atg2a	chr19:6241667-6262304	OtA5088-OtA5092	OtA5093-OtA5097
Cyp2u1	chr3:131290490-131303227	OtA5088-OtA5092	OtA5093-OtA5097
Lclat1	chr17:73107984-73243366	OtA5088-OtA5092	OtA5093-OtA5097
Mut	chr17:40934684-40961989	OtA5088-OtA5092	OtA5093-OtA5097
Nudcd1	chr15:44375226-44437685	OtA5088-OtA5092	OtA5093-OtA5097
Prkab1	chr5:116013589-116024428	OtA5088-OtA5092	OtA5093-OtA5097
Tpbg	chr9:85842379-85847055	OtA5088-OtA5092	OtA5093-OtA5097

Tstd2	chr4:46039221-46138475	OtA5088-OtA5092	OtA5093-OtA5097
Dmrtc1b	chrX:102707880-102715209	OtA5088-OtA5092	OtA5093-OtA5097
Phldb2	chr16:45746230-45844378	OtA5088-OtA5092	OtA5093-OtA5097
Tmco1	chr1:167308669-167333978	OtA5088-OtA5092	OtA5093-OtA5097
Slc3a1	chr17:85028346-85090274	OtA5088-OtA5092	OtA5093-OtA5097
Arhgap8	chr15:84714628-84772207	OtA5088-OtA5092	OtA5093-OtA5097
Clcn2	chr16:20695056-20716636	OtA5088-OtA5092	OtA5093-OtA5097
Tmem88	chr11:69395790-69398234	OtA5088-OtA5092	OtA5093-OtA5097
Cdc42bpg	chr19:6306456-6325652	OtA5088-OtA5092	OtA5093-OtA5097
Flt1	chr5:147562195-147725988	OtA5088-OtA5092	OtA5093-OtA5097
Gng10	chr4:59035155-59041899	OtA5088-OtA5092	OtA5093-OtA5097
Ubr4	chr4:139380658-139489532	OtA5088-OtA5092	OtA5093-OtA5097
Srsf6	chr2:162931507-162937120	OtA5088-OtA5092	OtA5093-OtA5097
Tmsb10	chr6:72957346-72958748	OtA5088-OtA5092	OtA5093-OtA5097
Dgka	chr10:128706257-128744056	OtA5088-OtA5092	OtA5093-OtA5097
Lphn1	chr8:83900097-83941954	OtA5088-OtA5092	OtA5093-OtA5097
Sfxn2	chr19:46573364-46596900	OtA5088-OtA5092	OtA5093-OtA5097
LOC545261	chr18:61639652-61665538	OtA5088-OtA5092	OtA5093-OtA5097
Rhpn1	chr15:75704287-75714419	OtA5088-OtA5092	OtA5093-OtA5097
Sh3rf2	chr18:42053709-42158695	OtA5088-OtA5092	OtA5093-OtA5097
Slc33a1	chr3:63942335-63964733	OtA5088-OtA5092	OtA5093-OtA5097
Tm4sf1	chr3:57287063-57301919	OtA5088-OtA5092	OtA5093-OtA5097
Tm9sf2	chr14:122107081-122159603	OtA5088-OtA5092	OtA5093-OtA5097
Umps	chr16:33955011-33967003	OtA5088-OtA5092	OtA5093-OtA5097
Btf3	chr13:98309896-98317006	OtA5088-OtA5092	OtA5093-OtA5097
Dhodh	chr8:109593247-109608673	OtA5088-OtA5092	OtA5093-OtA5097
Gtf2f2	chr14:75896936-76010865	OtA5088-OtA5092	OtA5093-OtA5097
Impad1	chr4:4764350-4793306	OtA5088-OtA5092	OtA5093-OtA5097
Itpripl2	chr7:118485111-118491975	OtA5088-OtA5092	OtA5093-OtA5097
Lsm7	chr10:80852824-80855209	OtA5088-OtA5092	OtA5093-OtA5097
Ptgs1	chr2:36230425-36252269	OtA5088-OtA5092	OtA5093-OtA5097
Rpl24	chr16:55966274-55971437	OtA5088-OtA5092	OtA5093-OtA5097
Sap30	chr8:57482701-57487860	OtA5088-OtA5092	OtA5093-OtA5097
Supt3h	chr17:44604000-45119284	OtA5088-OtA5092	OtA5093-OtA5097
Ythdf1	chr2:180904376-180920936	OtA5088-OtA5092	OtA5093-OtA5097
Anapc2	chr2:25272465-25285916	OtA5088-OtA5092	OtA5093-OtA5097
Kif9	chr9:110476957-110525174	OtA5088-OtA5092	OtA5093-OtA5097
Mtmr7	chr8:40511782-40634792	OtA5088-OtA5092	OtA5093-OtA5097
Cdt1	chr8:122568014-122573130	OtA5088-OtA5092	OtA5093-OtA5097
Ldoc1l	chr15:84553397-84557823	OtA5088-OtA5092	OtA5093-OtA5097
Mars2	chr1:55237176-55240058	OtA5088-OtA5092	OtA5093-OtA5097
Phb2	chr6:124712288-124716945	OtA5088-OtA5092	OtA5093-OtA5097
Cdc14b	chr13:64192544-64274988	OtA5088-OtA5092	OtA5093-OtA5097
Jup	chr11:100368855-100397790	OtA5088-OtA5092	OtA5093-OtA5097
Cpne2	chr8:94533027-94570529	OtA5088-OtA5092	OtA5093-OtA5097
Gas8	chr8:123518834-123536650	OtA5088-OtA5092	OtA5093-OtA5097
Gtpbp6	chr5:110099968-110108197	OtA5088-OtA5092	OtA5093-OtA5097

Tmem219	chr7:126886218-126898278	OtA5088-OtA5092	OtA5093-OtA5097
Aqp7	chr4:41033073-41048136	OtA5088-OtA5092	OtA5093-OtA5097
Armc6	chr8:70220192-70263105	OtA5088-OtA5092	OtA5093-OtA5097
Clic3	chr2:25456842-25458772	OtA5088-OtA5092	OtA5093-OtA5097
Cpt1c	chr7:44959371-44974851	OtA5088-OtA5092	OtA5093-OtA5097
Crkl	chr16:17451986-17486255	OtA5088-OtA5092	OtA5093-OtA5097
D19Ertd737e	chr19:60198585-60226697	OtA5088-OtA5092	OtA5093-OtA5097
Dcbld1	chr10:52233618-52321377	OtA5088-OtA5092	OtA5093-OtA5097
Dpm3	chr3:89266551-89267077	OtA5088-OtA5092	OtA5093-OtA5097
Eaf1	chr14:31495078-31509858	OtA5088-OtA5092	OtA5093-OtA5097
Efr3a	chr15:65787040-65873812	OtA5088-OtA5092	OtA5093-OtA5097
Elf1	chr14:79459936-79582476	OtA5088-OtA5092	OtA5093-OtA5097
Erp29	chr5:121444752-121452474	OtA5088-OtA5092	OtA5093-OtA5097
Fbxo44	chr4:148152799-148160064	OtA5088-OtA5092	OtA5093-OtA5097
Fcgrt	chr7:45092992-45103822	OtA5088-OtA5092	OtA5093-OtA5097
Fchsd1	chr18:37955558-37969731	OtA5088-OtA5092	OtA5093-OtA5097
Fpgt	chr3:155084918-155093378	OtA5088-OtA5092	OtA5093-OtA5097
Fus	chr7:127967478-127982031	OtA5088-OtA5092	OtA5093-OtA5097
Gm5918	chr9:72979724-72985376	OtA5088-OtA5092	OtA5093-OtA5097
Gpsm2	chr3:108653912-108722299	OtA5088-OtA5092	OtA5093-OtA5097
Hbs1l	chr10:21295978-21368889	OtA5088-OtA5092	OtA5093-OtA5097
Iars2	chr1:185204167-185329401	OtA5088-OtA5092	OtA5093-OtA5097
Kctd9	chr14:67716097-67742310	OtA5088-OtA5092	OtA5093-OtA5097
Lbr	chr1:181815314-181842401	OtA5088-OtA5092	OtA5093-OtA5097
Lrig1	chr6:94500313-94700145	OtA5088-OtA5092	OtA5093-OtA5097
Mark4	chr7:19426074-19458494	OtA5088-OtA5092	OtA5093-OtA5097
Mettl1	chr10:127041931-127045461	OtA5088-OtA5092	OtA5093-OtA5097
Mmrn2	chr14:34375503-34404286	OtA5088-OtA5092	OtA5093-OtA5097
Morf4l1	chr9:90091668-90114820	OtA5088-OtA5092	OtA5093-OtA5097
Mospd2	chrX:164938208-164980335	OtA5088-OtA5092	OtA5093-OtA5097
Naca	chr10:128035345-128048637	OtA5088-OtA5092	OtA5093-OtA5097
Nfix	chr8:84707598-84800340	OtA5088-OtA5092	OtA5093-OtA5097
Notch2	chr3:98013537-98150367	OtA5088-OtA5092	OtA5093-OtA5097
Nudt16l1	chr16:4886099-4941020	OtA5088-OtA5092	OtA5093-OtA5097
Osgin1	chr8:119437161-119446256	OtA5088-OtA5092	OtA5093-OtA5097
Pbx1	chr1:168119363-168432258	OtA5088-OtA5092	OtA5093-OtA5097
Pcbp4	chr9:106453837-106465940	OtA5088-OtA5092	OtA5093-OtA5097
Pik3ap1	chr19:41274217-41385070	OtA5088-OtA5092	OtA5093-OtA5097
Plrg1	chr3:83055537-83072291	OtA5088-OtA5092	OtA5093-OtA5097
Pmm2	chr16:8637706-8657524	OtA5088-OtA5092	OtA5093-OtA5097
Ppwd1	chr13:104205121-104228844	OtA5088-OtA5092	OtA5093-OtA5097
Prkaa1	chr15:5143860-5181899	OtA5088-OtA5092	OtA5093-OtA5097
Prpf8	chr11:75486776-75509447	OtA5088-OtA5092	OtA5093-OtA5097
Rarres2	chr6:48554798-48572670	OtA5088-OtA5092	OtA5093-OtA5097
Sdcbp2	chr2:151572631-151589999	OtA5088-OtA5092	OtA5093-OtA5097
Sdhaf1	chr7:30321408-30322375	OtA5088-OtA5092	OtA5093-OtA5097
Sdhaf2	chr19:10500511-10525209	OtA5088-OtA5092	OtA5093-OtA5097

Serbp1	chr6:67266978-67289302	OtA5088-OtA5092	OtA5093-OtA5097
Sipa1	chr19:5651184-5663707	OtA5088-OtA5092	OtA5093-OtA5097
Slc25a22	chr7:141429748-141437874	OtA5088-OtA5092	OtA5093-OtA5097
Sned1	chr1:93235896-93296448	OtA5088-OtA5092	OtA5093-OtA5097
Srf	chr17:46546838-46556162	OtA5088-OtA5092	OtA5093-OtA5097
St3gal2	chr8:110919864-110972497	OtA5088-OtA5092	OtA5093-OtA5097
St3gal5	chr6:72097612-72154570	OtA5088-OtA5092	OtA5093-OtA5097
Tgoln1,Tgoln2	chr6:72608420-72617000	OtA5088-OtA5092	OtA5093-OtA5097
Tmc4	chr7:3645268-3677553	OtA5088-OtA5092	OtA5093-OtA5097
Tmem50b	chr16:91574507-91597680	OtA5088-OtA5092	OtA5093-OtA5097
Tsr2	chrX:151047232-151096543	OtA5088-OtA5092	OtA5093-OtA5097
Vps16	chr2:130417683-130444269	OtA5088-OtA5092	OtA5093-OtA5097
Wars	chr12:108860029-108894174	OtA5088-OtA5092	OtA5093-OtA5097
Xpnpep1	chr19:52991182-53038542	OtA5088-OtA5092	OtA5093-OtA5097
Ythdf3	chr3:16183182-16217037	OtA5088-OtA5092	OtA5093-OtA5097
Zfp57	chr17:37001162-37010729	OtA5088-OtA5092	OtA5093-OtA5097
Fxyd1	chr7:31051677-31055656	OtA5088-OtA5092	OtA5093-OtA5097
Tmod1	chr4:46039221-46138475	OtA5088-OtA5092	OtA5093-OtA5097
Chmp3	chr6:71543853-71581574	OtA5088-OtA5092	OtA5093-OtA5097
C330018D20Rik	chr18:56955830-56975379	OtA5088-OtA5092	OtA5093-OtA5097
Lace1	chr10:42312584-42478565	OtA5088-OtA5092	OtA5093-OtA5097
Sirt3	chr7:140857396-140882309	OtA5088-OtA5092	OtA5093-OtA5097
Ccdc115	chr1:34436669-34439672	OtA5088-OtA5092	OtA5093-OtA5097
Cep63	chr9:102586577-102626124	OtA5088-OtA5092	OtA5093-OtA5097
Asah1	chr8:41340642-41374697	OtA5088-OtA5092	OtA5093-OtA5097
Gbas	chr5:129725074-129758325	OtA5088-OtA5092	OtA5093-OtA5097
Hspa1b	chr17:34956428-34959238	OtA5088-OtA5092	OtA5093-OtA5097
Adpgk	chr9:59291571-59316200	OtA5088-OtA5092	OtA5093-OtA5097
Nmt1	chr11:103028561-103066105	OtA5088-OtA5092	OtA5093-OtA5097
Agpat1	chr17:34605860-34615971	OtA5088-OtA5092	OtA5093-OtA5097
Proc	chr18:32123125-32139570	OtA5088-OtA5092	OtA5093-OtA5097
Seh1l	chr18:67774875-67799943	OtA5088-OtA5092	OtA5093-OtA5097
Anapc10	chr8:79711819-79777321	OtA5088-OtA5092	OtA5093-OtA5097
Arl16	chr11:120458128-120467600	OtA5088-OtA5092	OtA5093-OtA5097
Bace1	chr9:45838528-45862484	OtA5088-OtA5092	OtA5093-OtA5097
Dcaf5	chr12:80335846-80436601	OtA5088-OtA5092	OtA5093-OtA5097
Dpy19l4	chr4:11265078-11322131	OtA5088-OtA5092	OtA5093-OtA5097
Fam185a	chr5:21424902-21482124	OtA5088-OtA5092	OtA5093-OtA5097
Ltbr	chr6:125306570-125313870	OtA5088-OtA5092	OtA5093-OtA5097
Meaf6	chr4:125085146-125109889	OtA5088-OtA5092	OtA5093-OtA5097
Med7	chr11:46436946-46442721	OtA5088-OtA5092	OtA5093-OtA5097
Pcsk7	chr9:45863690-45936058	OtA5088-OtA5092	OtA5093-OtA5097
Pigo	chr4:43017637-43025756	OtA5088-OtA5092	OtA5093-OtA5097
Ppargc1b	chr18:61298135-61400431	OtA5088-OtA5092	OtA5093-OtA5097
Prkg1	chr19:30564486-31765033	OtA5088-OtA5092	OtA5093-OtA5097
Rnf187	chr11:58932287-58938906	OtA5088-OtA5092	OtA5093-OtA5097
Srprb	chr9:103188032-103202065	OtA5088-OtA5092	OtA5093-OtA5097

Stradb	chr1:58973570-58995121	OtA5088-OtA5092	OtA5093-OtA5097
Taok2	chr7:126865676-126884967	OtA5088-OtA5092	OtA5093-OtA5097
Tapbpl	chr6:125224211-125239934	OtA5088-OtA5092	OtA5093-OtA5097
Tcirg1	chr19:3896049-3907133	OtA5088-OtA5092	OtA5093-OtA5097
Thoc6	chr17:23660522-23677449	OtA5088-OtA5092	OtA5093-OtA5097
Tor1a	chr2:30960560-30967918	OtA5088-OtA5092	OtA5093-OtA5097
Wdfy3	chr5:101832952-102069921	OtA5088-OtA5092	OtA5093-OtA5097
Zfand5	chr19:21272277-21286840	OtA5088-OtA5092	OtA5093-OtA5097
Zfp282	chr6:47877554-47908484	OtA5088-OtA5092	OtA5093-OtA5097
Map2k7	chr8:4238739-4247897	OtA5088-OtA5092	OtA5093-OtA5097
Ptplb	chr16:35022420-35109175	OtA5088-OtA5092	OtA5093-OtA5097
Trim15	chr17:36860690-36867210	OtA5088-OtA5092	OtA5093-OtA5097
Trim47	chr11:116105749-116110235	OtA5088-OtA5092	OtA5093-OtA5097
Csf2rb	chr15:78325989-78351001	OtA5088-OtA5092	OtA5093-OtA5097
Fam203a	chr15:76368897-76371411	OtA5088-OtA5092	OtA5093-OtA5097
Itgbl1	chr14:123660139-123974079	OtA5088-OtA5092	OtA5093-OtA5097
Itm2c	chr1:85894509-85908698	OtA5088-OtA5092	OtA5093-OtA5097
Sac3d1	chr19:6116003-6118586	OtA5088-OtA5092	OtA5093-OtA5097
Stk24	chr14:121286340-121379230	OtA5088-OtA5092	OtA5093-OtA5097
Tubb6	chr18:67390730-67402749	OtA5088-OtA5092	OtA5093-OtA5097
Yipf6	chrX:98937780-98949020	OtA5088-OtA5092	OtA5093-OtA5097
Zswim4	chr8:84210941-84237042	OtA5088-OtA5092	OtA5093-OtA5097
Mbip	chr12:56328306-56345894	OtA5088-OtA5092	OtA5093-OtA5097
Sh3gl1	chr17:56009200-56036637	OtA5088-OtA5092	OtA5093-OtA5097
Gm12942	chr4:127126045-127129644	OtA5088-OtA5092	OtA5093-OtA5097
Eif4a1	chr11:69666935-69672423	OtA5088-OtA5092	OtA5093-OtA5097
Cdca4	chr12:112820234-112829389	OtA5088-OtA5092	OtA5093-OtA5097
E4f1	chr17:24443778-24455311	OtA5088-OtA5092	OtA5093-OtA5097
Fam108c	chr7:84109355-84151893	OtA5088-OtA5092	OtA5093-OtA5097
Tuba1a	chr15:98949846-98953501	OtA5088-OtA5092	OtA5093-OtA5097
Utp20	chr10:88746606-88826814	OtA5088-OtA5092	OtA5093-OtA5097
Cgrrf1	chr14:46832243-46854190	OtA5088-OtA5092	OtA5093-OtA5097
Huwe1	chrX:151803281-151935417	OtA5088-OtA5092	OtA5093-OtA5097
Rfwd3	chr8:111270943-111300222	OtA5088-OtA5092	OtA5093-OtA5097
Anapc5	chr5:122787460-122821339	OtA5088-OtA5092	OtA5093-OtA5097
Stxbp6	chr12:44852485-45074483	OtA5088-OtA5092	OtA5093-OtA5097
Acsl1	chr8:46471041-46536050	OtA5088-OtA5092	OtA5093-OtA5097
Arhgap10	chr8:77250365-77518578	OtA5088-OtA5092	OtA5093-OtA5097
Arih1	chr9:59388553-59486374	OtA5088-OtA5092	OtA5093-OtA5097
Arpp19	chr9:75037613-75060313	OtA5088-OtA5092	OtA5093-OtA5097
Banf1	chr19:5364639-5366645	OtA5088-OtA5092	OtA5093-OtA5097
Brd8	chr18:34598614-34651736	OtA5088-OtA5092	OtA5093-OtA5097
Camk2n1	chr4:138455147-138460126	OtA5088-OtA5092	OtA5093-OtA5097
Ccl2	chr11:82035576-82037452	OtA5088-OtA5092	OtA5093-OtA5097
Cops7b	chr1:86587099-86606500	OtA5088-OtA5092	OtA5093-OtA5097
Cox18	chr5:90214724-90223996	OtA5088-OtA5092	OtA5093-OtA5097
D8Ertd738e	chr8:84246234-84249761	OtA5088-OtA5092	OtA5093-OtA5097

Dkkl1	chr7:45207524-45211883	OtA5088-OtA5092	OtA5093-OtA5097
F13a1	chr13:36867177-37050244	OtA5088-OtA5092	OtA5093-OtA5097
F8a	chrX:73228305-73230795	OtA5088-OtA5092	OtA5093-OtA5097
Fbxw2	chr2:34804363-34826235	OtA5088-OtA5092	OtA5093-OtA5097
Fmo5	chr3:97628803-97655287	OtA5088-OtA5092	OtA5093-OtA5097
Gdi2	chr13:3538074-3611108	OtA5088-OtA5092	OtA5093-OtA5097
H13	chr2:152669460-152708668	OtA5088-OtA5092	OtA5093-OtA5097
Ift46	chr9:44773008-44792714	OtA5088-OtA5092	OtA5093-OtA5097
Irf3	chr7:44997647-45002848	OtA5088-OtA5092	OtA5093-OtA5097
Itgb7	chr15:102215994-102231935	OtA5088-OtA5092	OtA5093-OtA5097
Mcoln2	chr3:146149832-146195512	OtA5088-OtA5092	OtA5093-OtA5097
Mctp2	chr7:72077829-72306595	OtA5088-OtA5092	OtA5093-OtA5097
Mtx1	chr3:89209080-89214335	OtA5088-OtA5092	OtA5093-OtA5097
Myadm	chr7:3289037-3299349	OtA5088-OtA5092	OtA5093-OtA5097
Nup214	chr2:31974449-32053975	OtA5088-OtA5092	OtA5093-OtA5097
Pdia6	chr12:17266594-17324730	OtA5088-OtA5092	OtA5093-OtA5097
Pyroxd2	chr19:42725857-42752775	OtA5088-OtA5092	OtA5093-OtA5097
Rab13	chr3:90220816-90225963	OtA5088-OtA5092	OtA5093-OtA5097
Rheb	chr5:24802822-24842361	OtA5088-OtA5092	OtA5093-OtA5097
Rpl41	chr10:128548109-128549168	OtA5088-OtA5092	OtA5093-OtA5097
Rpusd2	chr2:119034789-119042197	OtA5088-OtA5092	OtA5093-OtA5097
Sco2	chr15:89355721-89377037	OtA5088-OtA5092	OtA5093-OtA5097
Slc7a1	chr5:148327409-148399904	OtA5088-OtA5092	OtA5093-OtA5097
Stx5a	chr19:8741423-8755642	OtA5088-OtA5092	OtA5093-OtA5097
Tes	chr6:17065148-17105825	OtA5088-OtA5092	OtA5093-OtA5097
Thra	chr11:98741872-98765111	OtA5088-OtA5092	OtA5093-OtA5097
Tmco3	chr8:13255962-13322924	OtA5088-OtA5092	OtA5093-OtA5097
Tmem33	chr5:67260651-67291461	OtA5088-OtA5092	OtA5093-OtA5097
Tnfsf12	chr11:69682576-69696098	OtA5088-OtA5092	OtA5093-OtA5097
Uhrf2	chr19:30030512-30093724	OtA5088-OtA5092	OtA5093-OtA5097
Vps26b	chr9:26999676-27030094	OtA5088-OtA5092	OtA5093-OtA5097
Wdsub1	chr2:59852364-59882606	OtA5088-OtA5092	OtA5093-OtA5097
Zbtb8os	chr4:129336025-129347029	OtA5088-OtA5092	OtA5093-OtA5097
Zw10	chr9:49055580-49078773	OtA5088-OtA5092	OtA5093-OtA5097
Tbc1d9b	chr11:50131359-50172785	OtA5088-OtA5092	OtA5093-OtA5097
Cdc37l1	chr19:28990493-29017569	OtA5088-OtA5092	OtA5093-OtA5097
Chka	chr19:3851772-3894367	OtA5088-OtA5092	OtA5093-OtA5097
Gmfg	chr7:28437446-28446895	OtA5088-OtA5092	OtA5093-OtA5097
Rccd1	chr7:80316615-80324454	OtA5088-OtA5092	OtA5093-OtA5097
Ace	chr11:105967944-105989964	OtA5088-OtA5092	OtA5093-OtA5097
Dtnb	chr12:3572390-3781398	OtA5088-OtA5092	OtA5093-OtA5097
Mettl9	chr7:121034444-121076835	OtA5088-OtA5092	OtA5093-OtA5097
Myh11	chr16:14194526-14291408	OtA5088-OtA5092	OtA5093-OtA5097
Pbx3	chr2:34171908-34372036	OtA5088-OtA5092	OtA5093-OtA5097
Xpc	chr6:91489306-91515888	OtA5088-OtA5092	OtA5093-OtA5097
Ap3s1	chr18:46741916-46790826	OtA5088-OtA5092	OtA5093-OtA5097
Dok4	chr8:94857449-94876312	OtA5088-OtA5092	OtA5093-OtA5097

Fat1	chr8:44950207-45052257	OtA5088-OtA5092	OtA5093-OtA5097
Kctd1	chr18:14968684-15151446	OtA5088-OtA5092	OtA5093-OtA5097
Kif3b	chr2:153291415-153333389	OtA5088-OtA5092	OtA5093-OtA5097
Lrp5	chr19:3584824-3686564	OtA5088-OtA5092	OtA5093-OtA5097
Nono	chrX:101429650-101448593	OtA5088-OtA5092	OtA5093-OtA5097
Pddc1	chr7:141408183-141414125	OtA5088-OtA5092	OtA5093-OtA5097
Polb	chr8:22628118-22653437	OtA5088-OtA5092	OtA5093-OtA5097
Sirt5	chr13:43370715-43395203	OtA5088-OtA5092	OtA5093-OtA5097
Insig1	chr5:28071411-28078662	OtA5088-OtA5092	OtA5093-OtA5097
Epm2a	chr10:11343444-11457477	OtA5088-OtA5092	OtA5093-OtA5097
Glrx5	chr12:105032688-105040910	OtA5088-OtA5092	OtA5093-OtA5097
Gzf1	chr2:148681119-148692949	OtA5088-OtA5092	OtA5093-OtA5097
Mbtps1	chr8:119508151-119558761	OtA5088-OtA5092	OtA5093-OtA5097
Psmd5	chr2:34852088-34870962	OtA5088-OtA5092	OtA5093-OtA5097
Rbp4	chr19:38116619-38125321	OtA5088-OtA5092	OtA5093-OtA5097
Tmem9b	chr7:109735835-109752263	OtA5088-OtA5092	OtA5093-OtA5097
Txnrd1	chr10:82833950-82897724	OtA5088-OtA5092	OtA5093-OtA5097
Ywhah	chr5:33018815-33027966	OtA5088-OtA5092	OtA5093-OtA5097
BC003331	chr1:150361310-150466165	OtA5088-OtA5092	OtA5093-OtA5097
Celf2	chr2:6539698-7396198	OtA5088-OtA5092	OtA5093-OtA5097
Ddx28	chr8:106009615-106011486	OtA5088-OtA5092	OtA5093-OtA5097
Ldha	chr7:46845803-46855627	OtA5088-OtA5092	OtA5093-OtA5097
Slc25a14	chrX:48623579-48662294	OtA5088-OtA5092	OtA5093-OtA5097
Tbc1d30	chr10:121263819-121311189	OtA5088-OtA5092	OtA5093-OtA5097
Wtip	chr7:34109549-34133268	OtA5088-OtA5092	OtA5093-OtA5097
Actb	chr5:142903115-142906724	OtA5088-OtA5092	OtA5093-OtA5097
Afg3l1	chr8:123477861-123503916	OtA5088-OtA5092	OtA5093-OtA5097
Brp44	chr1:165461207-165481214	OtA5088-OtA5092	OtA5093-OtA5097
D18Ertd653e	chr18:67933256-68255549	OtA5088-OtA5092	OtA5093-OtA5097
Eif3j,Gm9781	chr2:122028582-122118386	OtA5088-OtA5092	OtA5093-OtA5097
Gipc1	chr8:83652677-83664789	OtA5088-OtA5092	OtA5093-OtA5097
Gm10094,Sap18	chr14:57798188-57804980	OtA5088-OtA5092	OtA5093-OtA5097
Gnl1	chr17:35979954-35989462	OtA5088-OtA5092	OtA5093-OtA5097
Gstcd	chr3:132982550-133091740	OtA5088-OtA5092	OtA5093-OtA5097
Hdhd3	chr4:62499053-62502200	OtA5088-OtA5092	OtA5093-OtA5097
Katnb1	chr8:95081200-95142540	OtA5088-OtA5092	OtA5093-OtA5097
Mettl11a	chr2:30807976-30823014	OtA5088-OtA5092	OtA5093-OtA5097
Mrps24	chr11:5703982-5707699	OtA5088-OtA5092	OtA5093-OtA5097
Ppcs	chr4:119418532-119422420	OtA5088-OtA5092	OtA5093-OtA5097
Qars	chr9:108508004-108515941	OtA5088-OtA5092	OtA5093-OtA5097
Rab11fip2	chr19:59902883-59943654	OtA5088-OtA5092	OtA5093-OtA5097
Rnmt	chr18:68300354-68324852	OtA5088-OtA5092	OtA5093-OtA5097
Sec11a	chr7:80915378-80947550	OtA5088-OtA5092	OtA5093-OtA5097
Tmem107	chr11:69070808-69073293	OtA5088-OtA5092	OtA5093-OtA5097
Trim25	chr11:88999402-89020293	OtA5088-OtA5092	OtA5093-OtA5097
Tssc4	chr7:143069367-143071087	OtA5088-OtA5092	OtA5093-OtA5097
Ubtf	chr11:102304559-102319096	OtA5088-OtA5092	OtA5093-OtA5097

Vps13b	chr15:35371545-35931229	OtA5088-OtA5092	OtA5093-OtA5097
Zbtb24	chr10:41450395-41465574	OtA5088-OtA5092	OtA5093-OtA5097
Zwint	chr10:72654894-72669789	OtA5088-OtA5092	OtA5093-OtA5097
Agxt2l2	chr11:51584756-51606881	OtA5088-OtA5092	OtA5093-OtA5097
Usp24	chr4:106316212-106441327	OtA5088-OtA5092	OtA5093-OtA5097
Zfp688	chr7:127418965-127422034	OtA5088-OtA5092	OtA5093-OtA5097
Scrn3	chr2:73312651-73337807	OtA5088-OtA5092	OtA5093-OtA5097
Rab18	chr18:6765204-6790231	OtA5088-OtA5092	OtA5093-OtA5097
Cblc	chr7:19779717-19796809	OtA5088-OtA5092	OtA5093-OtA5097
Cd24a	chr10:43579168-43584265	OtA5088-OtA5092	OtA5093-OtA5097
Atp2b4	chr1:133702673-133753747	OtA5088-OtA5092	OtA5093-OtA5097
Lrrc8c	chr5:105519470-105608954	OtA5088-OtA5092	OtA5093-OtA5097
Thbd	chr2:148404470-148408188	OtA5088-OtA5092	OtA5093-OtA5097
C030006K11Rik	chr15:76715275-76723845	OtA5088-OtA5092	OtA5093-OtA5097
Myg1	chr15:102331708-102338138	OtA5088-OtA5092	OtA5093-OtA5097
Jazf1	chr6:52768067-53068624	OtA5088-OtA5092	OtA5093-OtA5097
Nfkbib	chr7:28756173-28766524	OtA5088-OtA5092	OtA5093-OtA5097
Pigq	chr17:25926423-25941962	OtA5088-OtA5092	OtA5093-OtA5097
Vapa	chr17:65580052-65613555	OtA5088-OtA5092	OtA5093-OtA5097
Bik	chr15:83526861-83544635	OtA5088-OtA5092	OtA5093-OtA5097
Marveld2	chr13:100595956-100616971	OtA5088-OtA5092	OtA5093-OtA5097
Mrpl27	chr11:94653790-94660087	OtA5088-OtA5092	OtA5093-OtA5097
Peli1	chr11:21091323-21150327	OtA5088-OtA5092	OtA5093-OtA5097
Rpl9	chr5:65388363-65391431	OtA5088-OtA5092	OtA5093-OtA5097
Ddx52	chr11:83942089-83963086	OtA5088-OtA5092	OtA5093-OtA5097
Ndufa12	chr10:94199008-94220948	OtA5088-OtA5092	OtA5093-OtA5097
Ang	chr14:51091076-51106151	OtA5088-OtA5092	OtA5093-OtA5097
Ap3m1	chr14:21033741-21052442	OtA5088-OtA5092	OtA5093-OtA5097
Birc5	chr11:117849236-117855743	OtA5088-OtA5092	OtA5093-OtA5097
Cbr1	chr16:93607836-93610349	OtA5088-OtA5092	OtA5093-OtA5097
Chn2	chr6:54039931-54430221	OtA5088-OtA5092	OtA5093-OtA5097
Cycs	chr6:50562562-50566474	OtA5088-OtA5092	OtA5093-OtA5097
Dtx3	chr10:127190377-127211622	OtA5088-OtA5092	OtA5093-OtA5097
Epb4.1l2	chr10:25359797-25523518	OtA5088-OtA5092	OtA5093-OtA5097
Esrp2	chr8:106131182-106138722	OtA5088-OtA5092	OtA5093-OtA5097
Fau	chr19:6057887-6059524	OtA5088-OtA5092	OtA5093-OtA5097
Hoxb6	chr11:96299170-96301569	OtA5088-OtA5092	OtA5093-OtA5097
Kif1b	chr4:149176320-149307698	OtA5088-OtA5092	OtA5093-OtA5097
Kifap3	chr1:163779678-163917100	OtA5088-OtA5092	OtA5093-OtA5097
Mcc	chr18:44425059-44812182	OtA5088-OtA5092	OtA5093-OtA5097
Metrn	chr17:25794570-25797045	OtA5088-OtA5092	OtA5093-OtA5097
Ndufc2	chr7:97400002-97407800	OtA5088-OtA5092	OtA5093-OtA5097
Nog	chr11:89300637-89302559	OtA5088-OtA5092	OtA5093-OtA5097
P2ry6	chr7:100937633-100964366	OtA5088-OtA5092	OtA5093-OtA5097
Ppp2r5b	chr19:6226400-6235840	OtA5088-OtA5092	OtA5093-OtA5097
Rbm8a	chr3:96629927-96633791	OtA5088-OtA5092	OtA5093-OtA5097
Rpain	chr11:70970199-70983026	OtA5088-OtA5092	OtA5093-OtA5097

Rrp36	chr17:46667452-46674255	OtA5088-OtA5092	OtA5093-OtA5097
Rxra	chr2:27677200-27763319	OtA5088-OtA5092	OtA5093-OtA5097
Scd2	chr19:44293675-44306862	OtA5088-OtA5092	OtA5093-OtA5097
Serpine2	chr1:79794320-79858665	OtA5088-OtA5092	OtA5093-OtA5097
Sesn3	chr9:14276300-14326134	OtA5088-OtA5092	OtA5093-OtA5097
Tfb2m	chr1:179528055-179546267	OtA5088-OtA5092	OtA5093-OtA5097
Tm2d3	chr7:65693416-65701913	OtA5088-OtA5092	OtA5093-OtA5097
Zfp161	chr17:69383977-69390544	OtA5088-OtA5092	OtA5093-OtA5097
Slc25a5	chrX:36795651-36798806	OtA5088-OtA5092	OtA5093-OtA5097
Tert	chr13:73627000-73649041	OtA5088-OtA5092	OtA5093-OtA5097
Acadm	chr3:153922358-153944432	OtA5088-OtA5092	OtA5093-OtA5097
Cib2	chr9:54545351-54560079	OtA5088-OtA5092	OtA5093-OtA5097
D430042O09Rik	chr7:125707875-125874797	OtA5088-OtA5092	OtA5093-OtA5097
Ube2s	chr7:4808013-4812340	OtA5088-OtA5092	OtA5093-OtA5097
Poc1b	chr10:99107170-99197988	OtA5088-OtA5092	OtA5093-OtA5097
Map2k4	chr11:65688243-65788297	OtA5088-OtA5092	OtA5093-OtA5097
D730040F13Rik	chr4:56876012-56947429	OtA5088-OtA5092	OtA5093-OtA5097
Dnaja3	chr16:4684069-4707693	OtA5088-OtA5092	OtA5093-OtA5097
Ep400	chr5:110664372-110770717	OtA5088-OtA5092	OtA5093-OtA5097
Hnrnph3	chr10:62980222-63023849	OtA5088-OtA5092	OtA5093-OtA5097
Prss16	chr13:22002175-22009741	OtA5088-OtA5092	OtA5093-OtA5097
Rpp21	chr17:36255672-36257846	OtA5088-OtA5092	OtA5093-OtA5097
Cd47	chr16:49855653-49911683	OtA5088-OtA5092	OtA5093-OtA5097
Zbtb20	chr16:43247283-43619123	OtA5088-OtA5092	OtA5093-OtA5097
Med6	chr12:81573563-81594958	OtA5088-OtA5092	OtA5093-OtA5097
Abt1	chr13:23418360-23423866	OtA5088-OtA5092	OtA5093-OtA5097
Arhgap32	chr9:32116135-32265511	OtA5088-OtA5092	OtA5093-OtA5097
Csf2ra	chr19:61224401-61228418	OtA5088-OtA5092	OtA5093-OtA5097
Ctdsp1	chr1:74391608-74397285	OtA5088-OtA5092	OtA5093-OtA5097
Dhdds	chr4:133969056-134000864	OtA5088-OtA5092	OtA5093-OtA5097
Dnaja2	chr8:85537639-85555271	OtA5088-OtA5092	OtA5093-OtA5097
Dusp13	chr14:21733394-21748622	OtA5088-OtA5092	OtA5093-OtA5097
Fbxo45	chr16:32230111-32247025	OtA5088-OtA5092	OtA5093-OtA5097
Gcsh	chr8:116981827-116993449	OtA5088-OtA5092	OtA5093-OtA5097
Grpel2	chr18:61712423-61726331	OtA5088-OtA5092	OtA5093-OtA5097
Lenep	chr3:89401895-89402650	OtA5088-OtA5092	OtA5093-OtA5097
Lrrc8b	chr5:105415774-105486189	OtA5088-OtA5092	OtA5093-OtA5097
Mtx2	chr2:74825811-74876748	OtA5088-OtA5092	OtA5093-OtA5097
Parp6	chr9:59617283-59650290	OtA5088-OtA5092	OtA5093-OtA5097
Phldb1	chr9:44686307-44735198	OtA5088-OtA5092	OtA5093-OtA5097
Pitpnm1	chr19:4100116-4113965	OtA5088-OtA5092	OtA5093-OtA5097
Pogk	chr1:166379166-166409828	OtA5088-OtA5092	OtA5093-OtA5097
Prps1	chrX:140456602-140476140	OtA5088-OtA5092	OtA5093-OtA5097
Prr15	chr6:54039931-54430221	OtA5088-OtA5092	OtA5093-OtA5097
Rg9mtd1	chr16:56033719-56037774	OtA5088-OtA5092	OtA5093-OtA5097
Sema6c	chr3:95164373-95173316	OtA5088-OtA5092	OtA5093-OtA5097
Sephs1	chr2:4881563-4910556	OtA5088-OtA5092	OtA5093-OtA5097

Sirt6	chr10:81621785-81627608	OtA5088-OtA5092	OtA5093-OtA5097
Tesk1	chr4:43442276-43454626	OtA5088-OtA5092	OtA5093-OtA5097
Tsc22d4	chr5:137745968-137768453	OtA5088-OtA5092	OtA5093-OtA5097
Ube2z	chr11:96047430-96065364	OtA5088-OtA5092	OtA5093-OtA5097
Vkorc1l1	chr5:129942112-129982890	OtA5088-OtA5092	OtA5093-OtA5097
Vps36	chr8:22192859-22218597	OtA5088-OtA5092	OtA5093-OtA5097
Zfp280b	chr10:76032611-76042969	OtA5088-OtA5092	OtA5093-OtA5097
Fam54b	chr4:134525554-134535387	OtA5088-OtA5092	OtA5093-OtA5097
Glt8d1	chr14:30999825-31019089	OtA5088-OtA5092	OtA5093-OtA5097
Inpp4a	chr1:37299892-37410735	OtA5088-OtA5092	OtA5093-OtA5097
Necap1	chr6:122874556-122888941	OtA5088-OtA5092	OtA5093-OtA5097
Ier5l	chr2:30472640-30474199	OtA5088-OtA5092	OtA5093-OtA5097
Zfp263	chr16:3744098-3750788	OtA5088-OtA5092	OtA5093-OtA5097
Zfp259	chr9:46273063-46282642	OtA5088-OtA5092	OtA5093-OtA5097
Nacc1	chr8:84670478-84687862	OtA5088-OtA5092	OtA5093-OtA5097
Adcy4	chr14:55769091-55784019	OtA5088-OtA5092	OtA5093-OtA5097
Kremen1	chr11:5191552-5261610	OtA5088-OtA5092	OtA5093-OtA5097
Pvrl3	chr16:46394857-46496967	OtA5088-OtA5092	OtA5093-OtA5097
Rhebl1	chr15:98877759-98881414	OtA5088-OtA5092	OtA5093-OtA5097
Agtpbp1	chr13:59449534-59557319	OtA5088-OtA5092	OtA5093-OtA5097
Cct2	chr10:117050997-117063814	OtA5088-OtA5092	OtA5093-OtA5097
Cdkn2aipnl	chr11:51967630-51977336	OtA5088-OtA5092	OtA5093-OtA5097
Fdxr	chr11:115268024-115276969	OtA5088-OtA5092	OtA5093-OtA5097
Naif1	chr2:32414486-32455476	OtA5088-OtA5092	OtA5093-OtA5097
Pnpla8	chr12:44269153-44313435	OtA5088-OtA5092	OtA5093-OtA5097
Rab7	chr6:88000434-88013690	OtA5088-OtA5092	OtA5093-OtA5097
Tatdn2	chr6:113697498-113711068	OtA5088-OtA5092	OtA5093-OtA5097
Tmprss4	chr9:45172725-45249985	OtA5088-OtA5092	OtA5093-OtA5097
Als2cl	chr9:110880173-110900530	OtA5088-OtA5092	OtA5093-OtA5097
Cox4i2	chr2:152754172-152765037	OtA5088-OtA5092	OtA5093-OtA5097
Dbt	chr3:116513078-116549981	OtA5088-OtA5092	OtA5093-OtA5097
Gtpbp8	chr16:44738878-44746363	OtA5088-OtA5092	OtA5093-OtA5097
Pycrl	chr15:75916462-75921560	OtA5088-OtA5092	OtA5093-OtA5097
Rqcd1	chr1:74409383-74588028	OtA5088-OtA5092	OtA5093-OtA5097
S100a4	chr3:90603769-90606045	OtA5088-OtA5092	OtA5093-OtA5097
Sec22a	chr16:35311130-35363918	OtA5088-OtA5092	OtA5093-OtA5097
Acrbp	chr6:125049926-125063265	OtA5088-OtA5092	OtA5093-OtA5097
Alpl	chr4:137741731-137796377	OtA5088-OtA5092	OtA5093-OtA5097
Arhgef17	chr7:100869745-100932161	OtA5088-OtA5092	OtA5093-OtA5097
Dnajc7	chr11:100582835-100620168	OtA5088-OtA5092	OtA5093-OtA5097
Fam45a	chr19:60811569-60836229	OtA5088-OtA5092	OtA5093-OtA5097
Hbxip	chr3:107278857-107284081	OtA5088-OtA5092	OtA5093-OtA5097
Nfe2l2	chr2:75675518-75704641	OtA5088-OtA5092	OtA5093-OtA5097
Rad9	chr19:4192173-4201603	OtA5088-OtA5092	OtA5093-OtA5097
Rnaseh2b	chr14:62332104-62372992	OtA5088-OtA5092	OtA5093-OtA5097
Slc10a6	chr5:103605710-103629403	OtA5088-OtA5092	OtA5093-OtA5097
Unc13b	chr4:43058983-43264887	OtA5088-OtA5092	OtA5093-OtA5097

Vdac1	chr11:52361114-52389396	OtA5088-OtA5092	OtA5093-OtA5097
Amotl2	chr9:102717803-102733417	OtA5088-OtA5092	OtA5093-OtA5097
Syvn1	chr19:6046575-6057751	OtA5088-OtA5092	OtA5093-OtA5097
Mcmbp	chr7:128696458-128740429	OtA5088-OtA5092	OtA5093-OtA5097
Als2cr4	chr1:59100592-59120096	OtA5088-OtA5092	OtA5093-OtA5097
Acvr1b	chr15:101174124-101212601	OtA5088-OtA5092	OtA5093-OtA5097
Afg3l2	chr18:67404763-67449136	OtA5088-OtA5092	OtA5093-OtA5097
Cldn6	chr17:23679364-23682446	OtA5088-OtA5092	OtA5093-OtA5097
Commd8	chr5:72156999-72168183	OtA5088-OtA5092	OtA5093-OtA5097
Gatc	chr5:115333241-115341161	OtA5088-OtA5092	OtA5093-OtA5097
Gna12	chr5:140759943-140830431	OtA5088-OtA5092	OtA5093-OtA5097
Gpsm3	chr17:34589805-34591754	OtA5088-OtA5092	OtA5093-OtA5097
Haus1	chr18:77757566-77767780	OtA5088-OtA5092	OtA5093-OtA5097
Hbp1	chr12:31654868-31950535	OtA5088-OtA5092	OtA5093-OtA5097
Ints8	chr4:11191353-11254259	OtA5088-OtA5092	OtA5093-OtA5097
Lamp1	chr8:13159134-13175338	OtA5088-OtA5092	OtA5093-OtA5097
Lysmd3	chr13:81657805-81671890	OtA5088-OtA5092	OtA5093-OtA5097
Mios	chr6:8209226-8236273	OtA5088-OtA5092	OtA5093-OtA5097
Mrrf	chr2:36116078-36190283	OtA5088-OtA5092	OtA5093-OtA5097
Nmnat3	chr9:98296582-98411428	OtA5088-OtA5092	OtA5093-OtA5097
Pank4	chr4:154964132-154980938	OtA5088-OtA5092	OtA5093-OtA5097
Pcx	chr19:4510471-4621752	OtA5088-OtA5092	OtA5093-OtA5097
Pdxp	chr15:78913918-78919517	OtA5088-OtA5092	OtA5093-OtA5097
Plin4	chr17:56079656-56109802	OtA5088-OtA5092	OtA5093-OtA5097
Prkaa2	chr4:105029649-105109898	OtA5088-OtA5092	OtA5093-OtA5097
Prl7d1	chr13:27708997-27716737	OtA5088-OtA5092	OtA5093-OtA5097
Puf60	chr15:76070181-76080946	OtA5088-OtA5092	OtA5093-OtA5097
Rnf13	chr3:57736065-57835233	OtA5088-OtA5092	OtA5093-OtA5097
Rnf5	chr17:34601098-34603561	OtA5088-OtA5092	OtA5093-OtA5097
Ror2	chr13:53109316-53286109	OtA5088-OtA5092	OtA5093-OtA5097
S100pbp	chr4:129150824-129189482	OtA5088-OtA5092	OtA5093-OtA5097
Sfrp1	chr8:23411501-23449632	OtA5088-OtA5092	OtA5093-OtA5097
Snrnp40	chr4:130360131-130390030	OtA5088-OtA5092	OtA5093-OtA5097
St3gal3	chr4:117932152-118134914	OtA5088-OtA5092	OtA5093-OtA5097
Stam2	chr2:52692205-52742149	OtA5088-OtA5092	OtA5093-OtA5097
Stk4	chr2:164074177-164155521	OtA5088-OtA5092	OtA5093-OtA5097
Trafd1	chr5:121371724-121385615	OtA5088-OtA5092	OtA5093-OtA5097
Trappc3	chr4:126262404-126275883	OtA5088-OtA5092	OtA5093-OtA5097
U2af2	chr7:5062142-5079945	OtA5088-OtA5092	OtA5093-OtA5097
Ubfd1	chr7:122067197-122082199	OtA5088-OtA5092	OtA5093-OtA5097
Zfp212	chr6:47920567-47932637	OtA5088-OtA5092	OtA5093-OtA5097
Zfp692	chr11:58307068-58314613	OtA5088-OtA5092	OtA5093-OtA5097
Zfp865	chr7:5020375-5033223	OtA5088-OtA5092	OtA5093-OtA5097
Zfp949	chr9:88548019-88571086	OtA5088-OtA5092	OtA5093-OtA5097
Xrcc6bp1	chr10:126868427-126901371	OtA5088-OtA5092	OtA5093-OtA5097
Zkscan5	chr5:145204558-145221750	OtA5088-OtA5092	OtA5093-OtA5097
Kpna6	chr4:129643978-129672767	OtA5088-OtA5092	OtA5093-OtA5097

Srd5a3	chr5:76140272-76155503	OtA5088-OtA5092	OtA5093-OtA5097
Accs	chr2:93835452-93849776	OtA5088-OtA5092	OtA5093-OtA5097
Aifm1	chrX:48474943-48513426	OtA5088-OtA5092	OtA5093-OtA5097
Akap8	chr17:32303675-32321153	OtA5088-OtA5092	OtA5093-OtA5097
Ankra2	chr13:98263241-98273913	OtA5088-OtA5092	OtA5093-OtA5097
Apbb3	chr18:36671158-36683862	OtA5088-OtA5092	OtA5093-OtA5097
Arf4	chr14:26638196-26657258	OtA5088-OtA5092	OtA5093-OtA5097
Arfgap1	chr2:180967224-180982524	OtA5088-OtA5092	OtA5093-OtA5097
Arl6	chr16:59613320-60605531	OtA5088-OtA5092	OtA5093-OtA5097
As3mt	chr19:46707442-46741095	OtA5088-OtA5092	OtA5093-OtA5097
BC017643	chr11:121177592-121229309	OtA5088-OtA5092	OtA5093-OtA5097
BC067068	chr10:105763184-105841380	OtA5088-OtA5092	OtA5093-OtA5097
Blvra	chr2:127070656-127097084	OtA5088-OtA5092	OtA5093-OtA5097
Bmp1	chr14:70474554-70520260	OtA5088-OtA5092	OtA5093-OtA5097
Bola2	chr7:126695999-126696693	OtA5088-OtA5092	OtA5093-OtA5097
C330013E15Rik	chr15:100614139-100615110	OtA5088-OtA5092	OtA5093-OtA5097
Cby1	chr15:79659226-79667660	OtA5088-OtA5092	OtA5093-OtA5097
Ccdc28b	chr4:129619273-129623908	OtA5088-OtA5092	OtA5093-OtA5097
Ccdc69	chr11:55049737-55078131	OtA5088-OtA5092	OtA5093-OtA5097
Cd93	chr2:148436650-148443535	OtA5088-OtA5092	OtA5093-OtA5097
Cfi	chr3:129836738-129875328	OtA5088-OtA5092	OtA5093-OtA5097
Chd3	chr11:69344350-69369391	OtA5088-OtA5092	OtA5093-OtA5097
Cideb	chr14:55745692-55758424	OtA5088-OtA5092	OtA5093-OtA5097
Cisd2	chr3:135406411-135423433	OtA5088-OtA5092	OtA5093-OtA5097
Ckmt1	chr2:121358640-121380940	OtA5088-OtA5092	OtA5093-OtA5097
Cnksr1	chr4:134228041-134238399	OtA5088-OtA5092	OtA5093-OtA5097
Commd1	chr11:22899727-22982284	OtA5088-OtA5092	OtA5093-OtA5097
Ctnnd1	chr2:84600780-84650740	OtA5088-OtA5092	OtA5093-OtA5097
Cyb561d1	chr3:108195770-108200834	OtA5088-OtA5092	OtA5093-OtA5097
D2hgdh	chr1:93825239-93852174	OtA5088-OtA5092	OtA5093-OtA5097
Dhx38	chr8:109548023-109565601	OtA5088-OtA5092	OtA5093-OtA5097
Dlst	chr12:85110832-85134091	OtA5088-OtA5092	OtA5093-OtA5097
Dpm2	chr2:32570857-32573571	OtA5088-OtA5092	OtA5093-OtA5097
Dpt	chr1:164796731-164824266	OtA5088-OtA5092	OtA5093-OtA5097
Dpy19l1	chr9:24411778-24503140	OtA5088-OtA5092	OtA5093-OtA5097
E030030I06Rik	chr10:22113049-22149270	OtA5088-OtA5092	OtA5093-OtA5097
E430018J23Rik	chr7:127390835-127393621	OtA5088-OtA5092	OtA5093-OtA5097
Elf4	chrX:48411048-48463132	OtA5088-OtA5092	OtA5093-OtA5097
Eps8	chr6:137477244-137649120	OtA5088-OtA5092	OtA5093-OtA5097
Espn	chr4:152120875-152152207	OtA5088-OtA5092	OtA5093-OtA5097
Etnk1	chr6:143167229-143208547	OtA5088-OtA5092	OtA5093-OtA5097
Exoc7	chr11:116287997-116306738	OtA5088-OtA5092	OtA5093-OtA5097
Fam120b	chr17:15396201-15433581	OtA5088-OtA5092	OtA5093-OtA5097
Fam167b	chr4:129576814-129578580	OtA5088-OtA5092	OtA5093-OtA5097
Fam20c	chr5:138755080-138810063	OtA5088-OtA5092	OtA5093-OtA5097
Fam38b	chr18:63010212-63387183	OtA5088-OtA5092	OtA5093-OtA5097
Fcgr2b	chr1:170960558-170976071	OtA5088-OtA5092	OtA5093-OtA5097

Foxo1	chr3:52268336-52350109	OtA5088-OtA5092	OtA5093-OtA5097
G0s2	chr1:193272159-193273188	OtA5088-OtA5092	OtA5093-OtA5097
Ggps1	chr13:14052444-14063401	OtA5088-OtA5092	OtA5093-OtA5097
Gm20604	chr12:102739829-102757810	OtA5088-OtA5092	OtA5093-OtA5097
Gm20605,Lrch4	chr5:137629122-137642902	OtA5088-OtA5092	OtA5093-OtA5097
Gmppa	chr1:75435942-75443176	OtA5088-OtA5092	OtA5093-OtA5097
Gnas	chr2:174281236-174346742	OtA5088-OtA5092	OtA5093-OtA5097
Gns	chr10:121365089-121397245	OtA5088-OtA5092	OtA5093-OtA5097
Gpr124	chr8:27085840-27123436	OtA5088-OtA5092	OtA5093-OtA5097
Gpr4	chr7:19212537-19224176	OtA5088-OtA5092	OtA5093-OtA5097
H2-Q4	chr17:35379616-35384674	OtA5088-OtA5092	OtA5093-OtA5097
H3f3b	chr11:116021960-116024504	OtA5088-OtA5092	OtA5093-OtA5097
H6pd	chr4:149979480-150009023	OtA5088-OtA5092	OtA5093-OtA5097
Harbi1	chr2:91710949-91721566	OtA5088-OtA5092	OtA5093-OtA5097
Hp1bp3	chr4:138216626-138243170	OtA5088-OtA5092	OtA5093-OtA5097
Hspa4l	chr3:40745612-40790365	OtA5088-OtA5092	OtA5093-OtA5097
Insr	chr8:3150921-3279617	OtA5088-OtA5092	OtA5093-OtA5097
Irs3	chr5:137643031-137645714	OtA5088-OtA5092	OtA5093-OtA5097
Jph2	chr2:163336241-163397993	OtA5088-OtA5092	OtA5093-OtA5097
Kat2b	chr17:53566970-53672721	OtA5088-OtA5092	OtA5093-OtA5097
Katna1	chr10:7725999-7763150	OtA5088-OtA5092	OtA5093-OtA5097
Kctd5	chr17:24047719-24073485	OtA5088-OtA5092	OtA5093-OtA5097
Kri1	chr9:21265298-21287969	OtA5088-OtA5092	OtA5093-OtA5097
Krt23	chr11:99477972-99493110	OtA5088-OtA5092	OtA5093-OtA5097
Lime1	chr2:181381234-181383628	OtA5088-OtA5092	OtA5093-OtA5097
Lin9	chr1:180641333-180690687	OtA5088-OtA5092	OtA5093-OtA5097
Lrfn4	chr19:4510471-4621752	OtA5088-OtA5092	OtA5093-OtA5097
Lrrn2	chr1:132880354-132940005	OtA5088-OtA5092	OtA5093-OtA5097
Ltn1	chr16:87376650-87432606	OtA5088-OtA5092	OtA5093-OtA5097
Mdm1	chr10:118141786-118168999	OtA5088-OtA5092	OtA5093-OtA5097
Mdp1	chr14:55657878-55660508	OtA5088-OtA5092	OtA5093-OtA5097
Med30	chr15:52712444-52730431	OtA5088-OtA5092	OtA5093-OtA5097
Megf11	chr9:64385625-64709205	OtA5088-OtA5092	OtA5093-OtA5097
Mkl1	chr15:80977764-81190757	OtA5088-OtA5092	OtA5093-OtA5097
Mon1a	chr9:107888128-107903139	OtA5088-OtA5092	OtA5093-OtA5097
Mrpl22	chr11:58171653-58179581	OtA5088-OtA5092	OtA5093-OtA5097
Mrpl50	chr4:49512596-49521083	OtA5088-OtA5092	OtA5093-OtA5097
Mrps17	chr5:129715527-129718691	OtA5088-OtA5092	OtA5093-OtA5097
Msrb2	chr2:19371635-19394971	OtA5088-OtA5092	OtA5093-OtA5097
Mtif3	chr5:146948656-146963797	OtA5088-OtA5092	OtA5093-OtA5097
N6amt1	chr16:87354184-87368649	OtA5088-OtA5092	OtA5093-OtA5097
Nat15	chr16:3884618-3904781	OtA5088-OtA5092	OtA5093-OtA5097
Nbn	chr4:15957966-15992589	OtA5088-OtA5092	OtA5093-OtA5097
Ndufa6	chr15:82350138-82354291	OtA5088-OtA5092	OtA5093-OtA5097
Ndufaf1	chr2:119655450-119662798	OtA5088-OtA5092	OtA5093-OtA5097
Ndufs4	chr13:114287794-114388094	OtA5088-OtA5092	OtA5093-OtA5097
Ndufs5	chr4:123712709-123718186	OtA5088-OtA5092	OtA5093-OtA5097

Nedd1	chr10:92684744-92722418	OtA5088-OtA5092	OtA5093-OtA5097
Nedd9	chr13:41309915-41487320	OtA5088-OtA5092	OtA5093-OtA5097
Neil2	chr14:63182444-63193525	OtA5088-OtA5092	OtA5093-OtA5097
Nfs1	chr2:156123636-156145794	OtA5088-OtA5092	OtA5093-OtA5097
Nucb1	chr7:45492673-45510408	OtA5088-OtA5092	OtA5093-OtA5097
Nup133	chr8:123897122-123949265	OtA5088-OtA5092	OtA5093-OtA5097
Optn	chr2:5020641-5063938	OtA5088-OtA5092	OtA5093-OtA5097
Ormdl2	chr10:128805675-128821631	OtA5088-OtA5092	OtA5093-OtA5097
Paip1	chr13:119428599-119460320	OtA5088-OtA5092	OtA5093-OtA5097
Pcca	chr14:122534327-122889944	OtA5088-OtA5092	OtA5093-OtA5097
Pcdhgb4	chr18:36930284-37841872	OtA5088-OtA5092	OtA5093-OtA5097
Pcif1	chr2:164879367-164891437	OtA5088-OtA5092	OtA5093-OtA5097
Pdss2	chr10:43221485-43464882	OtA5088-OtA5092	OtA5093-OtA5097
Per2	chr1:91415981-91459328	OtA5088-OtA5092	OtA5093-OtA5097
Pex11a	chr7:79737263-79743025	OtA5088-OtA5092	OtA5093-OtA5097
Pim2	chrX:7878305-7883432	OtA5088-OtA5092	OtA5093-OtA5097
Pip5k1b	chr19:24294795-24555827	OtA5088-OtA5092	OtA5093-OtA5097
Plekhj1	chr10:80796098-80798626	OtA5088-OtA5092	OtA5093-OtA5097
Poc5	chr13:96388293-96415587	OtA5088-OtA5092	OtA5093-OtA5097
Podxl2	chr6:88842557-88874044	OtA5088-OtA5092	OtA5093-OtA5097
Pomt1	chr2:32236682-32260105	OtA5088-OtA5092	OtA5093-OtA5097
Popdc3	chr10:45289304-45318450	OtA5088-OtA5092	OtA5093-OtA5097
Ppp2r5a	chr1:191325964-191397041	OtA5088-OtA5092	OtA5093-OtA5097
Prl7c1	chr13:27773494-27780804	OtA5088-OtA5092	OtA5093-OtA5097
Psma3	chr12:70974622-71015823	OtA5088-OtA5092	OtA5093-OtA5097
Psmd11	chr11:80428614-80472133	OtA5088-OtA5092	OtA5093-OtA5097
Ptpdc1	chr13:48577871-48625573	OtA5088-OtA5092	OtA5093-OtA5097
Rab3d	chr9:21907510-21918121	OtA5088-OtA5092	OtA5093-OtA5097
Rabl2	chr15:89582526-89591923	OtA5088-OtA5092	OtA5093-OtA5097
Rap2a	chr14:120478460-120507192	OtA5088-OtA5092	OtA5093-OtA5097
Rasal2	chr1:157135182-157420236	OtA5088-OtA5092	OtA5093-OtA5097
Rbm12	chr2:156071840-156111965	OtA5088-OtA5092	OtA5093-OtA5097
Rbm20	chr19:53677305-53867080	OtA5088-OtA5092	OtA5093-OtA5097
Rbm22	chr18:60560785-60572729	OtA5088-OtA5092	OtA5093-OtA5097
Rpe	chr1:66700892-66817595	OtA5088-OtA5092	OtA5093-OtA5097
Rpl10	chrX:74270815-74273135	OtA5088-OtA5092	OtA5093-OtA5097
Rplp1	chr9:61913282-61914510	OtA5088-OtA5092	OtA5093-OtA5097
Rplp2	chr7:141447649-141451342	OtA5088-OtA5092	OtA5093-OtA5097
Rps19bp1	chr15:80260613-80264306	OtA5088-OtA5092	OtA5093-OtA5097
Rps4y2	chr6:148047415-148444352	OtA5088-OtA5092	OtA5093-OtA5097
Rrp15	chr1:186721086-186749358	OtA5088-OtA5092	OtA5093-OtA5097
Samd1	chr8:83997671-84000386	OtA5088-OtA5092	OtA5093-OtA5097
Samd12	chr15:53461800-53902436	OtA5088-OtA5092	OtA5093-OtA5097
Scamp4	chr10:80602879-80615783	OtA5088-OtA5092	OtA5093-OtA5097
Sdf4	chr4:155992913-156013610	OtA5088-OtA5092	OtA5093-OtA5097
Sema7a	chr9:57940134-57962865	OtA5088-OtA5092	OtA5093-OtA5097
Sestd1	chr2:77180339-77280592	OtA5088-OtA5092	OtA5093-OtA5097

Sgpl1	chr10:61098641-61147665	OtA5088-OtA5092	OtA5093-OtA5097
Sh2d4b	chr14:40815867-40892965	OtA5088-OtA5092	OtA5093-OtA5097
Sik3	chr9:46012819-46224194	OtA5088-OtA5092	OtA5093-OtA5097
Sipa1l2	chr8:125418062-125492710	OtA5088-OtA5092	OtA5093-OtA5097
Slc35a3	chr3:116670797-116712280	OtA5088-OtA5092	OtA5093-OtA5097
Slc35b4	chr6:34155878-34177054	OtA5088-OtA5092	OtA5093-OtA5097
Slc6a19	chr13:73681156-73709856	OtA5088-OtA5092	OtA5093-OtA5097
Snrnp200	chr2:127208403-127240451	OtA5088-OtA5092	OtA5093-OtA5097
Son	chr16:91647823-91679192	OtA5088-OtA5092	OtA5093-OtA5097
Spata2	chr2:167481135-167516166	OtA5088-OtA5092	OtA5093-OtA5097
Spon2	chr5:33213517-33218238	OtA5088-OtA5092	OtA5093-OtA5097
Srsf1	chr11:88047670-88053757	OtA5088-OtA5092	OtA5093-OtA5097
Ssx2ip	chr3:146404641-146440137	OtA5088-OtA5092	OtA5093-OtA5097
Sulf2	chr2:166073898-166155683	OtA5088-OtA5092	OtA5093-OtA5097
Synb	chr14:69508647-69512549	OtA5088-OtA5092	OtA5093-OtA5097
Tacc2	chr7:130577483-130764784	OtA5088-OtA5092	OtA5093-OtA5097
Taf12	chr4:132274374-132293330	OtA5088-OtA5092	OtA5093-OtA5097
Taf9b	chrX:106206873-106221158	OtA5088-OtA5092	OtA5093-OtA5097
Tbc1d10b	chr7:127197458-127208468	OtA5088-OtA5092	OtA5093-OtA5097
Tchhl1	chr3:93468753-93471980	OtA5088-OtA5092	OtA5093-OtA5097
Tead2	chr7:45215752-45233619	OtA5088-OtA5092	OtA5093-OtA5097
Thap1	chr8:26158168-26164151	OtA5088-OtA5092	OtA5093-OtA5097
Ticam1	chr17:56269461-56276767	OtA5088-OtA5092	OtA5093-OtA5097
Timm17a	chr1:135301534-135313737	OtA5088-OtA5092	OtA5093-OtA5097
Timm8b	chr9:50603900-50625000	OtA5088-OtA5092	OtA5093-OtA5097
Tmem130	chr5:144735914-144761578	OtA5088-OtA5092	OtA5093-OtA5097
Tmem170	chr8:111864897-111876675	OtA5088-OtA5092	OtA5093-OtA5097
Tmem206	chr1:191325964-191397041	OtA5088-OtA5092	OtA5093-OtA5097
Tmem41b	chr7:109972186-109986230	OtA5088-OtA5092	OtA5093-OtA5097
Tnrc18	chr5:142724604-142817387	OtA5088-OtA5092	OtA5093-OtA5097
Trappc2	chrX:166440824-166452543	OtA5088-OtA5092	OtA5093-OtA5097
Triml2	chr8:43183121-43193884	OtA5088-OtA5092	OtA5093-OtA5097
Trpt1	chr19:6996130-7019469	OtA5088-OtA5092	OtA5093-OtA5097
Tshz1	chr18:84011626-84086562	OtA5088-OtA5092	OtA5093-OtA5097
Ttc30b	chr2:75935849-75938462	OtA5088-OtA5092	OtA5093-OtA5097
Ube2l6	chr2:84798827-84810003	OtA5088-OtA5092	OtA5093-OtA5097
Ube2n	chr10:95515161-95545658	OtA5088-OtA5092	OtA5093-OtA5097
Uhrf1	chr17:56303336-56323486	OtA5088-OtA5092	OtA5093-OtA5097
Uqcr10	chr11:4701967-4704344	OtA5088-OtA5092	OtA5093-OtA5097
Wdr33	chr18:31804056-31908987	OtA5088-OtA5092	OtA5093-OtA5097
Wdr35	chr12:8974000-9028847	OtA5088-OtA5092	OtA5093-OtA5097
Xpa	chr4:46175221-46196311	OtA5088-OtA5092	OtA5093-OtA5097
Ykt6	chr11:5955757-5967781	OtA5088-OtA5092	OtA5093-OtA5097
Zbtb42	chr12:112678839-112682747	OtA5088-OtA5092	OtA5093-OtA5097
Zcchc3	chr2:152411955-152415044	OtA5088-OtA5092	OtA5093-OtA5097
Zdhhc6	chr19:55298295-55316032	OtA5088-OtA5092	OtA5093-OtA5097
Znrd1	chr17:36954357-36958428	OtA5088-OtA5092	OtA5093-OtA5097

Acot8	chr2:164786020-164804881	OtA5088-OtA5092	OtA5093-OtA5097
Fahd2a	chr2:127436214-127444565	OtA5088-OtA5092	OtA5093-OtA5097
Tagln	chr9:45863690-45936058	OtA5088-OtA5092	OtA5093-OtA5097
Pdcd7	chr9:65346067-65359643	OtA5088-OtA5092	OtA5093-OtA5097
Rnaseh2a	chr8:84956609-84966011	OtA5088-OtA5092	OtA5093-OtA5097
Arrb2	chr11:70432652-70440674	OtA5088-OtA5092	OtA5093-OtA5097
Ppp1r15a	chr7:45522916-45526268	OtA5088-OtA5092	OtA5093-OtA5097
Ppp1r16b	chr2:158666732-158766334	OtA5088-OtA5092	OtA5093-OtA5097
Sppl3	chr5:115011523-115098790	OtA5088-OtA5092	OtA5093-OtA5097
Surf4	chr2:26913377-26933511	OtA5088-OtA5092	OtA5093-OtA5097
Cox7b	chrX:106015699-106022450	OtA5088-OtA5092	OtA5093-OtA5097
Mfng	chr15:78755882-78773445	OtA5088-OtA5092	OtA5093-OtA5097
Prkci	chr3:30995770-31052740	OtA5088-OtA5092	OtA5093-OtA5097
Rfc3	chr5:151642823-151651208	OtA5088-OtA5092	OtA5093-OtA5097
Slc46a1	chr11:78465700-78471945	OtA5088-OtA5092	OtA5093-OtA5097
Zfp771	chr7:127244525-127254801	OtA5088-OtA5092	OtA5093-OtA5097
Slc9a1	chr4:133369771-133423698	OtA5088-OtA5092	OtA5093-OtA5097
Palm3	chr8:84021473-84042575	OtA5088-OtA5092	OtA5093-OtA5097
Riok3	chr18:12128849-12157367	OtA5088-OtA5092	OtA5093-OtA5097
Tmub2	chr11:102284960-102296629	OtA5088-OtA5092	OtA5093-OtA5097
Vps37b	chr5:124004640-124032260	OtA5088-OtA5092	OtA5093-OtA5097
Bloc1s2	chr19:44139246-44146446	OtA5088-OtA5092	OtA5093-OtA5097
Dnajc30	chr5:135052956-135065365	OtA5088-OtA5092	OtA5093-OtA5097
Dnttip1	chr2:164746014-164768219	OtA5088-OtA5092	OtA5093-OtA5097
Ei24	chr9:36779152-36797393	OtA5088-OtA5092	OtA5093-OtA5097
Polr2d	chr18:31789158-31796701	OtA5088-OtA5092	OtA5093-OtA5097
Sctr	chr1:120006979-120063532	OtA5088-OtA5092	OtA5093-OtA5097
Ccdc92	chr5:124834431-124862221	OtA5088-OtA5092	OtA5093-OtA5097
D1Bwg0212e	chr1:39535801-39546877	OtA5088-OtA5092	OtA5093-OtA5097
Dffa	chr4:149104152-149120650	OtA5088-OtA5092	OtA5093-OtA5097
Dusp28	chr1:92906988-92908620	OtA5088-OtA5092	OtA5093-OtA5097
Elmod2	chr8:83312631-83332486	OtA5088-OtA5092	OtA5093-OtA5097
Hn1	chr11:115497352-115514370	OtA5088-OtA5092	OtA5093-OtA5097
Hoxd9	chr2:74697762-74700208	OtA5088-OtA5092	OtA5093-OtA5097
Itprip	chr19:47894595-47919299	OtA5088-OtA5092	OtA5093-OtA5097
Kbtbd7	chr14:79426510-79431038	OtA5088-OtA5092	OtA5093-OtA5097
Me1	chr9:86581362-86701253	OtA5088-OtA5092	OtA5093-OtA5097
Mpp2	chr11:102057016-102088515	OtA5088-OtA5092	OtA5093-OtA5097
Rasgrp2	chr19:6400582-6415216	OtA5088-OtA5092	OtA5093-OtA5097
Slc43a3	chr2:84936645-84958509	OtA5088-OtA5092	OtA5093-OtA5097
Ttc27	chr17:74717749-74863570	OtA5088-OtA5092	OtA5093-OtA5097
Ttll12	chr15:83575093-83595157	OtA5088-OtA5092	OtA5093-OtA5097
Zfp553	chr7:127233442-127237860	OtA5088-OtA5092	OtA5093-OtA5097
Zfp317	chr9:19622090-19649731	OtA5088-OtA5092	OtA5093-OtA5097
Arpc3	chr5:122391927-122406178	OtA5088-OtA5092	OtA5093-OtA5097
Chrac1	chr15:73090411-73094075	OtA5088-OtA5092	OtA5093-OtA5097
Cyyr1	chr16:85456243-85550373	OtA5088-OtA5092	OtA5093-OtA5097

Homer3	chr8:70282998-70302452	OtA5088-OtA5092	OtA5093-OtA5097
Hps1	chr19:42755195-42779976	OtA5088-OtA5092	OtA5093-OtA5097
Mutyh	chr4:116807733-116819431	OtA5088-OtA5092	OtA5093-OtA5097
Nbeal2	chr9:110624788-110654161	OtA5088-OtA5092	OtA5093-OtA5097
Ppp1r13b	chr12:111828457-111908055	OtA5088-OtA5092	OtA5093-OtA5097
Ntn3	chr17:24175430-24209387	OtA5088-OtA5092	OtA5093-OtA5097
Osbpl2	chr2:180119365-180162680	OtA5088-OtA5092	OtA5093-OtA5097
Edc4	chr8:105880950-105893207	OtA5088-OtA5092	OtA5093-OtA5097
Tmem101	chr11:102152546-102156407	OtA5088-OtA5092	OtA5093-OtA5097
Inpp5e	chr2:26389347-26409188	OtA5088-OtA5092	OtA5093-OtA5097
Pdlim1	chr19:40222238-40271616	OtA5088-OtA5092	OtA5093-OtA5097
Syf2	chr4:134930979-134937537	OtA5088-OtA5092	OtA5093-OtA5097
Thrsp	chr7:97412956-97417510	OtA5088-OtA5092	OtA5093-OtA5097
Tubgcp6	chr15:89089106-89123150	OtA5088-OtA5092	OtA5093-OtA5097
Ppm1a	chr12:72761210-72794940	OtA5088-OtA5092	OtA5093-OtA5097
Plagl2	chr2:153227768-153241358	OtA5088-OtA5092	OtA5093-OtA5097
Rab40c	chr17:25882113-25919714	OtA5088-OtA5092	OtA5093-OtA5097
Akr1b10	chr6:34384246-34396949	OtA5088-OtA5092	OtA5093-OtA5097
Psmb10	chr8:105935727-105938392	OtA5088-OtA5092	OtA5093-OtA5097
Zfp746	chr6:48062394-48086593	OtA5088-OtA5092	OtA5093-OtA5097
Adrbk1	chr19:4286000-4305955	OtA5088-OtA5092	OtA5093-OtA5097
Derl2	chr11:71007444-71019263	OtA5088-OtA5092	OtA5093-OtA5097
Slc25a39	chr11:102402975-102407517	OtA5088-OtA5092	OtA5093-OtA5097
Cmtm4	chr8:104348192-104395807	OtA5088-OtA5092	OtA5093-OtA5097
Cyb5rl	chr4:107070167-107084805	OtA5088-OtA5092	OtA5093-OtA5097
Dgkq	chr5:108629809-108660769	OtA5088-OtA5092	OtA5093-OtA5097
Nap1l4	chr7:143513578-143549090	OtA5088-OtA5092	OtA5093-OtA5097
Arv1	chr8:124722138-124734123	OtA5088-OtA5092	OtA5093-OtA5097
Ankrd54	chr15:79053093-79062859	OtA5088-OtA5092	OtA5093-OtA5097
Vash2	chr1:190947645-190979296	OtA5088-OtA5092	OtA5093-OtA5097
Adi1	chr12:28675206-28682174	OtA5088-OtA5092	OtA5093-OtA5097
Arnt	chr3:95434389-95497239	OtA5088-OtA5092	OtA5093-OtA5097
C630004H02Rik	chr11:115347708-115367719	OtA5088-OtA5092	OtA5093-OtA5097
Cdc16	chr8:13757689-13781882	OtA5088-OtA5092	OtA5093-OtA5097
Chmp2a	chr7:13032005-13034777	OtA5088-OtA5092	OtA5093-OtA5097
Fbxw11	chr11:32642874-32746814	OtA5088-OtA5092	OtA5093-OtA5097
Grb10	chr11:11930498-12037420	OtA5088-OtA5092	OtA5093-OtA5097
Igfbp6	chr15:102144185-102149512	OtA5088-OtA5092	OtA5093-OtA5097
Il10rb	chr16:91406234-91425834	OtA5088-OtA5092	OtA5093-OtA5097
Itsn2	chr12:4593007-4738383	OtA5088-OtA5092	OtA5093-OtA5097
Mettl21a	chr1:64606479-64617168	OtA5088-OtA5092	OtA5093-OtA5097
Nfatc2ip	chr7:126382853-126396737	OtA5088-OtA5092	OtA5093-OtA5097
Pgp	chr17:24470472-24471596	OtA5088-OtA5092	OtA5093-OtA5097
Plod3	chr5:136982200-136996646	OtA5088-OtA5092	OtA5093-OtA5097
Ppp2r5c	chr12:110447178-110583061	OtA5088-OtA5092	OtA5093-OtA5097
Scamp3	chr3:89177484-89182770	OtA5088-OtA5092	OtA5093-OtA5097
Sec31a	chr5:100361648-100416234	OtA5088-OtA5092	OtA5093-OtA5097

Snora52	chr7:141447649-141451342	OtA5088-OtA5092	OtA5093-OtA5097
Spns2	chr11:72451637-72489904	OtA5088-OtA5092	OtA5093-OtA5097
Spop	chr11:95414082-95493410	OtA5088-OtA5092	OtA5093-OtA5097
Tmem180	chr19:46356879-46375254	OtA5088-OtA5092	OtA5093-OtA5097
Trp53bp2	chr1:182409166-182462436	OtA5088-OtA5092	OtA5093-OtA5097
Ttc17	chr2:94300765-94406689	OtA5088-OtA5092	OtA5093-OtA5097
Usp14	chr18:9958179-10030149	OtA5088-OtA5092	OtA5093-OtA5097
Zbtb4	chr11:69765911-69784026	OtA5088-OtA5092	OtA5093-OtA5097
Zmynd19	chr2:24949801-24959413	OtA5088-OtA5092	OtA5093-OtA5097
Agrp	chr8:105566697-105568298	OtA5088-OtA5092	OtA5093-OtA5097
Calcoco1	chr15:102706776-102722178	OtA5088-OtA5092	OtA5093-OtA5097
Dstyk	chr1:132417452-132466959	OtA5088-OtA5092	OtA5093-OtA5097
F3	chr3:121723536-121735052	OtA5088-OtA5092	OtA5093-OtA5097
Hsd17b10	chrX:152001895-152004442	OtA5088-OtA5092	OtA5093-OtA5097
Irs2	chr8:10986963-11054541	OtA5088-OtA5092	OtA5093-OtA5097
Pak4	chr7:28558818-28598184	OtA5088-OtA5092	OtA5093-OtA5097
Prnp	chr2:131909927-131938431	OtA5088-OtA5092	OtA5093-OtA5097
Sh3glb2	chr2:30344808-30359231	OtA5088-OtA5092	OtA5093-OtA5097
Wdr37	chr13:8802967-8871730	OtA5088-OtA5092	OtA5093-OtA5097
B4galt4	chr16:38742263-38769045	OtA5088-OtA5092	OtA5093-OtA5097
Dhrs7b	chr11:60830630-60858423	OtA5088-OtA5092	OtA5093-OtA5097
Fkbp3	chr12:65036333-65073938	OtA5088-OtA5092	OtA5093-OtA5097
Rab3a	chr8:70754678-70760921	OtA5088-OtA5092	OtA5093-OtA5097
Trim2	chr3:84161873-84220877	OtA5088-OtA5092	OtA5093-OtA5097
Zbtb49	chr5:38200043-38220428	OtA5088-OtA5092	OtA5093-OtA5097
Zfp266	chr9:20495068-20521419	OtA5088-OtA5092	OtA5093-OtA5097
Apob	chr12:7977676-8016839	OtA5088-OtA5092	OtA5093-OtA5097
Ddx59	chr1:136411915-136440220	OtA5088-OtA5092	OtA5093-OtA5097
Dnajc21	chr15:10446762-10470516	OtA5088-OtA5092	OtA5093-OtA5097
Khsrp	chr17:57021048-57031507	OtA5088-OtA5092	OtA5093-OtA5097
Phf23	chr11:69995770-70000011	OtA5088-OtA5092	OtA5093-OtA5097
Prpf38a	chr4:108564866-108579336	OtA5088-OtA5092	OtA5093-OtA5097
Ralb	chr1:119470304-119504782	OtA5088-OtA5092	OtA5093-OtA5097
Ankrd27	chr7:35586246-35639237	OtA5088-OtA5092	OtA5093-OtA5097
Sdpr	chr1:51218385-51333779	OtA5088-OtA5092	OtA5093-OtA5097
Cherp	chr8:72443879-72475233	OtA5088-OtA5092	OtA5093-OtA5097
Nt5m	chr11:59848072-59876533	OtA5088-OtA5092	OtA5093-OtA5097
Samd4b	chr7:28399521-28436191	OtA5088-OtA5092	OtA5093-OtA5097
Slc35c2	chr2:165276521-165287838	OtA5088-OtA5092	OtA5093-OtA5097
Exoc3l	chr8:105289923-105296098	OtA5088-OtA5092	OtA5093-OtA5097
Rnf7	chr9:96470956-96478595	OtA5088-OtA5092	OtA5093-OtA5097
Sf3b5	chr10:13008449-13009183	OtA5088-OtA5092	OtA5093-OtA5097
Slc15a2	chr16:36750163-36784962	OtA5088-OtA5092	OtA5093-OtA5097
Col27a1	chr4:63215411-63334990	OtA5088-OtA5092	OtA5093-OtA5097
Mapk13	chr17:28769316-28778704	OtA5088-OtA5092	OtA5093-OtA5097
Mrpl52	chr14:54426908-54429750	OtA5088-OtA5092	OtA5093-OtA5097
Pus10	chr11:23646478-23732876	OtA5088-OtA5092	OtA5093-OtA5097

Adhfe1	chr1:9548045-9631092	OtA5088-OtA5092	OtA5093-OtA5097
Ccl21a,Gm1987	chr4_JH584294_random:120654-12178[illegible]	OtA5088-OtA5092	OtA5093-OtA5097
Dnajb6	chr5:29735333-29786478	OtA5088-OtA5092	OtA5093-OtA5097
Mettl2	chr11:105126424-105141146	OtA5088-OtA5092	OtA5093-OtA5097
Rnps1	chr17:24414674-24425897	OtA5088-OtA5092	OtA5093-OtA5097
Swi5	chr2:32278815-32288068	OtA5088-OtA5092	OtA5093-OtA5097
Ankrd13a	chr5:114775139-114805820	OtA5088-OtA5092	OtA5093-OtA5097
Antxr1	chr6:87133852-87335775	OtA5088-OtA5092	OtA5093-OtA5097
Atp5k	chr5:108433252-108434378	OtA5088-OtA5092	OtA5093-OtA5097
Cog6	chr3:52982122-53017223	OtA5088-OtA5092	OtA5093-OtA5097
Ifitm2	chr7:140954838-140955961	OtA5088-OtA5092	OtA5093-OtA5097
Lst1	chr17:35185094-35188440	OtA5088-OtA5092	OtA5093-OtA5097
Man2c1	chr9:57130776-57142210	OtA5088-OtA5092	OtA5093-OtA5097
Mau2	chr8:70016122-70042734	OtA5088-OtA5092	OtA5093-OtA5097
Ppard	chr17:28232753-28301469	OtA5088-OtA5092	OtA5093-OtA5097
Rpsa	chr9:120127765-120132369	OtA5088-OtA5092	OtA5093-OtA5097
Sft2d1	chr17:8311102-8327442	OtA5088-OtA5092	OtA5093-OtA5097
Stoml1	chr9:58253163-58262524	OtA5088-OtA5092	OtA5093-OtA5097
Vaultrc5	chr18:36801762-36802107	OtA5088-OtA5092	OtA5093-OtA5097
Sf1	chr19:6363689-6378038	OtA5088-OtA5092	OtA5093-OtA5097
Snx15	chr19:6119403-6128215	OtA5088-OtA5092	OtA5093-OtA5097
Tsga14	chr6:30653456-30693682	OtA5088-OtA5092	OtA5093-OtA5097
Coil	chr11:88973934-88991613	OtA5088-OtA5092	OtA5093-OtA5097
Higd1b	chr11:102836295-102838039	OtA5088-OtA5092	OtA5093-OtA5097
Jtb	chr3:90231596-90235840	OtA5088-OtA5092	OtA5093-OtA5097
Bcl7c	chr7:127704977-127708766	OtA5088-OtA5092	OtA5093-OtA5097
Itga1	chr13:114958080-115101964	OtA5088-OtA5092	OtA5093-OtA5097
Cdc123	chr2:5794293-5844960	OtA5088-OtA5092	OtA5093-OtA5097
Prr14	chr7:127471613-127476758	OtA5088-OtA5092	OtA5093-OtA5097
Nhej1	chr1:74967345-75046639	OtA5088-OtA5092	OtA5093-OtA5097
Ttll1	chr15:83483768-83510907	OtA5088-OtA5092	OtA5093-OtA5097
Actn4	chr7:28881655-28962280	OtA5088-OtA5092	OtA5093-OtA5097
Afap1	chr5:35893318-36003922	OtA5088-OtA5092	OtA5093-OtA5097
Agap3	chr5:24452176-24502047	OtA5088-OtA5092	OtA5093-OtA5097
Aoc3	chr11:101330605-101339430	OtA5088-OtA5092	OtA5093-OtA5097
Btbd2	chr10:80642616-80657071	OtA5088-OtA5092	OtA5093-OtA5097
Carm1	chr9:21546893-21595970	OtA5088-OtA5092	OtA5093-OtA5097
Ccdc120	chrX:7731713-7741324	OtA5088-OtA5092	OtA5093-OtA5097
Cd55	chr1:130439029-130462740	OtA5088-OtA5092	OtA5093-OtA5097
Cfl2	chr12:54858818-54862877	OtA5088-OtA5092	OtA5093-OtA5097
Chchd7	chr4:3938887-3943525	OtA5088-OtA5092	OtA5093-OtA5097
Clcf1	chr19:4214391-4222615	OtA5088-OtA5092	OtA5093-OtA5097
Cnn1	chr9:22055459-22389206	OtA5088-OtA5092	OtA5093-OtA5097
Coq10b	chr1:55052769-55072702	OtA5088-OtA5092	OtA5093-OtA5097
Csnk1e	chr15:79417851-79442057	OtA5088-OtA5092	OtA5093-OtA5097
Disp1	chr1:183086263-183221529	OtA5088-OtA5092	OtA5093-OtA5097
Dnm1	chr2:32308470-32353304	OtA5088-OtA5092	OtA5093-OtA5097

Egr1	chr18:34861206-34864956	OtA5088-OtA5092	OtA5093-OtA5097
Exd2	chr12:80463094-80498135	OtA5088-OtA5092	OtA5093-OtA5097
Foxo3	chr10:42185785-42276742	OtA5088-OtA5092	OtA5093-OtA5097
Fscn1	chr5:142960354-142973189	OtA5088-OtA5092	OtA5093-OtA5097
Gstm4	chr3:108040407-108044859	OtA5088-OtA5092	OtA5093-OtA5097
H2-Q9	chr17:35439154-35443773	OtA5088-OtA5092	OtA5093-OtA5097
Hspa9	chr18:34937414-34954351	OtA5088-OtA5092	OtA5093-OtA5097
Mbtps2	chrX:157547821-157598715	OtA5088-OtA5092	OtA5093-OtA5097
Mlph	chr1:90915099-90951142	OtA5088-OtA5092	OtA5093-OtA5097
Mospd3	chr5:137596646-137601040	OtA5088-OtA5092	OtA5093-OtA5097
Naa35	chr13:59585332-59634781	OtA5088-OtA5092	OtA5093-OtA5097
Narf	chr11:121237235-121255856	OtA5088-OtA5092	OtA5093-OtA5097
Nsf	chr11:103821782-103954056	OtA5088-OtA5092	OtA5093-OtA5097
Otop1	chr5:38277403-38304217	OtA5088-OtA5092	OtA5093-OtA5097
Pdpn	chr4:143267421-143299507	OtA5088-OtA5092	OtA5093-OtA5097
Plaa	chr4:94565138-94603247	OtA5088-OtA5092	OtA5093-OtA5097
Plcd3	chr11:103070295-103101658	OtA5088-OtA5092	OtA5093-OtA5097
Plekhb2	chr1:34849958-34879585	OtA5088-OtA5092	OtA5093-OtA5097
Pmm1	chr15:81951105-81960867	OtA5088-OtA5092	OtA5093-OtA5097
Pnpla3	chr15:84167815-84189521	OtA5088-OtA5092	OtA5093-OtA5097
Pofut2	chr10:77259299-77269586	OtA5088-OtA5092	OtA5093-OtA5097
Prdx1	chr4:116684964-116700000	OtA5088-OtA5092	OtA5093-OtA5097
Prpsap1	chr11:116470815-116490346	OtA5088-OtA5092	OtA5093-OtA5097
Rasa3	chr8:13567217-13677587	OtA5088-OtA5092	OtA5093-OtA5097
Rbbp5	chr1:132477366-132505665	OtA5088-OtA5092	OtA5093-OtA5097
Rcn1	chr2:105385947-105399319	OtA5088-OtA5092	OtA5093-OtA5097
Rhobtb1	chr10:69208551-69291784	OtA5088-OtA5092	OtA5093-OtA5097
Rpp30	chr19:36083715-36104773	OtA5088-OtA5092	OtA5093-OtA5097
Skp2	chr15:9111984-9140451	OtA5088-OtA5092	OtA5093-OtA5097
Slc35b3	chr13:38932139-38960875	OtA5088-OtA5092	OtA5093-OtA5097
Smpd2	chr10:41487171-41490340	OtA5088-OtA5092	OtA5093-OtA5097
Snupn	chr9:56950923-56983199	OtA5088-OtA5092	OtA5093-OtA5097
Spin4	chrX:95022506-95026682	OtA5088-OtA5092	OtA5093-OtA5097
Stk10	chr11:32533265-32624595	OtA5088-OtA5092	OtA5093-OtA5097
Ston1	chr17:88626554-88645724	OtA5088-OtA5092	OtA5093-OtA5097
Susd4	chr1:182764905-182895654	OtA5088-OtA5092	OtA5093-OtA5097
Tbc1d9	chr8:83165351-83272940	OtA5088-OtA5092	OtA5093-OtA5097
Tbl1x	chrX:77511226-77660265	OtA5088-OtA5092	OtA5093-OtA5097
Tmem198	chr1:75479531-75485693	OtA5088-OtA5092	OtA5093-OtA5097
Tmem48	chr4:107367783-107414338	OtA5088-OtA5092	OtA5093-OtA5097
Tsen2	chr6:115544703-115578336	OtA5088-OtA5092	OtA5093-OtA5097
Usp2	chr9:44067020-44095627	OtA5088-OtA5092	OtA5093-OtA5097
Vcpip1	chr1:9723195-9771256	OtA5088-OtA5092	OtA5093-OtA5097
Vwa5a	chr9:38718267-38743337	OtA5088-OtA5092	OtA5093-OtA5097
Was	chrX:8081465-8090491	OtA5088-OtA5092	OtA5093-OtA5097
Yipf2	chr9:21546893-21595970	OtA5088-OtA5092	OtA5093-OtA5097
Zc3h12c	chr9:52111984-52168111	OtA5088-OtA5092	OtA5093-OtA5097

Zfp821	chr8:109705548-109724932	OtA5088-OtA5092	OtA5093-OtA5097
Cript	chr17:87025560-87035808	OtA5088-OtA5092	OtA5093-OtA5097
Gtf3c5	chr2:28566262-28583279	OtA5088-OtA5092	OtA5093-OtA5097
Kat5	chr19:5603013-5610094	OtA5088-OtA5092	OtA5093-OtA5097
Rala	chr13:17880574-17944217	OtA5088-OtA5092	OtA5093-OtA5097
Raly	chr2:154791109-154867261	OtA5088-OtA5092	OtA5093-OtA5097
Sfpq	chr4:127021300-127037014	OtA5088-OtA5092	OtA5093-OtA5097
Ccdc80	chr16:45094052-45127924	OtA5088-OtA5092	OtA5093-OtA5097
Fam158a	chr14:55578493-55585254	OtA5088-OtA5092	OtA5093-OtA5097
Arhgef11	chr3:87618750-87748623	OtA5088-OtA5092	OtA5093-OtA5097
D7Ertd443e	chr7:134266261-134376828	OtA5088-OtA5092	OtA5093-OtA5097
Mrps26	chr2:130563756-130565394	OtA5088-OtA5092	OtA5093-OtA5097
Rps20	chr4:3834472-3835600	OtA5088-OtA5092	OtA5093-OtA5097
Scnm1	chr3:95129718-95134012	OtA5088-OtA5092	OtA5093-OtA5097
Supt4h1	chr11:87737564-87743617	OtA5088-OtA5092	OtA5093-OtA5097
Tfpi	chr2:84432859-84476775	OtA5088-OtA5092	OtA5093-OtA5097
Pex16	chr2:92374675-92381220	OtA5088-OtA5092	OtA5093-OtA5097
Hspa4	chr11:53259813-53300479	OtA5088-OtA5092	OtA5093-OtA5097
Ndufa4l2	chr10:127514938-127522444	OtA5088-OtA5092	OtA5093-OtA5097
Prmt6	chr3:110246109-110250998	OtA5088-OtA5092	OtA5093-OtA5097
Rad1	chr15:10486033-10493691	OtA5088-OtA5092	OtA5093-OtA5097
Syne2	chr12:75818317-76110928	OtA5088-OtA5092	OtA5093-OtA5097
Szt2	chr4:118359998-118409263	OtA5088-OtA5092	OtA5093-OtA5097
Tbc1d17	chr7:44812255-44849079	OtA5088-OtA5092	OtA5093-OtA5097
Alkbh4	chr5:136138780-136141614	OtA5088-OtA5092	OtA5093-OtA5097
Copb2	chr9:98563730-98588375	OtA5088-OtA5092	OtA5093-OtA5097
Dazap2	chr15:100615661-100620761	OtA5088-OtA5092	OtA5093-OtA5097
Ddx27	chr2:167015312-167034945	OtA5088-OtA5092	OtA5093-OtA5097
Ehd3	chr17:73804840-73832093	OtA5088-OtA5092	OtA5093-OtA5097
Gm14005	chr2:128298662-128429351	OtA5088-OtA5092	OtA5093-OtA5097
Hdac1	chr4:129516103-129542646	OtA5088-OtA5092	OtA5093-OtA5097
Iqsec1	chr6:90659597-90810123	OtA5088-OtA5092	OtA5093-OtA5097
Pml	chr9:58217179-58249786	OtA5088-OtA5092	OtA5093-OtA5097
Tbc1d12	chr19:38836578-38919923	OtA5088-OtA5092	OtA5093-OtA5097
Tmem126a	chr7:90450711-90457208	OtA5088-OtA5092	OtA5093-OtA5097
Trappc6b	chr12:59043091-59061461	OtA5088-OtA5092	OtA5093-OtA5097
Wbp4	chr14:79459936-79582476	OtA5088-OtA5092	OtA5093-OtA5097
Clta	chr4:44012642-44032846	OtA5088-OtA5092	OtA5093-OtA5097
Kin	chr2:10080611-10092701	OtA5088-OtA5092	OtA5093-OtA5097
Trim29	chr9:43310762-43336125	OtA5088-OtA5092	OtA5093-OtA5097
Klhl22	chr16:17759620-17793382	OtA5088-OtA5092	OtA5093-OtA5097
5-Mar	chr19:37207544-37224457	OtA5088-OtA5092	OtA5093-OtA5097
Add1	chr5:34573713-34632305	OtA5088-OtA5092	OtA5093-OtA5097
Ahsa1	chr12:87266727-87273952	OtA5088-OtA5092	OtA5093-OtA5097
Anks1	chr17:27909339-28062636	OtA5088-OtA5092	OtA5093-OtA5097
B3galt5	chr16:96235800-96319858	OtA5088-OtA5092	OtA5093-OtA5097
Ccni	chr5:93181932-93206495	OtA5088-OtA5092	OtA5093-OtA5097

Cd151	chr7:141467361-141471481	OtA5088-OtA5092	OtA5093-OtA5097
Chadl	chr15:81663935-81697287	OtA5088-OtA5092	OtA5093-OtA5097
Cklf	chr8:104250902-104264936	OtA5088-OtA5092	OtA5093-OtA5097
Cox17	chr16:38346998-38352763	OtA5088-OtA5092	OtA5093-OtA5097
Cpeb3	chr19:37021290-37207293	OtA5088-OtA5092	OtA5093-OtA5097
Det1	chr7:78827473-78847211	OtA5088-OtA5092	OtA5093-OtA5097
Ergic2	chr6:148047415-148444352	OtA5088-OtA5092	OtA5093-OtA5097
Fkbp4	chr6:128430106-128438631	OtA5088-OtA5092	OtA5093-OtA5097
Fkbp9	chr6:56832058-56879360	OtA5088-OtA5092	OtA5093-OtA5097
Hexim2	chr11:103133338-103139908	OtA5088-OtA5092	OtA5093-OtA5097
Kctd20	chr17:28953215-28967937	OtA5088-OtA5092	OtA5093-OtA5097
Klraq1	chr17:88530123-88588367	OtA5088-OtA5092	OtA5093-OtA5097
Lin52	chr12:84451507-84531533	OtA5088-OtA5092	OtA5093-OtA5097
Mpg	chr11:32226504-32267614	OtA5088-OtA5092	OtA5093-OtA5097
N4bp3	chr11:51643088-51657681	OtA5088-OtA5092	OtA5093-OtA5097
Oaz1-ps	chr10:80826655-80829290	OtA5088-OtA5092	OtA5093-OtA5097
Pdgfra	chr5:75152290-75198204	OtA5088-OtA5092	OtA5093-OtA5097
Prl2c3,Prl2c4	chr13:12790817-12800079	OtA5088-OtA5092	OtA5093-OtA5097
Psmc6	chr14:45329823-45349071	OtA5088-OtA5092	OtA5093-OtA5097
Ptprr	chr10:116018362-116274929	OtA5088-OtA5092	OtA5093-OtA5097
Sfxn3	chr19:45047575-45056383	OtA5088-OtA5092	OtA5093-OtA5097
Snx30	chr4:59805649-59904740	OtA5088-OtA5092	OtA5093-OtA5097
Swap70	chr7:110221702-110283506	OtA5088-OtA5092	OtA5093-OtA5097
Tbc1d2	chr4:46604389-46650199	OtA5088-OtA5092	OtA5093-OtA5097
Tmem138	chr19:10570884-10577097	OtA5088-OtA5092	OtA5093-OtA5097
Tram1	chr1:13564701-13589864	OtA5088-OtA5092	OtA5093-OtA5097
Ube2o	chr11:116537752-116581447	OtA5088-OtA5092	OtA5093-OtA5097
Utp6	chr11:79933955-79962387	OtA5088-OtA5092	OtA5093-OtA5097
Wdr73	chr7:80890722-80901269	OtA5088-OtA5092	OtA5093-OtA5097
Isoc2b	chr7:4844959-4866179	OtA5088-OtA5092	OtA5093-OtA5097
Bag2	chr1:33745483-33757750	OtA5088-OtA5092	OtA5093-OtA5097
Dnalc4	chr15:79761448-79774467	OtA5088-OtA5092	OtA5093-OtA5097
Fam160a2	chr7:105371210-105400054	OtA5088-OtA5092	OtA5093-OtA5097
Fam36a	chr1:178319152-178322693	OtA5088-OtA5092	OtA5093-OtA5097
Gorab	chr1:163384908-163403641	OtA5088-OtA5092	OtA5093-OtA5097
Ing2	chr8:47667177-47675159	OtA5088-OtA5092	OtA5093-OtA5097
Mprip	chr11:59662494-59780860	OtA5088-OtA5092	OtA5093-OtA5097
Mrps30	chr13:118380109-118387252	OtA5088-OtA5092	OtA5093-OtA5097
Paf1	chr7:28392995-28399383	OtA5088-OtA5092	OtA5093-OtA5097
Rnf214	chr9:45863690-45936058	OtA5088-OtA5092	OtA5093-OtA5097
Slc2a8	chr2:32972988-32982056	OtA5088-OtA5092	OtA5093-OtA5097
Nup54	chr5:92415539-92435199	OtA5088-OtA5092	OtA5093-OtA5097
Brox	chr1:183276341-183297008	OtA5088-OtA5092	OtA5093-OtA5097
Chmp7	chr14:69716978-69732570	OtA5088-OtA5092	OtA5093-OtA5097
Shfm1	chr6:6558274-6578658	OtA5088-OtA5092	OtA5093-OtA5097
Prickle2	chr6:92370891-92706184	OtA5088-OtA5092	OtA5093-OtA5097
Ctla2a	chr13:60934154-60936625	OtA5088-OtA5092	OtA5093-OtA5097

Arpc5l	chr2:39008138-39015872	OtA5088-OtA5092	OtA5093-OtA5097
Nr1d2	chr14:18204055-18239106	OtA5088-OtA5092	OtA5093-OtA5097
Serf2	chr2:121449227-121456764	OtA5088-OtA5092	OtA5093-OtA5097
Dapk2	chr9:66158225-66272242	OtA5088-OtA5092	OtA5093-OtA5097
Plcxd2	chr16:45959260-46010413	OtA5088-OtA5092	OtA5093-OtA5097
Zfp341	chr2:154613367-154646817	OtA5088-OtA5092	OtA5093-OtA5097
Gpkow	chrX:7697133-7710259	OtA5088-OtA5092	OtA5093-OtA5097
Kctd7	chr5:130144887-130155808	OtA5088-OtA5092	OtA5093-OtA5097
Pcyt1a	chr16:32430920-32475065	OtA5088-OtA5092	OtA5093-OtA5097
Akr1c18	chr13:4132626-4150631	OtA5088-OtA5092	OtA5093-OtA5097
Clstn3	chr6:124430755-124464784	OtA5088-OtA5092	OtA5093-OtA5097
Fam13b	chr18:34409422-34506823	OtA5088-OtA5092	OtA5093-OtA5097
Fgfr3	chr5:33721723-33737068	OtA5088-OtA5092	OtA5093-OtA5097
Mipep	chr14:60784565-60905480	OtA5088-OtA5092	OtA5093-OtA5097
Mosc2	chr1:184813067-184845847	OtA5088-OtA5092	OtA5093-OtA5097
Mrpl11	chr19:4962305-4966995	OtA5088-OtA5092	OtA5093-OtA5097
Rpl15	chr14:18267822-18270986	OtA5088-OtA5092	OtA5093-OtA5097
Setd3	chr12:108106430-108179284	OtA5088-OtA5092	OtA5093-OtA5097
U2af1l4	chr7:30563339-30565364	OtA5088-OtA5092	OtA5093-OtA5097
Ogfod1	chr8:94037197-94067922	OtA5088-OtA5092	OtA5093-OtA5097
Nup98	chr7:102134202-102210120	OtA5088-OtA5092	OtA5093-OtA5097
Prkca	chr11:107933386-108343888	OtA5088-OtA5092	OtA5093-OtA5097
Tmem216	chr19:10550465-10556238	OtA5088-OtA5092	OtA5093-OtA5097
Vrk1	chr12:106010262-106077410	OtA5088-OtA5092	OtA5093-OtA5097
Zc3h4	chr7:16401195-16437696	OtA5088-OtA5092	OtA5093-OtA5097
Cog5	chr12:31654868-31950535	OtA5088-OtA5092	OtA5093-OtA5097
Cxx1b	chrX:53669176-53670408	OtA5088-OtA5092	OtA5093-OtA5097
Gtf2b	chr3:142765246-142783605	OtA5088-OtA5092	OtA5093-OtA5097
Rnf40	chr7:127588697-127603605	OtA5088-OtA5092	OtA5093-OtA5097
Tagln2	chr1:172500245-172507375	OtA5088-OtA5092	OtA5093-OtA5097
Ushbp1	chr8:71384273-71395801	OtA5088-OtA5092	OtA5093-OtA5097
Gipc2	chr3:152093840-152165900	OtA5088-OtA5092	OtA5093-OtA5097
Leprotl1	chr8:34135571-34146739	OtA5088-OtA5092	OtA5093-OtA5097
Mocs3	chr2:168230621-168232303	OtA5088-OtA5092	OtA5093-OtA5097
Nat6	chr9:107580169-107587359	OtA5088-OtA5092	OtA5093-OtA5097
Rpl27	chr11:101442244-101445596	OtA5088-OtA5092	OtA5093-OtA5097
Timm23	chr14:32180165-32201891	OtA5088-OtA5092	OtA5093-OtA5097
BC021614	chr19:4057486-4059294	OtA5088-OtA5092	OtA5093-OtA5097
Copg2	chr6:30809558-30896760	OtA5088-OtA5092	OtA5093-OtA5097
Nmi	chr2:51948498-51973208	OtA5088-OtA5092	OtA5093-OtA5097
Prom2	chr2:127526952-127541417	OtA5088-OtA5092	OtA5093-OtA5097
Ppp2ca	chr11:52098823-52122749	OtA5088-OtA5092	OtA5093-OtA5097
Oxct2a	chr4:123312649-123343252	OtA5088-OtA5092	OtA5093-OtA5097
Nek1	chr8:60993219-61130234	OtA5088-OtA5092	OtA5093-OtA5097
Emb	chr13:117220572-117274415	OtA5088-OtA5092	OtA5093-OtA5097
Tmem17	chr11:22512282-22519231	OtA5088-OtA5092	OtA5093-OtA5097
Hnrnpa3	chr2:75659258-75669407	OtA5088-OtA5092	OtA5093-OtA5097

Myof	chr19:37899035-38043577	OtA5088-OtA5092	OtA5093-OtA5097
Ppfia1	chr7:144476754-144553729	OtA5088-OtA5092	OtA5093-OtA5097
Rbm47	chr5:66016556-66151924	OtA5088-OtA5092	OtA5093-OtA5097
Rest	chr5:77265493-77283697	OtA5088-OtA5092	OtA5093-OtA5097
Sct	chr7:141278337-141279131	OtA5088-OtA5092	OtA5093-OtA5097
Spnb2	chr11:30099394-30219772	OtA5088-OtA5092	OtA5093-OtA5097
Ttc19	chr11:62281472-62457332	OtA5088-OtA5092	OtA5093-OtA5097
Uhrf1bp1	chr17:27856506-27900040	OtA5088-OtA5092	OtA5093-OtA5097
Zfp639	chr3:32510549-32520833	OtA5088-OtA5092	OtA5093-OtA5097
Acap2	chr16:30312423-31275697	OtA5088-OtA5092	OtA5093-OtA5097
Ahsg	chr16:22892041-22899438	OtA5088-OtA5092	OtA5093-OtA5097
Bahd1	chr2:118901614-118924524	OtA5088-OtA5092	OtA5093-OtA5097
Ccdc85c	chr12:108206344-108275417	OtA5088-OtA5092	OtA5093-OtA5097
Ccnk	chr12:108179737-108203359	OtA5088-OtA5092	OtA5093-OtA5097
Clcn6	chr4:148006483-148038767	OtA5088-OtA5092	OtA5093-OtA5097
Clec1b	chr6:129397296-129405413	OtA5088-OtA5092	OtA5093-OtA5097
Clk2	chr3:89164804-89177087	OtA5088-OtA5092	OtA5093-OtA5097
Dact1	chr12:71309883-71320107	OtA5088-OtA5092	OtA5093-OtA5097
Ddit3	chr10:127290792-127311786	OtA5088-OtA5092	OtA5093-OtA5097
Dsp	chr13:38151293-38198577	OtA5088-OtA5092	OtA5093-OtA5097
Dtx4	chr19:12466335-12501996	OtA5088-OtA5092	OtA5093-OtA5097
Exosc4	chr15:76327396-76330670	OtA5088-OtA5092	OtA5093-OtA5097
Fam19a5	chr15:87544298-87759364	OtA5088-OtA5092	OtA5093-OtA5097
Fxc1	chr7:105640539-105641845	OtA5088-OtA5092	OtA5093-OtA5097
Gdi1	chrX:74305011-74311867	OtA5088-OtA5092	OtA5093-OtA5097
Gm10052,Hnrnpa1	chr15:103240431-103244955	OtA5088-OtA5092	OtA5093-OtA5097
Gzmm	chr10:79689019-79695260	OtA5088-OtA5092	OtA5093-OtA5097
Hist1h4i	chr13:22040815-22041352	OtA5088-OtA5092	OtA5093-OtA5097
Icmt	chr4:152297213-152318625	OtA5088-OtA5092	OtA5093-OtA5097
Inppl1	chr7:101818312-101838226	OtA5088-OtA5092	OtA5093-OtA5097
Kdelr2	chr5:143403819-143421904	OtA5088-OtA5092	OtA5093-OtA5097
Lpcat2	chr8:92855349-92919279	OtA5088-OtA5092	OtA5093-OtA5097
Maged2	chrX:150806420-150814339	OtA5088-OtA5092	OtA5093-OtA5097
Mettl22	chr16:8470812-8490197	OtA5088-OtA5092	OtA5093-OtA5097
Mrpl19	chr6:81923668-81965949	OtA5088-OtA5092	OtA5093-OtA5097
Nup43	chr10:7667503-7678886	OtA5088-OtA5092	OtA5093-OtA5097
Parvb	chr15:84232042-84315609	OtA5088-OtA5092	OtA5093-OtA5097
Pcid2	chr8:13077524-13105343	OtA5088-OtA5092	OtA5093-OtA5097
Polr2c	chr8:94857449-94876312	OtA5088-OtA5092	OtA5093-OtA5097
Prl8a2	chr13:27345688-27354204	OtA5088-OtA5092	OtA5093-OtA5097
Psma4	chr9:54950256-54958030	OtA5088-OtA5092	OtA5093-OtA5097
Pts	chr9:50521616-50528641	OtA5088-OtA5092	OtA5093-OtA5097
Rhot1	chr11:80209054-80267907	OtA5088-OtA5092	OtA5093-OtA5097
Rps9	chr7:3704040-3706897	OtA5088-OtA5092	OtA5093-OtA5097
Siah1a	chr8:86723937-86747060	OtA5088-OtA5092	OtA5093-OtA5097
Snhg7	chr2:26637175-26640244	OtA5088-OtA5092	OtA5093-OtA5097
Ssrp1	chr2:85037200-85047111	OtA5088-OtA5092	OtA5093-OtA5097

Tbca	chr13:94788942-94842899	OtA5088-OtA5092	OtA5093-OtA5097
Tnfaip3	chr10:19000909-19015410	OtA5088-OtA5092	OtA5093-OtA5097
Usp1	chr4:98923809-98935533	OtA5088-OtA5092	OtA5093-OtA5097
Zfp775	chr6:48613179-48623227	OtA5088-OtA5092	OtA5093-OtA5097
Zfr2	chr10:81233162-81252123	OtA5088-OtA5092	OtA5093-OtA5097
Abat	chr16:8513428-8621567	OtA5088-OtA5092	OtA5093-OtA5097
Ddx31	chr2:28840405-28905575	OtA5088-OtA5092	OtA5093-OtA5097
Eid1	chr2:125627446-125724148	OtA5088-OtA5092	OtA5093-OtA5097
Gsg1l	chr7:125878418-126082411	OtA5088-OtA5092	OtA5093-OtA5097
Manbal	chr2:157367593-157396763	OtA5088-OtA5092	OtA5093-OtA5097
Mettl6	chr14:31478797-31494977	OtA5088-OtA5092	OtA5093-OtA5097
Nt5c3l	chr11:100415693-100441089	OtA5088-OtA5092	OtA5093-OtA5097
Pla2g12b	chr10:59403684-59421976	OtA5088-OtA5092	OtA5093-OtA5097
Sergef	chr7:46443158-46639807	OtA5088-OtA5092	OtA5093-OtA5097
Tcea2	chr2:181680309-181688051	OtA5088-OtA5092	OtA5093-OtA5097
Tet2	chr3:133463676-133544390	OtA5088-OtA5092	OtA5093-OtA5097
Tfdp1	chr8:13339673-13377702	OtA5088-OtA5092	OtA5093-OtA5097
Tmem136	chr9:43108652-43116570	OtA5088-OtA5092	OtA5093-OtA5097
Tmem198b	chr10:128790909-128804370	OtA5088-OtA5092	OtA5093-OtA5097
Tsr1	chr11:74898079-74925798	OtA5088-OtA5092	OtA5093-OtA5097
Ubr3	chr2:69897245-70024013	OtA5088-OtA5092	OtA5093-OtA5097
Zfp787	chr7:6131488-6155971	OtA5088-OtA5092	OtA5093-OtA5097
Tmem168	chr6:13580688-13608063	OtA5088-OtA5092	OtA5093-OtA5097
Car5a	chr8:121907832-121944912	OtA5088-OtA5092	OtA5093-OtA5097
Comt	chr16:18348273-18479073	OtA5088-OtA5092	OtA5093-OtA5097
Ctbp1	chr5:33247722-33275004	OtA5088-OtA5092	OtA5093-OtA5097
Dgke	chr11:89037581-89060748	OtA5088-OtA5092	OtA5093-OtA5097
Gpc4	chrX:52053017-52164923	OtA5088-OtA5092	OtA5093-OtA5097
Mfap2	chr4:141010423-141015984	OtA5088-OtA5092	OtA5093-OtA5097
Myh9	chr15:77760588-77842115	OtA5088-OtA5092	OtA5093-OtA5097
Nnmt	chr9:48591889-48605077	OtA5088-OtA5092	OtA5093-OtA5097
Polr1b	chr2:129100995-129126595	OtA5088-OtA5092	OtA5093-OtA5097
Psmd7	chr8:107580379-107588482	OtA5088-OtA5092	OtA5093-OtA5097
Srebf1	chr11:60105012-60220604	OtA5088-OtA5092	OtA5093-OtA5097
Tfpi2	chr6:3962594-3968354	OtA5088-OtA5092	OtA5093-OtA5097
Tmem220	chr11:67025153-67034790	OtA5088-OtA5092	OtA5093-OtA5097
Usp19	chr9:108490675-108502337	OtA5088-OtA5092	OtA5093-OtA5097
Usp21	chr1:171281952-171287961	OtA5088-OtA5092	OtA5093-OtA5097
Zfp706	chr15:36997026-37007402	OtA5088-OtA5092	OtA5093-OtA5097
Capn10	chr1:92933542-92950590	OtA5088-OtA5092	OtA5093-OtA5097
Enox2	chrX:49010521-49288242	OtA5088-OtA5092	OtA5093-OtA5097
Magohb	chr6:131284388-131293244	OtA5088-OtA5092	OtA5093-OtA5097
Myo19	chr11:84876312-84916356	OtA5088-OtA5092	OtA5093-OtA5097
Wiz	chr17:32354049-32389439	OtA5088-OtA5092	OtA5093-OtA5097
Prdm15	chr16:97791466-97851227	OtA5088-OtA5092	OtA5093-OtA5097
Raver2	chr4:101069037-101265282	OtA5088-OtA5092	OtA5093-OtA5097
Tmem55a	chr4:14864218-14915260	OtA5088-OtA5092	OtA5093-OtA5097

F2r	chr13:95601788-95618433	OtA5088-OtA5092	OtA5093-OtA5097
Calml3	chr13:3802892-3804318	OtA5088-OtA5092	OtA5093-OtA5097
Ccpg1	chr9:72985550-73039699	OtA5088-OtA5092	OtA5093-OtA5097
Slc22a21	chr11:53950823-53980027	OtA5088-OtA5092	OtA5093-OtA5097
Obfc1	chr19:47501047-47537020	OtA5088-OtA5092	OtA5093-OtA5097
U2af1	chr17:31647081-31658754	OtA5088-OtA5092	OtA5093-OtA5097
Rps26	chr10:128624528-128626506	OtA5088-OtA5092	OtA5093-OtA5097
Rpl14	chr9:120571516-120574653	OtA5088-OtA5092	OtA5093-OtA5097
Fbxo4	chr15:3963563-3979573	OtA5088-OtA5092	OtA5093-OtA5097
Gpr52	chr1:160219173-160792938	OtA5088-OtA5092	OtA5093-OtA5097
Rad17	chr13:100617163-100651059	OtA5088-OtA5092	OtA5093-OtA5097
Gm9	chrX:37208501-37211041	OtA5088-OtA5092	OtA5093-OtA5097
Zdhhc1	chr8:105472424-105496870	OtA5088-OtA5092	OtA5093-OtA5097
Mbd2	chr18:70568291-70626131	OtA5088-OtA5092	OtA5093-OtA5097
Arfrp1	chr2:181357689-181380793	OtA5088-OtA5092	OtA5093-OtA5097
Cstf3	chr2:104590483-104665425	OtA5088-OtA5092	OtA5093-OtA5097
Mapk1ip1	chr7:138835817-138846267	OtA5088-OtA5092	OtA5093-OtA5097
Nckipsd	chr9:108808379-108818366	OtA5088-OtA5092	OtA5093-OtA5097
Nrf1	chr6:30047987-30153458	OtA5088-OtA5092	OtA5093-OtA5097
Nudt19	chr7:35547184-35555928	OtA5088-OtA5092	OtA5093-OtA5097
Pwwp2a	chr11:43681997-43722633	OtA5088-OtA5092	OtA5093-OtA5097
Rpl7l1	chr17:46773906-46782656	OtA5088-OtA5092	OtA5093-OtA5097
Spin1	chr13:51100897-51152546	OtA5088-OtA5092	OtA5093-OtA5097
Usf1	chr1:171411680-171418742	OtA5088-OtA5092	OtA5093-OtA5097
Usp28	chr9:48985384-49042517	OtA5088-OtA5092	OtA5093-OtA5097
Zfp358	chr8:3493137-3497208	OtA5088-OtA5092	OtA5093-OtA5097
Gpatch4	chr3:88043107-88055992	OtA5088-OtA5092	OtA5093-OtA5097
Hddc2	chr10:31313404-31328086	OtA5088-OtA5092	OtA5093-OtA5097
Polr2i	chr7:30232073-30233387	OtA5088-OtA5092	OtA5093-OtA5097
Nr1h4	chr10:89454233-89533622	OtA5088-OtA5092	OtA5093-OtA5097
Fam198b	chr3:79885929-79946278	OtA5088-OtA5092	OtA5093-OtA5097
Pqlc1	chr18:80255244-80292724	OtA5088-OtA5092	OtA5093-OtA5097
Rnf166	chr8:122466146-122476064	OtA5088-OtA5092	OtA5093-OtA5097
Acer3	chr7:98213659-98309527	OtA5088-OtA5092	OtA5093-OtA5097
Atad3a	chr4:155740639-155761098	OtA5088-OtA5092	OtA5093-OtA5097
Atf4	chr15:80255183-80257541	OtA5088-OtA5092	OtA5093-OtA5097
BC021767	chr3:94660588-94667152	OtA5088-OtA5092	OtA5093-OtA5097
Bbs4	chr9:59321965-59353508	OtA5088-OtA5092	OtA5093-OtA5097
Cbx4	chr11:119077570-119086237	OtA5088-OtA5092	OtA5093-OtA5097
Col8a2	chr4:126286793-126314330	OtA5088-OtA5092	OtA5093-OtA5097
Cxcr4	chr1:128588198-128592299	OtA5088-OtA5092	OtA5093-OtA5097
Dbr1	chr9:99575798-99584343	OtA5088-OtA5092	OtA5093-OtA5097
Ddx18	chr1:121553834-121567980	OtA5088-OtA5092	OtA5093-OtA5097
Dusp12	chr1:170874187-170885540	OtA5088-OtA5092	OtA5093-OtA5097
E130309D02Rik	chr5:143301071-143315360	OtA5088-OtA5092	OtA5093-OtA5097
Epb4.1l5	chr1:119545032-119649000	OtA5088-OtA5092	OtA5093-OtA5097
Faim	chr9:98986372-99002019	OtA5088-OtA5092	OtA5093-OtA5097

Fam169b	chr7:68266338-68363092	OtA5088-OtA5092	OtA5093-OtA5097
Fam78b	chr1:167001416-167091302	OtA5088-OtA5092	OtA5093-OtA5097
Fbxo28	chr1:182313101-182341606	OtA5088-OtA5092	OtA5093-OtA5097
Heatr2	chr5:139150222-139186505	OtA5088-OtA5092	OtA5093-OtA5097
Hook1	chr4:95959710-96024274	OtA5088-OtA5092	OtA5093-OtA5097
Hps3	chr3:19957053-20035310	OtA5088-OtA5092	OtA5093-OtA5097
Ints2	chr11:86210682-86257568	OtA5088-OtA5092	OtA5093-OtA5097
Lage3	chrX:74352161-74353618	OtA5088-OtA5092	OtA5093-OtA5097
Nab1	chr1:52455848-52500448	OtA5088-OtA5092	OtA5093-OtA5097
Pcgf5	chr19:36379066-36456204	OtA5088-OtA5092	OtA5093-OtA5097
Ropn1l	chr15:31441209-31453689	OtA5088-OtA5092	OtA5093-OtA5097
Smtnl2	chr11:72390113-72411713	OtA5088-OtA5092	OtA5093-OtA5097
Snx25	chr8:46033260-46124146	OtA5088-OtA5092	OtA5093-OtA5097
Wbscr16	chr5:134148057-134176767	OtA5088-OtA5092	OtA5093-OtA5097
Zfp748	chr13:67538653-67553152	OtA5088-OtA5092	OtA5093-OtA5097
Clic4	chr4:135213969-135272760	OtA5088-OtA5092	OtA5093-OtA5097
Dnajb11	chr16:22857844-22879634	OtA5088-OtA5092	OtA5093-OtA5097
Nme3	chr17:24896499-24936977	OtA5088-OtA5092	OtA5093-OtA5097
H2-T22,H2-T9	chr17:36038408-36042702	OtA5088-OtA5092	OtA5093-OtA5097
Pgrmc1	chrX:36598224-36606079	OtA5088-OtA5092	OtA5093-OtA5097
Ybey	chr10:76449080-76515857	OtA5088-OtA5092	OtA5093-OtA5097
Apool	chrX:112311407-112372430	OtA5088-OtA5092	OtA5093-OtA5097
Eif3e	chr15:43250039-43282736	OtA5088-OtA5092	OtA5093-OtA5097
Lztr1	chr16:17508970-17526330	OtA5088-OtA5092	OtA5093-OtA5097
Rplp2-ps1	chr12:75630593-75669537	OtA5088-OtA5092	OtA5093-OtA5097
Zfp598	chr17:24669751-24682016	OtA5088-OtA5092	OtA5093-OtA5097
Zfp768	chr7:127342794-127345314	OtA5088-OtA5092	OtA5093-OtA5097
Pfn2	chr3:57841894-57847757	OtA5088-OtA5092	OtA5093-OtA5097
Pold1	chr7:44532743-44548815	OtA5088-OtA5092	OtA5093-OtA5097
Dcxr	chr11:120725372-120727281	OtA5088-OtA5092	OtA5093-OtA5097
Fbxo36	chr1:84839840-84900486	OtA5088-OtA5092	OtA5093-OtA5097
Hnrnpa2b1	chr6:51460434-51469894	OtA5088-OtA5092	OtA5093-OtA5097
Rnf11	chr4:109452856-109476505	OtA5088-OtA5092	OtA5093-OtA5097
Slc7a6os	chr8:106200437-106210933	OtA5088-OtA5092	OtA5093-OtA5097
Lcp1	chr14:75004109-75388146	OtA5088-OtA5092	OtA5093-OtA5097
Ncbp1	chr4:46138510-46172402	OtA5088-OtA5092	OtA5093-OtA5097
Plekhf2	chr4:10988661-11007619	OtA5088-OtA5092	OtA5093-OtA5097
Shisa2	chr14:59625280-59631660	OtA5088-OtA5092	OtA5093-OtA5097
Npc1	chr18:12168729-12236386	OtA5088-OtA5092	OtA5093-OtA5097
Hyal2	chr9:107569162-107572778	OtA5088-OtA5092	OtA5093-OtA5097
Cldn10	chr14:118787870-118874525	OtA5088-OtA5092	OtA5093-OtA5097
Gng5	chr3:146499835-146505543	OtA5088-OtA5092	OtA5093-OtA5097
Gphn	chr12:78226654-78684769	OtA5088-OtA5092	OtA5093-OtA5097
Gspt1	chr16:11203382-11254325	OtA5088-OtA5092	OtA5093-OtA5097
Il1r1	chr1:40225079-40316177	OtA5088-OtA5092	OtA5093-OtA5097
Snrpd3	chr10:75518041-75535440	OtA5088-OtA5092	OtA5093-OtA5097
Vcl	chr14:20929432-21033673	OtA5088-OtA5092	OtA5093-OtA5097

Zfp322a	chr13:23353103-23369202	OtA5088-OtA5092	OtA5093-OtA5097
Hcfc2	chr10:82699006-82741392	OtA5088-OtA5092	OtA5093-OtA5097
Ppapdc2	chr19:28923063-28966801	OtA5088-OtA5092	OtA5093-OtA5097
Gsto1	chr19:47854988-47864788	OtA5088-OtA5092	OtA5093-OtA5097
Med10	chr13:69809881-69816094	OtA5088-OtA5092	OtA5093-OtA5097
Pdp2	chr8:104446718-104624396	OtA5088-OtA5092	OtA5093-OtA5097
Tspan9	chr6:127961399-128143578	OtA5088-OtA5092	OtA5093-OtA5097
Sox21	chr14:118233233-118237030	OtA5088-OtA5092	OtA5093-OtA5097
Mboat7	chr7:3677788-3693525	OtA5088-OtA5092	OtA5093-OtA5097
Tmem66	chr8:34154562-34170847	OtA5088-OtA5092	OtA5093-OtA5097
Acad12	chr5:121598280-121618938	OtA5088-OtA5092	OtA5093-OtA5097
Prkag1	chr15:98812796-98831508	OtA5088-OtA5092	OtA5093-OtA5097
Pwwp2b	chr7:139248481-139267253	OtA5088-OtA5092	OtA5093-OtA5097
Tmem186	chr16:8633730-8637701	OtA5088-OtA5092	OtA5093-OtA5097
Tmem231	chr8:111912017-111933791	OtA5088-OtA5092	OtA5093-OtA5097
Zfp503	chr14:21983961-21989601	OtA5088-OtA5092	OtA5093-OtA5097
Gars	chr6:55038000-55079504	OtA5088-OtA5092	OtA5093-OtA5097
Llph	chr10:120227059-120232070	OtA5088-OtA5092	OtA5093-OtA5097
Nol11	chr11:107166660-107189381	OtA5088-OtA5092	OtA5093-OtA5097
Phf13	chr4:151989630-151996179	OtA5088-OtA5092	OtA5093-OtA5097
Prdm11	chr2:92974905-93046144	OtA5088-OtA5092	OtA5093-OtA5097
Reep4	chr14:70545250-70548935	OtA5088-OtA5092	OtA5093-OtA5097
Rnf14	chr18:38296634-38317849	OtA5088-OtA5092	OtA5093-OtA5097
Sepx1	chr17:24736641-24742778	OtA5088-OtA5092	OtA5093-OtA5097
Suv420h2	chr7:4740126-4747514	OtA5088-OtA5092	OtA5093-OtA5097
Tusc3	chr8:39005866-39130817	OtA5088-OtA5092	OtA5093-OtA5097
Zc3h10	chr10:128543564-128547744	OtA5088-OtA5092	OtA5093-OtA5097
Zcchc17	chr4:130316084-130359943	OtA5088-OtA5092	OtA5093-OtA5097
Acsf2	chr11:94557101-94601786	OtA5088-OtA5092	OtA5093-OtA5097
Cln8	chr8:14888535-14901719	OtA5088-OtA5092	OtA5093-OtA5097
Nck2	chr1:43445750-43570518	OtA5088-OtA5092	OtA5093-OtA5097
Tceb2	chr17:23803186-23829109	OtA5088-OtA5092	OtA5093-OtA5097
Angptl7	chr4:148448581-148557685	OtA5088-OtA5092	OtA5093-OtA5097
Ankrd40	chr11:94328000-94341847	OtA5088-OtA5092	OtA5093-OtA5097
Cerk	chr15:86139100-86186141	OtA5088-OtA5092	OtA5093-OtA5097
Dalrd3	chr9:108569891-108578670	OtA5088-OtA5092	OtA5093-OtA5097
Dnajb5	chr4:42953093-42958732	OtA5088-OtA5092	OtA5093-OtA5097
Fsd1l	chr4:53631470-53707009	OtA5088-OtA5092	OtA5093-OtA5097
Glis3	chr19:28262447-28720027	OtA5088-OtA5092	OtA5093-OtA5097
Gna14	chr19:16435666-16610818	OtA5088-OtA5092	OtA5093-OtA5097
Grhpr	chr4:44981410-44990696	OtA5088-OtA5092	OtA5093-OtA5097
Hace1	chr10:45577828-45712345	OtA5088-OtA5092	OtA5093-OtA5097
Hnrpll	chr17:80029486-80062334	OtA5088-OtA5092	OtA5093-OtA5097
Ifi35	chr11:101448411-101458701	OtA5088-OtA5092	OtA5093-OtA5097
Impa1	chr3:10313539-10331439	OtA5088-OtA5092	OtA5093-OtA5097
Map3k6	chr4:133240817-133252928	OtA5088-OtA5092	OtA5093-OtA5097
Mapk7	chr11:61488811-61494203	OtA5088-OtA5092	OtA5093-OtA5097

Mppe1	chr18:67088335-67245830	OtA5088-OtA5092	OtA5093-OtA5097
Mrpl17	chr7:105803781-105811087	OtA5088-OtA5092	OtA5093-OtA5097
Nav3	chr10:109683438-110000219	OtA5088-OtA5092	OtA5093-OtA5097
P4ha1	chr10:59323295-59373304	OtA5088-OtA5092	OtA5093-OtA5097
Paqr3	chr5:96997688-97111596	OtA5088-OtA5092	OtA5093-OtA5097
Pigk	chr3:152714099-152789013	OtA5088-OtA5092	OtA5093-OtA5097
Smu1	chr4:40722467-40757885	OtA5088-OtA5092	OtA5093-OtA5097
Tada2a	chr11:84078919-84129568	OtA5088-OtA5092	OtA5093-OtA5097
Ttc35	chr15:43477228-43527777	OtA5088-OtA5092	OtA5093-OtA5097
Uxt	chrX:20951664-20987349	OtA5088-OtA5092	OtA5093-OtA5097
Zbtb5	chr4:44991242-45012412	OtA5088-OtA5092	OtA5093-OtA5097
Gp9	chr6:87778135-87779762	OtA5088-OtA5092	OtA5093-OtA5097
Ap3m2	chr8:22787353-22805654	OtA5088-OtA5092	OtA5093-OtA5097
Crot	chr5:8966047-8997146	OtA5088-OtA5092	OtA5093-OtA5097
Fchsd2	chr7:101108774-101284405	OtA5088-OtA5092	OtA5093-OtA5097
Hist1h1c	chr13:23738806-23740367	OtA5088-OtA5092	OtA5093-OtA5097
Ifit3	chr19:34583528-34588982	OtA5088-OtA5092	OtA5093-OtA5097
Itfg2	chr6:128409443-128424910	OtA5088-OtA5092	OtA5093-OtA5097
Mrpl33	chr5:31613950-31622644	OtA5088-OtA5092	OtA5093-OtA5097
Nfia	chr4:97567874-98118876	OtA5088-OtA5092	OtA5093-OtA5097
P2rx7	chr5:122643910-122691432	OtA5088-OtA5092	OtA5093-OtA5097
Rassf8	chr6:145808382-145817584	OtA5088-OtA5092	OtA5093-OtA5097
Rpl23a	chr11:78180935-78183584	OtA5088-OtA5092	OtA5093-OtA5097
Sf3a3	chr4:124714860-124732422	OtA5088-OtA5092	OtA5093-OtA5097
Snx4	chr16:33251455-33299562	OtA5088-OtA5092	OtA5093-OtA5097
Zfp512	chr5:31452435-31481753	OtA5088-OtA5092	OtA5093-OtA5097
Eif1ax	chrX:159372194-159385699	OtA5088-OtA5092	OtA5093-OtA5097
Klhl18	chr9:110425925-110476694	OtA5088-OtA5092	OtA5093-OtA5097
Ube2f	chr1:91250318-91286025	OtA5088-OtA5092	OtA5093-OtA5097
Myo5b	chr18:74442618-74771477	OtA5088-OtA5092	OtA5093-OtA5097
Clcn4-2	chr7:7282315-7299511	OtA5088-OtA5092	OtA5093-OtA5097
Eftud1	chr7:82648613-82777852	OtA5088-OtA5092	OtA5093-OtA5097
Ilkap	chr1:91375830-91398783	OtA5088-OtA5092	OtA5093-OtA5097
Psen1	chr12:83688562-83735199	OtA5088-OtA5092	OtA5093-OtA5097
Fancc	chr13:63304708-63431745	OtA5088-OtA5092	OtA5093-OtA5097
Clasrp	chr7:19581042-19604468	OtA5088-OtA5092	OtA5093-OtA5097
Traf3ip2	chr10:39612933-39732007	OtA5088-OtA5092	OtA5093-OtA5097
Farp2	chr1:93512103-93635727	OtA5088-OtA5092	OtA5093-OtA5097
Rmi1	chr13:58391950-58411149	OtA5088-OtA5092	OtA5093-OtA5097
Asf1a	chr10:53537323-53630439	OtA5088-OtA5092	OtA5093-OtA5097
BC023829	chrX:70460055-70477043	OtA5088-OtA5092	OtA5093-OtA5097
Casc3	chr11:98795768-98833807	OtA5088-OtA5092	OtA5093-OtA5097
Cep76	chr18:67617396-67641336	OtA5088-OtA5092	OtA5093-OtA5097
Dctn6	chr8:34090422-34108511	OtA5088-OtA5092	OtA5093-OtA5097
Ing5	chr1:93803964-93822100	OtA5088-OtA5092	OtA5093-OtA5097
Itm2b	chr14:73362231-73385271	OtA5088-OtA5092	OtA5093-OtA5097
Kdm4a	chr4:118137003-118180043	OtA5088-OtA5092	OtA5093-OtA5097

Map1lc3b	chr8:121590467-121598047	OtA5088-OtA5092	OtA5093-OtA5097
Nthl1	chr17:24632681-24638838	OtA5088-OtA5092	OtA5093-OtA5097
Orc3	chr4:34550614-34614942	OtA5088-OtA5092	OtA5093-OtA5097
Pqbp1	chrX:7894518-7899269	OtA5088-OtA5092	OtA5093-OtA5097
Rfxank	chr8:70130805-70139197	OtA5088-OtA5092	OtA5093-OtA5097
Cfl1	chr19:5490454-5494031	OtA5088-OtA5092	OtA5093-OtA5097
Krtcap2	chr3:89246437-89249722	OtA5088-OtA5092	OtA5093-OtA5097
Rbl2	chr8:91070093-91135496	OtA5088-OtA5092	OtA5093-OtA5097
Gpsm1	chr2:26315532-26352110	OtA5088-OtA5092	OtA5093-OtA5097
Sertad3	chr7:27473839-27477364	OtA5088-OtA5092	OtA5093-OtA5097
Arid1b	chr17:4995073-5347656	OtA5088-OtA5092	OtA5093-OtA5097
Lias	chr5:65391496-65409207	OtA5088-OtA5092	OtA5093-OtA5097
Boc	chr16:44485044-44558870	OtA5088-OtA5092	OtA5093-OtA5097
Zfp169	chr13:48487659-48513436	OtA5088-OtA5092	OtA5093-OtA5097
Tmem167	chr13:90089666-90114921	OtA5088-OtA5092	OtA5093-OtA5097
Fam63a	chr3:95282934-95307176	OtA5088-OtA5092	OtA5093-OtA5097
Fkbp1a	chr2:151542498-151561691	OtA5088-OtA5092	OtA5093-OtA5097
Flywch1	chr17:23755422-23771591	OtA5088-OtA5092	OtA5093-OtA5097
Slc6a6	chr6:91684066-91759063	OtA5088-OtA5092	OtA5093-OtA5097
Spats2l	chr1:57774860-57948397	OtA5088-OtA5092	OtA5093-OtA5097
Tmem29	chrX:150397772-150459150	OtA5088-OtA5092	OtA5093-OtA5097
Alkbh5	chr11:60537682-60558512	OtA5088-OtA5092	OtA5093-OtA5097
Cacybp	chr1:160202366-160212892	OtA5088-OtA5092	OtA5093-OtA5097
Cwc25	chr11:97745469-97766613	OtA5088-OtA5092	OtA5093-OtA5097
Dctn4	chr18:60526220-60558762	OtA5088-OtA5092	OtA5093-OtA5097
Dnmt1	chr9:20907205-20959888	OtA5088-OtA5092	OtA5093-OtA5097
E130203B14Rik	chr14:32856755-32939489	OtA5088-OtA5092	OtA5093-OtA5097
Emd	chrX:74254838-74257747	OtA5088-OtA5092	OtA5093-OtA5097
Gpx8	chr13:113042762-113046388	OtA5088-OtA5092	OtA5093-OtA5097
Gstm5	chr3:107877229-107898685	OtA5088-OtA5092	OtA5093-OtA5097
Hdgfrp2	chr17:56079656-56109802	OtA5088-OtA5092	OtA5093-OtA5097
Mars	chr10:127290792-127311786	OtA5088-OtA5092	OtA5093-OtA5097
Mertk	chr2:128698996-128802188	OtA5088-OtA5092	OtA5093-OtA5097
Ppp1r11	chr17:36948354-36951792	OtA5088-OtA5092	OtA5093-OtA5097
Rap1b	chr10:117814596-117845974	OtA5088-OtA5092	OtA5093-OtA5097
Rbms2	chr10:128129469-128180297	OtA5088-OtA5092	OtA5093-OtA5097
Romo1	chr2:156123636-156145794	OtA5088-OtA5092	OtA5093-OtA5097
Sae1	chr7:16327053-16387785	OtA5088-OtA5092	OtA5093-OtA5097
Socs6	chr18:88867879-88894207	OtA5088-OtA5092	OtA5093-OtA5097
Stx4a	chr7:127841807-127848965	OtA5088-OtA5092	OtA5093-OtA5097
Tnfrsf1b	chr4:145212367-145246870	OtA5088-OtA5092	OtA5093-OtA5097
Utp18	chr11:93859242-93885766	OtA5088-OtA5092	OtA5093-OtA5097
Zfp1	chr8:111643442-111671011	OtA5088-OtA5092	OtA5093-OtA5097
Zfp691	chr4:119169517-119174195	OtA5088-OtA5092	OtA5093-OtA5097
Tcf21	chr10:22817274-22820128	OtA5088-OtA5092	OtA5093-OtA5097
Prr3	chr17:35972538-35979825	OtA5088-OtA5092	OtA5093-OtA5097
Snx27	chr3:94497543-94582702	OtA5088-OtA5092	OtA5093-OtA5097

BC020402	chr10:7678970-7681158	OtA5088-OtA5092	OtA5093-OtA5097
Bhlhe40	chr6:108577038-108666925	OtA5088-OtA5092	OtA5093-OtA5097
Ceacam13	chr7:18009888-18019220	OtA5088-OtA5092	OtA5093-OtA5097
Ceacam5	chr7:17713251-17761121	OtA5088-OtA5092	OtA5093-OtA5097
Clk3	chr9:57750710-57765860	OtA5088-OtA5092	OtA5093-OtA5097
Dolpp1	chr2:30392415-30415748	OtA5088-OtA5092	OtA5093-OtA5097
Ercc8	chr13:108158737-108194981	OtA5088-OtA5092	OtA5093-OtA5097
Fbl	chr7:28169747-28179269	OtA5088-OtA5092	OtA5093-OtA5097
Hibadh	chr6:52546229-52640300	OtA5088-OtA5092	OtA5093-OtA5097
Hnrnpr	chr4:136310975-136340678	OtA5088-OtA5092	OtA5093-OtA5097
Lima1	chr15:99778467-99875456	OtA5088-OtA5092	OtA5093-OtA5097
Lsm11	chr11:45928268-45944935	OtA5088-OtA5092	OtA5093-OtA5097
Med14	chrX:12675370-12761973	OtA5088-OtA5092	OtA5093-OtA5097
Mrps36	chr13:100735939-100744659	OtA5088-OtA5092	OtA5093-OtA5097
Myl3	chr9:110763680-110769794	OtA5088-OtA5092	OtA5093-OtA5097
Nenf	chr1:191306796-191318118	OtA5088-OtA5092	OtA5093-OtA5097
Nhp2	chr11:51619772-51635896	OtA5088-OtA5092	OtA5093-OtA5097
Noc3l	chr19:38788127-38819237	OtA5088-OtA5092	OtA5093-OtA5097
Ntan1	chr16:13819276-13903135	OtA5088-OtA5092	OtA5093-OtA5097
Nup188	chr2:30286432-30344262	OtA5088-OtA5092	OtA5093-OtA5097
Pdk2	chr11:95026257-95041371	OtA5088-OtA5092	OtA5093-OtA5097
Pgap3	chr11:98388671-98400490	OtA5088-OtA5092	OtA5093-OtA5097
Pik3cb	chr9:99038401-99140211	OtA5088-OtA5092	OtA5093-OtA5097
Prr24	chr7:16272012-16273692	OtA5088-OtA5092	OtA5093-OtA5097
Psme3	chr11:101316250-101323530	OtA5088-OtA5092	OtA5093-OtA5097
R3hcc1	chr14:69697303-69707580	OtA5088-OtA5092	OtA5093-OtA5097
Rps15a	chr7:118104375-118116147	OtA5088-OtA5092	OtA5093-OtA5097
Scand1	chr2:156311845-156375638	OtA5088-OtA5092	OtA5093-OtA5097
Serp1	chr3:58521970-58557501	OtA5088-OtA5092	OtA5093-OtA5097
Snhg5	chr9:88521052-88522897	OtA5088-OtA5092	OtA5093-OtA5097
Stip1	chr19:7020695-7040026	OtA5088-OtA5092	OtA5093-OtA5097
Suox	chr10:128669886-128673918	OtA5088-OtA5092	OtA5093-OtA5097
Tcf19	chr17:35512734-35516824	OtA5088-OtA5092	OtA5093-OtA5097
Tcp11l2	chr10:84576946-84614355	OtA5088-OtA5092	OtA5093-OtA5097
Ttpal	chr2:163602313-163619013	OtA5088-OtA5092	OtA5093-OtA5097
Zadh2	chr18:84088157-84097514	OtA5088-OtA5092	OtA5093-OtA5097
Zfand2a	chr5:139471215-139484491	OtA5088-OtA5092	OtA5093-OtA5097
Zswim6	chr13:107724616-107890064	OtA5088-OtA5092	OtA5093-OtA5097
Elmod1	chr9:53911459-53975301	OtA5088-OtA5092	OtA5093-OtA5097
Mycbp	chr4:123905012-123912250	OtA5088-OtA5092	OtA5093-OtA5097
Rtcd1	chr3:116488963-116508175	OtA5088-OtA5092	OtA5093-OtA5097
Cxadr	chr16:78301690-78359785	OtA5088-OtA5092	OtA5093-OtA5097
Eif4e2	chr1:87213913-87240488	OtA5088-OtA5092	OtA5093-OtA5097
Igtp	chr11:58199555-58207592	OtA5088-OtA5092	OtA5093-OtA5097
C1d	chr11:17257617-17269176	OtA5088-OtA5092	OtA5093-OtA5097
Diablo	chr5:123510459-123524164	OtA5088-OtA5092	OtA5093-OtA5097
Tubgcp4	chr2:121171205-121271386	OtA5088-OtA5092	OtA5093-OtA5097

Prrx1	chr1:163245118-163313650	OtA5088-OtA5092	OtA5093-OtA5097
Ap1s3	chr1:79606875-79671972	OtA5088-OtA5092	OtA5093-OtA5097
Fam65b	chr13:24614609-24733806	OtA5088-OtA5092	OtA5093-OtA5097
Mon1b	chr8:113635585-113655533	OtA5088-OtA5092	OtA5093-OtA5097
Rogdi	chr16:5008728-5013553	OtA5088-OtA5092	OtA5093-OtA5097
Scarna6	chr1:87756010-87792428	OtA5088-OtA5092	OtA5093-OtA5097
Stx8	chr11:67966482-68207148	OtA5088-OtA5092	OtA5093-OtA5097
Tubb3	chr8:123411563-123422010	OtA5088-OtA5092	OtA5093-OtA5097
Anapc13	chr9:102626295-102634244	OtA5088-OtA5092	OtA5093-OtA5097
Gm16675	chr8:46730969-46739515	OtA5088-OtA5092	OtA5093-OtA5097
Slc25a35	chr11:68968130-68973820	OtA5088-OtA5092	OtA5093-OtA5097
Bcas3	chr11:85353163-85826058	OtA5088-OtA5092	OtA5093-OtA5097
Arpc5	chr1:152766541-152775580	OtA5088-OtA5092	OtA5093-OtA5097
Bag5	chr12:111709489-111713256	OtA5088-OtA5092	OtA5093-OtA5097
Fbxo10	chr4:45034247-45084604	OtA5088-OtA5092	OtA5093-OtA5097
Glb1l	chr1:75192134-75210778	OtA5088-OtA5092	OtA5093-OtA5097
Lrrc61	chr6:48554798-48572670	OtA5088-OtA5092	OtA5093-OtA5097
Oaz1	chr10:80826655-80829290	OtA5088-OtA5092	OtA5093-OtA5097
Reck	chr4:43875529-43944806	OtA5088-OtA5092	OtA5093-OtA5097
Snapc1	chr12:73964529-73984820	OtA5088-OtA5092	OtA5093-OtA5097
Timd2	chr11:46668959-46707104	OtA5088-OtA5092	OtA5093-OtA5097
Timp1	chrX:20848542-20920918	OtA5088-OtA5092	OtA5093-OtA5097
Baiap2	chr11:119943091-120006782	OtA5088-OtA5092	OtA5093-OtA5097
Cdc42se1	chr3:95228779-95236409	OtA5088-OtA5092	OtA5093-OtA5097
Gldc	chr19:30098446-30175418	OtA5088-OtA5092	OtA5093-OtA5097
Gm7367	chr11:116434093-116439077	OtA5088-OtA5092	OtA5093-OtA5097
Pcdhgb7	chr18:36930284-37841872	OtA5088-OtA5092	OtA5093-OtA5097
Ptrf	chr11:100956735-100970617	OtA5088-OtA5092	OtA5093-OtA5097
Rexo1	chr10:80540925-80561560	OtA5088-OtA5092	OtA5093-OtA5097
Scyl1	chr19:5740903-5771401	OtA5088-OtA5092	OtA5093-OtA5097
Wbp2	chr11:116078572-116086964	OtA5088-OtA5092	OtA5093-OtA5097
Eef2	chr10:81176630-81182509	OtA5088-OtA5092	OtA5093-OtA5097
Gan	chr8:117158134-117205186	OtA5088-OtA5092	OtA5093-OtA5097
Stk40	chr4:126103956-126141029	OtA5088-OtA5092	OtA5093-OtA5097
Tceal8	chrX:136168983-136172251	OtA5088-OtA5092	OtA5093-OtA5097
Pja1	chrX:99465733-99470735	OtA5088-OtA5092	OtA5093-OtA5097
Itgav	chr2:83724396-83806916	OtA5088-OtA5092	OtA5093-OtA5097
Krt8	chr15:101996710-102004342	OtA5088-OtA5092	OtA5093-OtA5097
Olfml2b	chr1:170644531-170682789	OtA5088-OtA5092	OtA5093-OtA5097
Smox	chr2:131127411-131604824	OtA5088-OtA5092	OtA5093-OtA5097
Zfp628	chr7:4915216-4922003	OtA5088-OtA5092	OtA5093-OtA5097
Irs1	chr1:82233104-82291439	OtA5088-OtA5092	OtA5093-OtA5097
Slc25a17	chr15:81318920-81360765	OtA5088-OtA5092	OtA5093-OtA5097
Gm16517	chr10:79669409-79676126	OtA5088-OtA5092	OtA5093-OtA5097
Hgs	chr11:120467634-120483984	OtA5088-OtA5092	OtA5093-OtA5097
Nipal3	chr4:135448900-135494504	OtA5088-OtA5092	OtA5093-OtA5097
Ppap2a	chr13:112800791-112867680	OtA5088-OtA5092	OtA5093-OtA5097

Ppp2r5d	chr17:46681136-46705002	OtA5088-OtA5092	OtA5093-OtA5097
Ube2d2	chr18:35771558-35807172	OtA5088-OtA5092	OtA5093-OtA5097
Zfp516	chr18:82910878-83005314	OtA5088-OtA5092	OtA5093-OtA5097
Mcm7	chr5:138164588-138171862	OtA5088-OtA5092	OtA5093-OtA5097
Rcl1	chr19:29101374-29143843	OtA5088-OtA5092	OtA5093-OtA5097
Wdr19	chr5:65199695-65260415	OtA5088-OtA5092	OtA5093-OtA5097
Herpud2	chr9:25108129-25151781	OtA5088-OtA5092	OtA5093-OtA5097
Acd	chr8:105690905-105701095	OtA5088-OtA5092	OtA5093-OtA5097
Fam131a	chr16:20695056-20716636	OtA5088-OtA5092	OtA5093-OtA5097
Psmd4	chr3:95032704-95042545	OtA5088-OtA5092	OtA5093-OtA5097
Snrnp27	chr6:86675168-86684491	OtA5088-OtA5092	OtA5093-OtA5097
Tchp	chr5:114707778-114722327	OtA5088-OtA5092	OtA5093-OtA5097
Med24	chr11:98704590-98729435	OtA5088-OtA5092	OtA5093-OtA5097
Sf3a2	chr10:80798734-80804922	OtA5088-OtA5092	OtA5093-OtA5097
Ddx55	chr5:124552863-124569660	OtA5088-OtA5092	OtA5093-OtA5097
Aard	chr15:52040106-52045722	OtA5088-OtA5092	OtA5093-OtA5097
Ip6k1	chr9:108002647-108048782	OtA5088-OtA5092	OtA5093-OtA5097
Yeats2	chr16:20141062-20232573	OtA5088-OtA5092	OtA5093-OtA5097
Nudt9	chr5:104047010-104065367	OtA5088-OtA5092	OtA5093-OtA5097
Aaas	chr15:102338246-102350759	OtA5088-OtA5092	OtA5093-OtA5097
Ache	chr5:137288276-137294463	OtA5088-OtA5092	OtA5093-OtA5097
Acp6	chr3:97158776-97176576	OtA5088-OtA5092	OtA5093-OtA5097
Adcy7	chr8:88272402-88329962	OtA5088-OtA5092	OtA5093-OtA5097
Amigo2	chr15:97244073-97385691	OtA5088-OtA5092	OtA5093-OtA5097
Arl2bp	chr8:94666754-94674423	OtA5088-OtA5092	OtA5093-OtA5097
Arvcf	chr16:18348273-18479073	OtA5088-OtA5092	OtA5093-OtA5097
Atg16l2	chr7:101289615-101302088	OtA5088-OtA5092	OtA5093-OtA5097
BC049762	chr11:51253650-51262951	OtA5088-OtA5092	OtA5093-OtA5097
Bag3	chr7:128523582-128546979	OtA5088-OtA5092	OtA5093-OtA5097
Bbs5	chr2:69647254-69667569	OtA5088-OtA5092	OtA5093-OtA5097
Bccip	chr7:133709332-133776803	OtA5088-OtA5092	OtA5093-OtA5097
Bckdk	chr7:127904072-127909664	OtA5088-OtA5092	OtA5093-OtA5097
Bmp6	chr13:38345715-38499728	OtA5088-OtA5092	OtA5093-OtA5097
Cdk2ap1	chr5:124345438-124354628	OtA5088-OtA5092	OtA5093-OtA5097
Chchd5	chr2:129129699-129134111	OtA5088-OtA5092	OtA5093-OtA5097
Crbn	chr6:106769137-106800074	OtA5088-OtA5092	OtA5093-OtA5097
Ctf1	chr7:127712735-127718185	OtA5088-OtA5092	OtA5093-OtA5097
Cuedc1	chr11:88098057-88194140	OtA5088-OtA5092	OtA5093-OtA5097
D15Ertd621e	chr15:58415467-58457801	OtA5088-OtA5092	OtA5093-OtA5097
D17H6S53E	chr17:35126401-35128855	OtA5088-OtA5092	OtA5093-OtA5097
Dab1	chr4:104367559-104744842	OtA5088-OtA5092	OtA5093-OtA5097
Dll4	chr2:119321198-119335666	OtA5088-OtA5092	OtA5093-OtA5097
Epb4.1l1	chr2:156421051-156543214	OtA5088-OtA5092	OtA5093-OtA5097
Fadd	chr7:144578322-144582436	OtA5088-OtA5092	OtA5093-OtA5097
Fbxo31	chr8:121549442-121578806	OtA5088-OtA5092	OtA5093-OtA5097
Fn3krp	chr11:121421372-121430768	OtA5088-OtA5092	OtA5093-OtA5097
Frs2	chr10:117070126-117148474	OtA5088-OtA5092	OtA5093-OtA5097

G630016D24Rik	chr14:51984832-52020733	OtA5088-OtA5092	OtA5093-OtA5097
Gabpa	chr16:84827870-84863778	OtA5088-OtA5092	OtA5093-OtA5097
Gm20594	chr6:79818030-79818364	OtA5088-OtA5092	OtA5093-OtA5097
Gpx7	chr4:108400216-108406713	OtA5088-OtA5092	OtA5093-OtA5097
Hspa8	chr9:40801272-40805199	OtA5088-OtA5092	OtA5093-OtA5097
Ikbkg	chrX:74393290-74453778	OtA5088-OtA5092	OtA5093-OtA5097
Kcnc4	chr3:107438302-107458898	OtA5088-OtA5092	OtA5093-OtA5097
Klhl9	chr4:88718291-88722508	OtA5088-OtA5092	OtA5093-OtA5097
Lman1	chr18:65980738-66002635	OtA5088-OtA5092	OtA5093-OtA5097
Mfsd3	chr15:76701541-76710559	OtA5088-OtA5092	OtA5093-OtA5097
Mtpap	chr18:4375591-4397330	OtA5088-OtA5092	OtA5093-OtA5097
Myst4	chr14:21499769-21672478	OtA5088-OtA5092	OtA5093-OtA5097
Nrbf2	chr10:67266688-67285281	OtA5088-OtA5092	OtA5093-OtA5097
Nudt22	chr19:6993018-6996037	OtA5088-OtA5092	OtA5093-OtA5097
Pard3b	chr1:61638823-62642284	OtA5088-OtA5092	OtA5093-OtA5097
Ppp1r2	chr16:30312423-31275697	OtA5088-OtA5092	OtA5093-OtA5097
Prickle3	chrX:7658532-7671278	OtA5088-OtA5092	OtA5093-OtA5097
Ptpn6	chr6:124720717-124738709	OtA5088-OtA5092	OtA5093-OtA5097
Pxn	chr5:115506701-115555986	OtA5088-OtA5092	OtA5093-OtA5097
Rassf1	chr9:107551554-107562267	OtA5088-OtA5092	OtA5093-OtA5097
Rpl12	chr2:32961711-32964045	OtA5088-OtA5092	OtA5093-OtA5097
Rrm1	chr7:102441694-102469771	OtA5088-OtA5092	OtA5093-OtA5097
Scarna3a	chr1:159232325-159347580	OtA5088-OtA5092	OtA5093-OtA5097
Sel1l	chr12:91806042-91849157	OtA5088-OtA5092	OtA5093-OtA5097
Sema4c	chr1:36548641-36558381	OtA5088-OtA5092	OtA5093-OtA5097
Sike1	chr3:102995739-103003914	OtA5088-OtA5092	OtA5093-OtA5097
Snora44	chr4:132308677-132311024	OtA5088-OtA5092	OtA5093-OtA5097
Snx6	chr12:54746356-54795662	OtA5088-OtA5092	OtA5093-OtA5097
Txnrd2	chr16:18348273-18479073	OtA5088-OtA5092	OtA5093-OtA5097
Uaca	chr9:60794547-60880370	OtA5088-OtA5092	OtA5093-OtA5097
Uchl5	chr1:143777277-143807466	OtA5088-OtA5092	OtA5093-OtA5097
Zfp36l1	chr12:80107759-80113013	OtA5088-OtA5092	OtA5093-OtA5097
Ado	chr10:67544510-67548955	OtA5088-OtA5092	OtA5093-OtA5097
BC055324	chr1:163930063-163994781	OtA5088-OtA5092	OtA5093-OtA5097
Cdc42	chr4:137319695-137357759	OtA5088-OtA5092	OtA5093-OtA5097
Eapp	chr12:54673466-54695865	OtA5088-OtA5092	OtA5093-OtA5097
Fkbp10	chr11:100415693-100441089	OtA5088-OtA5092	OtA5093-OtA5097
Gm9766	chr10:53278080-53379851	OtA5088-OtA5092	OtA5093-OtA5097
H2-Q6	chr17:35424876-35428361	OtA5088-OtA5092	OtA5093-OtA5097
Hexim1	chr11:103116324-103119724	OtA5088-OtA5092	OtA5093-OtA5097
Mapk3	chr7:126759625-126765816	OtA5088-OtA5092	OtA5093-OtA5097
Nedd8	chr14:55662266-55671906	OtA5088-OtA5092	OtA5093-OtA5097
Osbp	chr19:11965843-11994114	OtA5088-OtA5092	OtA5093-OtA5097
Rrp12	chr19:41862850-41896153	OtA5088-OtA5092	OtA5093-OtA5097
Taf1	chrX:101532734-101601789	OtA5088-OtA5092	OtA5093-OtA5097
Tead3	chr17:28331672-28350805	OtA5088-OtA5092	OtA5093-OtA5097
Fam134c	chr11:101096321-101119843	OtA5088-OtA5092	OtA5093-OtA5097

Abcf1	chr17:35956818-35969750	OtA5088-OtA5092	OtA5093-OtA5097
Aimp1	chr3:132660497-132683879	OtA5088-OtA5092	OtA5093-OtA5097
Ano8	chr8:71469193-71486067	OtA5088-OtA5092	OtA5093-OtA5097
Arf5	chr6:28423639-28426499	OtA5088-OtA5092	OtA5093-OtA5097
Atg13	chr2:91674611-91710592	OtA5088-OtA5092	OtA5093-OtA5097
Baiap2l1	chr5:144244436-144358112	OtA5088-OtA5092	OtA5093-OtA5097
Cd2bp2	chr7:127191659-127196000	OtA5088-OtA5092	OtA5093-OtA5097
Cdk2	chr10:128697938-128705051	OtA5088-OtA5092	OtA5093-OtA5097
Csnk2a2	chr8:95446095-95488820	OtA5088-OtA5092	OtA5093-OtA5097
Dnajc19	chr3:34020078-34081326	OtA5088-OtA5092	OtA5093-OtA5097
Dupd1	chr14:21676571-21714576	OtA5088-OtA5092	OtA5093-OtA5097
Dync1li1	chr9:114688830-114723777	OtA5088-OtA5092	OtA5093-OtA5097
Epb4.1l4a	chr18:33796326-34007206	OtA5088-OtA5092	OtA5093-OtA5097
Fads6	chr11:115283365-115297546	OtA5088-OtA5092	OtA5093-OtA5097
Fhdc1	chr3:84442195-84480439	OtA5088-OtA5092	OtA5093-OtA5097
Fip1l1	chr5:74535481-74702903	OtA5088-OtA5092	OtA5093-OtA5097
Fuz	chr7:44896078-44929490	OtA5088-OtA5092	OtA5093-OtA5097
Gba2	chr4:43562633-43587487	OtA5088-OtA5092	OtA5093-OtA5097
Gmpr	chr13:45507468-45546381	OtA5088-OtA5092	OtA5093-OtA5097
Hcfc1	chrX:73942794-73966315	OtA5088-OtA5092	OtA5093-OtA5097
Ing4	chr6:125039847-125049264	OtA5088-OtA5092	OtA5093-OtA5097
Megf9	chr4:70431926-70534928	OtA5088-OtA5092	OtA5093-OtA5097
Notch3	chr17:32120892-32166852	OtA5088-OtA5092	OtA5093-OtA5097
Pomp	chr5:147860627-147875778	OtA5088-OtA5092	OtA5093-OtA5097
Rps6ka3	chrX:159256119-159363485	OtA5088-OtA5092	OtA5093-OtA5097
Rps7	chr12:28630846-28635953	OtA5088-OtA5092	OtA5093-OtA5097
Samd10	chr2:181595217-181599147	OtA5088-OtA5092	OtA5093-OtA5097
Slc25a43	chrX:36743658-36777202	OtA5088-OtA5092	OtA5093-OtA5097
Smyd4	chr11:75348432-75405705	OtA5088-OtA5092	OtA5093-OtA5097
Sowahc	chr10:59221921-59226433	OtA5088-OtA5092	OtA5093-OtA5097
Trak1	chr9:121366957-121474918	OtA5088-OtA5092	OtA5093-OtA5097
Ube2w	chr1:16569405-16619286	OtA5088-OtA5092	OtA5093-OtA5097
Wash	chr17:66111545-66120503	OtA5088-OtA5092	OtA5093-OtA5097
X99384	chr10:61319656-61383523	OtA5088-OtA5092	OtA5093-OtA5097
Zfp260	chr7:30095075-30107614	OtA5088-OtA5092	OtA5093-OtA5097
Mrps35	chr6:147042769-147070902	OtA5088-OtA5092	OtA5093-OtA5097
Exoc6	chr19:37550417-37683249	OtA5088-OtA5092	OtA5093-OtA5097
Rgl1	chr1:152517529-152625111	OtA5088-OtA5092	OtA5093-OtA5097
Alkbh1	chr12:87428077-87472299	OtA5088-OtA5092	OtA5093-OtA5097
B230120H23Rik	chr2:72285636-72442610	OtA5088-OtA5092	OtA5093-OtA5097
C1qtnf1	chr11:118428456-118454995	OtA5088-OtA5092	OtA5093-OtA5097
Chst15	chr7:132236254-132318291	OtA5088-OtA5092	OtA5093-OtA5097
Coq4	chr2:29788262-29797743	OtA5088-OtA5092	OtA5093-OtA5097
Cox7a2l	chr17:83501916-83514333	OtA5088-OtA5092	OtA5093-OtA5097
D17Wsu104e	chr17:56176540-56183920	OtA5088-OtA5092	OtA5093-OtA5097
Esrra	chr19:6909697-6921808	OtA5088-OtA5092	OtA5093-OtA5097
Farsb	chr1:78424835-78488846	OtA5088-OtA5092	OtA5093-OtA5097

Fli1	chr9:32422207-32541368	OtA5088-OtA5092	OtA5093-OtA5097
Hsd17b4	chr18:50128200-50196270	OtA5088-OtA5092	OtA5093-OtA5097
Hspa12b	chr2:131127411-131604824	OtA5088-OtA5092	OtA5093-OtA5097
Mapk11	chr15:89142483-89149606	OtA5088-OtA5092	OtA5093-OtA5097
N4bp2l1	chr5:150571642-150594525	OtA5088-OtA5092	OtA5093-OtA5097
Ncald	chr15:37366174-37792428	OtA5088-OtA5092	OtA5093-OtA5097
Ndrg4	chr8:95703036-95715119	OtA5088-OtA5092	OtA5093-OtA5097
Praf2	chrX:7728570-7731063	OtA5088-OtA5092	OtA5093-OtA5097
Rbx1	chr15:81466315-81476369	OtA5088-OtA5092	OtA5093-OtA5097
Reep5	chr18:34344885-34373415	OtA5088-OtA5092	OtA5093-OtA5097
Rpl37a	chr1:72711259-72713813	OtA5088-OtA5092	OtA5093-OtA5097
Shisa5	chr9:109038626-109057766	OtA5088-OtA5092	OtA5093-OtA5097
Slc38a3	chr9:107651154-107668968	OtA5088-OtA5092	OtA5093-OtA5097
Tmem40	chr6:115729136-115762466	OtA5088-OtA5092	OtA5093-OtA5097
Tnfaip1	chr11:78522849-78536260	OtA5088-OtA5092	OtA5093-OtA5097
Trim8	chr19:46501647-46516455	OtA5088-OtA5092	OtA5093-OtA5097
Zfp783	chr6:47943174-47965299	OtA5088-OtA5092	OtA5093-OtA5097
Zyx	chr6:42349827-42358395	OtA5088-OtA5092	OtA5093-OtA5097
Aurkaip1	chr4:155831268-155833098	OtA5088-OtA5092	OtA5093-OtA5097
Elac2	chr11:64979034-65162961	OtA5088-OtA5092	OtA5093-OtA5097
Mtf2	chr5:108065673-108109219	OtA5088-OtA5092	OtA5093-OtA5097
Otub1	chr19:7198205-7206284	OtA5088-OtA5092	OtA5093-OtA5097
Prl7a1	chr13:27633363-27642493	OtA5088-OtA5092	OtA5093-OtA5097
Rab3gap1	chr1:127868772-127943876	OtA5088-OtA5092	OtA5093-OtA5097
Snhg1	chr19:8723486-8726326	OtA5088-OtA5092	OtA5093-OtA5097
Wwp2	chr8:107436397-107558595	OtA5088-OtA5092	OtA5093-OtA5097
Ldhb	chr6:142490248-142507943	OtA5088-OtA5092	OtA5093-OtA5097
Acp1	chr12:30884323-30911612	OtA5088-OtA5092	OtA5093-OtA5097
Bpgm	chr6:34476355-34505610	OtA5088-OtA5092	OtA5093-OtA5097
Ccdc132	chr6:3498392-3603531	OtA5088-OtA5092	OtA5093-OtA5097
Cyb5d2	chr11:72777231-72795839	OtA5088-OtA5092	OtA5093-OtA5097
Fiz1	chr7:5007055-5014728	OtA5088-OtA5092	OtA5093-OtA5097
Frs3	chr17:47695206-47704286	OtA5088-OtA5092	OtA5093-OtA5097
Gabarap	chr11:69991369-69994949	OtA5088-OtA5092	OtA5093-OtA5097
Kdelc1	chr1:44106545-44118773	OtA5088-OtA5092	OtA5093-OtA5097
Rad21	chr15:51962603-51991760	OtA5088-OtA5092	OtA5093-OtA5097
Twist2	chr1:91801476-91848027	OtA5088-OtA5092	OtA5093-OtA5097
Zfp12	chr5:143235207-143247970	OtA5088-OtA5092	OtA5093-OtA5097
Zfp383	chr7:29908516-29916813	OtA5088-OtA5092	OtA5093-OtA5097
Nid1	chr13:13437601-13512275	OtA5088-OtA5092	OtA5093-OtA5097
Pfdn1	chr18:36403678-36454495	OtA5088-OtA5092	OtA5093-OtA5097
Nek9	chr12:85299513-85339362	OtA5088-OtA5092	OtA5093-OtA5097
Cab39	chr1:85793446-85851577	OtA5088-OtA5092	OtA5093-OtA5097
Chchd4	chr6:91464275-91473423	OtA5088-OtA5092	OtA5093-OtA5097
D030056L22Rik	chr19:18713235-18718428	OtA5088-OtA5092	OtA5093-OtA5097
Gsk3a	chr7:25228258-25237851	OtA5088-OtA5092	OtA5093-OtA5097
Mycn	chr12:12936092-12941836	OtA5088-OtA5092	OtA5093-OtA5097

Nat9	chr11:115182831-115187316	OtA5088-OtA5092	OtA5093-OtA5097
Ogdhl	chr14:32322018-32347820	OtA5088-OtA5092	OtA5093-OtA5097
Slc35a1	chr4:34663256-34687438	OtA5088-OtA5092	OtA5093-OtA5097
Ube2v1	chr2:167607638-167632005	OtA5088-OtA5092	OtA5093-OtA5097
Upp1	chr11:9118007-9136170	OtA5088-OtA5092	OtA5093-OtA5097
Vezt	chr10:93968099-94035748	OtA5088-OtA5092	OtA5093-OtA5097
Zdhhc23	chr16:43969145-43979050	OtA5088-OtA5092	OtA5093-OtA5097
Thop1	chr10:81070082-81082360	OtA5088-OtA5092	OtA5093-OtA5097
Prr13	chr15:102459169-102462806	OtA5088-OtA5092	OtA5093-OtA5097
Zdhhc13	chr7:48789002-48827437	OtA5088-OtA5092	OtA5093-OtA5097
Spcs2	chr7:99837568-99858883	OtA5088-OtA5092	OtA5093-OtA5097
Acaca	chr11:84195437-84401651	OtA5088-OtA5092	OtA5093-OtA5097
Gyg	chr3:20122083-20155096	OtA5088-OtA5092	OtA5093-OtA5097
Zfp784	chr7:5034445-5038446	OtA5088-OtA5092	OtA5093-OtA5097
Phtf1	chr3:103914119-104007491	OtA5088-OtA5092	OtA5093-OtA5097
Nsun3	chr16:62734851-62786716	OtA5088-OtA5092	OtA5093-OtA5097
Topbp1	chr9:103305326-103350427	OtA5088-OtA5092	OtA5093-OtA5097
Gab1	chr8:80764433-80880479	OtA5088-OtA5092	OtA5093-OtA5097
Abhd5	chr9:122351615-122381523	OtA5088-OtA5092	OtA5093-OtA5097
Kif21a	chr15:90933275-91049948	OtA5088-OtA5092	OtA5093-OtA5097
Lsm1	chr8:25785590-25803975	OtA5088-OtA5092	OtA5093-OtA5097
Sertad2	chr11:20543252-20653023	OtA5088-OtA5092	OtA5093-OtA5097
Tmem38a	chr8:72572102-72587284	OtA5088-OtA5092	OtA5093-OtA5097
Xylb	chr9:119357380-119393797	OtA5088-OtA5092	OtA5093-OtA5097
Zfp954	chr7:7114682-7121476	OtA5088-OtA5092	OtA5093-OtA5097
Ano10	chr9:122175878-122294374	OtA5088-OtA5092	OtA5093-OtA5097
Ak1	chr2:32621757-32635058	OtA5088-OtA5092	OtA5093-OtA5097
Arhgap26	chr18:38993144-39376285	OtA5088-OtA5092	OtA5093-OtA5097
Blzf1	chr1:164289799-164307484	OtA5088-OtA5092	OtA5093-OtA5097
C030044B11Rik	chr13:48968111-48969905	OtA5088-OtA5092	OtA5093-OtA5097
Calhm2	chr19:47132231-47138294	OtA5088-OtA5092	OtA5093-OtA5097
Chchd6	chr6:89383145-89595652	OtA5088-OtA5092	OtA5093-OtA5097
Ctnnbip1	chr4:149518240-149566437	OtA5088-OtA5092	OtA5093-OtA5097
Elmo3	chr8:105305600-105310623	OtA5088-OtA5092	OtA5093-OtA5097
Fermt2	chr14:45458791-45530065	OtA5088-OtA5092	OtA5093-OtA5097
Hif1an	chr19:44562853-44576274	OtA5088-OtA5092	OtA5093-OtA5097
Igfbp4	chr11:99041259-99052643	OtA5088-OtA5092	OtA5093-OtA5097
Muted	chr13:38602705-38635109	OtA5088-OtA5092	OtA5093-OtA5097
Ncam1	chr9:49502145-49799069	OtA5088-OtA5092	OtA5093-OtA5097
Parm1	chr5:91517699-91623996	OtA5088-OtA5092	OtA5093-OtA5097
Pcyt2	chr11:120610086-120617890	OtA5088-OtA5092	OtA5093-OtA5097
Pla2g4a	chr1:149829621-149961284	OtA5088-OtA5092	OtA5093-OtA5097
Ppp1r15b	chr1:133131165-133139800	OtA5088-OtA5092	OtA5093-OtA5097
Ptprm	chr17:66666847-67354459	OtA5088-OtA5092	OtA5093-OtA5097
Rabl3	chr16:37539893-37572385	OtA5088-OtA5092	OtA5093-OtA5097
Rora	chr9:68653801-69379028	OtA5088-OtA5092	OtA5093-OtA5097
Slc36a1	chr11:55204339-55236330	OtA5088-OtA5092	OtA5093-OtA5097

Spna2	chr2:29965559-30031450	OtA5088-OtA5092	OtA5093-OtA5097
Tm4sf5	chr11:70505273-70511183	OtA5088-OtA5092	OtA5093-OtA5097
Tmsb15a	chrX:135718666-135720673	OtA5088-OtA5092	OtA5093-OtA5097
Trit1	chr4:123016596-123054934	OtA5088-OtA5092	OtA5093-OtA5097
Zbtb7a	chr10:81136270-81151657	OtA5088-OtA5092	OtA5093-OtA5097
Zhx3	chr2:160731309-160872990	OtA5088-OtA5092	OtA5093-OtA5097
Mknk1	chr4:115839245-115879248	OtA5088-OtA5092	OtA5093-OtA5097
Chst8	chr7:34674467-34812711	OtA5088-OtA5092	OtA5093-OtA5097
Ctps	chr4:120539867-120570276	OtA5088-OtA5092	OtA5093-OtA5097
Evi5	chr5:107744794-107875107	OtA5088-OtA5092	OtA5093-OtA5097
Pdxdc1	chr16:13819276-13903135	OtA5088-OtA5092	OtA5093-OtA5097
Eif2s1	chr12:78862071-78887010	OtA5088-OtA5092	OtA5093-OtA5097
Gm10033	chr8:69371024-69395544	OtA5088-OtA5092	OtA5093-OtA5097
Irgq	chr7:24530647-24538600	OtA5088-OtA5092	OtA5093-OtA5097
Lasp1	chr11:97799671-97838764	OtA5088-OtA5092	OtA5093-OtA5097
Leng1	chr7:3645268-3677553	OtA5088-OtA5092	OtA5093-OtA5097
Sec61b	chr4:47474660-47483233	OtA5088-OtA5092	OtA5093-OtA5097
Pigh	chr12:79029162-79089670	OtA5088-OtA5092	OtA5093-OtA5097
Dot1l	chr10:80755205-80794347	OtA5088-OtA5092	OtA5093-OtA5097
Snhg10	chr12:105030616-105032279	OtA5088-OtA5092	OtA5093-OtA5097
Arpc2	chr1:74236549-74268213	OtA5088-OtA5092	OtA5093-OtA5097
Mus81	chr19:5482839-5488336	OtA5088-OtA5092	OtA5093-OtA5097
Pgm1	chr5:64092949-64128158	OtA5088-OtA5092	OtA5093-OtA5097
Ap4m1	chr5:138172020-138187186	OtA5088-OtA5092	OtA5093-OtA5097
Arf2	chr11:103966870-103985336	OtA5088-OtA5092	OtA5093-OtA5097
Cntrob	chr11:69299495-69323873	OtA5088-OtA5092	OtA5093-OtA5097
H2afv	chr11:6427225-6444443	OtA5088-OtA5092	OtA5093-OtA5097
Map3k8	chr18:4331326-4352953	OtA5088-OtA5092	OtA5093-OtA5097
Nap1l1	chr10:111473191-111498150	OtA5088-OtA5092	OtA5093-OtA5097
Pacrgl	chr5:48372391-48388756	OtA5088-OtA5092	OtA5093-OtA5097
Pdgfc	chr3:81036415-81214031	OtA5088-OtA5092	OtA5093-OtA5097
Rad51l1	chr12:79297281-79814690	OtA5088-OtA5092	OtA5093-OtA5097
Rpl18	chr7:45718070-45720835	OtA5088-OtA5092	OtA5093-OtA5097
Sec11c	chr18:65800577-65817657	OtA5088-OtA5092	OtA5093-OtA5097
8-Sep	chr11:53519735-53544096	OtA5088-OtA5092	OtA5093-OtA5097
Slc37a3	chr6:39334770-39377707	OtA5088-OtA5092	OtA5093-OtA5097
Tmem80	chr7:141297175-141338783	OtA5088-OtA5092	OtA5093-OtA5097
Tor1aip1	chr1:156004598-156068859	OtA5088-OtA5092	OtA5093-OtA5097
Agps	chr2:75832176-75931350	OtA5088-OtA5092	OtA5093-OtA5097
Bcl3	chr7:19808461-19822755	OtA5088-OtA5092	OtA5093-OtA5097
C77080	chr4:129189794-129248443	OtA5088-OtA5092	OtA5093-OtA5097
Dstn	chr2:143915330-143943324	OtA5088-OtA5092	OtA5093-OtA5097
Map3k12	chr15:102470631-102517004	OtA5088-OtA5092	OtA5093-OtA5097
Nsmce1	chr7:125467639-125491542	OtA5088-OtA5092	OtA5093-OtA5097
Pgs1	chr11:117986856-118129219	OtA5088-OtA5092	OtA5093-OtA5097
Rabl5	chr5:136908149-136913244	OtA5088-OtA5092	OtA5093-OtA5097
Rbpms2	chr9:65630581-65660518	OtA5088-OtA5092	OtA5093-OtA5097

Rpl19	chr11:98026709-98030493	OtA5088-OtA5092	OtA5093-OtA5097
Rps6kb2	chr19:4156976-4163245	OtA5088-OtA5092	OtA5093-OtA5097
Slc17a5	chr9:78536508-78588027	OtA5088-OtA5092	OtA5093-OtA5097
Slc35e2	chr4:155601415-155624755	OtA5088-OtA5092	OtA5093-OtA5097
Ipo5	chr14:120911193-120948044	OtA5088-OtA5092	OtA5093-OtA5097
Uqcrb	chr13:66900620-66905350	OtA5088-OtA5092	OtA5093-OtA5097
Acadsb	chr7:131410600-131446211	OtA5088-OtA5092	OtA5093-OtA5097
D830031N03Rik	chr4:123349632-123684360	OtA5088-OtA5092	OtA5093-OtA5097
Abhd8	chr8:71456699-71463657	OtA5088-OtA5092	OtA5093-OtA5097
Actr5	chr2:158624912-158639211	OtA5088-OtA5092	OtA5093-OtA5097
Ankrd10	chr8:11611582-11635754	OtA5088-OtA5092	OtA5093-OtA5097
Arf1	chr11:59211411-59228267	OtA5088-OtA5092	OtA5093-OtA5097
Arhgdib	chr6:136923708-136941717	OtA5088-OtA5092	OtA5093-OtA5097
Atg4a	chr3:103643952-103646068	OtA5088-OtA5092	OtA5093-OtA5097
Atp1b1	chr1:164307674-164458355	OtA5088-OtA5092	OtA5093-OtA5097
Atpaf1	chr4:115784813-115812314	OtA5088-OtA5092	OtA5093-OtA5097
Atxn2	chr5:121711608-121814950	OtA5088-OtA5092	OtA5093-OtA5097
B3gnt9-ps	chr8:105225187-105255151	OtA5088-OtA5092	OtA5093-OtA5097
Bad	chr19:6938069-6951893	OtA5088-OtA5092	OtA5093-OtA5097
Beta-s	chr7:103826522-103827928	OtA5088-OtA5092	OtA5093-OtA5097
Brd1	chr15:88687034-88734219	OtA5088-OtA5092	OtA5093-OtA5097
Brwd3	chrX:108742207-108834355	OtA5088-OtA5092	OtA5093-OtA5097
Ccdc123	chr7:35397092-35438684	OtA5088-OtA5092	OtA5093-OtA5097
Cdca7	chr2:72476218-72486890	OtA5088-OtA5092	OtA5093-OtA5097
Chmp1b	chr18:67088335-67245830	OtA5088-OtA5092	OtA5093-OtA5097
Chst1	chr2:92599706-92615252	OtA5088-OtA5092	OtA5093-OtA5097
Cnpy4	chr5:138187534-138193894	OtA5088-OtA5092	OtA5093-OtA5097
Coq7	chr7:118525061-118533313	OtA5088-OtA5092	OtA5093-OtA5097
Creb3l2	chr6:37331020-37442148	OtA5088-OtA5092	OtA5093-OtA5097
Csnk1g2	chr10:80622779-80640771	OtA5088-OtA5092	OtA5093-OtA5097
Ctsf	chr19:4855128-4860912	OtA5088-OtA5092	OtA5093-OtA5097
D11Wsu99e	chr11:113649528-113684151	OtA5088-OtA5092	OtA5093-OtA5097
Ddx58	chr4:40203776-40239825	OtA5088-OtA5092	OtA5093-OtA5097
Edem1	chr6:108828640-108859356	OtA5088-OtA5092	OtA5093-OtA5097
Edf1	chr2:25557899-25562082	OtA5088-OtA5092	OtA5093-OtA5097
Fam122a	chr19:24294795-24555827	OtA5088-OtA5092	OtA5093-OtA5097
Fastkd5	chr2:130589995-130630038	OtA5088-OtA5092	OtA5093-OtA5097
Fhl3	chr4:124700698-124708611	OtA5088-OtA5092	OtA5093-OtA5097
Fis1	chr5:136953274-136966234	OtA5088-OtA5092	OtA5093-OtA5097
Fndc3b	chr3:27416161-27710439	OtA5088-OtA5092	OtA5093-OtA5097
Gabpb1	chr2:126628912-126675487	OtA5088-OtA5092	OtA5093-OtA5097
Gcc1	chr6:28416602-28421724	OtA5088-OtA5092	OtA5093-OtA5097
Gem	chr4:11704446-11714993	OtA5088-OtA5092	OtA5093-OtA5097
Gfpt2	chr11:49794154-49838620	OtA5088-OtA5092	OtA5093-OtA5097
Hgd	chr16:37580152-37632026	OtA5088-OtA5092	OtA5093-OtA5097
Hoxa11as	chr6:52242105-52249769	OtA5088-OtA5092	OtA5093-OtA5097
Ifitm1	chr7:140967428-140969827	OtA5088-OtA5092	OtA5093-OtA5097

Ighmbp2	chr19:3259075-3283010	OtA5088-OtA5092	OtA5093-OtA5097
Ipo8	chr6:148770683-148831467	OtA5088-OtA5092	OtA5093-OtA5097
Iqgap3	chr3:88082050-88121048	OtA5088-OtA5092	OtA5093-OtA5097
Isg20l2	chr3:87930313-87940686	OtA5088-OtA5092	OtA5093-OtA5097
Klc3	chr7:19382038-19399856	OtA5088-OtA5092	OtA5093-OtA5097
Ldlr	chr9:21723575-21749918	OtA5088-OtA5092	OtA5093-OtA5097
Leo1	chr9:75441523-75466432	OtA5088-OtA5092	OtA5093-OtA5097
Lhpp	chr7:132610642-132706419	OtA5088-OtA5092	OtA5093-OtA5097
Lrrc29	chr8:105312339-105326276	OtA5088-OtA5092	OtA5093-OtA5097
Mboat2	chr12:24831598-24960299	OtA5088-OtA5092	OtA5093-OtA5097
Mgat1	chr11:49244190-49263024	OtA5088-OtA5092	OtA5093-OtA5097
Mgst1	chr6:138140536-138156752	OtA5088-OtA5092	OtA5093-OtA5097
Mrps12	chr7:28739640-28741781	OtA5088-OtA5092	OtA5093-OtA5097
Myh14	chr7:44605819-44665503	OtA5088-OtA5092	OtA5093-OtA5097
Naa40	chr19:7225667-7241222	OtA5088-OtA5092	OtA5093-OtA5097
Neurl2	chr2:164830729-164857708	OtA5088-OtA5092	OtA5093-OtA5097
Nfe2l3	chr6:51432669-51458768	OtA5088-OtA5092	OtA5093-OtA5097
Nupl1	chr14:60219467-60251378	OtA5088-OtA5092	OtA5093-OtA5097
Ocln	chr13:100497366-100552498	OtA5088-OtA5092	OtA5093-OtA5097
Pam16	chr16:4616465-4624946	OtA5088-OtA5092	OtA5093-OtA5097
Phpt1	chr2:25573430-25574871	OtA5088-OtA5092	OtA5093-OtA5097
Polr2k	chr15:36174016-36177012	OtA5088-OtA5092	OtA5093-OtA5097
Ppm1g	chr5:31202667-31220545	OtA5088-OtA5092	OtA5093-OtA5097
Pramef8	chr4:143412425-143421087	OtA5088-OtA5092	OtA5093-OtA5097
Pspc1	chr14:56722448-56778316	OtA5088-OtA5092	OtA5093-OtA5097
Ptrh2	chr11:86683982-86692457	OtA5088-OtA5092	OtA5093-OtA5097
Rbbp8	chr18:11633275-11743207	OtA5088-OtA5092	OtA5093-OtA5097
Rbm4	chr19:4784292-4793877	OtA5088-OtA5092	OtA5093-OtA5097
Reep6	chr10:79697304-80369637	OtA5088-OtA5092	OtA5093-OtA5097
Rfk	chr19:17394042-17401349	OtA5088-OtA5092	OtA5093-OtA5097
Rnf113a2	chr12:84409067-84450950	OtA5088-OtA5092	OtA5093-OtA5097
Rnft2	chr5:118190735-118245025	OtA5088-OtA5092	OtA5093-OtA5097
Sap30l	chr11:57801636-57810615	OtA5088-OtA5092	OtA5093-OtA5097
Serpinb6a	chr13:33917917-34002794	OtA5088-OtA5092	OtA5093-OtA5097
Sipa1l3	chr7:29320377-29505460	OtA5088-OtA5092	OtA5093-OtA5097
Slc7a9	chr7:35449091-35466034	OtA5088-OtA5092	OtA5093-OtA5097
St5	chr7:109523910-109617147	OtA5088-OtA5092	OtA5093-OtA5097
Thap3	chr4:151982637-151988986	OtA5088-OtA5092	OtA5093-OtA5097
Them4	chr3:94310131-94332532	OtA5088-OtA5092	OtA5093-OtA5097
Tti2	chr8:31150315-31168724	OtA5088-OtA5092	OtA5093-OtA5097
Ube4b	chr4:149328415-149426631	OtA5088-OtA5092	OtA5093-OtA5097
Vgll4	chr6:114862091-114921752	OtA5088-OtA5092	OtA5093-OtA5097
Wdr3	chr3:99885416-100162403	OtA5088-OtA5092	OtA5093-OtA5097
Zbtb33	chrX:38189792-38197046	OtA5088-OtA5092	OtA5093-OtA5097
Zdhhc2	chr8:40423814-40484842	OtA5088-OtA5092	OtA5093-OtA5097
Zfp207	chr11:80383278-80405873	OtA5088-OtA5092	OtA5093-OtA5097
Caskin1	chr17:24488782-24527938	OtA5088-OtA5092	OtA5093-OtA5097

Tmbim4	chr10:120208825-120224897	OtA5088-OtA5092	OtA5093-OtA5097
Gopc	chr10:52337023-52382124	OtA5088-OtA5092	OtA5093-OtA5097
Tecpr2	chr12:110889263-110972394	OtA5088-OtA5092	OtA5093-OtA5097
Aasdhppt	chr9:4294792-4309494	OtA5088-OtA5092	OtA5093-OtA5097
Ap3b1	chr13:94358959-94566316	OtA5088-OtA5092	OtA5093-OtA5097
Cd274	chr19:29367437-29388094	OtA5088-OtA5092	OtA5093-OtA5097
Clasp2	chr9:113741472-113919697	OtA5088-OtA5092	OtA5093-OtA5097
Gsr	chr8:33653237-33698162	OtA5088-OtA5092	OtA5093-OtA5097
H2afj	chr6:136808247-136810074	OtA5088-OtA5092	OtA5093-OtA5097
Lrrc45	chr11:120713952-120721127	OtA5088-OtA5092	OtA5093-OtA5097
Polr3gl	chr3:96577873-96594158	OtA5088-OtA5092	OtA5093-OtA5097
Qtrtd1	chr16:43861412-43889676	OtA5088-OtA5092	OtA5093-OtA5097
Rab10	chr12:3235790-3309969	OtA5088-OtA5092	OtA5093-OtA5097
Rassf4	chr6:116633007-116673836	OtA5088-OtA5092	OtA5093-OtA5097
Dcp1a	chr14:30008999-30527056	OtA5088-OtA5092	OtA5093-OtA5097
Rhbdd1	chr1:82316578-82445367	OtA5088-OtA5092	OtA5093-OtA5097
Snx20	chr8:88626827-88636128	OtA5088-OtA5092	OtA5093-OtA5097
Adipor2	chr6:119353149-119417483	OtA5088-OtA5092	OtA5093-OtA5097
Cnot3	chr7:3645268-3677553	OtA5088-OtA5092	OtA5093-OtA5097
Ddx51	chr5:110653450-110660496	OtA5088-OtA5092	OtA5093-OtA5097
Dnajb12	chr10:59879590-59898016	OtA5088-OtA5092	OtA5093-OtA5097
Hsd17b2	chr8:117701945-117759029	OtA5088-OtA5092	OtA5093-OtA5097
Mtfr1	chr3:19187328-19220817	OtA5088-OtA5092	OtA5093-OtA5097
Nudt21	chr8:94019402-94037039	OtA5088-OtA5092	OtA5093-OtA5097
Srsf10	chr4:135856091-135869899	OtA5088-OtA5092	OtA5093-OtA5097
Taf5l	chr8:123996312-124021309	OtA5088-OtA5092	OtA5093-OtA5097
Wdr74	chr19:8735838-8740624	OtA5088-OtA5092	OtA5093-OtA5097
Armcx3	chrX:134756567-134761456	OtA5088-OtA5092	OtA5093-OtA5097
Ascc1	chr10:59987908-60099990	OtA5088-OtA5092	OtA5093-OtA5097
Atp5h	chr11:115415696-115419919	OtA5088-OtA5092	OtA5093-OtA5097
Cab39l	chr14:59440980-59548903	OtA5088-OtA5092	OtA5093-OtA5097
Calu	chr6:29348118-29380513	OtA5088-OtA5092	OtA5093-OtA5097
Camk2n2	chr16:20611600-20645915	OtA5088-OtA5092	OtA5093-OtA5097
Cspg4	chr9:56865103-56899870	OtA5088-OtA5092	OtA5093-OtA5097
Cst3	chr2:148871731-148875468	OtA5088-OtA5092	OtA5093-OtA5097
Dlk2	chr17:46297420-46328023	OtA5088-OtA5092	OtA5093-OtA5097
Dpp9	chr17:56186681-56218889	OtA5088-OtA5092	OtA5093-OtA5097
Fam149b	chr14:20348161-20383491	OtA5088-OtA5092	OtA5093-OtA5097
Fam82a2	chr2:119136997-119157034	OtA5088-OtA5092	OtA5093-OtA5097
Frmd4b	chr6:97286866-97617657	OtA5088-OtA5092	OtA5093-OtA5097
Glipr2	chr4:43957701-43979118	OtA5088-OtA5092	OtA5093-OtA5097
Iffo1	chr6:125145240-125161782	OtA5088-OtA5092	OtA5093-OtA5097
Krr1	chr10:111972694-111997264	OtA5088-OtA5092	OtA5093-OtA5097
Ltk	chr2:119751325-119760431	OtA5088-OtA5092	OtA5093-OtA5097
Maz	chr7:127022136-127026479	OtA5088-OtA5092	OtA5093-OtA5097
Mettl21d	chr12:69577627-69583028	OtA5088-OtA5092	OtA5093-OtA5097
Nprl2	chr9:107542208-107545706	OtA5088-OtA5092	OtA5093-OtA5097

Osbpl11	chr16:33185070-33243312	OtA5088-OtA5092	OtA5093-OtA5097
Ppdpf	chr2:181187342-181188504	OtA5088-OtA5092	OtA5093-OtA5097
Prdx3	chr19:60864065-60874538	OtA5088-OtA5092	OtA5093-OtA5097
Ptch1	chr13:63511532-63565520	OtA5088-OtA5092	OtA5093-OtA5097
Rap1gds1	chr3:138925905-139075199	OtA5088-OtA5092	OtA5093-OtA5097
Ric8b	chr10:84917615-85016447	OtA5088-OtA5092	OtA5093-OtA5097
Rundc1	chr11:101418813-101435665	OtA5088-OtA5092	OtA5093-OtA5097
Sec23a	chr12:58958383-59012017	OtA5088-OtA5092	OtA5093-OtA5097
Sepp1	chr15:3270766-3280508	OtA5088-OtA5092	OtA5093-OtA5097
Ss18	chr18:14625628-14682914	OtA5088-OtA5092	OtA5093-OtA5097
Tex2	chr11:106502138-106612930	OtA5088-OtA5092	OtA5093-OtA5097
Trim56	chr5:137111285-137116207	OtA5088-OtA5092	OtA5093-OtA5097
Zbtb10	chr3:9250566-9285332	OtA5088-OtA5092	OtA5093-OtA5097
Rtel1	chr2:181319723-181356616	OtA5088-OtA5092	OtA5093-OtA5097
Tipin	chr9:64281606-64304792	OtA5088-OtA5092	OtA5093-OtA5097
Zfp740	chr15:102208266-102215601	OtA5088-OtA5092	OtA5093-OtA5097
Cdon	chr9:35452075-35507652	OtA5088-OtA5092	OtA5093-OtA5097
Arl5b	chr2:15055361-15079191	OtA5088-OtA5092	OtA5093-OtA5097
Bid	chr6:120893118-120916820	OtA5088-OtA5092	OtA5093-OtA5097
Dctn2	chr10:127266261-127288771	OtA5088-OtA5092	OtA5093-OtA5097
Dctn3	chr4:41714797-41723163	OtA5088-OtA5092	OtA5093-OtA5097
Ddx19a	chr8:110974990-110997823	OtA5088-OtA5092	OtA5093-OtA5097
Dhrs4	chr14:55478757-55490340	OtA5088-OtA5092	OtA5093-OtA5097
Esyt2	chr12:116281221-116373098	OtA5088-OtA5092	OtA5093-OtA5097
Fam20a	chr11:109672925-109722256	OtA5088-OtA5092	OtA5093-OtA5097
Fgfr1	chr8:25518771-25575714	OtA5088-OtA5092	OtA5093-OtA5097
Lama1	chr17:67697264-67822645	OtA5088-OtA5092	OtA5093-OtA5097
Map3k3	chr11:106084901-106155434	OtA5088-OtA5092	OtA5093-OtA5097
Nop56	chr2:130274411-130284451	OtA5088-OtA5092	OtA5093-OtA5097
Otud5	chrX:7841830-7874858	OtA5088-OtA5092	OtA5093-OtA5097
Ruvbl2	chr7:45421897-45434464	OtA5088-OtA5092	OtA5093-OtA5097
St6galnac3	chr3:153202508-153725133	OtA5088-OtA5092	OtA5093-OtA5097
Tbc1d13	chr2:30133870-30152013	OtA5088-OtA5092	OtA5093-OtA5097
Tmem51	chr4:142030992-142088101	OtA5088-OtA5092	OtA5093-OtA5097
Itgb1bp1	chr12:21111755-21286237	OtA5088-OtA5092	OtA5093-OtA5097
Aldh3b2	chr19:3972327-3981665	OtA5088-OtA5092	OtA5093-OtA5097
Tsen15	chr1:152370735-152386682	OtA5088-OtA5092	OtA5093-OtA5097
Sh2d5	chr4:138250410-138260968	OtA5088-OtA5092	OtA5093-OtA5097
Ssh3	chr19:4261668-4269172	OtA5088-OtA5092	OtA5093-OtA5097
Wnt9b	chr11:103727362-103749821	OtA5088-OtA5092	OtA5093-OtA5097
Tssc1	chr12:28751827-28867491	OtA5088-OtA5092	OtA5093-OtA5097
S100a10	chr3:93555116-93564645	OtA5088-OtA5092	OtA5093-OtA5097
Usp54	chr14:20499313-20618354	OtA5088-OtA5092	OtA5093-OtA5097
Ints9	chr14:64950044-65039835	OtA5088-OtA5092	OtA5093-OtA5097
Ap4s1	chr12:51608540-51738939	OtA5088-OtA5092	OtA5093-OtA5097
BC005624	chr2:30972832-30981941	OtA5088-OtA5092	OtA5093-OtA5097
Cdx2	chr5:147300899-147307249	OtA5088-OtA5092	OtA5093-OtA5097

Dennd4c	chr4:86748554-86850602	OtA5088-OtA5092	OtA5093-OtA5097
Fpgs	chr2:32646594-32694175	OtA5088-OtA5092	OtA5093-OtA5097
Gatsl2	chr5:134099747-134141758	OtA5088-OtA5092	OtA5093-OtA5097
Kiss1r	chr10:79697304-80369637	OtA5088-OtA5092	OtA5093-OtA5097
Lztfl1	chr9:123697592-123717557	OtA5088-OtA5092	OtA5093-OtA5097
Nars	chr18:64499646-64516557	OtA5088-OtA5092	OtA5093-OtA5097
Pex11c	chr8:3458816-3467648	OtA5088-OtA5092	OtA5093-OtA5097
Prpsap2	chr11:61729649-61762088	OtA5088-OtA5092	OtA5093-OtA5097
Rarg	chr15:102234939-102257483	OtA5088-OtA5092	OtA5093-OtA5097
Tiam1	chr16:89787110-89974699	OtA5088-OtA5092	OtA5093-OtA5097
Wdr92	chr11:17211892-17235200	OtA5088-OtA5092	OtA5093-OtA5097
Atp9b	chr18:80734140-80934058	OtA5088-OtA5092	OtA5093-OtA5097
Atg3	chr16:45158828-45188538	OtA5088-OtA5092	OtA5093-OtA5097
Tcp11l1	chr2:104679979-104712162	OtA5088-OtA5092	OtA5093-OtA5097
Zfp7	chr15:76879275-76892394	OtA5088-OtA5092	OtA5093-OtA5097
Aloxe3	chr11:69126376-69149115	OtA5088-OtA5092	OtA5093-OtA5097
Ano6	chr15:95790842-95975472	OtA5088-OtA5092	OtA5093-OtA5097
Cln6	chr9:62838786-62875917	OtA5088-OtA5092	OtA5093-OtA5097
Cndp2	chr18:84667468-84685633	OtA5088-OtA5092	OtA5093-OtA5097
Cnp	chr11:100574938-100581739	OtA5088-OtA5092	OtA5093-OtA5097
Cox6a1	chr5:115345653-115348955	OtA5088-OtA5092	OtA5093-OtA5097
Dexi	chr16:10530206-10543054	OtA5088-OtA5092	OtA5093-OtA5097
Nfkbia	chr12:55280813-55492647	OtA5088-OtA5092	OtA5093-OtA5097
Nr1h2	chr7:44549632-44553923	OtA5088-OtA5092	OtA5093-OtA5097
Pold4	chr19:4231898-4233634	OtA5088-OtA5092	OtA5093-OtA5097
Sdc3	chr4:130792536-130826318	OtA5088-OtA5092	OtA5093-OtA5097
10-Sep	chr10:59141626-59221847	OtA5088-OtA5092	OtA5093-OtA5097
Smoc1	chr12:81026807-81186414	OtA5088-OtA5092	OtA5093-OtA5097
Stk19	chr17:34823992-34836903	OtA5088-OtA5092	OtA5093-OtA5097
Tenc1	chr15:102102987-102116401	OtA5088-OtA5092	OtA5093-OtA5097
Tfcp2	chr15:100502795-100551947	OtA5088-OtA5092	OtA5093-OtA5097
Tlr5	chr1:182954787-182976044	OtA5088-OtA5092	OtA5093-OtA5097
Parn	chr16:13537963-13668170	OtA5088-OtA5092	OtA5093-OtA5097
Exosc6	chr8:111033966-111057599	OtA5088-OtA5092	OtA5093-OtA5097
Cwf19l1	chr19:44108636-44135876	OtA5088-OtA5092	OtA5093-OtA5097
Fbxl15	chr19:46328183-46338660	OtA5088-OtA5092	OtA5093-OtA5097
Srrd	chr5:112326368-112343040	OtA5088-OtA5092	OtA5093-OtA5097
Lrch4	chr5:137629122-137642902	OtA5088-OtA5092	OtA5093-OtA5097
Otud3	chr4:138895378-138913947	OtA5088-OtA5092	OtA5093-OtA5097
Tbc1d23	chr16:57168863-57231466	OtA5088-OtA5092	OtA5093-OtA5097
Acta1	chr8:123891766-123894736	OtA5088-OtA5092	OtA5093-OtA5097
Actc1	chr2:114047288-114052811	OtA5088-OtA5092	OtA5093-OtA5097
Adamtsl5	chr10:79697304-80369637	OtA5088-OtA5092	OtA5093-OtA5097
Agpat4	chr17:12119283-12219640	OtA5088-OtA5092	OtA5093-OtA5097
Alkbh7	chr17:56997338-56999336	OtA5088-OtA5092	OtA5093-OtA5097
Appbp2	chr11:85191309-85235120	OtA5088-OtA5092	OtA5093-OtA5097
Arfip1	chr3:84496063-84582625	OtA5088-OtA5092	OtA5093-OtA5097

Arhgap22	chr14:33216822-33369936	OtA5088-OtA5092	OtA5093-OtA5097
Arid3a	chr10:79697304-80369637	OtA5088-OtA5092	OtA5093-OtA5097
Arsg	chr11:109440077-109573329	OtA5088-OtA5092	OtA5093-OtA5097
Atl1	chr12:69746847-69987002	OtA5088-OtA5092	OtA5093-OtA5097
B3gntl1	chr11:121451948-121673151	OtA5088-OtA5092	OtA5093-OtA5097
Bcl2l2	chr14:54883424-54888234	OtA5088-OtA5092	OtA5093-OtA5097
Bud13	chr9:46283011-46298783	OtA5088-OtA5092	OtA5093-OtA5097
Cand2	chr6:115774556-115808743	OtA5088-OtA5092	OtA5093-OtA5097
Ccdc109b	chr3:129914959-129970206	OtA5088-OtA5092	OtA5093-OtA5097
Cenpb	chr2:131127411-131604824	OtA5088-OtA5092	OtA5093-OtA5097
Cept1	chr3:106502259-106547761	OtA5088-OtA5092	OtA5093-OtA5097
Cpne8	chr15:90487480-90679388	OtA5088-OtA5092	OtA5093-OtA5097
Dcun1d5	chr9:7184565-7207031	OtA5088-OtA5092	OtA5093-OtA5097
Ddx24	chr12:103407975-103425867	OtA5088-OtA5092	OtA5093-OtA5097
Dnm2	chr9:21424907-21510186	OtA5088-OtA5092	OtA5093-OtA5097
Dyrk1b	chr7:28179482-28187294	OtA5088-OtA5092	OtA5093-OtA5097
Eral1	chr11:78073375-78080383	OtA5088-OtA5092	OtA5093-OtA5097
Exosc1	chr19:41922979-41933314	OtA5088-OtA5092	OtA5093-OtA5097
Fbxl19	chr7:127746774-127768928	OtA5088-OtA5092	OtA5093-OtA5097
Fbxo9	chr9:78081499-78109053	OtA5088-OtA5092	OtA5093-OtA5097
Fbxw4	chr19:45560614-45660193	OtA5088-OtA5092	OtA5093-OtA5097
Flnb	chr14:7817956-7951587	OtA5088-OtA5092	OtA5093-OtA5097
Fosl2	chr5:32136471-32157839	OtA5088-OtA5092	OtA5093-OtA5097
Galnt10	chr11:57645441-57787500	OtA5088-OtA5092	OtA5093-OtA5097
Gls2	chr10:128194634-128211794	OtA5088-OtA5092	OtA5093-OtA5097
Gm5480	chr16:5007287-5008308	OtA5088-OtA5092	OtA5093-OtA5097
Gpd2	chr2:57237677-57370719	OtA5088-OtA5092	OtA5093-OtA5097
Gtf2e1	chr16:37509795-37539769	OtA5088-OtA5092	OtA5093-OtA5097
H2-K1	chr17:33996011-34000333	OtA5088-OtA5092	OtA5093-OtA5097
Haghl	chr17:25773775-25785333	OtA5088-OtA5092	OtA5093-OtA5097
Hmga1	chr17:27556573-27563672	OtA5088-OtA5092	OtA5093-OtA5097
Hnrnpk	chr13:58391950-58411149	OtA5088-OtA5092	OtA5093-OtA5097
Icam2	chr11:106377655-106382641	OtA5088-OtA5092	OtA5093-OtA5097
Ict1	chr11:115403765-115410913	OtA5088-OtA5092	OtA5093-OtA5097
Igf2r	chr17:12682405-12868143	OtA5088-OtA5092	OtA5093-OtA5097
Ilf2	chr3:90476200-90491013	OtA5088-OtA5092	OtA5093-OtA5097
Ipo11	chr13:106794438-106936915	OtA5088-OtA5092	OtA5093-OtA5097
Kdm4c	chr4:74242496-74405864	OtA5088-OtA5092	OtA5093-OtA5097
Klf9	chr19:23141225-23166911	OtA5088-OtA5092	OtA5093-OtA5097
Lass4	chr8:4493404-4526079	OtA5088-OtA5092	OtA5093-OtA5097
Lect1	chr14:79637689-79662170	OtA5088-OtA5092	OtA5093-OtA5097
Map2k5	chr9:63163769-63377852	OtA5088-OtA5092	OtA5093-OtA5097
Med1	chr11:98152154-98193293	OtA5088-OtA5092	OtA5093-OtA5097
Med31	chr11:72211723-72218450	OtA5088-OtA5092	OtA5093-OtA5097
Mettl16	chr11:74770862-74818692	OtA5088-OtA5092	OtA5093-OtA5097
Mib2	chr4:155654469-155669254	OtA5088-OtA5092	OtA5093-OtA5097
Mmp2	chr8:92827327-92853420	OtA5088-OtA5092	OtA5093-OtA5097

Mob3a	chr10:80685252-80701820	OtA5088-OtA5092	OtA5093-OtA5097
Mob3b	chr4:34949073-35157484	OtA5088-OtA5092	OtA5093-OtA5097
Myl6	chr10:128490860-128493825	OtA5088-OtA5092	OtA5093-OtA5097
Myst1	chr7:127912516-127930113	OtA5088-OtA5092	OtA5093-OtA5097
Nom1	chr5:29434666-29452169	OtA5088-OtA5092	OtA5093-OtA5097
Nos2	chr11:78920858-78960124	OtA5088-OtA5092	OtA5093-OtA5097
Nt5dc1	chr10:34303611-34418528	OtA5088-OtA5092	OtA5093-OtA5097
Ntf5	chr7:45413694-45417179	OtA5088-OtA5092	OtA5093-OtA5097
Oma1	chr4:103313845-103366428	OtA5088-OtA5092	OtA5093-OtA5097
Osbpl10	chr9:115067278-115232223	OtA5088-OtA5092	OtA5093-OtA5097
P2rx2	chr5:110339811-110343035	OtA5088-OtA5092	OtA5093-OtA5097
Parp11	chr6:127453722-127494239	OtA5088-OtA5092	OtA5093-OtA5097
Pcmt1	chr10:7630235-7664623	OtA5088-OtA5092	OtA5093-OtA5097
Pdpk1	chr17:24073679-24141594	OtA5088-OtA5092	OtA5093-OtA5097
Phyhd1	chr2:30266528-30282149	OtA5088-OtA5092	OtA5093-OtA5097
Pitrm1	chr13:6548156-6648724	OtA5088-OtA5092	OtA5093-OtA5097
Prrc2b	chr2:32151147-32234537	OtA5088-OtA5092	OtA5093-OtA5097
Psg23	chr7:18606342-18616501	OtA5088-OtA5092	OtA5093-OtA5097
Psmd12	chr11:107479527-107498036	OtA5088-OtA5092	OtA5093-OtA5097
Qtrt1	chr9:21411836-21420279	OtA5088-OtA5092	OtA5093-OtA5097
Rab8a	chr8:72161199-72181366	OtA5088-OtA5092	OtA5093-OtA5097
Rbbp9	chr2:144542264-144550859	OtA5088-OtA5092	OtA5093-OtA5097
Rdm1	chr11:101627948-101636081	OtA5088-OtA5092	OtA5093-OtA5097
Rhou	chr8:123653928-123663880	OtA5088-OtA5092	OtA5093-OtA5097
Ripk3	chr14:55784994-55788857	OtA5088-OtA5092	OtA5093-OtA5097
Rnf4	chr5:34336389-34353430	OtA5088-OtA5092	OtA5093-OtA5097
Rnf8	chr17:29614830-29641659	OtA5088-OtA5092	OtA5093-OtA5097
Rom1	chr19:8927381-8929356	OtA5088-OtA5092	OtA5093-OtA5097
Rpl34-ps1	chr3:130726830-130730329	OtA5088-OtA5092	OtA5093-OtA5097
Sfn	chr4:133600555-133602168	OtA5088-OtA5092	OtA5093-OtA5097
Sh3bp5	chr14:31373963-31436033	OtA5088-OtA5092	OtA5093-OtA5097
Slc25a24	chr3:109123148-109168409	OtA5088-OtA5092	OtA5093-OtA5097
Slc25a26	chr6:94500313-94700145	OtA5088-OtA5092	OtA5093-OtA5097
Slc2a10	chr2:165503977-165519911	OtA5088-OtA5092	OtA5093-OtA5097
Snap29	chr16:17280350-17430826	OtA5088-OtA5092	OtA5093-OtA5097
Snord17	chr2:144250123-144270902	OtA5088-OtA5092	OtA5093-OtA5097
Snx21	chr2:164786020-164804881	OtA5088-OtA5092	OtA5093-OtA5097
Solh	chr17:25959555-25965505	OtA5088-OtA5092	OtA5093-OtA5097
Spp2	chr1:88407018-88426438	OtA5088-OtA5092	OtA5093-OtA5097
St7l	chr3:104864505-104930063	OtA5088-OtA5092	OtA5093-OtA5097
Stard7	chr2:127270228-127298934	OtA5088-OtA5092	OtA5093-OtA5097
Stau1	chr2:166948141-166996278	OtA5088-OtA5092	OtA5093-OtA5097
Stx3	chr19:11775117-11819403	OtA5088-OtA5092	OtA5093-OtA5097
Syde2	chr3:145987869-146021720	OtA5088-OtA5092	OtA5093-OtA5097
Taf2	chr15:55015128-55072152	OtA5088-OtA5092	OtA5093-OtA5097
Taf6	chr5:138172020-138187186	OtA5088-OtA5092	OtA5093-OtA5097
Tcf25	chr8:123373752-123404173	OtA5088-OtA5092	OtA5093-OtA5097

Tcf4	chr18:69344491-69687967	OtA5088-OtA5092	OtA5093-OtA5097
Timm17b	chrX:7899397-7907652	OtA5088-OtA5092	OtA5093-OtA5097
Timm44	chr8:4259730-4275905	OtA5088-OtA5092	OtA5093-OtA5097
Tmc7	chr7:118535843-118584686	OtA5088-OtA5092	OtA5093-OtA5097
Tmed8	chr12:87166241-87200229	OtA5088-OtA5092	OtA5093-OtA5097
Tmem203	chr2:25255438-25256352	OtA5088-OtA5092	OtA5093-OtA5097
Tmem98	chr11:80810414-80822033	OtA5088-OtA5092	OtA5093-OtA5097
Tnpo3	chr6:29540826-29609607	OtA5088-OtA5092	OtA5093-OtA5097
Toe1	chr4:116720954-116807559	OtA5088-OtA5092	OtA5093-OtA5097
Tomm34	chr2:164053540-164071102	OtA5088-OtA5092	OtA5093-OtA5097
Tsku	chr7:98350667-98361328	OtA5088-OtA5092	OtA5093-OtA5097
Tspan3	chr9:56135883-56161070	OtA5088-OtA5092	OtA5093-OtA5097
Txnl1	chr18:63662800-63692359	OtA5088-OtA5092	OtA5093-OtA5097
Wdr34	chr2:30031557-30048879	OtA5088-OtA5092	OtA5093-OtA5097
Wdr43	chr17:71616214-71659031	OtA5088-OtA5092	OtA5093-OtA5097
Wrap53	chr11:69561753-69579324	OtA5088-OtA5092	OtA5093-OtA5097
Wrb	chr16:96145418-96157852	OtA5088-OtA5092	OtA5093-OtA5097
Zbtb25	chr12:76348899-76369560	OtA5088-OtA5092	OtA5093-OtA5097
Zc3h7b	chr15:81744847-81796269	OtA5088-OtA5092	OtA5093-OtA5097
Zdhhc4	chr5:143316488-143329238	OtA5088-OtA5092	OtA5093-OtA5097
Zfp414	chr17:33629091-33631714	OtA5088-OtA5092	OtA5093-OtA5097
Zfp518b	chr5:38668483-38684826	OtA5088-OtA5092	OtA5093-OtA5097
Zscan22	chr7:12897814-12908239	OtA5088-OtA5092	OtA5093-OtA5097
Fam53c	chr18:34758905-34773760	OtA5088-OtA5092	OtA5093-OtA5097
Fbxl7	chr15:26540458-26895564	OtA5088-OtA5092	OtA5093-OtA5097
Mcfd2	chr17:87254442-87265947	OtA5088-OtA5092	OtA5093-OtA5097
Mical2	chr7:112225835-112355194	OtA5088-OtA5092	OtA5093-OtA5097
Mta1	chr12:113098277-113137206	OtA5088-OtA5092	OtA5093-OtA5097
Naa10	chrX:73916869-73921944	OtA5088-OtA5092	OtA5093-OtA5097
Ppme1	chr7:100326736-100371896	OtA5088-OtA5092	OtA5093-OtA5097
Slc13a3	chr2:165405294-165473197	OtA5088-OtA5092	OtA5093-OtA5097
Snapc5	chr9:64179296-64182688	OtA5088-OtA5092	OtA5093-OtA5097
Ssh1	chr5:113942218-113993757	OtA5088-OtA5092	OtA5093-OtA5097
Tle1	chr4:72117141-72200919	OtA5088-OtA5092	OtA5093-OtA5097
Tox4	chr14:52261758-52305124	OtA5088-OtA5092	OtA5093-OtA5097
Ttll3	chr6:113378112-113414571	OtA5088-OtA5092	OtA5093-OtA5097
Zc3h3	chr15:75754446-75841908	OtA5088-OtA5092	OtA5093-OtA5097
Exoc2	chr13:30813917-30974047	OtA5088-OtA5092	OtA5093-OtA5097
Mrpl41	chr2:24962421-24975098	OtA5088-OtA5092	OtA5093-OtA5097
Ppm1m	chr9:106194952-106199233	OtA5088-OtA5092	OtA5093-OtA5097
Psmc1	chr12:100112330-100123364	OtA5088-OtA5092	OtA5093-OtA5097
Alg5	chr3:54735538-54749795	OtA5088-OtA5092	OtA5093-OtA5097
Cyp11a1	chr9:58015016-58027023	OtA5088-OtA5092	OtA5093-OtA5097
Eif4ebp2	chr10:61432496-61452669	OtA5088-OtA5092	OtA5093-OtA5097
Ruvbl1	chr6:88465422-88497566	OtA5088-OtA5092	OtA5093-OtA5097
Abi2	chr1:60409618-60481163	OtA5088-OtA5092	OtA5093-OtA5097
Akna	chr4:63367127-63403445	OtA5088-OtA5092	OtA5093-OtA5097

Aqp8	chr7:123462293-123468003	OtA5088-OtA5092	OtA5093-OtA5097
Arf3	chr15:98737625-98763118	OtA5088-OtA5092	OtA5093-OtA5097
BC028528	chr3:95871521-95891930	OtA5088-OtA5092	OtA5093-OtA5097
C230052I12Rik	chr7:35334236-35396767	OtA5088-OtA5092	OtA5093-OtA5097
Casp6	chr3:129901424-129914102	OtA5088-OtA5092	OtA5093-OtA5097
Ccdc61	chr7:18890883-18910404	OtA5088-OtA5092	OtA5093-OtA5097
Ccng2	chr5:93267572-93276231	OtA5088-OtA5092	OtA5093-OtA5097
Ccnh	chr13:85189476-85213723	OtA5088-OtA5092	OtA5093-OtA5097
Cdc23	chr18:34598614-34651736	OtA5088-OtA5092	OtA5093-OtA5097
Chd4	chr6:125096162-125130501	OtA5088-OtA5092	OtA5093-OtA5097
Chpt1	chr10:88459586-88503970	OtA5088-OtA5092	OtA5093-OtA5097
Cox6b2	chr7:4751793-4753094	OtA5088-OtA5092	OtA5093-OtA5097
Crtc1	chr8:70382357-70439573	OtA5088-OtA5092	OtA5093-OtA5097
Ctnna1	chr18:35118911-35254775	OtA5088-OtA5092	OtA5093-OtA5097
Ctu2	chr8:122476142-122551329	OtA5088-OtA5092	OtA5093-OtA5097
Cyb561d2	chr9:107539010-107541865	OtA5088-OtA5092	OtA5093-OtA5097
Cyfip1	chr7:55842070-55962493	OtA5088-OtA5092	OtA5093-OtA5097
Dis3	chr14:99076633-99254493	OtA5088-OtA5092	OtA5093-OtA5097
Dmgdh	chr13:93674435-93752823	OtA5088-OtA5092	OtA5093-OtA5097
Donson	chr16:91679264-91688728	OtA5088-OtA5092	OtA5093-OtA5097
Dpy30	chr17:74299473-74323944	OtA5088-OtA5092	OtA5093-OtA5097
E2f5	chr3:14578670-14611256	OtA5088-OtA5092	OtA5093-OtA5097
Efhd1	chr1:87264363-87310791	OtA5088-OtA5092	OtA5093-OtA5097
Ercc3	chr18:32240330-32270147	OtA5088-OtA5092	OtA5093-OtA5097
Fkbp15	chr4:62300341-62360548	OtA5088-OtA5092	OtA5093-OtA5097
Folr2	chr7:101839987-101845331	OtA5088-OtA5092	OtA5093-OtA5097
Golt1b	chr6:142387242-142403858	OtA5088-OtA5092	OtA5093-OtA5097
Gpr35	chr1:92973118-92986391	OtA5088-OtA5092	OtA5093-OtA5097
H2-D1	chr17:35263093-35267497	OtA5088-OtA5092	OtA5093-OtA5097
Heca	chr10:17900465-17948067	OtA5088-OtA5092	OtA5093-OtA5097
Hes6	chr1:91411482-91413222	OtA5088-OtA5092	OtA5093-OtA5097
Hp	chr8:109575127-109579172	OtA5088-OtA5092	OtA5093-OtA5097
Iscu	chr5:113772811-113778282	OtA5088-OtA5092	OtA5093-OtA5097
Kcmf1	chr6:72841113-72899979	OtA5088-OtA5092	OtA5093-OtA5097
Kctd21	chr7:97332322-97350216	OtA5088-OtA5092	OtA5093-OtA5097
Klf16	chr10:80567120-80577296	OtA5088-OtA5092	OtA5093-OtA5097
Klhl17	chr4:156229043-156234857	OtA5088-OtA5092	OtA5093-OtA5097
Klhl8	chr5:103862049-103911229	OtA5088-OtA5092	OtA5093-OtA5097
Krt18	chr15:102028215-102032026	OtA5088-OtA5092	OtA5093-OtA5097
Map3k4	chr17:12227620-12318660	OtA5088-OtA5092	OtA5093-OtA5097
Mepce	chr5:137781905-137786701	OtA5088-OtA5092	OtA5093-OtA5097
Mettl4	chr17:94727079-94749892	OtA5088-OtA5092	OtA5093-OtA5097
Mnt	chr11:74830923-74845725	OtA5088-OtA5092	OtA5093-OtA5097
Mras	chr9:99385419-99436712	OtA5088-OtA5092	OtA5093-OtA5097
Mthfs	chr9:89211189-89240225	OtA5088-OtA5092	OtA5093-OtA5097
Myo1d	chr11:80482126-80780025	OtA5088-OtA5092	OtA5093-OtA5097
Nab2	chr10:127642985-127666703	OtA5088-OtA5092	OtA5093-OtA5097

Napg	chr18:62977915-62999451	OtA5088-OtA5092	OtA5093-OtA5097
Nsmce2	chr15:59374197-59601684	OtA5088-OtA5092	OtA5093-OtA5097
Ntn1	chr11:68209363-68386826	OtA5088-OtA5092	OtA5093-OtA5097
Nup155	chr15:8109312-8159859	OtA5088-OtA5092	OtA5093-OtA5097
Patz1	chr11:3202352-3479831	OtA5088-OtA5092	OtA5093-OtA5097
Pdha1	chrX:159988432-160138336	OtA5088-OtA5092	OtA5093-OtA5097
Peg10	chr6:4747305-4760516	OtA5088-OtA5092	OtA5093-OtA5097
Pld2	chr11:70540163-70558110	OtA5088-OtA5092	OtA5093-OtA5097
Pramef12	chr4:144391673-144408464	OtA5088-OtA5092	OtA5093-OtA5097
Prpf4	chr4:62408782-62426990	OtA5088-OtA5092	OtA5093-OtA5097
Ptpn1	chr2:167932326-167979385	OtA5088-OtA5092	OtA5093-OtA5097
Rab33b	chr3:51483965-51496212	OtA5088-OtA5092	OtA5093-OtA5097
Ramp3	chr11:6650147-6677475	OtA5088-OtA5092	OtA5093-OtA5097
Ranbp17	chr11:33211793-33513746	OtA5088-OtA5092	OtA5093-OtA5097
Rel	chr11:23741728-23770970	OtA5088-OtA5092	OtA5093-OtA5097
Reps2	chrX:162407199-163352564	OtA5088-OtA5092	OtA5093-OtA5097
Rgp1	chr4:43562633-43587487	OtA5088-OtA5092	OtA5093-OtA5097
Rilpl1	chr5:124493079-124531391	OtA5088-OtA5092	OtA5093-OtA5097
Rnf19b	chr4:129058270-129084526	OtA5088-OtA5092	OtA5093-OtA5097
Rpl35	chr2:38998308-39005131	OtA5088-OtA5092	OtA5093-OtA5097
Rps28	chr17:33823036-33824498	OtA5088-OtA5092	OtA5093-OtA5097
Rpusd3	chr6:113415318-113419340	OtA5088-OtA5092	OtA5093-OtA5097
Rrp7a	chr15:83100204-83122801	OtA5088-OtA5092	OtA5093-OtA5097
Rsu1	chr2:13077008-13271278	OtA5088-OtA5092	OtA5093-OtA5097
Scamp5	chr9:57441326-57468024	OtA5088-OtA5092	OtA5093-OtA5097
Scd4	chr19:44333325-44346743	OtA5088-OtA5092	OtA5093-OtA5097
15-Sep	chr3:144570426-144597676	OtA5088-OtA5092	OtA5093-OtA5097
Serpinb9	chr13:33004540-33017955	OtA5088-OtA5092	OtA5093-OtA5097
Slc4a4	chr5:88887259-89239656	OtA5088-OtA5092	OtA5093-OtA5097
Slco2a1	chr9:103008488-103087849	OtA5088-OtA5092	OtA5093-OtA5097
Snd1	chr6:28480347-28888832	OtA5088-OtA5092	OtA5093-OtA5097
Spsb2	chr6:124808940-124814296	OtA5088-OtA5092	OtA5093-OtA5097
Suv39h2	chr2:3424130-3474986	OtA5088-OtA5092	OtA5093-OtA5097
Telo2	chr17:25099568-25115967	OtA5088-OtA5092	OtA5093-OtA5097
Tigd5	chr15:75909734-75914535	OtA5088-OtA5092	OtA5093-OtA5097
Tmem134	chr19:4125959-4132307	OtA5088-OtA5092	OtA5093-OtA5097
Tmem63a	chr1:180942517-180975104	OtA5088-OtA5092	OtA5093-OtA5097
Tsc2	chr17:24549949-24632629	OtA5088-OtA5092	OtA5093-OtA5097
Txndc12	chr4:108834677-108862119	OtA5088-OtA5092	OtA5093-OtA5097
Uba5	chr9:104046587-104063121	OtA5088-OtA5092	OtA5093-OtA5097
Wwc1	chr11:35839177-35980089	OtA5088-OtA5092	OtA5093-OtA5097
Wwc2	chr8:47827605-47990551	OtA5088-OtA5092	OtA5093-OtA5097
Yap1	chr9:7932000-8004596	OtA5088-OtA5092	OtA5093-OtA5097
Zbtb22	chr17:33916175-33919325	OtA5088-OtA5092	OtA5093-OtA5097
Zbtb7b	chr3:89377645-89398779	OtA5088-OtA5092	OtA5093-OtA5097
Zfp277	chr12:40315045-40445790	OtA5088-OtA5092	OtA5093-OtA5097
Zfp524	chr7:5015507-5018488	OtA5088-OtA5092	OtA5093-OtA5097

Zscan2	chr7:80862107-80876513	OtA5088-OtA5092	OtA5093-OtA5097
Zyg11b	chr4:108227754-108301090	OtA5088-OtA5092	OtA5093-OtA5097
Cand1	chr10:119198811-119240055	OtA5088-OtA5092	OtA5093-OtA5097
Vasn	chr16:4626883-4679720	OtA5088-OtA5092	OtA5093-OtA5097
Dcaf4	chr12:83520465-83541992	OtA5088-OtA5092	OtA5093-OtA5097
Exosc3	chr4:45316629-45320603	OtA5088-OtA5092	OtA5093-OtA5097
Fam63b	chr9:70599013-70657174	OtA5088-OtA5092	OtA5093-OtA5097
Klhdc3	chr17:46674550-46680930	OtA5088-OtA5092	OtA5093-OtA5097
Nlgn2	chr11:69823122-69834849	OtA5088-OtA5092	OtA5093-OtA5097
Usp10	chr8:119910851-119957557	OtA5088-OtA5092	OtA5093-OtA5097
Zbtb12	chr17:34894558-34896844	OtA5088-OtA5092	OtA5093-OtA5097
Slc44a3	chr3:121459527-121532344	OtA5088-OtA5092	OtA5093-OtA5097
Dnlz	chr2:26315532-26352110	OtA5088-OtA5092	OtA5093-OtA5097
Atf1	chr15:100227858-100261248	OtA5088-OtA5092	OtA5093-OtA5097
BC022687	chr12:112808974-112816245	OtA5088-OtA5092	OtA5093-OtA5097
Bicd2	chr13:49341548-49387025	OtA5088-OtA5092	OtA5093-OtA5097
Ccbl1	chr2:30185130-30205699	OtA5088-OtA5092	OtA5093-OtA5097
D930015E06Rik	chr3:83898286-84040161	OtA5088-OtA5092	OtA5093-OtA5097
Gnpnat1	chr14:45351185-45388796	OtA5088-OtA5092	OtA5093-OtA5097
H2afz	chr3:137864598-137866922	OtA5088-OtA5092	OtA5093-OtA5097
Ispd	chr12:36381518-36689444	OtA5088-OtA5092	OtA5093-OtA5097
Large	chr8:72814598-73352556	OtA5088-OtA5092	OtA5093-OtA5097
Nkap	chrX:37126762-37168842	OtA5088-OtA5092	OtA5093-OtA5097
Rnft1	chr11:86415843-86544807	OtA5088-OtA5092	OtA5093-OtA5097
Timm50	chr7:28305825-28312046	OtA5088-OtA5092	OtA5093-OtA5097
Tmco7	chr8:106683067-106851439	OtA5088-OtA5092	OtA5093-OtA5097
Ube2i	chr17:25260510-25275528	OtA5088-OtA5092	OtA5093-OtA5097
Arl6ip1	chr7:118118889-118129625	OtA5088-OtA5092	OtA5093-OtA5097
Cald1	chr6:34709443-34775469	OtA5088-OtA5092	OtA5093-OtA5097
Capn6	chrX:143802236-143827412	OtA5088-OtA5092	OtA5093-OtA5097
Ccl6	chr11:83582060-83623693	OtA5088-OtA5092	OtA5093-OtA5097
Dctn1	chr6:83165923-83200118	OtA5088-OtA5092	OtA5093-OtA5097
Extl2	chr3:116007448-116029016	OtA5088-OtA5092	OtA5093-OtA5097
Fance	chr17:28313529-28326574	OtA5088-OtA5092	OtA5093-OtA5097
Hpcal1	chr12:17690813-17791926	OtA5088-OtA5092	OtA5093-OtA5097
Ipmk	chr10:71347792-71385885	OtA5088-OtA5092	OtA5093-OtA5097
Kcnj15	chr16:95257650-95300258	OtA5088-OtA5092	OtA5093-OtA5097
Kpnb1	chr11:97159709-97187892	OtA5088-OtA5092	OtA5093-OtA5097
Mat2a	chr6:72432798-72440615	OtA5088-OtA5092	OtA5093-OtA5097
Memo1	chr17:74200699-74294863	OtA5088-OtA5092	OtA5093-OtA5097
Mtmr10	chr7:64287669-64340806	OtA5088-OtA5092	OtA5093-OtA5097
Mycl1	chr4:122996098-123002480	OtA5088-OtA5092	OtA5093-OtA5097
Ncoa3	chr2:165992636-166073242	OtA5088-OtA5092	OtA5093-OtA5097
Noa1	chr5:77294168-77310086	OtA5088-OtA5092	OtA5093-OtA5097
Orc6	chr8:85299631-85308279	OtA5088-OtA5092	OtA5093-OtA5097
Pgf	chr12:85166640-85177285	OtA5088-OtA5092	OtA5093-OtA5097
Prkar1a	chr11:109650948-109669648	OtA5088-OtA5092	OtA5093-OtA5097

Rab20	chr8:11453976-11478499	OtA5088-OtA5092	OtA5093-OtA5097
Rbm15	chr3:107326109-107333289	OtA5088-OtA5092	OtA5093-OtA5097
Tbl2	chr5:135149710-135162662	OtA5088-OtA5092	OtA5093-OtA5097
Tmem63b	chr17:45660176-45686218	OtA5088-OtA5092	OtA5093-OtA5097
Trim71	chr9:114511272-114564369	OtA5088-OtA5092	OtA5093-OtA5097
Tspan6	chrX:133891069-133898429	OtA5088-OtA5092	OtA5093-OtA5097
Txnip	chr3:96555767-96566801	OtA5088-OtA5092	OtA5093-OtA5097
Ugdh	chr5:65413221-65435842	OtA5088-OtA5092	OtA5093-OtA5097
Vav3	chr3:109340682-109685694	OtA5088-OtA5092	OtA5093-OtA5097
Xpo5	chr17:46202854-46242299	OtA5088-OtA5092	OtA5093-OtA5097
Zmat3	chr3:32334793-32367438	OtA5088-OtA5092	OtA5093-OtA5097
Anxa9	chr3:95282934-95307176	OtA5088-OtA5092	OtA5093-OtA5097
Tmed1	chr9:21424907-21510186	OtA5088-OtA5092	OtA5093-OtA5097
Gpc3	chrX:52272426-52619047	OtA5088-OtA5092	OtA5093-OtA5097
Gadd45g	chr13:51846674-51848474	OtA5088-OtA5092	OtA5093-OtA5097
Spint2	chr7:29256329-29281977	OtA5088-OtA5092	OtA5093-OtA5097
Kpna4	chr3:69072220-69127092	OtA5088-OtA5092	OtA5093-OtA5097
Alg14	chr3:121291816-121362011	OtA5088-OtA5092	OtA5093-OtA5097
Als2	chr1:59120934-59237231	OtA5088-OtA5092	OtA5093-OtA5097
Anapc16	chr10:59987908-60099990	OtA5088-OtA5092	OtA5093-OtA5097
Ankzf1	chr1:75192134-75210778	OtA5088-OtA5092	OtA5093-OtA5097
BC029722	chr2:155775343-155819203	OtA5088-OtA5092	OtA5093-OtA5097
Bmp2	chr2:133553198-133562885	OtA5088-OtA5092	OtA5093-OtA5097
C87436	chr6:86438397-86470387	OtA5088-OtA5092	OtA5093-OtA5097
Ccnd2	chr6:127125708-127212419	OtA5088-OtA5092	OtA5093-OtA5097
Cep68	chr11:20227036-20249424	OtA5088-OtA5092	OtA5093-OtA5097
Cno	chr5:36747373-36748679	OtA5088-OtA5092	OtA5093-OtA5097
Coro7	chr16:4626883-4679720	OtA5088-OtA5092	OtA5093-OtA5097
Crlf3	chr11:80046498-80080955	OtA5088-OtA5092	OtA5093-OtA5097
Dcaf17	chr2:71055743-71099142	OtA5088-OtA5092	OtA5093-OtA5097
Dennd3	chr15:73512559-73572242	OtA5088-OtA5092	OtA5093-OtA5097
Dgcr6	chr16:18052859-18071342	OtA5088-OtA5092	OtA5093-OtA5097
E130311K13Rik	chr3:63914695-63929385	OtA5088-OtA5092	OtA5093-OtA5097
Ear1	chr14:43818764-43819639	OtA5088-OtA5092	OtA5093-OtA5097
Eif5a2	chr3:28781310-28798846	OtA5088-OtA5092	OtA5093-OtA5097
Eny2	chr15:44375226-44437685	OtA5088-OtA5092	OtA5093-OtA5097
Exosc8	chr3:54728678-54735364	OtA5088-OtA5092	OtA5093-OtA5097
Fam129a	chr1:151571372-151719347	OtA5088-OtA5092	OtA5093-OtA5097
Fcf1	chr12:84970929-84983303	OtA5088-OtA5092	OtA5093-OtA5097
Fem1b	chr9:62791829-62811648	OtA5088-OtA5092	OtA5093-OtA5097
Fkbpl	chr17:34644882-34646327	OtA5088-OtA5092	OtA5093-OtA5097
Galnt4	chr10:99107170-99197988	OtA5088-OtA5092	OtA5093-OtA5097
Glyr1	chr16:5013901-5049910	OtA5088-OtA5092	OtA5093-OtA5097
Hist1h4a	chr13:23760845-23761157	OtA5088-OtA5092	OtA5093-OtA5097
Kif1c	chr11:70700547-70731970	OtA5088-OtA5092	OtA5093-OtA5097
Klf10	chr15:38294412-38300707	OtA5088-OtA5092	OtA5093-OtA5097
Klhl2	chr8:64739861-64779865	OtA5088-OtA5092	OtA5093-OtA5097

Lrba	chr3:86224689-86782694	OtA5088-OtA5092	OtA5093-OtA5097
Lsm14b	chr2:180024986-180035461	OtA5088-OtA5092	OtA5093-OtA5097
Mink1	chr11:70562880-70614482	OtA5088-OtA5092	OtA5093-OtA5097
Mllt11	chr3:95218543-95228677	OtA5088-OtA5092	OtA5093-OtA5097
Mrap	chr16:90738323-90749776	OtA5088-OtA5092	OtA5093-OtA5097
Mrpl53	chr6:83101515-83109932	OtA5088-OtA5092	OtA5093-OtA5097
Notum	chr11:120653788-120660837	OtA5088-OtA5092	OtA5093-OtA5097
Nrp1	chr8:128359072-128505475	OtA5088-OtA5092	OtA5093-OtA5097
Pi4k2b	chr5:52741573-52769340	OtA5088-OtA5092	OtA5093-OtA5097
Pip4k2c	chr10:127190377-127211622	OtA5088-OtA5092	OtA5093-OtA5097
Plod2	chr9:92538800-92608427	OtA5088-OtA5092	OtA5093-OtA5097
Polr2a	chr11:69734409-69758223	OtA5088-OtA5092	OtA5093-OtA5097
Polr2f	chr15:79141366-79151767	OtA5088-OtA5092	OtA5093-OtA5097
Ppa2	chr3:133310115-133378069	OtA5088-OtA5092	OtA5093-OtA5097
Psg27	chr7:18556513-18567305	OtA5088-OtA5092	OtA5093-OtA5097
Rdbp	chr17:34850390-34856372	OtA5088-OtA5092	OtA5093-OtA5097
Rdh14	chr12:10390779-10395562	OtA5088-OtA5092	OtA5093-OtA5097
Rfx1	chr8:84066835-84096992	OtA5088-OtA5092	OtA5093-OtA5097
Rnf141	chr7:110816534-110844381	OtA5088-OtA5092	OtA5093-OtA5097
Rps17	chr7:81342732-81345234	OtA5088-OtA5092	OtA5093-OtA5097
Rwdd2b	chr16:87433330-87440592	OtA5088-OtA5092	OtA5093-OtA5097
Serinc2	chr4:130253496-130275586	OtA5088-OtA5092	OtA5093-OtA5097
Sik2	chr9:50856934-51009073	OtA5088-OtA5092	OtA5093-OtA5097
Thoc7	chr14:13949013-13961225	OtA5088-OtA5092	OtA5093-OtA5097
Tmem108	chr9:103482935-103761837	OtA5088-OtA5092	OtA5093-OtA5097
Tnks1bp1	chr2:85050459-85073048	OtA5088-OtA5092	OtA5093-OtA5097
Trp63	chr16:25683764-25892088	OtA5088-OtA5092	OtA5093-OtA5097
Tubgcp3	chr8:12614276-12672100	OtA5088-OtA5092	OtA5093-OtA5097
Vapb	chr2:173737570-173784336	OtA5088-OtA5092	OtA5093-OtA5097
Wars2	chr3:99141089-99220203	OtA5088-OtA5092	OtA5093-OtA5097
Whamm	chr7:81571291-81596836	OtA5088-OtA5092	OtA5093-OtA5097
Wnt2	chr6:17988939-18030445	OtA5088-OtA5092	OtA5093-OtA5097
Zer1	chr2:30097282-30124454	OtA5088-OtA5092	OtA5093-OtA5097
Numbl	chr7:27258760-27282150	OtA5088-OtA5092	OtA5093-OtA5097
Glg1	chr8:111157557-111259202	OtA5088-OtA5092	OtA5093-OtA5097
Gnai2	chr9:107614137-107635342	OtA5088-OtA5092	OtA5093-OtA5097
Sdk2	chr11:113780789-114065951	OtA5088-OtA5092	OtA5093-OtA5097
Rabac1	chr7:24969749-24972728	OtA5088-OtA5092	OtA5093-OtA5097
Brd2	chr17:34112018-34122607	OtA5088-OtA5092	OtA5093-OtA5097
Hist1h3a	chr13:23761852-23762386	OtA5088-OtA5092	OtA5093-OtA5097
Rgs12	chr5:34949447-35033593	OtA5088-OtA5092	OtA5093-OtA5097
Traf5	chr1:192054349-192092564	OtA5088-OtA5092	OtA5093-OtA5097
Mettl5	chr2:69861561-69885604	OtA5088-OtA5092	OtA5093-OtA5097
Wdr82	chr9:106170928-106191706	OtA5088-OtA5092	OtA5093-OtA5097
Gpam	chr19:55069733-55099447	OtA5088-OtA5092	OtA5093-OtA5097
Fgfr4	chr13:55152817-55168759	OtA5088-OtA5092	OtA5093-OtA5097
Arid5a	chr1:36307732-36324029	OtA5088-OtA5092	OtA5093-OtA5097

Asb3	chr11:30954397-31102704	OtA5088-OtA5092	OtA5093-OtA5097
Cnn2	chr10:79697304-80369637	OtA5088-OtA5092	OtA5093-OtA5097
Msto1	chr3:88835230-88913950	OtA5088-OtA5092	OtA5093-OtA5097
Pi4ka	chr16:17280350-17430826	OtA5088-OtA5092	OtA5093-OtA5097
Rnf24	chr2:131127411-131604824	OtA5088-OtA5092	OtA5093-OtA5097
Slc26a6	chr9:108854042-108862143	OtA5088-OtA5092	OtA5093-OtA5097
Gmcl1	chr6:86691767-86733378	OtA5088-OtA5092	OtA5093-OtA5097
Trp53	chr11:69580358-69591873	OtA5088-OtA5092	OtA5093-OtA5097
Adam9	chr8:24949610-25016922	OtA5088-OtA5092	OtA5093-OtA5097
Armc10	chr5:21645983-21662592	OtA5088-OtA5092	OtA5093-OtA5097
Bri3	chr5:144244436-144358112	OtA5088-OtA5092	OtA5093-OtA5097
Chkb	chr15:89416404-89429927	OtA5088-OtA5092	OtA5093-OtA5097
Comtd1	chr14:21831560-21848910	OtA5088-OtA5092	OtA5093-OtA5097
D16Ertd472e	chr16:78540335-78576688	OtA5088-OtA5092	OtA5093-OtA5097
Dusp8	chr7:142079486-142095284	OtA5088-OtA5092	OtA5093-OtA5097
Ece1	chr4:137862236-137965229	OtA5088-OtA5092	OtA5093-OtA5097
Egln2	chr7:27158657-27166802	OtA5088-OtA5092	OtA5093-OtA5097
Fxn	chr19:24261452-24280586	OtA5088-OtA5092	OtA5093-OtA5097
Immt	chr6:71831319-71875266	OtA5088-OtA5092	OtA5093-OtA5097
Msrb3	chr10:120781100-120898971	OtA5088-OtA5092	OtA5093-OtA5097
Mst1	chr9:108080435-108085027	OtA5088-OtA5092	OtA5093-OtA5097
Nol12	chr15:78934932-78941910	OtA5088-OtA5092	OtA5093-OtA5097
Ociad1	chr5:73292793-73314077	OtA5088-OtA5092	OtA5093-OtA5097
Oxnad1	chr14:32085727-32103203	OtA5088-OtA5092	OtA5093-OtA5097
Rab30	chr7:92741713-92837117	OtA5088-OtA5092	OtA5093-OtA5097
Rpl36al	chr12:69182733-69184067	OtA5088-OtA5092	OtA5093-OtA5097
Scrn2	chr11:97029951-97033960	OtA5088-OtA5092	OtA5093-OtA5097
Tbccd1	chr16:22813214-22857569	OtA5088-OtA5092	OtA5093-OtA5097
Trim3	chr7:105610469-105633499	OtA5088-OtA5092	OtA5093-OtA5097
Ywhaz	chr15:36770261-36796929	OtA5088-OtA5092	OtA5093-OtA5097
Slc25a2	chr18:36930284-37841872	OtA5088-OtA5092	OtA5093-OtA5097
Tex261	chr6:83770413-83775812	OtA5088-OtA5092	OtA5093-OtA5097
Lsp1	chr7:142460869-142516009	OtA5088-OtA5092	OtA5093-OtA5097
Ndufs7	chr10:79697304-80369637	OtA5088-OtA5092	OtA5093-OtA5097
Chchd2	chr5:129881160-129887470	OtA5088-OtA5092	OtA5093-OtA5097
Clic5	chr17:44188571-44280168	OtA5088-OtA5092	OtA5093-OtA5097
Fbln7	chr2:128863931-128897034	OtA5088-OtA5092	OtA5093-OtA5097
Igsf3	chr3:101377124-101463060	OtA5088-OtA5092	OtA5093-OtA5097
Sec24c	chr14:20674320-20694850	OtA5088-OtA5092	OtA5093-OtA5097
Sgcb	chr5:73632748-73647731	OtA5088-OtA5092	OtA5093-OtA5097
Slc35c1	chr2:92452764-92460518	OtA5088-OtA5092	OtA5093-OtA5097
Srp19	chr18:34331144-34336599	OtA5088-OtA5092	OtA5093-OtA5097
Csdc2	chr15:81936758-81950941	OtA5088-OtA5092	OtA5093-OtA5097
Nek4	chr14:30951487-30987401	OtA5088-OtA5092	OtA5093-OtA5097
Sema3f	chr9:107681501-107710475	OtA5088-OtA5092	OtA5093-OtA5097
Uck2	chr1:167226083-167285127	OtA5088-OtA5092	OtA5093-OtA5097
Ccdc72	chr9:109077987-109082381	OtA5088-OtA5092	OtA5093-OtA5097

Haus8	chr8:71251123-71272590	OtA5088-OtA5092	OtA5093-OtA5097
Ints4	chr7:97480955-97541398	OtA5088-OtA5092	OtA5093-OtA5097
Kdelc2	chr9:53384022-53401867	OtA5088-OtA5092	OtA5093-OtA5097
Nox4	chr7:87246794-87398606	OtA5088-OtA5092	OtA5093-OtA5097
Abhd15	chr11:77515116-77520628	OtA5088-OtA5092	OtA5093-OtA5097
Armcx2	chrX:134804141-134809221	OtA5088-OtA5092	OtA5093-OtA5097
Atp6v0a4	chr6:38048482-38254009	OtA5088-OtA5092	OtA5093-OtA5097
Bcdin3d	chr15:99470083-99474730	OtA5088-OtA5092	OtA5093-OtA5097
Casp2	chr6:42265038-42282496	OtA5088-OtA5092	OtA5093-OtA5097
Cdc26	chr4:62394588-62408623	OtA5088-OtA5092	OtA5093-OtA5097
Ces2b	chr8:104831643-104838652	OtA5088-OtA5092	OtA5093-OtA5097
Chd1l	chr3:97560747-97610190	OtA5088-OtA5092	OtA5093-OtA5097
Cox19	chr5:139337821-139345166	OtA5088-OtA5092	OtA5093-OtA5097
Ctbs	chr3:146450492-146499753	OtA5088-OtA5092	OtA5093-OtA5097
Ctdspl2	chr2:121956452-122013586	OtA5088-OtA5092	OtA5093-OtA5097
Cuedc2	chr19:46328183-46338660	OtA5088-OtA5092	OtA5093-OtA5097
Cyp26b1	chr6:84571413-84593908	OtA5088-OtA5092	OtA5093-OtA5097
Dtnbp1	chr13:44922079-45002096	OtA5088-OtA5092	OtA5093-OtA5097
Epc1	chr18:6435950-6516087	OtA5088-OtA5092	OtA5093-OtA5097
F2rl3	chr8:72761879-72763885	OtA5088-OtA5092	OtA5093-OtA5097
Fam86	chr16:5233620-5255956	OtA5088-OtA5092	OtA5093-OtA5097
Gpr77	chr7:16234584-16244154	OtA5088-OtA5092	OtA5093-OtA5097
Gtf2h5	chr17:6079827-6085485	OtA5088-OtA5092	OtA5093-OtA5097
Hist4h4	chr6:136801552-136804431	OtA5088-OtA5092	OtA5093-OtA5097
Kifc2	chr15:76660640-76668196	OtA5088-OtA5092	OtA5093-OtA5097
Lpcat1	chr13:73467382-73514538	OtA5088-OtA5092	OtA5093-OtA5097
Lrfn1	chr7:28451984-28482345	OtA5088-OtA5092	OtA5093-OtA5097
Lyar	chr5:38220481-38234306	OtA5088-OtA5092	OtA5093-OtA5097
Mkrn1	chr6:39397820-39420369	OtA5088-OtA5092	OtA5093-OtA5097
Mlh3	chr12:85234519-85270564	OtA5088-OtA5092	OtA5093-OtA5097
Mrpl24	chr3:87919543-87930195	OtA5088-OtA5092	OtA5093-OtA5097
Mtap6	chr7:99267446-99337137	OtA5088-OtA5092	OtA5093-OtA5097
Musk	chr4:58285961-58374303	OtA5088-OtA5092	OtA5093-OtA5097
Nos1	chr5:117867118-117953813	OtA5088-OtA5092	OtA5093-OtA5097
Pdcd6ip	chr9:113651743-113708259	OtA5088-OtA5092	OtA5093-OtA5097
Pdzd11	chrX:100622905-100625907	OtA5088-OtA5092	OtA5093-OtA5097
Pgcp	chr15:33083128-33653436	OtA5088-OtA5092	OtA5093-OtA5097
Plp2	chrX:7658532-7671278	OtA5088-OtA5092	OtA5093-OtA5097
Polr2l	chr7:141471859-141475153	OtA5088-OtA5092	OtA5093-OtA5097
Ppp1r9b	chr11:94991211-95006898	OtA5088-OtA5092	OtA5093-OtA5097
Prl8a1	chr13:27573921-27582171	OtA5088-OtA5092	OtA5093-OtA5097
Prmt10	chr8:77549396-77581338	OtA5088-OtA5092	OtA5093-OtA5097
Ptgds	chr2:25466711-25469749	OtA5088-OtA5092	OtA5093-OtA5097
Qsox1	chr1:155778154-155812899	OtA5088-OtA5092	OtA5093-OtA5097
Rpl32	chr6:115774556-115808743	OtA5088-OtA5092	OtA5093-OtA5097
Sars2	chr7:28741967-28753879	OtA5088-OtA5092	OtA5093-OtA5097
Sgsm3	chr15:80977764-81190757	OtA5088-OtA5092	OtA5093-OtA5097

Taf4b	chr18:14783244-14900359	OtA5088-OtA5092	OtA5093-OtA5097
Tcn2	chr11:3917077-3932078	OtA5088-OtA5092	OtA5093-OtA5097
Tm7sf3	chr6:146602275-146634592	OtA5088-OtA5092	OtA5093-OtA5097
Tmem123	chr9:7764076-7794332	OtA5088-OtA5092	OtA5093-OtA5097
Tnfrsf23	chr7:143665806-143685872	OtA5088-OtA5092	OtA5093-OtA5097
Txndc17	chr11:72207553-72210487	OtA5088-OtA5092	OtA5093-OtA5097
Ube2r2	chr4:41136020-41193370	OtA5088-OtA5092	OtA5093-OtA5097
Ube4a	chr9:44923126-44965600	OtA5088-OtA5092	OtA5093-OtA5097
Wnt5b	chr6:119432530-119544347	OtA5088-OtA5092	OtA5093-OtA5097
Zfp319	chr8:95326135-95331950	OtA5088-OtA5092	OtA5093-OtA5097
Mmp28	chr11:83441875-83462961	OtA5088-OtA5092	OtA5093-OtA5097
Ash2l	chr8:25816869-25847670	OtA5088-OtA5092	OtA5093-OtA5097
Akirin2	chr4:34550614-34614942	OtA5088-OtA5092	OtA5093-OtA5097
BC017158	chr7:128265696-128298131	OtA5088-OtA5092	OtA5093-OtA5097
Btbd11	chr10:85386813-85660292	OtA5088-OtA5092	OtA5093-OtA5097
Ccdc130	chr8:84257794-84270360	OtA5088-OtA5092	OtA5093-OtA5097
Cdc42ep5	chr7:4151259-4164699	OtA5088-OtA5092	OtA5093-OtA5097
Cenpv	chr11:62524943-62539261	OtA5088-OtA5092	OtA5093-OtA5097
Fam118b	chr9:35211202-35267780	OtA5088-OtA5092	OtA5093-OtA5097
Ganc	chr2:120355308-120504919	OtA5088-OtA5092	OtA5093-OtA5097
Gfm2	chr13:97137936-97198357	OtA5088-OtA5092	OtA5093-OtA5097
Gpr160	chr3:30855949-30897192	OtA5088-OtA5092	OtA5093-OtA5097
Hnf1a	chr5:114948979-114971062	OtA5088-OtA5092	OtA5093-OtA5097
Hnrnpc	chr14:52073379-52104028	OtA5088-OtA5092	OtA5093-OtA5097
Hspb11	chr4:107253933-107279888	OtA5088-OtA5092	OtA5093-OtA5097
Magt1	chrX:105968084-106011899	OtA5088-OtA5092	OtA5093-OtA5097
Mrpl40	chr16:18872017-18876637	OtA5088-OtA5092	OtA5093-OtA5097
Mrpl48	chr7:100549116-100641268	OtA5088-OtA5092	OtA5093-OtA5097
Ndufs6	chr13:73319875-73328482	OtA5088-OtA5092	OtA5093-OtA5097
Nlrx1	chr9:44252712-44268599	OtA5088-OtA5092	OtA5093-OtA5097
Nup93	chr8:94214600-94315066	OtA5088-OtA5092	OtA5093-OtA5097
Pde2a	chr7:101421690-101512829	OtA5088-OtA5092	OtA5093-OtA5097
Phyh	chr2:4918995-4938743	OtA5088-OtA5092	OtA5093-OtA5097
Rchy1	chr5:91948903-91972137	OtA5088-OtA5092	OtA5093-OtA5097
Ripk1	chr13:34002873-34035170	OtA5088-OtA5092	OtA5093-OtA5097
Rpl17	chr18:75000476-75003381	OtA5088-OtA5092	OtA5093-OtA5097
Rtp4	chr16:23609918-23614222	OtA5088-OtA5092	OtA5093-OtA5097
Snrpa	chr7:27187005-27204320	OtA5088-OtA5092	OtA5093-OtA5097
Ss18l1	chr2:180042482-180070201	OtA5088-OtA5092	OtA5093-OtA5097
Tbc1d16	chr11:119143042-119228499	OtA5088-OtA5092	OtA5093-OtA5097
Tgfbrap1	chr1:43047268-43098622	OtA5088-OtA5092	OtA5093-OtA5097
Top3b	chr16:16870890-16892986	OtA5088-OtA5092	OtA5093-OtA5097
Xylt2	chr11:94663846-94677493	OtA5088-OtA5092	OtA5093-OtA5097
Amdhd2	chr17:24155832-24163733	OtA5088-OtA5092	OtA5093-OtA5097
Camsap3	chr8:3587449-3609075	OtA5088-OtA5092	OtA5093-OtA5097
Elof1	chr9:22055459-22389206	OtA5088-OtA5092	OtA5093-OtA5097
Jam3	chr9:27097383-27155421	OtA5088-OtA5092	OtA5093-OtA5097

Xlr4c	chrX:73234075-73243130	OtA5088-OtA5092	OtA5093-OtA5097
B230312A22Rik	chr4:43032413-43046220	OtA5088-OtA5092	OtA5093-OtA5097
Abcb1a	chr5:8660091-8748570	OtA5088-OtA5092	OtA5093-OtA5097
Adck3	chr1:179961088-180196020	OtA5088-OtA5092	OtA5093-OtA5097
Atp5l	chr9:44913247-44920742	OtA5088-OtA5092	OtA5093-OtA5097
Atp8a1	chr5:67618140-67847431	OtA5088-OtA5092	OtA5093-OtA5097
Aup1	chr6:83054652-83057682	OtA5088-OtA5092	OtA5093-OtA5097
BC052040	chr2:115581715-115778768	OtA5088-OtA5092	OtA5093-OtA5097
Bre	chr5:31698049-32084739	OtA5088-OtA5092	OtA5093-OtA5097
Btbd3	chr2:138256583-138287422	OtA5088-OtA5092	OtA5093-OtA5097
Camta1	chr4:151059522-151861768	OtA5088-OtA5092	OtA5093-OtA5097
Ccdc157	chr11:4135159-4160293	OtA5088-OtA5092	OtA5093-OtA5097
Cds2	chr2:132263256-132312041	OtA5088-OtA5092	OtA5093-OtA5097
Crispld2	chr8:119992469-120052793	OtA5088-OtA5092	OtA5093-OtA5097
Cse1l	chr2:166906095-166946389	OtA5088-OtA5092	OtA5093-OtA5097
Dmrta2	chr4:109978024-109983684	OtA5088-OtA5092	OtA5093-OtA5097
Dnajb4	chr3:152183954-152210299	OtA5088-OtA5092	OtA5093-OtA5097
Dyrk2	chr10:118859348-118868903	OtA5088-OtA5092	OtA5093-OtA5097
E130307A14Rik	chr10:39612933-39732007	OtA5088-OtA5092	OtA5093-OtA5097
Erbb3	chr10:128569367-128589501	OtA5088-OtA5092	OtA5093-OtA5097
Extl1	chr4:134356372-134372547	OtA5088-OtA5092	OtA5093-OtA5097
Fbxl2	chr9:113930933-114026751	OtA5088-OtA5092	OtA5093-OtA5097
Golph3	chr15:12321495-12351267	OtA5088-OtA5092	OtA5093-OtA5097
Kalrn	chr16:33969072-34514027	OtA5088-OtA5092	OtA5093-OtA5097
Llgl1	chr11:60699689-60727221	OtA5088-OtA5092	OtA5093-OtA5097
Mks1	chr11:87853224-87863679	OtA5088-OtA5092	OtA5093-OtA5097
Mllt3	chr4:87769924-88033407	OtA5088-OtA5092	OtA5093-OtA5097
Mrpl30	chr1:37890552-37898333	OtA5088-OtA5092	OtA5093-OtA5097
Mtfp1	chr11:4091480-4095431	OtA5088-OtA5092	OtA5093-OtA5097
Mthfr	chr4:148039076-148059562	OtA5088-OtA5092	OtA5093-OtA5097
Nipa1	chr7:55962530-56019573	OtA5088-OtA5092	OtA5093-OtA5097
Notch1	chr2:26457901-26503822	OtA5088-OtA5092	OtA5093-OtA5097
Numb	chr12:83795438-83842343	OtA5088-OtA5092	OtA5093-OtA5097
Pdcd10	chr3:75516489-75556799	OtA5088-OtA5092	OtA5093-OtA5097
Pdxk	chr10:78436746-78464948	OtA5088-OtA5092	OtA5093-OtA5097
Polr1a	chr6:71909052-71979360	OtA5088-OtA5092	OtA5093-OtA5097
Ppp3ca	chr3:136670769-136935779	OtA5088-OtA5092	OtA5093-OtA5097
Ppp4r1	chr17:65783354-65841926	OtA5088-OtA5092	OtA5093-OtA5097
Ptpn2	chr18:67665500-67724621	OtA5088-OtA5092	OtA5093-OtA5097
Rad51l3	chr11:82873064-82890615	OtA5088-OtA5092	OtA5093-OtA5097
Rps16	chr7:28350688-28352698	OtA5088-OtA5092	OtA5093-OtA5097
Rtn2	chr7:19282666-19296160	OtA5088-OtA5092	OtA5093-OtA5097
Samd4	chr14:46882964-47105817	OtA5088-OtA5092	OtA5093-OtA5097
Sav1	chr12:69746847-69987002	OtA5088-OtA5092	OtA5093-OtA5097
Sod1	chr16:90220741-90226324	OtA5088-OtA5092	OtA5093-OtA5097
Stom	chr2:35313989-35337009	OtA5088-OtA5092	OtA5093-OtA5097
Stub1	chr17:25829042-25833361	OtA5088-OtA5092	OtA5093-OtA5097

Tctn1	chr5:122209736-122264460	OtA5088-OtA5092	OtA5093-OtA5097
Tdrd1	chr19:56826208-56870012	OtA5088-OtA5092	OtA5093-OtA5097
Tgfb3	chr12:86056742-86079041	OtA5088-OtA5092	OtA5093-OtA5097
Tm6sf1	chr7:81859021-81934459	OtA5088-OtA5092	OtA5093-OtA5097
Tmed5	chr5:108121646-108132591	OtA5088-OtA5092	OtA5093-OtA5097
Tmem37	chr1:120067376-120073780	OtA5088-OtA5092	OtA5093-OtA5097
Tmtc1	chr6:148047415-148444352	OtA5088-OtA5092	OtA5093-OtA5097
Tymp	chr15:89355721-89377037	OtA5088-OtA5092	OtA5093-OtA5097
Ubl5	chr9:20637786-20646789	OtA5088-OtA5092	OtA5093-OtA5097
Uqcc	chr2:155846885-155930310	OtA5088-OtA5092	OtA5093-OtA5097
Ybx1	chr4:119277326-119294513	OtA5088-OtA5092	OtA5093-OtA5097
Zfand6	chr7:84615053-84679351	OtA5088-OtA5092	OtA5093-OtA5097
Zfp275	chrX:73342619-73359080	OtA5088-OtA5092	OtA5093-OtA5097
Zfp622	chr15:25984365-25998482	OtA5088-OtA5092	OtA5093-OtA5097
Rps3	chr7:99477896-99483709	OtA5088-OtA5092	OtA5093-OtA5097
Dgkh	chr14:78569608-78725089	OtA5088-OtA5092	OtA5093-OtA5097
Mrc1	chr2:14229413-14332023	OtA5088-OtA5092	OtA5093-OtA5097
Sh3gl3	chr7:82260128-82307417	OtA5088-OtA5092	OtA5093-OtA5097
Lig1	chr7:13258966-13311427	OtA5088-OtA5092	OtA5093-OtA5097
BC048403	chr10:121739936-121752859	OtA5088-OtA5092	OtA5093-OtA5097
Adora2b	chr11:62248983-62266452	OtA5088-OtA5092	OtA5093-OtA5097
Arl6ip4	chr5:124116107-124118195	OtA5088-OtA5092	OtA5093-OtA5097
Armcx5	chrX:135742691-135803468	OtA5088-OtA5092	OtA5093-OtA5097
Elmo1	chr13:20090506-20608353	OtA5088-OtA5092	OtA5093-OtA5097
Igsf5	chr16:96361756-96422121	OtA5088-OtA5092	OtA5093-OtA5097
Ntpcr	chr8:125734202-125748235	OtA5088-OtA5092	OtA5093-OtA5097
Prepl	chr17:85028346-85090274	OtA5088-OtA5092	OtA5093-OtA5097
Scube3	chr17:28142525-28171345	OtA5088-OtA5092	OtA5093-OtA5097
Fosl1	chr19:5447697-5457563	OtA5088-OtA5092	OtA5093-OtA5097
Echdc3	chr2:6188464-6321611	OtA5088-OtA5092	OtA5093-OtA5097
Nop16	chr13:54584190-54590074	OtA5088-OtA5092	OtA5093-OtA5097
Pacs1	chr19:5133684-5273119	OtA5088-OtA5092	OtA5093-OtA5097
Ptar1	chr19:23687399-23721129	OtA5088-OtA5092	OtA5093-OtA5097
Ccdc137	chr11:120458128-120467600	OtA5088-OtA5092	OtA5093-OtA5097
Kif21b	chr1:136131400-136178014	OtA5088-OtA5092	OtA5093-OtA5097
Calm1	chr12:100199540-100209806	OtA5088-OtA5092	OtA5093-OtA5097
Dnajc25	chr4:59003192-59023398	OtA5088-OtA5092	OtA5093-OtA5097
Evi5l	chr8:4166566-4193701	OtA5088-OtA5092	OtA5093-OtA5097
Hfe	chr13:23703840-23710811	OtA5088-OtA5092	OtA5093-OtA5097
Kdelr3	chr15:79516407-79546741	OtA5088-OtA5092	OtA5093-OtA5097
Myzap	chr9:71504346-71592360	OtA5088-OtA5092	OtA5093-OtA5097
Ptpmt1	chr2:90910712-90918050	OtA5088-OtA5092	OtA5093-OtA5097
Rtn3	chr19:7417624-7483289	OtA5088-OtA5092	OtA5093-OtA5097
Setd4	chr16:93583460-93603815	OtA5088-OtA5092	OtA5093-OtA5097
Yipf3	chr17:46248079-46252537	OtA5088-OtA5092	OtA5093-OtA5097
Ppp1r8	chr4:132826924-132843169	OtA5088-OtA5092	OtA5093-OtA5097
Dusp9	chrX:73639440-73643514	OtA5088-OtA5092	OtA5093-OtA5097

Cdyl2	chr8:116568723-116732991	OtA5088-OtA5092	OtA5093-OtA5097
Gapvd1	chr2:34676982-34755232	OtA5088-OtA5092	OtA5093-OtA5097
S100a9	chr3:90692632-90695691	OtA5088-OtA5092	OtA5093-OtA5097
Vipar	chr12:87238874-87266286	OtA5088-OtA5092	OtA5093-OtA5097
Cdr2	chr7:120957036-120982312	OtA5088-OtA5092	OtA5093-OtA5097
Eif4h	chr5:134619875-134639328	OtA5088-OtA5092	OtA5093-OtA5097
Paqr4	chr17:23726335-23740330	OtA5088-OtA5092	OtA5093-OtA5097
Slc39a4	chr15:76612382-76616852	OtA5088-OtA5092	OtA5093-OtA5097
Snrpd2	chr7:19149837-19152726	OtA5088-OtA5092	OtA5093-OtA5097
Med12	chrX:101274090-101298934	OtA5088-OtA5092	OtA5093-OtA5097
Tmed2	chr5:124540790-124550503	OtA5088-OtA5092	OtA5093-OtA5097
Cic	chr7:25282178-25294148	OtA5088-OtA5092	OtA5093-OtA5097
Ide	chr19:37268740-37330613	OtA5088-OtA5092	OtA5093-OtA5097
Adcy6	chr15:98589985-98607633	OtA5088-OtA5092	OtA5093-OtA5097
Eme2	chr17:24892151-24895087	OtA5088-OtA5092	OtA5093-OtA5097
Por	chr5:135689213-135735325	OtA5088-OtA5092	OtA5093-OtA5097
Tcp1	chr17:12916464-12940396	OtA5088-OtA5092	OtA5093-OtA5097
Tmx4	chr2:134594501-134644121	OtA5088-OtA5092	OtA5093-OtA5097
Tns4	chr11:99065677-99089306	OtA5088-OtA5092	OtA5093-OtA5097
Erp44	chr4:48193330-48279589	OtA5088-OtA5092	OtA5093-OtA5097
Aars2	chr17:45506840-45520843	OtA5088-OtA5092	OtA5093-OtA5097
Hspa13	chr16:75755190-75766818	OtA5088-OtA5092	OtA5093-OtA5097
Stxbp5	chr10:9755546-9901040	OtA5088-OtA5092	OtA5093-OtA5097
Zfp219	chr14:51984832-52020733	OtA5088-OtA5092	OtA5093-OtA5097
Ahctf1	chr1:179744903-179804015	OtA5088-OtA5092	OtA5093-OtA5097
Apbb1ip	chr2:22774326-22875653	OtA5088-OtA5092	OtA5093-OtA5097
Atg10	chr13:90935348-91223987	OtA5088-OtA5092	OtA5093-OtA5097
Bag4	chr8:25764538-25785209	OtA5088-OtA5092	OtA5093-OtA5097
C1ra	chr6:124512620-124523440	OtA5088-OtA5092	OtA5093-OtA5097
Casp7	chr19:56397128-56442343	OtA5088-OtA5092	OtA5093-OtA5097
Casp8	chr1:58795373-58847503	OtA5088-OtA5092	OtA5093-OtA5097
Chordc1	chr9:18292266-18314000	OtA5088-OtA5092	OtA5093-OtA5097
Cnrip1	chr11:17051933-17079372	OtA5088-OtA5092	OtA5093-OtA5097
Cyb5r3	chr15:83153500-83172208	OtA5088-OtA5092	OtA5093-OtA5097
Dynlrb1	chr2:155236532-155250277	OtA5088-OtA5092	OtA5093-OtA5097
Eef1d	chr15:75894799-75909340	OtA5088-OtA5092	OtA5093-OtA5097
Ehmt1	chr2:24790772-24919590	OtA5088-OtA5092	OtA5093-OtA5097
Endog	chr2:30171523-30178459	OtA5088-OtA5092	OtA5093-OtA5097
Erf	chr7:25242559-25250758	OtA5088-OtA5092	OtA5093-OtA5097
Fam188b	chr6:55203382-55320222	OtA5088-OtA5092	OtA5093-OtA5097
Fbxo42	chr4:141147921-141204062	OtA5088-OtA5092	OtA5093-OtA5097
Fxyd6	chr9:45370184-45396159	OtA5088-OtA5092	OtA5093-OtA5097
Gins4	chr8:23226609-23237668	OtA5088-OtA5092	OtA5093-OtA5097
Gm505	chr8:124897885-124903813	OtA5088-OtA5092	OtA5093-OtA5097
Gpihbp1	chr15:75596657-75598213	OtA5088-OtA5092	OtA5093-OtA5097
Gt(ROSA)26Sor	chr6:113046326-113077244	OtA5088-OtA5092	OtA5093-OtA5097
Gtf3a	chr5:146948656-146963797	OtA5088-OtA5092	OtA5093-OtA5097

Haus2	chr2:120604237-120620818	OtA5088-OtA5092	OtA5093-OtA5097
Hps5	chr7:46760465-46795881	OtA5088-OtA5092	OtA5093-OtA5097
Ifitm3	chr7:141009589-141010744	OtA5088-OtA5092	OtA5093-OtA5097
Ino80c	chr18:24104760-24121819	OtA5088-OtA5092	OtA5093-OtA5097
Iqck	chr7:118842217-118995211	OtA5088-OtA5092	OtA5093-OtA5097
Kif26a	chr12:112146207-112181747	OtA5088-OtA5092	OtA5093-OtA5097
Leprel1	chr16:25960322-26105784	OtA5088-OtA5092	OtA5093-OtA5097
Lrg1	chr17:56119677-56121946	OtA5088-OtA5092	OtA5093-OtA5097
Lrrc40	chr3:158036702-158068478	OtA5088-OtA5092	OtA5093-OtA5097
Lrrc56	chr7:141194129-141214080	OtA5088-OtA5092	OtA5093-OtA5097
Map2k1	chr9:64185792-64253605	OtA5088-OtA5092	OtA5093-OtA5097
Mier2	chr10:79540244-79555091	OtA5088-OtA5092	OtA5093-OtA5097
Mina	chr16:59471774-59555752	OtA5088-OtA5092	OtA5093-OtA5097
Mtap	chr4:89137369-89181090	OtA5088-OtA5092	OtA5093-OtA5097
Mthfd1l	chr10:3973074-4167081	OtA5088-OtA5092	OtA5093-OtA5097
Ndufaf3	chr9:108565864-108567342	OtA5088-OtA5092	OtA5093-OtA5097
Nfx1	chr4:40970942-41025992	OtA5088-OtA5092	OtA5093-OtA5097
Oas1b	chr5:120812634-120824160	OtA5088-OtA5092	OtA5093-OtA5097
Opn3	chr1:175662427-175692590	OtA5088-OtA5092	OtA5093-OtA5097
Ost4	chr5:30888851-30907788	OtA5088-OtA5092	OtA5093-OtA5097
Pfkfb4	chr9:108991901-109032225	OtA5088-OtA5092	OtA5093-OtA5097
Psg26	chr7:18474581-18484149	OtA5088-OtA5092	OtA5093-OtA5097
Psmb6	chr11:70525356-70527858	OtA5088-OtA5092	OtA5093-OtA5097
Ptger4	chr15:5233403-5243831	OtA5088-OtA5092	OtA5093-OtA5097
Rad23b	chr4:55350041-55392237	OtA5088-OtA5092	OtA5093-OtA5097
Ranbp9	chr13:43398375-43480973	OtA5088-OtA5092	OtA5093-OtA5097
Rnf144b	chr13:47122719-47247991	OtA5088-OtA5092	OtA5093-OtA5097
Rnpep	chr1:135262698-135284084	OtA5088-OtA5092	OtA5093-OtA5097
Rufy2	chr10:62980222-63023849	OtA5088-OtA5092	OtA5093-OtA5097
S1pr1	chr3:115710432-115715055	OtA5088-OtA5092	OtA5093-OtA5097
Scube2	chr7:109798690-109865679	OtA5088-OtA5092	OtA5093-OtA5097
Slc25a23	chr17:57043710-57059863	OtA5088-OtA5092	OtA5093-OtA5097
Slc27a6	chr18:58556239-58612869	OtA5088-OtA5092	OtA5093-OtA5097
Slc35a4	chr18:36671158-36683862	OtA5088-OtA5092	OtA5093-OtA5097
Slc35e4	chr11:3907021-3914664	OtA5088-OtA5092	OtA5093-OtA5097
Smagp	chr15:100621339-100636865	OtA5088-OtA5092	OtA5093-OtA5097
Snora26	chr5:74093082-74094336	OtA5088-OtA5092	OtA5093-OtA5097
Stard3	chr11:98358367-98381112	OtA5088-OtA5092	OtA5093-OtA5097
Syap1	chrX:162407199-163352564	OtA5088-OtA5092	OtA5093-OtA5097
Tbk1	chr10:121546455-121586794	OtA5088-OtA5092	OtA5093-OtA5097
Tbkbp1	chr11:97136170-97149712	OtA5088-OtA5092	OtA5093-OtA5097
Tecr	chr8:83571697-83594491	OtA5088-OtA5092	OtA5093-OtA5097
Tmem191c	chr16:17276299-17278661	OtA5088-OtA5092	OtA5093-OtA5097
Tpd52	chr3:8929435-9004515	OtA5088-OtA5092	OtA5093-OtA5097
Vma21	chrX:71816778-71824706	OtA5088-OtA5092	OtA5093-OtA5097
Xrcc3	chr12:111758848-111813841	OtA5088-OtA5092	OtA5093-OtA5097
Zcchc24	chr14:25711639-25768856	OtA5088-OtA5092	OtA5093-OtA5097

Zfp191	chr18:24012266-24020771	OtA5088-OtA5092	OtA5093-OtA5097
Zfp384	chr6:125009237-125037870	OtA5088-OtA5092	OtA5093-OtA5097
Zfp444	chr7:6172512-6193104	OtA5088-OtA5092	OtA5093-OtA5097
Fam162b	chr10:51585449-51590460	OtA5088-OtA5092	OtA5093-OtA5097
Herc2	chr7:56050154-56231798	OtA5088-OtA5092	OtA5093-OtA5097
Tacc1	chr8:25154551-25201449	OtA5088-OtA5092	OtA5093-OtA5097
Ttc22	chr4:106622448-106640187	OtA5088-OtA5092	OtA5093-OtA5097
Rela	chr19:5637489-5648130	OtA5088-OtA5092	OtA5093-OtA5097
Dom3z	chr17:34837018-34839186	OtA5088-OtA5092	OtA5093-OtA5097
Fam102a	chr2:32535358-32569750	OtA5088-OtA5092	OtA5093-OtA5097
Fam173a	chr17:25786579-25792284	OtA5088-OtA5092	OtA5093-OtA5097
Ghitm	chr14:37120444-37135322	OtA5088-OtA5092	OtA5093-OtA5097
Glrx3	chr7:137437647-137468594	OtA5088-OtA5092	OtA5093-OtA5097
Gmps	chr3:63976142-64019078	OtA5088-OtA5092	OtA5093-OtA5097
Golph3l	chr3:95588933-95619247	OtA5088-OtA5092	OtA5093-OtA5097
H2-Ke6	chr17:34026032-34028055	OtA5088-OtA5092	OtA5093-OtA5097
Hdac4	chr1:91932748-92148393	OtA5088-OtA5092	OtA5093-OtA5097
Nckap1	chr2:80501294-80580965	OtA5088-OtA5092	OtA5093-OtA5097
Pcdhgb6	chr18:36930284-37841872	OtA5088-OtA5092	OtA5093-OtA5097
Serpinb1a	chr13:32842091-32851185	OtA5088-OtA5092	OtA5093-OtA5097
Sugt1	chr14:79587690-79629755	OtA5088-OtA5092	OtA5093-OtA5097
Tmed10	chr12:85340613-85374717	OtA5088-OtA5092	OtA5093-OtA5097
Ube2e3	chr2:78869046-78920583	OtA5088-OtA5092	OtA5093-OtA5097
Pold2	chr11:5861865-5878256	OtA5088-OtA5092	OtA5093-OtA5097
Abhd11	chr5:135009151-135013157	OtA5088-OtA5092	OtA5093-OtA5097
Adat2	chr10:13523841-13563376	OtA5088-OtA5092	OtA5093-OtA5097
Ddx1	chr12:13219306-13249174	OtA5088-OtA5092	OtA5093-OtA5097
Jund	chr8:70697738-70700616	OtA5088-OtA5092	OtA5093-OtA5097
P4htm	chr9:108578825-108597600	OtA5088-OtA5092	OtA5093-OtA5097
Poll	chr19:45552275-45560543	OtA5088-OtA5092	OtA5093-OtA5097
Sel1l3	chr5:53107082-53213452	OtA5088-OtA5092	OtA5093-OtA5097
Trim30a	chr7:104409025-104465193	OtA5088-OtA5092	OtA5093-OtA5097
Tubgcp2	chr7:139995954-140036350	OtA5088-OtA5092	OtA5093-OtA5097
Il18bp	chr7:102015076-102018155	OtA5088-OtA5092	OtA5093-OtA5097
S100g	chrX:162407199-163352564	OtA5088-OtA5092	OtA5093-OtA5097
Lamc2	chr1:153122755-153186447	OtA5088-OtA5092	OtA5093-OtA5097
Rab11a	chr9:64715299-64737756	OtA5088-OtA5092	OtA5093-OtA5097
Polr2e	chr10:79697304-80369637	OtA5088-OtA5092	OtA5093-OtA5097
Smug1	chr15:103153289-103163284	OtA5088-OtA5092	OtA5093-OtA5097
Fstl1	chr16:37777054-37836516	OtA5088-OtA5092	OtA5093-OtA5097
Tmem135	chr7:89139720-89338787	OtA5088-OtA5092	OtA5093-OtA5097
Pusl1	chr4:155869566-155891762	OtA5088-OtA5092	OtA5093-OtA5097
Tfap2a	chr13:40715674-40733823	OtA5088-OtA5092	OtA5093-OtA5097
Brms1	chr19:5041403-5049917	OtA5088-OtA5092	OtA5093-OtA5097
Slbp	chr5:33640061-33652306	OtA5088-OtA5092	OtA5093-OtA5097
Fam120a	chr13:48879216-48967828	OtA5088-OtA5092	OtA5093-OtA5097
Becn1	chr11:101279202-101302267	OtA5088-OtA5092	OtA5093-OtA5097

C2	chr17:34862601-34882100	OtA5088-OtA5092	OtA5093-OtA5097
Galnt3	chr2:66082765-66124793	OtA5088-OtA5092	OtA5093-OtA5097
Gbe1	chr16:70314086-70569716	OtA5088-OtA5092	OtA5093-OtA5097
Rae1	chr2:173000116-173015739	OtA5088-OtA5092	OtA5093-OtA5097
Wnt9a	chr11:59306929-59333552	OtA5088-OtA5092	OtA5093-OtA5097
Arfgap2	chr2:91265114-91277371	OtA5088-OtA5092	OtA5093-OtA5097
Arl2	chr19:6134388-6141137	OtA5088-OtA5092	OtA5093-OtA5097
Atf5	chr7:44812255-44849079	OtA5088-OtA5092	OtA5093-OtA5097
Atxn7l3	chr11:102284960-102296629	OtA5088-OtA5092	OtA5093-OtA5097
Btbd10	chr7:113315643-113369339	OtA5088-OtA5092	OtA5093-OtA5097
Caml	chr13:55623004-55632416	OtA5088-OtA5092	OtA5093-OtA5097
Ccne1	chr7:38097983-38107490	OtA5088-OtA5092	OtA5093-OtA5097
Cggbp1	chr16:64852084-64859491	OtA5088-OtA5092	OtA5093-OtA5097
Chac1	chr2:119351241-119354327	OtA5088-OtA5092	OtA5093-OtA5097
Coro2b	chr9:62419491-62537044	OtA5088-OtA5092	OtA5093-OtA5097
Ctr9	chr7:111028950-111056377	OtA5088-OtA5092	OtA5093-OtA5097
Cysltr2	chr14:73029127-73049114	OtA5088-OtA5092	OtA5093-OtA5097
Dnajc24	chr2:105966707-106003549	OtA5088-OtA5092	OtA5093-OtA5097
Epha4	chr1:77367184-77515088	OtA5088-OtA5092	OtA5093-OtA5097
Eppk1	chr15:76101487-76126556	OtA5088-OtA5092	OtA5093-OtA5097
Fahd1	chr17:24848895-24850302	OtA5088-OtA5092	OtA5093-OtA5097
Fjx1	chr2:102449365-102451792	OtA5088-OtA5092	OtA5093-OtA5097
Fzd7	chr1:59482146-59486955	OtA5088-OtA5092	OtA5093-OtA5097
Fzd8	chr18:9212855-9216201	OtA5088-OtA5092	OtA5093-OtA5097
G6pdx	chrX:74393290-74453778	OtA5088-OtA5092	OtA5093-OtA5097
Gdap10	chr12:32820334-32853369	OtA5088-OtA5092	OtA5093-OtA5097
Hadha	chr5:30103583-30154980	OtA5088-OtA5092	OtA5093-OtA5097
Lzic	chr4:149485332-149496667	OtA5088-OtA5092	OtA5093-OtA5097
Med16	chr10:79697304-80369637	OtA5088-OtA5092	OtA5093-OtA5097
Med9	chr11:59948213-59964942	OtA5088-OtA5092	OtA5093-OtA5097
Mrps11	chr7:78783130-78792988	OtA5088-OtA5092	OtA5093-OtA5097
Ndufs1	chr1:63114470-63176822	OtA5088-OtA5092	OtA5093-OtA5097
Nlk	chr11:78567167-78697425	OtA5088-OtA5092	OtA5093-OtA5097
Ormdl1	chr1:53297094-53310245	OtA5088-OtA5092	OtA5093-OtA5097
Peo1	chr19:45006557-45012762	OtA5088-OtA5092	OtA5093-OtA5097
Phgr1	chr2:118772768-118778164	OtA5088-OtA5092	OtA5093-OtA5097
Polr3k	chr2:181864359-181870826	OtA5088-OtA5092	OtA5093-OtA5097
Prkcsh	chr9:22003034-22014222	OtA5088-OtA5092	OtA5093-OtA5097
Psmf1	chr2:151716061-151741293	OtA5088-OtA5092	OtA5093-OtA5097
Ptpn4	chr1:119658092-119837071	OtA5088-OtA5092	OtA5093-OtA5097
Qdpr	chr5:45434031-45450229	OtA5088-OtA5092	OtA5093-OtA5097
Rab21	chr10:115289861-115315591	OtA5088-OtA5092	OtA5093-OtA5097
Rab5c	chr11:100715265-100738130	OtA5088-OtA5092	OtA5093-OtA5097
Rcn3	chr7:45082913-45092213	OtA5088-OtA5092	OtA5093-OtA5097
Rhbdf1	chr11:32209584-32222293	OtA5088-OtA5092	OtA5093-OtA5097
Sgms1	chr19:32122726-32388454	OtA5088-OtA5092	OtA5093-OtA5097
Slc50a1	chr3:89268245-89270570	OtA5088-OtA5092	OtA5093-OtA5097

Slc52a2	chr15:76538942-76542130	OtA5088-OtA5092	OtA5093-OtA5097
Smarca4	chr9:21616168-21704230	OtA5088-OtA5092	OtA5093-OtA5097
Snord15a	chr7:99477896-99483709	OtA5088-OtA5092	OtA5093-OtA5097
Snx16	chr3:10417816-10440124	OtA5088-OtA5092	OtA5093-OtA5097
Spag7	chr11:70663768-70669416	OtA5088-OtA5092	OtA5093-OtA5097
Tia1	chr6:86404218-86433405	OtA5088-OtA5092	OtA5093-OtA5097
Tmem106c	chr15:97964228-97970286	OtA5088-OtA5092	OtA5093-OtA5097
Tmem213	chr6:38048482-38254009	OtA5088-OtA5092	OtA5093-OtA5097
Tnfrsf14	chr4:154922209-154928077	OtA5088-OtA5092	OtA5093-OtA5097
Tprkb	chr6:85915718-85930284	OtA5088-OtA5092	OtA5093-OtA5097
Trnt1	chr6:106769137-106800074	OtA5088-OtA5092	OtA5093-OtA5097
Tulp3	chr6:128321160-128355851	OtA5088-OtA5092	OtA5093-OtA5097
Usp9x	chrX:13071497-13173327	OtA5088-OtA5092	OtA5093-OtA5097
Vps45	chr3:95999831-96058455	OtA5088-OtA5092	OtA5093-OtA5097
Wisp1	chr15:66891392-66923199	OtA5088-OtA5092	OtA5093-OtA5097
Ythdf2	chr4:132184915-132212256	OtA5088-OtA5092	OtA5093-OtA5097
Zfp60	chr7:27731408-27751689	OtA5088-OtA5092	OtA5093-OtA5097
Zfp707	chr15:75969184-75975865	OtA5088-OtA5092	OtA5093-OtA5097
Zfp791	chr8:85109166-85123095	OtA5088-OtA5092	OtA5093-OtA5097
Zfp874a	chr13:67440430-67451624	OtA5088-OtA5092	OtA5093-OtA5097
Zxdc	chr6:90369493-90403486	OtA5088-OtA5092	OtA5093-OtA5097
Abhd14a	chr9:106440050-106447678	OtA5088-OtA5092	OtA5093-OtA5097
Ccdc21	chr4:134129857-134187085	OtA5088-OtA5092	OtA5093-OtA5097
Ccdc58	chr16:36071659-36092118	OtA5088-OtA5092	OtA5093-OtA5097
Depdc7	chr2:104721786-104742801	OtA5088-OtA5092	OtA5093-OtA5097
Dnajc14	chr10:128805675-128821631	OtA5088-OtA5092	OtA5093-OtA5097
Dram2	chr3:106547826-106574845	OtA5088-OtA5092	OtA5093-OtA5097
Fars2	chr13:36117410-36537593	OtA5088-OtA5092	OtA5093-OtA5097
Gtdc1	chr2:44564421-44861622	OtA5088-OtA5092	OtA5093-OtA5097
Isyna1	chr8:70594480-70597290	OtA5088-OtA5092	OtA5093-OtA5097
Mapre2	chr18:23752332-23893861	OtA5088-OtA5092	OtA5093-OtA5097
Msh6	chr17:87975049-88065285	OtA5088-OtA5092	OtA5093-OtA5097
Rab2b	chr14:52261758-52305124	OtA5088-OtA5092	OtA5093-OtA5097
Rpl4	chr9:64173386-64178562	OtA5088-OtA5092	OtA5093-OtA5097
Tsc22d1	chr14:76415820-76507766	OtA5088-OtA5092	OtA5093-OtA5097
Heph	chrX:96455435-96574484	OtA5088-OtA5092	OtA5093-OtA5097
Snn	chr16:11066297-11134532	OtA5088-OtA5092	OtA5093-OtA5097
Trim37	chr11:87127076-87220682	OtA5088-OtA5092	OtA5093-OtA5097
Aarsd1	chr11:101406839-101417433	OtA5088-OtA5092	OtA5093-OtA5097
Crebzf	chr7:90442780-90447719	OtA5088-OtA5092	OtA5093-OtA5097
Ctsh	chr9:90054266-90076095	OtA5088-OtA5092	OtA5093-OtA5097
Cwc15	chr9:14500618-14510620	OtA5088-OtA5092	OtA5093-OtA5097
Fastkd1	chr2:69686823-69712606	OtA5088-OtA5092	OtA5093-OtA5097
Irak3	chr10:120141653-120201537	OtA5088-OtA5092	OtA5093-OtA5097
Nit1	chr1:171329144-171345645	OtA5088-OtA5092	OtA5093-OtA5097
Samd8	chr14:21750530-21798725	OtA5088-OtA5092	OtA5093-OtA5097
Tbc1d8	chr1:39367850-39478747	OtA5088-OtA5092	OtA5093-OtA5097

Thbs3	chr3:89215186-89226837	OtA5088-OtA5092	OtA5093-OtA5097
Lgmn	chr12:102394097-102439697	OtA5088-OtA5092	OtA5093-OtA5097
Lrfn3	chr7:30355513-30362772	OtA5088-OtA5092	OtA5093-OtA5097
Cstf1	chr2:172371002-172381086	OtA5088-OtA5092	OtA5093-OtA5097
Frg1	chr8:41397453-41417118	OtA5088-OtA5092	OtA5093-OtA5097
Gamt	chr10:79697304-80369637	OtA5088-OtA5092	OtA5093-OtA5097
Hecw2	chr1:53806873-54195034	OtA5088-OtA5092	OtA5093-OtA5097
Josd2	chr7:44467979-44471658	OtA5088-OtA5092	OtA5093-OtA5097
Mrps18c	chr5:100798758-100804467	OtA5088-OtA5092	OtA5093-OtA5097
Ranbp1	chr16:18239978-18248694	OtA5088-OtA5092	OtA5093-OtA5097
Gga3	chr11:115584254-115603916	OtA5088-OtA5092	OtA5093-OtA5097
Ap3d1	chr10:80706977-80742211	OtA5088-OtA5092	OtA5093-OtA5097
Arhgef6	chrX:57231484-57338729	OtA5088-OtA5092	OtA5093-OtA5097
BC017612	chr9:15505494-15545259	OtA5088-OtA5092	OtA5093-OtA5097
Bcl10	chr3:145924377-145934283	OtA5088-OtA5092	OtA5093-OtA5097
Bcl2l12	chr7:44986899-44997579	OtA5088-OtA5092	OtA5093-OtA5097
Cct4	chr11:22990592-23003336	OtA5088-OtA5092	OtA5093-OtA5097
Ccz1	chr5:143987908-144014853	OtA5088-OtA5092	OtA5093-OtA5097
Cdc42ep4	chr11:113726849-113751815	OtA5088-OtA5092	OtA5093-OtA5097
Cdhr5	chr7:141269084-141276786	OtA5088-OtA5092	OtA5093-OtA5097
Clpx	chr9:65294294-65330658	OtA5088-OtA5092	OtA5093-OtA5097
D11Wsu47e	chr11:113684411-113694647	OtA5088-OtA5092	OtA5093-OtA5097
Ddx10	chr9:53098453-53248112	OtA5088-OtA5092	OtA5093-OtA5097
Dnajc9	chr14:20384637-20388910	OtA5088-OtA5092	OtA5093-OtA5097
Dusp14	chr11:84048044-84068357	OtA5088-OtA5092	OtA5093-OtA5097
E030011O05Rik	chr9:96685422-96752831	OtA5088-OtA5092	OtA5093-OtA5097
Eif4enif1	chr11:3202352-3479831	OtA5088-OtA5092	OtA5093-OtA5097
Fam100a	chr16:4874778-4879851	OtA5088-OtA5092	OtA5093-OtA5097
Fbxl6	chr15:76535727-76538746	OtA5088-OtA5092	OtA5093-OtA5097
Glis1	chr4:107434718-107635059	OtA5088-OtA5092	OtA5093-OtA5097
Gm6251	chr10:20148919-20281590	OtA5088-OtA5092	OtA5093-OtA5097
Hars2	chr18:36783279-36792560	OtA5088-OtA5092	OtA5093-OtA5097
Hmha1	chr10:79697304-80369637	OtA5088-OtA5092	OtA5093-OtA5097
Igf2as	chr7:142650767-142670356	OtA5088-OtA5092	OtA5093-OtA5097
Immp1l	chr2:105904637-105965558	OtA5088-OtA5092	OtA5093-OtA5097
Ints6	chr14:62663666-62830126	OtA5088-OtA5092	OtA5093-OtA5097
Krt7	chr15:101412402-101427806	OtA5088-OtA5092	OtA5093-OtA5097
Lrrc57	chr2:120604237-120620818	OtA5088-OtA5092	OtA5093-OtA5097
Mad1l1	chr5:140008688-140321552	OtA5088-OtA5092	OtA5093-OtA5097
Mbnl1	chr3:60472829-60629748	OtA5088-OtA5092	OtA5093-OtA5097
Mis12	chr11:71019610-71027134	OtA5088-OtA5092	OtA5093-OtA5097
Mrpl12	chr11:120484668-120488754	OtA5088-OtA5092	OtA5093-OtA5097
Mrpl39	chr16:84717579-84735302	OtA5088-OtA5092	OtA5093-OtA5097
Msra	chr14:64122620-64455903	OtA5088-OtA5092	OtA5093-OtA5097
Npr1	chr3:90450591-90465866	OtA5088-OtA5092	OtA5093-OtA5097
Nsa2	chr13:97067286-97137926	OtA5088-OtA5092	OtA5093-OtA5097
Os9	chr10:127094258-127121160	OtA5088-OtA5092	OtA5093-OtA5097

Pick1	chr15:79229173-79249466	OtA5088-OtA5092	OtA5093-OtA5097
Pigl	chr11:62458459-62513900	OtA5088-OtA5092	OtA5093-OtA5097
Pkmyt1	chr17:23726335-23740330	OtA5088-OtA5092	OtA5093-OtA5097
Plk3	chr4:117126812-117133952	OtA5088-OtA5092	OtA5093-OtA5097
Ppid	chr3:79591388-79603650	OtA5088-OtA5092	OtA5093-OtA5097
Ppp6r2	chr15:89211558-89286261	OtA5088-OtA5092	OtA5093-OtA5097
Rab6a	chr7:100549116-100641268	OtA5088-OtA5092	OtA5093-OtA5097
Ring1	chr17:34020791-34024680	OtA5088-OtA5092	OtA5093-OtA5097
Rpl18a	chr8:70894721-70897443	OtA5088-OtA5092	OtA5093-OtA5097
Smarcd2	chr11:106263170-106272972	OtA5088-OtA5092	OtA5093-OtA5097
Snrpc	chr17:27840086-27851967	OtA5088-OtA5092	OtA5093-OtA5097
Spa17	chr9:37603293-37613720	OtA5088-OtA5092	OtA5093-OtA5097
Spg7	chr8:123065507-123097751	OtA5088-OtA5092	OtA5093-OtA5097
Srbd1	chr17:85984664-86145175	OtA5088-OtA5092	OtA5093-OtA5097
Suds3	chr5:117091679-117115993	OtA5088-OtA5092	OtA5093-OtA5097
Sycn	chr7:28540884-28542210	OtA5088-OtA5092	OtA5093-OtA5097
Tbx3	chr5:119670668-119684601	OtA5088-OtA5092	OtA5093-OtA5097
Tmem188	chr8:88118758-88135197	OtA5088-OtA5092	OtA5093-OtA5097
Tmem19	chr10:115340739-115362262	OtA5088-OtA5092	OtA5093-OtA5097
Tpst1	chr5:130073325-130135732	OtA5088-OtA5092	OtA5093-OtA5097
Trp53inp2	chr2:155381855-155389847	OtA5088-OtA5092	OtA5093-OtA5097
Uap1	chr1:170142002-170174946	OtA5088-OtA5092	OtA5093-OtA5097
Ube2d1	chr10:71254979-71285262	OtA5088-OtA5092	OtA5093-OtA5097
Ulk3	chr9:57589451-57596233	OtA5088-OtA5092	OtA5093-OtA5097
Unc5a	chr13:54949431-55021385	OtA5088-OtA5092	OtA5093-OtA5097
Vps33a	chr5:123528759-123573015	OtA5088-OtA5092	OtA5093-OtA5097
Wdr18	chr10:79697304-80369637	OtA5088-OtA5092	OtA5093-OtA5097
Wdr59	chr8:111448783-111522107	OtA5088-OtA5092	OtA5093-OtA5097
Zfp324	chr7:12965863-12973822	OtA5088-OtA5092	OtA5093-OtA5097
Glo1	chr17:30592861-30612659	OtA5088-OtA5092	OtA5093-OtA5097
Rab11b	chr17:33742483-33760486	OtA5088-OtA5092	OtA5093-OtA5097
Tbp	chr17:15499887-15517427	OtA5088-OtA5092	OtA5093-OtA5097
Tnpo1	chr13:98842080-98926384	OtA5088-OtA5092	OtA5093-OtA5097
Znhit1	chr5:136982200-136996646	OtA5088-OtA5092	OtA5093-OtA5097
Apol9b	chr15:77729071-77736382	OtA5088-OtA5092	OtA5093-OtA5097
Gnl3l	chrX:150983132-151017322	OtA5088-OtA5092	OtA5093-OtA5097
Gpat2	chr2:127425198-127436092	OtA5088-OtA5092	OtA5093-OtA5097
Jmjd7	chr2:120027482-120032604	OtA5088-OtA5092	OtA5093-OtA5097
Lars	chr18:42202348-42262071	OtA5088-OtA5092	OtA5093-OtA5097
Mrps22	chr9:98588729-98601679	OtA5088-OtA5092	OtA5093-OtA5097
Raver1-fdx1l	chr9:21067513-21092008	OtA5088-OtA5092	OtA5093-OtA5097
Tspan33	chr6:29694255-29718558	OtA5088-OtA5092	OtA5093-OtA5097
Ttc23	chr7:67647409-67726576	OtA5088-OtA5092	OtA5093-OtA5097
Csgalnact2	chr6:118107451-118139140	OtA5088-OtA5092	OtA5093-OtA5097
Bace2	chr16:97356727-97439012	OtA5088-OtA5092	OtA5093-OtA5097
Speg	chr1:75375296-75432306	OtA5088-OtA5092	OtA5093-OtA5097
B9d2	chr7:25681157-25686558	OtA5088-OtA5092	OtA5093-OtA5097

BC002230	chr12:100125451-100159653	OtA5088-OtA5092	OtA5093-OtA5097
Cdk9	chr2:32705781-32712784	OtA5088-OtA5092	OtA5093-OtA5097
D930014E17Rik	chr7:109703736-109723771	OtA5088-OtA5092	OtA5093-OtA5097
Ednrb	chr14:103814624-103844173	OtA5088-OtA5092	OtA5093-OtA5097
Fetub	chr16:22918381-22939768	OtA5088-OtA5092	OtA5093-OtA5097
Fto	chr8:91313524-91668433	OtA5088-OtA5092	OtA5093-OtA5097
Hsd17b12	chr2:94032696-94157909	OtA5088-OtA5092	OtA5093-OtA5097
Ldhd	chr8:111626270-111630322	OtA5088-OtA5092	OtA5093-OtA5097
Mmgt1	chrX:56585511-56597919	OtA5088-OtA5092	OtA5093-OtA5097
Mrps34	chr17:24895119-24896273	OtA5088-OtA5092	OtA5093-OtA5097
Rpl21	chr5:146832889-146837032	OtA5088-OtA5092	OtA5093-OtA5097
Ccdc90a	chr13:43538405-43560191	OtA5088-OtA5092	OtA5093-OtA5097
Ngly1	chr14:16238658-16311926	OtA5088-OtA5092	OtA5093-OtA5097
Spcs3	chr8:54520432-54529998	OtA5088-OtA5092	OtA5093-OtA5097
Wdfy1	chr1:79702261-79761769	OtA5088-OtA5092	OtA5093-OtA5097
Fam83b	chr9:76490704-76545804	OtA5088-OtA5092	OtA5093-OtA5097
Ralgds	chr2:28513166-28553082	OtA5088-OtA5092	OtA5093-OtA5097
Stxbp3a	chr3:108793179-108840502	OtA5088-OtA5092	OtA5093-OtA5097
Arhgef1	chr7:24902985-24926591	OtA5088-OtA5092	OtA5093-OtA5097
BC031781	chr1:180851150-180868114	OtA5088-OtA5092	OtA5093-OtA5097
BC088983	chr8:56531521-56550566	OtA5088-OtA5092	OtA5093-OtA5097
Crlf2	chr5:109554708-109558993	OtA5088-OtA5092	OtA5093-OtA5097
Gm9112	chrX:102706887-102707670	OtA5088-OtA5092	OtA5093-OtA5097
Nek8	chr11:78166105-78176666	OtA5088-OtA5092	OtA5093-OtA5097
Prcp	chr7:92875252-92934581	OtA5088-OtA5092	OtA5093-OtA5097
Slc39a9	chr12:80644214-80683342	OtA5088-OtA5092	OtA5093-OtA5097
Tacstd2	chr6:67534058-67535822	OtA5088-OtA5092	OtA5093-OtA5097
Tom1l2	chr11:60226713-60352905	OtA5088-OtA5092	OtA5093-OtA5097
Vangl1	chr3:102156695-102204693	OtA5088-OtA5092	OtA5093-OtA5097
Atp8a2	chr14:59647740-60086834	OtA5088-OtA5092	OtA5093-OtA5097
Dsn1	chr2:156995061-157007075	OtA5088-OtA5092	OtA5093-OtA5097
E130317F20Rik	chr10:79697304-80369637	OtA5088-OtA5092	OtA5093-OtA5097
Faf1	chr4:109676626-109963960	OtA5088-OtA5092	OtA5093-OtA5097
Hsf2	chr10:57486384-57513143	OtA5088-OtA5092	OtA5093-OtA5097
Ipo13	chr4:117894492-117914999	OtA5088-OtA5092	OtA5093-OtA5097
Mtm1	chrX:71210766-71315295	OtA5088-OtA5092	OtA5093-OtA5097
Ptk6	chr2:181195123-181202789	OtA5088-OtA5092	OtA5093-OtA5097
Rnf111	chr9:70425428-70503725	OtA5088-OtA5092	OtA5093-OtA5097
Wdr4	chr17:31494321-31512487	OtA5088-OtA5092	OtA5093-OtA5097
Ccnc	chr4:21727702-21750546	OtA5088-OtA5092	OtA5093-OtA5097
Cybasc3	chr19:10577722-10589830	OtA5088-OtA5092	OtA5093-OtA5097
Mrpl34	chr8:71464925-71465753	OtA5088-OtA5092	OtA5093-OtA5097
Utp14a	chrX:48256933-48285426	OtA5088-OtA5092	OtA5093-OtA5097
Ccdc12	chr9:110656502-110711593	OtA5088-OtA5092	OtA5093-OtA5097
Rrnad1	chr3:87919543-87930195	OtA5088-OtA5092	OtA5093-OtA5097
Ddx41	chr13:55530409-55536658	OtA5088-OtA5092	OtA5093-OtA5097
Incenp	chr19:9872296-9899533	OtA5088-OtA5092	OtA5093-OtA5097

Ccdc28a	chr10:18213684-18234981	OtA5088-OtA5092	OtA5093-OtA5097
Cmtm6	chr9:114731202-114749343	OtA5088-OtA5092	OtA5093-OtA5097
Dhx15	chr5:52150208-52190519	OtA5088-OtA5092	OtA5093-OtA5097
Fam40a	chr3:107612531-107631710	OtA5088-OtA5092	OtA5093-OtA5097
Glod4	chr11:76220394-76243699	OtA5088-OtA5092	OtA5093-OtA5097
Gorasp1	chr9:119894894-119937558	OtA5088-OtA5092	OtA5093-OtA5097
Gtf2h1	chr7:46796093-46823800	OtA5088-OtA5092	OtA5093-OtA5097
Mea1	chr17:46681136-46705002	OtA5088-OtA5092	OtA5093-OtA5097
Mfap4	chr11:61485443-61488704	OtA5088-OtA5092	OtA5093-OtA5097
Mrgprf	chr7:145300908-145309557	OtA5088-OtA5092	OtA5093-OtA5097
Mrpl46	chr7:78775340-78783089	OtA5088-OtA5092	OtA5093-OtA5097
Nol9	chr4:152039326-152061494	OtA5088-OtA5092	OtA5093-OtA5097
Ocrl	chrX:47912455-47965866	OtA5088-OtA5092	OtA5093-OtA5097
Osmr	chr15:6813576-6874313	OtA5088-OtA5092	OtA5093-OtA5097
Pcnx	chr12:81860029-82000924	OtA5088-OtA5092	OtA5093-OtA5097
Pot1a	chr6:25743734-25809226	OtA5088-OtA5092	OtA5093-OtA5097
Ptn	chr6:36715662-36811361	OtA5088-OtA5092	OtA5093-OtA5097
Rbbp4	chr4:129287620-129335370	OtA5088-OtA5092	OtA5093-OtA5097
Repin1	chr6:48593882-48599082	OtA5088-OtA5092	OtA5093-OtA5097
Sft2d3	chr18:31909093-31911903	OtA5088-OtA5092	OtA5093-OtA5097
Sirt7	chr11:120618371-120625002	OtA5088-OtA5092	OtA5093-OtA5097
Slc35a5	chr16:45139572-45158673	OtA5088-OtA5092	OtA5093-OtA5097
Stx18	chr5:38039234-38136818	OtA5088-OtA5092	OtA5093-OtA5097
Tmem126b	chr7:90468828-90475995	OtA5088-OtA5092	OtA5093-OtA5097
Traf2	chr2:25517981-25546894	OtA5088-OtA5092	OtA5093-OtA5097
Trmt2a	chr16:18248882-18289168	OtA5088-OtA5092	OtA5093-OtA5097
Ttf2	chr3:100938859-100969663	OtA5088-OtA5092	OtA5093-OtA5097
Unc45a	chr7:80325291-80340219	OtA5088-OtA5092	OtA5093-OtA5097
Utp23	chr15:51877440-51884622	OtA5088-OtA5092	OtA5093-OtA5097
Atf6	chr1:170704456-170867771	OtA5088-OtA5092	OtA5093-OtA5097
Nfya	chr17:48386884-48409820	OtA5088-OtA5092	OtA5093-OtA5097
Gsto2	chr19:47865544-47886305	OtA5088-OtA5092	OtA5093-OtA5097
Nudt5	chr2:5845033-5868736	OtA5088-OtA5092	OtA5093-OtA5097
Arl8a	chr1:135146833-135156268	OtA5088-OtA5092	OtA5093-OtA5097
Cux1	chr5:136248134-136567431	OtA5088-OtA5092	OtA5093-OtA5097
Anp32e	chr3:95929256-95947387	OtA5088-OtA5092	OtA5093-OtA5097
Arhgap23	chr11:97450159-97502400	OtA5088-OtA5092	OtA5093-OtA5097
Asrgl1	chr19:9111718-9135566	OtA5088-OtA5092	OtA5093-OtA5097
BC030336	chr7:120677619-120734854	OtA5088-OtA5092	OtA5093-OtA5097
Ccng1	chr11:40748551-40755286	OtA5088-OtA5092	OtA5093-OtA5097
Col6a1	chr10:76708791-76726044	OtA5088-OtA5092	OtA5093-OtA5097
Cope	chr8:70302784-70312990	OtA5088-OtA5092	OtA5093-OtA5097
Cpsf2	chr12:101975973-102005993	OtA5088-OtA5092	OtA5093-OtA5097
Csnk1d	chr11:120961740-120991333	OtA5088-OtA5092	OtA5093-OtA5097
Dab2ip	chr2:35549533-35730994	OtA5088-OtA5092	OtA5093-OtA5097
Dmxl2	chr9:54365157-54501626	OtA5088-OtA5092	OtA5093-OtA5097
Eif2b4	chr5:31187557-31193139	OtA5088-OtA5092	OtA5093-OtA5097

Eif4ebp1	chr8:27260326-27275656	OtA5088-OtA5092	OtA5093-OtA5097
Epcam	chr17:87635978-87651127	OtA5088-OtA5092	OtA5093-OtA5097
Fam176a	chr6:82041627-82093099	OtA5088-OtA5092	OtA5093-OtA5097
Fgd4	chr16:16421977-16560196	OtA5088-OtA5092	OtA5093-OtA5097
Gga1	chr15:78877189-78894585	OtA5088-OtA5092	OtA5093-OtA5097
Gnl3	chr14:30999825-31019089	OtA5088-OtA5092	OtA5093-OtA5097
Hdhd2	chr18:76944114-76972171	OtA5088-OtA5092	OtA5093-OtA5097
Hiat1	chr3:116631166-116662677	OtA5088-OtA5092	OtA5093-OtA5097
Hmox2	chr16:4726360-4789935	OtA5088-OtA5092	OtA5093-OtA5097
Ifngr1	chr10:19591957-19610225	OtA5088-OtA5092	OtA5093-OtA5097
Inadl	chr4:98395825-98719603	OtA5088-OtA5092	OtA5093-OtA5097
Itgb3	chr11:104607999-104670471	OtA5088-OtA5092	OtA5093-OtA5097
Itpkb	chr1:180326968-180423659	OtA5088-OtA5092	OtA5093-OtA5097
Lama2	chr10:26981284-27616942	OtA5088-OtA5092	OtA5093-OtA5097
Lmln	chr16:33062520-33125659	OtA5088-OtA5092	OtA5093-OtA5097
Mcts2	chr2:152669460-152708668	OtA5088-OtA5092	OtA5093-OtA5097
Mpnd	chr17:56009200-56036637	OtA5088-OtA5092	OtA5093-OtA5097
Mrpl18	chr17:12911354-12916091	OtA5088-OtA5092	OtA5093-OtA5097
Mrpl49	chr19:6046575-6057751	OtA5088-OtA5092	OtA5093-OtA5097
Nars2	chr7:96951526-97064757	OtA5088-OtA5092	OtA5093-OtA5097
Ndst1	chr18:60685975-60713389	OtA5088-OtA5092	OtA5093-OtA5097
Papss1	chr3:131564767-131643671	OtA5088-OtA5092	OtA5093-OtA5097
Pcdhgc3	chr18:36930284-37841872	OtA5088-OtA5092	OtA5093-OtA5097
Pigv	chr4:133661424-133672647	OtA5088-OtA5092	OtA5093-OtA5097
Plcg1	chr2:160731309-160872990	OtA5088-OtA5092	OtA5093-OtA5097
Poc1a	chr9:106281060-106349891	OtA5088-OtA5092	OtA5093-OtA5097
Polr3b	chr10:84622436-84727178	OtA5088-OtA5092	OtA5093-OtA5097
Ptcd2	chr13:99319648-99344678	OtA5088-OtA5092	OtA5093-OtA5097
Rab2a	chr4:8535643-8607702	OtA5088-OtA5092	OtA5093-OtA5097
Rbm45	chr2:76369983-76383767	OtA5088-OtA5092	OtA5093-OtA5097
Rbm7	chr9:48488696-48495330	OtA5088-OtA5092	OtA5093-OtA5097
Rbp2	chr9:98490536-98509771	OtA5088-OtA5092	OtA5093-OtA5097
Rpp14	chr14:8080312-8091846	OtA5088-OtA5092	OtA5093-OtA5097
Schip1	chr3:67892219-68626482	OtA5088-OtA5092	OtA5093-OtA5097
Sepn1	chr4:134537891-134552166	OtA5088-OtA5092	OtA5093-OtA5097
Slc22a17	chr14:54906726-54913132	OtA5088-OtA5092	OtA5093-OtA5097
Snx2	chr18:53176364-53220860	OtA5088-OtA5092	OtA5093-OtA5097
Srpr	chr9:35211202-35267780	OtA5088-OtA5092	OtA5093-OtA5097
Tbpl1	chr10:22645010-22731447	OtA5088-OtA5092	OtA5093-OtA5097
Tctn3	chr19:40596445-40612215	OtA5088-OtA5092	OtA5093-OtA5097
Th1l	chr2:174415803-174438992	OtA5088-OtA5092	OtA5093-OtA5097
Tmem184b	chr15:79360683-79403303	OtA5088-OtA5092	OtA5093-OtA5097
Tpm2	chr4:43514711-43523388	OtA5088-OtA5092	OtA5093-OtA5097
Ubr7	chr12:102757974-102777701	OtA5088-OtA5092	OtA5093-OtA5097
Prkrir	chr7:98703362-98718061	OtA5088-OtA5092	OtA5093-OtA5097
Itgb6	chr2:60598291-60722603	OtA5088-OtA5092	OtA5093-OtA5097
Rassf6	chr5:90603075-90640487	OtA5088-OtA5092	OtA5093-OtA5097

Acp5	chr9:22055459-22389206	OtA5088-OtA5092	OtA5093-OtA5097
Arfgef2	chr2:166805580-166898051	OtA5088-OtA5092	OtA5093-OtA5097
Atp6v1d	chr12:78842981-78861638	OtA5088-OtA5092	OtA5093-OtA5097
BC003965	chr17:25184560-25187662	OtA5088-OtA5092	OtA5093-OtA5097
Blcap	chr2:157556361-157566361	OtA5088-OtA5092	OtA5093-OtA5097
Cdc42se2	chr11:54717414-54787703	OtA5088-OtA5092	OtA5093-OtA5097
Depdc5	chr5:32863720-32994233	OtA5088-OtA5092	OtA5093-OtA5097
Evc2	chr5:37338477-37425054	OtA5088-OtA5092	OtA5093-OtA5097
Fam172a	chr13:77708689-78166240	OtA5088-OtA5092	OtA5093-OtA5097
Gramd1c	chr16:43980349-44027945	OtA5088-OtA5092	OtA5093-OtA5097
H3f3a	chr1:180802567-180813603	OtA5088-OtA5092	OtA5093-OtA5097
Hsp90b1	chr10:86690840-86705444	OtA5088-OtA5092	OtA5093-OtA5097
Mpi	chr9:57544267-57552752	OtA5088-OtA5092	OtA5093-OtA5097
Mrpl23	chr7:142533116-142540742	OtA5088-OtA5092	OtA5093-OtA5097
Nespas	chr2:174281236-174346742	OtA5088-OtA5092	OtA5093-OtA5097
Rhov	chr2:119269200-119271226	OtA5088-OtA5092	OtA5093-OtA5097
Rps15	chr10:79697304-80369637	OtA5088-OtA5092	OtA5093-OtA5097
Snhg8	chr3:123507551-123508336	OtA5088-OtA5092	OtA5093-OtA5097
Glud1	chr14:34310726-34345033	OtA5088-OtA5092	OtA5093-OtA5097
Gtf2e2	chr8:33731913-33777173	OtA5088-OtA5092	OtA5093-OtA5097
Mkrn2	chr6:115601937-115676635	OtA5088-OtA5092	OtA5093-OtA5097
Pdcl	chr2:37350073-37359332	OtA5088-OtA5092	OtA5093-OtA5097
Trhr2	chr8:122356966-122360746	OtA5088-OtA5092	OtA5093-OtA5097
Aacs	chr5:125475872-125517403	OtA5088-OtA5092	OtA5093-OtA5097
Abcd3	chr3:121758909-121815215	OtA5088-OtA5092	OtA5093-OtA5097
Acsl3	chr1:78657824-78707743	OtA5088-OtA5092	OtA5093-OtA5097
Ak2	chr4:128993264-129011529	OtA5088-OtA5092	OtA5093-OtA5097
Ak4	chr4:101419288-101467771	OtA5088-OtA5092	OtA5093-OtA5097
Amz2	chr11:109425945-109438148	OtA5088-OtA5092	OtA5093-OtA5097
Ankrd9	chr12:110975352-110979021	OtA5088-OtA5092	OtA5093-OtA5097
Ap2b1	chr11:83302696-83405033	OtA5088-OtA5092	OtA5093-OtA5097
Ap3s2	chr7:79875324-79920640	OtA5088-OtA5092	OtA5093-OtA5097
Apba3	chr10:81268171-81291267	OtA5088-OtA5092	OtA5093-OtA5097
Apc2	chr10:79697304-80369637	OtA5088-OtA5092	OtA5093-OtA5097
Aqp11	chr7:97726378-97738247	OtA5088-OtA5092	OtA5093-OtA5097
Arhgap44	chr11:64979034-65162961	OtA5088-OtA5092	OtA5093-OtA5097
Arhgef2	chr3:88616206-88648052	OtA5088-OtA5092	OtA5093-OtA5097
BC003266	chr4:127243783-127247809	OtA5088-OtA5092	OtA5093-OtA5097
Bbc3	chr7:16309582-16318334	OtA5088-OtA5092	OtA5093-OtA5097
Bgn	chrX:73483634-73495936	OtA5088-OtA5092	OtA5093-OtA5097
Bud31	chr5:145140396-145167104	OtA5088-OtA5092	OtA5093-OtA5097
C030048B08Rik	chr5:3571715-3637101	OtA5088-OtA5092	OtA5093-OtA5097
Cadm1	chr9:47530351-47853385	OtA5088-OtA5092	OtA5093-OtA5097
Capn7	chr14:31336723-31371983	OtA5088-OtA5092	OtA5093-OtA5097
Ccdc101	chr7:126649308-126672779	OtA5088-OtA5092	OtA5093-OtA5097
Ccdc43	chr11:102684687-102697725	OtA5088-OtA5092	OtA5093-OtA5097
Cebpz	chr17:78916498-78937070	OtA5088-OtA5092	OtA5093-OtA5097

Clcn3	chr8:60910388-60983311	OtA5088-OtA5092	OtA5093-OtA5097
Clstn1	chr4:149586637-149647899	OtA5088-OtA5092	OtA5093-OtA5097
Cox11	chr11:90638183-90687601	OtA5088-OtA5092	OtA5093-OtA5097
Cxcl16	chr11:70454233-70466199	OtA5088-OtA5092	OtA5093-OtA5097
D030028A08Rik	chr11:96944145-96977688	OtA5088-OtA5092	OtA5093-OtA5097
Dcps	chr9:35046578-35175987	OtA5088-OtA5092	OtA5093-OtA5097
Dedd	chr1:171329144-171345645	OtA5088-OtA5092	OtA5093-OtA5097
Dennd4b	chr3:90266533-90280665	OtA5088-OtA5092	OtA5093-OtA5097
Dmpk	chr7:19083848-19093820	OtA5088-OtA5092	OtA5093-OtA5097
Dnajb1	chr8:83608174-83611903	OtA5088-OtA5092	OtA5093-OtA5097
Dpf2	chr19:5897134-5912866	OtA5088-OtA5092	OtA5093-OtA5097
Dph5	chr3:115888182-115929323	OtA5088-OtA5092	OtA5093-OtA5097
Ebpl	chr14:61339762-61360445	OtA5088-OtA5092	OtA5093-OtA5097
Egln1	chr8:124908586-124949254	OtA5088-OtA5092	OtA5093-OtA5097
Eif1b	chr9:120492605-120495327	OtA5088-OtA5092	OtA5093-OtA5097
Enc1	chr13:97241104-97253040	OtA5088-OtA5092	OtA5093-OtA5097
Fam101b	chr11:76019194-76027782	OtA5088-OtA5092	OtA5093-OtA5097
Fam115a	chr6:42672546-42693059	OtA5088-OtA5092	OtA5093-OtA5097
Fam82b	chr4:19575065-19606932	OtA5088-OtA5092	OtA5093-OtA5097
Fancf	chr7:51860576-51862267	OtA5088-OtA5092	OtA5093-OtA5097
Fastkd2	chr1:63730624-63753385	OtA5088-OtA5092	OtA5093-OtA5097
Fbxl14	chr6:119432530-119544347	OtA5088-OtA5092	OtA5093-OtA5097
Foxa3	chr7:19013282-19023539	OtA5088-OtA5092	OtA5093-OtA5097
Frat1	chr19:41829969-41832583	OtA5088-OtA5092	OtA5093-OtA5097
Frk	chr10:34483399-34611230	OtA5088-OtA5092	OtA5093-OtA5097
Fth1	chr19:9982702-9985092	OtA5088-OtA5092	OtA5093-OtA5097
Ftsj2	chr5:140327673-140331898	OtA5088-OtA5092	OtA5093-OtA5097
Gadd45a	chr6:67035095-67080652	OtA5088-OtA5092	OtA5093-OtA5097
Gls	chr1:52163449-52233232	OtA5088-OtA5092	OtA5093-OtA5097
Gm16845	chr9:22055459-22389206	OtA5088-OtA5092	OtA5093-OtA5097
Gpatch1	chr7:35276543-35318440	OtA5088-OtA5092	OtA5093-OtA5097
Grb7	chr11:98446833-98455373	OtA5088-OtA5092	OtA5093-OtA5097
Gtf3c3	chr1:54397580-54438971	OtA5088-OtA5092	OtA5093-OtA5097
Gtpbp2	chr17:46161031-46169370	OtA5088-OtA5092	OtA5093-OtA5097
Hip1r	chr5:123973627-124003215	OtA5088-OtA5092	OtA5093-OtA5097
Hnrnpa0	chr13:58125878-58128556	OtA5088-OtA5092	OtA5093-OtA5097
Ildr1	chr16:36694037-36726741	OtA5088-OtA5092	OtA5093-OtA5097
Insc	chr7:114745765-114850380	OtA5088-OtA5092	OtA5093-OtA5097
Itih3	chr14:30908573-30923587	OtA5088-OtA5092	OtA5093-OtA5097
Jkamp	chr12:72085838-72185029	OtA5088-OtA5092	OtA5093-OtA5097
Kctd15	chr7:34639015-34652841	OtA5088-OtA5092	OtA5093-OtA5097
Kctd2	chr11:115420125-115431274	OtA5088-OtA5092	OtA5093-OtA5097
Khdrbs3	chr15:68928419-69093518	OtA5088-OtA5092	OtA5093-OtA5097
Khnyn	chr14:55884968-55896781	OtA5088-OtA5092	OtA5093-OtA5097
Krit1	chr5:3803164-3844515	OtA5088-OtA5092	OtA5093-OtA5097
Lysmd2	chr9:75625731-75637773	OtA5088-OtA5092	OtA5093-OtA5097
Maged1	chrX:94535473-94542074	OtA5088-OtA5092	OtA5093-OtA5097

Map3k9	chr12:81714949-81781170	OtA5088-OtA5092	OtA5093-OtA5097
Mcm9	chr10:53537323-53630439	OtA5088-OtA5092	OtA5093-OtA5097
Med8	chr4:118409336-118415782	OtA5088-OtA5092	OtA5093-OtA5097
Mettl3	chr14:52261758-52305124	OtA5088-OtA5092	OtA5093-OtA5097
Mgea5	chr19:45750258-45783291	OtA5088-OtA5092	OtA5093-OtA5097
Mib1	chr18:10725624-10812217	OtA5088-OtA5092	OtA5093-OtA5097
Mre11a	chr9:14784706-14834417	OtA5088-OtA5092	OtA5093-OtA5097
Mtap1s	chr8:70905973-70917529	OtA5088-OtA5092	OtA5093-OtA5097
Mtmr3	chr11:4480867-4594815	OtA5088-OtA5092	OtA5093-OtA5097
Nadk	chr4:155562391-155591001	OtA5088-OtA5092	OtA5093-OtA5097
Naf1	chr8:66860216-66890564	OtA5088-OtA5092	OtA5093-OtA5097
Nagk	chr6:83795056-83802556	OtA5088-OtA5092	OtA5093-OtA5097
Ndfip2	chr14:105258672-105309298	OtA5088-OtA5092	OtA5093-OtA5097
Ndufs3	chr2:90894635-90904721	OtA5088-OtA5092	OtA5093-OtA5097
Nfrkb	chr9:31386191-31421334	OtA5088-OtA5092	OtA5093-OtA5097
Nhlrc3	chr3:53451995-53463258	OtA5088-OtA5092	OtA5093-OtA5097
Nynrin	chr14:55854114-55874736	OtA5088-OtA5092	OtA5093-OtA5097
Pank2	chr2:131127411-131604824	OtA5088-OtA5092	OtA5093-OtA5097
Parl	chr16:20279820-20302362	OtA5088-OtA5092	OtA5093-OtA5097
Parp2	chr14:50807945-50821300	OtA5088-OtA5092	OtA5093-OtA5097
Pcdhga12	chr18:36930284-37841872	OtA5088-OtA5092	OtA5093-OtA5097
Pdf	chr8:107046289-107048614	OtA5088-OtA5092	OtA5093-OtA5097
Phax	chr18:56525735-56587712	OtA5088-OtA5092	OtA5093-OtA5097
Phrf1	chr7:141228787-141262751	OtA5088-OtA5092	OtA5093-OtA5097
Ppp1r10	chr17:35916910-35932283	OtA5088-OtA5092	OtA5093-OtA5097
Ppp2r2d	chr7:138846385-138883056	OtA5088-OtA5092	OtA5093-OtA5097
Pppde1	chr1:178187416-178252597	OtA5088-OtA5092	OtA5093-OtA5097
Prpf3	chr3:95830621-95855753	OtA5088-OtA5092	OtA5093-OtA5097
Prpf38b	chr3:108902806-108911704	OtA5088-OtA5092	OtA5093-OtA5097
Psmg1	chr16:95979934-95990903	OtA5088-OtA5092	OtA5093-OtA5097
Rabggtb	chr3:153907288-153912966	OtA5088-OtA5092	OtA5093-OtA5097
Rap2c	chrX:51003913-51018018	OtA5088-OtA5092	OtA5093-OtA5097
Rasal1	chr5:120648821-120679593	OtA5088-OtA5092	OtA5093-OtA5097
Rbm10	chrX:20615325-20650905	OtA5088-OtA5092	OtA5093-OtA5097
Rbm15b	chr9:106883984-106887000	OtA5088-OtA5092	OtA5093-OtA5097
Rer1	chr4:155074111-155086297	OtA5088-OtA5092	OtA5093-OtA5097
Rfc4	chr16:23107478-23127730	OtA5088-OtA5092	OtA5093-OtA5097
Ror1	chr4:100095790-100442545	OtA5088-OtA5092	OtA5093-OtA5097
Rpl36	chr17:56613394-56614246	OtA5088-OtA5092	OtA5093-OtA5097
Rpusd4	chr9:35267880-35275957	OtA5088-OtA5092	OtA5093-OtA5097
Rtn4ip1	chr10:43901806-43947862	OtA5088-OtA5092	OtA5093-OtA5097
Sbno2	chr10:79697304-80369637	OtA5088-OtA5092	OtA5093-OtA5097
Scyl3	chr1:163930063-163994781	OtA5088-OtA5092	OtA5093-OtA5097
Sdr39u1	chr14:55897284-55900232	OtA5088-OtA5092	OtA5093-OtA5097
Sec14l1	chr11:117115171-117159268	OtA5088-OtA5092	OtA5093-OtA5097
Sec16a	chr2:26409430-26445216	OtA5088-OtA5092	OtA5093-OtA5097
Setd7	chr3:51515317-51567116	OtA5088-OtA5092	OtA5093-OtA5097

Sgk1	chr10:21882183-21999902	OtA5088-OtA5092	OtA5093-OtA5097
Slc25a16	chr10:62920632-62946494	OtA5088-OtA5092	OtA5093-OtA5097
Snora7a	chr6:115774556-115808743	OtA5088-OtA5092	OtA5093-OtA5097
Sox18	chr2:181669836-181671640	OtA5088-OtA5092	OtA5093-OtA5097
Spata13	chr14:60634728-60764556	OtA5088-OtA5092	OtA5093-OtA5097
Sqle	chr15:59315091-59331193	OtA5088-OtA5092	OtA5093-OtA5097
Tatdn3	chr1:191045829-191062932	OtA5088-OtA5092	OtA5093-OtA5097
Tbcc	chr17:46890620-46892463	OtA5088-OtA5092	OtA5093-OtA5097
Tff3	chr17:31125305-31129611	OtA5088-OtA5092	OtA5093-OtA5097
Tm2d1	chr4:98355369-98383265	OtA5088-OtA5092	OtA5093-OtA5097
Tmem204	chr17:25016085-25099497	OtA5088-OtA5092	OtA5093-OtA5097
Twistnb	chr12:33429623-33439380	OtA5088-OtA5092	OtA5093-OtA5097
Txnl4b	chr8:109565985-109574051	OtA5088-OtA5092	OtA5093-OtA5097
Ube3c	chr5:29569241-29676077	OtA5088-OtA5092	OtA5093-OtA5097
Ubr1	chr2:120850655-120970715	OtA5088-OtA5092	OtA5093-OtA5097
Ufsp1	chr5:137294668-137295664	OtA5088-OtA5092	OtA5093-OtA5097
Ugp2	chr11:21321140-21370879	OtA5088-OtA5092	OtA5093-OtA5097
Usf2	chr7:30945247-30956803	OtA5088-OtA5092	OtA5093-OtA5097
Vps13c	chr9:67840395-67995634	OtA5088-OtA5092	OtA5093-OtA5097
Vps28	chr15:76622087-76626027	OtA5088-OtA5092	OtA5093-OtA5097
Vps39	chr2:120316460-120353133	OtA5088-OtA5092	OtA5093-OtA5097
Wnt4	chr4:137277634-137299501	OtA5088-OtA5092	OtA5093-OtA5097
Xpo6	chr7:126101718-126200408	OtA5088-OtA5092	OtA5093-OtA5097
Xpo7	chr14:70654245-70766628	OtA5088-OtA5092	OtA5093-OtA5097
Zfp426	chr9:20468548-20492746	OtA5088-OtA5092	OtA5093-OtA5097
Zfp568	chr7:29983954-30028282	OtA5088-OtA5092	OtA5093-OtA5097
Zfp961	chr8:71951065-71970333	OtA5088-OtA5092	OtA5093-OtA5097
Abl1	chr2:31688555-31804227	OtA5088-OtA5092	OtA5093-OtA5097
Adssl1	chr12:112620046-112641355	OtA5088-OtA5092	OtA5093-OtA5097
Amn1	chr6:149157576-149188712	OtA5088-OtA5092	OtA5093-OtA5097
Atp6v1c1	chr15:38661903-38692444	OtA5088-OtA5092	OtA5093-OtA5097
Bcas2	chr3:103171710-103179154	OtA5088-OtA5092	OtA5093-OtA5097
Ccdc84	chr9:44407713-44418007	OtA5088-OtA5092	OtA5093-OtA5097
Cdc42ep3	chr17:79334024-79355091	OtA5088-OtA5092	OtA5093-OtA5097
Cdh13	chr8:118283754-119323448	OtA5088-OtA5092	OtA5093-OtA5097
Chrnb1	chr11:69784035-69795937	OtA5088-OtA5092	OtA5093-OtA5097
D030016E14Rik	chr8:47490127-47533470	OtA5088-OtA5092	OtA5093-OtA5097
Ddx17	chr15:79516407-79546741	OtA5088-OtA5092	OtA5093-OtA5097
Dpysl3	chr18:43324226-43393331	OtA5088-OtA5092	OtA5093-OtA5097
Eif2a	chr3:58521970-58557501	OtA5088-OtA5092	OtA5093-OtA5097
Fam115c	chr6:42623042-42645041	OtA5088-OtA5092	OtA5093-OtA5097
Fbxo34	chr14:47472560-47531962	OtA5088-OtA5092	OtA5093-OtA5097
Gatad1	chr5:3639960-3647936	OtA5088-OtA5092	OtA5093-OtA5097
Gm221	chr10:4509871-4561111	OtA5088-OtA5092	OtA5093-OtA5097
Ifi27l2a	chr12:103442166-103443680	OtA5088-OtA5092	OtA5093-OtA5097
Kazn	chr4:142102389-142239401	OtA5088-OtA5092	OtA5093-OtA5097
Lig3	chr11:82781134-82871210	OtA5088-OtA5092	OtA5093-OtA5097

Lrrc59	chr11:94629823-94653754	OtA5088-OtA5092	OtA5093-OtA5097
Mad2l2	chr4:148140489-148145696	OtA5088-OtA5092	OtA5093-OtA5097
Mzt1	chr14:99034543-99046136	OtA5088-OtA5092	OtA5093-OtA5097
Nbas	chr12:13269126-13583811	OtA5088-OtA5092	OtA5093-OtA5097
Nme7	chr1:164307674-164458355	OtA5088-OtA5092	OtA5093-OtA5097
Nutf2	chr8:105860633-105880401	OtA5088-OtA5092	OtA5093-OtA5097
Polr2h	chr16:20717825-20722265	OtA5088-OtA5092	OtA5093-OtA5097
Prdm4	chr10:85891967-85916945	OtA5088-OtA5092	OtA5093-OtA5097
Prrc2a	chr17:35149085-35164877	OtA5088-OtA5092	OtA5093-OtA5097
Rnf44	chr13:54679398-54693960	OtA5088-OtA5092	OtA5093-OtA5097
Smek1	chr12:101039408-101088927	OtA5088-OtA5092	OtA5093-OtA5097
Srsf7	chr17:80200079-80207305	OtA5088-OtA5092	OtA5093-OtA5097
Stau2	chr1:16228809-16519302	OtA5088-OtA5092	OtA5093-OtA5097
Supt7l	chr5:31514568-31526762	OtA5088-OtA5092	OtA5093-OtA5097
Yars2	chr16:16302964-16309640	OtA5088-OtA5092	OtA5093-OtA5097
Yipf5	chr18:40204864-40219399	OtA5088-OtA5092	OtA5093-OtA5097
Zfp810	chr9:22055459-22389206	OtA5088-OtA5092	OtA5093-OtA5097
Cct8	chr16:87454984-87495869	OtA5088-OtA5092	OtA5093-OtA5097
Evl	chr12:108410654-108702306	OtA5088-OtA5092	OtA5093-OtA5097
Hint3	chr10:30608206-30618366	OtA5088-OtA5092	OtA5093-OtA5097
Nup50	chr15:84923427-84942963	OtA5088-OtA5092	OtA5093-OtA5097
Pttg1ip	chr10:77581766-77598732	OtA5088-OtA5092	OtA5093-OtA5097
Rab3gap2	chr1:185204167-185329401	OtA5088-OtA5092	OtA5093-OtA5097
Rab43	chr6:87788852-87811779	OtA5088-OtA5092	OtA5093-OtA5097
Xbp1	chr11:5520966-5525872	OtA5088-OtA5092	OtA5093-OtA5097
Zfp687	chr3:94974730-95015238	OtA5088-OtA5092	OtA5093-OtA5097
Abca3	chr17:24352045-24414513	OtA5088-OtA5092	OtA5093-OtA5097
Calml4	chr9:62838786-62875917	OtA5088-OtA5092	OtA5093-OtA5097
Cdan1	chr2:120716153-120731517	OtA5088-OtA5092	OtA5093-OtA5097
Elavl1	chr8:4284781-4325100	OtA5088-OtA5092	OtA5093-OtA5097
Plekha8	chr6:54595110-54645822	OtA5088-OtA5092	OtA5093-OtA5097
Rhobtb2	chr14:69784989-69805545	OtA5088-OtA5092	OtA5093-OtA5097
Zfp846	chr9:20581327-20595193	OtA5088-OtA5092	OtA5093-OtA5097
Fbxo8	chr8:56551133-56593939	OtA5088-OtA5092	OtA5093-OtA5097
Ccdc107	chr4:43493364-43495921	OtA5088-OtA5092	OtA5093-OtA5097
Rhod	chr19:4425456-4477143	OtA5088-OtA5092	OtA5093-OtA5097
Surf2	chr2:26913377-26933511	OtA5088-OtA5092	OtA5093-OtA5097
Hmg20b	chr10:81346045-81350457	OtA5088-OtA5092	OtA5093-OtA5097
Lrsam1	chr2:32876133-32961255	OtA5088-OtA5092	OtA5093-OtA5097
Nipal1	chr5:72647795-72671078	OtA5088-OtA5092	OtA5093-OtA5097
Sec23ip	chr7:128744869-128784835	OtA5088-OtA5092	OtA5093-OtA5097
Usp48	chr4:137594188-137658537	OtA5088-OtA5092	OtA5093-OtA5097
C1qtnf6	chr15:78523345-78529651	OtA5088-OtA5092	OtA5093-OtA5097
D4Wsu53e	chr4:134923624-134927370	OtA5088-OtA5092	OtA5093-OtA5097
Prune2	chr19:16956117-17223932	OtA5088-OtA5092	OtA5093-OtA5097
Rapgef3	chr15:97744769-97767666	OtA5088-OtA5092	OtA5093-OtA5097
Slc18a1	chr8:69037707-69113717	OtA5088-OtA5092	OtA5093-OtA5097

Zdhhc7	chr8:120081094-120101472	OtA5088-OtA5092	OtA5093-OtA5097
Zfp697	chr3:98382480-98431949	OtA5088-OtA5092	OtA5093-OtA5097
Ly96	chr1:16688455-16709605	OtA5088-OtA5092	OtA5093-OtA5097
Zbtb48	chr4:152019775-152027671	OtA5088-OtA5092	OtA5093-OtA5097
Amacr	chr15:10981755-10996624	OtA5088-OtA5092	OtA5093-OtA5097
Hba-a1,Hba-a2	chr11:32296488-32297310	OtA5088-OtA5092	OtA5093-OtA5097
Jam2	chr16:84774122-84826375	OtA5088-OtA5092	OtA5093-OtA5097
Neo1	chr9:58874679-59036441	OtA5088-OtA5092	OtA5093-OtA5097
Il6ra	chr3:89869323-89913162	OtA5088-OtA5092	OtA5093-OtA5097
Iqgap1	chr7:80711582-80803331	OtA5088-OtA5092	OtA5093-OtA5097
Morc4	chrX:139821634-139871654	OtA5088-OtA5092	OtA5093-OtA5097
Sccpdh	chr1:179668230-179687184	OtA5088-OtA5092	OtA5093-OtA5097
Surf6	chr2:26890771-26902813	OtA5088-OtA5092	OtA5093-OtA5097
Vtcn1	chr3:100825458-100896922	OtA5088-OtA5092	OtA5093-OtA5097
A2ld1	chr14:122890859-122913165	OtA5088-OtA5092	OtA5093-OtA5097
Cdc45	chr16:18780446-18811972	OtA5088-OtA5092	OtA5093-OtA5097
Fzd4	chr7:89404365-89410110	OtA5088-OtA5092	OtA5093-OtA5097
Mvk	chr5:114444305-114460590	OtA5088-OtA5092	OtA5093-OtA5097
Sh3bgrl	chrX:109095406-109162467	OtA5088-OtA5092	OtA5093-OtA5097
Thsd4	chr9:59966930-60511035	OtA5088-OtA5092	OtA5093-OtA5097
Stambp	chr6:83543205-83572504	OtA5088-OtA5092	OtA5093-OtA5097
Abtb1	chr6:88835913-88841935	OtA5088-OtA5092	OtA5093-OtA5097
Atp6v1g1	chr4:63544764-63550701	OtA5088-OtA5092	OtA5093-OtA5097
Exosc5	chr7:25659152-25668032	OtA5088-OtA5092	OtA5093-OtA5097
Foxo4	chrX:101254527-101260873	OtA5088-OtA5092	OtA5093-OtA5097
Ogfod2	chr5:124112337-124115476	OtA5088-OtA5092	OtA5093-OtA5097
Scgb1a1	chr19:9083641-9087956	OtA5088-OtA5092	OtA5093-OtA5097
Surf1	chr2:26913377-26933511	OtA5088-OtA5092	OtA5093-OtA5097
Ugcg	chr4:59189549-59222833	OtA5088-OtA5092	OtA5093-OtA5097
Use1	chr8:71366847-71369732	OtA5088-OtA5092	OtA5093-OtA5097
D10Wsu102e	chr10:83360220-83368835	OtA5088-OtA5092	OtA5093-OtA5097
Morf4l2	chrX:136732947-136803361	OtA5088-OtA5092	OtA5093-OtA5097
Polr3h	chr15:81872462-81926213	OtA5088-OtA5092	OtA5093-OtA5097
Thap6	chr5:91948903-91972137	OtA5088-OtA5092	OtA5093-OtA5097
Cbwd1	chr19:24919915-24961616	OtA5088-OtA5092	OtA5093-OtA5097
D330023K18Rik	chr2:31151048-31152291	OtA5088-OtA5092	OtA5093-OtA5097
Dock6	chr9:21800179-21852635	OtA5088-OtA5092	OtA5093-OtA5097
Exoc8	chr8:124890298-124897705	OtA5088-OtA5092	OtA5093-OtA5097
Fam160b1	chr19:57361008-57389594	OtA5088-OtA5092	OtA5093-OtA5097
Fam187b	chr7:30973803-30989725	OtA5088-OtA5092	OtA5093-OtA5097
Fam43a	chr16:30312423-31275697	OtA5088-OtA5092	OtA5093-OtA5097
Fam55c	chr16:55839952-55895285	OtA5088-OtA5092	OtA5093-OtA5097
Gm11127	chr17:36042960-36058645	OtA5088-OtA5092	OtA5093-OtA5097
Gna11	chr10:81528731-81545046	OtA5088-OtA5092	OtA5093-OtA5097
Isoc2a	chr7:4877052-4895716	OtA5088-OtA5092	OtA5093-OtA5097
Kctd17	chr15:78428627-78439303	OtA5088-OtA5092	OtA5093-OtA5097
Lrp11	chr10:7589799-7625477	OtA5088-OtA5092	OtA5093-OtA5097

Mafb	chr2:160363676-160367065	OtA5088-OtA5092	OtA5093-OtA5097
Mettl17	chr14:51884841-51891868	OtA5088-OtA5092	OtA5093-OtA5097
Mudeng	chr14:49012143-49087723	OtA5088-OtA5092	OtA5093-OtA5097
Nfu1	chr6:87009835-87028461	OtA5088-OtA5092	OtA5093-OtA5097
Ormdl3	chr11:98581293-98587245	OtA5088-OtA5092	OtA5093-OtA5097
Rcbtb1	chr14:59201227-59237265	OtA5088-OtA5092	OtA5093-OtA5097
Rps21	chr2:180257378-180258444	OtA5088-OtA5092	OtA5093-OtA5097
Sip1	chr12:59013392-59027988	OtA5088-OtA5092	OtA5093-OtA5097
Slmo2	chr2:174465090-174472941	OtA5088-OtA5092	OtA5093-OtA5097
Smap2	chr4:120968316-121017247	OtA5088-OtA5092	OtA5093-OtA5097
Tmem18	chr12:30584442-30591219	OtA5088-OtA5092	OtA5093-OtA5097
Trappc9	chr15:72589619-73061204	OtA5088-OtA5092	OtA5093-OtA5097
Trf	chr9:103208875-103230286	OtA5088-OtA5092	OtA5093-OtA5097
Tsnax	chr8:125012996-125034192	OtA5088-OtA5092	OtA5093-OtA5097
Zfp839	chr12:110850278-110869998	OtA5088-OtA5092	OtA5093-OtA5097
Aatf	chr11:84422855-84513501	OtA5088-OtA5092	OtA5093-OtA5097
Acsl5	chr19:55253368-55296628	OtA5088-OtA5092	OtA5093-OtA5097
Adal	chr2:121142140-121156678	OtA5088-OtA5092	OtA5093-OtA5097
B3gnt8	chr7:25627623-25629490	OtA5088-OtA5092	OtA5093-OtA5097
Bcar3	chr3:122419779-122530182	OtA5088-OtA5092	OtA5093-OtA5097
Bcat2	chr7:45567794-45589710	OtA5088-OtA5092	OtA5093-OtA5097
Blmh	chr11:76945655-76987389	OtA5088-OtA5092	OtA5093-OtA5097
C1galt1c1	chrX:38630782-38635143	OtA5088-OtA5092	OtA5093-OtA5097
Camk2g	chr14:20734874-20794088	OtA5088-OtA5092	OtA5093-OtA5097
Ccdc109a	chr10:59446983-59616692	OtA5088-OtA5092	OtA5093-OtA5097
Cdh24	chr14:54631991-54641364	OtA5088-OtA5092	OtA5093-OtA5097
D1Ertd622e	chr1:97643901-97662018	OtA5088-OtA5092	OtA5093-OtA5097
D930048N14Rik	chr11:51643088-51657681	OtA5088-OtA5092	OtA5093-OtA5097
Dennd4a	chr9:64811010-64919667	OtA5088-OtA5092	OtA5093-OtA5097
E130102H24Rik	chr4:101346523-101399185	OtA5088-OtA5092	OtA5093-OtA5097
E2f8	chr7:48866428-48881041	OtA5088-OtA5092	OtA5093-OtA5097
Ensa	chr3:95624979-95632117	OtA5088-OtA5092	OtA5093-OtA5097
Entpd5	chr12:84373856-84409029	OtA5088-OtA5092	OtA5093-OtA5097
Gemin5	chr11:58120000-58168539	OtA5088-OtA5092	OtA5093-OtA5097
Gins2	chr8:120488865-120589075	OtA5088-OtA5092	OtA5093-OtA5097
Gpr19	chr6:134869092-134897925	OtA5088-OtA5092	OtA5093-OtA5097
Klhl26	chr8:70450227-70476943	OtA5088-OtA5092	OtA5093-OtA5097
Ldoc1	chrX:61709615-61710950	OtA5088-OtA5092	OtA5093-OtA5097
Man2a1	chr17:64601648-64755110	OtA5088-OtA5092	OtA5093-OtA5097
Mki67ip	chr1:118321842-118333831	OtA5088-OtA5092	OtA5093-OtA5097
Myc	chr15:61985340-61990361	OtA5088-OtA5092	OtA5093-OtA5097
Ndufb11	chrX:20615325-20650905	OtA5088-OtA5092	OtA5093-OtA5097
Nudt1	chr5:140331921-140338135	OtA5088-OtA5092	OtA5093-OtA5097
Osbpl8	chr10:111164801-111297247	OtA5088-OtA5092	OtA5093-OtA5097
Ovca2	chr11:75175942-75190483	OtA5088-OtA5092	OtA5093-OtA5097
Pdia3	chr2:121413901-121438686	OtA5088-OtA5092	OtA5093-OtA5097
Pdpr	chr8:111094744-111145498	OtA5088-OtA5092	OtA5093-OtA5097

Pnpt1	chr11:29130750-29161828	OtA5088-OtA5092	OtA5093-OtA5097
Ppp1r3e	chr14:54875596-54877538	OtA5088-OtA5092	OtA5093-OtA5097
Rasgrp1	chr2:117279992-117342877	OtA5088-OtA5092	OtA5093-OtA5097
Rfng	chr11:120780744-120784204	OtA5088-OtA5092	OtA5093-OtA5097
Rnasel	chr1:153749425-153764221	OtA5088-OtA5092	OtA5093-OtA5097
Rnf185	chr11:3202352-3479831	OtA5088-OtA5092	OtA5093-OtA5097
Rnf220	chr4:117271463-117496915	OtA5088-OtA5092	OtA5093-OtA5097
Scp2	chr4:108043829-108118547	OtA5088-OtA5092	OtA5093-OtA5097
Sf3b4	chr3:96172549-96177564	OtA5088-OtA5092	OtA5093-OtA5097
Sgsm1	chr5:113243219-113310786	OtA5088-OtA5092	OtA5093-OtA5097
Slc30a7	chr3:115938972-116007406	OtA5088-OtA5092	OtA5093-OtA5097
Smcr8	chr11:60777524-60788287	OtA5088-OtA5092	OtA5093-OtA5097
Snx1	chr9:66088126-66124886	OtA5088-OtA5092	OtA5093-OtA5097
Spin2	chrX:153832292-153834240	OtA5088-OtA5092	OtA5093-OtA5097
Stam	chr2:14074111-14148330	OtA5088-OtA5092	OtA5093-OtA5097
Stx12	chr4:132854063-132884458	OtA5088-OtA5092	OtA5093-OtA5097
Sult1a1	chr7:126672869-126676357	OtA5088-OtA5092	OtA5093-OtA5097
Tcf3	chr10:80409164-80433653	OtA5088-OtA5092	OtA5093-OtA5097
Tfap2c	chr2:172549592-172558621	OtA5088-OtA5092	OtA5093-OtA5097
Tnk2	chr16:32644642-32683493	OtA5088-OtA5092	OtA5093-OtA5097
Trub1	chr19:57452905-57491005	OtA5088-OtA5092	OtA5093-OtA5097
Ube2j2	chr4:155943830-155959604	OtA5088-OtA5092	OtA5093-OtA5097
Ust	chr10:8204752-8518825	OtA5088-OtA5092	OtA5093-OtA5097
Vps37c	chr19:10688814-10714419	OtA5088-OtA5092	OtA5093-OtA5097
Wdr53	chr16:32247226-32257083	OtA5088-OtA5092	OtA5093-OtA5097
Whsc2	chr5:33898179-33936258	OtA5088-OtA5092	OtA5093-OtA5097
Ydjc	chr16:17146966-17148857	OtA5088-OtA5092	OtA5093-OtA5097
Dhx37	chr5:125414403-125434048	OtA5088-OtA5092	OtA5093-OtA5097
Amica1	chr9:45079182-45135606	OtA5088-OtA5092	OtA5093-OtA5097
Amot	chrX:145446423-145487046	OtA5088-OtA5092	OtA5093-OtA5097
Atp5j	chr16:84827870-84863778	OtA5088-OtA5092	OtA5093-OtA5097
Cmpk1	chr4:114960612-114987228	OtA5088-OtA5092	OtA5093-OtA5097
Egflam	chr15:7206119-7398304	OtA5088-OtA5092	OtA5093-OtA5097
Eif1ad	chr19:5366812-5371511	OtA5088-OtA5092	OtA5093-OtA5097
Fam98b	chr2:117249738-117271540	OtA5088-OtA5092	OtA5093-OtA5097
Fn1	chr1:71585522-71653171	OtA5088-OtA5092	OtA5093-OtA5097
Lrrc1	chr9:77430822-77544852	OtA5088-OtA5092	OtA5093-OtA5097
Mak16	chr8:31150315-31168724	OtA5088-OtA5092	OtA5093-OtA5097
Pcdhga10	chr18:36930284-37841872	OtA5088-OtA5092	OtA5093-OtA5097
Pcyox1l	chr18:61696836-61707635	OtA5088-OtA5092	OtA5093-OtA5097
Ppan	chr9:20888174-20892179	OtA5088-OtA5092	OtA5093-OtA5097
Tmem179b	chr19:8772521-8786417	OtA5088-OtA5092	OtA5093-OtA5097
Tmem50a	chr4:134897848-134914916	OtA5088-OtA5092	OtA5093-OtA5097
Ttc32	chr12:9029996-9036394	OtA5088-OtA5092	OtA5093-OtA5097
Zzef1	chr11:72796225-72927120	OtA5088-OtA5092	OtA5093-OtA5097
Alg6	chr4:99715629-99763460	OtA5088-OtA5092	OtA5093-OtA5097
Anks3	chr16:4941414-4964237	OtA5088-OtA5092	OtA5093-OtA5097

Anxa6	chr11:54978961-55033471	OtA5088-OtA5092	OtA5093-OtA5097
Aph1c	chr9:66814993-66834706	OtA5088-OtA5092	OtA5093-OtA5097
Atp5d	chr10:79697304-80369637	OtA5088-OtA5092	OtA5093-OtA5097
Atp7b	chr8:21994347-22060074	OtA5088-OtA5092	OtA5093-OtA5097
BC026590	chr4:56749679-56809605	OtA5088-OtA5092	OtA5093-OtA5097
BC029214	chr2:25459487-25461094	OtA5088-OtA5092	OtA5093-OtA5097
Cldn12	chr5:5505014-5514976	OtA5088-OtA5092	OtA5093-OtA5097
Cmklr1	chr5:113612354-113650390	OtA5088-OtA5092	OtA5093-OtA5097
Ctdp1	chr18:80407958-80469667	OtA5088-OtA5092	OtA5093-OtA5097
E330013P06	chr18:61639652-61665538	OtA5088-OtA5092	OtA5093-OtA5097
Emcn	chr3:137341077-137431069	OtA5088-OtA5092	OtA5093-OtA5097
Ephx1	chr1:180989555-181017495	OtA5088-OtA5092	OtA5093-OtA5097
Exog	chr9:119444922-119465518	OtA5088-OtA5092	OtA5093-OtA5097
Grb2	chr11:115644143-115708597	OtA5088-OtA5092	OtA5093-OtA5097
Homez	chr14:54852736-54864158	OtA5088-OtA5092	OtA5093-OtA5097
Jdp2	chr12:85599104-85639878	OtA5088-OtA5092	OtA5093-OtA5097
Klhl20	chr1:161088377-161131479	OtA5088-OtA5092	OtA5093-OtA5097
Lemd3	chr10:120923410-120979330	OtA5088-OtA5092	OtA5093-OtA5097
Lrrc51	chr7:101912988-101933857	OtA5088-OtA5092	OtA5093-OtA5097
Midn	chr10:79697304-80369637	OtA5088-OtA5092	OtA5093-OtA5097
Mlkl	chr8:111311799-111337903	OtA5088-OtA5092	OtA5093-OtA5097
Npr2	chr4:43631934-43651240	OtA5088-OtA5092	OtA5093-OtA5097
Parp16	chr9:65214689-65239219	OtA5088-OtA5092	OtA5093-OtA5097
Parva	chr7:112427705-112591688	OtA5088-OtA5092	OtA5093-OtA5097
Pdcd2	chr17:15521574-15527301	OtA5088-OtA5092	OtA5093-OtA5097
Pmpca	chr2:26389347-26409188	OtA5088-OtA5092	OtA5093-OtA5097
Pop4	chr7:38262819-38271348	OtA5088-OtA5092	OtA5093-OtA5097
Ppie	chr4:123127124-123139941	OtA5088-OtA5092	OtA5093-OtA5097
Ptpn23	chr9:110385088-110408210	OtA5088-OtA5092	OtA5093-OtA5097
Rbm41	chrX:139889510-139998595	OtA5088-OtA5092	OtA5093-OtA5097
Rexo4	chr2:26953562-26964386	OtA5088-OtA5092	OtA5093-OtA5097
Rgs10	chr7:128373624-128418172	OtA5088-OtA5092	OtA5093-OtA5097
Setd6	chr8:95715912-95719004	OtA5088-OtA5092	OtA5093-OtA5097
Smarcd3	chr5:24592621-24602002	OtA5088-OtA5092	OtA5093-OtA5097
Smo	chr6:29735496-29761366	OtA5088-OtA5092	OtA5093-OtA5097
Srp54a	chr12:55080495-55115367	OtA5088-OtA5092	OtA5093-OtA5097
Stbd1	chr5:92603050-92606579	OtA5088-OtA5092	OtA5093-OtA5097
Sun1	chr5:139200636-139249839	OtA5088-OtA5092	OtA5093-OtA5097
Taf10	chr7:105731729-105744338	OtA5088-OtA5092	OtA5093-OtA5097
Tk2	chr8:104226690-104248558	OtA5088-OtA5092	OtA5093-OtA5097
Tle3	chr9:61372365-61418497	OtA5088-OtA5092	OtA5093-OtA5097
Tor1b	chr2:30953000-30959015	OtA5088-OtA5092	OtA5093-OtA5097
Trmt12	chr15:58872648-58876781	OtA5088-OtA5092	OtA5093-OtA5097
Usp22	chr11:61151780-61175059	OtA5088-OtA5092	OtA5093-OtA5097
Vgf	chr5:137030294-137033351	OtA5088-OtA5092	OtA5093-OtA5097
Xpo4	chr14:57582270-57664956	OtA5088-OtA5092	OtA5093-OtA5097
Zfp3	chr11:70764446-70772928	OtA5088-OtA5092	OtA5093-OtA5097

Metap1	chr3:138458959-138489382	OtA5088-OtA5092	OtA5093-OtA5097
D2Wsu81e	chr2:30171523-30178459	OtA5088-OtA5092	OtA5093-OtA5097
Nt5dc2	chr14:31134852-31168641	OtA5088-OtA5092	OtA5093-OtA5097
Pls1	chr9:95752641-95845279	OtA5088-OtA5092	OtA5093-OtA5097
Selk	chr14:29968379-29975074	OtA5088-OtA5092	OtA5093-OtA5097
Stt3b	chr9:115242581-115310421	OtA5088-OtA5092	OtA5093-OtA5097
Ctnnal1	chr4:56810934-56865211	OtA5088-OtA5092	OtA5093-OtA5097
Ift140	chr17:25016085-25099497	OtA5088-OtA5092	OtA5093-OtA5097
Pop5	chr5:115235850-115240970	OtA5088-OtA5092	OtA5093-OtA5097
Cox4nb	chr8:120653913-120668112	OtA5088-OtA5092	OtA5093-OtA5097
Polrmt	chr10:79697304-80369637	OtA5088-OtA5092	OtA5093-OtA5097
Dsc2	chr18:20030797-20059505	OtA5088-OtA5092	OtA5093-OtA5097
Fam126a	chr5:23787743-24161231	OtA5088-OtA5092	OtA5093-OtA5097
Fbxo33	chr12:59200654-59219483	OtA5088-OtA5092	OtA5093-OtA5097
Pknox1	chr17:31564772-31607693	OtA5088-OtA5092	OtA5093-OtA5097
Sphk2	chr7:45705246-45718002	OtA5088-OtA5092	OtA5093-OtA5097
Tmtc3	chr10:100443901-100487347	OtA5088-OtA5092	OtA5093-OtA5097
Trim41	chr11:48806403-48817391	OtA5088-OtA5092	OtA5093-OtA5097
Zdhhc9	chrX:48171970-48208702	OtA5088-OtA5092	OtA5093-OtA5097
Cox15	chr19:43689688-43753000	OtA5088-OtA5092	OtA5093-OtA5097
Gm5617	chr9:48495344-48495972	OtA5088-OtA5092	OtA5093-OtA5097
Lyn	chr4:3678120-3791612	OtA5088-OtA5092	OtA5093-OtA5097
Mrpl15	chr1:4773199-4785726	OtA5088-OtA5092	OtA5093-OtA5097
Patl2	chr2:122120107-122186189	OtA5088-OtA5092	OtA5093-OtA5097
Phlpp1	chr1:106171868-106394245	OtA5088-OtA5092	OtA5093-OtA5097
Pigm	chr1:172376530-172384099	OtA5088-OtA5092	OtA5093-OtA5097
Ptbp1	chr10:79697304-80369637	OtA5088-OtA5092	OtA5093-OtA5097
Rps8	chr4:117153835-117156132	OtA5088-OtA5092	OtA5093-OtA5097
Terc	chr3:96414436-96414833	OtA5088-OtA5092	OtA5093-OtA5097
Tmem70	chr1:16665190-16678275	OtA5088-OtA5092	OtA5093-OtA5097
Wdr11	chr7:129591862-129635738	OtA5088-OtA5092	OtA5093-OtA5097
Zfp46	chr4:136286068-136293942	OtA5088-OtA5092	OtA5093-OtA5097
Hspb2	chr9:50751071-50752354	OtA5088-OtA5092	OtA5093-OtA5097
Ddr2	chr1:169969408-170088944	OtA5088-OtA5092	OtA5093-OtA5097
Gss	chr2:155563182-155592706	OtA5088-OtA5092	OtA5093-OtA5097
Tgfb1i1	chr7:128246870-128253712	OtA5088-OtA5092	OtA5093-OtA5097
Commd10	chr18:46958875-47087994	OtA5088-OtA5092	OtA5093-OtA5097
Ddx23	chr15:98645506-98662889	OtA5088-OtA5092	OtA5093-OtA5097
Dlgap4	chr2:156613704-156764363	OtA5088-OtA5092	OtA5093-OtA5097
Fam114a2	chr11:57482989-57518644	OtA5088-OtA5092	OtA5093-OtA5097
Fam92a	chr4:12153721-12172015	OtA5088-OtA5092	OtA5093-OtA5097
Fbxl5	chr5:43744617-43782149	OtA5088-OtA5092	OtA5093-OtA5097
Fbxl8	chr8:105264647-105269326	OtA5088-OtA5092	OtA5093-OtA5097
H2-T9	chr17:36038408-36042702	OtA5088-OtA5092	OtA5093-OtA5097
Il22ra1	chr4:135728158-135755383	OtA5088-OtA5092	OtA5093-OtA5097
Ldb1	chr19:46032600-46045211	OtA5088-OtA5092	OtA5093-OtA5097
Lysmd4	chr7:67222543-67228468	OtA5088-OtA5092	OtA5093-OtA5097

Ngrn	chr7:80261214-80265378	OtA5088-OtA5092	OtA5093-OtA5097
Paip2b	chr6:83806058-83831741	OtA5088-OtA5092	OtA5093-OtA5097
Phf10	chr17:14943183-14961260	OtA5088-OtA5092	OtA5093-OtA5097
Rps5	chr7:12922310-12926686	OtA5088-OtA5092	OtA5093-OtA5097
Wdr83	chr8:85071756-85080746	OtA5088-OtA5092	OtA5093-OtA5097
Sepw1	chr7:15917207-15922371	OtA5088-OtA5092	OtA5093-OtA5097
Aes	chr10:81559559-81566364	OtA5088-OtA5092	OtA5093-OtA5097
Epb4.1l3	chr17:69156809-69289987	OtA5088-OtA5092	OtA5093-OtA5097
Palm	chr10:79697304-80369637	OtA5088-OtA5092	OtA5093-OtA5097
Pan2	chr10:128303334-128321358	OtA5088-OtA5092	OtA5093-OtA5097
Parp12	chr6:39086411-39118349	OtA5088-OtA5092	OtA5093-OtA5097
Tbce	chr13:13954673-14039600	OtA5088-OtA5092	OtA5093-OtA5097
Aplp2	chr9:31149556-31211815	OtA5088-OtA5092	OtA5093-OtA5097
Dusp10	chr1:184034460-184075636	OtA5088-OtA5092	OtA5093-OtA5097
Hdac10	chr15:89123302-89128700	OtA5088-OtA5092	OtA5093-OtA5097
Rhog	chr7:102239122-102250118	OtA5088-OtA5092	OtA5093-OtA5097
Rnase4	chr14:51091076-51106151	OtA5088-OtA5092	OtA5093-OtA5097
Slc16a13	chr11:70216791-70220994	OtA5088-OtA5092	OtA5093-OtA5097
Yaf2	chr15:93283832-93336935	OtA5088-OtA5092	OtA5093-OtA5097
Alg11	chr8:22060720-22071627	OtA5088-OtA5092	OtA5093-OtA5097
Amigo3	chr9:108049289-108079375	OtA5088-OtA5092	OtA5093-OtA5097
Cpox	chr16:58670207-58680389	OtA5088-OtA5092	OtA5093-OtA5097
Cyhr1	chr15:76645766-76660108	OtA5088-OtA5092	OtA5093-OtA5097
Fdft1	chr14:63122461-63177793	OtA5088-OtA5092	OtA5093-OtA5097
Fyn	chr10:39369798-39565374	OtA5088-OtA5092	OtA5093-OtA5097
Hscb	chr5:110829069-110839777	OtA5088-OtA5092	OtA5093-OtA5097
Jmjd8	chr17:25829042-25833361	OtA5088-OtA5092	OtA5093-OtA5097
Mrpl1	chr5:96210114-96266709	OtA5088-OtA5092	OtA5093-OtA5097
Rhox5	chrX:37754607-37808878	OtA5088-OtA5092	OtA5093-OtA5097
Sf3b2	chr19:5273920-5295455	OtA5088-OtA5092	OtA5093-OtA5097
Srp9	chr1:182124736-182132415	OtA5088-OtA5092	OtA5093-OtA5097
Taf7	chr18:36930284-37841872	OtA5088-OtA5092	OtA5093-OtA5097
Tmem82	chr4:141614232-141618633	OtA5088-OtA5092	OtA5093-OtA5097
Tmpo	chr10:91147570-91171582	OtA5088-OtA5092	OtA5093-OtA5097
Tob1	chr11:94211453-94215492	OtA5088-OtA5092	OtA5093-OtA5097
Vill	chr9:119052777-119071525	OtA5088-OtA5092	OtA5093-OtA5097
Cyp2d22	chr15:82370526-82380260	OtA5088-OtA5092	OtA5093-OtA5097
Fam160b2	chr14:70583294-70599835	OtA5088-OtA5092	OtA5093-OtA5097
Xpnpep3	chr15:81400187-81454888	OtA5088-OtA5092	OtA5093-OtA5097
Rgs19	chr2:181688422-181693924	OtA5088-OtA5092	OtA5093-OtA5097
Tdg	chr10:82629837-82690650	OtA5088-OtA5092	OtA5093-OtA5097
Smpd4	chr16:17619353-17644830	OtA5088-OtA5092	OtA5093-OtA5097
Zkscan17	chr11:59485520-59511067	OtA5088-OtA5092	OtA5093-OtA5097
Acbd6	chr1:155558119-155687233	OtA5088-OtA5092	OtA5093-OtA5097
Chmp5	chr4:40948552-40965302	OtA5088-OtA5092	OtA5093-OtA5097
Eml4	chr17:83350930-83480359	OtA5088-OtA5092	OtA5093-OtA5097
Glt28d2	chr3:85869845-85887518	OtA5088-OtA5092	OtA5093-OtA5097

Gstt1	chr10:75783812-75798584	OtA5088-OtA5092	OtA5093-OtA5097
Hs2st1	chr3:144431106-144570216	OtA5088-OtA5092	OtA5093-OtA5097
Ptcd1	chr5:145140396-145167104	OtA5088-OtA5092	OtA5093-OtA5097
Rps25	chr9:44407713-44418007	OtA5088-OtA5092	OtA5093-OtA5097
Scarf2	chr16:17797281-17808287	OtA5088-OtA5092	OtA5093-OtA5097
Thap11	chr8:105855102-105856950	OtA5088-OtA5092	OtA5093-OtA5097
Tmem11	chr11:60864451-60879038	OtA5088-OtA5092	OtA5093-OtA5097
Tmem164	chrX:142681399-142843494	OtA5088-OtA5092	OtA5093-OtA5097
Trmt61b	chr17:71552060-71598761	OtA5088-OtA5092	OtA5093-OtA5097
Eif3h	chr15:51786562-51865461	OtA5088-OtA5092	OtA5093-OtA5097
Smc4	chr3:69004971-69034623	OtA5088-OtA5092	OtA5093-OtA5097
Cd81	chr7:143032620-143067930	OtA5088-OtA5092	OtA5093-OtA5097
Gpx4	chr10:79697304-80369637	OtA5088-OtA5092	OtA5093-OtA5097
Slc16a12	chr19:34668405-34747111	OtA5088-OtA5092	OtA5093-OtA5097
Tpm1	chr9:67022592-67049213	OtA5088-OtA5092	OtA5093-OtA5097
Uba2	chr7:34140696-34168529	OtA5088-OtA5092	OtA5093-OtA5097
Anp32b	chr4:46451116-46472523	OtA5088-OtA5092	OtA5093-OtA5097
Ccdc91	chr6:147475870-147632612	OtA5088-OtA5092	OtA5093-OtA5097
Wnt6	chr1:74771891-74785319	OtA5088-OtA5092	OtA5093-OtA5097
Ctso	chr3:81932615-81956725	OtA5088-OtA5092	OtA5093-OtA5097
Mrp63	chr14:57826238-57828745	OtA5088-OtA5092	OtA5093-OtA5097
Polr2b	chr5:77310483-77408045	OtA5088-OtA5092	OtA5093-OtA5097
Ube2e1	chr14:18271141-18331844	OtA5088-OtA5092	OtA5093-OtA5097
Zcchc9	chr13:91796532-91807696	OtA5088-OtA5092	OtA5093-OtA5097
Tril	chr6:53815467-53820825	OtA5088-OtA5092	OtA5093-OtA5097
Abca7	chr10:79697304-80369637	OtA5088-OtA5092	OtA5093-OtA5097
Adck5	chr15:76576358-76607591	OtA5088-OtA5092	OtA5093-OtA5097
Ahnak	chr19:8989283-9076919	OtA5088-OtA5092	OtA5093-OtA5097
Alas2	chrX:150547416-150570622	OtA5088-OtA5092	OtA5093-OtA5097
Angel1	chr12:86700501-86726460	OtA5088-OtA5092	OtA5093-OtA5097
Atp2a2	chr5:122453512-122502225	OtA5088-OtA5092	OtA5093-OtA5097
BC046331	chr4:153975560-154007225	OtA5088-OtA5092	OtA5093-OtA5097
Brix1	chr15:10474778-10485937	OtA5088-OtA5092	OtA5093-OtA5097
Chic2	chr5:75006423-75044626	OtA5088-OtA5092	OtA5093-OtA5097
Cln3	chr7:126571399-126584280	OtA5088-OtA5092	OtA5093-OtA5097
Cyr61	chr3:145646970-145649985	OtA5088-OtA5092	OtA5093-OtA5097
D10Wsu52e	chr10:85938636-85957793	OtA5088-OtA5092	OtA5093-OtA5097
Dcaf15	chr8:84097071-84104762	OtA5088-OtA5092	OtA5093-OtA5097
Dmwd	chr7:19076199-19082775	OtA5088-OtA5092	OtA5093-OtA5097
Ercc1	chr7:19345070-19359483	OtA5088-OtA5092	OtA5093-OtA5097
Erich1	chr8:14027564-14090327	OtA5088-OtA5092	OtA5093-OtA5097
Erlin1	chr19:44034942-44069785	OtA5088-OtA5092	OtA5093-OtA5097
Fam179b	chr12:64965741-65022573	OtA5088-OtA5092	OtA5093-OtA5097
Fdps	chr3:89093587-89101967	OtA5088-OtA5092	OtA5093-OtA5097
Fubp3	chr2:31572724-31617519	OtA5088-OtA5092	OtA5093-OtA5097
Gm6548	chr17:78850504-78852544	OtA5088-OtA5092	OtA5093-OtA5097
Gramd4	chr15:86057694-86137636	OtA5088-OtA5092	OtA5093-OtA5097

Hinfp	chr9:44295672-44305671	OtA5088-OtA5092	OtA5093-OtA5097
Il21r	chr7:125603428-125633570	OtA5088-OtA5092	OtA5093-OtA5097
Marveld1	chr19:42147388-42151703	OtA5088-OtA5092	OtA5093-OtA5097
Nhp2l1	chr15:82041344-82047598	OtA5088-OtA5092	OtA5093-OtA5097
Noc2l	chr4:156236009-156255338	OtA5088-OtA5092	OtA5093-OtA5097
Pear1	chr3:87749096-87768953	OtA5088-OtA5092	OtA5093-OtA5097
Phf1	chr17:26933126-26937890	OtA5088-OtA5092	OtA5093-OtA5097
Phyhipl	chr10:70440667-70599291	OtA5088-OtA5092	OtA5093-OtA5097
Pms2	chr5:143910000-143931756	OtA5088-OtA5092	OtA5093-OtA5097
Ppfibp2	chr7:107595050-107758032	OtA5088-OtA5092	OtA5093-OtA5097
Rab11fip5	chr6:85334961-85374634	OtA5088-OtA5092	OtA5093-OtA5097
Rragc	chr4:123917432-123936997	OtA5088-OtA5092	OtA5093-OtA5097
Sema6a	chr18:47245253-47368868	OtA5088-OtA5092	OtA5093-OtA5097
Shf	chr2:122348891-122368918	OtA5088-OtA5092	OtA5093-OtA5097
Sil1	chr18:35266395-35498925	OtA5088-OtA5092	OtA5093-OtA5097
Spata5l1	chr2:122630624-122632704	OtA5088-OtA5092	OtA5093-OtA5097
Thap7	chr16:17527981-17531052	OtA5088-OtA5092	OtA5093-OtA5097
Thyn1	chr9:26999676-27030094	OtA5088-OtA5092	OtA5093-OtA5097
Tmem141	chr2:25620065-25622005	OtA5088-OtA5092	OtA5093-OtA5097
Tmem184c	chr8:77595977-77610653	OtA5088-OtA5092	OtA5093-OtA5097
Ulk1	chr5:110784488-110810081	OtA5088-OtA5092	OtA5093-OtA5097
Vamp4	chr1:162570827-162599078	OtA5088-OtA5092	OtA5093-OtA5097
Wdr36	chr18:32663641-32866420	OtA5088-OtA5092	OtA5093-OtA5097
Yy1	chr12:108793310-108816632	OtA5088-OtA5092	OtA5093-OtA5097
Zfp110	chr7:12834809-12850584	OtA5088-OtA5092	OtA5093-OtA5097
Meis3	chr7:16175379-16186503	OtA5088-OtA5092	OtA5093-OtA5097
Abcc3	chr11:94343294-94392976	OtA5088-OtA5092	OtA5093-OtA5097
Abhd14b	chr9:106448639-106452918	OtA5088-OtA5092	OtA5093-OtA5097
Acss2	chr2:155518042-155562743	OtA5088-OtA5092	OtA5093-OtA5097
Aebp2	chr6:140622662-140677418	OtA5088-OtA5092	OtA5093-OtA5097
Agpat5	chr8:18846278-18884413	OtA5088-OtA5092	OtA5093-OtA5097
Ak3	chr19:29020831-29048729	OtA5088-OtA5092	OtA5093-OtA5097
Akr1e1	chr13:4592489-4609164	OtA5088-OtA5092	OtA5093-OtA5097
Ankfy1	chr11:72690001-72772146	OtA5088-OtA5092	OtA5093-OtA5097
Ankib1	chr5:3689999-3803109	OtA5088-OtA5092	OtA5093-OtA5097
Apoc1	chr7:19689480-19692658	OtA5088-OtA5092	OtA5093-OtA5097
Apold1	chr6:134982000-134986836	OtA5088-OtA5092	OtA5093-OtA5097
Aqr	chr2:114101171-114175339	OtA5088-OtA5092	OtA5093-OtA5097
Auh	chr13:52835109-52929677	OtA5088-OtA5092	OtA5093-OtA5097
B3gnt3	chr8:71691719-71701800	OtA5088-OtA5092	OtA5093-OtA5097
BC068281	chr12:4843302-4856967	OtA5088-OtA5092	OtA5093-OtA5097
Bbs9	chr9:22475714-22888280	OtA5088-OtA5092	OtA5093-OtA5097
Bend5	chr4:110397790-111657510	OtA5088-OtA5092	OtA5093-OtA5097
Bicc1	chr10:70925095-71159634	OtA5088-OtA5092	OtA5093-OtA5097
Bnip1	chr17:26781078-26792521	OtA5088-OtA5092	OtA5093-OtA5097
C1s	chr6:124530343-124542359	OtA5088-OtA5092	OtA5093-OtA5097
Camsap2	chr1:136268122-136346104	OtA5088-OtA5092	OtA5093-OtA5097

Cbr4	chr8:61487733-61503500	OtA5088-OtA5092	OtA5093-OtA5097
Cc2d1b	chr4:108619955-108634122	OtA5088-OtA5092	OtA5093-OtA5097
Ccdc77	chr6:120324321-120444574	OtA5088-OtA5092	OtA5093-OtA5097
Ccnj	chr19:40831278-40848570	OtA5088-OtA5092	OtA5093-OtA5097
Cflar	chr1:58713285-58758882	OtA5088-OtA5092	OtA5093-OtA5097
Chfr	chr5:110135839-110176504	OtA5088-OtA5092	OtA5093-OtA5097
Cldn25	chr16:58727909-58734247	OtA5088-OtA5092	OtA5093-OtA5097
Clmn	chr12:104763113-104865076	OtA5088-OtA5092	OtA5093-OtA5097
Cnot7	chr8:40493035-40511304	OtA5088-OtA5092	OtA5093-OtA5097
Cops6	chr5:138161101-138163984	OtA5088-OtA5092	OtA5093-OtA5097
Cox10	chr11:63962626-64079472	OtA5088-OtA5092	OtA5093-OtA5097
Cox6c	chr15:35931975-35938246	OtA5088-OtA5092	OtA5093-OtA5097
Cox7c	chr13:86044797-86046795	OtA5088-OtA5092	OtA5093-OtA5097
Cpne1	chr2:156071840-156111965	OtA5088-OtA5092	OtA5093-OtA5097
Cul4a	chr8:13105720-13147939	OtA5088-OtA5092	OtA5093-OtA5097
Dag1	chr9:108205957-108263736	OtA5088-OtA5092	OtA5093-OtA5097
Dbp	chr7:45705246-45718002	OtA5088-OtA5092	OtA5093-OtA5097
Dem1	chr4:120921201-120925005	OtA5088-OtA5092	OtA5093-OtA5097
Drg1	chr11:3202352-3479831	OtA5088-OtA5092	OtA5093-OtA5097
Dync1h1	chr12:110601394-110666944	OtA5088-OtA5092	OtA5093-OtA5097
E2f6	chr12:16810964-16826752	OtA5088-OtA5092	OtA5093-OtA5097
Eci2	chr13:34977747-34994144	OtA5088-OtA5092	OtA5093-OtA5097
Ecm1	chr3:95734147-95739569	OtA5088-OtA5092	OtA5093-OtA5097
Eif1a	chr18:46597703-46610225	OtA5088-OtA5092	OtA5093-OtA5097
Eif3m	chr2:104886323-105017027	OtA5088-OtA5092	OtA5093-OtA5097
Elp3	chr14:65530445-65593112	OtA5088-OtA5092	OtA5093-OtA5097
Emg1	chr6:124663103-124712178	OtA5088-OtA5092	OtA5093-OtA5097
Etv5	chr16:22381312-22439570	OtA5088-OtA5092	OtA5093-OtA5097
Eya3	chr4:132639045-132724763	OtA5088-OtA5092	OtA5093-OtA5097
Fam108b	chr19:21653308-21685637	OtA5088-OtA5092	OtA5093-OtA5097
Fam162a	chr16:36043843-36071515	OtA5088-OtA5092	OtA5093-OtA5097
Fancg	chr4:43002336-43010301	OtA5088-OtA5092	OtA5093-OtA5097
Fbxo38	chr18:62504058-62548743	OtA5088-OtA5092	OtA5093-OtA5097
Frmd8	chr19:5850973-5875208	OtA5088-OtA5092	OtA5093-OtA5097
Fstl3	chr10:79697304-80369637	OtA5088-OtA5092	OtA5093-OtA5097
Gas5	chr1:161035165-161038537	OtA5088-OtA5092	OtA5093-OtA5097
Gla	chrX:134588168-134601005	OtA5088-OtA5092	OtA5093-OtA5097
Gosr2	chr11:103676848-103697710	OtA5088-OtA5092	OtA5093-OtA5097
Gp1bb	chr16:18620318-18629938	OtA5088-OtA5092	OtA5093-OtA5097
Gpr155	chr2:73341506-73386398	OtA5088-OtA5092	OtA5093-OtA5097
H2-T23	chr17:35994503-36038174	OtA5088-OtA5092	OtA5093-OtA5097
Hipk3	chr2:104426481-104494489	OtA5088-OtA5092	OtA5093-OtA5097
Hist1h1e	chr13:23583669-23622558	OtA5088-OtA5092	OtA5093-OtA5097
Hpx	chr7:105591610-105600116	OtA5088-OtA5092	OtA5093-OtA5097
Hrct1	chr4:43727197-43728110	OtA5088-OtA5092	OtA5093-OtA5097
Hsdl2	chr4:59581562-59618694	OtA5088-OtA5092	OtA5093-OtA5097
Inpp5d	chr1:87620311-87720508	OtA5088-OtA5092	OtA5093-OtA5097

Inpp5f		chr7:128611364-128696436	OtA5088-OtA5092	OtA5093-OtA5097
Ints12		chr3:133091952-133110985	OtA5088-OtA5092	OtA5093-OtA5097
Irgm2		chr11:58214976-58222783	OtA5088-OtA5092	OtA5093-OtA5097
Isy1		chr6:87818447-87838759	OtA5088-OtA5092	OtA5093-OtA5097
Kank2		chr9:21766772-21798546	OtA5088-OtA5092	OtA5093-OtA5097
Kctd10		chr5:114314940-114380505	OtA5088-OtA5092	OtA5093-OtA5097
Klc2		chr19:5107745-5118408	OtA5088-OtA5092	OtA5093-OtA5097
Klhl7		chr5:23787743-24161231	OtA5088-OtA5092	OtA5093-OtA5097
Lactb2		chr1:13625899-13660509	OtA5088-OtA5092	OtA5093-OtA5097
Lix1l		chr3:96601132-96629819	OtA5088-OtA5092	OtA5093-OtA5097
Ltbp1		chr17:75005528-75392967	OtA5088-OtA5092	OtA5093-OtA5097
Mapkap1		chr2:34432024-34624950	OtA5088-OtA5092	OtA5093-OtA5097
	3-Mar	chr18:56761715-56925548	OtA5088-OtA5092	OtA5093-OtA5097
	8-Mar	chr6:116338122-116409540	OtA5088-OtA5092	OtA5093-OtA5097
Marcks		chr10:37133242-37138926	OtA5088-OtA5092	OtA5093-OtA5097
Mcm6		chr1:128331590-128359656	OtA5088-OtA5092	OtA5093-OtA5097
Mcts1		chrX:38600657-38613522	OtA5088-OtA5092	OtA5093-OtA5097
Med26		chr8:72494558-72548310	OtA5088-OtA5092	OtA5093-OtA5097
Mettl10		chr7:132827460-132852647	OtA5088-OtA5092	OtA5093-OtA5097
Mlf2		chr6:124931387-124936149	OtA5088-OtA5092	OtA5093-OtA5097
Mmab		chr5:114431033-114444027	OtA5088-OtA5092	OtA5093-OtA5097
Mon2		chr10:122992060-123076505	OtA5088-OtA5092	OtA5093-OtA5097
Mrps10		chr17:47368886-47378679	OtA5088-OtA5092	OtA5093-OtA5097
Mrps2		chr2:28468065-28471177	OtA5088-OtA5092	OtA5093-OtA5097
Mrps27		chr13:99344785-99415561	OtA5088-OtA5092	OtA5093-OtA5097
Myo5a		chr9:75071205-75223687	OtA5088-OtA5092	OtA5093-OtA5097
Nfkbiz		chr16:55811376-55838641	OtA5088-OtA5092	OtA5093-OtA5097
Nipal4		chr11:46148154-46166359	OtA5088-OtA5092	OtA5093-OtA5097
Nme5		chr18:34562640-34579106	OtA5088-OtA5092	OtA5093-OtA5097
Npc2		chr12:84754558-84773112	OtA5088-OtA5092	OtA5093-OtA5097
Nprl3		chr11:32226504-32267614	OtA5088-OtA5092	OtA5093-OtA5097
Nr2c1		chr10:94147930-94197214	OtA5088-OtA5092	OtA5093-OtA5097
Nradd		chr9:110621134-110624393	OtA5088-OtA5092	OtA5093-OtA5097
ORF61		chr10:79697304-80369637	OtA5088-OtA5092	OtA5093-OtA5097
Pard3		chr8:127064055-127612286	OtA5088-OtA5092	OtA5093-OtA5097
Pde8a		chr7:81213803-81333622	OtA5088-OtA5092	OtA5093-OtA5097
Pes1		chr11:3963974-3980004	OtA5088-OtA5092	OtA5093-OtA5097
Pfkp		chr13:6548156-6648724	OtA5088-OtA5092	OtA5093-OtA5097
Pigb		chr9:72985550-73039699	OtA5088-OtA5092	OtA5093-OtA5097
Pigf		chr17:86963110-87025401	OtA5088-OtA5092	OtA5093-OtA5097
Pih1d1		chr7:45141839-45160064	OtA5088-OtA5092	OtA5093-OtA5097
Pik3c3		chr18:30272895-30348120	OtA5088-OtA5092	OtA5093-OtA5097
Pik3ip1		chr11:3202352-3479831	OtA5088-OtA5092	OtA5093-OtA5097
Pkp2		chr16:16213344-16272712	OtA5088-OtA5092	OtA5093-OtA5097
Pnkd		chr1:74285033-74353692	OtA5088-OtA5092	OtA5093-OtA5097
Pole3		chr4:62523800-62525014	OtA5088-OtA5092	OtA5093-OtA5097
Polh		chr17:46171992-46202625	OtA5088-OtA5092	OtA5093-OtA5097

Ppat	chr5:76913248-76967505	OtA5088-OtA5092	OtA5093-OtA5097
Ppil2	chr16:17070310-17111252	OtA5088-OtA5092	OtA5093-OtA5097
Ppp6c	chr2:39196797-39226338	OtA5088-OtA5092	OtA5093-OtA5097
Pprc1	chr19:46056538-46072909	OtA5088-OtA5092	OtA5093-OtA5097
Prelid2	chr18:41875695-41951194	OtA5088-OtA5092	OtA5093-OtA5097
Prex1	chr2:166566344-166713832	OtA5088-OtA5092	OtA5093-OtA5097
Prickle1	chr15:93499113-93595891	OtA5088-OtA5092	OtA5093-OtA5097
Prosapip1	chr2:130632838-130642803	OtA5088-OtA5092	OtA5093-OtA5097
Psmb7	chr2:38588045-38643906	OtA5088-OtA5092	OtA5093-OtA5097
Psme1	chr14:55578493-55585254	OtA5088-OtA5092	OtA5093-OtA5097
Ptges	chr2:30889470-30903297	OtA5088-OtA5092	OtA5093-OtA5097
Ptprg	chr14:11553552-12242039	OtA5088-OtA5092	OtA5093-OtA5097
Qrich1	chr9:108517086-108560167	OtA5088-OtA5092	OtA5093-OtA5097
Qrsl1	chr10:43874189-43901736	OtA5088-OtA5092	OtA5093-OtA5097
Rab17	chr1:90958132-90969620	OtA5088-OtA5092	OtA5093-OtA5097
Rab1b	chr19:5099206-5106996	OtA5088-OtA5092	OtA5093-OtA5097
Rab9	chrX:166457251-166479867	OtA5088-OtA5092	OtA5093-OtA5097
Rbfa	chr18:80192263-80200619	OtA5088-OtA5092	OtA5093-OtA5097
Rbks	chr5:31624438-31697610	OtA5088-OtA5092	OtA5093-OtA5097
Rbm14	chr19:4800924-4811634	OtA5088-OtA5092	OtA5093-OtA5097
Rgl3	chr9:21971526-21989453	OtA5088-OtA5092	OtA5093-OtA5097
Rhbdf2	chr11:116598165-116627019	OtA5088-OtA5092	OtA5093-OtA5097
Rinl	chr7:28788968-28798966	OtA5088-OtA5092	OtA5093-OtA5097
Rnaseh2c	chr19:5601872-5602959	OtA5088-OtA5092	OtA5093-OtA5097
Rnf126	chr10:79697304-80369637	OtA5088-OtA5092	OtA5093-OtA5097
Rph3a	chr5:120940499-121009518	OtA5088-OtA5092	OtA5093-OtA5097
Rplp0	chr5:115559466-115563729	OtA5088-OtA5092	OtA5093-OtA5097
Rps10	chr17:27630428-27635242	OtA5088-OtA5092	OtA5093-OtA5097
Rps29	chr12:69157721-69159186	OtA5088-OtA5092	OtA5093-OtA5097
Rps6ka1	chr4:133847289-133887799	OtA5088-OtA5092	OtA5093-OtA5097
Rps6kc1	chr1:190772878-190911770	OtA5088-OtA5092	OtA5093-OtA5097
Rrp8	chr7:105731729-105744338	OtA5088-OtA5092	OtA5093-OtA5097
Rsl1d1	chr16:11193036-11203292	OtA5088-OtA5092	OtA5093-OtA5097
Sap30bp	chr11:115933658-115965534	OtA5088-OtA5092	OtA5093-OtA5097
Scoc	chr8:83434492-83458391	OtA5088-OtA5092	OtA5093-OtA5097
Sesn1	chr10:41810573-41908436	OtA5088-OtA5092	OtA5093-OtA5097
Sh3bgrl3	chr4:134127405-134128753	OtA5088-OtA5092	OtA5093-OtA5097
Sirt4	chr5:115478009-115484484	OtA5088-OtA5092	OtA5093-OtA5097
Slc19a2	chr1:164249054-164265368	OtA5088-OtA5092	OtA5093-OtA5097
Slc25a28	chr19:43663800-43674881	OtA5088-OtA5092	OtA5093-OtA5097
Slc25a45	chr19:5878465-5885768	OtA5088-OtA5092	OtA5093-OtA5097
Slc31a1	chr4:62360700-62391769	OtA5088-OtA5092	OtA5093-OtA5097
Slco3a1	chr7:74275417-74554780	OtA5088-OtA5092	OtA5093-OtA5097
Smarce1	chr11:99209047-99231017	OtA5088-OtA5092	OtA5093-OtA5097
Snrnp25	chr11:32205414-32208995	OtA5088-OtA5092	OtA5093-OtA5097
Sparc	chr11:55394500-55420080	OtA5088-OtA5092	OtA5093-OtA5097
Ssbp3	chr4:106911469-107049694	OtA5088-OtA5092	OtA5093-OtA5097

Stoml2	chr4:43027689-43031384	OtA5088-OtA5092	OtA5093-OtA5097
Tcof1	chr18:60813755-60848964	OtA5088-OtA5092	OtA5093-OtA5097
Tfg	chr16:56690328-56717450	OtA5088-OtA5092	OtA5093-OtA5097
Tgds	chr14:118111910-118132765	OtA5088-OtA5092	OtA5093-OtA5097
Tmbim6	chr15:99392946-99410049	OtA5088-OtA5092	OtA5093-OtA5097
Tmeff1	chr4:48585192-48663131	OtA5088-OtA5092	OtA5093-OtA5097
Tmem140	chr6:34863145-34878065	OtA5088-OtA5092	OtA5093-OtA5097
Tmem176b	chr6:48833811-48841374	OtA5088-OtA5092	OtA5093-OtA5097
Tmem8	chr17:26113315-26123253	OtA5088-OtA5092	OtA5093-OtA5097
Traf3ip1	chr1:91494667-91529307	OtA5088-OtA5092	OtA5093-OtA5097
Trim7	chr11:48826137-48850195	OtA5088-OtA5092	OtA5093-OtA5097
Trnau1ap	chr4:132311762-132329538	OtA5088-OtA5092	OtA5093-OtA5097
Trnp1	chr4:133491099-133498550	OtA5088-OtA5092	OtA5093-OtA5097
Ubqln4	chr3:88553715-88569725	OtA5088-OtA5092	OtA5093-OtA5097
Vopp1	chr6:57752263-57825125	OtA5088-OtA5092	OtA5093-OtA5097
Zc3h14	chr12:98746967-98901484	OtA5088-OtA5092	OtA5093-OtA5097
Zdhhc16	chr19:41933471-41981136	OtA5088-OtA5092	OtA5093-OtA5097
Zfp167	chr9:122888470-122896124	OtA5088-OtA5092	OtA5093-OtA5097
Zfp653	chr9:22055459-22389206	OtA5088-OtA5092	OtA5093-OtA5097
Zfp772	chr7:7202121-7209998	OtA5088-OtA5092	OtA5093-OtA5097
Zscan29	chr2:121157426-121171149	OtA5088-OtA5092	OtA5093-OtA5097
Zswim7	chr11:62267223-62281395	OtA5088-OtA5092	OtA5093-OtA5097
Cdkl2	chr5:92006074-92043018	OtA5088-OtA5092	OtA5093-OtA5097
Dusp19	chr2:80617213-80631661	OtA5088-OtA5092	OtA5093-OtA5097
Med19	chr2:84678401-84715164	OtA5088-OtA5092	OtA5093-OtA5097
Tmco6	chr18:36735069-36744548	OtA5088-OtA5092	OtA5093-OtA5097
Nfil3	chr13:52967208-52981039	OtA5088-OtA5092	OtA5093-OtA5097
Pitx2	chr3:129199917-129219590	OtA5088-OtA5092	OtA5093-OtA5097
Abi3	chr11:95824499-95845731	OtA5088-OtA5092	OtA5093-OtA5097
Afp	chr5:90490713-90508907	OtA5088-OtA5092	OtA5093-OtA5097
Fam189b	chr3:89183224-89189289	OtA5088-OtA5092	OtA5093-OtA5097
Las1l	chrX:95935312-95956959	OtA5088-OtA5092	OtA5093-OtA5097
Pgk1	chrX:106187123-106203699	OtA5088-OtA5092	OtA5093-OtA5097
Rint1	chr5:23787743-24161231	OtA5088-OtA5092	OtA5093-OtA5097
Slc16a4	chr3:107291291-107312116	OtA5088-OtA5092	OtA5093-OtA5097
Capn12	chr7:28881655-28962280	OtA5088-OtA5092	OtA5093-OtA5097
Sco1	chr11:67052669-67067440	OtA5088-OtA5092	OtA5093-OtA5097
Zfp809	chr9:22055459-22389206	OtA5088-OtA5092	OtA5093-OtA5097
Gjb5	chr4:127354808-127358164	OtA5088-OtA5092	OtA5093-OtA5097
Capzb	chr4:139192930-139291812	OtA5088-OtA5092	OtA5093-OtA5097
Ftsj1	chrX:8238667-8252406	OtA5088-OtA5092	OtA5093-OtA5097
Hars	chr18:36766527-36783205	OtA5088-OtA5092	OtA5093-OtA5097
Parp4	chr14:56575618-56659798	OtA5088-OtA5092	OtA5093-OtA5097
Slc10a7	chr8:78509334-78734005	OtA5088-OtA5092	OtA5093-OtA5097
Trim39	chr17:36258872-36272004	OtA5088-OtA5092	OtA5093-OtA5097
Tmtc4	chr14:122918974-122983261	OtA5088-OtA5092	OtA5093-OtA5097
Map3k10	chr7:27656376-27674598	OtA5088-OtA5092	OtA5093-OtA5097

Pigu	chr2:155278251-155357424	OtA5088-OtA5092	OtA5093-OtA5097
Gse1	chr8:120488865-120589075	OtA5088-OtA5092	OtA5093-OtA5097
Rdh12	chr12:79208913-79222661	OtA5088-OtA5092	OtA5093-OtA5097
Tmem65	chr15:58782268-58823427	OtA5088-OtA5092	OtA5093-OtA5097
Dhx8	chr11:101732955-101767357	OtA5088-OtA5092	OtA5093-OtA5097
Pfdn4	chr2:170482964-170519070	OtA5088-OtA5092	OtA5093-OtA5097
Klf15	chr6:90462625-90475209	OtA5088-OtA5092	OtA5093-OtA5097
Nsmaf	chr4:6396207-6454271	OtA5088-OtA5092	OtA5093-OtA5097
Tnfrsf1a	chr6:125349722-125362483	OtA5088-OtA5092	OtA5093-OtA5097
Nptn	chr9:58582239-58741559	OtA5088-OtA5092	OtA5093-OtA5097
Gfer	chr17:24693190-24696156	OtA5088-OtA5092	OtA5093-OtA5097
Gm11517	chr11:96789135-96808640	OtA5088-OtA5092	OtA5093-OtA5097
Atg16l1	chr1:87756010-87792428	OtA5088-OtA5092	OtA5093-OtA5097
Qsox2	chr2:26209129-26237420	OtA5088-OtA5092	OtA5093-OtA5097
Gngt2	chr11:95824499-95845731	OtA5088-OtA5092	OtA5093-OtA5097
Myo6	chr9:80165033-80311729	OtA5088-OtA5092	OtA5093-OtA5097
Prdm1	chr10:44437174-44458687	OtA5088-OtA5092	OtA5093-OtA5097
Atpaf2	chr11:60400623-60417099	OtA5088-OtA5092	OtA5093-OtA5097
D4Ertd22e	chr4:141113014-141139796	OtA5088-OtA5092	OtA5093-OtA5097
Epm2aip1	chr9:111271844-111279091	OtA5088-OtA5092	OtA5093-OtA5097
Mrpl32	chr13:14610300-14613037	OtA5088-OtA5092	OtA5093-OtA5097
Acad9	chr3:36065999-36092857	OtA5088-OtA5092	OtA5093-OtA5097
Arhgap9	chr10:127323726-127341579	OtA5088-OtA5092	OtA5093-OtA5097
Asns	chr6:7675170-7693182	OtA5088-OtA5092	OtA5093-OtA5097
C030046I01Rik	chr10:79697304-80369637	OtA5088-OtA5092	OtA5093-OtA5097
Dhx29	chr13:112927792-112969187	OtA5088-OtA5092	OtA5093-OtA5097
Dos	chr10:79697304-80369637	OtA5088-OtA5092	OtA5093-OtA5097
Dusp1	chr17:26505590-26508472	OtA5088-OtA5092	OtA5093-OtA5097
Eif4g1	chr16:20672748-20692883	OtA5088-OtA5092	OtA5093-OtA5097
Etd	chrX:53434917-53443576	OtA5088-OtA5092	OtA5093-OtA5097
Fkbp14	chr6:54577604-54593128	OtA5088-OtA5092	OtA5093-OtA5097
Foxk2	chr11:121259986-121309896	OtA5088-OtA5092	OtA5093-OtA5097
Gna13	chr11:109354777-109401369	OtA5088-OtA5092	OtA5093-OtA5097
Hif1a	chr12:73907866-73947530	OtA5088-OtA5092	OtA5093-OtA5097
Hnrnpul1	chr7:25722038-25754720	OtA5088-OtA5092	OtA5093-OtA5097
Hr	chr14:70554055-70573542	OtA5088-OtA5092	OtA5093-OtA5097
Ifi27l1	chr12:103434188-103440245	OtA5088-OtA5092	OtA5093-OtA5097
Ino80e	chr7:126847552-126861463	OtA5088-OtA5092	OtA5093-OtA5097
Itgb8	chr12:119162802-119238276	OtA5088-OtA5092	OtA5093-OtA5097
Kdm1a	chr4:136550532-136602723	OtA5088-OtA5092	OtA5093-OtA5097
Klhl5	chr5:65131230-65168142	OtA5088-OtA5092	OtA5093-OtA5097
Lsg1	chr16:30312423-31275697	OtA5088-OtA5092	OtA5093-OtA5097
Lynx1	chr15:74747855-74752979	OtA5088-OtA5092	OtA5093-OtA5097
Mark3	chr12:111574509-111656227	OtA5088-OtA5092	OtA5093-OtA5097
Nfe2l1	chr11:96817413-96829968	OtA5088-OtA5092	OtA5093-OtA5097
Nfyc	chr4:120757434-120831575	OtA5088-OtA5092	OtA5093-OtA5097
Odf2	chr2:29889719-29931746	OtA5088-OtA5092	OtA5093-OtA5097

Plagl1	chr10:13090787-13131695	OtA5088-OtA5092	OtA5093-OtA5097
Ppp1r3c	chr19:36731730-36736604	OtA5088-OtA5092	OtA5093-OtA5097
Rhot2	chr17:25838837-25861515	OtA5088-OtA5092	OtA5093-OtA5097
Rpusd1	chr17:25727750-25731456	OtA5088-OtA5092	OtA5093-OtA5097
Scamp1	chr13:94201432-94285281	OtA5088-OtA5092	OtA5093-OtA5097
Senp7	chr16:56075408-56190011	OtA5088-OtA5092	OtA5093-OtA5097
Serpinb9c	chr13:33149274-33159754	OtA5088-OtA5092	OtA5093-OtA5097
Snrnp35	chr5:124483154-124491122	OtA5088-OtA5092	OtA5093-OtA5097
Tspyl2	chrX:152336851-152342484	OtA5088-OtA5092	OtA5093-OtA5097
Usp3	chr9:66517880-66592980	OtA5088-OtA5092	OtA5093-OtA5097
Usp46	chr5:74000037-74068411	OtA5088-OtA5092	OtA5093-OtA5097
Utp15	chr13:98246844-98262992	OtA5088-OtA5092	OtA5093-OtA5097
Ywhaq	chr12:21390328-21417436	OtA5088-OtA5092	OtA5093-OtA5097
Zfat	chr15:68083737-68258856	OtA5088-OtA5092	OtA5093-OtA5097
Zfp651	chr9:121760032-121771742	OtA5088-OtA5092	OtA5093-OtA5097
Zgpat	chr2:181357689-181380793	OtA5088-OtA5092	OtA5093-OtA5097
Slc37a1	chr17:31295482-31350698	OtA5088-OtA5092	OtA5093-OtA5097
Fmo2	chr1:162875046-162898712	OtA5088-OtA5092	OtA5093-OtA5097
Cars2	chr8:11514016-11550771	OtA5088-OtA5092	OtA5093-OtA5097
Fam164a	chr3:7503425-7553848	OtA5088-OtA5092	OtA5093-OtA5097
Pbxip1	chr3:89436703-89450952	OtA5088-OtA5092	OtA5093-OtA5097
Pelo	chr13:114958080-115101964	OtA5088-OtA5092	OtA5093-OtA5097
Polr1d	chr5:147077540-147111359	OtA5088-OtA5092	OtA5093-OtA5097
Slc9a3r1	chr11:115163340-115181178	OtA5088-OtA5092	OtA5093-OtA5097
Spire1	chr18:67488208-67552721	OtA5088-OtA5092	OtA5093-OtA5097
Zfp64	chr2:168925360-168955587	OtA5088-OtA5092	OtA5093-OtA5097
Zrsr1	chr11:22899727-22982284	OtA5088-OtA5092	OtA5093-OtA5097
Arl8b	chr6:108783058-108823723	OtA5088-OtA5092	OtA5093-OtA5097
Bmp2k	chr5:96997688-97111596	OtA5088-OtA5092	OtA5093-OtA5097
Caskin2	chr11:115799351-115813592	OtA5088-OtA5092	OtA5093-OtA5097
Cxxc5	chr18:35829817-35861688	OtA5088-OtA5092	OtA5093-OtA5097
Rac3	chr11:120721467-120723969	OtA5088-OtA5092	OtA5093-OtA5097
Ssr1	chr13:37971400-37994190	OtA5088-OtA5092	OtA5093-OtA5097
Minos1	chr4:139101813-139131113	OtA5088-OtA5092	OtA5093-OtA5097
Abhd13	chr8:9977716-9992154	OtA5088-OtA5092	OtA5093-OtA5097
Asnsd1	chr1:53344643-53352699	OtA5088-OtA5092	OtA5093-OtA5097
B4galt3	chr1:171270327-171276895	OtA5088-OtA5092	OtA5093-OtA5097
Capns1	chr7:30186941-30195048	OtA5088-OtA5092	OtA5093-OtA5097
Cav2	chr6:17281340-17289011	OtA5088-OtA5092	OtA5093-OtA5097
Cdr2l	chr11:115381915-115396132	OtA5088-OtA5092	OtA5093-OtA5097
Chsy3	chr18:59175339-59411336	OtA5088-OtA5092	OtA5093-OtA5097
Exoc3	chr13:74169804-74208700	OtA5088-OtA5092	OtA5093-OtA5097
Fam108a	chr10:80583648-80590341	OtA5088-OtA5092	OtA5093-OtA5097
Fam136a	chr6:86365682-86370058	OtA5088-OtA5092	OtA5093-OtA5097
Fgfr2	chr7:130162450-130266808	OtA5088-OtA5092	OtA5093-OtA5097
Flrt3	chr2:140395429-142390050	OtA5088-OtA5092	OtA5093-OtA5097
Hdac8	chrX:102284639-102505148	OtA5088-OtA5092	OtA5093-OtA5097

Hrsp12	chr15:34484021-34495246	OtA5088-OtA5092	OtA5093-OtA5097
Hyi	chr4:118359998-118409263	OtA5088-OtA5092	OtA5093-OtA5097
Ifit2	chr19:34550693-34576534	OtA5088-OtA5092	OtA5093-OtA5097
Itpripl1	chr2:127138768-127143457	OtA5088-OtA5092	OtA5093-OtA5097
Kdr	chr5:75933269-75978428	OtA5088-OtA5092	OtA5093-OtA5097
L1td1	chr4:98726753-98738486	OtA5088-OtA5092	OtA5093-OtA5097
Mapk14	chr17:28691341-28748405	OtA5088-OtA5092	OtA5093-OtA5097
Mcl1	chr3:95658720-95663178	OtA5088-OtA5092	OtA5093-OtA5097
Mreg	chr1:72159232-72212307	OtA5088-OtA5092	OtA5093-OtA5097
Mterfd1	chr13:66912097-67032405	OtA5088-OtA5092	OtA5093-OtA5097
Mum1	chr10:79697304-80369637	OtA5088-OtA5092	OtA5093-OtA5097
Nsmce4a	chr7:130532525-130547381	OtA5088-OtA5092	OtA5093-OtA5097
Nup62	chr7:44812255-44849079	OtA5088-OtA5092	OtA5093-OtA5097
Orai1	chr5:123015073-123030452	OtA5088-OtA5092	OtA5093-OtA5097
Otud1	chr2:19658061-19660590	OtA5088-OtA5092	OtA5093-OtA5097
Phkb	chr8:85841001-86060642	OtA5088-OtA5092	OtA5093-OtA5097
Rbm28	chr6:29123572-29164724	OtA5088-OtA5092	OtA5093-OtA5097
Rpap2	chr5:107534710-107661838	OtA5088-OtA5092	OtA5093-OtA5097
Rpia	chr6:70765719-70792175	OtA5088-OtA5092	OtA5093-OtA5097
Sdccag3	chr2:26382799-26389316	OtA5088-OtA5092	OtA5093-OtA5097
Serf1	chr13:100108018-100114233	OtA5088-OtA5092	OtA5093-OtA5097
Slc35d2	chr13:64096309-64129330	OtA5088-OtA5092	OtA5093-OtA5097
Slc38a1	chr15:96571417-96642913	OtA5088-OtA5092	OtA5093-OtA5097
Slc41a3	chr6:90604879-90646412	OtA5088-OtA5092	OtA5093-OtA5097
Tiprl	chr1:165212285-165236958	OtA5088-OtA5092	OtA5093-OtA5097
Ttc5	chr14:50765414-50785518	OtA5088-OtA5092	OtA5093-OtA5097
Uap1l1	chr2:25361491-25365626	OtA5088-OtA5092	OtA5093-OtA5097
Wibg	chr10:128747878-128766568	OtA5088-OtA5092	OtA5093-OtA5097
Zxdb	chrX:94724568-94730191	OtA5088-OtA5092	OtA5093-OtA5097
Atg9a	chr1:75180865-75191923	OtA5088-OtA5092	OtA5093-OtA5097
Lamtor3	chr3:137918554-137928762	OtA5088-OtA5092	OtA5093-OtA5097
Ptprk	chr10:28074819-28597397	OtA5088-OtA5092	OtA5093-OtA5097
Serpine1	chr5:137061505-137072272	OtA5088-OtA5092	OtA5093-OtA5097
Clp1	chr2:84723121-84727268	OtA5088-OtA5092	OtA5093-OtA5097
Isoc1	chr18:58659481-58679570	OtA5088-OtA5092	OtA5093-OtA5097
Ufd1l	chr16:18812293-18835261	OtA5088-OtA5092	OtA5093-OtA5097
Acsf3	chr8:122775504-122817881	OtA5088-OtA5092	OtA5093-OtA5097
Acvr2a	chr2:48814108-48949267	OtA5088-OtA5092	OtA5093-OtA5097
Alkbh2	chr5:114123933-114128176	OtA5088-OtA5092	OtA5093-OtA5097
Anapc4	chr5:52834134-52866734	OtA5088-OtA5092	OtA5093-OtA5097
BC026585	chr1:157458581-157488733	OtA5088-OtA5092	OtA5093-OtA5097
Bbs2	chr8:94067953-94098811	OtA5088-OtA5092	OtA5093-OtA5097
Bnip2	chr9:69989500-70008317	OtA5088-OtA5092	OtA5093-OtA5097
Bzw2	chr12:36091834-36156825	OtA5088-OtA5092	OtA5093-OtA5097
Cdc37	chr9:21138540-21149906	OtA5088-OtA5092	OtA5093-OtA5097
Col4a1	chr8:11198422-11312826	OtA5088-OtA5092	OtA5093-OtA5097
Comp	chr8:70373547-70382066	OtA5088-OtA5092	OtA5093-OtA5097

Cradd	chr10:95174745-95324097	OtA5088-OtA5092	OtA5093-OtA5097
Csnk1g3	chr18:53862112-53955684	OtA5088-OtA5092	OtA5093-OtA5097
Ctps2	chrX:162407199-163352564	OtA5088-OtA5092	OtA5093-OtA5097
Cuta	chr17:26937971-26939478	OtA5088-OtA5092	OtA5093-OtA5097
D6Wsu163e	chr6:126939965-126975704	OtA5088-OtA5092	OtA5093-OtA5097
Dazap1	chr10:79697304-80369637	OtA5088-OtA5092	OtA5093-OtA5097
Dctn5	chr7:122133040-122149044	OtA5088-OtA5092	OtA5093-OtA5097
Dhx34	chr7:16197220-16222032	OtA5088-OtA5092	OtA5093-OtA5097
Dnajb9	chr12:44205896-44210068	OtA5088-OtA5092	OtA5093-OtA5097
Dpcd	chr19:45560614-45660193	OtA5088-OtA5092	OtA5093-OtA5097
Dram1	chr10:88322803-88357075	OtA5088-OtA5092	OtA5093-OtA5097
Drg2	chr11:60454616-60468705	OtA5088-OtA5092	OtA5093-OtA5097
Eif2s3y	chrY:1010611-1028598	OtA5088-OtA5092	OtA5093-OtA5097
Elmo2	chr2:165288030-165326479	OtA5088-OtA5092	OtA5093-OtA5097
Fabp4	chr3:10204342-10208576	OtA5088-OtA5092	OtA5093-OtA5097
Fam116b	chr15:89182212-89196474	OtA5088-OtA5092	OtA5093-OtA5097
Fam190a	chr6:61175603-62382863	OtA5088-OtA5092	OtA5093-OtA5097
Fosb	chr7:19302695-19310045	OtA5088-OtA5092	OtA5093-OtA5097
Galnt7	chr8:57523824-57663430	OtA5088-OtA5092	OtA5093-OtA5097
Gm9776	chr13:94356748-94358923	OtA5088-OtA5092	OtA5093-OtA5097
Gnaz	chr10:74957476-75032586	OtA5088-OtA5092	OtA5093-OtA5097
Hgfac	chr5:35041552-35048436	OtA5088-OtA5092	OtA5093-OtA5097
Homer1	chr13:93304494-93404962	OtA5088-OtA5092	OtA5093-OtA5097
Hpgd	chr8:56294551-56321046	OtA5088-OtA5092	OtA5093-OtA5097
Hspa14	chr2:3488853-3512814	OtA5088-OtA5092	OtA5093-OtA5097
Ids	chrX:70343069-70365085	OtA5088-OtA5092	OtA5093-OtA5097
Ifi27l2b	chr12:103450897-103457223	OtA5088-OtA5092	OtA5093-OtA5097
Il17d	chr14:57524828-57543166	OtA5088-OtA5092	OtA5093-OtA5097
Ints7	chr1:191575733-191621691	OtA5088-OtA5092	OtA5093-OtA5097
Kng2	chr16:22985851-23029101	OtA5088-OtA5092	OtA5093-OtA5097
Ly6k	chr15:74796873-74799968	OtA5088-OtA5092	OtA5093-OtA5097
Lyrm4	chr13:35978796-36117357	OtA5088-OtA5092	OtA5093-OtA5097
Lyrm5	chr6:145211133-145216542	OtA5088-OtA5092	OtA5093-OtA5097
Mast3	chr8:70778116-70792433	OtA5088-OtA5092	OtA5093-OtA5097
Mccc1	chr3:35959305-36053547	OtA5088-OtA5092	OtA5093-OtA5097
Micu1	chr10:59702634-59864122	OtA5088-OtA5092	OtA5093-OtA5097
Mphosph6	chr8:117791644-117801929	OtA5088-OtA5092	OtA5093-OtA5097
Msi2	chr11:88339381-88718267	OtA5088-OtA5092	OtA5093-OtA5097
Mta3	chr17:83706162-83814905	OtA5088-OtA5092	OtA5093-OtA5097
Mtpn	chr6:35508823-35539888	OtA5088-OtA5092	OtA5093-OtA5097
Nacc2	chr2:26055535-26122811	OtA5088-OtA5092	OtA5093-OtA5097
Nfkb1	chr3:135584654-135691547	OtA5088-OtA5092	OtA5093-OtA5097
Nsfl1c	chr2:151494307-151511304	OtA5088-OtA5092	OtA5093-OtA5097
Nsun2	chr13:69612016-69635779	OtA5088-OtA5092	OtA5093-OtA5097
Nsun5	chr5:135369952-135394546	OtA5088-OtA5092	OtA5093-OtA5097
Nuak1	chr10:84371318-84440471	OtA5088-OtA5092	OtA5093-OtA5097
Nudt16	chr9:105129337-105131805	OtA5088-OtA5092	OtA5093-OtA5097

Osbpl9	chr4:109000804-109202272	OtA5088-OtA5092	OtA5093-OtA5097
Pcbd2	chr13:55727367-55776830	OtA5088-OtA5092	OtA5093-OtA5097
Phtf2	chr5:20758663-20882124	OtA5088-OtA5092	OtA5093-OtA5097
Pigyl	chr9:22055459-22389206	OtA5088-OtA5092	OtA5093-OtA5097
Plec	chr15:76170973-76231378	OtA5088-OtA5092	OtA5093-OtA5097
Pmf1	chr3:88394142-88410316	OtA5088-OtA5092	OtA5093-OtA5097
Poldip3	chr15:83125977-83149336	OtA5088-OtA5092	OtA5093-OtA5097
Pop1	chr15:34495310-34530653	OtA5088-OtA5092	OtA5093-OtA5097
Ppt1	chr4:122836226-122859175	OtA5088-OtA5092	OtA5093-OtA5097
Prkar1b	chr5:139017303-139130386	OtA5088-OtA5092	OtA5093-OtA5097
Prl8a9	chr13:27558000-27564604	OtA5088-OtA5092	OtA5093-OtA5097
Prss36	chr7:127932637-127946725	OtA5088-OtA5092	OtA5093-OtA5097
Psg16	chr7:17074039-17098971	OtA5088-OtA5092	OtA5093-OtA5097
Psg21	chr7:18646653-18656725	OtA5088-OtA5092	OtA5093-OtA5097
Ptms	chr6:124913674-124917946	OtA5088-OtA5092	OtA5093-OtA5097
Pxk	chr14:8098212-8165111	OtA5088-OtA5092	OtA5093-OtA5097
Rgs5	chr1:169655500-169693526	OtA5088-OtA5092	OtA5093-OtA5097
Rhoa	chr9:108306204-108337939	OtA5088-OtA5092	OtA5093-OtA5097
Rnpepl1	chr1:92910824-92920585	OtA5088-OtA5092	OtA5093-OtA5097
Rpl26	chr11:68901565-68904534	OtA5088-OtA5092	OtA5093-OtA5097
Rpl6	chr5:121204500-121209241	OtA5088-OtA5092	OtA5093-OtA5097
Sdccag8	chr1:176814811-177020432	OtA5088-OtA5092	OtA5093-OtA5097
Sema6b	chr17:56123084-56140343	OtA5088-OtA5092	OtA5093-OtA5097
Slc35e3	chr10:117733677-117746358	OtA5088-OtA5092	OtA5093-OtA5097
Slc36a4	chr9:15709768-15738789	OtA5088-OtA5092	OtA5093-OtA5097
Sssca1	chr19:5730305-5731721	OtA5088-OtA5092	OtA5093-OtA5097
Syt14	chr1:192897306-193035698	OtA5088-OtA5092	OtA5093-OtA5097
Taf9	chr13:100651342-100666413	OtA5088-OtA5092	OtA5093-OtA5097
Tars	chr15:11383662-11399658	OtA5088-OtA5092	OtA5093-OtA5097
Terf1	chr1:15805657-15843429	OtA5088-OtA5092	OtA5093-OtA5097
Thap2	chr10:115369965-115384435	OtA5088-OtA5092	OtA5093-OtA5097
Tmem209	chr6:30481232-30509706	OtA5088-OtA5092	OtA5093-OtA5097
Tmem42	chr9:123021325-123023491	OtA5088-OtA5092	OtA5093-OtA5097
Tnfrsf12a	chr17:23660522-23677449	OtA5088-OtA5092	OtA5093-OtA5097
Tnk1	chr11:69846371-69858730	OtA5088-OtA5092	OtA5093-OtA5097
Top3a	chr11:60740058-60777365	OtA5088-OtA5092	OtA5093-OtA5097
Trim13	chr14:61598225-61682373	OtA5088-OtA5092	OtA5093-OtA5097
Trmu	chr15:85879326-85897393	OtA5088-OtA5092	OtA5093-OtA5097
Ttc9c	chr19:8809074-8819294	OtA5088-OtA5092	OtA5093-OtA5097
Txndc11	chr16:11066297-11134532	OtA5088-OtA5092	OtA5093-OtA5097
Uba52	chr8:70508265-70510367	OtA5088-OtA5092	OtA5093-OtA5097
Ube2q1	chr3:89773608-89783997	OtA5088-OtA5092	OtA5093-OtA5097
Ubxn8	chr8:33599620-33641976	OtA5088-OtA5092	OtA5093-OtA5097
Vwa1	chr4:155768494-155774561	OtA5088-OtA5092	OtA5093-OtA5097
Wee1	chr7:110122058-110143299	OtA5088-OtA5092	OtA5093-OtA5097
Xrcc6	chr15:82016368-82040084	OtA5088-OtA5092	OtA5093-OtA5097
Zfp185	chrX:72987338-73031543	OtA5088-OtA5092	OtA5093-OtA5097

Zfp192	chr13:21513220-21531114	OtA5088-OtA5092	OtA5093-OtA5097
Zfp511	chr7:140036390-140040605	OtA5088-OtA5092	OtA5093-OtA5097
Zfpl1	chr19:6080037-6084891	OtA5088-OtA5092	OtA5093-OtA5097
Col4a2	chr8:11312828-11449287	OtA5088-OtA5092	OtA5093-OtA5097
Crcp	chr5:130029305-130060783	OtA5088-OtA5092	OtA5093-OtA5097
Mrpl55	chr11:59202516-59206002	OtA5088-OtA5092	OtA5093-OtA5097
Il2rb	chr15:78480548-78495066	OtA5088-OtA5092	OtA5093-OtA5097
Ube2b	chr11:51985145-52000466	OtA5088-OtA5092	OtA5093-OtA5097
Rps3a	chr3:86137939-86142668	OtA5088-OtA5092	OtA5093-OtA5097
BC016495	chr19:18632015-18652184	OtA5088-OtA5092	OtA5093-OtA5097
BC005537	chr13:24801656-24812899	OtA5088-OtA5092	OtA5093-OtA5097
Heatr5b	chr17:78752905-78835381	OtA5088-OtA5092	OtA5093-OtA5097
Plekhg2	chr7:28359603-28372662	OtA5088-OtA5092	OtA5093-OtA5097
Stx11	chr10:12939982-12964259	OtA5088-OtA5092	OtA5093-OtA5097
Adat3	chr10:80602879-80615783	OtA5088-OtA5092	OtA5093-OtA5097
Ppp2r3c	chr12:55280813-55492647	OtA5088-OtA5092	OtA5093-OtA5097
Chmp4b	chr2:154657025-154694783	OtA5088-OtA5092	OtA5093-OtA5097
Tm7sf2	chr19:6062820-6077187	OtA5088-OtA5092	OtA5093-OtA5097
Stk11	chr10:79697304-80369637	OtA5088-OtA5092	OtA5093-OtA5097
Abcb9	chr5:124061856-124095798	OtA5088-OtA5092	OtA5093-OtA5097
Trim11	chr11:58978115-58991458	OtA5088-OtA5092	OtA5093-OtA5097
Abcb10	chr8:123952458-123983122	OtA5088-OtA5092	OtA5093-OtA5097
Btbd9	chr17:30215523-30576287	OtA5088-OtA5092	OtA5093-OtA5097
F11r	chr1:171437560-171464593	OtA5088-OtA5092	OtA5093-OtA5097
Lifr	chr15:7129571-7197489	OtA5088-OtA5092	OtA5093-OtA5097
Mavs	chr2:131127411-131604824	OtA5088-OtA5092	OtA5093-OtA5097
Mcm3ap	chr10:76449080-76515857	OtA5088-OtA5092	OtA5093-OtA5097
Myst2	chr11:95271852-95310246	OtA5088-OtA5092	OtA5093-OtA5097
Phf5a	chr15:81864515-81871892	OtA5088-OtA5092	OtA5093-OtA5097
Plekhh3	chr11:101162679-101171302	OtA5088-OtA5092	OtA5093-OtA5097
Psmd6	chr14:14112184-14120904	OtA5088-OtA5092	OtA5093-OtA5097
Rasl10a	chr11:5054130-5065327	OtA5088-OtA5092	OtA5093-OtA5097
Wdr46	chr17:33940722-33949695	OtA5088-OtA5092	OtA5093-OtA5097
Apex1	chr14:50924948-50930849	OtA5088-OtA5092	OtA5093-OtA5097
Cutc	chr19:43753022-43768638	OtA5088-OtA5092	OtA5093-OtA5097
Pldn	chr2:122738504-122749487	OtA5088-OtA5092	OtA5093-OtA5097
Dcaf8	chr1:172148014-172196393	OtA5088-OtA5092	OtA5093-OtA5097
Hadh	chr3:131233423-131272037	OtA5088-OtA5092	OtA5093-OtA5097
Map3k5	chr10:19934525-20142750	OtA5088-OtA5092	OtA5093-OtA5097
Rps27	chr3:90212666-90213648	OtA5088-OtA5092	OtA5093-OtA5097
Ube2m	chr7:13035119-13038275	OtA5088-OtA5092	OtA5093-OtA5097
Fam89a	chr8:124740256-124751809	OtA5088-OtA5092	OtA5093-OtA5097
Rnf26	chr9:44110780-44113051	OtA5088-OtA5092	OtA5093-OtA5097
Cx3cl1	chr8:94772179-94782426	OtA5088-OtA5092	OtA5093-OtA5097
Tbck	chr3:132684143-132838506	OtA5088-OtA5092	OtA5093-OtA5097
Ppp1r16a	chr15:76671679-76694915	OtA5088-OtA5092	OtA5093-OtA5097
Tor1aip2	chr1:156004598-156068859	OtA5088-OtA5092	OtA5093-OtA5097

Jun	chr4:95049035-95052222	OtA5088-OtA5092	OtA5093-OtA5097
Nipa2	chr7:55842070-55962493	OtA5088-OtA5092	OtA5093-OtA5097
Gpt2	chr8:85492616-85527558	OtA5088-OtA5092	OtA5093-OtA5097
Pik3r3	chr4:116221913-116303056	OtA5088-OtA5092	OtA5093-OtA5097
Lzts2	chr19:45015175-45045772	OtA5088-OtA5092	OtA5093-OtA5097
Mfsd1	chr3:67582767-67604231	OtA5088-OtA5092	OtA5093-OtA5097
Nhsl1	chr10:18407674-18533891	OtA5088-OtA5092	OtA5093-OtA5097
Zfand3	chr17:30005086-30210020	OtA5088-OtA5092	OtA5093-OtA5097
Cirbp	chr10:79697304-80369637	OtA5088-OtA5092	OtA5093-OtA5097
Fam57b	chr7:126816884-126830219	OtA5088-OtA5092	OtA5093-OtA5097
Nploc4	chr11:120379797-120437700	OtA5088-OtA5092	OtA5093-OtA5097
Tgfb2	chr1:186623185-186705992	OtA5088-OtA5092	OtA5093-OtA5097
A3galt2	chr4:128759257-128769298	OtA5088-OtA5092	OtA5093-OtA5097
A4gnt	chr9:99612501-99622367	OtA5088-OtA5092	OtA5093-OtA5097
Aadac	chr3:60031787-60040157	OtA5088-OtA5092	OtA5093-OtA5097
Aadacl2	chr3:60006742-60025420	OtA5088-OtA5092	OtA5093-OtA5097
Aadacl3	chr4:144453770-144463756	OtA5088-OtA5092	OtA5093-OtA5097
Aadat	chr8:60506123-60545677	OtA5088-OtA5092	OtA5093-OtA5097
Aanat	chr11:116593686-116597680	OtA5088-OtA5092	OtA5093-OtA5097
Aass	chr6:23072172-23132986	OtA5088-OtA5092	OtA5093-OtA5097
Abca12	chr1:71243089-71414910	OtA5088-OtA5092	OtA5093-OtA5097
Abca13	chr11:9191941-9684259	OtA5088-OtA5092	OtA5093-OtA5097
Abca14	chr7:120203963-120325352	OtA5088-OtA5092	OtA5093-OtA5097
Abca15	chr7:120328683-120407687	OtA5088-OtA5092	OtA5093-OtA5097
Abca16	chr7:120421700-120544811	OtA5088-OtA5092	OtA5093-OtA5097
Abca17	chr17:24264278-24347252	OtA5088-OtA5092	OtA5093-OtA5097
Abca6	chr11:110176820-110251776	OtA5088-OtA5092	OtA5093-OtA5097
Abca8a	chr11:110025633-110095937	OtA5088-OtA5092	OtA5093-OtA5097
Abca8b	chr11:109933405-109995816	OtA5088-OtA5092	OtA5093-OtA5097
Abca9	chr11:110100821-110168153	OtA5088-OtA5092	OtA5093-OtA5097
Abcb11	chr2:69238281-69342616	OtA5088-OtA5092	OtA5093-OtA5097
Abcb4	chr5:8893720-8959226	OtA5088-OtA5092	OtA5093-OtA5097
Abcb5	chr12:118867823-118966421	OtA5088-OtA5092	OtA5093-OtA5097
Abcb7	chrX:104280564-104413846	OtA5088-OtA5092	OtA5093-OtA5097
Abcc12	chr8:86504840-86566590	OtA5088-OtA5092	OtA5093-OtA5097
Abcc6	chr7:45976379-46030286	OtA5088-OtA5092	OtA5093-OtA5097
Abcc8	chr7:46104522-46180033	OtA5088-OtA5092	OtA5093-OtA5097
Abcd2	chr15:91145870-91191807	OtA5088-OtA5092	OtA5093-OtA5097
Abcg3	chr5:104935056-104982717	OtA5088-OtA5092	OtA5093-OtA5097
Abcg4	chr9:44273189-44288244	OtA5088-OtA5092	OtA5093-OtA5097
Abcg5	chr17:84658233-84682923	OtA5088-OtA5092	OtA5093-OtA5097
Abcg8	chr17:84683130-84700333	OtA5088-OtA5092	OtA5093-OtA5097
Abhd1	chr5:30950065-30960361	OtA5088-OtA5092	OtA5093-OtA5097
Abhd12b	chr12:70154170-70183206	OtA5088-OtA5092	OtA5093-OtA5097
Abhd16b	chr2:181493205-181494980	OtA5088-OtA5092	OtA5093-OtA5097
Abi3bp	chr16:56477845-56690135	OtA5088-OtA5092	OtA5093-OtA5097
Ablim3	chr18:61799392-61911852	OtA5088-OtA5092	OtA5093-OtA5097

Abo	chr2:26842502-26864979	OtA5088-OtA5092	OtA5093-OtA5097
Abpa	chr7:34021566-34022881	OtA5088-OtA5092	OtA5093-OtA5097
Abpb	chr7:34011931-34013942	OtA5088-OtA5092	OtA5093-OtA5097
Abpd	chr7:33364342-33366319	OtA5088-OtA5092	OtA5093-OtA5097
Abpe	chr7:31302768-31304977	OtA5088-OtA5092	OtA5093-OtA5097
Abpg	chr7:33942996-33944985	OtA5088-OtA5092	OtA5093-OtA5097
Abph	chr7:31290518-31291816	OtA5088-OtA5092	OtA5093-OtA5097
Abpz	chr7:33737192-33739295	OtA5088-OtA5092	OtA5093-OtA5097
Abra	chr15:41865292-41869720	OtA5088-OtA5092	OtA5093-OtA5097
Acaa1b	chr9:119148042-119157093	OtA5088-OtA5092	OtA5093-OtA5097
Acan	chr7:79053482-79115099	OtA5088-OtA5092	OtA5093-OtA5097
Acap1	chr11:69881566-69895539	OtA5088-OtA5092	OtA5093-OtA5097
Acat3	chr17:12916464-12940396	OtA5088-OtA5092	OtA5093-OtA5097
Acbd7	chr2:3336167-3340997	OtA5088-OtA5092	OtA5093-OtA5097
Accn1	chr11:80880164-81968396	OtA5088-OtA5092	OtA5093-OtA5097
Accn2	chr15:99670717-99701128	OtA5088-OtA5092	OtA5093-OtA5097
Accn3	chr5:24413450-24417834	OtA5088-OtA5092	OtA5093-OtA5097
Accn4	chr1:75450509-75474340	OtA5088-OtA5092	OtA5093-OtA5097
Accn5	chr3:81996921-82021233	OtA5088-OtA5092	OtA5093-OtA5097
Accsl	chr2:93855362-93869103	OtA5088-OtA5092	OtA5093-OtA5097
Ace3	chr11:105994674-106005443	OtA5088-OtA5092	OtA5093-OtA5097
Acer1	chr17:56953489-56982126	OtA5088-OtA5092	OtA5093-OtA5097
Acmsd	chr1:127729412-127774054	OtA5088-OtA5092	OtA5093-OtA5097
Acnat2	chr4:49379844-49408151	OtA5088-OtA5092	OtA5093-OtA5097
Acot10	chr15:20665213-20666750	OtA5088-OtA5092	OtA5093-OtA5097
Acot12	chr13:91461096-91786148	OtA5088-OtA5092	OtA5093-OtA5097
Acot3	chr12:84052150-84059564	OtA5088-OtA5092	OtA5093-OtA5097
Acot4	chr12:84038378-84044723	OtA5088-OtA5092	OtA5093-OtA5097
Acot5	chr12:84069324-84076019	OtA5088-OtA5092	OtA5093-OtA5097
Acot6	chr12:84100653-84109783	OtA5088-OtA5092	OtA5093-OtA5097
Acoxl	chr2:127854627-128123892	OtA5088-OtA5092	OtA5093-OtA5097
Acpl2	chr9:96823342-96889422	OtA5088-OtA5092	OtA5093-OtA5097
Acpp	chr9:104288239-104337722	OtA5088-OtA5092	OtA5093-OtA5097
Acpt	chr7:44253086-44257204	OtA5088-OtA5092	OtA5093-OtA5097
Acr	chr15:89568325-89574585	OtA5088-OtA5092	OtA5093-OtA5097
Acrv1	chr9:36693219-36698837	OtA5088-OtA5092	OtA5093-OtA5097
Acsbg2	chr17:56843103-56874447	OtA5088-OtA5092	OtA5093-OtA5097
Acsl6	chr11:54303823-54361539	OtA5088-OtA5092	OtA5093-OtA5097
Acsm2	chr7:119554339-119600694	OtA5088-OtA5092	OtA5093-OtA5097
Acsm3	chr7:119760922-119794058	OtA5088-OtA5092	OtA5093-OtA5097
Acsm4	chr7:119690025-119714566	OtA5088-OtA5092	OtA5093-OtA5097
Acsm5	chr7:119526264-119543360	OtA5088-OtA5092	OtA5093-OtA5097
Acss3	chr10:106936163-107123664	OtA5088-OtA5092	OtA5093-OtA5097
Actbl2	chr13:111255012-111257749	OtA5088-OtA5092	OtA5093-OtA5097
Actl6b	chr5:137553554-137569573	OtA5088-OtA5092	OtA5093-OtA5097
Actl7a	chr4:56743421-56744925	OtA5088-OtA5092	OtA5093-OtA5097
Actl7b	chr4:56740004-56741425	OtA5088-OtA5092	OtA5093-OtA5097

Actl9	chr17:33432898-33434267	OtA5088-OtA5092	OtA5093-OtA5097
Actn2	chr13:12269426-12340732	OtA5088-OtA5092	OtA5093-OtA5097
Actn3	chr19:4861222-4877884	OtA5088-OtA5092	OtA5093-OtA5097
Actr3b	chr5:25760025-25850341	OtA5088-OtA5092	OtA5093-OtA5097
Actrt1	chrX:46329006-46330345	OtA5088-OtA5092	OtA5093-OtA5097
Actrt2	chr4:154666427-154667867	OtA5088-OtA5092	OtA5093-OtA5097
Acvr1c	chr2:58267452-58357758	OtA5088-OtA5092	OtA5093-OtA5097
Acyp2	chr11:30505991-30649396	OtA5088-OtA5092	OtA5093-OtA5097
Adad1	chr3:37063655-37111512	OtA5088-OtA5092	OtA5093-OtA5097
Adad2	chr8:119612746-119616926	OtA5088-OtA5092	OtA5093-OtA5097
Adam11	chr11:102761438-102780262	OtA5088-OtA5092	OtA5093-OtA5097
Adam18	chr8:24602245-24674755	OtA5088-OtA5092	OtA5093-OtA5097
Adam1a	chr5:121518603-121521695	OtA5088-OtA5092	OtA5093-OtA5097
Adam1b	chr5:121500096-121502980	OtA5088-OtA5092	OtA5093-OtA5097
Adam2	chr14:66027328-66077733	OtA5088-OtA5092	OtA5093-OtA5097
Adam21	chr12:81558583-81568474	OtA5088-OtA5092	OtA5093-OtA5097
Adam22	chr5:8072351-8368081	OtA5088-OtA5092	OtA5093-OtA5097
Adam24	chr8:40675093-40682199	OtA5088-OtA5092	OtA5093-OtA5097
Adam25	chr8:40752207-40756176	OtA5088-OtA5092	OtA5093-OtA5097
Adam26a	chr8:43568275-43576707	OtA5088-OtA5092	OtA5093-OtA5097
Adam26b	chr8:43519863-43528137	OtA5088-OtA5092	OtA5093-OtA5097
Adam28	chr14:68606026-68655842	OtA5088-OtA5092	OtA5093-OtA5097
Adam29	chr8:55870911-55906964	OtA5088-OtA5092	OtA5093-OtA5097
Adam3	chr8:24677233-24725825	OtA5088-OtA5092	OtA5093-OtA5097
Adam30	chr3:98160811-98163173	OtA5088-OtA5092	OtA5093-OtA5097
Adam32	chr8:24836142-24948804	OtA5088-OtA5092	OtA5093-OtA5097
Adam33	chr2:131050816-131063814	OtA5088-OtA5092	OtA5093-OtA5097
Adam34	chr8:43650308-43665560	OtA5088-OtA5092	OtA5093-OtA5097
Adam39	chr8:40823008-40826861	OtA5088-OtA5092	OtA5093-OtA5097
Adam4	chr12:81419548-81421878	OtA5088-OtA5092	OtA5093-OtA5097
Adam5	chr8:24732984-24824369	OtA5088-OtA5092	OtA5093-OtA5097
Adam6a	chr12:113543907-113546414	OtA5088-OtA5092	OtA5093-OtA5097
Adam6b	chr12:113489564-113491835	OtA5088-OtA5092	OtA5093-OtA5097
Adam7	chr14:68497336-68533689	OtA5088-OtA5092	OtA5093-OtA5097
Adamdec1	chr14:68563386-68582072	OtA5088-OtA5092	OtA5093-OtA5097
Adamts12	chr15:11064789-11346867	OtA5088-OtA5092	OtA5093-OtA5097
Adamts13	chr2:26973415-27009625	OtA5088-OtA5092	OtA5093-OtA5097
Adamts16	chr13:70727807-70841810	OtA5088-OtA5092	OtA5093-OtA5097
Adamts17	chr7:66839734-67152625	OtA5088-OtA5092	OtA5093-OtA5097
Adamts18	chr8:113698136-113848839	OtA5088-OtA5092	OtA5093-OtA5097
Adamts19	chr18:58836763-59053678	OtA5088-OtA5092	OtA5093-OtA5097
Adamts20	chr15:94270162-94404350	OtA5088-OtA5092	OtA5093-OtA5097
Adamts6	chr13:104287872-104494763	OtA5088-OtA5092	OtA5093-OtA5097
Adamts8	chr9:30942562-30962858	OtA5088-OtA5092	OtA5093-OtA5097
Adamtsl2	chr2:27079380-27108613	OtA5088-OtA5092	OtA5093-OtA5097
Adamtsl3	chr7:82335693-82614448	OtA5088-OtA5092	OtA5093-OtA5097
Adarb2	chr13:8202908-8760308	OtA5088-OtA5092	OtA5093-OtA5097

Adat1	chr8:111966907-111992302	OtA5088-OtA5092	OtA5093-OtA5097
Adcy10	chr1:165485182-165576773	OtA5088-OtA5092	OtA5093-OtA5097
Adcy2	chr13:68620042-68999541	OtA5088-OtA5092	OtA5093-OtA5097
Adcy5	chr16:35155635-35304549	OtA5088-OtA5092	OtA5093-OtA5097
Adcy8	chr15:64699034-64922296	OtA5088-OtA5092	OtA5093-OtA5097
Adcyap1	chr17:93199421-93205489	OtA5088-OtA5092	OtA5093-OtA5097
Adh4	chr3:138415496-138430892	OtA5088-OtA5092	OtA5093-OtA5097
Adh6-ps1	chr3:138374120-138388291	OtA5088-OtA5092	OtA5093-OtA5097
Adh6a	chr3:138313285-138331134	OtA5088-OtA5092	OtA5093-OtA5097
Adh7	chr3:138210716-138239663	OtA5088-OtA5092	OtA5093-OtA5097
Adig	chr2:158502611-158508198	OtA5088-OtA5092	OtA5093-OtA5097
Adipoq	chr16:23146535-23157968	OtA5088-OtA5092	OtA5093-OtA5097
Adm2	chr15:89322971-89324730	OtA5088-OtA5092	OtA5093-OtA5097
Adora3	chr3:105870857-105924042	OtA5088-OtA5092	OtA5093-OtA5097
Adprhl1	chr8:13235661-13254162	OtA5088-OtA5092	OtA5093-OtA5097
Adra1a	chr14:66635257-66733329	OtA5088-OtA5092	OtA5093-OtA5097
Adra1b	chr11:43774605-43836332	OtA5088-OtA5092	OtA5093-OtA5097
Adra1d	chr2:131127411-131604824	OtA5088-OtA5092	OtA5093-OtA5097
Adra2a	chr19:54045181-54048982	OtA5088-OtA5092	OtA5093-OtA5097
Adra2c	chr5:35278565-35281763	OtA5088-OtA5092	OtA5093-OtA5097
Adrb3	chr8:27225775-27229588	OtA5088-OtA5092	OtA5093-OtA5097
Adrbk2	chr5:112910477-113015514	OtA5088-OtA5092	OtA5093-OtA5097
Afm	chr5:90518948-90553544	OtA5088-OtA5092	OtA5093-OtA5097
Agap2	chr10:127078906-127093169	OtA5088-OtA5092	OtA5093-OtA5097
Agbl1	chr7:76229886-77124698	OtA5088-OtA5092	OtA5093-OtA5097
Agbl2	chr2:90782743-90816231	OtA5088-OtA5092	OtA5093-OtA5097
Agbl3	chr6:34780431-34857932	OtA5088-OtA5092	OtA5093-OtA5097
Agbl4	chr4:110397790-111657510	OtA5088-OtA5092	OtA5093-OtA5097
Agmat	chr4:141746674-141759263	OtA5088-OtA5092	OtA5093-OtA5097
Agmo	chr12:37241638-37581932	OtA5088-OtA5092	OtA5093-OtA5097
Agphd1	chr9:54917289-54949924	OtA5088-OtA5092	OtA5093-OtA5097
Agr2	chr12:35992924-36004081	OtA5088-OtA5092	OtA5093-OtA5097
Agr3	chr12:35925620-35949730	OtA5088-OtA5092	OtA5093-OtA5097
Agtr1b	chr3:20314472-20367177	OtA5088-OtA5092	OtA5093-OtA5097
Agtr2	chrX:21484625-21488831	OtA5088-OtA5092	OtA5093-OtA5097
Agxt	chr1:93135275-93145421	OtA5088-OtA5092	OtA5093-OtA5097
Agxt2	chr15:10358578-10409738	OtA5088-OtA5092	OtA5093-OtA5097
Agxt2l1	chr3:130617447-130635750	OtA5088-OtA5092	OtA5093-OtA5097
Ahi1	chr10:20952546-21080429	OtA5088-OtA5092	OtA5093-OtA5097
Ahrr	chr13:74211117-74292309	OtA5088-OtA5092	OtA5093-OtA5097
Aicda	chr6:122553808-122564180	OtA5088-OtA5092	OtA5093-OtA5097
Aif1	chr17:35170991-35176001	OtA5088-OtA5092	OtA5093-OtA5097
Aim2	chr1:173420603-173466036	OtA5088-OtA5092	OtA5093-OtA5097
Aipl1	chr11:72028721-72037509	OtA5088-OtA5092	OtA5093-OtA5097
Aire	chr10:78030030-78063615	OtA5088-OtA5092	OtA5093-OtA5097
Ajap1	chr4:153373220-153482830	OtA5088-OtA5092	OtA5093-OtA5097
Ak5	chr3:152396009-152681432	OtA5088-OtA5092	OtA5093-OtA5097

Akap14	chrX:37126762-37168842	OtA5088-OtA5092	OtA5093-OtA5097
Akap17b	chrX:36608182-36645414	OtA5088-OtA5092	OtA5093-OtA5097
Akap3	chr6:126853097-126874308	OtA5088-OtA5092	OtA5093-OtA5097
Akap4	chrX:7067518-7078606	OtA5088-OtA5092	OtA5093-OtA5097
Akap5	chr12:76324890-76334151	OtA5088-OtA5092	OtA5093-OtA5097
Akap6	chr12:52699382-53151015	OtA5088-OtA5092	OtA5093-OtA5097
Aknad1	chr3:108739657-108782309	OtA5088-OtA5092	OtA5093-OtA5097
Akp3	chr1:87125007-87127912	OtA5088-OtA5092	OtA5093-OtA5097
Akr1c12	chr13:4268171-4279399	OtA5088-OtA5092	OtA5093-OtA5097
Akr1c13	chr13:4191186-4205603	OtA5088-OtA5092	OtA5093-OtA5097
Akr1c14	chr13:4059590-4090422	OtA5088-OtA5092	OtA5093-OtA5097
Akr1c19	chr13:4233739-4248359	OtA5088-OtA5092	OtA5093-OtA5097
Akr1c20	chr13:4507158-4523333	OtA5088-OtA5092	OtA5093-OtA5097
Akr1c21	chr13:4574074-4586543	OtA5088-OtA5092	OtA5093-OtA5097
Akr1c6	chr13:4434342-4457530	OtA5088-OtA5092	OtA5093-OtA5097
Akr1cl	chr1:65013085-65032748	OtA5088-OtA5092	OtA5093-OtA5097
Akr1d1	chr6:37530172-37568815	OtA5088-OtA5092	OtA5093-OtA5097
Alcam	chr16:52248995-52452997	OtA5088-OtA5092	OtA5093-OtA5097
Aldh1a1	chr19:20601981-20643462	OtA5088-OtA5092	OtA5093-OtA5097
Aldh1a7	chr19:20692952-20727556	OtA5088-OtA5092	OtA5093-OtA5097
Aldh1l1	chr6:90550847-90599171	OtA5088-OtA5092	OtA5093-OtA5097
Aldh1l2	chr10:83487446-83534140	OtA5088-OtA5092	OtA5093-OtA5097
Aldh3a1	chr11:61208741-61218416	OtA5088-OtA5092	OtA5093-OtA5097
Aldh5a1	chr13:24905599-24937661	OtA5088-OtA5092	OtA5093-OtA5097
Aldh8a1	chr10:21377299-21396578	OtA5088-OtA5092	OtA5093-OtA5097
Aldoart1	chr4:72850582-72852634	OtA5088-OtA5092	OtA5093-OtA5097
Aldoart2	chr12:55565134-55566709	OtA5088-OtA5092	OtA5093-OtA5097
Alg13	chrX:144317965-144374448	OtA5088-OtA5092	OtA5093-OtA5097
Alk	chr17:71868987-72604307	OtA5088-OtA5092	OtA5093-OtA5097
Allc	chr12:28553755-28582483	OtA5088-OtA5092	OtA5093-OtA5097
Alms1-ps2	chr6:85792116-85804057	OtA5088-OtA5092	OtA5093-OtA5097
Alox12	chr11:70241454-70255341	OtA5088-OtA5092	OtA5093-OtA5097
Alox12b	chr11:69157071-69169791	OtA5088-OtA5092	OtA5093-OtA5097
Alox12e	chr11:70315612-70322518	OtA5088-OtA5092	OtA5093-OtA5097
Alox15	chr11:70344146-70352031	OtA5088-OtA5092	OtA5093-OtA5097
Alox5	chr6:116410070-116461178	OtA5088-OtA5092	OtA5093-OtA5097
Alox8	chr11:69183884-69197839	OtA5088-OtA5092	OtA5093-OtA5097
Alpi	chr1:87098001-87101606	OtA5088-OtA5092	OtA5093-OtA5097
Alpk1	chr3:127670309-127780527	OtA5088-OtA5092	OtA5093-OtA5097
Alpk2	chr18:65265530-65393888	OtA5088-OtA5092	OtA5093-OtA5097
Alpk3	chr7:81057599-81105612	OtA5088-OtA5092	OtA5093-OtA5097
Alppl2	chr1:87086690-87089928	OtA5088-OtA5092	OtA5093-OtA5097
Als2cr11	chr1:59050505-59094900	OtA5088-OtA5092	OtA5093-OtA5097
Als2cr12	chr1:58658119-58695989	OtA5088-OtA5092	OtA5093-OtA5097
Alx1	chr10:103007846-103028777	OtA5088-OtA5092	OtA5093-OtA5097
Alx3	chr3:107595030-107605769	OtA5088-OtA5092	OtA5093-OtA5097
Alx4	chr2:93642433-93681339	OtA5088-OtA5092	OtA5093-OtA5097

Amac1	chr11:69759885-69761844	OtA5088-OtA5092	OtA5093-OtA5097
Ambn	chr5:88456010-88468529	OtA5088-OtA5092	OtA5093-OtA5097
Amd1,Amd2	chr10:40287457-40302188	OtA5088-OtA5092	OtA5093-OtA5097
Amdhd1	chr10:93523337-93540033	OtA5088-OtA5092	OtA5093-OtA5097
Amelx	chrX:168795098-169304435	OtA5088-OtA5092	OtA5093-OtA5097
Amh	chr10:80805247-80807648	OtA5088-OtA5092	OtA5093-OtA5097
Amhr2	chr15:102445366-102454639	OtA5088-OtA5092	OtA5093-OtA5097
Amigo1	chr3:108186334-108191777	OtA5088-OtA5092	OtA5093-OtA5097
Ampd1	chr3:103074013-103099720	OtA5088-OtA5092	OtA5093-OtA5097
Amph	chr13:18948370-19150915	OtA5088-OtA5092	OtA5093-OtA5097
Amtn	chr5:88376107-88385916	OtA5088-OtA5092	OtA5093-OtA5097
Amy1	chr3:113555951-113577750	OtA5088-OtA5092	OtA5093-OtA5097
Amy2a4,Amy2a5	chr3:113345048-113356658	OtA5088-OtA5092	OtA5093-OtA5097
Amy2a5	chr3:113345048-113356658	OtA5088-OtA5092	OtA5093-OtA5097
Amy2b	chr3:113147655-113156727	OtA5088-OtA5092	OtA5093-OtA5097
Ang2	chr14:51195485-51195923	OtA5088-OtA5092	OtA5093-OtA5097
Ang3,Ang5	chr14:43957068-43963080	OtA5088-OtA5092	OtA5093-OtA5097
Ang4	chr14:51763891-51773590	OtA5088-OtA5092	OtA5093-OtA5097
Ang5	chr14:43957068-43963080	OtA5088-OtA5092	OtA5093-OtA5097
Ang6	chr14:44001825-44002269	OtA5088-OtA5092	OtA5093-OtA5097
Angpt1	chr15:42424724-42676795	OtA5088-OtA5092	OtA5093-OtA5097
Angptl1	chr1:156804165-156939626	OtA5088-OtA5092	OtA5093-OtA5097
Angptl3	chr4:98936664-99120915	OtA5088-OtA5092	OtA5093-OtA5097
Angptl6	chr9:20868641-20879710	OtA5088-OtA5092	OtA5093-OtA5097
Ank1	chr8:22974881-23150496	OtA5088-OtA5092	OtA5093-OtA5097
Ankar	chr1:72642979-72700564	OtA5088-OtA5092	OtA5093-OtA5097
Ankdd1b	chr13:96416133-96471160	OtA5088-OtA5092	OtA5093-OtA5097
Ankfn1	chr11:89421085-89538555	OtA5088-OtA5092	OtA5093-OtA5097
Ankk1	chr9:49415221-49427021	OtA5088-OtA5092	OtA5093-OtA5097
Ankle1	chr8:71406011-71409904	OtA5088-OtA5092	OtA5093-OtA5097
Ankmy1	chr1:92870128-92902906	OtA5088-OtA5092	OtA5093-OtA5097
Ankrd1	chr19:36111964-36119844	OtA5088-OtA5092	OtA5093-OtA5097
Ankrd13b	chr11:77470486-77489678	OtA5088-OtA5092	OtA5093-OtA5097
Ankrd2	chr19:42036037-42045110	OtA5088-OtA5092	OtA5093-OtA5097
Ankrd22	chr19:34100942-34166041	OtA5088-OtA5092	OtA5093-OtA5097
Ankrd26	chr6:118502563-118562256	OtA5088-OtA5092	OtA5093-OtA5097
Ankrd29	chr18:12252356-12305720	OtA5088-OtA5092	OtA5093-OtA5097
Ankrd32	chr13:77043087-77135468	OtA5088-OtA5092	OtA5093-OtA5097
Ankrd33	chr15:101115754-101120024	OtA5088-OtA5092	OtA5093-OtA5097
Ankrd34a	chr3:96596635-96599778	OtA5088-OtA5092	OtA5093-OtA5097
Ankrd34b	chr13:92425968-92441658	OtA5088-OtA5092	OtA5093-OtA5097
Ankrd34c	chr9:89728248-89738475	OtA5088-OtA5092	OtA5093-OtA5097
Ankrd36	chr11:5569683-5689337	OtA5088-OtA5092	OtA5093-OtA5097
Ankrd45	chr1:161142711-161170507	OtA5088-OtA5092	OtA5093-OtA5097
Ankrd5	chr2:136532320-136555854	OtA5088-OtA5092	OtA5093-OtA5097
Ankrd53	chr6:83762645-83768326	OtA5088-OtA5092	OtA5093-OtA5097
Ankrd55	chr13:112288450-112384002	OtA5088-OtA5092	OtA5093-OtA5097

Ankrd60	chr2:173568660-173578341	OtA5088-OtA5092	OtA5093-OtA5097
Ankrd61	chr5:143871861-143909839	OtA5088-OtA5092	OtA5093-OtA5097
Ankrd7	chr6:18866317-18879584	OtA5088-OtA5092	OtA5093-OtA5097
Anks1b	chr10:89873508-90972984	OtA5088-OtA5092	OtA5093-OtA5097
Anks4b	chr7:120173857-120183716	OtA5088-OtA5092	OtA5093-OtA5097
Anks6	chr4:47015688-47057306	OtA5088-OtA5092	OtA5093-OtA5097
Anln	chr9:22055459-22389206	OtA5088-OtA5092	OtA5093-OtA5097
Ano2	chr6:125690418-126040128	OtA5088-OtA5092	OtA5093-OtA5097
Ano3	chr2:110658139-110950244	OtA5088-OtA5092	OtA5093-OtA5097
Ano4	chr10:88948993-89257790	OtA5088-OtA5092	OtA5093-OtA5097
Ano5	chr7:51511028-51598709	OtA5088-OtA5092	OtA5093-OtA5097
Ano7	chr1:93373895-93403502	OtA5088-OtA5092	OtA5093-OtA5097
Ano9	chr7:141101218-141117806	OtA5088-OtA5092	OtA5093-OtA5097
Antxrl	chr14:34051129-34102754	OtA5088-OtA5092	OtA5093-OtA5097
Anubl1	chr6:116264234-116329626	OtA5088-OtA5092	OtA5093-OtA5097
Anxa10	chr8:62057520-62123118	OtA5088-OtA5092	OtA5093-OtA5097
Anxa13	chr15:58341463-58389274	OtA5088-OtA5092	OtA5093-OtA5097
Aoah	chr13:20794118-21024252	OtA5088-OtA5092	OtA5093-OtA5097
Aox1	chr1:58029968-58106410	OtA5088-OtA5092	OtA5093-OtA5097
Aox3	chr1:58113135-58200452	OtA5088-OtA5092	OtA5093-OtA5097
Aox3l1	chr1:58278325-58379264	OtA5088-OtA5092	OtA5093-OtA5097
Aox4	chr1:58210396-58268596	OtA5088-OtA5092	OtA5093-OtA5097
Ap1s2	chrX:163909159-163929546	OtA5088-OtA5092	OtA5093-OtA5097
Apba2	chr7:64501705-64753876	OtA5088-OtA5092	OtA5093-OtA5097
Apcs	chr1:172893960-172895054	OtA5088-OtA5092	OtA5093-OtA5097
Apitd1	chr4:149128348-149137600	OtA5088-OtA5092	OtA5093-OtA5097
Apoa5	chr9:46268607-46271919	OtA5088-OtA5092	OtA5093-OtA5097
Apobec2	chr17:48419230-48432728	OtA5088-OtA5092	OtA5093-OtA5097
Apobec4	chr1:152750550-152757544	OtA5088-OtA5092	OtA5093-OtA5097
Apoc3	chr9:46233050-46235297	OtA5088-OtA5092	OtA5093-OtA5097
Apod	chr16:31296191-31314596	OtA5088-OtA5092	OtA5093-OtA5097
Apof	chr10:128267996-128270151	OtA5088-OtA5092	OtA5093-OtA5097
Apoh	chr11:108395296-108414396	OtA5088-OtA5092	OtA5093-OtA5097
Apol10a	chr15:77477046-77491069	OtA5088-OtA5092	OtA5093-OtA5097
Apol10b	chr15:77584157-77596125	OtA5088-OtA5092	OtA5093-OtA5097
Apol11a	chr15:77508270-77517319	OtA5088-OtA5092	OtA5093-OtA5097
Apol11b	chr15:77633950-77643286	OtA5088-OtA5092	OtA5093-OtA5097
Apol7a	chr15:77388216-77399110	OtA5088-OtA5092	OtA5093-OtA5097
Apol7b	chr15:77422208-77447460	OtA5088-OtA5092	OtA5093-OtA5097
Apol7c	chr15:77524866-77533315	OtA5088-OtA5092	OtA5093-OtA5097
Apol7d	chr1:71653334-71662843	OtA5088-OtA5092	OtA5093-OtA5097
Apol7e	chr15:77698888-77719288	OtA5088-OtA5092	OtA5093-OtA5097
Apol8	chr15:77748612-77755229	OtA5088-OtA5092	OtA5093-OtA5097
Apon	chr10:128254130-128255901	OtA5088-OtA5092	OtA5093-OtA5097
Aqp12	chr1:93006333-93012269	OtA5088-OtA5092	OtA5093-OtA5097
Aqp2	chr15:99579055-99584545	OtA5088-OtA5092	OtA5093-OtA5097
Aqp4	chr18:15389393-15403684	OtA5088-OtA5092	OtA5093-OtA5097

Aqp6	chr15:99601399-99605477	OtA5088-OtA5092	OtA5093-OtA5097
Aqp9	chr9:71110660-71163289	OtA5088-OtA5092	OtA5093-OtA5097
Ar	chrX:98149749-98317147	OtA5088-OtA5092	OtA5093-OtA5097
Arc	chr15:74669082-74672570	OtA5088-OtA5092	OtA5093-OtA5097
Areg	chr5:91139614-91148432	OtA5088-OtA5092	OtA5093-OtA5097
Arg2	chr12:79130787-79172458	OtA5088-OtA5092	OtA5093-OtA5097
Arhgap11a	chr2:113831491-113848661	OtA5088-OtA5092	OtA5093-OtA5097
Arhgap15	chr2:43748823-44387623	OtA5088-OtA5092	OtA5093-OtA5097
Arhgap19	chr19:41766587-41802084	OtA5088-OtA5092	OtA5093-OtA5097
Arhgap20	chr9:51765351-51875885	OtA5088-OtA5092	OtA5093-OtA5097
Arhgap30	chr1:171388959-171410239	OtA5088-OtA5092	OtA5093-OtA5097
Arhgap33	chr7:30522225-30535009	OtA5088-OtA5092	OtA5093-OtA5097
Arhgap36	chrX:49470449-49500250	OtA5088-OtA5092	OtA5093-OtA5097
Arhgap4	chrX:73892101-73911298	OtA5088-OtA5092	OtA5093-OtA5097
Arhgap40	chr2:158512795-158550628	OtA5088-OtA5092	OtA5093-OtA5097
Arhgdig	chr17:26199182-26201350	OtA5088-OtA5092	OtA5093-OtA5097
Arhgef33	chr17:80307406-80388689	OtA5088-OtA5092	OtA5093-OtA5097
Arhgef38	chr3:133159530-133234889	OtA5088-OtA5092	OtA5093-OtA5097
Arhgef4	chr1:34801721-34812754	OtA5088-OtA5092	OtA5093-OtA5097
Arhgef9	chrX:95048943-95166486	OtA5088-OtA5092	OtA5093-OtA5097
Arid3c	chr4:41723835-41731142	OtA5088-OtA5092	OtA5093-OtA5097
Arl11	chr14:61309752-61311936	OtA5088-OtA5092	OtA5093-OtA5097
Arl13a	chrX:134187500-134208030	OtA5088-OtA5092	OtA5093-OtA5097
Arl14	chr3:69222418-69223618	OtA5088-OtA5092	OtA5093-OtA5097
Arl5c	chr11:97989579-97996173	OtA5088-OtA5092	OtA5093-OtA5097
Arl9	chr5:77004054-77009478	OtA5088-OtA5092	OtA5093-OtA5097
Armc2	chr10:41914989-42018382	OtA5088-OtA5092	OtA5093-OtA5097
Armc3	chr2:19199117-19310234	OtA5088-OtA5092	OtA5093-OtA5097
Armc4	chr18:7088232-7297901	OtA5088-OtA5092	OtA5093-OtA5097
Armcx6	chrX:134748454-134751419	OtA5088-OtA5092	OtA5093-OtA5097
Arntl2	chr6:146805529-146832646	OtA5088-OtA5092	OtA5093-OtA5097
Arpm1	chr3:30597072-30599870	OtA5088-OtA5092	OtA5093-OtA5097
Arpp21	chr9:112065090-112348925	OtA5088-OtA5092	OtA5093-OtA5097
Arr3	chrX:100605496-100618493	OtA5088-OtA5092	OtA5093-OtA5097
Arrdc5	chr17:56294110-56300286	OtA5088-OtA5092	OtA5093-OtA5097
Arsj	chr3:126363851-126440374	OtA5088-OtA5092	OtA5093-OtA5097
Art1	chr7:102101742-102111148	OtA5088-OtA5092	OtA5093-OtA5097
Art2a-ps	chr7:101552452-101560865	OtA5088-OtA5092	OtA5093-OtA5097
Art2b	chr7:101578859-101581161	OtA5088-OtA5092	OtA5093-OtA5097
Art3	chr5:92331840-92414627	OtA5088-OtA5092	OtA5093-OtA5097
Art4	chr6:136848450-136857600	OtA5088-OtA5092	OtA5093-OtA5097
Art5	chr7:102096878-102100229	OtA5088-OtA5092	OtA5093-OtA5097
Artn	chr4:117926161-117929763	OtA5088-OtA5092	OtA5093-OtA5097
Arx	chrX:93286645-93298355	OtA5088-OtA5092	OtA5093-OtA5097
Arxes1	chrX:136033366-136034946	OtA5088-OtA5092	OtA5093-OtA5097
Arxes2	chrX:135993819-135995359	OtA5088-OtA5092	OtA5093-OtA5097
Asb12	chrX:95470198-95478129	OtA5088-OtA5092	OtA5093-OtA5097

Asb14	chr14:26894603-26915257	OtA5088-OtA5092	OtA5093-OtA5097
Asb15	chr6:24528143-24573164	OtA5088-OtA5092	OtA5093-OtA5097
Asb16	chr11:102268822-102278061	OtA5088-OtA5092	OtA5093-OtA5097
Asb17	chr3:153844246-153853615	OtA5088-OtA5092	OtA5093-OtA5097
Asb18	chr1:89952677-90014577	OtA5088-OtA5092	OtA5093-OtA5097
Asb2	chr12:103321141-103356001	OtA5088-OtA5092	OtA5093-OtA5097
Asb4	chr6:5383385-5433021	OtA5088-OtA5092	OtA5093-OtA5097
Asb5	chr8:54550330-54587836	OtA5088-OtA5092	OtA5093-OtA5097
Asb9	chrX:164497902-164539752	OtA5088-OtA5092	OtA5093-OtA5097
Ascl1	chr10:87491040-87493660	OtA5088-OtA5092	OtA5093-OtA5097
Ascl3	chr7:109727464-109731732	OtA5088-OtA5092	OtA5093-OtA5097
Ascl4	chr10:85928490-85929647	OtA5088-OtA5092	OtA5093-OtA5097
Asgr1	chr11:70054368-70057894	OtA5088-OtA5092	OtA5093-OtA5097
Asgr2	chr11:70092643-70106186	OtA5088-OtA5092	OtA5093-OtA5097
Asmt	chrX:170672643-170678054	OtA5088-OtA5092	OtA5093-OtA5097
Aspa	chr11:73304987-73324634	OtA5088-OtA5092	OtA5093-OtA5097
Aspdh	chr7:44465434-44467751	OtA5088-OtA5092	OtA5093-OtA5097
Asphd1	chr7:126946007-126949581	OtA5088-OtA5092	OtA5093-OtA5097
Asphd2	chr5:112385444-112392213	OtA5088-OtA5092	OtA5093-OtA5097
Aspm	chr1:139454772-139494088	OtA5088-OtA5092	OtA5093-OtA5097
Aspn	chr13:49464058-49652731	OtA5088-OtA5092	OtA5093-OtA5097
Astl	chr2:127338638-127357651	OtA5088-OtA5092	OtA5093-OtA5097
Astn1	chr1:158362303-158691786	OtA5088-OtA5092	OtA5093-OtA5097
Astn2	chr4:65380802-66404483	OtA5088-OtA5092	OtA5093-OtA5097
Asxl3	chr18:22345088-22530227	OtA5088-OtA5092	OtA5093-OtA5097
Asz1	chr6:18050963-18109061	OtA5088-OtA5092	OtA5093-OtA5097
Atad2	chr15:58094046-58135082	OtA5088-OtA5092	OtA5093-OtA5097
Atad5	chr11:80089399-80135791	OtA5088-OtA5092	OtA5093-OtA5097
Atcay	chr10:81194690-81230779	OtA5088-OtA5092	OtA5093-OtA5097
Atf7ip2	chr16:10192905-10243051	OtA5088-OtA5092	OtA5093-OtA5097
Atm	chr9:53437121-53536671	OtA5088-OtA5092	OtA5093-OtA5097
Atoh1	chr6:64729145-64731235	OtA5088-OtA5092	OtA5093-OtA5097
Atoh7	chr10:63100155-63100605	OtA5088-OtA5092	OtA5093-OtA5097
Atp10b	chr11:43149876-43262286	OtA5088-OtA5092	OtA5093-OtA5097
Atp10d	chr5:72203328-72298771	OtA5088-OtA5092	OtA5093-OtA5097
Atp11c	chrX:60223289-60403981	OtA5088-OtA5092	OtA5093-OtA5097
Atp12a	chr14:56365067-56388551	OtA5088-OtA5092	OtA5093-OtA5097
Atp13a4	chr16:29396037-29544864	OtA5088-OtA5092	OtA5093-OtA5097
Atp13a5	chr16:29231913-29378732	OtA5088-OtA5092	OtA5093-OtA5097
Atp1a2	chr1:172271708-172298064	OtA5088-OtA5092	OtA5093-OtA5097
Atp1a4	chr1:172223507-172258424	OtA5088-OtA5092	OtA5093-OtA5097
Atp1b2	chr11:69599749-69605960	OtA5088-OtA5092	OtA5093-OtA5097
Atp1b4	chrX:38316266-38336713	OtA5088-OtA5092	OtA5093-OtA5097
Atp2a1	chr7:126428766-126463073	OtA5088-OtA5092	OtA5093-OtA5097
Atp2b2	chr6:113745667-114042080	OtA5088-OtA5092	OtA5093-OtA5097
Atp2b3	chrX:73503085-73571005	OtA5088-OtA5092	OtA5093-OtA5097
Atp4a	chr7:30712231-30725534	OtA5088-OtA5092	OtA5093-OtA5097

Atp4b	chr8:13386208-13396778	OtA5088-OtA5092	OtA5093-OtA5097
Atp6ap1l	chr13:90883448-90905355	OtA5088-OtA5092	OtA5093-OtA5097
Atp6v0c-ps2	chr17:24163864-24169429	OtA5088-OtA5092	OtA5093-OtA5097
Atp6v1b1	chr6:83743016-83758810	OtA5088-OtA5092	OtA5093-OtA5097
Atp6v1c2	chr12:17266594-17324730	OtA5088-OtA5092	OtA5093-OtA5097
Atp6v1e2	chr17:86944108-86947887	OtA5088-OtA5092	OtA5093-OtA5097
Atp6v1g2	chr17:35236595-35238768	OtA5088-OtA5092	OtA5093-OtA5097
Atp6v1g3	chr1:138273737-138289462	OtA5088-OtA5092	OtA5093-OtA5097
Atp8b3	chr10:80519584-80539124	OtA5088-OtA5092	OtA5093-OtA5097
Atp8b4	chr2:126320972-126491553	OtA5088-OtA5092	OtA5093-OtA5097
Atp8b5	chr4:43267158-43373831	OtA5088-OtA5092	OtA5093-OtA5097
Atxn7l2	chr3:108202254-108210934	OtA5088-OtA5092	OtA5093-OtA5097
Aurkb	chr11:69045642-69051662	OtA5088-OtA5092	OtA5093-OtA5097
Aurkc	chr7:6995384-7003091	OtA5088-OtA5092	OtA5093-OtA5097
Avp	chr2:130580676-130582541	OtA5088-OtA5092	OtA5093-OtA5097
Avpr1a	chr10:122448498-122453453	OtA5088-OtA5092	OtA5093-OtA5097
Avpr1b	chr1:131599113-131612000	OtA5088-OtA5092	OtA5093-OtA5097
Avpr2	chrX:73892101-73911298	OtA5088-OtA5092	OtA5093-OtA5097
Awat1	chrX:100572246-100578206	OtA5088-OtA5092	OtA5093-OtA5097
Awat2	chrX:100402221-100412562	OtA5088-OtA5092	OtA5093-OtA5097
Aym1	chr5:113357293-113357821	OtA5088-OtA5092	OtA5093-OtA5097
Azgp1	chr5:137981520-137990232	OtA5088-OtA5092	OtA5093-OtA5097
B020004C17Rik	chr14:57015133-57018982	OtA5088-OtA5092	OtA5093-OtA5097
B020004J07Rik	chr4:101834968-101844022	OtA5088-OtA5092	OtA5093-OtA5097
B020014A21Rik	chr10:3125090-3133193	OtA5088-OtA5092	OtA5093-OtA5097
B020018J22Rik	chr12:112428283-112435338	OtA5088-OtA5092	OtA5093-OtA5097
B020031M17Rik	chr13:119949356-119950806	OtA5088-OtA5092	OtA5093-OtA5097
B130006D01Rik	chr11:95723585-95726773	OtA5088-OtA5092	OtA5093-OtA5097
B130024G19Rik	chr7:70351949-70411146	OtA5088-OtA5092	OtA5093-OtA5097
B130034C11Rik	chr16:87496072-87504038	OtA5088-OtA5092	OtA5093-OtA5097
B230118H07Rik	chr2:101560780-101632528	OtA5088-OtA5092	OtA5093-OtA5097
B230119M05Rik	chrX:134684561-134686462	OtA5088-OtA5092	OtA5093-OtA5097
B230206F22Rik	chrX:103560909-103623754	OtA5088-OtA5092	OtA5093-OtA5097
B230206H07Rik	chr7:141339001-141365110	OtA5088-OtA5092	OtA5093-OtA5097
B230208H11Rik	chr10:12916645-12923127	OtA5088-OtA5092	OtA5093-OtA5097
B230209E15Rik	chr7:61529409-61615327	OtA5088-OtA5092	OtA5093-OtA5097
B230209K01Rik	chr1:121087404-121120975	OtA5088-OtA5092	OtA5093-OtA5097
B230214G05	chr15:88314873-88372651	OtA5088-OtA5092	OtA5093-OtA5097
B230216N24Rik	chr1:97821093-98095632	OtA5088-OtA5092	OtA5093-OtA5097
B230217C12Rik	chr11:97840779-97843043	OtA5088-OtA5092	OtA5093-OtA5097
B230217O12Rik	chr19:57323196-57360899	OtA5088-OtA5092	OtA5093-OtA5097
B230319C09Rik	chr6:83441754-83448322	OtA5088-OtA5092	OtA5093-OtA5097
B230323A14Rik	chr9:69761145-69830199	OtA5088-OtA5092	OtA5093-OtA5097
B230378P21Rik	chr6:32144556-32588192	OtA5088-OtA5092	OtA5093-OtA5097
B330016D10Rik	chr4:141546161-141548313	OtA5088-OtA5092	OtA5093-OtA5097
B3galt1	chr2:68117712-68122690	OtA5088-OtA5092	OtA5093-OtA5097
B3galt2	chr1:143607498-143702684	OtA5088-OtA5092	OtA5093-OtA5097

B3gat1	chr9:26751561-26761338	OtA5088-OtA5092	OtA5093-OtA5097
B3gat2	chr1:23761925-23922289	OtA5088-OtA5092	OtA5093-OtA5097
B3gnt4	chr5:123510459-123524164	OtA5088-OtA5092	OtA5093-OtA5097
B3gnt6	chr7:98192414-98199475	OtA5088-OtA5092	OtA5093-OtA5097
B430010I23Rik	chr8:40990911-41133726	OtA5088-OtA5092	OtA5093-OtA5097
B430212C06Rik	chr18:67321208-67367794	OtA5088-OtA5092	OtA5093-OtA5097
B430306N03Rik	chr17:48316161-48326511	OtA5088-OtA5092	OtA5093-OtA5097
B430319G15Rik	chr9:92538800-92608427	OtA5088-OtA5092	OtA5093-OtA5097
B4galnt4	chr7:141061273-141072119	OtA5088-OtA5092	OtA5093-OtA5097
B4galt2	chr4:117872999-117883476	OtA5088-OtA5092	OtA5093-OtA5097
B630019K06Rik	chrX:8892352-8894964	OtA5088-OtA5092	OtA5093-OtA5097
B830017H08Rik	chr16:17833118-17835019	OtA5088-OtA5092	OtA5093-OtA5097
B930003M22Rik	chr17:10319918-10321141	OtA5088-OtA5092	OtA5093-OtA5097
B930007M17Rik	chr3:139205892-139710299	OtA5088-OtA5092	OtA5093-OtA5097
B930018H19	chr8:34589419-34640316	OtA5088-OtA5092	OtA5093-OtA5097
B930025P03Rik	chr8:10870421-10882454	OtA5088-OtA5092	OtA5093-OtA5097
B930059L03Rik	chr12:110591373-110592679	OtA5088-OtA5092	OtA5093-OtA5097
B930092H01Rik	chr9:61259226-61293809	OtA5088-OtA5092	OtA5093-OtA5097
BB014433	chr8:15041445-15046078	OtA5088-OtA5092	OtA5093-OtA5097
BB019430	chr10:58696744-58704277	OtA5088-OtA5092	OtA5093-OtA5097
BB031773	chr4:102760362-103011965	OtA5088-OtA5092	OtA5093-OtA5097
BB123696	chr13:52729369-52757205	OtA5088-OtA5092	OtA5093-OtA5097
BB283400	chr6:30176622-30178040	OtA5088-OtA5092	OtA5093-OtA5097
BB287469	chr12:87816576-87820676	OtA5088-OtA5092	OtA5093-OtA5097
BB557941	chr2:57127476-57181754	OtA5088-OtA5092	OtA5093-OtA5097
BC002163	chr4:123712709-123718186	OtA5088-OtA5092	OtA5093-OtA5097
BC005561	chr5:104508351-104522383	OtA5088-OtA5092	OtA5093-OtA5097
BC005764	chr10:79697304-80369637	OtA5088-OtA5092	OtA5093-OtA5097
BC006965	chr11:112663926-112711356	OtA5088-OtA5092	OtA5093-OtA5097
BC018242	chr9:21938273-21948907	OtA5088-OtA5092	OtA5093-OtA5097
BC018473	chr11:116752166-116757883	OtA5088-OtA5092	OtA5093-OtA5097
BC021785	chr10:39946911-39986646	OtA5088-OtA5092	OtA5093-OtA5097
BC024139	chr15:76101487-76126556	OtA5088-OtA5092	OtA5093-OtA5097
BC024386	chr7:140906063-140907967	OtA5088-OtA5092	OtA5093-OtA5097
BC024582	chr4:32417434-32586108	OtA5088-OtA5092	OtA5093-OtA5097
BC025920	chr10:81606307-81609836	OtA5088-OtA5092	OtA5093-OtA5097
BC027072	chr17:71743554-71752885	OtA5088-OtA5092	OtA5093-OtA5097
BC030307	chr10:86705810-86776843	OtA5088-OtA5092	OtA5093-OtA5097
BC030476	chr15:34453311-34473892	OtA5088-OtA5092	OtA5093-OtA5097
BC030500	chr8:58911754-58914298	OtA5088-OtA5092	OtA5093-OtA5097
BC030867	chr11:102248881-102265183	OtA5088-OtA5092	OtA5093-OtA5097
BC030870	chr8:65085614-65129537	OtA5088-OtA5092	OtA5093-OtA5097
BC031361	chr16:38085063-38246079	OtA5088-OtA5092	OtA5093-OtA5097
BC035044	chr6:128848043-128896848	OtA5088-OtA5092	OtA5093-OtA5097
BC037032	chr15:4020110-4155344	OtA5088-OtA5092	OtA5093-OtA5097
BC037704	chr19:43675177-43677170	OtA5088-OtA5092	OtA5093-OtA5097
BC039771	chr2:145701194-145753672	OtA5088-OtA5092	OtA5093-OtA5097

BC039966	chr4:153948454-153950476	OtA5088-OtA5092	OtA5093-OtA5097
BC048355	chr17:46496788-46499618	OtA5088-OtA5092	OtA5093-OtA5097
BC048502	chr15:103438959-103448459	OtA5088-OtA5092	OtA5093-OtA5097
BC048507	chr13:67863325-67863923	OtA5088-OtA5092	OtA5093-OtA5097
BC048546	chr6:128539821-128581606	OtA5088-OtA5092	OtA5093-OtA5097
BC048562	chr9:108436481-108446083	OtA5088-OtA5092	OtA5093-OtA5097
BC048602	chr15:35307009-35328535	OtA5088-OtA5092	OtA5093-OtA5097
BC048609	chr19:6080037-6084891	OtA5088-OtA5092	OtA5093-OtA5097
BC048644	chr8:121907832-121944912	OtA5088-OtA5092	OtA5093-OtA5097
BC048671	chr6:90301269-90305448	OtA5088-OtA5092	OtA5093-OtA5097
BC048679	chr7:81494273-81498330	OtA5088-OtA5092	OtA5093-OtA5097
BC048943	chr12:111971558-112068854	OtA5088-OtA5092	OtA5093-OtA5097
BC049352	chr9:45172725-45249985	OtA5088-OtA5092	OtA5093-OtA5097
BC049635	chr4:42868002-42874203	OtA5088-OtA5092	OtA5093-OtA5097
BC049715	chr6:136828842-136840557	OtA5088-OtA5092	OtA5093-OtA5097
BC049730	chr7:24709258-24714535	OtA5088-OtA5092	OtA5093-OtA5097
BC051019	chr7:109703736-109723771	OtA5088-OtA5092	OtA5093-OtA5097
BC051070	chr3:106387383-106406182	OtA5088-OtA5092	OtA5093-OtA5097
BC051142	chr17:34398819-34460734	OtA5088-OtA5092	OtA5093-OtA5097
BC051537	chr17:34083842-34095309	OtA5088-OtA5092	OtA5093-OtA5097
BC051628	chr2:181220015-181222851	OtA5088-OtA5092	OtA5093-OtA5097
BC051665	chr13:60781886-60786364	OtA5088-OtA5092	OtA5093-OtA5097
BC052688	chr13:61771579-61787282	OtA5088-OtA5092	OtA5093-OtA5097
BC053393	chr11:46571512-46589232	OtA5088-OtA5092	OtA5093-OtA5097
BC053749	chr7:30539133-30552272	OtA5088-OtA5092	OtA5093-OtA5097
BC055004	chr5:138225903-138251875	OtA5088-OtA5092	OtA5093-OtA5097
BC055111	chr4:106590908-106617238	OtA5088-OtA5092	OtA5093-OtA5097
BC055402	chr1:57200644-57214996	OtA5088-OtA5092	OtA5093-OtA5097
BC057022	chr5:114568250-114613218	OtA5088-OtA5092	OtA5093-OtA5097
BC060267	chr6:115810728-115838412	OtA5088-OtA5092	OtA5093-OtA5097
BC061194	chr2:18699021-18749605	OtA5088-OtA5092	OtA5093-OtA5097
BC061212	chr5_GL456354_random:157306-16138	OtA5088-OtA5092	OtA5093-OtA5097
BC061237	chr14:44500121-44506345	OtA5088-OtA5092	OtA5093-OtA5097
BC064078	chr6:128993019-129006649	OtA5088-OtA5092	OtA5093-OtA5097
BC065397	chrX:136732947-136803361	OtA5088-OtA5092	OtA5093-OtA5097
BC066135	chr2:180277645-180289879	OtA5088-OtA5092	OtA5093-OtA5097
BC068157	chr8:4209542-4217312	OtA5088-OtA5092	OtA5093-OtA5097
BC080695	chr4:143567466-143573798	OtA5088-OtA5092	OtA5093-OtA5097
BC089491	chr7:28284651-28291134	OtA5088-OtA5092	OtA5093-OtA5097
BC089597	chr10:127866475-127877319	OtA5088-OtA5092	OtA5093-OtA5097
BC090627,Chkb	chr15:89416404-89429927	OtA5088-OtA5092	OtA5093-OtA5097
BC094916	chr1:173521310-173535957	OtA5088-OtA5092	OtA5093-OtA5097
BC100451	chr11:118301060-118355411	OtA5088-OtA5092	OtA5093-OtA5097
BC100530	chr16:36359381-36367570	OtA5088-OtA5092	OtA5093-OtA5097
BC107364	chr3:96433787-96452306	OtA5088-OtA5092	OtA5093-OtA5097
BC117090	chr16:36321664-36334332	OtA5088-OtA5092	OtA5093-OtA5097
BC125332	chr2:112247947-112259291	OtA5088-OtA5092	OtA5093-OtA5097

BC147527	chr13:120300391-120308841	OtA5088-OtA5092	OtA5093-OtA5097
Baalc	chr15:38933908-38951264	OtA5088-OtA5092	OtA5093-OtA5097
Baat	chr4:49489417-49506558	OtA5088-OtA5092	OtA5093-OtA5097
Bai1	chr15:74516195-74589464	OtA5088-OtA5092	OtA5093-OtA5097
Bai2	chr4:129985077-130022633	OtA5088-OtA5092	OtA5093-OtA5097
Bai3	chr1:25067475-25829707	OtA5088-OtA5092	OtA5093-OtA5097
Baiap2l2	chr15:79258194-79285509	OtA5088-OtA5092	OtA5093-OtA5097
Baiap3	chr17:25240169-25256364	OtA5088-OtA5092	OtA5093-OtA5097
Bambi-ps1	chr2:122466582-122467797	OtA5088-OtA5092	OtA5093-OtA5097
Banf2	chr2:144033101-144073979	OtA5088-OtA5092	OtA5093-OtA5097
Bank1	chr3:136053363-136326046	OtA5088-OtA5092	OtA5093-OtA5097
Bard1	chr1:71027534-71102972	OtA5088-OtA5092	OtA5093-OtA5097
Barhl1	chr2:28907679-28916440	OtA5088-OtA5092	OtA5093-OtA5097
Barhl2	chr5:106452522-106458166	OtA5088-OtA5092	OtA5093-OtA5097
Barx1	chr13:48663035-48666507	OtA5088-OtA5092	OtA5093-OtA5097
Barx2	chr9:31846043-31913285	OtA5088-OtA5092	OtA5093-OtA5097
Batf	chr12:85686719-85709087	OtA5088-OtA5092	OtA5093-OtA5097
Batf2	chr19:6164457-6172475	OtA5088-OtA5092	OtA5093-OtA5097
Bbip1	chr19:53892230-54033278	OtA5088-OtA5092	OtA5093-OtA5097
Bbox1	chr2:110265082-110305725	OtA5088-OtA5092	OtA5093-OtA5097
Bbs1	chr19:4886881-4906627	OtA5088-OtA5092	OtA5093-OtA5097
Bbs10	chr10:111298678-111301736	OtA5088-OtA5092	OtA5093-OtA5097
Bbs12	chr3:37312553-37321451	OtA5088-OtA5092	OtA5093-OtA5097
Bbs7	chr3:36573142-36613389	OtA5088-OtA5092	OtA5093-OtA5097
Bc1	chrX:162407199-163352564	OtA5088-OtA5092	OtA5093-OtA5097
Bcan	chr3:87987530-88000356	OtA5088-OtA5092	OtA5093-OtA5097
Bcas1	chr2:170346990-170427845	OtA5088-OtA5092	OtA5093-OtA5097
Bche	chr3:73635808-73708415	OtA5088-OtA5092	OtA5093-OtA5097
Bcl11a	chr11:24078055-24173558	OtA5088-OtA5092	OtA5093-OtA5097
Bcl11b	chr12:107910402-108003414	OtA5088-OtA5092	OtA5093-OtA5097
Bcl2a1a	chr9:88956919-88962416	OtA5088-OtA5092	OtA5093-OtA5097
Bcl2a1b	chr9:89199272-89207838	OtA5088-OtA5092	OtA5093-OtA5097
Bcl2a1c	chr9:114330134-114330578	OtA5088-OtA5092	OtA5093-OtA5097
Bcl2a1d	chr9:88723287-88731850	OtA5088-OtA5092	OtA5093-OtA5097
Bcl2l10	chr9:75347757-75351640	OtA5088-OtA5092	OtA5093-OtA5097
Bcl2l14	chr6:134396328-134438724	OtA5088-OtA5092	OtA5093-OtA5097
Bcl2l15	chr3:103832695-103854618	OtA5088-OtA5092	OtA5093-OtA5097
Bcl7a	chr5:123344447-123374083	OtA5088-OtA5092	OtA5093-OtA5097
Bcmo1	chr8:117095864-117133720	OtA5088-OtA5092	OtA5093-OtA5097
Bco2	chr9:50533086-50555138	OtA5088-OtA5092	OtA5093-OtA5097
Bdh2	chr3:135281220-135304425	OtA5088-OtA5092	OtA5093-OtA5097
Bdkrb1	chr12:105604090-105605428	OtA5088-OtA5092	OtA5093-OtA5097
Bdnf	chr2:109674699-109727043	OtA5088-OtA5092	OtA5093-OtA5097
Bean1	chr8:104170512-104219097	OtA5088-OtA5092	OtA5093-OtA5097
Begain	chr12:109032181-109068217	OtA5088-OtA5092	OtA5093-OtA5097
Best1	chr19:9985171-10001633	OtA5088-OtA5092	OtA5093-OtA5097
Best2	chr8:85007202-85014408	OtA5088-OtA5092	OtA5093-OtA5097

Best3	chr10:116986313-117025040	OtA5088-OtA5092	OtA5093-OtA5097
Bet3l	chr10:34037596-34109815	OtA5088-OtA5092	OtA5093-OtA5097
Bex6	chr16:32179799-32186944	OtA5088-OtA5092	OtA5093-OtA5097
Bfsp1	chr2:143826527-143863173	OtA5088-OtA5092	OtA5093-OtA5097
Bfsp2	chr9:103423577-103480328	OtA5088-OtA5092	OtA5093-OtA5097
Bglap	chr3:88383494-88384464	OtA5088-OtA5092	OtA5093-OtA5097
Bglap2	chr3:88377735-88378699	OtA5088-OtA5092	OtA5093-OtA5097
Bhlha9	chr11:76672469-76673676	OtA5088-OtA5092	OtA5093-OtA5097
Bhlhe22	chr3:18054324-18057514	OtA5088-OtA5092	OtA5093-OtA5097
Bhlhe23	chr2:180774380-180776900	OtA5088-OtA5092	OtA5093-OtA5097
Bhmt	chr13:93616890-93637758	OtA5088-OtA5092	OtA5093-OtA5097
Bhmt2	chr13:93656096-93674302	OtA5088-OtA5092	OtA5093-OtA5097
Bicd1	chr6:149408885-149558857	OtA5088-OtA5092	OtA5093-OtA5097
Blk	chr14:63372836-63417187	OtA5088-OtA5092	OtA5093-OtA5097
Blm	chr7:80454992-80535119	OtA5088-OtA5092	OtA5093-OtA5097
Bmp15	chrX:6314105-6320723	OtA5088-OtA5092	OtA5093-OtA5097
Bmp3	chr5:98854438-98880960	OtA5088-OtA5092	OtA5093-OtA5097
Bmp5	chr9:75775364-75899017	OtA5088-OtA5092	OtA5093-OtA5097
Bmpr1b	chr3:141837135-141982251	OtA5088-OtA5092	OtA5093-OtA5097
Bmx	chrX:164192841-164258193	OtA5088-OtA5092	OtA5093-OtA5097
Bnc1	chr7:81966661-81992299	OtA5088-OtA5092	OtA5093-OtA5097
Bnc2	chr4:84272541-84675086	OtA5088-OtA5092	OtA5093-OtA5097
Bnipl	chr3:95241292-95251193	OtA5088-OtA5092	OtA5093-OtA5097
Boll	chr1:55300068-55363469	OtA5088-OtA5092	OtA5093-OtA5097
Bpi	chr2:158258240-158284531	OtA5088-OtA5092	OtA5093-OtA5097
Bpifa1	chr2:154142879-154149217	OtA5088-OtA5092	OtA5093-OtA5097
Bpifa2	chr2:154008275-154016073	OtA5088-OtA5092	OtA5093-OtA5097
Bpifa3	chr2:154130346-154138355	OtA5088-OtA5092	OtA5093-OtA5097
Bpifa5	chr2:154162606-154168446	OtA5088-OtA5092	OtA5093-OtA5097
Bpifa6	chr2:153974944-154000495	OtA5088-OtA5092	OtA5093-OtA5097
Bpifb1	chr2:154190817-154220343	OtA5088-OtA5092	OtA5093-OtA5097
Bpifb2	chr2:153875044-153895270	OtA5088-OtA5092	OtA5093-OtA5097
Bpifb3	chr2:153918229-153932996	OtA5088-OtA5092	OtA5093-OtA5097
Bpifb4	chr2:153940861-153963852	OtA5088-OtA5092	OtA5093-OtA5097
Bpifb5	chr2:154223741-154240902	OtA5088-OtA5092	OtA5093-OtA5097
Bpifb6	chr2:153900387-153912793	OtA5088-OtA5092	OtA5093-OtA5097
Bpifb9a	chr2:154257878-154271243	OtA5088-OtA5092	OtA5093-OtA5097
Bpifb9b	chr2:154307243-154320642	OtA5088-OtA5092	OtA5093-OtA5097
Bpifc	chr10:85959690-86011860	OtA5088-OtA5092	OtA5093-OtA5097
Brca1	chr11:101488763-101551955	OtA5088-OtA5092	OtA5093-OtA5097
Brca2	chr5:150522622-150569749	OtA5088-OtA5092	OtA5093-OtA5097
Brdt	chr5:107331193-107387058	OtA5088-OtA5092	OtA5093-OtA5097
Brip1	chr11:86058135-86201193	OtA5088-OtA5092	OtA5093-OtA5097
Brs3	chrX:57043073-57048758	OtA5088-OtA5092	OtA5093-OtA5097
Brsk1	chr7:4690927-4725251	OtA5088-OtA5092	OtA5093-OtA5097
Brsk2	chr7:141949750-142004243	OtA5088-OtA5092	OtA5093-OtA5097
Bsn	chr9:108096021-108190383	OtA5088-OtA5092	OtA5093-OtA5097

Bsnd	chr4:106483457-106492243	OtA5088-OtA5092	OtA5093-OtA5097
Bsph1	chr7:13450840-13473449	OtA5088-OtA5092	OtA5093-OtA5097
Bsph2	chr7:13554865-13571067	OtA5088-OtA5092	OtA5093-OtA5097
Bsx	chr9:40874126-40877972	OtA5088-OtA5092	OtA5093-OtA5097
Btbd16	chr7:130774068-130825899	OtA5088-OtA5092	OtA5093-OtA5097
Btbd17	chr11:114790668-114795892	OtA5088-OtA5092	OtA5093-OtA5097
Btbd19	chr4:117119217-117125725	OtA5088-OtA5092	OtA5093-OtA5097
Btbd8	chr5:107437996-107491596	OtA5088-OtA5092	OtA5093-OtA5097
Btc	chr5:91357260-91402994	OtA5088-OtA5092	OtA5093-OtA5097
Btg4	chr9:51116000-51119700	OtA5088-OtA5092	OtA5093-OtA5097
Btk	chrX:134542340-134583140	OtA5088-OtA5092	OtA5093-OtA5097
Btla	chr16:45224336-45252895	OtA5088-OtA5092	OtA5093-OtA5097
Btn1a1	chr13:23456992-23465901	OtA5088-OtA5092	OtA5093-OtA5097
Btn2a2	chr13:23477062-23488857	OtA5088-OtA5092	OtA5093-OtA5097
Btnl1	chr17:34377131-34386028	OtA5088-OtA5092	OtA5093-OtA5097
Btnl10	chr11:58918056-58926965	OtA5088-OtA5092	OtA5093-OtA5097
Btnl2	chr17:34354821-34369492	OtA5088-OtA5092	OtA5093-OtA5097
Btnl4	chr17:34469041-34475937	OtA5088-OtA5092	OtA5093-OtA5097
Btnl5	chr17:34487405-34497429	OtA5088-OtA5092	OtA5093-OtA5097
Btnl6	chr17:34507934-34517352	OtA5088-OtA5092	OtA5093-OtA5097
Btnl9	chr11:49168324-49187089	OtA5088-OtA5092	OtA5093-OtA5097
Bub1	chr2:127800199-127831859	OtA5088-OtA5092	OtA5093-OtA5097
Bves	chr10:45335761-45369708	OtA5088-OtA5092	OtA5093-OtA5097
Bzrap1	chr11:87760540-87785928	OtA5088-OtA5092	OtA5093-OtA5097
C030007H22Rik	chr1:89360343-89406133	OtA5088-OtA5092	OtA5093-OtA5097
C030016D13Rik	chr19:27430036-27432631	OtA5088-OtA5092	OtA5093-OtA5097
C030018K13Rik	chr5:64477007-64483966	OtA5088-OtA5092	OtA5093-OtA5097
C030019I05Rik	chr11:46193848-46312859	OtA5088-OtA5092	OtA5093-OtA5097
C030023E24Rik	chrX:61191292-61194164	OtA5088-OtA5092	OtA5093-OtA5097
C030030A07Rik	chr6:136828842-136840557	OtA5088-OtA5092	OtA5093-OtA5097
C030034I22Rik	chr17:69416446-69419192	OtA5088-OtA5092	OtA5093-OtA5097
C030034L19Rik	chr3:9403077-9413903	OtA5088-OtA5092	OtA5093-OtA5097
C030037D09Rik	chr11:88718642-88728572	OtA5088-OtA5092	OtA5093-OtA5097
C030039L03Rik	chr7:27674660-27706482	OtA5088-OtA5092	OtA5093-OtA5097
C130021I20Rik	chr2:33641192-33645663	OtA5088-OtA5092	OtA5093-OtA5097
C130026I21Rik	chr1:85246343-85270566	OtA5088-OtA5092	OtA5093-OtA5097
C130026L21Rik	chr5:111581560-111587153	OtA5088-OtA5092	OtA5093-OtA5097
C130030K03Rik	chr10:49093680-49095843	OtA5088-OtA5092	OtA5093-OtA5097
C130036L24Rik	chr1:86359573-86367964	OtA5088-OtA5092	OtA5093-OtA5097
C130050O18Rik	chr5:139359738-139460534	OtA5088-OtA5092	OtA5093-OtA5097
C130060C02Rik	chr19:15985074-16010912	OtA5088-OtA5092	OtA5093-OtA5097
C130060K24Rik	chr6:65381293-65458150	OtA5088-OtA5092	OtA5093-OtA5097
C130071C03Rik	chr13:83728105-83735669	OtA5088-OtA5092	OtA5093-OtA5097
C130079G13Rik	chr3:59925213-59937949	OtA5088-OtA5092	OtA5093-OtA5097
C130080G10Rik	chr2:114054395-114061502	OtA5088-OtA5092	OtA5093-OtA5097
C130083M11Rik	chr5:52199983-52216433	OtA5088-OtA5092	OtA5093-OtA5097
C1ql1	chr11:102939263-102946461	OtA5088-OtA5092	OtA5093-OtA5097

C1ql2	chr1:120340581-120343174	OtA5088-OtA5092	OtA5093-OtA5097
C1ql3	chr2:12924040-13010864	OtA5088-OtA5092	OtA5093-OtA5097
C1ql4	chr15:99084753-99087728	OtA5088-OtA5092	OtA5093-OtA5097
C1qtnf4	chr2:90885785-90890526	OtA5088-OtA5092	OtA5093-OtA5097
C1qtnf7	chr5:43515568-43616586	OtA5088-OtA5092	OtA5093-OtA5097
C1qtnf9	chr14:60768133-60780869	OtA5088-OtA5092	OtA5093-OtA5097
C1rb	chr6:124570429-124581044	OtA5088-OtA5092	OtA5093-OtA5097
C230004F18Rik	chrX:61116377-61139460	OtA5088-OtA5092	OtA5093-OtA5097
C230024C17	chr1:153721239-153737829	OtA5088-OtA5092	OtA5093-OtA5097
C230035I16Rik	chr13:23427975-23431017	OtA5088-OtA5092	OtA5093-OtA5097
C230079O03	chr7:136332215-136349415	OtA5088-OtA5092	OtA5093-OtA5097
C230091D08Rik	chr7:59307923-59324149	OtA5088-OtA5092	OtA5093-OtA5097
C2cd4a	chr9:67830531-67832330	OtA5088-OtA5092	OtA5093-OtA5097
C2cd4b	chr9:67759436-67760933	OtA5088-OtA5092	OtA5093-OtA5097
C2cd4c	chr10:79606853-79614025	OtA5088-OtA5092	OtA5093-OtA5097
C2cd4d	chr3:94362443-94364567	OtA5088-OtA5092	OtA5093-OtA5097
C330008G21Rik	chr2:161527622-162661147	OtA5088-OtA5092	OtA5093-OtA5097
C330011F03	chr17:50733126-51613053	OtA5088-OtA5092	OtA5093-OtA5097
C330013F16Rik	chrX:139240225-139357738	OtA5088-OtA5092	OtA5093-OtA5097
C330021F23Rik	chr8:3567997-3584776	OtA5088-OtA5092	OtA5093-OtA5097
C330022C24Rik	chr7:140835495-140846769	OtA5088-OtA5092	OtA5093-OtA5097
C330024C12Rik	chr17:87192215-87194942	OtA5088-OtA5092	OtA5093-OtA5097
C330024D21Rik	chr5:67463897-67470831	OtA5088-OtA5092	OtA5093-OtA5097
C330027C09Rik	chr16:48994187-49019705	OtA5088-OtA5092	OtA5093-OtA5097
C330046G13Rik	chr10:84547416-84553338	OtA5088-OtA5092	OtA5093-OtA5097
C430002E04Rik	chr3:41487680-41493199	OtA5088-OtA5092	OtA5093-OtA5097
C4a	chr17:34809091-34823454	OtA5088-OtA5092	OtA5093-OtA5097
C4bp	chr1:130635920-130661618	OtA5088-OtA5092	OtA5093-OtA5097
C4bp-ps1	chr1:130670201-130681571	OtA5088-OtA5092	OtA5093-OtA5097
C530005A16Rik	chr4:116557726-116600266	OtA5088-OtA5092	OtA5093-OtA5097
C530008M17Rik	chr5:76840598-76873554	OtA5088-OtA5092	OtA5093-OtA5097
C530044C16Rik	chr6:50776114-50814894	OtA5088-OtA5092	OtA5093-OtA5097
C6	chr15:4727209-4804045	OtA5088-OtA5092	OtA5093-OtA5097
C630020P19Rik	chr2:143535475-143816282	OtA5088-OtA5092	OtA5093-OtA5097
C630028M04Rik	chr4:51968093-52051110	OtA5088-OtA5092	OtA5093-OtA5097
C630031E19	chr12:29528383-29923209	OtA5088-OtA5092	OtA5093-OtA5097
C630043F03Rik	chr4:72201243-72203930	OtA5088-OtA5092	OtA5093-OtA5097
C7	chr15:4988761-5063773	OtA5088-OtA5092	OtA5093-OtA5097
C730002L08Rik	chr19:20492714-20556410	OtA5088-OtA5092	OtA5093-OtA5097
C730027H18Rik	chr10:71168736-71182040	OtA5088-OtA5092	OtA5093-OtA5097
C730036E19Rik	chr1:151138033-151144095	OtA5088-OtA5092	OtA5093-OtA5097
C77370	chrX:104077434-104201117	OtA5088-OtA5092	OtA5093-OtA5097
C86187	chr7:46967746-46975911	OtA5088-OtA5092	OtA5093-OtA5097
C86695	chr8:21958713-21964303	OtA5088-OtA5092	OtA5093-OtA5097
C87198	chr12:56591058-56594554	OtA5088-OtA5092	OtA5093-OtA5097
C87414	chr5:93635184-93671463	OtA5088-OtA5092	OtA5093-OtA5097
C87499	chr4:88627319-88634186	OtA5088-OtA5092	OtA5093-OtA5097

C87977	chr4:144206761-144213017	OtA5088-OtA5092	OtA5093-OtA5097
C8a	chr4:104815679-104876398	OtA5088-OtA5092	OtA5093-OtA5097
C8b	chr4:104766316-104804548	OtA5088-OtA5092	OtA5093-OtA5097
C9	chr15:6445332-6498476	OtA5088-OtA5092	OtA5093-OtA5097
C920006O11Rik	chr9:78175913-78178882	OtA5088-OtA5092	OtA5093-OtA5097
C920009B18Rik	chr10:22158608-22374139	OtA5088-OtA5092	OtA5093-OtA5097
C920021L13Rik	chr3:95871521-95891930	OtA5088-OtA5092	OtA5093-OtA5097
CK137956	chr4:127927591-127970841	OtA5088-OtA5092	OtA5093-OtA5097
Cabp1	chr5:115168690-115186121	OtA5088-OtA5092	OtA5093-OtA5097
Cabp2	chr19:4082487-4087339	OtA5088-OtA5092	OtA5093-OtA5097
Cabp4	chr19:4135422-4139609	OtA5088-OtA5092	OtA5093-OtA5097
Cabp5	chr7:13398154-13408878	OtA5088-OtA5092	OtA5093-OtA5097
Cabp7	chr11:4738820-4746778	OtA5088-OtA5092	OtA5093-OtA5097
Cabyr	chr18:12741354-12755142	OtA5088-OtA5092	OtA5093-OtA5097
Cacna1a	chr8:84415363-84640249	OtA5088-OtA5092	OtA5093-OtA5097
Cacna1b	chr2:24606374-24763152	OtA5088-OtA5092	OtA5093-OtA5097
Cacna1c	chr6:118592295-119221614	OtA5088-OtA5092	OtA5093-OtA5097
Cacna1d	chr14:30008999-30527056	OtA5088-OtA5092	OtA5093-OtA5097
Cacna1e	chr1:154392518-154725920	OtA5088-OtA5092	OtA5093-OtA5097
Cacna1f	chrX:7607102-7635196	OtA5088-OtA5092	OtA5093-OtA5097
Cacna1h	chr17:25370552-25433783	OtA5088-OtA5092	OtA5093-OtA5097
Cacna1i	chr15:80287237-80398292	OtA5088-OtA5092	OtA5093-OtA5097
Cacna1s	chr1:136052900-136119822	OtA5088-OtA5092	OtA5093-OtA5097
Cacna2d1	chr5:15934690-16374511	OtA5088-OtA5092	OtA5093-OtA5097
Cacna2d2	chr9:107399879-107529343	OtA5088-OtA5092	OtA5093-OtA5097
Cacna2d3	chr14:28904942-29721864	OtA5088-OtA5092	OtA5093-OtA5097
Cacna2d4	chr6:119236525-119352407	OtA5088-OtA5092	OtA5093-OtA5097
Cacnb1	chr11:98002900-98022627	OtA5088-OtA5092	OtA5093-OtA5097
Cacnb2	chr2:14604305-14987908	OtA5088-OtA5092	OtA5093-OtA5097
Cacnb4	chr2:52428319-52676582	OtA5088-OtA5092	OtA5093-OtA5097
Cacng1	chr11:107703217-107716476	OtA5088-OtA5092	OtA5093-OtA5097
Cacng2	chr15:77993622-78119280	OtA5088-OtA5092	OtA5093-OtA5097
Cacng3	chr7:122671743-122769394	OtA5088-OtA5092	OtA5093-OtA5097
Cacng4	chr11:107734779-107794464	OtA5088-OtA5092	OtA5093-OtA5097
Cacng5	chr11:107874604-107915055	OtA5088-OtA5092	OtA5093-OtA5097
Cacng6	chr7:3424661-3434940	OtA5088-OtA5092	OtA5093-OtA5097
Cacng7	chr7:3336584-3366948	OtA5088-OtA5092	OtA5093-OtA5097
Cacng8	chr7:3394116-3415605	OtA5088-OtA5092	OtA5093-OtA5097
Cadm2	chr16:66655420-67620908	OtA5088-OtA5092	OtA5093-OtA5097
Cadm3	chr1:173334253-173367695	OtA5088-OtA5092	OtA5093-OtA5097
Cadps	chr14:12372562-12823079	OtA5088-OtA5092	OtA5093-OtA5097
Cadps2	chr6:23262773-23839421	OtA5088-OtA5092	OtA5093-OtA5097
Cage1	chr13:38006051-38036937	OtA5088-OtA5092	OtA5093-OtA5097
Calb1	chr4:15881263-15906709	OtA5088-OtA5092	OtA5093-OtA5097
Calb2	chr8:110142537-110168206	OtA5088-OtA5092	OtA5093-OtA5097
Calca	chr7:114631477-114636357	OtA5088-OtA5092	OtA5093-OtA5097
Calcb	chr7:114718642-114723365	OtA5088-OtA5092	OtA5093-OtA5097

Calcoco2	chr11:96099325-96111962	OtA5088-OtA5092	OtA5093-OtA5097
Calcr	chr6:3685719-3764713	OtA5088-OtA5092	OtA5093-OtA5097
Calhm1	chr19:47141034-47144174	OtA5088-OtA5092	OtA5093-OtA5097
Calm5	chr13:3854172-3854761	OtA5088-OtA5092	OtA5093-OtA5097
Caln1	chr5:130369457-130840645	OtA5088-OtA5092	OtA5093-OtA5097
Calr3	chr8:72424182-72443778	OtA5088-OtA5092	OtA5093-OtA5097
Calr4	chr4:109235631-109254248	OtA5088-OtA5092	OtA5093-OtA5097
Caly	chr7:140069879-140082548	OtA5088-OtA5092	OtA5093-OtA5097
Camk1d	chr2:5293456-5714664	OtA5088-OtA5092	OtA5093-OtA5097
Camk1g	chr1:193346345-193370282	OtA5088-OtA5092	OtA5093-OtA5097
Camk2a	chr18:60925631-60988152	OtA5088-OtA5092	OtA5093-OtA5097
Camk2b	chr11:5969665-6065748	OtA5088-OtA5092	OtA5093-OtA5097
Camk4	chr18:32939040-33195767	OtA5088-OtA5092	OtA5093-OtA5097
Camkk1	chr11:73019007-73042065	OtA5088-OtA5092	OtA5093-OtA5097
Camkv	chr9:107935919-107949691	OtA5088-OtA5092	OtA5093-OtA5097
Camp	chr9:109847376-109849456	OtA5088-OtA5092	OtA5093-OtA5097
Cap2	chr13:46501902-46650281	OtA5088-OtA5092	OtA5093-OtA5097
Capn13	chr17:73306463-73399296	OtA5088-OtA5092	OtA5093-OtA5097
Capn3	chr2:120355308-120504919	OtA5088-OtA5092	OtA5093-OtA5097
Capn9	chr8:124576110-124618731	OtA5088-OtA5092	OtA5093-OtA5097
Capns2	chr8:92855349-92919279	OtA5088-OtA5092	OtA5093-OtA5097
Caprin2	chr6:148842511-148896126	OtA5088-OtA5092	OtA5093-OtA5097
Caps2	chr10:112165675-112216555	OtA5088-OtA5092	OtA5093-OtA5097
Capsl	chr15:9436027-9466035	OtA5088-OtA5092	OtA5093-OtA5097
Capza3	chr6:140041524-140042786	OtA5088-OtA5092	OtA5093-OtA5097
Car1	chr3:14766213-14808365	OtA5088-OtA5092	OtA5093-OtA5097
Car10	chr11:93099289-93601751	OtA5088-OtA5092	OtA5093-OtA5097
Car11	chr7:45699966-45704661	OtA5088-OtA5092	OtA5093-OtA5097
Car12	chr9:66713685-66766845	OtA5088-OtA5092	OtA5093-OtA5097
Car13	chr3:14641726-14663002	OtA5088-OtA5092	OtA5093-OtA5097
Car14	chr3:95893995-95904639	OtA5088-OtA5092	OtA5093-OtA5097
Car15	chr16:17835275-17838186	OtA5088-OtA5092	OtA5093-OtA5097
Car3	chr3:14863537-14872373	OtA5088-OtA5092	OtA5093-OtA5097
Car6	chr4:150187015-150201135	OtA5088-OtA5092	OtA5093-OtA5097
Car7	chr8:104446718-104624396	OtA5088-OtA5092	OtA5093-OtA5097
Car8	chr4:8141492-8239041	OtA5088-OtA5092	OtA5093-OtA5097
Car9	chr4:43507025-43513725	OtA5088-OtA5092	OtA5093-OtA5097
Card11	chr5:140872998-141000596	OtA5088-OtA5092	OtA5093-OtA5097
Card6	chr15:5097438-5108533	OtA5088-OtA5092	OtA5093-OtA5097
Card9	chr2:26352311-26359547	OtA5088-OtA5092	OtA5093-OtA5097
Carns1	chr19:4164323-4175479	OtA5088-OtA5092	OtA5093-OtA5097
Cartpt	chr13:99898483-99900683	OtA5088-OtA5092	OtA5093-OtA5097
Casc1	chr6:145121738-145210970	OtA5088-OtA5092	OtA5093-OtA5097
Casc5	chr2:119047118-119104121	OtA5088-OtA5092	OtA5093-OtA5097
Casp1	chr9:5298516-5307281	OtA5088-OtA5092	OtA5093-OtA5097
Casp12	chr9:5345475-5373034	OtA5088-OtA5092	OtA5093-OtA5097
Casp14	chr10:78711994-78718293	OtA5088-OtA5092	OtA5093-OtA5097

Casp4	chr9:5308859-5336791	OtA5088-OtA5092	OtA5093-OtA5097
Casq1	chr1:172209893-172219895	OtA5088-OtA5092	OtA5093-OtA5097
Casq2	chr3:102086509-102146512	OtA5088-OtA5092	OtA5093-OtA5097
Casr	chr16:36493695-36562134	OtA5088-OtA5092	OtA5093-OtA5097
Cass4	chr2:172393793-172432860	OtA5088-OtA5092	OtA5093-OtA5097
Catsper1	chr19:5335740-5344153	OtA5088-OtA5092	OtA5093-OtA5097
Catsper2	chr2:121394354-121413792	OtA5088-OtA5092	OtA5093-OtA5097
Catsper3	chr13:55784578-55809000	OtA5088-OtA5092	OtA5093-OtA5097
Catsper4	chr4:134211967-134227383	OtA5088-OtA5092	OtA5093-OtA5097
Catsperb	chr12:101404672-101626009	OtA5088-OtA5092	OtA5093-OtA5097
Catsperg1	chr7:29181531-29214033	OtA5088-OtA5092	OtA5093-OtA5097
Catsperg2	chr7:29697218-29727015	OtA5088-OtA5092	OtA5093-OtA5097
Cav3	chr6:112459504-112472872	OtA5088-OtA5092	OtA5093-OtA5097
Cbln1	chr8:87468852-87472592	OtA5088-OtA5092	OtA5093-OtA5097
Cbln2	chr18:86713047-86718283	OtA5088-OtA5092	OtA5093-OtA5097
Cbln3	chr14:55878919-55884256	OtA5088-OtA5092	OtA5093-OtA5097
Cbln4	chr2:172036335-172043466	OtA5088-OtA5092	OtA5093-OtA5097
Cbr2	chr11:120729484-120732021	OtA5088-OtA5092	OtA5093-OtA5097
Cbs	chr17:31612643-31637141	OtA5088-OtA5092	OtA5093-OtA5097
Cc2d2a	chr5:43662378-43740970	OtA5088-OtA5092	OtA5093-OtA5097
Ccbl2	chr3:142701079-142744910	OtA5088-OtA5092	OtA5093-OtA5097
Ccdc103	chr11:102881243-102886731	OtA5088-OtA5092	OtA5093-OtA5097
Ccdc105	chr10:78746923-78753067	OtA5088-OtA5092	OtA5093-OtA5097
Ccdc106	chr7:5056725-5060784	OtA5088-OtA5092	OtA5093-OtA5097
Ccdc108	chr1:74902079-74935599	OtA5088-OtA5092	OtA5093-OtA5097
Ccdc11	chr18:74283099-74359984	OtA5088-OtA5092	OtA5093-OtA5097
Ccdc110	chr8:45934648-45944145	OtA5088-OtA5092	OtA5093-OtA5097
Ccdc111	chr8:46552068-46617200	OtA5088-OtA5092	OtA5093-OtA5097
Ccdc112	chr18:46282150-46311928	OtA5088-OtA5092	OtA5093-OtA5097
Ccdc113	chr8:95534099-95558888	OtA5088-OtA5092	OtA5093-OtA5097
Ccdc114	chr7:45928397-45948956	OtA5088-OtA5092	OtA5093-OtA5097
Ccdc116	chr16:17139063-17144423	OtA5088-OtA5092	OtA5093-OtA5097
Ccdc121	chr1:181509632-181511451	OtA5088-OtA5092	OtA5093-OtA5097
Ccdc122	chr14:77036771-77112204	OtA5088-OtA5092	OtA5093-OtA5097
Ccdc125	chr13:100669480-100730942	OtA5088-OtA5092	OtA5093-OtA5097
Ccdc129	chr6:55837017-55978598	OtA5088-OtA5092	OtA5093-OtA5097
Ccdc13	chr9:121797626-121839461	OtA5088-OtA5092	OtA5093-OtA5097
Ccdc135	chr8:95055102-95078141	OtA5088-OtA5092	OtA5093-OtA5097
Ccdc136	chr6:29398925-29426995	OtA5088-OtA5092	OtA5093-OtA5097
Ccdc138	chr10:58497936-58576244	OtA5088-OtA5092	OtA5093-OtA5097
Ccdc14	chr16:34690615-34725194	OtA5088-OtA5092	OtA5093-OtA5097
Ccdc141	chr2:77009905-77170635	OtA5088-OtA5092	OtA5093-OtA5097
Ccdc142	chr6:83101515-83109932	OtA5088-OtA5092	OtA5093-OtA5097
Ccdc144b	chr3:35959305-36053547	OtA5088-OtA5092	OtA5093-OtA5097
Ccdc146	chr5:21292960-21424677	OtA5088-OtA5092	OtA5093-OtA5097
Ccdc147	chr19:47937711-48035379	OtA5088-OtA5092	OtA5093-OtA5097
Ccdc148	chr2:58821697-59018606	OtA5088-OtA5092	OtA5093-OtA5097

Ccdc149	chr5:52374650-52471543	OtA5088-OtA5092	OtA5093-OtA5097
Ccdc15	chr9:37275834-37348392	OtA5088-OtA5092	OtA5093-OtA5097
Ccdc150	chr1:54250682-54368727	OtA5088-OtA5092	OtA5093-OtA5097
Ccdc151	chr9:21989870-22002634	OtA5088-OtA5092	OtA5093-OtA5097
Ccdc152	chr15:3280626-3303526	OtA5088-OtA5092	OtA5093-OtA5097
Ccdc153	chr9:44240676-44247306	OtA5088-OtA5092	OtA5093-OtA5097
Ccdc154	chr17:25162460-25171913	OtA5088-OtA5092	OtA5093-OtA5097
Ccdc155	chr7:45183675-45204892	OtA5088-OtA5092	OtA5093-OtA5097
Ccdc158	chr5:92608294-92675127	OtA5088-OtA5092	OtA5093-OtA5097
Ccdc159	chr9:21921008-21935872	OtA5088-OtA5092	OtA5093-OtA5097
Ccdc160	chrX:52791199-52799468	OtA5088-OtA5092	OtA5093-OtA5097
Ccdc162	chr10:41538848-41587753	OtA5088-OtA5092	OtA5093-OtA5097
Ccdc164	chr5:30341662-30366708	OtA5088-OtA5092	OtA5093-OtA5097
Ccdc17	chr4:116557726-116600266	OtA5088-OtA5092	OtA5093-OtA5097
Ccdc18	chr5:108132913-108232949	OtA5088-OtA5092	OtA5093-OtA5097
Ccdc19	chr1:172521129-172550806	OtA5088-OtA5092	OtA5093-OtA5097
Ccdc24	chr4:117835257-117872470	OtA5088-OtA5092	OtA5093-OtA5097
Ccdc27	chr4:154026643-154042677	OtA5088-OtA5092	OtA5093-OtA5097
Ccdc30	chr4:119323838-119415521	OtA5088-OtA5092	OtA5093-OtA5097
Ccdc33	chr9:58028681-58118823	OtA5088-OtA5092	OtA5093-OtA5097
Ccdc34	chr2:110017816-110050479	OtA5088-OtA5092	OtA5093-OtA5097
Ccdc36	chr9:108403632-108428482	OtA5088-OtA5092	OtA5093-OtA5097
Ccdc37	chr6:90403735-90428480	OtA5088-OtA5092	OtA5093-OtA5097
Ccdc38	chr10:93540631-93605245	OtA5088-OtA5092	OtA5093-OtA5097
Ccdc39	chr3:33800194-33844310	OtA5088-OtA5092	OtA5093-OtA5097
Ccdc40	chr11:119228571-119265212	OtA5088-OtA5092	OtA5093-OtA5097
Ccdc42b	chr5:120613129-120634235	OtA5088-OtA5092	OtA5093-OtA5097
Ccdc46	chr11:108425265-108860615	OtA5088-OtA5092	OtA5093-OtA5097
Ccdc48	chr6:87730868-87755911	OtA5088-OtA5092	OtA5093-OtA5097
Ccdc54	chr16:50589858-50654166	OtA5088-OtA5092	OtA5093-OtA5097
Ccdc57	chr11:120826541-120932872	OtA5088-OtA5092	OtA5093-OtA5097
Ccdc60	chr5:116125580-116288985	OtA5088-OtA5092	OtA5093-OtA5097
Ccdc62	chr5:123930688-123969895	OtA5088-OtA5092	OtA5093-OtA5097
Ccdc63	chr5:122108055-122138062	OtA5088-OtA5092	OtA5093-OtA5097
Ccdc64b	chr17:23660522-23677449	OtA5088-OtA5092	OtA5093-OtA5097
Ccdc65	chr15:98708226-98723333	OtA5088-OtA5092	OtA5093-OtA5097
Ccdc67	chr9:15559863-15641220	OtA5088-OtA5092	OtA5093-OtA5097
Ccdc7	chr8:128980018-129065492	OtA5088-OtA5092	OtA5093-OtA5097
Ccdc70	chr8:21970595-21974041	OtA5088-OtA5092	OtA5093-OtA5097
Ccdc73	chr2:104886323-105017027	OtA5088-OtA5092	OtA5093-OtA5097
Ccdc74a	chr16:17646469-17650738	OtA5088-OtA5092	OtA5093-OtA5097
Ccdc75	chr17:78835515-78848308	OtA5088-OtA5092	OtA5093-OtA5097
Ccdc76	chr3:116562972-116630984	OtA5088-OtA5092	OtA5093-OtA5097
Ccdc78	chr17:25786579-25792284	OtA5088-OtA5092	OtA5093-OtA5097
Ccdc79	chr8:104446718-104624396	OtA5088-OtA5092	OtA5093-OtA5097
Ccdc81	chr7:89866147-89903629	OtA5088-OtA5092	OtA5093-OtA5097
Ccdc83	chr7:90223877-90265432	OtA5088-OtA5092	OtA5093-OtA5097

Ccdc85a	chr11:28385691-28584296	OtA5088-OtA5092	OtA5093-OtA5097
Ccdc87	chr19:4839365-4842528	OtA5088-OtA5092	OtA5093-OtA5097
Ccdc88a	chr11:29373774-29510811	OtA5088-OtA5092	OtA5093-OtA5097
Ccdc88b	chr19:6844622-6858211	OtA5088-OtA5092	OtA5093-OtA5097
Ccdc88c	chr12:100912699-101028983	OtA5088-OtA5092	OtA5093-OtA5097
Ccdc89	chr7:90426311-90428664	OtA5088-OtA5092	OtA5093-OtA5097
Ccdc96	chr5:36484587-36488171	OtA5088-OtA5092	OtA5093-OtA5097
Ccdc99	chr11:34809184-34833641	OtA5088-OtA5092	OtA5093-OtA5097
Cchcr1	chr17:35517115-35531015	OtA5088-OtA5092	OtA5093-OtA5097
Ccin	chr4:43983503-43985533	OtA5088-OtA5092	OtA5093-OtA5097
Cckar	chr5:53698484-53707704	OtA5088-OtA5092	OtA5093-OtA5097
Cckbr	chr7:105425819-105436338	OtA5088-OtA5092	OtA5093-OtA5097
Ccl1	chr11:82176665-82179812	OtA5088-OtA5092	OtA5093-OtA5097
Ccl11	chr11:82057831-82062955	OtA5088-OtA5092	OtA5093-OtA5097
Ccl12	chr11:82101844-82103399	OtA5088-OtA5092	OtA5093-OtA5097
Ccl17	chr8:94810452-94812036	OtA5088-OtA5092	OtA5093-OtA5097
Ccl19	chr4_JH584294_random:102333-10432[illegible]	OtA5088-OtA5092	OtA5093-OtA5097
Ccl20	chr1:83116765-83119167	OtA5088-OtA5092	OtA5093-OtA5097
Ccl21b,Ccl21c	chr4_JH584293_random:59768-60899	OtA5088-OtA5092	OtA5093-OtA5097
Ccl21c	chr4_JH584293_random:59768-60899	OtA5088-OtA5092	OtA5093-OtA5097
Ccl22	chr8:94745683-94751388	OtA5088-OtA5092	OtA5093-OtA5097
Ccl24	chr5:135569936-135573043	OtA5088-OtA5092	OtA5093-OtA5097
Ccl26	chr5:135560447-135563569	OtA5088-OtA5092	OtA5093-OtA5097
Ccl27b	chr4:41889794-42462993	OtA5088-OtA5092	OtA5093-OtA5097
Ccl28	chr13:119623818-119654358	OtA5088-OtA5092	OtA5093-OtA5097
Ccl3	chr11:83647842-83649378	OtA5088-OtA5092	OtA5093-OtA5097
Ccl4	chr11:83662583-83664683	OtA5088-OtA5092	OtA5093-OtA5097
Ccl5	chr11:83525778-83530518	OtA5088-OtA5092	OtA5093-OtA5097
Ccl7	chr11:82045711-82047523	OtA5088-OtA5092	OtA5093-OtA5097
Ccl8	chr11:82115184-82116799	OtA5088-OtA5092	OtA5093-OtA5097
Ccl9	chr11:83572916-83578636	OtA5088-OtA5092	OtA5093-OtA5097
Ccna1	chr3:55045468-55055055	OtA5088-OtA5092	OtA5093-OtA5097
Ccna2	chr3:36552605-36571996	OtA5088-OtA5092	OtA5093-OtA5097
Ccnb1	chr13:100778738-100786486	OtA5088-OtA5092	OtA5093-OtA5097
Ccnb1ip1	chr14:50789248-50795728	OtA5088-OtA5092	OtA5093-OtA5097
Ccnb2	chr9:70407688-70421554	OtA5088-OtA5092	OtA5093-OtA5097
Ccnb3	chrX:6979651-7041619	OtA5088-OtA5092	OtA5093-OtA5097
Ccne2	chr4:11191353-11254259	OtA5088-OtA5092	OtA5093-OtA5097
Ccno	chr13:112987801-112990778	OtA5088-OtA5092	OtA5093-OtA5097
Ccr1	chr9:123962125-123968692	OtA5088-OtA5092	OtA5093-OtA5097
Ccr10	chr11:101172997-101175443	OtA5088-OtA5092	OtA5093-OtA5097
Ccr2	chr9:124102182-124109140	OtA5088-OtA5092	OtA5093-OtA5097
Ccr3	chr9:124021970-124031689	OtA5088-OtA5092	OtA5093-OtA5097
Ccr4	chr9:114490315-114496544	OtA5088-OtA5092	OtA5093-OtA5097
Ccr5	chr9:124121542-124127183	OtA5088-OtA5092	OtA5093-OtA5097
Ccr6	chr17:8236042-8257129	OtA5088-OtA5092	OtA5093-OtA5097
Ccr7	chr11:99144198-99155077	OtA5088-OtA5092	OtA5093-OtA5097

Ccr8	chr9:120092132-120094906	OtA5088-OtA5092	OtA5093-OtA5097
Ccr9	chr9:123767210-123783457	OtA5088-OtA5092	OtA5093-OtA5097
Ccrl1	chr9:104063702-104127646	OtA5088-OtA5092	OtA5093-OtA5097
Cct8l1	chr5:25516066-25518027	OtA5088-OtA5092	OtA5093-OtA5097
Cd101	chr3:100993528-101029495	OtA5088-OtA5092	OtA5093-OtA5097
Cd160	chr3:96798762-96829351	OtA5088-OtA5092	OtA5093-OtA5097
Cd163	chr6:124304650-124330527	OtA5088-OtA5092	OtA5093-OtA5097
Cd163l1	chr7:140218266-140231145	OtA5088-OtA5092	OtA5093-OtA5097
Cd164l2	chr4:133220808-133224554	OtA5088-OtA5092	OtA5093-OtA5097
Cd177	chr7:24743982-24760311	OtA5088-OtA5092	OtA5093-OtA5097
Cd180	chr13:102693557-102706631	OtA5088-OtA5092	OtA5093-OtA5097
Cd19	chr7:126408447-126414870	OtA5088-OtA5092	OtA5093-OtA5097
Cd1d1	chr3:86995835-86999340	OtA5088-OtA5092	OtA5093-OtA5097
Cd1d2	chr3:86986585-86989532	OtA5088-OtA5092	OtA5093-OtA5097
Cd2	chr3:101275907-101287939	OtA5088-OtA5092	OtA5093-OtA5097
Cd200r1	chr16:44765735-44794977	OtA5088-OtA5092	OtA5093-OtA5097
Cd200r2	chr16:44867096-44915840	OtA5088-OtA5092	OtA5093-OtA5097
Cd200r3	chr16:44943677-44981380	OtA5088-OtA5092	OtA5093-OtA5097
Cd200r4	chr16:44820727-44839150	OtA5088-OtA5092	OtA5093-OtA5097
Cd207	chr6:83671206-83677857	OtA5088-OtA5092	OtA5093-OtA5097
Cd209a	chr8:3743394-3748984	OtA5088-OtA5092	OtA5093-OtA5097
Cd209b	chr8:3917656-3926814	OtA5088-OtA5092	OtA5093-OtA5097
Cd209c	chr8:3940221-3946863	OtA5088-OtA5092	OtA5093-OtA5097
Cd209d	chr8:3871823-3878499	OtA5088-OtA5092	OtA5093-OtA5097
Cd209e	chr8:3847972-3854286	OtA5088-OtA5092	OtA5093-OtA5097
Cd209f	chr8:4102792-4105764	OtA5088-OtA5092	OtA5093-OtA5097
Cd209g	chr8:4134735-4137707	OtA5088-OtA5092	OtA5093-OtA5097
Cd22	chr7:30865403-30880342	OtA5088-OtA5092	OtA5093-OtA5097
Cd226	chr18:89197426-89270327	OtA5088-OtA5092	OtA5093-OtA5097
Cd247	chr1:165788680-166002634	OtA5088-OtA5092	OtA5093-OtA5097
Cd27	chr6:125224211-125239934	OtA5088-OtA5092	OtA5093-OtA5097
Cd28	chr1:60746387-60773359	OtA5088-OtA5092	OtA5093-OtA5097
Cd300a	chr11:114890040-114904651	OtA5088-OtA5092	OtA5093-OtA5097
Cd300c	chr11:114956277-114960417	OtA5088-OtA5092	OtA5093-OtA5097
Cd300e	chr11:115051916-115062038	OtA5088-OtA5092	OtA5093-OtA5097
Cd300lb	chr11:114922780-114934386	OtA5088-OtA5092	OtA5093-OtA5097
Cd300ld	chr11:114982445-114989886	OtA5088-OtA5092	OtA5093-OtA5097
Cd300lf	chr11:115091430-115162240	OtA5088-OtA5092	OtA5093-OtA5097
Cd300lh	chr11:115042130-115048295	OtA5088-OtA5092	OtA5093-OtA5097
Cd302	chr2:60251992-60284418	OtA5088-OtA5092	OtA5093-OtA5097
Cd33	chr7:43527455-43533171	OtA5088-OtA5092	OtA5093-OtA5097
Cd37	chr7:45233631-45238839	OtA5088-OtA5092	OtA5093-OtA5097
Cd3d	chr9:44981785-44987052	OtA5088-OtA5092	OtA5093-OtA5097
Cd3e	chr9:44998742-45009590	OtA5088-OtA5092	OtA5093-OtA5097
Cd3g	chr9:44969571-44980431	OtA5088-OtA5092	OtA5093-OtA5097
Cd4	chr6:124864692-124888209	OtA5088-OtA5092	OtA5093-OtA5097
Cd40lg	chrX:57212142-57224042	OtA5088-OtA5092	OtA5093-OtA5097

Cd46	chr1:195041899-195092248	OtA5088-OtA5092	OtA5093-OtA5097
Cd48	chr1:171682054-171705257	OtA5088-OtA5092	OtA5093-OtA5097
Cd5	chr19:10718142-10738974	OtA5088-OtA5092	OtA5093-OtA5097
Cd53	chr3:106758860-106790149	OtA5088-OtA5092	OtA5093-OtA5097
Cd59b	chr2:104071009-104084957	OtA5088-OtA5092	OtA5093-OtA5097
Cd5l	chr3:87357880-87371074	OtA5088-OtA5092	OtA5093-OtA5097
Cd6	chr19:10789338-10830058	OtA5088-OtA5092	OtA5093-OtA5097
Cd69	chr6:129267324-129275369	OtA5088-OtA5092	OtA5093-OtA5097
Cd7	chr11:121036748-121039478	OtA5088-OtA5092	OtA5093-OtA5097
Cd70	chr17:57145996-57149777	OtA5088-OtA5092	OtA5093-OtA5097
Cd79a	chr7:24897510-24902197	OtA5088-OtA5092	OtA5093-OtA5097
Cd79b	chr11:106311340-106314529	OtA5088-OtA5092	OtA5093-OtA5097
Cd80	chr16:38458932-38486932	OtA5088-OtA5092	OtA5093-OtA5097
Cd84	chr1:171839696-171890718	OtA5088-OtA5092	OtA5093-OtA5097
Cd86	chr16:36603868-36666077	OtA5088-OtA5092	OtA5093-OtA5097
Cd8a	chr6:71373426-71379171	OtA5088-OtA5092	OtA5093-OtA5097
Cd8b1	chr6:71322811-71337451	OtA5088-OtA5092	OtA5093-OtA5097
Cd96	chr16:46035656-46120248	OtA5088-OtA5092	OtA5093-OtA5097
Cdc14a	chr3:116272552-116424032	OtA5088-OtA5092	OtA5093-OtA5097
Cdc25c	chr18:34732996-34751533	OtA5088-OtA5092	OtA5093-OtA5097
Cdc6	chr11:98907888-98923942	OtA5088-OtA5092	OtA5093-OtA5097
Cdc7	chr5:106964321-106984431	OtA5088-OtA5092	OtA5093-OtA5097
Cdca2	chr14:67676353-67715841	OtA5088-OtA5092	OtA5093-OtA5097
Cdca5	chr19:6085096-6091773	OtA5088-OtA5092	OtA5093-OtA5097
Cdca7l	chr12:117843860-118199043	OtA5088-OtA5092	OtA5093-OtA5097
Cdca8	chr4:124918464-124936917	OtA5088-OtA5092	OtA5093-OtA5097
Cdcp2	chr4:107096890-107107748	OtA5088-OtA5092	OtA5093-OtA5097
Cdh10	chr15:18820328-19014234	OtA5088-OtA5092	OtA5093-OtA5097
Cdh12	chr15:21111451-21589533	OtA5088-OtA5092	OtA5093-OtA5097
Cdh15	chr8:122848373-122867397	OtA5088-OtA5092	OtA5093-OtA5097
Cdh16	chr8:104446718-104624396	OtA5088-OtA5092	OtA5093-OtA5097
Cdh17	chr4:11758156-11817905	OtA5088-OtA5092	OtA5093-OtA5097
Cdh18	chr15:23036462-23474418	OtA5088-OtA5092	OtA5093-OtA5097
Cdh19	chr1:110889194-110977372	OtA5088-OtA5092	OtA5093-OtA5097
Cdh2	chr18:16588876-16809049	OtA5088-OtA5092	OtA5093-OtA5097
Cdh20	chr1:104768818-104995481	OtA5088-OtA5092	OtA5093-OtA5097
Cdh22	chr2:165111506-165234737	OtA5088-OtA5092	OtA5093-OtA5097
Cdh26	chr2:178430606-178487366	OtA5088-OtA5092	OtA5093-OtA5097
Cdh6	chr15:13034199-13173639	OtA5088-OtA5092	OtA5093-OtA5097
Cdh7	chr1:109983736-110139001	OtA5088-OtA5092	OtA5093-OtA5097
Cdh8	chr8:99028768-99416457	OtA5088-OtA5092	OtA5093-OtA5097
Cdh9	chr15:16778100-16856777	OtA5088-OtA5092	OtA5093-OtA5097
Cdhr1	chr14:37077848-37098311	OtA5088-OtA5092	OtA5093-OtA5097
Cdhr3	chr12:33033795-33092875	OtA5088-OtA5092	OtA5093-OtA5097
Cdk15	chr1:59256906-59352369	OtA5088-OtA5092	OtA5093-OtA5097
Cdk3-ps	chr11:116216001-116220287	OtA5088-OtA5092	OtA5093-OtA5097
Cdk5r2	chr1:74855028-74857732	OtA5088-OtA5092	OtA5093-OtA5097

Cdk8	chr5:146231674-146302874	OtA5088-OtA5092	OtA5093-OtA5097
Cdkl1	chr12:69746847-69987002	OtA5088-OtA5092	OtA5093-OtA5097
Cdkl3	chr11:52004220-52084667	OtA5088-OtA5092	OtA5093-OtA5097
Cdkl4	chr17:80523549-80563834	OtA5088-OtA5092	OtA5093-OtA5097
Cdkl5	chrX:160768012-160994681	OtA5088-OtA5092	OtA5093-OtA5097
Cdkn2c	chr4:109660875-109665372	OtA5088-OtA5092	OtA5093-OtA5097
Cdkn3	chr14:46760540-46771525	OtA5088-OtA5092	OtA5093-OtA5097
Cdnf	chr2:3513064-3526376	OtA5088-OtA5092	OtA5093-OtA5097
Cdr1	chrX:61183245-61185558	OtA5088-OtA5092	OtA5093-OtA5097
Cdrt4	chr11:62951192-62993095	OtA5088-OtA5092	OtA5093-OtA5097
Cdsn	chr17:35552127-35557180	OtA5088-OtA5092	OtA5093-OtA5097
Cdx4	chrX:103321397-103330224	OtA5088-OtA5092	OtA5093-OtA5097
Ceacam10	chr7:24777203-24784655	OtA5088-OtA5092	OtA5093-OtA5097
Ceacam16	chr7:19852096-19861299	OtA5088-OtA5092	OtA5093-OtA5097
Ceacam19	chr7:19875741-19887965	OtA5088-OtA5092	OtA5093-OtA5097
Ceacam2	chr7:25379526-25566417	OtA5088-OtA5092	OtA5093-OtA5097
Ceacam20	chr7:19965411-19991111	OtA5088-OtA5092	OtA5093-OtA5097
Ceacam9	chr7:16721928-16726110	OtA5088-OtA5092	OtA5093-OtA5097
Cebpe	chr14:54710362-54712174	OtA5088-OtA5092	OtA5093-OtA5097
Cecr6	chr6:120488938-120493807	OtA5088-OtA5092	OtA5093-OtA5097
Cel	chr2:28555819-28563362	OtA5088-OtA5092	OtA5093-OtA5097
Cela2a	chr4:141793611-141826003	OtA5088-OtA5092	OtA5093-OtA5097
Cela3b	chr4:137421007-137430520	OtA5088-OtA5092	OtA5093-OtA5097
Celf3	chr3:94478830-94492193	OtA5088-OtA5092	OtA5093-OtA5097
Celf4	chr18:25477619-25753983	OtA5088-OtA5092	OtA5093-OtA5097
Celf5	chr10:81459227-81482709	OtA5088-OtA5092	OtA5093-OtA5097
Celf6	chr9:59578336-59607292	OtA5088-OtA5092	OtA5093-OtA5097
Celsr2	chr3:108390847-108415494	OtA5088-OtA5092	OtA5093-OtA5097
Celsr3	chr9:108826319-108852969	OtA5088-OtA5092	OtA5093-OtA5097
Cend1	chr7:141426450-141429420	OtA5088-OtA5092	OtA5093-OtA5097
Cenpe	chr3:135212562-135273540	OtA5088-OtA5092	OtA5093-OtA5097
Cenpf	chr1:189640613-189688086	OtA5088-OtA5092	OtA5093-OtA5097
Cenph	chr13:100759685-100775899	OtA5088-OtA5092	OtA5093-OtA5097
Cenpi	chrX:134308162-134362639	OtA5088-OtA5092	OtA5093-OtA5097
Cenpj	chr14:56526760-56571846	OtA5088-OtA5092	OtA5093-OtA5097
Cenpk	chr13:104228954-104249617	OtA5088-OtA5092	OtA5093-OtA5097
Cenpl	chr1:161070891-161086343	OtA5088-OtA5092	OtA5093-OtA5097
Cenpm	chr15:82233775-82244747	OtA5088-OtA5092	OtA5093-OtA5097
Cenpn	chr8:116921739-116941503	OtA5088-OtA5092	OtA5093-OtA5097
Cenpo	chr12:4133396-4240123	OtA5088-OtA5092	OtA5093-OtA5097
Cenpp	chr13:49464058-49652731	OtA5088-OtA5092	OtA5093-OtA5097
Cenpq	chr17:40923054-40934551	OtA5088-OtA5092	OtA5093-OtA5097
Cenpw	chr10:30196008-30200540	OtA5088-OtA5092	OtA5093-OtA5097
Cep135	chr5:76591713-76646466	OtA5088-OtA5092	OtA5093-OtA5097
Cep152	chr2:125563087-125625113	OtA5088-OtA5092	OtA5093-OtA5097
Cep290	chr10:100488288-100589259	OtA5088-OtA5092	OtA5093-OtA5097
Cep55	chr19:38055024-38074425	OtA5088-OtA5092	OtA5093-OtA5097

Cep72	chr13:74036494-74062285	OtA5088-OtA5092	OtA5093-OtA5097
Cep97	chr16:55899887-55934848	OtA5088-OtA5092	OtA5093-OtA5097
Cer1	chr4:82881750-82885151	OtA5088-OtA5092	OtA5093-OtA5097
Cerkl	chr2:79255425-79428988	OtA5088-OtA5092	OtA5093-OtA5097
Ces1a	chr8:93020214-93048192	OtA5088-OtA5092	OtA5093-OtA5097
Ces1b	chr8:93056726-93080017	OtA5088-OtA5092	OtA5093-OtA5097
Ces1c	chr8:93099015-93131283	OtA5088-OtA5092	OtA5093-OtA5097
Ces1d	chr8:93166071-93197804	OtA5088-OtA5092	OtA5093-OtA5097
Ces1e	chr8:93201217-93229619	OtA5088-OtA5092	OtA5093-OtA5097
Ces1f	chr8:93256235-93279736	OtA5088-OtA5092	OtA5093-OtA5097
Ces1g	chr8:93302368-93337209	OtA5088-OtA5092	OtA5093-OtA5097
Ces2a	chr8:104734002-104741634	OtA5088-OtA5092	OtA5093-OtA5097
Ces2c	chr8:104847067-104854482	OtA5088-OtA5092	OtA5093-OtA5097
Ces2d-ps	chr8:104867423-104874082	OtA5088-OtA5092	OtA5093-OtA5097
Ces2e	chr8:104926259-104934672	OtA5088-OtA5092	OtA5093-OtA5097
Ces2f	chr8:104947355-104955962	OtA5088-OtA5092	OtA5093-OtA5097
Ces2g	chr8:104961717-104969537	OtA5088-OtA5092	OtA5093-OtA5097
Ces3a	chr8:105048598-105058414	OtA5088-OtA5092	OtA5093-OtA5097
Ces3b	chr8:105083754-105093591	OtA5088-OtA5092	OtA5093-OtA5097
Ces4a	chr8:105131799-105150109	OtA5088-OtA5092	OtA5093-OtA5097
Ces5a	chr8:93499377-93535707	OtA5088-OtA5092	OtA5093-OtA5097
Cetn1	chr18:9618418-9619469	OtA5088-OtA5092	OtA5093-OtA5097
Cetn4	chr3:37308626-37312446	OtA5088-OtA5092	OtA5093-OtA5097
Cfc1	chr1:34535647-34544311	OtA5088-OtA5092	OtA5093-OtA5097
Cfd	chr10:79697304-80369637	OtA5088-OtA5092	OtA5093-OtA5097
Cfhr1	chr1:139547063-139560222	OtA5088-OtA5092	OtA5093-OtA5097
Cfhr2	chr1:139810291-139858699	OtA5088-OtA5092	OtA5093-OtA5097
Cftr	chr6:18170686-18322769	OtA5088-OtA5092	OtA5093-OtA5097
Cga	chr4:34893778-34907374	OtA5088-OtA5092	OtA5093-OtA5097
Ch25h	chr19:34473783-34475135	OtA5088-OtA5092	OtA5093-OtA5097
Chac2	chr11:30954397-31102704	OtA5088-OtA5092	OtA5093-OtA5097
Chad	chr11:94557101-94601786	OtA5088-OtA5092	OtA5093-OtA5097
Chat	chr14:32408202-32465909	OtA5088-OtA5092	OtA5093-OtA5097
Chd5	chr4:152338650-152390194	OtA5088-OtA5092	OtA5093-OtA5097
Chek1	chr9:36708481-36726658	OtA5088-OtA5092	OtA5093-OtA5097
Chek2	chr5:110840016-110874133	OtA5088-OtA5092	OtA5093-OtA5097
Chga	chr12:102554968-102565027	OtA5088-OtA5092	OtA5093-OtA5097
Chgb	chr2:132781277-132795072	OtA5088-OtA5092	OtA5093-OtA5097
Chi3l1	chr1:134182403-134190031	OtA5088-OtA5092	OtA5093-OtA5097
Chi3l3	chr3:106147553-106167564	OtA5088-OtA5092	OtA5093-OtA5097
Chi3l4	chr3:106201490-106219479	OtA5088-OtA5092	OtA5093-OtA5097
Chia	chr3:106113381-106132116	OtA5088-OtA5092	OtA5093-OtA5097
Chic1	chrX:103356475-103396118	OtA5088-OtA5092	OtA5093-OtA5097
Chit1	chr1:134111241-134151436	OtA5088-OtA5092	OtA5093-OtA5097
Chl1	chr6:103510875-103733035	OtA5088-OtA5092	OtA5093-OtA5097
Chn1	chr2:73596525-73775346	OtA5088-OtA5092	OtA5093-OtA5097
Chodl	chr16:78930947-78951728	OtA5088-OtA5092	OtA5093-OtA5097

Chrdl1	chrX:143285673-143394262	OtA5088-OtA5092	OtA5093-OtA5097
Chrm1	chr19:8664004-8683602	OtA5088-OtA5092	OtA5093-OtA5097
Chrm2	chr6:36333137-36524774	OtA5088-OtA5092	OtA5093-OtA5097
Chrm3	chr13:9876612-10360803	OtA5088-OtA5092	OtA5093-OtA5097
Chrm4	chr2:91922188-91932282	OtA5088-OtA5092	OtA5093-OtA5097
Chrm5	chr2:112479071-112480817	OtA5088-OtA5092	OtA5093-OtA5097
Chrna1	chr2:73563280-73580306	OtA5088-OtA5092	OtA5093-OtA5097
Chrna10	chr7:102111265-102116713	OtA5088-OtA5092	OtA5093-OtA5097
Chrna2	chr14:66140959-66152948	OtA5088-OtA5092	OtA5093-OtA5097
Chrna3	chr9:55011342-55026559	OtA5088-OtA5092	OtA5093-OtA5097
Chrna5	chr9:54980879-55007779	OtA5088-OtA5092	OtA5093-OtA5097
Chrna6	chr8:27403211-27413944	OtA5088-OtA5092	OtA5093-OtA5097
Chrna7	chr7:63098691-63212526	OtA5088-OtA5092	OtA5093-OtA5097
Chrna9	chr5:65967123-65977486	OtA5088-OtA5092	OtA5093-OtA5097
Chrnb2	chr3:89715021-89764632	OtA5088-OtA5092	OtA5093-OtA5097
Chrnb3	chr8:27368710-27399730	OtA5088-OtA5092	OtA5093-OtA5097
Chrnb4	chr9:55028155-55048544	OtA5088-OtA5092	OtA5093-OtA5097
Chrnd	chr1:87190651-87200070	OtA5088-OtA5092	OtA5093-OtA5097
Chrne	chr11:70614882-70619194	OtA5088-OtA5092	OtA5093-OtA5097
Chrng	chr1:87205810-87211835	OtA5088-OtA5092	OtA5093-OtA5097
Chst10	chr1:38863872-38898160	OtA5088-OtA5092	OtA5093-OtA5097
Chst3	chr10:60181527-60219260	OtA5088-OtA5092	OtA5093-OtA5097
Chst4	chr8:110029074-110039334	OtA5088-OtA5092	OtA5093-OtA5097
Chst5	chr8:111889134-111910199	OtA5088-OtA5092	OtA5093-OtA5097
Chst9	chr18:15452174-15718046	OtA5088-OtA5092	OtA5093-OtA5097
Chtf18	chr17:25717171-25727415	OtA5088-OtA5092	OtA5093-OtA5097
Cib3	chr8:72204334-72212837	OtA5088-OtA5092	OtA5093-OtA5097
Cib4	chr5:30485583-30545836	OtA5088-OtA5092	OtA5093-OtA5097
Cidea	chr18:67321208-67367794	OtA5088-OtA5092	OtA5093-OtA5097
Ciita	chr16:10480071-10527564	OtA5088-OtA5092	OtA5093-OtA5097
Cilp	chr9:65265179-65280605	OtA5088-OtA5092	OtA5093-OtA5097
Cit	chr5:115845655-116006341	OtA5088-OtA5092	OtA5093-OtA5097
Ckap2	chr8:22168151-22185819	OtA5088-OtA5092	OtA5093-OtA5097
Ckap2l	chr2:129268209-129297212	OtA5088-OtA5092	OtA5093-OtA5097
Ckm	chr7:19411093-19421583	OtA5088-OtA5092	OtA5093-OtA5097
Ckmt2	chr13:91853386-91876885	OtA5088-OtA5092	OtA5093-OtA5097
Clca2	chr3:144796558-144819494	OtA5088-OtA5092	OtA5093-OtA5097
Clca3	chr3:145004536-145032776	OtA5088-OtA5092	OtA5093-OtA5097
Clca4	chr3:144822623-144849302	OtA5088-OtA5092	OtA5093-OtA5097
Clca5	chr3:145070264-145099041	OtA5088-OtA5092	OtA5093-OtA5097
Clca6	chr3:144952485-144975045	OtA5088-OtA5092	OtA5093-OtA5097
Clcn1	chr6:42286684-42314656	OtA5088-OtA5092	OtA5093-OtA5097
Clcnka	chr4:141384610-141398706	OtA5088-OtA5092	OtA5093-OtA5097
Clcnkb	chr4:141404356-141415988	OtA5088-OtA5092	OtA5093-OtA5097
Cldn13	chr5:134914249-134915530	OtA5088-OtA5092	OtA5093-OtA5097
Cldn14	chr16:93919030-94008837	OtA5088-OtA5092	OtA5093-OtA5097
Cldn15	chr5:136967868-136975845	OtA5088-OtA5092	OtA5093-OtA5097

Cldn16	chr16:26463134-26482764	OtA5088-OtA5092	OtA5093-OtA5097
Cldn17	chr16:88505806-88506978	OtA5088-OtA5092	OtA5093-OtA5097
Cldn18	chr9:99690796-99717267	OtA5088-OtA5092	OtA5093-OtA5097
Cldn19	chr4:119255440-119262438	OtA5088-OtA5092	OtA5093-OtA5097
Cldn20	chr17:3326572-3557713	OtA5088-OtA5092	OtA5093-OtA5097
Cldn22	chr8:47824481-47825475	OtA5088-OtA5092	OtA5093-OtA5097
Cldn24	chr8:47822142-47822805	OtA5088-OtA5092	OtA5093-OtA5097
Cldn26	chr16:8409275-8425136	OtA5088-OtA5092	OtA5093-OtA5097
Cldn27	chr11:117860751-117865543	OtA5088-OtA5092	OtA5093-OtA5097
Cldn8	chr16:88560825-88563183	OtA5088-OtA5092	OtA5093-OtA5097
Cldn9	chr17:23682583-23684026	OtA5088-OtA5092	OtA5093-OtA5097
Cldnd2	chr7:43440815-43443320	OtA5088-OtA5092	OtA5093-OtA5097
Clec10a	chr11:70166622-70170836	OtA5088-OtA5092	OtA5093-OtA5097
Clec11a	chr7:44303765-44306959	OtA5088-OtA5092	OtA5093-OtA5097
Clec12a	chr6:129350243-129365303	OtA5088-OtA5092	OtA5093-OtA5097
Clec18a	chr8:111069496-111081708	OtA5088-OtA5092	OtA5093-OtA5097
Clec2e	chr6:129091815-129100903	OtA5088-OtA5092	OtA5093-OtA5097
Clec2g	chr6:128934380-128984707	OtA5088-OtA5092	OtA5093-OtA5097
Clec2i	chr6:128848043-128896848	OtA5088-OtA5092	OtA5093-OtA5097
Clec2l	chr6:38663068-38680865	OtA5088-OtA5092	OtA5093-OtA5097
Clec3a	chr8:114418033-114428133	OtA5088-OtA5092	OtA5093-OtA5097
Clec3b	chr9:123150945-123157432	OtA5088-OtA5092	OtA5093-OtA5097
Clec4a1	chr6:122921847-122934619	OtA5088-OtA5092	OtA5093-OtA5097
Clec4a2	chr6:123122689-123143999	OtA5088-OtA5092	OtA5093-OtA5097
Clec4a3	chr6:122952514-122969878	OtA5088-OtA5092	OtA5093-OtA5097
Clec4a4	chr6:122990366-123024421	OtA5088-OtA5092	OtA5093-OtA5097
Clec4b1	chr6:123049961-123071555	OtA5088-OtA5092	OtA5093-OtA5097
Clec4b2	chr6:123173022-123204671	OtA5088-OtA5092	OtA5093-OtA5097
Clec4d	chr6:123262106-123275268	OtA5088-OtA5092	OtA5093-OtA5097
Clec4e	chr6:123281788-123289871	OtA5088-OtA5092	OtA5093-OtA5097
Clec4f	chr6:83644541-83656116	OtA5088-OtA5092	OtA5093-OtA5097
Clec4g	chr8:3716070-3720651	OtA5088-OtA5092	OtA5093-OtA5097
Clec4n	chr6:123229842-123247024	OtA5088-OtA5092	OtA5093-OtA5097
Clec5a	chr6:40529090-40910666	OtA5088-OtA5092	OtA5093-OtA5097
Clec7a	chr6:129461590-129472777	OtA5088-OtA5092	OtA5093-OtA5097
Clec9a	chr6:129408861-129424764	OtA5088-OtA5092	OtA5093-OtA5097
Clgn	chr8:83389890-83426829	OtA5088-OtA5092	OtA5093-OtA5097
Clip4	chr17:71769690-71858351	OtA5088-OtA5092	OtA5093-OtA5097
Clnk	chr5:38706456-38876693	OtA5088-OtA5092	OtA5093-OtA5097
Clrn1	chr3:58845214-58885212	OtA5088-OtA5092	OtA5093-OtA5097
Clrn2	chr5:45453750-45464149	OtA5088-OtA5092	OtA5093-OtA5097
Clspn	chr4:126556979-126593903	OtA5088-OtA5092	OtA5093-OtA5097
Clstn2	chr9:97444394-98033167	OtA5088-OtA5092	OtA5093-OtA5097
Clvs1	chr4:9269316-9669162	OtA5088-OtA5092	OtA5093-OtA5097
Clvs2	chr10:33512333-33624600	OtA5088-OtA5092	OtA5093-OtA5097
Cma2	chr14:55971427-55973995	OtA5088-OtA5092	OtA5093-OtA5097
Cmah	chr13:24327419-24477285	OtA5088-OtA5092	OtA5093-OtA5097

Cml1	chr6:85910153-85915677	OtA5088-OtA5092	OtA5093-OtA5097
Cml2	chr6:85865421-85869137	OtA5088-OtA5092	OtA5093-OtA5097
Cml3	chr6:85760630-85765744	OtA5088-OtA5092	OtA5093-OtA5097
Cml5	chr6:85817217-85820972	OtA5088-OtA5092	OtA5093-OtA5097
Cmtm1	chr8:104293541-104310145	OtA5088-OtA5092	OtA5093-OtA5097
Cmtm2a	chr8:104281041-104293181	OtA5088-OtA5092	OtA5093-OtA5097
Cmtm2b	chr8:104322236-104330756	OtA5088-OtA5092	OtA5093-OtA5097
Cmtm5	chr14:54936469-54939277	OtA5088-OtA5092	OtA5093-OtA5097
Cmya5	chr13:93040714-93144724	OtA5088-OtA5092	OtA5093-OtA5097
Cndp1	chr18:84610508-84650095	OtA5088-OtA5092	OtA5093-OtA5097
Cnfn	chr7:25367615-25369724	OtA5088-OtA5092	OtA5093-OtA5097
Cnga1	chr5:72603696-72642752	OtA5088-OtA5092	OtA5093-OtA5097
Cnga2	chrX:71991874-72010218	OtA5088-OtA5092	OtA5093-OtA5097
Cnga3	chr1:37219260-37263384	OtA5088-OtA5092	OtA5093-OtA5097
Cnga4	chr7:105404567-105408738	OtA5088-OtA5092	OtA5093-OtA5097
Cngb1	chr8:95239042-95306585	OtA5088-OtA5092	OtA5093-OtA5097
Cngb3	chr4:19280849-19510623	OtA5088-OtA5092	OtA5093-OtA5097
Cnih2	chr19:5088537-5098418	OtA5088-OtA5092	OtA5093-OtA5097
Cnih3	chr1:181352627-181460641	OtA5088-OtA5092	OtA5093-OtA5097
Cnksr2	chrX:157821572-158043110	OtA5088-OtA5092	OtA5093-OtA5097
Cnnm1	chr19:43440435-43497213	OtA5088-OtA5092	OtA5093-OtA5097
Cnpy1	chr5:28205462-28237873	OtA5088-OtA5092	OtA5093-OtA5097
Cnr1	chr4:33924631-33948831	OtA5088-OtA5092	OtA5093-OtA5097
Cnr2	chr4:135895403-135920210	OtA5088-OtA5092	OtA5093-OtA5097
Cntd1	chr11:101279202-101302267	OtA5088-OtA5092	OtA5093-OtA5097
Cntf	chr19:12763527-12796123	OtA5088-OtA5092	OtA5093-OtA5097
Cntln	chr4:84884326-85131918	OtA5088-OtA5092	OtA5093-OtA5097
Cntn1	chr15:92051164-92341967	OtA5088-OtA5092	OtA5093-OtA5097
Cntn2	chr1:132509424-132542940	OtA5088-OtA5092	OtA5093-OtA5097
Cntn3	chr6:102163305-102464667	OtA5088-OtA5092	OtA5093-OtA5097
Cntn4	chr6:105677744-106699305	OtA5088-OtA5092	OtA5093-OtA5097
Cntn5	chr9:9660890-10904775	OtA5088-OtA5092	OtA5093-OtA5097
Cntn6	chr6:104493043-104863405	OtA5088-OtA5092	OtA5093-OtA5097
Cntnap1	chr11:101176116-101190720	OtA5088-OtA5092	OtA5093-OtA5097
Cntnap2	chr6:45060060-47301371	OtA5088-OtA5092	OtA5093-OtA5097
Cntnap3	chr13:64737590-64903888	OtA5088-OtA5092	OtA5093-OtA5097
Cntnap4	chr8:112570042-112882707	OtA5088-OtA5092	OtA5093-OtA5097
Cntnap5a	chr1:115685136-116580674	OtA5088-OtA5092	OtA5093-OtA5097
Cntnap5b	chr1:99772764-100485942	OtA5088-OtA5092	OtA5093-OtA5097
Cntnap5c	chr17:57769569-58410342	OtA5088-OtA5092	OtA5093-OtA5097
Col10a1	chr10:34303611-34418528	OtA5088-OtA5092	OtA5093-OtA5097
Col11a2	chr17:34039436-34066242	OtA5088-OtA5092	OtA5093-OtA5097
Col17a1	chr19:47646340-47692042	OtA5088-OtA5092	OtA5093-OtA5097
Col19a1	chr1:24257682-24587437	OtA5088-OtA5092	OtA5093-OtA5097
Col22a1	chr15:71798475-72034227	OtA5088-OtA5092	OtA5093-OtA5097
Col24a1	chr3:145292471-145552005	OtA5088-OtA5092	OtA5093-OtA5097
Col25a1	chr3:130180844-130599883	OtA5088-OtA5092	OtA5093-OtA5097

Col28a1	chr6:7997807-8192617	OtA5088-OtA5092	OtA5093-OtA5097
Col2a1	chr15:97975601-98004724	OtA5088-OtA5092	OtA5093-OtA5097
Col4a3	chr1:82586920-82722059	OtA5088-OtA5092	OtA5093-OtA5097
Col4a4	chr1:82450722-82586849	OtA5088-OtA5092	OtA5093-OtA5097
Col4a6	chrX:141165402-141474076	OtA5088-OtA5092	OtA5093-OtA5097
Col6a4	chr9:105990317-106096691	OtA5088-OtA5092	OtA5093-OtA5097
Col6a5	chr9:105856069-105960643	OtA5088-OtA5092	OtA5093-OtA5097
Col6a6	chr9:105689416-105828085	OtA5088-OtA5092	OtA5093-OtA5097
Col7a1	chr9:108953744-108984875	OtA5088-OtA5092	OtA5093-OtA5097
Col8a1	chr16:57624255-57754737	OtA5088-OtA5092	OtA5093-OtA5097
Col9a1	chr1:24177609-24252738	OtA5088-OtA5092	OtA5093-OtA5097
Col9a3	chr2:180598221-180642691	OtA5088-OtA5092	OtA5093-OtA5097
Colec10	chr15:54410773-54466358	OtA5088-OtA5092	OtA5093-OtA5097
Colq	chr14:31523083-31577383	OtA5088-OtA5092	OtA5093-OtA5097
Coro6	chr11:77463912-77469501	OtA5088-OtA5092	OtA5093-OtA5097
Cort	chr4:149125190-149126741	OtA5088-OtA5092	OtA5093-OtA5097
Cox6a2	chr7:128173945-128206366	OtA5088-OtA5092	OtA5093-OtA5097
Cox7b2	chr5:71442822-71548205	OtA5088-OtA5092	OtA5093-OtA5097
Cox8b	chr7:140898941-140900446	OtA5088-OtA5092	OtA5093-OtA5097
Cox8c	chr12:102899305-102900534	OtA5088-OtA5092	OtA5093-OtA5097
Cpa1	chr6:30639220-30645361	OtA5088-OtA5092	OtA5093-OtA5097
Cpa2	chr6:30541641-30564473	OtA5088-OtA5092	OtA5093-OtA5097
Cpa3	chr3:20215615-20242181	OtA5088-OtA5092	OtA5093-OtA5097
Cpa4	chr6:30568375-30591747	OtA5088-OtA5092	OtA5093-OtA5097
Cpa5	chr6:30611009-30631521	OtA5088-OtA5092	OtA5093-OtA5097
Cpa6	chr1:10324726-10865874	OtA5088-OtA5092	OtA5093-OtA5097
Cpb1	chr3:20249483-20275729	OtA5088-OtA5092	OtA5093-OtA5097
Cpb2	chr14:75004109-75388146	OtA5088-OtA5092	OtA5093-OtA5097
Cphx	chr14:26221907-26235735	OtA5088-OtA5092	OtA5093-OtA5097
Cplx2	chr13:54371348-54383917	OtA5088-OtA5092	OtA5093-OtA5097
Cplx3	chr9:57599991-57606281	OtA5088-OtA5092	OtA5093-OtA5097
Cplx4	chr18:65955721-65970178	OtA5088-OtA5092	OtA5093-OtA5097
Cpn2	chr16:30256378-30267532	OtA5088-OtA5092	OtA5093-OtA5097
Cpne4	chr9:104569787-105034544	OtA5088-OtA5092	OtA5093-OtA5097
Cpne5	chr17:29156520-29237790	OtA5088-OtA5092	OtA5093-OtA5097
Cpne6	chr14:55510447-55517431	OtA5088-OtA5092	OtA5093-OtA5097
Cpne7	chr8:123117373-123135185	OtA5088-OtA5092	OtA5093-OtA5097
Cpne9	chr6:113282306-113305571	OtA5088-OtA5092	OtA5093-OtA5097
Cps1	chr1:67123065-67230448	OtA5088-OtA5092	OtA5093-OtA5097
Cpvl	chr6:53873380-53978662	OtA5088-OtA5092	OtA5093-OtA5097
Cpxcr1	chrX:116448943-116478783	OtA5088-OtA5092	OtA5093-OtA5097
Cpxm2	chr7:132042809-132154741	OtA5088-OtA5092	OtA5093-OtA5097
Cr2	chr1:195136810-195176715	OtA5088-OtA5092	OtA5093-OtA5097
Crb1	chr1:139198253-139377076	OtA5088-OtA5092	OtA5093-OtA5097
Crb2	chr2:37776248-38287384	OtA5088-OtA5092	OtA5093-OtA5097
Creb3l4	chr3:90237499-90243512	OtA5088-OtA5092	OtA5093-OtA5097
Creg2	chr1:39618405-39651182	OtA5088-OtA5092	OtA5093-OtA5097

Crh	chr3:19693400-19695396	OtA5088-OtA5092	OtA5093-OtA5097
Crhbp	chr13:95431375-95444831	OtA5088-OtA5092	OtA5093-OtA5097
Crhr1	chr11:104132881-104175518	OtA5088-OtA5092	OtA5093-OtA5097
Crhr2	chr6:55090047-55132972	OtA5088-OtA5092	OtA5093-OtA5097
Crip3	chr17:46428941-46431771	OtA5088-OtA5092	OtA5093-OtA5097
Crisp1	chr17:40293757-40319207	OtA5088-OtA5092	OtA5093-OtA5097
Crisp2	chr17:40764733-40794146	OtA5088-OtA5092	OtA5093-OtA5097
Crisp3	chr17:40221776-40242258	OtA5088-OtA5092	OtA5093-OtA5097
Crisp4	chr1:18115190-18137051	OtA5088-OtA5092	OtA5093-OtA5097
Crispld1	chr1:17727416-17766207	OtA5088-OtA5092	OtA5093-OtA5097
Crmp1	chr5:37242079-37292133	OtA5088-OtA5092	OtA5093-OtA5097
Crnn	chr3:93144786-93149471	OtA5088-OtA5092	OtA5093-OtA5097
Crp	chr1:172698055-172699966	OtA5088-OtA5092	OtA5093-OtA5097
Crtac1	chr19:42283036-42431783	OtA5088-OtA5092	OtA5093-OtA5097
Crtam	chr9:40972797-41004586	OtA5088-OtA5092	OtA5093-OtA5097
Crx	chr7:15865946-15879955	OtA5088-OtA5092	OtA5093-OtA5097
Crxos1	chr7:15882616-15904030	OtA5088-OtA5092	OtA5093-OtA5097
Cryba1	chr11:77718731-77725293	OtA5088-OtA5092	OtA5093-OtA5097
Cryba2	chr1:74889943-74893135	OtA5088-OtA5092	OtA5093-OtA5097
Cryba4	chr5:112246532-112252502	OtA5088-OtA5092	OtA5093-OtA5097
Crybb1	chr5:112255820-112269582	OtA5088-OtA5092	OtA5093-OtA5097
Crybb2	chr5:113058264-113068766	OtA5088-OtA5092	OtA5093-OtA5097
Crybb3	chr5:113075838-113081584	OtA5088-OtA5092	OtA5093-OtA5097
Cryga	chr1:65100388-65103363	OtA5088-OtA5092	OtA5093-OtA5097
Crygb	chr1:65080226-65082267	OtA5088-OtA5092	OtA5093-OtA5097
Crygc	chr1:65071530-65073521	OtA5088-OtA5092	OtA5093-OtA5097
Crygd	chr1:65061837-65063440	OtA5088-OtA5092	OtA5093-OtA5097
Cryge	chr1:65048556-65051163	OtA5088-OtA5092	OtA5093-OtA5097
Crygf	chr1:65926521-65928304	OtA5088-OtA5092	OtA5093-OtA5097
Crygn	chr5:24751001-24757843	OtA5088-OtA5092	OtA5093-OtA5097
Crygs	chr16:22805202-22811410	OtA5088-OtA5092	OtA5093-OtA5097
Csf2	chr11:54247269-54249899	OtA5088-OtA5092	OtA5093-OtA5097
Csf2rb2	chr15:78282509-78305603	OtA5088-OtA5092	OtA5093-OtA5097
Csf3	chr11:98701312-98703629	OtA5088-OtA5092	OtA5093-OtA5097
Csf3r	chr4:126024658-126044975	OtA5088-OtA5092	OtA5093-OtA5097
Csl	chr10:99757704-99759658	OtA5088-OtA5092	OtA5093-OtA5097
Csmd1	chr8:15892544-17535385	OtA5088-OtA5092	OtA5093-OtA5097
Csmd3	chr15:47580637-48791989	OtA5088-OtA5092	OtA5093-OtA5097
Csn1s1	chr5:87666223-87682574	OtA5088-OtA5092	OtA5093-OtA5097
Csn1s2a	chr5:87774566-87788797	OtA5088-OtA5092	OtA5093-OtA5097
Csn1s2b	chr5:87808121-87824421	OtA5088-OtA5092	OtA5093-OtA5097
Csn2	chr5:87692623-87699421	OtA5088-OtA5092	OtA5093-OtA5097
Csn3	chr5:87908587-87979351	OtA5088-OtA5092	OtA5093-OtA5097
Csnka2ip	chr16:64477810-64479134	OtA5088-OtA5092	OtA5093-OtA5097
Cspg5	chr9:110243782-110262576	OtA5088-OtA5092	OtA5093-OtA5097
Csprs	chr1_GL456221_random:111571-16301	OtA5088-OtA5092	OtA5093-OtA5097
Csrnp3	chr2:65845766-66031546	OtA5088-OtA5092	OtA5093-OtA5097

Csrp3	chr7:48830397-48848051	OtA5088-OtA5092	OtA5093-OtA5097
Cst10	chr2:149405248-149410278	OtA5088-OtA5092	OtA5093-OtA5097
Cst11	chr2:148768617-148771497	OtA5088-OtA5092	OtA5093-OtA5097
Cst12	chr2:148789360-148793437	OtA5088-OtA5092	OtA5093-OtA5097
Cst13	chr2:148820098-148830410	OtA5088-OtA5092	OtA5093-OtA5097
Cst6	chr19:5344704-5349574	OtA5088-OtA5092	OtA5093-OtA5097
Cst7	chr2:150570414-150578944	OtA5088-OtA5092	OtA5093-OtA5097
Cst8	chr2:148798838-148805584	OtA5088-OtA5092	OtA5093-OtA5097
Cst9	chr2:148835146-148838737	OtA5088-OtA5092	OtA5093-OtA5097
Csta	chr16:36119945-36131189	OtA5088-OtA5092	OtA5093-OtA5097
Cstad	chr2:30595043-30608945	OtA5088-OtA5092	OtA5093-OtA5097
Cstl1	chr2:148750360-148755378	OtA5088-OtA5092	OtA5093-OtA5097
Ctag2	chrX:65047643-65049017	OtA5088-OtA5092	OtA5093-OtA5097
Ctcfl	chr2:173093608-173119525	OtA5088-OtA5092	OtA5093-OtA5097
Ctf2	chr7:127718959-127725616	OtA5088-OtA5092	OtA5093-OtA5097
Ctla4	chr1:60909024-60915830	OtA5088-OtA5092	OtA5093-OtA5097
Ctnna2	chr6:76881636-77979667	OtA5088-OtA5092	OtA5093-OtA5097
Ctnna3	chr10:63430097-65003667	OtA5088-OtA5092	OtA5093-OtA5097
Ctnnd2	chr15:30172592-31029343	OtA5088-OtA5092	OtA5093-OtA5097
Ctrb1	chr8:111686509-111691010	OtA5088-OtA5092	OtA5093-OtA5097
Ctrc	chr4:141838239-141846990	OtA5088-OtA5092	OtA5093-OtA5097
Ctrl	chr8:105931993-105933862	OtA5088-OtA5092	OtA5093-OtA5097
Cts7	chr13:61352460-61358170	OtA5088-OtA5092	OtA5093-OtA5097
Cts8	chr13:61246746-61255348	OtA5088-OtA5092	OtA5093-OtA5097
Cts8-ps	chr13:61281379-61288850	OtA5088-OtA5092	OtA5093-OtA5097
Ctse	chr1:131638313-131675507	OtA5088-OtA5092	OtA5093-OtA5097
Ctsll3	chr13:60798249-60802844	OtA5088-OtA5092	OtA5093-OtA5097
Ctsw	chr19:5465239-5468498	OtA5088-OtA5092	OtA5093-OtA5097
Cttnbp2	chr6:18366476-18514825	OtA5088-OtA5092	OtA5093-OtA5097
Ctxn2	chr2:125136691-125147841	OtA5088-OtA5092	OtA5093-OtA5097
Ctxn3	chr18:57468485-57478134	OtA5088-OtA5092	OtA5093-OtA5097
Cux2	chr5:121860215-122047825	OtA5088-OtA5092	OtA5093-OtA5097
Cwf19l2	chr9:3404084-3479236	OtA5088-OtA5092	OtA5093-OtA5097
Cwh43	chr5:73406077-73453425	OtA5088-OtA5092	OtA5093-OtA5097
Cxcl1	chr5:90891244-90893115	OtA5088-OtA5092	OtA5093-OtA5097
Cxcl11	chr5:92331840-92414627	OtA5088-OtA5092	OtA5093-OtA5097
Cxcl13	chr5:95956938-95961068	OtA5088-OtA5092	OtA5093-OtA5097
Cxcl15	chr5:90794533-90803067	OtA5088-OtA5092	OtA5093-OtA5097
Cxcl17	chr7:25379526-25566417	OtA5088-OtA5092	OtA5093-OtA5097
Cxcl2	chr5:90903898-90905938	OtA5088-OtA5092	OtA5093-OtA5097
Cxcl3	chr5:90786102-90788090	OtA5088-OtA5092	OtA5093-OtA5097
Cxcl5	chr5:90759298-90761625	OtA5088-OtA5092	OtA5093-OtA5097
Cxcl9	chr5:92321330-92328079	OtA5088-OtA5092	OtA5093-OtA5097
Cxcr1	chr1:74191785-74194631	OtA5088-OtA5092	OtA5093-OtA5097
Cxcr3	chrX:101731535-101734147	OtA5088-OtA5092	OtA5093-OtA5097
Cxcr5	chr9:44511786-44526421	OtA5088-OtA5092	OtA5093-OtA5097
Cxcr6	chr9:123789509-123851899	OtA5088-OtA5092	OtA5093-OtA5097

Cxxc4	chr3:134236494-134262089	OtA5088-OtA5092	OtA5093-OtA5097
Cyb5r2	chr7:107595050-107758032	OtA5088-OtA5092	OtA5093-OtA5097
Cybb	chrX:9435251-9469324	OtA5088-OtA5092	OtA5093-OtA5097
Cybrd1	chr2:71118053-71142926	OtA5088-OtA5092	OtA5093-OtA5097
Cyct	chr2:76353942-76360448	OtA5088-OtA5092	OtA5093-OtA5097
Cygb	chr11:116645594-116654313	OtA5088-OtA5092	OtA5093-OtA5097
Cylc1	chrX:111110417-111123874	OtA5088-OtA5092	OtA5093-OtA5097
Cylc2	chr4:51216677-51229928	OtA5088-OtA5092	OtA5093-OtA5097
Cym	chr3:107211294-107221732	OtA5088-OtA5092	OtA5093-OtA5097
Cyp11b1	chr15:74834891-74841643	OtA5088-OtA5092	OtA5093-OtA5097
Cyp11b2	chr15:74851009-74856318	OtA5088-OtA5092	OtA5093-OtA5097
Cyp19a1	chr9:54165936-54193442	OtA5088-OtA5092	OtA5093-OtA5097
Cyp1a1	chr9:57687927-57703824	OtA5088-OtA5092	OtA5093-OtA5097
Cyp1a2	chr9:57676936-57683655	OtA5088-OtA5092	OtA5093-OtA5097
Cyp1b1	chr17:79706952-79715041	OtA5088-OtA5092	OtA5093-OtA5097
Cyp26a1	chr19:37697807-37701536	OtA5088-OtA5092	OtA5093-OtA5097
Cyp26c1	chr19:37685679-37693307	OtA5088-OtA5092	OtA5093-OtA5097
Cyp27b1	chr10:127048245-127053006	OtA5088-OtA5092	OtA5093-OtA5097
Cyp2a12	chr7:27029089-27036815	OtA5088-OtA5092	OtA5093-OtA5097
Cyp2a22	chr7:26931630-26939386	OtA5088-OtA5092	OtA5093-OtA5097
Cyp2a4	chr7:26307191-26315088	OtA5088-OtA5092	OtA5093-OtA5097
Cyp2a5	chr7:26835338-26843264	OtA5088-OtA5092	OtA5093-OtA5097
Cyp2ab1	chr16:20308386-20325404	OtA5088-OtA5092	OtA5093-OtA5097
Cyp2b10	chr7:25897663-25926624	OtA5088-OtA5092	OtA5093-OtA5097
Cyp2b13	chr7:26061494-26096196	OtA5088-OtA5092	OtA5093-OtA5097
Cyp2b19	chr7:26757186-26772630	OtA5088-OtA5092	OtA5093-OtA5097
Cyp2b23	chr7:26665226-26686430	OtA5088-OtA5092	OtA5093-OtA5097
Cyp2b9	chr7:26173408-26210660	OtA5088-OtA5092	OtA5093-OtA5097
Cyp2c29	chr19:39287084-39330713	OtA5088-OtA5092	OtA5093-OtA5097
Cyp2c37	chr19:39992423-40012243	OtA5088-OtA5092	OtA5093-OtA5097
Cyp2c38	chr19:39389555-39463075	OtA5088-OtA5092	OtA5093-OtA5097
Cyp2c39	chr19:39510821-39568529	OtA5088-OtA5092	OtA5093-OtA5097
Cyp2c40	chr19:39767072-39812814	OtA5088-OtA5092	OtA5093-OtA5097
Cyp2c44	chr19:44005021-44029247	OtA5088-OtA5092	OtA5093-OtA5097
Cyp2c50	chr19:40089678-40113955	OtA5088-OtA5092	OtA5093-OtA5097
Cyp2c53-ps	chr19:39229253-39274737	OtA5088-OtA5092	OtA5093-OtA5097
Cyp2c54	chr19:40037939-40073813	OtA5088-OtA5092	OtA5093-OtA5097
Cyp2c55	chr19:39007018-39042687	OtA5088-OtA5092	OtA5093-OtA5097
Cyp2c65	chr19:39061005-39093948	OtA5088-OtA5092	OtA5093-OtA5097
Cyp2c66	chr19:39113897-39186756	OtA5088-OtA5092	OtA5093-OtA5097
Cyp2c67	chr19:39608858-39649042	OtA5088-OtA5092	OtA5093-OtA5097
Cyp2c68	chr19:39688835-39741101	OtA5088-OtA5092	OtA5093-OtA5097
Cyp2c69	chr19:39842659-39886769	OtA5088-OtA5092	OtA5093-OtA5097
Cyp2c70	chr19:40153360-40187286	OtA5088-OtA5092	OtA5093-OtA5097
Cyp2d10	chr15:82402845-82407194	OtA5088-OtA5092	OtA5093-OtA5097
Cyp2d11	chr15:82389153-82394022	OtA5088-OtA5092	OtA5093-OtA5097
Cyp2d12	chr15:82555097-82559413	OtA5088-OtA5092	OtA5093-OtA5097

Cyp2d13	chr15:82636749-82642045	OtA5088-OtA5092	OtA5093-OtA5097
Cyp2d26	chr15:82790106-82794245	OtA5088-OtA5092	OtA5093-OtA5097
Cyp2d34	chr15:82615964-82620907	OtA5088-OtA5092	OtA5093-OtA5097
Cyp2d37-ps	chr15:82688749-82690058	OtA5088-OtA5092	OtA5093-OtA5097
Cyp2d40	chr15:82759832-82764122	OtA5088-OtA5092	OtA5093-OtA5097
Cyp2d9	chr15:82452376-82456827	OtA5088-OtA5092	OtA5093-OtA5097
Cyp2e1	chr7:140763831-140774977	OtA5088-OtA5092	OtA5093-OtA5097
Cyp2f2	chr7:27119954-27133660	OtA5088-OtA5092	OtA5093-OtA5097
Cyp2g1	chr7:26808926-26821197	OtA5088-OtA5092	OtA5093-OtA5097
Cyp2j11	chr4:96294508-96348680	OtA5088-OtA5092	OtA5093-OtA5097
Cyp2j12	chr4:96099317-96141152	OtA5088-OtA5092	OtA5093-OtA5097
Cyp2j13	chr4:96042659-96077540	OtA5088-OtA5092	OtA5093-OtA5097
Cyp2j5	chr4:96627431-96664119	OtA5088-OtA5092	OtA5093-OtA5097
Cyp2j6	chr4:96516137-96553651	OtA5088-OtA5092	OtA5093-OtA5097
Cyp2j8	chr4:96444587-96507386	OtA5088-OtA5092	OtA5093-OtA5097
Cyp2j9	chr4:96568428-96591485	OtA5088-OtA5092	OtA5093-OtA5097
Cyp2r1	chr7:114550167-114562972	OtA5088-OtA5092	OtA5093-OtA5097
Cyp2t4	chr7:27153713-27158564	OtA5088-OtA5092	OtA5093-OtA5097
Cyp2w1	chr5:139352616-139357033	OtA5088-OtA5092	OtA5093-OtA5097
Cyp3a11	chr5:145854606-145879854	OtA5088-OtA5092	OtA5093-OtA5097
Cyp3a13	chr5:137892932-137921619	OtA5088-OtA5092	OtA5093-OtA5097
Cyp3a16	chr5:145436308-145469723	OtA5088-OtA5092	OtA5093-OtA5097
Cyp3a25	chr5:145977193-146009618	OtA5088-OtA5092	OtA5093-OtA5097
Cyp3a41a	chr5:145694048-145720136	OtA5088-OtA5092	OtA5093-OtA5097
Cyp3a41b	chr5:145558663-145584730	OtA5088-OtA5092	OtA5093-OtA5097
Cyp3a44	chr5:145773982-145805874	OtA5088-OtA5092	OtA5093-OtA5097
Cyp3a57	chr5:145345269-145390512	OtA5088-OtA5092	OtA5093-OtA5097
Cyp3a59	chr5:146079257-146113283	OtA5088-OtA5092	OtA5093-OtA5097
Cyp46a1	chr12:108334380-108362234	OtA5088-OtA5092	OtA5093-OtA5097
Cyp4a10	chr4:115518286-115533649	OtA5088-OtA5092	OtA5093-OtA5097
Cyp4a12a	chr4:115299045-115332815	OtA5088-OtA5092	OtA5093-OtA5097
Cyp4a12b	chr4:115411623-115439034	OtA5088-OtA5092	OtA5093-OtA5097
Cyp4a14	chr4:115486199-115496141	OtA5088-OtA5092	OtA5093-OtA5097
Cyp4a29-ps	chr4:115242083-115254557	OtA5088-OtA5092	OtA5093-OtA5097
Cyp4a30b-ps	chr4:115452603-115471062	OtA5088-OtA5092	OtA5093-OtA5097
Cyp4a31	chr4:115563648-115579015	OtA5088-OtA5092	OtA5093-OtA5097
Cyp4a32	chr4:115600937-115622366	OtA5088-OtA5092	OtA5093-OtA5097
Cyp4b1	chr4:115624727-115647705	OtA5088-OtA5092	OtA5093-OtA5097
Cyp4f14	chr17:32905069-32917342	OtA5088-OtA5092	OtA5093-OtA5097
Cyp4f15	chr17:32685658-32703349	OtA5088-OtA5092	OtA5093-OtA5097
Cyp4f39	chr17:32452722-32493320	OtA5088-OtA5092	OtA5093-OtA5097
Cyp4f40	chr17:32659482-32676480	OtA5088-OtA5092	OtA5093-OtA5097
Cyp4f41-ps	chr17:32950960-32965716	OtA5088-OtA5092	OtA5093-OtA5097
Cyp4x1	chr4:115108681-115133977	OtA5088-OtA5092	OtA5093-OtA5097
Cyp7a1	chr4:6265611-6275631	OtA5088-OtA5092	OtA5093-OtA5097
Cyp7b1	chr3:18071949-18243338	OtA5088-OtA5092	OtA5093-OtA5097
Cyp8b1	chr9:121914355-121916305	OtA5088-OtA5092	OtA5093-OtA5097

Cypt1,Cypt8	chrX:16522871-16523689	OtA5088-OtA5092	OtA5093-OtA5097
Cypt10,Cypt4	chr9:24557047-24696293	OtA5088-OtA5092	OtA5093-OtA5097
Cypt12	chr3:17948443-17948952	OtA5088-OtA5092	OtA5093-OtA5097
Cypt14	chrX:39862918-39863604	OtA5088-OtA5092	OtA5093-OtA5097
Cypt15	chrX:39346265-39346961	OtA5088-OtA5092	OtA5093-OtA5097
Cypt2-ps	chrX:105499771-105500637	OtA5088-OtA5092	OtA5093-OtA5097
Cypt3	chrX:153558592-153559429	OtA5088-OtA5092	OtA5093-OtA5097
Cypt4	chr9:24557047-24696293	OtA5088-OtA5092	OtA5093-OtA5097
Cypt7	chrX:16522871-16523689	OtA5088-OtA5092	OtA5093-OtA5097
Cypt8	chrX:16522871-16523689	OtA5088-OtA5092	OtA5093-OtA5097
Cysltr1	chrX:106576516-106710557	OtA5088-OtA5092	OtA5093-OtA5097
Cytip	chr2:58129138-58160122	OtA5088-OtA5092	OtA5093-OtA5097
Cytl1	chr5:37735518-37739820	OtA5088-OtA5092	OtA5093-OtA5097
D030018L15Rik	chr15:96073336-96079552	OtA5088-OtA5092	OtA5093-OtA5097
D030024E09Rik	chr15:61699283-61774451	OtA5088-OtA5092	OtA5093-OtA5097
D030025E07Rik	chr3:128117014-128231605	OtA5088-OtA5092	OtA5093-OtA5097
D030025P21Rik	chr12:84783211-84879755	OtA5088-OtA5092	OtA5093-OtA5097
D030040B21Rik	chr1:16657551-16662278	OtA5088-OtA5092	OtA5093-OtA5097
D030045P18Rik	chr10:45807703-45852018	OtA5088-OtA5092	OtA5093-OtA5097
D030047H15Rik	chr7:4074124-4136926	OtA5088-OtA5092	OtA5093-OtA5097
D130009I18Rik	chr14:104639146-104966783	OtA5088-OtA5092	OtA5093-OtA5097
D130017N08Rik	chr5:143758353-143764942	OtA5088-OtA5092	OtA5093-OtA5097
D130020L05Rik	chr12:101039408-101088927	OtA5088-OtA5092	OtA5093-OtA5097
D130040H23Rik	chr8:69271079-69303375	OtA5088-OtA5092	OtA5093-OtA5097
D130043K22Rik	chr13:24845130-24901270	OtA5088-OtA5092	OtA5093-OtA5097
D14Ertd449e	chr14:26202666-26207041	OtA5088-OtA5092	OtA5093-OtA5097
D14Ertd668e	chr14:59347406-59365490	OtA5088-OtA5092	OtA5093-OtA5097
D16Ertd519e	chr16:70616424-70624822	OtA5088-OtA5092	OtA5093-OtA5097
D17Ertd648e	chr17:10840383-12063360	OtA5088-OtA5092	OtA5093-OtA5097
D17H6S56E-3	chr17:35016578-35028016	OtA5088-OtA5092	OtA5093-OtA5097
D1Pas1	chr1:186967415-186970627	OtA5088-OtA5092	OtA5093-OtA5097
D230030E09Rik	chr12:118518296-118530193	OtA5088-OtA5092	OtA5093-OtA5097
D2Ertd750e	chr2:118814002-118836212	OtA5088-OtA5092	OtA5093-OtA5097
D330022K07Rik	chr8:45507787-45827906	OtA5088-OtA5092	OtA5093-OtA5097
D330041H03Rik	chr17:24352045-24414513	OtA5088-OtA5092	OtA5093-OtA5097
D330045A20Rik	chrX:139480366-139554580	OtA5088-OtA5092	OtA5093-OtA5097
D330050G23Rik	chr2:116900151-116912791	OtA5088-OtA5092	OtA5093-OtA5097
D330050I16Rik	chr19:5377522-5390069	OtA5088-OtA5092	OtA5093-OtA5097
D3Bwg0562e	chr3:117319145-117360876	OtA5088-OtA5092	OtA5093-OtA5097
D3Ertd254e	chr3:36151079-36170341	OtA5088-OtA5092	OtA5093-OtA5097
D3Ertd751e	chr3:41742617-41758939	OtA5088-OtA5092	OtA5093-OtA5097
D430019H16Rik	chr12:105453855-105493095	OtA5088-OtA5092	OtA5093-OtA5097
D430020J02Rik	chr12:116401946-116405161	OtA5088-OtA5092	OtA5093-OtA5097
D430036J16Rik	chr9:81631391-81645156	OtA5088-OtA5092	OtA5093-OtA5097
D430041D05Rik	chr2:104143074-104410334	OtA5088-OtA5092	OtA5093-OtA5097
D4Ertd617e	chr4:118620798-118631915	OtA5088-OtA5092	OtA5093-OtA5097
D530049I02Rik	chr1:73391384-73430779	OtA5088-OtA5092	OtA5093-OtA5097

D5Ertd577e,Gm3286	chr5:95456805-95485589	OtA5088-OtA5092	OtA5093-OtA5097
D5Ertd605e	chr5:147418619-147423044	OtA5088-OtA5092	OtA5093-OtA5097
D630003M21Rik	chr2:158182532-158229222	OtA5088-OtA5092	OtA5093-OtA5097
D630010B17Rik	chr15:94247201-94255606	OtA5088-OtA5092	OtA5093-OtA5097
D630013N20Rik	chr10:70601048-70655963	OtA5088-OtA5092	OtA5093-OtA5097
D630023F18Rik	chr1:65107312-65123193	OtA5088-OtA5092	OtA5093-OtA5097
D630029K05Rik	chr10:116964739-116969107	OtA5088-OtA5092	OtA5093-OtA5097
D630032N06Rik	chr11:85235165-85238304	OtA5088-OtA5092	OtA5093-OtA5097
D630033O11Rik	chr9:43259878-43280075	OtA5088-OtA5092	OtA5093-OtA5097
D630037F22Rik	chr10:56014293-56228689	OtA5088-OtA5092	OtA5093-OtA5097
D630039A03Rik	chr4:57908383-57916264	OtA5088-OtA5092	OtA5093-OtA5097
D630041G03Rik	chr7:4464741-4479242	OtA5088-OtA5092	OtA5093-OtA5097
D630042F21Rik	chr6:129512556-129519227	OtA5088-OtA5092	OtA5093-OtA5097
D630042P16Rik	chr6:112359323-112388023	OtA5088-OtA5092	OtA5093-OtA5097
D630045M09Rik	chr13:73344664-73347384	OtA5088-OtA5092	OtA5093-OtA5097
D6Ertd474e	chr6:143245887-143297879	OtA5088-OtA5092	OtA5093-OtA5097
D6Ertd527e	chr6:87104745-87113003	OtA5088-OtA5092	OtA5093-OtA5097
D6Mm5e	chr6:82946921-83030309	OtA5088-OtA5092	OtA5093-OtA5097
D730001G18Rik	chr15:74770907-74778859	OtA5088-OtA5092	OtA5093-OtA5097
D730005E14Rik	chr15:79889531-79908429	OtA5088-OtA5092	OtA5093-OtA5097
D730039F16Rik	chr2:34874435-34892133	OtA5088-OtA5092	OtA5093-OtA5097
D730045A05Rik	chr18:73928485-74064949	OtA5088-OtA5092	OtA5093-OtA5097
D730048I06Rik	chr9:35788049-35790112	OtA5088-OtA5092	OtA5093-OtA5097
D730050B12Rik	chr13:72809600-72816763	OtA5088-OtA5092	OtA5093-OtA5097
D7Ertd715e	chr7:59969576-59974431	OtA5088-OtA5092	OtA5093-OtA5097
D830005E20Rik	chr10:33083482-33476709	OtA5088-OtA5092	OtA5093-OtA5097
D830013O20Rik	chr12:73364074-73409563	OtA5088-OtA5092	OtA5093-OtA5097
D830015G02Rik	chr14:54968786-54994549	OtA5088-OtA5092	OtA5093-OtA5097
D830029L11	chr18:16816406-16822783	OtA5088-OtA5092	OtA5093-OtA5097
D830030K20Rik	chr14:3846244-3856080	OtA5088-OtA5092	OtA5093-OtA5097
D830046C22Rik	chr5:139359738-139460534	OtA5088-OtA5092	OtA5093-OtA5097
D930007P13Rik	chr15:103123069-103146828	OtA5088-OtA5092	OtA5093-OtA5097
D930015M05Rik	chr2:92403646-92434069	OtA5088-OtA5092	OtA5093-OtA5097
D930016D06Rik	chr5:104525734-104554211	OtA5088-OtA5092	OtA5093-OtA5097
D930020B18Rik	chr10:121641700-121693914	OtA5088-OtA5092	OtA5093-OtA5097
D930028M14Rik	chr7:25152456-25156630	OtA5088-OtA5092	OtA5093-OtA5097
D930032P07Rik	chr19:28262447-28720027	OtA5088-OtA5092	OtA5093-OtA5097
DXBay18,Gm14685	chrX:73137231-73158399	OtA5088-OtA5092	OtA5093-OtA5097
Dach1	chr14:97786846-98169543	OtA5088-OtA5092	OtA5093-OtA5097
Dach2	chrX:113298255-113836386	OtA5088-OtA5092	OtA5093-OtA5097
Daf2	chr1:130388527-130422999	OtA5088-OtA5092	OtA5093-OtA5097
Dagla	chr19:10245264-10304877	OtA5088-OtA5092	OtA5093-OtA5097
Dapl1	chr2:59484652-59505020	OtA5088-OtA5092	OtA5093-OtA5097
Darc	chr1:173331885-173333503	OtA5088-OtA5092	OtA5093-OtA5097
Dazl	chr17:50279393-50293599	OtA5088-OtA5092	OtA5093-OtA5097
Dbc1	chr4:68761371-68954397	OtA5088-OtA5092	OtA5093-OtA5097
Dbh	chr2:27165506-27183204	OtA5088-OtA5092	OtA5093-OtA5097

Dbil5	chr11:76217612-76218665	OtA5088-OtA5092	OtA5093-OtA5097
Dbndd1	chr8:123504717-123515455	OtA5088-OtA5092	OtA5093-OtA5097
Dbpht2	chr12:74297473-74300468	OtA5088-OtA5092	OtA5093-OtA5097
Dbx1	chr7:49631498-49636835	OtA5088-OtA5092	OtA5093-OtA5097
Dbx2	chr15:95623562-95654771	OtA5088-OtA5092	OtA5093-OtA5097
Dcaf12l2	chrX:44365456-44368337	OtA5088-OtA5092	OtA5093-OtA5097
Dcc	chr18:71253631-72351069	OtA5088-OtA5092	OtA5093-OtA5097
Dcdc2a	chr13:25056003-25210706	OtA5088-OtA5092	OtA5093-OtA5097
Dcdc2b	chr4:129608330-129614257	OtA5088-OtA5092	OtA5093-OtA5097
Dcdc2c	chr12:28437794-28548337	OtA5088-OtA5092	OtA5093-OtA5097
Dclk1	chr3:55242525-55539068	OtA5088-OtA5092	OtA5093-OtA5097
Dclk2	chr3:86786149-86920884	OtA5088-OtA5092	OtA5093-OtA5097
Dclk3	chr9:111439080-111489611	OtA5088-OtA5092	OtA5093-OtA5097
Dcp1b	chr6:118592295-119221614	OtA5088-OtA5092	OtA5093-OtA5097
Dcpp1	chr17:23880875-23882853	OtA5088-OtA5092	OtA5093-OtA5097
Dcpp2	chr17:23898721-23900787	OtA5088-OtA5092	OtA5093-OtA5097
Dcpp3	chr17:23917457-23919441	OtA5088-OtA5092	OtA5093-OtA5097
Dcst1	chr3:89350234-89365239	OtA5088-OtA5092	OtA5093-OtA5097
Dcstamp	chr15:39745931-39760934	OtA5088-OtA5092	OtA5093-OtA5097
Dct	chr14:118012789-118052246	OtA5088-OtA5092	OtA5093-OtA5097
Dctd	chr8:48099091-48141667	OtA5088-OtA5092	OtA5093-OtA5097
Dcx	chrX:143855841-143933219	OtA5088-OtA5092	OtA5093-OtA5097
Ddi1	chr9:6168611-6377519	OtA5088-OtA5092	OtA5093-OtA5097
Ddit4l	chr3:137623671-137628332	OtA5088-OtA5092	OtA5093-OtA5097
Ddn	chr15:98803781-98807925	OtA5088-OtA5092	OtA5093-OtA5097
Ddo	chr10:40630010-40649931	OtA5088-OtA5092	OtA5093-OtA5097
Ddx11	chr17:66123519-66152168	OtA5088-OtA5092	OtA5093-OtA5097
Ddx25	chr9:35541847-35558470	OtA5088-OtA5092	OtA5093-OtA5097
Ddx4	chr13:112598332-112652310	OtA5088-OtA5092	OtA5093-OtA5097
Ddx43	chr9:78395776-78423589	OtA5088-OtA5092	OtA5093-OtA5097
Dear1	chr3:84815576-84979198	OtA5088-OtA5092	OtA5093-OtA5097
Defa-ps1	chr8:21695011-21695853	OtA5088-OtA5092	OtA5093-OtA5097
Defa-ps12	chr8:19210461-19212760	OtA5088-OtA5092	OtA5093-OtA5097
Defa-ps13	chr8:19300676-19304794	OtA5088-OtA5092	OtA5093-OtA5097
Defa-rs1	chr8:21325887-21327020	OtA5088-OtA5092	OtA5093-OtA5097
Defa-rs7,Defa23	chr8:21191572-21192550	OtA5088-OtA5092	OtA5093-OtA5097
Defa17	chr8:21655766-21656736	OtA5088-OtA5092	OtA5093-OtA5097
Defa20	chr8:21509255-21510237	OtA5088-OtA5092	OtA5093-OtA5097
Defa21	chr8:21025544-21026517	OtA5088-OtA5092	OtA5093-OtA5097
Defa22	chr8:21162276-21163249	OtA5088-OtA5092	OtA5093-OtA5097
Defa23	chr8:21191572-21192550	OtA5088-OtA5092	OtA5093-OtA5097
Defa24,Defa6	chr8:21734493-21735471	OtA5088-OtA5092	OtA5093-OtA5097
Defa25	chr8:21065037-21089644	OtA5088-OtA5092	OtA5093-OtA5097
Defa26	chr8:21618167-21618972	OtA5088-OtA5092	OtA5093-OtA5097
Defa3	chr8:21287408-21288377	OtA5088-OtA5092	OtA5093-OtA5097
Defa4	chr8:21065037-21089644	OtA5088-OtA5092	OtA5093-OtA5097
Defa5	chr8:21297393-21298375	OtA5088-OtA5092	OtA5093-OtA5097

Defa6	chr8:21734493-21735471	OtA5088-OtA5092	OtA5093-OtA5097
Defb1	chr8:21776554-21795185	OtA5088-OtA5092	OtA5093-OtA5097
Defb10	chr8:21858900-21862010	OtA5088-OtA5092	OtA5093-OtA5097
Defb11	chr8:21905373-21906432	OtA5088-OtA5092	OtA5093-OtA5097
Defb12	chr8:19111930-19114833	OtA5088-OtA5092	OtA5093-OtA5097
Defb13	chr8:21946761-21948622	OtA5088-OtA5092	OtA5093-OtA5097
Defb14	chr8:19194327-19195309	OtA5088-OtA5092	OtA5093-OtA5097
Defb15	chr8:21929812-21932710	OtA5088-OtA5092	OtA5093-OtA5097
Defb18	chr1:18236472-18237443	OtA5088-OtA5092	OtA5093-OtA5097
Defb19	chr2:152576085-152580312	OtA5088-OtA5092	OtA5093-OtA5097
Defb2	chr8:21839925-21843481	OtA5088-OtA5092	OtA5093-OtA5097
Defb20	chr2:152477062-152479934	OtA5088-OtA5092	OtA5093-OtA5097
Defb21	chr2:152572743-152574943	OtA5088-OtA5092	OtA5093-OtA5097
Defb22	chr2:152485665-152490138	OtA5088-OtA5092	OtA5093-OtA5097
Defb23	chr2:152459054-152464620	OtA5088-OtA5092	OtA5093-OtA5097
Defb25	chr2:152622355-152623053	OtA5088-OtA5092	OtA5093-OtA5097
Defb26	chr2:152507755-152511736	OtA5088-OtA5092	OtA5093-OtA5097
Defb28	chr2:152518254-152521447	OtA5088-OtA5092	OtA5093-OtA5097
Defb29	chr2:152538713-152540041	OtA5088-OtA5092	OtA5093-OtA5097
Defb3	chr8:19293360-19295339	OtA5088-OtA5092	OtA5093-OtA5097
Defb30	chr14:63034086-63037846	OtA5088-OtA5092	OtA5093-OtA5097
Defb33	chr8:20892666-20897723	OtA5088-OtA5092	OtA5093-OtA5097
Defb34	chr8:19123751-19126540	OtA5088-OtA5092	OtA5093-OtA5097
Defb35	chr8:21938351-21940878	OtA5088-OtA5092	OtA5093-OtA5097
Defb36	chr2:152604326-152612729	OtA5088-OtA5092	OtA5093-OtA5097
Defb37	chr8:18986232-18991055	OtA5088-OtA5092	OtA5093-OtA5097
Defb38	chr8:19023463-19026529	OtA5088-OtA5092	OtA5093-OtA5097
Defb39	chr8:19052825-19064810	OtA5088-OtA5092	OtA5093-OtA5097
Defb4	chr8:19198703-19201547	OtA5088-OtA5092	OtA5093-OtA5097
Defb40	chr8:18974939-18978116	OtA5088-OtA5092	OtA5093-OtA5097
Defb41	chr1:18250977-18265138	OtA5088-OtA5092	OtA5093-OtA5097
Defb42	chr14:63046990-63048606	OtA5088-OtA5092	OtA5093-OtA5097
Defb43	chr14:63011770-63018088	OtA5088-OtA5092	OtA5093-OtA5097
Defb44-ps	chr1:18210052-18223564	OtA5088-OtA5092	OtA5093-OtA5097
Defb45	chr2:152593190-152596485	OtA5088-OtA5092	OtA5093-OtA5097
Defb46	chr8:19239915-19242143	OtA5088-OtA5092	OtA5093-OtA5097
Defb47	chr14:62998076-63001160	OtA5088-OtA5092	OtA5093-OtA5097
Defb48	chr14:62977523-62984510	OtA5088-OtA5092	OtA5093-OtA5097
Defb48-ps	chr8:20876498-20889113	OtA5088-OtA5092	OtA5093-OtA5097
Defb5	chr8:19247591-19250828	OtA5088-OtA5092	OtA5093-OtA5097
Defb50	chr8:21823538-21831296	OtA5088-OtA5092	OtA5093-OtA5097
Defb6	chr8:19225477-19228209	OtA5088-OtA5092	OtA5093-OtA5097
Defb7	chr8:19495096-19497775	OtA5088-OtA5092	OtA5093-OtA5097
Defb8	chr8:19445769-19447606	OtA5088-OtA5092	OtA5093-OtA5097
Defb9	chr8:21881712-21885434	OtA5088-OtA5092	OtA5093-OtA5097
Dennd1c	chr17:57066054-57078510	OtA5088-OtA5092	OtA5093-OtA5097
Dennd2d	chr3:106481985-106501054	OtA5088-OtA5092	OtA5093-OtA5097

Depdc1a	chr3:159495432-159529955	OtA5088-OtA5092	OtA5093-OtA5097
Depdc1b	chr13:108316336-108389557	OtA5088-OtA5092	OtA5093-OtA5097
Dfna5	chr6:50207402-50261769	OtA5088-OtA5092	OtA5093-OtA5097
Dfnb59	chr2:76650272-76658556	OtA5088-OtA5092	OtA5093-OtA5097
Dgat2l6	chrX:100524837-100546108	OtA5088-OtA5092	OtA5093-OtA5097
Dgkb	chr12:37880704-38633410	OtA5088-OtA5092	OtA5093-OtA5097
Dgkg	chr16:22466568-22657231	OtA5088-OtA5092	OtA5093-OtA5097
Dgki	chr6:36846021-37299976	OtA5088-OtA5092	OtA5093-OtA5097
Dhdh	chr7:45473562-45488796	OtA5088-OtA5092	OtA5093-OtA5097
Dhfr	chr13:92211880-92389053	OtA5088-OtA5092	OtA5093-OtA5097
Dhh	chr15:98893026-98898540	OtA5088-OtA5092	OtA5093-OtA5097
Dhrs2	chr14:55222006-55241440	OtA5088-OtA5092	OtA5093-OtA5097
Dhrs7c	chr11:67798270-67816002	OtA5088-OtA5092	OtA5093-OtA5097
Dhrsx	chr4_GL456216_random:15880-36213	OtA5088-OtA5092	OtA5093-OtA5097
Diap3	chr14:86656322-87141114	OtA5088-OtA5092	OtA5093-OtA5097
Dio1	chr4:107291464-107307143	OtA5088-OtA5092	OtA5093-OtA5097
Dio2	chr12:90724551-90738438	OtA5088-OtA5092	OtA5093-OtA5097
Dio3	chr12:110279229-110281097	OtA5088-OtA5092	OtA5093-OtA5097
Dio3os	chr12:110275384-110278129	OtA5088-OtA5092	OtA5093-OtA5097
Diras1	chr10:81019589-81025377	OtA5088-OtA5092	OtA5093-OtA5097
Disc1	chr8:125054194-125261151	OtA5088-OtA5092	OtA5093-OtA5097
Dkc1	chrX:75095853-75130949	OtA5088-OtA5092	OtA5093-OtA5097
Dkk2	chr3:132085291-132180304	OtA5088-OtA5092	OtA5093-OtA5097
Dkk4	chr8:22624042-22627546	OtA5088-OtA5092	OtA5093-OtA5097
Dlec1	chr9:119102477-119147694	OtA5088-OtA5092	OtA5093-OtA5097
Dleu2	chr14:61598225-61682373	OtA5088-OtA5092	OtA5093-OtA5097
Dleu7	chr14:62276228-62292979	OtA5088-OtA5092	OtA5093-OtA5097
Dlg2	chr7:91090785-92449246	OtA5088-OtA5092	OtA5093-OtA5097
Dlgap1	chr17:70424791-70821413	OtA5088-OtA5092	OtA5093-OtA5097
Dlgap2	chr8:14095874-14847686	OtA5088-OtA5092	OtA5093-OtA5097
Dlgap3	chr4:127169270-127237022	OtA5088-OtA5092	OtA5093-OtA5097
Dlgap5	chr14:47387778-47418407	OtA5088-OtA5092	OtA5093-OtA5097
Dll3	chr7:28293554-28301785	OtA5088-OtA5092	OtA5093-OtA5097
Dlx1	chr2:71529444-71537891	OtA5088-OtA5092	OtA5093-OtA5097
Dlx1as	chr2:71529444-71537891	OtA5088-OtA5092	OtA5093-OtA5097
Dlx2	chr2:71543407-71546754	OtA5088-OtA5092	OtA5093-OtA5097
Dlx5	chr6:6877804-6882068	OtA5088-OtA5092	OtA5093-OtA5097
Dlx6	chr6:6820545-6869533	OtA5088-OtA5092	OtA5093-OtA5097
Dlx6as1	chr6:6820545-6869533	OtA5088-OtA5092	OtA5093-OtA5097
Dlx6as2	chr6:6820545-6869533	OtA5088-OtA5092	OtA5093-OtA5097
Dmbt1	chr7:131032075-131121628	OtA5088-OtA5092	OtA5093-OtA5097
Dmbx1	chr4:115915118-115939926	OtA5088-OtA5092	OtA5093-OtA5097
Dmc1	chr15:79561499-79605084	OtA5088-OtA5092	OtA5093-OtA5097
Dmp1	chr5:104202616-104214102	OtA5088-OtA5092	OtA5093-OtA5097
Dmrt1	chr19:25505705-25604328	OtA5088-OtA5092	OtA5093-OtA5097
Dmrt2	chr19:25672410-25678991	OtA5088-OtA5092	OtA5093-OtA5097
Dmrt3	chr19:25610536-25623921	OtA5088-OtA5092	OtA5093-OtA5097

Dmrta1	chr4:89688197-89694766	OtA5088-OtA5092	OtA5093-OtA5097
Dmrtb1	chr4:107676289-107684162	OtA5088-OtA5092	OtA5093-OtA5097
Dmrtc1c1	chrX:102802962-102809942	OtA5088-OtA5092	OtA5093-OtA5097
Dmrtc1c2	chrX:102847638-102854616	OtA5088-OtA5092	OtA5093-OtA5097
Dmrtc2	chr7:24870056-24877651	OtA5088-OtA5092	OtA5093-OtA5097
Dnahc1	chr14:31260374-31323896	OtA5088-OtA5092	OtA5093-OtA5097
Dnahc10	chr5:124725084-124834308	OtA5088-OtA5092	OtA5093-OtA5097
Dnahc11	chr12:117843860-118199043	OtA5088-OtA5092	OtA5093-OtA5097
Dnahc17	chr11:117986856-118129219	OtA5088-OtA5092	OtA5093-OtA5097
Dnahc2	chr11:69420808-69549108	OtA5088-OtA5092	OtA5093-OtA5097
Dnahc5	chr15:28203765-28472045	OtA5088-OtA5092	OtA5093-OtA5097
Dnahc6	chr6:73017606-73221631	OtA5088-OtA5092	OtA5093-OtA5097
Dnahc7a	chr1:53397001-53706784	OtA5088-OtA5092	OtA5093-OtA5097
Dnahc7b	chr1:46066737-46373550	OtA5088-OtA5092	OtA5093-OtA5097
Dnahc8	chr17:30626935-30875264	OtA5088-OtA5092	OtA5093-OtA5097
Dnahc9	chr11:65831323-66168551	OtA5088-OtA5092	OtA5093-OtA5097
Dnaic1	chr4:41569793-41640302	OtA5088-OtA5092	OtA5093-OtA5097
Dnaic2	chr11:114344525-114757886	OtA5088-OtA5092	OtA5093-OtA5097
Dnajb13	chr7:100503019-100514815	OtA5088-OtA5092	OtA5093-OtA5097
Dnajb7	chr15:81400187-81454888	OtA5088-OtA5092	OtA5093-OtA5097
Dnajb8	chr6:88222267-88223256	OtA5088-OtA5092	OtA5093-OtA5097
Dnajc28	chr16:91614256-91618999	OtA5088-OtA5092	OtA5093-OtA5097
Dnajc5b	chr3:19508594-19610862	OtA5088-OtA5092	OtA5093-OtA5097
Dnajc5g	chr5:31108318-31112524	OtA5088-OtA5092	OtA5093-OtA5097
Dnajc6	chr4:101496647-101642799	OtA5088-OtA5092	OtA5093-OtA5097
Dnalc1	chr12:84114327-84143510	OtA5088-OtA5092	OtA5093-OtA5097
Dnali1	chr4:125030013-125065657	OtA5088-OtA5092	OtA5093-OtA5097
Dnase1	chr16:4037144-4077810	OtA5088-OtA5092	OtA5093-OtA5097
Dnase1l2	chr17:24440767-24443101	OtA5088-OtA5092	OtA5093-OtA5097
Dnase2b	chr3:146581372-146631483	OtA5088-OtA5092	OtA5093-OtA5097
Dnd1	chr18:36760238-36766214	OtA5088-OtA5092	OtA5093-OtA5097
Dner	chr1:84369838-84696221	OtA5088-OtA5092	OtA5093-OtA5097
Dnm3	chr1:161987301-162478034	OtA5088-OtA5092	OtA5093-OtA5097
Dnm3os	chr1:161987301-162478034	OtA5088-OtA5092	OtA5093-OtA5097
Dntt	chr19:41029274-41059525	OtA5088-OtA5092	OtA5093-OtA5097
Doc2a	chr7:126847552-126861463	OtA5088-OtA5092	OtA5093-OtA5097
Doc2b	chr11:75769089-75796057	OtA5088-OtA5092	OtA5093-OtA5097
Dock10	chr1:80501072-80758553	OtA5088-OtA5092	OtA5093-OtA5097
Dock2	chr11:34226863-34514545	OtA5088-OtA5092	OtA5093-OtA5097
Dock3	chr9:106892824-107231909	OtA5088-OtA5092	OtA5093-OtA5097
Dok3	chr13:55523234-55528538	OtA5088-OtA5092	OtA5093-OtA5097
Dok5	chr2:170731806-170879775	OtA5088-OtA5092	OtA5093-OtA5097
Dok6	chr18:89301134-89769136	OtA5088-OtA5092	OtA5093-OtA5097
Dok7	chr5:35057083-35087831	OtA5088-OtA5092	OtA5093-OtA5097
Dpep2	chr8:105985056-105996423	OtA5088-OtA5092	OtA5093-OtA5097
Dpep3	chr8:105973519-105979419	OtA5088-OtA5092	OtA5093-OtA5097
Dpf1	chr7:29304004-29317586	OtA5088-OtA5092	OtA5093-OtA5097

Dpp10	chr1:123332137-124045559	OtA5088-OtA5092	OtA5093-OtA5097
Dpp4	chr2:62330072-62412231	OtA5088-OtA5092	OtA5093-OtA5097
Dpp6	chr5:26817356-27727500	OtA5088-OtA5092	OtA5093-OtA5097
Dppa1	chr11:46607009-46629274	OtA5088-OtA5092	OtA5093-OtA5097
Dppa2	chr16:48310273-48319513	OtA5088-OtA5092	OtA5093-OtA5097
Dppa3	chr6:122626423-122630271	OtA5088-OtA5092	OtA5093-OtA5097
Dppa4	chr16:48283734-48294292	OtA5088-OtA5092	OtA5093-OtA5097
Dpy19l2	chr9:24557047-24696293	OtA5088-OtA5092	OtA5093-OtA5097
Dpyd	chr3:118562177-119432918	OtA5088-OtA5092	OtA5093-OtA5097
Dpys	chr15:39768484-39857470	OtA5088-OtA5092	OtA5093-OtA5097
Dpysl4	chr7:139086000-139101791	OtA5088-OtA5092	OtA5093-OtA5097
Dpysl5	chr5:30711894-30799369	OtA5088-OtA5092	OtA5093-OtA5097
Drd1a	chr13:54051182-54055658	OtA5088-OtA5092	OtA5093-OtA5097
Drd2	chr9:49340661-49407214	OtA5088-OtA5092	OtA5093-OtA5097
Drd3	chr16:43762241-43822839	OtA5088-OtA5092	OtA5093-OtA5097
Drd5	chr5:38319508-38322310	OtA5088-OtA5092	OtA5093-OtA5097
Drp2	chrX:134404779-134456573	OtA5088-OtA5092	OtA5093-OtA5097
Dsc1	chr18:20084702-20114773	OtA5088-OtA5092	OtA5093-OtA5097
Dsc3	chr18:19960929-20002097	OtA5088-OtA5092	OtA5093-OtA5097
Dscam	chr16:96592078-97170735	OtA5088-OtA5092	OtA5093-OtA5097
Dscaml1	chr9:45416625-45753713	OtA5088-OtA5092	OtA5093-OtA5097
Dscc1	chr15:55076100-55090478	OtA5088-OtA5092	OtA5093-OtA5097
Dsg1a	chr18:20310872-20343353	OtA5088-OtA5092	OtA5093-OtA5097
Dsg1b	chr18:20376834-20409741	OtA5088-OtA5092	OtA5093-OtA5097
Dsg1c	chr18:20247339-20283923	OtA5088-OtA5092	OtA5093-OtA5097
Dsg3	chr18:20510303-20541310	OtA5088-OtA5092	OtA5093-OtA5097
Dsg4	chr18:20436174-20471821	OtA5088-OtA5092	OtA5093-OtA5097
Dspp	chr5:104170711-104180127	OtA5088-OtA5092	OtA5093-OtA5097
Dtl	chr1:191537364-191575534	OtA5088-OtA5092	OtA5093-OtA5097
Dub1	chr7:104857015-104862667	OtA5088-OtA5092	OtA5093-OtA5097
Dub1a	chr7:104840311-104842504	OtA5088-OtA5092	OtA5093-OtA5097
Dub2a	chr7:103250085-103252505	OtA5088-OtA5092	OtA5093-OtA5097
Dub3	chr7:104768048-104777470	OtA5088-OtA5092	OtA5093-OtA5097
Duox1	chr2:122315671-122347972	OtA5088-OtA5092	OtA5093-OtA5097
Duox2	chr2:122280436-122298165	OtA5088-OtA5092	OtA5093-OtA5097
Duoxa1	chr2:122303548-122313730	OtA5088-OtA5092	OtA5093-OtA5097
Duoxa2	chr2:122298899-122302885	OtA5088-OtA5092	OtA5093-OtA5097
Dus4l	chr12:31640054-31654826	OtA5088-OtA5092	OtA5093-OtA5097
Dusp15	chr2:152940994-152951582	OtA5088-OtA5092	OtA5093-OtA5097
Dusp21	chrX:18145869-18146692	OtA5088-OtA5092	OtA5093-OtA5097
Dusp26	chr8:31089661-31097047	OtA5088-OtA5092	OtA5093-OtA5097
Dusp27	chr1:166098147-166127898	OtA5088-OtA5092	OtA5093-OtA5097
Dux	chr10:58230650-58232675	OtA5088-OtA5092	OtA5093-OtA5097
Duxbl,Gm10391	chr14:26262257-26269434	OtA5088-OtA5092	OtA5093-OtA5097
Dydc1	chr14:41072910-41092197	OtA5088-OtA5092	OtA5093-OtA5097
Dydc2	chr14:41049208-41069074	OtA5088-OtA5092	OtA5093-OtA5097
Dync1i1	chr6:5725638-6028039	OtA5088-OtA5092	OtA5093-OtA5097

Dynlrb2	chr8:116505014-116515915	OtA5088-OtA5092	OtA5093-OtA5097
Dynlt1f	chr17:6646605-6655939	OtA5088-OtA5092	OtA5093-OtA5097
Dyrk4	chr6:126876198-126921839	OtA5088-OtA5092	OtA5093-OtA5097
Dytn	chr1:63622850-63686927	OtA5088-OtA5092	OtA5093-OtA5097
Dyx1c1	chr9:72958784-72973067	OtA5088-OtA5092	OtA5093-OtA5097
Dzip1	chr14:118875519-118923167	OtA5088-OtA5092	OtA5093-OtA5097
Dzip1l	chr9:99629594-99669256	OtA5088-OtA5092	OtA5093-OtA5097
Dzip3	chr16:48924227-48994112	OtA5088-OtA5092	OtA5093-OtA5097
E030002O03Rik	chr7:104153012-104164816	OtA5088-OtA5092	OtA5093-OtA5097
E030003E18Rik	chr19:20492714-20556410	OtA5088-OtA5092	OtA5093-OtA5097
E030013I19Rik	chr2:12106659-12312315	OtA5088-OtA5092	OtA5093-OtA5097
E030018B13Rik	chr7:63886350-63938915	OtA5088-OtA5092	OtA5093-OtA5097
E030019B06Rik	chr7:139600950-139616329	OtA5088-OtA5092	OtA5093-OtA5097
E030019B13Rik	chr12:56490427-56529420	OtA5088-OtA5092	OtA5093-OtA5097
E030025P04Rik	chr11:109139290-109144369	OtA5088-OtA5092	OtA5093-OtA5097
E030044B06Rik	chr19:40868315-40882639	OtA5088-OtA5092	OtA5093-OtA5097
E130003G02Rik	chr2:25289910-25319163	OtA5088-OtA5092	OtA5093-OtA5097
E130006D01Rik	chr5:111734283-111761728	OtA5088-OtA5092	OtA5093-OtA5097
E130008D07Rik	chr17:43146040-43158291	OtA5088-OtA5092	OtA5093-OtA5097
E130018N17Rik	chr2:168152602-168154513	OtA5088-OtA5092	OtA5093-OtA5097
E130112N10Rik	chr6:125224211-125239934	OtA5088-OtA5092	OtA5093-OtA5097
E130114P18Rik	chr4:97567874-98118876	OtA5088-OtA5092	OtA5093-OtA5097
E130201H02Rik	chr7:120597624-120598475	OtA5088-OtA5092	OtA5093-OtA5097
E130215H24Rik	chr2:150618110-150668932	OtA5088-OtA5092	OtA5093-OtA5097
E130218I03Rik	chr4:134243305-134245873	OtA5088-OtA5092	OtA5093-OtA5097
E130303B06Rik	chr8:105703651-105708168	OtA5088-OtA5092	OtA5093-OtA5097
E130304F04Rik	chr13:119710093-119755882	OtA5088-OtA5092	OtA5093-OtA5097
E130304I02Rik	chr7:35772345-35838074	OtA5088-OtA5092	OtA5093-OtA5097
E130306D19Rik	chr4:43496143-43499660	OtA5088-OtA5092	OtA5093-OtA5097
E130309D14Rik	chr11:74619604-74641516	OtA5088-OtA5092	OtA5093-OtA5097
E130309F12Rik	chr4:49059461-49340261	OtA5088-OtA5092	OtA5093-OtA5097
E130310I04Rik	chr16:34784949-35002434	OtA5088-OtA5092	OtA5093-OtA5097
E230008N13Rik	chr4:45890302-45950774	OtA5088-OtA5092	OtA5093-OtA5097
E230016K23Rik	chr11:83582060-83623693	OtA5088-OtA5092	OtA5093-OtA5097
E230016M11Rik	chr6:67035095-67080652	OtA5088-OtA5092	OtA5093-OtA5097
E230019M04Rik	chrX:140062878-140106797	OtA5088-OtA5092	OtA5093-OtA5097
E230025N22Rik	chr18:36684922-36695925	OtA5088-OtA5092	OtA5093-OtA5097
E230029C05Rik	chr7:90029158-90040280	OtA5088-OtA5092	OtA5093-OtA5097
E330009D11	chr3:36499803-36506809	OtA5088-OtA5092	OtA5093-OtA5097
E330009J07Rik	chr6:40407497-40436133	OtA5088-OtA5092	OtA5093-OtA5097
E330011O21Rik	chr16:78250862-78255454	OtA5088-OtA5092	OtA5093-OtA5097
E330012B07Rik	chr6:147180240-147208294	OtA5088-OtA5092	OtA5093-OtA5097
E330013P04Rik	chr19:60144674-60162591	OtA5088-OtA5092	OtA5093-OtA5097
E330014E10Rik,Gm328	chr5_GL456354_random:82558-110906	OtA5088-OtA5092	OtA5093-OtA5097
E330016A19Rik	chr9:78430517-78443147	OtA5088-OtA5092	OtA5093-OtA5097
E330017A01Rik	chr16:58635261-58638403	OtA5088-OtA5092	OtA5093-OtA5097
E330017L17Rik	chr4:129906225-129908996	OtA5088-OtA5092	OtA5093-OtA5097

E330021D16Rik	chr6:136400318-136415569	OtA5088-OtA5092	OtA5093-OtA5097
E330023G01	chr9:98748598-98820087	OtA5088-OtA5092	OtA5093-OtA5097
E330033B04Rik	chr15:96268563-96275275	OtA5088-OtA5092	OtA5093-OtA5097
E330033L03	chr9:58028681-58118823	OtA5088-OtA5092	OtA5093-OtA5097
E330034G19Rik	chr14:24293363-24309959	OtA5088-OtA5092	OtA5093-OtA5097
E430016F16Rik	chr7:78581065-78718220	OtA5088-OtA5092	OtA5093-OtA5097
E530001F21Rik	chrX:105164377-105165505	OtA5088-OtA5092	OtA5093-OtA5097
E530011L22Rik	chr9:121719180-121759943	OtA5088-OtA5092	OtA5093-OtA5097
EU599041	chr7:43214038-43226842	OtA5088-OtA5092	OtA5093-OtA5097
Eaf2	chr16:36792883-36874826	OtA5088-OtA5092	OtA5093-OtA5097
Ear10	chr14:43922897-43923368	OtA5088-OtA5092	OtA5093-OtA5097
Ear11	chr14:51255261-51256112	OtA5088-OtA5092	OtA5093-OtA5097
Ear12,Ear2	chr14:44102653-44103531	OtA5088-OtA5092	OtA5093-OtA5097
Ear14	chr14:51203689-51204156	OtA5088-OtA5092	OtA5093-OtA5097
Ear4	chr14:51203689-51204156	OtA5088-OtA5092	OtA5093-OtA5097
Ear5	chr14:51162259-51163018	OtA5088-OtA5092	OtA5093-OtA5097
Ear6,Ear7	chr14:51853767-51854642	OtA5088-OtA5092	OtA5093-OtA5097
Ear7	chr14:51853767-51854642	OtA5088-OtA5092	OtA5093-OtA5097
Ebf2	chr14:67233291-67429767	OtA5088-OtA5092	OtA5093-OtA5097
Ebf3	chr7:137193670-137314445	OtA5088-OtA5092	OtA5093-OtA5097
Ebi3	chr17:55952622-55957022	OtA5088-OtA5092	OtA5093-OtA5097
Ecel1	chr1:87147654-87155027	OtA5088-OtA5092	OtA5093-OtA5097
Echdc1	chr10:29313165-29362442	OtA5088-OtA5092	OtA5093-OtA5097
Eci3	chr13:34924408-34963809	OtA5088-OtA5092	OtA5093-OtA5097
Ecm2	chr13:49464058-49652731	OtA5088-OtA5092	OtA5093-OtA5097
Ect2	chr3:27097221-27153878	OtA5088-OtA5092	OtA5093-OtA5097
Ect2l	chr10:18129021-18210890	OtA5088-OtA5092	OtA5093-OtA5097
Eda	chrX:99975605-100400760	OtA5088-OtA5092	OtA5093-OtA5097
Eda2r	chrX:97333840-97377209	OtA5088-OtA5092	OtA5093-OtA5097
Edar	chr10:58600787-58675696	OtA5088-OtA5092	OtA5093-OtA5097
Edaradd	chr13:12471208-12520450	OtA5088-OtA5092	OtA5093-OtA5097
Eddm3b	chr14:51116552-51117791	OtA5088-OtA5092	OtA5093-OtA5097
Edil3	chr13:88821471-89323225	OtA5088-OtA5092	OtA5093-OtA5097
Edn3	chr2:174760772-174784042	OtA5088-OtA5092	OtA5093-OtA5097
Eef1a2	chr2:181147691-181157015	OtA5088-OtA5092	OtA5093-OtA5097
Efcab1	chr16:14906645-14924522	OtA5088-OtA5092	OtA5093-OtA5097
Efcab10	chr12:33394853-33401269	OtA5088-OtA5092	OtA5093-OtA5097
Efcab2	chr1:178405880-178483249	OtA5088-OtA5092	OtA5093-OtA5097
Efcab3	chr11:105092218-105117537	OtA5088-OtA5092	OtA5093-OtA5097
Efcab4b	chr6:127577974-127629938	OtA5088-OtA5092	OtA5093-OtA5097
Efcab5	chr11:77089914-77188968	OtA5088-OtA5092	OtA5093-OtA5097
Efcab6	chr15:83866711-84065349	OtA5088-OtA5092	OtA5093-OtA5097
Efcab7	chr4:99829499-99912783	OtA5088-OtA5092	OtA5093-OtA5097
Efcab8	chr2:153780878-153795182	OtA5088-OtA5092	OtA5093-OtA5097
Efcab9	chr11:32522732-32527574	OtA5088-OtA5092	OtA5093-OtA5097
Efemp1	chr11:28853204-28926743	OtA5088-OtA5092	OtA5093-OtA5097
Efha2	chr8:40308050-40386304	OtA5088-OtA5092	OtA5093-OtA5097

Efhb	chr17:53398888-53463321	OtA5088-OtA5092	OtA5093-OtA5097
Efhc2	chrX:17132048-17319368	OtA5088-OtA5092	OtA5093-OtA5097
Efna2	chr10:79697304-80369637	OtA5088-OtA5092	OtA5093-OtA5097
Efna3	chr3:89314950-89322879	OtA5088-OtA5092	OtA5093-OtA5097
Efna5	chr17:62602956-62881317	OtA5088-OtA5092	OtA5093-OtA5097
Efnb3	chr11:69554091-69560237	OtA5088-OtA5092	OtA5093-OtA5097
Egf	chr3:129677574-129763882	OtA5088-OtA5092	OtA5093-OtA5097
Egfbp2	chr7:44012677-44016969	OtA5088-OtA5092	OtA5093-OtA5097
Egfem1	chr3:29082576-29691056	OtA5088-OtA5092	OtA5093-OtA5097
Egfl6	chrX:166523006-166585716	OtA5088-OtA5092	OtA5093-OtA5097
Egfl8	chr17:34605860-34615971	OtA5088-OtA5092	OtA5093-OtA5097
Egr2	chr10:67537868-67542188	OtA5088-OtA5092	OtA5093-OtA5097
Egr3	chr14:70077444-70080157	OtA5088-OtA5092	OtA5093-OtA5097
Egr4	chr6:85511121-85513542	OtA5088-OtA5092	OtA5093-OtA5097
Ehf	chr2:103263432-103303196	OtA5088-OtA5092	OtA5093-OtA5097
Eid3	chr10:82833950-82897724	OtA5088-OtA5092	OtA5093-OtA5097
Eif4e1b	chr13:54784002-54788458	OtA5088-OtA5092	OtA5093-OtA5097
Elane	chr10:79697304-80369637	OtA5088-OtA5092	OtA5093-OtA5097
Elavl2	chr4:91250766-91399984	OtA5088-OtA5092	OtA5093-OtA5097
Elavl3	chr9:22015004-22052023	OtA5088-OtA5092	OtA5093-OtA5097
Elavl4	chr4:110203736-110351911	OtA5088-OtA5092	OtA5093-OtA5097
Elf5	chr2:103412097-103450988	OtA5088-OtA5092	OtA5093-OtA5097
Elfn1	chr5:139907942-139974724	OtA5088-OtA5092	OtA5093-OtA5097
Elfn2	chr15:78670006-78718113	OtA5088-OtA5092	OtA5093-OtA5097
Ell3	chr2:121439026-121442601	OtA5088-OtA5092	OtA5093-OtA5097
Elovl2	chr13:41182381-41220403	OtA5088-OtA5092	OtA5093-OtA5097
Elovl3	chr19:46131898-46148325	OtA5088-OtA5092	OtA5093-OtA5097
Elovl4	chr9:83778691-83806305	OtA5088-OtA5092	OtA5093-OtA5097
Elovl7	chr13:108214403-108287107	OtA5088-OtA5092	OtA5093-OtA5097
Eme1	chr11:94629823-94653754	OtA5088-OtA5092	OtA5093-OtA5097
Emid2	chr5:136741763-136883107	OtA5088-OtA5092	OtA5093-OtA5097
Eml5	chr12:98746967-98901484	OtA5088-OtA5092	OtA5093-OtA5097
Eml6	chr11:29692897-30026033	OtA5088-OtA5092	OtA5093-OtA5097
Emr4	chr17:55749983-55853662	OtA5088-OtA5092	OtA5093-OtA5097
Emx1	chr6:85187930-85204463	OtA5088-OtA5092	OtA5093-OtA5097
Emx2	chr19:59458689-59465357	OtA5088-OtA5092	OtA5093-OtA5097
Emx2os	chr19:59425103-59458635	OtA5088-OtA5092	OtA5093-OtA5097
En1	chr1:120602486-120607991	OtA5088-OtA5092	OtA5093-OtA5097
En2	chr5:28165695-28172166	OtA5088-OtA5092	OtA5093-OtA5097
Enam	chr5:88487974-88506049	OtA5088-OtA5092	OtA5093-OtA5097
Enkur	chr2:21180730-21205365	OtA5088-OtA5092	OtA5093-OtA5097
Eno4	chr19:58943424-58971421	OtA5088-OtA5092	OtA5093-OtA5097
Enox	chrX:103493575-103506425	OtA5088-OtA5092	OtA5093-OtA5097
Enox1	chr14:77156762-77721763	OtA5088-OtA5092	OtA5093-OtA5097
Enpp1	chr10:24641410-24712102	OtA5088-OtA5092	OtA5093-OtA5097
Enpp4	chr17:44096309-44105808	OtA5088-OtA5092	OtA5093-OtA5097
Enpp6	chr8:46986924-47094895	OtA5088-OtA5092	OtA5093-OtA5097

Enthd1	chr15:80452239-80560470	OtA5088-OtA5092	OtA5093-OtA5097
Entpd4	chr14:69555400-69584992	OtA5088-OtA5092	OtA5093-OtA5097
Entpd8	chr2:25080322-25095205	OtA5088-OtA5092	OtA5093-OtA5097
Eomes	chr9:118478188-118486132	OtA5088-OtA5092	OtA5093-OtA5097
Epb4.2	chr2:121018216-121036755	OtA5088-OtA5092	OtA5093-OtA5097
Epb4.9	chr14:70602183-70636048	OtA5088-OtA5092	OtA5093-OtA5097
Epgn	chr5:91027516-91035212	OtA5088-OtA5092	OtA5093-OtA5097
Epha10	chr4:124881784-124917800	OtA5088-OtA5092	OtA5093-OtA5097
Epha3	chr16:63545217-63864157	OtA5088-OtA5092	OtA5093-OtA5097
Epha5	chr5:84054764-84417382	OtA5088-OtA5092	OtA5093-OtA5097
Epha6	chr16:59613320-60605531	OtA5088-OtA5092	OtA5093-OtA5097
Epha7	chr4:28813144-28967499	OtA5088-OtA5092	OtA5093-OtA5097
Epha8	chr4:136929418-136956816	OtA5088-OtA5092	OtA5093-OtA5097
Ephb1	chr9:101922127-102354693	OtA5088-OtA5092	OtA5093-OtA5097
Ephb2	chr4:136653609-136835935	OtA5088-OtA5092	OtA5093-OtA5097
Ephx3	chr17:32183769-32189463	OtA5088-OtA5092	OtA5093-OtA5097
Ephx4	chr5:107403512-107430031	OtA5088-OtA5092	OtA5093-OtA5097
Epo	chr5:137483019-137485816	OtA5088-OtA5092	OtA5093-OtA5097
Epsti1	chr14:77904238-78002656	OtA5088-OtA5092	OtA5093-OtA5097
Epx	chr11:87863997-87875536	OtA5088-OtA5092	OtA5093-OtA5097
Epyc	chr10:97644067-97967090	OtA5088-OtA5092	OtA5093-OtA5097
Eras	chrX:7919821-7928607	OtA5088-OtA5092	OtA5093-OtA5097
Erbb4	chr1:68039965-69108059	OtA5088-OtA5092	OtA5093-OtA5097
Erc2	chr14:27622441-28478537	OtA5088-OtA5092	OtA5093-OtA5097
Ercc6l	chrX:102142819-102157091	OtA5088-OtA5092	OtA5093-OtA5097
Ereg	chr5:91074616-91093649	OtA5088-OtA5092	OtA5093-OtA5097
Eri2	chr7:119760922-119794058	OtA5088-OtA5092	OtA5093-OtA5097
Ermn	chr2:58045114-58052752	OtA5088-OtA5092	OtA5093-OtA5097
Ern2	chr7:122169892-122186216	OtA5088-OtA5092	OtA5093-OtA5097
Erp27	chr6:136907386-136922180	OtA5088-OtA5092	OtA5093-OtA5097
Esco2	chr14:65819026-65833969	OtA5088-OtA5092	OtA5093-OtA5097
Esm1	chr13:113209658-113218104	OtA5088-OtA5092	OtA5093-OtA5097
Esp24	chr17:39036694-39040227	OtA5088-OtA5092	OtA5093-OtA5097
Esp3	chr17:40632074-40637060	OtA5088-OtA5092	OtA5093-OtA5097
Esp38	chr17:39950519-39955287	OtA5088-OtA5092	OtA5093-OtA5097
Espl1	chr15:102296292-102324356	OtA5088-OtA5092	OtA5093-OtA5097
Espnl	chr1:91322074-91348303	OtA5088-OtA5092	OtA5093-OtA5097
Esr2	chr12:76120418-76177259	OtA5088-OtA5092	OtA5093-OtA5097
Esrrb	chr12:86361116-86521628	OtA5088-OtA5092	OtA5093-OtA5097
Esrrg	chr1:187609005-188214884	OtA5088-OtA5092	OtA5093-OtA5097
Esyt3	chr9:99309966-99358530	OtA5088-OtA5092	OtA5093-OtA5097
Etaa1	chr11:17938748-17953875	OtA5088-OtA5092	OtA5093-OtA5097
Etohd2	chr13:59769965-59823147	OtA5088-OtA5092	OtA5093-OtA5097
Etv1	chr12:38780257-38868215	OtA5088-OtA5092	OtA5093-OtA5097
Etv2	chr7:30633615-30635852	OtA5088-OtA5092	OtA5093-OtA5097
Etv4	chr11:101769741-101785310	OtA5088-OtA5092	OtA5093-OtA5097
Evc	chr5:37299170-37336881	OtA5088-OtA5092	OtA5093-OtA5097

Evi2a	chr11:79339891-79581609	OtA5088-OtA5092	OtA5093-OtA5097
Evi2b	chr11:79339891-79581609	OtA5088-OtA5092	OtA5093-OtA5097
Evx1	chr6:52308388-52318378	OtA5088-OtA5092	OtA5093-OtA5097
Evx2	chr2:74655615-74659419	OtA5088-OtA5092	OtA5093-OtA5097
Exd1	chr2:119519403-119547627	OtA5088-OtA5092	OtA5093-OtA5097
Exo1	chr1:175880777-175911396	OtA5088-OtA5092	OtA5093-OtA5097
Expi	chr11:83709003-83711360	OtA5088-OtA5092	OtA5093-OtA5097
Eya1	chr1:14168957-14310199	OtA5088-OtA5092	OtA5093-OtA5097
Eya2	chr2:165655297-165771631	OtA5088-OtA5092	OtA5093-OtA5097
Eya4	chr10:23104167-23349903	OtA5088-OtA5092	OtA5093-OtA5097
F10	chr8:13037307-13056676	OtA5088-OtA5092	OtA5093-OtA5097
F12	chr13:55417965-55426802	OtA5088-OtA5092	OtA5093-OtA5097
F13b	chr1:139501706-139523756	OtA5088-OtA5092	OtA5093-OtA5097
F2rl2	chr13:95627176-95891922	OtA5088-OtA5092	OtA5093-OtA5097
F420014N23Rik	chr10:127190377-127211622	OtA5088-OtA5092	OtA5093-OtA5097
F5	chr1:164115263-164220274	OtA5088-OtA5092	OtA5093-OtA5097
F630028O10Rik	chrX:96239925-96243642	OtA5088-OtA5092	OtA5093-OtA5097
F630042J09Rik	chr13:67278593-67283361	OtA5088-OtA5092	OtA5093-OtA5097
F630043A04Rik	chr14:57806560-57826163	OtA5088-OtA5092	OtA5093-OtA5097
F630111L10Rik	chr3:59006977-59344256	OtA5088-OtA5092	OtA5093-OtA5097
F630206G17Rik	chr11:45808082-45842878	OtA5088-OtA5092	OtA5093-OtA5097
F730035M05Rik	chr12:70227840-70234165	OtA5088-OtA5092	OtA5093-OtA5097
F730043M19Rik	chr12:33111710-33147586	OtA5088-OtA5092	OtA5093-OtA5097
F8	chrX:75172714-75380041	OtA5088-OtA5092	OtA5093-OtA5097
F830002L21Rik	chr10:43593426-43630913	OtA5088-OtA5092	OtA5093-OtA5097
F830016B08Rik	chr18:60293379-60303016	OtA5088-OtA5092	OtA5093-OtA5097
F830045P16Rik	chr2:129458358-129536602	OtA5088-OtA5092	OtA5093-OtA5097
F9	chrX:59999463-60030758	OtA5088-OtA5092	OtA5093-OtA5097
F930015N05Rik	chr11:64433134-64522835	OtA5088-OtA5092	OtA5093-OtA5097
Fabp1	chr6:71199887-71205023	OtA5088-OtA5092	OtA5093-OtA5097
Fabp12	chr3:10244208-10301183	OtA5088-OtA5092	OtA5093-OtA5097
Fabp2	chr3:122882591-122924116	OtA5088-OtA5092	OtA5093-OtA5097
Fabp6	chr11:43596111-43601511	OtA5088-OtA5092	OtA5093-OtA5097
Fabp7	chr10:57784922-57788450	OtA5088-OtA5092	OtA5093-OtA5097
Fabp9	chr3:10193623-10197222	OtA5088-OtA5092	OtA5093-OtA5097
Faim2	chr15:99497004-99528017	OtA5088-OtA5092	OtA5093-OtA5097
Faim3	chr1:130865776-130880790	OtA5088-OtA5092	OtA5093-OtA5097
Fam107a	chr14:8296277-8309776	OtA5088-OtA5092	OtA5093-OtA5097
Fam109b	chr15:82341178-82345710	OtA5088-OtA5092	OtA5093-OtA5097
Fam110c	chr12:31073967-31079940	OtA5088-OtA5092	OtA5093-OtA5097
Fam111a	chr19:12573524-12596566	OtA5088-OtA5092	OtA5093-OtA5097
Fam115e	chr6:42587212-42597372	OtA5088-OtA5092	OtA5093-OtA5097
Fam120c	chrX:151344222-151474134	OtA5088-OtA5092	OtA5093-OtA5097
Fam122c	chrX:53273432-53301501	OtA5088-OtA5092	OtA5093-OtA5097
Fam123a	chr14:60378285-60381003	OtA5088-OtA5092	OtA5093-OtA5097
Fam123c	chr1:34579692-34590944	OtA5088-OtA5092	OtA5093-OtA5097
Fam124b	chr1:80198698-80213944	OtA5088-OtA5092	OtA5093-OtA5097

Fam129c	chr8:71597645-71608149	OtA5088-OtA5092	OtA5093-OtA5097
Fam131b	chr6:42315311-42324640	OtA5088-OtA5092	OtA5093-OtA5097
Fam131c	chr4:141368219-141384175	OtA5088-OtA5092	OtA5093-OtA5097
Fam132b	chr1:91366429-91374217	OtA5088-OtA5092	OtA5093-OtA5097
Fam13c	chr10:70440667-70599291	OtA5088-OtA5092	OtA5093-OtA5097
Fam149a	chr8:45336714-45382291	OtA5088-OtA5092	OtA5093-OtA5097
Fam150a	chr1:6359330-6394731	OtA5088-OtA5092	OtA5093-OtA5097
Fam150b	chr12:30884323-30911612	OtA5088-OtA5092	OtA5093-OtA5097
Fam151a	chr4:106733914-106799831	OtA5088-OtA5092	OtA5093-OtA5097
Fam151b	chr13:92449619-92484031	OtA5088-OtA5092	OtA5093-OtA5097
Fam154a	chr4:86444697-86558303	OtA5088-OtA5092	OtA5093-OtA5097
Fam154b	chr7:82632959-82648528	OtA5088-OtA5092	OtA5093-OtA5097
Fam155a	chr8:9206009-9771023	OtA5088-OtA5092	OtA5093-OtA5097
Fam159b	chr13:104845282-104863893	OtA5088-OtA5092	OtA5093-OtA5097
Fam161a	chr11:23013386-23023741	OtA5088-OtA5092	OtA5093-OtA5097
Fam161b	chr12:84345316-84361821	OtA5088-OtA5092	OtA5093-OtA5097
Fam163a	chr1:156075965-156087888	OtA5088-OtA5092	OtA5093-OtA5097
Fam163b	chr2:27110378-27142477	OtA5088-OtA5092	OtA5093-OtA5097
Fam164b	chr10:13149643-13178023	OtA5088-OtA5092	OtA5093-OtA5097
Fam164c	chr12:85288590-85299358	OtA5088-OtA5092	OtA5093-OtA5097
Fam166a	chr2:25218744-25224702	OtA5088-OtA5092	OtA5093-OtA5097
Fam166b	chr4:43381981-43429134	OtA5088-OtA5092	OtA5093-OtA5097
Fam169a	chr13:97067286-97137926	OtA5088-OtA5092	OtA5093-OtA5097
Fam170a	chr18:50278368-50283019	OtA5088-OtA5092	OtA5093-OtA5097
Fam170b	chr14:32833961-32836788	OtA5088-OtA5092	OtA5093-OtA5097
Fam171b	chr2:83812727-83881358	OtA5088-OtA5092	OtA5093-OtA5097
Fam177a	chr12:55124527-55142082	OtA5088-OtA5092	OtA5093-OtA5097
Fam178b	chr1:36562695-36683183	OtA5088-OtA5092	OtA5093-OtA5097
Fam179a	chr17:71673260-71729669	OtA5088-OtA5092	OtA5093-OtA5097
Fam180a	chr6:35312745-35326141	OtA5088-OtA5092	OtA5093-OtA5097
Fam181a	chr12:103314958-103317065	OtA5088-OtA5092	OtA5093-OtA5097
Fam181b	chr7:93079878-93081721	OtA5088-OtA5092	OtA5093-OtA5097
Fam183b	chr11:58792801-58801960	OtA5088-OtA5092	OtA5093-OtA5097
Fam184a	chr10:53633144-53750909	OtA5088-OtA5092	OtA5093-OtA5097
Fam184b	chr5:45529704-45639501	OtA5088-OtA5092	OtA5093-OtA5097
Fam186b	chr15:99271017-99287180	OtA5088-OtA5092	OtA5093-OtA5097
Fam187a	chr11:102881243-102886731	OtA5088-OtA5092	OtA5093-OtA5097
Fam189a1	chr7:64756095-65156528	OtA5088-OtA5092	OtA5093-OtA5097
Fam18a	chr16:10411937-10447350	OtA5088-OtA5092	OtA5093-OtA5097
Fam194a	chr3:58616299-58637207	OtA5088-OtA5092	OtA5093-OtA5097
Fam196a	chr7:134670686-135173647	OtA5088-OtA5092	OtA5093-OtA5097
Fam196b	chr11:34226863-34514545	OtA5088-OtA5092	OtA5093-OtA5097
Fam198a	chr9:121950987-121980208	OtA5088-OtA5092	OtA5093-OtA5097
Fam19a1	chr6:96113153-96657198	OtA5088-OtA5092	OtA5093-OtA5097
Fam19a2	chr10:123264075-123741204	OtA5088-OtA5092	OtA5093-OtA5097
Fam19a3	chr3:104767404-104777547	OtA5088-OtA5092	OtA5093-OtA5097
Fam19a4	chr6:96831208-97060411	OtA5088-OtA5092	OtA5093-OtA5097

Fam23a	chr2:14174523-14221993	OtA5088-OtA5092	OtA5093-OtA5097
Fam24a	chr7:131334621-131336716	OtA5088-OtA5092	OtA5093-OtA5097
Fam25c	chr14:34351881-34355393	OtA5088-OtA5092	OtA5093-OtA5097
Fam26d	chr10:34037596-34109815	OtA5088-OtA5092	OtA5093-OtA5097
Fam26e	chr10:34037596-34109815	OtA5088-OtA5092	OtA5093-OtA5097
Fam26f	chr10:34126066-34127972	OtA5088-OtA5092	OtA5093-OtA5097
Fam33a	chr11:87109260-87122974	OtA5088-OtA5092	OtA5093-OtA5097
Fam3b	chr16:97471085-97504936	OtA5088-OtA5092	OtA5093-OtA5097
Fam43b	chr4:138394091-138396458	OtA5088-OtA5092	OtA5093-OtA5097
Fam46d	chrX:107816571-107872909	OtA5088-OtA5092	OtA5093-OtA5097
Fam47c	chrX:78737762-78739410	OtA5088-OtA5092	OtA5093-OtA5097
Fam47e	chr5:92571536-92591284	OtA5088-OtA5092	OtA5093-OtA5097
Fam50b	chr13:34739641-34747622	OtA5088-OtA5092	OtA5093-OtA5097
Fam54a	chr10:20347818-20361669	OtA5088-OtA5092	OtA5093-OtA5097
Fam55b	chr9:48318003-48340898	OtA5088-OtA5092	OtA5093-OtA5097
Fam55d	chr9:48362040-48400025	OtA5088-OtA5092	OtA5093-OtA5097
Fam59b	chr5:30103583-30154980	OtA5088-OtA5092	OtA5093-OtA5097
Fam5b	chr1:158245268-158356260	OtA5088-OtA5092	OtA5093-OtA5097
Fam5c	chr1:146495665-146902472	OtA5088-OtA5092	OtA5093-OtA5097
Fam64a	chr11:72042501-72047370	OtA5088-OtA5092	OtA5093-OtA5097
Fam69c	chr18:84720241-84740436	OtA5088-OtA5092	OtA5093-OtA5097
Fam70a	chrX:38197300-38252481	OtA5088-OtA5092	OtA5093-OtA5097
Fam70b	chr8:13435458-13461451	OtA5088-OtA5092	OtA5093-OtA5097
Fam71a	chr1:191162583-191164817	OtA5088-OtA5092	OtA5093-OtA5097
Fam71b	chr11:46404729-46407982	OtA5088-OtA5092	OtA5093-OtA5097
Fam71d	chr12:78691534-78734519	OtA5088-OtA5092	OtA5093-OtA5097
Fam71e1	chr7:44496587-44501134	OtA5088-OtA5092	OtA5093-OtA5097
Fam71e2	chr7:4753225-4771270	OtA5088-OtA5092	OtA5093-OtA5097
Fam71f1	chr6:29319139-29336022	OtA5088-OtA5092	OtA5093-OtA5097
Fam71f2	chr6:29281140-29290676	OtA5088-OtA5092	OtA5093-OtA5097
Fam72a	chr1:131527988-131539872	OtA5088-OtA5092	OtA5093-OtA5097
Fam75d3	chr13:59700082-59706197	OtA5088-OtA5092	OtA5093-OtA5097
Fam81a	chr9:70089309-70141557	OtA5088-OtA5092	OtA5093-OtA5097
Fam83a	chr15:57985902-58010702	OtA5088-OtA5092	OtA5093-OtA5097
Fam83c	chr2:155829182-155834854	OtA5088-OtA5092	OtA5093-OtA5097
Fam84a	chr12:14147597-14152038	OtA5088-OtA5092	OtA5093-OtA5097
Fam92b	chr8:120166396-120177469	OtA5088-OtA5092	OtA5093-OtA5097
Fancb	chrX:164980591-164997272	OtA5088-OtA5092	OtA5093-OtA5097
Fancd2	chr6:113531681-113596285	OtA5088-OtA5092	OtA5093-OtA5097
Fanci	chr7:79392337-79466273	OtA5088-OtA5092	OtA5093-OtA5097
Fancm	chr12:65075605-65132058	OtA5088-OtA5092	OtA5093-OtA5097
Fank1	chr7:133776890-133881532	OtA5088-OtA5092	OtA5093-OtA5097
Fap	chr2:62500944-62574021	OtA5088-OtA5092	OtA5093-OtA5097
Far2	chr6:148047415-148444352	OtA5088-OtA5092	OtA5093-OtA5097
Fasl	chr1:161780691-161788495	OtA5088-OtA5092	OtA5093-OtA5097
Fat2	chr11:55250609-55312257	OtA5088-OtA5092	OtA5093-OtA5097
Fat3	chr9:15910192-16378231	OtA5088-OtA5092	OtA5093-OtA5097

Fate1	chrX:71972985-71989046	OtA5088-OtA5092	OtA5093-OtA5097
Fbll1	chr11:35797379-35798884	OtA5088-OtA5092	OtA5093-OtA5097
Fbp1	chr13:62864752-62888236	OtA5088-OtA5092	OtA5093-OtA5097
Fbxl13	chr5:21483846-21645605	OtA5088-OtA5092	OtA5093-OtA5097
Fbxl16	chr17:25809084-25821265	OtA5088-OtA5092	OtA5093-OtA5097
Fbxl22	chr9:66350449-66514593	OtA5088-OtA5092	OtA5093-OtA5097
Fbxo15	chr18:84935024-84981392	OtA5088-OtA5092	OtA5093-OtA5097
Fbxo16	chr14:65266700-65321502	OtA5088-OtA5092	OtA5093-OtA5097
Fbxo17	chr7:28716789-28738140	OtA5088-OtA5092	OtA5093-OtA5097
Fbxo2	chr4:148160667-148166417	OtA5088-OtA5092	OtA5093-OtA5097
Fbxo24	chr5:137612504-137625078	OtA5088-OtA5092	OtA5093-OtA5097
Fbxo39	chr11:72314443-72319419	OtA5088-OtA5092	OtA5093-OtA5097
Fbxo40	chr16:36966072-36979136	OtA5088-OtA5092	OtA5093-OtA5097
Fbxo41	chr6:85469577-85502886	OtA5088-OtA5092	OtA5093-OtA5097
Fbxo43	chr15:36150059-36164884	OtA5088-OtA5092	OtA5093-OtA5097
Fbxo47	chr11:97854306-97884154	OtA5088-OtA5092	OtA5093-OtA5097
Fbxo48	chr11:16951409-16954772	OtA5088-OtA5092	OtA5093-OtA5097
Fbxo5	chr10:5799157-5805465	OtA5088-OtA5092	OtA5093-OtA5097
Fbxw10	chr11:62847122-62877462	OtA5088-OtA5092	OtA5093-OtA5097
Fbxw13	chr9:109179226-109195975	OtA5088-OtA5092	OtA5093-OtA5097
Fbxw14	chr9:109271124-109287676	OtA5088-OtA5092	OtA5093-OtA5097
Fbxw15	chr9:109552601-109568262	OtA5088-OtA5092	OtA5093-OtA5097
Fbxw16	chr9:109432317-109449140	OtA5088-OtA5092	OtA5093-OtA5097
Fbxw18	chr9:109676733-109702700	OtA5088-OtA5092	OtA5093-OtA5097
Fbxw19	chr9:109478574-109495854	OtA5088-OtA5092	OtA5093-OtA5097
Fbxw20	chr9:109217431-109234724	OtA5088-OtA5092	OtA5093-OtA5097
Fbxw21	chr9:109139453-109162022	OtA5088-OtA5092	OtA5093-OtA5097
Fbxw22	chr9:109378408-109404294	OtA5088-OtA5092	OtA5093-OtA5097
Fbxw24	chr9:109601115-109626059	OtA5088-OtA5092	OtA5093-OtA5097
Fbxw26	chr9:109717565-109746089	OtA5088-OtA5092	OtA5093-OtA5097
Fbxw28	chr9:109322885-109339659	OtA5088-OtA5092	OtA5093-OtA5097
Fcamr	chr1:130800901-130814740	OtA5088-OtA5092	OtA5093-OtA5097
Fcer1a	chr1:173221270-173227229	OtA5088-OtA5092	OtA5093-OtA5097
Fcer2a	chr8:3681736-3694174	OtA5088-OtA5092	OtA5093-OtA5097
Fcho1	chr8:71708386-71725681	OtA5088-OtA5092	OtA5093-OtA5097
Fcnb	chr2:28076478-28084878	OtA5088-OtA5092	OtA5093-OtA5097
Fcrl1	chr3:87376386-87392133	OtA5088-OtA5092	OtA5093-OtA5097
Fcrl5	chr3:87435781-87500678	OtA5088-OtA5092	OtA5093-OtA5097
Fcrl6	chr1:172596639-172602551	OtA5088-OtA5092	OtA5093-OtA5097
Fcrla	chr1:170917593-170927583	OtA5088-OtA5092	OtA5093-OtA5097
Fcrlb	chr1:170907272-170912941	OtA5088-OtA5092	OtA5093-OtA5097
Fer1l4	chr2:156019139-156052947	OtA5088-OtA5092	OtA5093-OtA5097
Ferd3l	chr12:33928424-33929309	OtA5088-OtA5092	OtA5093-OtA5097
Fermt1	chr2:132904178-132946036	OtA5088-OtA5092	OtA5093-OtA5097
Fev	chr1:74881508-74885408	OtA5088-OtA5092	OtA5093-OtA5097
Fezf1	chr6:23245046-23248264	OtA5088-OtA5092	OtA5093-OtA5097
Fezf2	chr14:12341891-12345865	OtA5088-OtA5092	OtA5093-OtA5097

Ffar1	chr7:30860567-30861470	OtA5088-OtA5092	OtA5093-OtA5097
Ffar2	chr7:30818356-30823775	OtA5088-OtA5092	OtA5093-OtA5097
Ffar3	chr7:30854329-30856178	OtA5088-OtA5092	OtA5093-OtA5097
Fgd2	chr17:29360913-29379535	OtA5088-OtA5092	OtA5093-OtA5097
Fgf1	chr18:38838672-38971274	OtA5088-OtA5092	OtA5093-OtA5097
Fgf10	chr13:118714698-118792573	OtA5088-OtA5092	OtA5093-OtA5097
Fgf11	chr11:69798201-69801625	OtA5088-OtA5092	OtA5093-OtA5097
Fgf12	chr16:28160097-28753202	OtA5088-OtA5092	OtA5093-OtA5097
Fgf13	chrX:59062149-59134436	OtA5088-OtA5092	OtA5093-OtA5097
Fgf14	chr14:123978290-124677127	OtA5088-OtA5092	OtA5093-OtA5097
Fgf15	chr7:144896531-144900951	OtA5088-OtA5092	OtA5093-OtA5097
Fgf16	chrX:105764476-105776532	OtA5088-OtA5092	OtA5093-OtA5097
Fgf17	chr14:70636204-70642268	OtA5088-OtA5092	OtA5093-OtA5097
Fgf18	chr11:33117430-33147400	OtA5088-OtA5092	OtA5093-OtA5097
Fgf20	chr8:40279165-40286953	OtA5088-OtA5092	OtA5093-OtA5097
Fgf21	chr7:45613889-45615490	OtA5088-OtA5092	OtA5093-OtA5097
Fgf22	chr10:79697304-80369637	OtA5088-OtA5092	OtA5093-OtA5097
Fgf23	chr6:127072901-127081408	OtA5088-OtA5092	OtA5093-OtA5097
Fgf3	chr7:144838611-144843348	OtA5088-OtA5092	OtA5093-OtA5097
Fgf4	chr7:144861385-144865243	OtA5088-OtA5092	OtA5093-OtA5097
Fgf5	chr5:98254183-98277030	OtA5088-OtA5092	OtA5093-OtA5097
Fgf6	chr6:127015541-127024718	OtA5088-OtA5092	OtA5093-OtA5097
Fgf7	chr2:125859217-126146251	OtA5088-OtA5092	OtA5093-OtA5097
Fgf8	chr19:45736797-45742884	OtA5088-OtA5092	OtA5093-OtA5097
Fgf9	chr14:58072685-58112720	OtA5088-OtA5092	OtA5093-OtA5097
Fgfbp1	chr5:43978860-43981762	OtA5088-OtA5092	OtA5093-OtA5097
Fgl1	chr8:41191433-41215156	OtA5088-OtA5092	OtA5093-OtA5097
Fgr	chr4:132974094-133001882	OtA5088-OtA5092	OtA5093-OtA5097
Fhad1	chr4:141890850-142011637	OtA5088-OtA5092	OtA5093-OtA5097
Fhl4	chr10:85097018-85102495	OtA5088-OtA5092	OtA5093-OtA5097
Fhl5	chr4:25199908-25242876	OtA5088-OtA5092	OtA5093-OtA5097
Fibcd1	chr2:31813289-31846005	OtA5088-OtA5092	OtA5093-OtA5097
Fibin	chr2:110360924-110362993	OtA5088-OtA5092	OtA5093-OtA5097
Figla	chr6:86017190-86020996	OtA5088-OtA5092	OtA5093-OtA5097
Fign	chr2:63972573-64098012	OtA5088-OtA5092	OtA5093-OtA5097
Fignl1	chr11:11800287-11808962	OtA5088-OtA5092	OtA5093-OtA5097
Filip1	chr9:79815561-79977882	OtA5088-OtA5092	OtA5093-OtA5097
Filip1l	chr16:57301999-57606867	OtA5088-OtA5092	OtA5093-OtA5097
Fitm1	chr14:55575673-55576952	OtA5088-OtA5092	OtA5093-OtA5097
Flg2	chr3:93197272-93221376	OtA5088-OtA5092	OtA5093-OtA5097
Flrt1	chr19:7056767-7198062	OtA5088-OtA5092	OtA5093-OtA5097
Flt3l	chr7:45131188-45136432	OtA5088-OtA5092	OtA5093-OtA5097
Flywch2	chr17:23776917-23786077	OtA5088-OtA5092	OtA5093-OtA5097
Fmn1	chr2:113220186-113716767	OtA5088-OtA5092	OtA5093-OtA5097
Fmn2	chr1:174501824-174822729	OtA5088-OtA5092	OtA5093-OtA5097
Fmo3	chr1:162953799-162984491	OtA5088-OtA5092	OtA5093-OtA5097
Fmo4	chr1:162793882-162812549	OtA5088-OtA5092	OtA5093-OtA5097

Fmo6	chr1:162916550-162937225	OtA5088-OtA5092	OtA5093-OtA5097
Fmo9	chr1:166662054-166681845	OtA5088-OtA5092	OtA5093-OtA5097
Fn3k	chr11:121434952-121450490	OtA5088-OtA5092	OtA5093-OtA5097
Fndc1	chr17:7738567-7804974	OtA5088-OtA5092	OtA5093-OtA5097
Fndc7	chr3:108853677-108890008	OtA5088-OtA5092	OtA5093-OtA5097
Fndc8	chr11:82892144-82900737	OtA5088-OtA5092	OtA5093-OtA5097
Folh1	chr7:86718975-86775864	OtA5088-OtA5092	OtA5093-OtA5097
Folr4	chr9:14885813-14903951	OtA5088-OtA5092	OtA5093-OtA5097
Foxa1	chr12:57540631-57546121	OtA5088-OtA5092	OtA5093-OtA5097
Foxb1	chr9:69757709-69760940	OtA5088-OtA5092	OtA5093-OtA5097
Foxb2	chr19:16872315-16873830	OtA5088-OtA5092	OtA5093-OtA5097
Foxc1	chr13:31806645-31810635	OtA5088-OtA5092	OtA5093-OtA5097
Foxc2	chr8:121116170-121118894	OtA5088-OtA5092	OtA5093-OtA5097
Foxd1	chr13:98354244-98356705	OtA5088-OtA5092	OtA5093-OtA5097
Foxd2	chr4:114906279-114908898	OtA5088-OtA5092	OtA5093-OtA5097
Foxd3	chr4:99656298-99658671	OtA5088-OtA5092	OtA5093-OtA5097
Foxd4	chr19:24898964-24901309	OtA5088-OtA5092	OtA5093-OtA5097
Foxe1	chr4:46344193-46345309	OtA5088-OtA5092	OtA5093-OtA5097
Foxe3	chr4:114925146-114926013	OtA5088-OtA5092	OtA5093-OtA5097
Foxg1	chr12:49382882-49386867	OtA5088-OtA5092	OtA5093-OtA5097
Foxh1	chr15:76668306-76669873	OtA5088-OtA5092	OtA5093-OtA5097
Foxi1	chr11:34204340-34208089	OtA5088-OtA5092	OtA5093-OtA5097
Foxi2	chr7:135410366-135413615	OtA5088-OtA5092	OtA5093-OtA5097
Foxi3	chr6:70956605-70961066	OtA5088-OtA5092	OtA5093-OtA5097
Foxj1	chr11:116330703-116335354	OtA5088-OtA5092	OtA5093-OtA5097
Foxl1	chr8:121127684-121130644	OtA5088-OtA5092	OtA5093-OtA5097
Foxl2	chr9:98955606-98958126	OtA5088-OtA5092	OtA5093-OtA5097
Foxl2os	chr9:98949154-98955310	OtA5088-OtA5092	OtA5093-OtA5097
Foxm1	chr6:128362993-128385144	OtA5088-OtA5092	OtA5093-OtA5097
Foxn1	chr11:78358287-78386558	OtA5088-OtA5092	OtA5093-OtA5097
Foxn4	chr5:114254163-114273761	OtA5088-OtA5092	OtA5093-OtA5097
Foxo6	chr4:120267077-120287261	OtA5088-OtA5092	OtA5093-OtA5097
Foxp2	chr6:14901348-15441977	OtA5088-OtA5092	OtA5093-OtA5097
Foxp3	chrX:7558561-7605420	OtA5088-OtA5092	OtA5093-OtA5097
Foxr1	chr9:44434233-44440868	OtA5088-OtA5092	OtA5093-OtA5097
Foxr2	chrX:153118787-153132861	OtA5088-OtA5092	OtA5093-OtA5097
Foxred2	chr15:77940521-77956722	OtA5088-OtA5092	OtA5093-OtA5097
Fpr-rs3	chr17:20623845-20624877	OtA5088-OtA5092	OtA5093-OtA5097
Fpr-rs4	chr17:18021732-18022704	OtA5088-OtA5092	OtA5093-OtA5097
Fpr-rs6	chr17:20182077-20183097	OtA5088-OtA5092	OtA5093-OtA5097
Fpr1	chr17:17876470-17883939	OtA5088-OtA5092	OtA5093-OtA5097
Fpr2	chr17:17887823-17893952	OtA5088-OtA5092	OtA5093-OtA5097
Fpr3	chr17:17970457-17971677	OtA5088-OtA5092	OtA5093-OtA5097
Frem3	chr8:80611038-80695557	OtA5088-OtA5092	OtA5093-OtA5097
Frmd3	chr4:74013602-74202214	OtA5088-OtA5092	OtA5093-OtA5097
Frmd5	chr2:121545528-121807057	OtA5088-OtA5092	OtA5093-OtA5097
Frmd7	chrX:50892644-50942710	OtA5088-OtA5092	OtA5093-OtA5097

Frmpd3	chrX:140381970-140394540	OtA5088-OtA5092	OtA5093-OtA5097
Frmpd4	chrX:167471308-168577233	OtA5088-OtA5092	OtA5093-OtA5097
Frzb	chr2:80411969-80447396	OtA5088-OtA5092	OtA5093-OtA5097
Fsbp	chr4:11558919-11615808	OtA5088-OtA5092	OtA5093-OtA5097
Fscb	chr12:64471332-64474898	OtA5088-OtA5092	OtA5093-OtA5097
Fscn2	chr11:120361533-120368173	OtA5088-OtA5092	OtA5093-OtA5097
Fscn3	chr6:28427900-28438622	OtA5088-OtA5092	OtA5093-OtA5097
Fsd1	chr17:55986510-55996881	OtA5088-OtA5092	OtA5093-OtA5097
Fsd2	chr7:81534353-81566981	OtA5088-OtA5092	OtA5093-OtA5097
Fshb	chr2:107056140-107059651	OtA5088-OtA5092	OtA5093-OtA5097
Fshr	chr17:88984951-89200675	OtA5088-OtA5092	OtA5093-OtA5097
Fsip1	chr2:118204887-118256966	OtA5088-OtA5092	OtA5093-OtA5097
Fstl4	chr11:52764705-53187347	OtA5088-OtA5092	OtA5093-OtA5097
Fstl5	chr3:76074560-76710005	OtA5088-OtA5092	OtA5093-OtA5097
Ftcd	chr10:76575647-76590338	OtA5088-OtA5092	OtA5093-OtA5097
Fthl17	chrX:9033485-9034333	OtA5088-OtA5092	OtA5093-OtA5097
Ftmt	chr18:52331535-52332996	OtA5088-OtA5092	OtA5093-OtA5097
Fundc2	chrX:75382398-75397158	OtA5088-OtA5092	OtA5093-OtA5097
Fut1	chr7:45617605-45621057	OtA5088-OtA5092	OtA5093-OtA5097
Fut2	chr7:45648594-45666308	OtA5088-OtA5092	OtA5093-OtA5097
Fut4-ps1	chr17:56734227-56739292	OtA5088-OtA5092	OtA5093-OtA5097
Fut7	chr2:25423693-25426373	OtA5088-OtA5092	OtA5093-OtA5097
Fut9	chr4:25609332-25800003	OtA5088-OtA5092	OtA5093-OtA5097
Fv1	chr4:147868978-147870358	OtA5088-OtA5092	OtA5093-OtA5097
Fxyd7	chr7:31042514-31051454	OtA5088-OtA5092	OtA5093-OtA5097
Fyb	chr15:6579870-6663312	OtA5088-OtA5092	OtA5093-OtA5097
Fzd10	chr5:128601105-128604093	OtA5088-OtA5092	OtA5093-OtA5097
Fzd3	chr14:65192440-65262463	OtA5088-OtA5092	OtA5093-OtA5097
Fzd9	chr5:135248937-135251047	OtA5088-OtA5092	OtA5093-OtA5097
G2e3	chr12:51348229-51376986	OtA5088-OtA5092	OtA5093-OtA5097
G530011O06Rik	chrX:169685246-169990797	OtA5088-OtA5092	OtA5093-OtA5097
G630008E18	chr5:119625941-119634462	OtA5088-OtA5092	OtA5093-OtA5097
G630025P09Rik	chr11:69803594-69806038	OtA5088-OtA5092	OtA5093-OtA5097
G630055G22Rik	chr18:47922840-48107980	OtA5088-OtA5092	OtA5093-OtA5097
G630071F17	chr18:60663409-60671624	OtA5088-OtA5092	OtA5093-OtA5097
G630093K05Rik	chr13:47910434-47934301	OtA5088-OtA5092	OtA5093-OtA5097
G6b	chr17:35062692-35066184	OtA5088-OtA5092	OtA5093-OtA5097
G6pc	chr11:101367729-101377903	OtA5088-OtA5092	OtA5093-OtA5097
G6pc2	chr2:69219997-69227988	OtA5088-OtA5092	OtA5093-OtA5097
G6pd2	chr5:61808842-61810477	OtA5088-OtA5092	OtA5093-OtA5097
G730013B05Rik	chr16:50526244-50559459	OtA5088-OtA5092	OtA5093-OtA5097
Gab3	chrX:74988544-75084905	OtA5088-OtA5092	OtA5093-OtA5097
Gabbr2	chr4:46663897-46991714	OtA5088-OtA5092	OtA5093-OtA5097
Gabra1	chr11:42131479-42182930	OtA5088-OtA5092	OtA5093-OtA5097
Gabra2	chr5:70961056-71095849	OtA5088-OtA5092	OtA5093-OtA5097
Gabra3	chrX:72432675-72656246	OtA5088-OtA5092	OtA5093-OtA5097
Gabra4	chr5:71569733-71658308	OtA5088-OtA5092	OtA5093-OtA5097

Gabra5	chr7:57387271-57510009	OtA5088-OtA5092	OtA5093-OtA5097
Gabra6	chr11:42306436-42321051	OtA5088-OtA5092	OtA5093-OtA5097
Gabrb1	chr5:71700015-72137244	OtA5088-OtA5092	OtA5093-OtA5097
Gabrb2	chr11:42419756-42632591	OtA5088-OtA5092	OtA5093-OtA5097
Gabrb3	chr7:57590533-57828801	OtA5088-OtA5092	OtA5093-OtA5097
Gabre	chrX:72257431-72274721	OtA5088-OtA5092	OtA5093-OtA5097
Gabrg1	chr5:70751046-70842617	OtA5088-OtA5092	OtA5093-OtA5097
Gabrg2	chr11:41910194-42000678	OtA5088-OtA5092	OtA5093-OtA5097
Gabrg3	chr7:56724241-57386871	OtA5088-OtA5092	OtA5093-OtA5097
Gabrq	chrX:72825435-72838544	OtA5088-OtA5092	OtA5093-OtA5097
Gabrr1	chr4:33132555-33163588	OtA5088-OtA5092	OtA5093-OtA5097
Gabrr2	chr4:33063111-33095865	OtA5088-OtA5092	OtA5093-OtA5097
Gabrr3	chr16:59407381-59461739	OtA5088-OtA5092	OtA5093-OtA5097
Gad1	chr2:70489939-70602012	OtA5088-OtA5092	OtA5093-OtA5097
Gad2	chr2:22622326-22693877	OtA5088-OtA5092	OtA5093-OtA5097
Gadl1	chr9:115909454-116076176	OtA5088-OtA5092	OtA5093-OtA5097
Gal	chr19:3409916-3414457	OtA5088-OtA5092	OtA5093-OtA5097
Gal3st2	chr1:93861343-93876494	OtA5088-OtA5092	OtA5093-OtA5097
Gal3st3	chr19:5298330-5308739	OtA5088-OtA5092	OtA5093-OtA5097
Gal3st4	chr5:138259657-138272754	OtA5088-OtA5092	OtA5093-OtA5097
Galnt13	chr2:54436386-55117744	OtA5088-OtA5092	OtA5093-OtA5097
Galnt14	chr17:73493750-73710451	OtA5088-OtA5092	OtA5093-OtA5097
Galnt5	chr2:57998154-58039171	OtA5088-OtA5092	OtA5093-OtA5097
Galnt9	chr5:110544344-110621382	OtA5088-OtA5092	OtA5093-OtA5097
Galntl5	chr5:25181479-25220289	OtA5088-OtA5092	OtA5093-OtA5097
Galntl6	chr8:57774051-58911627	OtA5088-OtA5092	OtA5093-OtA5097
Galp	chr7:6197089-6216137	OtA5088-OtA5092	OtA5093-OtA5097
Galr1	chr18:82392495-82406777	OtA5088-OtA5092	OtA5093-OtA5097
Galr2	chr11:116280938-116283938	OtA5088-OtA5092	OtA5093-OtA5097
Galr3	chr15:79030873-79043558	OtA5088-OtA5092	OtA5093-OtA5097
Gap43	chr16:42248560-42340651	OtA5088-OtA5092	OtA5093-OtA5097
Gapdhs	chr7:30729886-30739356	OtA5088-OtA5092	OtA5093-OtA5097
Gapt	chr13:110352614-110357172	OtA5088-OtA5092	OtA5093-OtA5097
Garnl3	chr2:32986365-33087204	OtA5088-OtA5092	OtA5093-OtA5097
Gas2	chr7:51879144-51994458	OtA5088-OtA5092	OtA5093-OtA5097
Gas2l2	chr11:83421901-83429455	OtA5088-OtA5092	OtA5093-OtA5097
Gas2l3	chr10:89408822-89443967	OtA5088-OtA5092	OtA5093-OtA5097
Gast	chr11:100334411-100336996	OtA5088-OtA5092	OtA5093-OtA5097
Gata5	chr2:180325087-180340732	OtA5088-OtA5092	OtA5093-OtA5097
Gbp1	chr3:142594846-142619176	OtA5088-OtA5092	OtA5093-OtA5097
Gbp10	chr5:105215698-105239533	OtA5088-OtA5092	OtA5093-OtA5097
Gbp11	chr5:105323025-105346472	OtA5088-OtA5092	OtA5093-OtA5097
Gbp8	chr5:105014152-105053561	OtA5088-OtA5092	OtA5093-OtA5097
Gbx1	chr5:24504425-24526848	OtA5088-OtA5092	OtA5093-OtA5097
Gbx2	chr1:89927961-89931176	OtA5088-OtA5092	OtA5093-OtA5097
Gcet2	chr16:45610439-45622867	OtA5088-OtA5092	OtA5093-OtA5097
Gcg	chr2:62474529-62483653	OtA5088-OtA5092	OtA5093-OtA5097

Gck	chr11:5900822-5949599	OtA5088-OtA5092	OtA5093-OtA5097
Gckr	chr5:31297580-31327302	OtA5088-OtA5092	OtA5093-OtA5097
Gcm2	chr13:41101426-41109988	OtA5088-OtA5092	OtA5093-OtA5097
Gcnt3	chr9:70031495-70038088	OtA5088-OtA5092	OtA5093-OtA5097
Gcnt7	chr2:172440577-172469899	OtA5088-OtA5092	OtA5093-OtA5097
Gdap1	chr1:17145372-17164270	OtA5088-OtA5092	OtA5093-OtA5097
Gdap1l1	chr2:163438466-163455324	OtA5088-OtA5092	OtA5093-OtA5097
Gdf1	chr8:70315774-70353273	OtA5088-OtA5092	OtA5093-OtA5097
Gdf11	chr10:128884545-128891718	OtA5088-OtA5092	OtA5093-OtA5097
Gdf2	chr14:33941038-33947198	OtA5088-OtA5092	OtA5093-OtA5097
Gdf3	chr6:122605402-122610071	OtA5088-OtA5092	OtA5093-OtA5097
Gdf5	chr2:155941024-155945364	OtA5088-OtA5092	OtA5093-OtA5097
Gdf6	chr4:9844371-9862345	OtA5088-OtA5092	OtA5093-OtA5097
Gdf7	chr12:8297917-8301954	OtA5088-OtA5092	OtA5093-OtA5097
Gdf9	chr11:53433285-53437902	OtA5088-OtA5092	OtA5093-OtA5097
Gdnf	chr15:7811010-7837575	OtA5088-OtA5092	OtA5093-OtA5097
Gdpd1	chr11:87033793-87074137	OtA5088-OtA5092	OtA5093-OtA5097
Gdpd2	chrX:100729846-100738899	OtA5088-OtA5092	OtA5093-OtA5097
Gdpd3	chr7:126766413-126775645	OtA5088-OtA5092	OtA5093-OtA5097
Gdpd4	chr7:97919957-98049663	OtA5088-OtA5092	OtA5093-OtA5097
Gemin8	chrX:166170479-166190512	OtA5088-OtA5092	OtA5093-OtA5097
Gen1	chr12:11240925-11265787	OtA5088-OtA5092	OtA5093-OtA5097
Gfap	chr11:102887335-102897200	OtA5088-OtA5092	OtA5093-OtA5097
Gfi1	chr5:107716654-107724343	OtA5088-OtA5092	OtA5093-OtA5097
Gfi1b	chr2:28609449-28621982	OtA5088-OtA5092	OtA5093-OtA5097
Gfra1	chr19:58236691-58454466	OtA5088-OtA5092	OtA5093-OtA5097
Gfra2	chr14:70890129-70979838	OtA5088-OtA5092	OtA5093-OtA5097
Gfra4	chr2:131040175-131043088	OtA5088-OtA5092	OtA5093-OtA5097
Gfral	chr9:76164101-76213657	OtA5088-OtA5092	OtA5093-OtA5097
Ggct	chr6:54985094-54992867	OtA5088-OtA5092	OtA5093-OtA5097
Ggn	chr7:29170209-29173933	OtA5088-OtA5092	OtA5093-OtA5097
Ggnbp1	chr17:27018034-27036378	OtA5088-OtA5092	OtA5093-OtA5097
Ggt6	chr11:72435525-72438404	OtA5088-OtA5092	OtA5093-OtA5097
Ggt7	chr2:155490379-155514846	OtA5088-OtA5092	OtA5093-OtA5097
Gh	chr11:106300267-106301810	OtA5088-OtA5092	OtA5093-OtA5097
Ghrhr	chr6:55376294-55388530	OtA5088-OtA5092	OtA5093-OtA5097
Ghrl	chr6:113716118-113719880	OtA5088-OtA5092	OtA5093-OtA5097
Ghsr	chr3:27371350-27378010	OtA5088-OtA5092	OtA5093-OtA5097
Gif	chr19:11747558-11763447	OtA5088-OtA5092	OtA5093-OtA5097
Gimap3	chr6:48764463-48770851	OtA5088-OtA5092	OtA5093-OtA5097
Gimap5	chr6:48746196-48754200	OtA5088-OtA5092	OtA5093-OtA5097
Gimap7	chr6:48718620-48724636	OtA5088-OtA5092	OtA5093-OtA5097
Gimap8	chr6:48647233-48660875	OtA5088-OtA5092	OtA5093-OtA5097
Gimap9	chr6:48676134-48678704	OtA5088-OtA5092	OtA5093-OtA5097
Gin1	chr1:97770178-97792971	OtA5088-OtA5092	OtA5093-OtA5097
Gins1	chr2:150909593-150931280	OtA5088-OtA5092	OtA5093-OtA5097
Gip	chr11:96024544-96030828	OtA5088-OtA5092	OtA5093-OtA5097

Gipc3	chr10:81337761-81343266	OtA5088-OtA5092	OtA5093-OtA5097
Gipr	chr7:19157124-19166127	OtA5088-OtA5092	OtA5093-OtA5097
Gja10	chr4:32600864-32602382	OtA5088-OtA5092	OtA5093-OtA5097
Gja3	chr14:57035659-57057945	OtA5088-OtA5092	OtA5093-OtA5097
Gja6	chrX:160768012-160994681	OtA5088-OtA5092	OtA5093-OtA5097
Gja8	chr3:96919021-96925930	OtA5088-OtA5092	OtA5093-OtA5097
Gjb6	chr14:57123302-57133577	OtA5088-OtA5092	OtA5093-OtA5097
Gjc2	chr11:59175563-59183213	OtA5088-OtA5092	OtA5093-OtA5097
Gjc3	chr5:137953808-137962959	OtA5088-OtA5092	OtA5093-OtA5097
Gjd2	chr2:114009600-114032292	OtA5088-OtA5092	OtA5093-OtA5097
Gjd3	chr11:98982179-98983016	OtA5088-OtA5092	OtA5093-OtA5097
Gjd4	chr18:9278606-9282809	OtA5088-OtA5092	OtA5093-OtA5097
Gje1	chr10:14705608-14759019	OtA5088-OtA5092	OtA5093-OtA5097
Gk2	chr5:97392416-97588126	OtA5088-OtA5092	OtA5093-OtA5097
Gkn3	chr6:87383318-87388935	OtA5088-OtA5092	OtA5093-OtA5097
Glb1l2	chr9:26763043-26806417	OtA5088-OtA5092	OtA5093-OtA5097
Glb1l3	chr9:26817952-26860823	OtA5088-OtA5092	OtA5093-OtA5097
Gldn	chr9:54268579-54341777	OtA5088-OtA5092	OtA5093-OtA5097
Gli1	chr10:127323726-127341579	OtA5088-OtA5092	OtA5093-OtA5097
Gli2	chr1:118834060-119053619	OtA5088-OtA5092	OtA5093-OtA5097
Glipr1l1	chr10:112060188-112078510	OtA5088-OtA5092	OtA5093-OtA5097
Glipr1l2	chr10:112083353-112108098	OtA5088-OtA5092	OtA5093-OtA5097
Glmn	chr5:107534710-107661838	OtA5088-OtA5092	OtA5093-OtA5097
Glod5	chrX:8004200-8018492	OtA5088-OtA5092	OtA5093-OtA5097
Glp2r	chr11:67706429-67771153	OtA5088-OtA5092	OtA5093-OtA5097
Glra1	chr11:55514812-55607790	OtA5088-OtA5092	OtA5093-OtA5097
Glra2	chrX:165129016-165326981	OtA5088-OtA5092	OtA5093-OtA5097
Glra3	chr8:55940824-56125352	OtA5088-OtA5092	OtA5093-OtA5097
Glra4	chrX:136732947-136803361	OtA5088-OtA5092	OtA5093-OtA5097
Glrb	chr3:80843603-80913624	OtA5088-OtA5092	OtA5093-OtA5097
Glrp1	chr1:88499870-88510066	OtA5088-OtA5092	OtA5093-OtA5097
Glt1d1	chr5:127632261-127707520	OtA5088-OtA5092	OtA5093-OtA5097
Glt25d2	chr1:152399866-152510695	OtA5088-OtA5092	OtA5093-OtA5097
Glt6d1	chr2:25793966-25815836	OtA5088-OtA5092	OtA5093-OtA5097
Glt8d2	chr10:82629837-82690650	OtA5088-OtA5092	OtA5093-OtA5097
Gltpd2	chr11:70519208-70520736	OtA5088-OtA5092	OtA5093-OtA5097
Glyat	chr19:12633307-12651737	OtA5088-OtA5092	OtA5093-OtA5097
Glyatl3	chr17:40904740-40914350	OtA5088-OtA5092	OtA5093-OtA5097
Glycam1	chr15:103562761-103565078	OtA5088-OtA5092	OtA5093-OtA5097
Gm10007	chr19:58236691-58454466	OtA5088-OtA5092	OtA5093-OtA5097
Gm10012	chr17:25882113-25919714	OtA5088-OtA5092	OtA5093-OtA5097
Gm10024	chr10:77711456-77712009	OtA5088-OtA5092	OtA5093-OtA5097
Gm10046	chr7:27765672-27770972	OtA5088-OtA5092	OtA5093-OtA5097
Gm10057	chrX:154043985-154055041	OtA5088-OtA5092	OtA5093-OtA5097
Gm10058,Gm10096,Gr	chrX:35404186-35427093	OtA5088-OtA5092	OtA5093-OtA5097
Gm10069	chr6:128438756-128526720	OtA5088-OtA5092	OtA5093-OtA5097
Gm10081	chr7:107105911-107106859	OtA5088-OtA5092	OtA5093-OtA5097

Gm10096,Gm10486	chrX:35404186-35427093	OtA5088-OtA5092	OtA5093-OtA5097
Gm101	chr1:119923518-119995210	OtA5088-OtA5092	OtA5093-OtA5097
Gm10100	chr10:77726485-77726848	OtA5088-OtA5092	OtA5093-OtA5097
Gm10104	chr8:21065037-21089644	OtA5088-OtA5092	OtA5093-OtA5097
Gm10125	chr18:5491500-5775468	OtA5088-OtA5092	OtA5093-OtA5097
Gm10142	chr10:77715806-77716169	OtA5088-OtA5092	OtA5093-OtA5097
Gm10147	chrX:35404186-35427093	OtA5088-OtA5092	OtA5093-OtA5097
Gm10190	chr17:80307406-80388689	OtA5088-OtA5092	OtA5093-OtA5097
Gm10220	chr5:26114763-26121425	OtA5088-OtA5092	OtA5093-OtA5097
Gm10229	chr16:89015276-89015846	OtA5088-OtA5092	OtA5093-OtA5097
Gm10230	chrX:30820524-30843469	OtA5088-OtA5092	OtA5093-OtA5097
Gm10248	chr14:22019711-23094571	OtA5088-OtA5092	OtA5093-OtA5097
Gm10267	chr18:44156403-44159303	OtA5088-OtA5092	OtA5093-OtA5097
Gm10272	chr10:77706586-77706986	OtA5088-OtA5092	OtA5093-OtA5097
Gm10280	chr8:113067261-113093333	OtA5088-OtA5092	OtA5093-OtA5097
Gm10318	chr10:77852858-77853788	OtA5088-OtA5092	OtA5093-OtA5097
Gm10319	chr6:122136627-122150958	OtA5088-OtA5092	OtA5093-OtA5097
Gm10324	chr13:66113452-66122836	OtA5088-OtA5092	OtA5093-OtA5097
Gm10334	chr6:41442213-41446097	OtA5088-OtA5092	OtA5093-OtA5097
Gm10336	chr13:12182716-12186488	OtA5088-OtA5092	OtA5093-OtA5097
Gm10345	chr15:76294433-76307245	OtA5088-OtA5092	OtA5093-OtA5097
Gm10373	chr15:43430942-43477036	OtA5088-OtA5092	OtA5093-OtA5097
Gm10375	chr14:43602626-43608013	OtA5088-OtA5092	OtA5093-OtA5097
Gm10377	chr14:43010187-43015576	OtA5088-OtA5092	OtA5093-OtA5097
Gm10389	chr15:10568977-10714631	OtA5088-OtA5092	OtA5093-OtA5097
Gm10390	chr5:120116512-120198971	OtA5088-OtA5092	OtA5093-OtA5097
Gm10391	chr14:26262257-26269434	OtA5088-OtA5092	OtA5093-OtA5097
Gm10394	chr14:25983004-25990166	OtA5088-OtA5092	OtA5093-OtA5097
Gm10400	chr6:141249268-141499351	OtA5088-OtA5092	OtA5093-OtA5097
Gm10406	chr14:7006114-7027449	OtA5088-OtA5092	OtA5093-OtA5097
Gm10408	chr14:3688718-3698681	OtA5088-OtA5092	OtA5093-OtA5097
Gm10413	chr14:3634511-3642952	OtA5088-OtA5092	OtA5093-OtA5097
Gm10415	chr6:126286648-126328686	OtA5088-OtA5092	OtA5093-OtA5097
Gm10416	chr5:109833500-109834142	OtA5088-OtA5092	OtA5093-OtA5097
Gm10421	chr12:116485719-117337012	OtA5088-OtA5092	OtA5093-OtA5097
Gm10432	chr12:100276608-100520822	OtA5088-OtA5092	OtA5093-OtA5097
Gm10433	chr12:100187967-100193538	OtA5088-OtA5092	OtA5093-OtA5097
Gm10436	chr12:88175588-88181894	OtA5088-OtA5092	OtA5093-OtA5097
Gm10439	chrX:149594600-149636621	OtA5088-OtA5092	OtA5093-OtA5097
Gm10440	chr5:54349991-54358542	OtA5088-OtA5092	OtA5093-OtA5097
Gm10445	chr6:84412941-84423838	OtA5088-OtA5092	OtA5093-OtA5097
Gm1045	chr5:91987472-91995317	OtA5088-OtA5092	OtA5093-OtA5097
Gm10451	chr12:76444496-76448698	OtA5088-OtA5092	OtA5093-OtA5097
Gm10466	chr11:24721005-24730722	OtA5088-OtA5092	OtA5093-OtA5097
Gm10471	chr5:26082171-26089264	OtA5088-OtA5092	OtA5093-OtA5097
Gm10474	chrX:68667513-68678399	OtA5088-OtA5092	OtA5093-OtA5097
Gm10485	chr12:3806979-3914443	OtA5088-OtA5092	OtA5093-OtA5097

Gm10486	chrX:35404186-35427093	OtA5088-OtA5092	OtA5093-OtA5097
Gm10487	chrX:35067383-35090316	OtA5088-OtA5092	OtA5093-OtA5097
Gm10488	chrX:35404186-35427093	OtA5088-OtA5092	OtA5093-OtA5097
Gm10494	chr17:79506048-79510486	OtA5088-OtA5092	OtA5093-OtA5097
Gm10509	chr17:21690211-21692213	OtA5088-OtA5092	OtA5093-OtA5097
Gm10510	chr17:15350812-15354461	OtA5088-OtA5092	OtA5093-OtA5097
Gm10512	chr17:13205041-13206211	OtA5088-OtA5092	OtA5093-OtA5097
Gm10516	chr1:192100461-192151025	OtA5088-OtA5092	OtA5093-OtA5097
Gm10536	chr18:56936259-56940518	OtA5088-OtA5092	OtA5093-OtA5097
Gm10538	chr1:132564689-132741797	OtA5088-OtA5092	OtA5093-OtA5097
Gm10548	chr18:34207774-34322190	OtA5088-OtA5092	OtA5093-OtA5097
Gm10549	chr18:33464162-33474710	OtA5088-OtA5092	OtA5093-OtA5097
Gm10556	chr18:4812485-4850959	OtA5088-OtA5092	OtA5093-OtA5097
Gm10560	chr4:156021644-156023824	OtA5088-OtA5092	OtA5093-OtA5097
Gm10565	chr4:142030992-142088101	OtA5088-OtA5092	OtA5093-OtA5097
Gm10578	chr7:137193670-137314445	OtA5088-OtA5092	OtA5093-OtA5097
Gm10584	chr7:132236254-132318291	OtA5088-OtA5092	OtA5093-OtA5097
Gm106	chr1:15853861-15892722	OtA5088-OtA5092	OtA5093-OtA5097
Gm10635	chr9:79444036-79519302	OtA5088-OtA5092	OtA5093-OtA5097
Gm10636	chr3:146367152-146378944	OtA5088-OtA5092	OtA5093-OtA5097
Gm10637	chr8:87169746-87199850	OtA5088-OtA5092	OtA5093-OtA5097
Gm10638	chr8:86723937-86747060	OtA5088-OtA5092	OtA5093-OtA5097
Gm10639	chr9:78289927-78305525	OtA5088-OtA5092	OtA5093-OtA5097
Gm10640	chr7:31162357-31170641	OtA5088-OtA5092	OtA5093-OtA5097
Gm10649	chr8:76749086-76900173	OtA5088-OtA5092	OtA5093-OtA5097
Gm10653	chr9:62838786-62875917	OtA5088-OtA5092	OtA5093-OtA5097
Gm10658	chr9:56994967-57071966	OtA5088-OtA5092	OtA5093-OtA5097
Gm10662,Gm5891	chr7:23132622-23144480	OtA5088-OtA5092	OtA5093-OtA5097
Gm10665	chr7:22463109-22464075	OtA5088-OtA5092	OtA5093-OtA5097
Gm10666	chr7:22133775-22134741	OtA5088-OtA5092	OtA5093-OtA5097
Gm10677	chr9:47530351-47853385	OtA5088-OtA5092	OtA5093-OtA5097
Gm10684	chr9:45079182-45135606	OtA5088-OtA5092	OtA5093-OtA5097
Gm10696	chr3:94174411-94178193	OtA5088-OtA5092	OtA5093-OtA5097
Gm10697	chr3:94071621-94072644	OtA5088-OtA5092	OtA5093-OtA5097
Gm10714	chr2:174125037-174183918	OtA5088-OtA5092	OtA5093-OtA5097
Gm10731	chr3:40846998-40875609	OtA5088-OtA5092	OtA5093-OtA5097
Gm10745	chr3:11823228-11844359	OtA5088-OtA5092	OtA5093-OtA5097
Gm10754	chr10:97644067-97967090	OtA5088-OtA5092	OtA5093-OtA5097
Gm10767	chr13:66905414-66909166	OtA5088-OtA5092	OtA5093-OtA5097
Gm10768	chr19:43838802-43840845	OtA5088-OtA5092	OtA5093-OtA5097
Gm10778	chr10:81812185-81826490	OtA5088-OtA5092	OtA5093-OtA5097
Gm1078	chr7:4965259-4970961	OtA5088-OtA5092	OtA5093-OtA5097
Gm10782	chr13:56362899-56368857	OtA5088-OtA5092	OtA5093-OtA5097
Gm10785	chr16:91688897-91728802	OtA5088-OtA5092	OtA5093-OtA5097
Gm10787	chr10:77011994-77022214	OtA5088-OtA5092	OtA5093-OtA5097
Gm10789	chr16:90142236-90146827	OtA5088-OtA5092	OtA5093-OtA5097
Gm10790	chr13:41865914-41913092	OtA5088-OtA5092	OtA5093-OtA5097

Gm10791	chr16:84954435-85173707	OtA5088-OtA5092	OtA5093-OtA5097
Gm10804	chr2:93419111-93469874	OtA5088-OtA5092	OtA5093-OtA5097
Gm10814	chr19:5988544-6018459	OtA5088-OtA5092	OtA5093-OtA5097
Gm10818	chr16:32644642-32683493	OtA5088-OtA5092	OtA5093-OtA5097
Gm1082	chr7:30515121-30522193	OtA5088-OtA5092	OtA5093-OtA5097
Gm10823	chr16:27849929-27926128	OtA5088-OtA5092	OtA5093-OtA5097
Gm10825	chr10:22402812-22407470	OtA5088-OtA5092	OtA5093-OtA5097
Gm10845	chr14:79860520-79869176	OtA5088-OtA5092	OtA5093-OtA5097
Gm10857	chr2:6132868-6140570	OtA5088-OtA5092	OtA5093-OtA5097
Gm10863	chr15:79166065-79216401	OtA5088-OtA5092	OtA5093-OtA5097
Gm10865	chr15:78947723-79011041	OtA5088-OtA5092	OtA5093-OtA5097
Gm10872	chr15:76266786-76292572	OtA5088-OtA5092	OtA5093-OtA5097
Gm10921	chrX:31381958-31383918	OtA5088-OtA5092	OtA5093-OtA5097
Gm10922	chrX:4370635-4372595	OtA5088-OtA5092	OtA5093-OtA5097
Gm10941	chr10:77257772-77259223	OtA5088-OtA5092	OtA5093-OtA5097
Gm11062	chr1:173274460-173277756	OtA5088-OtA5092	OtA5093-OtA5097
Gm1110	chr9:26879566-26923081	OtA5088-OtA5092	OtA5093-OtA5097
Gm11110	chr17:57092022-57107757	OtA5088-OtA5092	OtA5093-OtA5097
Gm11128	chr6:85808050-85809877	OtA5088-OtA5092	OtA5093-OtA5097
Gm11149	chr9:49502145-49799069	OtA5088-OtA5092	OtA5093-OtA5097
Gm11166	chr17:13096659-13098472	OtA5088-OtA5092	OtA5093-OtA5097
Gm11186	chr11:52764705-53187347	OtA5088-OtA5092	OtA5093-OtA5097
Gm11190	chr11:77912126-77929288	OtA5088-OtA5092	OtA5093-OtA5097
Gm11194	chr11:78394484-78422185	OtA5088-OtA5092	OtA5093-OtA5097
Gm11201	chr11:79014800-79146354	OtA5088-OtA5092	OtA5093-OtA5097
Gm11202	chr11:79591211-79694012	OtA5088-OtA5092	OtA5093-OtA5097
Gm11213	chr4:63596900-63605261	OtA5088-OtA5092	OtA5093-OtA5097
Gm1123	chr9:99006964-99035690	OtA5088-OtA5092	OtA5093-OtA5097
Gm11240	chr4:73791243-73798378	OtA5088-OtA5092	OtA5093-OtA5097
Gm11346	chr13:24598124-24604767	OtA5088-OtA5092	OtA5093-OtA5097
Gm11351	chr13:25921034-25966316	OtA5088-OtA5092	OtA5093-OtA5097
Gm1140,Gm14692	chrX:67695697-67706350	OtA5088-OtA5092	OtA5093-OtA5097
Gm1141	chrX:71932437-71940871	OtA5088-OtA5092	OtA5093-OtA5097
Gm11413	chr4:83378316-83390668	OtA5088-OtA5092	OtA5093-OtA5097
Gm11426	chr11:82633352-82636309	OtA5088-OtA5092	OtA5093-OtA5097
Gm11428	chr11:83704055-83706269	OtA5088-OtA5092	OtA5093-OtA5097
Gm11435	chr11:83511735-83522175	OtA5088-OtA5092	OtA5093-OtA5097
Gm11437	chr11:84148360-84167476	OtA5088-OtA5092	OtA5093-OtA5097
Gm11468	chr2:166273907-166294831	OtA5088-OtA5092	OtA5093-OtA5097
Gm11487	chr4:73401031-73405072	OtA5088-OtA5092	OtA5093-OtA5097
Gm11529	chr11:96446898-96464547	OtA5088-OtA5092	OtA5093-OtA5097
Gm11541	chr11:94694497-94704499	OtA5088-OtA5092	OtA5093-OtA5097
Gm11544	chr11:94844830-94849593	OtA5088-OtA5092	OtA5093-OtA5097
Gm11545	chr11:94755135-94761182	OtA5088-OtA5092	OtA5093-OtA5097
Gm11549	chr3:36515056-36521445	OtA5088-OtA5092	OtA5093-OtA5097
Gm11554	chr11:99798185-99809896	OtA5088-OtA5092	OtA5093-OtA5097
Gm11559	chr11:99864475-99865571	OtA5088-OtA5092	OtA5093-OtA5097

Gm11562	chr11:99619597-99620404	OtA5088-OtA5092	OtA5093-OtA5097
Gm11563	chr11:99657941-99658959	OtA5088-OtA5092	OtA5093-OtA5097
Gm11564	chr11:99814975-99815666	OtA5088-OtA5092	OtA5093-OtA5097
Gm11565	chr11:99914750-99915671	OtA5088-OtA5092	OtA5093-OtA5097
Gm11567	chr11:99879186-99880229	OtA5088-OtA5092	OtA5093-OtA5097
Gm11568	chr11:99857916-99859060	OtA5088-OtA5092	OtA5093-OtA5097
Gm11569	chr11:99798185-99809896	OtA5088-OtA5092	OtA5093-OtA5097
Gm11570	chr11:99984702-99986593	OtA5088-OtA5092	OtA5093-OtA5097
Gm11595	chr11:99771713-99772913	OtA5088-OtA5092	OtA5093-OtA5097
Gm11596	chr11:99792674-99793292	OtA5088-OtA5092	OtA5093-OtA5097
Gm11602	chr1:63114470-63176822	OtA5088-OtA5092	OtA5093-OtA5097
Gm11627	chr11:102576398-102579119	OtA5088-OtA5092	OtA5093-OtA5097
Gm11648	chr11:103331483-103363692	OtA5088-OtA5092	OtA5093-OtA5097
Gm11696	chr11:109354777-109401369	OtA5088-OtA5092	OtA5093-OtA5097
Gm11710	chr11:115031485-115037769	OtA5088-OtA5092	OtA5093-OtA5097
Gm11744	chr11:116657107-116668389	OtA5088-OtA5092	OtA5093-OtA5097
Gm11747	chr11:118428456-118454995	OtA5088-OtA5092	OtA5093-OtA5097
Gm11756	chr4:73916727-73920760	OtA5088-OtA5092	OtA5093-OtA5097
Gm11757,Gm13871	chr4:73901905-73905953	OtA5088-OtA5092	OtA5093-OtA5097
Gm11758	chr4:73901905-73905953	OtA5088-OtA5092	OtA5093-OtA5097
Gm11762	chr11:119548629-119569046	OtA5088-OtA5092	OtA5093-OtA5097
Gm11818	chr4:12906836-12981485	OtA5088-OtA5092	OtA5093-OtA5097
Gm11837	chr4:14930640-15149131	OtA5088-OtA5092	OtA5093-OtA5097
Gm11937	chr11:99609793-99610189	OtA5088-OtA5092	OtA5093-OtA5097
Gm11938	chr11:99602645-99603308	OtA5088-OtA5092	OtA5093-OtA5097
Gm11944	chr11:3202352-3479831	OtA5088-OtA5092	OtA5093-OtA5097
Gm11961	chr11:4620274-4630011	OtA5088-OtA5092	OtA5093-OtA5097
Gm11978	chr11:6650147-6677475	OtA5088-OtA5092	OtA5093-OtA5097
Gm11981	chr11:6716000-6720037	OtA5088-OtA5092	OtA5093-OtA5097
Gm11985	chr11:7183156-7188569	OtA5088-OtA5092	OtA5093-OtA5097
Gm11992	chr11:9016053-9069354	OtA5088-OtA5092	OtA5093-OtA5097
Gm12	chr11:98598290-98599586	OtA5088-OtA5092	OtA5093-OtA5097
Gm12060	chr11:23558059-23558842	OtA5088-OtA5092	OtA5093-OtA5097
Gm12070	chr11:26785107-26787859	OtA5088-OtA5092	OtA5093-OtA5097
Gm12130	chr11:38491547-38520045	OtA5088-OtA5092	OtA5093-OtA5097
Gm12159	chr11:45102865-45116928	OtA5088-OtA5092	OtA5093-OtA5097
Gm12169	chr11:46524250-46538156	OtA5088-OtA5092	OtA5093-OtA5097
Gm12171	chr11:46548412-46556068	OtA5088-OtA5092	OtA5093-OtA5097
Gm12185	chr11:48904655-48927159	OtA5088-OtA5092	OtA5093-OtA5097
Gm12216	chr11:53785488-53859256	OtA5088-OtA5092	OtA5093-OtA5097
Gm12238	chr11:55469751-55500887	OtA5088-OtA5092	OtA5093-OtA5097
Gm12250	chr11:58183842-58190198	OtA5088-OtA5092	OtA5093-OtA5097
Gm12253	chr11:58432558-58441622	OtA5088-OtA5092	OtA5093-OtA5097
Gm12295	chr11:65277653-65287833	OtA5088-OtA5092	OtA5093-OtA5097
Gm12298	chr11:66905631-66947086	OtA5088-OtA5092	OtA5093-OtA5097
Gm12359	chr11:98795768-98833807	OtA5088-OtA5092	OtA5093-OtA5097
Gm12409	chr4:45762376-45764223	OtA5088-OtA5092	OtA5093-OtA5097

Gm12504	chr4:44121892-44124123	OtA5088-OtA5092	OtA5093-OtA5097
Gm12505	chr4:55410442-55418842	OtA5088-OtA5092	OtA5093-OtA5097
Gm12522	chr3:108364910-108383741	OtA5088-OtA5092	OtA5093-OtA5097
Gm12530	chr4:57172070-57176554	OtA5088-OtA5092	OtA5093-OtA5097
Gm12597	chr4:88557672-88558245	OtA5088-OtA5092	OtA5093-OtA5097
Gm12603	chr4:89050306-89084610	OtA5088-OtA5092	OtA5093-OtA5097
Gm12633	chr4:90359624-90361314	OtA5088-OtA5092	OtA5093-OtA5097
Gm12657	chr4:94565138-94603247	OtA5088-OtA5092	OtA5093-OtA5097
Gm12695	chr4:96723886-96785160	OtA5088-OtA5092	OtA5093-OtA5097
Gm12709	chr4:102760362-103011965	OtA5088-OtA5092	OtA5093-OtA5097
Gm12769	chr7:33743798-33745103	OtA5088-OtA5092	OtA5093-OtA5097
Gm12776	chr7:34095430-34100837	OtA5088-OtA5092	OtA5093-OtA5097
Gm12789	chr4:101986839-101990231	OtA5088-OtA5092	OtA5093-OtA5097
Gm12794	chr4:101940406-101943183	OtA5088-OtA5092	OtA5093-OtA5097
Gm128	chr3:95236919-95241109	OtA5088-OtA5092	OtA5093-OtA5097
Gm12830	chr4:114821719-114856166	OtA5088-OtA5092	OtA5093-OtA5097
Gm12839	chr4:115582037-115583127	OtA5088-OtA5092	OtA5093-OtA5097
Gm12886	chr4:121414734-121423099	OtA5088-OtA5092	OtA5093-OtA5097
Gm12887	chr4:121614003-121622125	OtA5088-OtA5092	OtA5093-OtA5097
Gm12888	chr4:121316315-121324917	OtA5088-OtA5092	OtA5093-OtA5097
Gm129	chr3:95871521-95891930	OtA5088-OtA5092	OtA5093-OtA5097
Gm13003	chr4:137159081-137165066	OtA5088-OtA5092	OtA5093-OtA5097
Gm13011	chr4:137401553-137409791	OtA5088-OtA5092	OtA5093-OtA5097
Gm13023	chr4:143789333-143795585	OtA5088-OtA5092	OtA5093-OtA5097
Gm13031	chr4:140906359-140957902	OtA5088-OtA5092	OtA5093-OtA5097
Gm13032	chr4:140810992-140845778	OtA5088-OtA5092	OtA5093-OtA5097
Gm13034	chr4:146067523-146068718	OtA5088-OtA5092	OtA5093-OtA5097
Gm13040	chr4:143551699-143558236	OtA5088-OtA5092	OtA5093-OtA5097
Gm13043	chr4:143511311-143517833	OtA5088-OtA5092	OtA5093-OtA5097
Gm13051	chr4:146097311-146126623	OtA5088-OtA5092	OtA5093-OtA5097
Gm13078	chr4:143719454-143729158	OtA5088-OtA5092	OtA5093-OtA5097
Gm13083	chr4:143615002-143618595	OtA5088-OtA5092	OtA5093-OtA5097
Gm13084	chr4:143809990-143816093	OtA5088-OtA5092	OtA5093-OtA5097
Gm13088	chr4:143653759-143657246	OtA5088-OtA5092	OtA5093-OtA5097
Gm13102	chr4:144099879-144110101	OtA5088-OtA5092	OtA5093-OtA5097
Gm13103	chr4:143846496-143853637	OtA5088-OtA5092	OtA5093-OtA5097
Gm13109	chr4:143948579-143951016	OtA5088-OtA5092	OtA5093-OtA5097
Gm13119	chr4:144357963-144364419	OtA5088-OtA5092	OtA5093-OtA5097
Gm13124	chr4:144554999-144565134	OtA5088-OtA5092	OtA5093-OtA5097
Gm13125	chr4:144372759-144377933	OtA5088-OtA5092	OtA5093-OtA5097
Gm13128	chr4:144330248-144333465	OtA5088-OtA5092	OtA5093-OtA5097
Gm13139,Gm13251	chr4:146270384-146469440	OtA5088-OtA5092	OtA5093-OtA5097
Gm13152	chr4:147175865-147513420	OtA5088-OtA5092	OtA5093-OtA5097
Gm13154	chr4:147553276-147585201	OtA5088-OtA5092	OtA5093-OtA5097
Gm13157	chr4:147753973-147809788	OtA5088-OtA5092	OtA5093-OtA5097
Gm13177	chr4:144613706-144623398	OtA5088-OtA5092	OtA5093-OtA5097
Gm13178	chr4:144703190-144721404	OtA5088-OtA5092	OtA5093-OtA5097

Gm13212	chr4:145617145-145624394	OtA5088-OtA5092	OtA5093-OtA5097
Gm1322	chr2:67173833-67186212	OtA5088-OtA5092	OtA5093-OtA5097
Gm13238	chr4:145837218-145837992	OtA5088-OtA5092	OtA5093-OtA5097
Gm13242	chr4:145670696-145704437	OtA5088-OtA5092	OtA5093-OtA5097
Gm13247	chr4:146501999-146546873	OtA5088-OtA5092	OtA5093-OtA5097
Gm13251	chr4:146270384-146469440	OtA5088-OtA5092	OtA5093-OtA5097
Gm13271	chr4:88754867-88755416	OtA5088-OtA5092	OtA5093-OtA5097
Gm13272,Gm13285	chr4:88779849-88780660	OtA5088-OtA5092	OtA5093-OtA5097
Gm13275,Gm13287	chr4:88799574-88801130	OtA5088-OtA5092	OtA5093-OtA5097
Gm13276	chr4:88785053-88786515	OtA5088-OtA5092	OtA5093-OtA5097
Gm13277,Gm13285	chr4:88787976-88789438	OtA5088-OtA5092	OtA5093-OtA5097
Gm13278,Gm13279,Gr	chr4:88796741-88798204	OtA5088-OtA5092	OtA5093-OtA5097
Gm13279	chr4:88796741-88798204	OtA5088-OtA5092	OtA5093-OtA5097
Gm13280	chr4:88676286-88676856	OtA5088-OtA5092	OtA5093-OtA5097
Gm13283	chr4:88760773-88761584	OtA5088-OtA5092	OtA5093-OtA5097
Gm13285,Gm13288,Gr	chr4:88805530-88808380	OtA5088-OtA5092	OtA5093-OtA5097
Gm13286	chr4:88757843-88758392	OtA5088-OtA5092	OtA5093-OtA5097
Gm13287	chr4:88799574-88801130	OtA5088-OtA5092	OtA5093-OtA5097
Gm13288	chr4:88805530-88808380	OtA5088-OtA5092	OtA5093-OtA5097
Gm13289	chr4:88805530-88808380	OtA5088-OtA5092	OtA5093-OtA5097
Gm13290	chr4:88773833-88774801	OtA5088-OtA5092	OtA5093-OtA5097
Gm13293	chr2:11338365-11398500	OtA5088-OtA5092	OtA5093-OtA5097
Gm13298	chr4_GL456350_random:144078-14987	OtA5088-OtA5092	OtA5093-OtA5097
Gm13304	chr4_GL456350_random:211324-21245	OtA5088-OtA5092	OtA5093-OtA5097
Gm13305,Gm2002	chr4_JH584294_random:1717-13572	OtA5088-OtA5092	OtA5093-OtA5097
Gm13306	chr4_JH584294_random:1717-13572	OtA5088-OtA5092	OtA5093-OtA5097
Gm13308	chr4_GL456350_random:173251-17428	OtA5088-OtA5092	OtA5093-OtA5097
Gm13315	chr2:14604305-14987908	OtA5088-OtA5092	OtA5093-OtA5097
Gm1332	chr2:157087054-157096481	OtA5088-OtA5092	OtA5093-OtA5097
Gm1337	chr2:118699102-118703963	OtA5088-OtA5092	OtA5093-OtA5097
Gm13446	chr2:35549533-35730994	OtA5088-OtA5092	OtA5093-OtA5097
Gm13476	chr2:44983633-45114084	OtA5088-OtA5092	OtA5093-OtA5097
Gm13483	chr2:50296809-50433967	OtA5088-OtA5092	OtA5093-OtA5097
Gm13490	chr2:51656965-51732009	OtA5088-OtA5092	OtA5093-OtA5097
Gm13498	chr2:50909683-50911846	OtA5088-OtA5092	OtA5093-OtA5097
Gm13539	chr2:25784616-25787022	OtA5088-OtA5092	OtA5093-OtA5097
Gm13544	chr2:58267452-58357758	OtA5088-OtA5092	OtA5093-OtA5097
Gm13546	chr2:58163973-58177063	OtA5088-OtA5092	OtA5093-OtA5097
Gm13547	chr2:29761527-29764089	OtA5088-OtA5092	OtA5093-OtA5097
Gm13580	chr2:60411524-60412574	OtA5088-OtA5092	OtA5093-OtA5097
Gm13582	chr2:60964375-60975151	OtA5088-OtA5092	OtA5093-OtA5097
Gm136	chr4:34743787-34756259	OtA5088-OtA5092	OtA5093-OtA5097
Gm13629	chr2:66440879-66634952	OtA5088-OtA5092	OtA5093-OtA5097
Gm13710	chr2:84500747-84506873	OtA5088-OtA5092	OtA5093-OtA5097
Gm13718	chr2:84659078-84668781	OtA5088-OtA5092	OtA5093-OtA5097
Gm13749	chr1:63964726-63968999	OtA5088-OtA5092	OtA5093-OtA5097
Gm13752	chr2:80391363-80398216	OtA5088-OtA5092	OtA5093-OtA5097

Gm13807	chr2:93403546-93417830	OtA5088-OtA5092	OtA5093-OtA5097
Gm13845	chr6:31366884-31398773	OtA5088-OtA5092	OtA5093-OtA5097
Gm13871	chr4:73901905-73905953	OtA5088-OtA5092	OtA5093-OtA5097
Gm13939	chr2:109902395-109913319	OtA5088-OtA5092	OtA5093-OtA5097
Gm13944	chr2:77410626-77816816	OtA5088-OtA5092	OtA5093-OtA5097
Gm14015	chr2:106463822-106523103	OtA5088-OtA5092	OtA5093-OtA5097
Gm14023	chr2:129297369-129309972	OtA5088-OtA5092	OtA5093-OtA5097
Gm14047	chr2:129364579-129375733	OtA5088-OtA5092	OtA5093-OtA5097
Gm14057	chr2:130707852-130840110	OtA5088-OtA5092	OtA5093-OtA5097
Gm14085	chr2:122484940-122528040	OtA5088-OtA5092	OtA5093-OtA5097
Gm14092	chr2:145242610-145641939	OtA5088-OtA5092	OtA5093-OtA5097
Gm14124	chr2:150257516-150270300	OtA5088-OtA5092	OtA5093-OtA5097
Gm14137	chr2:119172499-119217050	OtA5088-OtA5092	OtA5093-OtA5097
Gm14139	chr2:150181754-150193279	OtA5088-OtA5092	OtA5093-OtA5097
Gm14151	chr2:151098244-151103809	OtA5088-OtA5092	OtA5093-OtA5097
Gm14164	chr2:152345668-152376566	OtA5088-OtA5092	OtA5093-OtA5097
Gm14169	chr2:156609197-156613422	OtA5088-OtA5092	OtA5093-OtA5097
Gm14204	chr2:158595471-158610723	OtA5088-OtA5092	OtA5093-OtA5097
Gm14207	chr2:119321198-119335666	OtA5088-OtA5092	OtA5093-OtA5097
Gm14288,Gm14435,Gr	chr2:175789356-175801038	OtA5088-OtA5092	OtA5093-OtA5097
Gm14295	chr2:176798599-176808704	OtA5088-OtA5092	OtA5093-OtA5097
Gm14306,Gm14436	chr2:175470024-175483322	OtA5088-OtA5092	OtA5093-OtA5097
Gm14308,Gm14430,O	chr2:176619932-176636319	OtA5088-OtA5092	OtA5093-OtA5097
Gm14316	chr2:164715304-164718068	OtA5088-OtA5092	OtA5093-OtA5097
Gm14318	chr2:180325087-180340732	OtA5088-OtA5092	OtA5093-OtA5097
Gm14322	chr2:177759287-177773275	OtA5088-OtA5092	OtA5093-OtA5097
Gm14325	chr2:177828990-177840318	OtA5088-OtA5092	OtA5093-OtA5097
Gm14326	chr2:177942610-177957289	OtA5088-OtA5092	OtA5093-OtA5097
Gm14327	chr2:177897162-177927850	OtA5088-OtA5092	OtA5093-OtA5097
Gm14346,Gm14351,Gr	chrX:34159624-34161584	OtA5088-OtA5092	OtA5093-OtA5097
Gm14347	chrX:4196575-4198535	OtA5088-OtA5092	OtA5093-OtA5097
Gm14351	chrX:34159624-34161584	OtA5088-OtA5092	OtA5093-OtA5097
Gm14354	chrX:31962043-31962821	OtA5088-OtA5092	OtA5093-OtA5097
Gm14374	chrX:5669066-5671026	OtA5088-OtA5092	OtA5093-OtA5097
Gm14378	chr8:4248213-4251423	OtA5088-OtA5092	OtA5093-OtA5097
Gm14379	chrX:7375924-7378042	OtA5088-OtA5092	OtA5093-OtA5097
Gm14391	chr2:175869237-175988574	OtA5088-OtA5092	OtA5093-OtA5097
Gm14393	chr2:175061548-175067763	OtA5088-OtA5092	OtA5093-OtA5097
Gm14403	chr2:177498225-177512311	OtA5088-OtA5092	OtA5093-OtA5097
Gm14405	chr2:176852791-176864437	OtA5088-OtA5092	OtA5093-OtA5097
Gm14420	chr2:177464741-177479194	OtA5088-OtA5092	OtA5093-OtA5097
Gm14430	chr2:176619932-176636319	OtA5088-OtA5092	OtA5093-OtA5097
Gm14431	chr2:176521003-176533821	OtA5088-OtA5092	OtA5093-OtA5097
Gm14434	chr2:175185164-175338212	OtA5088-OtA5092	OtA5093-OtA5097
Gm14435	chr2:176423660-176433086	OtA5088-OtA5092	OtA5093-OtA5097
Gm14436	chr2:175470024-175483322	OtA5088-OtA5092	OtA5093-OtA5097
Gm14440	chr2:175789356-175801038	OtA5088-OtA5092	OtA5093-OtA5097

Gm14446	chr19:34592887-34601968	OtA5088-OtA5092	OtA5093-OtA5097
Gm14458	chrX:8985899-8986755	OtA5088-OtA5092	OtA5093-OtA5097
Gm14459	chrX:8514051-8524954	OtA5088-OtA5092	OtA5093-OtA5097
Gm14461	chr2:78237546-78302230	OtA5088-OtA5092	OtA5093-OtA5097
Gm14474	chrX:11302431-11302921	OtA5088-OtA5092	OtA5093-OtA5097
Gm14475	chrX:11324658-11324976	OtA5088-OtA5092	OtA5093-OtA5097
Gm14476	chrX:11305654-11305972	OtA5088-OtA5092	OtA5093-OtA5097
Gm14477,Gm14478,Gr	chrX:11318255-11318764	OtA5088-OtA5092	OtA5093-OtA5097
Gm14478	chrX:11321422-11321931	OtA5088-OtA5092	OtA5093-OtA5097
Gm14482	chrX:11321422-11321931	OtA5088-OtA5092	OtA5093-OtA5097
Gm14483	chrX:11308754-11309263	OtA5088-OtA5092	OtA5093-OtA5097
Gm14484	chrX:11311933-11312427	OtA5088-OtA5092	OtA5093-OtA5097
Gm14492	chr7:142460869-142516009	OtA5088-OtA5092	OtA5093-OtA5097
Gm14496	chr2:181991225-182001087	OtA5088-OtA5092	OtA5093-OtA5097
Gm14501	chrX:9350598-9351137	OtA5088-OtA5092	OtA5093-OtA5097
Gm14511	chrX:8975711-8976559	OtA5088-OtA5092	OtA5093-OtA5097
Gm14525	chrX:26672777-26702638	OtA5088-OtA5092	OtA5093-OtA5097
Gm14548	chr7:3836809-3915501	OtA5088-OtA5092	OtA5093-OtA5097
Gm14625	chrX:55430234-55446892	OtA5088-OtA5092	OtA5093-OtA5097
Gm14632	chrX:35404186-35427093	OtA5088-OtA5092	OtA5093-OtA5097
Gm14634	chrX:12762277-12821492	OtA5088-OtA5092	OtA5093-OtA5097
Gm14635	chrX:12339778-12354996	OtA5088-OtA5092	OtA5093-OtA5097
Gm14685	chrX:73137231-73158399	OtA5088-OtA5092	OtA5093-OtA5097
Gm14692	chrX:67695697-67706350	OtA5088-OtA5092	OtA5093-OtA5097
Gm14718	chrX:57200636-57204376	OtA5088-OtA5092	OtA5093-OtA5097
Gm14725	chrX:70545557-70546385	OtA5088-OtA5092	OtA5093-OtA5097
Gm14743	chrX:78240461-78245921	OtA5088-OtA5092	OtA5093-OtA5097
Gm14744	chrX:77864757-77870033	OtA5088-OtA5092	OtA5093-OtA5097
Gm14781	chrX:91632193-91635671	OtA5088-OtA5092	OtA5093-OtA5097
Gm14812	chrX:99405330-99443797	OtA5088-OtA5092	OtA5093-OtA5097
Gm14819	chrX:35067383-35090316	OtA5088-OtA5092	OtA5093-OtA5097
Gm14827	chrX:94442731-94447727	OtA5088-OtA5092	OtA5093-OtA5097
Gm14850	chr8:21565385-21566350	OtA5088-OtA5092	OtA5093-OtA5097
Gm14851	chr8:21094970-21096050	OtA5088-OtA5092	OtA5093-OtA5097
Gm14872	chr17:47686644-47691092	OtA5088-OtA5092	OtA5093-OtA5097
Gm14873	chr17:47695206-47704286	OtA5088-OtA5092	OtA5093-OtA5097
Gm14920	chrX:117014756-117015104	OtA5088-OtA5092	OtA5093-OtA5097
Gm15008	chrX:36910833-36989695	OtA5088-OtA5092	OtA5093-OtA5097
Gm15023	chrX:135167623-135177632	OtA5088-OtA5092	OtA5093-OtA5097
Gm15056	chr8:20900605-20901973	OtA5088-OtA5092	OtA5093-OtA5097
Gm15070	chrX:144381670-144688180	OtA5088-OtA5092	OtA5093-OtA5097
Gm15085	chrX:149447616-149487784	OtA5088-OtA5092	OtA5093-OtA5097
Gm15091	chrX:149784478-149984982	OtA5088-OtA5092	OtA5093-OtA5097
Gm15093	chrX:149447616-149487784	OtA5088-OtA5092	OtA5093-OtA5097
Gm15097	chrX:149784478-149984982	OtA5088-OtA5092	OtA5093-OtA5097
Gm15104	chrX:150312950-150313364	OtA5088-OtA5092	OtA5093-OtA5097
Gm15107	chrX:148180723-148521446	OtA5088-OtA5092	OtA5093-OtA5097

Gm15114	chrX:148180723-148521446	OtA5088-OtA5092	OtA5093-OtA5097
Gm15127	chrX:149784478-149984982	OtA5088-OtA5092	OtA5093-OtA5097
Gm15179	chr1:75368209-75375015	OtA5088-OtA5092	OtA5093-OtA5097
Gm15217	chr14:46379523-46390599	OtA5088-OtA5092	OtA5093-OtA5097
Gm1527	chr3:28892616-28926724	OtA5088-OtA5092	OtA5093-OtA5097
Gm15284	chr8:21134649-21135598	OtA5088-OtA5092	OtA5093-OtA5097
Gm15292	chr8:21249761-21250461	OtA5088-OtA5092	OtA5093-OtA5097
Gm15293	chr8:21201603-21202446	OtA5088-OtA5092	OtA5093-OtA5097
Gm15299	chr8:21315545-21316387	OtA5088-OtA5092	OtA5093-OtA5097
Gm15308	chr8:21529029-21530012	OtA5088-OtA5092	OtA5093-OtA5097
Gm15315	chr8:21665796-21666733	OtA5088-OtA5092	OtA5093-OtA5097
Gm15319	chr8:20337663-20363202	OtA5088-OtA5092	OtA5093-OtA5097
Gm15348	chr8:12706943-12719127	OtA5088-OtA5092	OtA5093-OtA5097
Gm15350	chr8:12757015-12868728	OtA5088-OtA5092	OtA5093-OtA5097
Gm15386	chr1:18250977-18265138	OtA5088-OtA5092	OtA5093-OtA5097
Gm15401	chr6:72626379-72788956	OtA5088-OtA5092	OtA5093-OtA5097
Gm15408	chr5:149006961-149012776	OtA5088-OtA5092	OtA5093-OtA5097
Gm15412	chr7:96210636-96908554	OtA5088-OtA5092	OtA5093-OtA5097
Gm15413	chr7:96210636-96908554	OtA5088-OtA5092	OtA5093-OtA5097
Gm15417	chr3:89377645-89398779	OtA5088-OtA5092	OtA5093-OtA5097
Gm15421	chr5:22486488-22550331	OtA5088-OtA5092	OtA5093-OtA5097
Gm15441	chr3:96555767-96566801	OtA5088-OtA5092	OtA5093-OtA5097
Gm15455	chr1:33835898-33838914	OtA5088-OtA5092	OtA5093-OtA5097
Gm15471	chr3:103800604-103809387	OtA5088-OtA5092	OtA5093-OtA5097
Gm1553	chr10:82486532-82492618	OtA5088-OtA5092	OtA5093-OtA5097
Gm15545	chr7:44986899-44997579	OtA5088-OtA5092	OtA5093-OtA5097
Gm156	chr6:129766646-129775849	OtA5088-OtA5092	OtA5093-OtA5097
Gm15612	chr6:88842557-88874044	OtA5088-OtA5092	OtA5093-OtA5097
Gm1564	chr11:102665587-102682237	OtA5088-OtA5092	OtA5093-OtA5097
Gm15645	chr7:105861780-105863327	OtA5088-OtA5092	OtA5093-OtA5097
Gm15663	chr10:105574550-105583870	OtA5088-OtA5092	OtA5093-OtA5097
Gm1568	chr12:80755446-80760715	OtA5088-OtA5092	OtA5093-OtA5097
Gm15698	chr11:88964665-88966917	OtA5088-OtA5092	OtA5093-OtA5097
Gm15706	chr6:145250551-145251856	OtA5088-OtA5092	OtA5093-OtA5097
Gm15708	chr5:144244436-144358112	OtA5088-OtA5092	OtA5093-OtA5097
Gm1574	chr13:46273720-46300115	OtA5088-OtA5092	OtA5093-OtA5097
Gm15760	chr16:20545111-20548556	OtA5088-OtA5092	OtA5093-OtA5097
Gm15787	chr5:110135839-110176504	OtA5088-OtA5092	OtA5093-OtA5097
Gm1587	chr14:77793944-77798968	OtA5088-OtA5092	OtA5093-OtA5097
Gm15880	chr7:80586631-80688877	OtA5088-OtA5092	OtA5093-OtA5097
Gm15881	chr8:57774051-58911627	OtA5088-OtA5092	OtA5093-OtA5097
Gm15910	chr10:120781100-120898971	OtA5088-OtA5092	OtA5093-OtA5097
Gm15915	chr10:93641048-93745972	OtA5088-OtA5092	OtA5093-OtA5097
Gm15941	chr15:37366174-37792428	OtA5088-OtA5092	OtA5093-OtA5097
Gm15987	chr6:128934380-128984707	OtA5088-OtA5092	OtA5093-OtA5097
Gm15997	chr5:149489055-149611894	OtA5088-OtA5092	OtA5093-OtA5097
Gm16023	chr4:155601415-155624755	OtA5088-OtA5092	OtA5093-OtA5097

Gm1604b	chr17:8038509-8101228	OtA5088-OtA5092	OtA5093-OtA5097
Gm16062	chr11:59810079-59839767	OtA5088-OtA5092	OtA5093-OtA5097
Gm16119	chr2:181569152-181592461	OtA5088-OtA5092	OtA5093-OtA5097
Gm16157	chr7:68266338-68363092	OtA5088-OtA5092	OtA5093-OtA5097
Gm16197	chr17:29057473-29078961	OtA5088-OtA5092	OtA5093-OtA5097
Gm16287	chr4:139175406-139180655	OtA5088-OtA5092	OtA5093-OtA5097
Gm16291	chr15:39768484-39857470	OtA5088-OtA5092	OtA5093-OtA5097
Gm16294	chr15:40511321-40527277	OtA5088-OtA5092	OtA5093-OtA5097
Gm1631	chr2:71719416-71730966	OtA5088-OtA5092	OtA5093-OtA5097
Gm16325	chr3:146641931-146651715	OtA5088-OtA5092	OtA5093-OtA5097
Gm16336	chr7:110868266-110982095	OtA5088-OtA5092	OtA5093-OtA5097
Gm16367	chr5_JH584299_random:829033-84174	OtA5088-OtA5092	OtA5093-OtA5097
Gm16381	chr12:87841839-87846716	OtA5088-OtA5092	OtA5093-OtA5097
Gm16387	chr7:39256570-39264481	OtA5088-OtA5092	OtA5093-OtA5097
Gm16390	chrX:154886802-154890267	OtA5088-OtA5092	OtA5093-OtA5097
Gm16404	chrX:55551595-55568457	OtA5088-OtA5092	OtA5093-OtA5097
Gm16430	chrX:54622210-54639016	OtA5088-OtA5092	OtA5093-OtA5097
Gm16432	chr1:177991434-178048291	OtA5088-OtA5092	OtA5093-OtA5097
Gm16442	chr7:22117321-22118245	OtA5088-OtA5092	OtA5093-OtA5097
Gm16445	chrX:154152570-154156887	OtA5088-OtA5092	OtA5093-OtA5097
Gm16451	chr7:20121655-20122579	OtA5088-OtA5092	OtA5093-OtA5097
Gm1647	chr3:69131232-69157177	OtA5088-OtA5092	OtA5093-OtA5097
Gm16497	chr12:14261533-14285684	OtA5088-OtA5092	OtA5093-OtA5097
Gm16523	chr2:39066214-39190730	OtA5088-OtA5092	OtA5093-OtA5097
Gm1653	chr3:149274736-149279980	OtA5088-OtA5092	OtA5093-OtA5097
Gm16532	chr7:6415174-6431086	OtA5088-OtA5092	OtA5093-OtA5097
Gm16548	chr1:164115263-164220274	OtA5088-OtA5092	OtA5093-OtA5097
Gm16551	chr9:74848436-74852872	OtA5088-OtA5092	OtA5093-OtA5097
Gm16576	chr15:79742697-79757394	OtA5088-OtA5092	OtA5093-OtA5097
Gm16596	chr12:108410654-108702306	OtA5088-OtA5092	OtA5093-OtA5097
Gm166	chr7:127573347-127588595	OtA5088-OtA5092	OtA5093-OtA5097
Gm1661	chr4:117213332-117251917	OtA5088-OtA5092	OtA5093-OtA5097
Gm16617	chr14:51984832-52020733	OtA5088-OtA5092	OtA5093-OtA5097
Gm16677	chr14:69547956-69555387	OtA5088-OtA5092	OtA5093-OtA5097
Gm16701	chr1:166974022-166999730	OtA5088-OtA5092	OtA5093-OtA5097
Gm16702	chr17:8372395-8422726	OtA5088-OtA5092	OtA5093-OtA5097
Gm1679	chr5:110354095-110356087	OtA5088-OtA5092	OtA5093-OtA5097
Gm16793	chr8:35582502-35679449	OtA5088-OtA5092	OtA5093-OtA5097
Gm16796	chr2:170482964-170519070	OtA5088-OtA5092	OtA5093-OtA5097
Gm16833	chr9:8994952-9260885	OtA5088-OtA5092	OtA5093-OtA5097
Gm16853	chr9:21368002-21411720	OtA5088-OtA5092	OtA5093-OtA5097
Gm16861	chr16:4886099-4941020	OtA5088-OtA5092	OtA5093-OtA5097
Gm16880	chr1:136696210-136725728	OtA5088-OtA5092	OtA5093-OtA5097
Gm16894	chr1:40076211-40125226	OtA5088-OtA5092	OtA5093-OtA5097
Gm16897	chr1:194938820-194976959	OtA5088-OtA5092	OtA5093-OtA5097
Gm16907	chr13:63014933-63299656	OtA5088-OtA5092	OtA5093-OtA5097
Gm16973	chr14:56668247-56701000	OtA5088-OtA5092	OtA5093-OtA5097

Gm16982	chr7:141562179-141633011	OtA5088-OtA5092	OtA5093-OtA5097
Gm16998	chr10:54075921-54081081	OtA5088-OtA5092	OtA5093-OtA5097
Gm17019	chr5:15028949-15033007	OtA5088-OtA5092	OtA5093-OtA5097
Gm1715	chr9:52679862-52798772	OtA5088-OtA5092	OtA5093-OtA5097
Gm1720	chrX:166662024-166689812	OtA5088-OtA5092	OtA5093-OtA5097
Gm17252	chr9:35685078-35687371	OtA5088-OtA5092	OtA5093-OtA5097
Gm17296	chr8:126426651-126475065	OtA5088-OtA5092	OtA5093-OtA5097
Gm17359	chr3:79345375-79567679	OtA5088-OtA5092	OtA5093-OtA5097
Gm17365	chr9:35645112-35648202	OtA5088-OtA5092	OtA5093-OtA5097
Gm17384	chr5:62813822-62888318	OtA5088-OtA5092	OtA5093-OtA5097
Gm17455	chr10:60302749-60696490	OtA5088-OtA5092	OtA5093-OtA5097
Gm17495	chr17:40520021-40530702	OtA5088-OtA5092	OtA5093-OtA5097
Gm17660	chr5:104070059-104077608	OtA5088-OtA5092	OtA5093-OtA5097
Gm17677	chr9:35741048-35742252	OtA5088-OtA5092	OtA5093-OtA5097
Gm17689	chr9:36581277-36582635	OtA5088-OtA5092	OtA5093-OtA5097
Gm17727	chr9:35776523-35778109	OtA5088-OtA5092	OtA5093-OtA5097
Gm17745	chr10:93336231-93348879	OtA5088-OtA5092	OtA5093-OtA5097
Gm17746	chr12:25129173-25132274	OtA5088-OtA5092	OtA5093-OtA5097
Gm17751	chr1:76489132-76502028	OtA5088-OtA5092	OtA5093-OtA5097
Gm17757,Gm18853	chr7:106156535-106215340	OtA5088-OtA5092	OtA5093-OtA5097
Gm17762	chr2:18025188-18037741	OtA5088-OtA5092	OtA5093-OtA5097
Gm17769	chr10:77418375-77420939	OtA5088-OtA5092	OtA5093-OtA5097
Gm17801	chr17:25260510-25275528	OtA5088-OtA5092	OtA5093-OtA5097
Gm17821	chr12:67656741-67669271	OtA5088-OtA5092	OtA5093-OtA5097
Gm17830	chr17:49304478-49312960	OtA5088-OtA5092	OtA5093-OtA5097
Gm18409	chr10:99609170-99658134	OtA5088-OtA5092	OtA5093-OtA5097
Gm18853	chr7:106156535-106215340	OtA5088-OtA5092	OtA5093-OtA5097
Gm19276	chr15:10829050-10832713	OtA5088-OtA5092	OtA5093-OtA5097
Gm19277	chr15:85149253-85156132	OtA5088-OtA5092	OtA5093-OtA5097
Gm19299	chr9:67133560-67163788	OtA5088-OtA5092	OtA5093-OtA5097
Gm19303	chr15:51231639-51395610	OtA5088-OtA5092	OtA5093-OtA5097
Gm19395	chr10:53278080-53379851	OtA5088-OtA5092	OtA5093-OtA5097
Gm19402	chr10:77689956-77690757	OtA5088-OtA5092	OtA5093-OtA5097
Gm19424	chr19:41746960-41750097	OtA5088-OtA5092	OtA5093-OtA5097
Gm19434	chr6:135188479-135194959	OtA5088-OtA5092	OtA5093-OtA5097
Gm19461	chr1:133246096-133303435	OtA5088-OtA5092	OtA5093-OtA5097
Gm19466	chr18:53648150-53744547	OtA5088-OtA5092	OtA5093-OtA5097
Gm19510	chr15:59788416-59794959	OtA5088-OtA5092	OtA5093-OtA5097
Gm19522	chr16:42884369-42912070	OtA5088-OtA5092	OtA5093-OtA5097
Gm19557	chr19:47501047-47537020	OtA5088-OtA5092	OtA5093-OtA5097
Gm19583	chr5:75110320-75147107	OtA5088-OtA5092	OtA5093-OtA5097
Gm19589	chr1:88551516-88562166	OtA5088-OtA5092	OtA5093-OtA5097
Gm19619	chr5:91257570-91283076	OtA5088-OtA5092	OtA5093-OtA5097
Gm1965	chr6:89141004-89146016	OtA5088-OtA5092	OtA5093-OtA5097
Gm19668	chr10:77798386-77799133	OtA5088-OtA5092	OtA5093-OtA5097
Gm1968	chr16:29957926-29979124	OtA5088-OtA5092	OtA5093-OtA5097
Gm19689	chr17:83033591-83078225	OtA5088-OtA5092	OtA5093-OtA5097

Gm19705	chr1:136683395-136690805	OtA5088-OtA5092	OtA5093-OtA5097
Gm19710	chr3:89998759-90052515	OtA5088-OtA5092	OtA5093-OtA5097
Gm19757	chr6:105481265-105492668	OtA5088-OtA5092	OtA5093-OtA5097
Gm1976	chr17:94750099-94834799	OtA5088-OtA5092	OtA5093-OtA5097
Gm19782	chr15:69924232-70078204	OtA5088-OtA5092	OtA5093-OtA5097
Gm19784	chr18:66924761-66949961	OtA5088-OtA5092	OtA5093-OtA5097
Gm1987	chr4_JH584294_random:120654-12178[illegible]	OtA5088-OtA5092	OtA5093-OtA5097
Gm19897	chr7:28766751-28788665	OtA5088-OtA5092	OtA5093-OtA5097
Gm1993	chrX:25548295-25570452	OtA5088-OtA5092	OtA5093-OtA5097
Gm1995	chr12:87861349-87865690	OtA5088-OtA5092	OtA5093-OtA5097
Gm19990	chr12:56253242-56266381	OtA5088-OtA5092	OtA5093-OtA5097
Gm2002	chr4_JH584294_random:1717-13572	OtA5088-OtA5092	OtA5093-OtA5097
Gm20063	chr12:61499659-61521820	OtA5088-OtA5092	OtA5093-OtA5097
Gm20098	chr17:51882726-51965257	OtA5088-OtA5092	OtA5093-OtA5097
Gm2011	chr3:40846998-40875609	OtA5088-OtA5092	OtA5093-OtA5097
Gm20110	chr10:99609170-99658134	OtA5088-OtA5092	OtA5093-OtA5097
Gm2012	chrX:26200882-26231323	OtA5088-OtA5092	OtA5093-OtA5097
Gm20125	chr10:16951093-17095782	OtA5088-OtA5092	OtA5093-OtA5097
Gm20139	chr10:19141748-19207947	OtA5088-OtA5092	OtA5093-OtA5097
Gm2016	chr12:87874071-87877859	OtA5088-OtA5092	OtA5093-OtA5097
Gm20172	chr1:25067475-25829707	OtA5088-OtA5092	OtA5093-OtA5097
Gm20187	chr12:27090236-27114995	OtA5088-OtA5092	OtA5093-OtA5097
Gm20199	chr9:59388553-59486374	OtA5088-OtA5092	OtA5093-OtA5097
Gm2022	chr12:87892833-87896722	OtA5088-OtA5092	OtA5093-OtA5097
Gm20257	chr1:58652640-58655831	OtA5088-OtA5092	OtA5093-OtA5097
Gm20268	chr1:101155767-101175202	OtA5088-OtA5092	OtA5093-OtA5097
Gm2027	chr12:44221355-44224380	OtA5088-OtA5092	OtA5093-OtA5097
Gm2030	chrX:26286976-26310044	OtA5088-OtA5092	OtA5093-OtA5097
Gm20324	chr15:82808625-82912134	OtA5088-OtA5092	OtA5093-OtA5097
Gm20337	chr12:80942651-80945399	OtA5088-OtA5092	OtA5093-OtA5097
Gm20356	chr3:74009218-74017277	OtA5088-OtA5092	OtA5093-OtA5097
Gm20362	chr6:79032041-79035869	OtA5088-OtA5092	OtA5093-OtA5097
Gm20408	chr17:38416473-38420185	OtA5088-OtA5092	OtA5093-OtA5097
Gm20410	chr17:38554191-38560621	OtA5088-OtA5092	OtA5093-OtA5097
Gm20516	chr17:38639446-38645653	OtA5088-OtA5092	OtA5093-OtA5097
Gm20554	chr13:72623465-72628564	OtA5088-OtA5092	OtA5093-OtA5097
Gm20556	chr15:84714628-84772207	OtA5088-OtA5092	OtA5093-OtA5097
Gm20571	chr2:144368982-144407675	OtA5088-OtA5092	OtA5093-OtA5097
Gm20579	chr17:39406358-39410992	OtA5088-OtA5092	OtA5093-OtA5097
Gm20580	chr17:40598593-40602617	OtA5088-OtA5092	OtA5093-OtA5097
Gm20581	chr17:39073690-39077037	OtA5088-OtA5092	OtA5093-OtA5097
Gm20597	chr10:53498442-53518989	OtA5088-OtA5092	OtA5093-OtA5097
Gm2061	chr1:185447115-185468761	OtA5088-OtA5092	OtA5093-OtA5097
Gm2083,Mup19	chr4:61778324-61782224	OtA5088-OtA5092	OtA5093-OtA5097
Gm2109	chr9:83691474-83693247	OtA5088-OtA5092	OtA5093-OtA5097
Gm2115	chr7:84528953-84578339	OtA5088-OtA5092	OtA5093-OtA5097
Gm216	chr1:74362107-74369321	OtA5088-OtA5092	OtA5093-OtA5097

Gm2176	chr18:80541891-80544546	OtA5088-OtA5092	OtA5093-OtA5097
Gm2381	chr7:42816826-42867234	OtA5088-OtA5092	OtA5093-OtA5097
Gm239	chr10:116776544-116896879	OtA5088-OtA5092	OtA5093-OtA5097
Gm2447	chr3:52739723-52776654	OtA5088-OtA5092	OtA5093-OtA5097
Gm2506	chr4_JH584293_random:7077-22237	OtA5088-OtA5092	OtA5093-OtA5097
Gm2516	chr8:50368111-50416089	OtA5088-OtA5092	OtA5093-OtA5097
Gm2518	chr19:8772521-8786417	OtA5088-OtA5092	OtA5093-OtA5097
Gm266	chr12:111484608-111485823	OtA5088-OtA5092	OtA5093-OtA5097
Gm2663	chr6:40995821-40999479	OtA5088-OtA5092	OtA5093-OtA5097
Gm2694	chr8:87472811-87525554	OtA5088-OtA5092	OtA5093-OtA5097
Gm2696	chr10:77814681-77815404	OtA5088-OtA5092	OtA5093-OtA5097
Gm2721	chr12:105394855-105403760	OtA5088-OtA5092	OtA5093-OtA5097
Gm2762	chr13:53393688-53455630	OtA5088-OtA5092	OtA5093-OtA5097
Gm2799,Gmcl1l	chrX:32560054-32562009	OtA5088-OtA5092	OtA5093-OtA5097
Gm2825	chrX:32973896-32975846	OtA5088-OtA5092	OtA5093-OtA5097
Gm2837	chrX:33056285-33057063	OtA5088-OtA5092	OtA5093-OtA5097
Gm2848	chr13:52569879-52573225	OtA5088-OtA5092	OtA5093-OtA5097
Gm2863	chrX:33313337-33315288	OtA5088-OtA5092	OtA5093-OtA5097
Gm2897	chr14:3049285-3077025	OtA5088-OtA5092	OtA5093-OtA5097
Gm2913	chrX:33575386-33577346	OtA5088-OtA5092	OtA5093-OtA5097
Gm2933	chrX:33700648-33702349	OtA5088-OtA5092	OtA5093-OtA5097
Gm3002	chr14:3814912-3830681	OtA5088-OtA5092	OtA5093-OtA5097
Gm3020,Gm8348	chr14:3652029-3673272	OtA5088-OtA5092	OtA5093-OtA5097
Gm3086	chr12:69746847-69987002	OtA5088-OtA5092	OtA5093-OtA5097
Gm3139	chr5_JH584297_random:107878-11995[illegible]	OtA5088-OtA5092	OtA5093-OtA5097
Gm3143	chr3:34699639-34716662	OtA5088-OtA5092	OtA5093-OtA5097
Gm3219	chr14:34345054-34345658	OtA5088-OtA5092	OtA5093-OtA5097
Gm3230	chr2:19655805-19657897	OtA5088-OtA5092	OtA5093-OtA5097
Gm3238	chr10:77770632-77771325	OtA5088-OtA5092	OtA5093-OtA5097
Gm3258	chr10:31332379-31445921	OtA5088-OtA5092	OtA5093-OtA5097
Gm3264	chr14:4871155-4875851	OtA5088-OtA5092	OtA5093-OtA5097
Gm3279	chr6:55684570-55694574	OtA5088-OtA5092	OtA5093-OtA5097
Gm3285	chr10:77861974-77862638	OtA5088-OtA5092	OtA5093-OtA5097
Gm3286	chr5_GL456354_random:82558-110906	OtA5088-OtA5092	OtA5093-OtA5097
Gm3317	chr14:5516278-5522929	OtA5088-OtA5092	OtA5093-OtA5097
Gm3336	chr8:70718542-70722635	OtA5088-OtA5092	OtA5093-OtA5097
Gm3383	chr14:5791612-5801530	OtA5088-OtA5092	OtA5093-OtA5097
Gm3402,Gm3415	chr5:146556112-146558915	OtA5088-OtA5092	OtA5093-OtA5097
Gm3404	chr5:146525800-146528553	OtA5088-OtA5092	OtA5093-OtA5097
Gm3409	chr5:146537647-146540427	OtA5088-OtA5092	OtA5093-OtA5097
Gm3414	chr5:45669924-45857540	OtA5088-OtA5092	OtA5093-OtA5097
Gm3415	chr5:146556112-146558915	OtA5088-OtA5092	OtA5093-OtA5097
Gm3428	chr9:35848544-35849880	OtA5088-OtA5092	OtA5093-OtA5097
Gm3434	chr9:36134286-36135630	OtA5088-OtA5092	OtA5093-OtA5097
Gm3435	chr17:14964188-15022449	OtA5088-OtA5092	OtA5093-OtA5097
Gm3448	chr17:14964188-15022449	OtA5088-OtA5092	OtA5093-OtA5097
Gm3500	chr14:5718333-5741602	OtA5088-OtA5092	OtA5093-OtA5097

Gm3604	chr13:62367715-62383174	OtA5088-OtA5092	OtA5093-OtA5097
Gm362	chrX:43590988-43593530	OtA5088-OtA5092	OtA5093-OtA5097
Gm364	chrX:57409153-57488767	OtA5088-OtA5092	OtA5093-OtA5097
Gm3646	chr1:39804140-39805332	OtA5088-OtA5092	OtA5093-OtA5097
Gm3696	chr14:7083024-7105457	OtA5088-OtA5092	OtA5093-OtA5097
Gm3701	chrX:3441730-3443690	OtA5088-OtA5092	OtA5093-OtA5097
Gm3706	chrX:34159624-34161584	OtA5088-OtA5092	OtA5093-OtA5097
Gm3716	chr5:64593862-64610699	OtA5088-OtA5092	OtA5093-OtA5097
Gm3750	chrX:34159624-34161584	OtA5088-OtA5092	OtA5093-OtA5097
Gm3763	chrX:4952134-4954077	OtA5088-OtA5092	OtA5093-OtA5097
Gm3776	chr9:78257128-78269166	OtA5088-OtA5092	OtA5093-OtA5097
Gm382	chrX:127039971-127063986	OtA5088-OtA5092	OtA5093-OtA5097
Gm3833	chr14:9550093-11162032	OtA5088-OtA5092	OtA5093-OtA5097
Gm3852	chr1:46011546-46012263	OtA5088-OtA5092	OtA5093-OtA5097
Gm3893	chr4:41889794-42462993	OtA5088-OtA5092	OtA5093-OtA5097
Gm3925	chr5:143164778-143165751	OtA5088-OtA5092	OtA5093-OtA5097
Gm3985	chr8:32888504-32950026	OtA5088-OtA5092	OtA5093-OtA5097
Gm4013	chr18:42274379-42275197	OtA5088-OtA5092	OtA5093-OtA5097
Gm4027	chr12:87618867-87622955	OtA5088-OtA5092	OtA5093-OtA5097
Gm4070,Gvin1	chr7:106156535-106215340	OtA5088-OtA5092	OtA5093-OtA5097
Gm41	chrX:86345474-86354994	OtA5088-OtA5092	OtA5093-OtA5097
Gm410	chr3:57667421-57692537	OtA5088-OtA5092	OtA5093-OtA5097
Gm4133	chr7:22328793-22329717	OtA5088-OtA5092	OtA5093-OtA5097
Gm4172	chr7:22027976-22028897	OtA5088-OtA5092	OtA5093-OtA5097
Gm4175	chr7:22983728-22984673	OtA5088-OtA5092	OtA5093-OtA5097
Gm4177	chr7:23019005-23019923	OtA5088-OtA5092	OtA5093-OtA5097
Gm4179	chr7:23250761-23251682	OtA5088-OtA5092	OtA5093-OtA5097
Gm4201	chr7:22564422-22565343	OtA5088-OtA5092	OtA5093-OtA5097
Gm4224	chr7:12221315-12225807	OtA5088-OtA5092	OtA5093-OtA5097
Gm4251	chr14:71251272-71271059	OtA5088-OtA5092	OtA5093-OtA5097
Gm4262	chr16:11008897-11015184	OtA5088-OtA5092	OtA5093-OtA5097
Gm4265	chr7:129962164-129978669	OtA5088-OtA5092	OtA5093-OtA5097
Gm4278	chr14:74975085-74987104	OtA5088-OtA5092	OtA5093-OtA5097
Gm428	chr4:73683513-73687559	OtA5088-OtA5092	OtA5093-OtA5097
Gm4285	chr14:75842917-75844964	OtA5088-OtA5092	OtA5093-OtA5097
Gm4297	chrX:24552249-24574895	OtA5088-OtA5092	OtA5093-OtA5097
Gm4301	chr10:100366498-100367412	OtA5088-OtA5092	OtA5093-OtA5097
Gm4302	chr10:100345969-100362247	OtA5088-OtA5092	OtA5093-OtA5097
Gm4305	chr10:100345969-100362247	OtA5088-OtA5092	OtA5093-OtA5097
Gm4307	chr10:100345969-100362247	OtA5088-OtA5092	OtA5093-OtA5097
Gm4312	chr10:100381646-100382560	OtA5088-OtA5092	OtA5093-OtA5097
Gm4340	chr10:104142986-104188000	OtA5088-OtA5092	OtA5093-OtA5097
Gm4345	chr17:39536141-39540847	OtA5088-OtA5092	OtA5093-OtA5097
Gm4349	chr3:95427348-95431085	OtA5088-OtA5092	OtA5093-OtA5097
Gm436	chr4:144669936-144686368	OtA5088-OtA5092	OtA5093-OtA5097
Gm4371	chr15:96182799-96203470	OtA5088-OtA5092	OtA5093-OtA5097
Gm438	chr4:144777203-144786583	OtA5088-OtA5092	OtA5093-OtA5097

Gm44	chrX:90892141-90893134	OtA5088-OtA5092	OtA5093-OtA5097
Gm4461	chr17:33215260-33216351	OtA5088-OtA5092	OtA5093-OtA5097
Gm4477	chr6:85707172-85707858	OtA5088-OtA5092	OtA5093-OtA5097
Gm4489	chr10:122119696-122130558	OtA5088-OtA5092	OtA5093-OtA5097
Gm4498	chr7:20911426-20912347	OtA5088-OtA5092	OtA5093-OtA5097
Gm4532	chr7:127232417-127233130	OtA5088-OtA5092	OtA5093-OtA5097
Gm4541	chr7:23151760-23178761	OtA5088-OtA5092	OtA5093-OtA5097
Gm4566	chr17:71333200-71341804	OtA5088-OtA5092	OtA5093-OtA5097
Gm4567	chr7:22713597-22719710	OtA5088-OtA5092	OtA5093-OtA5097
Gm4598	chr7:24661293-24663109	OtA5088-OtA5092	OtA5093-OtA5097
Gm4636	chr7:27984470-28014115	OtA5088-OtA5092	OtA5093-OtA5097
Gm4684	chr7:31703778-31705754	OtA5088-OtA5092	OtA5093-OtA5097
Gm4710	chr17:75861022-75889039	OtA5088-OtA5092	OtA5093-OtA5097
Gm4719	chr17:89379240-89384993	OtA5088-OtA5092	OtA5093-OtA5097
Gm4724	chr2:176470368-176486749	OtA5088-OtA5092	OtA5093-OtA5097
Gm4736	chr6:132114220-132364135	OtA5088-OtA5092	OtA5093-OtA5097
Gm4745	chr7:14659707-14665996	OtA5088-OtA5092	OtA5093-OtA5097
Gm4759	chr7:106421549-106441073	OtA5088-OtA5092	OtA5093-OtA5097
Gm4763	chr7:24722493-24724290	OtA5088-OtA5092	OtA5093-OtA5097
Gm4776	chr1:46020414-46030437	OtA5088-OtA5092	OtA5093-OtA5097
Gm4787	chr12:81376990-81379486	OtA5088-OtA5092	OtA5093-OtA5097
Gm4788	chr1:139697918-139781239	OtA5088-OtA5092	OtA5093-OtA5097
Gm4791	chr9:46998740-47003860	OtA5088-OtA5092	OtA5093-OtA5097
Gm4792	chr10:94293662-94298703	OtA5088-OtA5092	OtA5093-OtA5097
Gm4794	chr10:33766423-33782115	OtA5088-OtA5092	OtA5093-OtA5097
Gm4814	chr13:93040714-93144724	OtA5088-OtA5092	OtA5093-OtA5097
Gm4827	chr16:50019657-50072852	OtA5088-OtA5092	OtA5093-OtA5097
Gm4832	chr17:88125511-88131116	OtA5088-OtA5092	OtA5093-OtA5097
Gm4836	chrX:28931803-28954702	OtA5088-OtA5092	OtA5093-OtA5097
Gm4841	chr18:60268300-60273267	OtA5088-OtA5092	OtA5093-OtA5097
Gm4846	chr1:166483612-166497588	OtA5088-OtA5092	OtA5093-OtA5097
Gm4847	chr1:166628970-166647693	OtA5088-OtA5092	OtA5093-OtA5097
Gm4850	chr1:31264846-31268061	OtA5088-OtA5092	OtA5093-OtA5097
Gm4858	chr3:93068822-93075505	OtA5088-OtA5092	OtA5093-OtA5097
Gm4861	chr3:137550044-137552622	OtA5088-OtA5092	OtA5093-OtA5097
Gm4871	chr5:145029599-145032764	OtA5088-OtA5092	OtA5093-OtA5097
Gm4884	chr7:41032718-41045302	OtA5088-OtA5092	OtA5093-OtA5097
Gm4890	chr8:79295077-79307774	OtA5088-OtA5092	OtA5093-OtA5097
Gm4894	chr9:49250530-49280594	OtA5088-OtA5092	OtA5093-OtA5097
Gm4902	chr14:59320963-59341330	OtA5088-OtA5092	OtA5093-OtA5097
Gm4906	chrX:11327821-11328151	OtA5088-OtA5092	OtA5093-OtA5097
Gm4907	chrX:23892762-23907550	OtA5088-OtA5092	OtA5093-OtA5097
Gm4922	chr10:18779726-18786793	OtA5088-OtA5092	OtA5093-OtA5097
Gm4925	chr10:88729540-88731101	OtA5088-OtA5092	OtA5093-OtA5097
Gm4926	chr11:46636505-46645006	OtA5088-OtA5092	OtA5093-OtA5097
Gm4934	chr13:59712283-59719289	OtA5088-OtA5092	OtA5093-OtA5097
Gm4937	chrX:76264305-76267673	OtA5088-OtA5092	OtA5093-OtA5097

Gm4944	chr17:22197264-22225614	OtA5088-OtA5092	OtA5093-OtA5097
Gm4951	chr18:60212076-60247820	OtA5088-OtA5092	OtA5093-OtA5097
Gm4952	chr19:12599983-12627616	OtA5088-OtA5092	OtA5093-OtA5097
Gm4956	chr1:21285245-21298312	OtA5088-OtA5092	OtA5093-OtA5097
Gm4961	chr5:30092953-30097681	OtA5088-OtA5092	OtA5093-OtA5097
Gm4971	chr7:73685491-73686563	OtA5088-OtA5092	OtA5093-OtA5097
Gm4975	chr8:63924693-63952170	OtA5088-OtA5092	OtA5093-OtA5097
Gm4981	chr10:58234847-58236660	OtA5088-OtA5092	OtA5093-OtA5097
Gm4984	chrX:12651882-12652820	OtA5088-OtA5092	OtA5093-OtA5097
Gm5	chr5:150499746-150502219	OtA5088-OtA5092	OtA5093-OtA5097
Gm5039	chr12:88320121-88321762	OtA5088-OtA5092	OtA5093-OtA5097
Gm5065	chr7:5298546-5360681	OtA5088-OtA5092	OtA5093-OtA5097
Gm5069	chr1:180326968-180423659	OtA5088-OtA5092	OtA5093-OtA5097
Gm5071	chrX:91931945-91932980	OtA5088-OtA5092	OtA5093-OtA5097
Gm5072	chrX:91480125-91481160	OtA5088-OtA5092	OtA5093-OtA5097
Gm5077	chr6:124624624-124636085	OtA5088-OtA5092	OtA5093-OtA5097
Gm5082	chr13:41649442-41656777	OtA5088-OtA5092	OtA5093-OtA5097
Gm5083	chr13:44121166-44125179	OtA5088-OtA5092	OtA5093-OtA5097
Gm5084	chr13:60205470-60216018	OtA5088-OtA5092	OtA5093-OtA5097
Gm5086	chr13:97559999-97583994	OtA5088-OtA5092	OtA5093-OtA5097
Gm5087	chr14:13157387-13284638	OtA5088-OtA5092	OtA5093-OtA5097
Gm5088	chr14:89896223-89899447	OtA5088-OtA5092	OtA5093-OtA5097
Gm5089	chr14:122181693-122451146	OtA5088-OtA5092	OtA5093-OtA5097
Gm5091	chr17:15231584-15240783	OtA5088-OtA5092	OtA5093-OtA5097
Gm5095	chr18:47537734-47718636	OtA5088-OtA5092	OtA5093-OtA5097
Gm5105	chr3:138048760-138067388	OtA5088-OtA5092	OtA5093-OtA5097
Gm5108	chr5:67933611-67977070	OtA5088-OtA5092	OtA5093-OtA5097
Gm5111	chr6:48589444-48590584	OtA5088-OtA5092	OtA5093-OtA5097
Gm5113	chr7:30169921-30180209	OtA5088-OtA5092	OtA5093-OtA5097
Gm5114	chr7:39407293-39413160	OtA5088-OtA5092	OtA5093-OtA5097
Gm5122	chr9:61017068-61019797	OtA5088-OtA5092	OtA5093-OtA5097
Gm5124	chrX:21360864-21364624	OtA5088-OtA5092	OtA5093-OtA5097
Gm5126	chrX:102932046-102934223	OtA5088-OtA5092	OtA5093-OtA5097
Gm5127	chrX:106576516-106710557	OtA5088-OtA5092	OtA5093-OtA5097
Gm5128	chrX:135373283-135383393	OtA5088-OtA5092	OtA5093-OtA5097
Gm5129	chr5:29735333-29786478	OtA5088-OtA5092	OtA5093-OtA5097
Gm5132	chrX:9571959-9572391	OtA5088-OtA5092	OtA5093-OtA5097
Gm5134	chr10:75954513-76009589	OtA5088-OtA5092	OtA5093-OtA5097
Gm5136	chr10:108497649-109010975	OtA5088-OtA5092	OtA5093-OtA5097
Gm514	chr9:65361059-65380377	OtA5088-OtA5092	OtA5093-OtA5097
Gm5141	chr13:62772199-62785808	OtA5088-OtA5092	OtA5093-OtA5097
Gm5142	chr14:59158502-59178749	OtA5088-OtA5092	OtA5093-OtA5097
Gm5148	chr3:37714189-37724360	OtA5088-OtA5092	OtA5093-OtA5097
Gm5150	chr3:15948069-16006332	OtA5088-OtA5092	OtA5093-OtA5097
Gm5151	chr4:155734803-155740564	OtA5088-OtA5092	OtA5093-OtA5097
Gm5166	chrX:102791195-102798389	OtA5088-OtA5092	OtA5093-OtA5097
Gm5168	chrX:26028213-26051041	OtA5088-OtA5092	OtA5093-OtA5097

Gm5169	chrX:25277482-25301455	OtA5088-OtA5092	OtA5093-OtA5097
Gm5176	chr10:111500787-111501345	OtA5088-OtA5092	OtA5093-OtA5097
Gm5177	chr10:10605944-10616313	OtA5088-OtA5092	OtA5093-OtA5097
Gm525	chr11:89073840-89093064	OtA5088-OtA5092	OtA5093-OtA5097
Gm527	chr12:64917910-64924591	OtA5088-OtA5092	OtA5093-OtA5097
Gm5294	chr5:138820079-138821619	OtA5088-OtA5092	OtA5093-OtA5097
Gm53	chr11:96251659-96264484	OtA5088-OtA5092	OtA5093-OtA5097
Gm5325	chr7:31375591-31376916	OtA5088-OtA5092	OtA5093-OtA5097
Gm5326	chr7:32441540-32442844	OtA5088-OtA5092	OtA5093-OtA5097
Gm5334	chr7:68618455-68620009	OtA5088-OtA5092	OtA5093-OtA5097
Gm5346	chr8:43624974-43627185	OtA5088-OtA5092	OtA5093-OtA5097
Gm5347	chr8:43673657-43699491	OtA5088-OtA5092	OtA5093-OtA5097
Gm5382	chrX:14211147-14211657	OtA5088-OtA5092	OtA5093-OtA5097
Gm5409	chr6:41415306-41419593	OtA5088-OtA5092	OtA5093-OtA5097
Gm5414	chr15:101624027-101628188	OtA5088-OtA5092	OtA5093-OtA5097
Gm5415	chr1:32172805-32657738	OtA5088-OtA5092	OtA5093-OtA5097
Gm5416	chr16:36210402-36217788	OtA5088-OtA5092	OtA5093-OtA5097
Gm5420	chr10:21690844-21693978	OtA5088-OtA5092	OtA5093-OtA5097
Gm5431	chr11:48887421-48902152	OtA5088-OtA5092	OtA5093-OtA5097
Gm5434	chr12:36090378-36091829	OtA5088-OtA5092	OtA5093-OtA5097
Gm5441	chr12:116485719-117337012	OtA5088-OtA5092	OtA5093-OtA5097
Gm5458	chr14:19594137-19602587	OtA5088-OtA5092	OtA5093-OtA5097
Gm5460	chr14:34041077-34046981	OtA5088-OtA5092	OtA5093-OtA5097
Gm5464	chr14:66868849-66871005	OtA5088-OtA5092	OtA5093-OtA5097
Gm5465	chr14:79451834-79454816	OtA5088-OtA5092	OtA5093-OtA5097
Gm5468	chr15:25414191-25452418	OtA5088-OtA5092	OtA5093-OtA5097
Gm5475	chr15:100423192-100428150	OtA5088-OtA5092	OtA5093-OtA5097
Gm5476	chr15:101587316-101588572	OtA5088-OtA5092	OtA5093-OtA5097
Gm5477	chr15:101608873-101610479	OtA5088-OtA5092	OtA5093-OtA5097
Gm5478	chr15:101643019-101647380	OtA5088-OtA5092	OtA5093-OtA5097
Gm5483	chr16:36184211-36188110	OtA5088-OtA5092	OtA5093-OtA5097
Gm5485	chr16:48399452-48412416	OtA5088-OtA5092	OtA5093-OtA5097
Gm5494	chr17:40561506-40565624	OtA5088-OtA5092	OtA5093-OtA5097
Gm5506	chr4:150237196-150248873	OtA5088-OtA5092	OtA5093-OtA5097
Gm5512	chr19:12905230-12906985	OtA5088-OtA5092	OtA5093-OtA5097
Gm5523	chr1:13785684-13786929	OtA5088-OtA5092	OtA5093-OtA5097
Gm5531	chr1:153874344-153876871	OtA5088-OtA5092	OtA5093-OtA5097
Gm5535	chr2:144173149-144189290	OtA5088-OtA5092	OtA5093-OtA5097
Gm5538	chr3:59729785-59752397	OtA5088-OtA5092	OtA5093-OtA5097
Gm5544	chr3:97930172-97967018	OtA5088-OtA5092	OtA5093-OtA5097
Gm5547	chr3:105815566-105817359	OtA5088-OtA5092	OtA5093-OtA5097
Gm5567	chr6:40110252-40248353	OtA5088-OtA5092	OtA5093-OtA5097
Gm5577	chr6:87981682-87984180	OtA5088-OtA5092	OtA5093-OtA5097
Gm5591	chr7:38518138-38528193	OtA5088-OtA5092	OtA5093-OtA5097
Gm5592	chr7:41284326-41290183	OtA5088-OtA5092	OtA5093-OtA5097
Gm5595	chr7:42660107-42692718	OtA5088-OtA5092	OtA5093-OtA5097
Gm5607	chr8:12385770-12436732	OtA5088-OtA5092	OtA5093-OtA5097

Gm5615	chr9:36532402-36541963	OtA5088-OtA5092	OtA5093-OtA5097
Gm5622	chr14:51552789-51662953	OtA5088-OtA5092	OtA5093-OtA5097
Gm5627	chr9:102739647-102756685	OtA5088-OtA5092	OtA5093-OtA5097
Gm5634	chrX:8962133-8962975	OtA5088-OtA5092	OtA5093-OtA5097
Gm5635	chrX:9063089-9063822	OtA5088-OtA5092	OtA5093-OtA5097
Gm5640	chrX:74639118-74645635	OtA5088-OtA5092	OtA5093-OtA5097
Gm5643	chrX:142297504-142299237	OtA5088-OtA5092	OtA5093-OtA5097
Gm5662	chr12:88270634-88274497	OtA5088-OtA5092	OtA5093-OtA5097
Gm5712	chr3:129433672-129434712	OtA5088-OtA5092	OtA5093-OtA5097
Gm572	chr4:148643316-148671572	OtA5088-OtA5092	OtA5093-OtA5097
Gm5728	chr7:22370121-22371039	OtA5088-OtA5092	OtA5093-OtA5097
Gm5741	chr8:85067567-85067982	OtA5088-OtA5092	OtA5093-OtA5097
Gm5766	chr16:4229228-4231205	OtA5088-OtA5092	OtA5093-OtA5097
Gm5771	chr6:41392355-41397256	OtA5088-OtA5092	OtA5093-OtA5097
Gm5779	chr10:75352054-75352961	OtA5088-OtA5092	OtA5093-OtA5097
Gm5796	chr14:4023940-4041368	OtA5088-OtA5092	OtA5093-OtA5097
Gm5797	chr14:7323987-7332395	OtA5088-OtA5092	OtA5093-OtA5097
Gm5800	chr14:51711643-51717132	OtA5088-OtA5092	OtA5093-OtA5097
Gm5801	chr4:155943830-155959604	OtA5088-OtA5092	OtA5093-OtA5097
Gm5803	chr15:22713819-22714984	OtA5088-OtA5092	OtA5093-OtA5097
Gm581	chr7:45176348-45179597	OtA5088-OtA5092	OtA5093-OtA5097
Gm5820	chr18:38838672-38971274	OtA5088-OtA5092	OtA5093-OtA5097
Gm5833	chr1:138640094-138682075	OtA5088-OtA5092	OtA5093-OtA5097
Gm5860	chr4:82065379-82102807	OtA5088-OtA5092	OtA5093-OtA5097
Gm5868	chr5:72581638-72587550	OtA5088-OtA5092	OtA5093-OtA5097
Gm5878	chr6:85111415-85126094	OtA5088-OtA5092	OtA5093-OtA5097
Gm5885	chr6:133529188-133532762	OtA5088-OtA5092	OtA5093-OtA5097
Gm5886	chr6:133763998-133767403	OtA5088-OtA5092	OtA5093-OtA5097
Gm5887	chr6:147075061-147087032	OtA5088-OtA5092	OtA5093-OtA5097
Gm5891	chr7:23132622-23144480	OtA5088-OtA5092	OtA5093-OtA5097
Gm5894	chr7:33278365-33280341	OtA5088-OtA5092	OtA5093-OtA5097
Gm590	chr9:110914738-110916861	OtA5088-OtA5092	OtA5093-OtA5097
Gm5901	chr7:105371210-105400054	OtA5088-OtA5092	OtA5093-OtA5097
Gm5916	chr9:36119933-36128779	OtA5088-OtA5092	OtA5093-OtA5097
Gm5925	chrX:4515588-4517543	OtA5088-OtA5092	OtA5093-OtA5097
Gm5934	chrX:24474307-24500760	OtA5088-OtA5092	OtA5093-OtA5097
Gm5935	chrX:24753161-24775164	OtA5088-OtA5092	OtA5093-OtA5097
Gm5936	chrX:74837195-74843355	OtA5088-OtA5092	OtA5093-OtA5097
Gm5938	chrX:78125466-78130403	OtA5088-OtA5092	OtA5093-OtA5097
Gm5941	chrX:92118878-92599572	OtA5088-OtA5092	OtA5093-OtA5097
Gm595	chrX:48841465-48877713	OtA5088-OtA5092	OtA5093-OtA5097
Gm597	chr1:28776121-28780252	OtA5088-OtA5092	OtA5093-OtA5097
Gm600	chr14:65605266-65648443	OtA5088-OtA5092	OtA5093-OtA5097
Gm6026	chrY:2720338-2720674	OtA5088-OtA5092	OtA5093-OtA5097
Gm6034	chr17:36042960-36058645	OtA5088-OtA5092	OtA5093-OtA5097
Gm6040	chr8:20916489-20922071	OtA5088-OtA5092	OtA5093-OtA5097
Gm6042	chr15:101503815-101506313	OtA5088-OtA5092	OtA5093-OtA5097

Gm606	chr16:26957234-26989974	OtA5088-OtA5092	OtA5093-OtA5097
Gm6084	chr17:40727119-40731782	OtA5088-OtA5092	OtA5093-OtA5097
Gm6086	chr1:93990508-94011539	OtA5088-OtA5092	OtA5093-OtA5097
Gm609	chr16:45416754-45492969	OtA5088-OtA5092	OtA5093-OtA5097
Gm6116	chr5:74949193-74966368	OtA5088-OtA5092	OtA5093-OtA5097
Gm6121	chrX:26912743-26935669	OtA5088-OtA5092	OtA5093-OtA5097
Gm614	chrX:101261376-101263992	OtA5088-OtA5092	OtA5093-OtA5097
Gm6150	chr10:9684721-9691909	OtA5088-OtA5092	OtA5093-OtA5097
Gm6164	chr7:22200340-22201264	OtA5088-OtA5092	OtA5093-OtA5097
Gm6194	chr3:8461386-8463098	OtA5088-OtA5092	OtA5093-OtA5097
Gm6213	chr8:39297979-39368268	OtA5088-OtA5092	OtA5093-OtA5097
Gm6222	chrX:103697413-103821988	OtA5088-OtA5092	OtA5093-OtA5097
Gm6225	chr18:3266353-3366863	OtA5088-OtA5092	OtA5093-OtA5097
Gm6249	chr7:136667954-136710604	OtA5088-OtA5092	OtA5093-OtA5097
Gm6260	chr3:143670891-143712143	OtA5088-OtA5092	OtA5093-OtA5097
Gm6268	chrX:36403476-36404654	OtA5088-OtA5092	OtA5093-OtA5097
Gm6277	chr18:11821087-11945627	OtA5088-OtA5092	OtA5093-OtA5097
Gm628	chr15:73786935-73839671	OtA5088-OtA5092	OtA5093-OtA5097
Gm6289	chr3:144879324-144896011	OtA5088-OtA5092	OtA5093-OtA5097
Gm6300	chr3:14363209-14377566	OtA5088-OtA5092	OtA5093-OtA5097
Gm6307	chr2:180370857-180401802	OtA5088-OtA5092	OtA5093-OtA5097
Gm6313	chr6:148606785-148614309	OtA5088-OtA5092	OtA5093-OtA5097
Gm6320	chr13:112993867-113000394	OtA5088-OtA5092	OtA5093-OtA5097
Gm6329	chr8:45160493-45165145	OtA5088-OtA5092	OtA5093-OtA5097
Gm6367	chr5_JH584299_random:587624-62551	OtA5088-OtA5092	OtA5093-OtA5097
Gm6370	chr5:146491362-146494132	OtA5088-OtA5092	OtA5093-OtA5097
Gm6377	chrX:109196755-109200445	OtA5088-OtA5092	OtA5093-OtA5097
Gm6402	chr17:30215523-30576287	OtA5088-OtA5092	OtA5093-OtA5097
Gm6406	chr9:98856590-98857352	OtA5088-OtA5092	OtA5093-OtA5097
Gm6408	chr5:146481942-146484702	OtA5088-OtA5092	OtA5093-OtA5097
Gm6416	chr13:117130024-117135884	OtA5088-OtA5092	OtA5093-OtA5097
Gm6432	chr9:99229375-99237239	OtA5088-OtA5092	OtA5093-OtA5097
Gm6455	chr5:10865028-10870808	OtA5088-OtA5092	OtA5093-OtA5097
Gm6460	chr5:11594955-11599480	OtA5088-OtA5092	OtA5093-OtA5097
Gm6484	chr9:21800179-21852635	OtA5088-OtA5092	OtA5093-OtA5097
Gm6498	chr14:48120868-48260883	OtA5088-OtA5092	OtA5093-OtA5097
Gm6524	chr8:11692981-11694514	OtA5088-OtA5092	OtA5093-OtA5097
Gm6525	chr3:84161873-84220877	OtA5088-OtA5092	OtA5093-OtA5097
Gm6531	chr8:85151923-85173622	OtA5088-OtA5092	OtA5093-OtA5097
Gm6537	chr7:28756173-28766524	OtA5088-OtA5092	OtA5093-OtA5097
Gm6567	chr7:73097751-73102068	OtA5088-OtA5092	OtA5093-OtA5097
Gm6568	chrX:157540684-157541782	OtA5088-OtA5092	OtA5093-OtA5097
Gm6578	chr6:12099520-12109580	OtA5088-OtA5092	OtA5093-OtA5097
Gm6583	chr5:112343094-112378424	OtA5088-OtA5092	OtA5093-OtA5097
Gm6588	chr5:112419150-112577198	OtA5088-OtA5092	OtA5093-OtA5097
Gm6592	chrX:8843993-8849607	OtA5088-OtA5092	OtA5093-OtA5097
Gm6602	chr3:111881902-112082001	OtA5088-OtA5092	OtA5093-OtA5097

Gm6607	chr9:22055459-22389206	OtA5088-OtA5092	OtA5093-OtA5097
Gm6614	chr6:141972195-142008745	OtA5088-OtA5092	OtA5093-OtA5097
Gm6623	chr17:36178776-36181364	OtA5088-OtA5092	OtA5093-OtA5097
Gm6634	chr3:70772378-70807291	OtA5088-OtA5092	OtA5093-OtA5097
Gm6639	chr3:35597151-35666163	OtA5088-OtA5092	OtA5093-OtA5097
Gm6642	chr19:30564486-31765033	OtA5088-OtA5092	OtA5093-OtA5097
Gm6654	chr6:146642578-146650600	OtA5088-OtA5092	OtA5093-OtA5097
Gm6682	chr12:4782169-4783512	OtA5088-OtA5092	OtA5093-OtA5097
Gm6696	chr8:21583026-21583867	OtA5088-OtA5092	OtA5093-OtA5097
Gm6710	chr2:175869237-175988574	OtA5088-OtA5092	OtA5093-OtA5097
Gm6760	chrX:64151404-64152198	OtA5088-OtA5092	OtA5093-OtA5097
Gm6772	chr12:86734368-86763795	OtA5088-OtA5092	OtA5093-OtA5097
Gm6787	chrX:8097086-8106701	OtA5088-OtA5092	OtA5093-OtA5097
Gm6792	chr7:6252709-6281861	OtA5088-OtA5092	OtA5093-OtA5097
Gm6812	chrX:68892372-68893053	OtA5088-OtA5092	OtA5093-OtA5097
Gm684	chr9:51270257-51278554	OtA5088-OtA5092	OtA5093-OtA5097
Gm6878	chr14:67233291-67429767	OtA5088-OtA5092	OtA5093-OtA5097
Gm6880	chrX:74480286-74481399	OtA5088-OtA5092	OtA5093-OtA5097
Gm6890	chrX:74740926-74742188	OtA5088-OtA5092	OtA5093-OtA5097
Gm6904	chr14:59244440-59260216	OtA5088-OtA5092	OtA5093-OtA5097
Gm6907	chr14:59380832-59393512	OtA5088-OtA5092	OtA5093-OtA5097
Gm6927	chrX:77674813-77675452	OtA5088-OtA5092	OtA5093-OtA5097
Gm6936	chr16:49980459-49997475	OtA5088-OtA5092	OtA5093-OtA5097
Gm6938	chrX:21312209-21334727	OtA5088-OtA5092	OtA5093-OtA5097
Gm694	chr4:141432671-141436105	OtA5088-OtA5092	OtA5093-OtA5097
Gm6981	chr9:52002060-52088738	OtA5088-OtA5092	OtA5093-OtA5097
Gm6994	chr14:77156762-77721763	OtA5088-OtA5092	OtA5093-OtA5097
Gm7008	chr12:40223363-40229183	OtA5088-OtA5092	OtA5093-OtA5097
Gm7030	chr17:36127608-36129425	OtA5088-OtA5092	OtA5093-OtA5097
Gm7056	chr5:89616435-89622611	OtA5088-OtA5092	OtA5093-OtA5097
Gm7073	chrX:60436051-60456195	OtA5088-OtA5092	OtA5093-OtA5097
Gm7092	chr7:25614620-25615892	OtA5088-OtA5092	OtA5093-OtA5097
Gm7102	chr19:61174685-61176309	OtA5088-OtA5092	OtA5093-OtA5097
Gm7104	chr12:88282866-88287070	OtA5088-OtA5092	OtA5093-OtA5097
Gm711	chr2:26934068-26953496	OtA5088-OtA5092	OtA5093-OtA5097
Gm7134	chrX:110372752-110375156	OtA5088-OtA5092	OtA5093-OtA5097
Gm7157	chrX:152563333-152563969	OtA5088-OtA5092	OtA5093-OtA5097
Gm7168	chr17:13948372-13950678	OtA5088-OtA5092	OtA5093-OtA5097
Gm7173	chrX:79482567-79517285	OtA5088-OtA5092	OtA5093-OtA5097
Gm7244	chr9:31271400-31275385	OtA5088-OtA5092	OtA5093-OtA5097
Gm7257	chr9:36431883-36434938	OtA5088-OtA5092	OtA5093-OtA5097
Gm7271	chr5:76484076-76516628	OtA5088-OtA5092	OtA5093-OtA5097
Gm732	chrX:107945734-107948436	OtA5088-OtA5092	OtA5093-OtA5097
Gm7325	chr17:45600966-45602102	OtA5088-OtA5092	OtA5093-OtA5097
Gm7337	chr5:87850358-87853012	OtA5088-OtA5092	OtA5093-OtA5097
Gm7444	chr9:56027635-56031573	OtA5088-OtA5092	OtA5093-OtA5097
Gm7457	chr6:142814230-142964452	OtA5088-OtA5092	OtA5093-OtA5097

Gm749	chr17:28549486-28552618	OtA5088-OtA5092	OtA5093-OtA5097
Gm7534	chr4:134190803-134203004	OtA5088-OtA5092	OtA5093-OtA5097
Gm7538	chr5:119194312-119200018	OtA5088-OtA5092	OtA5093-OtA5097
Gm7550	chr12:54387732-54391318	OtA5088-OtA5092	OtA5093-OtA5097
Gm757	chr2:25234475-25236787	OtA5088-OtA5092	OtA5093-OtA5097
Gm7609	chr1:85199610-85213762	OtA5088-OtA5092	OtA5093-OtA5097
Gm7616	chr9:59951293-59959044	OtA5088-OtA5092	OtA5093-OtA5097
Gm765	chr6:98238013-98342754	OtA5088-OtA5092	OtA5093-OtA5097
Gm766	chr6:142785780-142804466	OtA5088-OtA5092	OtA5093-OtA5097
Gm7714	chr5:88268918-88282835	OtA5088-OtA5092	OtA5093-OtA5097
Gm773	chrX:56189826-56212881	OtA5088-OtA5092	OtA5093-OtA5097
Gm7849,Gm7861	chr8:21593374-21594465	OtA5088-OtA5092	OtA5093-OtA5097
Gm7854	chr5:43151685-43289724	OtA5088-OtA5092	OtA5093-OtA5097
Gm7861	chr8:21593374-21594465	OtA5088-OtA5092	OtA5093-OtA5097
Gm7904	chr9:21607083-21608329	OtA5088-OtA5092	OtA5093-OtA5097
Gm7977	chr3:48026011-48026759	OtA5088-OtA5092	OtA5093-OtA5097
Gm805	chr12:86169340-86195102	OtA5088-OtA5092	OtA5093-OtA5097
Gm806	chr13:50467306-50475355	OtA5088-OtA5092	OtA5093-OtA5097
Gm8096	chr8:28083193-28084853	OtA5088-OtA5092	OtA5093-OtA5097
Gm813	chr16:58613685-58616978	OtA5088-OtA5092	OtA5093-OtA5097
Gm815	chr19:26885924-26888647	OtA5088-OtA5092	OtA5093-OtA5097
Gm8179	chr8:32151894-32181847	OtA5088-OtA5092	OtA5093-OtA5097
Gm8221	chr15:77618967-77627398	OtA5088-OtA5092	OtA5093-OtA5097
Gm8234	chr3:58652041-58654041	OtA5088-OtA5092	OtA5093-OtA5097
Gm826	chr2:160311400-160327494	OtA5088-OtA5092	OtA5093-OtA5097
Gm8267	chr14:44717166-44724987	OtA5088-OtA5092	OtA5093-OtA5097
Gm829	chr4:45683588-45723706	OtA5088-OtA5092	OtA5093-OtA5097
Gm8298	chr3:59861050-59877313	OtA5088-OtA5092	OtA5093-OtA5097
Gm8300	chr12:87514315-87518265	OtA5088-OtA5092	OtA5093-OtA5097
Gm833	chr4:152697539-152700922	OtA5088-OtA5092	OtA5093-OtA5097
Gm8348	chr14:3652029-3673272	OtA5088-OtA5092	OtA5093-OtA5097
Gm8369	chr19:11492037-11512577	OtA5088-OtA5092	OtA5093-OtA5097
Gm839	chr6:89212196-89216179	OtA5088-OtA5092	OtA5093-OtA5097
Gm8439	chr4:120588744-120609672	OtA5088-OtA5092	OtA5093-OtA5097
Gm853	chr4:130209108-130222295	OtA5088-OtA5092	OtA5093-OtA5097
Gm8579	chr6:3288518-3317019	OtA5088-OtA5092	OtA5093-OtA5097
Gm8580	chr10:93641048-93745972	OtA5088-OtA5092	OtA5093-OtA5097
Gm8615	chr5:149119564-149182036	OtA5088-OtA5092	OtA5093-OtA5097
Gm8633	chr10:98299926-98351867	OtA5088-OtA5092	OtA5093-OtA5097
Gm8660	chr7:20374492-20375410	OtA5088-OtA5092	OtA5093-OtA5097
Gm8677	chr7:22533963-22534887	OtA5088-OtA5092	OtA5093-OtA5097
Gm8693	chr7:22691396-22692317	OtA5088-OtA5092	OtA5093-OtA5097
Gm8709	chr10:26673445-26674651	OtA5088-OtA5092	OtA5093-OtA5097
Gm872	chr10:92796756-92836346	OtA5088-OtA5092	OtA5093-OtA5097
Gm8720	chr7:23066532-23067456	OtA5088-OtA5092	OtA5093-OtA5097
Gm8765	chr13:50698328-50703409	OtA5088-OtA5092	OtA5093-OtA5097
Gm8773	chr5:5573798-5576203	OtA5088-OtA5092	OtA5093-OtA5097

Gm8787	chrX:79330512-79358068	OtA5088-OtA5092	OtA5093-OtA5097
Gm88	chr17:46254164-46257316	OtA5088-OtA5092	OtA5093-OtA5097
Gm8801	chr17:35947153-35953317	OtA5088-OtA5092	OtA5093-OtA5097
Gm8817	chrX:167063448-167070370	OtA5088-OtA5092	OtA5093-OtA5097
Gm884	chr11:103534576-103614739	OtA5088-OtA5092	OtA5093-OtA5097
Gm885	chr11:106751267-106767568	OtA5088-OtA5092	OtA5093-OtA5097
Gm8882	chr6:132114220-132364135	OtA5088-OtA5092	OtA5093-OtA5097
Gm8884	chr9:48454343-48454727	OtA5088-OtA5092	OtA5093-OtA5097
Gm889	chr12:40176385-40199315	OtA5088-OtA5092	OtA5093-OtA5097
Gm8898	chr2:175869237-175988574	OtA5088-OtA5092	OtA5093-OtA5097
Gm8909	chr17:36164443-36168537	OtA5088-OtA5092	OtA5093-OtA5097
Gm8979	chr7:106075730-106079148	OtA5088-OtA5092	OtA5093-OtA5097
Gm8989	chr7:106322395-106324528	OtA5088-OtA5092	OtA5093-OtA5097
Gm8994	chr6:136327538-136329983	OtA5088-OtA5092	OtA5093-OtA5097
Gm904	chr13:50643227-50645838	OtA5088-OtA5092	OtA5093-OtA5097
Gm9041	chr17:39640956-39645667	OtA5088-OtA5092	OtA5093-OtA5097
Gm9047	chr6:29471436-29473429	OtA5088-OtA5092	OtA5093-OtA5097
Gm9054	chr3:95985509-95986967	OtA5088-OtA5092	OtA5093-OtA5097
Gm906	chr13:50245180-50250308	OtA5088-OtA5092	OtA5093-OtA5097
Gm9079	chr10:122078111-122079879	OtA5088-OtA5092	OtA5093-OtA5097
Gm9125	chr3:94047980-94054885	OtA5088-OtA5092	OtA5093-OtA5097
Gm9159	chrX:103545499-103547699	OtA5088-OtA5092	OtA5093-OtA5097
Gm9199	chr14:73025602-73026890	OtA5088-OtA5092	OtA5093-OtA5097
Gm9268	chr7:43018797-43048106	OtA5088-OtA5092	OtA5093-OtA5097
Gm9320	chr17:70875808-70876313	OtA5088-OtA5092	OtA5093-OtA5097
Gm933	chr16:32804887-32810446	OtA5088-OtA5092	OtA5093-OtA5097
Gm9376	chr14:118267157-118267770	OtA5088-OtA5092	OtA5093-OtA5097
Gm94	chr18:43777195-43792878	OtA5088-OtA5092	OtA5093-OtA5097
Gm949	chr18:46921814-46934222	OtA5088-OtA5092	OtA5093-OtA5097
Gm9513	chr9:36475637-36477180	OtA5088-OtA5092	OtA5093-OtA5097
Gm9524	chr9:122747345-122802074	OtA5088-OtA5092	OtA5093-OtA5097
Gm9573	chr17:35617922-35626637	OtA5088-OtA5092	OtA5093-OtA5097
Gm960	chr19:4625840-4698668	OtA5088-OtA5092	OtA5093-OtA5097
Gm9696	chr3:59952308-59973594	OtA5088-OtA5092	OtA5093-OtA5097
Gm97	chr19:11697054-11711858	OtA5088-OtA5092	OtA5093-OtA5097
Gm9705	chr17:32621318-32636184	OtA5088-OtA5092	OtA5093-OtA5097
Gm973	chr1:59516263-59634509	OtA5088-OtA5092	OtA5093-OtA5097
Gm9731	chr8:27296044-27297200	OtA5088-OtA5092	OtA5093-OtA5097
Gm9733	chr3:15296550-15332302	OtA5088-OtA5092	OtA5093-OtA5097
Gm9758	chr5:14910123-14914889	OtA5088-OtA5092	OtA5093-OtA5097
Gm9767	chr10:25991185-26079447	OtA5088-OtA5092	OtA5093-OtA5097
Gm9833	chr3:10088276-10092562	OtA5088-OtA5092	OtA5093-OtA5097
Gm9839	chr1:32172805-32657738	OtA5088-OtA5092	OtA5093-OtA5097
Gm9866	chr12:27140795-27160516	OtA5088-OtA5092	OtA5093-OtA5097
Gm9871	chr6:101774248-101801982	OtA5088-OtA5092	OtA5093-OtA5097
Gm9895	chr19:29067300-29069503	OtA5088-OtA5092	OtA5093-OtA5097
Gm9897	chr5:4192366-4197651	OtA5088-OtA5092	OtA5093-OtA5097

Gm9899	chr5:30573986-30625270	OtA5088-OtA5092	OtA5093-OtA5097
Gm9920	chr15:55099916-55254202	OtA5088-OtA5092	OtA5093-OtA5097
Gm9926	chr18:66504177-66511737	OtA5088-OtA5092	OtA5093-OtA5097
Gm9958	chr5:90366996-90368488	OtA5088-OtA5092	OtA5093-OtA5097
Gm996	chr2:25575415-25580099	OtA5088-OtA5092	OtA5093-OtA5097
Gm9961	chr16:11803720-11930594	OtA5088-OtA5092	OtA5093-OtA5097
Gm9962	chr7:57387271-57510009	OtA5088-OtA5092	OtA5093-OtA5097
Gm9994	chr1:93918436-93941239	OtA5088-OtA5092	OtA5093-OtA5097
Gm9999	chr7:46976631-46987803	OtA5088-OtA5092	OtA5093-OtA5097
Gmcl1l	chrX:32560054-32562009	OtA5088-OtA5092	OtA5093-OtA5097
Gml	chr15:74813454-74818815	OtA5088-OtA5092	OtA5093-OtA5097
Gna15	chr10:81502313-81524225	OtA5088-OtA5092	OtA5093-OtA5097
Gnal	chr18:67088335-67245830	OtA5088-OtA5092	OtA5093-OtA5097
Gnat1	chr9:107674473-107679592	OtA5088-OtA5092	OtA5093-OtA5097
Gnat2	chr3:108093067-108101285	OtA5088-OtA5092	OtA5093-OtA5097
Gnat3	chr5:17962569-18019668	OtA5088-OtA5092	OtA5093-OtA5097
Gnb1l	chr16:18498767-18566679	OtA5088-OtA5092	OtA5093-OtA5097
Gnb3	chr6:124834239-124840275	OtA5088-OtA5092	OtA5093-OtA5097
Gng13	chr17:25717171-25727415	OtA5088-OtA5092	OtA5093-OtA5097
Gng2	chr14:19872558-19977249	OtA5088-OtA5092	OtA5093-OtA5097
Gng3	chr19:8836928-8848683	OtA5088-OtA5092	OtA5093-OtA5097
Gng4	chr13:13784538-13827895	OtA5088-OtA5092	OtA5093-OtA5097
Gng7	chr10:80948623-81014925	OtA5088-OtA5092	OtA5093-OtA5097
Gng8	chr7:16891785-16895435	OtA5088-OtA5092	OtA5093-OtA5097
Gngt1	chr6:3994011-3997436	OtA5088-OtA5092	OtA5093-OtA5097
Gnmt	chr17:46725663-46729165	OtA5088-OtA5092	OtA5093-OtA5097
Gnrh1	chr14:67745228-67749436	OtA5088-OtA5092	OtA5093-OtA5097
Gnrhr	chr5:86181997-86197870	OtA5088-OtA5092	OtA5093-OtA5097
Got1l1	chr8:27197458-27202547	OtA5088-OtA5092	OtA5093-OtA5097
Gp1ba	chr11:70639121-70642055	OtA5088-OtA5092	OtA5093-OtA5097
Gp2	chr7:119442543-119459272	OtA5088-OtA5092	OtA5093-OtA5097
Gp49a	chr10:51480678-51486219	OtA5088-OtA5092	OtA5093-OtA5097
Gp5	chr16:30307692-30310779	OtA5088-OtA5092	OtA5093-OtA5097
Gp6	chr7:4368712-4397744	OtA5088-OtA5092	OtA5093-OtA5097
Gpa33	chr1:166130459-166166510	OtA5088-OtA5092	OtA5093-OtA5097
Gpbar1	chr1:74278599-74279589	OtA5088-OtA5092	OtA5093-OtA5097
Gpc5	chr14:115092214-116525192	OtA5088-OtA5092	OtA5093-OtA5097
Gpd1	chr15:99717592-99725007	OtA5088-OtA5092	OtA5093-OtA5097
Gpha2	chr19:6226400-6235840	OtA5088-OtA5092	OtA5093-OtA5097
Gphb5	chr12:75411719-75416781	OtA5088-OtA5092	OtA5093-OtA5097
Gpld1	chr13:24943151-24991936	OtA5088-OtA5092	OtA5093-OtA5097
Gpm6a	chr8:54779433-55060878	OtA5088-OtA5092	OtA5093-OtA5097
Gpr101	chrX:57496667-57503757	OtA5088-OtA5092	OtA5093-OtA5097
Gpr110	chr17:43270346-43324406	OtA5088-OtA5092	OtA5093-OtA5097
Gpr111	chr17:42708936-42742179	OtA5088-OtA5092	OtA5093-OtA5097
Gpr113	chr5:30193430-30205722	OtA5088-OtA5092	OtA5093-OtA5097
Gpr114	chr8:94923693-94943290	OtA5088-OtA5092	OtA5093-OtA5097

Gpr119	chrX:48667978-48674478	OtA5088-OtA5092	OtA5093-OtA5097
Gpr12	chr5:146582149-146585239	OtA5088-OtA5092	OtA5093-OtA5097
Gpr123	chr7:139834173-139878088	OtA5088-OtA5092	OtA5093-OtA5097
Gpr128	chr16:56724608-56795858	OtA5088-OtA5092	OtA5093-OtA5097
Gpr132	chr12:112850875-112860916	OtA5088-OtA5092	OtA5093-OtA5097
Gpr135	chr12:72069617-72070991	OtA5088-OtA5092	OtA5093-OtA5097
Gpr137c	chr14:45219716-45280976	OtA5088-OtA5092	OtA5093-OtA5097
Gpr139	chr7:119144322-119184374	OtA5088-OtA5092	OtA5093-OtA5097
Gpr141	chr13:19749681-19824257	OtA5088-OtA5092	OtA5093-OtA5097
Gpr142	chr11:114798923-114806745	OtA5088-OtA5092	OtA5093-OtA5097
Gpr143	chrX:152781920-152808646	OtA5088-OtA5092	OtA5093-OtA5097
Gpr149	chr3:62529962-62605140	OtA5088-OtA5092	OtA5093-OtA5097
Gpr15	chr16:58717434-58718724	OtA5088-OtA5092	OtA5093-OtA5097
Gpr150	chr13:76054850-76056996	OtA5088-OtA5092	OtA5093-OtA5097
Gpr151	chr18:42578019-42579652	OtA5088-OtA5092	OtA5093-OtA5097
Gpr152	chr19:4139798-4145740	OtA5088-OtA5092	OtA5093-OtA5097
Gpr158	chr2:21367566-21830542	OtA5088-OtA5092	OtA5093-OtA5097
Gpr162	chr6:124858444-124863917	OtA5088-OtA5092	OtA5093-OtA5097
Gpr165	chrX:96713467-96719388	OtA5088-OtA5092	OtA5093-OtA5097
Gpr17	chr18:31931506-31958619	OtA5088-OtA5092	OtA5093-OtA5097
Gpr171	chr3:59006977-59344256	OtA5088-OtA5092	OtA5093-OtA5097
Gpr173	chrX:152344645-152368111	OtA5088-OtA5092	OtA5093-OtA5097
Gpr174	chrX:107255877-107296769	OtA5088-OtA5092	OtA5093-OtA5097
Gpr176	chr2:118277097-118373419	OtA5088-OtA5092	OtA5093-OtA5097
Gpr179	chr11:97332108-97352073	OtA5088-OtA5092	OtA5093-OtA5097
Gpr18	chr14:121878605-122021035	OtA5088-OtA5092	OtA5093-OtA5097
Gpr183	chr14:121878605-122021035	OtA5088-OtA5092	OtA5093-OtA5097
Gpr20	chr15:73694606-73707505	OtA5088-OtA5092	OtA5093-OtA5097
Gpr21	chr2:37443284-37566437	OtA5088-OtA5092	OtA5093-OtA5097
Gpr22	chr12:31654868-31950535	OtA5088-OtA5092	OtA5093-OtA5097
Gpr25	chr1:136258913-136260873	OtA5088-OtA5092	OtA5093-OtA5097
Gpr26	chr7:131966459-131985633	OtA5088-OtA5092	OtA5093-OtA5097
Gpr3	chr4:133209339-133212536	OtA5088-OtA5092	OtA5093-OtA5097
Gpr30	chr5:139359738-139460534	OtA5088-OtA5092	OtA5093-OtA5097
Gpr31c	chr17:13051320-13052280	OtA5088-OtA5092	OtA5093-OtA5097
Gpr33	chr12:52023003-52028063	OtA5088-OtA5092	OtA5093-OtA5097
Gpr34	chrX:13517081-13846541	OtA5088-OtA5092	OtA5093-OtA5097
Gpr37	chr6:25668522-25689980	OtA5088-OtA5092	OtA5093-OtA5097
Gpr37l1	chr1:135160249-135167681	OtA5088-OtA5092	OtA5093-OtA5097
Gpr45	chr1:42952871-43035449	OtA5088-OtA5092	OtA5093-OtA5097
Gpr55	chr1:85939536-85961055	OtA5088-OtA5092	OtA5093-OtA5097
Gpr6	chr10:41070492-41071584	OtA5088-OtA5092	OtA5093-OtA5097
Gpr61	chr3:108148320-108154882	OtA5088-OtA5092	OtA5093-OtA5097
Gpr62	chr9:106453837-106465940	OtA5088-OtA5092	OtA5093-OtA5097
Gpr63	chr4:24973418-25009233	OtA5088-OtA5092	OtA5093-OtA5097
Gpr64	chrX:160390689-160498070	OtA5088-OtA5092	OtA5093-OtA5097
Gpr65	chr12:98268656-98276632	OtA5088-OtA5092	OtA5093-OtA5097

Gpr68	chr12:100876681-100908198	OtA5088-OtA5092	OtA5093-OtA5097
Gpr75	chr11:30885357-30893725	OtA5088-OtA5092	OtA5093-OtA5097
Gpr81	chr5:123876735-123880020	OtA5088-OtA5092	OtA5093-OtA5097
Gpr82	chrX:13517081-13846541	OtA5088-OtA5092	OtA5093-OtA5097
Gpr83	chr9:14860253-14869499	OtA5088-OtA5092	OtA5093-OtA5097
Gpr84	chr15:103308234-103310438	OtA5088-OtA5092	OtA5093-OtA5097
Gpr85	chr6:13835073-13839848	OtA5088-OtA5092	OtA5093-OtA5097
Gpr87	chr3:59006977-59344256	OtA5088-OtA5092	OtA5093-OtA5097
Gpr88	chr3:116249653-116253484	OtA5088-OtA5092	OtA5093-OtA5097
Gpr98	chr13:81095067-81633144	OtA5088-OtA5092	OtA5093-OtA5097
Gprasp2	chrX:135839033-135844730	OtA5088-OtA5092	OtA5093-OtA5097
Gprc5d	chr6:135105990-135118283	OtA5088-OtA5092	OtA5093-OtA5097
Gprc6a	chr10:51614822-51631458	OtA5088-OtA5092	OtA5093-OtA5097
Gprin1	chr13:54736672-54749669	OtA5088-OtA5092	OtA5093-OtA5097
Gprin2	chr14:34194440-34201633	OtA5088-OtA5092	OtA5093-OtA5097
Gpt	chr15:76696763-76699675	OtA5088-OtA5092	OtA5093-OtA5097
Gpx2	chr12:76792334-76795554	OtA5088-OtA5092	OtA5093-OtA5097
Gpx2-ps1	chr7:100227606-100278872	OtA5088-OtA5092	OtA5093-OtA5097
Gpx5	chr13:21286428-21292686	OtA5088-OtA5092	OtA5093-OtA5097
Gpx6	chr13:21312202-21319624	OtA5088-OtA5092	OtA5093-OtA5097
Grap2	chr15:80623482-80650298	OtA5088-OtA5092	OtA5093-OtA5097
Grb14	chr2:64912481-65022766	OtA5088-OtA5092	OtA5093-OtA5097
Greb1l	chr18:10325178-10562941	OtA5088-OtA5092	OtA5093-OtA5097
Grem1	chr2:113748674-113758648	OtA5088-OtA5092	OtA5093-OtA5097
Grhl3	chr4:135541887-135573620	OtA5088-OtA5092	OtA5093-OtA5097
Gria1	chr11:57011617-57330244	OtA5088-OtA5092	OtA5093-OtA5097
Gria2	chr3:80684935-80802791	OtA5088-OtA5092	OtA5093-OtA5097
Gria3	chrX:41401303-41678601	OtA5088-OtA5092	OtA5093-OtA5097
Gria4	chr9:4417892-4796234	OtA5088-OtA5092	OtA5093-OtA5097
Grid1	chr14:34820135-35581115	OtA5088-OtA5092	OtA5093-OtA5097
Grid2	chr6:63256856-64666279	OtA5088-OtA5092	OtA5093-OtA5097
Grid2ip	chr5:143357337-143391798	OtA5088-OtA5092	OtA5093-OtA5097
Grifin	chr5:140563188-140565067	OtA5088-OtA5092	OtA5093-OtA5097
Grik1	chr16:87895896-88290258	OtA5088-OtA5092	OtA5093-OtA5097
Grik2	chr10:49099462-49788754	OtA5088-OtA5092	OtA5093-OtA5097
Grik3	chr4:125490830-125714173	OtA5088-OtA5092	OtA5093-OtA5097
Grik4	chr9:42520411-42944371	OtA5088-OtA5092	OtA5093-OtA5097
Grin1	chr2:25289910-25319163	OtA5088-OtA5092	OtA5093-OtA5097
Grin2a	chr16:9577709-9992533	OtA5088-OtA5092	OtA5093-OtA5097
Grin2b	chr6:135729804-136173511	OtA5088-OtA5092	OtA5093-OtA5097
Grin2c	chr11:115249168-115267243	OtA5088-OtA5092	OtA5093-OtA5097
Grin2d	chr7:45832482-45866681	OtA5088-OtA5092	OtA5093-OtA5097
Grin3a	chr4:49665225-49845081	OtA5088-OtA5092	OtA5093-OtA5097
Grin3b	chr10:79697304-80369637	OtA5088-OtA5092	OtA5093-OtA5097
Grip1	chr10:119454313-120075593	OtA5088-OtA5092	OtA5093-OtA5097
Grip2	chr6:91761509-91807393	OtA5088-OtA5092	OtA5093-OtA5097
Grk1	chr8:13405080-13421949	OtA5088-OtA5092	OtA5093-OtA5097

Grk4	chr5:34660378-34755303	OtA5088-OtA5092	OtA5093-OtA5097
Grm1	chr10:10686058-11082331	OtA5088-OtA5092	OtA5093-OtA5097
Grm2	chr9:106644533-106656109	OtA5088-OtA5092	OtA5093-OtA5097
Grm3	chr5:9485235-9725352	OtA5088-OtA5092	OtA5093-OtA5097
Grm4	chr17:27422387-27503304	OtA5088-OtA5092	OtA5093-OtA5097
Grm5	chr7:87584167-88135063	OtA5088-OtA5092	OtA5093-OtA5097
Grm6	chr11:50850684-50866208	OtA5088-OtA5092	OtA5093-OtA5097
Grm7	chr6:110645597-111567230	OtA5088-OtA5092	OtA5093-OtA5097
Grm8	chr6:27275120-28134369	OtA5088-OtA5092	OtA5093-OtA5097
Grp	chr18:65873493-65886579	OtA5088-OtA5092	OtA5093-OtA5097
Grpr	chrX:163513903-163549647	OtA5088-OtA5092	OtA5093-OtA5097
Grxcr1	chr5:68031834-68166398	OtA5088-OtA5092	OtA5093-OtA5097
Grxcr2	chr18:41986200-41999049	OtA5088-OtA5092	OtA5093-OtA5097
Gsc2	chr16:17913553-17915059	OtA5088-OtA5092	OtA5093-OtA5097
Gsdma	chr11:98664350-98677708	OtA5088-OtA5092	OtA5093-OtA5097
Gsdma2	chr11:98646758-98657957	OtA5088-OtA5092	OtA5093-OtA5097
Gsdma3	chr11:98626359-98638089	OtA5088-OtA5092	OtA5093-OtA5097
Gsdmc	chr15:63775970-63808739	OtA5088-OtA5092	OtA5093-OtA5097
Gsdmc2	chr15:63824345-63845176	OtA5088-OtA5092	OtA5093-OtA5097
Gsdmc3	chr15:63857724-63878558	OtA5088-OtA5092	OtA5093-OtA5097
Gsdmc4	chr15:63891264-63912297	OtA5088-OtA5092	OtA5093-OtA5097
Gsdmcl-ps	chr15:63914460-63925453	OtA5088-OtA5092	OtA5093-OtA5097
Gsdmcl1	chr15:63847308-63850942	OtA5088-OtA5092	OtA5093-OtA5097
Gsg1	chr6:135237329-135254336	OtA5088-OtA5092	OtA5093-OtA5097
Gsg2	chr11:73090594-73147449	OtA5088-OtA5092	OtA5093-OtA5097
Gspt2	chrX:94636068-94638561	OtA5088-OtA5092	OtA5093-OtA5097
Gsta1	chr9:78230668-78242683	OtA5088-OtA5092	OtA5093-OtA5097
Gsta2	chr9:78331019-78347145	OtA5088-OtA5092	OtA5093-OtA5097
Gsta3	chr1:21240588-21265559	OtA5088-OtA5092	OtA5093-OtA5097
Gsta4	chr9:78191965-78209349	OtA5088-OtA5092	OtA5093-OtA5097
Gstm3	chr3:107963695-107969174	OtA5088-OtA5092	OtA5093-OtA5097
Gstm6	chr3:107938847-107943749	OtA5088-OtA5092	OtA5093-OtA5097
Gstm7	chr3:107926333-107931745	OtA5088-OtA5092	OtA5093-OtA5097
Gstt4	chr10:75814943-75822543	OtA5088-OtA5092	OtA5093-OtA5097
Gsx1	chr5:147188695-147190946	OtA5088-OtA5092	OtA5093-OtA5097
Gsx2	chr5:75075600-75077893	OtA5088-OtA5092	OtA5093-OtA5097
Gtf2a1l	chr17:88668659-88715150	OtA5088-OtA5092	OtA5093-OtA5097
Gtpbp10	chr5:5537456-5559501	OtA5088-OtA5092	OtA5093-OtA5097
Gtse1	chr15:85859706-85876573	OtA5088-OtA5092	OtA5093-OtA5097
Gtsf1	chr15:103402458-103430427	OtA5088-OtA5092	OtA5093-OtA5097
Gtsf1l	chr2:163087030-163089601	OtA5088-OtA5092	OtA5093-OtA5097
Guca1a	chr17:47394557-47400584	OtA5088-OtA5092	OtA5093-OtA5097
Guca1b	chr17:47385392-47392967	OtA5088-OtA5092	OtA5093-OtA5097
Guca2a	chr4:119637731-119639465	OtA5088-OtA5092	OtA5093-OtA5097
Gucy1a2	chr9:3532348-3905787	OtA5088-OtA5092	OtA5093-OtA5097
Gucy2c	chr6:136697283-136781742	OtA5088-OtA5092	OtA5093-OtA5097
Gucy2d	chr7:98443421-98477478	OtA5088-OtA5092	OtA5093-OtA5097

Gucy2e	chr11:69218116-69237022	OtA5088-OtA5092	OtA5093-OtA5097
Gucy2f	chrX:142079288-142196936	OtA5088-OtA5092	OtA5093-OtA5097
Gucy2g	chr19:55198097-55241236	OtA5088-OtA5092	OtA5093-OtA5097
Guf1	chr5:69556941-69573630	OtA5088-OtA5092	OtA5093-OtA5097
Gulp1	chr1:44551670-44796836	OtA5088-OtA5092	OtA5093-OtA5097
Gvin1	chr7:106156535-106215340	OtA5088-OtA5092	OtA5093-OtA5097
Gxylt2	chr6:100704733-100805081	OtA5088-OtA5092	OtA5093-OtA5097
Gyk	chrX:85701936-85776819	OtA5088-OtA5092	OtA5093-OtA5097
Gykl1	chr18:52693678-52695593	OtA5088-OtA5092	OtA5093-OtA5097
Gys2	chr6:142422612-142473109	OtA5088-OtA5092	OtA5093-OtA5097
Gzma	chr13:113093826-113100981	OtA5088-OtA5092	OtA5093-OtA5097
Gzmk	chr13:113171873-113180897	OtA5088-OtA5092	OtA5093-OtA5097
Gzmn	chr14:56165795-56174596	OtA5088-OtA5092	OtA5093-OtA5097
H1fnt	chr15:98255981-98257307	OtA5088-OtA5092	OtA5093-OtA5097
H1foo	chr6:115944938-115950233	OtA5088-OtA5092	OtA5093-OtA5097
H1fx	chr6:87980420-87981482	OtA5088-OtA5092	OtA5093-OtA5097
H2-Bl	chr17:36080188-36084249	OtA5088-OtA5092	OtA5093-OtA5097
H2-DMb1	chr17:34153190-34160229	OtA5088-OtA5092	OtA5093-OtA5097
H2-DMb2	chr17:34145415-34151095	OtA5088-OtA5092	OtA5093-OtA5097
H2-Ea-ps	chr17:34342211-34344645	OtA5088-OtA5092	OtA5093-OtA5097
H2-Eb2	chr17:34325674-34341410	OtA5088-OtA5092	OtA5093-OtA5097
H2-M1	chr17:36670007-36672197	OtA5088-OtA5092	OtA5093-OtA5097
H2-M10.1	chr17:36322859-36326150	OtA5088-OtA5092	OtA5093-OtA5097
H2-M10.2	chr17:36284280-36286421	OtA5088-OtA5092	OtA5093-OtA5097
H2-M10.3	chr17:36365003-36368417	OtA5088-OtA5092	OtA5093-OtA5097
H2-M10.4	chr17:36460163-36462329	OtA5088-OtA5092	OtA5093-OtA5097
H2-M10.5	chr17:36772909-36776235	OtA5088-OtA5092	OtA5093-OtA5097
H2-M10.6	chr17:36812174-36815566	OtA5088-OtA5092	OtA5093-OtA5097
H2-M11	chr17:36547074-36549252	OtA5088-OtA5092	OtA5093-OtA5097
H2-M2	chr17:37480850-37483529	OtA5088-OtA5092	OtA5093-OtA5097
H2-M5	chr17:36986854-36989504	OtA5088-OtA5092	OtA5093-OtA5097
H2-M9	chr17:36640424-36642644	OtA5088-OtA5092	OtA5093-OtA5097
H2-Oa	chr17:34083842-34095309	OtA5088-OtA5092	OtA5093-OtA5097
H2-Ob	chr17:34238904-34245908	OtA5088-OtA5092	OtA5093-OtA5097
H2-Q1	chr17:35320557-35325099	OtA5088-OtA5092	OtA5093-OtA5097
H2-Q10	chr17:35470088-35474563	OtA5088-OtA5092	OtA5093-OtA5097
H2-T10	chr17:36115870-36121444	OtA5088-OtA5092	OtA5093-OtA5097
H2-T3	chr17:36185570-36190151	OtA5088-OtA5092	OtA5093-OtA5097
H28	chr3:151758736-151762891	OtA5088-OtA5092	OtA5093-OtA5097
H2afb1	chr2:17996421-17996946	OtA5088-OtA5092	OtA5093-OtA5097
H2afy2	chr10:61715262-61783864	OtA5088-OtA5092	OtA5093-OtA5097
H2afy3	chr15:62037986-62250975	OtA5088-OtA5092	OtA5093-OtA5097
H60b	chr10:22158608-22374139	OtA5088-OtA5092	OtA5093-OtA5097
H60c	chr10:3256207-3267771	OtA5088-OtA5092	OtA5093-OtA5097
Haao	chr17:83831353-83846790	OtA5088-OtA5092	OtA5093-OtA5097
Hamp	chr7:30942368-30944017	OtA5088-OtA5092	OtA5093-OtA5097
Hamp2	chr7:30922371-30924181	OtA5088-OtA5092	OtA5093-OtA5097

Hao1	chr2:134497360-134554352	OtA5088-OtA5092	OtA5093-OtA5097
Hao2	chr3:98874518-98893230	OtA5088-OtA5092	OtA5093-OtA5097
Hapln1	chr13:89540635-89611832	OtA5088-OtA5092	OtA5093-OtA5097
Hapln2	chr3:88021749-88027511	OtA5088-OtA5092	OtA5093-OtA5097
Has1	chr17:17843325-17855188	OtA5088-OtA5092	OtA5093-OtA5097
Has2	chr15:56665626-56694546	OtA5088-OtA5092	OtA5093-OtA5097
Has2as	chr15:56665626-56694546	OtA5088-OtA5092	OtA5093-OtA5097
Has3	chr8:106870241-106882902	OtA5088-OtA5092	OtA5093-OtA5097
Haus3	chr5:34007199-34169448	OtA5088-OtA5092	OtA5093-OtA5097
Hba-a2	chr11:32296488-32297310	OtA5088-OtA5092	OtA5093-OtA5097
Hbb-bh1	chr7:103841637-103843162	OtA5088-OtA5092	OtA5093-OtA5097
Hbb-bh2	chr7:103839123-103840521	OtA5088-OtA5092	OtA5093-OtA5097
Hbq1a	chr11:32300068-32300873	OtA5088-OtA5092	OtA5093-OtA5097
Hbq1b	chr11:32286963-32287912	OtA5088-OtA5092	OtA5093-OtA5097
Hc	chr2:34983330-35061441	OtA5088-OtA5092	OtA5093-OtA5097
Hcn1	chr13:117602319-117981028	OtA5088-OtA5092	OtA5093-OtA5097
Hcn3	chr3:89146774-89160157	OtA5088-OtA5092	OtA5093-OtA5097
Hcn4	chr9:58823511-58860955	OtA5088-OtA5092	OtA5093-OtA5097
Hcrt	chr11:100761692-100762931	OtA5088-OtA5092	OtA5093-OtA5097
Hcrtr1	chr4:130130216-130139162	OtA5088-OtA5092	OtA5093-OtA5097
Hcrtr2	chr9:76225879-76323578	OtA5088-OtA5092	OtA5093-OtA5097
Hcst	chr7:30417711-30419854	OtA5088-OtA5092	OtA5093-OtA5097
Hdac11	chr6:91156814-91174683	OtA5088-OtA5092	OtA5093-OtA5097
Hdac9	chr12:34371496-34528889	OtA5088-OtA5092	OtA5093-OtA5097
Hdc	chr2:126593667-126618665	OtA5088-OtA5092	OtA5093-OtA5097
Hdgfl1	chr13:26768171-26770165	OtA5088-OtA5092	OtA5093-OtA5097
Hdgfrp3	chr7:81859021-81934459	OtA5088-OtA5092	OtA5093-OtA5097
Hdhd1a	chr18:50567655-50568699	OtA5088-OtA5092	OtA5093-OtA5097
Hdx	chrX:111575203-111697079	OtA5088-OtA5092	OtA5093-OtA5097
Heatr7b1	chr1:88227019-88277579	OtA5088-OtA5092	OtA5093-OtA5097
Heatr7b2	chr15:4898736-4962201	OtA5088-OtA5092	OtA5093-OtA5097
Heatr8	chr4:106680416-106727930	OtA5088-OtA5092	OtA5093-OtA5097
Hectd2	chr19:36554638-36689479	OtA5088-OtA5092	OtA5093-OtA5097
Hecw1	chr13:14226437-14523226	OtA5088-OtA5092	OtA5093-OtA5097
Helb	chr10:120083607-120112965	OtA5088-OtA5092	OtA5093-OtA5097
Hells	chr19:38930989-38968277	OtA5088-OtA5092	OtA5093-OtA5097
Helt	chr8:46292047-46294671	OtA5088-OtA5092	OtA5093-OtA5097
Hemgn	chr4:46393988-46404183	OtA5088-OtA5092	OtA5093-OtA5097
Hemt1	chr15:74819075-74824436	OtA5088-OtA5092	OtA5093-OtA5097
Hepacam	chr9:37367605-37386571	OtA5088-OtA5092	OtA5093-OtA5097
Hepacam2	chr6:3457088-3494498	OtA5088-OtA5092	OtA5093-OtA5097
Hephl1	chr9:15051840-15112108	OtA5088-OtA5092	OtA5093-OtA5097
Herc6	chr6:57580991-57665136	OtA5088-OtA5092	OtA5093-OtA5097
Hes2	chr4:152158866-152162469	OtA5088-OtA5092	OtA5093-OtA5097
Hes3	chr4:152285971-152291662	OtA5088-OtA5092	OtA5093-OtA5097
Hes5	chr4:154960922-154962371	OtA5088-OtA5092	OtA5093-OtA5097
Hes7	chr11:69120452-69123259	OtA5088-OtA5092	OtA5093-OtA5097

Hesx1	chr14:27000361-27002325	OtA5088-OtA5092	OtA5093-OtA5097
Hey2	chr10:30832358-30842783	OtA5088-OtA5092	OtA5093-OtA5097
Heyl	chr4:123233555-123249870	OtA5088-OtA5092	OtA5093-OtA5097
Hfe2	chr3:96525184-96529216	OtA5088-OtA5092	OtA5093-OtA5097
Hfm1	chr5:106840196-106925890	OtA5088-OtA5092	OtA5093-OtA5097
Hgf	chr5:16553549-16619439	OtA5088-OtA5092	OtA5093-OtA5097
Hhat	chr1:192512827-192771219	OtA5088-OtA5092	OtA5093-OtA5097
Hhatl	chr9:121784015-121792507	OtA5088-OtA5092	OtA5093-OtA5097
Hhex	chr19:37434840-37440731	OtA5088-OtA5092	OtA5093-OtA5097
Hhip	chr8:79965850-80058008	OtA5088-OtA5092	OtA5093-OtA5097
Hhipl2	chr1:183418502-183437053	OtA5088-OtA5092	OtA5093-OtA5097
Hhla1	chr15:65922442-65976804	OtA5088-OtA5092	OtA5093-OtA5097
Higd1c	chr15:100353199-100384435	OtA5088-OtA5092	OtA5093-OtA5097
Hils1	chr11:94962821-94976327	OtA5088-OtA5092	OtA5093-OtA5097
Hipk4	chr7:27523260-27531174	OtA5088-OtA5092	OtA5093-OtA5097
Hirip3	chr7:126861971-126865122	OtA5088-OtA5092	OtA5093-OtA5097
Hist1h1t	chr13:23695810-23696722	OtA5088-OtA5092	OtA5093-OtA5097
Hist1h2aa	chr13:23934461-23934851	OtA5088-OtA5092	OtA5093-OtA5097
Hist1h2ad	chr13:23574470-23574863	OtA5088-OtA5092	OtA5093-OtA5097
Hist1h2ah	chr13:22035165-22035552	OtA5088-OtA5092	OtA5093-OtA5097
Hist1h2ao,Hist1h2ap	chr13:21833061-21833575	OtA5088-OtA5092	OtA5093-OtA5097
Hist1h2ap	chr13:21833061-21833575	OtA5088-OtA5092	OtA5093-OtA5097
Hist1h2ba	chr13:23933772-23934156	OtA5088-OtA5092	OtA5093-OtA5097
Hist1h2br	chr13:21833742-21837530	OtA5088-OtA5092	OtA5093-OtA5097
Hist2h2aa1,Hist2h2aa2	chr3:96245537-96246115	OtA5088-OtA5092	OtA5093-OtA5097
Hist2h2aa2	chr3:96245537-96246115	OtA5088-OtA5092	OtA5093-OtA5097
Hist2h2be	chr3:96221120-96223738	OtA5088-OtA5092	OtA5093-OtA5097
Hist3h2ba	chr11:58948910-58949530	OtA5088-OtA5092	OtA5093-OtA5097
Hist3h2bb-ps	chr11:58954047-58954512	OtA5088-OtA5092	OtA5093-OtA5097
Hivep3	chr4:119814677-120135411	OtA5088-OtA5092	OtA5093-OtA5097
Hk3	chr13:54949431-55021385	OtA5088-OtA5092	OtA5093-OtA5097
Hlf	chr11:90336534-90390917	OtA5088-OtA5092	OtA5093-OtA5097
Hmcn1	chr1:150562501-150993435	OtA5088-OtA5092	OtA5093-OtA5097
Hmga1-rs1	chr17:27556573-27563672	OtA5088-OtA5092	OtA5093-OtA5097
Hmga2	chr10:120361274-120476935	OtA5088-OtA5092	OtA5093-OtA5097
Hmga2-ps1	chr1:176814811-177020432	OtA5088-OtA5092	OtA5093-OtA5097
Hmgb1-rs17	chr8:33484310-33485757	OtA5088-OtA5092	OtA5093-OtA5097
Hmgb4	chr4:128260211-128260895	OtA5088-OtA5092	OtA5093-OtA5097
Hmgcll1	chr9:76014976-76136350	OtA5088-OtA5092	OtA5093-OtA5097
Hmgn3	chr9:83109941-83146607	OtA5088-OtA5092	OtA5093-OtA5097
Hmgn5	chrX:109004536-109013380	OtA5088-OtA5092	OtA5093-OtA5097
Hmmr	chr11:40701387-40733437	OtA5088-OtA5092	OtA5093-OtA5097
Hmx1	chr5:35389116-35392872	OtA5088-OtA5092	OtA5093-OtA5097
Hmx2	chr7:131554061-131556582	OtA5088-OtA5092	OtA5093-OtA5097
Hmx3	chr7:131542962-131544931	OtA5088-OtA5092	OtA5093-OtA5097
Hnf4g	chr3:3508029-3659800	OtA5088-OtA5092	OtA5093-OtA5097
Hnmt	chr2:24002912-24049379	OtA5088-OtA5092	OtA5093-OtA5097

Hormad1	chr3:95559676-95587671	OtA5088-OtA5092	OtA5093-OtA5097
Hormad2	chr11:4346382-4441082	OtA5088-OtA5092	OtA5093-OtA5097
Hotair	chr15:102944061-102947946	OtA5088-OtA5092	OtA5093-OtA5097
Hoxa1	chr6:52155366-52158317	OtA5088-OtA5092	OtA5093-OtA5097
Hoxa2	chr6:52162510-52164831	OtA5088-OtA5092	OtA5093-OtA5097
Hoxa3	chr6:52169061-52213597	OtA5088-OtA5092	OtA5093-OtA5097
Hoxa4	chr6:52169061-52213597	OtA5088-OtA5092	OtA5093-OtA5097
Hoxa5	chr6:52169061-52213597	OtA5088-OtA5092	OtA5093-OtA5097
Hoxa6	chr6:52169061-52213597	OtA5088-OtA5092	OtA5093-OtA5097
Hoxa7	chr6:52215623-52218573	OtA5088-OtA5092	OtA5093-OtA5097
Hoxa9	chr6:52224053-52227370	OtA5088-OtA5092	OtA5093-OtA5097
Hoxb1	chr11:96365757-96368253	OtA5088-OtA5092	OtA5093-OtA5097
Hoxb13	chr11:96194360-96196599	OtA5088-OtA5092	OtA5093-OtA5097
Hoxb2	chr11:96351631-96354014	OtA5088-OtA5092	OtA5093-OtA5097
Hoxb3	chr11:96323125-96347930	OtA5088-OtA5092	OtA5093-OtA5097
Hoxb4	chr11:96318266-96321638	OtA5088-OtA5092	OtA5093-OtA5097
Hoxb5	chr11:96303511-96306121	OtA5088-OtA5092	OtA5093-OtA5097
Hoxb7	chr11:96286645-96290163	OtA5088-OtA5092	OtA5093-OtA5097
Hoxb8	chr11:96281904-96285325	OtA5088-OtA5092	OtA5093-OtA5097
Hoxb9	chr11:96271329-96276593	OtA5088-OtA5092	OtA5093-OtA5097
Hoxc10	chr15:102966795-102971897	OtA5088-OtA5092	OtA5093-OtA5097
Hoxc11	chr15:102954525-102956701	OtA5088-OtA5092	OtA5093-OtA5097
Hoxc12	chr15:102936852-102938517	OtA5088-OtA5092	OtA5093-OtA5097
Hoxc13	chr15:102921130-102928814	OtA5088-OtA5092	OtA5093-OtA5097
Hoxc4	chr15:103034394-103036852	OtA5088-OtA5092	OtA5093-OtA5097
Hoxc5	chr15:103014007-103017429	OtA5088-OtA5092	OtA5093-OtA5097
Hoxc6	chr15:103009561-103011881	OtA5088-OtA5092	OtA5093-OtA5097
Hoxc8	chr15:102990538-102994254	OtA5088-OtA5092	OtA5093-OtA5097
Hoxc9	chr15:102977031-102984444	OtA5088-OtA5092	OtA5093-OtA5097
Hoxd1	chr2:74762979-74765142	OtA5088-OtA5092	OtA5093-OtA5097
Hoxd11	chr2:74682392-74684138	OtA5088-OtA5092	OtA5093-OtA5097
Hoxd12	chr2:74675029-74677705	OtA5088-OtA5092	OtA5093-OtA5097
Hoxd13	chr2:74668309-74671598	OtA5088-OtA5092	OtA5093-OtA5097
Hoxd3	chr2:74710043-74748271	OtA5088-OtA5092	OtA5093-OtA5097
Hpcal4	chr4:123183503-123194699	OtA5088-OtA5092	OtA5093-OtA5097
Hpd	chr5:123171806-123182686	OtA5088-OtA5092	OtA5093-OtA5097
Hpdl	chr4:116819906-116821508	OtA5088-OtA5092	OtA5093-OtA5097
Hpgds	chr6:65117292-65144730	OtA5088-OtA5092	OtA5093-OtA5097
Hpse2	chr19:42788594-43388355	OtA5088-OtA5092	OtA5093-OtA5097
Hrasls	chr16:29209694-29230530	OtA5088-OtA5092	OtA5093-OtA5097
Hrasls5	chr19:7612568-7639238	OtA5088-OtA5092	OtA5093-OtA5097
Hrc	chr7:45335268-45338972	OtA5088-OtA5092	OtA5093-OtA5097
Hrg	chr16:22951071-22961659	OtA5088-OtA5092	OtA5093-OtA5097
Hrh1	chr6:114397935-114483296	OtA5088-OtA5092	OtA5093-OtA5097
Hrh2	chr13:54192128-54222432	OtA5088-OtA5092	OtA5093-OtA5097
Hrh3	chr2:180099472-180104222	OtA5088-OtA5092	OtA5093-OtA5097
Hrh4	chr18:13006989-13022882	OtA5088-OtA5092	OtA5093-OtA5097

Hrnr	chr3:93319748-93333572	OtA5088-OtA5092	OtA5093-OtA5097
Hs3st2	chr7:121392295-121501768	OtA5088-OtA5092	OtA5093-OtA5097
Hs3st3a1	chr11:64433134-64522835	OtA5088-OtA5092	OtA5093-OtA5097
Hs3st3b1	chr11:63884692-63922284	OtA5088-OtA5092	OtA5093-OtA5097
Hs3st4	chr7:123983180-124398989	OtA5088-OtA5092	OtA5093-OtA5097
Hs3st5	chr10:36506806-36834394	OtA5088-OtA5092	OtA5093-OtA5097
Hs3st6	chr17:24753002-24758684	OtA5088-OtA5092	OtA5093-OtA5097
Hs6st2	chrX:51387211-51681602	OtA5088-OtA5092	OtA5093-OtA5097
Hs6st3	chr14:119138264-119869815	OtA5088-OtA5092	OtA5093-OtA5097
Hsbp1l1	chr18:80229755-80247102	OtA5088-OtA5092	OtA5093-OtA5097
Hsd17b1	chr11:101078410-101080505	OtA5088-OtA5092	OtA5093-OtA5097
Hsd17b13	chr5:103955441-103977388	OtA5088-OtA5092	OtA5093-OtA5097
Hsd17b14	chr7:45554927-45567321	OtA5088-OtA5092	OtA5093-OtA5097
Hsd17b3	chr13:64058273-64089201	OtA5088-OtA5092	OtA5093-OtA5097
Hsd17b6	chr10:127990935-128007508	OtA5088-OtA5092	OtA5093-OtA5097
Hsd3b2	chr3:98711106-98724543	OtA5088-OtA5092	OtA5093-OtA5097
Hsd3b3	chr3:98741475-98763128	OtA5088-OtA5092	OtA5093-OtA5097
Hsd3b4	chr3:98552550-98564038	OtA5088-OtA5092	OtA5093-OtA5097
Hsd3b5	chr3:98618635-98645534	OtA5088-OtA5092	OtA5093-OtA5097
Hsd3b6	chr3:98805503-98814443	OtA5088-OtA5092	OtA5093-OtA5097
Hsf2bp	chr17:31944768-32034508	OtA5088-OtA5092	OtA5093-OtA5097
Hsf3	chrX:96306866-96357240	OtA5088-OtA5092	OtA5093-OtA5097
Hsf4	chr8:105269800-105275845	OtA5088-OtA5092	OtA5093-OtA5097
Hsf5	chr11:87617163-87659542	OtA5088-OtA5092	OtA5093-OtA5097
Hsfy2	chr1:56636050-56637451	OtA5088-OtA5092	OtA5093-OtA5097
Hsh2d	chr8:72189667-72200958	OtA5088-OtA5092	OtA5093-OtA5097
Hspa1l	chr17:34972702-34979228	OtA5088-OtA5092	OtA5093-OtA5097
Hspb3	chr13:113662895-113663676	OtA5088-OtA5092	OtA5093-OtA5097
Hspb9	chr11:100713849-100714575	OtA5088-OtA5092	OtA5093-OtA5097
Htr1a	chr13:105443692-105448133	OtA5088-OtA5092	OtA5093-OtA5097
Htr1b	chr9:81631391-81645156	OtA5088-OtA5092	OtA5093-OtA5097
Htr1f	chr16:64924728-65105784	OtA5088-OtA5092	OtA5093-OtA5097
Htr2a	chr14:74640839-74706859	OtA5088-OtA5092	OtA5093-OtA5097
Htr2c	chrX:146962512-147197277	OtA5088-OtA5092	OtA5093-OtA5097
Htr3a	chr9:48899212-48911099	OtA5088-OtA5092	OtA5093-OtA5097
Htr3b	chr9:48935007-48964990	OtA5088-OtA5092	OtA5093-OtA5097
Htr4	chr18:62324203-62467802	OtA5088-OtA5092	OtA5093-OtA5097
Htr5a	chr5:27841946-27855086	OtA5088-OtA5092	OtA5093-OtA5097
Htr5b	chr1:121509772-121528465	OtA5088-OtA5092	OtA5093-OtA5097
Htr6	chr4:139061408-139074789	OtA5088-OtA5092	OtA5093-OtA5097
Htr7	chr19:35958728-36057360	OtA5088-OtA5092	OtA5093-OtA5097
Hus1b	chr13:30813917-30974047	OtA5088-OtA5092	OtA5093-OtA5097
Hyal3	chr9:107580169-107587359	OtA5088-OtA5092	OtA5093-OtA5097
Hyal4	chr6:24748366-24766519	OtA5088-OtA5092	OtA5093-OtA5097
Hyal5	chr6:24857996-24891958	OtA5088-OtA5092	OtA5093-OtA5097
Hyal6	chr6:24733244-24745452	OtA5088-OtA5092	OtA5093-OtA5097
Hydin	chr8:110266976-110610253	OtA5088-OtA5092	OtA5093-OtA5097

Hyls1	chr9:35559465-35570069	OtA5088-OtA5092	OtA5093-OtA5097
I730028E13Rik	chr9:61138514-61145259	OtA5088-OtA5092	OtA5093-OtA5097
I730030J21Rik	chr15:100730504-100732737	OtA5088-OtA5092	OtA5093-OtA5097
I830012O16Rik	chr19:34607956-34613401	OtA5088-OtA5092	OtA5093-OtA5097
I830077J02Rik	chr3:105925890-105932664	OtA5088-OtA5092	OtA5093-OtA5097
Iapp	chr6:142234228-142322950	OtA5088-OtA5092	OtA5093-OtA5097
Ibsp	chr5:104299286-104311472	OtA5088-OtA5092	OtA5093-OtA5097
Ica1l	chr1:59989067-60043087	OtA5088-OtA5092	OtA5093-OtA5097
Icam5	chr9:21032037-21039036	OtA5088-OtA5092	OtA5093-OtA5097
Icos	chr1:60977913-61000321	OtA5088-OtA5092	OtA5093-OtA5097
Id4	chr13:48261426-48264036	OtA5088-OtA5092	OtA5093-OtA5097
Idi1	chr13:8885605-8892396	OtA5088-OtA5092	OtA5093-OtA5097
Idi2	chr13:8952862-8960935	OtA5088-OtA5092	OtA5093-OtA5097
Ido1	chr8:24584140-24597009	OtA5088-OtA5092	OtA5093-OtA5097
Ido2	chr8:24531891-24576333	OtA5088-OtA5092	OtA5093-OtA5097
Ifi202b	chr1:173962568-173982844	OtA5088-OtA5092	OtA5093-OtA5097
Ifi203	chr1:173920402-173942492	OtA5088-OtA5092	OtA5093-OtA5097
Ifi204	chr1:173747293-173766919	OtA5088-OtA5092	OtA5093-OtA5097
Ifi205	chr1:174011997-174031755	OtA5088-OtA5092	OtA5093-OtA5097
Ifitm5	chr7:140948961-140950239	OtA5088-OtA5092	OtA5093-OtA5097
Ifitm6	chr7:141015811-141016892	OtA5088-OtA5092	OtA5093-OtA5097
Ifitm7	chr16:13982798-13984621	OtA5088-OtA5092	OtA5093-OtA5097
Ifltd1	chr6:145397234-145434925	OtA5088-OtA5092	OtA5093-OtA5097
Ifna1	chr4:88850086-88850656	OtA5088-OtA5092	OtA5093-OtA5097
Ifna11	chr4:88819918-88820565	OtA5088-OtA5092	OtA5093-OtA5097
Ifna12	chr4:88602579-88603376	OtA5088-OtA5092	OtA5093-OtA5097
Ifna13	chr4:88643640-88644459	OtA5088-OtA5092	OtA5093-OtA5097
Ifna14	chr4:88571228-88571798	OtA5088-OtA5092	OtA5093-OtA5097
Ifna2	chr4:88683206-88683779	OtA5088-OtA5092	OtA5093-OtA5097
Ifna4	chr4:88841815-88842459	OtA5088-OtA5092	OtA5093-OtA5097
Ifna5	chr4:88835524-88836094	OtA5088-OtA5092	OtA5093-OtA5097
Ifna6	chr4:88827415-88827985	OtA5088-OtA5092	OtA5093-OtA5097
Ifna7	chr4:88816224-88816800	OtA5088-OtA5092	OtA5093-OtA5097
Ifna9	chr4:88591812-88592385	OtA5088-OtA5092	OtA5093-OtA5097
Ifnab	chr4:88690598-88691268	OtA5088-OtA5092	OtA5093-OtA5097
Ifnb1	chr4:88522024-88522794	OtA5088-OtA5092	OtA5093-OtA5097
Ifne	chr4:88879537-88880201	OtA5088-OtA5092	OtA5093-OtA5097
Ifng	chr10:118441046-118445892	OtA5088-OtA5092	OtA5093-OtA5097
Ifnk	chr4:34949073-35157484	OtA5088-OtA5092	OtA5093-OtA5097
Ifnz	chr4:88782130-88783592	OtA5088-OtA5092	OtA5093-OtA5097
Ift57	chr16:49699293-49765126	OtA5088-OtA5092	OtA5093-OtA5097
Ift80	chr3:68892498-69004570	OtA5088-OtA5092	OtA5093-OtA5097
Ift81	chr5:122550203-122614518	OtA5088-OtA5092	OtA5093-OtA5097
Igbp1b	chr6:138657091-138658444	OtA5088-OtA5092	OtA5093-OtA5097
Igf1	chr10:87859055-87937047	OtA5088-OtA5092	OtA5093-OtA5097
Igfbpl1	chr4:45809506-45826827	OtA5088-OtA5092	OtA5093-OtA5097
Igfl3	chr7:18176493-18181862	OtA5088-OtA5092	OtA5093-OtA5097

Igfn1	chr1:135953577-136006342	OtA5088-OtA5092	OtA5093-OtA5097
Igj	chr5:88519808-88527897	OtA5088-OtA5092	OtA5093-OtA5097
Igll1	chr16:16860670-16863985	OtA5088-OtA5092	OtA5093-OtA5097
Iglon5	chr7:43472906-43490023	OtA5088-OtA5092	OtA5093-OtA5097
Igsf10	chr3:59006977-59344256	OtA5088-OtA5092	OtA5093-OtA5097
Igsf21	chr4:140026851-140246811	OtA5088-OtA5092	OtA5093-OtA5097
Igsf6	chr7:121034444-121076835	OtA5088-OtA5092	OtA5093-OtA5097
Igsf9b	chr9:27299227-27334763	OtA5088-OtA5092	OtA5093-OtA5097
Ikzf1	chr11:11686212-11772926	OtA5088-OtA5092	OtA5093-OtA5097
Ikzf2	chr1:69531209-69685960	OtA5088-OtA5092	OtA5093-OtA5097
Ikzf3	chr11:98464692-98546031	OtA5088-OtA5092	OtA5093-OtA5097
Il10	chr1:131019844-131024970	OtA5088-OtA5092	OtA5093-OtA5097
Il11	chr7:4772377-4778141	OtA5088-OtA5092	OtA5093-OtA5097
Il11ra2	chr4_JH584293_random:7077-22237	OtA5088-OtA5092	OtA5093-OtA5097
Il12a	chr3:68690643-68698547	OtA5088-OtA5092	OtA5093-OtA5097
Il12b	chr11:44400062-44414017	OtA5088-OtA5092	OtA5093-OtA5097
Il12rb1	chr8:70808448-70821423	OtA5088-OtA5092	OtA5093-OtA5097
Il12rb2	chr6:67292017-67376137	OtA5088-OtA5092	OtA5093-OtA5097
Il13	chr11:53631322-53634702	OtA5088-OtA5092	OtA5093-OtA5097
Il13ra2	chrX:147383477-147403832	OtA5088-OtA5092	OtA5093-OtA5097
Il15ra	chr2:11705535-11733962	OtA5088-OtA5092	OtA5093-OtA5097
Il16	chr7:83643062-83735490	OtA5088-OtA5092	OtA5093-OtA5097
Il17a	chr1:20730904-20734496	OtA5088-OtA5092	OtA5093-OtA5097
Il17b	chr18:61687934-61692537	OtA5088-OtA5092	OtA5093-OtA5097
Il17c	chr8:122422084-122423639	OtA5088-OtA5092	OtA5093-OtA5097
Il17f	chr1:20777145-20784270	OtA5088-OtA5092	OtA5093-OtA5097
Il18	chr9:50565367-50581837	OtA5088-OtA5092	OtA5093-OtA5097
Il18r1	chr1:40465551-40500854	OtA5088-OtA5092	OtA5093-OtA5097
Il18rap	chr1:40515361-40549297	OtA5088-OtA5092	OtA5093-OtA5097
Il19	chr1:130932655-130939241	OtA5088-OtA5092	OtA5093-OtA5097
Il1a	chr2:129297369-129309972	OtA5088-OtA5092	OtA5093-OtA5097
Il1b	chr2:129364579-129375733	OtA5088-OtA5092	OtA5093-OtA5097
Il1f10	chr2:24291195-24293820	OtA5088-OtA5092	OtA5093-OtA5097
Il1f5	chr2:24276953-24282432	OtA5088-OtA5092	OtA5093-OtA5097
Il1f6	chr2:24215416-24225701	OtA5088-OtA5092	OtA5093-OtA5097
Il1f8	chr2:24153171-24160103	OtA5088-OtA5092	OtA5093-OtA5097
Il1f9	chr2:24186475-24193567	OtA5088-OtA5092	OtA5093-OtA5097
Il1rapl1	chrX:86747241-88115623	OtA5088-OtA5092	OtA5093-OtA5097
Il1rapl2	chrX:137571548-138846955	OtA5088-OtA5092	OtA5093-OtA5097
Il1rl1	chr1:40439648-40465414	OtA5088-OtA5092	OtA5093-OtA5097
Il1rl2	chr1:40324626-40365471	OtA5088-OtA5092	OtA5093-OtA5097
Il1rn	chr2:24336859-24351491	OtA5088-OtA5092	OtA5093-OtA5097
Il2	chr3:37120715-37125954	OtA5088-OtA5092	OtA5093-OtA5097
Il20	chr1:130907191-130911296	OtA5088-OtA5092	OtA5093-OtA5097
Il20ra	chr10:19712586-19760053	OtA5088-OtA5092	OtA5093-OtA5097
Il20rb	chr9:100457718-100486473	OtA5088-OtA5092	OtA5093-OtA5097
Il21	chr3:37222758-37232618	OtA5088-OtA5092	OtA5093-OtA5097

Il22	chr10:118204963-118210046	OtA5088-OtA5092	OtA5093-OtA5097
Il22ra2	chr10:19622027-19634681	OtA5088-OtA5092	OtA5093-OtA5097
Il23a	chr10:128296139-128298084	OtA5088-OtA5092	OtA5093-OtA5097
Il23r	chr6:67422931-67491855	OtA5088-OtA5092	OtA5093-OtA5097
Il24	chr1:130882073-130887411	OtA5088-OtA5092	OtA5093-OtA5097
Il25	chr14:54932694-54935836	OtA5088-OtA5092	OtA5093-OtA5097
Il27	chr7:126589294-126594910	OtA5088-OtA5092	OtA5093-OtA5097
Il27ra	chr8:84021473-84042575	OtA5088-OtA5092	OtA5093-OtA5097
Il28a	chr7:28508860-28510360	OtA5088-OtA5092	OtA5093-OtA5097
Il28b	chr7:28522835-28524322	OtA5088-OtA5092	OtA5093-OtA5097
Il2ra	chr2:11642791-11693194	OtA5088-OtA5092	OtA5093-OtA5097
Il3	chr11:54265084-54267279	OtA5088-OtA5092	OtA5093-OtA5097
Il31	chr5:123480152-123482101	OtA5088-OtA5092	OtA5093-OtA5097
Il31ra	chr13:112522806-112580662	OtA5088-OtA5092	OtA5093-OtA5097
Il33	chr19:29925113-29960715	OtA5088-OtA5092	OtA5093-OtA5097
Il4	chr11:53612460-53618665	OtA5088-OtA5092	OtA5093-OtA5097
Il4i1	chr7:44812255-44849079	OtA5088-OtA5092	OtA5093-OtA5097
Il5	chr11:53720793-53725103	OtA5088-OtA5092	OtA5093-OtA5097
Il5ra	chr6:106710378-106749037	OtA5088-OtA5092	OtA5093-OtA5097
Il6	chr5:30013160-30019968	OtA5088-OtA5092	OtA5093-OtA5097
Il7	chr3:7572076-7613427	OtA5088-OtA5092	OtA5093-OtA5097
Il7r	chr15:9506158-9529876	OtA5088-OtA5092	OtA5093-OtA5097
Il9	chr13:56479276-56482246	OtA5088-OtA5092	OtA5093-OtA5097
Il9r	chr11:32188996-32200279	OtA5088-OtA5092	OtA5093-OtA5097
Iltifb	chr10:118289629-118295038	OtA5088-OtA5092	OtA5093-OtA5097
Immp2l	chr12:41628763-41955586	OtA5088-OtA5092	OtA5093-OtA5097
Impg1	chr9:80313329-80465438	OtA5088-OtA5092	OtA5093-OtA5097
Impg2	chr16:56204312-56273753	OtA5088-OtA5092	OtA5093-OtA5097
Ina	chr19:46886830-47024346	OtA5088-OtA5092	OtA5093-OtA5097
Inhbc	chr10:127356325-127370544	OtA5088-OtA5092	OtA5093-OtA5097
Inhbe	chr10:127349401-127351772	OtA5088-OtA5092	OtA5093-OtA5097
Inpp4b	chr8:81715199-82122561	OtA5088-OtA5092	OtA5093-OtA5097
Inpp5j	chr11:3494271-3504821	OtA5088-OtA5092	OtA5093-OtA5097
Ins1	chr19:52264322-52265458	OtA5088-OtA5092	OtA5093-OtA5097
Ins2	chr7:142678655-142679708	OtA5088-OtA5092	OtA5093-OtA5097
Insl3	chr8:71676382-71690577	OtA5088-OtA5092	OtA5093-OtA5097
Insl5	chr4:103017872-103026728	OtA5088-OtA5092	OtA5093-OtA5097
Insm1	chr2:146221996-146225018	OtA5088-OtA5092	OtA5093-OtA5097
Insm2	chr12:55598916-55602017	OtA5088-OtA5092	OtA5093-OtA5097
Insrr	chr3:87796950-87816101	OtA5088-OtA5092	OtA5093-OtA5097
Intu	chr3:40653701-40704740	OtA5088-OtA5092	OtA5093-OtA5097
Ip6k3	chr17:27143970-27167764	OtA5088-OtA5092	OtA5093-OtA5097
Ipcef1	chr10:6788600-7038209	OtA5088-OtA5092	OtA5093-OtA5097
Ipw	chr7:59619157-59678878	OtA5088-OtA5092	OtA5093-OtA5097
Iqca	chr1:90042131-90153401	OtA5088-OtA5092	OtA5093-OtA5097
Iqcd	chr5:120589022-120607113	OtA5088-OtA5092	OtA5093-OtA5097
Iqcf1	chr9:106499966-106502250	OtA5088-OtA5092	OtA5093-OtA5097

Iqcf3	chr9:106543388-106561625	OtA5088-OtA5092	OtA5093-OtA5097
Iqcf4	chr9:106568318-106570967	OtA5088-OtA5092	OtA5093-OtA5097
Iqcf5	chr9:106514572-106516003	OtA5088-OtA5092	OtA5093-OtA5097
Iqcf6	chr9:106626581-106627612	OtA5088-OtA5092	OtA5093-OtA5097
Iqcg	chr16:32914099-33056186	OtA5088-OtA5092	OtA5093-OtA5097
Iqch	chr9:63421619-63602448	OtA5088-OtA5092	OtA5093-OtA5097
Iqcj	chr3:67892219-68626482	OtA5088-OtA5092	OtA5093-OtA5097
Iqcj-schip1	chr3:67892219-68626482	OtA5088-OtA5092	OtA5093-OtA5097
Iqsec3	chr6:121372932-121473678	OtA5088-OtA5092	OtA5093-OtA5097
Iqub	chr6:24444864-24515067	OtA5088-OtA5092	OtA5093-OtA5097
Irak1bp1	chr9:82829805-82847688	OtA5088-OtA5092	OtA5093-OtA5097
Irf4	chr13:30749257-30766927	OtA5088-OtA5092	OtA5093-OtA5097
Irg1	chr14:103047011-103056573	OtA5088-OtA5092	OtA5093-OtA5097
Irgc1	chr7:24431925-24445682	OtA5088-OtA5092	OtA5093-OtA5097
Irs4	chrX:141710997-141725217	OtA5088-OtA5092	OtA5093-OtA5097
Irx1	chr13:71958231-71963723	OtA5088-OtA5092	OtA5093-OtA5097
Irx5	chr8:92357795-92361456	OtA5088-OtA5092	OtA5093-OtA5097
Irx6	chr8:92674401-92680080	OtA5088-OtA5092	OtA5093-OtA5097
Isl1	chr13:116298269-116309688	OtA5088-OtA5092	OtA5093-OtA5097
Isl2	chr9:55541148-55546178	OtA5088-OtA5092	OtA5093-OtA5097
Isx	chr8:74873173-74893506	OtA5088-OtA5092	OtA5093-OtA5097
Itga10	chr3:96645583-96664519	OtA5088-OtA5092	OtA5093-OtA5097
Itga11	chr9:62677854-62783979	OtA5088-OtA5092	OtA5093-OtA5097
Itga4	chr2:79255425-79428988	OtA5088-OtA5092	OtA5093-OtA5097
Itgad	chr7:128173945-128206366	OtA5088-OtA5092	OtA5093-OtA5097
Itgae	chr11:73090594-73147449	OtA5088-OtA5092	OtA5093-OtA5097
Itgal	chr7:127296259-127335137	OtA5088-OtA5092	OtA5093-OtA5097
Itgax	chr7:128129567-128150657	OtA5088-OtA5092	OtA5093-OtA5097
Itgb1bp2	chrX:101449108-101453541	OtA5088-OtA5092	OtA5093-OtA5097
Itgb1bp3	chr10:81194690-81230779	OtA5088-OtA5092	OtA5093-OtA5097
Itgb2l	chr16:96422297-96443614	OtA5088-OtA5092	OtA5093-OtA5097
Itgb3bp	chr4:99765401-99829118	OtA5088-OtA5092	OtA5093-OtA5097
Itk	chr11:46325149-46389515	OtA5088-OtA5092	OtA5093-OtA5097
Itln1	chr1:171518123-171535294	OtA5088-OtA5092	OtA5093-OtA5097
Ivl	chr3:92570901-92573715	OtA5088-OtA5092	OtA5093-OtA5097
Iyd	chr10:3540278-3554877	OtA5088-OtA5092	OtA5093-OtA5097
Izumo1	chr7:45621810-45627242	OtA5088-OtA5092	OtA5093-OtA5097
Izumo2	chr7:44708742-44719842	OtA5088-OtA5092	OtA5093-OtA5097
Izumo3	chr4:92144317-92147221	OtA5088-OtA5092	OtA5093-OtA5097
Jakmip1	chr5:37050856-37125298	OtA5088-OtA5092	OtA5093-OtA5097
Jakmip2	chr18:43531407-43687773	OtA5088-OtA5092	OtA5093-OtA5097
Jakmip3	chr7:139062606-139083976	OtA5088-OtA5092	OtA5093-OtA5097
Jph1	chr1:16994939-17097889	OtA5088-OtA5092	OtA5093-OtA5097
Jph3	chr8:121730558-121791083	OtA5088-OtA5092	OtA5093-OtA5097
Jph4	chr14:55106825-55116935	OtA5088-OtA5092	OtA5093-OtA5097
Jrkl	chr9:13242789-13245741	OtA5088-OtA5092	OtA5093-OtA5097
Jsrp1	chr10:80808495-80813498	OtA5088-OtA5092	OtA5093-OtA5097

Katnal2	chr18:76993199-77047296	OtA5088-OtA5092	OtA5093-OtA5097
Kazald1	chr19:45076138-45079289	OtA5088-OtA5092	OtA5093-OtA5097
Kbtbd10	chr2:69670119-69684239	OtA5088-OtA5092	OtA5093-OtA5097
Kbtbd12	chr6:88613750-88627445	OtA5088-OtA5092	OtA5093-OtA5097
Kbtbd13	chr9:65388683-65391652	OtA5088-OtA5092	OtA5093-OtA5097
Kbtbd3	chr9:4309742-4331732	OtA5088-OtA5092	OtA5093-OtA5097
Kbtbd5	chr9:121777606-121783819	OtA5088-OtA5092	OtA5093-OtA5097
Kbtbd8	chr6:95117905-95129787	OtA5088-OtA5092	OtA5093-OtA5097
Kcna1	chr6:126636462-126645801	OtA5088-OtA5092	OtA5093-OtA5097
Kcna10	chr3:107183142-107195721	OtA5088-OtA5092	OtA5093-OtA5097
Kcna2	chr3:107101566-107115005	OtA5088-OtA5092	OtA5093-OtA5097
Kcna3	chr3:107036161-107038129	OtA5088-OtA5092	OtA5093-OtA5097
Kcna4	chr2:107290588-107298504	OtA5088-OtA5092	OtA5093-OtA5097
Kcna5	chr6:126532550-126535555	OtA5088-OtA5092	OtA5093-OtA5097
Kcna6	chr6:126708328-126740674	OtA5088-OtA5092	OtA5093-OtA5097
Kcna7	chr7:45405959-45411382	OtA5088-OtA5092	OtA5093-OtA5097
Kcnab1	chr3:65109642-65378219	OtA5088-OtA5092	OtA5093-OtA5097
Kcnab2	chr4:152390739-152477549	OtA5088-OtA5092	OtA5093-OtA5097
Kcnab3	chr11:69326257-69333041	OtA5088-OtA5092	OtA5093-OtA5097
Kcnb2	chr1:15312451-15714214	OtA5088-OtA5092	OtA5093-OtA5097
Kcnc1	chr7:46396467-46438704	OtA5088-OtA5092	OtA5093-OtA5097
Kcnc2	chr10:112271122-112466304	OtA5088-OtA5092	OtA5093-OtA5097
Kcnc3	chr7:44590885-44604751	OtA5088-OtA5092	OtA5093-OtA5097
Kcnd1	chrX:7823842-7836503	OtA5088-OtA5092	OtA5093-OtA5097
Kcnd2	chr6:21216108-21729805	OtA5088-OtA5092	OtA5093-OtA5097
Kcnd3	chr3:105448189-105674002	OtA5088-OtA5092	OtA5093-OtA5097
Kcne1	chr16:92346000-92359468	OtA5088-OtA5092	OtA5093-OtA5097
Kcne1l	chrX:142304752-142306198	OtA5088-OtA5092	OtA5093-OtA5097
Kcne2	chr16:92292388-92298133	OtA5088-OtA5092	OtA5093-OtA5097
Kcne3	chr7:100176669-100184869	OtA5088-OtA5092	OtA5093-OtA5097
Kcnf1	chr12:17172099-17176888	OtA5088-OtA5092	OtA5093-OtA5097
Kcng1	chr2:168261697-168269331	OtA5088-OtA5092	OtA5093-OtA5097
Kcng2	chr18:80294543-80364254	OtA5088-OtA5092	OtA5093-OtA5097
Kcng3	chr17:83585956-83631895	OtA5088-OtA5092	OtA5093-OtA5097
Kcng4	chr8:119623853-119635680	OtA5088-OtA5092	OtA5093-OtA5097
Kcnh2	chr5:24319588-24351604	OtA5088-OtA5092	OtA5093-OtA5097
Kcnh3	chr15:99224975-99262041	OtA5088-OtA5092	OtA5093-OtA5097
Kcnh4	chr11:100740377-100759778	OtA5088-OtA5092	OtA5093-OtA5097
Kcnh5	chr12:74897216-75177332	OtA5088-OtA5092	OtA5093-OtA5097
Kcnh6	chr11:106008202-106034064	OtA5088-OtA5092	OtA5093-OtA5097
Kcnh7	chr2:62702945-63184287	OtA5088-OtA5092	OtA5093-OtA5097
Kcnh8	chr17:52602762-52979033	OtA5088-OtA5092	OtA5093-OtA5097
Kcnip1	chr11:33629340-33993193	OtA5088-OtA5092	OtA5093-OtA5097
Kcnip2	chr19:45793669-45815803	OtA5088-OtA5092	OtA5093-OtA5097
Kcnip3	chr2:127456497-127521370	OtA5088-OtA5092	OtA5093-OtA5097
Kcnip4	chr5:48389502-49285659	OtA5088-OtA5092	OtA5093-OtA5097
Kcnj1	chr9:32372417-32399194	OtA5088-OtA5092	OtA5093-OtA5097

Kcnj10	chr1:172341209-172374085	OtA5088-OtA5092	OtA5093-OtA5097
Kcnj11	chr7:46097122-46100764	OtA5088-OtA5092	OtA5093-OtA5097
Kcnj14	chr7:45816466-45824747	OtA5088-OtA5092	OtA5093-OtA5097
Kcnj16	chr11:110968032-111027967	OtA5088-OtA5092	OtA5093-OtA5097
Kcnj2	chr11:111066163-111076825	OtA5088-OtA5092	OtA5093-OtA5097
Kcnj3	chr2:55437155-55595525	OtA5088-OtA5092	OtA5093-OtA5097
Kcnj4	chr15:79483713-79505241	OtA5088-OtA5092	OtA5093-OtA5097
Kcnj5	chr9:32314782-32344237	OtA5088-OtA5092	OtA5093-OtA5097
Kcnj6	chr16:94748682-94997696	OtA5088-OtA5092	OtA5093-OtA5097
Kcnj9	chr1:172321032-172329263	OtA5088-OtA5092	OtA5093-OtA5097
Kcnk10	chr12:98433993-98577940	OtA5088-OtA5092	OtA5093-OtA5097
Kcnk12	chr17:87745820-87797994	OtA5088-OtA5092	OtA5093-OtA5097
Kcnk13	chr12:99964498-100062682	OtA5088-OtA5092	OtA5093-OtA5097
Kcnk15	chr2:163853749-163858874	OtA5088-OtA5092	OtA5093-OtA5097
Kcnk18	chr19:59219647-59237370	OtA5088-OtA5092	OtA5093-OtA5097
Kcnk2	chr1:189207929-189344192	OtA5088-OtA5092	OtA5093-OtA5097
Kcnk3	chr5:30573986-30625270	OtA5088-OtA5092	OtA5093-OtA5097
Kcnk4	chr19:6925689-6934515	OtA5088-OtA5092	OtA5093-OtA5097
Kcnk7	chr19:5704475-5707101	OtA5088-OtA5092	OtA5093-OtA5097
Kcnk9	chr15:72512118-72546279	OtA5088-OtA5092	OtA5093-OtA5097
Kcnma1	chr14:23298693-24004205	OtA5088-OtA5092	OtA5093-OtA5097
Kcnmb1	chr11:33629340-33993193	OtA5088-OtA5092	OtA5093-OtA5097
Kcnmb2	chr3:31902702-32200180	OtA5088-OtA5092	OtA5093-OtA5097
Kcnmb3	chr3:32472320-32491969	OtA5088-OtA5092	OtA5093-OtA5097
Kcnmb4	chr10:116417867-116473523	OtA5088-OtA5092	OtA5093-OtA5097
Kcnn1	chr8:70842048-70857008	OtA5088-OtA5092	OtA5093-OtA5097
Kcnn2	chr18:45560153-46045260	OtA5088-OtA5092	OtA5093-OtA5097
Kcnn3	chr3:89520163-89672494	OtA5088-OtA5092	OtA5093-OtA5097
Kcnq1	chr7:143107253-143427042	OtA5088-OtA5092	OtA5093-OtA5097
Kcnq2	chr2:181075578-181135225	OtA5088-OtA5092	OtA5093-OtA5097
Kcnq3	chr15:65994914-66286224	OtA5088-OtA5092	OtA5093-OtA5097
Kcnq5	chr1:21398402-21961942	OtA5088-OtA5092	OtA5093-OtA5097
Kcnrg	chr14:61598225-61682373	OtA5088-OtA5092	OtA5093-OtA5097
Kcns1	chr2:164163618-164171113	OtA5088-OtA5092	OtA5093-OtA5097
Kcns2	chr15:34837380-34842337	OtA5088-OtA5092	OtA5093-OtA5097
Kcns3	chr12:11090201-11150842	OtA5088-OtA5092	OtA5093-OtA5097
Kcnt1	chr2:25863853-25918273	OtA5088-OtA5092	OtA5093-OtA5097
Kcnt2	chr1:140246256-140656402	OtA5088-OtA5092	OtA5093-OtA5097
Kcnu1	chr8:25849622-25937934	OtA5088-OtA5092	OtA5093-OtA5097
Kcnv1	chr15:45106283-45114934	OtA5088-OtA5092	OtA5093-OtA5097
Kcp	chr6:29482035-29507952	OtA5088-OtA5092	OtA5093-OtA5097
Kctd14	chr7:97451333-97459557	OtA5088-OtA5092	OtA5093-OtA5097
Kctd16	chr18:40258360-40531184	OtA5088-OtA5092	OtA5093-OtA5097
Kctd19	chr8:105375380-105413487	OtA5088-OtA5092	OtA5093-OtA5097
Kctd4	chr14:75896936-76010865	OtA5088-OtA5092	OtA5093-OtA5097
Kctd8	chr5:69109284-69341709	OtA5088-OtA5092	OtA5093-OtA5097
Kdm4d	chr9:14462580-14500482	OtA5088-OtA5092	OtA5093-OtA5097

Kdm5d	chrY:897787-943811	OtA5088-OtA5092	OtA5093-OtA5097
Keg1	chr19:12695789-12719896	OtA5088-OtA5092	OtA5093-OtA5097
Kel	chr6:41686329-41704325	OtA5088-OtA5092	OtA5093-OtA5097
Kera	chr10:97607204-97613688	OtA5088-OtA5092	OtA5093-OtA5097
Khdc1a	chr1:21349676-21352199	OtA5088-OtA5092	OtA5093-OtA5097
Khdc1b	chr1:21383555-21386384	OtA5088-OtA5092	OtA5093-OtA5097
Khdc1c	chr1:21368330-21369743	OtA5088-OtA5092	OtA5093-OtA5097
Khdrbs2	chr1:32172805-32657738	OtA5088-OtA5092	OtA5093-OtA5097
Kif11	chr19:37376402-37421859	OtA5088-OtA5092	OtA5093-OtA5097
Kif12	chr4:63165636-63172131	OtA5088-OtA5092	OtA5093-OtA5097
Kif14	chr1:136467957-136531511	OtA5088-OtA5092	OtA5093-OtA5097
Kif15	chr9:122951080-123018733	OtA5088-OtA5092	OtA5093-OtA5097
Kif17	chr4:138262250-138301973	OtA5088-OtA5092	OtA5093-OtA5097
Kif18a	chr2:109280737-109341746	OtA5088-OtA5092	OtA5093-OtA5097
Kif18b	chr11:102905518-102925124	OtA5088-OtA5092	OtA5093-OtA5097
Kif19a	chr11:114765388-114790575	OtA5088-OtA5092	OtA5093-OtA5097
Kif1a	chr1:93015455-93101865	OtA5088-OtA5092	OtA5093-OtA5097
Kif20b	chr19:34922357-34975731	OtA5088-OtA5092	OtA5093-OtA5097
Kif22	chr7:127027730-127042420	OtA5088-OtA5092	OtA5093-OtA5097
Kif23	chr9:61917277-61946799	OtA5088-OtA5092	OtA5093-OtA5097
Kif24	chr4:41390747-41464848	OtA5088-OtA5092	OtA5093-OtA5097
Kif26b	chr1:178529124-178932857	OtA5088-OtA5092	OtA5093-OtA5097
Kif27	chr13:58287515-58354862	OtA5088-OtA5092	OtA5093-OtA5097
Kif2b	chr11:91575285-91577555	OtA5088-OtA5092	OtA5093-OtA5097
Kif2c	chr4:117159635-117182624	OtA5088-OtA5092	OtA5093-OtA5097
Kif4	chrX:100626064-100727271	OtA5088-OtA5092	OtA5093-OtA5097
Kif4-ps	chr12:101145608-101149273	OtA5088-OtA5092	OtA5093-OtA5097
Kif6	chr17:49615171-49909847	OtA5088-OtA5092	OtA5093-OtA5097
Kif7	chr7:79660195-79714186	OtA5088-OtA5092	OtA5093-OtA5097
Kifc5b	chr17:26917090-26932579	OtA5088-OtA5092	OtA5093-OtA5097
Kir3dl1	chrX:136517998-136534308	OtA5088-OtA5092	OtA5093-OtA5097
Kir3dl2	chrX:136448106-136469041	OtA5088-OtA5092	OtA5093-OtA5097
Kirrel2	chr7:30447765-30457515	OtA5088-OtA5092	OtA5093-OtA5097
Kis2	chrX:52742502-52744593	OtA5088-OtA5092	OtA5093-OtA5097
Kiss1	chr1:133327211-133329722	OtA5088-OtA5092	OtA5093-OtA5097
Kl	chr5:150952606-150993817	OtA5088-OtA5092	OtA5093-OtA5097
Klf1	chr8:84901927-84905295	OtA5088-OtA5092	OtA5093-OtA5097
Klf12	chr14:99870639-100149796	OtA5088-OtA5092	OtA5093-OtA5097
Klf17	chr4:117756658-117765666	OtA5088-OtA5092	OtA5093-OtA5097
Klhdc7a	chr4:139962172-139968026	OtA5088-OtA5092	OtA5093-OtA5097
Klhdc7b	chr15:89386890-89388708	OtA5088-OtA5092	OtA5093-OtA5097
Klhdc8a	chr1:132298625-132307357	OtA5088-OtA5092	OtA5093-OtA5097
Klhdc9	chr1:171358448-171360798	OtA5088-OtA5092	OtA5093-OtA5097
Klhl1	chr14:96105264-96519034	OtA5088-OtA5092	OtA5093-OtA5097
Klhl10	chr11:100441923-100457024	OtA5088-OtA5092	OtA5093-OtA5097
Klhl14	chr18:21550376-21652368	OtA5088-OtA5092	OtA5093-OtA5097
Klhl23	chr2:69822369-69836651	OtA5088-OtA5092	OtA5093-OtA5097

Klhl3	chr13:58004956-58102428	OtA5088-OtA5092	OtA5093-OtA5097
Klhl31	chr9:77636731-77660122	OtA5088-OtA5092	OtA5093-OtA5097
Klhl32	chr4:24617272-24851086	OtA5088-OtA5092	OtA5093-OtA5097
Klhl33	chr14:50891388-50893255	OtA5088-OtA5092	OtA5093-OtA5097
Klhl34	chrX:157818434-157820369	OtA5088-OtA5092	OtA5093-OtA5097
Klhl35	chr7:99466003-99474020	OtA5088-OtA5092	OtA5093-OtA5097
Klhl38	chr15:58314572-58324169	OtA5088-OtA5092	OtA5093-OtA5097
Klhl4	chrX:114474519-114560698	OtA5088-OtA5092	OtA5093-OtA5097
Klhl6	chr16:19946491-19983049	OtA5088-OtA5092	OtA5093-OtA5097
Klk1	chr7:44225436-44229617	OtA5088-OtA5092	OtA5093-OtA5097
Klk11	chr7:43774616-43779262	OtA5088-OtA5092	OtA5093-OtA5097
Klk12	chr7:43769099-43773481	OtA5088-OtA5092	OtA5093-OtA5097
Klk13	chr7:43712566-43726758	OtA5088-OtA5092	OtA5093-OtA5097
Klk14	chr7:43690417-43695535	OtA5088-OtA5092	OtA5093-OtA5097
Klk1b1	chr7:43966767-43971315	OtA5088-OtA5092	OtA5093-OtA5097
Klk1b11	chr7:43995878-43999875	OtA5088-OtA5092	OtA5093-OtA5097
Klk1b16	chr7:44136766-44141604	OtA5088-OtA5092	OtA5093-OtA5097
Klk1b21	chr7:44102291-44106579	OtA5088-OtA5092	OtA5093-OtA5097
Klk1b22	chr7:44112682-44116876	OtA5088-OtA5092	OtA5093-OtA5097
Klk1b24	chr7:44188262-44192451	OtA5088-OtA5092	OtA5093-OtA5097
Klk1b26	chr7:44012677-44016969	OtA5088-OtA5092	OtA5093-OtA5097
Klk1b27	chr7:44052289-44056711	OtA5088-OtA5092	OtA5093-OtA5097
Klk1b3	chr7:44198190-44202351	OtA5088-OtA5092	OtA5093-OtA5097
Klk1b4	chr7:44207434-44211754	OtA5088-OtA5092	OtA5093-OtA5097
Klk1b5	chr7:44216474-44220703	OtA5088-OtA5092	OtA5093-OtA5097
Klk1b7-ps	chr7:43945011-43945927	OtA5088-OtA5092	OtA5093-OtA5097
Klk1b8	chr7:43950671-43954938	OtA5088-OtA5092	OtA5093-OtA5097
Klk1b9	chr7:43976060-43980376	OtA5088-OtA5092	OtA5093-OtA5097
Klk5	chr7:43842268-43851181	OtA5088-OtA5092	OtA5093-OtA5097
Klk9	chr7:43791890-43796756	OtA5088-OtA5092	OtA5093-OtA5097
Klkb1	chr8:45269450-45294835	OtA5088-OtA5092	OtA5093-OtA5097
Klra1	chr6:129972081-130386874	OtA5088-OtA5092	OtA5093-OtA5097
Klra10	chr6:129972081-130386874	OtA5088-OtA5092	OtA5093-OtA5097
Klra12	chr6:129972081-130386874	OtA5088-OtA5092	OtA5093-OtA5097
Klra13-ps	chr6:129972081-130386874	OtA5088-OtA5092	OtA5093-OtA5097
Klra14	chr6:129972081-130386874	OtA5088-OtA5092	OtA5093-OtA5097
Klra15	chr6:129972081-130386874	OtA5088-OtA5092	OtA5093-OtA5097
Klra17	chr6:129831153-129876672	OtA5088-OtA5092	OtA5093-OtA5097
Klra18,Klra33,Klra4	chr6:129972081-130386874	OtA5088-OtA5092	OtA5093-OtA5097
Klra19,Klra6	chr6:129972081-130386874	OtA5088-OtA5092	OtA5093-OtA5097
Klra2	chr6:131219234-131247362	OtA5088-OtA5092	OtA5093-OtA5097
Klra21	chr6:129972081-130386874	OtA5088-OtA5092	OtA5093-OtA5097
Klra22	chr6:129972081-130386874	OtA5088-OtA5092	OtA5093-OtA5097
Klra23	chr6:129972081-130386874	OtA5088-OtA5092	OtA5093-OtA5097
Klra3	chr6:129972081-130386874	OtA5088-OtA5092	OtA5093-OtA5097
Klra33	chr6:129972081-130386874	OtA5088-OtA5092	OtA5093-OtA5097
Klra4	chr6:129972081-130386874	OtA5088-OtA5092	OtA5093-OtA5097

Klra5	chr6:129898991-129913224	OtA5088-OtA5092	OtA5093-OtA5097
Klra6	chr6:129972081-130386874	OtA5088-OtA5092	OtA5093-OtA5097
Klra7	chr6:129972081-130386874	OtA5088-OtA5092	OtA5093-OtA5097
Klra8	chr6:129972081-130386874	OtA5088-OtA5092	OtA5093-OtA5097
Klra9	chr6:129972081-130386874	OtA5088-OtA5092	OtA5093-OtA5097
Klrb1a	chr6:128609226-128622934	OtA5088-OtA5092	OtA5093-OtA5097
Klrb1b	chr6:128813705-128826315	OtA5088-OtA5092	OtA5093-OtA5097
Klrb1c	chr6:128778484-128788641	OtA5088-OtA5092	OtA5093-OtA5097
Klrb1f	chr6:129045900-129057471	OtA5088-OtA5092	OtA5093-OtA5097
Klrc1	chr6:129674858-129678910	OtA5088-OtA5092	OtA5093-OtA5097
Klrc2	chr6:129656345-129660596	OtA5088-OtA5092	OtA5093-OtA5097
Klrc3	chr6:129639084-129643288	OtA5088-OtA5092	OtA5093-OtA5097
Klrd1	chr6:129591810-129598775	OtA5088-OtA5092	OtA5093-OtA5097
Klre1	chr6:129578285-129585827	OtA5088-OtA5092	OtA5093-OtA5097
Klrg1	chr6:122270596-122282833	OtA5088-OtA5092	OtA5093-OtA5097
Klrg2	chr6:38626659-38637239	OtA5088-OtA5092	OtA5093-OtA5097
Klri1	chr6:129697217-129717132	OtA5088-OtA5092	OtA5093-OtA5097
Klri2	chr6:129729040-129740484	OtA5088-OtA5092	OtA5093-OtA5097
Klrk1	chr6:129610320-129623841	OtA5088-OtA5092	OtA5093-OtA5097
Kmo	chr1:175632192-175660853	OtA5088-OtA5092	OtA5093-OtA5097
Kncn	chr4:115884399-115887964	OtA5088-OtA5092	OtA5093-OtA5097
Kndc1	chr7:139894695-139941540	OtA5088-OtA5092	OtA5093-OtA5097
Kntc1	chr5:123749725-123821593	OtA5088-OtA5092	OtA5093-OtA5097
Kpna7	chr5:144983743-145009636	OtA5088-OtA5092	OtA5093-OtA5097
Kprp	chr3:92823073-92827247	OtA5088-OtA5092	OtA5093-OtA5097
Kremen2	chr17:23741198-23745829	OtA5088-OtA5092	OtA5093-OtA5097
Krt1	chr15:101845425-101850786	OtA5088-OtA5092	OtA5093-OtA5097
Krt10	chr11:99385255-99389364	OtA5088-OtA5092	OtA5093-OtA5097
Krt12	chr11:99415663-99422259	OtA5088-OtA5092	OtA5093-OtA5097
Krt16	chr11:100246090-100248902	OtA5088-OtA5092	OtA5093-OtA5097
Krt17	chr11:100256216-100260989	OtA5088-OtA5092	OtA5093-OtA5097
Krt2	chr15:101810688-101818169	OtA5088-OtA5092	OtA5093-OtA5097
Krt20	chr11:99428402-99438153	OtA5088-OtA5092	OtA5093-OtA5097
Krt222	chr11:99233097-99244067	OtA5088-OtA5092	OtA5093-OtA5097
Krt24	chr11:99280092-99285238	OtA5088-OtA5092	OtA5093-OtA5097
Krt26	chr11:99328483-99337965	OtA5088-OtA5092	OtA5093-OtA5097
Krt27	chr11:99345565-99351094	OtA5088-OtA5092	OtA5093-OtA5097
Krt28	chr11:99365006-99374903	OtA5088-OtA5092	OtA5093-OtA5097
Krt31	chr11:100046645-100050551	OtA5088-OtA5092	OtA5093-OtA5097
Krt32	chr11:100080847-100088226	OtA5088-OtA5092	OtA5093-OtA5097
Krt33a	chr11:100011198-100016212	OtA5088-OtA5092	OtA5093-OtA5097
Krt33b	chr11:100023633-100029868	OtA5088-OtA5092	OtA5093-OtA5097
Krt34	chr11:100037350-100041554	OtA5088-OtA5092	OtA5093-OtA5097
Krt35	chr11:100092191-100096224	OtA5088-OtA5092	OtA5093-OtA5097
Krt36	chr11:100102012-100105626	OtA5088-OtA5092	OtA5093-OtA5097
Krt39	chr11:99514623-99521258	OtA5088-OtA5092	OtA5093-OtA5097
Krt4	chr15:101918534-101924735	OtA5088-OtA5092	OtA5093-OtA5097

Krt40	chr11:99537484-99543158	OtA5088-OtA5092	OtA5093-OtA5097
Krt42	chr11:100262881-100269871	OtA5088-OtA5092	OtA5093-OtA5097
Krt5	chr15:101707069-101712891	OtA5088-OtA5092	OtA5093-OtA5097
Krt6b	chr15:101676022-101680289	OtA5088-OtA5092	OtA5093-OtA5097
Krt71	chr15:101733948-101743097	OtA5088-OtA5092	OtA5093-OtA5097
Krt72-ps	chr15:101776559-101786458	OtA5088-OtA5092	OtA5093-OtA5097
Krt73	chr15:101793307-101802332	OtA5088-OtA5092	OtA5093-OtA5097
Krt74	chr15:101754258-101763504	OtA5088-OtA5092	OtA5093-OtA5097
Krt75	chr15:101563342-101573904	OtA5088-OtA5092	OtA5093-OtA5097
Krt76	chr15:101884350-101892920	OtA5088-OtA5092	OtA5093-OtA5097
Krt77	chr15:101859855-101869618	OtA5088-OtA5092	OtA5093-OtA5097
Krt78	chr15:101946003-101954287	OtA5088-OtA5092	OtA5093-OtA5097
Krt79	chr15:101929331-101940324	OtA5088-OtA5092	OtA5093-OtA5097
Krt81	chr15:101431013-101491512	OtA5088-OtA5092	OtA5093-OtA5097
Krt82	chr15:101541220-101550659	OtA5088-OtA5092	OtA5093-OtA5097
Krt83	chr15:101431013-101491512	OtA5088-OtA5092	OtA5093-OtA5097
Krt84	chr15:101525025-101532820	OtA5088-OtA5092	OtA5093-OtA5097
Krt85	chr15:101431013-101491512	OtA5088-OtA5092	OtA5093-OtA5097
Krt86	chr15:101431013-101491512	OtA5088-OtA5092	OtA5093-OtA5097
Krt9	chr11:100186780-100193246	OtA5088-OtA5092	OtA5093-OtA5097
Krtap1-3	chr11:99590460-99591339	OtA5088-OtA5092	OtA5093-OtA5097
Krtap1-4	chr11:99582548-99583677	OtA5088-OtA5092	OtA5093-OtA5097
Krtap1-5	chr11:99579976-99581016	OtA5088-OtA5092	OtA5093-OtA5097
Krtap10-10	chr10:77835946-77837251	OtA5088-OtA5092	OtA5093-OtA5097
Krtap10-4	chr10:77826168-77827070	OtA5088-OtA5092	OtA5093-OtA5097
Krtap11-1	chr16:89570175-89571183	OtA5088-OtA5092	OtA5093-OtA5097
Krtap12-1	chr10:77720585-77721256	OtA5088-OtA5092	OtA5093-OtA5097
Krtap13	chr16:88750743-88751628	OtA5088-OtA5092	OtA5093-OtA5097
Krtap13-1	chr16:88728861-88729611	OtA5088-OtA5092	OtA5093-OtA5097
Krtap14	chr16:88825290-88826145	OtA5088-OtA5092	OtA5093-OtA5097
Krtap15	chr16:88829008-88829844	OtA5088-OtA5092	OtA5093-OtA5097
Krtap16-1	chr16:88873664-88874269	OtA5088-OtA5092	OtA5093-OtA5097
Krtap16-10b	chr16:88931803-88932264	OtA5088-OtA5092	OtA5093-OtA5097
Krtap16-2	chr16:88868917-88869410	OtA5088-OtA5092	OtA5093-OtA5097
Krtap16-3	chr16:88962303-88962873	OtA5088-OtA5092	OtA5093-OtA5097
Krtap16-4	chr16:88884785-88885089	OtA5088-OtA5092	OtA5093-OtA5097
Krtap16-5	chr16:88877512-88878038	OtA5088-OtA5092	OtA5093-OtA5097
Krtap16-7	chr16:89403026-89403774	OtA5088-OtA5092	OtA5093-OtA5097
Krtap16-8	chr16:89047288-89047899	OtA5088-OtA5092	OtA5093-OtA5097
Krtap17-1	chr11:99993233-99993994	OtA5088-OtA5092	OtA5093-OtA5097
Krtap2-4	chr11:99614016-99614846	OtA5088-OtA5092	OtA5093-OtA5097
Krtap20-2	chr16:89205860-89206394	OtA5088-OtA5092	OtA5093-OtA5097
Krtap22-2	chr16:89010379-89010759	OtA5088-OtA5092	OtA5093-OtA5097
Krtap24-1	chr16:88610708-88612279	OtA5088-OtA5092	OtA5093-OtA5097
Krtap26-1	chr16:88646823-88647796	OtA5088-OtA5092	OtA5093-OtA5097
Krtap27-1	chr16:88671045-88671654	OtA5088-OtA5092	OtA5093-OtA5097
Krtap3-1	chr11:99566026-99566630	OtA5088-OtA5092	OtA5093-OtA5097

Krtap3-2	chr11:99555822-99556853	OtA5088-OtA5092	OtA5093-OtA5097
Krtap3-3	chr11:99550131-99550863	OtA5088-OtA5092	OtA5093-OtA5097
Krtap31-1	chr11:99907919-99908890	OtA5088-OtA5092	OtA5093-OtA5097
Krtap31-2	chr11:99936290-99937225	OtA5088-OtA5092	OtA5093-OtA5097
Krtap4-1	chr11:99627228-99628239	OtA5088-OtA5092	OtA5093-OtA5097
Krtap4-13	chr11:99798185-99809896	OtA5088-OtA5092	OtA5093-OtA5097
Krtap4-16	chr11:99850654-99851605	OtA5088-OtA5092	OtA5093-OtA5097
Krtap4-2	chr11:99634113-99635084	OtA5088-OtA5092	OtA5093-OtA5097
Krtap4-6	chr11:99665042-99665960	OtA5088-OtA5092	OtA5093-OtA5097
Krtap4-7	chr11:99643111-99644089	OtA5088-OtA5092	OtA5093-OtA5097
Krtap4-8	chr11:99780013-99780643	OtA5088-OtA5092	OtA5093-OtA5097
Krtap4-9	chr11:99785199-99786257	OtA5088-OtA5092	OtA5093-OtA5097
Krtap5-1	chr7:142296376-142297069	OtA5088-OtA5092	OtA5093-OtA5097
Krtap5-2	chr7:142174531-142176005	OtA5088-OtA5092	OtA5093-OtA5097
Krtap5-3	chr7:142201363-142203015	OtA5088-OtA5092	OtA5093-OtA5097
Krtap5-4	chr7:142303501-142304503	OtA5088-OtA5092	OtA5093-OtA5097
Krtap5-5	chr7:142228794-142229971	OtA5088-OtA5092	OtA5093-OtA5097
Krtap6-1	chr16:89031698-89032292	OtA5088-OtA5092	OtA5093-OtA5097
Krtap6-2	chr16:89419322-89420111	OtA5088-OtA5092	OtA5093-OtA5097
Krtap7-1	chr16:89507702-89508323	OtA5088-OtA5092	OtA5093-OtA5097
Krtap8-1	chr16:89487373-89487952	OtA5088-OtA5092	OtA5093-OtA5097
Krtap8-2	chr16:88895967-88896449	OtA5088-OtA5092	OtA5093-OtA5097
Krtap9-1	chr11:99873388-99874000	OtA5088-OtA5092	OtA5093-OtA5097
Krtap9-3	chr11:99597347-99598106	OtA5088-OtA5092	OtA5093-OtA5097
Krtap9-5	chr11:99948474-99949551	OtA5088-OtA5092	OtA5093-OtA5097
Krtdap	chr7:30787904-30791083	OtA5088-OtA5092	OtA5093-OtA5097
Ksr2	chr5:117414001-117767988	OtA5088-OtA5092	OtA5093-OtA5097
Ky	chr9:102506137-102546239	OtA5088-OtA5092	OtA5093-OtA5097
Kynu	chr2:43555328-43682715	OtA5088-OtA5092	OtA5093-OtA5097
L1cam	chrX:73853779-73880834	OtA5088-OtA5092	OtA5093-OtA5097
L3mbtl1	chr2:162943464-162974522	OtA5088-OtA5092	OtA5093-OtA5097
L3mbtl4	chr17:68273796-68780086	OtA5088-OtA5092	OtA5093-OtA5097
LOC100038947	chr3:15795146-15848487	OtA5088-OtA5092	OtA5093-OtA5097
LOC100039574	chrY:80756211-80757210	OtA5088-OtA5092	OtA5093-OtA5097
LOC100039614	chrY:72819514-72820507	OtA5088-OtA5092	OtA5093-OtA5097
LOC100039753	chrY:8832248-8835169	OtA5088-OtA5092	OtA5093-OtA5097
LOC100039810,LOC10(	chrY:64301780-64786299	OtA5088-OtA5092	OtA5093-OtA5097
LOC100040022	chrY:15882558-15883245	OtA5088-OtA5092	OtA5093-OtA5097
LOC100040031	chrY:72382444-72384781	OtA5088-OtA5092	OtA5093-OtA5097
LOC100040160	chrY:7636215-7636973	OtA5088-OtA5092	OtA5093-OtA5097
LOC100040223	chrY:17965159-17967598	OtA5088-OtA5092	OtA5093-OtA5097
LOC100040786	chrY:66739796-66742170	OtA5088-OtA5092	OtA5093-OtA5097
LOC100040911	chrY:85528524-85529519	OtA5088-OtA5092	OtA5093-OtA5097
LOC100041014	chrY:24188658-24214668	OtA5088-OtA5092	OtA5093-OtA5097
LOC100041033	chrY:26241091-26267093	OtA5088-OtA5092	OtA5093-OtA5097
LOC100041223	chrY:73239169-73240030	OtA5088-OtA5092	OtA5093-OtA5097
LOC100041256	chrY:64301780-64786299	OtA5088-OtA5092	OtA5093-OtA5097

LOC100041346	chrY:59606709-59632758	OtA5088-OtA5092	OtA5093-OtA5097
LOC100041550	chrY:17093878-17095887	OtA5088-OtA5092	OtA5093-OtA5097
LOC100042428	chrY:79148794-79149787	OtA5088-OtA5092	OtA5093-OtA5097
LOC100043315	chr15:95097621-95149318	OtA5088-OtA5092	OtA5093-OtA5097
LOC100044193	chr17:94864917-94878321	OtA5088-OtA5092	OtA5093-OtA5097
LOC100048884	chr4:61670177-61674094	OtA5088-OtA5092	OtA5093-OtA5097
LOC100302567	chr7:28350688-28352698	OtA5088-OtA5092	OtA5093-OtA5097
LOC100502924	chr4:73650909-73672397	OtA5088-OtA5092	OtA5093-OtA5097
LOC100503280	chrX:74966843-74969125	OtA5088-OtA5092	OtA5093-OtA5097
LOC100503496	chr11:109440077-109573329	OtA5088-OtA5092	OtA5093-OtA5097
LOC100504608	chr10:127032904-127041394	OtA5088-OtA5092	OtA5093-OtA5097
LOC100504703	chr10:127070480-127071101	OtA5088-OtA5092	OtA5093-OtA5097
LOC100642166	chr5:109933562-109941710	OtA5088-OtA5092	OtA5093-OtA5097
LOC100859962	chr7:80024813-80092751	OtA5088-OtA5092	OtA5093-OtA5097
LOC100861570	chr12:109603944-109661711	OtA5088-OtA5092	OtA5093-OtA5097
LOC100861571	chr12:109603944-109661711	OtA5088-OtA5092	OtA5093-OtA5097
LOC100861572	chr12:109603944-109661711	OtA5088-OtA5092	OtA5093-OtA5097
LOC171588	chr2:144459279-144467794	OtA5088-OtA5092	OtA5093-OtA5097
LOC319574	chr8:122443655-122451322	OtA5088-OtA5092	OtA5093-OtA5097
LOC329427	chr2:73596525-73775346	OtA5088-OtA5092	OtA5093-OtA5097
LOC330599	chr7:98121564-98184799	OtA5088-OtA5092	OtA5093-OtA5097
LOC380994	chrY:82225214-82251414	OtA5088-OtA5092	OtA5093-OtA5097
LOC382133	chrY:65205192-65207527	OtA5088-OtA5092	OtA5093-OtA5097
LOC432562	chr11:54819918-54824967	OtA5088-OtA5092	OtA5093-OtA5097
LOC432958	chr15:63414396-63479608	OtA5088-OtA5092	OtA5093-OtA5097
LOC433047	chr16:88811439-88812220	OtA5088-OtA5092	OtA5093-OtA5097
LOC433374	chr1:164863120-164871438	OtA5088-OtA5092	OtA5093-OtA5097
LOC433944	chr5:119685087-119691218	OtA5088-OtA5092	OtA5093-OtA5097
LOC434205	chr7:82070386-82081758	OtA5088-OtA5092	OtA5093-OtA5097
LOC434412	chr9:68017963-68025714	OtA5088-OtA5092	OtA5093-OtA5097
LOC434825	chrX:102252650-102257689	OtA5088-OtA5092	OtA5093-OtA5097
LOC434960	chrY:81799149-81801497	OtA5088-OtA5092	OtA5093-OtA5097
LOC545691	chr4:141890850-142011637	OtA5088-OtA5092	OtA5093-OtA5097
LOC622070	chr7:104299896-104324838	OtA5088-OtA5092	OtA5093-OtA5097
LOC622124	chr3:52906755-52927666	OtA5088-OtA5092	OtA5093-OtA5097
LOC622167	chr4:103382499-103492188	OtA5088-OtA5092	OtA5093-OtA5097
LOC622552	chr13:43716790-43719038	OtA5088-OtA5092	OtA5093-OtA5097
LOC624759	chr3:159076363-159308080	OtA5088-OtA5092	OtA5093-OtA5097
LOC626049	chr1:106152624-106171524	OtA5088-OtA5092	OtA5093-OtA5097
LOC626082	chr3:73066350-73594830	OtA5088-OtA5092	OtA5093-OtA5097
LOC626410	chr3:37898812-37992728	OtA5088-OtA5092	OtA5093-OtA5097
LOC626693	chr16:43247283-43619123	OtA5088-OtA5092	OtA5093-OtA5097
LOC626904	chr6:25931425-25977227	OtA5088-OtA5092	OtA5093-OtA5097
LOC627800	chr10:92171300-92375373	OtA5088-OtA5092	OtA5093-OtA5097
LOC629206	chr10:111854520-111874376	OtA5088-OtA5092	OtA5093-OtA5097
LOC629393	chr11:86002657-86024350	OtA5088-OtA5092	OtA5093-OtA5097
LOC639910	chr13:120140246-120155335	OtA5088-OtA5092	OtA5093-OtA5097

LOC666331	chr11:101610699-101615956	OtA5088-OtA5092	OtA5093-OtA5097
Lactbl1	chr4:136622620-136638110	OtA5088-OtA5092	OtA5093-OtA5097
Lag3	chr6:124904358-124911705	OtA5088-OtA5092	OtA5093-OtA5097
Lair1	chr7:4007072-4063204	OtA5088-OtA5092	OtA5093-OtA5097
Lalba	chr15:98480399-98482683	OtA5088-OtA5092	OtA5093-OtA5097
Lama3	chr18:12334023-12583012	OtA5088-OtA5092	OtA5093-OtA5097
Lamp3	chr16:19653380-19706365	OtA5088-OtA5092	OtA5093-OtA5097
Lancl3	chrX:9199972-9268085	OtA5088-OtA5092	OtA5093-OtA5097
Lao1	chr4:118961966-118968910	OtA5088-OtA5092	OtA5093-OtA5097
Lars2	chr9:123366939-123462664	OtA5088-OtA5092	OtA5093-OtA5097
Lass1	chr8:70315774-70353273	OtA5088-OtA5092	OtA5093-OtA5097
Lass3	chr7:66743503-66823692	OtA5088-OtA5092	OtA5093-OtA5097
Lat	chr7:126363827-126369534	OtA5088-OtA5092	OtA5093-OtA5097
Lax1	chr1:133679028-133690108	OtA5088-OtA5092	OtA5093-OtA5097
Lbx1	chr19:45233727-45235236	OtA5088-OtA5092	OtA5093-OtA5097
Lbx2	chr6:83086364-83088241	OtA5088-OtA5092	OtA5093-OtA5097
Lca5	chr9:83391354-83441127	OtA5088-OtA5092	OtA5093-OtA5097
Lca5l	chr16:96158405-96192257	OtA5088-OtA5092	OtA5093-OtA5097
Lce1a1	chr3:92646531-92648307	OtA5088-OtA5092	OtA5093-OtA5097
Lce1a2	chr3:92668612-92670315	OtA5088-OtA5092	OtA5093-OtA5097
Lce1b	chr3:92655649-92656926	OtA5088-OtA5092	OtA5093-OtA5097
Lce1c	chr3:92679246-92680918	OtA5088-OtA5092	OtA5093-OtA5097
Lce1d	chr3:92685498-92687210	OtA5088-OtA5092	OtA5093-OtA5097
Lce1e	chr3:92707399-92709074	OtA5088-OtA5092	OtA5093-OtA5097
Lce1f	chr3:92718695-92720350	OtA5088-OtA5092	OtA5093-OtA5097
Lce1g	chr3:92750150-92752338	OtA5088-OtA5092	OtA5093-OtA5097
Lce1h	chr3:92763214-92765065	OtA5088-OtA5092	OtA5093-OtA5097
Lce1i	chr3:92777209-92778899	OtA5088-OtA5092	OtA5093-OtA5097
Lce1k	chr3:92806290-92807891	OtA5088-OtA5092	OtA5093-OtA5097
Lce1l	chr3:92849948-92851286	OtA5088-OtA5092	OtA5093-OtA5097
Lce1m	chr3:93017806-93019060	OtA5088-OtA5092	OtA5093-OtA5097
Lce3a	chr3:92925286-92926230	OtA5088-OtA5092	OtA5093-OtA5097
Lce3b	chr3:92932978-92934096	OtA5088-OtA5092	OtA5093-OtA5097
Lce3c	chr3:92944485-92945730	OtA5088-OtA5092	OtA5093-OtA5097
Lce3f	chr3:92992225-92993426	OtA5088-OtA5092	OtA5093-OtA5097
Lce6a	chr3:92620084-92621660	OtA5088-OtA5092	OtA5093-OtA5097
Lck	chr4:129548343-129573641	OtA5088-OtA5092	OtA5093-OtA5097
Lcn10	chr2:25682725-25686081	OtA5088-OtA5092	OtA5093-OtA5097
Lcn11	chr2:25777016-25780279	OtA5088-OtA5092	OtA5093-OtA5097
Lcn12	chr2:25490844-25493911	OtA5088-OtA5092	OtA5093-OtA5097
Lcn13	chr2:25700073-25703326	OtA5088-OtA5092	OtA5093-OtA5097
Lcn14	chr2:25737008-25740097	OtA5088-OtA5092	OtA5093-OtA5097
Lcn3	chr2:25765568-25768099	OtA5088-OtA5092	OtA5093-OtA5097
Lcn4	chr2:26667673-26671282	OtA5088-OtA5092	OtA5093-OtA5097
Lcn5	chr2:25657954-25662230	OtA5088-OtA5092	OtA5093-OtA5097
Lcn6	chr2:25676785-25681607	OtA5088-OtA5092	OtA5093-OtA5097
Lcn8	chr2:25653117-25656216	OtA5088-OtA5092	OtA5093-OtA5097

Lcn9	chr2:25823152-25825537	OtA5088-OtA5092	OtA5093-OtA5097
Lcorl	chr5:45669924-45857540	OtA5088-OtA5092	OtA5093-OtA5097
Lcp2	chr11:34047200-34092280	OtA5088-OtA5092	OtA5093-OtA5097
Lct	chr1:128284763-128328318	OtA5088-OtA5092	OtA5093-OtA5097
Lctl	chr9:64117146-64172931	OtA5088-OtA5092	OtA5093-OtA5097
Ldb3	chr14:34526698-34588681	OtA5088-OtA5092	OtA5093-OtA5097
Ldhal6b	chr17:5410863-5440260	OtA5088-OtA5092	OtA5093-OtA5097
Ldhc	chr7:46861262-46878142	OtA5088-OtA5092	OtA5093-OtA5097
Ldlrad1	chr4:107209179-107217914	OtA5088-OtA5092	OtA5093-OtA5097
Ldlrad2	chr4:137570875-137575180	OtA5088-OtA5092	OtA5093-OtA5097
Leap2	chr11:53422180-53423136	OtA5088-OtA5092	OtA5093-OtA5097
Lect2	chr13:56542459-56548538	OtA5088-OtA5092	OtA5093-OtA5097
Lef1	chr3:131110470-131223331	OtA5088-OtA5092	OtA5093-OtA5097
Lefty1	chr1:180935038-180938401	OtA5088-OtA5092	OtA5093-OtA5097
Lefty2	chr1:180893118-180899102	OtA5088-OtA5092	OtA5093-OtA5097
Lekr1	chr3:65666227-65831162	OtA5088-OtA5092	OtA5093-OtA5097
Lelp1	chr3:92134993-92142754	OtA5088-OtA5092	OtA5093-OtA5097
Lemd1	chr1:132191435-132257382	OtA5088-OtA5092	OtA5093-OtA5097
Lep	chr6:29060220-29073876	OtA5088-OtA5092	OtA5093-OtA5097
Lgals12	chr19:7596660-7607176	OtA5088-OtA5092	OtA5093-OtA5097
Lgals6	chr7:28833793-28841703	OtA5088-OtA5092	OtA5093-OtA5097
Lgals7	chr7:28864385-28866284	OtA5088-OtA5092	OtA5093-OtA5097
Lgi1	chr19:38264781-38308939	OtA5088-OtA5092	OtA5093-OtA5097
Lgi2	chr5:52537863-52566280	OtA5088-OtA5092	OtA5093-OtA5097
Lgi3	chr14:70530820-70538324	OtA5088-OtA5092	OtA5093-OtA5097
Lgi4	chr7:31059934-31076697	OtA5088-OtA5092	OtA5093-OtA5097
Lgr5	chr10:115450313-115587780	OtA5088-OtA5092	OtA5093-OtA5097
Lgr6	chr1:134986352-135105276	OtA5088-OtA5092	OtA5093-OtA5097
Lgsn	chr1:31141077-31222656	OtA5088-OtA5092	OtA5093-OtA5097
Lhb	chr7:45420945-45421854	OtA5088-OtA5092	OtA5093-OtA5097
Lhcgr	chr17:88741548-88791976	OtA5088-OtA5092	OtA5093-OtA5097
Lhfpl1	chrX:145290358-145348894	OtA5088-OtA5092	OtA5093-OtA5097
Lhfpl3	chr5:22738910-23275597	OtA5088-OtA5092	OtA5093-OtA5097
Lhfpl4	chr6:113168086-113195384	OtA5088-OtA5092	OtA5093-OtA5097
Lhfpl5	chr17:28575718-28583593	OtA5088-OtA5092	OtA5093-OtA5097
Lhx1	chr11:84519378-84525534	OtA5088-OtA5092	OtA5093-OtA5097
Lhx3	chr2:26200211-26206556	OtA5088-OtA5092	OtA5093-OtA5097
Lhx4	chr1:155701693-155742027	OtA5088-OtA5092	OtA5093-OtA5097
Lhx5	chr5:120431885-120441457	OtA5088-OtA5092	OtA5093-OtA5097
Lhx8	chr3:154306293-154330560	OtA5088-OtA5092	OtA5093-OtA5097
Lhx9	chr1:138825185-138847579	OtA5088-OtA5092	OtA5093-OtA5097
Lif	chr11:4257567-4272514	OtA5088-OtA5092	OtA5093-OtA5097
Lig4	chr8:9970019-9976323	OtA5088-OtA5092	OtA5093-OtA5097
Lilra5	chr7:4237753-4243463	OtA5088-OtA5092	OtA5093-OtA5097
Lilra6	chr7:3836809-3915501	OtA5088-OtA5092	OtA5093-OtA5097
Lilrb3	chr7:3712504-3720382	OtA5088-OtA5092	OtA5093-OtA5097
Lim2	chr7:43430100-43435991	OtA5088-OtA5092	OtA5093-OtA5097

Lin28a	chr4:134003329-134018816	OtA5088-OtA5092	OtA5093-OtA5097
Lin28b	chr10:45376618-45470201	OtA5088-OtA5092	OtA5093-OtA5097
Lin7a	chr10:107271830-107425143	OtA5088-OtA5092	OtA5093-OtA5097
Lin7b	chr7:45339125-45370564	OtA5088-OtA5092	OtA5093-OtA5097
Lingo1	chr9:56618474-56685253	OtA5088-OtA5092	OtA5093-OtA5097
Lingo2	chr4:35706647-36951744	OtA5088-OtA5092	OtA5093-OtA5097
Lingo3	chr10:80832800-80844039	OtA5088-OtA5092	OtA5093-OtA5097
Lingo4	chr3:94399218-94404501	OtA5088-OtA5092	OtA5093-OtA5097
Lipc	chr9:70798127-70934808	OtA5088-OtA5092	OtA5093-OtA5097
Lipf	chr19:33961247-33976813	OtA5088-OtA5092	OtA5093-OtA5097
Lipi	chr16:75540513-75586061	OtA5088-OtA5092	OtA5093-OtA5097
Lipk	chr19:34008253-34047903	OtA5088-OtA5092	OtA5093-OtA5097
Lipm	chr19:34100942-34166041	OtA5088-OtA5092	OtA5093-OtA5097
Lipn	chr19:34067357-34084918	OtA5088-OtA5092	OtA5093-OtA5097
Lipo1	chr19:33555280-33590311	OtA5088-OtA5092	OtA5093-OtA5097
Lix1	chr17:17402685-17459388	OtA5088-OtA5092	OtA5093-OtA5097
Lman1l	chr9:57607032-57620774	OtA5088-OtA5092	OtA5093-OtA5097
Lmo1	chr7:109138571-109170308	OtA5088-OtA5092	OtA5093-OtA5097
Lmo3	chr6:138364480-138581968	OtA5088-OtA5092	OtA5093-OtA5097
Lmod2	chr6:24597770-24605414	OtA5088-OtA5092	OtA5093-OtA5097
Lmod3	chr6:97238527-97252780	OtA5088-OtA5092	OtA5093-OtA5097
Lmtk3	chr7:45783946-45804130	OtA5088-OtA5092	OtA5093-OtA5097
Lmx1a	chr1:167689557-167848733	OtA5088-OtA5092	OtA5093-OtA5097
Lmx1b	chr2:33564535-33640511	OtA5088-OtA5092	OtA5093-OtA5097
Lonrf2	chr1:38794508-38821215	OtA5088-OtA5092	OtA5093-OtA5097
Lor	chr3:92080270-92083142	OtA5088-OtA5092	OtA5093-OtA5097
Loxhd1	chr18:77281957-77442257	OtA5088-OtA5092	OtA5093-OtA5097
Lpar4	chrX:106920624-106933899	OtA5088-OtA5092	OtA5093-OtA5097
Lpcat2b	chr5:107431548-107435039	OtA5088-OtA5092	OtA5093-OtA5097
Lphn3	chr5:81021592-81795730	OtA5088-OtA5092	OtA5093-OtA5097
Lpo	chr11:87806427-87826114	OtA5088-OtA5092	OtA5093-OtA5097
Lpxn	chr19:12798608-12833808	OtA5088-OtA5092	OtA5093-OtA5097
Lrat	chr3:82876704-82943638	OtA5088-OtA5092	OtA5093-OtA5097
Lrch2	chrX:147471693-147554081	OtA5088-OtA5092	OtA5093-OtA5097
Lrdd	chr7:141438514-141443355	OtA5088-OtA5092	OtA5093-OtA5097
Lrfn2	chr17:48932581-49097590	OtA5088-OtA5092	OtA5093-OtA5097
Lrfn5	chr12:61524631-61845258	OtA5088-OtA5092	OtA5093-OtA5097
Lrguk	chr6:34029447-34134034	OtA5088-OtA5092	OtA5093-OtA5097
Lrit1	chr14:37054829-37064938	OtA5088-OtA5092	OtA5093-OtA5097
Lrit2	chr14:37068048-37073735	OtA5088-OtA5092	OtA5093-OtA5097
Lrit3	chr3:129788290-129804030	OtA5088-OtA5092	OtA5093-OtA5097
Lrmp	chr6:145121738-145210970	OtA5088-OtA5092	OtA5093-OtA5097
Lrp1b	chr2:40596772-42653598	OtA5088-OtA5092	OtA5093-OtA5097
Lrp2bp	chr8:46010601-46029476	OtA5088-OtA5092	OtA5093-OtA5097
Lrr1	chr12:69168813-69179010	OtA5088-OtA5092	OtA5093-OtA5097
Lrrc10	chr10:117045340-117046768	OtA5088-OtA5092	OtA5093-OtA5097
Lrrc10b	chr19:10455370-10457447	OtA5088-OtA5092	OtA5093-OtA5097

Lrrc14b	chr13:74350316-74365783	OtA5088-OtA5092	OtA5093-OtA5097
Lrrc15	chr16:30269301-30283254	OtA5088-OtA5092	OtA5093-OtA5097
Lrrc16b	chr14:55491092-55508264	OtA5088-OtA5092	OtA5093-OtA5097
Lrrc18	chr14:32959546-33185066	OtA5088-OtA5092	OtA5093-OtA5097
Lrrc19	chr4:94614519-94693233	OtA5088-OtA5092	OtA5093-OtA5097
Lrrc2	chr9:110951544-110984064	OtA5088-OtA5092	OtA5093-OtA5097
Lrrc23	chr6:124769862-124779718	OtA5088-OtA5092	OtA5093-OtA5097
Lrrc24	chr15:76715275-76723845	OtA5088-OtA5092	OtA5093-OtA5097
Lrrc26	chr2:25289910-25319163	OtA5088-OtA5092	OtA5093-OtA5097
Lrrc27	chr7:139103637-139242973	OtA5088-OtA5092	OtA5093-OtA5097
Lrrc3	chr10:77897575-77902536	OtA5088-OtA5092	OtA5093-OtA5097
Lrrc30	chr17:67630964-67632723	OtA5088-OtA5092	OtA5093-OtA5097
Lrrc34	chr3:30624266-30672431	OtA5088-OtA5092	OtA5093-OtA5097
Lrrc36	chr8:105413621-105464086	OtA5088-OtA5092	OtA5093-OtA5097
Lrrc38	chr4:143349749-143371028	OtA5088-OtA5092	OtA5093-OtA5097
Lrrc39	chr3:116562972-116630984	OtA5088-OtA5092	OtA5093-OtA5097
Lrrc3b	chr14:15357515-15438987	OtA5088-OtA5092	OtA5093-OtA5097
Lrrc4	chr6:28480347-28888832	OtA5088-OtA5092	OtA5093-OtA5097
Lrrc43	chr5:123489324-123508204	OtA5088-OtA5092	OtA5093-OtA5097
Lrrc46	chr11:97034601-97041369	OtA5088-OtA5092	OtA5093-OtA5097
Lrrc48	chr11:60353379-60394333	OtA5088-OtA5092	OtA5093-OtA5097
Lrrc49	chr9:60587234-60688134	OtA5088-OtA5092	OtA5093-OtA5097
Lrrc4b	chr7:44442486-44463344	OtA5088-OtA5092	OtA5093-OtA5097
Lrrc4c	chr2:97467673-97631664	OtA5088-OtA5092	OtA5093-OtA5097
Lrrc50	chr8:119575234-119605240	OtA5088-OtA5092	OtA5093-OtA5097
Lrrc52	chr1:167445674-167466780	OtA5088-OtA5092	OtA5093-OtA5097
Lrrc55	chr2:85188070-85196699	OtA5088-OtA5092	OtA5093-OtA5097
Lrrc6	chr15:66379857-66500910	OtA5088-OtA5092	OtA5093-OtA5097
Lrrc63	chr14:75004109-75388146	OtA5088-OtA5092	OtA5093-OtA5097
Lrrc66	chr5:73606641-73632421	OtA5088-OtA5092	OtA5093-OtA5097
Lrrc69	chr4:14665753-14796052	OtA5088-OtA5092	OtA5093-OtA5097
Lrrc7	chr3:158084438-158562221	OtA5088-OtA5092	OtA5093-OtA5097
Lrrc8e	chr8:4226826-4237470	OtA5088-OtA5092	OtA5093-OtA5097
Lrrc9	chr12:72441865-72510565	OtA5088-OtA5092	OtA5093-OtA5097
Lrrcc1	chr3:14533787-14572658	OtA5088-OtA5092	OtA5093-OtA5097
Lrriq1	chr10:103063197-103236322	OtA5088-OtA5092	OtA5093-OtA5097
Lrriq3	chr3:155093433-155194278	OtA5088-OtA5092	OtA5093-OtA5097
Lrriq4	chr3:30624266-30672431	OtA5088-OtA5092	OtA5093-OtA5097
Lrrk2	chr15:91673223-91816124	OtA5088-OtA5092	OtA5093-OtA5097
Lrrn1	chr6:107529725-107570228	OtA5088-OtA5092	OtA5093-OtA5097
Lrrn3	chr12:41451667-41485751	OtA5088-OtA5092	OtA5093-OtA5097
Lrrn4cl	chr19:8850784-8853909	OtA5088-OtA5092	OtA5093-OtA5097
Lrrtm1	chr6:76881636-77979667	OtA5088-OtA5092	OtA5093-OtA5097
Lrrtm2	chr18:35118911-35254775	OtA5088-OtA5092	OtA5093-OtA5097
Lrrtm3	chr10:63430097-65003667	OtA5088-OtA5092	OtA5093-OtA5097
Lrrtm4	chr6:80018876-80024932	OtA5088-OtA5092	OtA5093-OtA5097
Lrtm1	chr14:28904942-29721864	OtA5088-OtA5092	OtA5093-OtA5097

Lrtm2	chr6:119236525-119352407	OtA5088-OtA5092	OtA5093-OtA5097
Lsamp	chr16:41533341-42146213	OtA5088-OtA5092	OtA5093-OtA5097
Lta	chr17:35203164-35205351	OtA5088-OtA5092	OtA5093-OtA5097
Ltb	chr17:35194506-35196305	OtA5088-OtA5092	OtA5093-OtA5097
Ltb4r1	chr14:55765961-55768492	OtA5088-OtA5092	OtA5093-OtA5097
Ltb4r2	chr14:55761427-55763229	OtA5088-OtA5092	OtA5093-OtA5097
Luzp2	chr7:54835244-55268888	OtA5088-OtA5092	OtA5093-OtA5097
Luzp4	chrX:148882575-148924139	OtA5088-OtA5092	OtA5093-OtA5097
Ly6d	chr15:74762055-74763567	OtA5088-OtA5092	OtA5093-OtA5097
Ly6g5b	chr17:35113945-35115400	OtA5088-OtA5092	OtA5093-OtA5097
Ly6g5c	chr17:35108299-35111953	OtA5088-OtA5092	OtA5093-OtA5097
Ly6g6d	chr17:35071347-35074464	OtA5088-OtA5092	OtA5093-OtA5097
Ly6g6e	chr17:35076941-35078804	OtA5088-OtA5092	OtA5093-OtA5097
Ly6g6f	chr17:35080537-35085595	OtA5088-OtA5092	OtA5093-OtA5097
Ly6h	chr15:75564744-75567182	OtA5088-OtA5092	OtA5093-OtA5097
Ly6i	chr15:74979811-74983430	OtA5088-OtA5092	OtA5093-OtA5097
Ly9	chr1:171588612-171607360	OtA5088-OtA5092	OtA5093-OtA5097
Lyg1	chr1:37946737-37957759	OtA5088-OtA5092	OtA5093-OtA5097
Lyg2	chr1:37905922-37916493	OtA5088-OtA5092	OtA5093-OtA5097
Lyl1	chr8:84701456-84704716	OtA5088-OtA5092	OtA5093-OtA5097
Lypd1	chr1:125676995-125912214	OtA5088-OtA5092	OtA5093-OtA5097
Lypd2	chr15:74732251-74734313	OtA5088-OtA5092	OtA5093-OtA5097
Lypd3	chr7:24636569-24641118	OtA5088-OtA5092	OtA5093-OtA5097
Lypd4	chr7:24864619-24869691	OtA5088-OtA5092	OtA5093-OtA5097
Lypd5	chr7:24349223-24353833	OtA5088-OtA5092	OtA5093-OtA5097
Lypd6	chr2:50066428-50193569	OtA5088-OtA5092	OtA5093-OtA5097
Lypd6b	chr2:49787685-49948846	OtA5088-OtA5092	OtA5093-OtA5097
Lyplal1	chr1:186087731-186117310	OtA5088-OtA5092	OtA5093-OtA5097
Lyrm1	chr7:119896291-119916750	OtA5088-OtA5092	OtA5093-OtA5097
Lyrm7	chr11:54839288-54860591	OtA5088-OtA5092	OtA5093-OtA5097
Lysmd1	chr3:95134087-95142360	OtA5088-OtA5092	OtA5093-OtA5097
Lyz1	chr10:117287794-117292868	OtA5088-OtA5092	OtA5093-OtA5097
Lyzl1	chr18:4165831-4182236	OtA5088-OtA5092	OtA5093-OtA5097
Lyzl4	chr9:121577842-121641566	OtA5088-OtA5092	OtA5093-OtA5097
Lyzl6	chr11:103631074-103638874	OtA5088-OtA5092	OtA5093-OtA5097
MGC107098	chrY:73535407-73537718	OtA5088-OtA5092	OtA5093-OtA5097
Mab21l1	chr3:55625197-56183701	OtA5088-OtA5092	OtA5093-OtA5097
Mab21l2	chr3:86224689-86782694	OtA5088-OtA5092	OtA5093-OtA5097
Macc1	chr12:119443409-119466932	OtA5088-OtA5092	OtA5093-OtA5097
Macrod2	chr2:140395429-142390050	OtA5088-OtA5092	OtA5093-OtA5097
Mad2l1	chr6:66535467-66540991	OtA5088-OtA5092	OtA5093-OtA5097
Madcam1	chr10:79664573-79668536	OtA5088-OtA5092	OtA5093-OtA5097
Mael	chr1:166201384-166238744	OtA5088-OtA5092	OtA5093-OtA5097
Mafa	chr15:75746842-75747922	OtA5088-OtA5092	OtA5093-OtA5097
Mag	chr7:30899182-30914832	OtA5088-OtA5092	OtA5093-OtA5097
Magea1	chrX:155088518-155089790	OtA5088-OtA5092	OtA5093-OtA5097
Magea10	chrX:72381869-72386858	OtA5088-OtA5092	OtA5093-OtA5097

Magea2	chrX:155027200-155033292	OtA5088-OtA5092	OtA5093-OtA5097
Magea3	chrX:154948462-154949569	OtA5088-OtA5092	OtA5093-OtA5097
Magea4	chrX:72222016-72222964	OtA5088-OtA5092	OtA5093-OtA5097
Magea5	chrX:155053060-155061553	OtA5088-OtA5092	OtA5093-OtA5097
Magea6	chrX:154924011-154926115	OtA5088-OtA5092	OtA5093-OtA5097
Magea8	chrX:154985774-154995850	OtA5088-OtA5092	OtA5093-OtA5097
Mageb1	chrX:92015190-92016333	OtA5088-OtA5092	OtA5093-OtA5097
Mageb16	chrX:79623230-79671435	OtA5088-OtA5092	OtA5093-OtA5097
Mageb16-ps1	chrX:144381670-144688180	OtA5088-OtA5092	OtA5093-OtA5097
Mageb18	chrX:92118878-92599572	OtA5088-OtA5092	OtA5093-OtA5097
Mageb2	chrX:92015190-92016333	OtA5088-OtA5092	OtA5093-OtA5097
Mageb3	chr2:121953770-121956092	OtA5088-OtA5092	OtA5093-OtA5097
Mageb4	chrX:86250255-86256219	OtA5088-OtA5092	OtA5093-OtA5097
Mageb5	chrX:91779607-91782976	OtA5088-OtA5092	OtA5093-OtA5097
Magee2	chrX:104854951-104857267	OtA5088-OtA5092	OtA5093-OtA5097
Magi2	chr5:19227045-20704792	OtA5088-OtA5092	OtA5093-OtA5097
Magix	chrX:7673165-7681251	OtA5088-OtA5092	OtA5093-OtA5097
Mak	chr13:41025119-41079706	OtA5088-OtA5092	OtA5093-OtA5097
Mal2	chr15:54571365-54602846	OtA5088-OtA5092	OtA5093-OtA5097
Mamdc4	chr2:25563114-25571316	OtA5088-OtA5092	OtA5093-OtA5097
Mamld1	chrX:71050255-71154717	OtA5088-OtA5092	OtA5093-OtA5097
Mamstr	chr7:45639976-45645768	OtA5088-OtA5092	OtA5093-OtA5097
Maneal	chr4:124850758-124862171	OtA5088-OtA5092	OtA5093-OtA5097
Map3k13	chr16:21891968-21931877	OtA5088-OtA5092	OtA5093-OtA5097
Map3k14	chr11:103219763-103267401	OtA5088-OtA5092	OtA5093-OtA5097
Map3k15	chrX:159988432-160138336	OtA5088-OtA5092	OtA5093-OtA5097
Map4k1	chr7:28982853-29003278	OtA5088-OtA5092	OtA5093-OtA5097
Map6d1	chr16:20233308-20241358	OtA5088-OtA5092	OtA5093-OtA5097
Mapk10	chr5:102907947-103211334	OtA5088-OtA5092	OtA5093-OtA5097
Mapk12	chr15:89130583-89140703	OtA5088-OtA5092	OtA5093-OtA5097
Mapk15	chr15:75993768-75999153	OtA5088-OtA5092	OtA5093-OtA5097
Mapk4	chr18:73928485-74064949	OtA5088-OtA5092	OtA5093-OtA5097
Mapk8ip2	chr15:89453910-89462447	OtA5088-OtA5092	OtA5093-OtA5097
Mapt	chr11:104231435-104332093	OtA5088-OtA5092	OtA5093-OtA5097
1-Mar	chr8:65618039-66471637	OtA5088-OtA5092	OtA5093-OtA5097
10-Mar	chr11:105360797-105456735	OtA5088-OtA5092	OtA5093-OtA5097
11-Mar	chr15:26309071-26409573	OtA5088-OtA5092	OtA5093-OtA5097
4-Mar	chr1:72427111-72536564	OtA5088-OtA5092	OtA5093-OtA5097
Marcksl1-ps4	chr13:4248734-4249777	OtA5088-OtA5092	OtA5093-OtA5097
Marco	chr1:120474537-120505084	OtA5088-OtA5092	OtA5093-OtA5097
Mas1	chr17:12682405-12868143	OtA5088-OtA5092	OtA5093-OtA5097
Masp2	chr4:148602543-148626996	OtA5088-OtA5092	OtA5093-OtA5097
Mast1	chr8:84911852-84937353	OtA5088-OtA5092	OtA5093-OtA5097
Mastl	chr2:23116543-23156024	OtA5088-OtA5092	OtA5093-OtA5097
Mat1a	chr14:41105380-41124412	OtA5088-OtA5092	OtA5093-OtA5097
Matn1	chr4:130944384-130955476	OtA5088-OtA5092	OtA5093-OtA5097
Matn3	chr12:8947928-8972028	OtA5088-OtA5092	OtA5093-OtA5097

Matn4	chr2:164389392-164415448	OtA5088-OtA5092	OtA5093-OtA5097
Mb	chr15:77015486-77057108	OtA5088-OtA5092	OtA5093-OtA5097
Mbd3l1	chr9:18478394-18485292	OtA5088-OtA5092	OtA5093-OtA5097
Mbd3l2	chr9:18430614-18446316	OtA5088-OtA5092	OtA5093-OtA5097
Mbd4	chr6:115840696-115853341	OtA5088-OtA5092	OtA5093-OtA5097
Mbl1	chr14:41151455-41158959	OtA5088-OtA5092	OtA5093-OtA5097
Mbl2	chr19:30232956-30239682	OtA5088-OtA5092	OtA5093-OtA5097
Mblac2	chr13:81711416-81753275	OtA5088-OtA5092	OtA5093-OtA5097
Mboat4	chr8:34115029-34125185	OtA5088-OtA5092	OtA5093-OtA5097
Mc1r	chr8:123407081-123410744	OtA5088-OtA5092	OtA5093-OtA5097
Mc2r	chr18:68406906-68429259	OtA5088-OtA5092	OtA5093-OtA5097
Mc3r	chr2:172248491-172251114	OtA5088-OtA5092	OtA5093-OtA5097
Mc4r	chr18:66857707-66860472	OtA5088-OtA5092	OtA5093-OtA5097
Mc5r	chr18:68337602-68339711	OtA5088-OtA5092	OtA5093-OtA5097
Mcart6	chrX:136981115-137038302	OtA5088-OtA5092	OtA5093-OtA5097
Mcf2	chrX:60055955-60147643	OtA5088-OtA5092	OtA5093-OtA5097
Mchr1	chr15:81235498-81238964	OtA5088-OtA5092	OtA5093-OtA5097
Mcm10	chr2:4990723-5012791	OtA5088-OtA5092	OtA5093-OtA5097
Mcm8	chr2:132816334-132844188	OtA5088-OtA5092	OtA5093-OtA5097
Mcpt-ps1	chr14:55949808-55952371	OtA5088-OtA5092	OtA5093-OtA5097
Mcpt1	chr14:56017963-56020391	OtA5088-OtA5092	OtA5093-OtA5097
Mcpt2	chr14:56042122-56044634	OtA5088-OtA5092	OtA5093-OtA5097
Mcpt4	chr14:56059743-56062310	OtA5088-OtA5092	OtA5093-OtA5097
Mcpt8	chr14:56082163-56085197	OtA5088-OtA5092	OtA5093-OtA5097
Mcpt9	chr14:56026863-56030495	OtA5088-OtA5092	OtA5093-OtA5097
Mctp1	chr13:76384960-77031810	OtA5088-OtA5092	OtA5093-OtA5097
Mdga1	chr17:29827957-29887882	OtA5088-OtA5092	OtA5093-OtA5097
Mdga2	chr12:66466058-67222549	OtA5088-OtA5092	OtA5093-OtA5097
Mdh1b	chr1:63698826-63730318	OtA5088-OtA5092	OtA5093-OtA5097
Me3	chr7:89632817-89854359	OtA5088-OtA5092	OtA5093-OtA5097
Mecom	chr3:30236568-30509487	OtA5088-OtA5092	OtA5093-OtA5097
Mef2b	chr8:70152777-70167488	OtA5088-OtA5092	OtA5093-OtA5097
Mef2c	chr13:83504033-83667079	OtA5088-OtA5092	OtA5093-OtA5097
Mefv	chr16:3707214-3718124	OtA5088-OtA5092	OtA5093-OtA5097
Megf10	chr18:57133089-57297467	OtA5088-OtA5092	OtA5093-OtA5097
Mei1	chr15:82070116-82126814	OtA5088-OtA5092	OtA5093-OtA5097
Mei4	chr9:81863743-82027491	OtA5088-OtA5092	OtA5093-OtA5097
Meig1	chr2:3409042-3422648	OtA5088-OtA5092	OtA5093-OtA5097
Melk	chr4:44300916-44364675	OtA5088-OtA5092	OtA5093-OtA5097
Meox1	chr11:101877509-101894354	OtA5088-OtA5092	OtA5093-OtA5097
Meox2	chr12:37108545-37179528	OtA5088-OtA5092	OtA5093-OtA5097
Mep1a	chr17:43474328-43502773	OtA5088-OtA5092	OtA5093-OtA5097
Mep1b	chr18:21072343-21100199	OtA5088-OtA5092	OtA5093-OtA5097
Mepe	chr5:104325328-104338611	OtA5088-OtA5092	OtA5093-OtA5097
Mesp1	chr7:79792240-79793590	OtA5088-OtA5092	OtA5093-OtA5097
Mesp2	chr7:79810726-79813431	OtA5088-OtA5092	OtA5093-OtA5097
Mettl11b	chr1:163702992-163725232	OtA5088-OtA5092	OtA5093-OtA5097

Mettl7a2	chr15:100353199-100384435	OtA5088-OtA5092	OtA5093-OtA5097
Mettl7a3	chr15:100334928-100340169	OtA5088-OtA5092	OtA5093-OtA5097
Mex3a	chr3:88532394-88541394	OtA5088-OtA5092	OtA5093-OtA5097
Mfap3l	chr8:60632859-60676731	OtA5088-OtA5092	OtA5093-OtA5097
Mfsd2b	chr12:4862437-4874359	OtA5088-OtA5092	OtA5093-OtA5097
Mfsd4	chr1:132026620-132068062	OtA5088-OtA5092	OtA5093-OtA5097
Mfsd6l	chr11:68556185-68558245	OtA5088-OtA5092	OtA5093-OtA5097
Mgam	chr6:40529090-40910666	OtA5088-OtA5092	OtA5093-OtA5097
Mgat4c	chr10:101681486-102391468	OtA5088-OtA5092	OtA5093-OtA5097
Mgat5b	chr11:116918862-116986944	OtA5088-OtA5092	OtA5093-OtA5097
Mgl2	chr11:70130356-70137542	OtA5088-OtA5092	OtA5093-OtA5097
Mia1	chr7:27179740-27181149	OtA5088-OtA5092	OtA5093-OtA5097
Mia2	chr12:59095799-59109130	OtA5088-OtA5092	OtA5093-OtA5097
Miat	chr5:112213227-112228948	OtA5088-OtA5092	OtA5093-OtA5097
Micalcl	chr7:112368307-112413104	OtA5088-OtA5092	OtA5093-OtA5097
Mid1	chrX:169685246-169990797	OtA5088-OtA5092	OtA5093-OtA5097
Mill1	chr7:18245346-18266092	OtA5088-OtA5092	OtA5093-OtA5097
Miox	chr15:89334472-89337007	OtA5088-OtA5092	OtA5093-OtA5097
Mip	chr10:128225837-128231811	OtA5088-OtA5092	OtA5093-OtA5097
Mipol1	chr12:57230411-57457241	OtA5088-OtA5092	OtA5093-OtA5097
Mira	chr6:52214491-52215288	OtA5088-OtA5092	OtA5093-OtA5097
Mirlet7a-1	chr13:48538178-48538272	OtA5088-OtA5092	OtA5093-OtA5097
Mirlet7a-2	chr9:41536715-41536811	OtA5088-OtA5092	OtA5093-OtA5097
Mirlet7b	chr15:85707318-85707403	OtA5088-OtA5092	OtA5093-OtA5097
Mirlet7c-1	chr16:77599656-77599750	OtA5088-OtA5092	OtA5093-OtA5097
Mirlet7c-2	chr15:85706602-85706697	OtA5088-OtA5092	OtA5093-OtA5097
Mirlet7d	chr13:48536011-48536114	OtA5088-OtA5092	OtA5093-OtA5097
Mirlet7e	chr17:17830351-17830444	OtA5088-OtA5092	OtA5093-OtA5097
Mirlet7f-1	chr13:48537828-48537917	OtA5088-OtA5092	OtA5093-OtA5097
Mirlet7f-2	chrX:151803281-151935417	OtA5088-OtA5092	OtA5093-OtA5097
Mirlet7g	chr9:106170928-106191706	OtA5088-OtA5092	OtA5093-OtA5097
Mirlet7i	chr10:122985639-122985724	OtA5088-OtA5092	OtA5093-OtA5097
Mis18bp1	chr12:65132734-65172580	OtA5088-OtA5092	OtA5093-OtA5097
Mixl1	chr1:180693047-180697034	OtA5088-OtA5092	OtA5093-OtA5097
Mki67	chr7:135689787-135716379	OtA5088-OtA5092	OtA5093-OtA5097
Mkks	chr2:136873780-137069778	OtA5088-OtA5092	OtA5093-OtA5097
Mkrn3	chr7:62417592-62420139	OtA5088-OtA5092	OtA5093-OtA5097
Mkx	chr18:6934965-7004779	OtA5088-OtA5092	OtA5093-OtA5097
Mlana	chr19:29697940-29708306	OtA5088-OtA5092	OtA5093-OtA5097
Mlc1	chr15:88955883-88978553	OtA5088-OtA5092	OtA5093-OtA5097
Mlf1	chr3:67374096-67400000	OtA5088-OtA5092	OtA5093-OtA5097
Mlf1ip	chr8:46552068-46617200	OtA5088-OtA5092	OtA5093-OtA5097
Mlxipl	chr5:135106890-135138382	OtA5088-OtA5092	OtA5093-OtA5097
Mmd2	chr5:142563480-142608752	OtA5088-OtA5092	OtA5093-OtA5097
Mmel1	chr4:154869584-154895530	OtA5088-OtA5092	OtA5093-OtA5097
Mmp13	chr9:7272513-7283333	OtA5088-OtA5092	OtA5093-OtA5097
Mmp16	chr4:17853481-18118734	OtA5088-OtA5092	OtA5093-OtA5097

Mmp1a	chr9:7464140-7476869	OtA5088-OtA5092	OtA5093-OtA5097
Mmp1b	chr9:7367669-7388026	OtA5088-OtA5092	OtA5093-OtA5097
Mmp20	chr9:7628230-7674968	OtA5088-OtA5092	OtA5093-OtA5097
Mmp21	chr7:133674269-133680061	OtA5088-OtA5092	OtA5093-OtA5097
Mmp24	chr2:155775343-155819203	OtA5088-OtA5092	OtA5093-OtA5097
Mmp25	chr17:23629457-23645269	OtA5088-OtA5092	OtA5093-OtA5097
Mmp27	chr9:7571457-7581393	OtA5088-OtA5092	OtA5093-OtA5097
Mmp3	chr9:7445821-7455974	OtA5088-OtA5092	OtA5093-OtA5097
Mmp8	chr9:7558428-7568486	OtA5088-OtA5092	OtA5093-OtA5097
Mmp9	chr2:164948250-164955846	OtA5088-OtA5092	OtA5093-OtA5097
Mmrn1	chr6:60944316-60989378	OtA5088-OtA5092	OtA5093-OtA5097
Mms22l	chr4:24496461-24602948	OtA5088-OtA5092	OtA5093-OtA5097
Mnd1	chr3:84087933-84155786	OtA5088-OtA5092	OtA5093-OtA5097
Mnda	chr1:173896354-173912922	OtA5088-OtA5092	OtA5093-OtA5097
Mndal	chr1:173857219-173880187	OtA5088-OtA5092	OtA5093-OtA5097
Mns1	chr9:72438528-72491959	OtA5088-OtA5092	OtA5093-OtA5097
Mnx1	chr5:29473822-29478470	OtA5088-OtA5092	OtA5093-OtA5097
Moap1	chr12:102739829-102757810	OtA5088-OtA5092	OtA5093-OtA5097
Mobp	chr9:120149741-120176091	OtA5088-OtA5092	OtA5093-OtA5097
Mog	chr17:37010739-37023398	OtA5088-OtA5092	OtA5093-OtA5097
Mogat1	chr1:78511059-78538173	OtA5088-OtA5092	OtA5093-OtA5097
Morc1	chr16:48431236-48630905	OtA5088-OtA5092	OtA5093-OtA5097
Morc2b	chr17:33135589-33139683	OtA5088-OtA5092	OtA5093-OtA5097
Morn1	chr4:155086576-155145507	OtA5088-OtA5092	OtA5093-OtA5097
Morn3	chr5:123037126-123046820	OtA5088-OtA5092	OtA5093-OtA5097
Morn4	chr19:42074938-42086370	OtA5088-OtA5092	OtA5093-OtA5097
Morn5	chr2:36049472-36079709	OtA5088-OtA5092	OtA5093-OtA5097
Mos	chr4:3870657-3872105	OtA5088-OtA5092	OtA5093-OtA5097
Mospd4	chr18:46464933-46465816	OtA5088-OtA5092	OtA5093-OtA5097
Mov10l1	chr15:88982993-89055152	OtA5088-OtA5092	OtA5093-OtA5097
Moxd1	chr10:24223516-24302783	OtA5088-OtA5092	OtA5093-OtA5097
Moxd2	chr6:40529090-40910666	OtA5088-OtA5092	OtA5093-OtA5097
Mphosph9	chr5:124250958-124341844	OtA5088-OtA5092	OtA5093-OtA5097
Mpl	chr4:118442416-118457495	OtA5088-OtA5092	OtA5093-OtA5097
Mpo	chr11:87793783-87804412	OtA5088-OtA5092	OtA5093-OtA5097
Mpp3	chr11:101999652-102026955	OtA5088-OtA5092	OtA5093-OtA5097
Mpp4	chr1:59120934-59237231	OtA5088-OtA5092	OtA5093-OtA5097
Mpped1	chr15:83780022-83858474	OtA5088-OtA5092	OtA5093-OtA5097
Mpv17l	chr16:13940694-13949357	OtA5088-OtA5092	OtA5093-OtA5097
Mpz	chr1:171150712-171161123	OtA5088-OtA5092	OtA5093-OtA5097
Mr1	chr1:155127877-155146780	OtA5088-OtA5092	OtA5093-OtA5097
Mrap2	chr9:87144305-87184045	OtA5088-OtA5092	OtA5093-OtA5097
Mrgpra1	chr7:47334874-47354240	OtA5088-OtA5092	OtA5093-OtA5097
Mrgpra2a	chr7:47426328-47452139	OtA5088-OtA5092	OtA5093-OtA5097
Mrgpra2b	chr7:47463806-47489582	OtA5088-OtA5092	OtA5093-OtA5097
Mrgpra3	chr7:47588949-47601372	OtA5088-OtA5092	OtA5093-OtA5097
Mrgpra4	chr7:47980793-47982296	OtA5088-OtA5092	OtA5093-OtA5097

Mrgpra6	chr7:47185765-47189355	OtA5088-OtA5092	OtA5093-OtA5097
Mrgpra9	chr7:47234918-47252848	OtA5088-OtA5092	OtA5093-OtA5097
Mrgprb1	chr7:48444112-48456342	OtA5088-OtA5092	OtA5093-OtA5097
Mrgprb2	chr7:48550965-48558086	OtA5088-OtA5092	OtA5093-OtA5097
Mrgprb3	chr7:48642862-48643801	OtA5088-OtA5092	OtA5093-OtA5097
Mrgprb4	chr7:48198212-48199178	OtA5088-OtA5092	OtA5093-OtA5097
Mrgprb5	chr7:48168016-48168985	OtA5088-OtA5092	OtA5093-OtA5097
Mrgprb8	chr7:48388525-48389648	OtA5088-OtA5092	OtA5093-OtA5097
Mrgprd	chr7:145314834-145324059	OtA5088-OtA5092	OtA5093-OtA5097
Mrgprh	chr17:12876033-12877842	OtA5088-OtA5092	OtA5093-OtA5097
Mrgprx1	chr7:48020970-48027597	OtA5088-OtA5092	OtA5093-OtA5097
Mrgprx2	chr7:48478618-48499270	OtA5088-OtA5092	OtA5093-OtA5097
Mro	chr18:73859386-73879134	OtA5088-OtA5092	OtA5093-OtA5097
Mrps25	chr6:92091417-92184023	OtA5088-OtA5092	OtA5093-OtA5097
Ms4a1	chr19:11196816-11299884	OtA5088-OtA5092	OtA5093-OtA5097
Ms4a13	chr19:11169417-11196723	OtA5088-OtA5092	OtA5093-OtA5097
Ms4a15	chr19:10978306-10993250	OtA5088-OtA5092	OtA5093-OtA5097
Ms4a18	chr19:10997024-11017963	OtA5088-OtA5092	OtA5093-OtA5097
Ms4a2	chr19:11617192-11623643	OtA5088-OtA5092	OtA5093-OtA5097
Ms4a3	chr19:11629496-11640834	OtA5088-OtA5092	OtA5093-OtA5097
Ms4a4b	chr19:11443557-11463548	OtA5088-OtA5092	OtA5093-OtA5097
Ms4a4c	chr19:11407660-11427246	OtA5088-OtA5092	OtA5093-OtA5097
Ms4a4d	chr19:11536848-11558466	OtA5088-OtA5092	OtA5093-OtA5097
Ms4a5	chr19:11196816-11299884	OtA5088-OtA5092	OtA5093-OtA5097
Ms4a6b	chr19:11518558-11530403	OtA5088-OtA5092	OtA5093-OtA5097
Ms4a8a	chr19:11067470-11081103	OtA5088-OtA5092	OtA5093-OtA5097
Msc	chr1:14753345-14755966	OtA5088-OtA5092	OtA5093-OtA5097
Msgn1	chr12:11208381-11208948	OtA5088-OtA5092	OtA5093-OtA5097
Msh3	chr13:92211880-92389053	OtA5088-OtA5092	OtA5093-OtA5097
Msh4	chr3:153857146-153906138	OtA5088-OtA5092	OtA5093-OtA5097
Msh5	chr17:35028604-35046723	OtA5088-OtA5092	OtA5093-OtA5097
Msi1	chr5:115429684-115454202	OtA5088-OtA5092	OtA5093-OtA5097
Mslnl	chr17:25736039-25748330	OtA5088-OtA5092	OtA5093-OtA5097
Msmb	chr14:32142022-32158326	OtA5088-OtA5092	OtA5093-OtA5097
Msmp	chr4:43562633-43587487	OtA5088-OtA5092	OtA5093-OtA5097
Msr1	chr8:39467447-40227787	OtA5088-OtA5092	OtA5093-OtA5097
Mstn	chr1:53061662-53068079	OtA5088-OtA5092	OtA5093-OtA5097
Msx1	chr5:37820490-37824585	OtA5088-OtA5092	OtA5093-OtA5097
Msx1as	chr5:37820490-37824585	OtA5088-OtA5092	OtA5093-OtA5097
Msx3	chr7:140046156-140049088	OtA5088-OtA5092	OtA5093-OtA5097
Mt3	chr8:94152606-94154148	OtA5088-OtA5092	OtA5093-OtA5097
Mt4	chr8:94137203-94139031	OtA5088-OtA5092	OtA5093-OtA5097
Mtag2	chr7:45339125-45370564	OtA5088-OtA5092	OtA5093-OtA5097
Mtap1a	chr2:121289601-121351013	OtA5088-OtA5092	OtA5093-OtA5097
Mtap2	chr1:66175328-66442583	OtA5088-OtA5092	OtA5093-OtA5097
Mtap7d2	chrX:159414577-159498947	OtA5088-OtA5092	OtA5093-OtA5097
Mtap7d3	chrX:56797952-56822325	OtA5088-OtA5092	OtA5093-OtA5097

Mtap9	chr3:82358071-82395268	OtA5088-OtA5092	OtA5093-OtA5097
Mtbp	chr15:55557407-55626423	OtA5088-OtA5092	OtA5093-OtA5097
Mterf	chr5:3890580-3893933	OtA5088-OtA5092	OtA5093-OtA5097
Mterfd3	chr10:85119432-85128027	OtA5088-OtA5092	OtA5093-OtA5097
Mthfd2l	chr5:90931195-91021370	OtA5088-OtA5092	OtA5093-OtA5097
Mtl5	chr19:3388868-3407785	OtA5088-OtA5092	OtA5093-OtA5097
Mtnr1a	chr8:45069209-45088506	OtA5088-OtA5092	OtA5093-OtA5097
Mtnr1b	chr9:15862613-15874556	OtA5088-OtA5092	OtA5093-OtA5097
Mtrf1	chr14:79397771-79423650	OtA5088-OtA5092	OtA5093-OtA5097
Mtrf1l	chr10:5811886-5823943	OtA5088-OtA5092	OtA5093-OtA5097
Mtus2	chr5:147957319-148316065	OtA5088-OtA5092	OtA5093-OtA5097
Muc15	chr2:110658139-110950244	OtA5088-OtA5092	OtA5093-OtA5097
Muc19	chr15:91838325-91934555	OtA5088-OtA5092	OtA5093-OtA5097
Muc20	chr16:32735885-32797435	OtA5088-OtA5092	OtA5093-OtA5097
Muc4	chr16:32735885-32797435	OtA5088-OtA5092	OtA5093-OtA5097
Muc5ac	chr7:141789010-141819135	OtA5088-OtA5092	OtA5093-OtA5097
Muc5b	chr7:141839071-141873085	OtA5088-OtA5092	OtA5093-OtA5097
Muc6	chr7:141634048-141655308	OtA5088-OtA5092	OtA5093-OtA5097
Mucl1	chr15:103751922-103899312	OtA5088-OtA5092	OtA5093-OtA5097
Mug-ps1	chr6:122176052-122248566	OtA5088-OtA5092	OtA5093-OtA5097
Mug1	chr6:121838540-121889057	OtA5088-OtA5092	OtA5093-OtA5097
Mug2	chr6:122006797-122085967	OtA5088-OtA5092	OtA5093-OtA5097
Mup1	chr4:60498012-60501960	OtA5088-OtA5092	OtA5093-OtA5097
Mup10	chr4:60578260-60662395	OtA5088-OtA5092	OtA5093-OtA5097
Mup11	chr4:60578260-60662395	OtA5088-OtA5092	OtA5093-OtA5097
Mup12	chr4:60737380-60741281	OtA5088-OtA5092	OtA5093-OtA5097
Mup13	chr4:61224306-61228286	OtA5088-OtA5092	OtA5093-OtA5097
Mup14	chr4:61300036-61439704	OtA5088-OtA5092	OtA5093-OtA5097
Mup15	chr4:61300036-61439704	OtA5088-OtA5092	OtA5093-OtA5097
Mup16	chr4:61515592-61519486	OtA5088-OtA5092	OtA5093-OtA5097
Mup17	chr4:61591929-61595832	OtA5088-OtA5092	OtA5093-OtA5097
Mup19	chr4:61778324-61782224	OtA5088-OtA5092	OtA5093-OtA5097
Mup2	chr4:61300036-61439704	OtA5088-OtA5092	OtA5093-OtA5097
Mup20	chr4:62050234-62054117	OtA5088-OtA5092	OtA5093-OtA5097
Mup21	chr4:62147831-62150841	OtA5088-OtA5092	OtA5093-OtA5097
Mup3	chr4:62083475-62087312	OtA5088-OtA5092	OtA5093-OtA5097
Mup4	chr4:59956805-59960665	OtA5088-OtA5092	OtA5093-OtA5097
Mup5	chr4:61831318-61835180	OtA5088-OtA5092	OtA5093-OtA5097
Mup6	chr4:60003548-60006214	OtA5088-OtA5092	OtA5093-OtA5097
Mup7	chr4:60066468-60139858	OtA5088-OtA5092	OtA5093-OtA5097
Mup8	chr4:60218620-60222599	OtA5088-OtA5092	OtA5093-OtA5097
Mup9	chr4:60419159-60421952	OtA5088-OtA5092	OtA5093-OtA5097
Murc	chr4:48663513-48673492	OtA5088-OtA5092	OtA5093-OtA5097
Mx1	chr16:97447034-97462906	OtA5088-OtA5092	OtA5093-OtA5097
Mx2	chr16:97536080-97560901	OtA5088-OtA5092	OtA5093-OtA5097
Mxd3	chr13:55322054-55329730	OtA5088-OtA5092	OtA5093-OtA5097
Myadml2	chr11:120646030-120648337	OtA5088-OtA5092	OtA5093-OtA5097

Myb	chr10:21124929-21160984	OtA5088-OtA5092	OtA5093-OtA5097
Mybl1	chr1:9668834-9700209	OtA5088-OtA5092	OtA5093-OtA5097
Mybpc1	chr10:88518278-88605152	OtA5088-OtA5092	OtA5093-OtA5097
Mybpc2	chr7:44501698-44524669	OtA5088-OtA5092	OtA5093-OtA5097
Mybpc3	chr2:91118143-91136516	OtA5088-OtA5092	OtA5093-OtA5097
Mybph	chr1:134193447-134235431	OtA5088-OtA5092	OtA5093-OtA5097
Mybphl	chr3:108364910-108383741	OtA5088-OtA5092	OtA5093-OtA5097
Mycs	chrX:5466903-5469265	OtA5088-OtA5092	OtA5093-OtA5097
Myct1	chr10:5593727-5606791	OtA5088-OtA5092	OtA5093-OtA5097
Myf5	chr10:107482907-107486134	OtA5088-OtA5092	OtA5093-OtA5097
Myf6	chr10:107492859-107494729	OtA5088-OtA5092	OtA5093-OtA5097
Myh1	chr11:67200113-67224575	OtA5088-OtA5092	OtA5093-OtA5097
Myh13	chr11:67327102-67371502	OtA5088-OtA5092	OtA5093-OtA5097
Myh15	chr16:49057485-49199104	OtA5088-OtA5092	OtA5093-OtA5097
Myh2	chr11:67171026-67197517	OtA5088-OtA5092	OtA5093-OtA5097
Myh3	chr11:67078299-67102291	OtA5088-OtA5092	OtA5093-OtA5097
Myh4	chr11:67238028-67260446	OtA5088-OtA5092	OtA5093-OtA5097
Myh6	chr14:54941920-54966607	OtA5088-OtA5092	OtA5093-OtA5097
Myh7	chr14:54968786-54994549	OtA5088-OtA5092	OtA5093-OtA5097
Myh7b	chr2:155611211-155692384	OtA5088-OtA5092	OtA5093-OtA5097
Myh8	chr11:67277123-67308633	OtA5088-OtA5092	OtA5093-OtA5097
Myl1	chr1:66924295-66945056	OtA5088-OtA5092	OtA5093-OtA5097
Myl10	chr5:136693145-136701094	OtA5088-OtA5092	OtA5093-OtA5097
Myl2	chr5:122100979-122106854	OtA5088-OtA5092	OtA5093-OtA5097
Myl6b	chr10:128494156-128498685	OtA5088-OtA5092	OtA5093-OtA5097
Mylk2	chr2:152911351-152923065	OtA5088-OtA5092	OtA5093-OtA5097
Mylk4	chr13:32700826-32781779	OtA5088-OtA5092	OtA5093-OtA5097
Mylpf	chr7:127211607-127214287	OtA5088-OtA5092	OtA5093-OtA5097
Myo15	chr11:60469338-60528368	OtA5088-OtA5092	OtA5093-OtA5097
Myo16	chr8:10153922-10633950	OtA5088-OtA5092	OtA5093-OtA5097
Myo1a	chr10:127705255-127720940	OtA5088-OtA5092	OtA5093-OtA5097
Myo1h	chr5:114314940-114380505	OtA5088-OtA5092	OtA5093-OtA5097
Myo3a	chr2:22227502-22423370	OtA5088-OtA5092	OtA5093-OtA5097
Myo3b	chr2:70039125-70429195	OtA5088-OtA5092	OtA5093-OtA5097
Myo5c	chr9:75232013-75305450	OtA5088-OtA5092	OtA5093-OtA5097
Myo7b	chr18:31959233-32036931	OtA5088-OtA5092	OtA5093-OtA5097
Myoc	chr1:162639149-162658219	OtA5088-OtA5092	OtA5093-OtA5097
Myocd	chr11:65176570-65269989	OtA5088-OtA5092	OtA5093-OtA5097
Myod1	chr7:46376473-46379092	OtA5088-OtA5092	OtA5093-OtA5097
Myog	chr1:134290003-134292548	OtA5088-OtA5092	OtA5093-OtA5097
Myom2	chr8:15057652-15133419	OtA5088-OtA5092	OtA5093-OtA5097
Myot	chr18:44334073-44355722	OtA5088-OtA5092	OtA5093-OtA5097
Myoz1	chr14:20649101-20656540	OtA5088-OtA5092	OtA5093-OtA5097
Myoz2	chr3:123006205-123034987	OtA5088-OtA5092	OtA5093-OtA5097
Myoz3	chr18:60573716-60591716	OtA5088-OtA5092	OtA5093-OtA5097
Mypn	chr10:63115794-63203952	OtA5088-OtA5092	OtA5093-OtA5097
Myrip	chr9:120304072-120474834	OtA5088-OtA5092	OtA5093-OtA5097

Myt1	chr2:181763331-181827775	OtA5088-OtA5092	OtA5093-OtA5097
Myt1l	chr12:29528383-29923209	OtA5088-OtA5092	OtA5093-OtA5097
Mzf1	chr7:13042302-13054764	OtA5088-OtA5092	OtA5093-OtA5097
N28178	chr4:42917250-42941607	OtA5088-OtA5092	OtA5093-OtA5097
Naa11	chr5:97382208-97392330	OtA5088-OtA5092	OtA5093-OtA5097
Naalad2	chr9:18323020-18385928	OtA5088-OtA5092	OtA5093-OtA5097
Naaladl1	chr19:6105797-6115555	OtA5088-OtA5092	OtA5093-OtA5097
Nacad	chr11:6597822-6606054	OtA5088-OtA5092	OtA5093-OtA5097
Nags	chr11:102145586-102149476	OtA5088-OtA5092	OtA5093-OtA5097
Naip1	chr13:100407769-100452864	OtA5088-OtA5092	OtA5093-OtA5097
Naip2	chr13:100144062-100202092	OtA5088-OtA5092	OtA5093-OtA5097
Naip5	chr13:100211739-100247336	OtA5088-OtA5092	OtA5093-OtA5097
Naip6	chr13:100281120-100317688	OtA5088-OtA5092	OtA5093-OtA5097
Naip7	chr13:100281120-100317688	OtA5088-OtA5092	OtA5093-OtA5097
Nalcn	chr14:123276640-123627144	OtA5088-OtA5092	OtA5093-OtA5097
Nanog	chr6:122707567-122713778	OtA5088-OtA5092	OtA5093-OtA5097
Nanos1	chr19:60755986-60759914	OtA5088-OtA5092	OtA5093-OtA5097
Nanos2	chr7:18987523-18988962	OtA5088-OtA5092	OtA5093-OtA5097
Nanos3	chr8:84173732-84176552	OtA5088-OtA5092	OtA5093-OtA5097
Nanp	chr2:151029684-151039379	OtA5088-OtA5092	OtA5093-OtA5097
Nap1l2	chrX:103184058-103186664	OtA5088-OtA5092	OtA5093-OtA5097
Nap1l3	chrX:122394560-122397385	OtA5088-OtA5092	OtA5093-OtA5097
Nap1l5	chr6:58833699-58920396	OtA5088-OtA5092	OtA5093-OtA5097
Napb	chr2:148694656-148732420	OtA5088-OtA5092	OtA5093-OtA5097
Nat1	chr8:67490757-67492103	OtA5088-OtA5092	OtA5093-OtA5097
Nat14	chr7:4922250-4925006	OtA5088-OtA5092	OtA5093-OtA5097
Nat2	chr8:67494874-67502578	OtA5088-OtA5092	OtA5093-OtA5097
Nat3	chr8:67523853-67548627	OtA5088-OtA5092	OtA5093-OtA5097
Nat8	chr6:85830386-85831890	OtA5088-OtA5092	OtA5093-OtA5097
Nat8l	chr5:33995983-34005916	OtA5088-OtA5092	OtA5093-OtA5097
Nbea	chr3:55625197-56183701	OtA5088-OtA5092	OtA5093-OtA5097
Ncam2	chr16:81200696-81624287	OtA5088-OtA5092	OtA5093-OtA5097
Ncan	chr8:70093084-70120844	OtA5088-OtA5092	OtA5093-OtA5097
Ncapg	chr5:45669924-45857540	OtA5088-OtA5092	OtA5093-OtA5097
Ncapg2	chr12:116405401-116463531	OtA5088-OtA5092	OtA5093-OtA5097
Ncaph	chr2:127103809-127133954	OtA5088-OtA5092	OtA5093-OtA5097
Ncf4	chr15:78244810-78262580	OtA5088-OtA5092	OtA5093-OtA5097
Nckap5	chr1:125913635-126830632	OtA5088-OtA5092	OtA5093-OtA5097
Ncr1	chr7:4337723-4345164	OtA5088-OtA5092	OtA5093-OtA5097
Ncs1	chr2:31245922-31295471	OtA5088-OtA5092	OtA5093-OtA5097
Nctc1	chr7:142544608-142558598	OtA5088-OtA5092	OtA5093-OtA5097
Ndc80	chr17:71496099-71526857	OtA5088-OtA5092	OtA5093-OtA5097
Ndp	chrX:16885521-16911764	OtA5088-OtA5092	OtA5093-OtA5097
Ndst3	chr3:123526165-123672392	OtA5088-OtA5092	OtA5093-OtA5097
Ndst4	chr3:125404090-125724986	OtA5088-OtA5092	OtA5093-OtA5097
Ndufaf4	chr4:24898082-24905001	OtA5088-OtA5092	OtA5093-OtA5097
Neb	chr2:52136646-52338798	OtA5088-OtA5092	OtA5093-OtA5097

Nebl	chr2:17346733-17731068	OtA5088-OtA5092	OtA5093-OtA5097
Necab1	chr4:14930640-15149131	OtA5088-OtA5092	OtA5093-OtA5097
Necab2	chr8:119446718-119472635	OtA5088-OtA5092	OtA5093-OtA5097
Necab3	chr2:154544404-154558853	OtA5088-OtA5092	OtA5093-OtA5097
Nefh	chr11:4938755-5042794	OtA5088-OtA5092	OtA5093-OtA5097
Nefl	chr14:68083883-68087737	OtA5088-OtA5092	OtA5093-OtA5097
Nefm	chr14:68119544-68131371	OtA5088-OtA5092	OtA5093-OtA5097
Negr1	chr3:156561793-157316447	OtA5088-OtA5092	OtA5093-OtA5097
Neil3	chr8:53586866-53639065	OtA5088-OtA5092	OtA5093-OtA5097
Nek10	chr14:14820814-15006693	OtA5088-OtA5092	OtA5093-OtA5097
Nek11	chr9:105162466-105395287	OtA5088-OtA5092	OtA5093-OtA5097
Nek2	chr1:191821472-191833049	OtA5088-OtA5092	OtA5093-OtA5097
Nek3	chr8:22128282-22166435	OtA5088-OtA5092	OtA5093-OtA5097
Nek5	chr8:22073615-22125053	OtA5088-OtA5092	OtA5093-OtA5097
Nell1	chr7:49975349-50863289	OtA5088-OtA5092	OtA5093-OtA5097
Nell2	chr15:95219449-95528706	OtA5088-OtA5092	OtA5093-OtA5097
Nepn	chr10:52391607-52404604	OtA5088-OtA5092	OtA5093-OtA5097
Neto1	chr18:86394951-86501897	OtA5088-OtA5092	OtA5093-OtA5097
Neto2	chr8:85636587-85691009	OtA5088-OtA5092	OtA5093-OtA5097
Neu2	chr1:87574026-87597840	OtA5088-OtA5092	OtA5093-OtA5097
Neu4	chr1:94020492-94028330	OtA5088-OtA5092	OtA5093-OtA5097
Neurl3	chr1:36264601-36273425	OtA5088-OtA5092	OtA5093-OtA5097
Neurod1	chr2:79452640-79456636	OtA5088-OtA5092	OtA5093-OtA5097
Neurod2	chr11:98325416-98329645	OtA5088-OtA5092	OtA5093-OtA5097
Neurod4	chr10:130268151-130280240	OtA5088-OtA5092	OtA5093-OtA5097
Neurod6	chr6:55677818-55681263	OtA5088-OtA5092	OtA5093-OtA5097
Neurog1	chr13:56250497-56252163	OtA5088-OtA5092	OtA5093-OtA5097
Neurog3	chr10:62133089-62134763	OtA5088-OtA5092	OtA5093-OtA5097
Nexn	chr3:152236983-152266320	OtA5088-OtA5092	OtA5093-OtA5097
Nfam1	chr15:82997795-83023138	OtA5088-OtA5092	OtA5093-OtA5097
Nfasc	chr1:132564689-132741797	OtA5088-OtA5092	OtA5093-OtA5097
Nfatc2	chr2:168476409-168601655	OtA5088-OtA5092	OtA5093-OtA5097
Nfkbid	chr7:30423303-30428746	OtA5088-OtA5092	OtA5093-OtA5097
Ng23	chr17:35016578-35028016	OtA5088-OtA5092	OtA5093-OtA5097
Ngb	chr12:87097530-87102539	OtA5088-OtA5092	OtA5093-OtA5097
Ngef	chr1:87476828-87573870	OtA5088-OtA5092	OtA5093-OtA5097
Ngf	chr3:102469927-102521013	OtA5088-OtA5092	OtA5093-OtA5097
Ngfr	chr11:95568820-95587698	OtA5088-OtA5092	OtA5093-OtA5097
Ngp	chr9:110419807-110423012	OtA5088-OtA5092	OtA5093-OtA5097
Nhedc1	chr3:135348036-135397827	OtA5088-OtA5092	OtA5093-OtA5097
Nhlh1	chr1:172052291-172057596	OtA5088-OtA5092	OtA5093-OtA5097
Nhlh2	chr3:102010144-102015492	OtA5088-OtA5092	OtA5093-OtA5097
Nhlrc1	chr13:47012556-47014850	OtA5088-OtA5092	OtA5093-OtA5097
Nhlrc4	chr17:25942232-25944931	OtA5088-OtA5092	OtA5093-OtA5097
Niacr1	chr5:123863569-123865516	OtA5088-OtA5092	OtA5093-OtA5097
Ninj2	chr6:120093379-120200338	OtA5088-OtA5092	OtA5093-OtA5097
Ninl	chr2:150934518-151009398	OtA5088-OtA5092	OtA5093-OtA5097

Nipal2	chr15:34572798-34679204	OtA5088-OtA5092	OtA5093-OtA5097
Nipsnap1	chr11:4874002-4894200	OtA5088-OtA5092	OtA5093-OtA5097
Nipsnap3a	chr4:52989283-53000854	OtA5088-OtA5092	OtA5093-OtA5097
Nkain2	chr10:31689318-32889915	OtA5088-OtA5092	OtA5093-OtA5097
Nkain3	chr4:20243852-20778668	OtA5088-OtA5092	OtA5093-OtA5097
Nkain4	chr2:180934771-180954699	OtA5088-OtA5092	OtA5093-OtA5097
Nkapl	chr13:21467046-21468501	OtA5088-OtA5092	OtA5093-OtA5097
Nkd2	chr13:73819327-73847631	OtA5088-OtA5092	OtA5093-OtA5097
Nkg7	chr7:43437137-43438246	OtA5088-OtA5092	OtA5093-OtA5097
Nkiras1	chr14:18271141-18331844	OtA5088-OtA5092	OtA5093-OtA5097
Nkpd1	chr7:19518730-19525050	OtA5088-OtA5092	OtA5093-OtA5097
Nkrf	chrX:36887541-36902899	OtA5088-OtA5092	OtA5093-OtA5097
Nkx1-2	chr7:132596238-132599637	OtA5088-OtA5092	OtA5093-OtA5097
Nkx2-1	chr12:56531934-56536908	OtA5088-OtA5092	OtA5093-OtA5097
Nkx2-2	chr2:147177545-147331681	OtA5088-OtA5092	OtA5093-OtA5097
Nkx2-2as	chr2:147177545-147331681	OtA5088-OtA5092	OtA5093-OtA5097
Nkx2-3	chr19:43612324-43615892	OtA5088-OtA5092	OtA5093-OtA5097
Nkx2-4	chr2:147083875-147085345	OtA5088-OtA5092	OtA5093-OtA5097
Nkx2-5	chr17:26838664-26841565	OtA5088-OtA5092	OtA5093-OtA5097
Nkx2-6	chr14:69164798-69175540	OtA5088-OtA5092	OtA5093-OtA5097
Nkx2-9	chr12:56611396-56613284	OtA5088-OtA5092	OtA5093-OtA5097
Nkx3-1	chr14:69190691-69194658	OtA5088-OtA5092	OtA5093-OtA5097
Nkx3-2	chr5:41761482-41764220	OtA5088-OtA5092	OtA5093-OtA5097
Nkx6-1	chr5:101659196-101664711	OtA5088-OtA5092	OtA5093-OtA5097
Nkx6-2	chr7:139389108-139582797	OtA5088-OtA5092	OtA5093-OtA5097
Nkx6-3	chr8:23153270-23158948	OtA5088-OtA5092	OtA5093-OtA5097
Nlgn1	chr3:25431840-26331909	OtA5088-OtA5092	OtA5093-OtA5097
Nlgn3	chrX:101299178-101321350	OtA5088-OtA5092	OtA5093-OtA5097
Nlrc3	chr16:3946932-3976632	OtA5088-OtA5092	OtA5093-OtA5097
Nlrc4	chr17:74426294-74459108	OtA5088-OtA5092	OtA5093-OtA5097
Nlrp10	chr7:108921852-108930158	OtA5088-OtA5092	OtA5093-OtA5097
Nlrp12	chr7:3221509-3249740	OtA5088-OtA5092	OtA5093-OtA5097
Nlrp14	chr7:107166989-107198103	OtA5088-OtA5092	OtA5093-OtA5097
Nlrp1a	chr11:71091196-71144704	OtA5088-OtA5092	OtA5093-OtA5097
Nlrp1b	chr11:71153101-71230733	OtA5088-OtA5092	OtA5093-OtA5097
Nlrp1c-ps	chr11:71242429-71285232	OtA5088-OtA5092	OtA5093-OtA5097
Nlrp2	chr7:5298546-5360681	OtA5088-OtA5092	OtA5093-OtA5097
Nlrp3	chr11:59542685-59566956	OtA5088-OtA5092	OtA5093-OtA5097
Nlrp4a	chr7:26435112-26475458	OtA5088-OtA5092	OtA5093-OtA5097
Nlrp4b	chr7:10687792-10730158	OtA5088-OtA5092	OtA5093-OtA5097
Nlrp4c	chr7:6045160-6105149	OtA5088-OtA5092	OtA5093-OtA5097
Nlrp4e	chr7:23301191-23362277	OtA5088-OtA5092	OtA5093-OtA5097
Nlrp4f	chr13:65177110-65205716	OtA5088-OtA5092	OtA5093-OtA5097
Nlrp4g	chr9:124348830-124354483	OtA5088-OtA5092	OtA5093-OtA5097
Nlrp5	chr7:23385896-23441921	OtA5088-OtA5092	OtA5093-OtA5097
Nlrp6	chr7:140920901-140928540	OtA5088-OtA5092	OtA5093-OtA5097
Nlrp9a	chr7:26535064-26574146	OtA5088-OtA5092	OtA5093-OtA5097

Nlrp9b	chr7:20008022-20062938	OtA5088-OtA5092	OtA5093-OtA5097
Nlrp9c	chr7:26364886-26394243	OtA5088-OtA5092	OtA5093-OtA5097
Nmbr	chr10:14760288-14770556	OtA5088-OtA5092	OtA5093-OtA5097
Nmral1	chr16:4711317-4719059	OtA5088-OtA5092	OtA5093-OtA5097
Nms	chr1:38939148-38950276	OtA5088-OtA5092	OtA5093-OtA5097
Nmu	chr5:76183879-76469519	OtA5088-OtA5092	OtA5093-OtA5097
Nmur1	chr1:86386324-86388141	OtA5088-OtA5092	OtA5093-OtA5097
Nmur2	chr11:56024989-56040987	OtA5088-OtA5092	OtA5093-OtA5097
Nobox	chr6:43303673-43309554	OtA5088-OtA5092	OtA5093-OtA5097
Nod2	chr8:88647346-88688474	OtA5088-OtA5092	OtA5093-OtA5097
Nodal	chr10:61417971-61425337	OtA5088-OtA5092	OtA5093-OtA5097
Nol3	chr8:105276446-105289528	OtA5088-OtA5092	OtA5093-OtA5097
Nol4	chr18:22693154-23041653	OtA5088-OtA5092	OtA5093-OtA5097
Noto	chr6:85423885-85428877	OtA5088-OtA5092	OtA5093-OtA5097
Nova1	chr12:46694516-46818775	OtA5088-OtA5092	OtA5093-OtA5097
Nova2	chr7:18925887-18965319	OtA5088-OtA5092	OtA5093-OtA5097
Nox1	chrX:134087145-134111708	OtA5088-OtA5092	OtA5093-OtA5097
Nox3	chr17:3635239-3696261	OtA5088-OtA5092	OtA5093-OtA5097
Noxa1	chr2:25080322-25095205	OtA5088-OtA5092	OtA5093-OtA5097
Noxo1	chr17:24696233-24700529	OtA5088-OtA5092	OtA5093-OtA5097
Npas1	chr7:16455720-16476780	OtA5088-OtA5092	OtA5093-OtA5097
Npas3	chr12:53248676-54072175	OtA5088-OtA5092	OtA5093-OtA5097
Npas4	chr19:4984354-4989971	OtA5088-OtA5092	OtA5093-OtA5097
Npbwr1	chr1:5913706-5917398	OtA5088-OtA5092	OtA5093-OtA5097
Npc1l1	chr11:6211010-6230245	OtA5088-OtA5092	OtA5093-OtA5097
Npcd	chr15:79786350-79834333	OtA5088-OtA5092	OtA5093-OtA5097
Npff	chr15:102523838-102524942	OtA5088-OtA5092	OtA5093-OtA5097
Npffr1	chr10:61595483-61626385	OtA5088-OtA5092	OtA5093-OtA5097
Npffr2	chr5:89527428-89583740	OtA5088-OtA5092	OtA5093-OtA5097
Nphp1	chr2:127740731-127788854	OtA5088-OtA5092	OtA5093-OtA5097
Nphp4	chr4:152478141-152563184	OtA5088-OtA5092	OtA5093-OtA5097
Nphs1	chr7:30460057-30488609	OtA5088-OtA5092	OtA5093-OtA5097
Nphs1as	chr7:30460057-30488609	OtA5088-OtA5092	OtA5093-OtA5097
Nphs2	chr1:156310734-156328035	OtA5088-OtA5092	OtA5093-OtA5097
Npm2	chr14:70647301-70653084	OtA5088-OtA5092	OtA5093-OtA5097
Npm3-ps1	chr6:85076140-85077126	OtA5088-OtA5092	OtA5093-OtA5097
Nppa	chr4:148000745-148002067	OtA5088-OtA5092	OtA5093-OtA5097
Nps	chr7:135268618-135272942	OtA5088-OtA5092	OtA5093-OtA5097
Npsr1	chr9:24098017-24316398	OtA5088-OtA5092	OtA5093-OtA5097
Nptx2	chr5:144545886-144557478	OtA5088-OtA5092	OtA5093-OtA5097
Npvf	chr6:50650870-50654393	OtA5088-OtA5092	OtA5093-OtA5097
Npw	chr17:24657329-24658425	OtA5088-OtA5092	OtA5093-OtA5097
Npy1r	chr8:66697421-66706798	OtA5088-OtA5092	OtA5093-OtA5097
Npy2r	chr3:82538382-82548085	OtA5088-OtA5092	OtA5093-OtA5097
Npy5r	chr8:66679964-66688094	OtA5088-OtA5092	OtA5093-OtA5097
Npy6r	chr18:44270126-44277700	OtA5088-OtA5092	OtA5093-OtA5097
Nqo2	chr13:33917917-34002794	OtA5088-OtA5092	OtA5093-OtA5097

Nr0b1	chrX:86191774-86195944	OtA5088-OtA5092	OtA5093-OtA5097
Nr0b2	chr4:133553389-133556536	OtA5088-OtA5092	OtA5093-OtA5097
Nr1h5	chr3:102939657-102964133	OtA5088-OtA5092	OtA5093-OtA5097
Nr1i2	chr16:38248348-38294824	OtA5088-OtA5092	OtA5093-OtA5097
Nr1i3	chr1:171213969-171222514	OtA5088-OtA5092	OtA5093-OtA5097
Nr2e1	chr10:42561970-42583588	OtA5088-OtA5092	OtA5093-OtA5097
Nr2e3	chr9:59942770-59950079	OtA5088-OtA5092	OtA5093-OtA5097
Nr2f1	chr13:78188972-78236564	OtA5088-OtA5092	OtA5093-OtA5097
Nr3c2	chr8:76902507-77243639	OtA5088-OtA5092	OtA5093-OtA5097
Nr4a2	chr2:57107225-57124003	OtA5088-OtA5092	OtA5093-OtA5097
Nr4a3	chr4:48051247-48083352	OtA5088-OtA5092	OtA5093-OtA5097
Nr5a1	chr2:38692659-38714542	OtA5088-OtA5092	OtA5093-OtA5097
Nr5a2	chr1:136843583-136960448	OtA5088-OtA5092	OtA5093-OtA5097
Nrap	chr19:56287937-56390035	OtA5088-OtA5092	OtA5093-OtA5097
Nrg1	chr8:31818027-31918203	OtA5088-OtA5092	OtA5093-OtA5097
Nrg2	chr18:36017657-36197160	OtA5088-OtA5092	OtA5093-OtA5097
Nrg3	chr14:38368950-39473088	OtA5088-OtA5092	OtA5093-OtA5097
Nrg4	chr9:55208934-55283625	OtA5088-OtA5092	OtA5093-OtA5097
Nrip2	chr6:128399765-128408935	OtA5088-OtA5092	OtA5093-OtA5097
Nrip3	chr7:109758055-109781545	OtA5088-OtA5092	OtA5093-OtA5097
Nrl	chr14:55518977-55524965	OtA5088-OtA5092	OtA5093-OtA5097
Nrn1	chr13:36725624-36734454	OtA5088-OtA5092	OtA5093-OtA5097
Nron	chr2:33729955-33887946	OtA5088-OtA5092	OtA5093-OtA5097
Nrp	chr12:87428077-87472299	OtA5088-OtA5092	OtA5093-OtA5097
Nrsn1	chr13:25252038-25269996	OtA5088-OtA5092	OtA5093-OtA5097
Nrsn2	chr2:152345668-152376566	OtA5088-OtA5092	OtA5093-OtA5097
Nrxn1	chr17:90033643-91092802	OtA5088-OtA5092	OtA5093-OtA5097
Nrxn2	chr19:6418737-6533217	OtA5088-OtA5092	OtA5093-OtA5097
Nrxn3	chr12:88722875-90334933	OtA5088-OtA5092	OtA5093-OtA5097
Nsg1	chr5:38137192-38159467	OtA5088-OtA5092	OtA5093-OtA5097
Nsg2	chr11:32000458-32059202	OtA5088-OtA5092	OtA5093-OtA5097
Nsl1	chr1:191063020-191084558	OtA5088-OtA5092	OtA5093-OtA5097
Nsun7	chr5:66260124-66298016	OtA5088-OtA5092	OtA5093-OtA5097
Nt5c1a	chr4:123201552-123216207	OtA5088-OtA5092	OtA5093-OtA5097
Nt5c1b	chr12:10369970-10390174	OtA5088-OtA5092	OtA5093-OtA5097
Ntf3	chr6:126101411-126166744	OtA5088-OtA5092	OtA5093-OtA5097
Ntm	chr9:28995963-29963129	OtA5088-OtA5092	OtA5093-OtA5097
Ntn5	chr7:45684413-45694554	OtA5088-OtA5092	OtA5093-OtA5097
Ntng1	chr3:109780049-110143472	OtA5088-OtA5092	OtA5093-OtA5097
Ntng2	chr2:29194820-29252993	OtA5088-OtA5092	OtA5093-OtA5097
Ntrk1	chr3:87778243-87795162	OtA5088-OtA5092	OtA5093-OtA5097
Ntrk3	chr7:78192113-78577838	OtA5088-OtA5092	OtA5093-OtA5097
Nts	chr10:102481755-102490418	OtA5088-OtA5092	OtA5093-OtA5097
Ntsr1	chr2:180499975-180544979	OtA5088-OtA5092	OtA5093-OtA5097
Ntsr2	chr12:16653469-16660236	OtA5088-OtA5092	OtA5093-OtA5097
Nudt10	chrX:6168695-6172991	OtA5088-OtA5092	OtA5093-OtA5097
Nudt11	chrX:6047506-6054751	OtA5088-OtA5092	OtA5093-OtA5097

Nudt15	chr14:73519863-73548242	OtA5088-OtA5092	OtA5093-OtA5097
Nuf2	chr1:169497933-169531464	OtA5088-OtA5092	OtA5093-OtA5097
Nup210l	chr3:90104131-90212017	OtA5088-OtA5092	OtA5093-OtA5097
Nup62-il4i1	chr7:44812255-44849079	OtA5088-OtA5092	OtA5093-OtA5097
Nusap1	chr2:119609531-119650160	OtA5088-OtA5092	OtA5093-OtA5097
Nwd1	chr8:72646710-72714748	OtA5088-OtA5092	OtA5093-OtA5097
Nxf2	chrX:134944530-134964754	OtA5088-OtA5092	OtA5093-OtA5097
Nxf3	chrX:136072098-136085255	OtA5088-OtA5092	OtA5093-OtA5097
Nxnl1	chr8:71560554-71566649	OtA5088-OtA5092	OtA5093-OtA5097
Nxnl2	chr13:51171024-51175187	OtA5088-OtA5092	OtA5093-OtA5097
Nxph1	chr6:8950018-9248578	OtA5088-OtA5092	OtA5093-OtA5097
Nxph2	chr2:23321245-23401973	OtA5088-OtA5092	OtA5093-OtA5097
Nxph3	chr11:95509845-95514565	OtA5088-OtA5092	OtA5093-OtA5097
Nxph4	chr10:127525472-127534559	OtA5088-OtA5092	OtA5093-OtA5097
Nxt2	chrX:142227936-142239692	OtA5088-OtA5092	OtA5093-OtA5097
O3far1	chr19:38097078-38114263	OtA5088-OtA5092	OtA5093-OtA5097
ORF63	chr16:87553329-87595336	OtA5088-OtA5092	OtA5093-OtA5097
OTTMUSG0000001660	chr2:176619932-176636319	OtA5088-OtA5092	OtA5093-OtA5097
Oas1d	chr5:120914817-120921647	OtA5088-OtA5092	OtA5093-OtA5097
Oas1e	chr5:120786311-120795530	OtA5088-OtA5092	OtA5093-OtA5097
Oas1f	chr5:120847366-120857986	OtA5088-OtA5092	OtA5093-OtA5097
Oas1h	chr5:120861421-120873505	OtA5088-OtA5092	OtA5093-OtA5097
Oas2	chr5:120730332-120749848	OtA5088-OtA5092	OtA5093-OtA5097
Oaz3	chr3:94433165-94436616	OtA5088-OtA5092	OtA5093-OtA5097
Obox1	chr7:15547256-15556846	OtA5088-OtA5092	OtA5093-OtA5097
Obox2	chr7:15388850-15398543	OtA5088-OtA5092	OtA5093-OtA5097
Obox3	chr7:15625305-15639777	OtA5088-OtA5092	OtA5093-OtA5097
Obox5	chr7:15750369-15759274	OtA5088-OtA5092	OtA5093-OtA5097
Obox6	chr7:15833249-15839679	OtA5088-OtA5092	OtA5093-OtA5097
Obp1a	chrX:78085504-78091374	OtA5088-OtA5092	OtA5093-OtA5097
Obscn	chr11:58994255-59136375	OtA5088-OtA5092	OtA5093-OtA5097
Oc90	chr15:65876052-65912287	OtA5088-OtA5092	OtA5093-OtA5097
Oca2	chr7:56239770-56536517	OtA5088-OtA5092	OtA5093-OtA5097
Ociad2	chr5:73322198-73338639	OtA5088-OtA5092	OtA5093-OtA5097
Ocm	chr5:144019806-144050609	OtA5088-OtA5092	OtA5093-OtA5097
Ocstamp	chr2:165395449-165400394	OtA5088-OtA5092	OtA5093-OtA5097
Odam	chr5:87885694-87892742	OtA5088-OtA5092	OtA5093-OtA5097
Odf1	chr15:38219202-38226735	OtA5088-OtA5092	OtA5093-OtA5097
Odf3	chr7:140847915-140850925	OtA5088-OtA5092	OtA5093-OtA5097
Odf3b	chr15:89377448-89379254	OtA5088-OtA5092	OtA5093-OtA5097
Odf3l1	chr9:56848658-56851963	OtA5088-OtA5092	OtA5093-OtA5097
Odf3l2	chr10:79639525-79645738	OtA5088-OtA5092	OtA5093-OtA5097
Odf4	chr11:68921834-68927081	OtA5088-OtA5092	OtA5093-OtA5097
Odz1	chrX:42532392-43274786	OtA5088-OtA5092	OtA5093-OtA5097
Odz2	chr11:36006668-36944243	OtA5088-OtA5092	OtA5093-OtA5097
Odz3	chr8:48225664-48674690	OtA5088-OtA5092	OtA5093-OtA5097
Ofcc1	chr13:40001881-40288011	OtA5088-OtA5092	OtA5093-OtA5097

Ogfrl1	chr1:23366423-23383175	OtA5088-OtA5092	OtA5093-OtA5097
Ogn	chr13:49464058-49652731	OtA5088-OtA5092	OtA5093-OtA5097
Oip5	chr2:119609531-119650160	OtA5088-OtA5092	OtA5093-OtA5097
Oit1	chr14:8348947-8378763	OtA5088-OtA5092	OtA5093-OtA5097
Olah	chr2:3341987-3366569	OtA5088-OtA5092	OtA5093-OtA5097
Olfm2	chr9:20667985-20728214	OtA5088-OtA5092	OtA5093-OtA5097
Olfm3	chr3:114863252-115125256	OtA5088-OtA5092	OtA5093-OtA5097
Olfm4	chr14:80000301-80021930	OtA5088-OtA5092	OtA5093-OtA5097
Olfml2a	chr2:38931979-38960585	OtA5088-OtA5092	OtA5093-OtA5097
Olfr1	chr11:73395075-73399495	OtA5088-OtA5092	OtA5093-OtA5097
Olfr10	chr11:49317547-49318483	OtA5088-OtA5092	OtA5093-OtA5097
Olfr100,Olfr102	chr17:37313410-37314437	OtA5088-OtA5092	OtA5093-OtA5097
Olfr1000	chr2:85607963-85608908	OtA5088-OtA5092	OtA5093-OtA5097
Olfr1002	chr2:85647362-85648319	OtA5088-OtA5092	OtA5093-OtA5097
Olfr1006	chr2:85674210-85678741	OtA5088-OtA5092	OtA5093-OtA5097
Olfr1008	chr2:85689430-85690372	OtA5088-OtA5092	OtA5093-OtA5097
Olfr1009	chr2:85721406-85722351	OtA5088-OtA5092	OtA5093-OtA5097
Olfr101	chr17:37299493-37300420	OtA5088-OtA5092	OtA5093-OtA5097
Olfr1010	chr2:85753362-85754306	OtA5088-OtA5092	OtA5093-OtA5097
Olfr1012	chr2:85759438-85760374	OtA5088-OtA5092	OtA5093-OtA5097
Olfr1013	chr2:85769802-85770720	OtA5088-OtA5092	OtA5093-OtA5097
Olfr1014	chr2:85776585-85777503	OtA5088-OtA5092	OtA5093-OtA5097
Olfr1015	chr2:85785473-85786489	OtA5088-OtA5092	OtA5093-OtA5097
Olfr1016	chr2:85799338-85800268	OtA5088-OtA5092	OtA5093-OtA5097
Olfr1018	chr2:85822972-85823908	OtA5088-OtA5092	OtA5093-OtA5097
Olfr1019	chr2:85840856-85841789	OtA5088-OtA5092	OtA5093-OtA5097
Olfr102	chr17:37313410-37314437	OtA5088-OtA5092	OtA5093-OtA5097
Olfr1020	chr2:85849419-85850498	OtA5088-OtA5092	OtA5093-OtA5097
Olfr1022	chr2:85868593-85869541	OtA5088-OtA5092	OtA5093-OtA5097
Olfr1023	chr2:85886801-85887737	OtA5088-OtA5092	OtA5093-OtA5097
Olfr1024	chr2:85904068-85905052	OtA5088-OtA5092	OtA5093-OtA5097
Olfr1026	chr2:85923269-85924193	OtA5088-OtA5092	OtA5093-OtA5097
Olfr1028	chr2:85951064-85952039	OtA5088-OtA5092	OtA5093-OtA5097
Olfr1029	chr2:85975212-85976273	OtA5088-OtA5092	OtA5093-OtA5097
Olfr103	chr17:37336288-37337230	OtA5088-OtA5092	OtA5093-OtA5097
Olfr1030	chr2:85979311-85984798	OtA5088-OtA5092	OtA5093-OtA5097
Olfr1031	chr2:85991818-85992829	OtA5088-OtA5092	OtA5093-OtA5097
Olfr1032	chr2:86007777-86008710	OtA5088-OtA5092	OtA5093-OtA5097
Olfr1033	chr2:86020639-86044808	OtA5088-OtA5092	OtA5093-OtA5097
Olfr1034	chr2:86046443-86047466	OtA5088-OtA5092	OtA5093-OtA5097
Olfr1036	chr2:86074703-86075718	OtA5088-OtA5092	OtA5093-OtA5097
Olfr1037	chr2:86084729-86085854	OtA5088-OtA5092	OtA5093-OtA5097
Olfr1038-ps	chr2:86120223-86123037	OtA5088-OtA5092	OtA5093-OtA5097
Olfr1039	chr2:86130701-86131661	OtA5088-OtA5092	OtA5093-OtA5097
Olfr1040	chr2:86145790-86146732	OtA5088-OtA5092	OtA5093-OtA5097
Olfr1042	chr2:86159344-86160441	OtA5088-OtA5092	OtA5093-OtA5097
Olfr1043	chr2:86162002-86162947	OtA5088-OtA5092	OtA5093-OtA5097

Olfr1044	chr2:86170870-86171815	OtA5088-OtA5092	OtA5093-OtA5097
Olfr1045	chr2:86197802-86198750	OtA5088-OtA5092	OtA5093-OtA5097
Olfr1046	chr2:86216757-86217708	OtA5088-OtA5092	OtA5093-OtA5097
Olfr1047	chr2:86228009-86228969	OtA5088-OtA5092	OtA5093-OtA5097
Olfr1048	chr2:86235870-86236833	OtA5088-OtA5092	OtA5093-OtA5097
Olfr1049	chr2:86254764-86255691	OtA5088-OtA5092	OtA5093-OtA5097
Olfr1051	chr2:86275558-86276485	OtA5088-OtA5092	OtA5093-OtA5097
Olfr1052	chr2:86297817-86298756	OtA5088-OtA5092	OtA5093-OtA5097
Olfr1053	chr2:86314342-86315285	OtA5088-OtA5092	OtA5093-OtA5097
Olfr1054	chr2:86332415-86333354	OtA5088-OtA5092	OtA5093-OtA5097
Olfr1055	chr2:86346825-86347764	OtA5088-OtA5092	OtA5093-OtA5097
Olfr1056	chr2:86355358-86356437	OtA5088-OtA5092	OtA5093-OtA5097
Olfr1057	chr2:86374462-86375410	OtA5088-OtA5092	OtA5093-OtA5097
Olfr1058	chr2:86385465-86386416	OtA5088-OtA5092	OtA5093-OtA5097
Olfr1061	chr2:86413108-86414050	OtA5088-OtA5092	OtA5093-OtA5097
Olfr1062	chr2:86422718-86423674	OtA5088-OtA5092	OtA5093-OtA5097
Olfr1065	chr2:86445012-86446004	OtA5088-OtA5092	OtA5093-OtA5097
Olfr1066	chr2:86455327-86456269	OtA5088-OtA5092	OtA5093-OtA5097
Olfr107	chr17:37405476-37406509	OtA5088-OtA5092	OtA5093-OtA5097
Olfr1076	chr2:86508460-86509402	OtA5088-OtA5092	OtA5093-OtA5097
Olfr1077-ps1	chr2:86525661-86528427	OtA5088-OtA5092	OtA5093-OtA5097
Olfr1079	chr2:86537965-86538913	OtA5088-OtA5092	OtA5093-OtA5097
Olfr108	chr17:37445479-37446517	OtA5088-OtA5092	OtA5093-OtA5097
Olfr1080	chr2:86553180-86558138	OtA5088-OtA5092	OtA5093-OtA5097
Olfr1082	chr2:86593884-86598809	OtA5088-OtA5092	OtA5093-OtA5097
Olfr1084	chr2:86638764-86639718	OtA5088-OtA5092	OtA5093-OtA5097
Olfr1085	chr2:86657514-86658456	OtA5088-OtA5092	OtA5093-OtA5097
Olfr1086	chr2:86676398-86677331	OtA5088-OtA5092	OtA5093-OtA5097
Olfr1087	chr2:86690031-86690973	OtA5088-OtA5092	OtA5093-OtA5097
Olfr1089	chr2:86732674-86733610	OtA5088-OtA5092	OtA5093-OtA5097
Olfr109	chr17:37466207-37467152	OtA5088-OtA5092	OtA5093-OtA5097
Olfr1090	chr2:86753794-86754736	OtA5088-OtA5092	OtA5093-OtA5097
Olfr1093	chr2:86785731-86786700	OtA5088-OtA5092	OtA5093-OtA5097
Olfr1094	chr2:86828675-86829822	OtA5088-OtA5092	OtA5093-OtA5097
Olfr1095	chr2:86850769-86851696	OtA5088-OtA5092	OtA5093-OtA5097
Olfr1097	chr2:86890225-86892163	OtA5088-OtA5092	OtA5093-OtA5097
Olfr1098	chr2:86922582-86923530	OtA5088-OtA5092	OtA5093-OtA5097
Olfr1099	chr2:86958517-86959456	OtA5088-OtA5092	OtA5093-OtA5097
Olfr11	chr13:21638579-21639521	OtA5088-OtA5092	OtA5093-OtA5097
Olfr110	chr17:37492467-37499674	OtA5088-OtA5092	OtA5093-OtA5097
Olfr1100	chr2:86977828-86978794	OtA5088-OtA5092	OtA5093-OtA5097
Olfr1101	chr2:86988241-86989174	OtA5088-OtA5092	OtA5093-OtA5097
Olfr1102	chr2:87001931-87002994	OtA5088-OtA5092	OtA5093-OtA5097
Olfr1104	chr2:87021609-87022542	OtA5088-OtA5092	OtA5093-OtA5097
Olfr1105	chr2:87033280-87034219	OtA5088-OtA5092	OtA5093-OtA5097
Olfr1106	chr2:87048295-87049234	OtA5088-OtA5092	OtA5093-OtA5097
Olfr1107	chr2:87071115-87072223	OtA5088-OtA5092	OtA5093-OtA5097

Olfr1109	chr2:87092456-87093395	OtA5088-OtA5092	OtA5093-OtA5097
Olfr111	chr17:37529956-37530997	OtA5088-OtA5092	OtA5093-OtA5097
Olfr1110	chr2:87135380-87136319	OtA5088-OtA5092	OtA5093-OtA5097
Olfr1111	chr2:87149719-87150659	OtA5088-OtA5092	OtA5093-OtA5097
Olfr1112	chr2:87191688-87192645	OtA5088-OtA5092	OtA5093-OtA5097
Olfr1113	chr2:87212893-87213874	OtA5088-OtA5092	OtA5093-OtA5097
Olfr1115	chr2:87251839-87252941	OtA5088-OtA5092	OtA5093-OtA5097
Olfr1116-ps	chr2:87268845-87269769	OtA5088-OtA5092	OtA5093-OtA5097
Olfr1118	chr2:87308763-87309759	OtA5088-OtA5092	OtA5093-OtA5097
Olfr112	chr17:37563238-37569451	OtA5088-OtA5092	OtA5093-OtA5097
Olfr1120	chr2:87357445-87358390	OtA5088-OtA5092	OtA5093-OtA5097
Olfr1121	chr2:87371533-87372478	OtA5088-OtA5092	OtA5093-OtA5097
Olfr1122	chr2:87387706-87388687	OtA5088-OtA5092	OtA5093-OtA5097
Olfr1123	chr2:87418049-87419021	OtA5088-OtA5092	OtA5093-OtA5097
Olfr1124	chr2:87434488-87435445	OtA5088-OtA5092	OtA5093-OtA5097
Olfr1126	chr2:87457166-87458111	OtA5088-OtA5092	OtA5093-OtA5097
Olfr1128	chr2:87544606-87545542	OtA5088-OtA5092	OtA5093-OtA5097
Olfr1129	chr2:87575085-87576030	OtA5088-OtA5092	OtA5093-OtA5097
Olfr113	chr17:37574482-37575421	OtA5088-OtA5092	OtA5093-OtA5097
Olfr1130	chr2:87606211-87608334	OtA5088-OtA5092	OtA5093-OtA5097
Olfr1131	chr2:87628464-87629394	OtA5088-OtA5092	OtA5093-OtA5097
Olfr1132	chr2:87634818-87635745	OtA5088-OtA5092	OtA5093-OtA5097
Olfr1133	chr2:87645179-87646121	OtA5088-OtA5092	OtA5093-OtA5097
Olfr1134	chr2:87655924-87658457	OtA5088-OtA5092	OtA5093-OtA5097
Olfr1135	chr2:87671432-87672365	OtA5088-OtA5092	OtA5093-OtA5097
Olfr1136	chr2:87692880-87693947	OtA5088-OtA5092	OtA5093-OtA5097
Olfr1137	chr2:87710971-87711904	OtA5088-OtA5092	OtA5093-OtA5097
Olfr1138	chr2:87737386-87738322	OtA5088-OtA5092	OtA5093-OtA5097
Olfr114	chr17:37589412-37590351	OtA5088-OtA5092	OtA5093-OtA5097
Olfr1140	chr2:87746160-87747180	OtA5088-OtA5092	OtA5093-OtA5097
Olfr1141	chr2:87753055-87753991	OtA5088-OtA5092	OtA5093-OtA5097
Olfr1143	chr2:87802390-87803335	OtA5088-OtA5092	OtA5093-OtA5097
Olfr1145	chr2:87809821-87810799	OtA5088-OtA5092	OtA5093-OtA5097
Olfr1148	chr2:87833040-87833985	OtA5088-OtA5092	OtA5093-OtA5097
Olfr115	chr17:37609784-37610779	OtA5088-OtA5092	OtA5093-OtA5097
Olfr1151	chr2:87857176-87858103	OtA5088-OtA5092	OtA5093-OtA5097
Olfr1152	chr2:87867992-87868925	OtA5088-OtA5092	OtA5093-OtA5097
Olfr1153	chr2:87896176-87897133	OtA5088-OtA5092	OtA5093-OtA5097
Olfr1154	chr2:87902741-87903674	OtA5088-OtA5092	OtA5093-OtA5097
Olfr1155	chr2:87942681-87943626	OtA5088-OtA5092	OtA5093-OtA5097
Olfr1156	chr2:87949189-87950265	OtA5088-OtA5092	OtA5093-OtA5097
Olfr1157	chr2:87961951-87968257	OtA5088-OtA5092	OtA5093-OtA5097
Olfr1158	chr2:87990112-87991054	OtA5088-OtA5092	OtA5093-OtA5097
Olfr116	chr17:37623667-37624633	OtA5088-OtA5092	OtA5093-OtA5097
Olfr1160	chr2:88005816-88006776	OtA5088-OtA5092	OtA5093-OtA5097
Olfr1161	chr2:88024701-88025713	OtA5088-OtA5092	OtA5093-OtA5097
Olfr1162	chr2:88049677-88050622	OtA5088-OtA5092	OtA5093-OtA5097

Olfr1163	chr2:88070364-88071407	OtA5088-OtA5092	OtA5093-OtA5097
Olfr1164	chr2:88092854-88093966	OtA5088-OtA5092	OtA5093-OtA5097
Olfr1166	chr2:88123361-88124993	OtA5088-OtA5092	OtA5093-OtA5097
Olfr1167	chr2:88148983-88150083	OtA5088-OtA5092	OtA5093-OtA5097
Olfr1168	chr2:88184878-88185817	OtA5088-OtA5092	OtA5093-OtA5097
Olfr117	chr17:37659377-37660331	OtA5088-OtA5092	OtA5093-OtA5097
Olfr1170	chr2:88224079-88225030	OtA5088-OtA5092	OtA5093-OtA5097
Olfr1173	chr2:88274108-88275047	OtA5088-OtA5092	OtA5093-OtA5097
Olfr1176	chr2:88339566-88340514	OtA5088-OtA5092	OtA5093-OtA5097
Olfr1178	chr2:88391248-88392220	OtA5088-OtA5092	OtA5093-OtA5097
Olfr1179	chr2:88402008-88402932	OtA5088-OtA5092	OtA5093-OtA5097
Olfr118	chr17:37672024-37672990	OtA5088-OtA5092	OtA5093-OtA5097
Olfr1180	chr2:88411626-88412689	OtA5088-OtA5092	OtA5093-OtA5097
Olfr1181	chr2:88423087-88424023	OtA5088-OtA5092	OtA5093-OtA5097
Olfr1182	chr2:88446018-88449121	OtA5088-OtA5092	OtA5093-OtA5097
Olfr1183	chr2:88461341-88462253	OtA5088-OtA5092	OtA5093-OtA5097
Olfr1184	chr2:88486733-88487669	OtA5088-OtA5092	OtA5093-OtA5097
Olfr1186	chr2:88525500-88526590	OtA5088-OtA5092	OtA5093-OtA5097
Olfr1188	chr2:88559450-88560418	OtA5088-OtA5092	OtA5093-OtA5097
Olfr1189	chr2:88591805-88592726	OtA5088-OtA5092	OtA5093-OtA5097
Olfr119	chr17:37696684-37701637	OtA5088-OtA5092	OtA5093-OtA5097
Olfr1193	chr2:88677856-88678813	OtA5088-OtA5092	OtA5093-OtA5097
Olfr1195	chr2:88682803-88683730	OtA5088-OtA5092	OtA5093-OtA5097
Olfr1196	chr2:88700382-88701327	OtA5088-OtA5092	OtA5093-OtA5097
Olfr1197	chr2:88728652-88729597	OtA5088-OtA5092	OtA5093-OtA5097
Olfr1198	chr2:88745959-88746886	OtA5088-OtA5092	OtA5093-OtA5097
Olfr1199	chr2:88755740-88756673	OtA5088-OtA5092	OtA5093-OtA5097
Olfr12	chr1:92619880-92621001	OtA5088-OtA5092	OtA5093-OtA5097
Olfr120	chr17:37725998-37726991	OtA5088-OtA5092	OtA5093-OtA5097
Olfr1200	chr2:88767302-88768313	OtA5088-OtA5092	OtA5093-OtA5097
Olfr1201	chr2:88794383-88795307	OtA5088-OtA5092	OtA5093-OtA5097
Olfr1202	chr2:88817172-88818102	OtA5088-OtA5092	OtA5093-OtA5097
Olfr1204	chr2:88851951-88852881	OtA5088-OtA5092	OtA5093-OtA5097
Olfr1205	chr2:88831118-88832042	OtA5088-OtA5092	OtA5093-OtA5097
Olfr1206	chr2:88864606-88865530	OtA5088-OtA5092	OtA5093-OtA5097
Olfr1208	chr2:88896668-88897595	OtA5088-OtA5092	OtA5093-OtA5097
Olfr1209	chr2:88909458-88910391	OtA5088-OtA5092	OtA5093-OtA5097
Olfr121	chr17:37748865-37753004	OtA5088-OtA5092	OtA5093-OtA5097
Olfr1211	chr2:88929377-88930313	OtA5088-OtA5092	OtA5093-OtA5097
Olfr1212	chr2:88958467-88959403	OtA5088-OtA5092	OtA5093-OtA5097
Olfr1213	chr2:88972953-88980267	OtA5088-OtA5092	OtA5093-OtA5097
Olfr1214	chr2:88987264-88988200	OtA5088-OtA5092	OtA5093-OtA5097
Olfr1215	chr2:89001347-89002290	OtA5088-OtA5092	OtA5093-OtA5097
Olfr1216	chr2:89013126-89014062	OtA5088-OtA5092	OtA5093-OtA5097
Olfr1217	chr2:89022994-89024099	OtA5088-OtA5092	OtA5093-OtA5097
Olfr1218	chr2:89054488-89055424	OtA5088-OtA5092	OtA5093-OtA5097
Olfr1219	chr2:89074153-89075089	OtA5088-OtA5092	OtA5093-OtA5097

Olfr122	chr17:37768641-37772620	OtA5088-OtA5092	OtA5093-OtA5097
Olfr1220	chr2:89096937-89097963	OtA5088-OtA5092	OtA5093-OtA5097
Olfr1221	chr2:89111574-89112510	OtA5088-OtA5092	OtA5093-OtA5097
Olfr1222	chr2:89124793-89125729	OtA5088-OtA5092	OtA5093-OtA5097
Olfr1223	chr2:89144085-89151336	OtA5088-OtA5092	OtA5093-OtA5097
Olfr1225	chr2:89170218-89171245	OtA5088-OtA5092	OtA5093-OtA5097
Olfr1226	chr2:89193099-89194032	OtA5088-OtA5092	OtA5093-OtA5097
Olfr1228	chr2:89248720-89249692	OtA5088-OtA5092	OtA5093-OtA5097
Olfr1229	chr2:89282195-89283131	OtA5088-OtA5092	OtA5093-OtA5097
Olfr123	chr17:37795445-37796375	OtA5088-OtA5092	OtA5093-OtA5097
Olfr1230	chr2:89296350-89297268	OtA5088-OtA5092	OtA5093-OtA5097
Olfr1231	chr2:89302648-89303590	OtA5088-OtA5092	OtA5093-OtA5097
Olfr1232	chr2:89325242-89326178	OtA5088-OtA5092	OtA5093-OtA5097
Olfr1233	chr2:89339382-89340300	OtA5088-OtA5092	OtA5093-OtA5097
Olfr1234	chr2:89362482-89363427	OtA5088-OtA5092	OtA5093-OtA5097
Olfr1238	chr2:89406129-89407077	OtA5088-OtA5092	OtA5093-OtA5097
Olfr1239	chr2:89417493-89418411	OtA5088-OtA5092	OtA5093-OtA5097
Olfr124	chr17:37805068-37806190	OtA5088-OtA5092	OtA5093-OtA5097
Olfr1240	chr2:89439269-89440343	OtA5088-OtA5092	OtA5093-OtA5097
Olfr1241	chr2:89482188-89483133	OtA5088-OtA5092	OtA5093-OtA5097
Olfr1242	chr2:89493293-89494346	OtA5088-OtA5092	OtA5093-OtA5097
Olfr1243	chr2:89527490-89528408	OtA5088-OtA5092	OtA5093-OtA5097
Olfr1245	chr2:89574711-89575773	OtA5088-OtA5092	OtA5093-OtA5097
Olfr1246	chr2:89590068-89591146	OtA5088-OtA5092	OtA5093-OtA5097
Olfr1247	chr2:89609088-89610153	OtA5088-OtA5092	OtA5093-OtA5097
Olfr1248	chr2:89617167-89618245	OtA5088-OtA5092	OtA5093-OtA5097
Olfr1249	chr2:89629939-89630896	OtA5088-OtA5092	OtA5093-OtA5097
Olfr125	chr17:37834910-37836028	OtA5088-OtA5092	OtA5093-OtA5097
Olfr1250	chr2:89656494-89657439	OtA5088-OtA5092	OtA5093-OtA5097
Olfr1251	chr2:89666927-89667884	OtA5088-OtA5092	OtA5093-OtA5097
Olfr1252	chr2:89721164-89722109	OtA5088-OtA5092	OtA5093-OtA5097
Olfr1253	chr2:89751869-89752826	OtA5088-OtA5092	OtA5093-OtA5097
Olfr1254	chr2:89788405-89789350	OtA5088-OtA5092	OtA5093-OtA5097
Olfr1255	chr2:89816327-89817260	OtA5088-OtA5092	OtA5093-OtA5097
Olfr1256	chr2:89835022-89835943	OtA5088-OtA5092	OtA5093-OtA5097
Olfr1257	chr2:89880827-89881757	OtA5088-OtA5092	OtA5093-OtA5097
Olfr1258	chr2:89929810-89930746	OtA5088-OtA5092	OtA5093-OtA5097
Olfr1259	chr2:89943183-89944113	OtA5088-OtA5092	OtA5093-OtA5097
Olfr126	chr17:37850593-37851553	OtA5088-OtA5092	OtA5093-OtA5097
Olfr1260	chr2:89977779-89978712	OtA5088-OtA5092	OtA5093-OtA5097
Olfr1261	chr2:89993394-89994315	OtA5088-OtA5092	OtA5093-OtA5097
Olfr1262	chr2:90002407-90003322	OtA5088-OtA5092	OtA5093-OtA5097
Olfr1263	chr2:90014931-90015852	OtA5088-OtA5092	OtA5093-OtA5097
Olfr1264	chr2:90021137-90022064	OtA5088-OtA5092	OtA5093-OtA5097
Olfr1265	chr2:90036920-90037850	OtA5088-OtA5092	OtA5093-OtA5097
Olfr1269	chr2:90118666-90119596	OtA5088-OtA5092	OtA5093-OtA5097
Olfr127	chr17:37903547-37904519	OtA5088-OtA5092	OtA5093-OtA5097

Olfr1270	chr2:90149018-90150036	OtA5088-OtA5092	OtA5093-OtA5097
Olfr1271	chr2:90265510-90266428	OtA5088-OtA5092	OtA5093-OtA5097
Olfr1272	chr2:90281646-90282573	OtA5088-OtA5092	OtA5093-OtA5097
Olfr1273-ps	chr2:90295935-90296859	OtA5088-OtA5092	OtA5093-OtA5097
Olfr1274-ps	chr2:90400639-90401674	OtA5088-OtA5092	OtA5093-OtA5097
Olfr1275	chr2:111230852-111231791	OtA5088-OtA5092	OtA5093-OtA5097
Olfr1276	chr2:111257116-111258055	OtA5088-OtA5092	OtA5093-OtA5097
Olfr1277	chr2:111269453-111270365	OtA5088-OtA5092	OtA5093-OtA5097
Olfr1278	chr2:111292269-111293211	OtA5088-OtA5092	OtA5093-OtA5097
Olfr1279	chr2:111306200-111307142	OtA5088-OtA5092	OtA5093-OtA5097
Olfr128	chr17:37923567-37924494	OtA5088-OtA5092	OtA5093-OtA5097
Olfr1280	chr2:111315480-111316398	OtA5088-OtA5092	OtA5093-OtA5097
Olfr1281	chr2:111328420-111329338	OtA5088-OtA5092	OtA5093-OtA5097
Olfr1282	chr2:111335158-111336076	OtA5088-OtA5092	OtA5093-OtA5097
Olfr1283	chr2:111368633-111369551	OtA5088-OtA5092	OtA5093-OtA5097
Olfr1284	chr2:111379001-111379937	OtA5088-OtA5092	OtA5093-OtA5097
Olfr1286	chr2:111420031-111420949	OtA5088-OtA5092	OtA5093-OtA5097
Olfr1287	chr2:111449141-111450059	OtA5088-OtA5092	OtA5093-OtA5097
Olfr1288	chr2:111478785-111479724	OtA5088-OtA5092	OtA5093-OtA5097
Olfr1289	chr2:111483431-111484328	OtA5088-OtA5092	OtA5093-OtA5097
Olfr129	chr17:38054598-38059784	OtA5088-OtA5092	OtA5093-OtA5097
Olfr1290	chr2:111489217-111490156	OtA5088-OtA5092	OtA5093-OtA5097
Olfr1294	chr2:111537348-111538287	OtA5088-OtA5092	OtA5093-OtA5097
Olfr1295	chr2:111564503-111565442	OtA5088-OtA5092	OtA5093-OtA5097
Olfr1297	chr2:111621133-111622072	OtA5088-OtA5092	OtA5093-OtA5097
Olfr1298	chr2:111645056-111645995	OtA5088-OtA5092	OtA5093-OtA5097
Olfr1299	chr2:111661452-111665166	OtA5088-OtA5092	OtA5093-OtA5097
Olfr13	chr6:43173987-43174920	OtA5088-OtA5092	OtA5093-OtA5097
Olfr130	chr17:38067172-38068126	OtA5088-OtA5092	OtA5093-OtA5097
Olfr1300-ps1	chr2:111667436-111693319	OtA5088-OtA5092	OtA5093-OtA5097
Olfr1301	chr2:111754250-111755189	OtA5088-OtA5092	OtA5093-OtA5097
Olfr1302	chr2:111780312-111781260	OtA5088-OtA5092	OtA5093-OtA5097
Olfr1303	chr2:111813785-111814724	OtA5088-OtA5092	OtA5093-OtA5097
Olfr1305	chr2:111872914-111873853	OtA5088-OtA5092	OtA5093-OtA5097
Olfr1306	chr2:111911989-111912928	OtA5088-OtA5092	OtA5093-OtA5097
Olfr1307	chr2:111944515-111945454	OtA5088-OtA5092	OtA5093-OtA5097
Olfr1308	chr2:111960108-111961071	OtA5088-OtA5092	OtA5093-OtA5097
Olfr1309	chr2:111983133-111984096	OtA5088-OtA5092	OtA5093-OtA5097
Olfr131	chr17:38082031-38082976	OtA5088-OtA5092	OtA5093-OtA5097
Olfr1310	chr2:112008245-112009184	OtA5088-OtA5092	OtA5093-OtA5097
Olfr1311	chr2:112020913-112021852	OtA5088-OtA5092	OtA5093-OtA5097
Olfr1312	chr2:112042076-112043030	OtA5088-OtA5092	OtA5093-OtA5097
Olfr1313	chr2:112071645-112072581	OtA5088-OtA5092	OtA5093-OtA5097
Olfr1314	chr2:112091760-112092699	OtA5088-OtA5092	OtA5093-OtA5097
Olfr1316	chr2:112129870-112130815	OtA5088-OtA5092	OtA5093-OtA5097
Olfr1317	chr2:112141946-112142897	OtA5088-OtA5092	OtA5093-OtA5097
Olfr1318	chr2:112155897-112157007	OtA5088-OtA5092	OtA5093-OtA5097

Olfr132	chr17:38130248-38131190	OtA5088-OtA5092	OtA5093-OtA5097
Olfr1320	chrX:49683503-49684463	OtA5088-OtA5092	OtA5093-OtA5097
Olfr1321	chrX:49726972-49727932	OtA5088-OtA5092	OtA5093-OtA5097
Olfr1322	chrX:49885468-49886401	OtA5088-OtA5092	OtA5093-OtA5097
Olfr1323	chrX:50009305-50010235	OtA5088-OtA5092	OtA5093-OtA5097
Olfr1324	chrX:50425495-50426494	OtA5088-OtA5092	OtA5093-OtA5097
Olfr1325	chrX:74594327-74595275	OtA5088-OtA5092	OtA5093-OtA5097
Olfr1328	chr4:118933898-118934840	OtA5088-OtA5092	OtA5093-OtA5097
Olfr1329	chr4:118916523-118917465	OtA5088-OtA5092	OtA5093-OtA5097
Olfr133	chr17:38148589-38149528	OtA5088-OtA5092	OtA5093-OtA5097
Olfr1330	chr4:118893084-118894032	OtA5088-OtA5092	OtA5093-OtA5097
Olfr1331	chr4:118868782-118869736	OtA5088-OtA5092	OtA5093-OtA5097
Olfr1333	chr4:118829484-118830438	OtA5088-OtA5092	OtA5093-OtA5097
Olfr1335	chr4:118808854-118809862	OtA5088-OtA5092	OtA5093-OtA5097
Olfr1336	chr7:6460510-6461455	OtA5088-OtA5092	OtA5093-OtA5097
Olfr1337	chr4:118781635-118782583	OtA5088-OtA5092	OtA5093-OtA5097
Olfr1338	chr4:118753594-118754536	OtA5088-OtA5092	OtA5093-OtA5097
Olfr1339	chr4:118734530-118735478	OtA5088-OtA5092	OtA5093-OtA5097
Olfr134	chr17:38175085-38176024	OtA5088-OtA5092	OtA5093-OtA5097
Olfr1340	chr4:118726248-118727196	OtA5088-OtA5092	OtA5093-OtA5097
Olfr1341	chr4:118709408-118710347	OtA5088-OtA5092	OtA5093-OtA5097
Olfr1342	chr4:118689502-118690450	OtA5088-OtA5092	OtA5093-OtA5097
Olfr1344	chr7:6439901-6440867	OtA5088-OtA5092	OtA5093-OtA5097
Olfr1346	chr7:6474111-6475053	OtA5088-OtA5092	OtA5093-OtA5097
Olfr1347	chr7:6487912-6488872	OtA5088-OtA5092	OtA5093-OtA5097
Olfr1348	chr7:6501285-6502224	OtA5088-OtA5092	OtA5093-OtA5097
Olfr1349	chr7:6514473-6515427	OtA5088-OtA5092	OtA5093-OtA5097
Olfr135	chr17:38208246-38209185	OtA5088-OtA5092	OtA5093-OtA5097
Olfr1350	chr7:6569992-6570919	OtA5088-OtA5092	OtA5093-OtA5097
Olfr1351	chr10:79017323-79018283	OtA5088-OtA5092	OtA5093-OtA5097
Olfr1352	chr10:78981049-78984721	OtA5088-OtA5092	OtA5093-OtA5097
Olfr1353	chr10:78963308-78970612	OtA5088-OtA5092	OtA5093-OtA5097
Olfr1354	chr10:78916841-78917936	OtA5088-OtA5092	OtA5093-OtA5097
Olfr1355	chr10:78875548-78880106	OtA5088-OtA5092	OtA5093-OtA5097
Olfr1356	chr10:78846950-78847913	OtA5088-OtA5092	OtA5093-OtA5097
Olfr1357	chr10:78611668-78618074	OtA5088-OtA5092	OtA5093-OtA5097
Olfr1359	chr13:21703002-21703944	OtA5088-OtA5092	OtA5093-OtA5097
Olfr136	chr17:38335158-38336097	OtA5088-OtA5092	OtA5093-OtA5097
Olfr1360	chr13:21674000-21674980	OtA5088-OtA5092	OtA5093-OtA5097
Olfr1361	chr13:21658367-21659321	OtA5088-OtA5092	OtA5093-OtA5097
Olfr1362	chr13:21611019-21612004	OtA5088-OtA5092	OtA5093-OtA5097
Olfr1364	chr13:21573509-21574454	OtA5088-OtA5092	OtA5093-OtA5097
Olfr1366	chr13:21536973-21538027	OtA5088-OtA5092	OtA5093-OtA5097
Olfr1367	chr13:21346929-21347880	OtA5088-OtA5092	OtA5093-OtA5097
Olfr1368	chr13:21142110-21143055	OtA5088-OtA5092	OtA5093-OtA5097
Olfr137	chr17:38304520-38305459	OtA5088-OtA5092	OtA5093-OtA5097
Olfr1370	chr13:21072348-21073299	OtA5088-OtA5092	OtA5093-OtA5097

Olfr1371	chr11:52213051-52213987	OtA5088-OtA5092	OtA5093-OtA5097
Olfr1372-ps1	chr11:52154875-52157907	OtA5088-OtA5092	OtA5093-OtA5097
Olfr1373	chr11:52144592-52145528	OtA5088-OtA5092	OtA5093-OtA5097
Olfr1377	chr11:50984702-50985626	OtA5088-OtA5092	OtA5093-OtA5097
Olfr1378	chr11:50969019-50969967	OtA5088-OtA5092	OtA5093-OtA5097
Olfr138	chr17:38274772-38275711	OtA5088-OtA5092	OtA5093-OtA5097
Olfr1380	chr11:49563922-49564858	OtA5088-OtA5092	OtA5093-OtA5097
Olfr1381	chr11:49551748-49552684	OtA5088-OtA5092	OtA5093-OtA5097
Olfr1382	chr11:49535186-49536122	OtA5088-OtA5092	OtA5093-OtA5097
Olfr1383	chr11:49523724-49524660	OtA5088-OtA5092	OtA5093-OtA5097
Olfr1384	chr11:49513617-49514651	OtA5088-OtA5092	OtA5093-OtA5097
Olfr1385	chr11:49494534-49495464	OtA5088-OtA5092	OtA5093-OtA5097
Olfr1386	chr11:49470053-49471181	OtA5088-OtA5092	OtA5093-OtA5097
Olfr1387	chr11:49459680-49460616	OtA5088-OtA5092	OtA5093-OtA5097
Olfr1388	chr11:49443852-49444788	OtA5088-OtA5092	OtA5093-OtA5097
Olfr1389	chr11:49430426-49431499	OtA5088-OtA5092	OtA5093-OtA5097
Olfr139	chr11:74044324-74045272	OtA5088-OtA5092	OtA5093-OtA5097
Olfr1390	chr11:49340533-49341469	OtA5088-OtA5092	OtA5093-OtA5097
Olfr1391	chr11:49327412-49328348	OtA5088-OtA5092	OtA5093-OtA5097
Olfr1392	chr11:49293296-49294322	OtA5088-OtA5092	OtA5093-OtA5097
Olfr1393	chr11:49280149-49281085	OtA5088-OtA5092	OtA5093-OtA5097
Olfr1394	chr11:49160015-49160954	OtA5088-OtA5092	OtA5093-OtA5097
Olfr1395	chr11:49148258-49149212	OtA5088-OtA5092	OtA5093-OtA5097
Olfr1396	chr11:49037659-49135387	OtA5088-OtA5092	OtA5093-OtA5097
Olfr140	chr2:90051413-90052322	OtA5088-OtA5092	OtA5093-OtA5097
Olfr1402	chr3:97410216-97411179	OtA5088-OtA5092	OtA5093-OtA5097
Olfr1404	chr1:173215606-173216640	OtA5088-OtA5092	OtA5093-OtA5097
Olfr1406	chr1:173183441-173184504	OtA5088-OtA5092	OtA5093-OtA5097
Olfr1408	chr1:173130282-173131215	OtA5088-OtA5092	OtA5093-OtA5097
Olfr141	chr2:86806020-86806997	OtA5088-OtA5092	OtA5093-OtA5097
Olfr1410	chr1:92607838-92608807	OtA5088-OtA5092	OtA5093-OtA5097
Olfr1411	chr1:92596520-92597492	OtA5088-OtA5092	OtA5093-OtA5097
Olfr1412	chr1:92588331-92589297	OtA5088-OtA5092	OtA5093-OtA5097
Olfr1413	chr1:92573124-92574206	OtA5088-OtA5092	OtA5093-OtA5097
Olfr1414	chr1:92511047-92518515	OtA5088-OtA5092	OtA5093-OtA5097
Olfr1415	chr1:92490817-92491753	OtA5088-OtA5092	OtA5093-OtA5097
Olfr1416	chr1:92479680-92480619	OtA5088-OtA5092	OtA5093-OtA5097
Olfr1417	chr19:11828076-11829024	OtA5088-OtA5092	OtA5093-OtA5097
Olfr1418	chr19:11854994-11855951	OtA5088-OtA5092	OtA5093-OtA5097
Olfr1419	chr19:11870182-11871214	OtA5088-OtA5092	OtA5093-OtA5097
Olfr142	chr2:90252068-90252986	OtA5088-OtA5092	OtA5093-OtA5097
Olfr1420	chr19:11896022-11896952	OtA5088-OtA5092	OtA5093-OtA5097
Olfr1423	chr19:12035807-12036740	OtA5088-OtA5092	OtA5093-OtA5097
Olfr1424	chr19:12058808-12059750	OtA5088-OtA5092	OtA5093-OtA5097
Olfr1425	chr19:12071789-12413554	OtA5088-OtA5092	OtA5093-OtA5097
Olfr1426	chr19:12071789-12413554	OtA5088-OtA5092	OtA5093-OtA5097
Olfr1427	chr19:12071789-12413554	OtA5088-OtA5092	OtA5093-OtA5097

Olfr1428	chr19:12071789-12413554	OtA5088-OtA5092	OtA5093-OtA5097
Olfr143	chr9:38253418-38254360	OtA5088-OtA5092	OtA5093-OtA5097
Olfr1431	chr19:12071789-12413554	OtA5088-OtA5092	OtA5093-OtA5097
Olfr1433,Olfr1434	chr19:12071789-12413554	OtA5088-OtA5092	OtA5093-OtA5097
Olfr1434	chr19:12071789-12413554	OtA5088-OtA5092	OtA5093-OtA5097
Olfr1436	chr19:12071789-12413554	OtA5088-OtA5092	OtA5093-OtA5097
Olfr1437	chr19:12071789-12413554	OtA5088-OtA5092	OtA5093-OtA5097
Olfr1440	chr19:12071789-12413554	OtA5088-OtA5092	OtA5093-OtA5097
Olfr1441	chr19:12422310-12423267	OtA5088-OtA5092	OtA5093-OtA5097
Olfr1442	chr19:12674178-12675243	OtA5088-OtA5092	OtA5093-OtA5097
Olfr1443	chr19:12678165-12683845	OtA5088-OtA5092	OtA5093-OtA5097
Olfr1444	chr19:12861776-12862736	OtA5088-OtA5092	OtA5093-OtA5097
Olfr1445	chr19:12883882-12884827	OtA5088-OtA5092	OtA5093-OtA5097
Olfr1446	chr19:12889648-12890575	OtA5088-OtA5092	OtA5093-OtA5097
Olfr1447	chr19:12900848-12901778	OtA5088-OtA5092	OtA5093-OtA5097
Olfr1448	chr19:12919362-12920307	OtA5088-OtA5092	OtA5093-OtA5097
Olfr1449	chr19:12934739-12935684	OtA5088-OtA5092	OtA5093-OtA5097
Olfr145	chr9:37897405-37898338	OtA5088-OtA5092	OtA5093-OtA5097
Olfr1450	chr19:12953590-12954568	OtA5088-OtA5092	OtA5093-OtA5097
Olfr1451	chr19:12998987-12999920	OtA5088-OtA5092	OtA5093-OtA5097
Olfr1453	chr19:13027403-13028327	OtA5088-OtA5092	OtA5093-OtA5097
Olfr1454	chr19:13063412-13064336	OtA5088-OtA5092	OtA5093-OtA5097
Olfr1457	chr19:13094692-13095646	OtA5088-OtA5092	OtA5093-OtA5097
Olfr1459	chr19:13145733-13146657	OtA5088-OtA5092	OtA5093-OtA5097
Olfr146	chr9:39018618-39019539	OtA5088-OtA5092	OtA5093-OtA5097
Olfr1461	chr19:13165015-13165954	OtA5088-OtA5092	OtA5093-OtA5097
Olfr1462	chr19:13190668-13191592	OtA5088-OtA5092	OtA5093-OtA5097
Olfr1463	chr19:13234251-13235184	OtA5088-OtA5092	OtA5093-OtA5097
Olfr1465	chr19:13313359-13314283	OtA5088-OtA5092	OtA5093-OtA5097
Olfr1466	chr19:13341759-13342692	OtA5088-OtA5092	OtA5093-OtA5097
Olfr1467	chr19:13364629-13365556	OtA5088-OtA5092	OtA5093-OtA5097
Olfr1469	chr19:13410570-13411500	OtA5088-OtA5092	OtA5093-OtA5097
Olfr147	chr9:38401710-38403829	OtA5088-OtA5092	OtA5093-OtA5097
Olfr1471	chr19:13445013-13445958	OtA5088-OtA5092	OtA5093-OtA5097
Olfr1472	chr19:13453570-13454515	OtA5088-OtA5092	OtA5093-OtA5097
Olfr1474	chr19:13470971-13471916	OtA5088-OtA5092	OtA5093-OtA5097
Olfr1475	chr19:13479251-13480196	OtA5088-OtA5092	OtA5093-OtA5097
Olfr1477	chr19:13500545-13503292	OtA5088-OtA5092	OtA5093-OtA5097
Olfr148	chr9:39613568-39614501	OtA5088-OtA5092	OtA5093-OtA5097
Olfr1480	chr19:13529674-13530622	OtA5088-OtA5092	OtA5093-OtA5097
Olfr1484	chr19:13585305-13586253	OtA5088-OtA5092	OtA5093-OtA5097
Olfr1487	chr19:13619163-13620111	OtA5088-OtA5092	OtA5093-OtA5097
Olfr1489	chr19:13633112-13634054	OtA5088-OtA5092	OtA5093-OtA5097
Olfr149	chr9:39701831-39702767	OtA5088-OtA5092	OtA5093-OtA5097
Olfr1490	chr19:13654445-13655396	OtA5088-OtA5092	OtA5093-OtA5097
Olfr1491	chr19:13704828-13705788	OtA5088-OtA5092	OtA5093-OtA5097
Olfr1494	chr19:13749107-13750055	OtA5088-OtA5092	OtA5093-OtA5097

Olfr1495	chr19:13768343-13769303	OtA5088-OtA5092	OtA5093-OtA5097
Olfr1496	chr19:13780547-13781614	OtA5088-OtA5092	OtA5093-OtA5097
Olfr1497	chr19:13794664-13795609	OtA5088-OtA5092	OtA5093-OtA5097
Olfr1499	chr19:13814643-13815588	OtA5088-OtA5092	OtA5093-OtA5097
Olfr15	chr16:3838974-3839913	OtA5088-OtA5092	OtA5093-OtA5097
Olfr150	chr9:39736816-39737755	OtA5088-OtA5092	OtA5093-OtA5097
Olfr1500	chr19:13827458-13828394	OtA5088-OtA5092	OtA5093-OtA5097
Olfr1501	chr19:13838223-13839171	OtA5088-OtA5092	OtA5093-OtA5097
Olfr1502	chr19:13861794-13862745	OtA5088-OtA5092	OtA5093-OtA5097
Olfr1504	chr19:13887260-13888208	OtA5088-OtA5092	OtA5093-OtA5097
Olfr1505	chr19:13919021-13919972	OtA5088-OtA5092	OtA5093-OtA5097
Olfr1506	chr2:90322221-90325126	OtA5088-OtA5092	OtA5093-OtA5097
Olfr1507	chr14:52489934-52495695	OtA5088-OtA5092	OtA5093-OtA5097
Olfr1508	chr14:52462720-52467461	OtA5088-OtA5092	OtA5093-OtA5097
Olfr1509	chr14:52450392-52451402	OtA5088-OtA5092	OtA5093-OtA5097
Olfr151	chr9:37729976-37731061	OtA5088-OtA5092	OtA5093-OtA5097
Olfr1510	chr14:52409888-52410927	OtA5088-OtA5092	OtA5093-OtA5097
Olfr1511	chr14:52389814-52390771	OtA5088-OtA5092	OtA5093-OtA5097
Olfr1512	chr14:52372109-52373051	OtA5088-OtA5092	OtA5093-OtA5097
Olfr1513	chr14:52349102-52350044	OtA5088-OtA5092	OtA5093-OtA5097
Olfr152	chr2:87782535-87783486	OtA5088-OtA5092	OtA5093-OtA5097
Olfr153	chr2:87532034-87532958	OtA5088-OtA5092	OtA5093-OtA5097
Olfr1532-ps1	chr7:106914199-106915123	OtA5088-OtA5092	OtA5093-OtA5097
Olfr1535	chr13:21555039-21556020	OtA5088-OtA5092	OtA5093-OtA5097
Olfr1537-ps1	chr9:39237486-39238431	OtA5088-OtA5092	OtA5093-OtA5097
Olfr154	chr2:85663376-85664455	OtA5088-OtA5092	OtA5093-OtA5097
Olfr155	chr4:43854291-43855463	OtA5088-OtA5092	OtA5093-OtA5097
Olfr156	chr4:43820334-43821433	OtA5088-OtA5092	OtA5093-OtA5097
Olfr157	chr4:43834751-43836517	OtA5088-OtA5092	OtA5093-OtA5097
Olfr159	chr4:43770049-43771009	OtA5088-OtA5092	OtA5093-OtA5097
Olfr16	chr1:172956767-172957817	OtA5088-OtA5092	OtA5093-OtA5097
Olfr160	chr9:37711347-37712280	OtA5088-OtA5092	OtA5093-OtA5097
Olfr161	chr16:3592397-3593339	OtA5088-OtA5092	OtA5093-OtA5097
Olfr164	chr16:19285793-19286741	OtA5088-OtA5092	OtA5093-OtA5097
Olfr165	chr16:19407075-19408017	OtA5088-OtA5092	OtA5093-OtA5097
Olfr166	chr16:19486839-19487778	OtA5088-OtA5092	OtA5093-OtA5097
Olfr167	chr16:19514695-19515634	OtA5088-OtA5092	OtA5093-OtA5097
Olfr168	chr16:19529979-19530918	OtA5088-OtA5092	OtA5093-OtA5097
Olfr169	chr16:19565939-19566881	OtA5088-OtA5092	OtA5093-OtA5097
Olfr17	chr7:107097466-107098414	OtA5088-OtA5092	OtA5093-OtA5097
Olfr170	chr16:19605724-19606666	OtA5088-OtA5092	OtA5093-OtA5097
Olfr171	chr16:19624156-19625101	OtA5088-OtA5092	OtA5093-OtA5097
Olfr172	chr16:58760152-58761240	OtA5088-OtA5092	OtA5093-OtA5097
Olfr173	chr16:58796778-58797942	OtA5088-OtA5092	OtA5093-OtA5097
Olfr175-ps1	chr16:58823780-58826761	OtA5088-OtA5092	OtA5093-OtA5097
Olfr176	chr16:58872214-58873148	OtA5088-OtA5092	OtA5093-OtA5097
Olfr177	chr16:58872214-58873148	OtA5088-OtA5092	OtA5093-OtA5097

Olfr178	chr16:58889258-58890218	OtA5088-OtA5092	OtA5093-OtA5097
Olfr179	chr11:58665560-58666544	OtA5088-OtA5092	OtA5093-OtA5097
Olfr18	chr9:20313891-20336094	OtA5088-OtA5092	OtA5093-OtA5097
Olfr180	chr16:58915685-58918486	OtA5088-OtA5092	OtA5093-OtA5097
Olfr181	chr16:58925556-58928644	OtA5088-OtA5092	OtA5093-OtA5097
Olfr183	chr16:58995403-59000616	OtA5088-OtA5092	OtA5093-OtA5097
Olfr186	chr16:59026975-59027905	OtA5088-OtA5092	OtA5093-OtA5097
Olfr187	chr16:59035779-59039749	OtA5088-OtA5092	OtA5093-OtA5097
Olfr19	chr16:16673049-16673979	OtA5088-OtA5092	OtA5093-OtA5097
Olfr190	chr16:59074154-59075078	OtA5088-OtA5092	OtA5093-OtA5097
Olfr191	chr16:59085551-59086481	OtA5088-OtA5092	OtA5093-OtA5097
Olfr192	chr16:59098065-59098990	OtA5088-OtA5092	OtA5093-OtA5097
Olfr193	chr16:59109678-59110608	OtA5088-OtA5092	OtA5093-OtA5097
Olfr194	chr16:59119147-59120068	OtA5088-OtA5092	OtA5093-OtA5097
Olfr195	chr16:59148851-59149778	OtA5088-OtA5092	OtA5093-OtA5097
Olfr196	chr16:59167211-59168141	OtA5088-OtA5092	OtA5093-OtA5097
Olfr197	chr16:59185555-59186481	OtA5088-OtA5092	OtA5093-OtA5097
Olfr198	chr16:59201503-59202424	OtA5088-OtA5092	OtA5093-OtA5097
Olfr199	chr16:59215684-59216611	OtA5088-OtA5092	OtA5093-OtA5097
Olfr2	chr7:107000874-107002605	OtA5088-OtA5092	OtA5093-OtA5097
Olfr20	chr11:73350858-73354699	OtA5088-OtA5092	OtA5093-OtA5097
Olfr201	chr16:59268738-59269665	OtA5088-OtA5092	OtA5093-OtA5097
Olfr202	chr16:59283571-59284495	OtA5088-OtA5092	OtA5093-OtA5097
Olfr203	chr16:59303154-59304075	OtA5088-OtA5092	OtA5093-OtA5097
Olfr204	chr16:59314487-59315405	OtA5088-OtA5092	OtA5093-OtA5097
Olfr205	chr16:59328589-59329507	OtA5088-OtA5092	OtA5093-OtA5097
Olfr206	chr16:59344778-59345699	OtA5088-OtA5092	OtA5093-OtA5097
Olfr208	chr16:59361298-59362216	OtA5088-OtA5092	OtA5093-OtA5097
Olfr211	chr6:116493610-116494540	OtA5088-OtA5092	OtA5093-OtA5097
Olfr212	chr6:116506515-116517965	OtA5088-OtA5092	OtA5093-OtA5097
Olfr213	chr6:116540454-116541438	OtA5088-OtA5092	OtA5093-OtA5097
Olfr214	chr6:116556426-116557389	OtA5088-OtA5092	OtA5093-OtA5097
Olfr215	chr6:116582011-116582944	OtA5088-OtA5092	OtA5093-OtA5097
Olfr218	chr1:173203357-173204299	OtA5088-OtA5092	OtA5093-OtA5097
Olfr220	chr1:174448624-174449602	OtA5088-OtA5092	OtA5093-OtA5097
Olfr221	chr14:52035173-52036109	OtA5088-OtA5092	OtA5093-OtA5097
Olfr222	chr11:59570781-59571738	OtA5088-OtA5092	OtA5093-OtA5097
Olfr223	chr11:59589127-59590087	OtA5088-OtA5092	OtA5093-OtA5097
Olfr224	chr11:58566398-58567343	OtA5088-OtA5092	OtA5093-OtA5097
Olfr225	chr11:59612965-59614225	OtA5088-OtA5092	OtA5093-OtA5097
Olfr228	chr2:86482798-86483740	OtA5088-OtA5092	OtA5093-OtA5097
Olfr229	chr9:39909804-39910728	OtA5088-OtA5092	OtA5093-OtA5097
Olfr23	chr11:73940247-73941225	OtA5088-OtA5092	OtA5093-OtA5097
Olfr230-ps1	chr1:174117090-174118047	OtA5088-OtA5092	OtA5093-OtA5097
Olfr231	chr1:174117090-174118047	OtA5088-OtA5092	OtA5093-OtA5097
Olfr235	chr19:12071789-12413554	OtA5088-OtA5092	OtA5093-OtA5097
Olfr237-ps1	chr6:43153306-43154239	OtA5088-OtA5092	OtA5093-OtA5097

Olfr239	chr17:33199061-33200009	OtA5088-OtA5092	OtA5093-OtA5097
Olfr24	chr9:18754691-18755633	OtA5088-OtA5092	OtA5093-OtA5097
Olfr242	chr9:39128166-39145070	OtA5088-OtA5092	OtA5093-OtA5097
Olfr243	chr7:103716595-103717546	OtA5088-OtA5092	OtA5093-OtA5097
Olfr247	chr10:129983479-129984424	OtA5088-OtA5092	OtA5093-OtA5097
Olfr248	chr1:174391045-174392055	OtA5088-OtA5092	OtA5093-OtA5097
Olfr25	chr9:38329491-38330559	OtA5088-OtA5092	OtA5093-OtA5097
Olfr250	chr9:38367547-38368543	OtA5088-OtA5092	OtA5093-OtA5097
Olfr251	chr9:38377882-38378833	OtA5088-OtA5092	OtA5093-OtA5097
Olfr259	chr2:87107446-87108385	OtA5088-OtA5092	OtA5093-OtA5097
Olfr26	chr9:38855059-38855990	OtA5088-OtA5092	OtA5093-OtA5097
Olfr262	chr19:12071789-12413554	OtA5088-OtA5092	OtA5093-OtA5097
Olfr263	chr13:21132776-21133730	OtA5088-OtA5092	OtA5093-OtA5097
Olfr266	chr3:106821606-106822557	OtA5088-OtA5092	OtA5093-OtA5097
Olfr267	chr4:58784778-58785720	OtA5088-OtA5092	OtA5093-OtA5097
Olfr27	chr9:39128166-39145070	OtA5088-OtA5092	OtA5093-OtA5097
Olfr270	chr4:52970628-52971567	OtA5088-OtA5092	OtA5093-OtA5097
Olfr272	chr4:52910832-52911792	OtA5088-OtA5092	OtA5093-OtA5097
Olfr273	chr4:52855557-52856511	OtA5088-OtA5092	OtA5093-OtA5097
Olfr275	chr4:52814603-52828269	OtA5088-OtA5092	OtA5093-OtA5097
Olfr279	chr15:98497473-98498406	OtA5088-OtA5092	OtA5093-OtA5097
Olfr281	chr15:98456311-98457247	OtA5088-OtA5092	OtA5093-OtA5097
Olfr282	chr15:98437469-98438397	OtA5088-OtA5092	OtA5093-OtA5097
Olfr283	chr15:98378178-98379108	OtA5088-OtA5092	OtA5093-OtA5097
Olfr284	chr15:98340021-98340939	OtA5088-OtA5092	OtA5093-OtA5097
Olfr285	chr15:98312588-98313548	OtA5088-OtA5092	OtA5093-OtA5097
Olfr286	chr15:98226674-98234450	OtA5088-OtA5092	OtA5093-OtA5097
Olfr287	chr15:98206970-98221056	OtA5088-OtA5092	OtA5093-OtA5097
Olfr288	chr15:98186012-98195542	OtA5088-OtA5092	OtA5093-OtA5097
Olfr29-ps1	chr4:43781364-43782327	OtA5088-OtA5092	OtA5093-OtA5097
Olfr290	chr7:84915780-84916728	OtA5088-OtA5092	OtA5093-OtA5097
Olfr291	chr7:84853570-84857318	OtA5088-OtA5092	OtA5093-OtA5097
Olfr292	chr7:86688340-86695384	OtA5088-OtA5092	OtA5093-OtA5097
Olfr293	chr7:86663663-86664674	OtA5088-OtA5092	OtA5093-OtA5097
Olfr294	chr7:86615635-86616643	OtA5088-OtA5092	OtA5093-OtA5097
Olfr295	chr7:86585276-86586206	OtA5088-OtA5092	OtA5093-OtA5097
Olfr297	chr7:86526758-86527691	OtA5088-OtA5092	OtA5093-OtA5097
Olfr298	chr7:86488550-86489549	OtA5088-OtA5092	OtA5093-OtA5097
Olfr299	chr7:86465412-86466405	OtA5088-OtA5092	OtA5093-OtA5097
Olfr3	chr2:36812148-36813090	OtA5088-OtA5092	OtA5093-OtA5097
Olfr30	chr11:58454914-58455980	OtA5088-OtA5092	OtA5093-OtA5097
Olfr301	chr7:86403848-86413299	OtA5088-OtA5092	OtA5093-OtA5097
Olfr303	chr7:86394536-86395496	OtA5088-OtA5092	OtA5093-OtA5097
Olfr304	chr7:86385656-86386658	OtA5088-OtA5092	OtA5093-OtA5097
Olfr305	chr7:86363375-86364335	OtA5088-OtA5092	OtA5093-OtA5097
Olfr307	chr7:86335452-86336394	OtA5088-OtA5092	OtA5093-OtA5097
Olfr308	chr7:86321023-86321950	OtA5088-OtA5092	OtA5093-OtA5097

Olfr309	chr7:86306184-86307111	OtA5088-OtA5092	OtA5093-OtA5097
Olfr31	chr14:14328112-14329066	OtA5088-OtA5092	OtA5093-OtA5097
Olfr310	chr7:86268729-86269811	OtA5088-OtA5092	OtA5093-OtA5097
Olfr311	chr11:58841115-58842042	OtA5088-OtA5092	OtA5093-OtA5097
Olfr312	chr11:58831155-58832082	OtA5088-OtA5092	OtA5093-OtA5097
Olfr313	chr11:58816927-58818001	OtA5088-OtA5092	OtA5093-OtA5097
Olfr314	chr11:58786138-58787269	OtA5088-OtA5092	OtA5093-OtA5097
Olfr315	chr11:58778088-58779086	OtA5088-OtA5092	OtA5093-OtA5097
Olfr316	chr11:58757666-58758587	OtA5088-OtA5092	OtA5093-OtA5097
Olfr317	chr11:58732095-58733223	OtA5088-OtA5092	OtA5093-OtA5097
Olfr318	chr11:58720068-58721094	OtA5088-OtA5092	OtA5093-OtA5097
Olfr319	chr11:58701702-58702623	OtA5088-OtA5092	OtA5093-OtA5097
Olfr32	chr2:90138179-90142293	OtA5088-OtA5092	OtA5093-OtA5097
Olfr320	chr11:58683874-58684795	OtA5088-OtA5092	OtA5093-OtA5097
Olfr323	chr11:58625072-58626044	OtA5088-OtA5092	OtA5093-OtA5097
Olfr324	chr11:58597344-58598466	OtA5088-OtA5092	OtA5093-OtA5097
Olfr325	chr11:58580836-58581877	OtA5088-OtA5092	OtA5093-OtA5097
Olfr328	chr11:58551304-58552237	OtA5088-OtA5092	OtA5093-OtA5097
Olfr329-ps	chr11:58542446-58544938	OtA5088-OtA5092	OtA5093-OtA5097
Olfr33	chr7:102713454-102714411	OtA5088-OtA5092	OtA5093-OtA5097
Olfr330	chr11:58528928-58534825	OtA5088-OtA5092	OtA5093-OtA5097
Olfr331	chr11:58501597-58502554	OtA5088-OtA5092	OtA5093-OtA5097
Olfr332	chr11:58489718-58492499	OtA5088-OtA5092	OtA5093-OtA5097
Olfr338	chr2:36376777-36377698	OtA5088-OtA5092	OtA5093-OtA5097
Olfr339	chr2:36421399-36422329	OtA5088-OtA5092	OtA5093-OtA5097
Olfr340	chr2:36452586-36453525	OtA5088-OtA5092	OtA5093-OtA5097
Olfr341	chr2:36479186-36480128	OtA5088-OtA5092	OtA5093-OtA5097
Olfr342	chr2:36527413-36528352	OtA5088-OtA5092	OtA5093-OtA5097
Olfr344	chr2:36568599-36569529	OtA5088-OtA5092	OtA5093-OtA5097
Olfr345	chr2:36640040-36640976	OtA5088-OtA5092	OtA5093-OtA5097
Olfr346	chr2:36688003-36688933	OtA5088-OtA5092	OtA5093-OtA5097
Olfr347	chr2:36734322-36735261	OtA5088-OtA5092	OtA5093-OtA5097
Olfr348	chr2:36786526-36787468	OtA5088-OtA5092	OtA5093-OtA5097
Olfr350	chr2:36850047-36850986	OtA5088-OtA5092	OtA5093-OtA5097
Olfr351	chr2:36859413-36860346	OtA5088-OtA5092	OtA5093-OtA5097
Olfr352	chr2:36869567-36870515	OtA5088-OtA5092	OtA5093-OtA5097
Olfr353	chr2:36889910-36890846	OtA5088-OtA5092	OtA5093-OtA5097
Olfr354	chr2:36906947-36907901	OtA5088-OtA5092	OtA5093-OtA5097
Olfr355	chr2:36927179-36928112	OtA5088-OtA5092	OtA5093-OtA5097
Olfr356	chr2:36937120-36938068	OtA5088-OtA5092	OtA5093-OtA5097
Olfr357	chr2:36996811-36997738	OtA5088-OtA5092	OtA5093-OtA5097
Olfr358	chr2:37004619-37005612	OtA5088-OtA5092	OtA5093-OtA5097
Olfr360	chr2:37068306-37069260	OtA5088-OtA5092	OtA5093-OtA5097
Olfr361	chr2:37084777-37085746	OtA5088-OtA5092	OtA5093-OtA5097
Olfr362	chr2:37104694-37105648	OtA5088-OtA5092	OtA5093-OtA5097
Olfr365	chr2:37201242-37202181	OtA5088-OtA5092	OtA5093-OtA5097
Olfr366	chr2:37219490-37220420	OtA5088-OtA5092	OtA5093-OtA5097

Olfr367-ps	chr2:37266950-37271387	OtA5088-OtA5092	OtA5093-OtA5097
Olfr368	chr2:37331748-37332732	OtA5088-OtA5092	OtA5093-OtA5097
Olfr370	chr8:83541092-83542188	OtA5088-OtA5092	OtA5093-OtA5097
Olfr371	chr8:85230496-85231435	OtA5088-OtA5092	OtA5093-OtA5097
Olfr372	chr8:72057659-72058675	OtA5088-OtA5092	OtA5093-OtA5097
Olfr373	chr8:72099761-72100706	OtA5088-OtA5092	OtA5093-OtA5097
Olfr374	chr8:72107039-72110509	OtA5088-OtA5092	OtA5093-OtA5097
Olfr376	chr11:73371245-73375704	OtA5088-OtA5092	OtA5093-OtA5097
Olfr378	chr11:73425036-73428477	OtA5088-OtA5092	OtA5093-OtA5097
Olfr38	chr6:42762053-42763007	OtA5088-OtA5092	OtA5093-OtA5097
Olfr380	chr11:73453274-73454210	OtA5088-OtA5092	OtA5093-OtA5097
Olfr381	chr11:73485886-73486822	OtA5088-OtA5092	OtA5093-OtA5097
Olfr382	chr11:73516258-73517197	OtA5088-OtA5092	OtA5093-OtA5097
Olfr384	chr11:73602581-73603520	OtA5088-OtA5092	OtA5093-OtA5097
Olfr385	chr11:73588797-73589736	OtA5088-OtA5092	OtA5093-OtA5097
Olfr389	chr11:73776386-73780589	OtA5088-OtA5092	OtA5093-OtA5097
Olfr39	chr9:20282350-20286648	OtA5088-OtA5092	OtA5093-OtA5097
Olfr390	chr11:73786939-73787875	OtA5088-OtA5092	OtA5093-OtA5097
Olfr391-ps	chr11:73798752-73802984	OtA5088-OtA5092	OtA5093-OtA5097
Olfr392	chr11:73814141-73816877	OtA5088-OtA5092	OtA5093-OtA5097
Olfr393	chr11:73847184-73848123	OtA5088-OtA5092	OtA5093-OtA5097
Olfr394	chr11:73887437-73888370	OtA5088-OtA5092	OtA5093-OtA5097
Olfr395	chr11:73906551-73907490	OtA5088-OtA5092	OtA5093-OtA5097
Olfr397	chr11:73964609-73965557	OtA5088-OtA5092	OtA5093-OtA5097
Olfr398	chr11:73983661-73984606	OtA5088-OtA5092	OtA5093-OtA5097
Olfr399	chr11:74053775-74054778	OtA5088-OtA5092	OtA5093-OtA5097
Olfr401	chr11:74121290-74122238	OtA5088-OtA5092	OtA5093-OtA5097
Olfr402	chr11:74155155-74156103	OtA5088-OtA5092	OtA5093-OtA5097
Olfr403	chr11:74195504-74196446	OtA5088-OtA5092	OtA5093-OtA5097
Olfr406-ps	chr11:74269390-74270362	OtA5088-OtA5092	OtA5093-OtA5097
Olfr410	chr11:74334281-74335229	OtA5088-OtA5092	OtA5093-OtA5097
Olfr411	chr11:74346623-74347672	OtA5088-OtA5092	OtA5093-OtA5097
Olfr412	chr11:74364670-74365609	OtA5088-OtA5092	OtA5093-OtA5097
Olfr414	chr1:174430429-174431384	OtA5088-OtA5092	OtA5093-OtA5097
Olfr417	chr1:174368918-174369848	OtA5088-OtA5092	OtA5093-OtA5097
Olfr418-ps1	chr1:173270146-173271137	OtA5088-OtA5092	OtA5093-OtA5097
Olfr419	chr1:174249888-174250976	OtA5088-OtA5092	OtA5093-OtA5097
Olfr420	chr1:174158721-174159767	OtA5088-OtA5092	OtA5093-OtA5097
Olfr421-ps1	chr1:174151480-174152530	OtA5088-OtA5092	OtA5093-OtA5097
Olfr424	chr1:174136745-174137693	OtA5088-OtA5092	OtA5093-OtA5097
Olfr426	chr1:174099459-174100410	OtA5088-OtA5092	OtA5093-OtA5097
Olfr427	chr1:174099459-174100410	OtA5088-OtA5092	OtA5093-OtA5097
Olfr429	chr1:174089037-174089980	OtA5088-OtA5092	OtA5093-OtA5097
Olfr43	chr11:74206197-74207289	OtA5088-OtA5092	OtA5093-OtA5097
Olfr430	chr1:174069299-174070253	OtA5088-OtA5092	OtA5093-OtA5097
Olfr432	chr1:174050374-174051313	OtA5088-OtA5092	OtA5093-OtA5097
Olfr433	chr1:174041932-174042936	OtA5088-OtA5092	OtA5093-OtA5097

Olfr434	chr6:43216914-43217880	OtA5088-OtA5092	OtA5093-OtA5097
Olfr435	chr6:43201645-43202587	OtA5088-OtA5092	OtA5093-OtA5097
Olfr437	chr6:43167059-43167992	OtA5088-OtA5092	OtA5093-OtA5097
Olfr44	chr9:39484203-39493988	OtA5088-OtA5092	OtA5093-OtA5097
Olfr441	chr6:43115743-43116676	OtA5088-OtA5092	OtA5093-OtA5097
Olfr444	chr6:42955499-42956432	OtA5088-OtA5092	OtA5093-OtA5097
Olfr446	chr6:42927232-42928159	OtA5088-OtA5092	OtA5093-OtA5097
Olfr447	chr6:42911524-42912457	OtA5088-OtA5092	OtA5093-OtA5097
Olfr448	chr6:42896452-42897385	OtA5088-OtA5092	OtA5093-OtA5097
Olfr449	chr6:42837882-42838818	OtA5088-OtA5092	OtA5093-OtA5097
Olfr45	chr7:140690906-140691842	OtA5088-OtA5092	OtA5093-OtA5097
Olfr450	chr6:42817472-42818405	OtA5088-OtA5092	OtA5093-OtA5097
Olfr452	chr6:42790040-42790994	OtA5088-OtA5092	OtA5093-OtA5097
Olfr453	chr6:42744038-42744992	OtA5088-OtA5092	OtA5093-OtA5097
Olfr455	chr6:42538066-42539020	OtA5088-OtA5092	OtA5093-OtA5097
Olfr456	chr6:42486162-42487214	OtA5088-OtA5092	OtA5093-OtA5097
Olfr457	chr6:42471234-42472176	OtA5088-OtA5092	OtA5093-OtA5097
Olfr458	chr6:42460075-42461017	OtA5088-OtA5092	OtA5093-OtA5097
Olfr459	chr6:41771352-41772297	OtA5088-OtA5092	OtA5093-OtA5097
Olfr46	chr7:140601339-140611124	OtA5088-OtA5092	OtA5093-OtA5097
Olfr460	chr6:40529090-40910666	OtA5088-OtA5092	OtA5093-OtA5097
Olfr461	chr6:40529090-40910666	OtA5088-OtA5092	OtA5093-OtA5097
Olfr462	chr11:87888958-87889894	OtA5088-OtA5092	OtA5093-OtA5097
Olfr463	chr11:87892986-87893922	OtA5088-OtA5092	OtA5093-OtA5097
Olfr464	chr11:87913919-87915003	OtA5088-OtA5092	OtA5093-OtA5097
Olfr466	chr13:65152225-65153152	OtA5088-OtA5092	OtA5093-OtA5097
Olfr467	chr7:107814585-107815506	OtA5088-OtA5092	OtA5093-OtA5097
Olfr469	chr7:107822522-107823467	OtA5088-OtA5092	OtA5093-OtA5097
Olfr47	chr6:43235609-43236575	OtA5088-OtA5092	OtA5093-OtA5097
Olfr470	chr7:107844786-107845731	OtA5088-OtA5092	OtA5093-OtA5097
Olfr472	chr7:107902718-107903651	OtA5088-OtA5092	OtA5093-OtA5097
Olfr473	chr7:107933521-107934454	OtA5088-OtA5092	OtA5093-OtA5097
Olfr474	chr7:107954642-107955575	OtA5088-OtA5092	OtA5093-OtA5097
Olfr476	chr7:107967398-107968331	OtA5088-OtA5092	OtA5093-OtA5097
Olfr477	chr7:107990366-107991299	OtA5088-OtA5092	OtA5093-OtA5097
Olfr478	chr7:108031396-108032341	OtA5088-OtA5092	OtA5093-OtA5097
Olfr479	chr7:108054983-108055967	OtA5088-OtA5092	OtA5093-OtA5097
Olfr48	chr2:89844065-89844971	OtA5088-OtA5092	OtA5093-OtA5097
Olfr480	chr7:108065767-108066796	OtA5088-OtA5092	OtA5093-OtA5097
Olfr481	chr7:108080795-108081734	OtA5088-OtA5092	OtA5093-OtA5097
Olfr482	chr7:108094596-108095568	OtA5088-OtA5092	OtA5093-OtA5097
Olfr483	chr7:108103310-108104258	OtA5088-OtA5092	OtA5093-OtA5097
Olfr484	chr7:108124295-108125261	OtA5088-OtA5092	OtA5093-OtA5097
Olfr485	chr7:108158917-108159871	OtA5088-OtA5092	OtA5093-OtA5097
Olfr486	chr7:108171797-108172742	OtA5088-OtA5092	OtA5093-OtA5097
Olfr487	chr7:108211582-108212527	OtA5088-OtA5092	OtA5093-OtA5097
Olfr488	chr7:108255191-108256136	OtA5088-OtA5092	OtA5093-OtA5097

Olfr49	chr14:54281895-54282925	OtA5088-OtA5092	OtA5093-OtA5097
Olfr490	chr7:108286179-108287124	OtA5088-OtA5092	OtA5093-OtA5097
Olfr491	chr7:108316895-108317828	OtA5088-OtA5092	OtA5093-OtA5097
Olfr492	chr7:108322729-108323674	OtA5088-OtA5092	OtA5093-OtA5097
Olfr493	chr7:108346034-108346979	OtA5088-OtA5092	OtA5093-OtA5097
Olfr494	chr7:108367491-108368436	OtA5088-OtA5092	OtA5093-OtA5097
Olfr495	chr7:108395121-108396114	OtA5088-OtA5092	OtA5093-OtA5097
Olfr497	chr7:108422572-108423517	OtA5088-OtA5092	OtA5093-OtA5097
Olfr498	chr7:108465325-108466318	OtA5088-OtA5092	OtA5093-OtA5097
Olfr5	chr7:6480215-6486813	OtA5088-OtA5092	OtA5093-OtA5097
Olfr50	chr2:36793237-36794176	OtA5088-OtA5092	OtA5093-OtA5097
Olfr502	chr7:108523003-108523948	OtA5088-OtA5092	OtA5093-OtA5097
Olfr503	chr7:108544526-108545498	OtA5088-OtA5092	OtA5093-OtA5097
Olfr504	chr7:108564836-108565793	OtA5088-OtA5092	OtA5093-OtA5097
Olfr506	chr7:108612308-108613253	OtA5088-OtA5092	OtA5093-OtA5097
Olfr507	chr7:108621813-108622764	OtA5088-OtA5092	OtA5093-OtA5097
Olfr508	chr7:108629993-108630926	OtA5088-OtA5092	OtA5093-OtA5097
Olfr509	chr7:108645608-108646574	OtA5088-OtA5092	OtA5093-OtA5097
Olfr51	chr11:51006973-51007897	OtA5088-OtA5092	OtA5093-OtA5097
Olfr510	chr7:108667417-108668362	OtA5088-OtA5092	OtA5093-OtA5097
Olfr512	chr7:108713354-108714335	OtA5088-OtA5092	OtA5093-OtA5097
Olfr513	chr7:108754857-108755787	OtA5088-OtA5092	OtA5093-OtA5097
Olfr514	chr7:108825064-108825997	OtA5088-OtA5092	OtA5093-OtA5097
Olfr516	chr7:108845063-108846008	OtA5088-OtA5092	OtA5093-OtA5097
Olfr517	chr7:108868207-108869152	OtA5088-OtA5092	OtA5093-OtA5097
Olfr518	chr7:108880602-108881604	OtA5088-OtA5092	OtA5093-OtA5097
Olfr519	chr7:108893460-108894405	OtA5088-OtA5092	OtA5093-OtA5097
Olfr52	chr2:86181149-86182109	OtA5088-OtA5092	OtA5093-OtA5097
Olfr520	chr7:99735144-99736095	OtA5088-OtA5092	OtA5093-OtA5097
Olfr521	chr7:99767163-99768129	OtA5088-OtA5092	OtA5093-OtA5097
Olfr522	chr7:140162009-140162948	OtA5088-OtA5092	OtA5093-OtA5097
Olfr523	chr7:140176103-140177057	OtA5088-OtA5092	OtA5093-OtA5097
Olfr524	chr7:140201811-140202768	OtA5088-OtA5092	OtA5093-OtA5097
Olfr525	chr7:140322700-140323630	OtA5088-OtA5092	OtA5093-OtA5097
Olfr527	chr7:140335863-140336781	OtA5088-OtA5092	OtA5093-OtA5097
Olfr53	chr7:140646451-140652919	OtA5088-OtA5092	OtA5093-OtA5097
Olfr530	chr7:140372684-140373608	OtA5088-OtA5092	OtA5093-OtA5097
Olfr531	chr7:140400129-140401044	OtA5088-OtA5092	OtA5093-OtA5097
Olfr532	chr7:140418841-140419771	OtA5088-OtA5092	OtA5093-OtA5097
Olfr533	chr7:140466202-140467165	OtA5088-OtA5092	OtA5093-OtA5097
Olfr535	chr7:140492639-140493578	OtA5088-OtA5092	OtA5093-OtA5097
Olfr536	chr7:140500852-140507318	OtA5088-OtA5092	OtA5093-OtA5097
Olfr538	chr7:140574154-140575087	OtA5088-OtA5092	OtA5093-OtA5097
Olfr539	chr7:140667288-140668248	OtA5088-OtA5092	OtA5093-OtA5097
Olfr54	chr11:51027003-51027945	OtA5088-OtA5092	OtA5093-OtA5097
Olfr541	chr7:140704252-140705191	OtA5088-OtA5092	OtA5093-OtA5097
Olfr543	chr7:102476772-102477902	OtA5088-OtA5092	OtA5093-OtA5097

Olfr544	chr7:102484113-102488307	OtA5088-OtA5092	OtA5093-OtA5097
Olfr545	chr7:102493822-102494773	OtA5088-OtA5092	OtA5093-OtA5097
Olfr547	chr7:102534748-102535688	OtA5088-OtA5092	OtA5093-OtA5097
Olfr549	chr7:102554285-102555236	OtA5088-OtA5092	OtA5093-OtA5097
Olfr55	chr17:33176415-33177363	OtA5088-OtA5092	OtA5093-OtA5097
Olfr550	chr7:102578468-102579506	OtA5088-OtA5092	OtA5093-OtA5097
Olfr551	chr7:102587740-102588819	OtA5088-OtA5092	OtA5093-OtA5097
Olfr552	chr7:102604355-102605309	OtA5088-OtA5092	OtA5093-OtA5097
Olfr553	chr7:102614006-102614987	OtA5088-OtA5092	OtA5093-OtA5097
Olfr554	chr7:102640247-102641201	OtA5088-OtA5092	OtA5093-OtA5097
Olfr555	chr7:102658822-102659770	OtA5088-OtA5092	OtA5093-OtA5097
Olfr556	chr7:102669900-102670923	OtA5088-OtA5092	OtA5093-OtA5097
Olfr557	chr7:102698215-102699272	OtA5088-OtA5092	OtA5093-OtA5097
Olfr558	chr7:102702322-102712054	OtA5088-OtA5092	OtA5093-OtA5097
Olfr559	chr7:102723531-102724488	OtA5088-OtA5092	OtA5093-OtA5097
Olfr56	chr11:49037659-49135387	OtA5088-OtA5092	OtA5093-OtA5097
Olfr560	chr7:102740720-102759471	OtA5088-OtA5092	OtA5093-OtA5097
Olfr561	chr7:102774525-102775470	OtA5088-OtA5092	OtA5093-OtA5097
Olfr564	chr7:102803479-102804430	OtA5088-OtA5092	OtA5093-OtA5097
Olfr566	chr7:102856320-102857271	OtA5088-OtA5092	OtA5093-OtA5097
Olfr568	chr7:102877121-102878063	OtA5088-OtA5092	OtA5093-OtA5097
Olfr569	chr7:102887206-102888151	OtA5088-OtA5092	OtA5093-OtA5097
Olfr57	chr10:79034722-79035802	OtA5088-OtA5092	OtA5093-OtA5097
Olfr570	chr7:102900368-102901307	OtA5088-OtA5092	OtA5093-OtA5097
Olfr571	chr7:102908868-102909837	OtA5088-OtA5092	OtA5093-OtA5097
Olfr572	chr7:102927629-102928586	OtA5088-OtA5092	OtA5093-OtA5097
Olfr574	chr7:102948466-102949507	OtA5088-OtA5092	OtA5093-OtA5097
Olfr575	chr7:102954663-102955620	OtA5088-OtA5092	OtA5093-OtA5097
Olfr576	chr7:102965101-102966040	OtA5088-OtA5092	OtA5093-OtA5097
Olfr577	chr7:102973051-102973990	OtA5088-OtA5092	OtA5093-OtA5097
Olfr578	chr7:102984220-102985162	OtA5088-OtA5092	OtA5093-OtA5097
Olfr58	chr9:19780282-19784064	OtA5088-OtA5092	OtA5093-OtA5097
Olfr582	chr7:103041480-103042440	OtA5088-OtA5092	OtA5093-OtA5097
Olfr583	chr7:103051299-103052259	OtA5088-OtA5092	OtA5093-OtA5097
Olfr584	chr7:103085519-103086479	OtA5088-OtA5092	OtA5093-OtA5097
Olfr585	chr7:103097742-103098699	OtA5088-OtA5092	OtA5093-OtA5097
Olfr586	chr7:103121828-103122782	OtA5088-OtA5092	OtA5093-OtA5097
Olfr589	chr7:103154791-103155745	OtA5088-OtA5092	OtA5093-OtA5097
Olfr59	chr11:74283808-74289646	OtA5088-OtA5092	OtA5093-OtA5097
Olfr591	chr7:103172690-103173635	OtA5088-OtA5092	OtA5093-OtA5097
Olfr592	chr7:103186602-103187541	OtA5088-OtA5092	OtA5093-OtA5097
Olfr593	chr7:103211861-103212845	OtA5088-OtA5092	OtA5093-OtA5097
Olfr594	chr7:103219719-103220655	OtA5088-OtA5092	OtA5093-OtA5097
Olfr596	chr7:103309722-103310661	OtA5088-OtA5092	OtA5093-OtA5097
Olfr597	chr7:103320412-103321360	OtA5088-OtA5092	OtA5093-OtA5097
Olfr598	chr7:103328487-103329447	OtA5088-OtA5092	OtA5093-OtA5097
Olfr599	chr7:103338055-103339003	OtA5088-OtA5092	OtA5093-OtA5097

Olfr6	chr7:106955983-106956934	OtA5088-OtA5092	OtA5093-OtA5097
Olfr60	chr7:140345051-140345987	OtA5088-OtA5092	OtA5093-OtA5097
Olfr600	chr7:103345981-103346926	OtA5088-OtA5092	OtA5093-OtA5097
Olfr601	chr7:103358223-103359213	OtA5088-OtA5092	OtA5093-OtA5097
Olfr603	chr7:103383061-103384000	OtA5088-OtA5092	OtA5093-OtA5097
Olfr605	chr7:103442139-103443150	OtA5088-OtA5092	OtA5093-OtA5097
Olfr606	chr7:103451338-103452298	OtA5088-OtA5092	OtA5093-OtA5097
Olfr608	chr7:103470040-103470991	OtA5088-OtA5092	OtA5093-OtA5097
Olfr609	chr7:103491916-103492876	OtA5088-OtA5092	OtA5093-OtA5097
Olfr61	chr7:140637702-140638638	OtA5088-OtA5092	OtA5093-OtA5097
Olfr610	chr7:103505996-103506944	OtA5088-OtA5092	OtA5093-OtA5097
Olfr611	chr7:103517410-103518382	OtA5088-OtA5092	OtA5093-OtA5097
Olfr612	chr7:103538260-103539232	OtA5088-OtA5092	OtA5093-OtA5097
Olfr613	chr7:103550367-103555504	OtA5088-OtA5092	OtA5093-OtA5097
Olfr615	chr7:103560478-103561420	OtA5088-OtA5092	OtA5093-OtA5097
Olfr616	chr7:103564323-103565277	OtA5088-OtA5092	OtA5093-OtA5097
Olfr617	chr7:103584023-103584980	OtA5088-OtA5092	OtA5093-OtA5097
Olfr618	chr7:103597317-103598274	OtA5088-OtA5092	OtA5093-OtA5097
Olfr619	chr7:103603588-103604711	OtA5088-OtA5092	OtA5093-OtA5097
Olfr62	chr4:118665518-118666466	OtA5088-OtA5092	OtA5093-OtA5097
Olfr620	chr7:103611409-103612351	OtA5088-OtA5092	OtA5093-OtA5097
Olfr622	chr7:103639184-103640138	OtA5088-OtA5092	OtA5093-OtA5097
Olfr623	chr7:103660243-103661318	OtA5088-OtA5092	OtA5093-OtA5097
Olfr624	chr7:103670102-103671029	OtA5088-OtA5092	OtA5093-OtA5097
Olfr628	chr7:103731927-103732878	OtA5088-OtA5092	OtA5093-OtA5097
Olfr629	chr7:103740200-103741322	OtA5088-OtA5092	OtA5093-OtA5097
Olfr63	chr17:33268725-33269676	OtA5088-OtA5092	OtA5093-OtA5097
Olfr630	chr7:103754149-103757350	OtA5088-OtA5092	OtA5093-OtA5097
Olfr631	chr7:103915061-103929911	OtA5088-OtA5092	OtA5093-OtA5097
Olfr632	chr7:103937381-103938335	OtA5088-OtA5092	OtA5093-OtA5097
Olfr633	chr7:103946567-103947506	OtA5088-OtA5092	OtA5093-OtA5097
Olfr635	chr7:103979175-103980141	OtA5088-OtA5092	OtA5093-OtA5097
Olfr638	chr7:104003258-104004224	OtA5088-OtA5092	OtA5093-OtA5097
Olfr639	chr7:104011749-104012700	OtA5088-OtA5092	OtA5093-OtA5097
Olfr64	chr7:103892809-103894471	OtA5088-OtA5092	OtA5093-OtA5097
Olfr640	chr7:104021371-104022316	OtA5088-OtA5092	OtA5093-OtA5097
Olfr641	chr7:104039797-104040736	OtA5088-OtA5092	OtA5093-OtA5097
Olfr642	chr7:104049407-104050352	OtA5088-OtA5092	OtA5093-OtA5097
Olfr643	chr7:104058655-104059600	OtA5088-OtA5092	OtA5093-OtA5097
Olfr644	chr7:104068084-104069029	OtA5088-OtA5092	OtA5093-OtA5097
Olfr645	chr7:104084130-104085078	OtA5088-OtA5092	OtA5093-OtA5097
Olfr646	chr7:104106280-104107219	OtA5088-OtA5092	OtA5093-OtA5097
Olfr648	chr7:104179455-104180406	OtA5088-OtA5092	OtA5093-OtA5097
Olfr649	chr7:104189266-104190205	OtA5088-OtA5092	OtA5093-OtA5097
Olfr65	chr7:103906341-103907447	OtA5088-OtA5092	OtA5093-OtA5097
Olfr651	chr7:104552883-104553952	OtA5088-OtA5092	OtA5093-OtA5097
Olfr652	chr7:104564151-104565212	OtA5088-OtA5092	OtA5093-OtA5097

Olfr653	chr7:104579623-104580669	OtA5088-OtA5092	OtA5093-OtA5097
Olfr654	chr7:104587754-104588780	OtA5088-OtA5092	OtA5093-OtA5097
Olfr655	chr7:104596252-104597179	OtA5088-OtA5092	OtA5093-OtA5097
Olfr656	chr7:104617656-104618646	OtA5088-OtA5092	OtA5093-OtA5097
Olfr657	chr7:104635675-104636635	OtA5088-OtA5092	OtA5093-OtA5097
Olfr658	chr7:104642879-104647305	OtA5088-OtA5092	OtA5093-OtA5097
Olfr659	chr7:104670703-104671672	OtA5088-OtA5092	OtA5093-OtA5097
Olfr66	chr7:103881305-103882241	OtA5088-OtA5092	OtA5093-OtA5097
Olfr661	chr7:104688016-104688976	OtA5088-OtA5092	OtA5093-OtA5097
Olfr663	chr7:104703568-104704603	OtA5088-OtA5092	OtA5093-OtA5097
Olfr665	chr7:104880708-104881659	OtA5088-OtA5092	OtA5093-OtA5097
Olfr666	chr7:104892669-104893626	OtA5088-OtA5092	OtA5093-OtA5097
Olfr667	chr7:104916313-104917294	OtA5088-OtA5092	OtA5093-OtA5097
Olfr668	chr7:104924805-104925762	OtA5088-OtA5092	OtA5093-OtA5097
Olfr669	chr7:104938527-104939481	OtA5088-OtA5092	OtA5093-OtA5097
Olfr67	chr7:103787211-103791830	OtA5088-OtA5092	OtA5093-OtA5097
Olfr670	chr7:104959791-104960730	OtA5088-OtA5092	OtA5093-OtA5097
Olfr671	chr7:104975041-104975983	OtA5088-OtA5092	OtA5093-OtA5097
Olfr672	chr7:104995963-104996902	OtA5088-OtA5092	OtA5093-OtA5097
Olfr675	chr7:105024024-105024966	OtA5088-OtA5092	OtA5093-OtA5097
Olfr676	chr7:105035199-105036153	OtA5088-OtA5092	OtA5093-OtA5097
Olfr677	chr7:105056247-105057186	OtA5088-OtA5092	OtA5093-OtA5097
Olfr678	chr7:105069468-105070410	OtA5088-OtA5092	OtA5093-OtA5097
Olfr679	chr7:105085717-105086665	OtA5088-OtA5092	OtA5093-OtA5097
Olfr68	chr7:103777395-103778343	OtA5088-OtA5092	OtA5093-OtA5097
Olfr681	chr7:105121458-105122406	OtA5088-OtA5092	OtA5093-OtA5097
Olfr683	chr7:105143349-105144309	OtA5088-OtA5092	OtA5093-OtA5097
Olfr684	chr7:105156741-105157680	OtA5088-OtA5092	OtA5093-OtA5097
Olfr685	chr7:105180360-105181311	OtA5088-OtA5092	OtA5093-OtA5097
Olfr686	chr7:105203387-105204341	OtA5088-OtA5092	OtA5093-OtA5097
Olfr688	chr7:105288094-105289060	OtA5088-OtA5092	OtA5093-OtA5097
Olfr689	chr7:105314005-105314968	OtA5088-OtA5092	OtA5093-OtA5097
Olfr69	chr7:103767276-103771594	OtA5088-OtA5092	OtA5093-OtA5097
Olfr690	chr7:105329216-105330278	OtA5088-OtA5092	OtA5093-OtA5097
Olfr691	chr7:105336745-105337714	OtA5088-OtA5092	OtA5093-OtA5097
Olfr692	chr7:105368327-105369317	OtA5088-OtA5092	OtA5093-OtA5097
Olfr693	chr7:106677533-106678485	OtA5088-OtA5092	OtA5093-OtA5097
Olfr694	chr7:106688781-106689729	OtA5088-OtA5092	OtA5093-OtA5097
Olfr695	chr7:106713731-106716345	OtA5088-OtA5092	OtA5093-OtA5097
Olfr697	chr7:106740907-106741956	OtA5088-OtA5092	OtA5093-OtA5097
Olfr698	chr7:106752450-106753386	OtA5088-OtA5092	OtA5093-OtA5097
Olfr699	chr7:106790048-106790999	OtA5088-OtA5092	OtA5093-OtA5097
Olfr70	chr4:43694999-43700807	OtA5088-OtA5092	OtA5093-OtA5097
Olfr700	chr7:106805509-106806460	OtA5088-OtA5092	OtA5093-OtA5097
Olfr701	chr7:106814143-106820709	OtA5088-OtA5092	OtA5093-OtA5097
Olfr702	chr7:106823488-106826743	OtA5088-OtA5092	OtA5093-OtA5097
Olfr703	chr7:106844612-106845572	OtA5088-OtA5092	OtA5093-OtA5097

Olfr704	chr7:106864981-106865929	OtA5088-OtA5092	OtA5093-OtA5097
Olfr705	chr7:106873292-106874243	OtA5088-OtA5092	OtA5093-OtA5097
Olfr706	chr7:106885864-106886815	OtA5088-OtA5092	OtA5093-OtA5097
Olfr707	chr7:106891183-106892107	OtA5088-OtA5092	OtA5093-OtA5097
Olfr71	chr4:43705627-43706566	OtA5088-OtA5092	OtA5093-OtA5097
Olfr710	chr7:106944066-106948312	OtA5088-OtA5092	OtA5093-OtA5097
Olfr711	chr7:106971367-106975451	OtA5088-OtA5092	OtA5093-OtA5097
Olfr713	chr7:107036135-107037110	OtA5088-OtA5092	OtA5093-OtA5097
Olfr714	chr7:107073829-107074783	OtA5088-OtA5092	OtA5093-OtA5097
Olfr715	chr7:107128351-107129482	OtA5088-OtA5092	OtA5093-OtA5097
Olfr716	chr7:107147317-107148262	OtA5088-OtA5092	OtA5093-OtA5097
Olfr720	chr14:14175129-14176080	OtA5088-OtA5092	OtA5093-OtA5097
Olfr722	chr14:49894257-49901941	OtA5088-OtA5092	OtA5093-OtA5097
Olfr723	chr14:49928562-49929568	OtA5088-OtA5092	OtA5093-OtA5097
Olfr724	chr14:49960090-49961096	OtA5088-OtA5092	OtA5093-OtA5097
Olfr725	chr14:50034390-50035500	OtA5088-OtA5092	OtA5093-OtA5097
Olfr726	chr14:50083713-50084679	OtA5088-OtA5092	OtA5093-OtA5097
Olfr727	chr14:50126552-50127587	OtA5088-OtA5092	OtA5093-OtA5097
Olfr728	chr14:50139701-50140637	OtA5088-OtA5092	OtA5093-OtA5097
Olfr729	chr14:50147900-50148872	OtA5088-OtA5092	OtA5093-OtA5097
Olfr73	chr2:88034195-88035137	OtA5088-OtA5092	OtA5093-OtA5097
Olfr730	chr14:50186261-50187218	OtA5088-OtA5092	OtA5093-OtA5097
Olfr731	chr14:50237902-50238883	OtA5088-OtA5092	OtA5093-OtA5097
Olfr732	chr14:50281227-50282271	OtA5088-OtA5092	OtA5093-OtA5097
Olfr733	chr14:50298336-50299330	OtA5088-OtA5092	OtA5093-OtA5097
Olfr734	chr14:50319891-50320833	OtA5088-OtA5092	OtA5093-OtA5097
Olfr735	chr14:50345375-50346440	OtA5088-OtA5092	OtA5093-OtA5097
Olfr736	chr14:50392757-50393696	OtA5088-OtA5092	OtA5093-OtA5097
Olfr738	chr14:50413545-50414481	OtA5088-OtA5092	OtA5093-OtA5097
Olfr739	chr14:50424520-50425450	OtA5088-OtA5092	OtA5093-OtA5097
Olfr74	chr2:87973706-87974663	OtA5088-OtA5092	OtA5093-OtA5097
Olfr740	chr14:50453053-50453989	OtA5088-OtA5092	OtA5093-OtA5097
Olfr741	chr14:50473056-50486395	OtA5088-OtA5092	OtA5093-OtA5097
Olfr742	chr14:50503768-50520943	OtA5088-OtA5092	OtA5093-OtA5097
Olfr743	chr14:50533413-50534349	OtA5088-OtA5092	OtA5093-OtA5097
Olfr744	chr14:50618223-50619195	OtA5088-OtA5092	OtA5093-OtA5097
Olfr745	chr14:50642194-50643369	OtA5088-OtA5092	OtA5093-OtA5097
Olfr746	chr14:50653238-50654183	OtA5088-OtA5092	OtA5093-OtA5097
Olfr747	chr14:50680690-50681645	OtA5088-OtA5092	OtA5093-OtA5097
Olfr748	chr14:50710331-50711255	OtA5088-OtA5092	OtA5093-OtA5097
Olfr749	chr14:50736218-50744324	OtA5088-OtA5092	OtA5093-OtA5097
Olfr75-ps1	chr11:73405449-73409858	OtA5088-OtA5092	OtA5093-OtA5097
Olfr750	chr14:51070310-51071442	OtA5088-OtA5092	OtA5093-OtA5097
Olfr76	chr19:12071789-12413554	OtA5088-OtA5092	OtA5093-OtA5097
Olfr761	chr17:37952050-37953022	OtA5088-OtA5092	OtA5093-OtA5097
Olfr763	chr10:129011286-129012216	OtA5088-OtA5092	OtA5093-OtA5097
Olfr765	chr10:129046131-129047061	OtA5088-OtA5092	OtA5093-OtA5097

Olfr767	chr10:129079031-129079961	OtA5088-OtA5092	OtA5093-OtA5097
Olfr768	chr10:129093033-129093972	OtA5088-OtA5092	OtA5093-OtA5097
Olfr769	chr10:129111484-129112423	OtA5088-OtA5092	OtA5093-OtA5097
Olfr77	chr9:19917228-19922510	OtA5088-OtA5092	OtA5093-OtA5097
Olfr770	chr10:129132830-129133766	OtA5088-OtA5092	OtA5093-OtA5097
Olfr771	chr10:129160028-129160982	OtA5088-OtA5092	OtA5093-OtA5097
Olfr772	chr10:129174050-129175054	OtA5088-OtA5092	OtA5093-OtA5097
Olfr773	chr10:129186483-129187419	OtA5088-OtA5092	OtA5093-OtA5097
Olfr774	chr10:129238150-129239089	OtA5088-OtA5092	OtA5093-OtA5097
Olfr775	chr10:129250535-129251474	OtA5088-OtA5092	OtA5093-OtA5097
Olfr776	chr10:129260962-129261901	OtA5088-OtA5092	OtA5093-OtA5097
Olfr777	chr10:129268385-129269321	OtA5088-OtA5092	OtA5093-OtA5097
Olfr78	chr7:102740720-102759471	OtA5088-OtA5092	OtA5093-OtA5097
Olfr780	chr10:129321624-129322563	OtA5088-OtA5092	OtA5093-OtA5097
Olfr781	chr10:129332882-129333818	OtA5088-OtA5092	OtA5093-OtA5097
Olfr782	chr10:129350564-129351509	OtA5088-OtA5092	OtA5093-OtA5097
Olfr784	chr10:129387634-129388594	OtA5088-OtA5092	OtA5093-OtA5097
Olfr786	chr10:129436813-129437752	OtA5088-OtA5092	OtA5093-OtA5097
Olfr787	chr10:129462677-129463616	OtA5088-OtA5092	OtA5093-OtA5097
Olfr788	chr10:129472693-129473629	OtA5088-OtA5092	OtA5093-OtA5097
Olfr790	chr10:129500885-129501854	OtA5088-OtA5092	OtA5093-OtA5097
Olfr791	chr10:129526228-129527167	OtA5088-OtA5092	OtA5093-OtA5097
Olfr792	chr10:129540538-129541474	OtA5088-OtA5092	OtA5093-OtA5097
Olfr794	chr10:129570656-129571619	OtA5088-OtA5092	OtA5093-OtA5097
Olfr796	chr10:129607546-129608479	OtA5088-OtA5092	OtA5093-OtA5097
Olfr798	chr10:129625123-129626059	OtA5088-OtA5092	OtA5093-OtA5097
Olfr799	chr10:129647129-129648065	OtA5088-OtA5092	OtA5093-OtA5097
Olfr8	chr10:78955206-78956139	OtA5088-OtA5092	OtA5093-OtA5097
Olfr800	chr10:129659807-129660743	OtA5088-OtA5092	OtA5093-OtA5097
Olfr801	chr10:129669560-129670517	OtA5088-OtA5092	OtA5093-OtA5097
Olfr802	chr10:129681798-129682737	OtA5088-OtA5092	OtA5093-OtA5097
Olfr803	chr10:129691112-129692039	OtA5088-OtA5092	OtA5093-OtA5097
Olfr804	chr10:129704879-129705824	OtA5088-OtA5092	OtA5093-OtA5097
Olfr805	chr10:129722582-129723542	OtA5088-OtA5092	OtA5093-OtA5097
Olfr806	chr10:129737973-129738915	OtA5088-OtA5092	OtA5093-OtA5097
Olfr807	chr10:129754512-129755448	OtA5088-OtA5092	OtA5093-OtA5097
Olfr808	chr10:129767497-129768436	OtA5088-OtA5092	OtA5093-OtA5097
Olfr809	chr10:129775870-129776857	OtA5088-OtA5092	OtA5093-OtA5097
Olfr810	chr10:129790648-129791587	OtA5088-OtA5092	OtA5093-OtA5097
Olfr811	chr10:129801563-129802523	OtA5088-OtA5092	OtA5093-OtA5097
Olfr812	chr10:129842107-129843040	OtA5088-OtA5092	OtA5093-OtA5097
Olfr813	chr10:129856519-129857452	OtA5088-OtA5092	OtA5093-OtA5097
Olfr814	chr10:129873822-129874755	OtA5088-OtA5092	OtA5093-OtA5097
Olfr815	chr10:129901775-129902726	OtA5088-OtA5092	OtA5093-OtA5097
Olfr816	chr10:129911337-129912276	OtA5088-OtA5092	OtA5093-OtA5097
Olfr818	chr10:129945103-129946060	OtA5088-OtA5092	OtA5093-OtA5097
Olfr819	chr10:129965735-129966698	OtA5088-OtA5092	OtA5093-OtA5097

Olfr820	chr10:130017362-130018301	OtA5088-OtA5092	OtA5093-OtA5097
Olfr821	chr10:130033627-130034560	OtA5088-OtA5092	OtA5093-OtA5097
Olfr822	chr10:130074411-130075350	OtA5088-OtA5092	OtA5093-OtA5097
Olfr823	chr10:130111840-130112788	OtA5088-OtA5092	OtA5093-OtA5097
Olfr824	chr10:130126107-130127055	OtA5088-OtA5092	OtA5093-OtA5097
Olfr825	chr10:130162361-130163324	OtA5088-OtA5092	OtA5093-OtA5097
Olfr826	chr10:130179936-130180878	OtA5088-OtA5092	OtA5093-OtA5097
Olfr827	chr10:130210159-130211128	OtA5088-OtA5092	OtA5093-OtA5097
Olfr828	chr9:18815353-18816292	OtA5088-OtA5092	OtA5093-OtA5097
Olfr829	chr9:18856626-18857592	OtA5088-OtA5092	OtA5093-OtA5097
Olfr830	chr9:18875328-18876276	OtA5088-OtA5092	OtA5093-OtA5097
Olfr832	chr9:18944649-18945588	OtA5088-OtA5092	OtA5093-OtA5097
Olfr834	chr9:18987989-18988928	OtA5088-OtA5092	OtA5093-OtA5097
Olfr835	chr9:19035124-19036060	OtA5088-OtA5092	OtA5093-OtA5097
Olfr836	chr9:19120956-19121911	OtA5088-OtA5092	OtA5093-OtA5097
Olfr837	chr9:19136927-19137967	OtA5088-OtA5092	OtA5093-OtA5097
Olfr843	chr9:19248356-19249463	OtA5088-OtA5092	OtA5093-OtA5097
Olfr845	chr9:19338458-19339400	OtA5088-OtA5092	OtA5093-OtA5097
Olfr846	chr9:19360414-19361356	OtA5088-OtA5092	OtA5093-OtA5097
Olfr847	chr9:19374940-19375879	OtA5088-OtA5092	OtA5093-OtA5097
Olfr849	chr9:19440914-19441853	OtA5088-OtA5092	OtA5093-OtA5097
Olfr850	chr9:19477300-19478248	OtA5088-OtA5092	OtA5093-OtA5097
Olfr851	chr9:19496749-19497688	OtA5088-OtA5092	OtA5093-OtA5097
Olfr853	chr9:19537010-19537928	OtA5088-OtA5092	OtA5093-OtA5097
Olfr854	chr9:19566434-19567382	OtA5088-OtA5092	OtA5093-OtA5097
Olfr855	chr9:19584510-19585502	OtA5088-OtA5092	OtA5093-OtA5097
Olfr856-ps1	chr9:19657038-19658680	OtA5088-OtA5092	OtA5093-OtA5097
Olfr857	chr9:19712828-19713758	OtA5088-OtA5092	OtA5093-OtA5097
Olfr859	chr9:19808319-19809249	OtA5088-OtA5092	OtA5093-OtA5097
Olfr860	chr9:19845622-19849747	OtA5088-OtA5092	OtA5093-OtA5097
Olfr862	chr9:19883382-19884303	OtA5088-OtA5092	OtA5093-OtA5097
Olfr866	chr9:20026958-20028035	OtA5088-OtA5092	OtA5093-OtA5097
Olfr867	chr9:20054465-20055461	OtA5088-OtA5092	OtA5093-OtA5097
Olfr868	chr9:20098625-20101690	OtA5088-OtA5092	OtA5093-OtA5097
Olfr869	chr9:20129119-20138089	OtA5088-OtA5092	OtA5093-OtA5097
Olfr870	chr9:20170633-20171569	OtA5088-OtA5092	OtA5093-OtA5097
Olfr871	chr9:20212206-20213353	OtA5088-OtA5092	OtA5093-OtA5097
Olfr872	chr9:20237161-20260913	OtA5088-OtA5092	OtA5093-OtA5097
Olfr873	chr9:20300210-20301170	OtA5088-OtA5092	OtA5093-OtA5097
Olfr874	chr9:37746135-37747068	OtA5088-OtA5092	OtA5093-OtA5097
Olfr875	chr9:37772636-37773640	OtA5088-OtA5092	OtA5093-OtA5097
Olfr876	chr9:37803888-37804936	OtA5088-OtA5092	OtA5093-OtA5097
Olfr877	chr9:37854819-37855755	OtA5088-OtA5092	OtA5093-OtA5097
Olfr878	chr9:37918547-37919612	OtA5088-OtA5092	OtA5093-OtA5097
Olfr881	chr9:37992491-37993439	OtA5088-OtA5092	OtA5093-OtA5097
Olfr883	chr9:38025807-38026737	OtA5088-OtA5092	OtA5093-OtA5097
Olfr884	chr9:38047223-38048153	OtA5088-OtA5092	OtA5093-OtA5097

Olfr885	chr9:38061321-38062251	OtA5088-OtA5092	OtA5093-OtA5097
Olfr887	chr9:38084837-38085767	OtA5088-OtA5092	OtA5093-OtA5097
Olfr888	chr9:38108687-38109632	OtA5088-OtA5092	OtA5093-OtA5097
Olfr889	chr9:38115797-38116727	OtA5088-OtA5092	OtA5093-OtA5097
Olfr890	chr9:38143136-38144081	OtA5088-OtA5092	OtA5093-OtA5097
Olfr891	chr9:38179870-38180821	OtA5088-OtA5092	OtA5093-OtA5097
Olfr893	chr9:38209051-38209993	OtA5088-OtA5092	OtA5093-OtA5097
Olfr894	chr9:38218817-38219766	OtA5088-OtA5092	OtA5093-OtA5097
Olfr895	chr9:38268514-38269465	OtA5088-OtA5092	OtA5093-OtA5097
Olfr898	chr9:38349084-38350035	OtA5088-OtA5092	OtA5093-OtA5097
Olfr9	chr10:128989913-128990852	OtA5088-OtA5092	OtA5093-OtA5097
Olfr90	chr17:37085190-37086236	OtA5088-OtA5092	OtA5093-OtA5097
Olfr901	chr9:38430283-38431219	OtA5088-OtA5092	OtA5093-OtA5097
Olfr902	chr9:38448791-38449847	OtA5088-OtA5092	OtA5093-OtA5097
Olfr904	chr9:38464042-38464975	OtA5088-OtA5092	OtA5093-OtA5097
Olfr905	chr9:38472748-38473681	OtA5088-OtA5092	OtA5093-OtA5097
Olfr906	chr9:38488030-38488966	OtA5088-OtA5092	OtA5093-OtA5097
Olfr907	chr9:38498670-38499603	OtA5088-OtA5092	OtA5093-OtA5097
Olfr908	chr9:38516033-38516965	OtA5088-OtA5092	OtA5093-OtA5097
Olfr91	chr17:37092933-37093872	OtA5088-OtA5092	OtA5093-OtA5097
Olfr910	chr9:38537881-38539829	OtA5088-OtA5092	OtA5093-OtA5097
Olfr911-ps1	chr9:38523729-38524669	OtA5088-OtA5092	OtA5093-OtA5097
Olfr912	chr9:38580243-38582211	OtA5088-OtA5092	OtA5093-OtA5097
Olfr913	chr9:38592802-38595161	OtA5088-OtA5092	OtA5093-OtA5097
Olfr914	chr9:38606466-38607417	OtA5088-OtA5092	OtA5093-OtA5097
Olfr915	chr9:38646589-38647522	OtA5088-OtA5092	OtA5093-OtA5097
Olfr916	chr9:38657457-38658390	OtA5088-OtA5092	OtA5093-OtA5097
Olfr917	chr9:38664912-38665842	OtA5088-OtA5092	OtA5093-OtA5097
Olfr918	chr9:38672503-38673481	OtA5088-OtA5092	OtA5093-OtA5097
Olfr919	chr9:38697428-38698376	OtA5088-OtA5092	OtA5093-OtA5097
Olfr92	chr17:37111041-37111980	OtA5088-OtA5092	OtA5093-OtA5097
Olfr920	chr9:38752795-38756863	OtA5088-OtA5092	OtA5093-OtA5097
Olfr921	chr9:38773087-38776354	OtA5088-OtA5092	OtA5093-OtA5097
Olfr922	chr9:38815407-38816539	OtA5088-OtA5092	OtA5093-OtA5097
Olfr923	chr9:38827614-38828682	OtA5088-OtA5092	OtA5093-OtA5097
Olfr924	chr9:38848115-38849042	OtA5088-OtA5092	OtA5093-OtA5097
Olfr926	chr9:38877177-38878104	OtA5088-OtA5092	OtA5093-OtA5097
Olfr93	chr17:37151031-37151970	OtA5088-OtA5092	OtA5093-OtA5097
Olfr930	chr9:38930172-38931099	OtA5088-OtA5092	OtA5093-OtA5097
Olfr933	chr9:38975677-38976604	OtA5088-OtA5092	OtA5093-OtA5097
Olfr934	chr9:38982109-38983042	OtA5088-OtA5092	OtA5093-OtA5097
Olfr935	chr9:38994506-38995433	OtA5088-OtA5092	OtA5093-OtA5097
Olfr936	chr9:39046613-39047549	OtA5088-OtA5092	OtA5093-OtA5097
Olfr937	chr9:39059728-39060664	OtA5088-OtA5092	OtA5093-OtA5097
Olfr938	chr9:39077795-39078743	OtA5088-OtA5092	OtA5093-OtA5097
Olfr94	chr17:37196805-37197966	OtA5088-OtA5092	OtA5093-OtA5097
Olfr943	chr9:39166309-39185124	OtA5088-OtA5092	OtA5093-OtA5097

Olfr944	chr9:39217358-39218294	OtA5088-OtA5092	OtA5093-OtA5097
Olfr945	chr9:39257719-39258679	OtA5088-OtA5092	OtA5093-OtA5097
Olfr947-ps1	chr9:39287923-39299481	OtA5088-OtA5092	OtA5093-OtA5097
Olfr948	chr9:39318646-39319612	OtA5088-OtA5092	OtA5093-OtA5097
Olfr95	chr17:37210912-37211851	OtA5088-OtA5092	OtA5093-OtA5097
Olfr951	chr9:39393792-39394737	OtA5088-OtA5092	OtA5093-OtA5097
Olfr952	chr9:39426124-39427069	OtA5088-OtA5092	OtA5093-OtA5097
Olfr954	chr9:39461432-39462377	OtA5088-OtA5092	OtA5093-OtA5097
Olfr955	chr9:39469779-39470724	OtA5088-OtA5092	OtA5093-OtA5097
Olfr957	chr9:39510782-39511718	OtA5088-OtA5092	OtA5093-OtA5097
Olfr958	chr9:39549930-39550869	OtA5088-OtA5092	OtA5093-OtA5097
Olfr959	chr9:39572321-39573257	OtA5088-OtA5092	OtA5093-OtA5097
Olfr96	chr17:37225126-37226068	OtA5088-OtA5092	OtA5093-OtA5097
Olfr960	chr9:39623124-39624075	OtA5088-OtA5092	OtA5093-OtA5097
Olfr961	chr9:39646727-39647672	OtA5088-OtA5092	OtA5093-OtA5097
Olfr963	chr9:39669058-39669994	OtA5088-OtA5092	OtA5093-OtA5097
Olfr965	chr9:39719228-39720167	OtA5088-OtA5092	OtA5093-OtA5097
Olfr967	chr9:39750387-39751320	OtA5088-OtA5092	OtA5093-OtA5097
Olfr968	chr9:39771771-39772798	OtA5088-OtA5092	OtA5093-OtA5097
Olfr969	chr9:39795376-39796312	OtA5088-OtA5092	OtA5093-OtA5097
Olfr97	chr17:37231435-37232368	OtA5088-OtA5092	OtA5093-OtA5097
Olfr970	chr9:39819640-39820576	OtA5088-OtA5092	OtA5093-OtA5097
Olfr971	chr9:39839435-39840359	OtA5088-OtA5092	OtA5093-OtA5097
Olfr972	chr9:39873276-39874221	OtA5088-OtA5092	OtA5093-OtA5097
Olfr974	chr9:39942261-39943194	OtA5088-OtA5092	OtA5093-OtA5097
Olfr975	chr9:39949836-39950769	OtA5088-OtA5092	OtA5093-OtA5097
Olfr976	chr9:39954256-39957069	OtA5088-OtA5092	OtA5093-OtA5097
Olfr978	chr9:39993811-39994747	OtA5088-OtA5092	OtA5093-OtA5097
Olfr979	chr9:40000205-40001250	OtA5088-OtA5092	OtA5093-OtA5097
Olfr98	chr17:37262732-37263662	OtA5088-OtA5092	OtA5093-OtA5097
Olfr980	chr9:40005993-40007029	OtA5088-OtA5092	OtA5093-OtA5097
Olfr981	chr9:40022394-40023327	OtA5088-OtA5092	OtA5093-OtA5097
Olfr982	chr9:40074296-40075262	OtA5088-OtA5092	OtA5093-OtA5097
Olfr983	chr9:40092016-40093558	OtA5088-OtA5092	OtA5093-OtA5097
Olfr984	chr9:40100543-40101488	OtA5088-OtA5092	OtA5093-OtA5097
Olfr985	chr9:40126923-40128039	OtA5088-OtA5092	OtA5093-OtA5097
Olfr986	chr9:40187078-40188094	OtA5088-OtA5092	OtA5093-OtA5097
Olfr987	chr2:85330966-85331896	OtA5088-OtA5092	OtA5093-OtA5097
Olfr988	chr2:85352994-85353924	OtA5088-OtA5092	OtA5093-OtA5097
Olfr99	chr17:37279500-37280418	OtA5088-OtA5092	OtA5093-OtA5097
Olfr992	chr2:85399601-85400531	OtA5088-OtA5092	OtA5093-OtA5097
Olfr993	chr2:85413932-85414877	OtA5088-OtA5092	OtA5093-OtA5097
Olfr994	chr2:85429882-85430827	OtA5088-OtA5092	OtA5093-OtA5097
Olfr995	chr2:85438208-85439156	OtA5088-OtA5092	OtA5093-OtA5097
Olfr996	chr2:85579214-85580282	OtA5088-OtA5092	OtA5093-OtA5097
Olfr998	chr2:85590509-85591521	OtA5088-OtA5092	OtA5093-OtA5097
Olig1	chr16:91269768-91271939	OtA5088-OtA5092	OtA5093-OtA5097

Olig2	chr16:91225549-91228677	OtA5088-OtA5092	OtA5093-OtA5097
Olig3	chr10:19356538-19358604	OtA5088-OtA5092	OtA5093-OtA5097
Omd	chr13:49464058-49652731	OtA5088-OtA5092	OtA5093-OtA5097
Omg	chr11:79339891-79581609	OtA5088-OtA5092	OtA5093-OtA5097
Omp	chr7:98121564-98184799	OtA5088-OtA5092	OtA5093-OtA5097
Omt2a	chr9:78311971-78314797	OtA5088-OtA5092	OtA5093-OtA5097
Omt2b	chr9:78328029-78329620	OtA5088-OtA5092	OtA5093-OtA5097
Onecut1	chr9:74861920-74889647	OtA5088-OtA5092	OtA5093-OtA5097
Onecut2	chr18:64340363-64398488	OtA5088-OtA5092	OtA5093-OtA5097
Onecut3	chr10:80494905-80517260	OtA5088-OtA5092	OtA5093-OtA5097
Ooep	chr9:78376902-78378588	OtA5088-OtA5092	OtA5093-OtA5097
Oog1	chr12:87602687-87608845	OtA5088-OtA5092	OtA5093-OtA5097
Oog2	chr4:144190716-144196934	OtA5088-OtA5092	OtA5093-OtA5097
Oog3	chr4:144157555-144162651	OtA5088-OtA5092	OtA5093-OtA5097
Oog4	chr4:143437163-143450302	OtA5088-OtA5092	OtA5093-OtA5097
Oosp1	chr19:11667459-11691051	OtA5088-OtA5092	OtA5093-OtA5097
Opalin	chr19:41063419-41077113	OtA5088-OtA5092	OtA5093-OtA5097
Opcml	chr9:27791268-28925048	OtA5088-OtA5092	OtA5093-OtA5097
Ophn1	chrX:98557514-98890985	OtA5088-OtA5092	OtA5093-OtA5097
Opn1mw	chrX:74127465-74150755	OtA5088-OtA5092	OtA5093-OtA5097
Opn1sw	chr6:29348118-29380513	OtA5088-OtA5092	OtA5093-OtA5097
Opn4	chr14:34590617-34600142	OtA5088-OtA5092	OtA5093-OtA5097
Opn5	chr17:42556782-42611313	OtA5088-OtA5092	OtA5093-OtA5097
Oprd1	chr4:132110725-132144486	OtA5088-OtA5092	OtA5093-OtA5097
Oprk1	chr1:5588492-5606133	OtA5088-OtA5092	OtA5093-OtA5097
Oprl1	chr2:181715015-181720985	OtA5088-OtA5092	OtA5093-OtA5097
Oprm1	chr10:6788600-7038209	OtA5088-OtA5092	OtA5093-OtA5097
Optc	chr1:133897193-133907999	OtA5088-OtA5092	OtA5093-OtA5097
Orai2	chr5:136147460-136170656	OtA5088-OtA5092	OtA5093-OtA5097
Orc1	chr4:108579453-108614831	OtA5088-OtA5092	OtA5093-OtA5097
Orm1	chr4:63344565-63348162	OtA5088-OtA5092	OtA5093-OtA5097
Orm2	chr4:63362448-63365877	OtA5088-OtA5092	OtA5093-OtA5097
Orm3	chr4:63356161-63359511	OtA5088-OtA5092	OtA5093-OtA5097
Oscar	chr7:3609814-3616133	OtA5088-OtA5092	OtA5093-OtA5097
Oscp1	chr4:126058564-126089334	OtA5088-OtA5092	OtA5093-OtA5097
Osgepl1	chr1:53313662-53324064	OtA5088-OtA5092	OtA5093-OtA5097
Osm	chr11:4236784-4241026	OtA5088-OtA5092	OtA5093-OtA5097
Osr1	chr12:9574441-9581500	OtA5088-OtA5092	OtA5093-OtA5097
Ostb	chr9:65398487-65422773	OtA5088-OtA5092	OtA5093-OtA5097
Ostn	chr16:27307640-27351209	OtA5088-OtA5092	OtA5093-OtA5097
Otc	chrX:10252358-10321024	OtA5088-OtA5092	OtA5093-OtA5097
Otoa	chr7:121083437-121163089	OtA5088-OtA5092	OtA5093-OtA5097
Otof	chr5:30367065-30461932	OtA5088-OtA5092	OtA5093-OtA5097
Otog	chr7:46240986-46311434	OtA5088-OtA5092	OtA5093-OtA5097
Otogl	chr10:107762309-107912134	OtA5088-OtA5092	OtA5093-OtA5097
Otol1	chr3:70007612-70028708	OtA5088-OtA5092	OtA5093-OtA5097
Otop2	chr11:115307162-115332303	OtA5088-OtA5092	OtA5093-OtA5097

Otop3	chr11:115334733-115346926	OtA5088-OtA5092	OtA5093-OtA5097
Otor	chr2:143078491-143081699	OtA5088-OtA5092	OtA5093-OtA5097
Otos	chr1:92644217-92648841	OtA5088-OtA5092	OtA5093-OtA5097
Otp	chr13:94875626-94883681	OtA5088-OtA5092	OtA5093-OtA5097
Ott	chrX:149088314-149305003	OtA5088-OtA5092	OtA5093-OtA5097
Otud6a	chrX:100429012-100429885	OtA5088-OtA5092	OtA5093-OtA5097
Otud7a	chr7:63444771-63759027	OtA5088-OtA5092	OtA5093-OtA5097
Otx1	chr11:21994763-22001651	OtA5088-OtA5092	OtA5093-OtA5097
Otx2	chr14:48658183-48662872	OtA5088-OtA5092	OtA5093-OtA5097
Otx2os1	chr14:48669287-48793348	OtA5088-OtA5092	OtA5093-OtA5097
Ovch2	chr7:107781543-107801179	OtA5088-OtA5092	OtA5093-OtA5097
Ovgp1	chr3:105973801-105987423	OtA5088-OtA5092	OtA5093-OtA5097
Oxct2b	chr4:123105164-123126091	OtA5088-OtA5092	OtA5093-OtA5097
Oxgr1	chr14:120019584-120042435	OtA5088-OtA5092	OtA5093-OtA5097
Oxt	chr2:130576172-130576988	OtA5088-OtA5092	OtA5093-OtA5097
Oxtr	chr6:112473683-112489808	OtA5088-OtA5092	OtA5093-OtA5097
P2rx3	chr2:84996551-85035834	OtA5088-OtA5092	OtA5093-OtA5097
P2rx6	chr16:17561884-17572012	OtA5088-OtA5092	OtA5093-OtA5097
P2ry10	chrX:107089334-107104970	OtA5088-OtA5092	OtA5093-OtA5097
P2ry12	chr3:59006977-59344256	OtA5088-OtA5092	OtA5093-OtA5097
P2ry13	chr3:59006977-59344256	OtA5088-OtA5092	OtA5093-OtA5097
P2ry14	chr3:59006977-59344256	OtA5088-OtA5092	OtA5093-OtA5097
P2ry4	chrX:100590153-100594869	OtA5088-OtA5092	OtA5093-OtA5097
Pabpc1l	chr2:164025449-164050538	OtA5088-OtA5092	OtA5093-OtA5097
Pabpc2	chr18:39773496-39776082	OtA5088-OtA5092	OtA5093-OtA5097
Pabpc4l	chr3:46442196-46447941	OtA5088-OtA5092	OtA5093-OtA5097
Pabpc5	chrX:119927195-119930165	OtA5088-OtA5092	OtA5093-OtA5097
Pabpc6	chr17:9666496-9669704	OtA5088-OtA5092	OtA5093-OtA5097
Pabpn1l	chr8:122619470-122622735	OtA5088-OtA5092	OtA5093-OtA5097
Pacrg	chr17:10403011-10840191	OtA5088-OtA5092	OtA5093-OtA5097
Pacsin1	chr17:27655681-27711106	OtA5088-OtA5092	OtA5093-OtA5097
Padi3	chr4:140785368-140810648	OtA5088-OtA5092	OtA5093-OtA5097
Padi4	chr4:140745507-140774203	OtA5088-OtA5092	OtA5093-OtA5097
Padi6	chr4:140727354-140742643	OtA5088-OtA5092	OtA5093-OtA5097
Pah	chr10:87521794-87584137	OtA5088-OtA5092	OtA5093-OtA5097
Pak3	chrX:143518590-143797796	OtA5088-OtA5092	OtA5093-OtA5097
Pak7	chr2:136081087-136387967	OtA5088-OtA5092	OtA5093-OtA5097
Palb2	chr7:122107264-122132946	OtA5088-OtA5092	OtA5093-OtA5097
Palm2	chr4:57568247-57717128	OtA5088-OtA5092	OtA5093-OtA5097
Palmd	chr3:116918261-116984406	OtA5088-OtA5092	OtA5093-OtA5097
Pank1	chr19:34810893-34879455	OtA5088-OtA5092	OtA5093-OtA5097
Panx2	chr15:89059725-89070907	OtA5088-OtA5092	OtA5093-OtA5097
Panx3	chr9:37659901-37669222	OtA5088-OtA5092	OtA5093-OtA5097
Papolb	chr5:142484840-142551077	OtA5088-OtA5092	OtA5093-OtA5097
Pappa	chr4:65124173-65357509	OtA5088-OtA5092	OtA5093-OtA5097
Paqr5	chr9:61953737-62026790	OtA5088-OtA5092	OtA5093-OtA5097
Paqr6	chr3:88364588-88368541	OtA5088-OtA5092	OtA5093-OtA5097

Paqr9	chr9:95559816-95562121	OtA5088-OtA5092	OtA5093-OtA5097
Park2	chr17:10840383-12063360	OtA5088-OtA5092	OtA5093-OtA5097
Parp8	chr13:116854823-117025516	OtA5088-OtA5092	OtA5093-OtA5097
Parvg	chr15:84324719-84342978	OtA5088-OtA5092	OtA5093-OtA5097
Pate2	chr9:35669638-35672889	OtA5088-OtA5092	OtA5093-OtA5097
Pate4	chr9:35607092-35611885	OtA5088-OtA5092	OtA5093-OtA5097
Pax1	chr2:147364993-147375048	OtA5088-OtA5092	OtA5093-OtA5097
Pax2	chr19:44757393-44837869	OtA5088-OtA5092	OtA5093-OtA5097
Pax3	chr1:78101266-78197136	OtA5088-OtA5092	OtA5093-OtA5097
Pax4	chr6:28442333-28449340	OtA5088-OtA5092	OtA5093-OtA5097
Pax5	chr4:44531505-44710440	OtA5088-OtA5092	OtA5093-OtA5097
Pax6	chr2:105536079-105697364	OtA5088-OtA5092	OtA5093-OtA5097
Pax6os1	chr2:105536079-105697364	OtA5088-OtA5092	OtA5093-OtA5097
Pax7	chr4:139738080-139832968	OtA5088-OtA5092	OtA5093-OtA5097
Pax9	chr12:56695638-56711230	OtA5088-OtA5092	OtA5093-OtA5097
Pbk	chr14:65805910-65817822	OtA5088-OtA5092	OtA5093-OtA5097
Pbld2	chr10:63024511-63058812	OtA5088-OtA5092	OtA5093-OtA5097
Pbp2	chr6:135309128-135310384	OtA5088-OtA5092	OtA5093-OtA5097
Pbsn	chrX:77837897-77853500	OtA5088-OtA5092	OtA5093-OtA5097
Pbx4	chr8:69832703-69872292	OtA5088-OtA5092	OtA5093-OtA5097
Pcdh10	chr3:45378397-45439309	OtA5088-OtA5092	OtA5093-OtA5097
Pcdh11x	chrX:120290326-120906450	OtA5088-OtA5092	OtA5093-OtA5097
Pcdh15	chr10:73821866-74649732	OtA5088-OtA5092	OtA5093-OtA5097
Pcdh17	chr14:84443562-84537060	OtA5088-OtA5092	OtA5093-OtA5097
Pcdh19	chrX:133582860-133688993	OtA5088-OtA5092	OtA5093-OtA5097
Pcdh20	chr14:88464746-88471396	OtA5088-OtA5092	OtA5093-OtA5097
Pcdh8	chr14:79766771-79771312	OtA5088-OtA5092	OtA5093-OtA5097
Pcdh9	chr14:93013700-93888732	OtA5088-OtA5092	OtA5093-OtA5097
Pcdha1	chr18:36930284-37841872	OtA5088-OtA5092	OtA5093-OtA5097
Pcdha10	chr18:36930284-37841872	OtA5088-OtA5092	OtA5093-OtA5097
Pcdha11	chr18:36930284-37841872	OtA5088-OtA5092	OtA5093-OtA5097
Pcdha12	chr18:36930284-37841872	OtA5088-OtA5092	OtA5093-OtA5097
Pcdha2	chr18:36930284-37841872	OtA5088-OtA5092	OtA5093-OtA5097
Pcdha3	chr18:36930284-37841872	OtA5088-OtA5092	OtA5093-OtA5097
Pcdha4	chr18:36930284-37841872	OtA5088-OtA5092	OtA5093-OtA5097
Pcdha4-g	chr18:36930284-37841872	OtA5088-OtA5092	OtA5093-OtA5097
Pcdha5	chr18:36930284-37841872	OtA5088-OtA5092	OtA5093-OtA5097
Pcdha6	chr18:36930284-37841872	OtA5088-OtA5092	OtA5093-OtA5097
Pcdha7	chr18:36930284-37841872	OtA5088-OtA5092	OtA5093-OtA5097
Pcdha8	chr18:36930284-37841872	OtA5088-OtA5092	OtA5093-OtA5097
Pcdha9	chr18:36930284-37841872	OtA5088-OtA5092	OtA5093-OtA5097
Pcdhac1	chr18:36930284-37841872	OtA5088-OtA5092	OtA5093-OtA5097
Pcdhac2	chr18:36930284-37841872	OtA5088-OtA5092	OtA5093-OtA5097
Pcdhb1	chr18:36930284-37841872	OtA5088-OtA5092	OtA5093-OtA5097
Pcdhb10	chr18:36930284-37841872	OtA5088-OtA5092	OtA5093-OtA5097
Pcdhb11	chr18:36930284-37841872	OtA5088-OtA5092	OtA5093-OtA5097
Pcdhb12	chr18:36930284-37841872	OtA5088-OtA5092	OtA5093-OtA5097

Pcdhb13	chr18:36930284-37841872	OtA5088-OtA5092	OtA5093-OtA5097
Pcdhb14	chr18:36930284-37841872	OtA5088-OtA5092	OtA5093-OtA5097
Pcdhb15	chr18:36930284-37841872	OtA5088-OtA5092	OtA5093-OtA5097
Pcdhb16	chr18:36930284-37841872	OtA5088-OtA5092	OtA5093-OtA5097
Pcdhb17	chr18:36930284-37841872	OtA5088-OtA5092	OtA5093-OtA5097
Pcdhb18	chr18:36930284-37841872	OtA5088-OtA5092	OtA5093-OtA5097
Pcdhb19	chr18:36930284-37841872	OtA5088-OtA5092	OtA5093-OtA5097
Pcdhb2	chr18:36930284-37841872	OtA5088-OtA5092	OtA5093-OtA5097
Pcdhb20	chr18:36930284-37841872	OtA5088-OtA5092	OtA5093-OtA5097
Pcdhb21	chr18:36930284-37841872	OtA5088-OtA5092	OtA5093-OtA5097
Pcdhb22	chr18:36930284-37841872	OtA5088-OtA5092	OtA5093-OtA5097
Pcdhb3	chr18:36930284-37841872	OtA5088-OtA5092	OtA5093-OtA5097
Pcdhb4	chr18:36930284-37841872	OtA5088-OtA5092	OtA5093-OtA5097
Pcdhb5	chr18:36930284-37841872	OtA5088-OtA5092	OtA5093-OtA5097
Pcdhb6	chr18:36930284-37841872	OtA5088-OtA5092	OtA5093-OtA5097
Pcdhb7	chr18:36930284-37841872	OtA5088-OtA5092	OtA5093-OtA5097
Pcdhb8	chr18:36930284-37841872	OtA5088-OtA5092	OtA5093-OtA5097
Pcdhb9	chr18:36930284-37841872	OtA5088-OtA5092	OtA5093-OtA5097
Pcdhga1	chr18:36930284-37841872	OtA5088-OtA5092	OtA5093-OtA5097
Pcdhga2	chr18:36930284-37841872	OtA5088-OtA5092	OtA5093-OtA5097
Pcdhga3	chr18:36930284-37841872	OtA5088-OtA5092	OtA5093-OtA5097
Pcdhga4	chr18:36930284-37841872	OtA5088-OtA5092	OtA5093-OtA5097
Pcdhga5	chr18:36930284-37841872	OtA5088-OtA5092	OtA5093-OtA5097
Pcdhga6	chr18:36930284-37841872	OtA5088-OtA5092	OtA5093-OtA5097
Pcdhga7	chr18:36930284-37841872	OtA5088-OtA5092	OtA5093-OtA5097
Pcdhga8	chr18:36930284-37841872	OtA5088-OtA5092	OtA5093-OtA5097
Pcdhga9	chr18:36930284-37841872	OtA5088-OtA5092	OtA5093-OtA5097
Pcdhgb1	chr18:36930284-37841872	OtA5088-OtA5092	OtA5093-OtA5097
Pcdhgb2	chr18:36930284-37841872	OtA5088-OtA5092	OtA5093-OtA5097
Pcdhgb5	chr18:36930284-37841872	OtA5088-OtA5092	OtA5093-OtA5097
Pcdhgb8	chr18:36930284-37841872	OtA5088-OtA5092	OtA5093-OtA5097
Pcdhgc4	chr18:36930284-37841872	OtA5088-OtA5092	OtA5093-OtA5097
Pcdhgc5	chr18:36930284-37841872	OtA5088-OtA5092	OtA5093-OtA5097
Pck1	chr2:173153072-173159253	OtA5088-OtA5092	OtA5093-OtA5097
Pclo	chr5:14514917-14863459	OtA5088-OtA5092	OtA5093-OtA5097
Pcnxl2	chr8:125751507-125898293	OtA5088-OtA5092	OtA5093-OtA5097
Pcolce2	chr9:95637627-95695551	OtA5088-OtA5092	OtA5093-OtA5097
Pcp2	chr8:3621550-3625545	OtA5088-OtA5092	OtA5093-OtA5097
Pcp4	chr16:96467605-96525793	OtA5088-OtA5092	OtA5093-OtA5097
Pcsk1	chr13:75089986-75132498	OtA5088-OtA5092	OtA5093-OtA5097
Pcsk1n	chrX:7919821-7928607	OtA5088-OtA5092	OtA5093-OtA5097
Pcsk2	chr2:143535475-143816282	OtA5088-OtA5092	OtA5093-OtA5097
Pcsk4	chr10:79697304-80369637	OtA5088-OtA5092	OtA5093-OtA5097
Pcyt1b	chrX:93654862-93749948	OtA5088-OtA5092	OtA5093-OtA5097
Pdc	chr1:150319416-150346771	OtA5088-OtA5092	OtA5093-OtA5097
Pdcd1	chr1:94038304-94052553	OtA5088-OtA5092	OtA5093-OtA5097
Pdcd1lg2	chr19:29410918-29472927	OtA5088-OtA5092	OtA5093-OtA5097

Pdcl2	chr5:76183879-76469519	OtA5088-OtA5092	OtA5093-OtA5097
Pde11a	chr2:75991131-76338653	OtA5088-OtA5092	OtA5093-OtA5097
Pde1a	chr2:79834452-80129412	OtA5088-OtA5092	OtA5093-OtA5097
Pde1c	chr6:56069803-56369634	OtA5088-OtA5092	OtA5093-OtA5097
Pde4c	chr8:70724063-70751176	OtA5088-OtA5092	OtA5093-OtA5097
Pde6a	chr18:61220498-61289718	OtA5088-OtA5092	OtA5093-OtA5097
Pde6b	chr5:108388372-108431742	OtA5088-OtA5092	OtA5093-OtA5097
Pde6c	chr19:38132780-38183951	OtA5088-OtA5092	OtA5093-OtA5097
Pde6g	chr11:120447609-120453483	OtA5088-OtA5092	OtA5093-OtA5097
Pde6h	chr6:136954522-136963483	OtA5088-OtA5092	OtA5093-OtA5097
Pde7b	chr10:20398003-20725068	OtA5088-OtA5092	OtA5093-OtA5097
Pde8b	chr13:95024104-95250304	OtA5088-OtA5092	OtA5093-OtA5097
Pdgfd	chr9:6168611-6377519	OtA5088-OtA5092	OtA5093-OtA5097
Pdgfrl	chr8:40926232-40990770	OtA5088-OtA5092	OtA5093-OtA5097
Pdha2	chr3:141210003-141212355	OtA5088-OtA5092	OtA5093-OtA5097
Pdia2	chr17:26195998-26199087	OtA5088-OtA5092	OtA5093-OtA5097
Pdilt	chr7:119486586-119523482	OtA5088-OtA5092	OtA5093-OtA5097
Pdp1	chr4:11958184-11966450	OtA5088-OtA5092	OtA5093-OtA5097
Pdss1	chr2:22895521-23040240	OtA5088-OtA5092	OtA5093-OtA5097
Pdx1	chr5:147228597-147275149	OtA5088-OtA5092	OtA5093-OtA5097
Pdxk-ps	chr17:32076082-32091423	OtA5088-OtA5092	OtA5093-OtA5097
Pdyn	chr2:129688000-129689868	OtA5088-OtA5092	OtA5093-OtA5097
Pdzd4	chrX:73793356-73824969	OtA5088-OtA5092	OtA5093-OtA5097
Pdzd7	chr19:45015175-45045772	OtA5088-OtA5092	OtA5093-OtA5097
Pdzd9	chr7:120635188-120670343	OtA5088-OtA5092	OtA5093-OtA5097
Pdzrn4	chr15:92396809-92771819	OtA5088-OtA5092	OtA5093-OtA5097
Pea15b	chr5:77511194-77511748	OtA5088-OtA5092	OtA5093-OtA5097
Pebp4	chr14:69840419-70059886	OtA5088-OtA5092	OtA5093-OtA5097
Peg12	chr7:62461870-62464510	OtA5088-OtA5092	OtA5093-OtA5097
Peg3as	chr7:6705959-6730419	OtA5088-OtA5092	OtA5093-OtA5097
Peli3	chr19:4931854-4943092	OtA5088-OtA5092	OtA5093-OtA5097
Pet2	chrX:89403847-89409689	OtA5088-OtA5092	OtA5093-OtA5097
Pfn3	chr13:55399647-55415232	OtA5088-OtA5092	OtA5093-OtA5097
Pfn4	chr12:4769294-4778266	OtA5088-OtA5092	OtA5093-OtA5097
Pfpl	chr19:12427904-12432110	OtA5088-OtA5092	OtA5093-OtA5097
Pgam2	chr11:5801636-5803796	OtA5088-OtA5092	OtA5093-OtA5097
Pgap1	chr1:54472999-54557684	OtA5088-OtA5092	OtA5093-OtA5097
Pgbd1	chr13:21421274-21441053	OtA5088-OtA5092	OtA5093-OtA5097
Pgbd5	chr8:124369048-124433936	OtA5088-OtA5092	OtA5093-OtA5097
Pgk2	chr17:40207017-40208609	OtA5088-OtA5092	OtA5093-OtA5097
Pglyrp2	chr17:32413099-32420975	OtA5088-OtA5092	OtA5093-OtA5097
Pglyrp3	chr3:92014582-92031584	OtA5088-OtA5092	OtA5093-OtA5097
Pglyrp4	chr3:90726905-90741517	OtA5088-OtA5092	OtA5093-OtA5097
Pgm5	chr19:24674007-24861842	OtA5088-OtA5092	OtA5093-OtA5097
Pgpep1l	chr7:68236607-68264233	OtA5088-OtA5092	OtA5093-OtA5097
Pgr15l	chrX:97072595-97082104	OtA5088-OtA5092	OtA5093-OtA5097
Phactr3	chr2:178118974-178338492	OtA5088-OtA5092	OtA5093-OtA5097

Phex	chrX:157162074-157415286	OtA5088-OtA5092	OtA5093-OtA5097
Phf11	chr14:59276912-59297522	OtA5088-OtA5092	OtA5093-OtA5097
Phf21b	chr15:84785375-84856129	OtA5088-OtA5092	OtA5093-OtA5097
Phf6	chrX:52912265-52956943	OtA5088-OtA5092	OtA5093-OtA5097
Phf7	chr14:31237695-31251218	OtA5088-OtA5092	OtA5093-OtA5097
Phox2a	chr7:101818312-101838226	OtA5088-OtA5092	OtA5093-OtA5097
Phox2b	chr5:67094396-67099126	OtA5088-OtA5092	OtA5093-OtA5097
Phxr4	chr9:13431360-13432740	OtA5088-OtA5092	OtA5093-OtA5097
Phyhip	chr14:70457516-70468824	OtA5088-OtA5092	OtA5093-OtA5097
Pi15	chr1:17601900-17630939	OtA5088-OtA5092	OtA5093-OtA5097
Pi16	chr17:29318881-29328902	OtA5088-OtA5092	OtA5093-OtA5097
Pif1	chr9:65587204-65595962	OtA5088-OtA5092	OtA5093-OtA5097
Piga	chrX:164419786-164433915	OtA5088-OtA5092	OtA5093-OtA5097
Pigw	chr11:84876312-84916356	OtA5088-OtA5092	OtA5093-OtA5097
Pigy	chr6:57684738-57692078	OtA5088-OtA5092	OtA5093-OtA5097
Pigz	chr16:31933850-31946046	OtA5088-OtA5092	OtA5093-OtA5097
Pih1d2	chr9:50603900-50625000	OtA5088-OtA5092	OtA5093-OtA5097
Pik3c2g	chr6:139715858-139969283	OtA5088-OtA5092	OtA5093-OtA5097
Pik3cg	chr12:32173396-32208649	OtA5088-OtA5092	OtA5093-OtA5097
Pik3r5	chr11:68432124-68497846	OtA5088-OtA5092	OtA5093-OtA5097
Pik3r6	chr11:68503018-68552698	OtA5088-OtA5092	OtA5093-OtA5097
Pilra	chr5:137787801-137836278	OtA5088-OtA5092	OtA5093-OtA5097
Pilrb1	chr5:137852146-137858049	OtA5088-OtA5092	OtA5093-OtA5097
Pilrb2	chr5:137865828-137871758	OtA5088-OtA5092	OtA5093-OtA5097
Pin1-ps1	chr2:104713925-104716290	OtA5088-OtA5092	OtA5093-OtA5097
Pinc	chr1:73391384-73430779	OtA5088-OtA5092	OtA5093-OtA5097
Pinx1	chr14:63860311-63919859	OtA5088-OtA5092	OtA5093-OtA5097
Pion	chr5:21186266-21291701	OtA5088-OtA5092	OtA5093-OtA5097
Pip	chr6:41847548-41852062	OtA5088-OtA5092	OtA5093-OtA5097
Pip5kl1	chr2:32575818-32583779	OtA5088-OtA5092	OtA5093-OtA5097
Pipox	chr11:77880614-77893872	OtA5088-OtA5092	OtA5093-OtA5097
Pir	chrX:164269430-164373013	OtA5088-OtA5092	OtA5093-OtA5097
Pira1	chr7:3836809-3915501	OtA5088-OtA5092	OtA5093-OtA5097
Pira11	chr7:3836809-3915501	OtA5088-OtA5092	OtA5093-OtA5097
Pira2	chr7:3836809-3915501	OtA5088-OtA5092	OtA5093-OtA5097
Pira4,Pira7	chr7:3836809-3915501	OtA5088-OtA5092	OtA5093-OtA5097
Pira6	chr7:4274142-4282928	OtA5088-OtA5092	OtA5093-OtA5097
Pira7	chr7:3836809-3915501	OtA5088-OtA5092	OtA5093-OtA5097
Pirt	chr11:66905631-66947086	OtA5088-OtA5092	OtA5093-OtA5097
Pisd-ps3	chrUn_JH584304:52673-59689	OtA5088-OtA5092	OtA5093-OtA5097
Pitpnm3	chr11:72047527-72135889	OtA5088-OtA5092	OtA5093-OtA5097
Pitx3	chr19:46131898-46148325	OtA5088-OtA5092	OtA5093-OtA5097
Piwil1	chr5:128736245-128755474	OtA5088-OtA5092	OtA5093-OtA5097
Piwil2	chr14:70372479-70429094	OtA5088-OtA5092	OtA5093-OtA5097
Piwil4	chr9:14702357-14740733	OtA5088-OtA5092	OtA5093-OtA5097
Pkd1l2	chr8:116995678-117082449	OtA5088-OtA5092	OtA5093-OtA5097
Pkd1l3	chr8:109614516-109693294	OtA5088-OtA5092	OtA5093-OtA5097

Pkd2l1	chr19:44147636-44192442	OtA5088-OtA5092	OtA5093-OtA5097
Pkd2l2	chr18:34409422-34506823	OtA5088-OtA5092	OtA5093-OtA5097
Pkdrej	chr15:85814675-85821733	OtA5088-OtA5092	OtA5093-OtA5097
Pkhd1	chr1:20057778-20618057	OtA5088-OtA5092	OtA5093-OtA5097
Pkhd1l1	chr15:44457552-44597135	OtA5088-OtA5092	OtA5093-OtA5097
Pkia	chr3:7366603-7445365	OtA5088-OtA5092	OtA5093-OtA5097
Pkib	chr10:57631980-57741112	OtA5088-OtA5092	OtA5093-OtA5097
Pkp1	chr1:135871394-135919024	OtA5088-OtA5092	OtA5093-OtA5097
Pla2g10	chr16:13715061-13739471	OtA5088-OtA5092	OtA5093-OtA5097
Pla2g1b	chr5:115466265-115474717	OtA5088-OtA5092	OtA5093-OtA5097
Pla2g2a	chr4:138799246-138863469	OtA5088-OtA5092	OtA5093-OtA5097
Pla2g2c	chr4:138725324-138744575	OtA5088-OtA5092	OtA5093-OtA5097
Pla2g2d	chr4:138775734-138782143	OtA5088-OtA5092	OtA5093-OtA5097
Pla2g2f	chr4:138750532-138757598	OtA5088-OtA5092	OtA5093-OtA5097
Pla2g3	chr11:3488226-3494166	OtA5088-OtA5092	OtA5093-OtA5097
Pla2g4b	chr2:120033432-120043032	OtA5088-OtA5092	OtA5093-OtA5097
Pla2g4c	chr7:13325666-13360668	OtA5088-OtA5092	OtA5093-OtA5097
Pla2g5	chr4:138799246-138863469	OtA5088-OtA5092	OtA5093-OtA5097
Plac1l	chr19:11647283-11660559	OtA5088-OtA5092	OtA5093-OtA5097
Plac8l1	chr18:42178674-42196709	OtA5088-OtA5092	OtA5093-OtA5097
Plac9	chr14:26167786-26182273	OtA5088-OtA5092	OtA5093-OtA5097
Plag1	chr4:3901157-3938405	OtA5088-OtA5092	OtA5093-OtA5097
Plcb1	chr2:134786163-135475258	OtA5088-OtA5092	OtA5093-OtA5097
Plcb2	chr2:118707516-118728438	OtA5088-OtA5092	OtA5093-OtA5097
Plcd4	chr1:74409383-74588028	OtA5088-OtA5092	OtA5093-OtA5097
Plce1	chr19:38524196-38785100	OtA5088-OtA5092	OtA5093-OtA5097
Plch1	chr3:63696233-63850991	OtA5088-OtA5092	OtA5093-OtA5097
Plcl1	chr1:55405945-55754285	OtA5088-OtA5092	OtA5093-OtA5097
Plcxd1	chr5:110099968-110108197	OtA5088-OtA5092	OtA5093-OtA5097
Plcxd3	chr15:4375490-4575579	OtA5088-OtA5092	OtA5093-OtA5097
Plcz1	chr6:139989721-140041417	OtA5088-OtA5092	OtA5093-OtA5097
Pld5	chr1:175962305-176275312	OtA5088-OtA5092	OtA5093-OtA5097
Pld6	chr11:59783892-59787657	OtA5088-OtA5092	OtA5093-OtA5097
Pldi	chr10:60928225-60938132	OtA5088-OtA5092	OtA5093-OtA5097
Plek2	chr12:78888696-78906938	OtA5088-OtA5092	OtA5093-OtA5097
Plekhb1	chr7:100643895-100662394	OtA5088-OtA5092	OtA5093-OtA5097
Plekhn1	chr4:156221455-156228542	OtA5088-OtA5092	OtA5093-OtA5097
Plin1	chr7:79721163-79732776	OtA5088-OtA5092	OtA5093-OtA5097
Plin5	chr17:56111600-56117548	OtA5088-OtA5092	OtA5093-OtA5097
Plk1	chr7:122159436-122169875	OtA5088-OtA5092	OtA5093-OtA5097
Plk4	chr3:40799950-40816883	OtA5088-OtA5092	OtA5093-OtA5097
Plk5	chr10:79697304-80369637	OtA5088-OtA5092	OtA5093-OtA5097
Pln	chr10:53278080-53379851	OtA5088-OtA5092	OtA5093-OtA5097
Plp1	chrX:136822835-136837742	OtA5088-OtA5092	OtA5093-OtA5097
Plscr2	chr9:92275601-92297752	OtA5088-OtA5092	OtA5093-OtA5097
Plscr4	chr9:92457377-92492516	OtA5088-OtA5092	OtA5093-OtA5097
Plscr5	chr9:92192935-92209698	OtA5088-OtA5092	OtA5093-OtA5097

Plxdc1	chr11:97923236-97986446	OtA5088-OtA5092	OtA5093-OtA5097
Plxna3	chrX:74329065-74344689	OtA5088-OtA5092	OtA5093-OtA5097
Plxnb3	chrX:73757102-73772510	OtA5088-OtA5092	OtA5093-OtA5097
Plxnc1	chr10:94790865-94944578	OtA5088-OtA5092	OtA5093-OtA5097
Pm20d1	chr1:131797394-131818115	OtA5088-OtA5092	OtA5093-OtA5097
Pm20d2	chr4:33170405-33189737	OtA5088-OtA5092	OtA5093-OtA5097
Pmch	chr10:88091071-88146941	OtA5088-OtA5092	OtA5093-OtA5097
Pmfbp1	chr8:109494026-109542642	OtA5088-OtA5092	OtA5093-OtA5097
Pmp2	chr3:10179850-10183885	OtA5088-OtA5092	OtA5093-OtA5097
Pms1	chr1:53189188-53296996	OtA5088-OtA5092	OtA5093-OtA5097
Pnck	chrX:73655991-73660070	OtA5088-OtA5092	OtA5093-OtA5097
Pnldc1	chr17:12888901-12910000	OtA5088-OtA5092	OtA5093-OtA5097
Pnlip	chr19:58670364-58681788	OtA5088-OtA5092	OtA5093-OtA5097
Pnliprp1	chr19:58728886-58744169	OtA5088-OtA5092	OtA5093-OtA5097
Pnliprp2	chr19:58759722-58777534	OtA5088-OtA5092	OtA5093-OtA5097
Pnma1	chr12:84146130-84148489	OtA5088-OtA5092	OtA5093-OtA5097
Pnma2	chr14:66911207-66920061	OtA5088-OtA5092	OtA5093-OtA5097
Pnma3	chrX:73064786-73068191	OtA5088-OtA5092	OtA5093-OtA5097
Pnma5	chrX:73033980-73037103	OtA5088-OtA5092	OtA5093-OtA5097
Pnmal1	chr7:16959794-16962320	OtA5088-OtA5092	OtA5093-OtA5097
Pnmal2	chr7:16944681-16948828	OtA5088-OtA5092	OtA5093-OtA5097
Pnmt	chr11:98386631-98388097	OtA5088-OtA5092	OtA5093-OtA5097
Pnoc	chr14:65400672-65425472	OtA5088-OtA5092	OtA5093-OtA5097
Pnpla1	chr17:28858410-28890308	OtA5088-OtA5092	OtA5093-OtA5097
Pnpla5	chr15:84112620-84123175	OtA5088-OtA5092	OtA5093-OtA5097
Podnl1	chr8:84125988-84132517	OtA5088-OtA5092	OtA5093-OtA5097
Pof1b	chrX:112638426-112698651	OtA5088-OtA5092	OtA5093-OtA5097
Pola1	chrX:93304765-93632155	OtA5088-OtA5092	OtA5093-OtA5097
Pole	chr5:110286318-110337453	OtA5088-OtA5092	OtA5093-OtA5097
Pole2	chr12:69201778-69228190	OtA5088-OtA5092	OtA5093-OtA5097
Poln	chr5:34007199-34169448	OtA5088-OtA5092	OtA5093-OtA5097
Polq	chr16:37011785-37095417	OtA5088-OtA5092	OtA5093-OtA5097
Polr1e	chr4:45018624-45033692	OtA5088-OtA5092	OtA5093-OtA5097
Pom121l12	chr11:14599239-14600316	OtA5088-OtA5092	OtA5093-OtA5097
Pom121l2	chr13:21981180-21988734	OtA5088-OtA5092	OtA5093-OtA5097
Pon1	chr6:5168089-5193946	OtA5088-OtA5092	OtA5093-OtA5097
Pot1b	chr17:55652024-55712628	OtA5088-OtA5092	OtA5093-OtA5097
Poteg	chr8:27447669-27495172	OtA5088-OtA5092	OtA5093-OtA5097
Pou1f1	chr16:65520628-65533981	OtA5088-OtA5092	OtA5093-OtA5097
Pou2af1	chr9:51213689-51240079	OtA5088-OtA5092	OtA5093-OtA5097
Pou2f2	chr7:25091114-25132460	OtA5088-OtA5092	OtA5093-OtA5097
Pou3f2	chr4:22482094-22488366	OtA5088-OtA5092	OtA5093-OtA5097
Pou3f3	chr1:42697145-42700209	OtA5088-OtA5092	OtA5093-OtA5097
Pou3f4	chrX:110814464-110815550	OtA5088-OtA5092	OtA5093-OtA5097
Pou4f1	chr14:104462324-104467999	OtA5088-OtA5092	OtA5093-OtA5097
Pou4f2	chr8:78433008-78436652	OtA5088-OtA5092	OtA5093-OtA5097
Pou4f3	chr18:42394596-42396093	OtA5088-OtA5092	OtA5093-OtA5097

Pou5f1	chr17:35506031-35510777	OtA5088-OtA5092	OtA5093-OtA5097
Pou5f2	chr13:77708689-78166240	OtA5088-OtA5092	OtA5093-OtA5097
Pou6f2	chr13:18124959-18382039	OtA5088-OtA5092	OtA5093-OtA5097
Ppapdc1a	chr7:129257093-129391307	OtA5088-OtA5092	OtA5093-OtA5097
Ppara	chr15:85735563-85806851	OtA5088-OtA5092	OtA5093-OtA5097
Ppargc1a	chr5:51454248-51553921	OtA5088-OtA5092	OtA5093-OtA5097
Ppef1	chrX:160623093-160719972	OtA5088-OtA5092	OtA5093-OtA5097
Ppef2	chr5:92226973-92253159	OtA5088-OtA5092	OtA5093-OtA5097
Ppfia2	chr10:106470309-106933468	OtA5088-OtA5092	OtA5093-OtA5097
Ppfia3	chr7:45339125-45370564	OtA5088-OtA5092	OtA5093-OtA5097
Ppifos	chr14:25694169-25701282	OtA5088-OtA5092	OtA5093-OtA5097
Ppil6	chr10:41490438-41514288	OtA5088-OtA5092	OtA5093-OtA5097
Ppm1j	chr3:104781055-104786017	OtA5088-OtA5092	OtA5093-OtA5097
Ppm1n	chr7:19276806-19280049	OtA5088-OtA5092	OtA5093-OtA5097
Ppp1r14c	chr10:3366149-3464975	OtA5088-OtA5092	OtA5093-OtA5097
Ppp1r14d	chr2:119218122-119229865	OtA5088-OtA5092	OtA5093-OtA5097
Ppp1r17	chr6:56017514-56032688	OtA5088-OtA5092	OtA5093-OtA5097
Ppp1r1a	chr15:103530278-103537992	OtA5088-OtA5092	OtA5093-OtA5097
Ppp1r1b	chr11:98348737-98358283	OtA5088-OtA5092	OtA5093-OtA5097
Ppp1r1c	chr2:79707779-79810313	OtA5088-OtA5092	OtA5093-OtA5097
Ppp1r2-ps3	chr19:30538875-30539679	OtA5088-OtA5092	OtA5093-OtA5097
Ppp1r2-ps7	chrX:22461595-22463543	OtA5088-OtA5092	OtA5093-OtA5097
Ppp1r2-ps9	chrX:15110585-15111489	OtA5088-OtA5092	OtA5093-OtA5097
Ppp1r26	chr2:28447940-28455508	OtA5088-OtA5092	OtA5093-OtA5097
Ppp1r27	chr11:120549974-120551132	OtA5088-OtA5092	OtA5093-OtA5097
Ppp1r32	chr19:10474256-10482897	OtA5088-OtA5092	OtA5093-OtA5097
Ppp1r36	chr12:76417598-76439491	OtA5088-OtA5092	OtA5093-OtA5097
Ppp1r3a	chr6:14713821-14755274	OtA5088-OtA5092	OtA5093-OtA5097
Ppp1r42	chr1:9968625-10009130	OtA5088-OtA5092	OtA5093-OtA5097
Ppp2r2b	chr18:42645268-43059471	OtA5088-OtA5092	OtA5093-OtA5097
Ppp2r3d	chr9:124474345-124476862	OtA5088-OtA5092	OtA5093-OtA5097
Ppp3r2	chr4:49665225-49845081	OtA5088-OtA5092	OtA5093-OtA5097
Ppp4r1l-ps	chr2:173579319-173659539	OtA5088-OtA5092	OtA5093-OtA5097
Ppp4r4	chr12:103532564-103613832	OtA5088-OtA5092	OtA5093-OtA5097
Ppyr1	chr14:34145645-34152419	OtA5088-OtA5092	OtA5093-OtA5097
Pram1	chr17:33638055-33645706	OtA5088-OtA5092	OtA5093-OtA5097
Prame	chrX:135613001-135627705	OtA5088-OtA5092	OtA5093-OtA5097
Pramef17	chr4:143991118-143994369	OtA5088-OtA5092	OtA5093-OtA5097
Pramef6	chr4:143894236-143900380	OtA5088-OtA5092	OtA5093-OtA5097
Pramel3	chrX:135302315-135312636	OtA5088-OtA5092	OtA5093-OtA5097
Pramel4	chr4:144059125-144069318	OtA5088-OtA5092	OtA5093-OtA5097
Pramel5	chr4:144270632-144280466	OtA5088-OtA5092	OtA5093-OtA5097
Pramel6	chr2:87508457-87510865	OtA5088-OtA5092	OtA5093-OtA5097
Pramel7	chr2:87489087-87492418	OtA5088-OtA5092	OtA5093-OtA5097
Prb1	chr6:132114220-132364135	OtA5088-OtA5092	OtA5093-OtA5097
Prc1	chr7:80294464-80316259	OtA5088-OtA5092	OtA5093-OtA5097
Prdm12	chr2:31640036-31655795	OtA5088-OtA5092	OtA5093-OtA5097

Prdm13	chr4:21677479-21685963	OtA5088-OtA5092	OtA5093-OtA5097
Prdm14	chr1:13113427-13127163	OtA5088-OtA5092	OtA5093-OtA5097
Prdm16	chr4:154316124-154636873	OtA5088-OtA5092	OtA5093-OtA5097
Prdm5	chr6:65778961-65936377	OtA5088-OtA5092	OtA5093-OtA5097
Prdm8	chr5:98180868-98187448	OtA5088-OtA5092	OtA5093-OtA5097
Prdm9	chr17:15543078-15563323	OtA5088-OtA5092	OtA5093-OtA5097
Prdx6b	chr2:80292469-80295358	OtA5088-OtA5092	OtA5093-OtA5097
Prg2	chr2:84980460-84983632	OtA5088-OtA5092	OtA5093-OtA5097
Prg3	chr2:84988214-84993886	OtA5088-OtA5092	OtA5093-OtA5097
Prg4	chr1:150361310-150466165	OtA5088-OtA5092	OtA5093-OtA5097
Prh1	chr6:132569841-132572401	OtA5088-OtA5092	OtA5093-OtA5097
Prhoxnb	chr5:147314983-147322440	OtA5088-OtA5092	OtA5093-OtA5097
Prim1	chr10:128015214-128030030	OtA5088-OtA5092	OtA5093-OtA5097
Prima1	chr12:103196907-103242146	OtA5088-OtA5092	OtA5093-OtA5097
Prkag3	chr1:74738921-74748955	OtA5088-OtA5092	OtA5093-OtA5097
Prkcb	chr7:122289124-122634401	OtA5088-OtA5092	OtA5093-OtA5097
Prkcc	chr7:3303531-3330696	OtA5088-OtA5092	OtA5093-OtA5097
Prkcq	chr2:11172381-11301226	OtA5088-OtA5092	OtA5093-OtA5097
Prkd1	chr12:50341231-50649223	OtA5088-OtA5092	OtA5093-OtA5097
Prkdc	chr16:15637865-15842239	OtA5088-OtA5092	OtA5093-OtA5097
Prkg2	chr5:98929772-99037079	OtA5088-OtA5092	OtA5093-OtA5097
Prl	chr13:27057569-27065203	OtA5088-OtA5092	OtA5093-OtA5097
Prl2c4	chr13:12790817-12800079	OtA5088-OtA5092	OtA5093-OtA5097
Prl3d2	chr13:27121703-27127482	OtA5088-OtA5092	OtA5093-OtA5097
Prl3d3	chr13:27156798-27162520	OtA5088-OtA5092	OtA5093-OtA5097
Prlh	chr1:90953107-90954012	OtA5088-OtA5092	OtA5093-OtA5097
Prlhr	chr19:60466732-60468304	OtA5088-OtA5092	OtA5093-OtA5097
Prm1	chr16:10796331-10796823	OtA5088-OtA5092	OtA5093-OtA5097
Prm2	chr16:10791380-10792097	OtA5088-OtA5092	OtA5093-OtA5097
Prm3	chr16:10790507-10790914	OtA5088-OtA5092	OtA5093-OtA5097
Prmt8	chr6:127689008-127769159	OtA5088-OtA5092	OtA5093-OtA5097
Prnd	chr2:131950860-131956130	OtA5088-OtA5092	OtA5093-OtA5097
Proca1	chr11:78193391-78205763	OtA5088-OtA5092	OtA5093-OtA5097
Prodh2	chr7:30493657-30513402	OtA5088-OtA5092	OtA5093-OtA5097
Prok1	chr3:107235530-107239707	OtA5088-OtA5092	OtA5093-OtA5097
Prok2	chr6:99711298-99726392	OtA5088-OtA5092	OtA5093-OtA5097
Prokr1	chr6:87578591-87590701	OtA5088-OtA5092	OtA5093-OtA5097
Prokr2	chr2:132370328-132385447	OtA5088-OtA5092	OtA5093-OtA5097
Prol1	chr5:88317311-88328817	OtA5088-OtA5092	OtA5093-OtA5097
Prop1	chr11:50950805-50953632	OtA5088-OtA5092	OtA5093-OtA5097
Prorsd1	chr11:29511756-29515033	OtA5088-OtA5092	OtA5093-OtA5097
Prox1	chr1:190121776-190170680	OtA5088-OtA5092	OtA5093-OtA5097
Prox2	chr12:85086813-85106431	OtA5088-OtA5092	OtA5093-OtA5097
Prp2	chr6:132595945-132600702	OtA5088-OtA5092	OtA5093-OtA5097
Prph2	chr17:46910484-46924926	OtA5088-OtA5092	OtA5093-OtA5097
Prpmp5	chr6:132114220-132364135	OtA5088-OtA5092	OtA5093-OtA5097
Prps1l1	chr12:34984760-34986436	OtA5088-OtA5092	OtA5093-OtA5097

Prr11	chr11:87089155-87108714	OtA5088-OtA5092	OtA5093-OtA5097
Prr16	chr18:51117897-51304641	OtA5088-OtA5092	OtA5093-OtA5097
Prr18	chr17:8340405-8344113	OtA5088-OtA5092	OtA5093-OtA5097
Prr19	chr7:25301358-25304133	OtA5088-OtA5092	OtA5093-OtA5097
Prr22	chr17:56770275-56772134	OtA5088-OtA5092	OtA5093-OtA5097
Prr23a	chr9:98842586-98843645	OtA5088-OtA5092	OtA5093-OtA5097
Prr7	chr13:55464266-55473155	OtA5088-OtA5092	OtA5093-OtA5097
Prr9	chr3:92122203-92123947	OtA5088-OtA5092	OtA5093-OtA5097
Prrg1	chrX:78449612-78583896	OtA5088-OtA5092	OtA5093-OtA5097
Prrt1	chr17:34629685-34632260	OtA5088-OtA5092	OtA5093-OtA5097
Prrt2	chr7:127017541-127021211	OtA5088-OtA5092	OtA5093-OtA5097
Prrt3	chr6:113494094-113501818	OtA5088-OtA5092	OtA5093-OtA5097
Prrt4	chr6:29169229-29179584	OtA5088-OtA5092	OtA5093-OtA5097
Prrx2	chr2:30845366-30881247	OtA5088-OtA5092	OtA5093-OtA5097
Prrxl1	chr14:32599926-32649246	OtA5088-OtA5092	OtA5093-OtA5097
Prss1	chr6:41458929-41463786	OtA5088-OtA5092	OtA5093-OtA5097
Prss2	chr6:41521775-41525079	OtA5088-OtA5092	OtA5093-OtA5097
Prss21	chr17:23868071-23873113	OtA5088-OtA5092	OtA5093-OtA5097
Prss22	chr17:23993533-23998100	OtA5088-OtA5092	OtA5093-OtA5097
Prss27	chr17:24038242-24045949	OtA5088-OtA5092	OtA5093-OtA5097
Prss28	chr17:25308645-25311876	OtA5088-OtA5092	OtA5093-OtA5097
Prss29	chr17:25318653-25322684	OtA5088-OtA5092	OtA5093-OtA5097
Prss3	chr6:41373758-41377613	OtA5088-OtA5092	OtA5093-OtA5097
Prss30	chr17:23972126-23975230	OtA5088-OtA5092	OtA5093-OtA5097
Prss32	chr17:23853771-23859776	OtA5088-OtA5092	OtA5093-OtA5097
Prss33	chr17:23833360-23835767	OtA5088-OtA5092	OtA5093-OtA5097
Prss34	chr17:25298393-25300161	OtA5088-OtA5092	OtA5093-OtA5097
Prss37	chr6:40514823-40519508	OtA5088-OtA5092	OtA5093-OtA5097
Prss38	chr11:59372668-59375653	OtA5088-OtA5092	OtA5093-OtA5097
Prss39	chr1:34498429-34503062	OtA5088-OtA5092	OtA5093-OtA5097
Prss40	chr1:34552330-34560943	OtA5088-OtA5092	OtA5093-OtA5097
Prss41	chr17:23836784-23844156	OtA5088-OtA5092	OtA5093-OtA5097
Prss42	chr9:110798184-110803744	OtA5088-OtA5092	OtA5093-OtA5097
Prss43	chr9:110826689-110831504	OtA5088-OtA5092	OtA5093-OtA5097
Prss44	chr9:110813993-110817999	OtA5088-OtA5092	OtA5093-OtA5097
Prss45	chr9:110834587-110841310	OtA5088-OtA5092	OtA5093-OtA5097
Prss46	chr9:110844505-110856522	OtA5088-OtA5092	OtA5093-OtA5097
Prss48	chr3:85993809-86002491	OtA5088-OtA5092	OtA5093-OtA5097
Prss50	chr9:110857966-110864628	OtA5088-OtA5092	OtA5093-OtA5097
Prss51	chr14:64093695-64097672	OtA5088-OtA5092	OtA5093-OtA5097
Prss52	chr14:64104322-64113751	OtA5088-OtA5092	OtA5093-OtA5097
Prss53	chr7:127877700-127890970	OtA5088-OtA5092	OtA5093-OtA5097
Prss54	chr8:95559291-95575197	OtA5088-OtA5092	OtA5093-OtA5097
Prss55	chr14:64075442-64085389	OtA5088-OtA5092	OtA5093-OtA5097
Prss56	chr1:87183492-87188404	OtA5088-OtA5092	OtA5093-OtA5097
Prss57	chr10:79697304-80369637	OtA5088-OtA5092	OtA5093-OtA5097
Prss58	chr6:40529090-40910666	OtA5088-OtA5092	OtA5093-OtA5097

Prtg	chr9:72807273-72917307	OtA5088-OtA5092	OtA5093-OtA5097
Prtn3	chr10:79697304-80369637	OtA5088-OtA5092	OtA5093-OtA5097
Psapl1	chr5:36017180-36398139	OtA5088-OtA5092	OtA5093-OtA5097
Psd	chr19:46304736-46327156	OtA5088-OtA5092	OtA5093-OtA5097
Psd2	chr18:35964834-36014716	OtA5088-OtA5092	OtA5093-OtA5097
Psma8	chr18:14706150-14762299	OtA5088-OtA5092	OtA5093-OtA5097
Psmb11	chr14:54625309-54629556	OtA5088-OtA5092	OtA5093-OtA5097
Psmc3ip	chr11:101087289-101095435	OtA5088-OtA5092	OtA5093-OtA5097
Psors1c2	chr17:35533200-35534648	OtA5088-OtA5092	OtA5093-OtA5097
Pspn	chr17:56999456-57000018	OtA5088-OtA5092	OtA5093-OtA5097
Pstpip2	chr18:77794549-77882879	OtA5088-OtA5092	OtA5093-OtA5097
Ptch2	chr4:117096355-117114831	OtA5088-OtA5092	OtA5093-OtA5097
Ptchd1	chrX:155569735-155623327	OtA5088-OtA5092	OtA5093-OtA5097
Ptchd2	chr4:148236856-148287965	OtA5088-OtA5092	OtA5093-OtA5097
Ptchd3	chr11:121830217-121843436	OtA5088-OtA5092	OtA5093-OtA5097
Ptcra	chr17:46755662-46763712	OtA5088-OtA5092	OtA5093-OtA5097
Pter	chr2:12924040-13010864	OtA5088-OtA5092	OtA5093-OtA5097
Ptf1a	chr2:19445662-19447501	OtA5088-OtA5092	OtA5093-OtA5097
Ptgdr	chr14:44851234-44859375	OtA5088-OtA5092	OtA5093-OtA5097
Ptger1	chr8:83666639-83699179	OtA5088-OtA5092	OtA5093-OtA5097
Ptger2	chr14:44988110-45003820	OtA5088-OtA5092	OtA5093-OtA5097
Ptgfr	chr3:151798609-151837528	OtA5088-OtA5092	OtA5093-OtA5097
Ptgir	chr7:16906489-16910905	OtA5088-OtA5092	OtA5093-OtA5097
Pth	chr7:113385575-113388573	OtA5088-OtA5092	OtA5093-OtA5097
Pth2	chr7:45180994-45181838	OtA5088-OtA5092	OtA5093-OtA5097
Pth2r	chr1:65311256-65389244	OtA5088-OtA5092	OtA5093-OtA5097
Pthlh	chr6:147252109-147264085	OtA5088-OtA5092	OtA5093-OtA5097
Ptpn20	chr14:33589269-33640754	OtA5088-OtA5092	OtA5093-OtA5097
Ptpn22	chr3:103860291-103912247	OtA5088-OtA5092	OtA5093-OtA5097
Ptpn5	chr7:47077799-47133684	OtA5088-OtA5092	OtA5093-OtA5097
Ptpn7	chr1:135132724-135145320	OtA5088-OtA5092	OtA5093-OtA5097
Ptprc	chr1:138062860-138175305	OtA5088-OtA5092	OtA5093-OtA5097
Ptprcap	chr19:4154645-4156710	OtA5088-OtA5092	OtA5093-OtA5097
Ptprd	chr4:75941238-76594299	OtA5088-OtA5092	OtA5093-OtA5097
Ptprh	chr7:4548613-4604041	OtA5088-OtA5092	OtA5093-OtA5097
Ptpro	chr6:137252298-137464633	OtA5088-OtA5092	OtA5093-OtA5097
Ptprq	chr10:107517359-107720027	OtA5088-OtA5092	OtA5093-OtA5097
Ptprt	chr2:161527622-162661147	OtA5088-OtA5092	OtA5093-OtA5097
Ptprv	chr1:135108497-135132575	OtA5088-OtA5092	OtA5093-OtA5097
Ptprz1	chr6:22875501-23052916	OtA5088-OtA5092	OtA5093-OtA5097
Ptx3	chr3:66054825-66296819	OtA5088-OtA5092	OtA5093-OtA5097
Ptx4	chr17:25120759-25125268	OtA5088-OtA5092	OtA5093-OtA5097
Pus3	chr9:35559465-35570069	OtA5088-OtA5092	OtA5093-OtA5097
Pus7l	chr15:94522639-94543507	OtA5088-OtA5092	OtA5093-OtA5097
Pvalb	chr15:78191117-78206351	OtA5088-OtA5092	OtA5093-OtA5097
Pvrl4	chr1:171370172-171388287	OtA5088-OtA5092	OtA5093-OtA5097
Pvt1	chr15:62037986-62250975	OtA5088-OtA5092	OtA5093-OtA5097

Pxt1	chr17:28933985-28942262	OtA5088-OtA5092	OtA5093-OtA5097
Pycard	chr7:127991372-127993867	OtA5088-OtA5092	OtA5093-OtA5097
Pycr1	chr11:120635711-120643670	OtA5088-OtA5092	OtA5093-OtA5097
Pydc3	chr1:173673679-173698392	OtA5088-OtA5092	OtA5093-OtA5097
Pydc4	chr1:173591956-173599274	OtA5088-OtA5092	OtA5093-OtA5097
Pygm	chr19:6384428-6398459	OtA5088-OtA5092	OtA5093-OtA5097
Pygo1	chr9:72925649-72946015	OtA5088-OtA5092	OtA5093-OtA5097
Pyhin1	chr1:173630858-173647928	OtA5088-OtA5092	OtA5093-OtA5097
Pyy	chr11:102106675-102107776	OtA5088-OtA5092	OtA5093-OtA5097
Pzp	chr6:128438756-128526720	OtA5088-OtA5092	OtA5093-OtA5097
Qrfp	chr2:31806167-31810518	OtA5088-OtA5092	OtA5093-OtA5097
Qrfpr	chr3:36179425-36222275	OtA5088-OtA5092	OtA5093-OtA5097
Qrich2	chr11:116441324-116454347	OtA5088-OtA5092	OtA5093-OtA5097
R3hdml	chr2:163492317-163541721	OtA5088-OtA5092	OtA5093-OtA5097
R74862	chr7:143032620-143067930	OtA5088-OtA5092	OtA5093-OtA5097
Rab19	chr6:39381427-39390379	OtA5088-OtA5092	OtA5093-OtA5097
Rab26	chr17:24529053-24533747	OtA5088-OtA5092	OtA5093-OtA5097
Rab27b	chr18:69979130-70141590	OtA5088-OtA5092	OtA5093-OtA5097
Rab33a	chrX:48519469-48530232	OtA5088-OtA5092	OtA5093-OtA5097
Rab36	chr10:75037088-75054100	OtA5088-OtA5092	OtA5093-OtA5097
Rab37	chr11:115091430-115162240	OtA5088-OtA5092	OtA5093-OtA5097
Rab38	chr7:88430272-88491572	OtA5088-OtA5092	OtA5093-OtA5097
Rab39	chr9:53684109-53706232	OtA5088-OtA5092	OtA5093-OtA5097
Rab39b	chrX:75572057-75578204	OtA5088-OtA5092	OtA5093-OtA5097
Rab3c	chr13:110054186-110280206	OtA5088-OtA5092	OtA5093-OtA5097
Rab40b	chr11:121356120-121388251	OtA5088-OtA5092	OtA5093-OtA5097
Rab42-ps	chr4:132302193-132303356	OtA5088-OtA5092	OtA5093-OtA5097
Rab44	chr17:29135055-29148976	OtA5088-OtA5092	OtA5093-OtA5097
Rab9b	chrX:136858150-136868540	OtA5088-OtA5092	OtA5093-OtA5097
Racgap1	chr15:99620495-99651656	OtA5088-OtA5092	OtA5093-OtA5097
Rad18	chr6:112619850-112696670	OtA5088-OtA5092	OtA5093-OtA5097
Rad21l	chr2:151645403-151668533	OtA5088-OtA5092	OtA5093-OtA5097
Rad51	chr2:119112816-119136070	OtA5088-OtA5092	OtA5093-OtA5097
Rad51ap1	chr6:126923418-126939555	OtA5088-OtA5092	OtA5093-OtA5097
Rad51ap2	chr12:11456078-11462928	OtA5088-OtA5092	OtA5093-OtA5097
Rad51c	chr11:87377606-87404495	OtA5088-OtA5092	OtA5093-OtA5097
Rad54b	chr4:11558919-11615808	OtA5088-OtA5092	OtA5093-OtA5097
Rad54l	chr4:116075464-116123690	OtA5088-OtA5092	OtA5093-OtA5097
Rad9b	chr5:122325507-122354195	OtA5088-OtA5092	OtA5093-OtA5097
Radil	chr5:142484840-142551077	OtA5088-OtA5092	OtA5093-OtA5097
Raet1a,Raet1b	chr10:22158608-22374139	OtA5088-OtA5092	OtA5093-OtA5097
Raet1b	chr10:22158608-22374139	OtA5088-OtA5092	OtA5093-OtA5097
Raet1c,Raet1e	chr10:22158608-22374139	OtA5088-OtA5092	OtA5093-OtA5097
Raet1d	chr10:22158608-22374139	OtA5088-OtA5092	OtA5093-OtA5097
Raet1e	chr10:22158608-22374139	OtA5088-OtA5092	OtA5093-OtA5097
Rag1	chr2:101638251-101649532	OtA5088-OtA5092	OtA5093-OtA5097
Rag2	chr2:101560780-101632528	OtA5088-OtA5092	OtA5093-OtA5097

Ralyl	chr3:13471654-14182287	OtA5088-OtA5092	OtA5093-OtA5097
Ranbp3l	chr15:9055681-9067333	OtA5088-OtA5092	OtA5093-OtA5097
Rapgefl1	chr11:98836784-98853005	OtA5088-OtA5092	OtA5093-OtA5097
Rapsn	chr2:91035626-91045729	OtA5088-OtA5092	OtA5093-OtA5097
Rarb	chr14:16430839-16575210	OtA5088-OtA5092	OtA5093-OtA5097
Rasal3	chr17:32390660-32404808	OtA5088-OtA5092	OtA5093-OtA5097
Rasgef1a	chr6:118066384-118091546	OtA5088-OtA5092	OtA5093-OtA5097
Rasgef1c	chr11:49901834-49980223	OtA5088-OtA5092	OtA5093-OtA5097
Rasgrf2	chr13:91880406-91988042	OtA5088-OtA5092	OtA5093-OtA5097
Rasgrp4	chr7:29134932-29156210	OtA5088-OtA5092	OtA5093-OtA5097
Rasl10b	chr11:83410071-83421038	OtA5088-OtA5092	OtA5093-OtA5097
Rasl2-9-ps	chr7:5124941-5125950	OtA5088-OtA5092	OtA5093-OtA5097
Rassf2	chr2:131992849-132029988	OtA5088-OtA5092	OtA5093-OtA5097
Rassf9	chr10:102512221-102546560	OtA5088-OtA5092	OtA5093-OtA5097
Rax	chr18:65934638-65939089	OtA5088-OtA5092	OtA5093-OtA5097
Rbfox1	chr16:5884792-7412480	OtA5088-OtA5092	OtA5093-OtA5097
Rbfox3	chr11:118489759-118909572	OtA5088-OtA5092	OtA5093-OtA5097
Rbm11	chr16:75592890-75602825	OtA5088-OtA5092	OtA5093-OtA5097
Rbm12b	chr4:12140116-12146746	OtA5088-OtA5092	OtA5093-OtA5097
Rbm24	chr13:46418299-46431099	OtA5088-OtA5092	OtA5093-OtA5097
Rbm31y	chrY:17400760-17402718	OtA5088-OtA5092	OtA5093-OtA5097
Rbm43	chr2:51924448-51935009	OtA5088-OtA5092	OtA5093-OtA5097
Rbm44	chr1:91145102-91170795	OtA5088-OtA5092	OtA5093-OtA5097
Rbm46	chr3:82837232-82876483	OtA5088-OtA5092	OtA5093-OtA5097
Rbmx2	chrX:48695003-48710719	OtA5088-OtA5092	OtA5093-OtA5097
Rbmxl2	chr7:107209444-107210916	OtA5088-OtA5092	OtA5093-OtA5097
Rbmy1a1	chrY:3771672-3783271	OtA5088-OtA5092	OtA5093-OtA5097
Rbp3	chr14:33954021-33964216	OtA5088-OtA5092	OtA5093-OtA5097
Rbp7	chr4:149449701-149454968	OtA5088-OtA5092	OtA5093-OtA5097
Rbpjl	chr2:164389392-164415448	OtA5088-OtA5092	OtA5093-OtA5097
Rcan2	chr17:43801850-44039516	OtA5088-OtA5092	OtA5093-OtA5097
Rcvrn	chr11:67695325-67703355	OtA5088-OtA5092	OtA5093-OtA5097
Rd3	chr1:191977372-191988282	OtA5088-OtA5092	OtA5093-OtA5097
Rdh1	chr10:127759762-127768299	OtA5088-OtA5092	OtA5093-OtA5097
Rdh16	chr10:127801152-127815839	OtA5088-OtA5092	OtA5093-OtA5097
Rdh18-ps	chr10:127824215-127846565	OtA5088-OtA5092	OtA5093-OtA5097
Rdh19	chr10:127849927-127861176	OtA5088-OtA5092	OtA5093-OtA5097
Rdh5	chr10:128913590-128919297	OtA5088-OtA5092	OtA5093-OtA5097
Rdh7	chr10:127884026-127888733	OtA5088-OtA5092	OtA5093-OtA5097
Rdh8	chr9:20818503-20826163	OtA5088-OtA5092	OtA5093-OtA5097
Rdh9	chr10:127776404-127792697	OtA5088-OtA5092	OtA5093-OtA5097
Recql4	chr15:76701541-76710559	OtA5088-OtA5092	OtA5093-OtA5097
Reep2	chr18:34840588-34847463	OtA5088-OtA5092	OtA5093-OtA5097
Reg1	chr6:78425982-78428666	OtA5088-OtA5092	OtA5093-OtA5097
Reg2	chr6:78405154-78408097	OtA5088-OtA5092	OtA5093-OtA5097
Reg3a	chr6:78380708-78383839	OtA5088-OtA5092	OtA5093-OtA5097
Reg3b	chr6:78370884-78373466	OtA5088-OtA5092	OtA5093-OtA5097

Reg3d	chr6:78375873-78378865	OtA5088-OtA5092	OtA5093-OtA5097
Reg3g	chr6:78466267-78468874	OtA5088-OtA5092	OtA5093-OtA5097
Reg4	chr3:98222155-98236748	OtA5088-OtA5092	OtA5093-OtA5097
Rell2	chr18:37955558-37969731	OtA5088-OtA5092	OtA5093-OtA5097
Reln	chr5:21884453-22344705	OtA5088-OtA5092	OtA5093-OtA5097
Rem1	chr2:152627007-152635191	OtA5088-OtA5092	OtA5093-OtA5097
Rem2	chr14:54476099-54480434	OtA5088-OtA5092	OtA5093-OtA5097
Ren1	chr1:133350565-133360323	OtA5088-OtA5092	OtA5093-OtA5097
Ren2	chr1:133350565-133360323	OtA5088-OtA5092	OtA5093-OtA5097
Rep15	chr6:147032536-147033518	OtA5088-OtA5092	OtA5093-OtA5097
Rergl	chr6:139493181-139501909	OtA5088-OtA5092	OtA5093-OtA5097
Resp18	chr1:75272201-75278380	OtA5088-OtA5092	OtA5093-OtA5097
Ret	chr6:118151747-118197744	OtA5088-OtA5092	OtA5093-OtA5097
Retn	chr8:3655769-3659818	OtA5088-OtA5092	OtA5093-OtA5097
Retnla	chr16:48842551-48844461	OtA5088-OtA5092	OtA5093-OtA5097
Retnlb	chr16:48816855-48818892	OtA5088-OtA5092	OtA5093-OtA5097
Retnlg	chr16:48872607-48874498	OtA5088-OtA5092	OtA5093-OtA5097
Rex2	chr4:147021849-147060799	OtA5088-OtA5092	OtA5093-OtA5097
Rfpl3s	chr9:55962588-55980932	OtA5088-OtA5092	OtA5093-OtA5097
Rfpl4	chr7:5109786-5116911	OtA5088-OtA5092	OtA5093-OtA5097
Rfpl4b	chr10:38820540-38821779	OtA5088-OtA5092	OtA5093-OtA5097
Rftn2	chr1:55170159-55226782	OtA5088-OtA5092	OtA5093-OtA5097
Rfx3	chr19:27761720-28011166	OtA5088-OtA5092	OtA5093-OtA5097
Rfx4	chr10:84756047-84906538	OtA5088-OtA5092	OtA5093-OtA5097
Rfx6	chr10:51677759-51730429	OtA5088-OtA5092	OtA5093-OtA5097
Rfx8	chr1:39665300-39720989	OtA5088-OtA5092	OtA5093-OtA5097
Rg9mtd2	chr3:138143537-138159820	OtA5088-OtA5092	OtA5093-OtA5097
Rgag1	chrX:143099602-143104335	OtA5088-OtA5092	OtA5093-OtA5097
Rgag4	chrX:101849384-102092055	OtA5088-OtA5092	OtA5093-OtA5097
Rgn	chrX:20549817-20562087	OtA5088-OtA5092	OtA5093-OtA5097
Rgr	chr14:37037489-37048964	OtA5088-OtA5092	OtA5093-OtA5097
Rgs1	chr1:144244668-144249104	OtA5088-OtA5092	OtA5093-OtA5097
Rgs11	chr17:26202961-26211324	OtA5088-OtA5092	OtA5093-OtA5097
Rgs13	chr1:144138666-144177372	OtA5088-OtA5092	OtA5093-OtA5097
Rgs14	chr13:55369731-55384687	OtA5088-OtA5092	OtA5093-OtA5097
Rgs18	chr1:144752840-144775421	OtA5088-OtA5092	OtA5093-OtA5097
Rgs20	chr1:4909575-5070285	OtA5088-OtA5092	OtA5093-OtA5097
Rgs22	chr15:36009476-36140400	OtA5088-OtA5092	OtA5093-OtA5097
Rgs6	chr12:82617037-83158812	OtA5088-OtA5092	OtA5093-OtA5097
Rgs7	chr1:175059075-175492545	OtA5088-OtA5092	OtA5093-OtA5097
Rgs7bp	chr13:104947152-105054930	OtA5088-OtA5092	OtA5093-OtA5097
Rgs8	chr1:153653036-153697665	OtA5088-OtA5092	OtA5093-OtA5097
Rgs9	chr11:109225354-109298181	OtA5088-OtA5092	OtA5093-OtA5097
Rgs9bp	chr7:35578995-35585582	OtA5088-OtA5092	OtA5093-OtA5097
Rgsl1	chr1:153779386-153844141	OtA5088-OtA5092	OtA5093-OtA5097
Rhag	chr17:40811148-40840754	OtA5088-OtA5092	OtA5093-OtA5097
Rhbdl1	chr17:25834464-25837127	OtA5088-OtA5092	OtA5093-OtA5097

Rhbdl2	chr4:123787874-123829904	OtA5088-OtA5092	OtA5093-OtA5097
Rhbdl3	chr11:80300911-80355986	OtA5088-OtA5092	OtA5093-OtA5097
Rhbg	chr3:88242873-88254684	OtA5088-OtA5092	OtA5093-OtA5097
Rhcg	chr7:79593362-79617657	OtA5088-OtA5092	OtA5093-OtA5097
Rhd	chr4:134864535-134896172	OtA5088-OtA5092	OtA5093-OtA5097
Rho	chr6:115931926-115938830	OtA5088-OtA5092	OtA5093-OtA5097
Rhobtb3	chr13:75869536-75943824	OtA5088-OtA5092	OtA5093-OtA5097
Rhoh	chr5:65863568-65896700	OtA5088-OtA5092	OtA5093-OtA5097
Rhox1	chrX:37213804-37222258	OtA5088-OtA5092	OtA5093-OtA5097
Rhox11	chrX:38076597-38085139	OtA5088-OtA5092	OtA5093-OtA5097
Rhox13	chrX:38120839-38129966	OtA5088-OtA5092	OtA5093-OtA5097
Rhox2a	chrX:37244991-37249690	OtA5088-OtA5092	OtA5093-OtA5097
Rhox2b	chrX:37412104-37416806	OtA5088-OtA5092	OtA5093-OtA5097
Rhox2c	chrX:37453658-37458375	OtA5088-OtA5092	OtA5093-OtA5097
Rhox2d	chrX:37493409-37497715	OtA5088-OtA5092	OtA5093-OtA5097
Rhox2e	chrX:37530467-37541809	OtA5088-OtA5092	OtA5093-OtA5097
Rhox2f	chrX:37571420-37576159	OtA5088-OtA5092	OtA5093-OtA5097
Rhox2g	chrX:37639111-37643470	OtA5088-OtA5092	OtA5093-OtA5097
Rhox2h	chrX:37668996-37673277	OtA5088-OtA5092	OtA5093-OtA5097
Rhox3-ps	chrX:37503843-37507806	OtA5088-OtA5092	OtA5093-OtA5097
Rhox3a,Rhox3e	chrX:37249918-37258978	OtA5088-OtA5092	OtA5093-OtA5097
Rhox3c	chrX:37469867-37473779	OtA5088-OtA5092	OtA5093-OtA5097
Rhox3e	chrX:37546974-37550897	OtA5088-OtA5092	OtA5093-OtA5097
Rhox3f	chrX:37581351-37585496	OtA5088-OtA5092	OtA5093-OtA5097
Rhox3g-ps	chrX:37623431-37628269	OtA5088-OtA5092	OtA5093-OtA5097
Rhox3h	chrX:37657687-37668764	OtA5088-OtA5092	OtA5093-OtA5097
Rhox4a	chrX:37390863-37395620	OtA5088-OtA5092	OtA5093-OtA5097
Rhox4b	chrX:37432493-37437278	OtA5088-OtA5092	OtA5093-OtA5097
Rhox4c	chrX:37480336-37485124	OtA5088-OtA5092	OtA5093-OtA5097
Rhox4d	chrX:37514534-37519175	OtA5088-OtA5092	OtA5093-OtA5097
Rhox4e	chrX:37557411-37562282	OtA5088-OtA5092	OtA5093-OtA5097
Rhox4f	chrX:37602894-37607686	OtA5088-OtA5092	OtA5093-OtA5097
Rhox4g	chrX:37646500-37651327	OtA5088-OtA5092	OtA5093-OtA5097
Rhox7	chrX:37831685-37841171	OtA5088-OtA5092	OtA5093-OtA5097
Rhox8	chrX:37874775-37878944	OtA5088-OtA5092	OtA5093-OtA5097
Ribc1	chrX:152004583-152016295	OtA5088-OtA5092	OtA5093-OtA5097
Ribc2	chr15:85132098-85144569	OtA5088-OtA5092	OtA5093-OtA5097
Ric3	chr7:109010879-109083324	OtA5088-OtA5092	OtA5093-OtA5097
Riiad1	chr3:94464984-94473591	OtA5088-OtA5092	OtA5093-OtA5097
Rimbp2	chr5:128760400-128953362	OtA5088-OtA5092	OtA5093-OtA5097
Rimbp3	chr16:17208134-17213982	OtA5088-OtA5092	OtA5093-OtA5097
Rimkla	chr4:119465284-119492598	OtA5088-OtA5092	OtA5093-OtA5097
Rims1	chr1:22468190-22805724	OtA5088-OtA5092	OtA5093-OtA5097
Rims2	chr15:39198285-39684372	OtA5088-OtA5092	OtA5093-OtA5097
Rims3	chr4:120877868-120891560	OtA5088-OtA5092	OtA5093-OtA5097
Rims4	chr2:163863880-163918683	OtA5088-OtA5092	OtA5093-OtA5097
Ripply2	chr9:87015536-87019916	OtA5088-OtA5092	OtA5093-OtA5097

Rit2	chr18:30974313-31317128	OtA5088-OtA5092	OtA5093-OtA5097
Rlbp1	chr7:79374869-79387027	OtA5088-OtA5092	OtA5093-OtA5097
Rln1	chr19:29331754-29334670	OtA5088-OtA5092	OtA5093-OtA5097
Rln3	chr8:84043066-84044979	OtA5088-OtA5092	OtA5093-OtA5097
Rltpr	chr8:105690905-105701095	OtA5088-OtA5092	OtA5093-OtA5097
Rmst	chr10:92081745-92165178	OtA5088-OtA5092	OtA5093-OtA5097
Rn4.5s	chr6:47752751-47752925	OtA5088-OtA5092	OtA5093-OtA5097
Rn45s	chr17:39842996-39848829	OtA5088-OtA5092	OtA5093-OtA5097
Rnase1	chr14:51145001-51146767	OtA5088-OtA5092	OtA5093-OtA5097
Rnase10	chr14:51007750-51010758	OtA5088-OtA5092	OtA5093-OtA5097
Rnase11	chr14:51049516-51050095	OtA5088-OtA5092	OtA5093-OtA5097
Rnase12	chr14:51056782-51057220	OtA5088-OtA5092	OtA5093-OtA5097
Rnase13	chr14:51922159-51922773	OtA5088-OtA5092	OtA5093-OtA5097
Rnase6	chr14:51129067-51131121	OtA5088-OtA5092	OtA5093-OtA5097
Rnase9	chr14:51038458-51041867	OtA5088-OtA5092	OtA5093-OtA5097
Rnaset2a,Rnaset2b	chr17:8128590-8147832	OtA5088-OtA5092	OtA5093-OtA5097
Rnd1	chr15:98669204-98677461	OtA5088-OtA5092	OtA5093-OtA5097
Rnf112	chr11:61448441-61453886	OtA5088-OtA5092	OtA5093-OtA5097
Rnf122	chr8:31111845-31131473	OtA5088-OtA5092	OtA5093-OtA5097
Rnf133	chr6:23262773-23839421	OtA5088-OtA5092	OtA5093-OtA5097
Rnf148	chr6:23262773-23839421	OtA5088-OtA5092	OtA5093-OtA5097
Rnf150	chr8:82863355-83091271	OtA5088-OtA5092	OtA5093-OtA5097
Rnf151	chr17:24715839-24718057	OtA5088-OtA5092	OtA5093-OtA5097
Rnf152	chr1:105276916-105356710	OtA5088-OtA5092	OtA5093-OtA5097
Rnf165	chr18:77456109-77565136	OtA5088-OtA5092	OtA5093-OtA5097
Rnf17	chr14:56402696-56525031	OtA5088-OtA5092	OtA5093-OtA5097
Rnf180	chr13:105147493-105293014	OtA5088-OtA5092	OtA5093-OtA5097
Rnf182	chr13:43615796-43670944	OtA5088-OtA5092	OtA5093-OtA5097
Rnf183	chr4:62427541-62434726	OtA5088-OtA5092	OtA5093-OtA5097
Rnf186	chr4:138967111-138968366	OtA5088-OtA5092	OtA5093-OtA5097
Rnf207	chr4:152297213-152318625	OtA5088-OtA5092	OtA5093-OtA5097
Rnf219	chr14:104477533-104522666	OtA5088-OtA5092	OtA5093-OtA5097
Rnf32	chr5:29198005-29225291	OtA5088-OtA5092	OtA5093-OtA5097
Rnf43	chr11:87663086-87735539	OtA5088-OtA5092	OtA5093-OtA5097
Rnls	chr19:33137744-33392295	OtA5088-OtA5092	OtA5093-OtA5097
Rnu11	chr4:132270078-132270186	OtA5088-OtA5092	OtA5093-OtA5097
Rnu6	chr19:14438479-14438506	OtA5088-OtA5092	OtA5093-OtA5097
Rnu7	chr5:53698484-53707704	OtA5088-OtA5092	OtA5093-OtA5097
Rnu73b	chr3:86137939-86142668	OtA5088-OtA5092	OtA5093-OtA5097
Robo2	chr16:73892305-74410912	OtA5088-OtA5092	OtA5093-OtA5097
Robo3	chr9:37416044-37433175	OtA5088-OtA5092	OtA5093-OtA5097
Ropn1	chr16:34651210-34678610	OtA5088-OtA5092	OtA5093-OtA5097
Rorb	chr19:18930608-19111196	OtA5088-OtA5092	OtA5093-OtA5097
Rorc	chr3:94372825-94398239	OtA5088-OtA5092	OtA5093-OtA5097
Ros1	chr10:52045925-52195244	OtA5088-OtA5092	OtA5093-OtA5097
Rp1	chr1:4290845-4409241	OtA5088-OtA5092	OtA5093-OtA5097
Rp1l1	chr14:63992430-64033506	OtA5088-OtA5092	OtA5093-OtA5097

Rpe65	chr3:159599180-159625307	OtA5088-OtA5092	OtA5093-OtA5097
Rpgr	chrX:10158215-10216795	OtA5088-OtA5092	OtA5093-OtA5097
Rpgrip1	chr14:52110902-52197239	OtA5088-OtA5092	OtA5093-OtA5097
Rpgrip1l	chr8:91217029-91313222	OtA5088-OtA5092	OtA5093-OtA5097
Rph3al	chr11:75832157-75911026	OtA5088-OtA5092	OtA5093-OtA5097
Rpl10l	chr12:66283378-66284401	OtA5088-OtA5092	OtA5093-OtA5097
Rpl31-ps12	chr16:16753015-16829363	OtA5088-OtA5092	OtA5093-OtA5097
Rpl39l	chr16:10170227-10174911	OtA5088-OtA5092	OtA5093-OtA5097
Rpl3l	chr17:24727828-24736149	OtA5088-OtA5092	OtA5093-OtA5097
Rpp25	chr9:57504101-57505447	OtA5088-OtA5092	OtA5093-OtA5097
Rpp40	chr13:35895103-35906347	OtA5088-OtA5092	OtA5093-OtA5097
Rprl1	chr6:69326936-69327174	OtA5088-OtA5092	OtA5093-OtA5097
Rprl2	chr3:22251369-22251607	OtA5088-OtA5092	OtA5093-OtA5097
Rprl3	chr8:3803124-3803361	OtA5088-OtA5092	OtA5093-OtA5097
Rprm	chr2:54084092-54085552	OtA5088-OtA5092	OtA5093-OtA5097
Rps15a-ps4	chr4:132219892-132220589	OtA5088-OtA5092	OtA5093-OtA5097
Rps15a-ps6	chr11:6105691-6200451	OtA5088-OtA5092	OtA5093-OtA5097
Rps6ka5	chr12:100549777-100725028	OtA5088-OtA5092	OtA5093-OtA5097
Rps6kl1	chr12:85135595-85151264	OtA5088-OtA5092	OtA5093-OtA5097
Rptn	chr3:93393698-93399442	OtA5088-OtA5092	OtA5093-OtA5097
Rragb	chrX:153139957-153171943	OtA5088-OtA5092	OtA5093-OtA5097
Rrh	chr3:129808574-129822505	OtA5088-OtA5092	OtA5093-OtA5097
Rs1	chrX:160768012-160994681	OtA5088-OtA5092	OtA5093-OtA5097
Rsc1a1	chr4:141683562-141723419	OtA5088-OtA5092	OtA5093-OtA5097
Rsl1	chr13:67173181-67183498	OtA5088-OtA5092	OtA5093-OtA5097
Rslcan18	chr13:67069397-67114028	OtA5088-OtA5092	OtA5093-OtA5097
Rsph1	chr17:31255019-31277356	OtA5088-OtA5092	OtA5093-OtA5097
Rsph4a	chr10:33905110-33916021	OtA5088-OtA5092	OtA5093-OtA5097
Rsph6a	chr7:19054686-19074447	OtA5088-OtA5092	OtA5093-OtA5097
Rsph9	chr17:46129276-46144198	OtA5088-OtA5092	OtA5093-OtA5097
Rspo1	chr4:124986429-125009099	OtA5088-OtA5092	OtA5093-OtA5097
Rspo2	chr15:43020794-43170818	OtA5088-OtA5092	OtA5093-OtA5097
Rspo3	chr10:29453106-29535867	OtA5088-OtA5092	OtA5093-OtA5097
Rspo4	chr2:151842926-151874668	OtA5088-OtA5092	OtA5093-OtA5097
Rtbdn	chr8:84946990-84956603	OtA5088-OtA5092	OtA5093-OtA5097
Rtdr1	chr10:74957476-75032586	OtA5088-OtA5092	OtA5093-OtA5097
Rtkn2	chr10:67979597-68043864	OtA5088-OtA5092	OtA5093-OtA5097
Rtn4r	chr16:18127705-18152408	OtA5088-OtA5092	OtA5093-OtA5097
Rtn4rl2	chr2:84871945-84886692	OtA5088-OtA5092	OtA5093-OtA5097
Rtp1	chr16:23429132-23433960	OtA5088-OtA5092	OtA5093-OtA5097
Rtp2	chr16:23925547-23930794	OtA5088-OtA5092	OtA5093-OtA5097
Rtp3	chr9:110984942-110989713	OtA5088-OtA5092	OtA5093-OtA5097
Rttn	chr18:88971789-89131014	OtA5088-OtA5092	OtA5093-OtA5097
Rubie	chr14:46568330-46575851	OtA5088-OtA5092	OtA5093-OtA5097
Rundc3a	chr11:102393402-102402939	OtA5088-OtA5092	OtA5093-OtA5097
Rundc3b	chr5:8490335-8622952	OtA5088-OtA5092	OtA5093-OtA5097
Runx1t1	chr4:13743301-13893643	OtA5088-OtA5092	OtA5093-OtA5097

Runx2	chr17:44604000-45119284	OtA5088-OtA5092	OtA5093-OtA5097
Runx3	chr4:135120644-135177990	OtA5088-OtA5092	OtA5093-OtA5097
Rwdd3	chr3:121155401-121171695	OtA5088-OtA5092	OtA5093-OtA5097
Rxfp1	chr3:79644715-79737794	OtA5088-OtA5092	OtA5093-OtA5097
Rxfp2	chr5:150018674-150082186	OtA5088-OtA5092	OtA5093-OtA5097
Rxfp3	chr15:11033716-11037968	OtA5088-OtA5092	OtA5093-OtA5097
Rxfp4	chr3:88651897-88653142	OtA5088-OtA5092	OtA5093-OtA5097
Rxrg	chr1:167598361-167639623	OtA5088-OtA5092	OtA5093-OtA5097
Ryr1	chr7:29003339-29125151	OtA5088-OtA5092	OtA5093-OtA5097
Ryr2	chr13:11553102-12106945	OtA5088-OtA5092	OtA5093-OtA5097
Ryr3	chr2:112631381-113030331	OtA5088-OtA5092	OtA5093-OtA5097
S100a2	chr3:90590246-90591508	OtA5088-OtA5092	OtA5093-OtA5097
S100a3	chr3:90600214-90602702	OtA5088-OtA5092	OtA5093-OtA5097
S100a5	chr3:90608521-90611780	OtA5088-OtA5092	OtA5093-OtA5097
S100a7a	chr3:90654301-90658130	OtA5088-OtA5092	OtA5093-OtA5097
S100b	chr10:76253835-76261319	OtA5088-OtA5092	OtA5093-OtA5097
S100z	chr13:95477300-95478655	OtA5088-OtA5092	OtA5093-OtA5097
S1pr4	chr10:81497744-81500137	OtA5088-OtA5092	OtA5093-OtA5097
S1pr5	chr9:21242916-21248443	OtA5088-OtA5092	OtA5093-OtA5097
Saa1	chr7:46740498-46742980	OtA5088-OtA5092	OtA5093-OtA5097
Saa2	chr7:46751832-46754314	OtA5088-OtA5092	OtA5093-OtA5097
Saa4	chr7:46727998-46732543	OtA5088-OtA5092	OtA5093-OtA5097
Sacs	chr14:61138456-61258490	OtA5088-OtA5092	OtA5093-OtA5097
Sag	chr1:87803679-87845157	OtA5088-OtA5092	OtA5093-OtA5097
Sall2	chr14:52311176-52328670	OtA5088-OtA5092	OtA5093-OtA5097
Sall3	chr18:80966373-80986578	OtA5088-OtA5092	OtA5093-OtA5097
Samd11	chr4:156236009-156255338	OtA5088-OtA5092	OtA5093-OtA5097
Samd3	chr10:26229706-26272172	OtA5088-OtA5092	OtA5093-OtA5097
Samd7	chr3:30746292-30767174	OtA5088-OtA5092	OtA5093-OtA5097
Samsn1	chr16:75858793-75909266	OtA5088-OtA5092	OtA5093-OtA5097
Samt1	chrX:154022779-154033829	OtA5088-OtA5092	OtA5093-OtA5097
Samt2	chrX:154575227-154579330	OtA5088-OtA5092	OtA5093-OtA5097
Samt3	chrX:86044198-86047313	OtA5088-OtA5092	OtA5093-OtA5097
Samt4	chrX:154482001-154484682	OtA5088-OtA5092	OtA5093-OtA5097
Sap25	chr5:137629122-137642902	OtA5088-OtA5092	OtA5093-OtA5097
Sarm1	chr11:78472329-78497754	OtA5088-OtA5092	OtA5093-OtA5097
Sash3	chrX:48146526-48161563	OtA5088-OtA5092	OtA5093-OtA5097
Sass6	chr3:116562972-116630984	OtA5088-OtA5092	OtA5093-OtA5097
Satb1	chr17:51736186-51833290	OtA5088-OtA5092	OtA5093-OtA5097
Satl1	chrX:112384304-112406779	OtA5088-OtA5092	OtA5093-OtA5097
Sbk2	chr7:4957080-4964348	OtA5088-OtA5092	OtA5093-OtA5097
Sbp	chr17:23941995-23945607	OtA5088-OtA5092	OtA5093-OtA5097
Sbpl	chr17:23953084-23955273	OtA5088-OtA5092	OtA5093-OtA5097
Scai	chr2:39066214-39190730	OtA5088-OtA5092	OtA5093-OtA5097
Scara3	chr14:65919394-65953744	OtA5088-OtA5092	OtA5093-OtA5097
Scarna10	chr6:125168006-125191586	OtA5088-OtA5092	OtA5093-OtA5097
Scarna17	chr18:74778393-74778455	OtA5088-OtA5092	OtA5093-OtA5097

Scarna2	chr3:108554341-108554402	OtA5088-OtA5092	OtA5093-OtA5097
Scarna3b	chr12:80335846-80436601	OtA5088-OtA5092	OtA5093-OtA5097
Scarna8	chr4:86581284-86612022	OtA5088-OtA5092	OtA5093-OtA5097
Scarna9	chr9:15283336-15357788	OtA5088-OtA5092	OtA5093-OtA5097
Scd3	chr19:44203287-44244016	OtA5088-OtA5092	OtA5093-OtA5097
Scg2	chr1:79434668-79440090	OtA5088-OtA5092	OtA5093-OtA5097
Scg3	chr9:75643365-75684056	OtA5088-OtA5092	OtA5093-OtA5097
Scgb1c1	chr7:140835495-140846769	OtA5088-OtA5092	OtA5093-OtA5097
Scgb2b1-ps	chr7:33625144-33653281	OtA5088-OtA5092	OtA5093-OtA5097
Scgb3a1	chr11:49663609-49665118	OtA5088-OtA5092	OtA5093-OtA5097
Scgb3a2	chr18:43764326-43767399	OtA5088-OtA5092	OtA5093-OtA5097
Scgn	chr13:23953456-23991214	OtA5088-OtA5092	OtA5093-OtA5097
Scin	chr12:40059770-40134228	OtA5088-OtA5092	OtA5093-OtA5097
Sclt1	chr3:41626704-41742514	OtA5088-OtA5092	OtA5093-OtA5097
Scml2	chrX:161162771-161258213	OtA5088-OtA5092	OtA5093-OtA5097
Scml4	chr10:42860511-42960782	OtA5088-OtA5092	OtA5093-OtA5097
Scn10a	chr9:119608455-119719032	OtA5088-OtA5092	OtA5093-OtA5097
Scn11a	chr9:119753764-119825456	OtA5088-OtA5092	OtA5093-OtA5097
Scn1a	chr2:66270781-66440837	OtA5088-OtA5092	OtA5093-OtA5097
Scn2a1	chr2:65670444-65767447	OtA5088-OtA5092	OtA5093-OtA5097
Scn2b	chr9:45079182-45135606	OtA5088-OtA5092	OtA5093-OtA5097
Scn3a	chr2:65457117-65567492	OtA5088-OtA5092	OtA5093-OtA5097
Scn3b	chr9:40269215-40291618	OtA5088-OtA5092	OtA5093-OtA5097
Scn4a	chr11:106318592-106349390	OtA5088-OtA5092	OtA5093-OtA5097
Scn4b	chr9:45139041-45154061	OtA5088-OtA5092	OtA5093-OtA5097
Scn7a	chr2:66673425-66784910	OtA5088-OtA5092	OtA5093-OtA5097
Scn8a	chr15:100870682-101045929	OtA5088-OtA5092	OtA5093-OtA5097
Scrg1	chr8:57455922-57477585	OtA5088-OtA5092	OtA5093-OtA5097
Scrt1	chr15:76516202-76522129	OtA5088-OtA5092	OtA5093-OtA5097
Scrt2	chr2:152081528-152095802	OtA5088-OtA5092	OtA5093-OtA5097
Scube1	chr15:83602582-83725021	OtA5088-OtA5092	OtA5093-OtA5097
Scx	chr15:76380512-76477269	OtA5088-OtA5092	OtA5093-OtA5097
Sdr16c5	chr4:3995941-4019663	OtA5088-OtA5092	OtA5093-OtA5097
Sdr16c6	chr4:4056665-4077514	OtA5088-OtA5092	OtA5093-OtA5097
Sdr9c7	chr10:127898534-127911759	OtA5088-OtA5092	OtA5093-OtA5097
Sds	chr5:120476546-120503623	OtA5088-OtA5092	OtA5093-OtA5097
Sdsl	chr5:120458201-120472763	OtA5088-OtA5092	OtA5093-OtA5097
Sebox	chr11:78503512-78505081	OtA5088-OtA5092	OtA5093-OtA5097
Sec1	chr7:45677686-45679558	OtA5088-OtA5092	OtA5093-OtA5097
Sec14l3	chr11:4064852-4078990	OtA5088-OtA5092	OtA5093-OtA5097
Sec14l4	chr11:4031781-4048009	OtA5088-OtA5092	OtA5093-OtA5097
Sec14l5	chr16:5147108-5183934	OtA5088-OtA5092	OtA5093-OtA5097
Sec31b	chr19:44516956-44545848	OtA5088-OtA5092	OtA5093-OtA5097
Sectm1a	chr11:121067402-121081139	OtA5088-OtA5092	OtA5093-OtA5097
Sectm1b	chr11:121053422-121063569	OtA5088-OtA5092	OtA5093-OtA5097
Sel1l2	chr2:140229857-140389710	OtA5088-OtA5092	OtA5093-OtA5097
Sell	chr1:164062075-164080785	OtA5088-OtA5092	OtA5093-OtA5097

Selp	chr1:164115263-164220274	OtA5088-OtA5092	OtA5093-OtA5097
Sema3a	chr5:13396783-13603485	OtA5088-OtA5092	OtA5093-OtA5097
Sema3d	chr5:12383165-12588943	OtA5088-OtA5092	OtA5093-OtA5097
Sema4f	chr6:82911884-82939750	OtA5088-OtA5092	OtA5093-OtA5097
Sema5b	chr16:35541361-35664258	OtA5088-OtA5092	OtA5093-OtA5097
Senp8	chr9:59734258-59750649	OtA5088-OtA5092	OtA5093-OtA5097
1-Sep	chr7:127214441-127218445	OtA5088-OtA5092	OtA5093-OtA5097
12-Sep	chr16:4986857-4997852	OtA5088-OtA5092	OtA5093-OtA5097
14-Sep	chr5:129683390-129708511	OtA5088-OtA5092	OtA5093-OtA5097
3-Sep	chr15:82274934-82294442	OtA5088-OtA5092	OtA5093-OtA5097
Serac1	chr17:5941279-6079739	OtA5088-OtA5092	OtA5093-OtA5097
Serinc4	chr2:121449227-121456764	OtA5088-OtA5092	OtA5093-OtA5097
Serpina11	chr12:103980242-103989957	OtA5088-OtA5092	OtA5093-OtA5097
Serpina12	chr12:104028768-104044443	OtA5088-OtA5092	OtA5093-OtA5097
Serpina1d	chr12:103763586-103773633	OtA5088-OtA5092	OtA5093-OtA5097
Serpina1f	chr12:103688043-103695529	OtA5088-OtA5092	OtA5093-OtA5097
Serpina3a	chr12:104112723-104121896	OtA5088-OtA5092	OtA5093-OtA5097
Serpina3b	chr12:104127995-104139545	OtA5088-OtA5092	OtA5093-OtA5097
Serpina3c	chr12:104146906-104153872	OtA5088-OtA5092	OtA5093-OtA5097
Serpina3f	chr12:104214543-104241934	OtA5088-OtA5092	OtA5093-OtA5097
Serpina3g	chr12:104214543-104241934	OtA5088-OtA5092	OtA5093-OtA5097
Serpina3h	chr12:104247895-104254405	OtA5088-OtA5092	OtA5093-OtA5097
Serpina3i	chr12:104263121-104269365	OtA5088-OtA5092	OtA5093-OtA5097
Serpina3j	chr12:104314569-104320586	OtA5088-OtA5092	OtA5093-OtA5097
Serpina3k	chr12:104338485-104345739	OtA5088-OtA5092	OtA5093-OtA5097
Serpina3m	chr12:104387163-104394257	OtA5088-OtA5092	OtA5093-OtA5097
Serpina4-ps1	chr12:104077958-104086920	OtA5088-OtA5092	OtA5093-OtA5097
Serpina6	chr12:103646631-103657071	OtA5088-OtA5092	OtA5093-OtA5097
Serpina7	chrX:139079249-139085236	OtA5088-OtA5092	OtA5093-OtA5097
Serpina9	chr12:103996619-104013652	OtA5088-OtA5092	OtA5093-OtA5097
Serpinb10-ps	chr1:107529002-107549271	OtA5088-OtA5092	OtA5093-OtA5097
Serpinb11	chr1:107362313-107380475	OtA5088-OtA5092	OtA5093-OtA5097
Serpinb12	chr1:106934448-106957080	OtA5088-OtA5092	OtA5093-OtA5097
Serpinb13	chr1:106980983-107001195	OtA5088-OtA5092	OtA5093-OtA5097
Serpinb1b	chr13:33084102-33094380	OtA5088-OtA5092	OtA5093-OtA5097
Serpinb1c	chr13:32881396-32898140	OtA5088-OtA5092	OtA5093-OtA5097
Serpinb2	chr1:107511422-107525600	OtA5088-OtA5092	OtA5093-OtA5097
Serpinb3a	chr1:107045586-107052303	OtA5088-OtA5092	OtA5093-OtA5097
Serpinb3b	chr1:107153960-107161114	OtA5088-OtA5092	OtA5093-OtA5097
Serpinb3c	chr1:107271200-107278371	OtA5088-OtA5092	OtA5093-OtA5097
Serpinb3d	chr1:107078192-107083480	OtA5088-OtA5092	OtA5093-OtA5097
Serpinb5	chr1:106861179-106883348	OtA5088-OtA5092	OtA5093-OtA5097
Serpinb6d	chr13:33661404-33671585	OtA5088-OtA5092	OtA5093-OtA5097
Serpinb6e	chr13:33832344-33843408	OtA5088-OtA5092	OtA5093-OtA5097
Serpinb7	chr1:107422688-107452689	OtA5088-OtA5092	OtA5093-OtA5097
Serpinb8	chr1:107590005-107608978	OtA5088-OtA5092	OtA5093-OtA5097
Serpinc1	chr1:160978605-161003014	OtA5088-OtA5092	OtA5093-OtA5097

Serpine3	chr14:62663666-62830126	OtA5088-OtA5092	OtA5093-OtA5097
Serpini1	chr3:75557532-75642523	OtA5088-OtA5092	OtA5093-OtA5097
Serpini2	chr3:75242370-75270078	OtA5088-OtA5092	OtA5093-OtA5097
Sertad4	chr1:192844487-192855752	OtA5088-OtA5092	OtA5093-OtA5097
Setmar	chr6:108065045-108077120	OtA5088-OtA5092	OtA5093-OtA5097
Sez6	chr11:77930942-77979042	OtA5088-OtA5092	OtA5093-OtA5097
Sez6l2	chr7:126950534-126970606	OtA5088-OtA5092	OtA5093-OtA5097
Sfrp2	chr3:83766320-83774314	OtA5088-OtA5092	OtA5093-OtA5097
Sfta2	chr17:35649707-35650569	OtA5088-OtA5092	OtA5093-OtA5097
Sftpa1	chr14:41131787-41136373	OtA5088-OtA5092	OtA5093-OtA5097
Sftpb	chr6:72303247-72314370	OtA5088-OtA5092	OtA5093-OtA5097
Sftpc	chr14:70520941-70524081	OtA5088-OtA5092	OtA5093-OtA5097
Sfxn4	chr19:60837276-60861430	OtA5088-OtA5092	OtA5093-OtA5097
Sgca	chr11:94962821-94976327	OtA5088-OtA5092	OtA5093-OtA5097
Sgcd	chr11:46978782-47379302	OtA5088-OtA5092	OtA5093-OtA5097
Sgcg	chr14:61138456-61258490	OtA5088-OtA5092	OtA5093-OtA5097
Sgcz	chr8:37522553-38661508	OtA5088-OtA5092	OtA5093-OtA5097
Sgip1	chr4:102760362-103011965	OtA5088-OtA5092	OtA5093-OtA5097
Sgol1	chr17:53674786-53689315	OtA5088-OtA5092	OtA5093-OtA5097
Sgol2	chr1:57985339-58026277	OtA5088-OtA5092	OtA5093-OtA5097
Sgtb	chr13:104109789-104141441	OtA5088-OtA5092	OtA5093-OtA5097
Sh2d1a	chrX:42502607-42522075	OtA5088-OtA5092	OtA5093-OtA5097
Sh2d1b1	chr1:170277323-170285662	OtA5088-OtA5092	OtA5093-OtA5097
Sh2d1b2	chr1:170232870-170251587	OtA5088-OtA5092	OtA5093-OtA5097
Sh2d2a	chr3:87846754-87855722	OtA5088-OtA5092	OtA5093-OtA5097
Sh2d7	chr9:54538983-54545020	OtA5088-OtA5092	OtA5093-OtA5097
Sh3bgr	chr16:96200469-96228933	OtA5088-OtA5092	OtA5093-OtA5097
Sh3gl2	chr4:85205455-85389379	OtA5088-OtA5092	OtA5093-OtA5097
Sh3rf3	chr10:58813358-59138916	OtA5088-OtA5092	OtA5093-OtA5097
Shank1	chr7:44310263-44358353	OtA5088-OtA5092	OtA5093-OtA5097
Shank2	chr7:143948404-144470935	OtA5088-OtA5092	OtA5093-OtA5097
Shbg	chr11:69614815-69617905	OtA5088-OtA5092	OtA5093-OtA5097
Shc3	chr13:51431041-51567084	OtA5088-OtA5092	OtA5093-OtA5097
Shc4	chr2:125627446-125724148	OtA5088-OtA5092	OtA5093-OtA5097
Shcbp1	chr8:4735979-4779534	OtA5088-OtA5092	OtA5093-OtA5097
Shd	chr17:55970481-55976619	OtA5088-OtA5092	OtA5093-OtA5097
Shh	chr5:28456839-28719209	OtA5088-OtA5092	OtA5093-OtA5097
Shisa6	chr11:66211724-66526126	OtA5088-OtA5092	OtA5093-OtA5097
Shisa7	chr7:4825551-4844696	OtA5088-OtA5092	OtA5093-OtA5097
Shisa9	chr16:11984112-12270904	OtA5088-OtA5092	OtA5093-OtA5097
Shox2	chr3:66973265-66981771	OtA5088-OtA5092	OtA5093-OtA5097
Siah1b	chrX:164070702-164076049	OtA5088-OtA5092	OtA5093-OtA5097
Siah3	chr14:75455981-75526141	OtA5088-OtA5092	OtA5093-OtA5097
Sidt1	chr16:44240179-44332838	OtA5088-OtA5092	OtA5093-OtA5097
Siglec15	chr18:78043613-78057268	OtA5088-OtA5092	OtA5093-OtA5097
Siglec5	chr7:43351404-43359470	OtA5088-OtA5092	OtA5093-OtA5097
Siglece	chr7:43651069-43660161	OtA5088-OtA5092	OtA5093-OtA5097

Siglecg	chr7:43408279-43418349	OtA5088-OtA5092	OtA5093-OtA5097
Siglech	chr7:55768177-55778833	OtA5088-OtA5092	OtA5093-OtA5097
Sim1	chr10:50895650-50989152	OtA5088-OtA5092	OtA5093-OtA5097
Sim2	chr16:94085259-94127032	OtA5088-OtA5092	OtA5093-OtA5097
Sirpb1a	chr3:15371826-15426427	OtA5088-OtA5092	OtA5093-OtA5097
Sirpb1b	chr3:15495753-15575065	OtA5088-OtA5092	OtA5093-OtA5097
Sis	chr3:72888559-72966866	OtA5088-OtA5092	OtA5093-OtA5097
Sit1	chr4:43482082-43483709	OtA5088-OtA5092	OtA5093-OtA5097
Six1	chr12:73041826-73046712	OtA5088-OtA5092	OtA5093-OtA5097
Six2	chr17:85684267-85688254	OtA5088-OtA5092	OtA5093-OtA5097
Six3	chr17:85620833-85626191	OtA5088-OtA5092	OtA5093-OtA5097
Six3os1	chr17:85601931-85618396	OtA5088-OtA5092	OtA5093-OtA5097
Six4	chr12:73100258-73113245	OtA5088-OtA5092	OtA5093-OtA5097
Six6	chr12:72939944-72944899	OtA5088-OtA5092	OtA5093-OtA5097
Ska1	chr18:74195298-74207818	OtA5088-OtA5092	OtA5093-OtA5097
Skap1	chr11:96464590-96759622	OtA5088-OtA5092	OtA5093-OtA5097
Skint1	chr4:112006268-112029538	OtA5088-OtA5092	OtA5093-OtA5097
Skint10	chr4:112710829-112774861	OtA5088-OtA5092	OtA5093-OtA5097
Skint11	chr4:114163383-114245028	OtA5088-OtA5092	OtA5093-OtA5097
Skint2	chr4:112557193-112647840	OtA5088-OtA5092	OtA5093-OtA5097
Skint3	chr4:112232244-112300468	OtA5088-OtA5092	OtA5093-OtA5097
Skint4	chr4:112081469-112168076	OtA5088-OtA5092	OtA5093-OtA5097
Skint5	chr4:113477890-113999503	OtA5088-OtA5092	OtA5093-OtA5097
Skint6	chr4:112804615-113286973	OtA5088-OtA5092	OtA5093-OtA5097
Skint7	chr4:111972926-111988221	OtA5088-OtA5092	OtA5093-OtA5097
Skint8	chr4:111919554-111950356	OtA5088-OtA5092	OtA5093-OtA5097
Skint9	chr4:112385968-112433985	OtA5088-OtA5092	OtA5093-OtA5097
Skor1	chr9:63138163-63148961	OtA5088-OtA5092	OtA5093-OtA5097
Skor2	chr18:76856404-76900342	OtA5088-OtA5092	OtA5093-OtA5097
Sla	chr15:66670769-66850720	OtA5088-OtA5092	OtA5093-OtA5097
Sla2	chr2:156863121-156887078	OtA5088-OtA5092	OtA5093-OtA5097
Slain1	chr14:103650242-103704799	OtA5088-OtA5092	OtA5093-OtA5097
Slamf1	chr1:171767131-171801184	OtA5088-OtA5092	OtA5093-OtA5097
Slamf6	chr1:171917536-171943868	OtA5088-OtA5092	OtA5093-OtA5097
Slamf7	chr1:171632402-171653037	OtA5088-OtA5092	OtA5093-OtA5097
Slamf8	chr1:172581376-172590568	OtA5088-OtA5092	OtA5093-OtA5097
Slamf9	chr1:172475359-172478409	OtA5088-OtA5092	OtA5093-OtA5097
Slc10a1	chr12:80953184-80968079	OtA5088-OtA5092	OtA5093-OtA5097
Slc10a2	chr8:5085622-5105232	OtA5088-OtA5092	OtA5093-OtA5097
Slc10a4	chr5:73006903-73012955	OtA5088-OtA5092	OtA5093-OtA5097
Slc10a5	chr3:10331733-10335656	OtA5088-OtA5092	OtA5093-OtA5097
Slc12a1	chr2:125152599-125230001	OtA5088-OtA5092	OtA5093-OtA5097
Slc12a3	chr8:94329207-94366221	OtA5088-OtA5092	OtA5093-OtA5097
Slc12a5	chr2:164967987-164999731	OtA5088-OtA5092	OtA5093-OtA5097
Slc12a8	chr16:33518328-33664135	OtA5088-OtA5092	OtA5093-OtA5097
Slc13a1	chr6:24088282-24168092	OtA5088-OtA5092	OtA5093-OtA5097
Slc13a2	chr11:78394484-78422185	OtA5088-OtA5092	OtA5093-OtA5097

Slc13a5	chr11:72241993-72266604	OtA5088-OtA5092	OtA5093-OtA5097
Slc14a2	chr18:78146143-78596950	OtA5088-OtA5092	OtA5093-OtA5097
Slc15a1	chr14:121459620-121505254	OtA5088-OtA5092	OtA5093-OtA5097
Slc15a5	chr6:137983589-138079916	OtA5088-OtA5092	OtA5093-OtA5097
Slc16a11	chr11:70213909-70216414	OtA5088-OtA5092	OtA5093-OtA5097
Slc16a14	chr1:84906704-84935083	OtA5088-OtA5092	OtA5093-OtA5097
Slc16a2	chrX:103697413-103821988	OtA5088-OtA5092	OtA5093-OtA5097
Slc16a5	chr11:115462472-115474398	OtA5088-OtA5092	OtA5093-OtA5097
Slc16a7	chr10:125227484-125328535	OtA5088-OtA5092	OtA5093-OtA5097
Slc16a8	chr15:79251015-79254748	OtA5088-OtA5092	OtA5093-OtA5097
Slc17a1	chr13:23870272-23895730	OtA5088-OtA5092	OtA5093-OtA5097
Slc17a2	chr13:23807026-23823525	OtA5088-OtA5092	OtA5093-OtA5097
Slc17a3	chr13:23839433-23860714	OtA5088-OtA5092	OtA5093-OtA5097
Slc17a4	chr13:23897888-23915007	OtA5088-OtA5092	OtA5093-OtA5097
Slc17a6	chr7:51621829-51671126	OtA5088-OtA5092	OtA5093-OtA5097
Slc17a7	chr7:45163920-45176139	OtA5088-OtA5092	OtA5093-OtA5097
Slc17a8	chr10:89574019-89621249	OtA5088-OtA5092	OtA5093-OtA5097
Slc17a9	chr2:180725338-180742278	OtA5088-OtA5092	OtA5093-OtA5097
Slc18a3	chr14:32408202-32465909	OtA5088-OtA5092	OtA5093-OtA5097
Slc1a2	chr2:102658682-102790784	OtA5088-OtA5092	OtA5093-OtA5097
Slc1a3	chr15:8634123-8710807	OtA5088-OtA5092	OtA5093-OtA5097
Slc1a6	chr10:78780495-78814825	OtA5088-OtA5092	OtA5093-OtA5097
Slc1a7	chr4:107968333-108013532	OtA5088-OtA5092	OtA5093-OtA5097
Slc22a1	chr17:12648874-12675838	OtA5088-OtA5092	OtA5093-OtA5097
Slc22a12	chr19:6535855-6543070	OtA5088-OtA5092	OtA5093-OtA5097
Slc22a13	chr9:119192977-119209105	OtA5088-OtA5092	OtA5093-OtA5097
Slc22a13b	chr9:119220490-119231692	OtA5088-OtA5092	OtA5093-OtA5097
Slc22a14	chr9:119169455-119190393	OtA5088-OtA5092	OtA5093-OtA5097
Slc22a16	chr10:40570361-40604132	OtA5088-OtA5092	OtA5093-OtA5097
Slc22a19	chr19:7673075-7711310	OtA5088-OtA5092	OtA5093-OtA5097
Slc22a20	chr19:5970233-5986143	OtA5088-OtA5092	OtA5093-OtA5097
Slc22a22	chr15:57243770-57477625	OtA5088-OtA5092	OtA5093-OtA5097
Slc22a26	chr19:7781980-7802667	OtA5088-OtA5092	OtA5093-OtA5097
Slc22a27	chr19:7864388-7966027	OtA5088-OtA5092	OtA5093-OtA5097
Slc22a28	chr19:8062208-8131982	OtA5088-OtA5092	OtA5093-OtA5097
Slc22a29	chr19:8160167-8218839	OtA5088-OtA5092	OtA5093-OtA5097
Slc22a30	chr19:8335622-8405105	OtA5088-OtA5092	OtA5093-OtA5097
Slc22a6	chr19:8617995-8628299	OtA5088-OtA5092	OtA5093-OtA5097
Slc22a7	chr17:46432184-46438477	OtA5088-OtA5092	OtA5093-OtA5097
Slc22a8	chr19:8591253-8611835	OtA5088-OtA5092	OtA5093-OtA5097
Slc23a1	chr18:35598666-35627227	OtA5088-OtA5092	OtA5093-OtA5097
Slc24a1	chr9:64922860-64951607	OtA5088-OtA5092	OtA5093-OtA5097
Slc24a2	chr4:86983125-87230540	OtA5088-OtA5092	OtA5093-OtA5097
Slc24a4	chr12:102129418-102266749	OtA5088-OtA5092	OtA5093-OtA5097
Slc24a5	chr2:125068126-125123660	OtA5088-OtA5092	OtA5093-OtA5097
Slc25a18	chr6:120773767-120793982	OtA5088-OtA5092	OtA5093-OtA5097
Slc25a21	chr12:56712633-57197472	OtA5088-OtA5092	OtA5093-OtA5097

Slc25a27	chr17:43641899-43667015	OtA5088-OtA5092	OtA5093-OtA5097
Slc25a31	chr3:40708870-40726094	OtA5088-OtA5092	OtA5093-OtA5097
Slc25a34	chr4:141618824-141623834	OtA5088-OtA5092	OtA5093-OtA5097
Slc25a41	chr17:57032771-57041654	OtA5088-OtA5092	OtA5093-OtA5097
Slc26a10	chr10:127172425-127180645	OtA5088-OtA5092	OtA5093-OtA5097
Slc26a3	chr12:31438300-31473719	OtA5088-OtA5092	OtA5093-OtA5097
Slc26a4	chr12:31519818-31559969	OtA5088-OtA5092	OtA5093-OtA5097
Slc26a5	chr5:21810654-21865604	OtA5088-OtA5092	OtA5093-OtA5097
Slc26a9	chr1:131744021-131770405	OtA5088-OtA5092	OtA5093-OtA5097
Slc27a5	chr7:12988345-12998192	OtA5088-OtA5092	OtA5093-OtA5097
Slc28a1	chr7:81114798-81170416	OtA5088-OtA5092	OtA5093-OtA5097
Slc2a4	chr11:69942285-69948190	OtA5088-OtA5092	OtA5093-OtA5097
Slc2a4rg-ps	chr2:181384249-181387596	OtA5088-OtA5092	OtA5093-OtA5097
Slc2a5	chr4:150119343-150144168	OtA5088-OtA5092	OtA5093-OtA5097
Slc2a6	chr2:27021364-27027998	OtA5088-OtA5092	OtA5093-OtA5097
Slc2a7	chr4:150148971-150168482	OtA5088-OtA5092	OtA5093-OtA5097
Slc2a9	chr5:38349272-38502152	OtA5088-OtA5092	OtA5093-OtA5097
Slc30a10	chr1:185447115-185468761	OtA5088-OtA5092	OtA5093-OtA5097
Slc30a8	chr15:52295552-52335733	OtA5088-OtA5092	OtA5093-OtA5097
Slc32a1	chr2:158610757-158615747	OtA5088-OtA5092	OtA5093-OtA5097
Slc34a1	chr13:55399647-55415232	OtA5088-OtA5092	OtA5093-OtA5097
Slc34a3	chr2:25228896-25234234	OtA5088-OtA5092	OtA5093-OtA5097
Slc35d3	chr10:19847916-19851459	OtA5088-OtA5092	OtA5093-OtA5097
Slc35f1	chr10:52690500-53111622	OtA5088-OtA5092	OtA5093-OtA5097
Slc35f3	chr8:126298578-126395977	OtA5088-OtA5092	OtA5093-OtA5097
Slc35f4	chr14:49298519-49525837	OtA5088-OtA5092	OtA5093-OtA5097
Slc36a2	chr11:55158467-55185077	OtA5088-OtA5092	OtA5093-OtA5097
Slc36a3	chr11:55124822-55151706	OtA5088-OtA5092	OtA5093-OtA5097
Slc37a2	chr9:37229148-37255738	OtA5088-OtA5092	OtA5093-OtA5097
Slc38a11	chr2:65316632-65364026	OtA5088-OtA5092	OtA5093-OtA5097
Slc38a5	chrX:8271391-8280176	OtA5088-OtA5092	OtA5093-OtA5097
Slc38a8	chr8:119479602-119501698	OtA5088-OtA5092	OtA5093-OtA5097
Slc39a12	chr2:14388397-14494977	OtA5088-OtA5092	OtA5093-OtA5097
Slc43a1	chr2:84839407-84863586	OtA5088-OtA5092	OtA5093-OtA5097
Slc44a5	chr3:153973435-154271720	OtA5088-OtA5092	OtA5093-OtA5097
Slc45a1	chr4:150629395-150652174	OtA5088-OtA5092	OtA5093-OtA5097
Slc45a2	chr15:11000720-11029233	OtA5088-OtA5092	OtA5093-OtA5097
Slc46a2	chr4:59905898-59915056	OtA5088-OtA5092	OtA5093-OtA5097
Slc47a1	chr11:61343399-61378075	OtA5088-OtA5092	OtA5093-OtA5097
Slc47a2	chr11:61301630-61342860	OtA5088-OtA5092	OtA5093-OtA5097
Slc4a10	chr2:62046514-62326743	OtA5088-OtA5092	OtA5093-OtA5097
Slc4a9	chr18:36528151-36544608	OtA5088-OtA5092	OtA5093-OtA5097
Slc5a10	chr11:61672781-61720799	OtA5088-OtA5092	OtA5093-OtA5097
Slc5a11	chr7:123214865-123273251	OtA5088-OtA5092	OtA5093-OtA5097
Slc5a12	chr2:110597298-110649345	OtA5088-OtA5092	OtA5093-OtA5097
Slc5a2	chr7:128265696-128298131	OtA5088-OtA5092	OtA5093-OtA5097
Slc5a4a	chr10:76147450-76189265	OtA5088-OtA5092	OtA5093-OtA5097

Slc5a4b	chr10:76058620-76111018	OtA5088-OtA5092	OtA5093-OtA5097
Slc5a7	chr17:54273589-54299034	OtA5088-OtA5092	OtA5093-OtA5097
Slc5a8	chr10:88885991-88929515	OtA5088-OtA5092	OtA5093-OtA5097
Slc5a9	chr4:111875376-111902796	OtA5088-OtA5092	OtA5093-OtA5097
Slc6a1	chr6:114282634-114317525	OtA5088-OtA5092	OtA5093-OtA5097
Slc6a11	chr6:114131240-114249886	OtA5088-OtA5092	OtA5093-OtA5097
Slc6a14	chrX:21714899-21742357	OtA5088-OtA5092	OtA5093-OtA5097
Slc6a15	chr10:103367807-103419379	OtA5088-OtA5092	OtA5093-OtA5097
Slc6a17	chr3:107467547-107518018	OtA5088-OtA5092	OtA5093-OtA5097
Slc6a18	chr13:73661749-73678023	OtA5088-OtA5092	OtA5093-OtA5097
Slc6a20a	chr9:123636906-123678832	OtA5088-OtA5092	OtA5093-OtA5097
Slc6a20b	chr9:123593819-123632565	OtA5088-OtA5092	OtA5093-OtA5097
Slc6a3	chr13:73536746-73578672	OtA5088-OtA5092	OtA5093-OtA5097
Slc6a5	chr7:49910298-49959493	OtA5088-OtA5092	OtA5093-OtA5097
Slc6a7	chr18:60995379-61014199	OtA5088-OtA5092	OtA5093-OtA5097
Slc6a9	chr4:117835257-117872470	OtA5088-OtA5092	OtA5093-OtA5097
Slc7a10	chr7:35186384-35215345	OtA5088-OtA5092	OtA5093-OtA5097
Slc7a11	chr3:50364935-50443613	OtA5088-OtA5092	OtA5093-OtA5097
Slc7a12	chr3:14480698-14505818	OtA5088-OtA5092	OtA5093-OtA5097
Slc7a13	chr4:19818726-19842213	OtA5088-OtA5092	OtA5093-OtA5097
Slc7a14	chr3:31202855-31310319	OtA5088-OtA5092	OtA5093-OtA5097
Slc7a15	chr12:8528482-8599066	OtA5088-OtA5092	OtA5093-OtA5097
Slc7a3	chrX:101079220-101085352	OtA5088-OtA5092	OtA5093-OtA5097
Slc7a4	chr16:17572017-17576671	OtA5088-OtA5092	OtA5093-OtA5097
Slc8a1	chr17:81373104-81738377	OtA5088-OtA5092	OtA5093-OtA5097
Slc8a2	chr7:16130299-16160511	OtA5088-OtA5092	OtA5093-OtA5097
Slc8a3	chr12:81197914-81333180	OtA5088-OtA5092	OtA5093-OtA5097
Slc9a10	chr16:45535265-45607001	OtA5088-OtA5092	OtA5093-OtA5097
Slc9a4	chr1:40580226-40630731	OtA5088-OtA5092	OtA5093-OtA5097
Slc9a5	chr8:105348257-105369881	OtA5088-OtA5092	OtA5093-OtA5097
Slc9a7	chrX:20105754-20291764	OtA5088-OtA5092	OtA5093-OtA5097
Slc9a9	chr9:94669891-95230452	OtA5088-OtA5092	OtA5093-OtA5097
Slco1a1	chr6:141907280-141946962	OtA5088-OtA5092	OtA5093-OtA5097
Slco1a4	chr6:141805439-141856171	OtA5088-OtA5092	OtA5093-OtA5097
Slco1a5	chr6:142234228-142322950	OtA5088-OtA5092	OtA5093-OtA5097
Slco1a6	chr6:142085767-142186149	OtA5088-OtA5092	OtA5093-OtA5097
Slco1b2	chr6:141629517-141686635	OtA5088-OtA5092	OtA5093-OtA5097
Slco1c1	chr6:141524385-141570177	OtA5088-OtA5092	OtA5093-OtA5097
Slco4c1	chr1:96818783-96872171	OtA5088-OtA5092	OtA5093-OtA5097
Slco6b1	chr1:96906175-96997560	OtA5088-OtA5092	OtA5093-OtA5097
Slco6c1	chr1:97059448-97128303	OtA5088-OtA5092	OtA5093-OtA5097
Slco6d1	chr1:98421123-98509380	OtA5088-OtA5092	OtA5093-OtA5097
Slfn1	chr11:83116844-83122659	OtA5088-OtA5092	OtA5093-OtA5097
Slfn10-ps	chr11:83028125-83040533	OtA5088-OtA5092	OtA5093-OtA5097
Slfn14-ps	chr11:83275111-83286726	OtA5088-OtA5092	OtA5093-OtA5097
Slfn3	chr11:83191329-83215154	OtA5088-OtA5092	OtA5093-OtA5097
Slfn4	chr11:83175185-83190216	OtA5088-OtA5092	OtA5093-OtA5097

Slfn8	chr11:83002157-83020810	OtA5088-OtA5092	OtA5093-OtA5097
Slfn9	chr11:82980302-82991830	OtA5088-OtA5092	OtA5093-OtA5097
Slfnl1	chr4:120532230-120536661	OtA5088-OtA5092	OtA5093-OtA5097
Slit2	chr5:47983154-48305778	OtA5088-OtA5092	OtA5093-OtA5097
Slitrk1	chr14:108909988-108914239	OtA5088-OtA5092	OtA5093-OtA5097
Slitrk2	chrX:66649317-66661402	OtA5088-OtA5092	OtA5093-OtA5097
Slitrk3	chr3:73048124-73056943	OtA5088-OtA5092	OtA5093-OtA5097
Slitrk4	chrX:64269443-64276996	OtA5088-OtA5092	OtA5093-OtA5097
Slitrk5	chr14:111675114-111683135	OtA5088-OtA5092	OtA5093-OtA5097
Slitrk6	chr14:110748577-110755149	OtA5088-OtA5092	OtA5093-OtA5097
Slmo1	chr18:67464848-67480581	OtA5088-OtA5092	OtA5093-OtA5097
Sln	chr9:53850250-53853849	OtA5088-OtA5092	OtA5093-OtA5097
Slurp1	chr15:74726643-74728026	OtA5088-OtA5092	OtA5093-OtA5097
Slx	chrX:26522656-26545565	OtA5088-OtA5092	OtA5093-OtA5097
Slxl1	chrX:55226875-55243736	OtA5088-OtA5092	OtA5093-OtA5097
Sly	chrY:75195855-75222053	OtA5088-OtA5092	OtA5093-OtA5097
Smad9	chr3:54755581-54801257	OtA5088-OtA5092	OtA5093-OtA5097
Smarca1	chrX:47809369-47892552	OtA5088-OtA5092	OtA5093-OtA5097
Smarca5-ps	chr4:145464209-145467961	OtA5088-OtA5092	OtA5093-OtA5097
Smc1b	chr15:85064688-85131957	OtA5088-OtA5092	OtA5093-OtA5097
Smc2	chr4:52439248-52488259	OtA5088-OtA5092	OtA5093-OtA5097
Smcp	chr3:92583865-92589024	OtA5088-OtA5092	OtA5093-OtA5097
Smgc	chr15:91838325-91934555	OtA5088-OtA5092	OtA5093-OtA5097
Smok2a	chr17:13221187-13227658	OtA5088-OtA5092	OtA5093-OtA5097
Smok2b	chr17:13234870-13236454	OtA5088-OtA5092	OtA5093-OtA5097
Smok3a	chr5:138021275-138034665	OtA5088-OtA5092	OtA5093-OtA5097
Smok3b	chr5:138037223-138050633	OtA5088-OtA5092	OtA5093-OtA5097
Smok4a	chr17:13487020-13554094	OtA5088-OtA5092	OtA5093-OtA5097
Smpx	chrX:157698972-157752591	OtA5088-OtA5092	OtA5093-OtA5097
Smr2	chr5:88086555-88109053	OtA5088-OtA5092	OtA5093-OtA5097
Smr3a	chr5:88002524-88008534	OtA5088-OtA5092	OtA5093-OtA5097
Smtnl1	chr2:84811175-84822652	OtA5088-OtA5092	OtA5093-OtA5097
Smyd1	chr6:71213939-71262281	OtA5088-OtA5092	OtA5093-OtA5097
Snai2	chr16:14705858-14709382	OtA5088-OtA5092	OtA5093-OtA5097
Snai3	chr8:122454205-122460692	OtA5088-OtA5092	OtA5093-OtA5097
Snap25	chr2:136713449-136782428	OtA5088-OtA5092	OtA5093-OtA5097
Sncaip	chr18:52767810-52915931	OtA5088-OtA5092	OtA5093-OtA5097
Sncb	chr13:54758859-54766440	OtA5088-OtA5092	OtA5093-OtA5097
Sncg	chr14:34370273-34374669	OtA5088-OtA5092	OtA5093-OtA5097
Snhg11	chr2:158375637-158386145	OtA5088-OtA5092	OtA5093-OtA5097
Snhg4	chr18:35553409-35558316	OtA5088-OtA5092	OtA5093-OtA5097
Snhg9	chr17:24719530-24719965	OtA5088-OtA5092	OtA5093-OtA5097
Snora15	chr5:129794560-129794676	OtA5088-OtA5092	OtA5093-OtA5097
Snora16a	chr4:132308677-132311024	OtA5088-OtA5092	OtA5093-OtA5097
Snora17	chr2:26637175-26640244	OtA5088-OtA5092	OtA5093-OtA5097
Snora19	chr19:60761115-60790693	OtA5088-OtA5092	OtA5093-OtA5097
Snora20	chr17:12916464-12940396	OtA5088-OtA5092	OtA5093-OtA5097

Snora21	chr11:97777525-97782439	OtA5088-OtA5092	OtA5093-OtA5097
Snora23	chr7:110018424-110055114	OtA5088-OtA5092	OtA5093-OtA5097
Snora24	chr3:123507551-123508336	OtA5088-OtA5092	OtA5093-OtA5097
Snora28	chr12:111538100-111546753	OtA5088-OtA5092	OtA5093-OtA5097
Snora2b	chr15:98519100-98534221	OtA5088-OtA5092	OtA5093-OtA5097
Snora3	chr7:109519194-109522369	OtA5088-OtA5092	OtA5093-OtA5097
Snora30	chr7:127527896-127528003	OtA5088-OtA5092	OtA5093-OtA5097
Snora31	chr14:75845255-75848303	OtA5088-OtA5092	OtA5093-OtA5097
Snora33	chr10:23785182-23787209	OtA5088-OtA5092	OtA5093-OtA5097
Snora34	chr15:98519100-98534221	OtA5088-OtA5092	OtA5093-OtA5097
Snora35	chrX:146962512-147197277	OtA5088-OtA5092	OtA5093-OtA5097
Snora36b	chr1:185204167-185329401	OtA5088-OtA5092	OtA5093-OtA5097
Snora41	chr1:63176830-63180486	OtA5088-OtA5092	OtA5093-OtA5097
Snora43	chr2:26637175-26640244	OtA5088-OtA5092	OtA5093-OtA5097
Snora47	chr13:95325236-95337841	OtA5088-OtA5092	OtA5093-OtA5097
Snora5c	chr11:6615597-6626067	OtA5088-OtA5092	OtA5093-OtA5097
Snora61	chr4:132308677-132311024	OtA5088-OtA5092	OtA5093-OtA5097
Snora62	chr9:120127765-120132369	OtA5088-OtA5092	OtA5093-OtA5097
Snora64	chr17:24720062-24721927	OtA5088-OtA5092	OtA5093-OtA5097
Snora65	chr2:32961711-32964045	OtA5088-OtA5092	OtA5093-OtA5097
Snora68	chr8:70894721-70897443	OtA5088-OtA5092	OtA5093-OtA5097
Snora69	chrX:37082519-37085184	OtA5088-OtA5092	OtA5093-OtA5097
Snora75	chr1:86344718-86359455	OtA5088-OtA5092	OtA5093-OtA5097
Snora81	chr16:23107478-23127730	OtA5088-OtA5092	OtA5093-OtA5097
Snord100	chr10:23785182-23787209	OtA5088-OtA5092	OtA5093-OtA5097
Snord104	chr11:106500990-106501063	OtA5088-OtA5092	OtA5093-OtA5097
Snord11	chr1:59685005-59711510	OtA5088-OtA5092	OtA5093-OtA5097
Snord110	chr2:130274411-130284451	OtA5088-OtA5092	OtA5093-OtA5097
Snord111	chr8:110810491-110846803	OtA5088-OtA5092	OtA5093-OtA5097
Snord116	chr7:59751490-59751580	OtA5088-OtA5092	OtA5093-OtA5097
Snord116l1	chr7:59700879-59700969	OtA5088-OtA5092	OtA5093-OtA5097
Snord118	chr19:7417624-7483289	OtA5088-OtA5092	OtA5093-OtA5097
Snord12	chr2:167035796-167065862	OtA5088-OtA5092	OtA5093-OtA5097
Snord123	chr15:32240567-32244662	OtA5088-OtA5092	OtA5093-OtA5097
Snord14a	chr2:32775820-32784405	OtA5088-OtA5092	OtA5093-OtA5097
Snord14c	chr10:46244747-46244770	OtA5088-OtA5092	OtA5093-OtA5097
Snord14d	chr10:46244747-46244770	OtA5088-OtA5092	OtA5093-OtA5097
Snord16a	chr9:64173386-64178562	OtA5088-OtA5092	OtA5093-OtA5097
Snord19	chr14:30999825-31019089	OtA5088-OtA5092	OtA5093-OtA5097
Snord1a	chr11:116671659-116675798	OtA5088-OtA5092	OtA5093-OtA5097
Snord1b	chr11:116671659-116675798	OtA5088-OtA5092	OtA5093-OtA5097
Snord1c	chr11:116671659-116675798	OtA5088-OtA5092	OtA5093-OtA5097
Snord2	chr16:23107478-23127730	OtA5088-OtA5092	OtA5093-OtA5097
Snord23	chr7:15937835-15946108	OtA5088-OtA5092	OtA5093-OtA5097
Snord32a	chr7:45125562-45128745	OtA5088-OtA5092	OtA5093-OtA5097
Snord33	chr7:45125562-45128745	OtA5088-OtA5092	OtA5093-OtA5097
Snord34	chr7:45125562-45128745	OtA5088-OtA5092	OtA5093-OtA5097

Snord35a	chr7:45125562-45128745	OtA5088-OtA5092	OtA5093-OtA5097
Snord35b	chr7:45122387-45124389	OtA5088-OtA5092	OtA5093-OtA5097
Snord37	chr10:81176630-81182509	OtA5088-OtA5092	OtA5093-OtA5097
Snord38a	chr4:117153835-117156132	OtA5088-OtA5092	OtA5093-OtA5097
Snord42a	chr11:78180935-78183584	OtA5088-OtA5092	OtA5093-OtA5097
Snord42b	chr11:78180935-78183584	OtA5088-OtA5092	OtA5093-OtA5097
Snord43	chr15:80077780-80083406	OtA5088-OtA5092	OtA5093-OtA5097
Snord45b	chr3:153907288-153912966	OtA5088-OtA5092	OtA5093-OtA5097
Snord45c	chr3:153907288-153912966	OtA5088-OtA5092	OtA5093-OtA5097
Snord47	chr1:161035165-161038537	OtA5088-OtA5092	OtA5093-OtA5097
Snord49a	chr11:62602876-62604806	OtA5088-OtA5092	OtA5093-OtA5097
Snord49b	chr11:62602876-62604806	OtA5088-OtA5092	OtA5093-OtA5097
Snord4a	chr11:78180935-78183584	OtA5088-OtA5092	OtA5093-OtA5097
Snord52	chr17:34950235-34952471	OtA5088-OtA5092	OtA5093-OtA5097
Snord53	chr7:110848295-110848315	OtA5088-OtA5092	OtA5093-OtA5097
Snord55	chr4:117153835-117156132	OtA5088-OtA5092	OtA5093-OtA5097
Snord57	chr2:130274411-130284451	OtA5088-OtA5092	OtA5093-OtA5097
Snord58b	chr18:75000476-75003381	OtA5088-OtA5092	OtA5093-OtA5097
Snord61	chrX:57383347-57393036	OtA5088-OtA5092	OtA5093-OtA5097
Snord64	chr7:59978810-59978856	OtA5088-OtA5092	OtA5093-OtA5097
Snord65	chr11:62602876-62604806	OtA5088-OtA5092	OtA5093-OtA5097
Snord66	chr16:20672748-20692883	OtA5088-OtA5092	OtA5093-OtA5097
Snord67	chr2:91546321-91620665	OtA5088-OtA5092	OtA5093-OtA5097
Snord68	chr8:123102349-123105242	OtA5088-OtA5092	OtA5093-OtA5097
Snord69	chr14:30999825-31019089	OtA5088-OtA5092	OtA5093-OtA5097
Snord7	chr11:83291698-83298977	OtA5088-OtA5092	OtA5093-OtA5097
Snord70	chr1:59685005-59711510	OtA5088-OtA5092	OtA5093-OtA5097
Snord71	chr8:109778639-109864208	OtA5088-OtA5092	OtA5093-OtA5097
Snord72	chr15:5116612-5119140	OtA5088-OtA5092	OtA5093-OtA5097
Snord73a	chr3:86137939-86142668	OtA5088-OtA5092	OtA5093-OtA5097
Snord8	chr14:52198150-52237572	OtA5088-OtA5092	OtA5093-OtA5097
Snord82	chr1:86344718-86359455	OtA5088-OtA5092	OtA5093-OtA5097
Snord83b	chr15:80077780-80083406	OtA5088-OtA5092	OtA5093-OtA5097
Snord85	chr4:130663358-130781565	OtA5088-OtA5092	OtA5093-OtA5097
Snord87	chr1:9942024-9944118	OtA5088-OtA5092	OtA5093-OtA5097
Snord88a	chr7:44246721-44252319	OtA5088-OtA5092	OtA5093-OtA5097
Snord88c	chr7:44246721-44252319	OtA5088-OtA5092	OtA5093-OtA5097
Snord89	chr1:39548746-39548840	OtA5088-OtA5092	OtA5093-OtA5097
Snord90	chr2:37370070-37422903	OtA5088-OtA5092	OtA5093-OtA5097
Snord91a	chr11:74898079-74925798	OtA5088-OtA5092	OtA5093-OtA5097
Snord92	chr17:71616214-71659031	OtA5088-OtA5092	OtA5093-OtA5097
Snord93	chr5:23787743-24161231	OtA5088-OtA5092	OtA5093-OtA5097
Snord95	chr11:48800359-48806241	OtA5088-OtA5092	OtA5093-OtA5097
Snord96a	chr11:48800359-48806241	OtA5088-OtA5092	OtA5093-OtA5097
Snord98	chr10:62743927-62792368	OtA5088-OtA5092	OtA5093-OtA5097
Snord99	chr4:132308677-132311024	OtA5088-OtA5092	OtA5093-OtA5097
Snph	chr2:151590549-151632533	OtA5088-OtA5092	OtA5093-OtA5097

Sntg1	chr1:8362660-9299730	OtA5088-OtA5092	OtA5093-OtA5097
Sntg2	chr12:30174556-30373375	OtA5088-OtA5092	OtA5093-OtA5097
Sntn	chr14:13670875-13683148	OtA5088-OtA5092	OtA5093-OtA5097
Snx22	chr9:66060168-66069731	OtA5088-OtA5092	OtA5093-OtA5097
Snx31	chr15:36504061-36555572	OtA5088-OtA5092	OtA5093-OtA5097
Snx32	chr19:5495277-5510489	OtA5088-OtA5092	OtA5093-OtA5097
Sobp	chr10:43002499-43174530	OtA5088-OtA5092	OtA5093-OtA5097
Sohlh1	chr2:25842996-25847248	OtA5088-OtA5092	OtA5093-OtA5097
Sohlh2	chr3:55182043-55209957	OtA5088-OtA5092	OtA5093-OtA5097
Sorcs1	chr19:50143300-50678646	OtA5088-OtA5092	OtA5093-OtA5097
Sorcs3	chr19:48206024-48805505	OtA5088-OtA5092	OtA5093-OtA5097
Sost	chr11:101962457-101967015	OtA5088-OtA5092	OtA5093-OtA5097
Sostdc1	chr12:36314168-36318452	OtA5088-OtA5092	OtA5093-OtA5097
Sowaha	chr11:53476577-53480195	OtA5088-OtA5092	OtA5093-OtA5097
Sox1	chr8:12385770-12436732	OtA5088-OtA5092	OtA5093-OtA5097
Sox10	chr15:79154912-79164490	OtA5088-OtA5092	OtA5093-OtA5097
Sox12	chr2:152393611-152398046	OtA5088-OtA5092	OtA5093-OtA5097
Sox14	chr9:99874105-99876170	OtA5088-OtA5092	OtA5093-OtA5097
Sox2	chr3:34560380-34677993	OtA5088-OtA5092	OtA5093-OtA5097
Sox2ot	chr3:34560380-34677993	OtA5088-OtA5092	OtA5093-OtA5097
Sox3	chrX:60891365-60893430	OtA5088-OtA5092	OtA5093-OtA5097
Sox30	chr11:45980309-46017992	OtA5088-OtA5092	OtA5093-OtA5097
Sox6	chr7:115471343-116038744	OtA5088-OtA5092	OtA5093-OtA5097
Sox8	chr17:25565892-25570686	OtA5088-OtA5092	OtA5093-OtA5097
Sp110	chr1:85576898-85645036	OtA5088-OtA5092	OtA5093-OtA5097
Sp140	chr1:85576898-85645036	OtA5088-OtA5092	OtA5093-OtA5097
Sp4	chr12:118234932-118301440	OtA5088-OtA5092	OtA5093-OtA5097
Sp5	chr2:70474922-70477726	OtA5088-OtA5092	OtA5093-OtA5097
Sp7	chr15:102357176-102366271	OtA5088-OtA5092	OtA5093-OtA5097
Sp8	chr12:118846328-118852578	OtA5088-OtA5092	OtA5093-OtA5097
Sp9	chr2:73271925-73275771	OtA5088-OtA5092	OtA5093-OtA5097
Spaca1	chr4:34028313-34050067	OtA5088-OtA5092	OtA5093-OtA5097
Spaca3	chr11:80858388-80867814	OtA5088-OtA5092	OtA5093-OtA5097
Spaca4	chr7:45721219-45729492	OtA5088-OtA5092	OtA5093-OtA5097
Spaca5	chrX:21068405-21077963	OtA5088-OtA5092	OtA5093-OtA5097
Spaca7	chr8:12573048-12600738	OtA5088-OtA5092	OtA5093-OtA5097
Spag11a	chr8:19157886-19159578	OtA5088-OtA5092	OtA5093-OtA5097
Spag11b	chr8:19140758-19143010	OtA5088-OtA5092	OtA5093-OtA5097
Spag16	chr1:69826987-70725132	OtA5088-OtA5092	OtA5093-OtA5097
Spag17	chr3:99885416-100162403	OtA5088-OtA5092	OtA5093-OtA5097
Spag4	chr2:156065212-156069499	OtA5088-OtA5092	OtA5093-OtA5097
Spag5	chr11:78301590-78322454	OtA5088-OtA5092	OtA5093-OtA5097
Spag6	chr16:16753015-16829363	OtA5088-OtA5092	OtA5093-OtA5097
Spag8	chr4:43651728-43653552	OtA5088-OtA5092	OtA5093-OtA5097
Spam1	chr6:24791198-24801048	OtA5088-OtA5092	OtA5093-OtA5097
Spata1	chr3:146450492-146499753	OtA5088-OtA5092	OtA5093-OtA5097
Spata16	chr3:26637630-26983212	OtA5088-OtA5092	OtA5093-OtA5097

Spata17	chr1:187048406-187215446	OtA5088-OtA5092	OtA5093-OtA5097
Spata18	chr5:73651379-73679484	OtA5088-OtA5092	OtA5093-OtA5097
Spata19	chr9:27396820-27401710	OtA5088-OtA5092	OtA5093-OtA5097
Spata22	chr11:73329740-73346044	OtA5088-OtA5092	OtA5093-OtA5097
Spata24	chr18:35656688-35662186	OtA5088-OtA5092	OtA5093-OtA5097
Spata25	chr2:164827381-164828534	OtA5088-OtA5092	OtA5093-OtA5097
Spata3	chr1:86021941-86029958	OtA5088-OtA5092	OtA5093-OtA5097
Spata4	chr8:54600780-54610098	OtA5088-OtA5092	OtA5093-OtA5097
Spata7	chr12:98628183-98669814	OtA5088-OtA5092	OtA5093-OtA5097
Spata9	chr13:75967738-75998968	OtA5088-OtA5092	OtA5093-OtA5097
Spatc1	chr15:76266786-76292572	OtA5088-OtA5092	OtA5093-OtA5097
Spats1	chr17:45448936-45474938	OtA5088-OtA5092	OtA5093-OtA5097
Spc24	chr9:21755441-21760286	OtA5088-OtA5092	OtA5093-OtA5097
Spc25	chr2:69193894-69206190	OtA5088-OtA5092	OtA5093-OtA5097
Spdef	chr17:27714446-27728951	OtA5088-OtA5092	OtA5093-OtA5097
Spdya	chr17:71552060-71598761	OtA5088-OtA5092	OtA5093-OtA5097
Spdyb	chr5:143216315-143225882	OtA5088-OtA5092	OtA5093-OtA5097
Speer1-ps1	chr5:11340407-11346273	OtA5088-OtA5092	OtA5093-OtA5097
Speer2	chr16:69856873-69863744	OtA5088-OtA5092	OtA5093-OtA5097
Speer3	chr5:13791618-13796819	OtA5088-OtA5092	OtA5093-OtA5097
Speer4a	chr5:26034269-26039505	OtA5088-OtA5092	OtA5093-OtA5097
Speer4b	chr5:26817356-27727500	OtA5088-OtA5092	OtA5093-OtA5097
Speer4d	chr5:15619098-15623864	OtA5088-OtA5092	OtA5093-OtA5097
Speer4e	chr5:14933630-14938475	OtA5088-OtA5092	OtA5093-OtA5097
Speer4f	chr5:17476121-17480936	OtA5088-OtA5092	OtA5093-OtA5097
Speer5-ps1	chr10:44170446-44219585	OtA5088-OtA5092	OtA5093-OtA5097
Speer6-ps1	chr13:3149474-3189678	OtA5088-OtA5092	OtA5093-OtA5097
Speer7-ps1	chr5:15680709-15714596	OtA5088-OtA5092	OtA5093-OtA5097
Speer8-ps1	chr5:14945293-14978541	OtA5088-OtA5092	OtA5093-OtA5097
Speer9-ps1	chr7:3128145-3144992	OtA5088-OtA5092	OtA5093-OtA5097
Spef1	chr2:131127411-131604824	OtA5088-OtA5092	OtA5093-OtA5097
Spef2	chr15:9662735-9748806	OtA5088-OtA5092	OtA5093-OtA5097
Spem1	chr11:69820870-69822165	OtA5088-OtA5092	OtA5093-OtA5097
Spert	chr14:75582833-75593116	OtA5088-OtA5092	OtA5093-OtA5097
Spesp1	chr9:62270728-62282179	OtA5088-OtA5092	OtA5093-OtA5097
Sphkap	chr1:83255780-83408200	OtA5088-OtA5092	OtA5093-OtA5097
Spib	chr7:44525994-44532071	OtA5088-OtA5092	OtA5093-OtA5097
Spic	chr10:88675269-88683023	OtA5088-OtA5092	OtA5093-OtA5097
Spink10	chr18:62548910-62741387	OtA5088-OtA5092	OtA5093-OtA5097
Spink11	chr18:44190044-44196177	OtA5088-OtA5092	OtA5093-OtA5097
Spink12	chr18:44104522-44108543	OtA5088-OtA5092	OtA5093-OtA5097
Spink13	chr18:62548910-62741387	OtA5088-OtA5092	OtA5093-OtA5097
Spink14	chr18:44027868-44032208	OtA5088-OtA5092	OtA5093-OtA5097
Spink2	chr5:77205107-77211453	OtA5088-OtA5092	OtA5093-OtA5097
Spink4	chr4:40920055-40931395	OtA5088-OtA5092	OtA5093-OtA5097
Spink5	chr18:43963240-44022487	OtA5088-OtA5092	OtA5093-OtA5097
Spink6	chr18:44071392-44083610	OtA5088-OtA5092	OtA5093-OtA5097

Spink7	chr18:62548910-62741387	OtA5088-OtA5092	OtA5093-OtA5097
Spink8	chr9:109816626-109826627	OtA5088-OtA5092	OtA5093-OtA5097
Spinkl	chr18:44166357-44175073	OtA5088-OtA5092	OtA5093-OtA5097
Spinlw1	chr2:164588342-164593571	OtA5088-OtA5092	OtA5093-OtA5097
Spint3	chr2:164569694-164573456	OtA5088-OtA5092	OtA5093-OtA5097
Spint4	chr2:164698500-164702448	OtA5088-OtA5092	OtA5093-OtA5097
Spn-ps	chr15:90891395-90898476	OtA5088-OtA5092	OtA5093-OtA5097
Spna1	chr1:174172775-174248449	OtA5088-OtA5092	OtA5093-OtA5097
Spnb1	chr12:76580487-76710547	OtA5088-OtA5092	OtA5093-OtA5097
Spnb3	chr19:4711222-4752352	OtA5088-OtA5092	OtA5093-OtA5097
Spnb4	chr7:27356382-27446589	OtA5088-OtA5092	OtA5093-OtA5097
Spns3	chr11:72498155-72550246	OtA5088-OtA5092	OtA5093-OtA5097
Spo11	chr2:172979841-172993576	OtA5088-OtA5092	OtA5093-OtA5097
Spock2	chr10:60106256-60133913	OtA5088-OtA5092	OtA5093-OtA5097
Spock3	chr8:62951231-63357096	OtA5088-OtA5092	OtA5093-OtA5097
Spred3	chr7:29158828-29168647	OtA5088-OtA5092	OtA5093-OtA5097
Sprn	chr7:140137563-140154659	OtA5088-OtA5092	OtA5093-OtA5097
Sprr1a	chr3:92483953-92485881	OtA5088-OtA5092	OtA5093-OtA5097
Sprr1b	chr3:92436808-92438779	OtA5088-OtA5092	OtA5093-OtA5097
Sprr2a2	chr3:92250646-92257304	OtA5088-OtA5092	OtA5093-OtA5097
Sprr2b	chr3:92316704-92318085	OtA5088-OtA5092	OtA5093-OtA5097
Sprr2d	chr3:92339139-92340873	OtA5088-OtA5092	OtA5093-OtA5097
Sprr2e	chr3:92352142-92353449	OtA5088-OtA5092	OtA5093-OtA5097
Sprr2f	chr3:92365186-92366442	OtA5088-OtA5092	OtA5093-OtA5097
Sprr2g	chr3:92373914-92375229	OtA5088-OtA5092	OtA5093-OtA5097
Sprr2h	chr3:92385684-92387319	OtA5088-OtA5092	OtA5093-OtA5097
Sprr2i	chr3:92407990-92409271	OtA5088-OtA5092	OtA5093-OtA5097
Sprr2j-ps	chr3:92418086-92419396	OtA5088-OtA5092	OtA5093-OtA5097
Sprr2k	chr3:92432581-92433927	OtA5088-OtA5092	OtA5093-OtA5097
Sprr3	chr3:92456501-92458720	OtA5088-OtA5092	OtA5093-OtA5097
Sprr4	chr3:92500262-92500493	OtA5088-OtA5092	OtA5093-OtA5097
Spry3	chrX_GL456233_random:101351-10639	OtA5088-OtA5092	OtA5093-OtA5097
Spt1	chr15:103751922-103899312	OtA5088-OtA5092	OtA5093-OtA5097
Sptlc3	chr2:139493919-139637674	OtA5088-OtA5092	OtA5093-OtA5097
Spz1	chr13:92574631-92576232	OtA5088-OtA5092	OtA5093-OtA5097
Srcin1	chr11:97509339-97575126	OtA5088-OtA5092	OtA5093-OtA5097
Srcrb4d	chr5:135960222-135974476	OtA5088-OtA5092	OtA5093-OtA5097
Srd5a2	chr17:74017705-74047916	OtA5088-OtA5092	OtA5093-OtA5097
Srl	chr16:4480227-4523053	OtA5088-OtA5092	OtA5093-OtA5097
Srms	chr2:181205562-181213171	OtA5088-OtA5092	OtA5093-OtA5097
Srp54b,Srp54c	chr12:55230167-55263480	OtA5088-OtA5092	OtA5093-OtA5097
Srp54c	chr12:55230167-55263480	OtA5088-OtA5092	OtA5093-OtA5097
Srpk3	chrX:73774421-73778924	OtA5088-OtA5092	OtA5093-OtA5097
Srpx2	chrX:133908425-133932450	OtA5088-OtA5092	OtA5093-OtA5097
Srrm3	chr5:135818105-135874772	OtA5088-OtA5092	OtA5093-OtA5097
Srrm4	chr5:116438721-116591817	OtA5088-OtA5092	OtA5093-OtA5097
Srsf12	chr4:33208990-33233340	OtA5088-OtA5092	OtA5093-OtA5097

Sry	chrY:2662470-2663658	OtA5088-OtA5092	OtA5093-OtA5097
Ssbp2	chr13:91461096-91786148	OtA5088-OtA5092	OtA5093-OtA5097
Sspn	chr6:145934146-145965225	OtA5088-OtA5092	OtA5093-OtA5097
Sspo	chr6:48448228-48501250	OtA5088-OtA5092	OtA5093-OtA5097
Sst	chr16:23889580-23890844	OtA5088-OtA5092	OtA5093-OtA5097
Sstr1	chr12:58211803-58216036	OtA5088-OtA5092	OtA5093-OtA5097
Sstr2	chr11:113624256-113625636	OtA5088-OtA5092	OtA5093-OtA5097
Sstr3	chr15:78537014-78544345	OtA5088-OtA5092	OtA5093-OtA5097
Sstr4	chr2:148395376-148396764	OtA5088-OtA5092	OtA5093-OtA5097
Sstr5	chr17:25489874-25497288	OtA5088-OtA5092	OtA5093-OtA5097
Ssty1	chrY:21095330-21097715	OtA5088-OtA5092	OtA5093-OtA5097
Ssty2	chrY:80463105-80465434	OtA5088-OtA5092	OtA5093-OtA5097
Ssx9	chrX:8748429-8754587	OtA5088-OtA5092	OtA5093-OtA5097
Ssxb1	chrX:8413304-8422157	OtA5088-OtA5092	OtA5093-OtA5097
Ssxb10	chrX:8327423-8336235	OtA5088-OtA5092	OtA5093-OtA5097
Ssxb2	chrX:8454342-8461726	OtA5088-OtA5092	OtA5093-OtA5097
Ssxb3	chrX:8583543-8589246	OtA5088-OtA5092	OtA5093-OtA5097
Ssxb5	chrX:8803690-8809386	OtA5088-OtA5092	OtA5093-OtA5097
Ssxb6	chrX:8542603-8548255	OtA5088-OtA5092	OtA5093-OtA5097
Ssxb8	chrX:8685330-8690844	OtA5088-OtA5092	OtA5093-OtA5097
Ssxb9	chrX:8366977-8375388	OtA5088-OtA5092	OtA5093-OtA5097
St18	chr1:6487230-6860940	OtA5088-OtA5092	OtA5093-OtA5097
St6gal2	chr17:55445716-55499226	OtA5088-OtA5092	OtA5093-OtA5097
St6galnac1	chr11:116765024-116775507	OtA5088-OtA5092	OtA5093-OtA5097
St6galnac5	chr3:152820709-152982207	OtA5088-OtA5092	OtA5093-OtA5097
St8sia1	chr6:142814230-142964452	OtA5088-OtA5092	OtA5093-OtA5097
St8sia3	chr18:64157723-64277180	OtA5088-OtA5092	OtA5093-OtA5097
St8sia4	chr1:95587681-95667594	OtA5088-OtA5092	OtA5093-OtA5097
St8sia6	chr2:13654935-13793520	OtA5088-OtA5092	OtA5093-OtA5097
Stac	chr9:111561433-111690216	OtA5088-OtA5092	OtA5093-OtA5097
Stac3	chr10:127501716-127508815	OtA5088-OtA5092	OtA5093-OtA5097
Stap1	chr5:86071827-86103993	OtA5088-OtA5092	OtA5093-OtA5097
Stard6	chr18:70472544-70501063	OtA5088-OtA5092	OtA5093-OtA5097
Stat4	chr1:52008243-52107184	OtA5088-OtA5092	OtA5093-OtA5097
Stc1	chr14:69029288-69041401	OtA5088-OtA5092	OtA5093-OtA5097
Stc2	chr11:31359440-31370061	OtA5088-OtA5092	OtA5093-OtA5097
Steap1	chr5:5736321-5749317	OtA5088-OtA5092	OtA5093-OtA5097
Steap2	chr5:5664830-5694568	OtA5088-OtA5092	OtA5093-OtA5097
Stfa1	chr16:36277147-36285371	OtA5088-OtA5092	OtA5093-OtA5097
Stfa2	chr16:36403945-36408363	OtA5088-OtA5092	OtA5093-OtA5097
Stfa2l1	chr16:36156810-36161948	OtA5088-OtA5092	OtA5093-OtA5097
Stfa3	chr16:36450536-36455392	OtA5088-OtA5092	OtA5093-OtA5097
Stil	chr4:115000117-115043198	OtA5088-OtA5092	OtA5093-OtA5097
Stk30	chr12:110807797-110840939	OtA5088-OtA5092	OtA5093-OtA5097
Stk32a	chr18:43207696-43317481	OtA5088-OtA5092	OtA5093-OtA5097
Stk32b	chr5:37446824-37717153	OtA5088-OtA5092	OtA5093-OtA5097
Stk32c	chr7:139103637-139242973	OtA5088-OtA5092	OtA5093-OtA5097

Stk33	chr7:109279215-109439053	OtA5088-OtA5092	OtA5093-OtA5097
Stk36	chr1:74601454-74636893	OtA5088-OtA5092	OtA5093-OtA5097
Stmn1-rs1	chr9:115242581-115310421	OtA5088-OtA5092	OtA5093-OtA5097
Stmn3	chr2:181306458-181314500	OtA5088-OtA5092	OtA5093-OtA5097
Stmn4	chr14:66344374-66361680	OtA5088-OtA5092	OtA5093-OtA5097
Stoml3	chr3:53488792-53507652	OtA5088-OtA5092	OtA5093-OtA5097
Stra8	chr6:34920959-34939342	OtA5088-OtA5092	OtA5093-OtA5097
Strbp	chr2:37569867-37647285	OtA5088-OtA5092	OtA5093-OtA5097
Strc	chr2:121358640-121380940	OtA5088-OtA5092	OtA5093-OtA5097
Stx19	chr16:62793688-62847040	OtA5088-OtA5092	OtA5093-OtA5097
Stx1a	chr5:135023571-135051099	OtA5088-OtA5092	OtA5093-OtA5097
Stxbp4	chr11:90476492-90638108	OtA5088-OtA5092	OtA5093-OtA5097
Stxbp5l	chr16:37107309-37384958	OtA5088-OtA5092	OtA5093-OtA5097
Styk1	chr6:131299143-131313827	OtA5088-OtA5092	OtA5093-OtA5097
Styx	chr14:45351185-45388796	OtA5088-OtA5092	OtA5093-OtA5097
Styxl1	chr5:135747219-135778385	OtA5088-OtA5092	OtA5093-OtA5097
Sucnr1	chr3:60081868-60087566	OtA5088-OtA5092	OtA5093-OtA5097
Sult1b1	chr5:87513338-87538195	OtA5088-OtA5092	OtA5093-OtA5097
Sult1c1	chr17:53961614-53990631	OtA5088-OtA5092	OtA5093-OtA5097
Sult1c2	chr17:53829636-53845958	OtA5088-OtA5092	OtA5093-OtA5097
Sult1d1	chr5:87554649-87569006	OtA5088-OtA5092	OtA5093-OtA5097
Sult2a1	chr7:13796245-13837410	OtA5088-OtA5092	OtA5093-OtA5097
Sult2a2	chr7:13733505-13779637	OtA5088-OtA5092	OtA5093-OtA5097
Sult2a3	chr7:14067554-14122993	OtA5088-OtA5092	OtA5093-OtA5097
Sult2a4	chr7:13909676-13989588	OtA5088-OtA5092	OtA5093-OtA5097
Sult2a5	chr7:13623966-13670807	OtA5088-OtA5092	OtA5093-OtA5097
Sult2a6	chr7:14222402-14254870	OtA5088-OtA5092	OtA5093-OtA5097
Sult2a7	chr7:14465158-14492926	OtA5088-OtA5092	OtA5093-OtA5097
Sult3a1	chr10:33857721-33879475	OtA5088-OtA5092	OtA5093-OtA5097
Sult4a1	chr15:84076096-84105754	OtA5088-OtA5092	OtA5093-OtA5097
Sult5a1	chr8:123142846-123158280	OtA5088-OtA5092	OtA5093-OtA5097
Sult6b1	chr17:78883937-78906992	OtA5088-OtA5092	OtA5093-OtA5097
Sun3	chr11:9016053-9069354	OtA5088-OtA5092	OtA5093-OtA5097
Sun5	chr2:153856189-153871084	OtA5088-OtA5092	OtA5093-OtA5097
Susd5	chr9:114057353-114098733	OtA5088-OtA5092	OtA5093-OtA5097
Sv2a	chr3:96181226-96195180	OtA5088-OtA5092	OtA5093-OtA5097
Sv2b	chr7:75114894-75331419	OtA5088-OtA5092	OtA5093-OtA5097
Sv2c	chr13:95959442-96132577	OtA5088-OtA5092	OtA5093-OtA5097
Sva	chr6:42038393-42042851	OtA5088-OtA5092	OtA5093-OtA5097
Sval1	chr6:41951627-41956098	OtA5088-OtA5092	OtA5093-OtA5097
Sval2	chr6:41860338-41864322	OtA5088-OtA5092	OtA5093-OtA5097
Sval3	chr6:41968139-41973090	OtA5088-OtA5092	OtA5093-OtA5097
Svip	chr7:51997160-52006018	OtA5088-OtA5092	OtA5093-OtA5097
Svop	chr5:114026912-114091380	OtA5088-OtA5092	OtA5093-OtA5097
Svopl	chr6:37983738-38046996	OtA5088-OtA5092	OtA5093-OtA5097
Svs1	chr6:48986860-48991724	OtA5088-OtA5092	OtA5093-OtA5097
Svs2	chr2:164235928-164238341	OtA5088-OtA5092	OtA5093-OtA5097

Svs3a	chr2:164289267-164291500	OtA5088-OtA5092	OtA5093-OtA5097
Svs3b	chr2:164254362-164256643	OtA5088-OtA5092	OtA5093-OtA5097
Svs4	chr2:164275955-164278307	OtA5088-OtA5092	OtA5093-OtA5097
Svs5	chr2:164332764-164334394	OtA5088-OtA5092	OtA5093-OtA5097
Svs6	chr2:164316750-164318450	OtA5088-OtA5092	OtA5093-OtA5097
Sybu	chr15:44671855-44805829	OtA5088-OtA5092	OtA5093-OtA5097
Syce1	chr7:140777228-140787854	OtA5088-OtA5092	OtA5093-OtA5097
Syce1l	chr8:113635585-113655533	OtA5088-OtA5092	OtA5093-OtA5097
Syce3	chr15:89390173-89410503	OtA5088-OtA5092	OtA5093-OtA5097
Sycp1	chr3:102818498-102936100	OtA5088-OtA5092	OtA5093-OtA5097
Sycp1-ps1	chr7:18786301-18798510	OtA5088-OtA5092	OtA5093-OtA5097
Sycp2	chr2:178345295-178407658	OtA5088-OtA5092	OtA5093-OtA5097
Sycp3	chr10:88459586-88503970	OtA5088-OtA5092	OtA5093-OtA5097
Syn1	chrX:20848542-20920918	OtA5088-OtA5092	OtA5093-OtA5097
Syn2	chr6:115134901-115282626	OtA5088-OtA5092	OtA5093-OtA5097
Syn3	chr10:86048745-86498896	OtA5088-OtA5092	OtA5093-OtA5097
Syne1	chr10:5020195-5194707	OtA5088-OtA5092	OtA5093-OtA5097
Syngr3	chr17:24685091-24689949	OtA5088-OtA5092	OtA5093-OtA5097
Syngr4	chr7:45886844-45896711	OtA5088-OtA5092	OtA5093-OtA5097
Synm	chr7:67730160-67759742	OtA5088-OtA5092	OtA5093-OtA5097
Synpo2l	chr14:20659417-20668306	OtA5088-OtA5092	OtA5093-OtA5097
Synpr	chr14:13284779-13615469	OtA5088-OtA5092	OtA5093-OtA5097
Sypl2	chr3:108212265-108226599	OtA5088-OtA5092	OtA5093-OtA5097
Syt1	chr10:108497649-109010975	OtA5088-OtA5092	OtA5093-OtA5097
Syt10	chr15:89782392-89841860	OtA5088-OtA5092	OtA5093-OtA5097
Syt12	chr19:4425456-4477143	OtA5088-OtA5092	OtA5093-OtA5097
Syt15	chr14:34220045-34230421	OtA5088-OtA5092	OtA5093-OtA5097
Syt16	chr12:73997760-74267916	OtA5088-OtA5092	OtA5093-OtA5097
Syt17	chr7:118381855-118443552	OtA5088-OtA5092	OtA5093-OtA5097
Syt2	chr1:134646680-134749417	OtA5088-OtA5092	OtA5093-OtA5097
Syt3	chr7:44384125-44400030	OtA5088-OtA5092	OtA5093-OtA5097
Syt4	chr18:31437807-31447415	OtA5088-OtA5092	OtA5093-OtA5097
Syt5	chr7:4539764-4546567	OtA5088-OtA5092	OtA5093-OtA5097
Syt6	chr3:103575280-103635179	OtA5088-OtA5092	OtA5093-OtA5097
Syt7	chr19:10389089-10453181	OtA5088-OtA5092	OtA5093-OtA5097
Syt9	chr7:107284774-107591365	OtA5088-OtA5092	OtA5093-OtA5097
Sytl2	chr7:90348698-90410439	OtA5088-OtA5092	OtA5093-OtA5097
Sytl3	chr17:6673609-6738044	OtA5088-OtA5092	OtA5093-OtA5097
Sytl4	chrX:133936384-133981783	OtA5088-OtA5092	OtA5093-OtA5097
Sytl5	chrX:9885621-9994543	OtA5088-OtA5092	OtA5093-OtA5097
T	chr17:8434422-8442496	OtA5088-OtA5092	OtA5093-OtA5097
T2	chr17:8372395-8422726	OtA5088-OtA5092	OtA5093-OtA5097
Taar1	chr10:23920405-23921404	OtA5088-OtA5092	OtA5093-OtA5097
Taar2	chr10:23938571-23941583	OtA5088-OtA5092	OtA5093-OtA5097
Taar3	chr10:23949557-23950589	OtA5088-OtA5092	OtA5093-OtA5097
Taar4	chr10:23960493-23961537	OtA5088-OtA5092	OtA5093-OtA5097
Taar5	chr10:23970705-23971719	OtA5088-OtA5092	OtA5093-OtA5097

Taar6	chr10:23984608-23985646	OtA5088-OtA5092	OtA5093-OtA5097
Taar7a	chr10:23992404-23993481	OtA5088-OtA5092	OtA5093-OtA5097
Taar7b	chr10:23999938-24001015	OtA5088-OtA5092	OtA5093-OtA5097
Taar7d	chr10:24027221-24028298	OtA5088-OtA5092	OtA5093-OtA5097
Taar7e	chr10:24037613-24038690	OtA5088-OtA5092	OtA5093-OtA5097
Taar7f	chr10:24049509-24050586	OtA5088-OtA5092	OtA5093-OtA5097
Taar8a	chr10:24076499-24077534	OtA5088-OtA5092	OtA5093-OtA5097
Taar8b	chr10:24091259-24092294	OtA5088-OtA5092	OtA5093-OtA5097
Taar8c	chr10:24100842-24101951	OtA5088-OtA5092	OtA5093-OtA5097
Taar9	chr10:24108487-24109534	OtA5088-OtA5092	OtA5093-OtA5097
Tac1	chr6:7555070-7562973	OtA5088-OtA5092	OtA5093-OtA5097
Tac2	chr10:127724477-127731768	OtA5088-OtA5092	OtA5093-OtA5097
Tac4	chr11:95261528-95269261	OtA5088-OtA5092	OtA5093-OtA5097
Tacr1	chr6:82402474-82560104	OtA5088-OtA5092	OtA5093-OtA5097
Tacr2	chr10:62252437-62265990	OtA5088-OtA5092	OtA5093-OtA5097
Tacr3	chr3:134829006-134934581	OtA5088-OtA5092	OtA5093-OtA5097
Tagln3	chr16:45711229-45724531	OtA5088-OtA5092	OtA5093-OtA5097
Tal1	chr4:115059526-115071755	OtA5088-OtA5092	OtA5093-OtA5097
Tal2	chr4:53779704-53786885	OtA5088-OtA5092	OtA5093-OtA5097
Tarm1	chr7:3489075-3502552	OtA5088-OtA5092	OtA5093-OtA5097
Tarsl2	chr7:65644897-65692093	OtA5088-OtA5092	OtA5093-OtA5097
Tas1r1	chr4:152027913-152038490	OtA5088-OtA5092	OtA5093-OtA5097
Tas1r2	chr4:139653537-139670279	OtA5088-OtA5092	OtA5093-OtA5097
Tas1r3	chr4:155847411-155863353	OtA5088-OtA5092	OtA5093-OtA5097
Tas2r102	chr6:132762130-132763174	OtA5088-OtA5092	OtA5093-OtA5097
Tas2r103	chr6:133036162-133037101	OtA5088-OtA5092	OtA5093-OtA5097
Tas2r104	chr6:131684835-131685744	OtA5088-OtA5092	OtA5093-OtA5097
Tas2r105	chr6:131686560-131687463	OtA5088-OtA5092	OtA5093-OtA5097
Tas2r106	chr6:131677959-131678886	OtA5088-OtA5092	OtA5093-OtA5097
Tas2r107	chr6:131659157-131660084	OtA5088-OtA5092	OtA5093-OtA5097
Tas2r108	chr6:40493591-40494485	OtA5088-OtA5092	OtA5093-OtA5097
Tas2r109	chr6:132980014-132980965	OtA5088-OtA5092	OtA5093-OtA5097
Tas2r110	chr6:132868007-132869009	OtA5088-OtA5092	OtA5093-OtA5097
Tas2r113	chr6:132893010-132893940	OtA5088-OtA5092	OtA5093-OtA5097
Tas2r114	chr6:131689133-131690063	OtA5088-OtA5092	OtA5093-OtA5097
Tas2r115	chr6:132737053-132737986	OtA5088-OtA5092	OtA5093-OtA5097
Tas2r116	chr6:132855437-132856355	OtA5088-OtA5092	OtA5093-OtA5097
Tas2r117	chr6:132802900-132803893	OtA5088-OtA5092	OtA5093-OtA5097
Tas2r118	chr6:23969160-23970060	OtA5088-OtA5092	OtA5093-OtA5097
Tas2r119	chr15:32177288-32178294	OtA5088-OtA5092	OtA5093-OtA5097
Tas2r120	chr6:132656956-132657844	OtA5088-OtA5092	OtA5093-OtA5097
Tas2r121	chr6:132700089-132701007	OtA5088-OtA5092	OtA5093-OtA5097
Tas2r122	chr6:132710998-132711928	OtA5088-OtA5092	OtA5093-OtA5097
Tas2r123	chr6:132847141-132848143	OtA5088-OtA5092	OtA5093-OtA5097
Tas2r124	chr6:132754729-132755659	OtA5088-OtA5092	OtA5093-OtA5097
Tas2r125	chr6:132909650-132910586	OtA5088-OtA5092	OtA5093-OtA5097
Tas2r126	chr6:42434534-42435461	OtA5088-OtA5092	OtA5093-OtA5097

Tas2r129	chr6:132951101-132952064	OtA5088-OtA5092	OtA5093-OtA5097
Tas2r130	chr6:131629891-131630830	OtA5088-OtA5092	OtA5093-OtA5097
Tas2r131	chr6:132956911-132957844	OtA5088-OtA5092	OtA5093-OtA5097
Tas2r134	chr2:51627510-51628407	OtA5088-OtA5092	OtA5093-OtA5097
Tas2r135	chr6:42405528-42406494	OtA5088-OtA5092	OtA5093-OtA5097
Tas2r136	chr6:132777178-132778162	OtA5088-OtA5092	OtA5093-OtA5097
Tas2r137	chr6:40491237-40492239	OtA5088-OtA5092	OtA5093-OtA5097
Tas2r138	chr6:40529090-40910666	OtA5088-OtA5092	OtA5093-OtA5097
Tas2r139	chr6:42140935-42141895	OtA5088-OtA5092	OtA5093-OtA5097
Tas2r140	chr6:133054854-133055793	OtA5088-OtA5092	OtA5093-OtA5097
Tas2r143	chr6:42400237-42401119	OtA5088-OtA5092	OtA5093-OtA5097
Tas2r144	chr6:42215327-42216287	OtA5088-OtA5092	OtA5093-OtA5097
Tat	chr8:109990435-109999804	OtA5088-OtA5092	OtA5093-OtA5097
Tatdn1	chr15:58889228-58933730	OtA5088-OtA5092	OtA5093-OtA5097
Tbata	chr10:61171963-61188841	OtA5088-OtA5092	OtA5093-OtA5097
Tbc1d10c	chr19:4184356-4191047	OtA5088-OtA5092	OtA5093-OtA5097
Tbc1d21	chr9:58359803-58370369	OtA5088-OtA5092	OtA5093-OtA5097
Tbpl2	chr2:24071720-24096595	OtA5088-OtA5092	OtA5093-OtA5097
Tbr1	chr2:61804452-61814113	OtA5088-OtA5092	OtA5093-OtA5097
Tbx1	chr16:18581712-18586969	OtA5088-OtA5092	OtA5093-OtA5097
Tbx10	chr19:3992751-3999512	OtA5088-OtA5092	OtA5093-OtA5097
Tbx15	chr3:99253759-99354260	OtA5088-OtA5092	OtA5093-OtA5097
Tbx18	chr9:87702799-87731260	OtA5088-OtA5092	OtA5093-OtA5097
Tbx19	chr1:165137852-165160773	OtA5088-OtA5092	OtA5093-OtA5097
Tbx21	chr11:97098006-97115331	OtA5088-OtA5092	OtA5093-OtA5097
Tbx22	chrX:107667963-107688980	OtA5088-OtA5092	OtA5093-OtA5097
Tbx5	chr5:119834662-119885218	OtA5088-OtA5092	OtA5093-OtA5097
Tbx6	chr7:126781482-126785548	OtA5088-OtA5092	OtA5093-OtA5097
Tbxa2r	chr10:81328730-81335172	OtA5088-OtA5092	OtA5093-OtA5097
Tbxas1	chr6:38918985-39084579	OtA5088-OtA5092	OtA5093-OtA5097
Tc2n	chr12:101648601-101718502	OtA5088-OtA5092	OtA5093-OtA5097
Tcam1	chr11:106276671-106288143	OtA5088-OtA5092	OtA5093-OtA5097
Tcap	chr11:98383810-98384953	OtA5088-OtA5092	OtA5093-OtA5097
Tceal3	chrX:136666374-136668377	OtA5088-OtA5092	OtA5093-OtA5097
Tceal5	chrX:136200950-136203851	OtA5088-OtA5092	OtA5093-OtA5097
Tceal6	chrX:135208685-135210687	OtA5088-OtA5092	OtA5093-OtA5097
Tceal7	chrX:136224040-136226100	OtA5088-OtA5092	OtA5093-OtA5097
Tcerg1l	chr7:138208971-138397730	OtA5088-OtA5092	OtA5093-OtA5097
Tcf7	chr11:52252603-52282571	OtA5088-OtA5092	OtA5093-OtA5097
Tcfl5	chr2:180598221-180642691	OtA5088-OtA5092	OtA5093-OtA5097
Tcl1	chr12:105216754-105222737	OtA5088-OtA5092	OtA5093-OtA5097
Tcl1b1	chr12:105159703-105166625	OtA5088-OtA5092	OtA5093-OtA5097
Tcl1b2	chr12:105147032-105155225	OtA5088-OtA5092	OtA5093-OtA5097
Tcl1b3	chr12:105191044-105195399	OtA5088-OtA5092	OtA5093-OtA5097
Tcl1b4	chr12:105202421-105206993	OtA5088-OtA5092	OtA5093-OtA5097
Tcl1b5	chr12:105176357-105181145	OtA5088-OtA5092	OtA5093-OtA5097
Tcp10a	chr17:7324659-7345860	OtA5088-OtA5092	OtA5093-OtA5097

Tcp10b	chr17:13061110-13082226	OtA5088-OtA5092	OtA5093-OtA5097
Tcp10c	chr17:13354571-13377223	OtA5088-OtA5092	OtA5093-OtA5097
Tcp11	chr17:28066746-28080639	OtA5088-OtA5092	OtA5093-OtA5097
Tcstv1	chr13:119893387-119894767	OtA5088-OtA5092	OtA5093-OtA5097
Tcstv3	chr13:120316861-120318261	OtA5088-OtA5092	OtA5093-OtA5097
Tcte1	chr17:45523433-45549677	OtA5088-OtA5092	OtA5093-OtA5097
Tcte2	chr17:13716435-13905794	OtA5088-OtA5092	OtA5093-OtA5097
Tcte3	chr17:14964188-15022449	OtA5088-OtA5092	OtA5093-OtA5097
Tctex1d1	chr4:102760362-103011965	OtA5088-OtA5092	OtA5093-OtA5097
Tctex1d4	chr4:117126812-117133952	OtA5088-OtA5092	OtA5093-OtA5097
Tdgf1	chr9:110939607-110946158	OtA5088-OtA5092	OtA5093-OtA5097
Tdh	chr14:63492346-63509092	OtA5088-OtA5092	OtA5093-OtA5097
Tdpoz1	chr3:93669332-93676283	OtA5088-OtA5092	OtA5093-OtA5097
Tdpoz2	chr3:93651570-93652663	OtA5088-OtA5092	OtA5093-OtA5097
Tdpoz3	chr3:93826019-93827117	OtA5088-OtA5092	OtA5093-OtA5097
Tdpoz4	chr3:93796397-93797510	OtA5088-OtA5092	OtA5093-OtA5097
Tdpoz5	chr3:93960291-93961314	OtA5088-OtA5092	OtA5093-OtA5097
Tdrd12	chr7:35493609-35537744	OtA5088-OtA5092	OtA5093-OtA5097
Tdrd5	chr1:156255295-156303348	OtA5088-OtA5092	OtA5093-OtA5097
Tdrd6	chr17:43615334-43630299	OtA5088-OtA5092	OtA5093-OtA5097
Tdrd9	chr12:111971558-112068854	OtA5088-OtA5092	OtA5093-OtA5097
Tdrkh	chr3:94413317-94431499	OtA5088-OtA5092	OtA5093-OtA5097
Tecrl	chr5:83278121-83355195	OtA5088-OtA5092	OtA5093-OtA5097
Tecta	chr9:42329621-42399929	OtA5088-OtA5092	OtA5093-OtA5097
Tectb	chr19:55180884-55196313	OtA5088-OtA5092	OtA5093-OtA5097
Teddm1	chr1:153891645-153893060	OtA5088-OtA5092	OtA5093-OtA5097
Tekt1	chr11:72344719-72361880	OtA5088-OtA5092	OtA5093-OtA5097
Tekt2	chr4:126322120-126325199	OtA5088-OtA5092	OtA5093-OtA5097
Tekt3	chr11:63061658-63094960	OtA5088-OtA5092	OtA5093-OtA5097
Tekt4	chr17:25471589-25476594	OtA5088-OtA5092	OtA5093-OtA5097
Tekt5	chr16:10361253-10395448	OtA5088-OtA5092	OtA5093-OtA5097
Tepp	chr8:95311514-95321328	OtA5088-OtA5092	OtA5093-OtA5097
Tesc	chr5:118027823-118061870	OtA5088-OtA5092	OtA5093-OtA5097
Tespa1	chr10:130322851-130362642	OtA5088-OtA5092	OtA5093-OtA5097
Tet1	chr10:62804569-62880014	OtA5088-OtA5092	OtA5093-OtA5097
Tex101	chr7:24668011-24672050	OtA5088-OtA5092	OtA5093-OtA5097
Tex11	chrX:100838647-101059639	OtA5088-OtA5092	OtA5093-OtA5097
Tex12	chr9:50557147-50561268	OtA5088-OtA5092	OtA5093-OtA5097
Tex13a	chrX:137571548-138846955	OtA5088-OtA5092	OtA5093-OtA5097
Tex14	chr11:87405064-87555823	OtA5088-OtA5092	OtA5093-OtA5097
Tex15	chr8:33570543-33585585	OtA5088-OtA5092	OtA5093-OtA5097
Tex16	chrX:112093519-112127326	OtA5088-OtA5092	OtA5093-OtA5097
Tex19.2	chr11:121116214-121118677	OtA5088-OtA5092	OtA5093-OtA5097
Tex21	chr12:76198689-76246746	OtA5088-OtA5092	OtA5093-OtA5097
Tex22	chr12:113074501-113088914	OtA5088-OtA5092	OtA5093-OtA5097
Tex24	chr8:27344393-27349188	OtA5088-OtA5092	OtA5093-OtA5097
Tex28	chrX:74150943-74167838	OtA5088-OtA5092	OtA5093-OtA5097

Tex9	chr9:72438528-72491959	OtA5088-OtA5092	OtA5093-OtA5097
Tfap2b	chr1:19208913-19238734	OtA5088-OtA5092	OtA5093-OtA5097
Tfap2d	chr1:19103021-19166332	OtA5088-OtA5092	OtA5093-OtA5097
Tfap2e	chr4:126716002-126736269	OtA5088-OtA5092	OtA5093-OtA5097
Tfap4	chr16:4544660-4559720	OtA5088-OtA5092	OtA5093-OtA5097
Tfec	chr6:16833380-16898441	OtA5088-OtA5092	OtA5093-OtA5097
Tff2	chr17:31141061-31144282	OtA5088-OtA5092	OtA5093-OtA5097
Tfr2	chr5:137569850-137587481	OtA5088-OtA5092	OtA5093-OtA5097
Tg	chr15:66670769-66850720	OtA5088-OtA5092	OtA5093-OtA5097
Tgif2lx1,Tgif2lx2	chrX:118479705-118480737	OtA5088-OtA5092	OtA5093-OtA5097
Tgif2lx2	chrX:118479705-118480737	OtA5088-OtA5092	OtA5093-OtA5097
Tgm3	chr2:130012373-130050399	OtA5088-OtA5092	OtA5093-OtA5097
Tgm4	chr9:123034740-123067558	OtA5088-OtA5092	OtA5093-OtA5097
Tgm5	chr2:121046110-121085759	OtA5088-OtA5092	OtA5093-OtA5097
Tgm6	chr2:130123284-130154225	OtA5088-OtA5092	OtA5093-OtA5097
Tgm7	chr2:121093588-121109791	OtA5088-OtA5092	OtA5093-OtA5097
Tgtp1	chr11:48985328-48992246	OtA5088-OtA5092	OtA5093-OtA5097
Tgtp2	chr11:49037659-49135387	OtA5088-OtA5092	OtA5093-OtA5097
Th	chr7:142892775-142899966	OtA5088-OtA5092	OtA5093-OtA5097
Tha1	chr11:117867948-117873526	OtA5088-OtA5092	OtA5093-OtA5097
Thbs4	chr13:92751585-92794818	OtA5088-OtA5092	OtA5093-OtA5097
Theg	chr10:79576476-79587136	OtA5088-OtA5092	OtA5093-OtA5097
Them5	chr3:94342098-94347352	OtA5088-OtA5092	OtA5093-OtA5097
Themis	chr10:28668394-28882749	OtA5088-OtA5092	OtA5093-OtA5097
Thnsl1	chr2:21205723-21215009	OtA5088-OtA5092	OtA5093-OtA5097
Thpo	chr16:20724453-20742384	OtA5088-OtA5092	OtA5093-OtA5097
Thrb	chr14:17660959-18038088	OtA5088-OtA5092	OtA5093-OtA5097
Thsd7b	chr1:129273303-130219278	OtA5088-OtA5092	OtA5093-OtA5097
Thumpd2	chr17:81026326-81065085	OtA5088-OtA5092	OtA5093-OtA5097
Ticam2	chr18:46558230-46574533	OtA5088-OtA5092	OtA5093-OtA5097
Tifa	chr3:127789912-127798389	OtA5088-OtA5092	OtA5093-OtA5097
Tifab	chr13:56173702-56178885	OtA5088-OtA5092	OtA5093-OtA5097
Tigd3	chr19:5891137-5894107	OtA5088-OtA5092	OtA5093-OtA5097
Tigd4	chr3:84593573-84597032	OtA5088-OtA5092	OtA5093-OtA5097
Tigit	chr16:43648860-43664184	OtA5088-OtA5092	OtA5093-OtA5097
Timd4	chr11:46810798-46844333	OtA5088-OtA5092	OtA5093-OtA5097
Timm8a1	chrX:134537257-134541629	OtA5088-OtA5092	OtA5093-OtA5097
Timm8a2	chr14:122034673-122038422	OtA5088-OtA5092	OtA5093-OtA5097
Timp4	chr6:115134901-115282626	OtA5088-OtA5092	OtA5093-OtA5097
Tktl1	chrX:74177258-74208498	OtA5088-OtA5092	OtA5093-OtA5097
Tktl2	chr8:66511739-66513778	OtA5088-OtA5092	OtA5093-OtA5097
Tle2	chr10:81575286-81590845	OtA5088-OtA5092	OtA5093-OtA5097
Tll1	chr8:64014769-64205993	OtA5088-OtA5092	OtA5093-OtA5097
Tll2	chr19:41083980-41206774	OtA5088-OtA5092	OtA5093-OtA5097
Tlr1	chr5:64924833-64932699	OtA5088-OtA5092	OtA5093-OtA5097
Tlr11	chr14:50360501-50363663	OtA5088-OtA5092	OtA5093-OtA5097
Tlr13	chrX:106143274-106160493	OtA5088-OtA5092	OtA5093-OtA5097

Tlr3	chr8:45395664-45410539	OtA5088-OtA5092	OtA5093-OtA5097
Tlr6	chr5:64953094-64960034	OtA5088-OtA5092	OtA5093-OtA5097
Tlr7	chrX:167304930-167330528	OtA5088-OtA5092	OtA5093-OtA5097
Tlr8	chrX:167242731-167263788	OtA5088-OtA5092	OtA5093-OtA5097
Tlr9	chr9:106222597-106226876	OtA5088-OtA5092	OtA5093-OtA5097
Tlx1	chr19:45150714-45156943	OtA5088-OtA5092	OtA5093-OtA5097
Tlx2	chr6:83068324-83070225	OtA5088-OtA5092	OtA5093-OtA5097
Tlx3	chr11:33200751-33203588	OtA5088-OtA5092	OtA5093-OtA5097
Tm4sf19	chr16:32400505-32408227	OtA5088-OtA5092	OtA5093-OtA5097
Tm4sf20	chr1:82756648-82768456	OtA5088-OtA5092	OtA5093-OtA5097
Tm4sf4	chr3:57425409-57441675	OtA5088-OtA5092	OtA5093-OtA5097
Tm6sf2	chr8:70072931-70080052	OtA5088-OtA5092	OtA5093-OtA5097
Tmc1	chr19:20783457-20954202	OtA5088-OtA5092	OtA5093-OtA5097
Tmc2	chr2:130195193-130264445	OtA5088-OtA5092	OtA5093-OtA5097
Tmc3	chr7:83584930-83623709	OtA5088-OtA5092	OtA5093-OtA5097
Tmco2	chr4:121105650-121109226	OtA5088-OtA5092	OtA5093-OtA5097
Tmco5	chr2:116878690-116892500	OtA5088-OtA5092	OtA5093-OtA5097
Tmco5b	chr2:113220186-113716767	OtA5088-OtA5092	OtA5093-OtA5097
Tmed11	chr5:108777234-108795363	OtA5088-OtA5092	OtA5093-OtA5097
Tmed6	chr8:107061483-107065644	OtA5088-OtA5092	OtA5093-OtA5097
Tmeff2	chr1:50927522-51187270	OtA5088-OtA5092	OtA5093-OtA5097
Tmem117	chr15:94629184-95096097	OtA5088-OtA5092	OtA5093-OtA5097
Tmem121	chr12:113185902-113189522	OtA5088-OtA5092	OtA5093-OtA5097
Tmem132b	chr5:125532417-125792583	OtA5088-OtA5092	OtA5093-OtA5097
Tmem132c	chr5:127241825-127565790	OtA5088-OtA5092	OtA5093-OtA5097
Tmem132d	chr5:127783490-128433077	OtA5088-OtA5092	OtA5093-OtA5097
Tmem145	chr7:25306107-25316195	OtA5088-OtA5092	OtA5093-OtA5097
Tmem146	chr17:56628142-56664456	OtA5088-OtA5092	OtA5093-OtA5097
Tmem149	chr7:30565426-30567962	OtA5088-OtA5092	OtA5093-OtA5097
Tmem14a	chr1:21218630-21230167	OtA5088-OtA5092	OtA5093-OtA5097
Tmem150b	chr7:4690927-4725251	OtA5088-OtA5092	OtA5093-OtA5097
Tmem150c	chr5:100077872-100159808	OtA5088-OtA5092	OtA5093-OtA5097
Tmem151a	chr19:5079336-5085477	OtA5088-OtA5092	OtA5093-OtA5097
Tmem151b	chr17:45523433-45549677	OtA5088-OtA5092	OtA5093-OtA5097
Tmem163	chr1:127490341-127678021	OtA5088-OtA5092	OtA5093-OtA5097
Tmem169	chr1:72284372-72302995	OtA5088-OtA5092	OtA5093-OtA5097
Tmem171	chr13:98686237-98694831	OtA5088-OtA5092	OtA5093-OtA5097
Tmem174	chr13:98634977-98637410	OtA5088-OtA5092	OtA5093-OtA5097
Tmem179	chr12:112500183-112511160	OtA5088-OtA5092	OtA5093-OtA5097
Tmem181c-ps	chr17:6610102-6620925	OtA5088-OtA5092	OtA5093-OtA5097
Tmem182	chr1:40805600-40855267	OtA5088-OtA5092	OtA5093-OtA5097
Tmem190	chr7:4782939-4784340	OtA5088-OtA5092	OtA5093-OtA5097
Tmem194b	chr1:52630704-52651919	OtA5088-OtA5092	OtA5093-OtA5097
Tmem196	chr12:119945961-120018706	OtA5088-OtA5092	OtA5093-OtA5097
Tmem200a	chr10:25991185-26079447	OtA5088-OtA5092	OtA5093-OtA5097
Tmem202	chr9:59518684-59525501	OtA5088-OtA5092	OtA5093-OtA5097
Tmem207	chr16:26503792-26526771	OtA5088-OtA5092	OtA5093-OtA5097

Tmem210	chr2:25288144-25289187	OtA5088-OtA5092	OtA5093-OtA5097
Tmem211	chr5:113226908-113239263	OtA5088-OtA5092	OtA5093-OtA5097
Tmem212	chr3:27866064-27896368	OtA5088-OtA5092	OtA5093-OtA5097
Tmem215	chr4:40472179-40475653	OtA5088-OtA5092	OtA5093-OtA5097
Tmem217	chr17:29526017-29549593	OtA5088-OtA5092	OtA5093-OtA5097
Tmem22	chr9:100552187-100571085	OtA5088-OtA5092	OtA5093-OtA5097
Tmem221	chr8:71554302-71558871	OtA5088-OtA5092	OtA5093-OtA5097
Tmem225	chr9:40148121-40150878	OtA5088-OtA5092	OtA5093-OtA5097
Tmem229a	chr6:24951140-24956125	OtA5088-OtA5092	OtA5093-OtA5097
Tmem232	chr17:65256004-65540782	OtA5088-OtA5092	OtA5093-OtA5097
Tmem233	chr5:116040533-116083246	OtA5088-OtA5092	OtA5093-OtA5097
Tmem25	chr9:44793778-44799216	OtA5088-OtA5092	OtA5093-OtA5097
Tmem26	chr10:68723745-68782654	OtA5088-OtA5092	OtA5093-OtA5097
Tmem27	chrX:164090186-164118859	OtA5088-OtA5092	OtA5093-OtA5097
Tmem28	chrX:99821068-99846345	OtA5088-OtA5092	OtA5093-OtA5097
Tmem30c	chr16:57266138-57292851	OtA5088-OtA5092	OtA5093-OtA5097
Tmem35	chrX:134295224-134305969	OtA5088-OtA5092	OtA5093-OtA5097
Tmem44	chr16:30312423-31275697	OtA5088-OtA5092	OtA5093-OtA5097
Tmem45a	chr16:56805160-56886163	OtA5088-OtA5092	OtA5093-OtA5097
Tmem47	chrX:81070643-81097875	OtA5088-OtA5092	OtA5093-OtA5097
Tmem52	chr4:155469113-155470858	OtA5088-OtA5092	OtA5093-OtA5097
Tmem56	chr3:121202009-121263316	OtA5088-OtA5092	OtA5093-OtA5097
Tmem63c	chr12:87026563-87090039	OtA5088-OtA5092	OtA5093-OtA5097
Tmem69	chr4:116551527-116555943	OtA5088-OtA5092	OtA5093-OtA5097
Tmem71	chr15:66526211-66561046	OtA5088-OtA5092	OtA5093-OtA5097
Tmem72	chr6:116692724-116716758	OtA5088-OtA5092	OtA5093-OtA5097
Tmem74	chr15:43866694-43870029	OtA5088-OtA5092	OtA5093-OtA5097
Tmem81	chr1:132506229-132508639	OtA5088-OtA5092	OtA5093-OtA5097
Tmem86b	chr7:4628039-4630482	OtA5088-OtA5092	OtA5093-OtA5097
Tmem88b	chr4:155781590-155785874	OtA5088-OtA5092	OtA5093-OtA5097
Tmem89	chr9:108914618-108915563	OtA5088-OtA5092	OtA5093-OtA5097
Tmem8c	chr2:27061635-27072161	OtA5088-OtA5092	OtA5093-OtA5097
Tmem90a	chr12:84677277-84698807	OtA5088-OtA5092	OtA5093-OtA5097
Tmem90b	chr2:149830782-150004392	OtA5088-OtA5092	OtA5093-OtA5097
Tmem91	chr7:25669139-25675166	OtA5088-OtA5092	OtA5093-OtA5097
Tmem92	chr11:94777216-94782703	OtA5088-OtA5092	OtA5093-OtA5097
Tmem95	chr11:69876683-69878018	OtA5088-OtA5092	OtA5093-OtA5097
Tmie	chr9:110866045-110880083	OtA5088-OtA5092	OtA5093-OtA5097
Tmigd1	chr11:76904544-76916586	OtA5088-OtA5092	OtA5093-OtA5097
Tmlhe	chrX_GL456233_random:159646-33418	OtA5088-OtA5092	OtA5093-OtA5097
Tmod2	chr9:75565621-75611325	OtA5088-OtA5092	OtA5093-OtA5097
Tmod4	chr3:95124513-95129208	OtA5088-OtA5092	OtA5093-OtA5097
Tmprss11a	chr5:86410409-86468990	OtA5088-OtA5092	OtA5093-OtA5097
Tmprss11bnl	chr5:86659521-86676298	OtA5088-OtA5092	OtA5093-OtA5097
Tmprss11c	chr5:86231480-86289308	OtA5088-OtA5092	OtA5093-OtA5097
Tmprss11d	chr5:86302853-86373387	OtA5088-OtA5092	OtA5093-OtA5097
Tmprss11e	chr5:86705185-86745807	OtA5088-OtA5092	OtA5093-OtA5097

Tmprss11f	chr5:86521225-86632424	OtA5088-OtA5092	OtA5093-OtA5097
Tmprss11g	chr5:86485876-86518600	OtA5088-OtA5092	OtA5093-OtA5097
Tmprss12	chr15:100280836-100293053	OtA5088-OtA5092	OtA5093-OtA5097
Tmprss13	chr9:45318265-45347580	OtA5088-OtA5092	OtA5093-OtA5097
Tmprss15	chr16:78953463-79091097	OtA5088-OtA5092	OtA5093-OtA5097
Tmprss3	chr17:31179267-31198974	OtA5088-OtA5092	OtA5093-OtA5097
Tmprss5	chr9:49102778-49117587	OtA5088-OtA5092	OtA5093-OtA5097
Tmprss7	chr16:45656316-45693658	OtA5088-OtA5092	OtA5093-OtA5097
Tmprss9	chr10:80879815-80900969	OtA5088-OtA5092	OtA5093-OtA5097
Tmsb15b1	chrX:136954987-136976874	OtA5088-OtA5092	OtA5093-OtA5097
Tmsb15b2	chrX:136954987-136976874	OtA5088-OtA5092	OtA5093-OtA5097
Tmsb15l	chrX:136954987-136976874	OtA5088-OtA5092	OtA5093-OtA5097
Tmtc2	chr10:105187663-105574479	OtA5088-OtA5092	OtA5093-OtA5097
Tnfaip6	chr2:52038112-52056681	OtA5088-OtA5092	OtA5093-OtA5097
Tnfaip8l2	chr3:95134087-95142360	OtA5088-OtA5092	OtA5093-OtA5097
Tnfaip8l3	chr9:54025605-54068411	OtA5088-OtA5092	OtA5093-OtA5097
Tnfrsf11a	chr1:105780722-105847981	OtA5088-OtA5092	OtA5093-OtA5097
Tnfrsf13b	chr11:61140834-61147642	OtA5088-OtA5092	OtA5093-OtA5097
Tnfrsf13c	chr15:82221743-82224336	OtA5088-OtA5092	OtA5093-OtA5097
Tnfrsf17	chr16:11313808-11320068	OtA5088-OtA5092	OtA5093-OtA5097
Tnfrsf18	chr4:156026341-156028891	OtA5088-OtA5092	OtA5093-OtA5097
Tnfrsf19	chr14:60963833-61046855	OtA5088-OtA5092	OtA5093-OtA5097
Tnfrsf22	chr7:143636724-143649638	OtA5088-OtA5092	OtA5093-OtA5097
Tnfrsf25	chr4:152116342-152120111	OtA5088-OtA5092	OtA5093-OtA5097
Tnfrsf26	chr7:143607684-143627845	OtA5088-OtA5092	OtA5093-OtA5097
Tnfrsf4	chr4:156013694-156016589	OtA5088-OtA5092	OtA5093-OtA5097
Tnfrsf8	chr4:145268975-145315147	OtA5088-OtA5092	OtA5093-OtA5097
Tnfsf11	chr14:78277445-78308043	OtA5088-OtA5092	OtA5093-OtA5097
Tnfsf13	chr11:69682576-69696098	OtA5088-OtA5092	OtA5093-OtA5097
Tnfsf13b	chr8:10006632-10035999	OtA5088-OtA5092	OtA5093-OtA5097
Tnfsf14	chr17:57189491-57194186	OtA5088-OtA5092	OtA5093-OtA5097
Tnfsf15	chr4:63724602-63745113	OtA5088-OtA5092	OtA5093-OtA5097
Tnfsf18	chr1:161494656-161505290	OtA5088-OtA5092	OtA5093-OtA5097
Tnfsf4	chr1:161395437-161418206	OtA5088-OtA5092	OtA5093-OtA5097
Tnfsf8	chr4:63832823-63861284	OtA5088-OtA5092	OtA5093-OtA5097
Tnik	chr3:28263213-28670585	OtA5088-OtA5092	OtA5093-OtA5097
Tnip3	chr6:65590397-65634040	OtA5088-OtA5092	OtA5093-OtA5097
Tnmd	chrX:133851007-133865578	OtA5088-OtA5092	OtA5093-OtA5097
Tnn	chr1:160085031-160153575	OtA5088-OtA5092	OtA5093-OtA5097
Tnnc2	chr2:164777161-164779734	OtA5088-OtA5092	OtA5093-OtA5097
Tnni1	chr1:135799420-135810989	OtA5088-OtA5092	OtA5093-OtA5097
Tnni2	chr7:142442467-142444405	OtA5088-OtA5092	OtA5093-OtA5097
Tnni3	chr7:4518307-4522443	OtA5088-OtA5092	OtA5093-OtA5097
Tnni3k	chr3:154786290-155055407	OtA5088-OtA5092	OtA5093-OtA5097
Tnnt1	chr7:4504662-4515975	OtA5088-OtA5092	OtA5093-OtA5097
Tnnt3	chr7:142460869-142516009	OtA5088-OtA5092	OtA5093-OtA5097
Tnp1	chr1:73015074-73015899	OtA5088-OtA5092	OtA5093-OtA5097

Tnp2	chr16:10787934-10788655	OtA5088-OtA5092	OtA5093-OtA5097
Tnr	chr1:159523768-159924922	OtA5088-OtA5092	OtA5093-OtA5097
Tomm20l	chr12:71111427-71136675	OtA5088-OtA5092	OtA5093-OtA5097
Tomt	chr7:101899807-101911903	OtA5088-OtA5092	OtA5093-OtA5097
Top2a	chr11:98992946-99024189	OtA5088-OtA5092	OtA5093-OtA5097
Tox	chr4:6687385-6990723	OtA5088-OtA5092	OtA5093-OtA5097
Tox3	chr8:90247111-90348252	OtA5088-OtA5092	OtA5093-OtA5097
Tpd52l1	chr10:31332379-31445921	OtA5088-OtA5092	OtA5093-OtA5097
Tph2	chr10:115078640-115185022	OtA5088-OtA5092	OtA5093-OtA5097
Tpmt	chr13:47023542-47043217	OtA5088-OtA5092	OtA5093-OtA5097
Tpo	chr12:30054660-30132609	OtA5088-OtA5092	OtA5093-OtA5097
Tppp2	chr14:51918760-51920700	OtA5088-OtA5092	OtA5093-OtA5097
Tprg	chr16:25286816-25422344	OtA5088-OtA5092	OtA5093-OtA5097
Tpsab1	chr17:25343244-25345562	OtA5088-OtA5092	OtA5093-OtA5097
Tpsb2	chr17:25366332-25368092	OtA5088-OtA5092	OtA5093-OtA5097
Tpsg1	chr17:25370552-25433783	OtA5088-OtA5092	OtA5093-OtA5097
Tpte	chr8:22283440-22371418	OtA5088-OtA5092	OtA5093-OtA5097
Tpx2	chr2:152847963-152895321	OtA5088-OtA5092	OtA5093-OtA5097
Traf1	chr2:34943257-34961772	OtA5088-OtA5092	OtA5093-OtA5097
Traf3ip3	chr1:193173468-193201546	OtA5088-OtA5092	OtA5093-OtA5097
Traip	chr9:107950962-107972268	OtA5088-OtA5092	OtA5093-OtA5097
Tram1l1	chr3:124321036-124323260	OtA5088-OtA5092	OtA5093-OtA5097
Trat1	chr16:48734689-48771956	OtA5088-OtA5092	OtA5093-OtA5097
Trcg1	chr9:57236555-57249864	OtA5088-OtA5092	OtA5093-OtA5097
Trdmt1	chr2:13510160-13544664	OtA5088-OtA5092	OtA5093-OtA5097
Trdn	chr10:33083482-33476709	OtA5088-OtA5092	OtA5093-OtA5097
Treh	chr9:44673237-44686304	OtA5088-OtA5092	OtA5093-OtA5097
Trem1	chr17:48232738-48246924	OtA5088-OtA5092	OtA5093-OtA5097
Trem3	chr17:48247776-48258841	OtA5088-OtA5092	OtA5093-OtA5097
Treml1	chr17:48359915-48367146	OtA5088-OtA5092	OtA5093-OtA5097
Treml2	chr17:48300037-48312534	OtA5088-OtA5092	OtA5093-OtA5097
Treml4	chr17:48264294-48275358	OtA5088-OtA5092	OtA5093-OtA5097
Trex2	chrX:73433704-73435343	OtA5088-OtA5092	OtA5093-OtA5097
Trh	chr6:92242061-92244648	OtA5088-OtA5092	OtA5093-OtA5097
Trhde	chr10:114398820-114801370	OtA5088-OtA5092	OtA5093-OtA5097
Trhr	chr15:44196134-44235912	OtA5088-OtA5092	OtA5093-OtA5097
Trim12a	chr7:104299896-104324838	OtA5088-OtA5092	OtA5093-OtA5097
Trim17	chr11:58963780-58971729	OtA5088-OtA5092	OtA5093-OtA5097
Trim30b	chr7:104355397-104358646	OtA5088-OtA5092	OtA5093-OtA5097
Trim30d	chr7:104470013-104507849	OtA5088-OtA5092	OtA5093-OtA5097
Trim30e-ps1	chr7:104532958-104535361	OtA5088-OtA5092	OtA5093-OtA5097
Trim31	chr17:36898129-36910217	OtA5088-OtA5092	OtA5093-OtA5097
Trim34b	chr7:104329470-104336617	OtA5088-OtA5092	OtA5093-OtA5097
Trim36	chr18:46165299-46212607	OtA5088-OtA5092	OtA5093-OtA5097
Trim38	chr13:23782571-23791495	OtA5088-OtA5092	OtA5093-OtA5097
Trim40	chr17:36881597-36890125	OtA5088-OtA5092	OtA5093-OtA5097
Trim42	chr9:97349561-97369958	OtA5088-OtA5092	OtA5093-OtA5097

Trim43a	chr9:88581035-88588819	OtA5088-OtA5092	OtA5093-OtA5097
Trim43b	chr9:89084623-89092835	OtA5088-OtA5092	OtA5093-OtA5097
Trim43c	chr9:88839163-88858870	OtA5088-OtA5092	OtA5093-OtA5097
Trim50	chr5:135353295-135367654	OtA5088-OtA5092	OtA5093-OtA5097
Trim52	chr14:106106197-106109280	OtA5088-OtA5092	OtA5093-OtA5097
Trim54	chr5:31116611-31137626	OtA5088-OtA5092	OtA5093-OtA5097
Trim55	chr3:19644459-19691599	OtA5088-OtA5092	OtA5093-OtA5097
Trim58	chr11:58640464-58652404	OtA5088-OtA5092	OtA5093-OtA5097
Trim59	chr3:69035293-69044742	OtA5088-OtA5092	OtA5093-OtA5097
Trim60	chr8:64998447-65018688	OtA5088-OtA5092	OtA5093-OtA5097
Trim61	chr8:64998447-65018688	OtA5088-OtA5092	OtA5093-OtA5097
Trim66	chr7:109449000-109508134	OtA5088-OtA5092	OtA5093-OtA5097
Trim67	chr8:124793018-124834704	OtA5088-OtA5092	OtA5093-OtA5097
Trim69	chr2:122120107-122186189	OtA5088-OtA5092	OtA5093-OtA5097
Trim72	chr7:128004377-128011393	OtA5088-OtA5092	OtA5093-OtA5097
Trim75	chr8:64981650-64987644	OtA5088-OtA5092	OtA5093-OtA5097
Trim9	chr12:70244534-70669399	OtA5088-OtA5092	OtA5093-OtA5097
Trip13	chr13:73912461-73937767	OtA5088-OtA5092	OtA5093-OtA5097
Trmt11	chr10:30534224-30600749	OtA5088-OtA5092	OtA5093-OtA5097
Troap	chr15:99074972-99083409	OtA5088-OtA5092	OtA5093-OtA5097
Trp53rk	chr2:166793766-166799492	OtA5088-OtA5092	OtA5093-OtA5097
Trp73	chr4:154056248-154140208	OtA5088-OtA5092	OtA5093-OtA5097
Trpa1	chr1:14872647-14918862	OtA5088-OtA5092	OtA5093-OtA5097
Trpc1	chr9:95706628-95750358	OtA5088-OtA5092	OtA5093-OtA5097
Trpc2	chr7:102065712-102096416	OtA5088-OtA5092	OtA5093-OtA5097
Trpc3	chr3:36620481-36690167	OtA5088-OtA5092	OtA5093-OtA5097
Trpc4	chr3:54156056-54318471	OtA5088-OtA5092	OtA5093-OtA5097
Trpc5	chrX:144381670-144688180	OtA5088-OtA5092	OtA5093-OtA5097
Trpc6	chr9:8544141-8680741	OtA5088-OtA5092	OtA5093-OtA5097
Trpc7	chr13:56773107-56895588	OtA5088-OtA5092	OtA5093-OtA5097
Trpd52l3	chr19:30003789-30006020	OtA5088-OtA5092	OtA5093-OtA5097
Trpm1	chr7:64153834-64269759	OtA5088-OtA5092	OtA5093-OtA5097
Trpm2	chr10:77907721-77969872	OtA5088-OtA5092	OtA5093-OtA5097
Trpm3	chr19:22139116-22989884	OtA5088-OtA5092	OtA5093-OtA5097
Trpm5	chr7:143071528-143094642	OtA5088-OtA5092	OtA5093-OtA5097
Trpm8	chr1:88306711-88388851	OtA5088-OtA5092	OtA5093-OtA5097
Trps1	chr15:50654758-50890041	OtA5088-OtA5092	OtA5093-OtA5097
Trpv1	chr11:73238258-73260507	OtA5088-OtA5092	OtA5093-OtA5097
Trpv3	chr11:73267619-73297200	OtA5088-OtA5092	OtA5093-OtA5097
Trpv4	chr5:114622153-114658421	OtA5088-OtA5092	OtA5093-OtA5097
Trpv5	chr6:41652769-41680723	OtA5088-OtA5092	OtA5093-OtA5097
Try10	chr6:41354104-41357944	OtA5088-OtA5092	OtA5093-OtA5097
Try4	chr6:41302271-41305533	OtA5088-OtA5092	OtA5093-OtA5097
Try5	chr6:41311231-41314710	OtA5088-OtA5092	OtA5093-OtA5097
Tsga10	chr1:37754775-37865079	OtA5088-OtA5092	OtA5093-OtA5097
Tsga13	chr6:30896980-30915573	OtA5088-OtA5092	OtA5093-OtA5097
Tsga8	chrX:82948869-85205050	OtA5088-OtA5092	OtA5093-OtA5097

Tshb	chr3:102777397-102782714	OtA5088-OtA5092	OtA5093-OtA5097
Tshr	chr12:91400992-91540509	OtA5088-OtA5092	OtA5093-OtA5097
Tsix	chrX:103431516-103484957	OtA5088-OtA5092	OtA5093-OtA5097
Tsks	chr7:44943239-44958037	OtA5088-OtA5092	OtA5093-OtA5097
Tslp	chr18:32663641-32866420	OtA5088-OtA5092	OtA5093-OtA5097
Tsnaxip1	chr8:105827743-105844676	OtA5088-OtA5092	OtA5093-OtA5097
Tspan10	chr11:120442630-120447321	OtA5088-OtA5092	OtA5093-OtA5097
Tspan11	chr6:127887621-127953031	OtA5088-OtA5092	OtA5093-OtA5097
Tspan32	chr7:143005045-143019485	OtA5088-OtA5092	OtA5093-OtA5097
Tspy-ps	chrY:1055763-1058868	OtA5088-OtA5092	OtA5093-OtA5097
Tspyl3	chr2:153222369-153225441	OtA5088-OtA5092	OtA5093-OtA5097
Tspyl4	chr10:34297420-34301320	OtA5088-OtA5092	OtA5093-OtA5097
Tspyl5	chr15:33683874-33687883	OtA5088-OtA5092	OtA5093-OtA5097
Tssk1	chr16:17894202-17895653	OtA5088-OtA5092	OtA5093-OtA5097
Tssk2	chr16:17898636-17900024	OtA5088-OtA5092	OtA5093-OtA5097
Tssk3	chr4:129489007-129490770	OtA5088-OtA5092	OtA5093-OtA5097
Tssk4	chr14:55650183-55652539	OtA5088-OtA5092	OtA5093-OtA5097
Tssk5	chr15:76371957-76374938	OtA5088-OtA5092	OtA5093-OtA5097
Tssk6	chr8:69894181-69903518	OtA5088-OtA5092	OtA5093-OtA5097
Tstd1	chr1:171419032-171420352	OtA5088-OtA5092	OtA5093-OtA5097
Tsx	chrX:103414466-103424583	OtA5088-OtA5092	OtA5093-OtA5097
Ttbk1	chr17:46442447-46487675	OtA5088-OtA5092	OtA5093-OtA5097
Ttc12	chr9:49436960-49486225	OtA5088-OtA5092	OtA5093-OtA5097
Ttc16	chr2:32757233-32775383	OtA5088-OtA5092	OtA5093-OtA5097
Ttc18	chr14:20394189-20452225	OtA5088-OtA5092	OtA5093-OtA5097
Ttc21a	chr9:119937605-119967793	OtA5088-OtA5092	OtA5093-OtA5097
Ttc21b	chr2:66184328-66256617	OtA5088-OtA5092	OtA5093-OtA5097
Ttc23l	chr15:10503946-10558668	OtA5088-OtA5092	OtA5093-OtA5097
Ttc24	chr3:88069409-88078304	OtA5088-OtA5092	OtA5093-OtA5097
Ttc25	chr11:100545631-100572566	OtA5088-OtA5092	OtA5093-OtA5097
Ttc26	chr6:38381523-38427647	OtA5088-OtA5092	OtA5093-OtA5097
Ttc29	chr8:78213341-78394326	OtA5088-OtA5092	OtA5093-OtA5097
Ttc30a1	chr2:75979105-75981967	OtA5088-OtA5092	OtA5093-OtA5097
Ttc30a2	chr2:75976171-75978179	OtA5088-OtA5092	OtA5093-OtA5097
Ttc34	chr4:154856199-154867127	OtA5088-OtA5092	OtA5093-OtA5097
Ttc36	chr9:44799399-44802951	OtA5088-OtA5092	OtA5093-OtA5097
Ttc39d	chr17:80215913-80217936	OtA5088-OtA5092	OtA5093-OtA5097
Ttc9	chr12:81631368-81664941	OtA5088-OtA5092	OtA5093-OtA5097
Ttc9b	chr7:27653923-27656207	OtA5088-OtA5092	OtA5093-OtA5097
Ttk	chr9:83834688-83872390	OtA5088-OtA5092	OtA5093-OtA5097
Ttll10	chr4:156034836-156050817	OtA5088-OtA5092	OtA5093-OtA5097
Ttll11	chr2:35751239-35979624	OtA5088-OtA5092	OtA5093-OtA5097
Ttll13	chr7:80246375-80260821	OtA5088-OtA5092	OtA5093-OtA5097
Ttll2	chr17:7350903-7352696	OtA5088-OtA5092	OtA5093-OtA5097
Ttll6	chr11:96133785-96165452	OtA5088-OtA5092	OtA5093-OtA5097
Ttll7	chr3:146852366-146982750	OtA5088-OtA5092	OtA5093-OtA5097
Ttll8	chr15:88913897-88954418	OtA5088-OtA5092	OtA5093-OtA5097

Ttll9	chr2:152962484-153008482	OtA5088-OtA5092	OtA5093-OtA5097
Ttn	chr2:76703983-76982547	OtA5088-OtA5092	OtA5093-OtA5097
Ttpa	chr4:20008427-20030785	OtA5088-OtA5092	OtA5093-OtA5097
Ttyh1	chr7:4074124-4136926	OtA5088-OtA5092	OtA5093-OtA5097
Tub	chr7:109010879-109083324	OtA5088-OtA5092	OtA5093-OtA5097
Tuba3a	chr6:125278273-125286042	OtA5088-OtA5092	OtA5093-OtA5097
Tuba3b	chr6:145615962-145621477	OtA5088-OtA5092	OtA5093-OtA5097
Tubal3	chr13:3924694-3935277	OtA5088-OtA5092	OtA5093-OtA5097
Tubb2a-ps2	chr12:11882195-11882899	OtA5088-OtA5092	OtA5093-OtA5097
Tube1	chr10:39134022-39163794	OtA5088-OtA5092	OtA5093-OtA5097
Tubg2	chr11:101155883-101161787	OtA5088-OtA5092	OtA5093-OtA5097
Tulp2	chr7:45513705-45522603	OtA5088-OtA5092	OtA5093-OtA5097
Twist1	chr12:33957670-33959831	OtA5088-OtA5092	OtA5093-OtA5097
Txk	chr5:72695977-72752773	OtA5088-OtA5092	OtA5093-OtA5097
Txlnb	chr10:17796218-17845663	OtA5088-OtA5092	OtA5093-OtA5097
Txlng	chrX:162407199-163352564	OtA5088-OtA5092	OtA5093-OtA5097
Txndc2	chr17:65637504-65642204	OtA5088-OtA5092	OtA5093-OtA5097
Txndc3	chr13:19645078-19697760	OtA5088-OtA5092	OtA5093-OtA5097
Txndc6	chr9:99456242-99568899	OtA5088-OtA5092	OtA5093-OtA5097
Txndc8	chr4:57984028-58009124	OtA5088-OtA5092	OtA5093-OtA5097
Tyms	chr5:30058199-30073625	OtA5088-OtA5092	OtA5093-OtA5097
Tyms-ps	chr10:87966670-87968017	OtA5088-OtA5092	OtA5093-OtA5097
Tyr	chr7:87427404-87493411	OtA5088-OtA5092	OtA5093-OtA5097
Tyrp1	chr4:80834229-80851718	OtA5088-OtA5092	OtA5093-OtA5097
Tyw3	chr3:154576519-154623182	OtA5088-OtA5092	OtA5093-OtA5097
U05342,Zfp91	chr19:12763527-12796123	OtA5088-OtA5092	OtA5093-OtA5097
U90926	chr5:92209894-92215408	OtA5088-OtA5092	OtA5093-OtA5097
Ubash3a	chr17:31208065-31242403	OtA5088-OtA5092	OtA5093-OtA5097
Ubd	chr17:37193891-37196101	OtA5088-OtA5092	OtA5093-OtA5097
Ube1y1	chrY:818712-844224	OtA5088-OtA5092	OtA5093-OtA5097
Ube2cbp	chr9:86307233-86464916	OtA5088-OtA5092	OtA5093-OtA5097
Ube2dnl	chrX:114905011-114905941	OtA5088-OtA5092	OtA5093-OtA5097
Ube2t	chr1:134962576-134974137	OtA5088-OtA5092	OtA5093-OtA5097
Ube2u	chr4:100478866-100550145	OtA5088-OtA5092	OtA5093-OtA5097
Ube2v2	chr16:15550985-15594518	OtA5088-OtA5092	OtA5093-OtA5097
Ubl4b	chr3:107553697-107555073	OtA5088-OtA5092	OtA5093-OtA5097
Ubqln3	chr7:104140622-104143272	OtA5088-OtA5092	OtA5093-OtA5097
Ubqlnl	chr7:104148258-104150556	OtA5088-OtA5092	OtA5093-OtA5097
Ubtfl1	chr9:18404417-18411502	OtA5088-OtA5092	OtA5093-OtA5097
Ubxn10	chr4:138718525-138725251	OtA5088-OtA5092	OtA5093-OtA5097
Ubxn11	chr4:134102582-134126780	OtA5088-OtA5092	OtA5093-OtA5097
Ubxn2b	chr4:6191104-6219788	OtA5088-OtA5092	OtA5093-OtA5097
Uchl4	chr9:64185792-64253605	OtA5088-OtA5092	OtA5093-OtA5097
Ucma	chr2:4976121-4985748	OtA5088-OtA5092	OtA5093-OtA5097
Ucn	chr5:31137988-31138895	OtA5088-OtA5092	OtA5093-OtA5097
Ucn2	chr9:108986162-108987164	OtA5088-OtA5092	OtA5093-OtA5097
Ucn3	chr13:3940687-3945349	OtA5088-OtA5092	OtA5093-OtA5097

Ucp1	chr8:83290347-83298456	OtA5088-OtA5092	OtA5093-OtA5097
Ucp3	chr7:100472990-100486432	OtA5088-OtA5092	OtA5093-OtA5097
Uggt2	chr14:118984984-119099434	OtA5088-OtA5092	OtA5093-OtA5097
Ugt1a1	chr1:88055410-88220002	OtA5088-OtA5092	OtA5093-OtA5097
Ugt1a10	chr1:88055410-88220002	OtA5088-OtA5092	OtA5093-OtA5097
Ugt1a2	chr1:88055410-88220002	OtA5088-OtA5092	OtA5093-OtA5097
Ugt1a5	chr1:88055410-88220002	OtA5088-OtA5092	OtA5093-OtA5097
Ugt1a6a	chr1:88055410-88220002	OtA5088-OtA5092	OtA5093-OtA5097
Ugt1a6b	chr1:88055410-88220002	OtA5088-OtA5092	OtA5093-OtA5097
Ugt1a9	chr1:88055410-88220002	OtA5088-OtA5092	OtA5093-OtA5097
Ugt2a1	chr5:87459489-87490871	OtA5088-OtA5092	OtA5093-OtA5097
Ugt2a2	chr5:87459489-87490871	OtA5088-OtA5092	OtA5093-OtA5097
Ugt2a3	chr5:87324971-87337195	OtA5088-OtA5092	OtA5093-OtA5097
Ugt2b1	chr5:86916638-86926503	OtA5088-OtA5092	OtA5093-OtA5097
Ugt2b34	chr5:86889769-86906937	OtA5088-OtA5092	OtA5093-OtA5097
Ugt2b35	chr5:87000859-87013274	OtA5088-OtA5092	OtA5093-OtA5097
Ugt2b36	chr5:87065926-87092555	OtA5088-OtA5092	OtA5093-OtA5097
Ugt2b37	chr5:87240491-87254788	OtA5088-OtA5092	OtA5093-OtA5097
Ugt2b38	chr5:87409939-87424203	OtA5088-OtA5092	OtA5093-OtA5097
Ugt2b5	chr5:87124946-87140340	OtA5088-OtA5092	OtA5093-OtA5097
Ugt3a1	chr15:9279828-9315036	OtA5088-OtA5092	OtA5093-OtA5097
Ugt3a2	chr15:9335597-9370870	OtA5088-OtA5092	OtA5093-OtA5097
Ugt8a	chr3:125865342-125938550	OtA5088-OtA5092	OtA5093-OtA5097
Ulbp1	chr10:7444872-7473477	OtA5088-OtA5092	OtA5093-OtA5097
Ulk4	chr9:120964453-121277172	OtA5088-OtA5092	OtA5093-OtA5097
Umod	chr7:119462865-119479255	OtA5088-OtA5092	OtA5093-OtA5097
Umodl1	chr17:30954682-31010710	OtA5088-OtA5092	OtA5093-OtA5097
Unc13a	chr8:71626711-71671757	OtA5088-OtA5092	OtA5093-OtA5097
Unc13c	chr9:73479423-73933567	OtA5088-OtA5092	OtA5093-OtA5097
Unc45b	chr11:82911252-82964850	OtA5088-OtA5092	OtA5093-OtA5097
Unc5c	chr3:141465563-141834922	OtA5088-OtA5092	OtA5093-OtA5097
Unc5d	chr8:28646716-29219636	OtA5088-OtA5092	OtA5093-OtA5097
Unc79	chr12:102948858-103183997	OtA5088-OtA5092	OtA5093-OtA5097
Unc80	chr1:66468446-66699148	OtA5088-OtA5092	OtA5093-OtA5097
Unc93a	chr17:13108616-13131791	OtA5088-OtA5092	OtA5093-OtA5097
Uncx	chr5:139543897-139548178	OtA5088-OtA5092	OtA5093-OtA5097
Ung	chr5:114130434-114139321	OtA5088-OtA5092	OtA5093-OtA5097
Uox	chr3:146581372-146631483	OtA5088-OtA5092	OtA5093-OtA5097
Upb1	chr10:75407033-75440195	OtA5088-OtA5092	OtA5093-OtA5097
Upk2	chr9:44452714-44454767	OtA5088-OtA5092	OtA5093-OtA5097
Upk3a	chr15:85017140-85022560	OtA5088-OtA5092	OtA5093-OtA5097
Upp2	chr2:58755184-58790668	OtA5088-OtA5092	OtA5093-OtA5097
Uprt	chrX:104482781-104506262	OtA5088-OtA5092	OtA5093-OtA5097
Uroc1	chr6:90333288-90364548	OtA5088-OtA5092	OtA5093-OtA5097
Ush1c	chr7:46195350-46238490	OtA5088-OtA5092	OtA5093-OtA5097
Ush1g	chr11:115307162-115332303	OtA5088-OtA5092	OtA5093-OtA5097
Ush2a	chr1:188262837-188965039	OtA5088-OtA5092	OtA5093-OtA5097

Usp13	chr3:32817625-32935257	OtA5088-OtA5092	OtA5093-OtA5097
Usp17l5	chr7:103416695-103419174	OtA5088-OtA5092	OtA5093-OtA5097
Usp26	chrX:51753958-51801233	OtA5088-OtA5092	OtA5093-OtA5097
Usp29	chr7:6730740-6976662	OtA5088-OtA5092	OtA5093-OtA5097
Usp43	chr11:67854522-67922153	OtA5088-OtA5092	OtA5093-OtA5097
Usp44	chr10:93831554-93858087	OtA5088-OtA5092	OtA5093-OtA5097
Usp50	chr2:126761049-126783460	OtA5088-OtA5092	OtA5093-OtA5097
Usp51	chrX:153006468-153009459	OtA5088-OtA5092	OtA5093-OtA5097
Usp9y	chrY:1298960-1459782	OtA5088-OtA5092	OtA5093-OtA5097
Utf1	chr7:139943855-139945112	OtA5088-OtA5092	OtA5093-OtA5097
Uts2	chr4:150997096-151001810	OtA5088-OtA5092	OtA5093-OtA5097
Uts2d	chr16:27353321-27370239	OtA5088-OtA5092	OtA5093-OtA5097
Uts2r	chr11:121160270-121161973	OtA5088-OtA5092	OtA5093-OtA5097
V1ra8	chr6:90202816-90203656	OtA5088-OtA5092	OtA5093-OtA5097
V1rd18,V1rd19	chr7:24003090-24004247	OtA5088-OtA5092	OtA5093-OtA5097
V1rd19	chr7:24003090-24004247	OtA5088-OtA5092	OtA5093-OtA5097
V1rg10	chr5:3000100-3001111	OtA5088-OtA5092	OtA5093-OtA5097
Vamp1	chr6:125215580-125222306	OtA5088-OtA5092	OtA5093-OtA5097
Vamp7	chrX_GL456233_random:10737-39148	OtA5088-OtA5092	OtA5093-OtA5097
Vat1l	chr8:114205639-114374070	OtA5088-OtA5092	OtA5093-OtA5097
Vav1	chr17:57279099-57329236	OtA5088-OtA5092	OtA5093-OtA5097
Vax1	chr19:59166186-59170029	OtA5088-OtA5092	OtA5093-OtA5097
Vax2os1	chr6:83692805-83738304	OtA5088-OtA5092	OtA5093-OtA5097
Vax2os2	chr6:83692805-83738304	OtA5088-OtA5092	OtA5093-OtA5097
Vegfc	chr8:54077531-54186454	OtA5088-OtA5092	OtA5093-OtA5097
Veph1	chr3:66054825-66296819	OtA5088-OtA5092	OtA5093-OtA5097
Vgll2	chr10:52022685-52028471	OtA5088-OtA5092	OtA5093-OtA5097
Vip	chr10:5639217-5647614	OtA5088-OtA5092	OtA5093-OtA5097
Vipr1	chr9:121642715-121672954	OtA5088-OtA5092	OtA5093-OtA5097
Vipr2	chr12:116077725-116146261	OtA5088-OtA5092	OtA5093-OtA5097
Vit	chr17:78508062-78627409	OtA5088-OtA5092	OtA5093-OtA5097
Vmn1r-ps103	chr13:22441271-22442243	OtA5088-OtA5092	OtA5093-OtA5097
Vmn1r-ps79	chr7:22352262-22354555	OtA5088-OtA5092	OtA5093-OtA5097
Vmn1r1	chr1:182157177-182158098	OtA5088-OtA5092	OtA5093-OtA5097
Vmn1r10	chr6:57113424-57114360	OtA5088-OtA5092	OtA5093-OtA5097
Vmn1r100,Vmn1r148	chr7:22412230-22414668	OtA5088-OtA5092	OtA5093-OtA5097
Vmn1r101	chr7:22437305-22438229	OtA5088-OtA5092	OtA5093-OtA5097
Vmn1r103	chr7:20509616-20510534	OtA5088-OtA5092	OtA5093-OtA5097
Vmn1r104	chr7:22522966-22523890	OtA5088-OtA5092	OtA5093-OtA5097
Vmn1r11	chr6:57137352-57138252	OtA5088-OtA5092	OtA5093-OtA5097
Vmn1r111	chr7:20702295-20703216	OtA5088-OtA5092	OtA5093-OtA5097
Vmn1r112	chr7:20771184-20772078	OtA5088-OtA5092	OtA5093-OtA5097
Vmn1r113	chr7:20787284-20788208	OtA5088-OtA5092	OtA5093-OtA5097
Vmn1r114	chr7:20811220-20812186	OtA5088-OtA5092	OtA5093-OtA5097
Vmn1r115	chr7:20844097-20844985	OtA5088-OtA5092	OtA5093-OtA5097
Vmn1r116	chr7:20872255-20873179	OtA5088-OtA5092	OtA5093-OtA5097
Vmn1r117	chr7:20883197-20884121	OtA5088-OtA5092	OtA5093-OtA5097

Vmn1r118	chr7:22228563-22229484	OtA5088-OtA5092	OtA5093-OtA5097
Vmn1r119	chr7:21011531-21012455	OtA5088-OtA5092	OtA5093-OtA5097
Vmn1r12	chr6:57158919-57159843	OtA5088-OtA5092	OtA5093-OtA5097
Vmn1r120	chr7:21052866-21053784	OtA5088-OtA5092	OtA5093-OtA5097
Vmn1r121	chr7:21097568-21098513	OtA5088-OtA5092	OtA5093-OtA5097
Vmn1r122	chr7:21133210-21134128	OtA5088-OtA5092	OtA5093-OtA5097
Vmn1r123	chr7:21162184-21163108	OtA5088-OtA5092	OtA5093-OtA5097
Vmn1r124	chr7:21259693-21260617	OtA5088-OtA5092	OtA5093-OtA5097
Vmn1r125	chr7:21272178-21273102	OtA5088-OtA5092	OtA5093-OtA5097
Vmn1r126	chr7:21300501-21301467	OtA5088-OtA5092	OtA5093-OtA5097
Vmn1r127	chr7:21318943-21319861	OtA5088-OtA5092	OtA5093-OtA5097
Vmn1r128	chr7:21349372-21350296	OtA5088-OtA5092	OtA5093-OtA5097
Vmn1r129	chr7:21360367-21361291	OtA5088-OtA5092	OtA5093-OtA5097
Vmn1r13	chr6:57209857-57210760	OtA5088-OtA5092	OtA5093-OtA5097
Vmn1r132,Vmn1r139	chr7:22095519-22097921	OtA5088-OtA5092	OtA5093-OtA5097
Vmn1r135	chr7:23055539-23056463	OtA5088-OtA5092	OtA5093-OtA5097
Vmn1r137	chr7:22658017-22658941	OtA5088-OtA5092	OtA5093-OtA5097
Vmn1r139	chr7:22095519-22097921	OtA5088-OtA5092	OtA5093-OtA5097
Vmn1r14	chr6:57233438-57234350	OtA5088-OtA5092	OtA5093-OtA5097
Vmn1r142	chr7:20167459-20168377	OtA5088-OtA5092	OtA5093-OtA5097
Vmn1r143	chr7:20193709-20194633	OtA5088-OtA5092	OtA5093-OtA5097
Vmn1r148	chr7:22412230-22414668	OtA5088-OtA5092	OtA5093-OtA5097
Vmn1r15	chr6:57258148-57259048	OtA5088-OtA5092	OtA5093-OtA5097
Vmn1r151	chr7:22498760-22499678	OtA5088-OtA5092	OtA5093-OtA5097
Vmn1r155	chr7:21994586-21995510	OtA5088-OtA5092	OtA5093-OtA5097
Vmn1r157	chr7:22761696-22762590	OtA5088-OtA5092	OtA5093-OtA5097
Vmn1r158	chr7:22789858-22790782	OtA5088-OtA5092	OtA5093-OtA5097
Vmn1r159	chr7:22842687-22843605	OtA5088-OtA5092	OtA5093-OtA5097
Vmn1r16	chr6:57322723-57323635	OtA5088-OtA5092	OtA5093-OtA5097
Vmn1r160	chr7:22871223-22872147	OtA5088-OtA5092	OtA5093-OtA5097
Vmn1r165	chr7:23217331-23218255	OtA5088-OtA5092	OtA5093-OtA5097
Vmn1r167	chr7:23504641-23505589	OtA5088-OtA5092	OtA5093-OtA5097
Vmn1r168	chr7:23540719-23541649	OtA5088-OtA5092	OtA5093-OtA5097
Vmn1r169	chr7:23577184-23578099	OtA5088-OtA5092	OtA5093-OtA5097
Vmn1r17	chr6:57360466-57361378	OtA5088-OtA5092	OtA5093-OtA5097
Vmn1r170	chr7:23606174-23607089	OtA5088-OtA5092	OtA5093-OtA5097
Vmn1r171	chr7:23631987-23633568	OtA5088-OtA5092	OtA5093-OtA5097
Vmn1r172	chr7:23658315-23660663	OtA5088-OtA5092	OtA5093-OtA5097
Vmn1r173	chr7:23702341-23703283	OtA5088-OtA5092	OtA5093-OtA5097
Vmn1r174	chr7:23753910-23754852	OtA5088-OtA5092	OtA5093-OtA5097
Vmn1r175	chr7:23808285-23809200	OtA5088-OtA5092	OtA5093-OtA5097
Vmn1r176	chr7:23834811-23835726	OtA5088-OtA5092	OtA5093-OtA5097
Vmn1r177	chr7:23865519-23866449	OtA5088-OtA5092	OtA5093-OtA5097
Vmn1r178	chr7:23893528-23894443	OtA5088-OtA5092	OtA5093-OtA5097
Vmn1r179	chr7:23928385-23929357	OtA5088-OtA5092	OtA5093-OtA5097
Vmn1r18	chr6:57389667-57390567	OtA5088-OtA5092	OtA5093-OtA5097
Vmn1r180	chr7:23952386-23953356	OtA5088-OtA5092	OtA5093-OtA5097

Vmn1r181	chr7:23983960-23985048	OtA5088-OtA5092	OtA5093-OtA5097
Vmn1r183	chr7:24054773-24055691	OtA5088-OtA5092	OtA5093-OtA5097
Vmn1r184	chr7:26266830-26267775	OtA5088-OtA5092	OtA5093-OtA5097
Vmn1r185	chr7:26611118-26612078	OtA5088-OtA5092	OtA5093-OtA5097
Vmn1r186,Vmn1r62	chr7_GL456219_random:93810-95806	OtA5088-OtA5092	OtA5093-OtA5097
Vmn1r187,Vmn1r63	chr7_GL456219_random:148880-15197	OtA5088-OtA5092	OtA5093-OtA5097
Vmn1r188	chr13:22087877-22088804	OtA5088-OtA5092	OtA5093-OtA5097
Vmn1r189	chr13:22101726-22102665	OtA5088-OtA5092	OtA5093-OtA5097
Vmn1r19	chr6:57404463-57405390	OtA5088-OtA5092	OtA5093-OtA5097
Vmn1r191	chr13:22178685-22179582	OtA5088-OtA5092	OtA5093-OtA5097
Vmn1r192	chr13:22187145-22188048	OtA5088-OtA5092	OtA5093-OtA5097
Vmn1r193	chr13:22218860-22219820	OtA5088-OtA5092	OtA5093-OtA5097
Vmn1r194	chr13:22244214-22245105	OtA5088-OtA5092	OtA5093-OtA5097
Vmn1r195	chr13:22278316-22279404	OtA5088-OtA5092	OtA5093-OtA5097
Vmn1r196	chr13:22293192-22294098	OtA5088-OtA5092	OtA5093-OtA5097
Vmn1r197	chr13:22327910-22328807	OtA5088-OtA5092	OtA5093-OtA5097
Vmn1r198	chr13:22354345-22355248	OtA5088-OtA5092	OtA5093-OtA5097
Vmn1r199	chr13:22382537-22383641	OtA5088-OtA5092	OtA5093-OtA5097
Vmn1r2	chr4:3172082-3173003	OtA5088-OtA5092	OtA5093-OtA5097
Vmn1r20	chr6:57431690-57432602	OtA5088-OtA5092	OtA5093-OtA5097
Vmn1r200	chr13:22395028-22395967	OtA5088-OtA5092	OtA5093-OtA5097
Vmn1r201	chr13:22474617-22475520	OtA5088-OtA5092	OtA5093-OtA5097
Vmn1r202	chr13:22501336-22502245	OtA5088-OtA5092	OtA5093-OtA5097
Vmn1r203	chr13:22524050-22524986	OtA5088-OtA5092	OtA5093-OtA5097
Vmn1r204	chr13:22556200-22557109	OtA5088-OtA5092	OtA5093-OtA5097
Vmn1r205	chr13:22591979-22592930	OtA5088-OtA5092	OtA5093-OtA5097
Vmn1r206	chr13:22620096-22621035	OtA5088-OtA5092	OtA5093-OtA5097
Vmn1r207-ps	chr13:22725683-22726622	OtA5088-OtA5092	OtA5093-OtA5097
Vmn1r208	chr13:22772398-22773325	OtA5088-OtA5092	OtA5093-OtA5097
Vmn1r209	chr13:22805579-22806518	OtA5088-OtA5092	OtA5093-OtA5097
Vmn1r21	chr6:57843563-57844457	OtA5088-OtA5092	OtA5093-OtA5097
Vmn1r210	chr13:22827193-22828114	OtA5088-OtA5092	OtA5093-OtA5097
Vmn1r211	chr13:22851598-22852495	OtA5088-OtA5092	OtA5093-OtA5097
Vmn1r212	chr13:22883087-22884161	OtA5088-OtA5092	OtA5093-OtA5097
Vmn1r213	chr13:23011248-23012403	OtA5088-OtA5092	OtA5093-OtA5097
Vmn1r214	chr13:23034337-23035441	OtA5088-OtA5092	OtA5093-OtA5097
Vmn1r215	chr13:23075791-23076694	OtA5088-OtA5092	OtA5093-OtA5097
Vmn1r216	chr13:23099148-23100045	OtA5088-OtA5092	OtA5093-OtA5097
Vmn1r217	chr13:23113833-23114730	OtA5088-OtA5092	OtA5093-OtA5097
Vmn1r218	chr13:23136484-23137381	OtA5088-OtA5092	OtA5093-OtA5097
Vmn1r219	chr13:23162642-23163581	OtA5088-OtA5092	OtA5093-OtA5097
Vmn1r22	chr6:57900081-57900990	OtA5088-OtA5092	OtA5093-OtA5097
Vmn1r220	chr13:23183627-23184524	OtA5088-OtA5092	OtA5093-OtA5097
Vmn1r221	chr13:23217372-23218311	OtA5088-OtA5092	OtA5093-OtA5097
Vmn1r222	chr13:23232114-23233041	OtA5088-OtA5092	OtA5093-OtA5097
Vmn1r223	chr13:23249237-23250323	OtA5088-OtA5092	OtA5093-OtA5097
Vmn1r224	chr17:20419162-20420059	OtA5088-OtA5092	OtA5093-OtA5097

Vmn1r225	chr17:20502298-20503195	OtA5088-OtA5092	OtA5093-OtA5097
Vmn1r226	chr17:20687507-20688404	OtA5088-OtA5092	OtA5093-OtA5097
Vmn1r227	chr17:20735099-20736126	OtA5088-OtA5092	OtA5093-OtA5097
Vmn1r228	chr17:20776058-20777501	OtA5088-OtA5092	OtA5093-OtA5097
Vmn1r229	chr17:20814494-20815415	OtA5088-OtA5092	OtA5093-OtA5097
Vmn1r23	chr6:57925882-57926791	OtA5088-OtA5092	OtA5093-OtA5097
Vmn1r230	chr17:20846550-20847501	OtA5088-OtA5092	OtA5093-OtA5097
Vmn1r231	chr17:20889715-20890651	OtA5088-OtA5092	OtA5093-OtA5097
Vmn1r232	chr17:20913204-20914363	OtA5088-OtA5092	OtA5093-OtA5097
Vmn1r233	chr17:20993726-20994686	OtA5088-OtA5092	OtA5093-OtA5097
Vmn1r234	chr17:21228825-21229815	OtA5088-OtA5092	OtA5093-OtA5097
Vmn1r235	chr17:21260426-21262863	OtA5088-OtA5092	OtA5093-OtA5097
Vmn1r236	chr17:21286532-21287653	OtA5088-OtA5092	OtA5093-OtA5097
Vmn1r237	chr17:21314016-21314886	OtA5088-OtA5092	OtA5093-OtA5097
Vmn1r238	chr18:3122491-3123412	OtA5088-OtA5092	OtA5093-OtA5097
Vmn1r24	chr6:57955640-57956531	OtA5088-OtA5092	OtA5093-OtA5097
Vmn1r25	chr6:57978393-57979302	OtA5088-OtA5092	OtA5093-OtA5097
Vmn1r26	chr6:58008182-58009202	OtA5088-OtA5092	OtA5093-OtA5097
Vmn1r27	chr6:58215105-58216017	OtA5088-OtA5092	OtA5093-OtA5097
Vmn1r28	chr6:58265173-58266082	OtA5088-OtA5092	OtA5093-OtA5097
Vmn1r29	chr6:58307296-58308208	OtA5088-OtA5092	OtA5093-OtA5097
Vmn1r3	chr4:3184384-3185305	OtA5088-OtA5092	OtA5093-OtA5097
Vmn1r30	chr6:58434936-58435845	OtA5088-OtA5092	OtA5093-OtA5097
Vmn1r31	chr6:58471966-58472878	OtA5088-OtA5092	OtA5093-OtA5097
Vmn1r32	chr6:66552182-66559708	OtA5088-OtA5092	OtA5093-OtA5097
Vmn1r33	chr6:66611650-66612568	OtA5088-OtA5092	OtA5093-OtA5097
Vmn1r34	chr6:66636822-66637752	OtA5088-OtA5092	OtA5093-OtA5097
Vmn1r35	chr6:66678793-66679684	OtA5088-OtA5092	OtA5093-OtA5097
Vmn1r36	chr6:66715971-66716889	OtA5088-OtA5092	OtA5093-OtA5097
Vmn1r37	chr6:66731391-66732300	OtA5088-OtA5092	OtA5093-OtA5097
Vmn1r38	chr6:66776221-66777130	OtA5088-OtA5092	OtA5093-OtA5097
Vmn1r39	chr6:66804414-66805332	OtA5088-OtA5092	OtA5093-OtA5097
Vmn1r4	chr6:56956512-56957406	OtA5088-OtA5092	OtA5093-OtA5097
Vmn1r40	chr6:89714202-89715135	OtA5088-OtA5092	OtA5093-OtA5097
Vmn1r41	chr6:89740887-89747414	OtA5088-OtA5092	OtA5093-OtA5097
Vmn1r42	chr6:89844517-89845615	OtA5088-OtA5092	OtA5093-OtA5097
Vmn1r43	chr6:89869460-89870529	OtA5088-OtA5092	OtA5093-OtA5097
Vmn1r44	chr6:89893273-89894206	OtA5088-OtA5092	OtA5093-OtA5097
Vmn1r45	chr6:89931649-89940507	OtA5088-OtA5092	OtA5093-OtA5097
Vmn1r46	chr6:89976170-89977100	OtA5088-OtA5092	OtA5093-OtA5097
Vmn1r47	chr6:90021887-90022820	OtA5088-OtA5092	OtA5093-OtA5097
Vmn1r48	chr6:90035932-90036841	OtA5088-OtA5092	OtA5093-OtA5097
Vmn1r49	chr6:90072085-90073018	OtA5088-OtA5092	OtA5093-OtA5097
Vmn1r5	chr6:56985341-56986289	OtA5088-OtA5092	OtA5093-OtA5097
Vmn1r50	chr6:90107274-90108207	OtA5088-OtA5092	OtA5093-OtA5097
Vmn1r51	chr6:90122642-90130645	OtA5088-OtA5092	OtA5093-OtA5097
Vmn1r52	chr6:90178715-90179645	OtA5088-OtA5092	OtA5093-OtA5097

Vmn1r53	chr6:90223316-90224438	OtA5088-OtA5092	OtA5093-OtA5097
Vmn1r54	chr6:90269105-90270053	OtA5088-OtA5092	OtA5093-OtA5097
Vmn1r55	chr7:5146489-5147422	OtA5088-OtA5092	OtA5093-OtA5097
Vmn1r56	chr7:5194915-5196747	OtA5088-OtA5092	OtA5093-OtA5097
Vmn1r57	chr7:5220477-5221410	OtA5088-OtA5092	OtA5093-OtA5097
Vmn1r58	chr7:5408889-5413145	OtA5088-OtA5092	OtA5093-OtA5097
Vmn1r59	chr7:5453826-5454759	OtA5088-OtA5092	OtA5093-OtA5097
Vmn1r6	chr6:57002354-57003266	OtA5088-OtA5092	OtA5093-OtA5097
Vmn1r60	chr7:5544196-5545099	OtA5088-OtA5092	OtA5093-OtA5097
Vmn1r61	chr7:5610410-5611313	OtA5088-OtA5092	OtA5093-OtA5097
Vmn1r62	chr7_GL456219_random:93810-95806	OtA5088-OtA5092	OtA5093-OtA5097
Vmn1r63	chr7_GL456219_random:148880-15197	OtA5088-OtA5092	OtA5093-OtA5097
Vmn1r64	chr7:5883579-5884542	OtA5088-OtA5092	OtA5093-OtA5097
Vmn1r65	chr7:6007749-6011010	OtA5088-OtA5092	OtA5093-OtA5097
Vmn1r66	chr7:10273827-10275351	OtA5088-OtA5092	OtA5093-OtA5097
Vmn1r67	chr7:10446788-10447787	OtA5088-OtA5092	OtA5093-OtA5097
Vmn1r68	chr7:10527209-10528169	OtA5088-OtA5092	OtA5093-OtA5097
Vmn1r69	chr7:10579755-10581487	OtA5088-OtA5092	OtA5093-OtA5097
Vmn1r7	chr6:57024337-57025273	OtA5088-OtA5092	OtA5093-OtA5097
Vmn1r70	chr7:10633586-10634483	OtA5088-OtA5092	OtA5093-OtA5097
Vmn1r71	chr7:10747501-10749538	OtA5088-OtA5092	OtA5093-OtA5097
Vmn1r72	chr7:11669598-11670519	OtA5088-OtA5092	OtA5093-OtA5097
Vmn1r73	chr7:11756256-11757168	OtA5088-OtA5092	OtA5093-OtA5097
Vmn1r74	chr7:11846774-11847689	OtA5088-OtA5092	OtA5093-OtA5097
Vmn1r75	chr7:11880342-11881260	OtA5088-OtA5092	OtA5093-OtA5097
Vmn1r76	chr7:11930310-11931285	OtA5088-OtA5092	OtA5093-OtA5097
Vmn1r77	chr7:12041298-12042219	OtA5088-OtA5092	OtA5093-OtA5097
Vmn1r78	chr7:12152463-12153405	OtA5088-OtA5092	OtA5093-OtA5097
Vmn1r79	chr7:12176192-12177113	OtA5088-OtA5092	OtA5093-OtA5097
Vmn1r8	chr6:57035912-57037125	OtA5088-OtA5092	OtA5093-OtA5097
Vmn1r80	chr7:12192964-12193891	OtA5088-OtA5092	OtA5093-OtA5097
Vmn1r81	chr7:12259758-12260679	OtA5088-OtA5092	OtA5093-OtA5097
Vmn1r82	chr7:12304804-12305719	OtA5088-OtA5092	OtA5093-OtA5097
Vmn1r83	chr7:12321207-12322128	OtA5088-OtA5092	OtA5093-OtA5097
Vmn1r84	chr7:12361807-12362764	OtA5088-OtA5092	OtA5093-OtA5097
Vmn1r85	chr7:13084288-13085215	OtA5088-OtA5092	OtA5093-OtA5097
Vmn1r86	chr7:13101996-13102947	OtA5088-OtA5092	OtA5093-OtA5097
Vmn1r87	chr7:13131470-13132358	OtA5088-OtA5092	OtA5093-OtA5097
Vmn1r88	chr7:13177718-13178669	OtA5088-OtA5092	OtA5093-OtA5097
Vmn1r89	chr7:13219338-13220289	OtA5088-OtA5092	OtA5093-OtA5097
Vmn1r9	chr6:57070894-57071945	OtA5088-OtA5092	OtA5093-OtA5097
Vmn1r90	chr7:14530653-14623066	OtA5088-OtA5092	OtA5093-OtA5097
Vmn1r91	chr7:20101157-20102081	OtA5088-OtA5092	OtA5093-OtA5097
Vmn1r94	chr7:22163117-22164035	OtA5088-OtA5092	OtA5093-OtA5097
Vmn1r95	chr7:22189376-22190300	OtA5088-OtA5092	OtA5093-OtA5097
Vmn2r-ps11	chr3:64632862-64844743	OtA5088-OtA5092	OtA5093-OtA5097
Vmn2r-ps129	chr17:23004970-23006659	OtA5088-OtA5092	OtA5093-OtA5097

Vmn2r-ps159	chr4:156331102-156339499	OtA5088-OtA5092	OtA5093-OtA5097
Vmn2r-ps54	chr7:41668013-41727193	OtA5088-OtA5092	OtA5093-OtA5097
Vmn2r-ps60	chr7:42430104-42430369	OtA5088-OtA5092	OtA5093-OtA5097
Vmn2r1	chr3:64081641-64105458	OtA5088-OtA5092	OtA5093-OtA5097
Vmn2r10	chr5:108995538-109006436	OtA5088-OtA5092	OtA5093-OtA5097
Vmn2r100	chr17:19504810-19532060	OtA5088-OtA5092	OtA5093-OtA5097
Vmn2r101	chr17:19577230-19612317	OtA5088-OtA5092	OtA5093-OtA5097
Vmn2r102	chr17:19660398-19694748	OtA5088-OtA5092	OtA5093-OtA5097
Vmn2r103	chr17:19773362-19812536	OtA5088-OtA5092	OtA5093-OtA5097
Vmn2r104	chr17:20029424-20048205	OtA5088-OtA5092	OtA5093-OtA5097
Vmn2r105	chr17:20208229-20234872	OtA5088-OtA5092	OtA5093-OtA5097
Vmn2r106	chr17:20267546-20285430	OtA5088-OtA5092	OtA5093-OtA5097
Vmn2r107	chr17:20345424-20375772	OtA5088-OtA5092	OtA5093-OtA5097
Vmn2r108	chr17:20462372-20481236	OtA5088-OtA5092	OtA5093-OtA5097
Vmn2r109	chr17:20540516-20564756	OtA5088-OtA5092	OtA5093-OtA5097
Vmn2r11	chr5:109046872-109059452	OtA5088-OtA5092	OtA5093-OtA5097
Vmn2r110	chr17:20573828-20596259	OtA5088-OtA5092	OtA5093-OtA5097
Vmn2r111	chr17:22547940-22573273	OtA5088-OtA5092	OtA5093-OtA5097
Vmn2r112	chr17:22601147-22619133	OtA5088-OtA5092	OtA5093-OtA5097
Vmn2r113	chr17:22943183-22958814	OtA5088-OtA5092	OtA5093-OtA5097
Vmn2r114	chr17:23290933-23312313	OtA5088-OtA5092	OtA5093-OtA5097
Vmn2r115	chr17:23343976-23360128	OtA5088-OtA5092	OtA5093-OtA5097
Vmn2r116	chr17:23384802-23401864	OtA5088-OtA5092	OtA5093-OtA5097
Vmn2r117	chr17:23459674-23479597	OtA5088-OtA5092	OtA5093-OtA5097
Vmn2r118	chr17:55592340-55624672	OtA5088-OtA5092	OtA5093-OtA5097
Vmn2r12	chr5:109085848-109097864	OtA5088-OtA5092	OtA5093-OtA5097
Vmn2r120	chr17:57508782-57545314	OtA5088-OtA5092	OtA5093-OtA5097
Vmn2r121	chrX:124127338-124135910	OtA5088-OtA5092	OtA5093-OtA5097
Vmn2r122	chr4_JH584292_random:3535-11935	OtA5088-OtA5092	OtA5093-OtA5097
Vmn2r123	chr4:156331102-156339499	OtA5088-OtA5092	OtA5093-OtA5097
Vmn2r13	chr5:109156067-109192107	OtA5088-OtA5092	OtA5093-OtA5097
Vmn2r14	chr5:109215501-109224622	OtA5088-OtA5092	OtA5093-OtA5097
Vmn2r15	chr5:109286268-109297556	OtA5088-OtA5092	OtA5093-OtA5097
Vmn2r16	chr5:109330380-109364481	OtA5088-OtA5092	OtA5093-OtA5097
Vmn2r17	chr5:109420012-109453387	OtA5088-OtA5092	OtA5093-OtA5097
Vmn2r18	chr5:151561660-151586924	OtA5088-OtA5092	OtA5093-OtA5097
Vmn2r19	chr6:123308332-123336537	OtA5088-OtA5092	OtA5093-OtA5097
Vmn2r2	chr3:64116431-64137480	OtA5088-OtA5092	OtA5093-OtA5097
Vmn2r20	chr6:123385261-123418061	OtA5088-OtA5092	OtA5093-OtA5097
Vmn2r21	chr6:123492507-123533406	OtA5088-OtA5092	OtA5093-OtA5097
Vmn2r22	chr6:123609757-123650635	OtA5088-OtA5092	OtA5093-OtA5097
Vmn2r23	chr6:123702820-123742239	OtA5088-OtA5092	OtA5093-OtA5097
Vmn2r24	chr6:123778970-123816280	OtA5088-OtA5092	OtA5093-OtA5097
Vmn2r25	chr6:123822813-123853190	OtA5088-OtA5092	OtA5093-OtA5097
Vmn2r26	chr6:124024757-124062035	OtA5088-OtA5092	OtA5093-OtA5097
Vmn2r27	chr6:124191595-124231784	OtA5088-OtA5092	OtA5093-OtA5097
Vmn2r28	chr7:5480455-5493851	OtA5088-OtA5092	OtA5093-OtA5097

Vmn2r29	chr7:7212995-7278289	OtA5088-OtA5092	OtA5093-OtA5097
Vmn2r3	chr3:64258960-64287417	OtA5088-OtA5092	OtA5093-OtA5097
Vmn2r30	chr7:7312273-7337493	OtA5088-OtA5092	OtA5093-OtA5097
Vmn2r31	chr7:7383984-7399627	OtA5088-OtA5092	OtA5093-OtA5097
Vmn2r32	chr7:7463968-7479973	OtA5088-OtA5092	OtA5093-OtA5097
Vmn2r33	chr7:7550966-7566786	OtA5088-OtA5092	OtA5093-OtA5097
Vmn2r34	chr7:7671828-7689398	OtA5088-OtA5092	OtA5093-OtA5097
Vmn2r35	chr7:7786150-7819867	OtA5088-OtA5092	OtA5093-OtA5097
Vmn2r36	chr7:7876652-7902462	OtA5088-OtA5092	OtA5093-OtA5097
Vmn2r37	chr7:9205548-9223653	OtA5088-OtA5092	OtA5093-OtA5097
Vmn2r38	chr7:9074795-9097765	OtA5088-OtA5092	OtA5093-OtA5097
Vmn2r39	chr7:9014749-9030682	OtA5088-OtA5092	OtA5093-OtA5097
Vmn2r4	chr3:64388620-64410057	OtA5088-OtA5092	OtA5093-OtA5097
Vmn2r40	chr7:8907733-8931402	OtA5088-OtA5092	OtA5093-OtA5097
Vmn2r41	chr7:8137904-8161551	OtA5088-OtA5092	OtA5093-OtA5097
Vmn2r42	chr7:8183268-8200320	OtA5088-OtA5092	OtA5093-OtA5097
Vmn2r43	chr7:8244350-8260599	OtA5088-OtA5092	OtA5093-OtA5097
Vmn2r44	chr7:8367459-8383238	OtA5088-OtA5092	OtA5093-OtA5097
Vmn2r45	chr7:8471468-8488959	OtA5088-OtA5092	OtA5093-OtA5097
Vmn2r46	chr7:9754635-9770571	OtA5088-OtA5092	OtA5093-OtA5097
Vmn2r47	chr7:9816781-9841325	OtA5088-OtA5092	OtA5093-OtA5097
Vmn2r48	chr7:9927623-9953585	OtA5088-OtA5092	OtA5093-OtA5097
Vmn2r49	chr7:9976244-9992139	OtA5088-OtA5092	OtA5093-OtA5097
Vmn2r5	chr3:64490820-64507685	OtA5088-OtA5092	OtA5093-OtA5097
Vmn2r50	chr7:10037234-10053178	OtA5088-OtA5092	OtA5093-OtA5097
Vmn2r51	chr7:10087197-10105659	OtA5088-OtA5092	OtA5093-OtA5097
Vmn2r52	chr7:10158651-10176286	OtA5088-OtA5092	OtA5093-OtA5097
Vmn2r53	chr7:12581469-12606544	OtA5088-OtA5092	OtA5093-OtA5097
Vmn2r54	chr7:12615232-12636134	OtA5088-OtA5092	OtA5093-OtA5097
Vmn2r55	chr7:12651705-12684991	OtA5088-OtA5092	OtA5093-OtA5097
Vmn2r56	chr7:12693997-12733105	OtA5088-OtA5092	OtA5093-OtA5097
Vmn2r57	chr7:41399731-41448641	OtA5088-OtA5092	OtA5093-OtA5097
Vmn2r58	chr7:41836880-41872670	OtA5088-OtA5092	OtA5093-OtA5097
Vmn2r59	chr7:42011791-42058981	OtA5088-OtA5092	OtA5093-OtA5097
Vmn2r6	chr3:64537560-64559818	OtA5088-OtA5092	OtA5093-OtA5097
Vmn2r60	chr7:42116470-42195776	OtA5088-OtA5092	OtA5093-OtA5097
Vmn2r61	chr7:42260052-42300755	OtA5088-OtA5092	OtA5093-OtA5097
Vmn2r62	chr7:42764437-42793496	OtA5088-OtA5092	OtA5093-OtA5097
Vmn2r63	chr7:42903250-42933789	OtA5088-OtA5092	OtA5093-OtA5097
Vmn2r65	chr7:84940168-84964009	OtA5088-OtA5092	OtA5093-OtA5097
Vmn2r66	chr7:84994644-85012020	OtA5088-OtA5092	OtA5093-OtA5097
Vmn2r67	chr7:85136239-85155902	OtA5088-OtA5092	OtA5093-OtA5097
Vmn2r68-ps	chr7:85221517-85237704	OtA5088-OtA5092	OtA5093-OtA5097
Vmn2r69	chr7:85406375-85415676	OtA5088-OtA5092	OtA5093-OtA5097
Vmn2r7	chr3:64632862-64844743	OtA5088-OtA5092	OtA5093-OtA5097
Vmn2r70	chr7:85558702-85569088	OtA5088-OtA5092	OtA5093-OtA5097
Vmn2r71	chr7:85615461-85624547	OtA5088-OtA5092	OtA5093-OtA5097

Vmn2r72-ps	chr7:85737783-85754981	OtA5088-OtA5092	OtA5093-OtA5097
Vmn2r73	chr7:85857546-85875938	OtA5088-OtA5092	OtA5093-OtA5097
Vmn2r74	chr7:85951866-85961482	OtA5088-OtA5092	OtA5093-OtA5097
Vmn2r75	chr7:86148041-86171724	OtA5088-OtA5092	OtA5093-OtA5097
Vmn2r76	chr7:86225205-86246201	OtA5088-OtA5092	OtA5093-OtA5097
Vmn2r77	chr7:86795140-86812032	OtA5088-OtA5092	OtA5093-OtA5097
Vmn2r78	chr7:86915348-86955177	OtA5088-OtA5092	OtA5093-OtA5097
Vmn2r79	chr7:86996464-87037968	OtA5088-OtA5092	OtA5093-OtA5097
Vmn2r8	chr5:108797192-108808754	OtA5088-OtA5092	OtA5093-OtA5097
Vmn2r80	chr10:79148815-79194933	OtA5088-OtA5092	OtA5093-OtA5097
Vmn2r81	chr10:79247776-79294535	OtA5088-OtA5092	OtA5093-OtA5097
Vmn2r82	chr10:79356590-79396766	OtA5088-OtA5092	OtA5093-OtA5097
Vmn2r83	chr10:79468957-79492154	OtA5088-OtA5092	OtA5093-OtA5097
Vmn2r84	chr10:130385799-130394241	OtA5088-OtA5092	OtA5093-OtA5097
Vmn2r85	chr10:130418260-130429612	OtA5088-OtA5092	OtA5093-OtA5097
Vmn2r86	chr10:130446198-130455894	OtA5088-OtA5092	OtA5093-OtA5097
Vmn2r87	chr10:130471820-130497379	OtA5088-OtA5092	OtA5093-OtA5097
Vmn2r88	chr14:51411000-51418882	OtA5088-OtA5092	OtA5093-OtA5097
Vmn2r89	chr14:51451961-51461293	OtA5088-OtA5092	OtA5093-OtA5097
Vmn2r9	chr5:108842946-108852510	OtA5088-OtA5092	OtA5093-OtA5097
Vmn2r90	chr17:17703940-17734167	OtA5088-OtA5092	OtA5093-OtA5097
Vmn2r91	chr17:18085056-18136643	OtA5088-OtA5092	OtA5093-OtA5097
Vmn2r92	chr17:18151929-18185178	OtA5088-OtA5092	OtA5093-OtA5097
Vmn2r93	chr17:18298280-18326441	OtA5088-OtA5092	OtA5093-OtA5097
Vmn2r94	chr17:18243569-18277566	OtA5088-OtA5092	OtA5093-OtA5097
Vmn2r95	chr17:18424103-18452324	OtA5088-OtA5092	OtA5093-OtA5097
Vmn2r96	chr17:18581726-18598157	OtA5088-OtA5092	OtA5093-OtA5097
Vmn2r97	chr17:18914321-18948071	OtA5088-OtA5092	OtA5093-OtA5097
Vmn2r98	chr17:19053492-19081311	OtA5088-OtA5092	OtA5093-OtA5097
Vmn2r99	chr17:19362134-19394590	OtA5088-OtA5092	OtA5093-OtA5097
Vmo1	chr11:70513515-70514616	OtA5088-OtA5092	OtA5093-OtA5097
Vnn3	chr10:23851461-23869843	OtA5088-OtA5092	OtA5093-OtA5097
Vpreb1	chr16:16868400-16869255	OtA5088-OtA5092	OtA5093-OtA5097
Vpreb2	chr16:17980564-17981080	OtA5088-OtA5092	OtA5093-OtA5097
Vpreb3	chr10:75948311-75949646	OtA5088-OtA5092	OtA5093-OtA5097
Vps37d	chr5:135072899-135078266	OtA5088-OtA5092	OtA5093-OtA5097
Vrtn	chr12:84641018-84651455	OtA5088-OtA5092	OtA5093-OtA5097
Vsig1	chrX:140907607-140939472	OtA5088-OtA5092	OtA5093-OtA5097
Vsig4	chrX:96247202-96293438	OtA5088-OtA5092	OtA5093-OtA5097
Vsig8	chr1:172555937-172563717	OtA5088-OtA5092	OtA5093-OtA5097
Vsnl1	chr12:11325244-11436649	OtA5088-OtA5092	OtA5093-OtA5097
Vstm2a	chr11:16257723-16284551	OtA5088-OtA5092	OtA5093-OtA5097
Vstm2b	chr7:40899277-40929968	OtA5088-OtA5092	OtA5093-OtA5097
Vstm2l	chr2:157914652-157944719	OtA5088-OtA5092	OtA5093-OtA5097
Vsx1	chr2:150680701-150689137	OtA5088-OtA5092	OtA5093-OtA5097
Vsx2	chr12:84569851-84595457	OtA5088-OtA5092	OtA5093-OtA5097
Vwa2	chr19:56874415-56912078	OtA5088-OtA5092	OtA5093-OtA5097

Vwa3a	chr7:120739556-120805540	OtA5088-OtA5092	OtA5093-OtA5097
Vwa5b1	chr4:138568968-138623992	OtA5088-OtA5092	OtA5093-OtA5097
Vwa5b2	chr16:20589581-20605377	OtA5088-OtA5092	OtA5093-OtA5097
Vwc2	chr11:11114015-11263526	OtA5088-OtA5092	OtA5093-OtA5097
Vwc2l	chr1:70725714-70885397	OtA5088-OtA5092	OtA5093-OtA5097
Vwce	chr19:10634232-10665210	OtA5088-OtA5092	OtA5093-OtA5097
Vwde	chr6:13185610-13224965	OtA5088-OtA5092	OtA5093-OtA5097
Wap	chr11:6635482-6638649	OtA5088-OtA5092	OtA5093-OtA5097
Wasf3	chr5:146385005-146471125	OtA5088-OtA5092	OtA5093-OtA5097
Wbp2nl	chr15:82298983-82314558	OtA5088-OtA5092	OtA5093-OtA5097
Wbscr17	chr5:130874950-131307522	OtA5088-OtA5092	OtA5093-OtA5097
Wbscr25	chr5:134986213-135001350	OtA5088-OtA5092	OtA5093-OtA5097
Wbscr28	chr5:134901593-134906733	OtA5088-OtA5092	OtA5093-OtA5097
Wdfy4	chr14:32959546-33185066	OtA5088-OtA5092	OtA5093-OtA5097
Wdhd1	chr14:47240943-47276857	OtA5088-OtA5092	OtA5093-OtA5097
Wdpcp	chr11:21572280-21898686	OtA5088-OtA5092	OtA5093-OtA5097
Wdr16	chr11:67924805-67965642	OtA5088-OtA5092	OtA5093-OtA5097
Wdr17	chr8:54629615-54724368	OtA5088-OtA5092	OtA5093-OtA5097
Wdr20b	chr12:65225516-65228454	OtA5088-OtA5092	OtA5093-OtA5097
Wdr27	chr17:14818671-14943124	OtA5088-OtA5092	OtA5093-OtA5097
Wdr31	chr4:62453733-62470872	OtA5088-OtA5092	OtA5093-OtA5097
Wdr38	chr2:38998308-39005131	OtA5088-OtA5092	OtA5093-OtA5097
Wdr44	chrX:23693116-23806001	OtA5088-OtA5092	OtA5093-OtA5097
Wdr52	chr16:44394798-44482428	OtA5088-OtA5092	OtA5093-OtA5097
Wdr54	chr6:83152709-83156379	OtA5088-OtA5092	OtA5093-OtA5097
Wdr63	chr3:146040525-146108036	OtA5088-OtA5092	OtA5093-OtA5097
Wdr64	chr1:175698592-175815733	OtA5088-OtA5092	OtA5093-OtA5097
Wdr65	chr4:118554695-118620405	OtA5088-OtA5092	OtA5093-OtA5097
Wdr67	chr15:57912198-57970068	OtA5088-OtA5092	OtA5093-OtA5097
Wdr69	chr1:83159761-83210572	OtA5088-OtA5092	OtA5093-OtA5097
Wdr72	chr9:74110333-74283203	OtA5088-OtA5092	OtA5093-OtA5097
Wdr76	chr2:121506722-121544859	OtA5088-OtA5092	OtA5093-OtA5097
Wdr78	chr4:103038064-103114299	OtA5088-OtA5092	OtA5093-OtA5097
Wdr93	chr7:79743162-79785950	OtA5088-OtA5092	OtA5093-OtA5097
Wdr95	chr5:149489055-149611894	OtA5088-OtA5092	OtA5093-OtA5097
Wdr96	chr19:47736856-47837361	OtA5088-OtA5092	OtA5093-OtA5097
Wee2	chr6:40442862-40466815	OtA5088-OtA5092	OtA5093-OtA5097
Wfdc10	chr2:164656045-164657368	OtA5088-OtA5092	OtA5093-OtA5097
Wfdc11	chr2:164662893-164674087	OtA5088-OtA5092	OtA5093-OtA5097
Wfdc12	chr2:164189230-164190558	OtA5088-OtA5092	OtA5093-OtA5097
Wfdc13	chr2:164685106-164687706	OtA5088-OtA5092	OtA5093-OtA5097
Wfdc15a	chr2:164198871-164200117	OtA5088-OtA5092	OtA5093-OtA5097
Wfdc15b	chr2:164214453-164221663	OtA5088-OtA5092	OtA5093-OtA5097
Wfdc16	chr2:164634707-164638802	OtA5088-OtA5092	OtA5093-OtA5097
Wfdc3	chr2:164731225-164743267	OtA5088-OtA5092	OtA5093-OtA5097
Wfdc5	chr2:164176324-164182742	OtA5088-OtA5092	OtA5093-OtA5097
Wfdc6a	chr2:164579518-164585447	OtA5088-OtA5092	OtA5093-OtA5097

Wfdc6b	chr2:164613699-164618213	OtA5088-OtA5092	OtA5093-OtA5097
Wfdc8	chr2:164597212-164613626	OtA5088-OtA5092	OtA5093-OtA5097
Wfdc9	chr2:164649624-164654966	OtA5088-OtA5092	OtA5093-OtA5097
Wfikkn1	chr17:25877627-25880858	OtA5088-OtA5092	OtA5093-OtA5097
Wfikkn2	chr11:94235951-94242579	OtA5088-OtA5092	OtA5093-OtA5097
Wif1	chr10:121034003-121100642	OtA5088-OtA5092	OtA5093-OtA5097
Wisp2	chr2:163820833-163833147	OtA5088-OtA5092	OtA5093-OtA5097
Wisp3	chr10:39134022-39163794	OtA5088-OtA5092	OtA5093-OtA5097
Wnt1	chr15:98789856-98793830	OtA5088-OtA5092	OtA5093-OtA5097
Wnt10a	chr1:74792018-74804175	OtA5088-OtA5092	OtA5093-OtA5097
Wnt11	chr7:98838844-98854747	OtA5088-OtA5092	OtA5093-OtA5097
Wnt16	chr6:22288226-22298522	OtA5088-OtA5092	OtA5093-OtA5097
Wnt2b	chr3:104944804-104961709	OtA5088-OtA5092	OtA5093-OtA5097
Wnt3	chr11:103774174-103818021	OtA5088-OtA5092	OtA5093-OtA5097
Wnt3a	chr11:59248041-59290751	OtA5088-OtA5092	OtA5093-OtA5097
Wnt7a	chr6:91363982-91411369	OtA5088-OtA5092	OtA5093-OtA5097
Wnt8a	chr18:34542327-34548061	OtA5088-OtA5092	OtA5093-OtA5097
Wnt8b	chr19:44493471-44514273	OtA5088-OtA5092	OtA5093-OtA5097
Xcl1	chr1:164931647-164935510	OtA5088-OtA5092	OtA5093-OtA5097
Xcr1	chr9:123852314-123862029	OtA5088-OtA5092	OtA5093-OtA5097
Xirp1	chr9:120013754-120023598	OtA5088-OtA5092	OtA5093-OtA5097
Xirp2	chr2:67446001-67526606	OtA5088-OtA5092	OtA5093-OtA5097
Xkr4	chr1:3214481-3671498	OtA5088-OtA5092	OtA5093-OtA5097
Xkr5	chr8:18932728-18950973	OtA5088-OtA5092	OtA5093-OtA5097
Xkr6	chr14:63606529-63820410	OtA5088-OtA5092	OtA5093-OtA5097
Xkr7	chr2:153031851-153055775	OtA5088-OtA5092	OtA5093-OtA5097
Xkr8	chr4:132724903-132732546	OtA5088-OtA5092	OtA5093-OtA5097
Xkr9	chr1:13668770-13701723	OtA5088-OtA5092	OtA5093-OtA5097
Xkrx	chrX:134149044-134161928	OtA5088-OtA5092	OtA5093-OtA5097
Xlr	chrX:53783747-53797696	OtA5088-OtA5092	OtA5093-OtA5097
Xlr3c	chrX:73254539-73265390	OtA5088-OtA5092	OtA5093-OtA5097
Xlr5a	chrX:73107634-73129260	OtA5088-OtA5092	OtA5093-OtA5097
Xlr5b	chrX:73137231-73158399	OtA5088-OtA5092	OtA5093-OtA5097
Xlr5c	chrX:73285196-73290515	OtA5088-OtA5092	OtA5093-OtA5097
Xpnpep2	chrX:48108724-48136981	OtA5088-OtA5092	OtA5093-OtA5097
Xrra1	chr7:99859217-99917824	OtA5088-OtA5092	OtA5093-OtA5097
Ybx2	chr11:69935898-69941599	OtA5088-OtA5092	OtA5093-OtA5097
Yipf7	chr5:69516669-69542647	OtA5088-OtA5092	OtA5093-OtA5097
Ypel4	chr2:84734203-84737877	OtA5088-OtA5092	OtA5093-OtA5097
Ysk4	chr1:127815270-127840290	OtA5088-OtA5092	OtA5093-OtA5097
Ythdc2	chr18:44828664-44889720	OtA5088-OtA5092	OtA5093-OtA5097
Yy2	chrX:157547821-157598715	OtA5088-OtA5092	OtA5093-OtA5097
Zan	chr5:137378636-137477064	OtA5088-OtA5092	OtA5093-OtA5097
Zap70	chr1:36761860-36782816	OtA5088-OtA5092	OtA5093-OtA5097
Zar1	chr5:72577113-72581084	OtA5088-OtA5092	OtA5093-OtA5097
Zar1l	chr5:150507068-150518159	OtA5088-OtA5092	OtA5093-OtA5097
Zbbx	chr3:75037906-75143723	OtA5088-OtA5092	OtA5093-OtA5097

Zc3h12b	chrX:95711677-95927970	OtA5088-OtA5092	OtA5093-OtA5097
Zc3h12d	chr10:7832469-7870397	OtA5088-OtA5092	OtA5093-OtA5097
Zc3h6	chr2:128967401-129018563	OtA5088-OtA5092	OtA5093-OtA5097
Zc3h8	chr2:128926267-128944020	OtA5088-OtA5092	OtA5093-OtA5097
Zc4h2	chrX:95639196-95658432	OtA5088-OtA5092	OtA5093-OtA5097
Zcchc10	chr11:53324688-53333301	OtA5088-OtA5092	OtA5093-OtA5097
Zcchc12	chrX:36195903-36199158	OtA5088-OtA5092	OtA5093-OtA5097
Zcchc13	chrX:103630585-103631664	OtA5088-OtA5092	OtA5093-OtA5097
Zcchc16	chrX:144688906-145122410	OtA5088-OtA5092	OtA5093-OtA5097
Zcchc18	chrX:136981115-137038302	OtA5088-OtA5092	OtA5093-OtA5097
Zcchc5	chrX:106837081-106840643	OtA5088-OtA5092	OtA5093-OtA5097
Zcwpw1	chr5:137787801-137836278	OtA5088-OtA5092	OtA5093-OtA5097
Zdhhc11	chr13:73963861-73992840	OtA5088-OtA5092	OtA5093-OtA5097
Zdhhc15	chrX:104536969-104671064	OtA5088-OtA5092	OtA5093-OtA5097
Zdhhc19	chr16:32496280-32507214	OtA5088-OtA5092	OtA5093-OtA5097
Zdhhc22	chr12:86983380-86988676	OtA5088-OtA5092	OtA5093-OtA5097
Zdhhc25	chr15:88600301-88601542	OtA5088-OtA5092	OtA5093-OtA5097
Zf12	chrX_GL456233_random:159646-33418	OtA5088-OtA5092	OtA5093-OtA5097
Zfa	chr10:52542319-52545739	OtA5088-OtA5092	OtA5093-OtA5097
Zfhx2	chr14:55061658-55092048	OtA5088-OtA5092	OtA5093-OtA5097
Zfhx2as	chr14:55061658-55092048	OtA5088-OtA5092	OtA5093-OtA5097
Zfhx4	chr3:5177827-5415855	OtA5088-OtA5092	OtA5093-OtA5097
Zfp101	chr17:33380178-33394637	OtA5088-OtA5092	OtA5093-OtA5097
Zfp105	chr9:122923077-122931028	OtA5088-OtA5092	OtA5093-OtA5097
Zfp108	chr7:24254793-24262444	OtA5088-OtA5092	OtA5093-OtA5097
Zfp109	chr7:24227794-24237598	OtA5088-OtA5092	OtA5093-OtA5097
Zfp11	chr5:129654594-129670088	OtA5088-OtA5092	OtA5093-OtA5097
Zfp111	chr7:24193214-24208149	OtA5088-OtA5092	OtA5093-OtA5097
Zfp112	chr7:24112319-24127952	OtA5088-OtA5092	OtA5093-OtA5097
Zfp113	chr5:138139701-138155744	OtA5088-OtA5092	OtA5093-OtA5097
Zfp114	chr7:24175044-24182318	OtA5088-OtA5092	OtA5093-OtA5097
Zfp119a	chr17:55864926-55878930	OtA5088-OtA5092	OtA5093-OtA5097
Zfp119b	chr17:55938372-55945259	OtA5088-OtA5092	OtA5093-OtA5097
Zfp120	chr2:150114406-150136678	OtA5088-OtA5092	OtA5093-OtA5097
Zfp128	chr7:12881177-12893422	OtA5088-OtA5092	OtA5093-OtA5097
Zfp133-ps	chr2:144459279-144467794	OtA5088-OtA5092	OtA5093-OtA5097
Zfp14	chr7:30036358-30051396	OtA5088-OtA5092	OtA5093-OtA5097
Zfp157	chr5:138441475-138460694	OtA5088-OtA5092	OtA5093-OtA5097
Zfp160	chr17:21008940-21028856	OtA5088-OtA5092	OtA5093-OtA5097
Zfp174	chr16:3847222-3858880	OtA5088-OtA5092	OtA5093-OtA5097
Zfp182	chrX:21026183-21062038	OtA5088-OtA5092	OtA5093-OtA5097
Zfp184	chr13:21945093-21960485	OtA5088-OtA5092	OtA5093-OtA5097
Zfp189	chr4:49521216-49531545	OtA5088-OtA5092	OtA5093-OtA5097
Zfp2	chr11:50898711-50916161	OtA5088-OtA5092	OtA5093-OtA5097
Zfp239	chr6:117863076-117872766	OtA5088-OtA5092	OtA5093-OtA5097
Zfp248	chr6:118427318-118455506	OtA5088-OtA5092	OtA5093-OtA5097
Zfp273	chr13:67796593-67830985	OtA5088-OtA5092	OtA5093-OtA5097

Zfp28	chr7:6383317-6396637	OtA5088-OtA5092	OtA5093-OtA5097
Zfp280c	chrX:48541625-48594452	OtA5088-OtA5092	OtA5093-OtA5097
Zfp286	chr11:62778386-62789417	OtA5088-OtA5092	OtA5093-OtA5097
Zfp287	chr11:62711485-62729093	OtA5088-OtA5092	OtA5093-OtA5097
Zfp296	chr7:19577286-19580656	OtA5088-OtA5092	OtA5093-OtA5097
Zfp30	chr7:29784789-29794540	OtA5088-OtA5092	OtA5093-OtA5097
Zfp300	chrX:21079149-21089229	OtA5088-OtA5092	OtA5093-OtA5097
Zfp329	chr7:12803779-12818860	OtA5088-OtA5092	OtA5093-OtA5097
Zfp345	chr2:150470990-150485063	OtA5088-OtA5092	OtA5093-OtA5097
Zfp352	chr4:90218819-90225687	OtA5088-OtA5092	OtA5093-OtA5097
Zfp354a	chr11:51059256-51072799	OtA5088-OtA5092	OtA5093-OtA5097
Zfp354b	chr11:50921785-50931635	OtA5088-OtA5092	OtA5093-OtA5097
Zfp354c	chr11:50811084-50827731	OtA5088-OtA5092	OtA5093-OtA5097
Zfp365	chr10:67886104-67912662	OtA5088-OtA5092	OtA5093-OtA5097
Zfp367	chr13:64133056-64153199	OtA5088-OtA5092	OtA5093-OtA5097
Zfp382	chr7:30121947-30135032	OtA5088-OtA5092	OtA5093-OtA5097
Zfp385b	chr2:77410626-77816816	OtA5088-OtA5092	OtA5093-OtA5097
Zfp389	chr13:21504315-21506524	OtA5088-OtA5092	OtA5093-OtA5097
Zfp39	chr11:58888152-58904225	OtA5088-OtA5092	OtA5093-OtA5097
Zfp40	chr17:23173868-23193228	OtA5088-OtA5092	OtA5093-OtA5097
Zfp418	chr7:7171352-7183560	OtA5088-OtA5092	OtA5093-OtA5097
Zfp420	chr7:29859978-29877302	OtA5088-OtA5092	OtA5093-OtA5097
Zfp429	chr13:67389327-67399767	OtA5088-OtA5092	OtA5093-OtA5097
Zfp433	chr10:81863933-81881086	OtA5088-OtA5092	OtA5093-OtA5097
Zfp438	chr18:5210030-5334439	OtA5088-OtA5092	OtA5093-OtA5097
Zfp442	chr2:150407136-150451494	OtA5088-OtA5092	OtA5093-OtA5097
Zfp449	chrX:56346399-56365674	OtA5088-OtA5092	OtA5093-OtA5097
Zfp454	chr11:50872722-50887443	OtA5088-OtA5092	OtA5093-OtA5097
Zfp455	chr13:67194505-67209298	OtA5088-OtA5092	OtA5093-OtA5097
Zfp456	chr13:67363583-67375763	OtA5088-OtA5092	OtA5093-OtA5097
Zfp457	chr13:67292450-67306412	OtA5088-OtA5092	OtA5093-OtA5097
Zfp458	chr13:67254917-67269068	OtA5088-OtA5092	OtA5093-OtA5097
Zfp459	chr13:67405712-67421418	OtA5088-OtA5092	OtA5093-OtA5097
Zfp472	chr17:32965830-32979211	OtA5088-OtA5092	OtA5093-OtA5097
Zfp473	chr7:44731485-44748617	OtA5088-OtA5092	OtA5093-OtA5097
Zfp474	chr18:52615914-52639830	OtA5088-OtA5092	OtA5093-OtA5097
Zfp488	chr14:33967069-33978764	OtA5088-OtA5092	OtA5093-OtA5097
Zfp493	chr13:67779692-67789080	OtA5088-OtA5092	OtA5093-OtA5097
Zfp51	chr17:21450351-21465589	OtA5088-OtA5092	OtA5093-OtA5097
Zfp52	chr17:21535538-21562601	OtA5088-OtA5092	OtA5093-OtA5097
Zfp521	chr18:13687013-13972733	OtA5088-OtA5092	OtA5093-OtA5097
Zfp53	chr17:21488987-21510477	OtA5088-OtA5092	OtA5093-OtA5097
Zfp534	chr4:147673502-147702553	OtA5088-OtA5092	OtA5093-OtA5097
Zfp536	chr7:37479108-37769752	OtA5088-OtA5092	OtA5093-OtA5097
Zfp54	chr17:21423226-21435384	OtA5088-OtA5092	OtA5093-OtA5097
Zfp541	chr7:16071941-16096328	OtA5088-OtA5092	OtA5093-OtA5097
Zfp551	chr7:12415148-12422491	OtA5088-OtA5092	OtA5093-OtA5097

Zfp558	chr9:18454053-18473559	OtA5088-OtA5092	OtA5093-OtA5097
Zfp560	chr9:20345135-20385158	OtA5088-OtA5092	OtA5093-OtA5097
Zfp563	chr17:33089366-33110704	OtA5088-OtA5092	OtA5093-OtA5097
Zfp566	chr7:30077336-30090510	OtA5088-OtA5092	OtA5093-OtA5097
Zfp572	chr15:59306947-59312013	OtA5088-OtA5092	OtA5093-OtA5097
Zfp575	chr7:24583837-24608926	OtA5088-OtA5092	OtA5093-OtA5097
Zfp58	chr13:67490166-67500522	OtA5088-OtA5092	OtA5093-OtA5097
Zfp580	chr7:5051531-5053723	OtA5088-OtA5092	OtA5093-OtA5097
Zfp583	chr7:6315664-6330434	OtA5088-OtA5092	OtA5093-OtA5097
Zfp59	chr7:27838583-27856774	OtA5088-OtA5092	OtA5093-OtA5097
Zfp595	chr13:67312997-67332560	OtA5088-OtA5092	OtA5093-OtA5097
Zfp599	chr9:22055459-22389206	OtA5088-OtA5092	OtA5093-OtA5097
Zfp600	chr4:146156823-146198749	OtA5088-OtA5092	OtA5093-OtA5097
Zfp605	chr5:110110091-110129794	OtA5088-OtA5092	OtA5093-OtA5097
Zfp607	chr7:27860584-27880825	OtA5088-OtA5092	OtA5093-OtA5097
Zfp61	chr7:24291045-24299549	OtA5088-OtA5092	OtA5093-OtA5097
Zfp612	chr8:110079733-110092752	OtA5088-OtA5092	OtA5093-OtA5097
Zfp616	chr11:74070169-74087301	OtA5088-OtA5092	OtA5093-OtA5097
Zfp618	chr4:62965573-63134030	OtA5088-OtA5092	OtA5093-OtA5097
Zfp619	chr7:39517765-39540415	OtA5088-OtA5092	OtA5093-OtA5097
Zfp641	chr15:98286121-98296083	OtA5088-OtA5092	OtA5093-OtA5097
Zfp647	chr15:76910369-76925448	OtA5088-OtA5092	OtA5093-OtA5097
Zfp648	chr1:154201186-154205674	OtA5088-OtA5092	OtA5093-OtA5097
Zfp658	chr7:43562369-43575461	OtA5088-OtA5092	OtA5093-OtA5097
Zfp661	chr2:127575532-127584677	OtA5088-OtA5092	OtA5093-OtA5097
Zfp663	chr2:165351296-165362119	OtA5088-OtA5092	OtA5093-OtA5097
Zfp677	chr17:21383747-21399265	OtA5088-OtA5092	OtA5093-OtA5097
Zfp68	chr5:138603651-138619743	OtA5088-OtA5092	OtA5093-OtA5097
Zfp689	chr7:127442135-127449158	OtA5088-OtA5092	OtA5093-OtA5097
Zfp69	chr4:120930136-120951701	OtA5088-OtA5092	OtA5093-OtA5097
Zfp708	chr13:67069397-67114028	OtA5088-OtA5092	OtA5093-OtA5097
Zfp709	chr8:71882067-71892565	OtA5088-OtA5092	OtA5093-OtA5097
Zfp71-rs1	chr13:67705305-67729173	OtA5088-OtA5092	OtA5093-OtA5097
Zfp711	chrX:112600525-112635062	OtA5088-OtA5092	OtA5093-OtA5097
Zfp712	chr13:67038593-67061170	OtA5088-OtA5092	OtA5093-OtA5097
Zfp719	chr7:43579585-43593710	OtA5088-OtA5092	OtA5093-OtA5097
Zfp72	chr13:74371425-74376566	OtA5088-OtA5092	OtA5093-OtA5097
Zfp735	chr11:73688777-73713808	OtA5088-OtA5092	OtA5093-OtA5097
Zfp738	chr13:67667440-67683512	OtA5088-OtA5092	OtA5093-OtA5097
Zfp750	chr11:121451948-121673151	OtA5088-OtA5092	OtA5093-OtA5097
Zfp758	chr17:22361452-22377281	OtA5088-OtA5092	OtA5093-OtA5097
Zfp759	chr13:67128227-67141787	OtA5088-OtA5092	OtA5093-OtA5097
Zfp760	chr17:21707740-21725636	OtA5088-OtA5092	OtA5093-OtA5097
Zfp763	chr17:33016863-33033381	OtA5088-OtA5092	OtA5093-OtA5097
Zfp770	chr2:114193460-114201432	OtA5088-OtA5092	OtA5093-OtA5097
Zfp773	chr7:7130677-7136755	OtA5088-OtA5092	OtA5093-OtA5097
Zfp78	chr7:6363307-6382605	OtA5088-OtA5092	OtA5093-OtA5097

Zfp780b	chr7:27959799-27979157	OtA5088-OtA5092	OtA5093-OtA5097
Zfp781	chr10:81901910-81930480	OtA5088-OtA5092	OtA5093-OtA5097
Zfp786	chr6:47819265-47830505	OtA5088-OtA5092	OtA5093-OtA5097
Zfp788	chr7:41633530-41651532	OtA5088-OtA5092	OtA5093-OtA5097
Zfp799	chr17:32817203-32830248	OtA5088-OtA5092	OtA5093-OtA5097
Zfp804a	chr2:82053657-82259878	OtA5088-OtA5092	OtA5093-OtA5097
Zfp804b	chr5:6769029-7344378	OtA5088-OtA5092	OtA5093-OtA5097
Zfp808	chr13:62129889-62173936	OtA5088-OtA5092	OtA5093-OtA5097
Zfp81	chr17:33333727-33358878	OtA5088-OtA5092	OtA5093-OtA5097
Zfp811	chr17:32797010-32809919	OtA5088-OtA5092	OtA5093-OtA5097
Zfp819	chr7:43607168-43618279	OtA5088-OtA5092	OtA5093-OtA5097
Zfp820	chr17:21816875-21845759	OtA5088-OtA5092	OtA5093-OtA5097
Zfp825	chr13:74480056-74493950	OtA5088-OtA5092	OtA5093-OtA5097
Zfp831	chr2:174643533-174710830	OtA5088-OtA5092	OtA5093-OtA5097
Zfp85-rs1	chr13:67729261-67756838	OtA5088-OtA5092	OtA5093-OtA5097
Zfp867	chr11:59461196-59472474	OtA5088-OtA5092	OtA5093-OtA5097
Zfp870	chr17:32879220-32891603	OtA5088-OtA5092	OtA5093-OtA5097
Zfp872	chr9:22055459-22389206	OtA5088-OtA5092	OtA5093-OtA5097
Zfp873	chr10:82048126-82061586	OtA5088-OtA5092	OtA5093-OtA5097
Zfp879	chr11:50832034-50841552	OtA5088-OtA5092	OtA5093-OtA5097
Zfp882	chr8:71908605-71918851	OtA5088-OtA5092	OtA5093-OtA5097
Zfp92	chrX:73411102-73426987	OtA5088-OtA5092	OtA5093-OtA5097
Zfp93	chr7:24270417-24277794	OtA5088-OtA5092	OtA5093-OtA5097
Zfp930	chr8:69209045-69230539	OtA5088-OtA5092	OtA5093-OtA5097
Zfp931	chr2:178066877-178078425	OtA5088-OtA5092	OtA5093-OtA5097
Zfp932	chr5:109996526-110010411	OtA5088-OtA5092	OtA5093-OtA5097
Zfp933	chr4:147822985-147848375	OtA5088-OtA5092	OtA5093-OtA5097
Zfp934	chr13:62516796-62558599	OtA5088-OtA5092	OtA5093-OtA5097
Zfp936	chr7:43177587-43192109	OtA5088-OtA5092	OtA5093-OtA5097
Zfp937	chr2:150218098-150244874	OtA5088-OtA5092	OtA5093-OtA5097
Zfp938	chr10:82224855-82241275	OtA5088-OtA5092	OtA5093-OtA5097
Zfp939	chr7:39449517-39477416	OtA5088-OtA5092	OtA5093-OtA5097
Zfp94	chr7:24301703-24316666	OtA5088-OtA5092	OtA5093-OtA5097
Zfp940	chr7:29843935-29853648	OtA5088-OtA5092	OtA5093-OtA5097
Zfp941	chr7:140809676-140822178	OtA5088-OtA5092	OtA5093-OtA5097
Zfp943	chr17:21962558-21994366	OtA5088-OtA5092	OtA5093-OtA5097
Zfp944	chr17:22337988-22361400	OtA5088-OtA5092	OtA5093-OtA5097
Zfp945	chr17:22846696-22867134	OtA5088-OtA5092	OtA5093-OtA5097
Zfp946	chr17:22424267-22456689	OtA5088-OtA5092	OtA5093-OtA5097
Zfp947	chr17:22144358-22165977	OtA5088-OtA5092	OtA5093-OtA5097
Zfp951	chr5:104813167-104860068	OtA5088-OtA5092	OtA5093-OtA5097
Zfp952	chr17:32993138-33005457	OtA5088-OtA5092	OtA5093-OtA5097
Zfp953	chr13:67339308-67360572	OtA5088-OtA5092	OtA5093-OtA5097
Zfp955a	chr17:33239506-33255145	OtA5088-OtA5092	OtA5093-OtA5097
Zfp955b	chr17:33289543-33304689	OtA5088-OtA5092	OtA5093-OtA5097
Zfp957	chr14:79212354-79247367	OtA5088-OtA5092	OtA5093-OtA5097
Zfp958	chr8:4613169-4630231	OtA5088-OtA5092	OtA5093-OtA5097

Zfp959	chr17:55892092-55898930	OtA5088-OtA5092	OtA5093-OtA5097
Zfp960	chr17:17064102-17089631	OtA5088-OtA5092	OtA5093-OtA5097
Zfp963	chr8:69741638-69749962	OtA5088-OtA5092	OtA5093-OtA5097
Zfp97	chr17:17121382-17146878	OtA5088-OtA5092	OtA5093-OtA5097
Zfpm2	chr15:40655041-41104592	OtA5088-OtA5092	OtA5093-OtA5097
Zfy1	chrY:725206-797409	OtA5088-OtA5092	OtA5093-OtA5097
Zfy2	chrY:2106174-2170409	OtA5088-OtA5092	OtA5093-OtA5097
Zfyve28	chr5:34194893-34288324	OtA5088-OtA5092	OtA5093-OtA5097
Zg16	chr7:127050155-127051977	OtA5088-OtA5092	OtA5093-OtA5097
Zglp1	chr9:21062392-21067093	OtA5088-OtA5092	OtA5093-OtA5097
Zic1	chr9:91360344-91365799	OtA5088-OtA5092	OtA5093-OtA5097
Zic2	chr14:122475383-122480328	OtA5088-OtA5092	OtA5093-OtA5097
Zic4	chr9:91368971-91389348	OtA5088-OtA5092	OtA5093-OtA5097
Zic5	chr14:122459159-122465658	OtA5088-OtA5092	OtA5093-OtA5097
Zik1	chr7:10487223-10495381	OtA5088-OtA5092	OtA5093-OtA5097
Zim3	chr7:6730740-6976662	OtA5088-OtA5092	OtA5093-OtA5097
Zkscan16	chr4:58943627-58958355	OtA5088-OtA5092	OtA5093-OtA5097
Zkscan2	chr7:123478638-123500449	OtA5088-OtA5092	OtA5093-OtA5097
Zkscan4	chr13:21478848-21485505	OtA5088-OtA5092	OtA5093-OtA5097
Zmynd10	chr9:107547309-107551319	OtA5088-OtA5092	OtA5093-OtA5097
Zmynd12	chr4:119422683-119453899	OtA5088-OtA5092	OtA5093-OtA5097
Zmynd15	chr11:70454233-70466199	OtA5088-OtA5092	OtA5093-OtA5097
Zmynd17	chr14:20482863-20496901	OtA5088-OtA5092	OtA5093-OtA5097
Znhit6	chr3:145576207-145605245	OtA5088-OtA5092	OtA5093-OtA5097
Znrd1as	chr17:36958591-36965623	OtA5088-OtA5092	OtA5093-OtA5097
Znrf4	chr17:56511247-56512483	OtA5088-OtA5092	OtA5093-OtA5097
Zp1	chr19:10914295-10920601	OtA5088-OtA5092	OtA5093-OtA5097
Zp2	chr7:120132360-120145290	OtA5088-OtA5092	OtA5093-OtA5097
Zp3	chr5:135980104-135988624	OtA5088-OtA5092	OtA5093-OtA5097
Zp3r	chr1:130576705-130629606	OtA5088-OtA5092	OtA5093-OtA5097
Zp4-ps	chr13:11522051-11525682	OtA5088-OtA5092	OtA5093-OtA5097
Zpbp	chr11:11280039-11462419	OtA5088-OtA5092	OtA5093-OtA5097
Zpbp2	chr11:98551096-98558665	OtA5088-OtA5092	OtA5093-OtA5097
Zpld1	chr16:55225174-55283237	OtA5088-OtA5092	OtA5093-OtA5097
Zranb3	chr1:127954185-128102408	OtA5088-OtA5092	OtA5093-OtA5097
Zscan10	chr17:23600855-23611019	OtA5088-OtA5092	OtA5093-OtA5097
Zscan12	chr13:21362819-21372302	OtA5088-OtA5092	OtA5093-OtA5097
Zscan18	chr7:12768091-12775658	OtA5088-OtA5092	OtA5093-OtA5097
Zscan4a	chr7:10794240-10799065	OtA5088-OtA5092	OtA5093-OtA5097
Zscan4b	chr7:10900739-10905050	OtA5088-OtA5092	OtA5093-OtA5097
Zscan4c	chr7:11005744-11010547	OtA5088-OtA5092	OtA5093-OtA5097
Zscan4d	chr7:11161642-11166148	OtA5088-OtA5092	OtA5093-OtA5097
Zscan4e	chr7:11306377-11310682	OtA5088-OtA5092	OtA5093-OtA5097
Zscan4f	chr7:11397914-11402318	OtA5088-OtA5092	OtA5093-OtA5097
Zscan5b	chr7:6222277-6239412	OtA5088-OtA5092	OtA5093-OtA5097
Zswim2	chr2:83915078-83941226	OtA5088-OtA5092	OtA5093-OtA5097
Zswim5	chr4:116877401-116989105	OtA5088-OtA5092	OtA5093-OtA5097

Zwilch	chr9:64117146-64172931	OtA5088-OtA5092	OtA5093-OtA5097
Zxda	chrX:94791284-94798289	OtA5088-OtA5092	OtA5093-OtA5097
a	chr2:155013569-155051012	OtA5088-OtA5092	OtA5093-OtA5097

Controls	Nulls	log2(fold_chang	test_stat	p_value	q_value	significant
66.1985	103.409	0.643494	2.16569	5.00E-05	0.00427132	yes
16.3639	23.8259	0.542014	1.86419	5.00E-05	0.00427132	yes
47.0839	34.3026	-0.456914	-1.69931	5.00E-05	0.00427132	yes
33.6468	47.8317	0.507498	1.88376	5.00E-05	0.00427132	yes
3.95095	7.72024	0.966444	2.43357	5.00E-05	0.00427132	yes
54.0482	83.1913	0.622186	2.19612	5.00E-05	0.00427132	yes
48.5349	75.6639	0.640584	1.83073	5.00E-05	0.00427132	yes
83.2061	137.167	0.72117	1.88079	5.00E-05	0.00427132	yes
2.64447	5.11388	0.951441	1.86522	5.00E-05	0.00427132	yes
2.15276	6.75054	1.64881	2.92974	5.00E-05	0.00427132	yes
2.66527	5.30616	0.993386	2.32007	5.00E-05	0.00427132	yes
12.9286	18.1183	0.48688	1.73969	5.00E-05	0.00427132	yes
28.3536	20.3103	-0.481322	-1.79007	5.00E-05	0.00427132	yes
3.44377	2.27582	-0.597604	-1.81921	5.00E-05	0.00427132	yes
18.7973	13.5269	-0.474695	-1.77553	5.00E-05	0.00427132	yes
5.28206	8.58165	0.700154	2.03656	5.00E-05	0.00427132	yes
62.822	109.097	0.796272	2.88372	5.00E-05	0.00427132	yes
16.5621	23.33	0.494298	1.80241	5.00E-05	0.00427132	yes
18.2311	8.01129	-1.18629	-2.16972	5.00E-05	0.00427132	yes
53.5056	34.7086	-0.624398	-2.16919	5.00E-05	0.00427132	yes
5.1991	1.9419	-1.42079	-2.24111	5.00E-05	0.00427132	yes
3.98909	1.68916	-1.23975	-2.3425	5.00E-05	0.00427132	yes
16.1178	23.7622	0.560017	1.77923	5.00E-05	0.00427132	yes
1.11198	2.19316	0.979877	1.76247	5.00E-05	0.00427132	yes
6.35975	3.77869	-0.751085	-2.20909	5.00E-05	0.00427132	yes
478.075	227.02	-1.07442	-3.48158	5.00E-05	0.00427132	yes
1595.27	812.588	-0.973206	-2.48318	5.00E-05	0.00427132	yes
145.338	85.6483	-0.762915	-2.80371	5.00E-05	0.00427132	yes
8.51951	14.6822	0.78523	1.87312	5.00E-05	0.00427132	yes
12.1403	18.9932	0.645676	1.89533	5.00E-05	0.00427132	yes
12.6698	18.7181	0.563047	1.81797	5.00E-05	0.00427132	yes
3.48028	1.35385	-1.36213	-2.62466	5.00E-05	0.00427132	yes
8.67783	4.88429	-0.829185	-2.76485	5.00E-05	0.00427132	yes
3.63899	2.20437	-0.723171	-1.90971	5.00E-05	0.00427132	yes
859.794	466.507	-0.882092	-2.22794	5.00E-05	0.00427132	yes
13.2777	8.71212	-0.607907	-2.18594	5.00E-05	0.00427132	yes
28.275	40.1261	0.505014	1.83725	5.00E-05	0.00427132	yes
2.70622	1.32974	-1.02514	-1.81291	5.00E-05	0.00427132	yes
7.48406	4.76306	-0.651931	-1.91463	5.00E-05	0.00427132	yes
13.3643	9.4505	-0.499926	-1.81646	5.00E-05	0.00427132	yes
1.37214	3.45335	1.33157	2.62677	5.00E-05	0.00427132	yes
56.1593	37.9311	-0.566146	-1.79795	5.00E-05	0.00427132	yes
73.2032	42.5481	-0.782811	-2.35811	5.00E-05	0.00427132	yes
9.93144	18.9614	0.932989	2.1824	5.00E-05	0.00427132	yes
64.2647	40.7259	-0.658079	-2.38973	5.00E-05	0.00427132	yes

96.7399	150.656	0.639076	2.36334	5.00E-05	0.00427132	yes
20.2887	14.1411	-0.520788	-1.93289	5.00E-05	0.00427132	yes
0.666721	1.43657	1.10748	2.69371	5.00E-05	0.00427132	yes
6.63399	12.9747	0.967757	2.51131	5.00E-05	0.00427132	yes
22.5873	33.6866	0.576664	2.11416	5.00E-05	0.00427132	yes
15.8573	24.8787	0.649765	2.21131	5.00E-05	0.00427132	yes
7.78028	21.3266	1.45476	2.93719	5.00E-05	0.00427132	yes
17.9036	49.2007	1.45843	3.65272	5.00E-05	0.00427132	yes
7.74831	25.4397	1.71513	3.50197	5.00E-05	0.00427132	yes
20.9375	56.2058	1.42463	3.67734	5.00E-05	0.00427132	yes
18.0109	55.0577	1.61207	4.00188	5.00E-05	0.00427132	yes
4.15879	12.158	1.54766	2.17845	5.00E-05	0.00427132	yes
13.3697	8.74499	-0.61244	-2.08104	5.00E-05	0.00427132	yes
7.2816	11.518	0.661562	1.86469	5.00E-05	0.00427132	yes
8.65482	25.5094	1.55945	2.23274	5.00E-05	0.00427132	yes
13.1462	28.4943	1.11602	2.00532	5.00E-05	0.00427132	yes
13.1224	36.1832	1.46329	2.41704	5.00E-05	0.00427132	yes
10.9354	7.44908	-0.55387	-2.02105	5.00E-05	0.00427132	yes
3.50775	1.086	-1.69152	-3.18178	5.00E-05	0.00427132	yes
24.9448	10.0336	-1.3139	-3.2305	5.00E-05	0.00427132	yes
59.5265	43.0992	-0.465871	-1.73509	5.00E-05	0.00427132	yes
29.196	43.6555	0.580393	1.91078	5.00E-05	0.00427132	yes
42.6839	66.6501	0.642916	2.34127	5.00E-05	0.00427132	yes
6.97587	4.42955	-0.655213	-1.85748	5.00E-05	0.00427132	yes
2.657	1.76479	-0.590301	-2.1305	5.00E-05	0.00427132	yes
0.418652	1.51712	1.85752	2.28283	5.00E-05	0.00427132	yes
4.32971	1.57921	-1.45507	-2.35279	5.00E-05	0.00427132	yes
39.994	87.431	1.12836	2.84653	5.00E-05	0.00427132	yes
11.273	5.61888	-1.00452	-2.01309	5.00E-05	0.00427132	yes
9.16762	4.88028	-0.909582	-2.91279	5.00E-05	0.00427132	yes
30.906	89.316	1.53103	3.19019	5.00E-05	0.00427132	yes
2.92968	1.72963	-0.760277	-1.76973	5.00E-05	0.00427132	yes
38.1116	52.4978	0.462028	1.71515	5.00E-05	0.00427132	yes
29.7357	13.1283	-1.17952	-2.67928	5.00E-05	0.00427132	yes
6.28779	3.25733	-0.948865	-2.89498	5.00E-05	0.00427132	yes
8.99049	16.0995	0.840547	2.02865	5.00E-05	0.00427132	yes
10.0853	15.566	0.626135	2.12655	5.00E-05	0.00427132	yes
15.645	11.042	-0.502696	-1.85801	5.00E-05	0.00427132	yes
17.6518	25.8079	0.547993	1.89031	5.00E-05	0.00427132	yes
30.347	52.0171	0.77743	2.79082	5.00E-05	0.00427132	yes
3.70541	6.42099	0.793161	2.17963	5.00E-05	0.00427132	yes
5.37631	8.90081	0.727321	2.00466	5.00E-05	0.00427132	yes
21.0382	35.9819	0.774265	1.85437	5.00E-05	0.00427132	yes
28.1836	40.1336	0.509955	1.83595	5.00E-05	0.00427132	yes
4.62761	3.07004	-0.59201	-1.97176	5.00E-05	0.00427132	yes
3.00561	10.004	1.73485	3.38907	5.00E-05	0.00427132	yes
20.5157	39.3595	0.939981	2.5345	5.00E-05	0.00427132	yes

36.2787	20.414	-0.829566	-2.37466	5.00E-05	0.00427132	yes
2.85591	4.52625	0.664369	2.05707	5.00E-05	0.00427132	yes
47.1569	76.6115	0.700092	2.50791	5.00E-05	0.00427132	yes
13.1777	25.1068	0.929984	3.10425	5.00E-05	0.00427132	yes
37.039	52.8954	0.514098	1.89316	5.00E-05	0.00427132	yes
30.0961	46.4137	0.624974	2.2951	5.00E-05	0.00427132	yes
16.7813	11.8318	-0.504182	-1.85285	5.00E-05	0.00427132	yes
11.2086	18.0678	0.688814	1.87049	5.00E-05	0.00427132	yes
15.828	10.2373	-0.628635	-1.7758	5.00E-05	0.00427132	yes
1.3948	3.89712	1.48235	2.46186	5.00E-05	0.00427132	yes
7.71002	11.8102	0.615226	1.77432	5.00E-05	0.00427132	yes
7.50059	12.675	0.756915	1.90301	5.00E-05	0.00427132	yes
6.19752	11.225	0.856958	2.75538	5.00E-05	0.00427132	yes
27.1856	42.1936	0.634182	2.32413	5.00E-05	0.00427132	yes
1.62072	4.23611	1.38611	2.3195	5.00E-05	0.00427132	yes
117.23	170.236	0.538195	1.79123	5.00E-05	0.00427132	yes
168.402	406.054	1.26976	2.8485	5.00E-05	0.00427132	yes
3.94874	1.85148	-1.09271	-3.0693	5.00E-05	0.00427132	yes
24.0927	55.8406	1.21272	3.81907	5.00E-05	0.00427132	yes
7.02599	12.5722	0.83946	2.48774	5.00E-05	0.00427132	yes
4.33767	8.63782	0.993749	2.40779	5.00E-05	0.00427132	yes
28.8667	19.8832	-0.537856	-1.86587	5.00E-05	0.00427132	yes
120.256	49.9363	-1.26795	-3.87678	5.00E-05	0.00427132	yes
71.4341	48.8627	-0.547878	-1.96428	5.00E-05	0.00427132	yes
22.0761	14.4435	-0.612065	-1.88149	5.00E-05	0.00427132	yes
33.7658	7.46478	-2.17739	-5.69585	5.00E-05	0.00427132	yes
3.64485	1.80197	-1.01628	-2.35919	5.00E-05	0.00427132	yes
30.2573	42.6382	0.49486	1.83382	5.00E-05	0.00427132	yes
18.9726	29.6688	0.645029	1.81167	5.00E-05	0.00427132	yes
24.124	36.7469	0.607153	2.15316	5.00E-05	0.00427132	yes
65.9786	91.2728	0.468187	1.72847	5.00E-05	0.00427132	yes
4.56515	7.6299	0.741002	2.11253	5.00E-05	0.00427132	yes
9.07218	4.70686	-0.946685	-2.61023	5.00E-05	0.00427132	yes
62.1009	99.6133	0.681723	2.48705	5.00E-05	0.00427132	yes
18.835	35.2586	0.904561	1.92239	5.00E-05	0.00427132	yes
6.70909	4.10203	-0.709781	-2.52505	5.00E-05	0.00427132	yes
171.981	239.204	0.475991	1.73006	0.0001	0.00749548	yes
67.3677	91.5014	0.441737	1.63348	0.0001	0.00749548	yes
19.7109	28.4366	0.528754	1.81672	0.0001	0.00749548	yes
2.05233	4.12957	1.00873	2.05472	0.0001	0.00749548	yes
57.5	40.3069	-0.512538	-1.70802	0.0001	0.00749548	yes
8.88313	6.0115	-0.563344	-1.7635	0.0001	0.00749548	yes
1.59011	3.25975	1.03563	2.02376	0.0001	0.00749548	yes
9.08214	17.843	0.974254	1.79216	0.0001	0.00749548	yes
4.83153	3.28674	-0.555822	-1.87671	0.0001	0.00749548	yes
11.5464	4.96379	-1.21793	-2.15518	0.0001	0.00749548	yes
11.635	16.7298	0.523941	1.8356	0.0001	0.00749548	yes

15.386	21.9242	0.510899	1.76881	0.0001	0.00749548	yes
12.4615	8.60358	-0.534464	-1.90515	0.0001	0.00749548	yes
3.84749	6.1896	0.685929	1.87268	0.0001	0.00749548	yes
96.6471	70.5513	-0.454053	-1.68356	0.0001	0.00749548	yes
69.3863	49.2491	-0.494552	-1.72648	0.0001	0.00749548	yes
75.5838	111.738	0.56397	2.0052	0.0001	0.00749548	yes
50.8636	71.423	0.489756	1.74833	0.0001	0.00749548	yes
8.47175	5.58017	-0.602349	-1.77234	0.0001	0.00749548	yes
1.84881	3.76754	1.02703	1.82749	0.00015	0.0105618	yes
11.7127	8.23957	-0.507428	-1.59014	0.00015	0.0105618	yes
4.07426	1.86223	-1.12951	-1.82692	0.00015	0.0105618	yes
23.0398	35.6661	0.630426	1.83374	0.00015	0.0105618	yes
342.326	468.129	0.451534	1.67896	0.00015	0.0105618	yes
10.5871	7.48634	-0.499971	-1.79497	0.00015	0.0105618	yes
14.4848	10.6515	-0.443484	-1.62665	0.00015	0.0105618	yes
19.7088	27.6453	0.488198	1.71129	0.00015	0.0105618	yes
11.4836	15.9288	0.47206	1.65607	0.00015	0.0105618	yes
9.91179	7.17004	-0.467165	-1.69679	0.00015	0.0105618	yes
4.11216	2.80972	-0.54947	-1.68788	0.0002	0.0132023	yes
22.8093	16.2038	-0.493287	-1.61667	0.0002	0.0132023	yes
26.1674	35.724	0.449119	1.57726	0.0002	0.0132023	yes
0.844505	2.86557	1.76264	2.02263	0.0002	0.0132023	yes
5.62646	3.17251	-0.826603	-1.86195	0.0002	0.0132023	yes
28.0504	37.7178	0.427221	1.55328	0.0002	0.0132023	yes
12.0736	16.364	0.438665	1.63334	0.0002	0.0132023	yes
24.9733	35.0762	0.490108	1.75754	0.0002	0.0132023	yes
0.529594	1.9606	1.88834	2.03515	0.0002	0.0132023	yes
11.5019	7.7253	-0.574205	-1.67186	0.00025	0.0153677	yes
16.6031	12.3864	-0.422695	-1.58409	0.00025	0.0153677	yes
18.745	13.2789	-0.497371	-1.72028	0.00025	0.0153677	yes
320.212	216.925	-0.561827	-1.66287	0.00025	0.0153677	yes
13.8763	9.9881	-0.474345	-1.65349	0.00025	0.0153677	yes
16.9389	12.4818	-0.440514	-1.63586	0.00025	0.0153677	yes
38.4523	52.485	0.448837	1.67207	0.00025	0.0153677	yes
11.7587	6.28886	-0.902855	-1.8464	0.00025	0.0153677	yes
32.6402	44.5117	0.447535	1.65418	0.00025	0.0153677	yes
6.32469	3.54111	-0.836795	-1.70874	0.00025	0.0153677	yes
16.2649	23.1787	0.511033	1.62932	0.00025	0.0153677	yes
5.71113	4.03804	-0.500122	-1.5668	0.00025	0.0153677	yes
17.8419	13.1374	-0.441591	-1.64246	0.0003	0.017966	yes
24.6243	18.5749	-0.406727	-1.51542	0.0003	0.017966	yes
3.84036	2.64218	-0.539513	-1.67359	0.0003	0.017966	yes
22.5227	30.839	0.453377	1.67663	0.0003	0.017966	yes
1.50447	0.656009	-1.19747	-1.80909	0.0003	0.017966	yes
118.836	161.207	0.439939	1.60383	0.00035	0.019644	yes
19.1239	13.978	-0.452218	-1.65961	0.00035	0.019644	yes
25.1069	33.4037	0.411925	1.54261	0.00035	0.019644	yes

11.2765	8.03106	-0.489654	-1.58193	0.00035	0.019644	yes
3.25246	5.19926	0.676774	1.77136	0.00035	0.019644	yes
6.72758	4.68637	-0.521617	-1.53446	0.00035	0.019644	yes
46.5342	64.1578	0.463332	1.60946	0.00035	0.019644	yes
1.04328	2.04914	0.973891	1.79949	0.00035	0.019644	yes
56.2692	75.955	0.432799	1.61354	0.00035	0.019644	yes
1.16092	2.39451	1.04447	1.66619	0.00035	0.019644	yes
16.6377	22.6605	0.445727	1.63766	0.00035	0.019644	yes
53.73	73.1478	0.445088	1.65853	0.00035	0.019644	yes
1.39898	3.01723	1.10885	1.75503	0.00035	0.019644	yes
11.8771	8.23937	-0.527578	-1.56253	0.0004	0.0216149	yes
2040.23	1257.67	-0.697976	-1.6734	0.0004	0.0216149	yes
4.3427	2.97616	-0.54514	-1.58947	0.0004	0.0216149	yes
32.6037	43.7022	0.422669	1.54386	0.0004	0.0216149	yes
4.23123	8.86745	1.06744	1.59756	0.0004	0.0216149	yes
4.23699	3.00019	-0.497987	-1.6275	0.0004	0.0216149	yes
11.698	17.0107	0.540182	1.61891	0.0004	0.0216149	yes
28.2652	37.9639	0.425601	1.55304	0.0004	0.0216149	yes
7.70366	10.5878	0.458781	1.54428	0.00045	0.0238726	yes
7.09243	14.7476	1.05613	1.70207	0.00045	0.0238726	yes
16.7699	22.841	0.44575	1.52999	0.00045	0.0238726	yes
15.6813	21.7042	0.468932	1.60876	0.00045	0.0238726	yes
3.89153	2.74239	-0.5049	-1.51197	0.0005	0.0257035	yes
1.11134	2.81337	1.33999	1.64509	0.0005	0.0257035	yes
65.3715	46.8189	-0.48157	-1.53509	0.0005	0.0257035	yes
8.95745	6.76614	-0.404754	-1.51398	0.0005	0.0257035	yes
2.10128	3.50843	0.73956	1.55049	0.0005	0.0257035	yes
5.3516	3.53264	-0.599225	-1.53996	0.0005	0.0257035	yes
24.8047	33.5328	0.434961	1.58989	0.0005	0.0257035	yes
8.93552	6.69849	-0.415717	-1.51743	0.00055	0.0269616	yes
10.8336	8.04698	-0.428999	-1.56946	0.00055	0.0269616	yes
2.92725	5.96535	1.02706	1.61196	0.00055	0.0269616	yes
18.2013	13.4871	-0.432459	-1.58108	0.00055	0.0269616	yes
2.75208	4.62767	0.749763	1.59009	0.00055	0.0269616	yes
1.1678	2.23234	0.934764	1.66146	0.00055	0.0269616	yes
3.7568	2.51836	-0.577022	-1.5724	0.00055	0.0269616	yes
40.1224	27.4574	-0.547212	-1.5796	0.00055	0.0269616	yes
1.42743	2.99236	1.06787	1.73572	0.00055	0.0269616	yes
6.79253	10.6647	0.650818	1.62225	0.00055	0.0269616	yes
17.3976	23.3755	0.426106	1.47497	0.00055	0.0269616	yes
53.2685	70.8302	0.41108	1.53117	0.0006	0.0285689	yes
3.2745	4.70689	0.523499	1.50874	0.0006	0.0285689	yes
6.76155	5.01487	-0.431143	-1.46709	0.0006	0.0285689	yes
2.62781	4.82665	0.877159	1.6092	0.0006	0.0285689	yes
105.95	78.8132	-0.42687	-1.51103	0.0006	0.0285689	yes
0.662746	1.55416	1.2296	1.70472	0.0006	0.0285689	yes
4.43477	2.8993	-0.613154	-1.53736	0.0006	0.0285689	yes

6.27738	9.85699	0.650984	1.57011	0.00065	0.0299671	yes
6.93285	5.02685	-0.463793	-1.48959	0.00065	0.0299671	yes
5.70532	14.2822	1.32384	1.59625	0.00065	0.0299671	yes
12.2221	8.98657	-0.443649	-1.48095	0.00065	0.0299671	yes
43.1219	32.3198	-0.416002	-1.53913	0.00065	0.0299671	yes
10.0131	16.14	0.688757	1.55967	0.00065	0.0299671	yes
11.7448	17.3431	0.562342	1.57621	0.00065	0.0299671	yes
19.9779	36.6807	0.876616	1.60685	0.0007	0.0315217	yes
0.726905	1.50429	1.04924	1.54314	0.0007	0.0315217	yes
15.4935	11.7402	-0.400215	-1.47899	0.0007	0.0315217	yes
6.19606	8.76567	0.500515	1.47683	0.0007	0.0315217	yes
0.776434	1.73049	1.15625	1.60636	0.0007	0.0315217	yes
2.86858	4.56724	0.670989	1.54825	0.0007	0.0315217	yes
47.7842	64.3539	0.429492	1.50655	0.00075	0.0331312	yes
3.18192	1.94625	-0.7092	-1.60734	0.00075	0.0331312	yes
10.8277	15.4867	0.516306	1.50714	0.00075	0.0331312	yes
15.2471	11.5061	-0.406134	-1.51108	0.00075	0.0331312	yes
8.88388	13.8004	0.635447	1.57717	0.00075	0.0331312	yes
5.29669	3.46396	-0.61267	-1.54792	0.0008	0.0348105	yes
23.7582	17.8928	-0.409049	-1.51489	0.0008	0.0348105	yes
13.9534	18.7138	0.423484	1.5407	0.0008	0.0348105	yes
61.001	46.5166	-0.391089	-1.43415	0.0008	0.0348105	yes
1.22224	2.34361	0.939199	1.55353	0.00085	0.0367112	yes
13.7706	10.4406	-0.399395	-1.47862	0.00085	0.0367112	yes
3.97915	2.84453	-0.484269	-1.47956	0.0009	0.0381613	yes
46.7671	69.3386	0.568164	1.49893	0.0009	0.0381613	yes
7.72892	14.1795	0.87547	1.56863	0.0009	0.0381613	yes
32.3744	43.5665	0.428366	1.53053	0.0009	0.0381613	yes
1.88911	2.84364	0.590034	1.49341	0.0009	0.0381613	yes
6.46783	4.73246	-0.45069	-1.50147	0.00095	0.0384568	yes
4.58363	6.35026	0.470324	1.49901	0.00095	0.0384568	yes
1.65114	0.768722	-1.10293	-1.58179	0.00095	0.0384568	yes
5.68753	4.05519	-0.488034	-1.47521	0.00095	0.0384568	yes
70.1794	100.644	0.520141	1.49513	0.00095	0.0384568	yes
36.697	48.8259	0.411982	1.4195	0.00095	0.0384568	yes
77.1371	103.794	0.428223	1.4861	0.00095	0.0384568	yes
15.7677	11.8781	-0.40867	-1.45088	0.00095	0.0384568	yes
36.8583	28.2791	-0.382257	-1.41376	0.00095	0.0384568	yes
306.516	536.388	0.807315	1.50742	0.00095	0.0384568	yes
2.94186	4.12363	0.487188	1.48477	0.00095	0.0384568	yes
14.04	19.0868	0.443034	1.43357	0.00095	0.0384568	yes
9.87427	7.47627	-0.401356	-1.46736	0.00095	0.0384568	yes
2.44999	3.74038	0.610412	1.47149	0.001	0.039517	yes
10.0239	7.52786	-0.413139	-1.46722	0.001	0.039517	yes
19.548	14.7221	-0.409036	-1.44411	0.001	0.039517	yes
4.41637	7.33243	0.73143	1.51376	0.001	0.039517	yes
1.50645	2.33801	0.634128	1.52389	0.001	0.039517	yes

50.1036	67.8472	0.437375	1.47239	0.001	0.039517	yes
11.7265	8.35353	-0.489316	-1.47536	0.001	0.039517	yes
35.8348	46.975	0.390531	1.4557	0.00105	0.040799	yes
2.88905	1.5865	-0.864745	-1.54354	0.00105	0.040799	yes
26.2198	19.8	-0.405158	-1.48211	0.00105	0.040799	yes
13.268	9.81085	-0.435499	-1.52548	0.00105	0.040799	yes
10.4361	7.79652	-0.420684	-1.46887	0.00105	0.040799	yes
1.47241	0.967474	-0.605887	-1.48936	0.0011	0.041901	yes
22.2215	30.6597	0.464386	1.4838	0.0011	0.041901	yes
5.17321	2.33908	-1.14512	-1.6738	0.0011	0.041901	yes
7.67243	13.948	0.862301	1.50463	0.0011	0.041901	yes
64.8982	87.6407	0.433423	1.481	0.0011	0.041901	yes
87.6506	115.701	0.400562	1.44744	0.0011	0.041901	yes
47.3484	34.2304	-0.468036	-1.431	0.00115	0.0432385	yes
7.68441	5.58117	-0.461367	-1.4067	0.00115	0.0432385	yes
5.47866	2.97164	-0.882564	-1.48378	0.00115	0.0432385	yes
24.4392	33.4449	0.452592	1.4603	0.00115	0.0432385	yes
8.29874	4.49051	-0.886011	-1.52024	0.0012	0.0446846	yes
14.3396	10.9875	-0.384131	-1.41778	0.0012	0.0446846	yes
2.27924	1.56542	-0.542002	-1.47829	0.0012	0.0446846	yes
14.2212	19.1038	0.425817	1.46326	0.00125	0.0456682	yes
40.2215	52.9506	0.39668	1.46379	0.00125	0.0456682	yes
6.25203	8.37207	0.42126	1.40503	0.00125	0.0456682	yes
9.30928	12.8048	0.459947	1.4431	0.00125	0.0456682	yes
9.6868	12.6956	0.390232	1.42458	0.00125	0.0456682	yes
6.52054	9.86443	0.597244	1.49576	0.00125	0.0456682	yes
6.70921	4.79797	-0.483719	-1.4525	0.0013	0.046905	yes
41.039	55.1872	0.427339	1.39481	0.0013	0.046905	yes
6.06532	4.37115	-0.472569	-1.47271	0.0013	0.046905	yes
8.17743	5.92449	-0.464956	-1.46389	0.0013	0.046905	yes
2.21302	3.33287	0.590748	1.4477	0.00135	0.0479642	yes
22.3346	29.1155	0.38251	1.4088	0.00135	0.0479642	yes
46.8823	61.3319	0.387596	1.43845	0.00135	0.0479642	yes
8.44687	11.7999	0.482285	1.41037	0.00135	0.0479642	yes
8.50049	12.6949	0.578637	1.45626	0.0014	0.0489916	yes
22.1767	29.141	0.394005	1.39961	0.0014	0.0489916	yes
5.7477	4.22864	-0.442788	-1.47186	0.0014	0.0489916	yes
6.85771	4.9296	-0.476256	-1.42241	0.0014	0.0489916	yes
36.234	47.0883	0.378022	1.39524	0.0015	0.0515592	no
12.2582	9.02528	-0.441705	-1.41979	0.0015	0.0515592	no
2.32415	1.28043	-0.860069	-1.52131	0.0015	0.0515592	no
10.7512	14.4249	0.424065	1.44154	0.0015	0.0515592	no
15.3361	20.0035	0.383323	1.36799	0.0015	0.0515592	no
87.4363	114.042	0.383255	1.37506	0.0015	0.0515592	no
84.2915	111.589	0.404736	1.43857	0.00155	0.0528091	no
29.7192	38.7216	0.381745	1.42647	0.00155	0.0528091	no
12.2139	8.88789	-0.458606	-1.45281	0.00155	0.0528091	no

201.819	292.626	0.535998	1.51587	0.0016	0.0543532	no
3.85863	6.09176	0.658772	1.44288	0.00165	0.0557259	no
16.3747	12.6006	-0.377977	-1.41062	0.00165	0.0557259	no
0.936657	1.6508	0.81757	1.43733	0.0017	0.056592	no
77.2814	102.555	0.408201	1.42359	0.0017	0.056592	no
23.4216	17.7516	-0.399886	-1.46751	0.0017	0.056592	no
2.15657	3.62801	0.750441	1.46801	0.0017	0.056592	no
17.2505	13.0188	-0.406043	-1.41083	0.0017	0.056592	no
80.2317	105.585	0.396161	1.40531	0.00175	0.0575963	no
25.3124	36.9286	0.544892	1.44413	0.00175	0.0575963	no
19.5202	25.9126	0.408684	1.40985	0.00175	0.0575963	no
37.7788	49.2904	0.383731	1.43223	0.00175	0.0575963	no
10.3392	13.8911	0.426041	1.4278	0.0018	0.0589082	no
23.0006	17.707	-0.377352	-1.41563	0.0018	0.0589082	no
21.7804	28.744	0.400233	1.4096	0.00185	0.0598699	no
4.3064	2.62778	-0.712641	-1.40618	0.00185	0.0598699	no
56.0391	72.6299	0.374129	1.35598	0.00185	0.0598699	no
10.3	13.8439	0.426611	1.38863	0.00185	0.0598699	no
7.48793	5.79805	-0.368999	-1.3561	0.0019	0.0606434	no
22.4825	17.4533	-0.365303	-1.35545	0.0019	0.0606434	no
25.2495	33.3392	0.400964	1.37909	0.0019	0.0606434	no
12.2662	9.33095	-0.394597	-1.38624	0.0019	0.0606434	no
24.4296	18.8056	-0.377472	-1.39253	0.0019	0.0606434	no
0.750494	1.7688	1.23686	1.57897	0.00195	0.0617305	no
9.9181	7.09434	-0.483395	-1.36115	0.00195	0.0617305	no
44.9705	58.2236	0.372623	1.37152	0.00195	0.0617305	no
2.11675	1.13955	-0.893392	-1.43109	0.002	0.0628	no
33.4308	45.6599	0.449753	1.37687	0.002	0.0628	no
15.7383	20.973	0.41425	1.36549	0.002	0.0628	no
339.638	467.41	0.460689	1.36307	0.00205	0.0640239	no
8.93932	11.8509	0.406759	1.37871	0.00205	0.0640239	no
133.543	172.473	0.369067	1.36607	0.0021	0.0642047	no
72.3578	94.0527	0.378321	1.35837	0.0021	0.0642047	no
6.07146	4.40934	-0.461482	-1.38719	0.0021	0.0642047	no
30.2259	44.0267	0.542593	1.41212	0.0021	0.0642047	no
77.7611	100.994	0.377145	1.40946	0.0021	0.0642047	no
13.2227	10.1872	-0.376261	-1.37489	0.0021	0.0642047	no
16.1909	12.2747	-0.399497	-1.39724	0.0021	0.0642047	no
7.43333	5.53942	-0.424274	-1.40403	0.0021	0.0642047	no
7.72939	11.6736	0.594821	1.38123	0.00215	0.0647117	no
0.39705	1.53036	1.94648	1.7319	0.00215	0.0647117	no
25.2294	19.5566	-0.367449	-1.36255	0.00215	0.0647117	no
4.96973	3.75914	-0.402764	-1.37197	0.00215	0.0647117	no
67.9871	89.1739	0.391359	1.37813	0.00215	0.0647117	no
12.7438	18.3198	0.523608	1.42269	0.00215	0.0647117	no
20.2569	15.7228	-0.365556	-1.36772	0.0022	0.0655374	no
99.0968	127.975	0.368951	1.36833	0.0022	0.0655374	no

391.783	524.852	0.421856	1.38174	0.0022	0.0655374	no
12.2282	20.3449	0.734456	1.37207	0.0022	0.0655374	no
2.74479	2.01622	-0.445043	-1.33602	0.00225	0.0668555	no
8.381	5.96693	-0.490134	-1.36137	0.0023	0.0679934	no
6.27524	4.2021	-0.578559	-1.3529	0.0023	0.0679934	no
27.5558	21.2039	-0.378022	-1.35058	0.00235	0.0685987	no
15.3845	11.8749	-0.373562	-1.30322	0.00235	0.0685987	no
22.6194	29.2336	0.370068	1.37873	0.00235	0.0685987	no
4.86737	3.51715	-0.468733	-1.35947	0.00235	0.0685987	no
106.578	137.652	0.369114	1.36321	0.00235	0.0685987	no
3.24675	2.33008	-0.478615	-1.37288	0.0024	0.0691891	no
27.0527	21.0488	-0.362037	-1.35068	0.0024	0.0691891	no
4.74777	7.62519	0.683523	1.3971	0.0024	0.0691891	no
4.15863	6.75595	0.700052	1.36002	0.0024	0.0691891	no
34.1969	26.1221	-0.388593	-1.36032	0.0024	0.0691891	no
66.2979	51.3373	-0.368957	-1.36211	0.00245	0.0699364	no
37.3991	28.4732	-0.393401	-1.36109	0.00245	0.0699364	no
14.0505	10.6056	-0.405795	-1.35466	0.00245	0.0699364	no
241.262	332.559	0.46301	1.34686	0.00245	0.0699364	no
1.80345	3.1023	0.782577	1.417	0.0025	0.0708415	no
18.1732	23.7478	0.385985	1.33865	0.0025	0.0708415	no
12.2027	16.9528	0.474323	1.39404	0.00255	0.0715601	no
2.16874	1.41027	-0.620886	-1.33993	0.00255	0.0715601	no
58.0069	44.765	-0.373853	-1.35345	0.00255	0.0715601	no
56.0673	72.2639	0.366114	1.3641	0.00255	0.0715601	no
5.17544	2.92928	-0.821136	-1.42113	0.0026	0.0726125	no
45.0065	57.7065	0.358601	1.30519	0.0026	0.0726125	no
1.04944	2.00813	0.936236	1.391	0.0027	0.0748654	no
73.169	93.7675	0.357857	1.33729	0.0027	0.0748654	no
1.46551	3.50983	1.26	1.41897	0.0027	0.0748654	no
7.53441	5.85225	-0.364503	-1.3245	0.00275	0.0758895	no
5.41336	4.16521	-0.378135	-1.34875	0.00275	0.0758896	no
1.26678	2.14706	0.761193	1.3543	0.0028	0.0770863	no
36.2229	59.4088	0.713773	1.36755	0.00285	0.0779089	no
3.58479	2.38062	-0.590551	-1.34672	0.00285	0.0779089	no
10.9562	7.9606	-0.460803	-1.34548	0.00285	0.0779089	no
10.9231	8.35883	-0.386013	-1.31803	0.0029	0.0785366	no
16.0681	12.1302	-0.405589	-1.31605	0.0029	0.0785366	no
6.4504	4.31077	-0.581443	-1.3452	0.0029	0.0785366	no
11.5355	15.6833	0.443151	1.33947	0.00295	0.07952	no
69.4332	53.552	-0.374685	-1.30629	0.00295	0.07952	no
8.1895	6.17316	-0.407766	-1.35984	0.003	0.0804942	no
236.825	182.651	-0.374728	-1.3135	0.003	0.0804942	no
1.89531	3.34774	0.820752	1.33792	0.00305	0.0816472	no
12.7654	19.5388	0.6141	1.35813	0.0031	0.0827949	no
27.7426	35.5524	0.357844	1.31076	0.00315	0.0839374	no
1.75967	3.26736	0.892819	1.42147	0.0032	0.0844945	no

5.03779	3.653	-0.463706	-1.35105	0.0032	0.0844945	no
870.282	554.13	-0.651259	-1.3538	0.0032	0.0844945	no
20.0056	25.8532	0.369941	1.30885	0.0032	0.0844945	no
88.351	114.399	0.37276	1.30616	0.00325	0.0856202	no
8.35929	6.13349	-0.446674	-1.30814	0.0033	0.0867407	no
5.932	4.51147	-0.394921	-1.28305	0.00335	0.0876583	no
6.81906	4.89918	-0.477033	-1.29589	0.00335	0.0876583	no
4.50646	2.87964	-0.646104	-1.28676	0.0034	0.0887667	no
61.2138	78.2724	0.354647	1.32607	0.00345	0.0896691	no
13.8102	18.3338	0.40877	1.30226	0.00345	0.0896691	no
2.7814	1.91747	-0.536609	-1.31561	0.0035	0.0903622	no
10.8319	8.45875	-0.356775	-1.2972	0.0035	0.0903622	no
39.8787	31.0893	-0.3592	-1.28826	0.0035	0.0903622	no
68.6238	53.3733	-0.362591	-1.29006	0.00355	0.0912476	no
4.45869	3.48588	-0.355096	-1.28177	0.00355	0.0912476	no
9.29019	6.92376	-0.424153	-1.27787	0.0036	0.0923285	no
6.74348	5.14817	-0.389434	-1.28808	0.00365	0.092995	no
42.4484	33.0845	-0.359552	-1.31306	0.00365	0.092995	no
34.9128	44.3367	0.344744	1.28023	0.00365	0.092995	no
230.442	297.772	0.369805	1.29885	0.00375	0.0953337	no
17.3611	22.9299	0.401369	1.27152	0.0038	0.0963939	no
12.8915	17.2862	0.423208	1.30029	0.00385	0.0972376	no
6.82343	4.99378	-0.450365	-1.30693	0.00385	0.0972376	no
15.0658	19.1037	0.342573	1.26974	0.0039	0.0976513	no
11.2236	8.64569	-0.376486	-1.2984	0.0039	0.0976513	no
3.18578	2.34088	-0.444597	-1.30177	0.0039	0.0976513	no
4.08103	6.19739	0.602726	1.27846	0.0039	0.0976513	no
2.79895	1.96474	-0.510545	-1.29498	0.00395	0.0980579	no
7.93025	6.18798	-0.357898	-1.27892	0.00395	0.0980579	no
21.9046	27.947	0.351462	1.28103	0.00395	0.0980579	no
11.4566	8.78813	-0.382545	-1.28437	0.00395	0.0980579	no
20.2043	15.9532	-0.340813	-1.26822	0.004	0.0984576	no
2.22923	1.49335	-0.577998	-1.31854	0.004	0.0984576	no
76.5164	59.7866	-0.355946	-1.30722	0.004	0.0984576	no
9.26608	6.8868	-0.428125	-1.28238	0.00405	0.0988506	no
97.0356	123.79	0.351307	1.29564	0.00405	0.0988506	no
1.97637	0.961997	-1.03875	-1.37261	0.00405	0.0988506	no
7.11373	5.0773	-0.486544	-1.30264	0.00405	0.0988506	no
14.7098	19.1742	0.382391	1.28941	0.0041	0.0992371	no
32.2165	41.1322	0.35247	1.27643	0.0041	0.0992371	no
0.981708	2.17587	1.14823	1.35611	0.0041	0.0992371	no
30.5882	24.0862	-0.344769	-1.2648	0.0041	0.0992371	no
9.09515	7.02282	-0.373048	-1.26643	0.00415	0.100238	no
12.6653	9.64916	-0.392402	-1.26851	0.0042	0.101236	no
6.74701	5.16932	-0.384275	-1.26459	0.00425	0.101807	no
6.41221	4.78573	-0.422083	-1.32276	0.00425	0.101807	no
0.916015	1.90055	1.05297	1.31827	0.00425	0.101807	no

11.125	8.68511	-0.357188	-1.27788	0.0043	0.102793	no
25.4989	32.652	0.356738	1.25329	0.00435	0.103562	no
6.95488	9.4656	0.444668	1.28905	0.00435	0.103562	no
38.5653	48.7489	0.338068	1.26254	0.0044	0.104112	no
45.8659	35.7286	-0.360341	-1.27707	0.0044	0.104112	no
23.9638	30.6866	0.356752	1.28971	0.00445	0.104656	no
9.40863	13.8271	0.555445	1.30306	0.00445	0.104656	no
1.56278	0.816836	-0.935998	-1.35789	0.00445	0.104656	no
35.3761	27.8706	-0.344032	-1.27516	0.0045	0.104772	no
36.6136	46.4405	0.343005	1.28004	0.0045	0.104772	no
31.3604	24.7532	-0.34133	-1.26338	0.0045	0.104772	no
38.5969	49.0376	0.345402	1.29184	0.0045	0.104772	no
2.09457	2.99057	0.513768	1.28919	0.0045	0.104772	no
15.9114	12.1744	-0.386215	-1.27287	0.00455	0.105513	no
18.7756	24.2795	0.370886	1.24226	0.00455	0.105513	no
5.05622	6.89298	0.447068	1.27256	0.0046	0.106037	no
7.59596	5.35139	-0.505318	-1.29851	0.0046	0.106037	no
29.0637	22.4466	-0.372722	-1.27643	0.0046	0.106037	no
1.00157	2.11991	1.08174	1.34842	0.00465	0.106978	no
9.68221	6.82491	-0.504526	-1.29356	0.0047	0.107701	no
27.5497	21.6234	-0.349447	-1.29981	0.0047	0.107701	no
14.8416	11.609	-0.354398	-1.2806	0.00475	0.108419	no
9.4357	12.4004	0.394181	1.27155	0.00475	0.108419	no
2.64075	1.93229	-0.450635	-1.26688	0.00485	0.110269	no
4.87663	3.69968	-0.398483	-1.25347	0.00485	0.110269	no
2.26216	1.62207	-0.479867	-1.2954	0.0049	0.111188	no
53.7263	68.3171	0.346619	1.27134	0.00495	0.111885	no
3.98145	2.50991	-0.665661	-1.2557	0.00495	0.111885	no
5.17872	3.83504	-0.433354	-1.2735	0.005	0.11236	no
1886.62	1382.83	-0.448183	-1.23274	0.005	0.11236	no
5.13998	7.86815	0.614262	1.2516	0.005	0.11236	no
10.678	8.47327	-0.333654	-1.23339	0.00505	0.112829	no
28.2585	35.7881	0.340799	1.26318	0.00505	0.112829	no
19.1374	15.1884	-0.333427	-1.24079	0.00505	0.112829	no
6.34782	4.85061	-0.388097	-1.27856	0.0051	0.113727	no
11.415	8.93499	-0.353398	-1.24547	0.00515	0.114622	no
57.4276	74.6522	0.37844	1.23951	0.0052	0.115293	no
8.76518	6.70966	-0.385545	-1.24441	0.0052	0.115293	no
11.329	14.9693	0.401992	1.2488	0.00525	0.11618	no
2.87286	2.1306	-0.431224	-1.23491	0.0053	0.1164	no
1.41851	2.25757	0.670401	1.27375	0.0053	0.1164	no
3.93203	2.42898	-0.694926	-1.30421	0.0053	0.1164	no
37.6754	29.805	-0.338067	-1.21709	0.0054	0.118149	no
69.2904	87.1329	0.330563	1.23668	0.0054	0.118149	no
2.44732	1.64853	-0.570028	-1.24704	0.00545	0.119019	no
11.7316	15.1347	0.367465	1.23504	0.0055	0.119886	no
22.3649	29.0782	0.378699	1.23086	0.00555	0.120523	no

11.6838	15.7549	0.43129	1.25006	0.00555	0.120523	no
5.72859	8.30263	0.535388	1.2632	0.0056	0.121382	no
18.7869	25.8287	0.459247	1.2415	0.0057	0.122182	no
246.628	321.685	0.383312	1.22259	0.0057	0.122182	no
23.4152	18.4763	-0.341774	-1.22484	0.0057	0.122182	no
39.6413	29.8	-0.41169	-1.25813	0.0057	0.122182	no
49.9511	62.8624	0.331681	1.24017	0.00565	0.122182	no
3.94498	2.78603	-0.501805	-1.23189	0.00575	0.123027	no
19.9678	27.0451	0.437694	1.23438	0.0058	0.123641	no
9.61481	5.59262	-0.781734	-1.24514	0.0058	0.123641	no
1.56922	1.06765	-0.555606	-1.25786	0.00585	0.124024	no
19.9243	15.7998	-0.334619	-1.22764	0.00585	0.124024	no
15.3391	19.4166	0.340074	1.23358	0.00585	0.124024	no
6.39654	4.7895	-0.417416	-1.24249	0.0059	0.124856	no
4.63095	3.40011	-0.44573	-1.249	0.006	0.126742	no
33.4619	42.4915	0.344655	1.23242	0.0061	0.12862	no
70.1611	91.2319	0.378867	1.22135	0.00615	0.129206	no
84.8773	107.16	0.336321	1.23354	0.00615	0.129206	no
48.9102	38.832	-0.332889	-1.22006	0.0062	0.130021	no
202.227	262.808	0.378029	1.21615	0.00625	0.130364	no
78.1466	62.5372	-0.321469	-1.1843	0.00625	0.130364	no
20.0212	25.0575	0.323714	1.2029	0.00625	0.130364	no
10.9718	8.69658	-0.335282	-1.23486	0.00635	0.132212	no
27.1172	19.7104	-0.460251	-1.20751	0.0065	0.135093	no
17.6948	22.6	0.352996	1.20343	0.00655	0.135406	no
13.689	17.4829	0.352926	1.22344	0.00655	0.135406	no
18.8138	14.7357	-0.352469	-1.20643	0.00655	0.135406	no
7.11891	11.922	0.743902	1.22052	0.0066	0.135475	no
22.898	28.6672	0.324182	1.19469	0.0066	0.135475	no
6.96649	9.31172	0.418615	1.22608	0.0066	0.135475	no
6.39957	4.77781	-0.421627	-1.23499	0.0066	0.135475	no
45.114	35.6084	-0.341358	-1.23515	0.00665	0.136021	no
6.1335	8.53804	0.477195	1.21428	0.00665	0.136021	no
9.25876	12.1142	0.387807	1.17708	0.0067	0.136802	no
26.3429	33.002	0.325142	1.21069	0.0068	0.137157	no
8.48447	6.6431	-0.352968	-1.21107	0.0068	0.137157	no
20.2971	16.2352	-0.322145	-1.19516	0.0068	0.137157	no
11.7932	9.42364	-0.323597	-1.19988	0.0068	0.137157	no
1.75478	1.26259	-0.474911	-1.21816	0.00675	0.137157	no
52.7922	66.1023	0.324376	1.20708	0.0068	0.137157	no
1.74243	3.06183	0.813295	1.26637	0.0068	0.137157	no
223.555	280.524	0.327495	1.20431	0.0069	0.138692	no
17.851	13.9746	-0.353197	-1.21063	0.0069	0.138692	no
46.1538	58.9479	0.352992	1.22405	0.00695	0.138737	no
65.5846	51.8616	-0.338691	-1.19942	0.00695	0.138737	no
21.5987	17.2649	-0.323106	-1.19769	0.00695	0.138737	no
27.3393	34.3911	0.331056	1.20175	0.00695	0.138737	no

4.14761	2.88727	-0.522572	-1.21751	0.00705	0.140492	no
2.7538	1.72124	-0.677974	-1.2213	0.0071	0.140764	no
71.5891	101.958	0.510169	1.21874	0.0071	0.140764	no
4.39427	3.36792	-0.383763	-1.20503	0.0071	0.140764	no
15.4403	12.339	-0.323469	-1.20376	0.00715	0.141514	no
69.9065	87.606	0.325602	1.21457	0.00725	0.143249	no
17.3772	13.8927	-0.322867	-1.19557	0.0073	0.143505	no
42.5082	54.5203	0.359053	1.20477	0.0073	0.143505	no
24.5276	19.5954	-0.323891	-1.18891	0.0073	0.143505	no
9.15062	7.18274	-0.349335	-1.20084	0.00735	0.144001	no
224.686	178.41	-0.332717	-1.19483	0.00735	0.144001	no
7.71319	5.55112	-0.474549	-1.18968	0.0074	0.144493	no
12.8892	9.84592	-0.388565	-1.21531	0.0074	0.144493	no
60.1797	75.6195	0.329483	1.21275	0.0075	0.145225	no
12.01	9.43872	-0.347569	-1.18767	0.0075	0.145225	no
13.1534	7.75283	-0.762642	-1.2379	0.0075	0.145225	no
9.68377	7.75631	-0.320197	-1.17707	0.0075	0.145225	no
12.5176	9.99417	-0.324795	-1.18416	0.0075	0.145225	no
28.7801	36.073	0.325847	1.19232	0.00755	0.145707	no
6.50754	5.00156	-0.379734	-1.19967	0.00755	0.145707	no
8.69165	6.0532	-0.521933	-1.20635	0.0076	0.146429	no
20.6888	26.069	0.333484	1.17984	0.00765	0.147149	no
110.888	138.586	0.321674	1.19058	0.0077	0.147621	no
58.5978	73.2545	0.322069	1.17456	0.0077	0.147621	no
28.1605	35.7613	0.344726	1.19967	0.0079	0.150463	no
6.99159	10.1315	0.535161	1.18735	0.0079	0.150463	no
95.5649	123.224	0.366726	1.19346	0.0079	0.150463	no
12.5889	9.97468	-0.335808	-1.17017	0.0079	0.150463	no
9.35457	12.0007	0.359376	1.16001	0.008	0.151128	no
6.43542	8.73568	0.440887	1.20036	0.008	0.151128	no
13.9496	11.0216	-0.339882	-1.18686	0.008	0.151128	no
17.9999	14.2922	-0.332763	-1.18164	0.008	0.151128	no
9.71374	7.67713	-0.33946	-1.17958	0.00795	0.151128	no
11.4262	14.512	0.344895	1.18074	0.00805	0.15158	no
30.5979	38.3717	0.326608	1.18099	0.00805	0.15158	no
9.66478	7.07347	-0.45032	-1.17648	0.0081	0.152275	no
17.4866	22.3403	0.353401	1.18688	0.0082	0.153906	no
5.97654	7.88387	0.399595	1.16846	0.00825	0.154594	no
4.83708	3.74288	-0.369988	-1.16794	0.0083	0.155031	no
137.737	108.828	-0.339868	-1.18394	0.0083	0.155031	no
3.20607	5.12256	0.676062	1.19027	0.00835	0.155216	no
17.3405	13.4222	-0.369527	-1.18328	0.00835	0.155216	no
4.57957	3.47489	-0.398246	-1.1746	0.00835	0.155216	no
8.55371	11.2084	0.389953	1.16775	0.0084	0.155648	no
12.7625	16.4593	0.366991	1.17398	0.0084	0.155648	no
30.4927	37.8709	0.312626	1.16986	0.00845	0.156076	no
2.9394	3.96392	0.431409	1.20217	0.00845	0.156076	no

22.8465	18.0806	-0.33753	-1.17733	0.00855	0.156678	no
9.92958	7.87032	-0.335311	-1.16985	0.00855	0.156678	no
6.34597	4.18836	-0.599455	-1.18819	0.0085	0.156678	no
16.4298	20.7036	0.333569	1.1792	0.00855	0.156678	no
44.5578	55.3255	0.312266	1.16644	0.00855	0.156678	no
19.3902	15.3644	-0.33574	-1.15205	0.0086	0.156852	no
2.97886	2.19689	-0.439299	-1.1851	0.0086	0.156852	no
53.3046	67.4682	0.339948	1.14727	0.0086	0.156852	no
7.86796	5.998	-0.391508	-1.17442	0.00865	0.15727	no
36.0286	44.8111	0.314712	1.17533	0.00865	0.15727	no
1.03914	2.03682	0.970931	1.20105	0.0087	0.157932	no
31.3404	38.8926	0.311473	1.14853	0.0088	0.15925	no
1.43511	2.13188	0.570966	1.16746	0.0088	0.15925	no
4.88783	3.4371	-0.508005	-1.17628	0.0089	0.160809	no
1.73593	3.22993	0.895796	1.22576	0.00895	0.161461	no
6.05605	4.32091	-0.487041	-1.17732	0.009	0.161861	no
28.9879	37.5082	0.371756	1.1508	0.009	0.161861	no
8.65028	11.4581	0.405543	1.17434	0.00905	0.162008	no
12.4529	15.7095	0.335165	1.15183	0.00905	0.162008	no
2.85697	1.87818	-0.605156	-1.16078	0.00905	0.162008	no
119.941	153.71	0.35789	1.17406	0.0091	0.162652	no
52.363	39.4701	-0.40779	-1.17874	0.00915	0.163294	no
7.98104	6.03776	-0.402564	-1.17596	0.0092	0.163434	no
0.852584	1.58392	0.893586	1.17831	0.0092	0.163434	no
343.258	428.894	0.321329	1.14539	0.0092	0.163434	no
4.09327	3.19468	-0.357585	-1.14347	0.00925	0.163821	no
0.393469	1.54737	1.9755	1.44934	0.00925	0.163821	no
38.4934	47.9025	0.315491	1.15313	0.0093	0.163957	no
17.0694	13.6898	-0.318306	-1.16142	0.0093	0.163957	no
29.8485	37.0331	0.311159	1.15059	0.0093	0.163957	no
1.75236	1.21664	-0.526397	-1.15353	0.00945	0.166097	no
16.5494	13.3792	-0.306785	-1.14446	0.00945	0.166097	no
12.7902	10.3124	-0.310663	-1.15083	0.00955	0.167601	no
14.561	11.2794	-0.36841	-1.15229	0.0096	0.168224	no
6.30591	9.30161	0.560776	1.1602	0.00965	0.168592	no
3.42509	2.51292	-0.446776	-1.1496	0.00965	0.168592	no
5.85043	4.09299	-0.515388	-1.17361	0.00975	0.170083	no
18.1063	23.4026	0.370175	1.12671	0.0098	0.170444	no
0.995435	1.72338	0.791841	1.18044	0.0098	0.170444	no
8.63888	10.8778	0.332475	1.14897	0.00985	0.171057	no
4.51082	3.47454	-0.376567	-1.14105	0.0099	0.171669	no
83.5487	67.167	-0.314865	-1.15471	0.00995	0.171767	no
7.53494	9.43066	0.323763	1.15244	0.00995	0.171767	no
7.16326	5.44803	-0.394882	-1.16886	0.00995	0.171767	no
9.72267	13.3693	0.459495	1.1612	0.01	0.172119	no
32.9486	26.3184	-0.324142	-1.16016	0.01	0.172119	no
4.75906	3.41203	-0.480046	-1.17546	0.0101	0.173583	no

11.6365	9.22707	-0.334717	-1.15317	0.01025	0.175641	no
15.7802	12.7423	-0.308486	-1.14343	0.0103	0.176238	no
7.93724	5.98799	-0.406565	-1.15769	0.01035	0.176573	no
3.86923	2.90746	-0.412285	-1.14013	0.0104	0.176907	no
5.40801	6.75467	0.320788	1.12031	0.0104	0.176907	no
7.38723	5.88467	-0.328073	-1.13656	0.01045	0.17698	no
1.46272	3.17033	1.11598	1.195	0.01045	0.17698	no
7.52642	5.63708	-0.417016	-1.14573	0.01045	0.17698	no
0.700528	1.44998	1.04952	1.16776	0.0105	0.177052	no
3.02823	1.6252	-0.897851	-1.26694	0.0105	0.177052	no
0.732136	1.45754	0.993356	1.21388	0.0105	0.177052	no
16.652	13.5247	-0.300098	-1.12282	0.01055	0.17738	no
26.8421	34.5101	0.36252	1.13521	0.01055	0.17738	no
111.356	141.599	0.346624	1.13402	0.01075	0.180221	no
89.8951	111.416	0.309637	1.12625	0.01075	0.180222	no
48.9252	39.6403	-0.30361	-1.13257	0.01085	0.181375	no
12.3564	16.7509	0.438985	1.16577	0.01085	0.181375	no
13.1219	9.75919	-0.42714	-1.13288	0.01095	0.182521	no
2.4383	1.63594	-0.575753	-1.14416	0.01095	0.182521	no
1284.71	1683.59	0.390102	1.13426	0.011	0.182569	no
36.3001	28.9241	-0.327701	-1.13629	0.011	0.182569	no
231.713	288.027	0.313864	1.14722	0.011	0.182569	no
69.2421	55.1855	-0.32736	-1.13395	0.01105	0.182616	no
61.677	76.0978	0.303121	1.13782	0.01105	0.182616	no
7.85643	10.1505	0.369607	1.13189	0.01105	0.182616	no
11.8962	15.2706	0.360252	1.12678	0.0111	0.182922	no
2.44284	1.44566	-0.756826	-1.18196	0.0111	0.182922	no
21.6553	17.5819	-0.300626	-1.12535	0.01115	0.183226	no
392.738	316.532	-0.311218	-1.12844	0.01115	0.183226	no
303.632	245.291	-0.307834	-1.13975	0.0112	0.183528	no
13.6466	10.883	-0.326461	-1.13114	0.0112	0.183528	no
3.32923	2.3157	-0.52374	-1.11881	0.01125	0.183829	no
1.91132	1.41944	-0.429242	-1.1407	0.01125	0.183829	no
3.77319	4.78152	0.341687	1.11243	0.0113	0.184128	no
18.8677	13.9772	-0.432848	-1.14511	0.0113	0.184128	no
8.16296	6.43101	-0.344048	-1.12103	0.01135	0.184426	no
13.7032	11.095	-0.304602	-1.13161	0.01135	0.184426	no
17.5069	14.057	-0.316638	-1.12171	0.0114	0.184721	no
6.41241	4.75415	-0.431679	-1.1466	0.0114	0.184721	no
4.43931	7.65912	0.786843	1.16704	0.01145	0.185273	no
174.732	239.231	0.453264	1.12954	0.0115	0.185565	no
29.7272	23.7019	-0.326781	-1.12835	0.0115	0.185565	no
2.32184	3.78071	0.703389	1.1248	0.01155	0.185856	no
15.2702	18.8468	0.303596	1.13393	0.01155	0.185856	no
6.17073	4.92189	-0.326228	-1.1056	0.01165	0.187206	no
6.24614	4.92939	-0.341554	-1.12969	0.01175	0.188552	no
6.15446	4.75235	-0.372991	-1.13233	0.0118	0.188573	no

5.70319	9.03104	0.663124	1.20274	0.0118	0.188573	no
2.11888	1.35742	-0.64244	-1.12792	0.0118	0.188573	no
17.3183	21.9855	0.344257	1.15198	0.01185	0.189112	no
1.49752	2.32694	0.635862	1.14796	0.0119	0.189389	no
3.49045	5.52494	0.662548	1.13063	0.0119	0.189389	no
18.3257	22.6074	0.302924	1.12466	0.01195	0.189407	no
7.71676	6.09092	-0.341335	-1.11839	0.01195	0.189407	no
210.68	292.535	0.47356	1.15999	0.012	0.18994	no
24.0464	30.0034	0.319306	1.12404	0.01205	0.190213	no
10.8287	13.5147	0.319672	1.10684	0.01205	0.190213	no
10.2561	8.33687	-0.298911	-1.11445	0.01215	0.191531	no
1.0907	1.77281	0.700782	1.15564	0.0122	0.191799	no
46.7502	58.3982	0.320951	1.12847	0.0122	0.191799	no
12.8664	17.7302	0.462608	1.1215	0.01225	0.192325	no
7.57782	6.11557	-0.309297	-1.12321	0.0123	0.192849	no
7.56072	5.93768	-0.348625	-1.1017	0.01235	0.193372	no
61.5138	76.8993	0.32206	1.11171	0.0124	0.193633	no
5.60989	4.358	-0.364307	-1.10745	0.0124	0.193633	no
2.66757	1.80508	-0.56346	-1.12357	0.0125	0.194933	no
5.48955	4.20353	-0.385086	-1.10157	0.01255	0.195189	no
6.68881	9.00401	0.42882	1.11242	0.01255	0.195189	no
8.66635	5.99423	-0.531851	-1.1375	0.0126	0.195704	no
17.6674	14.2722	-0.307888	-1.11232	0.01265	0.195957	no
1.47772	0.962342	-0.618747	-1.12232	0.01265	0.195957	no
1.10505	1.74093	0.655745	1.10102	0.0127	0.19647	no
10.1856	8.17676	-0.316929	-1.12848	0.0128	0.197753	no
8.66925	7.08157	-0.291839	-1.07781	0.0129	0.197982	no
66.0919	81.7406	0.306581	1.11499	0.0129	0.197982	no
5.92524	7.57313	0.354015	1.10019	0.01285	0.197982	no
27.999	36.0366	0.364085	1.09464	0.0129	0.197982	no
17.7537	11.6488	-0.60794	-1.13683	0.01285	0.197982	no
15.1332	12.2421	-0.305867	-1.10714	0.01295	0.198487	no
56.1403	68.9147	0.295775	1.10057	0.013	0.198991	no
150.091	186.058	0.309911	1.11718	0.01315	0.201022	no
89.1604	109.631	0.298176	1.10661	0.01335	0.201954	no
3.03051	2.401	-0.335928	-1.10666	0.01335	0.201954	no
1.63571	2.6632	0.70324	1.12333	0.0133	0.201954	no
120.035	152.09	0.341464	1.09984	0.0133	0.201954	no
30.7045	37.6463	0.294055	1.09816	0.01335	0.201954	no
8.60127	6.7635	-0.346781	-1.11281	0.0133	0.201954	no
11.2122	14.7617	0.396784	1.11678	0.0133	0.201954	no
17.5232	21.9125	0.322489	1.08349	0.0133	0.201954	no
1.50817	2.21784	0.55635	1.11542	0.0134	0.202183	no
15.0408	12.1886	-0.303348	-1.11111	0.0134	0.202183	no
13.8958	17.6476	0.344829	1.09303	0.01345	0.202412	no
22.2285	29.8731	0.426438	1.11731	0.01345	0.202412	no
204.768	253.631	0.308741	1.10746	0.0135	0.202902	no

23.4344	30.0871	0.360518	1.11018	0.0136	0.204141	no
6.49732	5.06784	-0.358475	-1.08044	0.01365	0.204627	no
6.66952	9.2818	0.476822	1.10505	0.0137	0.205112	no
5.86934	4.74392	-0.307118	-1.08757	0.01385	0.20709	no
1.21853	2.04656	0.748064	1.132	0.014	0.208796	no
30.124	24.5906	-0.292806	-1.09315	0.014	0.208796	no
5.3298	4.31084	-0.306113	-1.08518	0.01415	0.209154	no
9.12955	7.22886	-0.336777	-1.06683	0.0141	0.209154	no
46.8247	57.524	0.296894	1.09662	0.01415	0.209154	no
2.1451	1.35387	-0.663956	-1.11898	0.01415	0.209154	no
7.02562	10.0965	0.523153	1.07502	0.0141	0.209154	no
81.9167	101.297	0.306368	1.09135	0.01415	0.209154	no
27.0783	34.176	0.335846	1.08739	0.0141	0.209154	no
30.3363	24.7835	-0.291664	-1.09187	0.0142	0.209626	no
72.649	89.1235	0.294864	1.10501	0.0143	0.210567	no
400.37	515.613	0.364954	1.09386	0.0143	0.210567	no
172.868	139.946	-0.304794	-1.08634	0.01435	0.211036	no
40.4824	57.7208	0.511796	1.11443	0.0144	0.211236	no
16.3197	20.9685	0.361606	1.10695	0.0144	0.211236	no
27.0272	22.0357	-0.294569	-1.10035	0.01445	0.211703	no
42.0878	51.8624	0.301287	1.09584	0.0145	0.212168	no
16.1806	13.2054	-0.29314	-1.08602	0.01455	0.212631	no
16.1438	19.8407	0.297482	1.08683	0.01465	0.213748	no
1.38085	2.32254	0.750148	1.11632	0.0147	0.213748	no
50.632	62.5064	0.303954	1.08105	0.0147	0.213748	no
6.99224	5.63799	-0.310572	-1.08835	0.0147	0.213748	no
10.0176	12.7096	0.343376	1.08021	0.01485	0.21539	no
14.5877	17.907	0.295774	1.10213	0.01485	0.21539	no
15.1242	12.2316	-0.306241	-1.09163	0.01495	0.215681	no
30.9421	42.8456	0.469576	1.09454	0.0149	0.215681	no
10.3652	14.4919	0.483504	1.07385	0.015	0.215681	no
12.5105	15.7552	0.332686	1.09576	0.01495	0.215681	no
3.9993	2.79216	-0.518366	-1.08342	0.015	0.215681	no
2.63686	3.81399	0.532479	1.09424	0.015	0.215681	no
12.1491	9.80237	-0.309647	-1.07032	0.01495	0.215681	no
302.687	372.126	0.297964	1.08744	0.0151	0.216049	no
7.2894	5.3114	-0.456707	-1.09158	0.0151	0.216049	no
1.69964	0.94868	-0.841235	-1.11189	0.0151	0.216049	no
3.88253	2.78561	-0.479006	-1.11805	0.01505	0.216049	no
23.0023	29.3043	0.349331	1.06119	0.0152	0.217212	no
3.02196	6.25081	1.04856	1.14534	0.01525	0.217392	no
243.86	332.604	0.447754	1.08723	0.01525	0.217392	no
36.2021	29.6213	-0.28944	-1.07979	0.0154	0.219261	no
2.83895	1.79486	-0.661483	-1.08399	0.0155	0.220415	no
0.984256	2.16165	1.13503	1.15854	0.0156	0.220488	no
31.8411	41.7113	0.389547	1.08485	0.0156	0.220488	no
6.47145	8.84397	0.450605	1.08873	0.0156	0.220488	no

8.79216	7.13804	-0.300689	-1.08164	0.01555	0.220488	no
4.71184	3.8146	-0.304759	-1.0931	0.01555	0.220488	no
17.8569	22.1863	0.313184	1.06374	0.01565	0.220657	no
3.93403	2.8959	-0.441997	-1.08259	0.01565	0.220657	no
5.95416	4.78515	-0.315333	-1.08058	0.0157	0.221094	no
73.7686	91.151	0.305252	1.07099	0.01575	0.22153	no
11.0321	9.00868	-0.292316	-1.05976	0.01585	0.222398	no
26.0233	20.8472	-0.319953	-1.06087	0.01585	0.222398	no
54.5925	44.6855	-0.288897	-1.07752	0.01595	0.222457	no
0.698358	1.64956	1.24004	1.14826	0.01595	0.222457	no
24.1181	29.8893	0.309515	1.07693	0.0159	0.222457	no
88.3905	107.921	0.288017	1.07711	0.0159	0.222457	no
1.48859	1.06319	-0.485557	-1.08315	0.01595	0.222458	no
20.0198	24.5269	0.292937	1.07487	0.016	0.222887	no
7.95663	6.41035	-0.311756	-1.07876	0.01605	0.223049	no
17.0149	14.0045	-0.280907	-1.04383	0.0161	0.22321	no
14.5969	11.9037	-0.294255	-1.05522	0.0161	0.22321	no
4.78647	6.99261	0.546871	1.0576	0.0163	0.223739	no
36.2325	44.8391	0.307472	1.06375	0.01635	0.223739	no
15.0348	12.0715	-0.316697	-1.0677	0.0163	0.223739	no
41.2174	50.9872	0.306881	1.08041	0.0162	0.223739	no
4.62208	5.77279	0.320726	1.06588	0.01635	0.223739	no
2.17043	2.98246	0.458524	1.08247	0.0163	0.223739	no
5.00399	9.29894	0.893987	1.09582	0.01625	0.223739	no
6.45387	4.90219	-0.396738	-1.08146	0.0162	0.223739	no
7.96106	6.30985	-0.335355	-1.05192	0.0163	0.223739	no
6.72167	5.18684	-0.373964	-1.0731	0.01635	0.223739	no
11.9702	9.72082	-0.300292	-1.0741	0.01645	0.224578	no
47.7631	58.3429	0.288659	1.0715	0.01645	0.224578	no
10.6938	13.2782	0.31228	1.0611	0.0165	0.224777	no
14.4795	19.5028	0.429671	1.07312	0.0166	0.224777	no
13.0179	17.5596	0.431766	1.07915	0.01655	0.224777	no
9.43528	12.1643	0.366522	1.07311	0.01655	0.224777	no
45.6513	37.1453	-0.297475	-1.05418	0.0166	0.224777	no
12.9324	10.3137	-0.32642	-1.07849	0.0166	0.224777	no
8.38037	6.63343	-0.337259	-1.05791	0.0166	0.224777	no
5.3252	3.69686	-0.526537	-1.07217	0.0167	0.224821	no
15.8498	20.4763	0.369486	1.09141	0.0167	0.224821	no
11.0122	13.6537	0.310186	1.03303	0.0167	0.224821	no
34.0761	41.8671	0.297058	1.06771	0.01665	0.224821	no
17.2143	13.7815	-0.320876	-1.03691	0.0168	0.225124	no
8.21915	10.7259	0.384039	1.06944	0.01675	0.225124	no
50.2493	40.7446	-0.302496	-1.05548	0.0168	0.225124	no
10.9612	8.15498	-0.426651	-1.07422	0.0168	0.225124	no
9.05281	7.07006	-0.356644	-1.05389	0.017	0.22728	no
16.2484	13.3401	-0.284526	-1.05423	0.017	0.22728	no
7.3092	5.79912	-0.333879	-1.06654	0.01705	0.227425	no

95.1931	123.386	0.374249	1.06795	0.01705	0.227425	no
35.4654	28.4637	-0.317286	-1.0649	0.0171	0.22783	no
8.69314	6.99883	-0.312763	-1.05681	0.01715	0.227973	no
81.1833	99.0716	0.287288	1.06631	0.01715	0.227973	no
6.1939	4.84692	-0.353778	-1.05738	0.0173	0.229704	no
21.251	25.9504	0.288226	1.06068	0.01735	0.229843	no
17.4708	14.3729	-0.281598	-1.05101	0.01735	0.229843	no
145.523	179.777	0.304969	1.05857	0.0174	0.229981	no
17.5718	22.4839	0.355632	1.07005	0.0174	0.229981	no
104.398	129.379	0.309512	1.0651	0.0175	0.23104	no
9.87411	7.95456	-0.311868	-1.04001	0.01765	0.231966	no
63.6526	78.7375	0.30683	1.06161	0.0176	0.231966	no
3.88021	2.81708	-0.461935	-1.06349	0.01765	0.231966	no
5.26979	3.94498	-0.417726	-1.06828	0.01765	0.231966	no
18.9864	14.9908	-0.340888	-1.05411	0.01775	0.232753	no
1.71415	0.882281	-0.958179	-1.09577	0.01775	0.232753	no
47.6116	58.0597	0.286223	1.06348	0.0178	0.233146	no
5.18562	3.972	-0.384653	-1.07469	0.01785	0.233538	no
19.9448	25.1207	0.332866	1.05447	0.0179	0.233665	no
2.46756	1.79218	-0.461375	-1.05872	0.0179	0.233665	no
9.78003	12.1952	0.318408	1.04486	0.01795	0.233793	no
5.16351	4.07007	-0.343298	-1.06918	0.01795	0.233793	no
6.14563	4.55304	-0.432731	-1.04934	0.018	0.234181	no
1.95254	1.37425	-0.506706	-1.06367	0.01805	0.234307	no
1.00296	1.80265	0.845854	1.05988	0.01805	0.234307	no
2.63178	6.07108	1.20591	1.12134	0.0182	0.235991	no
121.545	152.631	0.328553	1.04215	0.01825	0.236112	no
8.19871	6.70175	-0.29086	-1.04792	0.01825	0.236112	no
1.87775	2.76746	0.559556	1.07135	0.01845	0.238434	no
19.8115	24.2851	0.293734	1.04004	0.0185	0.238814	no
5.81932	4.69872	-0.308581	-1.0514	0.01855	0.239194	no
5.58801	4.39164	-0.347575	-1.05365	0.0186	0.239573	no
39.4696	48.8362	0.307209	1.05942	0.01875	0.240971	no
19.8003	16.167	-0.292475	-1.06382	0.01875	0.240971	no
21.2642	16.9943	-0.323369	-1.02743	0.01885	0.241721	no
20.8431	17.0933	-0.286139	-1.05204	0.01885	0.241721	no
29.6594	24.4702	-0.277463	-1.03893	0.0191	0.243685	no
69.2828	84.9227	0.293652	1.04825	0.01915	0.243685	no
8.57022	10.8312	0.337792	1.02452	0.0191	0.243685	no
29.8815	24.5892	-0.281229	-1.04144	0.01915	0.243685	no
10.0354	12.6495	0.333982	1.05124	0.01915	0.243685	no
22.6143	18.4314	-0.295069	-1.05161	0.01905	0.243685	no
9.45175	7.71345	-0.293205	-1.05959	0.0194	0.244722	no
15.1134	12.3286	-0.293811	-1.041	0.0194	0.244722	no
1.61386	2.28825	0.503731	1.03414	0.0193	0.244722	no
24.7651	20.4287	-0.277711	-1.03516	0.0193	0.244722	no
28.6364	34.9972	0.289391	1.04609	0.0193	0.244722	no

4.48173	3.6126	-0.311015	-1.02894	0.01935	0.244722	no
47.6695	58.1829	0.287528	1.03009	0.0194	0.244722	no
32.6147	39.5445	0.277957	1.03213	0.0194	0.244722	no
3.43895	2.63232	-0.385635	-1.05032	0.0195	0.245283	no
1.30008	2.5366	0.964293	1.05724	0.0195	0.245283	no
5.7888	4.60336	-0.330576	-1.03436	0.01955	0.245283	no
26.5431	21.8652	-0.2797	-1.03717	0.01955	0.245283	no
8.60718	6.8284	-0.333993	-1.02159	0.01955	0.245283	no
4.95024	4.00327	-0.306318	-1.03019	0.0196	0.24538	no
8.71334	7.11127	-0.293119	-1.02996	0.0196	0.24538	no
13.9778	11.4045	-0.293538	-1.04368	0.0197	0.246102	no
26.661	21.8471	-0.287291	-1.03072	0.0197	0.246102	no
20.463	32.8098	0.681111	1.06313	0.0199	0.248067	no
7.8211	5.85354	-0.418063	-1.04781	0.01995	0.248423	no
2.92281	2.1713	-0.428799	-1.03883	0.02	0.248779	no
16.5751	21.2133	0.355945	1.02816	0.0201	0.249756	no
44.4623	54.5159	0.294096	1.01356	0.0202	0.250463	no
11.055	8.96236	-0.302747	-1.03283	0.0202	0.250463	no
2.80053	2.01345	-0.476029	-1.03226	0.0203	0.251434	no
20.5922	16.9527	-0.280581	-1.02609	0.02035	0.251785	no
17.3218	14.1338	-0.293439	-1.01969	0.0204	0.252135	no
22.3063	18.1515	-0.297363	-1.04011	0.02055	0.253181	no
2.42314	3.5176	0.537709	1.04013	0.02055	0.253181	no
1.50865	2.7356	0.858603	1.06372	0.02055	0.253181	no
7.10952	9.56447	0.427934	1.04371	0.0206	0.25326	no
2.79176	4.24356	0.604098	1.04254	0.0206	0.25326	no
3.48036	2.76382	-0.332572	-1.0312	0.0208	0.255449	no
13.1435	16.2541	0.306458	1.04606	0.02085	0.255792	no
153.346	188.085	0.294594	1.02793	0.02095	0.255938	no
14.2465	11.6758	-0.287089	-1.03491	0.02095	0.255938	no
6.57475	5.03499	-0.384948	-1.02444	0.02095	0.255938	no
1.51459	1.04641	-0.533475	-1.03863	0.02095	0.255938	no
13.5795	11.1825	-0.280185	-1.0177	0.021	0.256279	no
6.92502	5.58058	-0.311402	-1.04147	0.02105	0.256351	no
28.274	34.3239	0.279736	1.03048	0.02105	0.256351	no
1.8611	1.20086	-0.632095	-1.02051	0.02125	0.257437	no
24.1638	19.4504	-0.313047	-1.01439	0.0212	0.257437	no
3.56326	2.44753	-0.541875	-1.03938	0.02125	0.257437	no
15.8001	13.0254	-0.2786	-1.00374	0.02125	0.257437	no
7.0279	5.59317	-0.329426	-1.0238	0.02125	0.257437	no
4.30276	3.46994	-0.310351	-1.02457	0.02135	0.258111	no
83.0085	100.852	0.280902	1.04143	0.02135	0.258111	no
3.64811	7.68702	1.07528	1.08575	0.0215	0.259654	no
34.6045	28.6302	-0.273425	-1.02211	0.02165	0.260923	no
1.10689	1.71713	0.633492	1.04042	0.02165	0.260923	no
7.41656	5.59429	-0.406796	-1.0213	0.02175	0.261045	no
3.91586	3.07727	-0.347679	-1.00729	0.02175	0.261045	no

1.95493	1.3753	-0.507365	-1.02086	0.02175	0.261045	no
20.3062	16.784	-0.274838	-1.01468	0.02175	0.261045	no
26.5218	32.5711	0.296413	1.02026	0.02185	0.261975	no
40.4455	49.5721	0.293548	1.00436	0.0219	0.262303	no
90.4684	110.103	0.283369	1.02455	0.02195	0.262631	no
18.2682	22.1307	0.276715	1.02235	0.022	0.262959	no
11.9142	14.5456	0.2879	1.02467	0.0221	0.263341	no
3.10796	2.2132	-0.489834	-1.01826	0.0221	0.263341	no
9.95386	7.72758	-0.365239	-1.0245	0.0221	0.263341	no
3.02982	4.48403	0.565565	1.01951	0.02215	0.263397	no
72.0913	87.6019	0.281136	1.03464	0.02215	0.263397	no
394.548	497.825	0.335438	1.02764	0.0222	0.263721	no
1.5366	1.15884	-0.407064	-1.02818	0.0223	0.264639	no
14.5064	12.0307	-0.269971	-1.0082	0.02235	0.264962	no
14.6382	12.1183	-0.272554	-1.02095	0.0224	0.265284	no
2.11198	3.17875	0.589861	1.00155	0.02245	0.265335	no
59.2719	48.8354	-0.279421	-1.01561	0.02245	0.265335	no
66.0527	81.4572	0.302425	1.00922	0.0227	0.266662	no
4.38518	6.22659	0.505806	1.01067	0.0227	0.266662	no
3.93637	2.51728	-0.645	-0.971918	0.0226	0.266662	no
24.658	20.4425	-0.270485	-1.0033	0.02265	0.266662	no
9.73469	7.77031	-0.325164	-1.02684	0.02265	0.266662	no
13.9171	17.0067	0.289241	1.01656	0.0227	0.266662	no
21.2511	25.6265	0.270096	1.00294	0.0228	0.267566	no
16.223	13.0162	-0.317728	-1.00003	0.0229	0.268468	no
37.3324	30.6122	-0.286321	-1.01431	0.02305	0.268601	no
0.862169	1.66376	0.948404	1.06992	0.023	0.268601	no
3.48383	5.56829	0.67656	1.03177	0.02295	0.268601	no
15.5879	12.8282	-0.281107	-1.01026	0.02305	0.268601	no
5.85222	7.30447	0.319794	1.00052	0.02305	0.268601	no
131.218	158.528	0.272769	0.996838	0.023	0.268601	no
87.7418	106.031	0.273152	1.01327	0.02315	0.269226	no
11.6531	14.4742	0.312772	1.00456	0.02315	0.269226	no
8.3748	11.4678	0.453458	1.02511	0.02325	0.269579	no
1.72511	2.58022	0.580804	1.01567	0.02325	0.269579	no
14.2668	11.7234	-0.283271	-1.00707	0.02325	0.269579	no
170.097	208.085	0.290817	0.997958	0.0233	0.269621	no
94.125	114.384	0.28124	1.01029	0.0233	0.269621	no
31.7509	38.3725	0.273276	1.0115	0.02335	0.269931	no
9.90752	12.6703	0.354857	1.00786	0.0234	0.27024	no
3.13223	2.25125	-0.476462	-1.0007	0.02345	0.27028	no
8.34669	6.85145	-0.284796	-1.01392	0.02345	0.27028	no
10.5191	13.8054	0.392222	1.01802	0.02355	0.270895	no
2.44064	1.75551	-0.475369	-1.00758	0.02355	0.270895	no
13.2754	10.7081	-0.310055	-0.994598	0.0236	0.270934	no
8.15209	6.68027	-0.287264	-0.992472	0.0236	0.270934	no
7.10939	5.56516	-0.353303	-1.00763	0.02365	0.27124	no

1.6364	2.73403	0.740504	0.956227	0.0237	0.271277	no
6.57691	5.17104	-0.346957	-0.997044	0.0237	0.271277	no
1.64576	2.63402	0.678513	1.01584	0.0238	0.271353	no
27.0804	22.4069	-0.273305	-1.00074	0.0238	0.271353	no
31.956	26.4278	-0.274033	-1.01382	0.0238	0.271353	no
2.25652	1.69859	-0.409763	-1.01311	0.0239	0.271959	no
18.3698	22.6564	0.302582	0.987144	0.024	0.272031	no
1.2165	1.81023	0.573428	0.996972	0.024	0.272031	no
32.69	39.3746	0.268417	1.00144	0.024	0.272031	no
31.084	37.6919	0.278082	1.00397	0.02395	0.272031	no
10.5831	12.9313	0.289109	1.00301	0.02405	0.272067	no
7.2615	9.15753	0.334691	1.00288	0.02405	0.272067	no
89.6208	108.485	0.275585	0.993785	0.0242	0.273498	no
0.896659	1.45203	0.695443	1.0134	0.02425	0.273796	no
5.32732	3.79949	-0.487604	-1.01373	0.0244	0.273893	no
43.5579	52.6541	0.273611	1.00167	0.02435	0.273893	no
3.6704	2.72328	-0.430593	-0.99975	0.0244	0.273893	no
2.09631	1.48333	-0.499012	-1.01634	0.02435	0.273893	no
5.23121	6.59245	0.333669	0.997784	0.0244	0.273893	no
2.54003	1.78602	-0.508099	-1.01457	0.02435	0.273893	no
9.18868	11.2633	0.293704	0.99836	0.0245	0.274221	no
11.4605	9.48052	-0.273627	-1.00039	0.0245	0.274221	no
28.3226	34.5458	0.286559	1.01304	0.0246	0.275075	no
3.7529	2.84674	-0.398694	-1.02307	0.0247	0.275927	no
19.8831	15.9423	-0.318687	-0.998539	0.0248	0.275983	no
53.9946	44.7448	-0.271096	-1.00718	0.0248	0.275983	no
9.9584	8.06108	-0.30494	-0.99488	0.02475	0.275983	no
2.22943	1.61308	-0.46685	-0.998509	0.0248	0.275983	no
48.5402	58.416	0.267184	0.999196	0.0249	0.276831	no
2.05317	3.13242	0.609422	1.0203	0.02495	0.276857	no
48.3022	59.1486	0.292254	0.981187	0.02495	0.276857	no
9.05944	11.218	0.308317	0.983806	0.025	0.277147	no
4.11687	3.2696	-0.332432	-1.00674	0.0251	0.277726	no
7.54834	5.27363	-0.517362	-1.02058	0.0251	0.277726	no
13.2291	16.2333	0.295236	0.986003	0.02535	0.277869	no
11.1798	8.41563	-0.409757	-1.00526	0.0254	0.277869	no
22.5217	18.6379	-0.273073	-1.00604	0.02535	0.277869	no
1.15917	2.12955	0.877454	1.02451	0.02535	0.277869	no
18.6529	22.5659	0.274744	1.00405	0.02535	0.277869	no
11.8462	9.73985	-0.282458	-0.999838	0.0254	0.277869	no
2.58973	3.36783	0.379021	1.00653	0.0252	0.277869	no
1.76181	2.70677	0.619511	1.01668	0.0254	0.277869	no
17.6356	14.4075	-0.291672	-1.01024	0.0253	0.277869	no
13.4179	16.3135	0.281909	0.988366	0.0252	0.277869	no
6.96777	8.60006	0.303648	0.983682	0.02515	0.277869	no
14.9166	12.3844	-0.268398	-0.994593	0.02545	0.277893	no
7.97425	6.48775	-0.297631	-0.980559	0.02545	0.277893	no

3.43486	4.49259	0.387297	1.0234	0.0255	0.278177	no
8.64148	11.1572	0.368624	1.00437	0.02555	0.2782	no
11.7462	14.5741	0.311213	0.999811	0.02555	0.2782	no
10.7477	14.0168	0.383126	0.999136	0.0256	0.278223	no
13.2787	10.9099	-0.283474	-1.00698	0.0256	0.278223	no
35.1583	29.17	-0.269383	-1.00804	0.0258	0.280135	no
20.5137	25.4646	0.311902	0.978856	0.02585	0.280416	no
26.1741	21.7325	-0.268286	-0.992809	0.0259	0.280696	no
25.1376	30.3544	0.272058	1.01143	0.02595	0.280976	no
8.1231	10.1651	0.323529	1.00724	0.026	0.281255	no
21.8937	18.1053	-0.274104	-0.989461	0.02605	0.281272	no
4.06005	3.15813	-0.362427	-0.995025	0.02605	0.281272	no
6.51306	5.38466	-0.274482	-0.990234	0.0262	0.282367	no
2.77498	2.20338	-0.33276	-0.999637	0.0262	0.282367	no
2.61015	3.50679	0.42602	0.985594	0.02625	0.282382	no
36.642	44.1674	0.269483	1.00326	0.02625	0.282382	no
3.17514	4.72004	0.571978	1.00057	0.0264	0.282948	no
37.2859	44.6762	0.260878	0.975923	0.0264	0.282948	no
78.8702	65.5898	-0.266008	-0.986903	0.0264	0.282948	no
21.1168	26.4181	0.323137	0.987215	0.0264	0.282948	no
2.04351	1.41981	-0.525358	-1.00307	0.02645	0.282961	no
10.9761	15.1834	0.468137	0.991543	0.02645	0.282961	no
11.2176	9.30587	-0.269548	-0.988398	0.02655	0.283509	no
24.6492	29.8284	0.275144	0.995605	0.02655	0.283509	no
6.58626	7.98955	0.278655	0.976304	0.02665	0.284055	no
11.4078	14.0071	0.296145	0.97783	0.02665	0.284055	no
52.9229	43.7818	-0.273563	-0.998297	0.0267	0.284066	no
6.39208	8.26164	0.370142	0.98495	0.0267	0.284066	no
407.278	492.139	0.273052	0.986856	0.0268	0.284609	no
2.95753	4.37929	0.566304	1.00092	0.0268	0.284609	no
6.0945	4.37743	-0.477423	-0.968485	0.0269	0.28541	no
30.3648	24.6017	-0.303643	-0.980241	0.02695	0.28568	no
5.67317	7.71521	0.443551	0.989613	0.027	0.285949	no
2.12654	1.20473	-0.819798	-1.01035	0.02705	0.286218	no
4.10803	5.43933	0.404983	0.98865	0.0271	0.286225	no
43.2274	35.6896	-0.27644	-0.986916	0.0271	0.286225	no
5.42159	8.54965	0.65715	1.004	0.02715	0.286233	no
31.1147	37.6069	0.273402	0.974027	0.02715	0.286233	no
3.76232	3.0151	-0.319419	-0.967074	0.0272	0.286241	no
1.88553	3.27732	0.797542	1.02487	0.0272	0.286241	no
17.1609	20.7028	0.270705	0.992955	0.02735	0.287299	no
6.37147	8.62523	0.436938	0.98995	0.02735	0.287299	no
5.95678	4.7696	-0.320666	-0.968282	0.0274	0.287564	no
7.82936	6.44345	-0.28106	-0.981461	0.0275	0.288353	no
50.5362	60.6687	0.263634	0.98662	0.02755	0.288357	no
90.0379	108.19	0.26496	0.980734	0.02755	0.288357	no
34.7577	41.6335	0.260415	0.970622	0.02765	0.289143	no

21.4319	26.3476	0.29791	0.962569	0.0277	0.289405	no
15.4861	18.9479	0.291062	0.986618	0.02785	0.29045	no
1.20325	2.20119	0.871346	1.0019	0.02795	0.29071	no
101.421	121.466	0.260192	0.97369	0.02795	0.29071	no
2.78523	2.02316	-0.461188	-0.987758	0.02795	0.29071	no
3.27004	2.63663	-0.310609	-0.978979	0.028	0.29097	no
0.783913	1.63804	1.0632	1.02338	0.0281	0.291487	no
2.83465	2.27522	-0.317167	-0.97203	0.0281	0.291487	no
33.869	28.0678	-0.271047	-0.978897	0.02825	0.29226	no
11.6306	15.7002	0.432857	0.980321	0.02825	0.29226	no
1.97036	3.0114	0.611974	0.978865	0.0282	0.29226	no
16.1875	20.6286	0.34976	0.984837	0.02845	0.294068	no
41.5318	34.72	-0.258451	-0.964345	0.0285	0.294323	no
3.44368	2.74212	-0.328659	-0.974493	0.0286	0.294831	no
67.1423	80.833	0.267722	0.980733	0.0286	0.294831	no
7.93728	9.93734	0.324215	0.96291	0.02875	0.295591	no
1.12714	1.5039	0.416038	0.966637	0.02875	0.295591	no
7.34783	6.11158	-0.265774	-0.973266	0.0287	0.295591	no
320.6	389.745	0.281753	0.95718	0.02885	0.296357	no
20.3335	16.5552	-0.296577	-0.97862	0.0289	0.296608	no
8.21318	6.39822	-0.360271	-0.975194	0.029	0.297109	no
13.1885	16.1918	0.295982	0.962841	0.029	0.297109	no
10.53	8.63309	-0.286561	-0.953277	0.02905	0.297347	no
12.6008	10.3549	-0.283194	-0.96368	0.0291	0.297347	no
15.4646	19.0065	0.297525	0.972388	0.0291	0.297347	no
3.24972	4.38188	0.431237	0.986734	0.0292	0.297832	no
22.1258	18.2597	-0.277066	-0.972059	0.02925	0.297832	no
4.4041	3.33258	-0.402208	-0.979423	0.0292	0.297832	no
40.8899	49.0589	0.262768	0.969911	0.02925	0.297832	no
53.1853	44.4172	-0.259911	-0.971745	0.0294	0.29856	no
139.95	170.794	0.287347	0.964245	0.0294	0.29856	no
7.3491	8.97317	0.28805	0.951731	0.02945	0.29856	no
15.2782	12.7024	-0.266374	-0.972372	0.02945	0.29856	no
5.33914	4.44337	-0.264954	-0.959974	0.02945	0.29856	no
0.790672	1.57318	0.992531	1.00864	0.0295	0.298806	no
22.9	19.1602	-0.257237	-0.959971	0.02965	0.299803	no
11.7435	9.62399	-0.287157	-0.96567	0.02975	0.300291	no
1.15776	1.81562	0.649122	0.964336	0.02975	0.300291	no
21.0537	26.4942	0.331601	0.972546	0.0298	0.300535	no
2.48538	1.714	-0.536101	-0.979844	0.03	0.30229	no
10.3241	7.6323	-0.435822	-0.99206	0.03005	0.302531	no
12.3098	9.97617	-0.303244	-0.967821	0.0301	0.302772	no
53.8106	64.9125	0.270604	0.973759	0.0302	0.302991	no
25.5291	31.6823	0.311533	0.995511	0.0302	0.302991	no
21.1029	30.8035	0.54565	0.96906	0.03015	0.302991	no
17.6932	14.6249	-0.274772	-0.942517	0.0303	0.30347	no
14.4352	11.9247	-0.275634	-0.990003	0.0303	0.30347	no

32.4702	39.1642	0.270419	0.967708	0.0304	0.303664	no
6.45037	5.25217	-0.296469	-0.950786	0.03045	0.303664	no
1.87905	2.83532	0.593505	0.953251	0.03045	0.303664	no
5.38473	4.2317	-0.347636	-0.979488	0.0304	0.303664	no
10.8703	8.85555	-0.295734	-0.96552	0.03035	0.303664	no
14.1582	16.9912	0.263158	0.972746	0.03055	0.3044	no
150.22	180.64	0.266041	0.958393	0.0306	0.304637	no
6.8092	5.67071	-0.263956	-0.943685	0.03075	0.305868	no
12.3689	10.2667	-0.268738	-0.962274	0.03095	0.307594	no
16.1249	13.4142	-0.265534	-0.96664	0.031	0.307827	no
11.7912	14.2126	0.269461	0.978608	0.0311	0.308557	no
3.56019	4.82469	0.43848	0.951882	0.03125	0.309516	no
86.2507	71.3419	-0.273786	-0.955474	0.03125	0.309516	no
18.0708	22.8159	0.33638	0.967748	0.03135	0.309715	no
16.5558	13.7905	-0.263668	-0.941891	0.03135	0.309715	no
29.1679	24.359	-0.259922	-0.971958	0.0313	0.309715	no
10.8682	16.4941	0.601833	0.971655	0.03145	0.310175	no
5.67622	4.72365	-0.265027	-0.956187	0.03145	0.310175	no
22.6412	18.8121	-0.26729	-0.958866	0.0315	0.310405	no
19.5421	16.0657	-0.282603	-0.938906	0.0316	0.3106	no
13.0434	10.2744	-0.344273	-0.967029	0.03155	0.3106	no
3.10289	2.2787	-0.445402	-0.95642	0.0316	0.3106	no
15.5181	12.812	-0.276462	-0.957033	0.03165	0.310828	no
9.2752	7.20515	-0.36435	-0.959958	0.03175	0.311547	no
2.27391	1.6497	-0.462973	-0.974238	0.03185	0.312264	no
3.07247	4.03981	0.394887	0.962203	0.03195	0.312981	no
11.0653	13.3342	0.269082	0.958459	0.03205	0.313696	no
30.1583	36.3133	0.267939	0.970117	0.0322	0.314899	no
10.6302	8.4099	-0.33801	-0.962418	0.0323	0.315346	no
5.68789	4.23241	-0.426416	-0.965848	0.0323	0.315346	no
4.79678	3.9289	-0.287939	-0.950661	0.03245	0.315886	no
12.6632	10.4908	-0.271518	-0.959506	0.0325	0.315886	no
36.7516	43.9313	0.257445	0.961106	0.0324	0.315886	no
210.336	255.081	0.278258	0.946109	0.03245	0.315886	no
14.4356	11.7707	-0.294439	-0.93575	0.03255	0.315886	no
4.47643	3.52153	-0.346148	-0.968817	0.0326	0.315886	no
22.7	27.5687	0.280344	0.950806	0.0325	0.315886	no
2.17677	1.55235	-0.487738	-0.950234	0.03255	0.315886	no
3.13765	2.35159	-0.416048	-0.960673	0.0326	0.315886	no
21.8483	26.1517	0.25938	0.946946	0.0327	0.31659	no
3.29583	4.71861	0.517718	0.961697	0.03275	0.316767	no
8.58874	6.21192	-0.467407	-0.934023	0.0328	0.316767	no
13.2949	10.9811	-0.275849	-0.951165	0.0328	0.316767	no
104.539	125.692	0.265854	0.948844	0.03285	0.316986	no
1.64899	2.39549	0.538742	0.953238	0.0329	0.317205	no
30.2749	36.2138	0.258421	0.948633	0.033	0.317379	no
6.36749	7.94688	0.319663	0.953265	0.033	0.317379	no

32.0103	40.2178	0.329296	0.941841	0.033	0.317379	no
9.91935	12.3389	0.314902	0.957383	0.0332	0.319039	no
6.01146	4.45607	-0.431945	-0.944247	0.0333	0.319735	no
13.0182	16.122	0.308506	0.925902	0.03345	0.320645	no
18.1041	22.1237	0.28928	0.946708	0.03355	0.321339	no
3.69166	2.75733	-0.420996	-0.952917	0.0336	0.321553	no
16.6494	21.7632	0.386418	0.947137	0.03365	0.321766	no
2.7242	1.83421	-0.570671	-0.950412	0.0337	0.321979	no
4.97864	3.52444	-0.49836	-0.924122	0.0338	0.322669	no
4.57464	6.65051	0.539805	0.957103	0.0339	0.323358	no
6.48918	5.37517	-0.271724	-0.950245	0.03405	0.324522	no
5.29745	6.72916	0.345129	0.95197	0.03415	0.324942	no
1.92346	2.53967	0.400935	0.95701	0.03415	0.324942	no
38.0924	45.343	0.251378	0.937653	0.03435	0.326311	no
46.7235	55.5417	0.249423	0.933704	0.03455	0.326875	no
13.819	11.6185	-0.250231	-0.933107	0.03455	0.326875	no
27.464	22.994	-0.256281	-0.936791	0.03455	0.326875	no
2.11987	1.28984	-0.716785	-0.938606	0.03445	0.326875	no
4.17715	3.39791	-0.297872	-0.935435	0.0345	0.326875	no
3.25892	2.21817	-0.555023	-0.957291	0.03465	0.327288	no
12.6534	15.6347	0.305232	0.942518	0.03465	0.327288	no
10.1204	13.6732	0.434093	0.934804	0.0348	0.328171	no
4.02973	3.17364	-0.344545	-0.942586	0.0348	0.328171	no
60.9269	72.5285	0.251469	0.9413	0.03485	0.328314	no
2.07136	2.65431	0.357758	0.951237	0.0349	0.328314	no
3.18576	2.45795	-0.374182	-0.952806	0.0349	0.328314	no
2.25279	3.42903	0.606088	0.944934	0.035	0.328926	no
13.7368	16.4797	0.262644	0.929641	0.03505	0.328926	no
13.1115	10.7346	-0.288569	-0.947849	0.03505	0.328926	no
1.45883	2.04892	0.490056	0.936325	0.0351	0.32913	no
13.5885	16.4068	0.271903	0.936077	0.0352	0.329801	no
8.55455	7.09454	-0.269983	-0.925174	0.0353	0.330206	no
43.678	35.7073	-0.29069	-0.938562	0.0353	0.330206	no
16.1028	13.2266	-0.283872	-0.936423	0.0354	0.330875	no
4.72256	3.58548	-0.397403	-0.957358	0.03545	0.33101	no
6.42462	5.36608	-0.259744	-0.933502	0.0355	0.33101	no
30.0151	24.0255	-0.321121	-0.945525	0.0355	0.33101	no
17.0481	20.5808	0.271686	0.928281	0.03555	0.331211	no
7.91754	9.71545	0.295228	0.942258	0.0356	0.331411	no
36.393	30.517	-0.254045	-0.938911	0.03575	0.332541	no
13.1017	15.6731	0.258535	0.931479	0.0359	0.333669	no
146.45	174.503	0.252837	0.928077	0.0362	0.333718	no
5.81497	4.77657	-0.283795	-0.92592	0.03605	0.333718	no
7.30247	6.12111	-0.254591	-0.932332	0.0361	0.333718	no
20.0615	24.1073	0.265039	0.931701	0.03625	0.333718	no
347.15	435.334	0.326567	0.950794	0.036	0.333718	no
10.1438	8.52665	-0.250547	-0.93389	0.036	0.333718	no

27.8884	39.8932	0.516476	0.937139	0.03595	0.333718	no
3.89413	5.23897	0.427982	0.937956	0.03605	0.333718	no
14.2679	17.2187	0.271208	0.92531	0.03625	0.333718	no
7.70985	9.31168	0.272337	0.951713	0.0362	0.333718	no
19.7548	16.6745	-0.244558	-0.919131	0.0362	0.333718	no
14.0326	16.7939	0.259159	0.924918	0.0363	0.333914	no
9.86523	13.8183	0.486154	0.925558	0.0364	0.334097	no
5.90408	7.90147	0.420409	0.935591	0.03655	0.334097	no
212.964	266.638	0.324275	0.943706	0.03635	0.334097	no
4.45972	3.73557	-0.255626	-0.933776	0.03655	0.334097	no
14.1397	11.8951	-0.249391	-0.930589	0.03645	0.334097	no
10.2024	8.50053	-0.263285	-0.923381	0.03655	0.334097	no
4.70116	7.20615	0.616212	0.928414	0.03665	0.334485	no
14.8997	12.5637	-0.246026	-0.922999	0.03665	0.334485	no
1.86716	1.35947	-0.457806	-0.932892	0.0367	0.334679	no
15.0711	17.9729	0.254041	0.923156	0.03675	0.334872	no
47.4874	57.545	0.277144	0.948956	0.0368	0.335065	no
7.89887	6.36246	-0.312061	-0.930408	0.03695	0.335904	no
15.218	18.4641	0.278951	0.92308	0.03695	0.335904	no
70.1923	83.8886	0.257162	0.938995	0.03705	0.33655	no
5.66502	7.54433	0.413312	0.929494	0.0371	0.33674	no
46.7722	55.7421	0.253115	0.917964	0.0373	0.338028	no
55.7857	46.8584	-0.251588	-0.927372	0.0373	0.338028	no
5.04725	4.02585	-0.326204	-0.920825	0.0374	0.33867	no
35.0487	27.9409	-0.326984	-0.928085	0.0375	0.339311	no
155.636	187.564	0.269207	0.919966	0.03755	0.339423	no
25.6862	21.4431	-0.260475	-0.933558	0.0376	0.339423	no
10.4978	8.78626	-0.25676	-0.922329	0.0376	0.339423	no
9.19004	11.5032	0.323896	0.925999	0.0379	0.340729	no
8.64752	6.83101	-0.340188	-0.923566	0.0379	0.340729	no
1.0583	1.67478	0.662232	0.91143	0.0379	0.340729	no
5.80007	4.68611	-0.307679	-0.934282	0.03795	0.340729	no
64.4485	76.6212	0.249596	0.926944	0.03785	0.340729	no
1.66205	2.27014	0.449815	0.927687	0.03795	0.340729	no
38.6603	32.5417	-0.248563	-0.926363	0.03825	0.343157	no
2.96987	2.3747	-0.322654	-0.927202	0.0383	0.343341	no
15.8559	13.3022	-0.253356	-0.929595	0.03835	0.343524	no
53.4626	63.9181	0.257694	0.920321	0.0385	0.344602	no
11.9821	14.6454	0.289561	0.920744	0.03875	0.344715	no
7.54448	6.33981	-0.250982	-0.91186	0.0387	0.344715	no
9.19709	11.1213	0.27407	0.917277	0.03865	0.344715	no
8.15461	6.76019	-0.270552	-0.909727	0.03875	0.344715	no
26.0046	30.9688	0.252049	0.918965	0.03875	0.344715	no
35.9832	42.7928	0.250043	0.930658	0.03875	0.344715	no
3.5884	5.07935	0.501303	0.917219	0.03865	0.344715	no
4.21058	3.26845	-0.365413	-0.917969	0.03875	0.344715	no
1.70979	0.976608	-0.807964	-0.935873	0.03905	0.346587	no

4.47423	5.84224	0.384883	0.936329	0.03905	0.346587	no
3.86527	4.75623	0.29925	0.929124	0.03905	0.346587	no
2.2498	2.94962	0.39073	0.928422	0.03925	0.347831	no
5.08476	3.46964	-0.551394	-0.916534	0.0393	0.348009	no
19.3106	16.0113	-0.270303	-0.908933	0.03935	0.348186	no
35.3874	41.9813	0.246512	0.916922	0.0394	0.348363	no
37.5874	44.5783	0.246095	0.917947	0.03945	0.34854	no
11.803	9.33371	-0.338636	-0.918819	0.03955	0.348893	no
1.95162	2.54304	0.38188	0.915649	0.03955	0.348893	no
29.5326	24.9163	-0.245218	-0.909021	0.03965	0.34951	no
1.52116	1.00372	-0.599812	-0.914686	0.0397	0.349685	no
12.9362	10.7272	-0.27014	-0.920037	0.03985	0.35074	no
62.8428	74.5616	0.246684	0.913928	0.0399	0.350915	no
81.9571	68.8354	-0.251718	-0.90533	0.04	0.351262	no
34.8183	41.6292	0.257751	0.909008	0.04005	0.351436	no
7.89763	11.1945	0.5033	0.908007	0.0401	0.351609	no
5.36922	4.07553	-0.397727	-0.932631	0.04025	0.352658	no
121.531	143.823	0.242974	0.904738	0.0403	0.35283	no
25.7633	30.5068	0.243816	0.907484	0.04035	0.353002	no
1.90666	1.23236	-0.629621	-0.915005	0.04045	0.353079	no
0.991471	1.76723	0.833851	0.927202	0.04045	0.353079	no
186.858	221.612	0.246095	0.91494	0.04045	0.353079	no
9.16575	11.2159	0.291214	0.908025	0.0405	0.35325	no
13.7223	17.2212	0.327663	0.91671	0.0406	0.353478	no
14.9081	12.5372	-0.249888	-0.894194	0.04075	0.353478	no
5.98195	4.61805	-0.373332	-0.932941	0.04065	0.353478	no
3.78764	5.1618	0.446576	0.899713	0.04065	0.353478	no
24.8749	29.4029	0.241266	0.900917	0.0408	0.353478	no
2.21775	1.62617	-0.44762	-0.913672	0.04075	0.353478	no
22.1266	18.4337	-0.263436	-0.92274	0.0406	0.353478	no
17.6752	14.768	-0.259256	-0.918452	0.0408	0.353478	no
8.82806	7.35598	-0.26318	-0.907309	0.0408	0.353478	no
31.7208	37.7034	0.249264	0.906083	0.04085	0.353554	no
1.61878	1.23156	-0.394427	-0.908132	0.0409	0.353554	no
5.67113	4.59947	-0.302167	-0.913141	0.041	0.354155	no
6.5882	5.05495	-0.382188	-0.928092	0.04115	0.354923	no
27.739	33.8097	0.285521	0.892208	0.04115	0.354923	no
38.5594	32.5313	-0.245252	-0.915321	0.04125	0.355521	no
7.12603	5.87119	-0.279446	-0.913887	0.0414	0.356549	no
3.6989	2.75802	-0.423461	-0.914643	0.04165	0.357279	no
13.8857	17.2533	0.31327	0.910125	0.0417	0.357279	no
5.4452	6.63774	0.285708	0.902909	0.0416	0.357279	no
1.45879	0.412043	-1.8239	-1.06208	0.0417	0.357279	no
12.0975	14.4103	0.25239	0.903428	0.0417	0.357279	no
12.9688	16.6725	0.362426	0.914366	0.04155	0.357279	no
21.7877	18.4409	-0.240608	-0.902643	0.0417	0.357279	no
10.7207	13.0683	0.285666	0.90553	0.04175	0.357444	no

7.00498	8.79061	0.327583	0.910596	0.04185	0.358036	no
5.25602	6.31535	0.264892	0.911791	0.04195	0.358628	no
6.37485	5.1508	-0.307593	-0.912437	0.0421	0.359645	no
3.0987	2.50613	-0.306203	-0.906584	0.0422	0.359706	no
36.6434	30.6537	-0.257491	-0.901084	0.0422	0.359706	no
32.464	27.3453	-0.247545	-0.906698	0.0422	0.359706	no
72.7759	86.2552	0.245152	0.901554	0.0424	0.359786	no
6.34431	5.31209	-0.256184	-0.915847	0.04235	0.359786	no
25.6277	21.6648	-0.242352	-0.90553	0.04235	0.359786	no
24.8774	30.7416	0.305353	0.897471	0.0425	0.359786	no
21.9902	18.5989	-0.241648	-0.902892	0.04235	0.359786	no
5.39885	9.44252	0.80652	0.922746	0.04235	0.359786	no
4.19359	3.00702	-0.479854	-0.90089	0.04245	0.359786	no
19.6988	16.5773	-0.248893	-0.898967	0.04245	0.359786	no
15.1205	12.7052	-0.251077	-0.904765	0.04255	0.359786	no
22.8049	18.9938	-0.263812	-0.906559	0.0425	0.359786	no
22.9151	19.2143	-0.25412	-0.90061	0.04255	0.359786	no
124.251	150.928	0.2806	0.893187	0.04265	0.360107	no
24.2359	28.8291	0.250383	0.896476	0.04265	0.360107	no
13.8029	11.3917	-0.276992	-0.90676	0.0427	0.360268	no
3.48446	4.51782	0.37469	0.903789	0.04275	0.360428	no
12.8274	10.3965	-0.303137	-0.892291	0.04285	0.360907	no
5.40321	7.0932	0.39262	0.895119	0.0429	0.360907	no
21.1326	25.603	0.276841	0.905084	0.0429	0.360907	no
9.35222	11.105	0.247829	0.898873	0.043	0.360964	no
2.89693	3.88368	0.422898	0.892519	0.043	0.360964	no
7.1734	5.8268	-0.299953	-0.896327	0.043	0.360964	no
1.9175	2.99064	0.641229	0.924802	0.04325	0.361913	no
1.59554	1.25114	-0.350805	-0.903706	0.0433	0.361913	no
16.1121	13.5998	-0.244558	-0.904324	0.04325	0.361913	no
58.2753	68.8644	0.240873	0.900173	0.04325	0.361913	no
251.421	305.709	0.282055	0.90646	0.04325	0.361913	no
7.25746	6.10096	-0.250426	-0.898719	0.0433	0.361913	no
7.76741	6.3915	-0.281279	-0.894002	0.04345	0.362385	no
4.68483	3.83315	-0.289466	-0.880482	0.0434	0.362385	no
34.6276	40.9245	0.241043	0.896488	0.04345	0.362385	no
18.926	22.7986	0.268576	0.894106	0.04355	0.362648	no
14.2981	11.866	-0.268988	-0.876058	0.0437	0.362648	no
9.22158	7.61429	-0.276304	-0.895181	0.04365	0.362648	no
1.84979	1.37766	-0.425147	-0.895423	0.0437	0.362648	no
4.11303	5.28563	0.361872	0.903485	0.04365	0.362648	no
25.9073	30.6353	0.241836	0.895442	0.04355	0.362648	no
3.37166	2.76701	-0.285129	-0.893167	0.04365	0.362648	no
13.1288	11.0189	-0.252758	-0.895073	0.04375	0.362803	no
14.4734	11.5167	-0.329672	-0.911608	0.04385	0.363269	no
2.76697	3.86564	0.482403	0.890607	0.0439	0.363269	no
8.35674	10.6345	0.347741	0.896326	0.0439	0.363269	no

15.0297	18.4814	0.298258	0.904632	0.04405	0.364251	no
19.0539	15.4214	-0.305153	-0.910908	0.04425	0.365645	no
5.90076	4.79969	-0.29796	-0.901247	0.0443	0.365798	no
25.1945	30.2381	0.263259	0.905034	0.04465	0.367643	no
6.46808	9.80268	0.599839	0.900185	0.04465	0.367643	no
103.08	121.846	0.241298	0.893986	0.04465	0.367643	no
24.644	29.1236	0.240952	0.900797	0.04475	0.367684	no
48.7459	40.6253	-0.262904	-0.900144	0.04475	0.367684	no
26.6633	22.6317	-0.236514	-0.884343	0.0448	0.367835	no
20.1956	23.8185	0.238042	0.892061	0.04495	0.368806	no
113.995	136.85	0.263633	0.901826	0.045	0.368956	no
7.49678	6.06338	-0.30615	-0.894439	0.04505	0.369105	no
39.3061	33.2303	-0.242252	-0.893114	0.04515	0.369403	no
16.1186	13.4579	-0.260272	-0.87173	0.04515	0.369403	no
53.83	63.5214	0.238832	0.891941	0.04525	0.369849	no
2.37202	4.54173	0.937126	0.903703	0.0453	0.369849	no
12.0281	42.4391	1.81899	1.66417	0.0453	0.369849	no
4.49158	5.90186	0.393946	0.910331	0.0454	0.370145	no
10.7035	12.8154	0.259802	0.89251	0.0454	0.370145	no
64.258	78.4934	0.288694	0.891949	0.04565	0.371662	no
13.5623	11.2594	-0.268474	-0.883021	0.04565	0.371662	no
5.23163	4.15742	-0.331572	-0.895087	0.04575	0.372215	no
7.77394	6.19701	-0.327075	-0.899198	0.04595	0.373059	no
45.8125	38.3373	-0.256994	-0.896355	0.04595	0.373059	no
5.13257	4.10347	-0.322837	-0.890811	0.04595	0.373059	no
37.1383	31.4398	-0.240319	-0.888474	0.046	0.373204	no
25.8056	30.5702	0.244445	0.899393	0.04605	0.373349	no
52.7805	62.6163	0.246532	0.890311	0.0461	0.373494	no
2.80305	3.64362	0.378377	0.887008	0.04635	0.374996	no
134.11	161.837	0.271127	0.878937	0.0466	0.376756	no
4.86836	3.7953	-0.359225	-0.898211	0.0467	0.377302	no
6.10522	4.82728	-0.338833	-0.889263	0.04675	0.377444	no
11.3189	9.17072	-0.303621	-0.882384	0.04685	0.377464	no
20.183	23.9825	0.248845	0.890348	0.04685	0.377464	no
515.664	632.921	0.295593	0.892943	0.04685	0.377464	no
1.86213	1.39903	-0.412524	-0.873209	0.047	0.37841	no
21.1482	17.547	-0.269304	-0.857877	0.0471	0.378429	no
16.3302	12.2036	-0.420239	-0.889705	0.0471	0.378429	no
490.08	411.99	-0.250409	-0.888831	0.04705	0.378429	no
210.411	252.325	0.262071	0.877657	0.0472	0.378708	no
3.64305	4.43581	0.28405	0.866225	0.0472	0.378708	no
8.06668	6.68984	-0.270003	-0.884229	0.0473	0.378987	no
9.97863	12.0908	0.276991	0.880305	0.04745	0.379927	no
4.52768	5.61185	0.309705	0.873822	0.04755	0.380465	no
22.4557	19.0228	-0.239348	-0.890745	0.04765	0.381003	no
23.0709	27.8284	0.270478	0.890648	0.0477	0.381141	no
12.7225	15.3312	0.269086	0.873216	0.04775	0.381278	no

6.73895	5.02255	-0.424104	-0.875412	0.04805	0.38341	no
6.16388	5.03934	-0.290603	-0.884152	0.0481	0.383546	no
6.13893	9.10976	0.569424	0.890473	0.04825	0.384478	no
23.6805	27.9589	0.239609	0.877199	0.0483	0.384612	no
34.0365	40.3112	0.244099	0.885053	0.0484	0.384881	no
3.33055	4.62239	0.472881	0.882778	0.0484	0.384881	no
13.3138	10.527	-0.338831	-0.884852	0.04845	0.385015	no
2.39215	1.95615	-0.290294	-0.886237	0.04865	0.38634	no
9.31029	7.84649	-0.246778	-0.878512	0.0487	0.386473	no
81.9551	97.1416	0.245256	0.870768	0.04875	0.386606	no
40.0979	47.4777	0.243724	0.887135	0.0488	0.386738	no
4.54222	6.29633	0.471114	0.874544	0.04885	0.386871	no
83.0233	70.4712	-0.236482	-0.869153	0.0489	0.387003	no
8.8433	7.38343	-0.260295	-0.856371	0.049	0.387003	no
6.11773	5.16657	-0.243789	-0.872013	0.049	0.387003	no
4.30481	3.58836	-0.262624	-0.871429	0.04905	0.387135	no
16.0463	13.5682	-0.24201	-0.883565	0.0491	0.387267	no
4.41218	3.42964	-0.363433	-0.879701	0.0493	0.387529	no
71.9074	87.7656	0.287516	0.87201	0.0493	0.387529	no
58.1457	68.4637	0.235665	0.883149	0.0493	0.387529	no
7.27801	9.48429	0.381996	0.887588	0.0492	0.387529	no
11.591	13.6827	0.239343	0.873441	0.04925	0.387529	no
85.1572	100.381	0.237288	0.882407	0.04945	0.388445	no
9.29355	7.67914	-0.275285	-0.871718	0.04955	0.388967	no
8.59453	10.9414	0.348305	0.88467	0.04965	0.389223	no
10.4558	8.86685	-0.237807	-0.864674	0.04975	0.389223	no
47.4377	40.1756	-0.239715	-0.878272	0.04975	0.389223	no
15.3889	13.0674	-0.235915	-0.874227	0.04975	0.389223	no
5.91963	4.93255	-0.263173	-0.849745	0.0497	0.389223	no
20.0499	17.0002	-0.238042	-0.880953	0.0498	0.389352	no
309.82	372.949	0.267548	0.871399	0.0499	0.389471	no
12.4051	10.3451	-0.261986	-0.871389	0.05005	0.389471	no
3.75984	2.49074	-0.594101	-0.869667	0.04985	0.389471	no
163.161	193.183	0.24367	0.870025	0.05005	0.389471	no
9.21077	7.70551	-0.25743	-0.865582	0.05005	0.389471	no
1.1012	1.64877	0.582305	0.874503	0.04995	0.389471	no
2.91447	2.25068	-0.372873	-0.86643	0.04995	0.389471	no
7.01027	8.44118	0.267975	0.862997	0.05015	0.389576	no
96.3814	113.572	0.236777	0.873667	0.05045	0.389576	no
20.8395	24.554	0.236636	0.869598	0.05045	0.389576	no
8.87076	10.6869	0.268719	0.871439	0.0503	0.389576	no
5.01884	6.38804	0.348021	0.885155	0.0501	0.389576	no
11.5593	9.52573	-0.279148	-0.865025	0.0506	0.389576	no
37.1937	43.8962	0.23904	0.879979	0.05035	0.389576	no
18.1155	21.7302	0.262476	0.866865	0.0503	0.389576	no
18.9629	16.0523	-0.240395	-0.870941	0.0505	0.389576	no
68.0018	80.1866	0.237789	0.878024	0.05055	0.389576	no

1.83383	2.52555	0.461732	0.878651	0.0505	0.389576	no
1.51319	2.16896	0.519411	0.872301	0.0502	0.389576	no
12.0709	15.3612	0.347763	0.88283	0.05035	0.389576	no
3.46379	2.8333	-0.289869	-0.859379	0.0505	0.389576	no
2.43308	1.67592	-0.537833	-0.864837	0.0506	0.389576	no
10.4675	8.35892	-0.324524	-0.876435	0.05035	0.389576	no
39.4398	33.393	-0.240106	-0.861633	0.0509	0.391234	no
44.5303	37.8729	-0.233621	-0.871368	0.05095	0.391234	no
24.7306	29.1203	0.235726	0.872418	0.05095	0.391234	no
50.5327	59.2558	0.229738	0.85631	0.05105	0.391484	no
1.42952	2.16836	0.601069	0.881366	0.05105	0.391484	no
3.74493	2.9223	-0.357832	-0.869291	0.05125	0.391974	no
47.7533	55.9392	0.228258	0.85768	0.0513	0.391974	no
50.0888	58.9852	0.235865	0.871189	0.05135	0.391974	no
1.91319	1.1339	-0.754679	-0.87653	0.05135	0.391974	no
13.8751	16.5777	0.256751	0.861996	0.0512	0.391974	no
19.324	22.9808	0.250036	0.864822	0.05135	0.391974	no
2.66726	1.57233	-0.762449	-0.889343	0.05135	0.391974	no
35.6923	41.9725	0.233833	0.861996	0.05165	0.394005	no
4.50217	5.7104	0.342972	0.871563	0.0517	0.394128	no
31.4534	26.7278	-0.234875	-0.871686	0.05175	0.39425	no
8.66505	6.99208	-0.309486	-0.869947	0.05185	0.394753	no
7.37897	9.41922	0.352188	0.875302	0.0519	0.394875	no
19.6805	23.2517	0.240567	0.86016	0.052	0.395118	no
27.5719	23.4656	-0.23265	-0.870478	0.052	0.395118	no
2.61096	1.93921	-0.429113	-0.855281	0.05215	0.395999	no
10.2512	12.0599	0.234424	0.861508	0.05255	0.398256	no
264.472	321.533	0.281856	0.868195	0.0525	0.398256	no
6.85651	5.83139	-0.233635	-0.861975	0.05255	0.398256	no
18.6144	15.7845	-0.237911	-0.858385	0.05305	0.398922	no
17.3594	20.853	0.26454	0.856611	0.05295	0.398922	no
20.8025	17.4314	-0.25507	-0.874349	0.0528	0.398922	no
47.9906	56.4591	0.234454	0.866455	0.0527	0.398922	no
1.1983	1.91825	0.678801	0.873576	0.05295	0.398922	no
1.91096	1.35622	-0.494709	-0.849327	0.053	0.398922	no
5.77585	4.64495	-0.314371	-0.856324	0.0529	0.398922	no
11.7582	14.3926	0.291658	0.860015	0.05285	0.398922	no
1.15386	1.5617	0.436648	0.872312	0.05305	0.398922	no
145.489	171.771	0.239571	0.867351	0.053	0.398922	no
4.68579	3.69244	-0.343717	-0.872595	0.05285	0.398922	no
3.21521	2.40604	-0.418248	-0.872175	0.05315	0.399416	no
1.92084	1.21656	-0.65893	-0.878126	0.0533	0.400025	no
33.877	40.2101	0.247253	0.854218	0.0533	0.400025	no
28.7049	34.1972	0.252583	0.855669	0.05355	0.401383	no
7.45138	9.50033	0.35047	0.860909	0.0536	0.401499	no
43.3556	50.9951	0.234139	0.864986	0.0537	0.40173	no
2.37206	1.64485	-0.52818	-0.858636	0.0537	0.40173	no

67.2181	57.2806	-0.230803	-0.858427	0.05375	0.401845	no
1.47603	2.24323	0.603859	0.866894	0.0539	0.402449	no
69.6514	59.0705	-0.237713	-0.86558	0.0539	0.402449	no
16.2448	13.8576	-0.2293	-0.852188	0.05395	0.402563	no
11.5687	14.1185	0.287356	0.854145	0.054	0.402678	no
25.3812	30.2908	0.255121	0.8685	0.0541	0.403165	no
12.0421	10.208	-0.238386	-0.869941	0.05415	0.403279	no
1.66388	2.4553	0.561345	0.872646	0.0544	0.403814	no
6.8698	8.29049	0.271189	0.848725	0.0545	0.403814	no
1.9792	1.32838	-0.575247	-0.868094	0.05445	0.403814	no
6.89448	9.95738	0.530325	0.863036	0.05445	0.403814	no
50.3656	58.8828	0.225407	0.846427	0.0545	0.403814	no
23.1322	19.4364	-0.251141	-0.845042	0.0545	0.403814	no
119.423	140.169	0.231094	0.860879	0.0543	0.403814	no
2.71824	2.07281	-0.391087	-0.852411	0.05445	0.403814	no
13.2269	11.0622	-0.257838	-0.851122	0.05475	0.404233	no
18.1826	15.2964	-0.24937	-0.852663	0.05475	0.404233	no
5.11776	6.78276	0.406359	0.8628	0.05465	0.404233	no
19.3912	24.296	0.325315	0.851622	0.0547	0.404233	no
2.23477	1.69555	-0.398374	-0.856854	0.05475	0.404233	no
12.825	15.5945	0.282078	0.84767	0.0548	0.404233	no
29.7993	34.9061	0.228201	0.857799	0.0549	0.404713	no
28.3616	24.1707	-0.230682	-0.846657	0.055	0.405193	no
13.819	11.3502	-0.28393	-0.84231	0.05515	0.406041	no
6.11497	5.15313	-0.246896	-0.843568	0.05525	0.40652	no
21.0751	27.156	0.365735	0.863505	0.0553	0.40663	no
161.552	191.66	0.246549	0.848036	0.05535	0.40674	no
17.8134	15.1715	-0.231594	-0.858839	0.05545	0.407218	no
76.3812	89.5825	0.23	0.854119	0.0555	0.407327	no
3.68439	2.63225	-0.485128	-0.8591	0.05555	0.407437	no
2.54935	1.81317	-0.491617	-0.840025	0.0556	0.407546	no
19.4713	23.4063	0.265548	0.849656	0.05565	0.407656	no
23.0194	27.1374	0.237432	0.84304	0.0557	0.407765	no
142.366	169.357	0.250462	0.845121	0.0558	0.4082	no
28.5651	23.5123	-0.280837	-0.847475	0.05585	0.4082	no
10.7467	9.16583	-0.229551	-0.843588	0.0559	0.4082	no
11.9752	10.1601	-0.237146	-0.861837	0.0559	0.4082	no
27.8752	32.9885	0.242983	0.860043	0.05625	0.408298	no
35.7813	41.9711	0.230189	0.852403	0.05615	0.408298	no
4.87448	3.23201	-0.592814	-0.859648	0.05615	0.408298	no
13.6097	11.6524	-0.224012	-0.838686	0.0562	0.408298	no
12.7946	15.2131	0.249787	0.851674	0.05615	0.408298	no
7.40031	6.14952	-0.267111	-0.848413	0.0563	0.408298	no
12.5837	10.6962	-0.234462	-0.847469	0.0563	0.408298	no
12.3248	10.1407	-0.281414	-0.844859	0.0562	0.408298	no
13.4132	11.4433	-0.229154	-0.853622	0.05625	0.408298	no
10.5199	8.98039	-0.228277	-0.850647	0.05625	0.408298	no

51.3848	43.7853	-0.230897	-0.856995	0.056	0.408298	no
7.29468	8.88925	0.285215	0.836594	0.05635	0.408406	no
20.4153	24.9247	0.287927	0.870001	0.0565	0.409238	no
28.2156	33.183	0.23395	0.843113	0.05655	0.409344	no
10.2303	7.93626	-0.366318	-0.85259	0.0567	0.410175	no
7.29781	9.09684	0.317902	0.856716	0.0568	0.410642	no
10.9328	14.2246	0.379734	0.857458	0.05685	0.410748	no
14.1065	17.3382	0.297602	0.848756	0.057	0.410916	no
11.7198	13.8103	0.2368	0.847277	0.057	0.410916	no
22.6776	19.1153	-0.246543	-0.836262	0.05705	0.410916	no
10.5054	8.75891	-0.262308	-0.83635	0.05695	0.410916	no
5.57463	4.37426	-0.349836	-0.845915	0.05705	0.410916	no
23.4539	27.5138	0.230327	0.847726	0.0572	0.411486	no
41.8113	50.2486	0.265189	0.848014	0.0572	0.411486	no
45.5729	38.9677	-0.225897	-0.843454	0.05725	0.411591	no
35.1342	41.4839	0.239673	0.8402	0.05745	0.411664	no
41.9516	33.3522	-0.330942	-0.899401	0.0574	0.411664	no
12.7268	15.3583	0.271145	0.837837	0.05755	0.411664	no
1.52872	1.23358	-0.309467	-0.843248	0.05765	0.411664	no
6.98266	5.87697	-0.248703	-0.831996	0.05755	0.411664	no
2.43473	3.72396	0.613074	0.85694	0.05755	0.411664	no
15.113	12.788	-0.241	-0.849829	0.05735	0.411664	no
18.8797	15.9076	-0.247121	-0.846308	0.0575	0.411664	no
7.33818	5.68559	-0.368112	-0.854744	0.05765	0.411664	no
48.5432	40.9175	-0.246554	-0.844488	0.0576	0.411664	no
4.36701	3.50763	-0.31615	-0.857439	0.0575	0.411664	no
2.26316	1.55545	-0.541007	-0.829696	0.0577	0.411768	no
15.793	13.4052	-0.236494	-0.858258	0.05785	0.412332	no
7.90027	6.32339	-0.321203	-0.844784	0.05785	0.412332	no
5.93041	4.9739	-0.253755	-0.855197	0.0579	0.412435	no
29.5701	25.2228	-0.229412	-0.849098	0.05805	0.413251	no
10.8582	13.4567	0.309544	0.852912	0.0582	0.413305	no
0.957222	1.84266	0.944867	0.843489	0.0582	0.413305	no
7.93543	10.011	0.335206	0.849133	0.0582	0.413305	no
1.29031	1.75214	0.441405	0.821065	0.05835	0.414117	no
50.3326	62.4312	0.310775	0.844404	0.05855	0.41503	no
9.93896	8.22397	-0.27326	-0.839249	0.0586	0.415131	no
2.71787	2.13181	-0.350402	-0.854898	0.0588	0.41518	no
5.27604	4.26135	-0.308145	-0.849727	0.059	0.41518	no
3.29934	3.97424	0.2685	0.835727	0.05895	0.41518	no
6.56926	8.82918	0.42655	0.833311	0.05895	0.41518	no
63.0268	74.051	0.232555	0.859812	0.0588	0.41518	no
4.9337	4.0502	-0.28468	-0.839732	0.0589	0.41518	no
44.0402	51.8627	0.235876	0.846012	0.0589	0.41518	no
12.9751	11.0551	-0.231035	-0.840555	0.05875	0.41518	no
8.61973	7.15036	-0.269627	-0.842923	0.0589	0.41518	no
45.1299	38.3253	-0.235786	-0.849066	0.05875	0.41518	no

10.8653	9.24362	-0.233194	-0.842404	0.059	0.41518	no
26.839	22.9897	-0.22334	-0.830581	0.05905	0.41528	no
6.50649	10.3066	0.663622	0.825153	0.05935	0.41588	no
7.45706	6.27358	-0.249319	-0.849496	0.05925	0.41588	no
18.083	15.4162	-0.230188	-0.841479	0.05925	0.41588	no
27.9885	33.7492	0.270017	0.842992	0.05935	0.41588	no
28.3738	24.2103	-0.228938	-0.845718	0.05925	0.41588	no
3.31197	2.4482	-0.435968	-0.843994	0.0593	0.41588	no
10.3591	8.68852	-0.253715	-0.836642	0.05945	0.416329	no
20.0611	17	-0.238866	-0.831563	0.0595	0.416428	no
87.316	102.244	0.227705	0.835092	0.05965	0.416725	no
61.0633	71.6875	0.231416	0.847477	0.05965	0.416725	no
10.7747	9.13536	-0.238112	-0.843243	0.05965	0.416725	no
3.09315	2.10838	-0.552943	-0.846537	0.0597	0.416824	no
56.6938	46.8938	-0.273793	-0.837737	0.0598	0.417271	no
14.8116	12.6451	-0.228156	-0.851596	0.05995	0.418067	no
6.2038	4.74765	-0.385938	-0.84492	0.06005	0.418513	no
6.00139	4.73209	-0.342821	-0.848855	0.06035	0.420292	no
9.01997	7.60012	-0.2471	-0.830239	0.06045	0.420292	no
10.0456	12.4337	0.307693	0.839756	0.06045	0.420292	no
8.46271	7.21792	-0.229538	-0.829792	0.06045	0.420292	no
6.88832	8.24885	0.260041	0.83361	0.0606	0.420365	no
17.3345	14.852	-0.222993	-0.831024	0.06055	0.420365	no
2.90582	2.37503	-0.291	-0.835486	0.06065	0.420365	no
9.38527	11.7838	0.328335	0.835053	0.06075	0.420365	no
38.8364	46.0022	0.244295	0.826973	0.06055	0.420365	no
3.04442	2.39565	-0.345752	-0.836561	0.06075	0.420365	no
3.4506	4.61841	0.420551	0.851157	0.06075	0.420365	no
17.8965	15.241	-0.23172	-0.835304	0.06075	0.420365	no
51.1992	42.9745	-0.252641	-0.831172	0.06095	0.421498	no
51.3549	43.8349	-0.228419	-0.833417	0.06115	0.422128	no
6.15558	7.46558	0.27836	0.834267	0.06115	0.422128	no
2.54566	1.90915	-0.415112	-0.835537	0.0615	0.423537	no
4.92037	3.80485	-0.370927	-0.832138	0.06145	0.423537	no
1.25816	1.72614	0.456233	0.815223	0.0615	0.423537	no
7.80435	5.91992	-0.398699	-0.832534	0.06155	0.42363	no
72.6832	85.2174	0.229524	0.839692	0.06165	0.423816	no
13.0738	15.6666	0.261015	0.82667	0.06165	0.423816	no
1.26044	1.84443	0.549247	0.829322	0.0617	0.423909	no
6.26422	4.73707	-0.403139	-0.822602	0.0619	0.425032	no
1.44592	0.96795	-0.578978	-0.847057	0.06205	0.42581	no
2.29832	1.80995	-0.344627	-0.846034	0.06215	0.425834	no
1.04817	1.44564	0.463835	0.832103	0.0621	0.425834	no
7.15897	5.90069	-0.278869	-0.829188	0.0622	0.425834	no
9.96604	8.46166	-0.236081	-0.849362	0.0622	0.425834	no
2.26961	1.77017	-0.358559	-0.831026	0.06225	0.425925	no
23.4336	20.0356	-0.226015	-0.819568	0.0624	0.426449	no

42.3017	50.4024	0.252779	0.82615	0.0625	0.426881	no
37.6224	32.2721	-0.221303	-0.82921	0.06265	0.427654	no
19.9764	23.4097	0.228811	0.823825	0.0627	0.427744	no
146.692	185.524	0.338822	0.825341	0.0628	0.427763	no
55.7412	65.0435	0.222661	0.83687	0.06285	0.427763	no
28.3173	35.0263	0.306753	0.829618	0.06285	0.427763	no
35.2537	41.172	0.22389	0.828191	0.06295	0.428193	no
15.3442	19.3409	0.333961	0.834907	0.063	0.428282	no
7.40581	6.1556	-0.266759	-0.819236	0.0632	0.428388	no
89.488	104.491	0.223617	0.820476	0.0632	0.428388	no
19.3848	16.6374	-0.220496	-0.820095	0.06315	0.428388	no
14.7401	12.5732	-0.2294	-0.83321	0.0632	0.428388	no
8.4959	6.64123	-0.355316	-0.828853	0.0633	0.428816	no
17.3979	14.082	-0.305055	-0.827423	0.06335	0.428905	no
58.0372	49.7515	-0.222237	-0.834053	0.0636	0.430096	no
4.01547	3.32748	-0.271137	-0.810185	0.0636	0.430096	no
6.23536	5.06701	-0.299338	-0.822958	0.06365	0.430184	no
15.7749	18.7103	0.246196	0.819103	0.06375	0.430359	no
14.0572	17.2119	0.292101	0.828829	0.06375	0.430359	no
2.98227	2.34938	-0.34413	-0.832013	0.06385	0.430784	no
2.13724	1.70585	-0.325261	-0.818988	0.06405	0.430882	no
1.5064	1.1582	-0.37922	-0.826556	0.064	0.430882	no
34.9776	29.6267	-0.239535	-0.827703	0.06395	0.430882	no
66.5326	78.6651	0.241661	0.817503	0.06405	0.430882	no
7.64125	9.40207	0.29917	0.828831	0.06395	0.430882	no
5.20493	4.36305	-0.25454	-0.814438	0.06425	0.431727	no
71.2198	60.532	-0.234579	-0.816551	0.06425	0.431727	no
1248.8	1549.85	0.311581	0.834931	0.0644	0.432485	no
1.1806	1.6461	0.479534	0.838117	0.06445	0.432571	no
31.5367	26.7373	-0.238179	-0.823905	0.0645	0.432656	no
20.06	16.8178	-0.254331	-0.826147	0.0648	0.434418	no
81.7255	95.6729	0.227324	0.815391	0.065	0.434424	no
19.1381	22.3525	0.223992	0.824409	0.0649	0.434424	no
13.4986	11.5137	-0.229458	-0.820924	0.0651	0.434424	no
15.5784	13.2847	-0.229781	-0.828376	0.065	0.434424	no
270.172	321.955	0.252982	0.825344	0.06485	0.434424	no
25.2518	29.5199	0.225302	0.823082	0.06505	0.434424	no
18.753	15.9797	-0.230888	-0.812483	0.06495	0.434424	no
37.4251	43.6589	0.222271	0.819633	0.0651	0.434424	no
56.8276	66.2199	0.220672	0.825269	0.0652	0.434841	no
1.6561	2.28499	0.4644	0.823205	0.0653	0.435009	no
8.05941	6.85801	-0.232885	-0.819785	0.0653	0.435009	no
25.4694	29.747	0.22398	0.825669	0.06535	0.435092	no
1.57045	1.16405	-0.432016	-0.830715	0.0654	0.435176	no
4.82033	3.88482	-0.311286	-0.820241	0.06545	0.435259	no
20.1222	24.7323	0.297604	0.824858	0.0655	0.435343	no
77.5098	91.7442	0.243237	0.814306	0.06565	0.43609	no

247.031	295.134	0.256677	0.820521	0.0657	0.436173	no
33.5745	39.6182	0.238798	0.813393	0.06575	0.436256	no
53.1637	61.958	0.220849	0.828685	0.0659	0.436752	no
71.5684	83.8354	0.228236	0.81382	0.0659	0.436752	no
27.9455	23.7729	-0.233299	-0.827958	0.066	0.437165	no
94.2519	109.609	0.217769	0.815978	0.06615	0.43766	no
2.88037	2.14892	-0.422645	-0.826011	0.06615	0.43766	no
102.567	88.0361	-0.220403	-0.817441	0.06645	0.438396	no
15.6158	13.3874	-0.222129	-0.824989	0.06645	0.438396	no
10.9828	13.2558	0.271376	0.81994	0.0664	0.438396	no
13.5742	11.2481	-0.271184	-0.80552	0.0664	0.438396	no
15.1028	12.9701	-0.219624	-0.822031	0.0663	0.438396	no
19.0885	15.9927	-0.255289	-0.805353	0.0665	0.438477	no
7.35522	6.12919	-0.263073	-0.816566	0.06665	0.439217	no
29.8296	25.4714	-0.227864	-0.821824	0.06675	0.439378	no
28.2334	24.0951	-0.228663	-0.811378	0.06675	0.439378	no
15.962	13.7029	-0.220156	-0.814284	0.067	0.440524	no
1.55565	1.18851	-0.388363	-0.797345	0.06705	0.440603	no
47.1399	55.2378	0.228708	0.812987	0.0671	0.440683	no
1.87921	2.85794	0.604848	0.822774	0.0672	0.44109	no
44.5666	54.0206	0.277549	0.814998	0.0673	0.441248	no
10.558	8.98496	-0.232747	-0.819299	0.0673	0.441248	no
1.54681	0.982381	-0.654938	-0.822828	0.06745	0.441982	no
41.9441	48.7633	0.217326	0.812211	0.0676	0.442466	no
6.5829	5.43512	-0.27641	-0.804868	0.0676	0.442466	no
8.66053	7.28636	-0.249258	-0.802872	0.06775	0.443198	no
1.25639	2.38617	0.925406	0.79981	0.0678	0.443275	no
16.3319	14.006	-0.221647	-0.802744	0.0679	0.443507	no
3.05954	2.2522	-0.441979	-0.800208	0.06795	0.443507	no
7.36409	8.81212	0.258981	0.803347	0.06795	0.443507	no
73.205	62.7996	-0.221187	-0.824987	0.06805	0.443911	no
1.5661	2.13391	0.44632	0.802599	0.0681	0.443969	no
1.90887	1.36625	-0.482495	-0.8044	0.06815	0.443969	no
18.1309	21.0854	0.217794	0.800495	0.06825	0.443969	no
16.3199	13.8848	-0.233125	-0.802225	0.0682	0.443969	no
45.0106	38.4798	-0.226166	-0.801377	0.06825	0.443969	no
10.3863	8.67311	-0.260063	-0.817958	0.0684	0.444447	no
9.19637	11.3132	0.29887	0.819773	0.0684	0.444447	no
17.2541	14.7842	-0.222885	-0.810316	0.06855	0.444924	no
9.06739	10.8793	0.262827	0.815564	0.06855	0.444924	no
21.2725	25.289	0.249521	0.814609	0.0687	0.445151	no
58.3574	67.8928	0.218344	0.812119	0.0687	0.445151	no
113.888	97.9295	-0.2178	-0.809237	0.0687	0.445151	no
1.35273	2.01942	0.57806	0.819124	0.0688	0.445551	no
1.82601	2.43164	0.413235	0.792092	0.06885	0.445626	no
9.76534	8.28348	-0.237433	-0.817975	0.0689	0.445702	no
10.7793	9.20511	-0.227754	-0.806699	0.06895	0.445777	no

15.4023	17.9804	0.223278	0.810051	0.069	0.445852	no
5.02794	4.04257	-0.314696	-0.81002	0.0691	0.445882	no
16.4905	14.2184	-0.213874	-0.793577	0.06925	0.445882	no
11.0537	12.8446	0.216631	0.792778	0.0692	0.445882	no
11.7872	9.95598	-0.24359	-0.79045	0.06935	0.445882	no
5.80374	4.59096	-0.338189	-0.816586	0.0693	0.445882	no
17.924	20.988	0.227667	0.814206	0.0692	0.445882	no
85.904	99.7808	0.216036	0.811169	0.0692	0.445882	no
8.10888	6.28508	-0.367571	-0.81309	0.06935	0.445882	no
7.80624	6.70548	-0.219288	-0.804533	0.0697	0.446157	no
2.1304	1.50268	-0.503583	-0.811459	0.06965	0.446157	no
1.86045	1.01999	-0.867092	-0.944031	0.0697	0.446157	no
2.97213	2.18524	-0.443707	-0.814866	0.06965	0.446157	no
493.21	423.714	-0.219111	-0.805266	0.06955	0.446157	no
10.1235	8.56186	-0.241714	-0.813373	0.0696	0.446157	no
24.452	21.0088	-0.218957	-0.807354	0.06965	0.446157	no
1.16473	2.05553	0.819513	0.808709	0.0695	0.446157	no
1.83984	2.77791	0.594422	0.811487	0.0699	0.446945	no
15.2061	12.5661	-0.275108	-0.805818	0.0701	0.447484	no
17.776	20.9123	0.234416	0.806856	0.0701	0.447484	no
15.5149	18.9642	0.289622	0.807804	0.0701	0.447485	no
3.5908	2.95153	-0.282843	-0.806542	0.07015	0.447558	no
8.84047	10.3646	0.229473	0.807621	0.0703	0.448269	no
8.70712	7.30818	-0.252682	-0.811907	0.07035	0.448341	no
6.09136	4.01224	-0.602357	-0.822314	0.07045	0.448733	no
80.2818	95.8509	0.25572	0.793279	0.0705	0.448805	no
65.3405	75.8935	0.215997	0.807525	0.07055	0.448877	no
83.8417	70.713	-0.245694	-0.78939	0.0707	0.449094	no
4.08036	5.28664	0.373655	0.806928	0.0707	0.449094	no
20.0333	23.2519	0.214942	0.797565	0.0708	0.449483	no
15.8907	13.3925	-0.246761	-0.799934	0.07095	0.449811	no
13.8342	11.5478	-0.260619	-0.801993	0.071	0.449811	no
3.00314	4.60734	0.617465	0.818641	0.0712	0.449811	no
2.48107	1.8987	-0.385945	-0.813089	0.07105	0.449811	no
3.85258	5.17977	0.427066	0.799794	0.07115	0.449811	no
9.62731	8.07289	-0.254047	-0.807287	0.07115	0.449811	no
15.442	19.8388	0.361467	0.822088	0.0712	0.449811	no
7.17433	5.96011	-0.267505	-0.801449	0.0712	0.449811	no
6.95417	5.73045	-0.279232	-0.800048	0.0712	0.449811	no
14.0307	12.0293	-0.222035	-0.803179	0.0714	0.450095	no
22.18	18.9949	-0.223649	-0.810923	0.07135	0.450095	no
12.9915	15.5766	0.26181	0.797851	0.0713	0.450095	no
26.4352	31.2436	0.241105	0.812135	0.0714	0.450095	no
4.35764	3.62957	-0.263749	-0.806693	0.07165	0.451426	no
9.21632	10.9729	0.251689	0.80083	0.0718	0.452126	no
15.5065	13.3691	-0.213975	-0.798228	0.07195	0.452825	no
3.3237	4.2001	0.337634	0.804253	0.0722	0.452995	no

1.54928	2.3126	0.577922	0.79867	0.07215	0.452995	no
26.4393	31.1916	0.238476	0.805032	0.07215	0.452995	no
2.67288	1.7348	-0.623627	-0.793779	0.0722	0.452995	no
11.5541	9.58826	-0.269067	-0.79932	0.0721	0.452995	no
14.2202	16.5385	0.217891	0.804336	0.07225	0.452995	no
879.654	1072.94	0.286556	0.795293	0.07225	0.452995	no
4.73927	3.98565	-0.24985	-0.786496	0.07235	0.453133	no
5.44548	7.00877	0.364102	0.790121	0.07235	0.453133	no
5.25366	6.46461	0.29924	0.799282	0.07245	0.453515	no
95.4733	112.206	0.232975	0.797056	0.07255	0.453652	no
13.5282	11.6253	-0.218705	-0.80143	0.07255	0.453652	no
12.2055	10.2679	-0.24939	-0.790417	0.07265	0.453789	no
1.93171	2.88903	0.580706	0.800639	0.07265	0.453789	no
4.92521	3.99308	-0.302686	-0.805101	0.0727	0.453857	no
30.0821	36.4742	0.277971	0.798068	0.07275	0.453926	no
39.3506	45.8101	0.219279	0.809802	0.07285	0.454022	no
80.9104	69.236	-0.224802	-0.792737	0.07285	0.454022	no
10.9869	8.93416	-0.298378	-0.80874	0.07295	0.454022	no
4.73209	3.49413	-0.437545	-0.79816	0.0729	0.454022	no
50.5698	58.762	0.216608	0.794105	0.073	0.454022	no
29.3945	34.2273	0.219598	0.79225	0.073	0.454022	no
5.96512	4.86579	-0.293877	-0.81058	0.07315	0.454712	no
2.79047	2.13559	-0.385875	-0.794645	0.07325	0.45509	no
2.40082	1.92875	-0.315864	-0.802571	0.0735	0.455912	no
24.5451	29.818	0.280745	0.80439	0.0735	0.455912	no
70.9779	82.6922	0.220381	0.805355	0.07345	0.455912	no
36.7531	42.6262	0.213877	0.795917	0.0736	0.456289	no
82.0383	95.2195	0.214958	0.801201	0.0737	0.456665	no
2.06382	1.61897	-0.350238	-0.814264	0.07385	0.456863	no
13.5974	11.6394	-0.224321	-0.802178	0.0738	0.456863	no
59.2338	68.9215	0.218532	0.800049	0.07385	0.456863	no
19.6357	24.3531	0.310629	0.802605	0.0739	0.456929	no
38.148	32.8167	-0.217179	-0.791867	0.074	0.457304	no
1.99561	2.8776	0.528035	0.816673	0.07405	0.45737	no
2.95024	2.2911	-0.364791	-0.798914	0.0741	0.457436	no
23.0964	28.8886	0.32283	0.792874	0.0742	0.457567	no
1.98997	2.85463	0.520556	0.802153	0.0742	0.457567	no
58.9187	68.3715	0.214671	0.798975	0.0744	0.458557	no
10.4647	8.90579	-0.232709	-0.798889	0.0745	0.458929	no
26.3072	31.0053	0.237058	0.794167	0.07485	0.46011	no
28.7798	33.4752	0.218037	0.795474	0.0748	0.46011	no
4.36053	3.42206	-0.349637	-0.800226	0.07485	0.46011	no
6.89987	10.068	0.54513	0.803729	0.07475	0.46011	no
23.261	19.7383	-0.236915	-0.796757	0.07495	0.460237	no
1.77724	2.80209	0.65686	0.796153	0.07495	0.460237	no
5.48076	4.57026	-0.262101	-0.794293	0.0754	0.46178	no
11.4806	14.2236	0.309088	0.811041	0.07535	0.46178	no

49.9944	58.168	0.218459	0.78619	0.0753	0.46178	no
11.0972	9.54592	-0.217243	-0.79233	0.0754	0.46178	no
16.8067	19.9156	0.244856	0.794274	0.07555	0.462455	no
3.46034	4.45098	0.363206	0.80532	0.07575	0.462461	no
32.0864	23.3428	-0.458987	-0.801084	0.07575	0.462461	no
483.475	562.637	0.218764	0.786454	0.07565	0.462461	no
3.47731	4.8876	0.491152	0.80416	0.0757	0.462461	no
2.58831	1.90476	-0.442401	-0.798918	0.07585	0.462585	no
11.4342	9.69858	-0.237505	-0.798488	0.07585	0.462585	no
13.0645	11.2328	-0.217942	-0.7906	0.07595	0.462952	no
13.3198	17.2006	0.368885	0.797474	0.07605	0.463076	no
22.8665	26.5175	0.213705	0.794861	0.07605	0.463076	no
11.1063	13.1032	0.238539	0.781166	0.07625	0.463808	no
24.0592	28.0107	0.219388	0.784379	0.07625	0.463808	no
11.6459	9.72124	-0.260614	-0.792438	0.07635	0.46393	no
130.026	151.486	0.220383	0.794721	0.07645	0.464295	no
26.7657	31.4093	0.230805	0.787187	0.07675	0.465144	no
46.8458	54.6165	0.221416	0.784902	0.0767	0.465144	no
17.8905	21.0544	0.234925	0.79393	0.07665	0.465144	no
17.0989	20.0139	0.227098	0.789092	0.07675	0.465144	no
22.6886	19.5541	-0.214501	-0.793371	0.0768	0.465205	no
90.9661	105.455	0.213229	0.783893	0.0771	0.46605	no
3.31085	4.05901	0.293923	0.795931	0.07705	0.46605	no
22.4116	26.8105	0.258549	0.786881	0.077	0.46605	no
11.7438	14.5164	0.305791	0.788334	0.07715	0.46611	no
18.1584	21.394	0.236566	0.790652	0.07725	0.466471	no
4.26796	3.57631	-0.255074	-0.782359	0.0773	0.466531	no
6.64245	5.64082	-0.235811	-0.781025	0.0774	0.466892	no
15.5083	11.6129	-0.417313	-0.773839	0.07745	0.466951	no
14.8108	12.785	-0.212193	-0.786789	0.0775	0.46701	no
2.736	2.14473	-0.351273	-0.784819	0.0776	0.46737	no
18.2066	15.687	-0.214893	-0.781491	0.0779	0.468934	no
1.32648	1.99131	0.586116	0.77952	0.07795	0.468992	no
1.41826	2.18512	0.623593	0.807965	0.078	0.46905	no
1.7199	1.31505	-0.387211	-0.777561	0.07805	0.469108	no
38.5403	33.3438	-0.208953	-0.778778	0.07815	0.469466	no
3.39567	4.57242	0.429261	0.788525	0.0782	0.469523	no
7.02092	8.73734	0.315535	0.781567	0.07825	0.469581	no
45.0891	52.2308	0.212123	0.794518	0.07835	0.469938	no
61.5114	53.2811	-0.20723	-0.776493	0.0784	0.469995	no
3.53682	2.83477	-0.31922	-0.783293	0.0786	0.470951	no
2.15511	3.02047	0.487008	0.79062	0.0788	0.471291	no
76.9545	64.7974	-0.248071	-0.779873	0.0789	0.471291	no
12.4261	10.5656	-0.233991	-0.776869	0.0789	0.471291	no
10.3316	12.2912	0.250563	0.782679	0.0789	0.471291	no
5.37736	4.37384	-0.297997	-0.78977	0.0787	0.471291	no
15.4547	13.2821	-0.218559	-0.783305	0.0791	0.472	no

3.77755	3.16851	-0.253646	-0.774754	0.0791	0.472	no
16.1918	13.9489	-0.215109	-0.785905	0.0792	0.472112	no
6.30943	8.54234	0.437121	0.77433	0.0792	0.472112	no
6.58935	5.55393	-0.246626	-0.786916	0.07955	0.473955	no
9.03032	7.63227	-0.242665	-0.766142	0.07965	0.474307	no
2.50948	2.02244	-0.311289	-0.776202	0.0798	0.474714	no
2.10456	1.698	-0.309681	-0.791931	0.07985	0.474768	no
9.63655	11.5327	0.259142	0.775268	0.07995	0.47512	no
36.2395	31.3653	-0.208393	-0.778166	0.08	0.475174	no
41.9918	48.5142	0.2083	0.780627	0.0801	0.475525	no
38.4542	33.1485	-0.214198	-0.770643	0.08035	0.475604	no
0.904615	1.58762	0.811488	0.734169	0.08025	0.475604	no
139.022	120.199	-0.209883	-0.779543	0.0802	0.475604	no
24.1457	20.8968	-0.208483	-0.776908	0.08035	0.475604	no
12.7591	10.5325	-0.276668	-0.771344	0.0804	0.475604	no
1.92337	1.57324	-0.289893	-0.775049	0.0804	0.475604	no
153.276	132.006	-0.215532	-0.780328	0.0804	0.475604	no
220.007	255.874	0.217884	0.77745	0.0806	0.476545	no
18.3239	21.4312	0.22599	0.779551	0.08085	0.477537	no
31.403	36.5052	0.217201	0.77689	0.08085	0.477537	no
8.69154	7.37292	-0.237376	-0.773658	0.0811	0.47877	no
78.0406	66.5431	-0.229937	-0.779097	0.0812	0.479117	no
47.7963	55.6243	0.218816	0.7758	0.08125	0.479169	no
16.1256	19.5245	0.275933	0.769108	0.08175	0.480168	no
6.61448	8.3356	0.333659	0.781509	0.08175	0.480168	no
53.9678	46.413	-0.217569	-0.782352	0.0816	0.480168	no
36.7247	42.5029	0.210809	0.779972	0.08175	0.480168	no
9.16378	10.5975	0.209708	0.775725	0.0817	0.480168	no
16.4403	14.1909	-0.21227	-0.775129	0.0816	0.480168	no
16.6483	19.336	0.215917	0.772264	0.0815	0.480168	no
5.91694	5.01532	-0.23851	-0.770803	0.08175	0.480168	no
13.0915	15.5259	0.246041	0.766068	0.08205	0.480281	no
7.54916	6.42659	-0.232262	-0.780875	0.08195	0.480281	no
102.09	119.956	0.232668	0.776612	0.0819	0.480281	no
11.9832	13.9616	0.220442	0.774588	0.0821	0.480281	no
1.03596	1.54907	0.580436	0.775496	0.0821	0.480281	no
21.9562	25.5217	0.217098	0.774381	0.08185	0.480281	no
7.90621	6.65827	-0.247839	-0.776436	0.0821	0.480281	no
78.6683	90.7845	0.206663	0.775325	0.08195	0.480281	no
42.11	36.5013	-0.206216	-0.769542	0.08225	0.480916	no
7.98089	9.80465	0.296916	0.774157	0.08235	0.481067	no
1065.8	1286.44	0.271446	0.772272	0.0824	0.481067	no
5.18367	4.33718	-0.257216	-0.775359	0.0824	0.481067	no
14.3587	12.1563	-0.240216	-0.772505	0.08265	0.482284	no
6.76553	8.17112	0.272331	0.762564	0.08285	0.48258	no
9.14137	10.7324	0.231496	0.765848	0.08295	0.48258	no
11.2687	13.8206	0.294503	0.772432	0.0829	0.48258	no

25.1566	29.3184	0.220873	0.775121	0.0828	0.48258	no
14.1855	12.21	-0.216345	-0.775218	0.08295	0.48258	no
17.0367	14.6471	-0.218032	-0.775539	0.08295	0.48258	no
3.19899	2.53717	-0.334395	-0.778848	0.08305	0.48292	no
84.652	98.8565	0.223792	0.781168	0.0831	0.482969	no
35.2579	41.2925	0.227932	0.760028	0.0833	0.48389	no
13.7628	11.7971	-0.222343	-0.759167	0.08375	0.486018	no
3.11227	2.46244	-0.337875	-0.772448	0.08375	0.486018	no
2.24148	1.71681	-0.384718	-0.761139	0.0839	0.486645	no
23.9282	27.8496	0.218942	0.763911	0.08395	0.486692	no
35.4837	40.9211	0.205688	0.769518	0.08405	0.486786	no
2.4761	1.84801	-0.422096	-0.762652	0.08405	0.486786	no
31.1949	35.9715	0.205542	0.763555	0.08435	0.487309	no
1.70169	2.33742	0.457942	0.779048	0.0843	0.487309	no
93.7996	109.804	0.22728	0.766064	0.08425	0.487309	no
17.3312	19.9596	0.20371	0.762703	0.0842	0.487309	no
62.0962	72.4748	0.222974	0.758918	0.08435	0.487309	no
2.61946	3.89828	0.573569	0.764211	0.08445	0.487644	no
35.4695	41.03	0.210101	0.763907	0.0845	0.487691	no
11.1683	13.3528	0.257735	0.767409	0.08455	0.487737	no
7.0434	6.00003	-0.231303	-0.760532	0.0847	0.48836	no
37.8779	44.0207	0.216826	0.773857	0.0849	0.489027	no
3.02935	2.42857	-0.318897	-0.768033	0.0849	0.489027	no
4.7752	5.7488	0.267699	0.77775	0.08495	0.489073	no
14.3622	12.4402	-0.207267	-0.766102	0.08515	0.489255	no
41.5189	36.0626	-0.203267	-0.760577	0.08515	0.489255	no
12.4541	10.4892	-0.247716	-0.755033	0.0851	0.489255	no
136.018	156.984	0.20682	0.77311	0.08515	0.489255	no
6.38475	5.44277	-0.230289	-0.769344	0.0852	0.4893	no
17.3378	20.2651	0.225074	0.769341	0.0855	0.490538	no
11.3883	9.79305	-0.217716	-0.762939	0.0855	0.490538	no
27.2583	31.4582	0.20674	0.759223	0.08565	0.491156	no
12.9497	11.0228	-0.23243	-0.779047	0.08575	0.491487	no
12.1884	14.6008	0.260541	0.763857	0.0858	0.491531	no
14.3682	12.2496	-0.230146	-0.7563	0.086	0.492191	no
22.845	19.8242	-0.204617	-0.767216	0.086	0.492191	no
2.08638	2.74495	0.395782	0.751199	0.08605	0.492235	no
73.4755	84.6769	0.204705	0.765426	0.0861	0.492278	no
54.7513	62.9943	0.202327	0.758345	0.08615	0.492322	no
28.285	22.4987	-0.330191	-0.771184	0.0862	0.492366	no
20.7843	24.0797	0.212327	0.77248	0.08625	0.492409	no
50.7466	44.0242	-0.205013	-0.762644	0.08635	0.492496	no
61.7387	71.2109	0.205923	0.764932	0.08635	0.492496	no
36.426	31.5815	-0.205889	-0.762513	0.08645	0.492583	no
73.1395	84.8674	0.214559	0.768073	0.0865	0.492626	no
32.4579	37.5305	0.209492	0.763441	0.0867	0.49304	no
11.9618	10.0655	-0.249022	-0.772566	0.0867	0.49304	no

7.44335	6.23429	-0.255725	-0.763682	0.08665	0.49304	no
11.46	9.47655	-0.274176	-0.762132	0.08675	0.493083	no
9.60066	8.07813	-0.249114	-0.764856	0.08685	0.49341	no
2.56417	3.56178	0.474108	0.771244	0.087	0.493538	no
8.54965	10.1614	0.249164	0.774279	0.087	0.493538	no
3.19687	1.98504	-0.687492	-0.774685	0.087	0.493538	no
18.8737	21.8946	0.214196	0.762516	0.0871	0.493864	no
8.65578	6.93099	-0.320604	-0.76897	0.0873	0.494034	no
2.26129	1.78115	-0.344341	-0.766692	0.0873	0.494034	no
9.25633	7.55003	-0.293957	-0.775151	0.08725	0.494034	no
10.223	8.70016	-0.232701	-0.757441	0.0872	0.494034	no
7.20707	5.92208	-0.283308	-0.772655	0.08735	0.494076	no
1.77607	2.50341	0.495207	0.740704	0.0875	0.494684	no
107.939	124.636	0.207504	0.758437	0.08775	0.495374	no
5.04501	4.16715	-0.275797	-0.769795	0.08775	0.495374	no
9.74756	8.33866	-0.225226	-0.762804	0.0877	0.495374	no
10.9039	21.1377	0.95497	0.778705	0.08785	0.495698	no
16.6405	14.3338	-0.215275	-0.766369	0.08805	0.496585	no
1.55308	1.22608	-0.341079	-0.762662	0.08815	0.496667	no
2.64121	1.74693	-0.596376	-0.758673	0.08815	0.496667	no
35.6988	41.3074	0.210526	0.757041	0.0882	0.496708	no
9.73778	8.11737	-0.262581	-0.755541	0.0884	0.497593	no
8.47372	7.32224	-0.210712	-0.760174	0.08845	0.497633	no
1.16324	1.78996	0.621772	0.761827	0.0885	0.497673	no
11.3195	13.2966	0.232241	0.744438	0.08865	0.498035	no
23.1463	19.7448	-0.22931	-0.751894	0.08865	0.498035	no
1.11065	1.65692	0.577098	0.751896	0.08875	0.498115	no
4.97997	6.08128	0.288237	0.766313	0.08875	0.498115	no
42.9948	49.5192	0.203826	0.749664	0.08895	0.498755	no
14.4637	16.7312	0.210102	0.750793	0.08895	0.498755	no
14.5598	12.5262	-0.217039	-0.741098	0.0892	0.499916	no
4.10448	3.44133	-0.254234	-0.748371	0.0893	0.500111	no
3.72992	2.4581	-0.601599	-0.762137	0.08935	0.500111	no
21.4096	18.0468	-0.246508	-0.758136	0.08945	0.500111	no
5.01832	5.94276	0.243928	0.750061	0.08945	0.500111	no
2.76904	1.95789	-0.500084	-0.769587	0.0894	0.500111	no
15.121	18.1052	0.259854	0.757389	0.0895	0.50015	no
21.6061	18.6168	-0.214832	-0.760416	0.08985	0.501623	no
16.2598	19.2491	0.243487	0.753113	0.08995	0.50194	no
1.52894	0.936161	-0.707701	-0.767374	0.0901	0.502054	no
5.46986	8.53785	0.642368	0.753986	0.0901	0.502054	no
194.784	162.743	-0.259279	-0.755971	0.09005	0.502054	no
95.4377	110.308	0.208902	0.750035	0.09015	0.502091	no
5.33365	4.51459	-0.24053	-0.750897	0.09025	0.502408	no
24.2686	29.3438	0.273966	0.761367	0.0904	0.50252	no
4.77674	7.38415	0.628405	0.767715	0.09035	0.50252	no
13.767	11.9064	-0.209475	-0.744507	0.0904	0.50252	no

8.8302	10.6501	0.270347	0.747392	0.09045	0.502542	no
12.2974	15.2513	0.310588	0.759714	0.09075	0.502542	no
1.06951	1.56694	0.551001	0.748577	0.09055	0.502542	no
30.4701	26.4794	-0.202523	-0.753863	0.09055	0.502542	no
9.729	11.411	0.230067	0.742839	0.09075	0.502542	no
7.85204	9.51069	0.276483	0.745951	0.09065	0.502542	no
5.66777	4.69807	-0.270713	-0.760937	0.0906	0.502542	no
4.24791	5.47203	0.365324	0.74877	0.0907	0.502542	no
61.0711	70.2699	0.202416	0.754491	0.0908	0.50258	no
1.72192	2.32869	0.435497	0.753742	0.09085	0.502617	no
9.78672	11.3926	0.219194	0.743407	0.091	0.502968	no
2.03248	2.55968	0.332727	0.7502	0.0911	0.503281	no
45.0031	52.1528	0.212719	0.753544	0.0913	0.504146	no
20.0258	23.8192	0.250261	0.74787	0.09135	0.504183	no
17.6761	20.9121	0.242543	0.74666	0.09155	0.504807	no
16.5205	19.0905	0.208594	0.74565	0.09175	0.50519	no
2.97625	2.17756	-0.450787	-0.751372	0.0918	0.505226	no
8.36093	7.06898	-0.242161	-0.753923	0.0921	0.506637	no
31.28	36.2012	0.210799	0.744606	0.09225	0.507222	no
129.184	148.596	0.201961	0.755036	0.09265	0.507365	no
3.62457	2.75598	-0.395243	-0.757525	0.09265	0.507365	no
7.36376	6.33198	-0.217784	-0.742034	0.0925	0.507365	no
7.90391	6.74289	-0.229199	-0.734787	0.09275	0.507365	no
64.8399	74.5958	0.202213	0.746498	0.0928	0.507365	no
1.10748	1.66376	0.587165	0.759747	0.0924	0.507365	no
183.522	217.52	0.245192	0.744867	0.0927	0.507365	no
1.67993	2.32968	0.47173	0.762441	0.09255	0.507365	no
5.50729	6.8145	0.307266	0.75529	0.0927	0.507365	no
4.04638	3.17942	-0.347866	-0.735601	0.0928	0.507365	no
7.14706	8.79368	0.299118	0.748573	0.09275	0.507365	no
40.7862	46.901	0.201536	0.758002	0.09275	0.507365	no
18.9896	21.8473	0.202249	0.740412	0.09305	0.508492	no
5.44849	4.59055	-0.24719	-0.741246	0.0931	0.508526	no
4.45092	3.12741	-0.509136	-0.748543	0.0934	0.509925	no
43.6569	51.4714	0.237559	0.751075	0.09345	0.509959	no
67.7008	77.7646	0.199941	0.750069	0.09355	0.510265	no
7.29667	9.93343	0.445054	0.737725	0.0938	0.510669	no
16.3747	14.2458	-0.200934	-0.748502	0.09375	0.510669	no
2.33105	3.194	0.454384	0.742428	0.0938	0.510669	no
19.7496	22.975	0.218241	0.747924	0.09375	0.510669	no
11.7287	14.124	0.268108	0.751965	0.0939	0.510974	no
4.85802	5.62417	0.211272	0.743171	0.09405	0.511072	no
8.76046	10.2236	0.222818	0.738843	0.09405	0.511072	no
37.5889	43.1522	0.199126	0.744639	0.0942	0.51117	no
7.93968	9.18878	0.210792	0.737518	0.0942	0.51117	no
13.1769	10.6603	-0.305757	-0.756175	0.09415	0.51117	no
3.38152	2.69941	-0.325027	-0.749213	0.09435	0.511745	no

8.1942	6.94227	-0.239196	-0.743852	0.09445	0.51181	no
4.03606	2.96944	-0.442755	-0.731465	0.09445	0.51181	no
1.47479	1.15841	-0.348359	-0.750086	0.0945	0.511842	no
1.41619	0.861435	-0.717197	-0.747295	0.09455	0.511874	no
2.92845	3.95455	0.433375	0.736533	0.09485	0.512543	no
3.15949	4.28949	0.441113	0.743483	0.0948	0.512543	no
2.70062	2.04171	-0.403516	-0.745238	0.09485	0.512543	no
88.6292	101.989	0.202554	0.741294	0.09475	0.512543	no
129.489	153.114	0.241781	0.747884	0.0949	0.512575	no
1.4444	2.08275	0.528015	0.730428	0.0953	0.513066	no
17.2725	20.164	0.223308	0.73583	0.0953	0.513066	no
10.0446	11.7051	0.220724	0.741209	0.09515	0.513066	no
3.52239	5.92736	0.750834	0.71819	0.0951	0.513066	no
4.80142	3.9998	-0.263532	-0.743663	0.0953	0.513066	no
30.8673	35.4191	0.198446	0.740376	0.0953	0.513066	no
2.43729	1.99089	-0.29186	-0.748815	0.09515	0.513066	no
8.72532	7.60335	-0.198574	-0.737595	0.09545	0.513635	no
6.72859	5.14406	-0.387397	-0.744928	0.0956	0.514204	no
14.3214	16.5326	0.207137	0.74712	0.09565	0.514235	no
1047.83	1346.28	0.361568	0.738776	0.0957	0.514266	no
35.3596	40.5471	0.197495	0.734459	0.09605	0.51567	no
11.5443	13.6726	0.244101	0.744103	0.09605	0.51567	no
25.058	21.7685	-0.203034	-0.73844	0.09615	0.51573	no
8.4179	10.0743	0.259141	0.744798	0.09615	0.51573	no
4.7037	6.9724	0.56786	0.743089	0.09625	0.516028	no
39.6697	47.0043	0.244755	0.750637	0.0963	0.516058	no
12.2247	10.6402	-0.200274	-0.751227	0.0965	0.516653	no
15.8355	19.7473	0.318491	0.742934	0.0965	0.516653	no
15.5166	18.4051	0.246294	0.735356	0.0968	0.51683	no
2.20397	1.46844	-0.585823	-0.728074	0.0968	0.51683	no
43.5772	50.0418	0.199561	0.740813	0.09675	0.51683	no
11.5034	13.5467	0.235885	0.732295	0.09675	0.51683	no
3.96056	3.11271	-0.347533	-0.741827	0.097	0.51766	no
86.7554	99.9137	0.203728	0.737423	0.09735	0.519289	no
5.94785	4.81221	-0.305669	-0.739546	0.0974	0.519318	no
10.2883	8.89088	-0.210603	-0.741438	0.09745	0.519346	no
15.317	13.3286	-0.200616	-0.739755	0.0975	0.519374	no
1.2904	1.80399	0.483365	0.73967	0.09775	0.519976	no
2.12252	2.72244	0.359128	0.734735	0.0978	0.519976	no
1.7419	2.35881	0.437398	0.731578	0.0978	0.519976	no
4.01122	4.86837	0.279396	0.746967	0.0981	0.519976	no
1.95752	1.59477	-0.295674	-0.736789	0.09815	0.519976	no
2.83807	2.32879	-0.28533	-0.744087	0.098	0.519976	no
13.0814	11.1977	-0.224316	-0.730643	0.09805	0.519976	no
3.80291	4.79147	0.333365	0.735974	0.09795	0.519976	no
1.69278	2.49494	0.559611	0.729758	0.09805	0.519976	no
15.3288	18.2474	0.251445	0.736803	0.0977	0.519976	no

1.61584	1.22705	-0.39709	-0.706528	0.09805	0.519976	no
35.1396	30.6599	-0.196747	-0.732071	0.09815	0.519976	no
7.34481	6.25092	-0.232657	-0.733164	0.0982	0.520003	no
33.1489	37.9414	0.19481	0.730824	0.09825	0.520031	no
129.591	148.967	0.201025	0.731881	0.0983	0.520059	no
6.30782	5.23905	-0.267836	-0.748777	0.09835	0.520087	no
5.26242	4.31183	-0.287426	-0.73665	0.0984	0.520114	no
8.92941	11.0192	0.303385	0.744688	0.0986	0.520225	no
5.62878	6.98948	0.312362	0.743651	0.0986	0.520225	no
41.1026	35.6896	-0.203727	-0.738073	0.0985	0.520225	no
54.1221	47.2221	-0.196757	-0.737	0.09855	0.520225	no
11.0867	13.4767	0.28164	0.737389	0.09875	0.520335	no
1.54231	1.06192	-0.538427	-0.7308	0.09875	0.520335	no
1.00242	2.0199	1.0108	0.691433	0.0988	0.520335	no
3.46927	2.78759	-0.315616	-0.739394	0.0988	0.520335	no
4.64113	3.2988	-0.492534	-0.741676	0.09885	0.520362	no
1.46301	0.868667	-0.752067	-0.753154	0.09895	0.520653	no
5.09082	4.36397	-0.222256	-0.729157	0.09905	0.520789	no
28.8658	25.1409	-0.199324	-0.738175	0.0991	0.520789	no
10.3065	9.00014	-0.195529	-0.727784	0.0992	0.520789	no
2.19335	1.83053	-0.260877	-0.731449	0.09915	0.520789	no
16.2306	13.6086	-0.254206	-0.74092	0.0993	0.520843	no
10.8812	12.7477	0.228404	0.738792	0.0993	0.520843	no
15.0644	18.9277	0.32936	0.732861	0.0994	0.521132	no
95.6843	111.092	0.215396	0.731551	0.09945	0.521159	no
18.7775	21.5681	0.199892	0.730238	0.0995	0.521186	no
2.7236	1.94917	-0.482656	-0.735647	0.0997	0.521763	no
1.7513	2.86309	0.709152	0.715232	0.0997	0.521763	no
16.0298	13.9962	-0.195716	-0.727641	0.09975	0.52179	no
14.4581	12.607	-0.197659	-0.741144	0.0999	0.522339	no
19.0747	16.559	-0.204046	-0.732751	0.09995	0.522366	no
1.47027	1.19194	-0.302761	-0.735145	0.1001	0.522679	no
10.4094	12.5316	0.26768	0.744139	0.1001	0.522679	no
7.69543	9.4328	0.293684	0.734407	0.1006	0.524583	no
831.953	1036.79	0.317556	0.716628	0.1006	0.524583	no
3.05226	2.3289	-0.390232	-0.719159	0.10055	0.524583	no
0.846496	1.55255	0.875064	0.729361	0.10065	0.524608	no
29.3791	25.5911	-0.199147	-0.737969	0.1007	0.524633	no
57.9318	66.6409	0.202052	0.731231	0.10075	0.524659	no
3.90226	5.08684	0.382459	0.738811	0.1008	0.524684	no
13.8397	16.0631	0.214931	0.726964	0.10105	0.525044	no
120.77	101.965	-0.244192	-0.737186	0.101	0.525044	no
4.82488	3.94768	-0.28949	-0.73313	0.10105	0.525044	no
60.8418	69.9866	0.202016	0.735467	0.10105	0.525044	no
10.7038	8.66762	-0.304414	-0.744931	0.10135	0.52623	no
7.94199	9.26488	0.222271	0.73019	0.1014	0.52623	no
14.3378	12.3992	-0.209581	-0.733066	0.10155	0.52623	no

1.92982	2.75907	0.515714	0.706989	0.10155	0.52623	no
12.2279	14.2986	0.225702	0.729773	0.10145	0.52623	no
8.67777	7.34422	-0.240716	-0.738981	0.1015	0.52623	no
732.204	905.977	0.307229	0.727229	0.1017	0.526562	no
11.5889	13.9936	0.272024	0.728307	0.10175	0.526562	no
1.68692	1.24869	-0.433984	-0.731012	0.10175	0.526562	no
11.8463	13.7039	0.210151	0.729265	0.10195	0.527362	no
55.3333	64.1227	0.212688	0.730453	0.10205	0.527433	no
1.42511	2.46955	0.79318	0.73761	0.1021	0.527433	no
2.53262	1.9497	-0.377379	-0.728771	0.1021	0.527433	no
75.586	86.7617	0.198939	0.731894	0.1024	0.528513	no
5.35375	4.64279	-0.205557	-0.727161	0.1024	0.528513	no
4.58203	3.73348	-0.295467	-0.731005	0.1025	0.528794	no
32.6905	37.6105	0.202263	0.729339	0.10255	0.528818	no
11.3266	13.183	0.218962	0.728339	0.10315	0.529093	no
46.1685	52.8024	0.193694	0.7187	0.1031	0.529093	no
28.4918	32.8267	0.204322	0.718094	0.103	0.529093	no
1.50868	1.18074	-0.353593	-0.708712	0.10275	0.529093	no
6.68899	9.14873	0.451783	0.741677	0.10265	0.529093	no
189.918	218.776	0.204084	0.721415	0.10305	0.529093	no
1.8754	1.41831	-0.403023	-0.730219	0.10315	0.529093	no
10.2794	8.87999	-0.211124	-0.722245	0.10285	0.529093	no
14.2018	12.3992	-0.195825	-0.72022	0.1027	0.529093	no
30.8682	25.5132	-0.274877	-0.731224	0.10295	0.529093	no
3.09177	2.40833	-0.360402	-0.712822	0.10305	0.529093	no
1.4326	0.947743	-0.596067	-0.646572	0.10285	0.529093	no
27.2375	23.7573	-0.197222	-0.729744	0.10325	0.529139	no
9.00585	12.231	0.441609	0.72199	0.10325	0.529139	no
2.57731	3.41656	0.406679	0.71643	0.1034	0.529674	no
3.70457	2.84846	-0.379121	-0.713201	0.10345	0.529697	no
12.3591	10.7885	-0.196086	-0.709717	0.10365	0.530487	no
5.98711	7.16004	0.258107	0.728968	0.10375	0.530765	no
22.0455	25.3149	0.1995	0.720368	0.1039	0.531299	no
10.9942	7.33889	-0.583112	-0.876996	0.10405	0.531831	no
3.53962	5.98016	0.756587	0.730784	0.10415	0.531875	no
3.34394	2.84222	-0.234533	-0.719112	0.10415	0.531875	no
11.7828	10.2822	-0.196534	-0.721185	0.1042	0.531896	no
2.87183	2.09661	-0.453913	-0.733395	0.1043	0.532173	no
1.53027	1.01314	-0.594958	-0.73554	0.1044	0.532449	no
5.12115	4.02675	-0.346851	-0.71499	0.10445	0.53247	no
21.2547	24.4773	0.203667	0.729625	0.1045	0.532492	no
8.06475	6.91999	-0.220861	-0.708537	0.10455	0.532513	no
3.44358	4.16448	0.274226	0.713305	0.1046	0.532534	no
20.4882	17.6002	-0.219201	-0.717979	0.10475	0.533064	no
12.8879	10.9803	-0.231101	-0.724252	0.1049	0.533594	no
3.30615	2.00462	-0.721827	-0.721129	0.105	0.533869	no
21.6766	18.8761	-0.199579	-0.720841	0.1052	0.534418	no

19.7727	24.1	0.285524	0.729148	0.10535	0.534499	no
1.41274	1.0915	-0.372189	-0.719326	0.10535	0.534499	no
18.5523	16.2077	-0.194913	-0.721638	0.1054	0.534499	no
342.393	411.949	0.266811	0.727202	0.1054	0.534499	no
3.67873	5.26399	0.516949	0.725351	0.1056	0.534853	no
20.0081	22.9555	0.198252	0.720107	0.10565	0.534853	no
2.95465	2.4134	-0.291924	-0.720083	0.1057	0.534853	no
3.90806	3.29647	-0.245528	-0.724897	0.1057	0.534853	no
5.2951	4.58353	-0.208201	-0.715705	0.1056	0.534853	no
27.8629	31.9041	0.195396	0.72241	0.10585	0.535146	no
27.15	23.7349	-0.193943	-0.713451	0.10585	0.535146	no
15.6802	18.2111	0.215874	0.713083	0.1061	0.535478	no
29.1092	33.9895	0.223611	0.712098	0.1061	0.535478	no
3.01291	2.46011	-0.292435	-0.724634	0.106	0.535478	no
5.21194	6.42511	0.301901	0.718757	0.10615	0.535497	no
5.07835	6.18387	0.284149	0.726505	0.1062	0.535517	no
13.1352	15.1472	0.205613	0.713212	0.1064	0.535596	no
2.17594	2.90693	0.417862	0.716323	0.1064	0.535596	no
6.36645	5.09565	-0.321225	-0.706558	0.1064	0.535596	no
15.5578	17.9381	0.205393	0.712299	0.1064	0.535596	no
0.999046	1.44914	0.53657	0.730764	0.1065	0.535635	no
1.95462	1.31052	-0.576752	-0.728792	0.1065	0.535635	no
6.395	7.41951	0.214381	0.708664	0.1066	0.535906	no
27.2132	31.1659	0.19566	0.711427	0.1067	0.535945	no
21.4667	18.7573	-0.194648	-0.711509	0.1067	0.535945	no
8.79283	10.6388	0.27494	0.72847	0.10675	0.535964	no
2.35487	3.22413	0.453261	0.732976	0.1069	0.536486	no
15.6127	13.6767	-0.190995	-0.709619	0.1071	0.537257	no
1.42102	2.03787	0.520136	0.717404	0.1074	0.53853	no
143.952	164.411	0.191722	0.719301	0.1075	0.538799	no
26.9767	23.5732	-0.194568	-0.711204	0.1077	0.538871	no
17.7138	15.2307	-0.21789	-0.712653	0.1076	0.538871	no
6.41472	8.43967	0.3958	0.718079	0.10765	0.538871	no
31.1625	27.284	-0.191756	-0.710259	0.1077	0.538871	no
16.7711	14.5318	-0.206765	-0.719849	0.10775	0.538889	no
5.59591	4.55303	-0.297548	-0.722764	0.10795	0.539425	no
293.118	335.099	0.193104	0.710931	0.10795	0.539425	no
1.68461	1.22287	-0.46214	-0.716056	0.108	0.539443	no
5.71707	6.80192	0.250666	0.711399	0.10805	0.539461	no
26.6789	23.1732	-0.203244	-0.7173	0.10835	0.540262	no
6.67722	5.80903	-0.200952	-0.717385	0.10835	0.540262	no
13.2886	11.5587	-0.201209	-0.705388	0.10835	0.540262	no
31.7566	37.9527	0.257144	0.708501	0.10845	0.540529	no
7.89222	6.67418	-0.241841	-0.716224	0.1086	0.54058	no
5.50057	4.25549	-0.370257	-0.71745	0.1086	0.54058	no
8.97166	7.3037	-0.296746	-0.729836	0.10855	0.54058	no
0.970022	1.42804	0.557942	0.731367	0.1087	0.540847	no

158.896	181.74	0.193796	0.704575	0.10885	0.541361	no
26.1287	29.8141	0.190359	0.704557	0.1089	0.541378	no
32.8569	37.6707	0.197248	0.710407	0.10895	0.541395	no
1.08503	1.65644	0.610355	0.693748	0.1091	0.541908	no
14.8875	17.1582	0.204804	0.711993	0.1093	0.54267	no
108.546	94.6783	-0.1972	-0.715099	0.10955	0.543679	no
3.26493	2.6359	-0.308759	-0.708466	0.1097	0.543726	no
13.0865	11.4195	-0.196581	-0.72204	0.10965	0.543726	no
11.4388	9.97905	-0.196965	-0.714666	0.1097	0.543726	no
27.5814	31.8876	0.209299	0.707231	0.10975	0.543742	no
199.147	227.133	0.189703	0.705592	0.10995	0.544501	no
17.7386	20.5879	0.214904	0.713498	0.1101	0.544547	no
51.3763	58.653	0.191104	0.710591	0.1101	0.544547	no
108.151	123.524	0.191744	0.703384	0.11035	0.545552	no
48.6517	55.7084	0.195406	0.711941	0.1104	0.545567	no
7.62046	6.25233	-0.285483	-0.7172	0.1105	0.545597	no
11.4522	9.75439	-0.231499	-0.714395	0.1105	0.545597	no
13.9282	15.9394	0.194592	0.716376	0.1106	0.545858	no
16.1123	12.135	-0.408988	-0.701925	0.11075	0.545903	no
187.106	156.624	-0.256553	-0.72151	0.11075	0.545903	no
1.2327	1.70486	0.467834	0.70929	0.1107	0.545903	no
23.1176	26.8302	0.214864	0.713061	0.11095	0.546657	no
10.7247	12.6553	0.238808	0.715058	0.11105	0.546686	no
24.6007	30.0872	0.290449	0.71006	0.1111	0.5467	no
46.4137	52.8879	0.188387	0.70415	0.1112	0.546744	no
1.12176	1.78804	0.672609	0.698199	0.11125	0.546744	no
59.7942	68.6763	0.199808	0.702795	0.11125	0.546744	no
14.3311	12.5274	-0.194066	-0.714278	0.11155	0.547755	no
6.02456	7.55965	0.327462	0.70858	0.1116	0.547769	no
65.3642	57.2562	-0.191069	-0.710509	0.11165	0.547783	no
2.16829	1.77905	-0.285454	-0.70669	0.1126	0.547872	no
101.231	116.331	0.200586	0.701624	0.1127	0.547872	no
6.07156	4.70831	-0.405125	-0.846344	0.11215	0.547872	no
10.8019	12.3392	0.191965	0.714325	0.1128	0.547872	no
21.4242	18.7464	-0.19263	-0.705164	0.11215	0.547872	no
1.27811	1.83491	0.521701	0.713546	0.11245	0.547872	no
313.011	273.863	-0.192754	-0.702529	0.1128	0.547872	no
7.25657	5.86187	-0.307928	-0.697088	0.11245	0.547872	no
5.91083	5.03618	-0.23103	-0.709999	0.1122	0.547872	no
43.8925	50.5246	0.203013	0.705559	0.1127	0.547872	no
6.18914	4.45972	-0.472788	-0.69482	0.1127	0.547872	no
2.85325	2.28161	-0.322557	-0.704235	0.1121	0.547872	no
7.77313	6.29045	-0.305332	-0.710999	0.1122	0.547872	no
22.9948	26.8539	0.223828	0.707689	0.11185	0.547872	no
39.6383	45.8941	0.211412	0.705508	0.11225	0.547872	no
7.03588	5.85995	-0.263842	-0.713725	0.11195	0.547872	no
135.75	154.778	0.189245	0.709696	0.1125	0.547872	no

3.88257	3.03073	-0.357346	-0.702255	0.1122	0.547872	no
46.5974	53.4326	0.19747	0.704838	0.11175	0.547872	no
18.6521	21.5261	0.206749	0.70109	0.1119	0.547872	no
8.43623	7.35486	-0.197901	-0.707288	0.11245	0.547872	no
3.52928	2.79326	-0.337425	-0.694022	0.1128	0.547872	no
20.795	18.2448	-0.188748	-0.704489	0.1129	0.5479	no
2.62118	3.69455	0.495184	0.711105	0.1129	0.5479	no
2.6787	2.10757	-0.34595	-0.698832	0.11295	0.547914	no
5.59542	4.52622	-0.305942	-0.703694	0.113	0.547927	no
5.09599	6.10708	0.261121	0.700052	0.1132	0.548211	no
190.241	243.455	0.355823	0.701625	0.11315	0.548211	no
11.8675	13.7414	0.211512	0.698022	0.1132	0.548211	no
1.84789	2.54032	0.459129	0.704608	0.1133	0.548466	no
15.8612	18.0763	0.188602	0.706083	0.1134	0.548722	no
2.26837	1.7923	-0.339844	-0.69866	0.11345	0.548735	no
16.3961	14.4112	-0.186164	-0.69963	0.1135	0.548749	no
42.7789	37.4538	-0.19179	-0.703522	0.1137	0.549487	no
5.25135	4.48704	-0.226923	-0.702852	0.11385	0.549983	no
5.12608	6.35499	0.310035	0.698793	0.114	0.55025	no
14.8448	12.7971	-0.214144	-0.697535	0.114	0.55025	no
51.799	59.2262	0.193313	0.706559	0.11405	0.550263	no
16.1583	13.7409	-0.233794	-0.697364	0.11435	0.551481	no
29.2675	33.5222	0.195816	0.71001	0.1144	0.551493	no
55.7877	63.7303	0.192033	0.699322	0.11445	0.551506	no
452.984	517.367	0.191729	0.700607	0.1146	0.551542	no
28.0284	31.9631	0.189519	0.708429	0.1146	0.551542	no
42.5139	37.2036	-0.192492	-0.690001	0.11455	0.551542	no
7.02512	8.34534	0.248447	0.705099	0.11485	0.552516	no
61.4438	70.1527	0.191229	0.696559	0.11515	0.55373	no
9.415	8.07717	-0.22111	-0.701959	0.11525	0.553982	no
5.15635	6.30285	0.289656	0.703284	0.11545	0.554026	no
9.34205	11.0336	0.240098	0.704926	0.1154	0.554026	no
10.3046	8.52394	-0.273692	-0.707258	0.1154	0.554026	no
4.69611	3.49317	-0.426929	-0.700411	0.11545	0.554026	no
36.2905	41.3874	0.189599	0.706417	0.1155	0.554038	no
77.7316	88.7386	0.191061	0.700019	0.1156	0.554288	no
4.93503	4.14944	-0.25014	-0.703532	0.11565	0.554299	no
3.13347	3.80792	0.281242	0.711722	0.1157	0.55431	no
58.5937	67.0942	0.195443	0.68748	0.11585	0.5548	no
328.224	288.057	-0.188324	-0.69271	0.1159	0.554811	no
29.1259	25.5685	-0.187937	-0.697616	0.116	0.555061	no
22.5976	26.188	0.212736	0.694077	0.11605	0.555072	no
0	24.2892	inf	#NAME?	0.11615	0.555321	no
1.16595	1.49508	0.358722	0.688241	0.11625	0.555342	no
2.50132	3.16439	0.339234	0.681658	0.11625	0.555342	no
21.7739	25.4405	0.224529	0.695577	0.11645	0.556069	no
20.4661	23.6702	0.209836	0.68649	0.11655	0.556318	no

5.60155	4.68847	-0.256709	-0.700657	0.1166	0.556328	no
11.6979	10.2432	-0.191585	-0.692343	0.11685	0.557292	no
17.6178	23.6845	0.426907	0.700317	0.11695	0.55754	no
8.77851	7.67123	-0.194517	-0.689992	0.117	0.55755	no
14.9412	13.0282	-0.197661	-0.697168	0.11705	0.557559	no
20.2675	17.595	-0.204007	-0.690556	0.1171	0.557569	no
27.3619	23.6786	-0.208581	-0.691218	0.11725	0.558054	no
14.6741	16.9083	0.204458	0.691067	0.1185	0.559211	no
9.23994	7.97159	-0.213015	-0.693995	0.11855	0.559211	no
5.2401	4.31871	-0.278995	-0.701878	0.1186	0.559211	no
28.2364	24.5859	-0.199728	-0.702639	0.1182	0.559211	no
48.8292	42.895	-0.186934	-0.69796	0.1182	0.559211	no
9.9422	8.74751	-0.184693	-0.693038	0.11855	0.559211	no
12.7457	11.1131	-0.197751	-0.693882	0.11845	0.559211	no
14.9307	17.1414	0.199198	0.70285	0.1186	0.559211	no
11.2778	9.90025	-0.187947	-0.691164	0.11825	0.559211	no
18.6723	22.0469	0.239676	0.700048	0.11775	0.559211	no
6.05623	4.78446	-0.340063	-0.692136	0.118	0.559211	no
19.612	15.8471	-0.307519	-0.693236	0.11855	0.559211	no
8.87025	10.2884	0.213973	0.680248	0.1179	0.559211	no
7.69568	9.40042	0.288676	0.689988	0.11845	0.559211	no
9.23708	10.7095	0.213382	0.696447	0.118	0.559211	no
2.8522	2.22278	-0.35971	-0.698634	0.1183	0.559211	no
24.8854	21.8371	-0.188517	-0.700507	0.1176	0.559211	no
20.0457	17.5821	-0.189186	-0.69748	0.11785	0.559211	no
3.05318	1.99047	-0.617202	-0.707465	0.1183	0.559211	no
88.492	100.597	0.184971	0.690954	0.1185	0.559211	no
1.77085	1.22538	-0.531204	-0.692755	0.1182	0.559211	no
29.7293	34.0544	0.195955	0.694248	0.1189	0.56017	no
26.497	23.1021	-0.197802	-0.689129	0.1189	0.56017	no
2.77421	3.50461	0.337176	0.677959	0.11895	0.560179	no
3.01428	2.3715	-0.346008	-0.689576	0.119	0.560187	no
7.65062	6.27644	-0.28563	-0.697281	0.1192	0.560674	no
29.793	26.1982	-0.185508	-0.694216	0.1192	0.560674	no
19.7599	22.6099	0.194377	0.696387	0.1193	0.560691	no
9.24151	8.10648	-0.189052	-0.68886	0.1194	0.560707	no
24.9312	28.4186	0.188886	0.69875	0.1194	0.560707	no
3.82586	5.1977	0.442089	0.699705	0.11945	0.560715	no
208.409	244.52	0.230537	0.690496	0.1195	0.560723	no
64.1155	72.876	0.184771	0.692832	0.1197	0.561435	no
9.35031	8.18515	-0.192005	-0.683684	0.1198	0.561451	no
10.4513	8.95236	-0.22334	-0.68311	0.1198	0.561451	no
82.6049	93.9962	0.186375	0.69777	0.11985	0.561459	no
60.7413	53.419	-0.185325	-0.689211	0.11995	0.561701	no
87.4803	99.7704	0.189653	0.697148	0.1202	0.562191	no
9.44071	8.17532	-0.207621	-0.690027	0.1202	0.562191	no
7.90226	9.69598	0.295122	0.691177	0.12015	0.562191	no

19.0233	21.9946	0.209382	0.683497	0.1203	0.562433	no
39.7655	45.1723	0.183923	0.686814	0.1204	0.562674	no
10.2823	8.93145	-0.203198	-0.693338	0.1205	0.562915	no
382.695	443.28	0.212021	0.699216	0.12055	0.562922	no
2.67353	1.92352	-0.474999	-0.693001	0.1206	0.562929	no
7.44981	8.62741	0.211724	0.685052	0.12065	0.562936	no
35.2346	30.9962	-0.184901	-0.687	0.1207	0.562944	no
36.5607	42.1912	0.20665	0.67648	0.12115	0.564615	no
23.2841	20.4847	-0.184801	-0.68685	0.1213	0.564615	no
3.56097	2.95445	-0.269378	-0.695487	0.12135	0.564615	no
41.3163	46.8465	0.18123	0.681251	0.1213	0.564615	no
1.56108	2.14134	0.455972	0.683955	0.12125	0.564615	no
342.521	398.535	0.218515	0.684412	0.12135	0.564615	no
30.9597	35.3199	0.190091	0.681405	0.12155	0.564867	no
30.4233	34.8292	0.195123	0.689266	0.1215	0.564867	no
12.8094	11.0831	-0.20885	-0.686403	0.12155	0.564867	no
10.4435	9.14791	-0.191097	-0.686591	0.12165	0.56488	no
2.66338	4.00968	0.590231	0.679594	0.12165	0.56488	no
3.32032	4.46749	0.42814	0.692973	0.1219	0.564925	no
14.9932	19.4423	0.374892	0.697334	0.12195	0.564925	no
1.64515	0.975966	-0.753316	-0.71235	0.1218	0.564925	no
2.33505	1.8057	-0.370896	-0.68715	0.12175	0.564925	no
60.233	68.4399	0.184284	0.68706	0.12195	0.564925	no
1.09991	1.53682	0.482556	0.686017	0.122	0.564925	no
55.0555	62.8696	0.191477	0.692832	0.122	0.564925	no
20.1438	17.6717	-0.188898	-0.69545	0.1221	0.564937	no
47.2669	54.2109	0.197751	0.684363	0.12215	0.564944	no
4.15523	5.88359	0.50177	0.681435	0.1224	0.565875	no
27.0456	30.7465	0.185028	0.682716	0.1227	0.565916	no
10.0106	8.77066	-0.190764	-0.695224	0.1226	0.565916	no
95.193	108.116	0.183659	0.687212	0.12275	0.565916	no
39.2381	44.5529	0.183265	0.684792	0.1225	0.565916	no
4.12014	3.43367	-0.262944	-0.691583	0.1227	0.565916	no
7.30084	6.20241	-0.235235	-0.696423	0.12265	0.565916	no
17.1894	15.0684	-0.189989	-0.691002	0.12275	0.565916	no
62.5304	72.9484	0.222319	0.687912	0.1228	0.565922	no
107.44	123.297	0.19861	0.684821	0.1229	0.566159	no
2.76223	1.65484	-0.739138	-0.677157	0.123	0.56617	no
11.5518	13.1304	0.184789	0.686401	0.123	0.56617	no
9.73992	8.56289	-0.185812	-0.683975	0.12305	0.566176	no
32.1406	36.7105	0.191798	0.688437	0.1231	0.566182	no
16.2015	18.7492	0.210699	0.673594	0.12315	0.566188	no
15.7011	13.796	-0.186619	-0.685229	0.12325	0.566423	no
19.5436	17.1266	-0.190455	-0.686082	0.1234	0.566889	no
90.0048	102.222	0.183634	0.688275	0.12345	0.566894	no
219.685	249.599	0.18418	0.685206	0.1235	0.5669	no
12.0584	10.6069	-0.185037	-0.681198	0.12365	0.56714	no

5.0755	4.05115	-0.32522	-0.685994	0.12375	0.567375	no
61.4781	70.0159	0.187611	0.682571	0.12395	0.567843	no
1.63902	1.15081	-0.510181	-0.682794	0.12395	0.567843	no
2.10779	1.68633	-0.32185	-0.682688	0.12405	0.568078	no
5.28543	6.39145	0.274123	0.690879	0.1241	0.568083	no
10.1353	12.084	0.253713	0.676836	0.12415	0.568088	no
17.717	22.7756	0.362357	0.671966	0.1242	0.568093	no
1.84551	2.4191	0.390452	0.672508	0.12425	0.568098	no
7.63809	9.59084	0.328445	0.687659	0.1244	0.568336	no
2.81134	3.83056	0.446299	0.684135	0.1244	0.568336	no
16.237	18.5043	0.188574	0.686355	0.1245	0.568346	no
284.735	323.705	0.185059	0.684408	0.1245	0.568346	no
41.5586	36.5774	-0.184195	-0.685913	0.12455	0.568351	no
280.208	318.494	0.184769	0.68795	0.1246	0.568356	no
2.52201	2.03444	-0.309945	-0.683536	0.1247	0.568589	no
2.47159	1.97633	-0.322613	-0.687561	0.1248	0.568822	no
38.9977	44.2888	0.183552	0.674418	0.12535	0.569105	no
131.679	150.289	0.190707	0.676794	0.1254	0.569105	no
11.6078	9.87027	-0.233936	-0.649071	0.1253	0.569105	no
8.56558	7.25096	-0.24038	-0.672379	0.1254	0.569105	no
9.6336	11.3092	0.231347	0.686329	0.1254	0.569105	no
5.99195	7.39883	0.304272	0.679871	0.1253	0.569105	no
26.7754	30.4592	0.18597	0.689885	0.12495	0.569105	no
15.0195	13.1757	-0.188953	-0.679276	0.12545	0.569105	no
5.8119	7.14345	0.297611	0.683729	0.12505	0.569105	no
15.2993	13.226	-0.210094	-0.673999	0.12545	0.569105	no
7.38831	6.32269	-0.224705	-0.681375	0.12525	0.569105	no
35.4905	40.4258	0.187846	0.683522	0.12525	0.569105	no
2.28464	1.58276	-0.529521	-0.668184	0.1256	0.569563	no
23.7721	27.3219	0.200789	0.664975	0.12565	0.569568	no
83.6078	95.0112	0.18446	0.686437	0.1257	0.569572	no
20.3074	17.9034	-0.181771	-0.681723	0.12585	0.570029	no
14.8617	17.1253	0.204537	0.681219	0.1259	0.570034	no
2.97231	2.47771	-0.262576	-0.680701	0.12595	0.570038	no
6.7504	5.1052	-0.403006	-0.67443	0.126	0.570042	no
95.667	109.739	0.197988	0.674737	0.12605	0.570046	no
14.1848	12.3509	-0.199728	-0.680859	0.1261	0.570051	no
14.5869	16.9839	0.219496	0.680107	0.1262	0.570281	no
35.1941	40.6064	0.206374	0.673189	0.1263	0.570511	no
7.53828	8.89531	0.238811	0.681474	0.12635	0.570515	no
11.0816	9.73896	-0.186329	-0.681601	0.12655	0.571196	no
16.8494	14.8587	-0.181391	-0.673781	0.12675	0.571876	no
1.68107	1.24233	-0.436332	-0.66494	0.12685	0.571883	no
71.0852	80.9337	0.187192	0.683524	0.12685	0.571883	no
9.53825	11	0.205702	0.676381	0.127	0.571897	no
8.5813	11.6987	0.447075	0.68177	0.12705	0.571897	no
89.927	103	0.195822	0.684328	0.12705	0.571897	no

6.35395	5.51786	-0.203546	-0.671266	0.12695	0.571897	no
10.8931	12.8928	0.243151	0.683021	0.1272	0.571911	no
13.1836	15.1252	0.198209	0.674396	0.12725	0.571911	no
12.8856	11.1382	-0.21024	-0.673317	0.12725	0.571911	no
2.7496	3.60434	0.390512	0.661498	0.12725	0.571911	no
98.9377	112.38	0.183796	0.676556	0.1273	0.571915	no
2.87046	2.22	-0.370727	-0.681374	0.1276	0.573041	no
7.73817	6.37891	-0.278683	-0.681772	0.12765	0.573044	no
3.65854	4.32175	0.240347	0.682034	0.1278	0.573496	no
13.2277	15.1057	0.191525	0.678462	0.1279	0.573723	no
9.55459	8.3779	-0.189605	-0.678338	0.12805	0.574174	no
93.8837	107.284	0.19249	0.673785	0.1282	0.574625	no
1.47326	2.01121	0.449054	0.677455	0.12825	0.574627	no
21.9993	26.0427	0.243423	0.679803	0.1284	0.575078	no
25.5765	22.2864	-0.198656	-0.665649	0.12845	0.57508	no
5.82822	6.93956	0.25179	0.680872	0.1287	0.575755	no
35.9585	31.1094	-0.20898	-0.677266	0.12905	0.576877	no
14.2478	12.3573	-0.205372	-0.672034	0.1292	0.577325	no
27.0338	30.6927	0.183131	0.674121	0.1294	0.577997	no
7.15668	8.79446	0.297305	0.677693	0.12945	0.577998	no
2.23473	1.79633	-0.315048	-0.668232	0.12955	0.578013	no
1.93294	2.60065	0.428077	0.673151	0.13005	0.578013	no
1.41942	1.849	0.381452	0.670745	0.13015	0.578013	no
27.0147	23.5196	-0.199882	-0.660526	0.1298	0.578013	no
20.0038	17.2852	-0.210735	-0.532634	0.13015	0.578013	no
2.15621	1.78378	-0.273559	-0.66933	0.12985	0.578013	no
3.02843	2.37848	-0.348532	-0.687684	0.13015	0.578013	no
6.24539	7.58208	0.279802	0.682095	0.12965	0.578013	no
7.61774	6.45867	-0.238126	-0.681474	0.1297	0.578013	no
21.4122	25.1885	0.234328	0.666054	0.13015	0.578013	no
1.59454	2.80877	0.816797	0.712033	0.13015	0.578013	no
4.12009	4.98541	0.275036	0.680455	0.1298	0.578013	no
4.50756	5.47812	0.281335	0.684008	0.13015	0.578013	no
17.5411	15.4046	-0.18738	-0.679501	0.12975	0.578013	no
8.58279	7.0672	-0.280307	-0.673353	0.13025	0.578236	no
10.0336	11.5893	0.207965	0.67003	0.1306	0.578905	no
71.1053	80.5289	0.179549	0.67422	0.1306	0.578905	no
111.166	126.429	0.185622	0.67792	0.1305	0.578905	no
5.81381	4.92165	-0.24034	-0.667005	0.1306	0.578905	no
24.4826	21.0602	-0.217235	-0.673985	0.13065	0.578906	no
6.54464	5.62276	-0.219035	-0.681965	0.1307	0.578907	no
22.13	19.5219	-0.18091	-0.670887	0.1308	0.579129	no
27.6426	24.2609	-0.18826	-0.676186	0.1309	0.57913	no
5.16698	4.50119	-0.199014	-0.661174	0.13085	0.57913	no
3.61519	2.73782	-0.401045	-0.656974	0.131	0.579352	no
7.71306	6.52165	-0.242066	-0.672554	0.13135	0.580458	no
1.74987	1.28484	-0.445656	-0.682702	0.13165	0.581121	no

27.3844	24.0805	-0.185489	-0.681293	0.13165	0.581121	no
12.4042	10.8724	-0.190162	-0.675602	0.13165	0.581121	no
12.2291	10.448	-0.227089	-0.678481	0.13185	0.58156	no
2.54139	1.74154	-0.545253	-0.665174	0.1319	0.58156	no
7.90999	9.20502	0.218746	0.668952	0.1321	0.58156	no
31.6029	27.7577	-0.18717	-0.658182	0.1321	0.58156	no
7.11997	10.6825	0.585303	0.680194	0.13215	0.58156	no
29.3332	33.4362	0.188876	0.667612	0.1322	0.58156	no
1.80832	1.41515	-0.353696	-0.663222	0.13195	0.58156	no
22.8932	25.9973	0.183445	0.673165	0.13205	0.58156	no
8.74699	10.1369	0.212764	0.663566	0.13215	0.58156	no
2.70456	1.91548	-0.497689	-0.666685	0.1326	0.582217	no
311.229	361.887	0.217562	0.678245	0.1324	0.582217	no
1.89045	1.48944	-0.343963	-0.653648	0.13245	0.582217	no
42.2656	48.0501	0.185058	0.672796	0.1326	0.582217	no
6.58636	5.654	-0.22021	-0.666333	0.13255	0.582217	no
42.8673	49.0675	0.194889	0.656033	0.1328	0.582875	no
28.4499	25.0215	-0.185256	-0.663563	0.13295	0.583313	no
8.84646	7.58481	-0.221987	-0.670459	0.13305	0.583531	no
9.59917	8.37362	-0.197059	-0.663785	0.1333	0.584187	no
5.90243	6.92348	0.230189	0.662494	0.13345	0.584403	no
13.7824	15.9879	0.214153	0.665438	0.13345	0.584403	no
4.53124	3.74587	-0.274608	-0.678947	0.1339	0.584831	no
8.45935	9.88536	0.224748	0.665987	0.13385	0.584831	no
17.6618	20.0787	0.185034	0.662618	0.1339	0.584831	no
2.3422	1.79277	-0.385672	-0.658282	0.1336	0.584831	no
6.67568	5.25063	-0.346424	-0.662522	0.1337	0.584831	no
4.70491	4.06855	-0.209652	-0.666345	0.13385	0.584831	no
1.88773	1.33877	-0.495743	-0.663133	0.13385	0.584831	no
4.25587	3.10921	-0.452906	-0.674003	0.13415	0.585703	no
10.2386	11.8433	0.210046	0.661584	0.1343	0.585917	no
59.8752	67.7023	0.177247	0.660154	0.13425	0.585917	no
6.1613	5.33009	-0.209076	-0.670392	0.1344	0.586133	no
2.2026	1.5688	-0.489545	-0.680552	0.13455	0.586567	no
5.14363	5.88329	0.193836	0.663926	0.1347	0.586999	no
16.9691	14.8663	-0.190868	-0.660763	0.13475	0.586999	no
4.54573	5.48618	0.27129	0.663413	0.13485	0.587214	no
25.7817	29.7421	0.206159	0.663653	0.13505	0.587425	no
7.33752	6.16663	-0.25081	-0.669302	0.13505	0.587425	no
37.57	42.7739	0.187147	0.659657	0.13505	0.587425	no
456.715	554.123	0.278911	0.659923	0.1352	0.587637	no
14.0589	16.0395	0.190141	0.667402	0.13515	0.587637	no
19.4024	21.9557	0.178361	0.669892	0.13555	0.588486	no
6.34732	4.84558	-0.389476	-0.67933	0.1357	0.588486	no
139.587	158.634	0.184543	0.663413	0.1355	0.588486	no
14.6073	12.8954	-0.179835	-0.663613	0.1357	0.588486	no
7.32521	6.11002	-0.261695	-0.672186	0.13575	0.588486	no

11.3535	13.3701	0.235875	0.665997	0.1356	0.588486	no
4.96729	4.18375	-0.247662	-0.666852	0.1355	0.588486	no
25.7811	22.4601	-0.198951	-0.661736	0.13585	0.5887	no
39.9357	45.692	0.194262	0.65592	0.1365	0.589538	no
141.516	124.727	-0.182183	-0.670068	0.13635	0.589538	no
20.962	18.4142	-0.186958	-0.66009	0.1364	0.589538	no
67.7891	78.1309	0.20484	0.659282	0.1364	0.589538	no
22.3791	25.6633	0.197553	0.651709	0.1365	0.589538	no
7.96382	6.95939	-0.1945	-0.656352	0.1363	0.589538	no
42.7546	48.4595	0.1807	0.658535	0.13615	0.589538	no
6.64692	7.72857	0.217516	0.657593	0.1361	0.589538	no
36.7096	32.3117	-0.184104	-0.653122	0.13625	0.589538	no
83.5071	94.8913	0.184376	0.657977	0.13675	0.589718	no
30.2022	26.5332	-0.186853	-0.666573	0.1367	0.589718	no
2.43956	3.03853	0.316755	0.662584	0.1371	0.589718	no
4.31274	3.66808	-0.233581	-0.659689	0.13705	0.589718	no
6.8962	5.87706	-0.230706	-0.656554	0.13705	0.589718	no
4.4108	3.5895	-0.297257	-0.673931	0.1367	0.589718	no
22.6058	19.7841	-0.192348	-0.659271	0.13685	0.589718	no
7.48331	6.50577	-0.201956	-0.655408	0.1368	0.589718	no
24.1356	27.7937	0.203595	0.665263	0.1371	0.589718	no
5.73147	4.98203	-0.202171	-0.666416	0.13695	0.589718	no
7.48449	6.35367	-0.236314	-0.661038	0.1376	0.590768	no
171.457	193.926	0.177661	0.664509	0.13765	0.590768	no
14.0847	16.4842	0.226953	0.663122	0.1375	0.590768	no
93.558	105.974	0.179781	0.667003	0.1375	0.590768	no
34.7321	39.3225	0.179086	0.661487	0.13755	0.590768	no
13.7466	18.3525	0.416902	0.658997	0.13745	0.590768	no
15.0914	17.2547	0.193264	0.651106	0.138	0.591836	no
6.63491	5.63839	-0.234793	-0.657735	0.13825	0.592462	no
11.7109	13.5098	0.206158	0.652869	0.13835	0.592462	no
67.8492	57.595	-0.236388	-0.664171	0.1382	0.592462	no
11.0035	13.537	0.298949	0.666666	0.13835	0.592462	no
14.242	12.3316	-0.207794	-0.663925	0.13845	0.592672	no
16.5524	14.4933	-0.191655	-0.662371	0.13865	0.592873	no
5.63696	6.61988	0.231887	0.645978	0.1386	0.592873	no
29.6755	33.5977	0.179088	0.660771	0.13855	0.592873	no
3.14957	2.51206	-0.326288	-0.647588	0.1388	0.593296	no
1.78736	1.26277	-0.501232	-0.662427	0.1389	0.593505	no
19.3526	16.6955	-0.213073	-0.654555	0.13905	0.593928	no
16.5224	14.6191	-0.176569	-0.652003	0.13915	0.594136	no
13.4095	11.7878	-0.185961	-0.649306	0.13935	0.594335	no
20.3683	17.918	-0.184918	-0.660727	0.13935	0.594335	no
13.2132	15.2298	0.204914	0.656139	0.1393	0.594335	no
44.2343	51.2531	0.212474	0.663992	0.13955	0.59497	no
15.6908	13.8695	-0.178006	-0.66314	0.13995	0.596456	no
13.7656	15.7783	0.196873	0.661015	0.14005	0.596663	no

8.25763	9.63048	0.221881	0.657855	0.14015	0.59687	no
14.3413	12.6454	-0.181568	-0.659176	0.14035	0.597066	no
7.81693	6.77161	-0.207103	-0.65164	0.14035	0.597066	no
6.9704	6.08446	-0.196112	-0.65859	0.1403	0.597066	no
61.1902	69.2559	0.178636	0.661444	0.14085	0.597225	no
3.02766	3.64365	0.267181	0.662785	0.1406	0.597225	no
5.62872	6.79326	0.271298	0.652438	0.14085	0.597225	no
8.24057	7.21242	-0.192261	-0.651632	0.1407	0.597225	no
2.58988	3.3015	0.350234	0.649605	0.14065	0.597225	no
3.44658	2.81304	-0.293034	-0.656379	0.14075	0.597225	no
19.7228	22.2809	0.175943	0.655228	0.1405	0.597225	no
76.0555	67.1138	-0.180444	-0.665316	0.1407	0.597225	no
6.23844	4.70964	-0.40557	-0.653234	0.14065	0.597225	no
26.6132	30.1057	0.17789	0.661019	0.141	0.597642	no
20.146	23.0199	0.192389	0.666868	0.14135	0.597738	no
4.73803	4.10663	-0.20633	-0.655978	0.14155	0.597738	no
2.5862	3.23493	0.3229	0.651429	0.142	0.597738	no
10.3172	12.7124	0.301183	0.659037	0.1416	0.597738	no
31.8311	27.1532	-0.229312	-0.653585	0.14195	0.597738	no
45.7644	53.0741	0.213781	0.659706	0.14135	0.597738	no
15.2881	18.8454	0.3018	0.657315	0.14115	0.597738	no
43.2284	38.1816	-0.179104	-0.658281	0.14175	0.597738	no
2.92247	3.94817	0.433995	0.663758	0.1415	0.597738	no
14.6958	12.9379	-0.183808	-0.65142	0.14155	0.597738	no
21.2608	24.2631	0.190568	0.647283	0.14135	0.597738	no
3.46792	2.73805	-0.340921	-0.658472	0.14195	0.597738	no
2.2149	3.02519	0.449781	0.656832	0.142	0.597738	no
20.1041	22.9008	0.18791	0.657847	0.1419	0.597738	no
6.74901	5.73778	-0.234184	-0.642187	0.1419	0.597738	no
8.17575	6.91704	-0.241195	-0.657725	0.14185	0.597738	no
1.1715	1.62366	0.470887	0.654707	0.14165	0.597738	no
8.79899	6.85284	-0.360637	-0.655911	0.1414	0.597738	no
16.7722	19.1606	0.192069	0.644921	0.1423	0.598567	no
9.3461	7.72993	-0.27391	-0.655639	0.1423	0.598567	no
2.92916	2.27306	-0.365854	-0.645793	0.14265	0.598738	no
27.5614	24.2084	-0.187138	-0.647733	0.14255	0.598738	no
1.02137	1.45249	0.508013	0.647365	0.1425	0.598738	no
58.1782	65.868	0.1791	0.660762	0.1426	0.598738	no
23.5779	27.2442	0.208514	0.646476	0.14265	0.598738	no
2.8365	2.34987	-0.27153	-0.665839	0.14265	0.598738	no
17.8721	15.7223	-0.1849	-0.648383	0.1431	0.60041	no
66.1462	58.4453	-0.178571	-0.656079	0.1434	0.601444	no
11.2398	9.81284	-0.195868	-0.639088	0.14345	0.601444	no
10.5628	12.2714	0.216306	0.644847	0.1436	0.601856	no
5.18391	4.17451	-0.312432	-0.650745	0.14385	0.602686	no
135.771	155.527	0.195988	0.657695	0.14415	0.603073	no
5.35347	6.32626	0.240878	0.654284	0.14415	0.603073	no

2.34759	1.93161	-0.281378	-0.652377	0.14405	0.603073	no
17.9324	15.3801	-0.221502	-0.648558	0.14435	0.603475	no
1.49034	1.18198	-0.334437	-0.649255	0.14445	0.603668	no
4.0828	5.2696	0.368133	0.642732	0.1445	0.603668	no
6.82237	5.97206	-0.192044	-0.651554	0.1447	0.604286	no
139.462	157.983	0.179889	0.645477	0.14485	0.604695	no
15.5772	13.7388	-0.181175	-0.657732	0.14495	0.604896	no
87.6028	77.4794	-0.177165	-0.647224	0.14505	0.605095	no
3.24407	2.85075	-0.186461	-0.646281	0.1452	0.605278	no
4.92542	4.24343	-0.215019	-0.643929	0.1452	0.605278	no
12.5887	11.1377	-0.176677	-0.649879	0.14525	0.605278	no
26.572	30.0798	0.178887	0.64497	0.1457	0.605606	no
2.44822	2.02914	-0.270862	-0.640836	0.14585	0.605606	no
33.8696	38.1822	0.172913	0.6493	0.1457	0.605606	no
38.9725	44.3909	0.187808	0.640669	0.14575	0.605606	no
9.04402	10.3661	0.196844	0.647367	0.14565	0.605606	no
56.6569	64.075	0.177508	0.643906	0.14585	0.605606	no
1.12063	1.47178	0.393261	0.646726	0.1457	0.605606	no
9.35251	8.25904	-0.17938	-0.653549	0.14585	0.605606	no
5.02178	4.19523	-0.259451	-0.651818	0.14545	0.605606	no
7.15546	5.90795	-0.276388	-0.64347	0.14585	0.605606	no
14.7548	12.9246	-0.19107	-0.653243	0.14605	0.606003	no
20.1281	22.7443	0.176291	0.645583	0.14625	0.606616	no
42.7501	48.2672	0.175115	0.647844	0.1465	0.60722	no
5.81116	6.95572	0.259373	0.655821	0.1465	0.60722	no
30.1527	26.7629	-0.172053	-0.64347	0.14665	0.607408	no
12.8406	14.5339	0.178703	0.648426	0.1466	0.607408	no
75.4861	85.0672	0.172393	0.642322	0.1468	0.607803	no
61.4228	53.5632	-0.197531	-0.646527	0.14685	0.607803	no
10.3191	9.10008	-0.18137	-0.641434	0.14695	0.608	no
47.0834	53.149	0.174822	0.6435	0.14715	0.608395	no
8.27398	7.32213	-0.176318	-0.647431	0.14715	0.608395	no
6.45779	7.49244	0.214396	0.643994	0.1475	0.609408	no
3.03465	4.17875	0.461541	0.650473	0.14745	0.609408	no
32.4781	28.6859	-0.179126	-0.650834	0.1479	0.610409	no
42.578	48.0704	0.175041	0.645729	0.14785	0.610409	no
5.42861	4.42407	-0.295211	-0.658077	0.1479	0.610409	no
1.58689	1.1472	-0.468087	-0.64095	0.1482	0.61078	no
3.60697	2.93656	-0.296658	-0.644925	0.14805	0.61078	no
1.41383	1.9803	0.486108	0.639299	0.14815	0.61078	no
6.44685	7.9851	0.308716	0.637068	0.14815	0.61078	no
6.14537	7.29777	0.247958	0.646849	0.14875	0.610879	no
48.5603	55.1483	0.183539	0.643366	0.14875	0.610879	no
1.96711	2.74152	0.478902	0.654838	0.1486	0.610879	no
4.13511	3.48368	-0.247315	-0.645719	0.14835	0.610879	no
2.54593	1.89209	-0.428215	-0.643884	0.1486	0.610879	no
3.41548	4.30013	0.33229	0.640302	0.1483	0.610879	no

21.8851	19.2149	-0.187724	-0.644435	0.14875	0.610879	no
7.03835	8.45182	0.264025	0.644231	0.1484	0.610879	no
4.10849	3.41853	-0.265233	-0.643447	0.14855	0.610879	no
4.51409	5.52339	0.291119	0.650698	0.1486	0.610879	no
17.6409	15.4371	-0.192515	-0.633646	0.1489	0.611279	no
18.8244	16.6912	-0.173515	-0.632751	0.14915	0.611862	no
12.3393	10.8859	-0.180802	-0.642846	0.1491	0.611862	no
1.5612	1.19967	-0.380016	-0.637135	0.1492	0.611862	no
14.737	17.2034	0.223252	0.648667	0.14935	0.612045	no
1.65927	2.16597	0.384459	0.633544	0.1493	0.612045	no
3.66604	3.17529	-0.207336	-0.633627	0.14975	0.613218	no
7.26612	6.36649	-0.190688	-0.631181	0.1499	0.613218	no
36.6363	44.9165	0.293972	0.640806	0.1499	0.613218	no
4.31387	5.30672	0.29884	0.638755	0.1499	0.613218	no
8.91147	7.79929	-0.192321	-0.644065	0.1499	0.613218	no
41.0698	35.8797	-0.194911	-0.650213	0.15	0.613411	no
66.4965	74.922	0.17211	0.638941	0.1501	0.613581	no
1.79924	2.21983	0.303061	0.634943	0.1502	0.613581	no
15.6223	13.8954	-0.169005	-0.631727	0.15015	0.613581	no
2.08686	1.71224	-0.285453	-0.646353	0.1504	0.613966	no
4.57388	3.80133	-0.266915	-0.635388	0.15035	0.613966	no
8.13626	9.39157	0.207001	0.641333	0.1506	0.614123	no
35.4082	39.9497	0.174102	0.636075	0.1506	0.614123	no
9.15959	7.89014	-0.215231	-0.639486	0.1506	0.614123	no
1.74522	2.39	0.453603	0.631874	0.15065	0.614123	no
32.6252	36.9537	0.179731	0.639372	0.1508	0.614519	no
18.1765	16.0974	-0.175253	-0.641408	0.1509	0.614711	no
12.4025	14.4936	0.224781	0.643502	0.1512	0.615465	no
13.2957	15.2826	0.200926	0.630236	0.15135	0.615465	no
2.39194	1.85488	-0.366854	-0.638312	0.1513	0.615465	no
144.441	164.121	0.184274	0.634619	0.15135	0.615465	no
6.97415	5.83153	-0.258143	-0.64086	0.15155	0.615847	no
25.1122	28.4651	0.180801	0.637695	0.1515	0.615847	no
5.40875	6.87298	0.345641	0.641034	0.1517	0.616241	no
21.0974	18.7098	-0.173266	-0.635831	0.1518	0.616432	no
38.0662	43.0528	0.177598	0.632724	0.1521	0.616788	no
39.8213	45.0648	0.178462	0.639402	0.15205	0.616788	no
34.5508	38.9832	0.174131	0.638711	0.1521	0.616788	no
3.01352	4.17824	0.471444	0.636553	0.15195	0.616788	no
43.8558	38.8919	-0.173299	-0.639531	0.15235	0.616928	no
9.31224	8.25678	-0.173548	-0.636158	0.15225	0.616928	no
6.88768	5.86775	-0.231212	-0.640937	0.1524	0.616928	no
16.978	19.29	0.184182	0.633906	0.15225	0.616928	no
9.51212	8.36477	-0.185441	-0.638637	0.1524	0.616928	no
16.8777	19.3603	0.19799	0.637447	0.1527	0.617927	no
149.386	168.005	0.169464	0.629901	0.15285	0.618091	no
4.81002	3.95864	-0.281038	-0.633884	0.1529	0.618091	no

22.0934	19.6261	-0.170839	-0.63905	0.15285	0.618091	no
1.66058	2.20008	0.405872	0.636544	0.1533	0.618794	no
1.77154	2.38272	0.427603	0.645802	0.1533	0.618794	no
16.2501	14.4377	-0.170602	-0.635641	0.1533	0.618794	no
68.5514	60.4358	-0.181783	-0.641746	0.1535	0.618794	no
4.14077	4.7584	0.200578	0.632775	0.1532	0.618794	no
20.247	17.9554	-0.173294	-0.641632	0.1535	0.618794	no
14.7931	16.7123	0.175987	0.634999	0.1535	0.618794	no
18.7155	16.6157	-0.171687	-0.639308	0.1535	0.618794	no
47.6463	53.6015	0.169908	0.635372	0.15365	0.619183	no
78.6037	88.5193	0.171396	0.635316	0.15375	0.619372	no
16.4577	19.197	0.222117	0.63353	0.15385	0.619546	no
3.98241	3.50281	-0.185132	-0.632204	0.1539	0.619546	no
13.2639	10.3041	-0.364293	-0.634428	0.1542	0.620324	no
2.01519	1.58811	-0.343604	-0.619651	0.15415	0.620324	no
31.6829	28.1753	-0.169274	-0.628599	0.1543	0.620511	no
23.3668	20.748	-0.171489	-0.630841	0.1546	0.621246	no
95.959	85.1787	-0.171927	-0.632724	0.1546	0.621246	no
47.8737	42.5113	-0.171389	-0.631317	0.15475	0.621246	no
16.0467	14.2005	-0.176332	-0.643221	0.15465	0.621246	no
30.7909	34.836	0.178072	0.632607	0.1547	0.621246	no
12.0379	10.6393	-0.17818	-0.622198	0.1549	0.621633	no
2.32312	1.62981	-0.511359	-0.630027	0.15525	0.621749	no
10.5893	9.26451	-0.192825	-0.641036	0.15505	0.621749	no
2.11725	1.75409	-0.271472	-0.634957	0.15515	0.621749	no
170.411	193.334	0.182072	0.634747	0.155	0.621749	no
3.10058	2.43151	-0.350683	-0.62325	0.1551	0.621749	no
14.7037	12.3578	-0.250758	-0.630288	0.1552	0.621749	no
24.0046	27.1034	0.17516	0.621824	0.15545	0.622336	no
8.76814	9.98145	0.186978	0.636872	0.15615	0.622777	no
11.1213	9.87725	-0.17114	-0.624357	0.15605	0.622777	no
28.2585	31.7624	0.168634	0.629826	0.1559	0.622777	no
17.8153	20.4159	0.196577	0.639313	0.15565	0.622777	no
3.35936	3.9819	0.245268	0.629813	0.15595	0.622777	no
19.8088	22.9795	0.214207	0.632814	0.1561	0.622777	no
32.6107	36.7209	0.171254	0.633027	0.15605	0.622777	no
11.1957	9.92453	-0.17388	-0.63465	0.15585	0.622777	no
7.71184	6.73746	-0.194869	-0.623633	0.1559	0.622777	no
2.73516	2.35797	-0.214076	-0.632185	0.1558	0.622777	no
4.74646	5.69528	0.262915	0.638967	0.1561	0.622777	no
6.82589	8.06156	0.240041	0.638424	0.1571	0.622917	no
1.80782	2.23536	0.30626	0.626883	0.1567	0.622917	no
8.78964	7.62383	-0.205289	-0.62834	0.15655	0.622917	no
3.37815	2.62006	-0.366634	-0.638179	0.15665	0.622917	no
2.45658	1.84377	-0.413989	-0.636025	0.15705	0.622917	no
8.81835	7.71618	-0.192622	-0.616066	0.1564	0.622917	no
19.2081	16.6808	-0.203527	-0.628338	0.15705	0.622917	no

11.4169	12.9481	0.18157	0.627326	0.1569	0.622917	no
3.19709	3.85609	0.270381	0.629804	0.15635	0.622917	no
12.152	13.7906	0.182484	0.628989	0.1566	0.622917	no
7.54002	8.81535	0.225451	0.633615	0.1568	0.622917	no
2.31546	1.87932	-0.301092	-0.627236	0.15675	0.622917	no
25.3128	22.0566	-0.19866	-0.628397	0.15705	0.622917	no
9.02455	7.32099	-0.301816	-0.634739	0.15715	0.622917	no
3.45948	4.00164	0.210036	0.624027	0.1571	0.622917	no
18.3223	16.268	-0.171558	-0.630848	0.15715	0.622917	no
9.69009	11.0378	0.187864	0.62976	0.15665	0.622917	no
43.6133	38.7844	-0.169293	-0.629044	0.1575	0.623878	no
2.04539	2.67554	0.387454	0.628504	0.1575	0.623878	no
7.81637	9.12528	0.22337	0.63349	0.1582	0.625159	no
13.7718	16.3496	0.24754	0.623654	0.15815	0.625159	no
24.5916	21.7636	-0.176249	-0.629644	0.15815	0.625159	no
12.8412	14.6449	0.189615	0.627466	0.1581	0.625159	no
4.78775	3.44058	-0.476697	-0.61454	0.15805	0.625159	no
16.3587	14.5349	-0.170531	-0.633432	0.15815	0.625159	no
3.91863	3.16989	-0.305917	-0.624749	0.15795	0.625159	no
26.825	30.3769	0.179397	0.617841	0.1584	0.625524	no
12.3418	14.1888	0.201194	0.621991	0.15835	0.625524	no
4.10476	3.2746	-0.325979	-0.628112	0.15855	0.625888	no
2.25579	1.87323	-0.268102	-0.634514	0.1586	0.625888	no
29.3616	33.121	0.173816	0.630082	0.1587	0.62607	no
4.7346	4.06273	-0.220793	-0.625667	0.1593	0.626461	no
5.17625	6.10835	0.238876	0.624454	0.1591	0.626461	no
101.273	114.354	0.175264	0.629841	0.159	0.626461	no
23.4877	26.8533	0.193193	0.61934	0.15945	0.626461	no
17.324	20.2973	0.228514	0.625375	0.15895	0.626461	no
27.4762	24.4868	-0.166176	-0.620931	0.15925	0.626461	no
7.30365	6.08552	-0.263238	-0.63103	0.15925	0.626461	no
17.4585	19.8327	0.183951	0.62699	0.15945	0.626461	no
25.7596	29.5531	0.1982	0.624386	0.1593	0.626461	no
30.2322	33.9865	0.168872	0.629195	0.15905	0.626461	no
135.612	153.861	0.182145	0.634308	0.1595	0.626461	no
28.9036	32.8716	0.185593	0.629268	0.15915	0.626461	no
6.11243	5.00371	-0.288748	-0.625245	0.15945	0.626461	no
14.4011	16.6273	0.207374	0.616559	0.1596	0.626642	no
10.3159	12.4641	0.272911	0.634574	0.15995	0.627788	no
28.6858	25.3573	-0.177932	-0.621758	0.16	0.627788	no
7.47904	8.57451	0.197202	0.623754	0.16065	0.628624	no
2.14178	2.57937	0.268204	0.623073	0.1606	0.628624	no
14.9983	13.2902	-0.174436	-0.62152	0.1606	0.628624	no
2.85454	2.26579	-0.33324	-0.619459	0.16045	0.628624	no
20.1013	17.56	-0.19499	-0.629546	0.1607	0.628624	no
20.7989	26.6835	0.35944	0.628554	0.16035	0.628624	no
10.5121	12.0122	0.19246	0.617639	0.1606	0.628624	no

9.48673	11.0547	0.220679	0.619123	0.16045	0.628624	no
2.87415	3.8059	0.405102	0.620372	0.16095	0.628966	no
34.5255	38.7641	0.167057	0.625696	0.16085	0.628966	no
8.13749	9.81173	0.269924	0.623342	0.1609	0.628966	no
7.41875	6.45457	-0.200855	-0.62083	0.1613	0.629276	no
5.72706	6.5252	0.188227	0.624077	0.16115	0.629276	no
272.269	305.812	0.16761	0.618228	0.16125	0.629276	no
5.9957	5.19561	-0.206635	-0.618357	0.1612	0.629276	no
9.07251	10.5452	0.217019	0.621646	0.16155	0.629812	no
15.7576	17.748	0.171606	0.624662	0.1616	0.629812	no
10.3372	11.7831	0.188878	0.622719	0.1616	0.629812	no
42.4636	37.8826	-0.164692	-0.619396	0.16185	0.629941	no
16.929	14.6634	-0.207278	-0.620945	0.16185	0.629941	no
25.004	31.4768	0.332131	0.621328	0.1618	0.629941	no
2.34324	2.94938	0.331908	0.617142	0.16185	0.629941	no
12.1214	13.9241	0.200026	0.62215	0.16235	0.63083	no
6.00192	5.26793	-0.18819	-0.627134	0.16235	0.63083	no
40.2768	35.839	-0.168418	-0.624774	0.16215	0.63083	no
14.5323	16.5866	0.190752	0.618718	0.16235	0.63083	no
11.1925	13.0326	0.219586	0.618469	0.1623	0.63083	no
12.1503	10.6431	-0.191067	-0.61203	0.16285	0.631001	no
3.38797	2.84526	-0.25186	-0.628219	0.1626	0.631001	no
6.67757	5.60476	-0.252669	-0.622623	0.1626	0.631001	no
8.01084	9.4372	0.236406	0.62539	0.1625	0.631001	no
7.21626	6.30341	-0.19512	-0.614291	0.16275	0.631001	no
12.5513	10.7425	-0.224516	-0.62294	0.16265	0.631001	no
1.87063	1.51315	-0.305969	-0.609151	0.163	0.631001	no
5.7545	6.9578	0.273942	0.629434	0.1631	0.631001	no
37.0714	42.0546	0.181957	0.628879	0.1631	0.631001	no
1.85265	2.38476	0.364253	0.621423	0.1627	0.631001	no
8.05757	5.9738	-0.431696	-0.627157	0.1629	0.631001	no
28.534	32.002	0.165478	0.621241	0.1628	0.631001	no
10.189	11.5207	0.177213	0.619347	0.1633	0.631564	no
19.8761	22.4909	0.178307	0.62176	0.1634	0.631741	no
6.01867	6.92567	0.202508	0.616866	0.1642	0.631873	no
9.09269	10.9379	0.266553	0.617507	0.1637	0.631873	no
344.113	386.417	0.167275	0.623718	0.16375	0.631873	no
7.66504	6.75518	-0.182299	-0.618262	0.1636	0.631873	no
49.8604	44.3547	-0.168808	-0.615817	0.1641	0.631873	no
1.67586	1.4	-0.259477	-0.620747	0.1642	0.631873	no
24.8282	28.0073	0.173824	0.621428	0.16415	0.631873	no
2.53891	3.8511	0.601065	0.616892	0.1638	0.631873	no
86.9017	98.4536	0.180058	0.617521	0.16425	0.631873	no
84.2949	94.5135	0.165076	0.616239	0.1641	0.631873	no
4.39486	5.22571	0.24981	0.617402	0.1635	0.631873	no
186.495	211.769	0.183356	0.61512	0.16385	0.631873	no
19.8251	17.6617	-0.166705	-0.623618	0.164	0.631873	no

1.8974	2.58333	0.445209	0.615161	0.16395	0.631873	no
4.01598	5.35183	0.414281	0.624468	0.16365	0.631873	no
44.0681	49.5839	0.170138	0.614517	0.16435	0.632048	no
6.77191	7.94345	0.230204	0.613713	0.1645	0.632207	no
17.974	15.963	-0.171185	-0.613537	0.16445	0.632207	no
11.6396	13.5324	0.217379	0.625976	0.1652	0.632285	no
2.12958	1.51919	-0.487266	-0.61728	0.1655	0.632285	no
17.018	15.1565	-0.167125	-0.618325	0.1654	0.632285	no
65.0511	57.7861	-0.170851	-0.620379	0.16485	0.632285	no
8.67613	10.1982	0.233185	0.612974	0.1653	0.632285	no
295.684	258.542	-0.193657	-0.6229	0.1651	0.632285	no
3.49032	4.11812	0.238627	0.614169	0.16535	0.632285	no
63.6448	71.3937	0.165754	0.615581	0.16495	0.632285	no
107.862	121.05	0.166424	0.614647	0.1646	0.632285	no
24.4545	21.7621	-0.168283	-0.614594	0.1655	0.632285	no
9.53301	8.42289	-0.178616	-0.612115	0.165	0.632285	no
19.0856	21.7324	0.187361	0.607274	0.1649	0.632285	no
5.44329	4.49444	-0.276338	-0.620453	0.16475	0.632285	no
4.58059	3.98925	-0.199414	-0.621202	0.16475	0.632285	no
3.14708	3.83174	0.283986	0.623665	0.16475	0.632285	no
10.4189	12.4287	0.254477	0.623649	0.1655	0.632285	no
6.96584	6.09375	-0.192967	-0.609539	0.1655	0.632285	no
2.98662	3.76291	0.333334	0.622755	0.16535	0.632285	no
9.96707	8.8018	-0.17937	-0.611014	0.1657	0.632841	no
15.2823	17.379	0.185491	0.612087	0.1658	0.633015	no
29.6978	33.4966	0.17366	0.622832	0.16595	0.633379	no
29.4684	35.099	0.252264	0.610547	0.1665	0.634176	no
2.94079	2.19661	-0.420923	-0.620608	0.16665	0.634176	no
5.44242	4.59418	-0.24444	-0.615294	0.1666	0.634176	no
21.8164	24.4407	0.163868	0.612439	0.1666	0.634176	no
25.1321	22.3397	-0.169921	-0.614828	0.16645	0.634176	no
3.42268	2.88096	-0.248578	-0.616593	0.1663	0.634176	no
19.1966	17.1306	-0.164277	-0.612726	0.1664	0.634176	no
13.4221	15.3184	0.190651	0.607768	0.16735	0.634519	no
12.2102	10.5162	-0.21547	-0.61468	0.1674	0.634519	no
61.9414	70.1258	0.179039	0.610557	0.16715	0.634519	no
33.4916	29.8409	-0.166505	-0.611323	0.16735	0.634519	no
8.10707	6.62626	-0.290986	-0.614459	0.16745	0.634519	no
2.89744	2.25545	-0.361364	-0.613378	0.1674	0.634519	no
15.4262	13.7678	-0.164083	-0.608422	0.16705	0.634519	no
45.9888	52.2564	0.184324	0.609791	0.1671	0.634519	no
1.97452	2.41097	0.288113	0.599981	0.16695	0.634519	no
19.9493	22.6511	0.183247	0.619472	0.1673	0.634519	no
18.5869	16.5746	-0.165317	-0.612576	0.1672	0.634519	no
7.42486	6.52881	-0.185544	-0.61545	0.16715	0.634519	no
8.52589	7.28934	-0.226063	-0.611271	0.16695	0.634519	no
3.90928	3.17008	-0.302385	-0.613704	0.1678	0.63543	no

82.8221	73.8088	-0.166224	-0.605614	0.16775	0.63543	no
32.2636	36.7539	0.18799	0.604394	0.1679	0.635602	no
2.85252	3.25394	0.189951	0.605995	0.1681	0.635909	no
41.3968	46.5889	0.170466	0.609134	0.16805	0.635909	no
9.1607	10.5099	0.198217	0.613126	0.1682	0.635909	no
4.13677	3.47699	-0.250664	-0.608088	0.1682	0.635909	no
7.48839	8.59286	0.198483	0.606305	0.1685	0.636422	no
5.01055	6.02679	0.266421	0.608797	0.1685	0.636422	no
5.21552	4.48135	-0.21888	-0.60661	0.16845	0.636422	no
19.8136	22.2823	0.169403	0.61416	0.16875	0.637159	no
22.9382	25.6531	0.161382	0.602562	0.16895	0.637651	no
17.3231	19.4992	0.170718	0.600252	0.1691	0.637651	no
41.8124	48.2568	0.206803	0.607394	0.16905	0.637651	no
13.9279	12.391	-0.168683	-0.608212	0.1691	0.637651	no
35.6371	39.9416	0.16451	0.605947	0.1693	0.637973	no
34.7077	39.3911	0.182612	0.610841	0.16935	0.637973	no
7.22905	8.4109	0.218454	0.612137	0.1693	0.637973	no
1.82414	2.34983	0.36534	0.597859	0.1695	0.638069	no
8.19869	9.37059	0.192746	0.604106	0.16965	0.638069	no
33.4888	29.8551	-0.165702	-0.614705	0.16965	0.638069	no
44.1582	39.1896	-0.17221	-0.614368	0.16955	0.638069	no
646.892	453.237	-0.513259	-0.623895	0.16945	0.638069	no
31.4038	35.2602	0.167101	0.618186	0.16985	0.638614	no
2.42203	3.16891	0.38777	0.616736	0.1702	0.639704	no
37.6882	42.2374	0.16441	0.606336	0.1704	0.640061	no
9.16518	7.99725	-0.196659	-0.616569	0.17075	0.640547	no
9.77001	8.54275	-0.193658	-0.606975	0.1707	0.640547	no
37.0852	41.641	0.16716	0.601522	0.1707	0.640547	no
3.82942	4.72206	0.302291	0.614855	0.1706	0.640547	no
3.70517	3.1443	-0.236798	-0.616524	0.171	0.641278	no
25.305	22.4366	-0.173567	-0.604245	0.1711	0.641446	no
2.37574	3.23757	0.446535	0.605779	0.1712	0.641613	no
3.16831	3.71236	0.22862	0.606601	0.17165	0.642264	no
33.5756	29.5749	-0.18304	-0.607502	0.17155	0.642264	no
62.0642	55.3969	-0.163956	-0.610165	0.17165	0.642264	no
8.70115	7.36235	-0.24104	-0.612457	0.17145	0.642264	no
10.4843	12.0312	0.198551	0.604139	0.1722	0.64244	no
4.28193	6.9592	0.700662	0.583364	0.17225	0.64244	no
6.24868	5.02292	-0.315025	-0.599142	0.17195	0.64244	no
1.62896	2.13159	0.387977	0.604991	0.17205	0.64244	no
2.4503	3.06539	0.323111	0.588652	0.17215	0.64244	no
5.15631	9.92818	0.94519	0.6585	0.1721	0.64244	no
13.4691	11.8916	-0.179708	-0.609919	0.17215	0.64244	no
2.31666	2.83706	0.292353	0.599669	0.17215	0.64244	no
3.77578	3.1345	-0.26854	-0.603129	0.17215	0.64244	no
21.7494	18.3674	-0.243831	-0.612183	0.17225	0.64244	no
5.23435	4.10455	-0.350787	-0.608847	0.1724	0.642793	no

7.29036	8.46622	0.215727	0.611241	0.1725	0.64292	no
3.27483	3.91168	0.256366	0.610699	0.17255	0.64292	no
4.21656	3.61508	-0.22204	-0.604958	0.1729	0.643418	no
102.244	117.244	0.197507	0.608677	0.17285	0.643418	no
18.0119	20.3014	0.17263	0.599666	0.17285	0.643418	no
3.8088	3.21659	-0.243806	-0.601235	0.173	0.643584	no
24.5907	27.6008	0.166595	0.605801	0.1731	0.64375	no
9.20405	10.8018	0.230937	0.607292	0.17345	0.644433	no
51.4838	57.8131	0.167279	0.605228	0.17345	0.644433	no
8.8764	7.29396	-0.283272	-0.611436	0.1734	0.644433	no
14.1889	15.932	0.167166	0.604677	0.17355	0.644598	no
37.4744	41.8795	0.160341	0.598663	0.1737	0.644949	no
168.588	189.321	0.167327	0.610794	0.1739	0.645424	no
8.48861	10.0077	0.237517	0.604973	0.17395	0.645424	no
189.993	213.091	0.165523	0.598458	0.17425	0.645753	no
26.8119	21.0872	-0.346504	-0.603719	0.17425	0.645753	no
48.5453	54.3372	0.162608	0.600977	0.1746	0.645814	no
12.3415	15.6166	0.339559	0.607529	0.1745	0.645814	no
48.1303	42.8093	-0.169021	-0.604369	0.17455	0.645814	no
26.8842	30.0882	0.162439	0.601778	0.1745	0.645814	no
19.7654	22.3767	0.179017	0.601935	0.17445	0.645814	no
5.20678	4.62282	-0.171619	-0.608663	0.17445	0.645814	no
35.8838	41.1748	0.198432	0.600338	0.17475	0.645958	no
25.4825	28.8426	0.178699	0.605691	0.1747	0.645958	no
7.90195	6.9707	-0.180906	-0.607836	0.17485	0.646122	no
4.39032	3.84775	-0.190313	-0.596138	0.175	0.646471	no
17.6141	20.0983	0.190342	0.59969	0.17525	0.647188	no
2.2252	3.04936	0.454572	0.619731	0.1754	0.647331	no
9.10444	11.0887	0.284441	0.601056	0.1754	0.647331	no
3.43682	4.1629	0.276515	0.602275	0.1757	0.648026	no
33.0021	29.4156	-0.165975	-0.599198	0.17565	0.648026	no
16.1088	14.1877	-0.183204	-0.602275	0.1759	0.648558	no
2.53102	2.05374	-0.301467	-0.607188	0.1761	0.648884	no
58.2072	65.2022	0.163723	0.604693	0.17625	0.64923	no
11.9603	10.1611	-0.235191	-0.602509	0.17635	0.649393	no
1.15273	1.5265	0.405177	0.609926	0.1767	0.650247	no
1.8769	1.41568	-0.406857	-0.589488	0.1767	0.650247	no
9.28078	10.548	0.184647	0.604484	0.17675	0.650247	no
21.5705	19.2649	-0.163089	-0.600583	0.17695	0.650777	no
163.491	184.471	0.174182	0.597063	0.1772	0.65149	no
22.5715	19.7565	-0.192173	-0.602252	0.17735	0.651629	no
14.5656	12.9586	-0.168659	-0.598654	0.1773	0.651629	no
5.5729	4.5967	-0.27783	-0.597827	0.17755	0.652158	no
28.8283	25.2743	-0.189817	-0.601185	0.17765	0.652319	no
22.7262	19.3779	-0.22994	-0.60346	0.17795	0.652325	no
10.3483	11.6002	0.164767	0.602454	0.1779	0.652325	no
17.4571	19.8779	0.18735	0.60487	0.1781	0.652325	no

37.874	42.3202	0.16014	0.5995	0.17795	0.652325	no
4.37669	3.73431	-0.228999	-0.601053	0.1783	0.652325	no
153.771	173.717	0.175959	0.594364	0.1785	0.652325	no
6.75404	5.69474	-0.246121	-0.606794	0.1778	0.652325	no
8.88895	10.3839	0.224269	0.602124	0.17815	0.652325	no
12.9235	11.3798	-0.183517	-0.592906	0.17855	0.652325	no
6.50687	5.51677	-0.238139	-0.598211	0.17835	0.652325	no
2.2643	3.2288	0.511935	0.585687	0.1782	0.652325	no
3.30634	4.52722	0.453391	0.601939	0.17845	0.652325	no
7.82701	6.87374	-0.187365	-0.60193	0.1779	0.652325	no
7.92088	9.29846	0.231331	0.598226	0.17845	0.652325	no
25.5047	28.6309	0.166811	0.606383	0.1783	0.652325	no
4.97237	3.87673	-0.359094	-0.588524	0.1783	0.652325	no
5.09159	4.33241	-0.232948	-0.596471	0.1788	0.652828	no
10.0746	8.92101	-0.175444	-0.586003	0.17875	0.652828	no
86.1284	96.454	0.163352	0.590068	0.1789	0.652988	no
12.0756	14.7395	0.28759	0.587552	0.1792	0.653832	no
2.8001	2.38504	-0.231466	-0.604283	0.1793	0.653832	no
103.175	92.3878	-0.159325	-0.590569	0.17925	0.653832	no
260.654	292.13	0.164477	0.593812	0.1796	0.654309	no
3.08468	2.61465	-0.238502	-0.603692	0.17955	0.654309	no
81.564	72.7691	-0.164607	-0.598154	0.17955	0.654309	no
1.7213	1.44317	-0.254255	-0.598122	0.1799	0.654353	no
3.49443	2.76273	-0.338961	-0.602146	0.1798	0.654353	no
2.88346	2.37109	-0.282251	-0.575035	0.1799	0.654353	no
2.49707	1.96125	-0.34846	-0.596347	0.17985	0.654353	no
3.3008	2.84031	-0.216766	-0.598269	0.17995	0.654353	no
42.9075	47.9544	0.160431	0.597185	0.1804	0.655579	no
7.5268	6.45808	-0.220929	-0.60112	0.18035	0.655579	no
12.835	11.4174	-0.168846	-0.596498	0.1808	0.656622	no
2.26387	1.80171	-0.329428	-0.591727	0.1808	0.656622	no
26.3572	23.3728	-0.173371	-0.583545	0.181	0.657095	no
7.17291	8.2624	0.204002	0.590144	0.1811	0.657096	no
31.2435	34.9211	0.160544	0.601429	0.1814	0.657773	no
1.82373	2.26815	0.314628	0.58859	0.18135	0.657773	no
22.1969	24.7649	0.157939	0.586692	0.1819	0.658545	no
13.3939	15.0876	0.171787	0.588411	0.1826	0.658545	no
280.405	323.739	0.207319	0.59504	0.18205	0.658545	no
75.957	84.997	0.162229	0.588796	0.1828	0.658545	no
2.18298	2.82219	0.370516	0.604678	0.18235	0.658545	no
29.4849	26.3954	-0.159688	-0.589207	0.18275	0.658545	no
27.8602	24.9488	-0.159236	-0.588748	0.1832	0.658545	no
16.5067	19.2846	0.224396	0.589953	0.1832	0.658545	no
14.2118	15.9195	0.163703	0.595521	0.1831	0.658545	no
20.643	23.301	0.174739	0.577138	0.18295	0.658545	no
121.731	141.934	0.221523	0.583602	0.1824	0.658545	no
889.18	1054.58	0.246116	0.591172	0.1823	0.658545	no

8.11516	9.29233	0.195421	0.592996	0.18305	0.658545	no
1.56448	1.88291	0.267284	0.587057	0.18315	0.658545	no
10.3137	9.10471	-0.179877	-0.593691	0.1824	0.658545	no
2.18757	2.8474	0.38032	0.583446	0.1832	0.658545	no
29.1023	25.3042	-0.201757	-0.591368	0.18235	0.658545	no
56.3819	62.9332	0.158589	0.593594	0.1819	0.658545	no
10.6299	8.22831	-0.369457	-0.595081	0.1817	0.658545	no
3.53177	3.01724	-0.227159	-0.597023	0.1825	0.658545	no
5.20791	4.58821	-0.182774	-0.594948	0.18255	0.658545	no
38.4958	43.0219	0.160367	0.599627	0.1826	0.658545	no
34.3406	28.3617	-0.275973	-0.589004	0.1829	0.658545	no
8.07182	9.38908	0.218089	0.594535	0.18285	0.658545	no
48.0454	53.848	0.164495	0.593182	0.18225	0.658545	no
1.45227	1.05041	-0.467358	-0.391483	0.1823	0.658545	no
63.0148	70.3585	0.159036	0.589678	0.18205	0.658545	no
3.0965	2.49289	-0.31282	-0.581989	0.18175	0.658545	no
3.92613	4.62933	0.237696	0.594594	0.18335	0.658677	no
1.23804	1.63822	0.404067	0.594676	0.1833	0.658677	no
9.49117	8.4074	-0.174927	-0.592738	0.184	0.659033	no
17.891	15.9608	-0.164706	-0.594038	0.18385	0.659033	no
6.04025	4.56809	-0.40302	-0.584718	0.18375	0.659033	no
13.2727	17.774	0.421303	0.586032	0.1842	0.659033	no
7.034	6.2508	-0.170304	-0.588879	0.18405	0.659033	no
22.3341	20.0224	-0.157633	-0.590328	0.1843	0.659033	no
5.50376	4.73379	-0.217422	-0.593575	0.1843	0.659033	no
36.5573	32.2576	-0.180518	-0.585378	0.1839	0.659033	no
35.5415	31.7024	-0.164912	-0.589671	0.18375	0.659033	no
22.2761	19.9628	-0.158186	-0.592658	0.1843	0.659033	no
9.14055	8.1692	-0.162086	-0.594734	0.18355	0.659033	no
2.90294	2.44082	-0.250146	-0.592715	0.18395	0.659033	no
30.473	33.9598	0.156299	0.587209	0.18365	0.659033	no
2.99056	2.58186	-0.212007	-0.595668	0.1842	0.659033	no
2.63717	3.87885	0.556635	0.599869	0.1844	0.659187	no
9.82704	11.9701	0.284608	0.584692	0.1847	0.65928	no
25.0977	29.1715	0.217005	0.59563	0.18475	0.65928	no
34.5174	38.7913	0.168409	0.589914	0.18455	0.65928	no
22.5081	25.1289	0.158899	0.583217	0.185	0.65928	no
4.00736	3.32508	-0.269264	-0.589774	0.18465	0.65928	no
10.8582	9.42383	-0.204401	-0.595081	0.1849	0.65928	no
37.2751	33.383	-0.1591	-0.594854	0.18505	0.65928	no
58.6346	65.5053	0.159858	0.589363	0.18485	0.65928	no
16.8895	15.067	-0.164732	-0.590784	0.18505	0.65928	no
41.1559	46.145	0.165074	0.593783	0.18475	0.65928	no
56.3204	49.6197	-0.182744	-0.585991	0.1852	0.659612	no
2.1073	2.605	0.305886	0.576314	0.18545	0.659693	no
5.05063	5.90826	0.226269	0.590841	0.18535	0.659693	no
16.3736	14.5211	-0.173223	-0.579881	0.1853	0.659693	no

34.23	39.5193	0.207296	0.58991	0.18555	0.659847	no
21.7271	24.4511	0.170401	0.586337	0.18575	0.659904	no
12.4873	14.7693	0.242142	0.580567	0.18585	0.659904	no
81.3241	91.492	0.169962	0.588694	0.18565	0.659904	no
1.49687	1.07082	-0.483238	-0.599644	0.18585	0.659904	no
11.0852	13.1801	0.249721	0.594473	0.18585	0.659904	no
117.612	134.655	0.195241	0.588957	0.18625	0.661122	no
11.9188	13.3086	0.159118	0.586069	0.1864	0.661452	no
3.18163	2.7216	-0.225315	-0.586517	0.18655	0.661581	no
16.9767	19.0079	0.163047	0.594521	0.18655	0.661581	no
17.1259	15.2563	-0.166772	-0.580396	0.1871	0.662647	no
1.43831	1.99856	0.474585	0.567475	0.187	0.662647	no
106.449	118.871	0.159231	0.583113	0.18725	0.662647	no
56.43	63.3448	0.166763	0.591126	0.18725	0.662647	no
116.988	131.088	0.164184	0.58337	0.18725	0.662647	no
9.89924	8.49114	-0.221359	-0.588001	0.187	0.662647	no
25.2173	28.1015	0.156234	0.579459	0.1877	0.663204	no
4.28663	3.54377	-0.274559	-0.586667	0.18775	0.663204	no
31.0579	34.5942	0.155569	0.579267	0.1876	0.663204	no
30.5029	34.1619	0.16344	0.584655	0.18765	0.663204	no
22.9402	26.7095	0.219475	0.583696	0.18755	0.663204	no
1.20563	1.5928	0.401781	0.583589	0.18775	0.663204	no
1.75503	2.37396	0.435804	0.595624	0.18785	0.663306	no
13.8144	16.149	0.225275	0.594953	0.1879	0.663306	no
5.17357	4.60986	-0.166437	-0.58361	0.18795	0.663306	no
7.44292	6.52801	-0.189225	-0.583782	0.1883	0.664138	no
7.45617	8.56654	0.200279	0.582147	0.1883	0.664138	no
8.2585	9.44002	0.19291	0.584408	0.18845	0.664465	no
4.47697	3.71149	-0.270523	-0.585846	0.18855	0.664616	no
1.65556	1.17689	-0.492339	-0.590305	0.1889	0.664841	no
3.32981	2.81093	-0.244395	-0.590849	0.18885	0.664841	no
13.8504	16.0077	0.208831	0.586417	0.1887	0.664841	no
28.5506	25.5986	-0.157452	-0.58586	0.18925	0.665065	no
23.8487	26.7349	0.164816	0.587358	0.1892	0.665065	no
273.289	309.37	0.178906	0.585152	0.1892	0.665065	no
49.5245	55.1716	0.155783	0.579266	0.18915	0.665065	no
1.50863	1.21867	-0.307933	-0.560908	0.1892	0.665065	no
2.99944	2.6172	-0.196672	-0.582636	0.1895	0.665541	no
4.3996	3.4015	-0.3712	-0.584084	0.18945	0.665541	no
45.069	50.4813	0.163612	0.578512	0.1896	0.665691	no
18.2043	16.3469	-0.155262	-0.578806	0.1897	0.665841	no
33.5569	30.1526	-0.154328	-0.572088	0.18985	0.66594	no
2.91896	2.34289	-0.317164	-0.573397	0.1899	0.66594	no
3.88437	5.27007	0.44014	0.580007	0.1898	0.66594	no
3.13557	3.80262	0.278268	0.58074	0.19	0.666064	no
2.63078	1.99332	-0.400317	-0.582023	0.19005	0.666064	no
14.7749	13.0581	-0.178207	-0.570291	0.19055	0.666208	no

10.3379	9.01882	-0.196939	-0.57921	0.1902	0.666208	no
467.226	528.234	0.177057	0.575882	0.1905	0.666208	no
15.3761	12.6442	-0.282205	-0.570828	0.1904	0.666208	no
11.8705	13.8707	0.224653	0.585088	0.19035	0.666208	no
59.3601	66.1099	0.155374	0.583462	0.1903	0.666208	no
3.45825	4.14378	0.260905	0.580365	0.19035	0.666208	no
29.3709	32.8243	0.160375	0.583399	0.1905	0.666208	no
15.0698	13.4981	-0.158904	-0.588301	0.19095	0.667406	no
18.731	20.9032	0.158299	0.587006	0.19145	0.667747	no
1819.46	1457.27	-0.320241	-0.580498	0.19135	0.667747	no
22.9168	20.5652	-0.156198	-0.584751	0.19145	0.667747	no
4.60483	5.77447	0.326542	0.573018	0.1912	0.667747	no
13.664	15.3991	0.172472	0.577574	0.19115	0.667747	no
4.16378	3.60962	-0.206048	-0.582278	0.1913	0.667747	no
2.54525	3.11375	0.290844	0.568098	0.1917	0.668419	no
37.7079	43.7657	0.214933	0.591151	0.1918	0.668567	no
6.91813	7.98929	0.207687	0.578386	0.1922	0.668878	no
5.35577	6.54444	0.289174	0.587179	0.192	0.668878	no
11.6063	12.9756	0.160882	0.583966	0.1923	0.668878	no
33.8363	37.6612	0.154503	0.576696	0.1921	0.668878	no
38.1422	42.5928	0.159222	0.581691	0.1923	0.668878	no
10.3813	9.32357	-0.155029	-0.573452	0.1923	0.668878	no
1.63214	1.27429	-0.35707	-0.580058	0.19235	0.668878	no
20.8148	18.3029	-0.185538	-0.585256	0.19265	0.66912	no
8.47052	7.50422	-0.174748	-0.575866	0.19255	0.66912	no
17.278	19.2814	0.158275	0.584274	0.19265	0.66912	no
7.07406	6.2598	-0.176423	-0.575521	0.19285	0.669388	no
11.7612	10.5317	-0.159306	-0.584122	0.19285	0.669388	no
13.7866	12.3357	-0.160426	-0.576079	0.1929	0.669388	no
6.5953	7.75179	0.233092	0.58062	0.1931	0.669882	no
5.80904	5.1591	-0.17118	-0.567228	0.1932	0.670029	no
1.32185	1.81052	0.45385	0.585939	0.1934	0.670323	no
22.283	25.1021	0.171867	0.583366	0.19335	0.670323	no
12.5833	14.1149	0.165709	0.580023	0.19365	0.670589	no
56.9422	63.4025	0.155041	0.570476	0.19365	0.670589	no
5.03728	3.87912	-0.376918	-0.582311	0.1936	0.670589	no
16.4854	14.7263	-0.162792	-0.578163	0.1938	0.670908	no
5.69912	6.61372	0.214723	0.577056	0.19395	0.671028	no
6.77167	8.0512	0.249694	0.576805	0.1942	0.671293	no
1.906	1.37351	-0.47268	-0.564516	0.1941	0.671293	no
11.8229	10.5498	-0.164367	-0.579702	0.19415	0.671293	no
5.66697	6.49804	0.197426	0.573582	0.19435	0.671612	no
38.5816	34.2963	-0.169861	-0.573327	0.19455	0.672022	no
38.7459	43.2823	0.159733	0.572235	0.1947	0.672022	no
3.40678	3.88472	0.189404	0.579395	0.1946	0.672022	no
3.85715	3.16997	-0.283065	-0.581967	0.1947	0.672022	no
2.33284	1.89697	-0.298391	-0.565156	0.1948	0.672167	no

4.64817	3.87993	-0.260632	-0.577873	0.195	0.672657	no
18.195	22.0534	0.277458	0.573956	0.19535	0.673665	no
8.1596	9.43218	0.209094	0.581336	0.19555	0.674127	no
50.4666	45.2967	-0.155923	-0.580268	0.1956	0.674127	no
6.93295	7.79029	0.168207	0.57161	0.1957	0.674271	no
38.911	43.3593	0.156163	0.571135	0.1963	0.674715	no
28.6544	32.0683	0.162389	0.577494	0.19665	0.674715	no
5.21863	6.33387	0.279417	0.56892	0.19665	0.674715	no
8.42397	7.13934	-0.238709	-0.580502	0.1966	0.674715	no
18.5494	16.6032	-0.15991	-0.577761	0.19625	0.674715	no
27.7936	31.2451	0.16888	0.574613	0.196	0.674715	no
2.87645	3.30048	0.198385	0.578232	0.1965	0.674715	no
10.8758	9.22032	-0.238234	-0.577496	0.19615	0.674715	no
9.44192	8.44867	-0.160356	-0.576939	0.1961	0.674715	no
12.5657	14.7465	0.230892	0.576288	0.1967	0.674715	no
15.1368	13.4798	-0.167263	-0.574675	0.1965	0.674715	no
1.78983	1.36428	-0.391688	-0.576096	0.1959	0.674715	no
37.8197	42.0385	0.152574	0.572131	0.1962	0.674715	no
10.7187	12.1057	0.175551	0.569673	0.1967	0.674715	no
1.9987	1.60825	-0.313568	-0.566272	0.1965	0.674715	no
5.71313	4.86289	-0.232467	-0.569965	0.1969	0.674975	no
19.8295	22.7621	0.198988	0.572643	0.19695	0.674975	no
15.3557	17.3738	0.178139	0.568078	0.19735	0.675294	no
11.5574	10.2786	-0.169174	-0.573829	0.1973	0.675294	no
23.4097	26.3499	0.170694	0.576271	0.1972	0.675294	no
20.2767	22.8089	0.169774	0.571763	0.1974	0.675294	no
6.10384	5.33878	-0.193208	-0.580388	0.1973	0.675294	no
31.3384	34.8812	0.154519	0.582105	0.19745	0.675294	no
4.18762	4.92413	0.23374	0.575381	0.19725	0.675294	no
13.5462	11.6917	-0.212394	-0.575439	0.1976	0.675608	no
7.97873	7.06764	-0.17493	-0.571154	0.19775	0.675723	no
14.5817	13.1057	-0.153969	-0.573813	0.19775	0.675723	no
5.36862	4.45978	-0.267579	-0.574701	0.19785	0.675866	no
2.43644	1.84847	-0.398445	-0.579444	0.19795	0.676009	no
2.73287	2.17035	-0.332484	-0.569012	0.19815	0.676239	no
2.20822	1.70608	-0.372196	-0.575136	0.19815	0.676239	no
13.0326	14.6864	0.172358	0.575076	0.19825	0.676239	no
20.2899	22.7885	0.167543	0.575619	0.1982	0.676239	no
3.92792	4.7544	0.275496	0.569421	0.1986	0.676439	no
1.7069	2.15567	0.33676	0.574183	0.1986	0.676439	no
2.7298	2.06587	-0.402047	-0.571776	0.19845	0.676439	no
5.80213	4.61907	-0.328978	-0.573048	0.19855	0.676439	no
40.2055	44.8928	0.159091	0.562407	0.1987	0.676554	no
79.3266	89.3491	0.171649	0.568865	0.19875	0.676554	no
5.78187	5.01952	-0.203988	-0.579957	0.19895	0.677036	no
15.7543	17.9372	0.187214	0.569834	0.19905	0.677178	no
31.4556	35.0492	0.156068	0.581736	0.19945	0.678226	no

3.32849	2.75068	-0.275078	-0.571717	0.19955	0.678226	no
4.13006	5.36219	0.37666	0.582673	0.1996	0.678226	no
45.7458	41.0566	-0.156024	-0.576159	0.19965	0.678226	no
10.7866	12.227	0.180823	0.570062	0.19955	0.678226	no
23.5688	21.2117	-0.152021	-0.565309	0.1999	0.678282	no
14.7878	13.2725	-0.155969	-0.574262	0.19975	0.678282	no
45.7758	50.9169	0.153559	0.565473	0.1999	0.678282	no
18.23	16.2016	-0.170182	-0.5675	0.1999	0.678282	no
536.21	605.351	0.174974	0.568553	0.20005	0.678451	no
8.07598	9.26137	0.197589	0.566193	0.20025	0.678451	no
5.10434	4.5259	-0.173522	-0.564832	0.20025	0.678451	no
49.9594	56.0761	0.166632	0.564654	0.2002	0.678451	no
46.2009	51.4485	0.155207	0.571133	0.2001	0.678451	no
3.44662	4.07453	0.241452	0.571119	0.2003	0.678451	no
7.34491	6.55436	-0.164291	-0.568558	0.2005	0.67893	no
11.8461	10.6241	-0.157072	-0.573082	0.2008	0.679748	no
23.4981	26.0777	0.150273	0.559945	0.201	0.680029	no
7.19996	8.4547	0.231765	0.56558	0.201	0.680029	no
4.98194	5.86162	0.234594	0.576544	0.20125	0.68028	no
10.2697	11.5242	0.166275	0.57335	0.2012	0.68028	no
9.3662	10.868	0.21455	0.574101	0.20115	0.68028	no
6.34245	7.2639	0.195703	0.571626	0.2014	0.680362	no
5.85901	5.01905	-0.223243	-0.568436	0.20135	0.680362	no
21.6621	24.0702	0.152075	0.566065	0.20145	0.680362	no
7.37717	6.58205	-0.16453	-0.564503	0.20155	0.680502	no
9.51121	11.5331	0.278076	0.56852	0.20165	0.680613	no
21.6117	24.4624	0.178752	0.56004	0.2017	0.680613	no
1416.75	1722.21	0.28167	0.565448	0.20185	0.680863	no
11.3419	10.1561	-0.159326	-0.563981	0.20195	0.680863	no
6.58973	5.82731	-0.177389	-0.559216	0.20195	0.680863	no
28.4624	25.0769	-0.182698	-0.568563	0.2022	0.681392	no
41.8226	36.8174	-0.183898	-0.564484	0.2023	0.681392	no
9.02461	11.3157	0.326385	0.569034	0.2024	0.681392	no
24.3962	21.7038	-0.168709	-0.568624	0.20235	0.681392	no
17.4571	15.1858	-0.201086	-0.570528	0.20255	0.681699	no
3.4642	2.97651	-0.218898	-0.567	0.2027	0.682007	no
22.2957	19.857	-0.167123	-0.555218	0.2028	0.682116	no
30.7106	34.1969	0.155131	0.562195	0.20285	0.682116	no
26.6392	29.7792	0.160754	0.559623	0.20305	0.682591	no
1.99597	2.65724	0.412836	0.555444	0.2032	0.6827	no
51.0794	56.7617	0.152175	0.564749	0.2032	0.6827	no
3.24173	3.77836	0.220996	0.566203	0.2034	0.682771	no
92.0856	102.187	0.150165	0.559597	0.2037	0.682771	no
12.1375	10.825	-0.165104	-0.565803	0.20355	0.682771	no
4.48102	3.71541	-0.270306	-0.562057	0.2033	0.682771	no
9.57971	10.7611	0.167765	0.565487	0.2036	0.682771	no
3.07274	2.59415	-0.244264	-0.564291	0.20375	0.682771	no

51.9302	57.7178	0.152442	0.562878	0.20365	0.682771	no
3.08104	2.59147	-0.249648	-0.558837	0.20365	0.682771	no
15.443	17.8699	0.210575	0.569765	0.20365	0.682771	no
9.06606	10.3675	0.193527	0.565981	0.20385	0.682909	no
3.1528	2.60153	-0.277273	-0.564969	0.20395	0.683047	no
2.87629	2.43505	-0.240262	-0.574623	0.20405	0.683185	no
90.5354	101.241	0.161237	0.564894	0.2042	0.683294	no
39.5511	43.9127	0.150923	0.565651	0.2052	0.683294	no
5.86782	6.92394	0.238769	0.562639	0.2047	0.683294	no
11.7292	13.4213	0.194425	0.564603	0.20465	0.683294	no
7.25399	5.71197	-0.344785	-0.558299	0.2052	0.683294	no
8.27507	9.52466	0.202896	0.564172	0.20515	0.683294	no
0.967681	1.45504	0.588453	0.576229	0.2051	0.683294	no
26.4563	29.541	0.159109	0.558816	0.205	0.683294	no
13.967	15.5257	0.152634	0.559144	0.2047	0.683294	no
3.37918	2.7405	-0.302236	-0.551757	0.2049	0.683294	no
23.3244	20.8848	-0.159389	-0.558239	0.20505	0.683294	no
17.1109	13.9181	-0.297949	-0.560944	0.20455	0.683294	no
59.659	53.454	-0.158443	-0.568377	0.2044	0.683294	no
50.1507	45.1321	-0.152117	-0.566812	0.205	0.683294	no
73.4671	81.6295	0.151992	0.569004	0.20435	0.683294	no
10.5905	12.4594	0.234459	0.567465	0.2047	0.683294	no
34.1763	38.8876	0.186314	0.566042	0.20475	0.683294	no
3.4587	2.95924	-0.225	-0.56633	0.2043	0.683294	no
8.53674	7.68026	-0.15253	-0.563584	0.2054	0.683373	no
38.2715	42.7002	0.157974	0.568202	0.2053	0.683373	no
6.72015	7.94622	0.241776	0.572829	0.2054	0.683373	no
1.25688	1.5744	0.324959	0.562585	0.20565	0.683617	no
8.85276	7.80526	-0.18168	-0.562436	0.2056	0.683617	no
12.1822	10.8414	-0.16823	-0.557565	0.2056	0.683617	no
1405.97	1229.01	-0.194073	-0.567866	0.2059	0.684252	no
52.7405	58.5456	0.150649	0.562733	0.20625	0.684437	no
1.55778	1.97373	0.341437	0.567347	0.20615	0.684437	no
13.0708	10.7393	-0.283453	-0.563332	0.20605	0.684437	no
3.05607	2.54625	-0.263307	-0.572102	0.20615	0.684437	no
51.5761	57.2445	0.150435	0.562595	0.2062	0.684437	no
2.67464	2.38486	-0.165442	-0.562571	0.20635	0.684573	no
1.44108	1.98824	0.464337	0.567728	0.20665	0.685372	no
7.15894	5.69888	-0.329069	-0.556005	0.20695	0.686172	no
8.2952	9.24648	0.156628	0.559484	0.20725	0.686744	no
9.30556	7.68636	-0.275792	-0.560163	0.2073	0.686744	no
3.3415	2.93212	-0.18855	-0.558619	0.20745	0.687045	no
25.4622	28.5745	0.166373	0.561801	0.20755	0.68718	no
32.9347	36.5911	0.151881	0.56788	0.2077	0.687285	no
5.67147	6.67762	0.235612	0.569393	0.20765	0.687285	no
2.60294	2.09938	-0.310179	-0.565113	0.20785	0.68739	no
6.99493	6.22071	-0.169229	-0.567385	0.20795	0.687525	no

9.70743	8.55057	-0.183069	-0.549413	0.2082	0.688156	no
4.15907	5.14378	0.306569	0.558159	0.2084	0.688229	no
81.0715	72.8231	-0.154797	-0.55365	0.2084	0.688229	no
16.6809	18.8591	0.177059	0.556518	0.2083	0.688229	no
11.8583	13.8266	0.221551	0.556666	0.2089	0.688748	no
5.9495	5.22595	-0.187077	-0.555249	0.20875	0.688748	no
1.74394	1.28919	-0.435892	-0.568388	0.2089	0.688748	no
11.5549	12.8895	0.157691	0.559328	0.20895	0.688748	no
6.56629	10.2362	0.640537	0.613325	0.2091	0.688748	no
29.7947	33.1598	0.154381	0.557596	0.20915	0.688748	no
6.00712	7.02262	0.225336	0.565221	0.20905	0.688748	no
135.197	119.591	-0.176957	-0.554676	0.2091	0.688748	no
2.04498	2.60159	0.347308	0.559248	0.2091	0.688748	no
138.959	161.591	0.21769	0.561408	0.2094	0.688955	no
3.32635	2.80675	-0.24504	-0.560886	0.20935	0.688955	no
34.9462	38.785	0.150366	0.561481	0.2093	0.688955	no
8.84143	10.1791	0.20326	0.5576	0.20945	0.688955	no
41.5849	46.6418	0.165562	0.548858	0.2098	0.689737	no
2.43239	2.02539	-0.264178	-0.560286	0.2103	0.689737	no
9.98516	8.82071	-0.178891	-0.554486	0.2103	0.689737	no
26.927	29.9543	0.15371	0.554829	0.21025	0.689737	no
4.7147	5.4407	0.206628	0.558348	0.21015	0.689737	no
64.3625	71.3553	0.1488	0.557295	0.2102	0.689737	no
1.5132	1.27456	-0.247602	-0.548068	0.2101	0.689737	no
16.0007	14.3912	-0.152952	-0.563374	0.21025	0.689737	no
72.9112	65.5757	-0.152981	-0.551886	0.2102	0.689737	no
498.033	568.343	0.19052	0.562719	0.21	0.689737	no
9.82995	11.1211	0.178046	0.561901	0.21035	0.689737	no
11.6426	10.4397	-0.157338	-0.564416	0.2104	0.689737	no
12.6543	11.2242	-0.173012	-0.558488	0.21055	0.690034	no
1.95037	2.32576	0.253954	0.561102	0.21065	0.690167	no
20.2425	22.652	0.162252	0.560458	0.21075	0.6903	no
29.599	33.3272	0.171149	0.551735	0.21085	0.690433	no
3.77784	4.62084	0.290596	0.542487	0.2111	0.691057	no
9.39878	11.0816	0.237625	0.559601	0.2113	0.691322	no
780.923	919.858	0.23623	0.555506	0.2113	0.691322	no
39.894	35.8616	-0.153732	-0.55177	0.21145	0.691618	no
35.3547	39.2541	0.150941	0.552318	0.21155	0.691688	no
7.70728	6.90174	-0.159262	-0.555274	0.2116	0.691688	no
7.29662	6.39649	-0.189948	-0.56146	0.21165	0.691688	no
5.51663	4.93488	-0.160773	-0.559935	0.2118	0.691758	no
3.01148	2.32142	-0.375462	-0.560801	0.21185	0.691758	no
7.48421	8.51688	0.186476	0.555939	0.21175	0.691758	no
7.44917	8.42646	0.177846	0.546023	0.2122	0.692317	no
4.83416	3.61924	-0.41758	-0.552888	0.2122	0.692317	no
5.3215	4.71267	-0.175288	-0.56176	0.21215	0.692317	no
8.76801	7.64965	-0.196854	-0.559765	0.21235	0.692417	no

3.67564	3.1409	-0.226816	-0.55983	0.21235	0.692417	no
19.3924	17.4856	-0.149323	-0.557017	0.2125	0.692712	no
20.4266	22.6661	0.150086	0.554363	0.2127	0.693169	no
803.849	931.942	0.213315	0.552166	0.213	0.693306	no
4.26763	5.09904	0.256789	0.554886	0.2131	0.693306	no
62.7434	56.412	-0.153462	-0.554812	0.2131	0.693306	no
22.9223	25.588	0.158713	0.553444	0.213	0.693306	no
11.7903	13.3412	0.178285	0.552999	0.21295	0.693306	no
36.8029	41.1198	0.160012	0.549655	0.2131	0.693306	no
3.67158	4.63454	0.336026	0.559054	0.21345	0.693862	no
6.54277	8.45851	0.370502	0.560829	0.2134	0.693862	no
9.00345	7.22486	-0.317508	-0.550592	0.21345	0.693862	no
5.13836	4.34707	-0.241265	-0.549009	0.21365	0.693929	no
1.03454	1.42024	0.457141	0.556471	0.21355	0.693929	no
17.8498	15.8952	-0.167316	-0.540939	0.2136	0.693929	no
28.5309	31.9618	0.163824	0.551543	0.21385	0.693997	no
5.8702	4.79683	-0.291329	-0.549649	0.21385	0.693997	no
15.3311	17.367	0.179886	0.552145	0.21375	0.693997	no
45.1726	40.761	-0.148259	-0.554029	0.21405	0.694258	no
104.573	115.917	0.148579	0.555413	0.21425	0.694519	no
12.8852	15.1219	0.230928	0.557749	0.21425	0.694519	no
2.43509	1.90107	-0.357162	-0.56052	0.21445	0.694974	no
12.0038	10.8557	-0.145043	-0.544201	0.2147	0.695396	no
32.2561	28.9384	-0.15659	-0.552686	0.21465	0.695396	no
4.94293	4.22079	-0.227855	-0.554196	0.2148	0.695526	no
4.46296	3.84562	-0.214784	-0.553574	0.21495	0.69556	no
2.46543	2.04409	-0.27038	-0.542885	0.21505	0.69556	no
24.721	27.7442	0.166449	0.547767	0.215	0.69556	no
1.70496	2.64097	0.631331	0.562566	0.2155	0.696434	no
27.6709	24.9141	-0.151409	-0.554015	0.21545	0.696434	no
8.24696	9.33973	0.179519	0.557268	0.2157	0.696499	no
31.2111	34.599	0.148672	0.552366	0.21565	0.696499	no
15.0751	13.5651	-0.152261	-0.553189	0.21565	0.696499	no
4.18813	3.3813	-0.308725	-0.541567	0.21635	0.696822	no
10.6944	12.1753	0.187106	0.54763	0.2163	0.696822	no
4.78624	2.41148	-0.988973	-0.576535	0.2164	0.696822	no
60.0023	66.8483	0.155874	0.550513	0.2161	0.696822	no
5.08168	4.30788	-0.238328	-0.551284	0.2162	0.696822	no
2.36859	1.90814	-0.311862	-0.555962	0.2161	0.696822	no
28.3308	31.4318	0.149855	0.549422	0.21615	0.696822	no
3.52081	2.94991	-0.255238	-0.549641	0.2159	0.696822	no
684.759	614.726	-0.155655	-0.558182	0.21635	0.696822	no
47.2516	52.4915	0.151721	0.55218	0.2164	0.696822	no
2.89722	3.50775	0.275879	0.550718	0.21655	0.696919	no
10.0614	11.4551	0.187168	0.54119	0.21655	0.696919	no
7.03622	6.25489	-0.169816	-0.540657	0.2171	0.698431	no
14.3487	16.4655	0.198528	0.554836	0.2172	0.698431	no

23.9461	20.9858	-0.190378	-0.553711	0.2172	0.698431	no
15.0365	17.2965	0.20201	0.546806	0.21755	0.698943	no
12.5172	10.5338	-0.248887	-0.547779	0.2176	0.698943	no
4.29197	3.527	-0.283197	-0.539018	0.2175	0.698943	no
5.61401	6.58109	0.229294	0.546726	0.2176	0.698943	no
3.08926	2.40099	-0.363631	-0.553633	0.2177	0.699071	no
37.2251	33.6181	-0.147037	-0.546719	0.21785	0.699359	no
2.87549	2.20963	-0.380003	-0.547157	0.2182	0.699837	no
2.11546	2.62104	0.309167	0.550179	0.2183	0.699837	no
27.572	24.7938	-0.153226	-0.545447	0.2183	0.699837	no
3.94382	5.08969	0.367984	0.557415	0.21815	0.699837	no
2.82642	3.54874	0.32833	0.54122	0.2181	0.699837	no
6.81165	7.88778	0.211615	0.547387	0.2185	0.699858	no
34.3697	38.0646	0.147313	0.546323	0.2186	0.699858	no
8.30861	7.49204	-0.149249	-0.539296	0.2191	0.699858	no
14.7323	16.3666	0.151764	0.551283	0.2191	0.699858	no
4.82772	4.11461	-0.230586	-0.546287	0.21885	0.699858	no
28.2441	31.7678	0.169617	0.538662	0.21855	0.699858	no
64.9324	74.3864	0.196098	0.547212	0.2191	0.699858	no
29.8897	26.6372	-0.166205	-0.537625	0.21875	0.699858	no
4.27176	3.66736	-0.220088	-0.54139	0.21905	0.699858	no
7.94571	9.33002	0.231703	0.549891	0.21915	0.699858	no
9.55549	10.9789	0.200333	0.547691	0.2189	0.699858	no
5.89955	5.1568	-0.194127	-0.549721	0.2191	0.699858	no
5.82415	5.1006	-0.191381	-0.548745	0.21845	0.699858	no
17.8464	19.9501	0.160764	0.539643	0.2193	0.700145	no
24.5696	21.9107	-0.16524	-0.549174	0.2197	0.701037	no
111.213	99.1861	-0.165118	-0.543904	0.2199	0.701257	no
6.64241	7.52277	0.179558	0.543783	0.2199	0.701257	no
12.644	11.0483	-0.194626	-0.552789	0.21995	0.701257	no
17.1581	18.9767	0.145338	0.541436	0.22005	0.701383	no
11.3353	10.2056	-0.151459	-0.546605	0.2203	0.70141	no
9.2271	10.4266	0.176313	0.543991	0.2203	0.70141	no
17.8254	16.0069	-0.15524	-0.542909	0.2202	0.70141	no
46.1379	51.2921	0.152784	0.550516	0.22025	0.70141	no
7.13497	7.95856	0.157601	0.546938	0.2206	0.702173	no
29.7003	32.9595	0.150218	0.544177	0.2207	0.702299	no
4.20468	4.83027	0.200106	0.548446	0.2208	0.702425	no
175.217	195.538	0.158308	0.538836	0.22095	0.702709	no
158.598	176.845	0.157111	0.542452	0.2212	0.702927	no
5.2965	4.5765	-0.210796	-0.545963	0.2212	0.702927	no
1.27862	1.60693	0.329723	0.548794	0.2211	0.702927	no
62.1401	69.7346	0.16635	0.540664	0.22135	0.703211	no
20.0761	22.3773	0.156553	0.550358	0.22145	0.703303	no
9.39336	10.6804	0.185247	0.545346	0.2215	0.703303	no
30.8321	27.8705	-0.145696	-0.543526	0.2218	0.703487	no
6.12369	5.46574	-0.163985	-0.54288	0.22165	0.703487	no

4.74612	3.94301	-0.267452	-0.543255	0.2217	0.703487	no
27.1796	30.1683	0.150507	0.547644	0.2218	0.703487	no
19.3975	25.1548	0.374958	0.546917	0.22205	0.703512	no
41.2375	48.9095	0.246158	0.543428	0.2219	0.703512	no
9.3135	7.8039	-0.255129	-0.547226	0.22205	0.703512	no
17.2063	19.0219	0.144727	0.537763	0.22195	0.703512	no
23.4893	21.2209	-0.146515	-0.548068	0.22225	0.703761	no
2.19554	1.79839	-0.287863	-0.534114	0.22225	0.703761	no
4.89738	4.1282	-0.246495	-0.539214	0.22265	0.704644	no
8.54759	7.60296	-0.168956	-0.542806	0.22265	0.704644	no
2.34254	1.98728	-0.237278	-0.543842	0.2229	0.705243	no
20.2657	22.6601	0.16111	0.543403	0.223	0.705367	no
9.84989	8.60512	-0.194912	-0.542803	0.2232	0.705808	no
20.6168	22.7795	0.143915	0.537917	0.22345	0.70583	no
1.48279	1.24241	-0.255175	-0.5393	0.2233	0.70583	no
12.4288	10.7054	-0.215349	-0.535526	0.22345	0.70583	no
3.88432	3.19301	-0.282745	-0.538359	0.22335	0.70583	no
15.7425	13.8872	-0.180906	-0.546024	0.2236	0.70592	no
27.784	30.8178	0.149507	0.53975	0.2236	0.70592	no
14.1745	15.6628	0.144042	0.540414	0.22475	0.7065	no
6.63105	5.82986	-0.185777	-0.540759	0.225	0.7065	no
29.4576	33.2677	0.17548	0.540601	0.2249	0.7065	no
12.5141	14.9266	0.25433	0.5376	0.2242	0.7065	no
2.95971	3.87726	0.389582	0.53777	0.22495	0.7065	no
16.9038	19.3663	0.196201	0.536386	0.2247	0.7065	no
15.2041	13.7562	-0.144384	-0.536865	0.2246	0.7065	no
3.13092	2.53634	-0.30384	-0.541001	0.22435	0.7065	no
5.68386	4.87625	-0.221099	-0.544598	0.22495	0.7065	no
12.119	13.4901	0.154621	0.537895	0.22465	0.7065	no
7.15207	5.90318	-0.276867	-0.538323	0.2248	0.7065	no
5.51575	4.93617	-0.160163	-0.535856	0.2243	0.7065	no
22.7984	20.5953	-0.146616	-0.544866	0.22495	0.7065	no
1.58813	1.17378	-0.436173	-0.532241	0.22385	0.7065	no
8.894	10.1816	0.195058	0.545222	0.2245	0.7065	no
30.7669	34.0235	0.145155	0.541732	0.22485	0.7065	no
44.1268	48.795	0.145079	0.536865	0.22465	0.7065	no
78.4513	86.9396	0.148216	0.542686	0.22465	0.7065	no
1.92469	1.48324	-0.375869	-0.548122	0.22515	0.70678	no
8.52936	7.55454	-0.175094	-0.535976	0.2254	0.707339	no
31.7723	35.1037	0.143853	0.535688	0.22545	0.707339	no
13.0856	14.7398	0.171737	0.535834	0.2258	0.708246	no
2.20788	1.72604	-0.355193	-0.545125	0.2259	0.708369	no
9.15981	8.22023	-0.156139	-0.533649	0.22645	0.709519	no
14.2816	12.2445	-0.222029	-0.534086	0.22645	0.709519	no
4.76969	4.13668	-0.205423	-0.543361	0.2266	0.709797	no
102.381	113.207	0.145016	0.53519	0.2267	0.709919	no
2.87927	3.38787	0.234673	0.532256	0.2274	0.710509	no

6.87259	6.17251	-0.154997	-0.545194	0.22735	0.710509	no
2.54884	3.31911	0.380955	0.536308	0.22725	0.710509	no
20.4378	18.471	-0.14598	-0.537698	0.2275	0.710509	no
6.30418	7.16267	0.184189	0.539561	0.22725	0.710509	no
14.5987	13.0653	-0.160102	-0.528244	0.2274	0.710509	no
23.1114	20.8124	-0.151156	-0.535653	0.22745	0.710509	no
2.21251	1.92358	-0.201887	-0.537679	0.2273	0.710509	no
7.51802	8.45178	0.168902	0.53004	0.22765	0.710596	no
23.5223	21.21	-0.149286	-0.539175	0.22765	0.710596	no
2.89688	2.43185	-0.25245	-0.530721	0.22795	0.711271	no
1.0027	1.50688	0.587678	0.546575	0.228	0.711271	no
31.9808	35.7084	0.159057	0.52904	0.22835	0.711996	no
18.076	20.5811	0.187243	0.536955	0.22865	0.711996	no
3.02328	3.70265	0.292446	0.530485	0.22855	0.711996	no
19.3407	21.6397	0.162045	0.540002	0.22845	0.711996	no
4.93235	4.31138	-0.194126	-0.534824	0.22855	0.711996	no
31.5672	34.9846	0.148296	0.532114	0.2286	0.711996	no
5.5584	4.56282	-0.284742	-0.526213	0.22875	0.712116	no
18.8464	20.8633	0.146675	0.535702	0.22905	0.712251	no
1.64275	1.30972	-0.326847	-0.526158	0.229	0.712251	no
1.26396	1.49234	0.239622	0.535877	0.2291	0.712251	no
3.71768	3.01201	-0.303677	-0.532674	0.2291	0.712251	no
15.7757	13.7819	-0.194931	-0.53737	0.22905	0.712251	no
1.75425	1.50196	-0.224013	-0.539495	0.2292	0.712372	no
49.5275	54.8383	0.146953	0.538285	0.22935	0.712647	no
1.9445	2.44638	0.331248	0.531514	0.2296	0.712852	no
17.3874	19.2212	0.144659	0.534868	0.2296	0.712852	no
3.33754	2.75175	-0.278433	-0.536656	0.2296	0.712852	no
21.403	23.8561	0.156544	0.527895	0.22995	0.713381	no
28.7885	32.0464	0.154673	0.527402	0.2302	0.713381	no
6.54504	5.59606	-0.225991	-0.536862	0.2301	0.713381	no
79.3046	87.6215	0.143879	0.535589	0.23005	0.713381	no
3.69609	3.26563	-0.178637	-0.536076	0.2302	0.713381	no
15.959	20.3126	0.348005	0.530219	0.22985	0.713381	no
7.04496	6.04831	-0.220059	-0.535974	0.2306	0.713443	no
8.47448	9.75788	0.203444	0.539081	0.2306	0.713443	no
1.47852	1.92055	0.377369	0.535691	0.2303	0.713443	no
35.3078	39.1794	0.15011	0.533441	0.23055	0.713443	no
8.66967	10.005	0.206673	0.544401	0.23065	0.713443	no
22.9633	20.7016	-0.149584	-0.528707	0.2306	0.713443	no
18.0789	19.9915	0.145079	0.526695	0.23125	0.714538	no
22.3507	24.9905	0.161063	0.53165	0.2312	0.714538	no
46.7107	51.8907	0.151724	0.539258	0.23115	0.714538	no
21.2874	23.5396	0.145093	0.526539	0.2312	0.714538	no
1.68463	1.32626	-0.345064	-0.531175	0.23135	0.714657	no
20.5167	22.7359	0.148174	0.531242	0.23145	0.714776	no
78.3424	86.8812	0.14925	0.531112	0.2316	0.714859	no

17.8942	21.0531	0.234541	0.540497	0.2316	0.714859	no
58.0873	64.0336	0.140606	0.527072	0.2321	0.715226	no
6.93073	8.22561	0.247116	0.531881	0.2319	0.715226	no
4.94942	5.54135	0.162978	0.533941	0.2319	0.715226	no
50.9598	56.3388	0.144769	0.531403	0.23215	0.715226	no
7.29665	6.55745	-0.154098	-0.526191	0.23205	0.715226	no
109.38	120.706	0.142143	0.533821	0.2321	0.715226	no
9.78314	10.8519	0.149577	0.530506	0.232	0.715226	no
1.53474	1.19578	-0.360043	-0.527964	0.23225	0.715345	no
14.0093	15.8963	0.182308	0.535073	0.23245	0.715546	no
30.6564	33.9123	0.145622	0.532155	0.2324	0.715546	no
5.33686	6.33522	0.247402	0.531013	0.2325	0.715546	no
3.95629	4.78188	0.273431	0.533664	0.2328	0.715604	no
6.1004	5.3105	-0.200056	-0.526818	0.23295	0.715604	no
12.914	14.4441	0.161538	0.525331	0.23285	0.715604	no
1.51927	1.90652	0.327569	0.539639	0.2329	0.715604	no
2.88205	3.51731	0.287374	0.530939	0.23275	0.715604	no
4.40624	3.88401	-0.182002	-0.526485	0.23265	0.715604	no
5.80565	6.70005	0.206715	0.534137	0.23315	0.71584	no
27.2789	30.0915	0.141571	0.528916	0.23325	0.715957	no
3.83139	3.37793	-0.18173	-0.531892	0.2334	0.716229	no
3.40772	2.46987	-0.464372	-0.520494	0.2335	0.716346	no
1.38872	1.7346	0.320841	0.531659	0.2336	0.716464	no
9.54583	10.7081	0.165756	0.529052	0.2337	0.716581	no
4.7367	5.66856	0.259101	0.527577	0.23385	0.716852	no
14.776	16.9323	0.196522	0.530342	0.23405	0.717276	no
20.7405	22.9138	0.143768	0.524463	0.23435	0.717708	no
28.357	31.2292	0.139188	0.514772	0.23445	0.717708	no
9.5256	8.61771	-0.144506	-0.533912	0.2345	0.717708	no
22.5846	19.4103	-0.218516	-0.530609	0.23445	0.717708	no
26.7793	29.5233	0.140736	0.52649	0.23475	0.718095	no
3.82558	3.21012	-0.253052	-0.518714	0.23475	0.718095	no
44.9767	49.7591	0.145782	0.533165	0.2349	0.718176	no
24.3416	26.8246	0.140135	0.524713	0.23485	0.718176	no
14.3415	12.9753	-0.144422	-0.538726	0.235	0.718293	no
16.2541	18.059	0.15192	0.527083	0.23525	0.718868	no
2.74937	2.36081	-0.219821	-0.541656	0.2354	0.718948	no
5.34327	6.34552	0.248014	0.519533	0.2354	0.718948	no
1.65341	1.19362	-0.470104	-0.530379	0.23565	0.719333	no
2.25549	2.79766	0.310777	0.536594	0.23575	0.719413	no
7.70795	6.60468	-0.222859	-0.527273	0.2358	0.719413	no
9.02315	8.05555	-0.163648	-0.521014	0.23595	0.719682	no
10.5671	11.7278	0.150361	0.527743	0.2362	0.720255	no
11.2427	10.0018	-0.168735	-0.524977	0.23715	0.720625	no
4.84965	4.01844	-0.271244	-0.521308	0.2371	0.720625	no
4.94794	5.70285	0.204856	0.520229	0.2373	0.720625	no
7.99479	6.72411	-0.249716	-0.527421	0.2372	0.720625	no

19.3696	16.6009	-0.222528	-0.528087	0.23735	0.720625	no
1.55959	1.29037	-0.273377	-0.530257	0.2368	0.720625	no
5.84295	5.01179	-0.22137	-0.52823	0.237	0.720625	no
9.90317	12.1097	0.290196	0.520492	0.2364	0.720625	no
113.56	125.777	0.147414	0.526226	0.23685	0.720625	no
31.7327	28.7891	-0.140449	-0.524225	0.2373	0.720625	no
5.06003	6.44079	0.348091	0.526884	0.2372	0.720625	no
2.15636	1.71285	-0.332198	-0.516427	0.2375	0.720625	no
767.064	861.91	0.168189	0.534676	0.23675	0.720625	no
55.7428	61.4957	0.141701	0.526919	0.237	0.720625	no
7.5306	6.74969	-0.157944	-0.525132	0.2375	0.720625	no
1.20707	1.79307	0.570924	0.529254	0.2367	0.720625	no
11.0973	12.2791	0.145998	0.525997	0.23735	0.720625	no
9.03703	10.2405	0.180369	0.522628	0.23745	0.720625	no
45.4068	41.2071	-0.140016	-0.524357	0.2371	0.720625	no
24.4791	22.2008	-0.140942	-0.522711	0.2376	0.720668	no
30.0519	33.6955	0.165099	0.522294	0.2377	0.720668	no
2.39004	2.01138	-0.248851	-0.522905	0.23765	0.720668	no
3.59864	2.94262	-0.290353	-0.529224	0.23795	0.721201	no
56.6846	51.4588	-0.139539	-0.520998	0.238	0.721201	no
8.67742	7.64692	-0.182388	-0.523056	0.2383	0.721357	no
13.223	11.9829	-0.142074	-0.523242	0.23825	0.721357	no
18.0901	16.2554	-0.154274	-0.525739	0.2382	0.721357	no
6.29835	7.22899	0.198819	0.522818	0.23815	0.721357	no
375.318	431.55	0.201413	0.523393	0.23855	0.72155	no
2360.93	3494.42	0.565696	0.515554	0.23855	0.72155	no
4.32289	5.22217	0.272653	0.50881	0.23845	0.72155	no
20.7235	22.918	0.145215	0.526319	0.23885	0.722269	no
10.65	9.39399	-0.181049	-0.526219	0.2395	0.723294	no
4.48989	5.20389	0.21291	0.522312	0.2393	0.723294	no
17.0839	19.2872	0.175011	0.522659	0.23945	0.723294	no
4.83051	4.19863	-0.202258	-0.534469	0.2395	0.723294	no
10.0154	11.2365	0.165975	0.526354	0.2395	0.723294	no
9.36276	11.9141	0.34767	0.522949	0.2397	0.72371	no
7.60312	6.26913	-0.278325	-0.512819	0.2402	0.72409	no
15.4959	17.158	0.147001	0.517871	0.24015	0.72409	no
39.3817	43.339	0.13814	0.519047	0.2402	0.72409	no
19.4739	22.6147	0.215718	0.528117	0.2401	0.72409	no
41.6519	45.872	0.139233	0.516963	0.24005	0.72409	no
15.9601	14.2207	-0.166471	-0.525934	0.2399	0.72409	no
17.9135	15.9617	-0.166433	-0.519701	0.2404	0.724505	no
21.322	24.0072	0.171124	0.520985	0.24055	0.724544	no
3.52634	4.14161	0.232018	0.518767	0.2406	0.724544	no
28.0911	32.9727	0.231161	0.524228	0.2405	0.724544	no
5.07833	5.8955	0.21526	0.521461	0.2409	0.725072	no
9.57203	10.6714	0.15685	0.515972	0.24085	0.725072	no
24.0092	26.9004	0.164042	0.526326	0.24105	0.725335	no

71.7695	79.2076	0.142269	0.517565	0.24135	0.725674	no
19.3187	21.3248	0.142534	0.515159	0.24135	0.725674	no
17.3458	15.739	-0.140237	-0.516462	0.2413	0.725674	no
10.1407	11.4783	0.178751	0.521569	0.2415	0.725749	no
6.27485	5.50518	-0.188791	-0.521246	0.24145	0.725749	no
16.1706	18.6195	0.203437	0.522695	0.2422	0.726162	no
14.751	13.3292	-0.146217	-0.517331	0.2417	0.726162	no
17.5712	19.3389	0.138298	0.517392	0.242	0.726162	no
35.8997	39.5685	0.14038	0.518361	0.2421	0.726162	no
2.6427	2.10095	-0.330974	-0.518372	0.2421	0.726162	no
41.5082	46.2885	0.157258	0.517563	0.2422	0.726162	no
6.49747	8.02254	0.30418	0.521304	0.242	0.726162	no
1.15841	1.48721	0.360467	0.523113	0.2421	0.726162	no
18.8172	20.7644	0.142066	0.521316	0.24215	0.726162	no
7.77985	9.46278	0.282521	0.514658	0.2424	0.726387	no
253.728	288.834	0.186956	0.523383	0.24235	0.726387	no
16.2713	18.3731	0.175266	0.520005	0.24255	0.726574	no
30.2435	33.7833	0.159684	0.515346	0.2426	0.726574	no
1.97584	1.65018	-0.259848	-0.516998	0.24265	0.726574	no
8.1624	9.19299	0.171541	0.52411	0.2429	0.72691	no
65.0067	72.4579	0.156553	0.525519	0.24295	0.72691	no
5.49116	6.40347	0.221744	0.526503	0.24295	0.72691	no
27.6412	24.9276	-0.149076	-0.49365	0.2433	0.727396	no
344.328	379.805	0.141477	0.515515	0.2433	0.727396	no
3.12004	2.69486	-0.211353	-0.512333	0.2433	0.727396	no
6.20535	7.23441	0.221364	0.525629	0.2435	0.727806	no
3.23639	4.72	0.544401	0.522567	0.24365	0.728067	no
37.2971	41.081	0.139408	0.518776	0.24495	0.728728	no
10.9495	9.8432	-0.153665	-0.521508	0.2447	0.728728	no
38.0023	41.9789	0.143576	0.512376	0.245	0.728728	no
6.87489	6.01731	-0.192218	-0.517054	0.2447	0.728728	no
48.6768	53.5441	0.137494	0.513517	0.245	0.728728	no
23.3663	25.7244	0.138714	0.509761	0.2447	0.728728	no
7.05149	8.09483	0.199073	0.522536	0.2449	0.728728	no
32.7114	36.3066	0.150435	0.521686	0.24425	0.728728	no
9.75494	8.57615	-0.185801	-0.519846	0.24445	0.728728	no
28.248	24.9773	-0.17753	-0.520602	0.2449	0.728728	no
10.7866	12.3975	0.200804	0.51733	0.2449	0.728728	no
9.28952	10.9286	0.234433	0.520512	0.2449	0.728728	no
2.31648	1.77212	-0.386457	-0.515266	0.2448	0.728728	no
4.14996	3.65788	-0.182092	-0.521243	0.2447	0.728728	no
26.9503	30.0781	0.15841	0.517292	0.2442	0.728728	no
3.70776	4.25425	0.198356	0.513971	0.2442	0.728728	no
8.56247	7.67009	-0.158783	-0.513392	0.244	0.728728	no
7.57363	6.68011	-0.181115	-0.514047	0.2452	0.728949	no
24.3389	27.4833	0.175293	0.513546	0.24515	0.728949	no
4.15909	5.11441	0.298299	0.510323	0.24545	0.729506	no

5.91039	5.23487	-0.175099	-0.522192	0.2458	0.730172	no
3.68038	4.47561	0.28223	0.517979	0.2459	0.730283	no
69.0502	76.0303	0.138927	0.519765	0.24615	0.730834	no
29.9944	27.2696	-0.137402	-0.513804	0.2463	0.730834	no
30.5964	34.9114	0.190335	0.519686	0.2464	0.730834	no
1.96894	3.09748	0.653674	0.512078	0.2463	0.730834	no
33.8485	37.2585	0.138476	0.517246	0.24635	0.730834	no
16.5372	18.8419	0.188229	0.51318	0.2468	0.731646	no
112.648	123.944	0.137873	0.504805	0.2468	0.731646	no
4.54174	3.83107	-0.245496	-0.504973	0.24755	0.732111	no
68.13	75.9369	0.156511	0.508679	0.24765	0.732111	no
3833.24	4603.23	0.264082	0.502283	0.24725	0.732111	no
11.9442	10.6507	-0.165366	-0.517912	0.24705	0.732111	no
11.278	12.7224	0.173861	0.513786	0.2474	0.732111	no
5.04013	4.51198	-0.1597	-0.510663	0.2473	0.732111	no
15.1858	13.7694	-0.141266	-0.516766	0.2472	0.732111	no
11.0327	12.8649	0.221659	0.518828	0.2474	0.732111	no
6.69853	5.86313	-0.192175	-0.515763	0.2472	0.732111	no
2.50149	1.97022	-0.344427	-0.523625	0.24805	0.732735	no
12.4913	11.3533	-0.137812	-0.506103	0.24805	0.732735	no
2.04048	1.61774	-0.33493	-0.512983	0.248	0.732735	no
14.5757	16.1713	0.14987	0.512195	0.2482	0.732805	no
5.81249	6.61004	0.185504	0.51635	0.24815	0.732805	no
81.5606	90.1121	0.143849	0.510397	0.2486	0.733388	no
6.00174	5.31464	-0.175409	-0.507561	0.2485	0.733388	no
1.17074	1.55927	0.413453	0.509188	0.24865	0.733388	no
4.81311	4.0245	-0.258161	-0.507316	0.2486	0.733388	no
5.04452	4.38216	-0.203075	-0.515384	0.2494	0.733923	no
37.9232	31.9189	-0.24867	-0.510879	0.2493	0.733923	no
6.07277	5.36601	-0.178504	-0.514789	0.24915	0.733923	no
15.5916	13.8103	-0.175023	-0.515725	0.24935	0.733923	no
7.39057	8.28316	0.164496	0.506641	0.2493	0.733923	no
35.049	38.5205	0.136255	0.511224	0.2492	0.733923	no
12.8731	11.2563	-0.193625	-0.518047	0.249	0.733923	no
17.2761	15.6501	-0.142604	-0.513578	0.24905	0.733923	no
11.9417	13.5627	0.183642	0.514963	0.24965	0.734473	no
8.43435	7.57656	-0.154734	-0.506288	0.2499	0.735022	no
2.96502	3.50687	0.242141	0.515919	0.2501	0.735238	no
1.4253	1.95182	0.45355	0.51645	0.2501	0.735238	no
9.17877	10.6851	0.219226	0.511729	0.2502	0.735307	no
1.54011	1.89205	0.296916	0.511429	0.25025	0.735307	no
94.6702	105.669	0.158571	0.511361	0.2508	0.735543	no
36.5027	33.0073	-0.145219	-0.512131	0.2506	0.735543	no
1.94346	1.62655	-0.256811	-0.503203	0.25065	0.735543	no
85.3395	94.8512	0.152452	0.506294	0.25085	0.735543	no
8.55583	9.60051	0.166204	0.507821	0.25075	0.735543	no
41.094	37.3916	-0.13621	-0.507916	0.2504	0.735543	no

12.1968	13.4636	0.142557	0.505793	0.25055	0.735543	no
7.60514	6.85277	-0.150287	-0.509033	0.25055	0.735543	no
9.59685	8.67324	-0.14599	-0.512954	0.2509	0.735543	no
3.85832	3.20175	-0.269112	-0.50974	0.25125	0.736383	no
37.6154	32.9359	-0.191661	-0.513161	0.25135	0.73649	no
3.52926	3.05407	-0.208631	-0.510808	0.25145	0.736598	no
26.3735	30.6513	0.216855	0.506903	0.25165	0.736626	no
42.6285	46.9294	0.138673	0.50953	0.25155	0.736626	no
44.9701	49.7111	0.144602	0.510414	0.25165	0.736626	no
36.9702	40.6663	0.137473	0.50934	0.2519	0.737172	no
7.69875	6.60522	-0.221019	-0.518751	0.252	0.737279	no
7.70481	6.89901	-0.15937	-0.505966	0.2524	0.737892	no
7.97816	7.03585	-0.18133	-0.505849	0.2523	0.737892	no
17.9991	20.0907	0.158598	0.510126	0.25235	0.737892	no
33.8344	30.7474	-0.138029	-0.511605	0.25255	0.738145	no
1.49453	1.2692	-0.235772	-0.506742	0.2538	0.738469	no
3.49833	4.35839	0.317129	0.510257	0.25405	0.738469	no
41.3087	45.5429	0.140779	0.505229	0.2535	0.738469	no
8.51162	9.78597	0.201282	0.511816	0.2533	0.738469	no
14.2971	16.1683	0.17744	0.50652	0.253	0.738469	no
9.7343	8.70921	-0.160535	-0.505164	0.25305	0.738469	no
13.7239	16.2985	0.248048	0.515084	0.25305	0.738469	no
2.34587	2.7755	0.242624	0.507814	0.254	0.738469	no
7.93944	9.13816	0.202867	0.507597	0.25385	0.738469	no
8.88095	11.136	0.326448	0.522178	0.2528	0.738469	no
80.6572	72.691	-0.150026	-0.511427	0.25425	0.738469	no
3.31765	3.92695	0.243247	0.504399	0.2533	0.738469	no
4.1563	4.96192	0.2556	0.504035	0.25385	0.738469	no
5.40626	6.72829	0.315607	0.509295	0.2533	0.738469	no
3.42078	2.70417	-0.339141	-0.510482	0.2537	0.738469	no
20.4351	22.4955	0.138592	0.509026	0.25285	0.738469	no
7.16006	6.50543	-0.138326	-0.507631	0.2541	0.738469	no
27.0032	29.7358	0.139074	0.504845	0.25325	0.738469	no
20.9033	18.6467	-0.16481	-0.508499	0.2542	0.738469	no
33.6916	37.0193	0.135891	0.513515	0.2537	0.738469	no
8.01313	9.03222	0.172715	0.508802	0.25425	0.738469	no
1.48025	1.14013	-0.376636	-0.512159	0.254	0.738469	no
15.1344	13.7814	-0.135106	-0.505684	0.2532	0.738469	no
15.7155	14.1261	-0.153826	-0.509418	0.25345	0.738469	no
38.0159	41.8185	0.137538	0.511024	0.25375	0.738469	no
34.1743	31.0542	-0.138123	-0.508091	0.2546	0.739064	no
4.91451	4.19404	-0.228707	-0.502899	0.25465	0.739064	no
22.5467	25.0086	0.149507	0.505425	0.25475	0.739064	no
37.8832	41.6528	0.136854	0.501473	0.25475	0.739064	no
6.17886	7.10609	0.201715	0.512749	0.25485	0.739064	no
84.292	92.8661	0.139756	0.511326	0.2548	0.739064	no
14.9923	13.4544	-0.156148	-0.502411	0.2549	0.739064	no

17.4338	19.3169	0.147978	0.504539	0.25525	0.739709	no
8.29798	9.34465	0.17138	0.506039	0.25525	0.739709	no
2.19106	1.68704	-0.377137	-0.504844	0.25565	0.740023	no
5.72057	6.73314	0.23512	0.499357	0.2562	0.740023	no
21.057	24.0394	0.191102	0.507383	0.25575	0.740023	no
28.9099	26.3064	-0.136154	-0.508304	0.2561	0.740023	no
14.2018	11.4569	-0.309859	-0.50148	0.256	0.740023	no
20.4319	18.6419	-0.132275	-0.494761	0.25625	0.740023	no
44.3177	48.7619	0.137872	0.511587	0.2556	0.740023	no
11.8604	13.3943	0.175465	0.51179	0.2562	0.740023	no
28.116	30.9757	0.139745	0.502837	0.256	0.740023	no
39.957	36.3745	-0.13552	-0.507307	0.2562	0.740023	no
7.75769	6.88468	-0.172236	-0.500603	0.2557	0.740023	no
1.70168	1.39516	-0.286528	-0.510656	0.2557	0.740023	no
3.06884	2.34879	-0.385778	-0.508402	0.25585	0.740023	no
41.5918	36.7697	-0.177783	-0.507601	0.2564	0.740272	no
2.10587	1.83924	-0.195308	-0.506671	0.25655	0.740521	no
76.4648	84.4163	0.142725	0.508699	0.2568	0.740611	no
264.582	298.159	0.17237	0.512005	0.2569	0.740611	no
13.8754	15.5291	0.162448	0.501271	0.25685	0.740611	no
21.3656	24.0573	0.171185	0.506935	0.25685	0.740611	no
16.0465	17.6347	0.136162	0.501258	0.25675	0.740611	no
6.58682	7.9502	0.271408	0.500281	0.25715	0.740964	no
4.45054	5.16689	0.215314	0.505325	0.25715	0.740964	no
9.35141	10.5129	0.168903	0.501437	0.25725	0.741069	no
16.3705	14.4389	-0.181132	-0.504743	0.25755	0.741382	no
68.9384	76.3077	0.146521	0.50079	0.25745	0.741382	no
1.84838	1.44858	-0.351623	-0.511785	0.2575	0.741382	no
3.29536	2.85142	-0.208753	-0.504119	0.25795	0.741535	no
37.2149	33.866	-0.13604	-0.494757	0.25775	0.741535	no
8.48024	9.932	0.227981	0.502346	0.258	0.741535	no
82.4974	91.3974	0.147805	0.500371	0.2578	0.741535	no
2.57171	2.13982	-0.265236	-0.491932	0.2579	0.741535	no
6.77268	5.80917	-0.221396	-0.506773	0.25805	0.741535	no
55.7365	50.5628	-0.140545	-0.498231	0.25785	0.741535	no
161.734	177.89	0.137364	0.502459	0.25815	0.741639	no
9.77115	11.0537	0.177929	0.50468	0.2584	0.74199	no
360.643	397.485	0.140329	0.499528	0.2584	0.74199	no
27.4456	31.0341	0.177277	0.502916	0.2586	0.742197	no
6.38844	5.54356	-0.204651	-0.499871	0.25855	0.742197	no
1.6994	0.947658	-0.842589	-0.533669	0.2587	0.742301	no
4.60553	5.1163	0.151733	0.498439	0.2589	0.742508	no
117.847	130.864	0.151155	0.504287	0.2589	0.742508	no
43.9422	39.8568	-0.140781	-0.505003	0.25905	0.742572	no
11.9836	10.8384	-0.14491	-0.503695	0.25905	0.742572	no
18.4379	16.742	-0.139206	-0.496958	0.2592	0.742635	no
6.32393	5.67418	-0.156409	-0.497426	0.25915	0.742635	no

1.70915	1.44618	-0.241031	-0.490137	0.2593	0.742739	no
7.60951	6.73066	-0.177056	-0.502283	0.2595	0.743128	no
23.0526	27.4725	0.253059	0.494751	0.2596	0.743231	no
9.21819	10.3519	0.167335	0.494878	0.26	0.744193	no
46.8939	51.6908	0.140509	0.506182	0.26015	0.744318	no
4.28916	3.8496	-0.155988	-0.496687	0.2603	0.744318	no
6.50304	5.00728	-0.377087	-0.484578	0.26025	0.744318	no
73.3428	64.3359	-0.189032	-0.507446	0.26025	0.744318	no
74.1005	81.8188	0.142948	0.494578	0.2605	0.744707	no
3.90141	4.95218	0.344068	0.516882	0.26095	0.744919	no
18.399	21.4493	0.221305	0.501473	0.2609	0.744919	no
3.18169	2.76597	-0.202007	-0.506773	0.26115	0.744919	no
3.82188	3.40064	-0.168477	-0.501332	0.26155	0.744919	no
6.71923	5.49532	-0.290092	-0.490512	0.2612	0.744919	no
79.0429	71.4363	-0.145979	-0.496573	0.2609	0.744919	no
22.1031	24.4221	0.143936	0.501427	0.2613	0.744919	no
15.8002	13.3911	-0.238671	-0.49956	0.26145	0.744919	no
20.9853	23.0676	0.136483	0.502391	0.26095	0.744919	no
11.8895	14.0889	0.244865	0.503058	0.2615	0.744919	no
27.5643	30.2737	0.135265	0.504633	0.2612	0.744919	no
1.30177	1.82862	0.490273	0.497623	0.2616	0.744919	no
5.5038	6.39103	0.215619	0.499954	0.26125	0.744919	no
21.1159	19.1842	-0.138413	-0.503924	0.2613	0.744919	no
39.6245	36.0861	-0.134949	-0.505235	0.26105	0.744919	no
45.6214	50.0743	0.134358	0.502399	0.2615	0.744919	no
7.80955	6.96058	-0.166032	-0.497922	0.2617	0.745021	no
13.3311	14.9462	0.164986	0.496592	0.2618	0.745123	no
87.1632	95.5549	0.13261	0.497502	0.26215	0.745936	no
33.7058	30.7457	-0.132615	-0.497738	0.2623	0.746181	no
4.37925	5.04855	0.205185	0.503686	0.26275	0.746364	no
4.0991	4.60859	0.169017	0.500924	0.2627	0.746364	no
10.8069	12.7546	0.239063	0.490881	0.2625	0.746364	no
12.2949	13.7855	0.165092	0.501828	0.2625	0.746364	no
70.5075	77.2821	0.132357	0.490955	0.26275	0.746364	no
4.39052	3.69459	-0.248977	-0.489764	0.26265	0.746364	no
37.3226	34.0141	-0.133918	-0.50018	0.26285	0.746425	no
14.4328	15.9602	0.145128	0.498665	0.2629	0.746425	no
14.5325	16.8976	0.217537	0.499564	0.2634	0.746488	no
27.664	25.232	-0.132753	-0.497488	0.26395	0.746488	no
43.8668	48.1138	0.133321	0.49736	0.26305	0.746488	no
8.41176	7.40644	-0.183627	-0.502451	0.2638	0.746488	no
5.64424	4.75782	-0.246478	-0.500386	0.26345	0.746488	no
9.35488	8.47207	-0.143004	-0.501056	0.26385	0.746488	no
3.05469	2.44001	-0.324141	-0.495604	0.26365	0.746488	no
4.36802	5.15983	0.240343	0.499935	0.26385	0.746488	no
179.833	163.978	-0.133151	-0.495208	0.26325	0.746488	no
27.5752	30.2042	0.131381	0.487761	0.26335	0.746488	no

12.3941	14.2666	0.202988	0.499411	0.26365	0.746488	no
25.8891	28.7686	0.152146	0.494564	0.2635	0.746488	no
14.0162	16.4552	0.231442	0.501496	0.2639	0.746488	no
16.7359	14.717	-0.185461	-0.495902	0.26395	0.746488	no
10.721	9.23971	-0.214522	-0.494983	0.2642	0.747013	no
14.2699	15.6821	0.136145	0.496868	0.2644	0.747396	no
3.56727	4.25276	0.253579	0.489502	0.2648	0.747799	no
3.26807	2.78015	-0.233276	-0.493515	0.26465	0.747799	no
2.17337	2.57889	0.246818	0.489403	0.2647	0.747799	no
31.4257	34.5804	0.138009	0.489283	0.265	0.748	no
25.9935	28.5307	0.134361	0.495636	0.265	0.748001	no
25.7807	28.4858	0.143947	0.492772	0.26515	0.748242	no
75.5212	82.8465	0.13356	0.492778	0.26525	0.748343	no
60.4302	66.2928	0.133583	0.488439	0.2655	0.748503	no
1.4195	1.08594	-0.386432	-0.489793	0.26545	0.748503	no
10.3851	8.93943	-0.216267	-0.495224	0.26545	0.748503	no
6.62288	7.31342	0.143086	0.484355	0.26575	0.748662	no
4.63809	3.98766	-0.217988	-0.500676	0.26575	0.748662	no
33.8106	37.0274	0.131115	0.49107	0.2657	0.748662	no
26.585	29.8469	0.166968	0.490571	0.26615	0.749607	no
18.7575	20.7884	0.148311	0.503183	0.26635	0.749989	no
56.0971	63.9386	0.188763	0.494543	0.2666	0.750511	no
9.29528	7.88756	-0.236921	-0.49441	0.26695	0.751009	no
10.0829	9.05428	-0.155243	-0.493299	0.2671	0.751009	no
1.78257	1.37546	-0.374045	-0.49442	0.26705	0.751009	no
1.75486	1.44283	-0.282455	-0.502411	0.26685	0.751009	no
6.25852	7.02765	0.167221	0.494956	0.2673	0.751251	no
30.3895	33.3782	0.135332	0.495177	0.26815	0.751251	no
1.66632	1.37979	-0.272213	-0.493475	0.26745	0.751251	no
119.215	132.102	0.148082	0.485624	0.26835	0.751251	no
25.965	28.7131	0.145141	0.495211	0.2676	0.751251	no
4.66764	4.10529	-0.185208	-0.493273	0.26815	0.751251	no
9.5109	8.50093	-0.161962	-0.492988	0.268	0.751251	no
2.79138	3.41111	0.28926	0.48642	0.2679	0.751251	no
3.35834	2.86167	-0.230892	-0.496244	0.26755	0.751251	no
17.0435	19.0305	0.159092	0.497617	0.26785	0.751251	no
6.91049	6.18202	-0.16071	-0.487262	0.2674	0.751251	no
86.873	78.9096	-0.138706	-0.492533	0.26735	0.751251	no
34.0522	31.0482	-0.133239	-0.500247	0.26805	0.751251	no
10.8565	9.88355	-0.135458	-0.491019	0.2683	0.751251	no
8.70543	7.59576	-0.196722	-0.488633	0.26795	0.751251	no
42.248	47.6131	0.172477	0.491005	0.2677	0.751251	no
20.4367	22.6217	0.146543	0.492454	0.2683	0.751251	no
6.6124	5.85235	-0.176157	-0.490803	0.26835	0.751251	no
21.8686	24.2276	0.147787	0.490641	0.2685	0.751309	no
60.1427	65.8807	0.131465	0.487856	0.2685	0.751309	no
10.6881	9.67265	-0.144025	-0.490179	0.2687	0.751687	no

13.9915	16.1021	0.202691	0.489658	0.26895	0.751843	no
11.6327	10.5902	-0.135447	-0.496851	0.26895	0.751843	no
46.1701	41.6222	-0.149607	-0.488943	0.2689	0.751843	no
17.2927	14.8959	-0.215254	-0.493052	0.2692	0.75218	no
22.3555	24.5332	0.134106	0.497831	0.26915	0.75218	no
2.07172	2.65962	0.36039	0.487296	0.2694	0.752377	no
1.25387	1.66048	0.405219	0.504991	0.2694	0.752377	no
24.344	26.7421	0.135549	0.49003	0.2695	0.752476	no
6.73428	8.07712	0.262317	0.484083	0.2698	0.75259	no
1.79695	2.15471	0.261943	0.479531	0.26965	0.75259	no
7.09428	7.94211	0.162866	0.494364	0.26975	0.75259	no
3.60865	4.00374	0.149889	0.482727	0.2697	0.75259	no
38.1386	34.4828	-0.145373	-0.495223	0.27025	0.753623	no
5.36887	4.79416	-0.163341	-0.490206	0.2703	0.753623	no
65.6295	59.5777	-0.139571	-0.491969	0.2708	0.754836	no
34.8261	31.7566	-0.133111	-0.491301	0.271	0.755031	no
2.21454	2.86288	0.370461	0.491265	0.271	0.755031	no
12.071	10.9991	-0.134158	-0.491091	0.2712	0.755407	no
23.091	21.0138	-0.135991	-0.493595	0.2715	0.755434	no
8.17748	7.22034	-0.17959	-0.481529	0.27155	0.755434	no
18.3129	20.0665	0.131935	0.487664	0.2716	0.755434	no
5.19569	4.6132	-0.171548	-0.486772	0.2716	0.755434	no
1.46372	1.03608	-0.498496	-0.478714	0.2715	0.755434	no
1.97808	2.33102	0.236856	0.489855	0.2716	0.755434	no
49.2267	44.8872	-0.133138	-0.487379	0.27175	0.75549	no
23.9568	26.6429	0.153313	0.483924	0.2717	0.75549	no
72.8899	80.2145	0.138144	0.482858	0.27185	0.755587	no
138.75	126.626	-0.131914	-0.4948	0.27195	0.755684	no
5.15159	4.5825	-0.168882	-0.490874	0.2726	0.756405	no
11.3779	10.3354	-0.138639	-0.490016	0.27255	0.756405	no
6.09615	5.09317	-0.259335	-0.480934	0.27245	0.756405	no
23.4912	21.3728	-0.136343	-0.489339	0.27235	0.756405	no
37.9031	34.5985	-0.131607	-0.488364	0.2726	0.756405	no
3.11952	2.68269	-0.217644	-0.490818	0.27275	0.75664	no
3.41791	2.80366	-0.285806	-0.461244	0.27295	0.756972	no
4.72922	4.16659	-0.182735	-0.488488	0.273	0.756972	no
16.2846	14.8746	-0.130653	-0.487317	0.27315	0.757027	no
21.8528	19.866	-0.137516	-0.49156	0.2731	0.757027	no
13.8763	12.1534	-0.191264	-0.485095	0.2733	0.757262	no
2.67566	2.25001	-0.249967	-0.486131	0.2735	0.757329	no
81.8405	89.5149	0.129312	0.481669	0.2734	0.757329	no
8.14903	7.43342	-0.132602	-0.489136	0.2736	0.757329	no
43.5843	47.9356	0.137288	0.494008	0.27365	0.757329	no
33.0737	36.7493	0.152035	0.484681	0.27355	0.757329	no
6.53651	7.21813	0.143104	0.482068	0.27455	0.757528	no
31.1152	28.1053	-0.146776	-0.4814	0.27405	0.757528	no
3.877	3.2118	-0.271561	-0.478907	0.2743	0.757528	no

6.01715	6.95383	0.208727	0.481859	0.2746	0.757528	no
4.10956	4.67307	0.185384	0.4866	0.2743	0.757528	no
29.488	32.6144	0.14538	0.487098	0.27385	0.757528	no
51.8599	57.1876	0.141084	0.489781	0.27465	0.757528	no
7.75739	9.10817	0.231589	0.487865	0.27465	0.757528	no
1.83572	2.30853	0.33063	0.484337	0.2747	0.757528	no
1.60541	1.35527	-0.244356	-0.478033	0.2744	0.757528	no
9.53069	8.69777	-0.131935	-0.485424	0.27465	0.757528	no
11.038	9.62374	-0.197815	-0.496733	0.27465	0.757528	no
13.0724	14.4506	0.144606	0.48086	0.27455	0.757528	no
6.97117	7.97361	0.193832	0.492747	0.27455	0.757528	no
6.6056	7.7737	0.234913	0.476502	0.274	0.757528	no
19.397	17.7354	-0.129203	-0.479179	0.27495	0.758037	no
4.70253	4.12501	-0.189039	-0.488946	0.2752	0.758367	no
23.4079	26.1953	0.162309	0.480351	0.2756	0.758887	no
10.9601	12.1368	0.147118	0.476013	0.27555	0.758887	no
34.0315	31.0831	-0.130742	-0.487328	0.2756	0.758887	no
27.7337	30.4791	0.136181	0.490096	0.27565	0.758887	no
8.6429	7.84824	-0.139147	-0.488457	0.27585	0.759078	no
23.6711	26.9394	0.186589	0.483936	0.27585	0.759078	no
16.7205	15.2071	-0.136877	-0.484465	0.27605	0.759448	no
1.86888	1.55857	-0.261949	-0.486114	0.2764	0.760231	no
9.24963	10.2731	0.151398	0.480933	0.27655	0.760463	no
299.613	333.419	0.154236	0.475099	0.2767	0.760696	no
6.5834	7.35209	0.15932	0.480007	0.2769	0.761066	no
12.6654	14.0227	0.146876	0.479711	0.27705	0.761118	no
14.5138	13.288	-0.127303	-0.474973	0.27705	0.761118	no
29.2743	32.0004	0.128455	0.479762	0.2772	0.76135	no
14.5089	13.0091	-0.157407	-0.481162	0.27735	0.761582	no
3.98365	4.59938	0.207347	0.479183	0.2776	0.761728	no
4.19328	3.64711	-0.201328	-0.486951	0.2775	0.761728	no
11.9964	10.801	-0.151445	-0.484812	0.2776	0.761728	no
10.1083	9.00139	-0.16732	-0.487476	0.2778	0.762097	no
17.1981	18.9997	0.143725	0.476103	0.2779	0.762191	no
23.9567	26.5232	0.146824	0.474266	0.278	0.762286	no
12.6685	14.0203	0.146274	0.472613	0.2782	0.762654	no
269.415	294.596	0.12891	0.483828	0.27845	0.763116	no
5.47715	6.31068	0.20437	0.480389	0.2785	0.763116	no
10.3431	11.8793	0.199778	0.486987	0.27895	0.763629	no
290.611	318.262	0.131126	0.479138	0.27895	0.763629	no
15.0209	13.3868	-0.16616	-0.484151	0.27895	0.763629	no
26.8121	29.3272	0.129357	0.477966	0.2788	0.763629	no
2.26061	1.67754	-0.430362	-0.483212	0.27905	0.763723	no
1.47302	1.1807	-0.319128	-0.483681	0.2795	0.764774	no
18.2706	16.457	-0.150829	-0.481491	0.28015	0.765651	no
66.9245	73.5621	0.136427	0.4857	0.27995	0.765651	no
6.6497	7.73575	0.218253	0.47931	0.28015	0.765651	no

20.6618	18.8836	-0.129835	-0.484338	0.28005	0.765651	no
3.34639	3.85418	0.203818	0.485344	0.28	0.765651	no
3.19987	3.831	0.25971	0.478725	0.2803	0.765881	no
28.4293	31.0706	0.128173	0.478957	0.28045	0.76593	no
1.38203	1.74511	0.336534	0.4834	0.2804	0.76593	no
11.4225	10.0775	-0.180735	-0.478721	0.28055	0.766023	no
5.04738	4.2258	-0.256311	-0.482809	0.28065	0.766116	no
1.48008	2.09535	0.50151	0.470205	0.28075	0.766209	no
28.4839	31.1135	0.127394	0.472527	0.28095	0.766215	no
39.7793	36.361	-0.129625	-0.479918	0.28095	0.766215	no
27.8686	30.505	0.130406	0.47895	0.2809	0.766215	no
17.7684	16.2416	-0.129625	-0.482119	0.28115	0.766581	no
4.3081	5.02711	0.222676	0.488693	0.28125	0.766674	no
10.5988	11.6529	0.136793	0.481976	0.2815	0.767088	no
22.1045	24.5207	0.149661	0.481739	0.28155	0.767088	no
30.016	27.4816	-0.127266	-0.472762	0.2816	0.767088	no
53.5249	58.6265	0.131343	0.482977	0.28175	0.767137	no
6.10768	5.50122	-0.150871	-0.466651	0.2817	0.767137	no
2.52909	2.16693	-0.222963	-0.477608	0.2819	0.767365	no
9.61743	10.6454	0.146509	0.469085	0.2822	0.767822	no
16.0893	14.0514	-0.195386	-0.480698	0.28215	0.767822	no
1.71046	2.30954	0.433218	0.469601	0.2823	0.767914	no
14.0902	12.8008	-0.138468	-0.466377	0.28255	0.768011	no
1.75176	2.05952	0.233507	0.473181	0.2826	0.768011	no
2.72323	3.22456	0.243782	0.478156	0.28245	0.768011	no
12.8433	11.6946	-0.135167	-0.474983	0.2826	0.768011	no
36.4855	39.8791	0.128311	0.480364	0.2827	0.768103	no
67.3613	76.0445	0.174925	0.472936	0.283	0.768379	no
16.9971	19.6476	0.209059	0.479446	0.283	0.768379	no
2.67723	3.14521	0.232415	0.467535	0.283	0.768379	no
8.14505	6.98615	-0.221427	-0.477662	0.2832	0.768742	no
2.35604	1.7935	-0.393586	-0.442806	0.28345	0.768794	no
11.7182	10.6434	-0.13879	-0.472664	0.2835	0.768794	no
1.54684	1.01392	-0.609378	-0.50324	0.2833	0.768794	no
3.44901	3.92591	0.186844	0.479668	0.2834	0.768794	no
4.18038	3.72013	-0.168281	-0.475277	0.28355	0.768794	no
42.5059	38.905	-0.127705	-0.477806	0.2837	0.768842	no
3.78142	4.45449	0.236332	0.473215	0.2837	0.768842	no
10.7918	9.86837	-0.129057	-0.4813	0.2846	0.768862	no
50.613	46.3264	-0.127672	-0.477403	0.28405	0.768862	no
86.4572	94.952	0.135212	0.475092	0.28445	0.768862	no
58.39	53.152	-0.135599	-0.474692	0.2846	0.768862	no
40.9589	36.9952	-0.146839	-0.481333	0.28435	0.768862	no
18.7298	21.7719	0.217132	0.480434	0.2846	0.768862	no
6.15013	7.0853	0.204212	0.474364	0.2841	0.768862	no
23.7979	21.6803	-0.134453	-0.483217	0.2847	0.768862	no
334.354	365.626	0.12899	0.469417	0.28455	0.768862	no

12.282	10.6665	-0.203459	-0.474622	0.2847	0.768862	no
4.19309	3.64687	-0.201355	-0.476373	0.2843	0.768862	no
51.5391	58.2308	0.176113	0.476541	0.28455	0.768862	no
4.41256	5.13937	0.219976	0.474418	0.2838	0.768862	no
22.5431	24.669	0.13001	0.474026	0.28435	0.768862	no
17.2819	15.7656	-0.132479	-0.470421	0.2846	0.768862	no
4.53059	5.32035	0.231824	0.475576	0.2849	0.769001	no
1.35389	1.6857	0.316236	0.476305	0.2849	0.769001	no
15.4873	16.9699	0.131894	0.472842	0.28495	0.769001	no
94.8606	104.072	0.133696	0.477585	0.28505	0.769048	no
142.808	156.416	0.131315	0.480738	0.2851	0.769048	no
22.9843	20.9014	-0.137049	-0.475756	0.2852	0.76914	no
6.08025	5.18724	-0.229162	-0.474671	0.2853	0.769231	no
7.8852	9.25228	0.230662	0.47469	0.2854	0.769278	no
4.79468	4.00597	-0.259281	-0.467311	0.28545	0.769278	no
5.64589	4.99312	-0.177259	-0.478804	0.2856	0.769504	no
5.75116	6.39814	0.1538	0.469402	0.28585	0.76982	no
15.6653	14.2645	-0.135143	-0.47636	0.2858	0.76982	no
50.1187	45.6411	-0.135016	-0.480898	0.2861	0.769871	no
25.7892	23.4211	-0.138963	-0.472868	0.28615	0.769871	no
20.8005	18.8512	-0.141959	-0.479459	0.286	0.769871	no
44.6709	48.8187	0.128099	0.478115	0.28605	0.769871	no
12.7408	14.0859	0.144798	0.479547	0.2865	0.770143	no
6.88248	8.10266	0.235467	0.46645	0.2864	0.770143	no
3.78576	3.15116	-0.264699	-0.47512	0.28645	0.770143	no
1.20712	1.54759	0.358459	0.471718	0.2868	0.770193	no
2.89808	3.35292	0.21032	0.467423	0.28665	0.770193	no
12.4866	11.3956	-0.131914	-0.475004	0.28675	0.770193	no
29.6264	32.4219	0.130086	0.472567	0.28675	0.770193	no
8.92348	8.12108	-0.135934	-0.469619	0.28705	0.770552	no
2.65839	2.13545	-0.31601	-0.474922	0.28745	0.771359	no
8.25009	9.4802	0.200508	0.474863	0.28755	0.771359	no
2.72804	2.33284	-0.22578	-0.4719	0.28755	0.771359	no
31.8617	29.2009	-0.12581	-0.467316	0.2926	0.771852	no
1.44722	1.83022	0.338734	0.470932	0.2919	0.771852	no
4.25414	5.06505	0.251707	0.463997	0.2901	0.771852	no
4.84485	5.37473	0.14974	0.465697	0.2894	0.771852	no
2.93301	3.53086	0.267637	0.46583	0.2899	0.771852	no
6.48105	5.21106	-0.314652	-0.463149	0.2906	0.771852	no
30.3122	33.0918	0.126577	0.471675	0.2881	0.771852	no
9.86297	8.80031	-0.164467	-0.469353	0.289	0.771852	no
12.0271	10.9021	-0.141683	-0.463657	0.29205	0.771852	no
1.2243	2.16164	0.820171	0.489119	0.29145	0.771852	no
2.71212	2.08502	-0.379361	-0.450204	0.29105	0.771852	no
17.0722	18.7194	0.132888	0.472905	0.28975	0.771852	no
6.06121	5.48692	-0.14361	-0.468917	0.2924	0.771852	no
1.50989	1.24254	-0.281144	-0.466465	0.2904	0.771852	no

20.2617	22.2922	0.137783	0.465217	0.29245	0.771852	no
11.5089	12.8581	0.159923	0.470914	0.29135	0.771852	no
10.7468	9.74771	-0.140768	-0.473139	0.2918	0.771852	no
1.97629	1.71823	-0.201874	-0.467429	0.29245	0.771852	no
5.09856	5.75745	0.175339	0.473387	0.2923	0.771852	no
5.4916	4.10644	-0.419338	-0.476275	0.2898	0.771852	no
8.2064	9.1145	0.151414	0.473052	0.29155	0.771852	no
4.44567	5.34183	0.264932	0.463663	0.28915	0.771852	no
57.0761	51.9912	-0.134618	-0.464385	0.29115	0.771852	no
6.77315	7.46123	0.139587	0.460382	0.29005	0.771852	no
10.8033	9.83489	-0.135496	-0.474107	0.2907	0.771852	no
2.26404	1.99027	-0.185934	-0.474975	0.29265	0.771852	no
35.6965	38.9374	0.125372	0.46906	0.28985	0.771852	no
49.0124	53.7169	0.132231	0.47117	0.29025	0.771852	no
44.0376	48.2471	0.131704	0.4744	0.2914	0.771852	no
4.07732	3.45399	-0.239356	-0.468504	0.2899	0.771852	no
8.11322	9.52765	0.231846	0.459813	0.29095	0.771852	no
69.894	63.6583	-0.134821	-0.473151	0.2887	0.771852	no
10.5617	11.8237	0.162842	0.471869	0.2905	0.771852	no
23.2662	21.2849	-0.128406	-0.475239	0.288	0.771852	no
44.1823	40.4266	-0.128166	-0.470508	0.29195	0.771852	no
4.69835	5.57539	0.246919	0.471876	0.29045	0.771852	no
5.97567	4.9087	-0.28376	-0.46852	0.291	0.771852	no
2.70749	2.32208	-0.221536	-0.459637	0.29255	0.771852	no
7.44924	8.58054	0.203975	0.470577	0.2908	0.771852	no
17.3927	15.8992	-0.129528	-0.471847	0.2879	0.771852	no
17.0806	15.6177	-0.129177	-0.468824	0.2902	0.771852	no
17.794	19.802	0.154254	0.465827	0.2926	0.771852	no
23.8705	26.9779	0.176546	0.474593	0.289	0.771852	no
14.4929	11.9656	-0.276447	-0.466079	0.2894	0.771852	no
47.3703	43.4148	-0.125796	-0.467751	0.29035	0.771852	no
17.0437	15.25	-0.160434	-0.469277	0.2904	0.771852	no
24.9487	27.2564	0.127631	0.472495	0.29045	0.771852	no
1.62487	2.16262	0.412459	0.473065	0.2882	0.771852	no
3.87567	3.28661	-0.237844	-0.472371	0.29065	0.771852	no
17.9333	19.9424	0.153194	0.463195	0.29175	0.771852	no
231.349	252.522	0.126338	0.464607	0.29235	0.771852	no
119.384	109.311	-0.12717	-0.468859	0.29215	0.771852	no
31.0294	34.0497	0.134006	0.475911	0.2881	0.771852	no
2.51088	2.12822	-0.238549	-0.461313	0.2919	0.771852	no
259.512	286.085	0.140641	0.472748	0.28825	0.771852	no
4.61219	5.1024	0.145726	0.464971	0.2926	0.771852	no
35.8685	39.3423	0.133365	0.473419	0.292	0.771852	no
5.45589	4.77589	-0.192044	-0.475269	0.28835	0.771852	no
1.88615	1.59693	-0.240141	-0.459016	0.2926	0.771852	no
1.83318	2.15165	0.231088	0.470071	0.29065	0.771852	no
26.658	24.4315	-0.125829	-0.472331	0.2885	0.771852	no

15.2118	13.8521	-0.135095	-0.46961	0.2918	0.771852	no
5.62065	5.0464	-0.155483	-0.467859	0.2893	0.771852	no
9.27294	7.97229	-0.218033	-0.466005	0.288	0.771852	no
20.7635	22.874	0.139663	0.46749	0.2921	0.771852	no
1.73622	2.31641	0.415944	0.467124	0.29225	0.771852	no
28.4053	31.4439	0.146622	0.476489	0.29065	0.771852	no
20.7535	23.2055	0.16111	0.472483	0.2884	0.771852	no
4.20024	3.70401	-0.181386	-0.468398	0.29255	0.771852	no
5.72043	4.96119	-0.205438	-0.468563	0.2883	0.771852	no
1.15469	1.62385	0.491913	0.461566	0.29275	0.77194	no
2.57942	2.12531	-0.279371	-0.464661	0.29285	0.772029	no
1.00164	1.56691	0.645549	0.472601	0.293	0.772074	no
29.145	32.0016	0.134896	0.461922	0.293	0.772074	no
1.86822	1.58582	-0.236443	-0.464552	0.29315	0.772294	no
101.162	110.287	0.124601	0.462086	0.29325	0.772382	no
18.1542	19.8673	0.13009	0.469165	0.29345	0.772734	no
28.6459	31.5807	0.140718	0.464198	0.29385	0.772781	no
21.7509	24.0478	0.144825	0.468914	0.29375	0.772781	no
41.0951	37.5875	-0.128713	-0.466054	0.2939	0.772781	no
18.2222	20.5218	0.171453	0.470811	0.2936	0.772781	no
14.0476	15.3181	0.124913	0.461316	0.29395	0.772781	no
5.04731	4.51961	-0.159319	-0.465891	0.2938	0.772781	no
74.8567	68.4273	-0.12956	-0.468506	0.294	0.772781	no
12.5304	11.364	-0.140963	-0.457586	0.29395	0.772781	no
12.1898	13.6315	0.161262	0.468886	0.2941	0.772869	no
14.9661	13.688	-0.128781	-0.467855	0.2943	0.773045	no
10.5078	12.0792	0.201069	0.46671	0.2943	0.773045	no
5.49427	6.50446	0.243502	0.466143	0.29445	0.773224	no
19.2339	16.7629	-0.198378	-0.473062	0.2953	0.773224	no
6.4053	5.82081	-0.138047	-0.460286	0.2952	0.773224	no
6.03198	7.3648	0.288013	0.462031	0.2948	0.773224	no
5.85374	6.80727	0.217719	0.47378	0.2953	0.773224	no
41.8247	47.0885	0.171018	0.467603	0.2946	0.773224	no
3.95849	4.54098	0.198053	0.469496	0.2951	0.773224	no
13.5284	12.2487	-0.143368	-0.464568	0.29495	0.773224	no
5.10802	4.47365	-0.191311	-0.467179	0.29465	0.773224	no
28.2708	30.9293	0.129661	0.467776	0.295	0.773224	no
4.12808	3.65575	-0.175304	-0.468215	0.295	0.773224	no
27.9486	25.3341	-0.141693	-0.462956	0.29455	0.773224	no
4.66585	3.88955	-0.262537	-0.466299	0.2948	0.773224	no
7.38111	6.69527	-0.140695	-0.463785	0.2955	0.773399	no
7.32047	8.16953	0.158317	0.457386	0.2955	0.773399	no
3.08346	2.561	-0.267841	-0.466708	0.2956	0.773487	no
5.06787	4.43349	-0.192937	-0.473084	0.2958	0.773836	no
3.32329	2.28877	-0.53804	-0.487515	0.2959	0.77388	no
1.94885	2.24006	0.200913	0.466168	0.29595	0.77388	no
2.47121	2.1625	-0.192513	-0.465474	0.29615	0.774228	no

12.3081	13.5847	0.142383	0.460342	0.2966	0.774272	no
6.89682	7.78378	0.174541	0.462463	0.29665	0.774272	no
7.9639	9.23818	0.214134	0.469903	0.29645	0.774272	no
42.102	47.2543	0.166555	0.464595	0.29645	0.774272	no
13.7821	12.3149	-0.162385	-0.457906	0.29645	0.774272	no
12.3544	11.2203	-0.138905	-0.462026	0.2967	0.774272	no
23.957	26.12	0.124708	0.46503	0.2965	0.774272	no
1.55872	1.27234	-0.292876	-0.468638	0.2964	0.774272	no
11.6929	10.7236	-0.124844	-0.465882	0.29795	0.77449	no
5.3011	6.12786	0.209091	0.46558	0.29755	0.77449	no
7.10137	6.08292	-0.223335	-0.465506	0.2978	0.77449	no
7.74778	6.7846	-0.191519	-0.470868	0.2981	0.77449	no
14.8392	12.5917	-0.236945	-0.464237	0.2975	0.77449	no
15.4666	12.8032	-0.27265	-0.464749	0.29745	0.77449	no
26.4849	29.6552	0.163119	0.459961	0.2975	0.77449	no
9.52262	8.63057	-0.141902	-0.456301	0.29735	0.77449	no
8.34385	9.15389	0.133672	0.464335	0.29785	0.77449	no
1.38985	1.81955	0.388651	0.461055	0.2973	0.77449	no
69.8891	76.3041	0.126694	0.463497	0.2974	0.77449	no
57.5994	52.7973	-0.125591	-0.464971	0.2982	0.77449	no
50.4374	54.9565	0.123798	0.457708	0.29815	0.77449	no
2.4714	2.12981	-0.214602	-0.463702	0.29745	0.77449	no
12.9822	11.7916	-0.138776	-0.453401	0.2974	0.77449	no
8.54043	7.6835	-0.152546	-0.464027	0.29815	0.77449	no
16.3977	18.4966	0.173768	0.46368	0.29755	0.77449	no
26.2072	28.6608	0.129115	0.456056	0.2977	0.77449	no
134.482	150.903	0.166201	0.463639	0.29825	0.77449	no
14.7294	13.3114	-0.146035	-0.45844	0.29805	0.77449	no
28.3036	34.5041	0.285783	0.464637	0.29825	0.77449	no
4.3253	3.76924	-0.198524	-0.462841	0.29815	0.77449	no
42.8822	46.6644	0.121943	0.457181	0.29835	0.774577	no
17.1589	15.7412	-0.124411	-0.458622	0.2985	0.774793	no
41.3832	38.0158	-0.122447	-0.457721	0.2986	0.774879	no
14.3301	13.0935	-0.130194	-0.459039	0.2987	0.774966	no
12.4276	11.3359	-0.132654	-0.457658	0.299	0.775571	no
1.50678	1.82874	0.279379	0.457407	0.29915	0.775787	no
27.9907	30.6231	0.129671	0.461103	0.2997	0.77613	no
1.17218	1.70125	0.537404	0.447943	0.2996	0.77613	no
3.56523	3.02519	-0.236969	-0.460228	0.2995	0.77613	no
113.981	124.69	0.129551	0.458224	0.29965	0.77613	no
2.07903	2.83572	0.447807	0.470087	0.29945	0.77613	no
5.11109	5.93463	0.215526	0.459706	0.29935	0.77613	no
6.36279	5.68205	-0.163248	-0.462161	0.2999	0.776345	no
27.0221	24.7077	-0.129176	-0.466372	0.3002	0.776559	no
65.6064	59.9571	-0.129905	-0.461609	0.3002	0.776559	no
1.76227	2.1179	0.265202	0.462455	0.30005	0.776559	no
9.88605	11.1436	0.172745	0.464969	0.30025	0.776559	no

5.24212	6.02708	0.201308	0.460753	0.30045	0.776731	no
6.32313	7.26846	0.201011	0.464117	0.3004	0.776731	no
15.9205	14.4234	-0.142471	-0.458934	0.30065	0.777075	no
13.359	11.7223	-0.188556	-0.462962	0.301	0.77759	no
4.40687	5.1891	0.235731	0.460903	0.30105	0.77759	no
32.4875	37.4338	0.204457	0.462755	0.30105	0.77759	no
10.2607	9.2865	-0.143919	-0.455035	0.30125	0.777933	no
4.43576	3.75663	-0.239742	-0.458262	0.3021	0.777966	no
5.70225	6.76591	0.246753	0.462813	0.30145	0.777966	no
23.3557	25.4181	0.122085	0.458085	0.3015	0.777966	no
20.65	22.5522	0.127128	0.45081	0.30195	0.777966	no
1.24093	1.44166	0.216312	0.45284	0.30215	0.777966	no
17.9981	16.0563	-0.16471	-0.458931	0.30205	0.777966	no
22.5506	24.8163	0.138127	0.455	0.3018	0.777966	no
110.229	120.679	0.130666	0.458893	0.3019	0.777966	no
30.794	28.289	-0.122406	-0.457	0.3022	0.777966	no
6.626	5.95497	-0.154045	-0.461859	0.3019	0.777966	no
41.9789	45.6834	0.122004	0.457343	0.30165	0.777966	no
12.1587	11.0738	-0.134833	-0.455364	0.30175	0.777966	no
17.6948	19.2779	0.123623	0.453661	0.30215	0.777966	no
1.12423	1.4274	0.344462	0.457226	0.30195	0.777966	no
11.3899	12.8495	0.173959	0.460102	0.3023	0.778051	no
1575.72	1360.7	-0.211663	-0.44427	0.30265	0.778779	no
57.4741	52.8193	-0.121847	-0.455972	0.30275	0.778864	no
8.50725	7.68483	-0.146678	-0.458239	0.30295	0.779206	no
9.6501	10.5501	0.12864	0.460411	0.30315	0.779548	no
153.244	166.723	0.121622	0.4483	0.30335	0.779889	no
37.3665	42.4873	0.185289	0.463983	0.30345	0.779974	no
21.5848	23.9581	0.150496	0.455103	0.30375	0.780227	no
3.00291	3.68287	0.294467	0.454447	0.3037	0.780227	no
8.93079	10.0999	0.177479	0.458586	0.3037	0.780227	no
31.5608	28.6832	-0.137927	-0.455598	0.3042	0.78121	no
2.11419	1.68393	-0.328274	-0.458978	0.3049	0.781277	no
13.226	14.5773	0.140348	0.46252	0.3048	0.781277	no
27.1431	29.7392	0.131782	0.453066	0.30465	0.781277	no
6.11968	5.12806	-0.255045	-0.448424	0.3051	0.781277	no
24.0357	26.3628	0.133322	0.447686	0.30445	0.781277	no
36.1659	39.6424	0.132414	0.45754	0.30465	0.781277	no
15.1991	13.8867	-0.130278	-0.457619	0.30435	0.781277	no
31.8414	29.187	-0.125579	-0.454178	0.30485	0.781277	no
3.99823	4.53622	0.182129	0.457961	0.3051	0.781277	no
45.8775	40.3808	-0.184117	-0.455636	0.3051	0.781277	no
5.6493	5.13787	-0.1369	-0.459456	0.30505	0.781277	no
5.1258	4.15165	-0.304091	-0.450529	0.30465	0.781277	no
145.181	158.418	0.125881	0.462156	0.3054	0.781484	no
159.722	173.925	0.122905	0.45466	0.30545	0.781484	no
3.00964	2.627	-0.196176	-0.459482	0.3054	0.781484	no

239.721	220.265	-0.122119	-0.4547	0.30565	0.781823	no
28.1589	25.8319	-0.124437	-0.454846	0.30575	0.781863	no
6.09709	5.2695	-0.210454	-0.457263	0.3058	0.781863	no
11.1801	10.2581	-0.124179	-0.461932	0.306	0.782202	no
8.98543	10.7041	0.252499	0.456079	0.30625	0.78228	no
1.97905	1.72089	-0.201656	-0.454212	0.3063	0.78228	no
3.28231	2.7442	-0.258329	-0.458461	0.3061	0.78228	no
5.75339	5.00601	-0.200751	-0.45276	0.3063	0.78228	no
5.24492	6.19274	0.239657	0.454729	0.3064	0.782319	no
27.6205	25.3754	-0.122312	-0.456643	0.30645	0.78232	no
17.9821	20.3542	0.178772	0.453468	0.3067	0.782741	no
5.24066	4.50551	-0.218059	-0.453617	0.30675	0.782741	no
15.2989	13.8912	-0.139256	-0.453547	0.30685	0.782825	no
24.2593	26.7171	0.139224	0.448928	0.30715	0.783113	no
3.88664	3.30181	-0.235265	-0.441461	0.30715	0.783113	no
7.39261	6.59085	-0.165619	-0.454706	0.3073	0.783113	no
1.41352	2.16421	0.614551	0.464092	0.30725	0.783113	no
211.305	230.06	0.122686	0.445123	0.30765	0.783788	no
32.258	35.154	0.124029	0.460677	0.3077	0.783788	no
4.34945	3.70911	-0.229759	-0.439845	0.3078	0.783871	no
27.1485	29.5211	0.120873	0.449023	0.3081	0.783948	no
14.2248	13.0676	-0.122419	-0.448267	0.308	0.783948	no
41.05	44.7075	0.123136	0.456099	0.3081	0.783948	no
9.81807	11.5131	0.229763	0.462275	0.3081	0.783948	no
1.08169	1.84368	0.769299	0.569708	0.3083	0.784285	no
1.46939	1.78942	0.284278	0.456124	0.3085	0.784622	no
15.7429	14.0638	-0.162723	-0.451876	0.3092	0.784798	no
2.39124	2.10419	-0.184496	-0.453264	0.3095	0.784798	no
16.224	17.7373	0.128658	0.454558	0.30965	0.784798	no
120.437	109.765	-0.133859	-0.457543	0.30895	0.784798	no
112.436	122.658	0.125527	0.446773	0.30905	0.784798	no
6.04307	6.78647	0.16738	0.454375	0.3096	0.784798	no
41.4403	45.0544	0.120633	0.444972	0.3096	0.784798	no
47.4651	52.2229	0.137814	0.454617	0.30895	0.784798	no
17.9865	19.6265	0.125885	0.453317	0.3094	0.784798	no
2.25988	1.94963	-0.213045	-0.453394	0.3089	0.784798	no
3.36358	4.03273	0.261761	0.448397	0.3093	0.784798	no
2.0495	2.50079	0.287107	0.45579	0.3089	0.784798	no
46.7075	50.8716	0.123208	0.44242	0.3098	0.784836	no
6.01036	6.63047	0.141661	0.44378	0.3098	0.784836	no
5.08593	4.38676	-0.213355	-0.452368	0.31005	0.785298	no
10.6283	9.57586	-0.150441	-0.449024	0.31045	0.785574	no
14.2875	13.0864	-0.126692	-0.456608	0.3107	0.785574	no
5.64313	5.17088	-0.126086	-0.452915	0.3105	0.785574	no
16.9697	15.4719	-0.133313	-0.448733	0.31065	0.785574	no
7.13104	6.21394	-0.198605	-0.453949	0.31035	0.785574	no
12.5022	13.7699	0.139341	0.448045	0.3106	0.785574	no

3.63402	3.02613	-0.264092	-0.452076	0.311	0.786161	no
4.77303	5.63106	0.238502	0.452563	0.3112	0.786265	no
2.8207	2.35425	-0.260787	-0.433248	0.3116	0.786265	no
9.57107	8.28179	-0.208739	-0.451545	0.31165	0.786265	no
2.63619	2.27131	-0.214929	-0.44726	0.3116	0.786265	no
10.2025	11.2333	0.138865	0.440785	0.31155	0.786265	no
14.0173	15.6529	0.15922	0.453763	0.3115	0.786265	no
15.0498	13.8312	-0.121826	-0.45287	0.3116	0.786265	no
2.90446	3.87233	0.414931	0.456666	0.31165	0.786265	no
18.1181	19.7472	0.124219	0.444316	0.3114	0.786265	no
5.3958	4.71141	-0.195678	-0.445761	0.31175	0.786346	no
7.48311	6.72791	-0.153478	-0.450833	0.31205	0.786932	no
148.345	162.219	0.128982	0.444242	0.3122	0.786969	no
5.58725	6.20095	0.150352	0.449775	0.31215	0.786969	no
2.66321	2.13931	-0.316017	-0.458955	0.31245	0.787087	no
33.2045	36.1505	0.122635	0.450225	0.31245	0.787087	no
354.5	384.904	0.118712	0.446002	0.31235	0.787087	no
22.9353	25.1829	0.134876	0.445	0.3135	0.787423	no
20.6484	18.9693	-0.122365	-0.454663	0.3129	0.787423	no
12.0031	13.5752	0.177565	0.453004	0.3136	0.787423	no
5.38156	6.31519	0.230803	0.449419	0.31325	0.787423	no
15.6643	13.9127	-0.171078	-0.446802	0.3134	0.787423	no
2.17496	1.80953	-0.265373	-0.44467	0.31285	0.787423	no
1.47648	1.26923	-0.218212	-0.442763	0.313	0.787423	no
6.1592	7.54651	0.293068	0.45164	0.31335	0.787423	no
1.59543	1.36607	-0.22391	-0.447564	0.31355	0.787423	no
41.8072	46.0835	0.140499	0.445877	0.31275	0.787423	no
13.871	15.4463	0.155192	0.451449	0.31315	0.787423	no
22.6093	20.7948	-0.120691	-0.450629	0.31295	0.787423	no
2.09824	1.81674	-0.207828	-0.453535	0.31345	0.787423	no
19.6011	17.7907	-0.139815	-0.444156	0.3131	0.787423	no
5.75449	5.0166	-0.197979	-0.451869	0.3128	0.787423	no
559.543	622.326	0.15342	0.446682	0.31415	0.787782	no
2.86381	3.44271	0.265607	0.452348	0.3139	0.787782	no
59.9653	54.6327	-0.134363	-0.447449	0.3141	0.787782	no
16.6757	18.4386	0.144979	0.441632	0.31385	0.787782	no
10.4514	11.4984	0.137739	0.443824	0.31395	0.787782	no
3.72354	3.20225	-0.217592	-0.440515	0.31415	0.787782	no
9.55323	8.68406	-0.137619	-0.447507	0.3143	0.787988	no
16.3966	18.249	0.154419	0.44947	0.3146	0.788185	no
8.1596	7.49967	-0.121671	-0.447547	0.3146	0.788185	no
31.7909	34.5108	0.118433	0.44413	0.3145	0.788185	no
16.3959	15.0957	-0.119197	-0.446742	0.31465	0.788185	no
9.01697	8.22328	-0.132928	-0.441332	0.3148	0.788391	no
24.4	26.8273	0.136823	0.443619	0.31505	0.788507	no
15.3757	16.9862	0.143716	0.451409	0.31495	0.788507	no
5.16995	6.11928	0.243213	0.438116	0.315	0.788507	no

30.7733	33.4545	0.120523	0.449787	0.3152	0.788543	no
3.62021	4.34976	0.264862	0.437692	0.3152	0.788543	no
4.68588	3.85469	-0.281705	-0.443217	0.3153	0.788623	no
17.6969	19.2829	0.123822	0.444447	0.3156	0.788695	no
12.2348	13.509	0.14293	0.448033	0.3155	0.788695	no
4.80216	4.30882	-0.156391	-0.447547	0.3154	0.788695	no
13.1346	14.3223	0.124886	0.441832	0.31555	0.788695	no
3.28661	3.79008	0.205629	0.44513	0.31595	0.789355	no
34.3518	31.635	-0.118864	-0.445564	0.316	0.789355	no
3.25192	2.7915	-0.220253	-0.434157	0.3162	0.789515	no
22.0347	23.9207	0.11848	0.439686	0.31615	0.789515	no
20.8688	23.1586	0.150195	0.441386	0.31635	0.789719	no
10.6516	9.68778	-0.136836	-0.44224	0.31675	0.789994	no
4.55499	5.68074	0.318631	0.443842	0.31665	0.789994	no
2.32684	2.02751	-0.198664	-0.450984	0.3167	0.789994	no
1.56591	1.28776	-0.282136	-0.451568	0.31665	0.789994	no
2.54878	3.03446	0.251631	0.449618	0.3168	0.789994	no
29.1596	26.8584	-0.118597	-0.444033	0.31695	0.790044	no
7.95232	7.2546	-0.13248	-0.445435	0.3175	0.790044	no
3.78779	3.18082	-0.251958	-0.443502	0.3174	0.790044	no
7.02665	7.96734	0.181261	0.445665	0.31735	0.790044	no
29.965	32.5693	0.120235	0.449745	0.3174	0.790044	no
10.1022	8.84681	-0.191436	-0.448889	0.3172	0.790044	no
25.0891	27.5998	0.137601	0.444245	0.3175	0.790044	no
7.11667	7.73804	0.120765	0.448422	0.3174	0.790044	no
35.3018	32.4923	-0.119643	-0.440532	0.3173	0.790044	no
124.008	135.264	0.125343	0.444581	0.31715	0.790044	no
11.2702	8.21968	-0.455359	-0.459316	0.31775	0.790069	no
6.43999	7.74016	0.265305	0.448443	0.31785	0.790069	no
44.0341	40.4861	-0.121195	-0.444086	0.3176	0.790069	no
10.8181	12.3003	0.185241	0.446937	0.31775	0.790069	no
47.3376	51.4103	0.119072	0.441746	0.31785	0.790069	no
16.1742	17.6521	0.126145	0.440835	0.318	0.790228	no
19.6723	18.0359	-0.125296	-0.44303	0.31815	0.790307	no
30.121	33.007	0.132001	0.441337	0.3186	0.791256	no
22.9134	21.0943	-0.119339	-0.444215	0.3189	0.791832	no
33.9047	36.7913	0.117881	0.442214	0.31905	0.79199	no
29.8141	27.2273	-0.13094	-0.44144	0.3191	0.79199	no
14.9305	13.7034	-0.123731	-0.440258	0.31925	0.792193	no
23.4691	21.3271	-0.13807	-0.4379	0.3197	0.792452	no
58.8014	64.0149	0.122557	0.446324	0.3199	0.792452	no
19.3659	23.2474	0.263551	0.445437	0.31985	0.792452	no
3.84829	3.17471	-0.277593	-0.438793	0.31985	0.792452	no
20.8953	22.9184	0.133328	0.439627	0.31945	0.792452	no
44.8601	48.9228	0.125074	0.437264	0.31985	0.792452	no
34.1723	37.2269	0.12352	0.453087	0.31965	0.792452	no
10.5176	8.8309	-0.252177	-0.444914	0.3199	0.792452	no

2.58677	2.1018	-0.299522	-0.440949	0.3201	0.792778	no
6.57541	5.67664	-0.212042	-0.440192	0.32035	0.793228	no
169.722	184.941	0.123892	0.439767	0.32135	0.793494	no
45.4357	49.9131	0.13559	0.436322	0.3213	0.793494	no
94.9467	87.017	-0.12582	-0.444315	0.32125	0.793494	no
41.3341	45.008	0.12285	0.443078	0.32145	0.793494	no
6.27083	7.17487	0.194296	0.443023	0.32115	0.793494	no
7.47746	8.2287	0.138117	0.435695	0.32055	0.793494	no
11.2762	10.369	-0.121004	-0.436094	0.3208	0.793494	no
26.2777	24.1474	-0.121971	-0.439335	0.3208	0.793494	no
14.4679	13.2705	-0.124628	-0.436881	0.32155	0.793494	no
11.0349	9.69323	-0.187026	-0.446311	0.32155	0.793494	no
4.74043	4.34019	-0.12726	-0.445321	0.32095	0.793494	no
66.3902	61.1213	-0.119295	-0.443403	0.32135	0.793494	no
6.24028	6.96537	0.158589	0.440137	0.32135	0.793494	no
13.1883	14.4176	0.128579	0.442304	0.32155	0.793494	no
2.47737	2.17382	-0.188573	-0.447907	0.32075	0.793494	no
4.33556	4.75105	0.132027	0.438785	0.3217	0.793605	no
6.86794	7.59675	0.145504	0.443981	0.32175	0.793605	no
32.8425	35.8727	0.127322	0.440386	0.3218	0.793605	no
1.62688	1.96648	0.27351	0.437694	0.3219	0.793683	no
49.4569	45.5999	-0.117143	-0.438168	0.322	0.793761	no
11.1214	12.7494	0.197087	0.438697	0.32255	0.79388	no
64.2367	52.311	-0.296282	-0.430632	0.3226	0.79388	no
16.2625	15.0109	-0.115538	-0.434373	0.3227	0.79388	no
5.09714	4.36593	-0.223399	-0.431534	0.3226	0.79388	no
13.6873	12.3805	-0.144772	-0.437377	0.32275	0.79388	no
8.52023	9.7453	0.193813	0.440381	0.3224	0.79388	no
2.90804	2.43971	-0.253342	-0.446093	0.32275	0.79388	no
353.305	383.996	0.120178	0.435832	0.32255	0.79388	no
4.9296	4.09712	-0.26686	-0.442162	0.3225	0.79388	no
10.8029	11.7527	0.121576	0.438996	0.32245	0.79388	no
8.79695	9.82903	0.160045	0.439306	0.32295	0.793913	no
17.0224	15.6258	-0.123504	-0.437895	0.32295	0.793913	no
5.68008	5.17609	-0.134047	-0.430691	0.3234	0.794347	no
6.34842	7.03424	0.147997	0.440062	0.3234	0.794347	no
3.97682	4.82273	0.278234	0.446806	0.3232	0.794347	no
1.71079	1.45076	-0.237853	-0.439684	0.3235	0.794425	no
13.6106	12.0091	-0.180601	-0.445013	0.32375	0.794703	no
8.35436	9.3046	0.155415	0.436566	0.3237	0.794703	no
33.6519	36.8357	0.130415	0.437031	0.3239	0.794755	no
141.815	129.947	-0.126083	-0.433889	0.3242	0.794755	no
18.1885	20.2274	0.15328	0.439104	0.324	0.794755	no
7.17899	6.32824	-0.181976	-0.441192	0.3242	0.794755	no
9.77607	10.8724	0.15334	0.439389	0.3242	0.794755	no
21.595	19.9105	-0.117168	-0.433827	0.32425	0.794755	no
4.84259	4.23065	-0.1949	-0.43592	0.32515	0.794858	no

46.4403	50.4054	0.1182	0.439375	0.3249	0.794858	no
35.1188	38.6536	0.13836	0.441689	0.32465	0.794858	no
3.48788	4.05812	0.218463	0.435984	0.32525	0.794858	no
2.14138	2.49292	0.2193	0.440404	0.32465	0.794858	no
2.90472	3.29864	0.183472	0.439739	0.3251	0.794858	no
73.5847	80.5073	0.129715	0.436277	0.3245	0.794858	no
32.048	35.6586	0.154015	0.442034	0.32455	0.794858	no
14.3172	13.1163	-0.126391	-0.439238	0.32485	0.794858	no
6.28693	7.09482	0.174411	0.436524	0.3252	0.794858	no
2.39704	2.036	-0.235515	-0.428738	0.3245	0.794858	no
7.70307	6.75429	-0.189631	-0.437567	0.3251	0.794858	no
3.24578	2.67889	-0.276932	-0.434056	0.3251	0.794858	no
23.0577	25.0825	0.121434	0.435425	0.32545	0.795012	no
1.89429	1.51644	-0.320973	-0.435403	0.32555	0.795089	no
49.2883	53.4731	0.117567	0.438996	0.32635	0.795242	no
8.12461	7.36334	-0.141938	-0.43672	0.32685	0.795242	no
13.5343	14.9567	0.144174	0.439234	0.3265	0.795242	no
13.3853	12.2801	-0.124321	-0.43878	0.32575	0.795242	no
10.6057	9.544	-0.152173	-0.440322	0.3261	0.795242	no
1.6004	1.30562	-0.293694	-0.433797	0.32635	0.795242	no
9.79645	8.88234	-0.141319	-0.43744	0.32705	0.795242	no
11.4518	10.5329	-0.120679	-0.429589	0.32695	0.795242	no
49.747	53.9459	0.116902	0.438135	0.32655	0.795242	no
12.843	14.0361	0.128159	0.439467	0.32625	0.795242	no
15.9898	17.5257	0.132319	0.430552	0.32635	0.795242	no
45.3916	41.6514	-0.124062	-0.435603	0.327	0.795242	no
43.6558	47.3316	0.11663	0.433821	0.3258	0.795242	no
3.71094	4.24363	0.193512	0.431528	0.32655	0.795242	no
12.099	10.9617	-0.142423	-0.439077	0.3266	0.795242	no
55.2478	50.9268	-0.117493	-0.431068	0.32655	0.795242	no
2.33453	2.71681	0.218783	0.432187	0.32695	0.795242	no
6.85744	7.5259	0.134194	0.428654	0.3268	0.795242	no
2.59598	2.97792	0.198023	0.437107	0.32625	0.795242	no
150.753	163.444	0.116609	0.43085	0.3273	0.795683	no
2.70521	2.1204	-0.351407	-0.438542	0.32755	0.795958	no
5.89414	5.3157	-0.149021	-0.432248	0.32755	0.795958	no
9.81565	11.3149	0.205064	0.433381	0.328	0.796718	no
7.85064	7.15467	-0.133925	-0.426412	0.32795	0.796718	no
30.6211	28.2651	-0.115504	-0.430711	0.3282	0.797038	no
2.90546	2.50281	-0.215219	-0.426328	0.32835	0.797235	no
2.3727	2.01077	-0.23878	-0.437729	0.3286	0.797283	no
2.81601	2.46242	-0.193574	-0.440593	0.32845	0.797283	no
2.06586	1.78535	-0.210536	-0.431204	0.3288	0.797283	no
1.57412	1.312	-0.262779	-0.433451	0.3287	0.797283	no
2.43846	2.03202	-0.263058	-0.429666	0.3288	0.797283	no
14.6355	13.4567	-0.121146	-0.431057	0.3287	0.797283	no
14.1453	15.6654	0.147258	0.437175	0.32885	0.797283	no

4.82445	4.12876	-0.224654	-0.420266	0.329	0.79748	no
2.51705	2.18446	-0.204456	-0.437188	0.32925	0.797753	no
8.85612	10.1054	0.190385	0.439425	0.32925	0.797753	no
5.76424	6.51917	0.177559	0.439481	0.32935	0.797829	no
2.88288	3.37485	0.227312	0.43265	0.32945	0.797905	no
4.42771	5.27031	0.251327	0.435203	0.32955	0.797981	no
10.887	9.89322	-0.138093	-0.429792	0.3299	0.798662	no
29.6113	26.904	-0.138326	-0.428591	0.3301	0.79898	no
13.3852	14.7719	0.142215	0.436326	0.33025	0.79901	no
27.15	24.9203	-0.123633	-0.433886	0.33025	0.79901	no
12.1147	13.8032	0.188245	0.435722	0.3305	0.799282	no
10.1923	11.3617	0.156697	0.433978	0.33045	0.799282	no
1.70719	1.35085	-0.337757	-0.415361	0.33075	0.79972	no
5.13855	4.38663	-0.228248	-0.425627	0.331	0.800158	no
12.6403	11.4662	-0.140644	-0.431022	0.3312	0.800475	no
19.2514	23.5082	0.288201	0.431003	0.3313	0.80055	no
7.08667	7.73891	0.127023	0.431284	0.33145	0.800746	no
30.2434	32.8332	0.118534	0.43867	0.3318	0.800892	no
7.89497	7.03559	-0.166262	-0.43016	0.33215	0.800892	no
22.6758	20.8654	-0.120042	-0.432319	0.33205	0.800892	no
1.52822	1.93652	0.341616	0.44814	0.33175	0.800892	no
42.5358	39.2819	-0.114811	-0.427494	0.33175	0.800892	no
11.6259	10.709	-0.118529	-0.428833	0.33205	0.800892	no
15.8788	14.6321	-0.117969	-0.428529	0.3322	0.800892	no
2.34542	2.07295	-0.178157	-0.438573	0.33165	0.800892	no
11.4548	10.5402	-0.120048	-0.427549	0.3321	0.800892	no
12.0663	11.0296	-0.129596	-0.432757	0.3325	0.801358	no
6.28106	5.66766	-0.148256	-0.431867	0.3326	0.801358	no
34.2886	31.6151	-0.117113	-0.428144	0.3326	0.801358	no
4.03295	3.52184	-0.195508	-0.43386	0.33275	0.801553	no
3.28757	3.86281	0.232631	0.432622	0.33295	0.801868	no
13.9957	12.6492	-0.145937	-0.433913	0.3331	0.801897	no
13.2499	15.6734	0.242333	0.4237	0.3331	0.801897	no
2.15596	2.72344	0.337095	0.414453	0.3334	0.802287	no
17.8312	19.649	0.140055	0.428453	0.3334	0.802287	no
2.37864	2.04582	-0.217461	-0.42173	0.33375	0.802373	no
2.8317	2.41444	-0.229978	-0.419399	0.33375	0.802373	no
46.3339	42.8117	-0.114065	-0.426858	0.3338	0.802373	no
12.8036	11.7979	-0.118013	-0.430091	0.33385	0.802373	no
52.4077	48.3934	-0.114969	-0.431813	0.3337	0.802373	no
3.58792	4.1839	0.221702	0.424698	0.33395	0.802447	no
3.21994	3.88597	0.271242	0.42997	0.33405	0.802475	no
1.79741	2.09838	0.223363	0.422171	0.33435	0.802744	no
2.76435	3.20912	0.21524	0.418642	0.33435	0.802744	no
14.6167	13.2036	-0.146688	-0.428944	0.33465	0.802801	no
119.622	129.705	0.116754	0.436885	0.33465	0.802801	no
1.54007	1.32421	-0.217858	-0.420997	0.3346	0.802801	no

3.34755	2.96123	-0.176912	-0.431085	0.33455	0.802801	no
3.28883	2.82502	-0.219313	-0.421222	0.3349	0.803069	no
14.4333	16.0734	0.155274	0.432848	0.335	0.803143	no
14.6216	13.4616	-0.119251	-0.427932	0.33575	0.803283	no
187.127	203.239	0.119161	0.429445	0.3354	0.803283	no
1.31806	1.59647	0.276461	0.430425	0.3355	0.803283	no
17.6819	19.4414	0.136857	0.426314	0.3355	0.803283	no
9.50944	10.7346	0.174832	0.42779	0.33535	0.803283	no
2.74456	2.36326	-0.215799	-0.431959	0.3357	0.803283	no
6.07519	7.12834	0.230636	0.425759	0.33565	0.803283	no
20.4691	18.8539	-0.118583	-0.432108	0.33555	0.803283	no
6.38657	5.71479	-0.160342	-0.429969	0.33545	0.803283	no
5.03915	4.54686	-0.148311	-0.429276	0.33535	0.803283	no
1.65075	1.30831	-0.335417	-0.428658	0.336	0.803294	no
29.4197	32.7631	0.155288	0.425364	0.3361	0.803294	no
6.28748	5.46628	-0.201922	-0.426922	0.33605	0.803294	no
23.3401	26.6411	0.190843	0.424837	0.33605	0.803294	no
25.2546	28.3475	0.166675	0.434129	0.33595	0.803294	no
5.92429	5.16778	-0.197098	-0.430356	0.3364	0.80368	no
3.00849	3.50997	0.22242	0.420538	0.33655	0.803873	no
107.928	157.965	0.549541	0.430326	0.33685	0.804259	no
1.26956	1.54308	0.281482	0.42385	0.33685	0.804259	no
1.12192	1.46846	0.388333	0.422208	0.33705	0.80436	no
32.2652	36.7543	0.187936	0.430255	0.33705	0.80436	no
4.21124	3.55752	-0.243375	-0.432645	0.3371	0.80436	no
6.84133	6.25119	-0.130145	-0.418868	0.33725	0.804507	no
7.77116	6.88215	-0.175271	-0.429756	0.3373	0.804507	no
47.4371	51.2997	0.112937	0.423781	0.3374	0.80458	no
4.18481	4.70776	0.169878	0.427798	0.3375	0.804653	no
7.56549	6.45024	-0.230081	-0.419496	0.33795	0.805469	no
9.93181	9.11255	-0.124201	-0.425773	0.338	0.805469	no
7.31814	5.9234	-0.305053	-0.424588	0.33825	0.805615	no
193.946	176.741	-0.134022	-0.424308	0.3384	0.805807	no
23.2556	25.167	0.113956	0.423059	0.3385	0.80588	no
2.53382	2.80994	0.149226	0.417735	0.3387	0.806191	no
11.0043	10.1746	-0.113096	-0.41788	0.33915	0.806436	no
51.8327	47.8867	-0.114237	-0.422771	0.3389	0.806436	no
1.12195	1.41828	0.338137	0.425684	0.33905	0.806436	no
9.22243	10.2724	0.155554	0.427198	0.33915	0.806436	no
7770.82	6493.81	-0.259003	-0.407395	0.33915	0.806436	no
12.3035	13.6421	0.148994	0.42481	0.33945	0.806489	no
20.632	23.1504	0.166152	0.429733	0.3393	0.806489	no
9.84686	10.7183	0.122344	0.428962	0.3394	0.806489	no
9.846	8.44571	-0.221319	-0.414239	0.3394	0.806489	no
2.7199	2.28254	-0.252911	-0.421306	0.3396	0.806515	no
61.421	67.3362	0.132652	0.428937	0.33955	0.806515	no
6.70043	7.4375	0.150565	0.426657	0.33985	0.806944	no

27.9163	30.8021	0.141918	0.428692	0.33995	0.807017	no
404.949	440.614	0.121774	0.425925	0.34075	0.807365	no
12.2604	13.5834	0.147839	0.42448	0.34055	0.807365	no
10.1717	9.37072	-0.118322	-0.426329	0.34045	0.807365	no
24.3704	22.317	-0.126985	-0.421805	0.34025	0.807365	no
22.2614	24.1542	0.117733	0.419539	0.3407	0.807365	no
10.2544	9.44961	-0.117916	-0.423905	0.34055	0.807365	no
4.13271	3.4229	-0.271868	-0.427518	0.3404	0.807365	no
24.7395	26.8349	0.117292	0.422971	0.3409	0.807365	no
13.0825	12.0029	-0.12425	-0.419429	0.3408	0.807365	no
7.98768	8.86619	0.150539	0.423143	0.3407	0.807365	no
32.2373	34.9811	0.117845	0.418177	0.341	0.807365	no
1.46192	1.14886	-0.347668	-0.426168	0.34085	0.807365	no
14.3673	15.6596	0.124255	0.418862	0.34095	0.807365	no
9.39635	10.735	0.192148	0.42564	0.3412	0.807417	no
8.09195	7.21902	-0.164684	-0.424483	0.34125	0.807417	no
46.4124	42.7847	-0.117416	-0.422855	0.3411	0.807417	no
19.5385	17.9821	-0.119762	-0.431252	0.3413	0.807417	no
5.42518	6.16343	0.184062	0.425425	0.34165	0.80808	no
3.20505	3.76781	0.233378	0.435312	0.3419	0.808085	no
6.92019	5.48211	-0.336081	-0.428645	0.342	0.808085	no
10.6424	9.38396	-0.181555	-0.424008	0.342	0.808085	no
5.61381	6.24664	0.1541	0.423496	0.34175	0.808085	no
11.0982	10.2155	-0.119571	-0.424862	0.342	0.808085	no
21.0649	23.2802	0.14426	0.421268	0.3421	0.808157	no
20.7829	22.9795	0.144949	0.425628	0.34245	0.808526	no
18.3819	16.7826	-0.131319	-0.419323	0.3432	0.808526	no
5.89511	8.73204	0.566801	0.421211	0.3427	0.808526	no
12.2618	13.5768	0.146975	0.419696	0.3433	0.808526	no
3.63125	4.3286	0.253435	0.419475	0.343	0.808526	no
45.366	41.7631	-0.119382	-0.417981	0.3424	0.808526	no
20.7874	19.2073	-0.11405	-0.420592	0.34315	0.808526	no
15.126	13.9675	-0.114955	-0.419862	0.343	0.808526	no
8.71857	7.98822	-0.126217	-0.421751	0.34265	0.808526	no
1.17012	1.51031	0.368194	0.425791	0.34275	0.808526	no
12.0054	11.0068	-0.125289	-0.423343	0.34315	0.808526	no
31.5931	29.2044	-0.113428	-0.42208	0.34315	0.808526	no
2.03115	2.51365	0.307488	0.420914	0.3426	0.808526	no
1.85008	2.0946	0.179088	0.428943	0.34255	0.808526	no
46.4577	50.2341	0.112748	0.422047	0.3436	0.808909	no
21.4187	18.802	-0.187989	-0.428299	0.3439	0.808909	no
14.2014	15.5475	0.13065	0.41788	0.3437	0.808909	no
115.798	125.651	0.117811	0.425895	0.34395	0.808909	no
254.772	275.577	0.113249	0.416614	0.34395	0.808909	no
76.3836	70.5299	-0.115029	-0.427838	0.34375	0.808909	no
5.52225	4.9054	-0.170885	-0.427722	0.3437	0.808909	no
2.10743	2.51919	0.257474	0.425116	0.34405	0.808981	no

7.22803	6.59722	-0.131744	-0.424513	0.3443	0.809405	no
19.8838	18.3341	-0.117063	-0.419907	0.34465	0.809736	no
17.2068	18.7157	0.121269	0.422358	0.34465	0.809736	no
2.45797	2.11626	-0.215954	-0.411812	0.34465	0.809736	no
43.6762	40.4459	-0.110855	-0.414446	0.34495	0.809739	no
6.26658	5.72252	-0.131028	-0.418189	0.34485	0.809739	no
6.3767	7.0558	0.145999	0.417328	0.345	0.809739	no
4.34262	5.29668	0.286523	0.416832	0.3448	0.809739	no
7.5699	8.4711	0.162275	0.416516	0.345	0.809739	no
8.29711	9.13168	0.138271	0.416808	0.3451	0.809811	no
59.8812	65.3514	0.126114	0.419316	0.34525	0.809953	no
29.8364	32.3221	0.115451	0.419296	0.3453	0.809953	no
22.3818	24.582	0.135276	0.426018	0.3454	0.810024	no
11.477	12.4242	0.114411	0.421551	0.3462	0.810061	no
7.07736	7.73932	0.128995	0.415402	0.3458	0.810061	no
37.6339	40.8705	0.119029	0.415133	0.3469	0.810061	no
14.4374	16.2469	0.170357	0.42201	0.3467	0.810061	no
9.6826	10.4874	0.115197	0.424727	0.3466	0.810061	no
7.46078	6.70452	-0.154193	-0.421146	0.3463	0.810061	no
10.8891	12.2244	0.166878	0.422523	0.34695	0.810061	no
15.9008	14.6536	-0.117846	-0.42439	0.3467	0.810061	no
321.096	380.592	0.245241	0.415034	0.3469	0.810061	no
2.41154	2.06574	-0.223295	-0.415623	0.34565	0.810061	no
2.13651	2.51778	0.236899	0.421764	0.3468	0.810061	no
1.63469	1.34132	-0.285364	-0.418619	0.3469	0.810061	no
24.876	23.0146	-0.112203	-0.41988	0.34615	0.810061	no
7.59211	8.94509	0.236594	0.417701	0.3464	0.810061	no
27.9472	25.8234	-0.114029	-0.420847	0.3469	0.810061	no
31.3604	28.9581	-0.114978	-0.419558	0.3461	0.810061	no
53.7036	49.6837	-0.112246	-0.41986	0.34555	0.810061	no
26.6374	24.647	-0.112038	-0.416118	0.34595	0.810061	no
9.70826	10.6708	0.13638	0.414197	0.3466	0.810061	no
12.3363	11.3581	-0.119191	-0.416137	0.3458	0.810061	no
13.108	11.8907	-0.140607	-0.416034	0.3473	0.810227	no
93.2198	86.0544	-0.115387	-0.416513	0.34725	0.810227	no
9.63209	8.84734	-0.122605	-0.416521	0.3472	0.810227	no
4.8367	4.38753	-0.140615	-0.415095	0.3472	0.810227	no
6.73599	7.50938	0.156804	0.425327	0.34755	0.810648	no
3.96893	3.53153	-0.168455	-0.420513	0.3479	0.81065	no
6.48562	7.33597	0.177744	0.418042	0.3479	0.81065	no
158.964	146.446	-0.118338	-0.416919	0.3479	0.81065	no
18.1435	19.7349	0.121296	0.418705	0.3478	0.81065	no
5.62021	4.51103	-0.31717	-0.422092	0.34835	0.811351	no
10.7142	11.8557	0.146066	0.416174	0.3485	0.811351	no
22.7572	25.0887	0.140715	0.41747	0.34845	0.811351	no
13.3679	12.3918	-0.109394	-0.409328	0.34855	0.811351	no
6.5422	7.43688	0.18492	0.424088	0.3483	0.811351	no

2.35834	2.70661	0.198714	0.419217	0.34875	0.811654	no
10.6228	11.7682	0.147735	0.414546	0.34905	0.811702	no
10.8388	10.0132	-0.114312	-0.41753	0.349	0.811702	no
15.7958	14.6015	-0.113425	-0.418805	0.34895	0.811702	no
1.872	1.58762	-0.237716	-0.412503	0.34895	0.811702	no
142.257	154.054	0.114942	0.417805	0.3494	0.811703	no
6.90493	6.15258	-0.166435	-0.412274	0.34935	0.811704	no
2.569	2.29553	-0.162375	-0.416587	0.34935	0.811704	no
4.60769	5.18738	0.170962	0.417168	0.34915	0.811704	no
3.43261	2.96784	-0.209889	-0.411312	0.3493	0.811704	no
16.0895	14.7968	-0.120834	-0.418966	0.3498	0.81194	no
41.4153	38.2911	-0.113157	-0.412295	0.3502	0.81194	no
36.0704	46.4362	0.364437	0.418998	0.35	0.81194	no
23.9105	26.1691	0.130221	0.406126	0.35045	0.81194	no
4.57798	4.06555	-0.171259	-0.424735	0.35055	0.81194	no
7.36054	6.70286	-0.135036	-0.416848	0.3496	0.81194	no
7.86381	7.11714	-0.14393	-0.414915	0.3502	0.81194	no
30.5532	33.0004	0.111159	0.407821	0.3502	0.81194	no
13.3302	11.7627	-0.180476	-0.412058	0.3505	0.81194	no
2.45498	2.13555	-0.201103	-0.413263	0.3505	0.81194	no
21.2832	23.0738	0.116543	0.418629	0.35045	0.81194	no
8.97408	8.24892	-0.12156	-0.418545	0.3502	0.81194	no
113.484	122.918	0.115199	0.41482	0.3497	0.81194	no
30.7376	28.4331	-0.112435	-0.416771	0.35025	0.81194	no
1.54131	1.31186	-0.232545	-0.408341	0.3501	0.81194	no
2.13366	1.85348	-0.203093	-0.402503	0.35125	0.812751	no
14.6515	13.4952	-0.118602	-0.416935	0.35125	0.812751	no
6.83413	7.85747	0.201307	0.411855	0.35105	0.812751	no
3.32921	4.01077	0.2687	0.424141	0.3512	0.812751	no
3.68999	2.81342	-0.391293	-0.41029	0.35195	0.812914	no
9.36646	6.18393	-0.59898	-0.101749	0.3519	0.812914	no
22.411	20.7692	-0.109761	-0.410364	0.35185	0.812914	no
45.501	49.1794	0.112154	0.420759	0.3514	0.812914	no
52.691	56.9003	0.110879	0.413991	0.3518	0.812914	no
24.4605	22.567	-0.11624	-0.404463	0.35195	0.812914	no
44.7906	50.2694	0.166485	0.411916	0.3518	0.812914	no
1.96681	1.69914	-0.211055	-0.408831	0.3519	0.812914	no
16.2656	17.5987	0.113649	0.418617	0.3517	0.812914	no
27.7222	29.9984	0.113844	0.413919	0.35225	0.813445	no
16.9837	15.6291	-0.119915	-0.408321	0.35255	0.813976	no
22.663	25.0233	0.142934	0.412905	0.3528	0.814391	no
9.57815	10.4518	0.125939	0.414806	0.3538	0.814502	no
17.2722	18.7584	0.119086	0.408076	0.35355	0.814502	no
1.1685	1.54265	0.400746	0.417524	0.35425	0.814502	no
27.5126	29.941	0.122029	0.41458	0.3537	0.814502	no
5.4021	6.0316	0.159022	0.418189	0.3542	0.814502	no
16.6403	15.3755	-0.114048	-0.410663	0.35405	0.814502	no

10.7888	9.49273	-0.184635	-0.411962	0.35365	0.814502	no
4.4068	4.8224	0.130019	0.410564	0.3533	0.814502	no
14.2186	15.5466	0.128817	0.414006	0.35385	0.814502	no
6.38154	7.92259	0.312067	0.40085	0.35325	0.814502	no
8.31914	7.66786	-0.117609	-0.40992	0.3542	0.814502	no
11.804	13.2918	0.171268	0.41326	0.35375	0.814502	no
24.1685	26.162	0.114348	0.410473	0.3534	0.814502	no
3.82543	3.2752	-0.224043	-0.404912	0.35415	0.814502	no
25.6086	23.7288	-0.109986	-0.411781	0.3539	0.814502	no
5.42611	4.94169	-0.134912	-0.408962	0.35355	0.814502	no
5.81765	5.20935	-0.159331	-0.417602	0.3542	0.814502	no
17.3203	16.0411	-0.11069	-0.415478	0.35405	0.814502	no
4.61636	3.98305	-0.21288	-0.407037	0.35315	0.814502	no
166.642	154.058	-0.113275	-0.411142	0.35465	0.815099	no
7.28873	6.70826	-0.119729	-0.413565	0.3546	0.815099	no
1.19797	1.45818	0.283583	0.413528	0.35485	0.815189	no
4.23159	5.4131	0.355255	0.416701	0.3549	0.815189	no
13.3754	14.5934	0.125731	0.409271	0.3548	0.815189	no
78.194	84.443	0.110919	0.407396	0.3555	0.815254	no
2.44507	2.02369	-0.272885	-0.416166	0.3555	0.815254	no
11.0113	10.0006	-0.138904	-0.410602	0.35565	0.815254	no
44.83	48.5348	0.114555	0.412017	0.3557	0.815254	no
10.6291	11.5863	0.124402	0.412356	0.35565	0.815254	no
10.0714	9.2771	-0.118525	-0.409444	0.3554	0.815254	no
5.78116	6.45578	0.159233	0.410752	0.35525	0.815254	no
5.77841	5.26329	-0.134708	-0.406645	0.3551	0.815254	no
1.98568	1.75145	-0.181082	-0.413583	0.35555	0.815254	no
1.95684	1.68065	-0.219503	-0.38034	0.35555	0.815254	no
1.29915	1.50278	0.210063	0.400718	0.35595	0.815666	no
17.1267	18.7087	0.127463	0.403501	0.35665	0.81589	no
4.41285	6.05946	0.457478	0.406072	0.3566	0.81589	no
49.7982	46.0425	-0.113126	-0.409245	0.35675	0.81589	no
11.8239	12.9361	0.129694	0.40822	0.35645	0.81589	no
4.26884	3.84543	-0.150698	-0.418094	0.35645	0.81589	no
51.3628	55.3407	0.107617	0.403051	0.3567	0.81589	no
20.6313	22.3238	0.113747	0.411253	0.35635	0.81589	no
9.36178	8.62195	-0.118768	-0.40901	0.35665	0.81589	no
1.49395	1.32813	-0.169733	-0.413437	0.3571	0.816323	no
7.83877	7.11081	-0.140614	-0.407672	0.35715	0.816323	no
55.0741	59.7279	0.11703	0.414224	0.35705	0.816323	no
4.35996	3.91321	-0.155965	-0.40445	0.3574	0.816687	no
76.6414	70.8726	-0.112896	-0.4054	0.35745	0.816687	no
2.71404	3.14369	0.212021	0.401756	0.35755	0.816755	no
2.62505	3.04224	0.212794	0.39872	0.35775	0.817051	no
16.8577	18.2786	0.116743	0.407371	0.3584	0.817525	no
55.1713	59.504	0.10907	0.405969	0.35815	0.817525	no
19.7324	21.3248	0.111961	0.409577	0.3583	0.817525	no

8.36611	9.33454	0.158021	0.411914	0.3584	0.817525	no
1.3234	1.61684	0.288924	0.407025	0.35845	0.817525	no
6.04925	5.21198	-0.214925	-0.411557	0.35845	0.817525	no
2.8452	3.2842	0.207016	0.407328	0.35855	0.817546	no
2.07905	1.82394	-0.188867	-0.401409	0.35915	0.818272	no
31.2412	28.8271	-0.116024	-0.406707	0.3592	0.818272	no
2.91742	2.60684	-0.162387	-0.410157	0.35945	0.818681	no
7.22244	6.67788	-0.113097	-0.406193	0.35955	0.818748	no
7.00531	6.35568	-0.140402	-0.40602	0.3605	0.818959	no
58.8957	64.0962	0.122078	0.40645	0.36065	0.818959	no
5.90216	5.30659	-0.153457	-0.407426	0.3607	0.818959	no
3.97898	3.64453	-0.126668	-0.398956	0.3603	0.818959	no
23.0097	25.7067	0.159901	0.411289	0.3604	0.818959	no
10.4636	9.01399	-0.215136	-0.403549	0.3607	0.818959	no
13.7935	14.8732	0.108731	0.407105	0.36055	0.818959	no
11.9912	10.6354	-0.173106	-0.407882	0.36035	0.818959	no
1.6899	1.47735	-0.193927	-0.410521	0.3606	0.818959	no
103.041	111.258	0.110688	0.405636	0.36005	0.818959	no
4.07098	4.63387	0.186842	0.402435	0.36005	0.818959	no
32.2591	29.898	-0.109654	-0.407883	0.3605	0.818959	no
18.6658	17.2737	-0.11182	-0.397535	0.3604	0.818959	no
4.81904	4.18682	-0.202891	-0.402295	0.36045	0.818959	no
7.80138	8.80989	0.175395	0.404852	0.36085	0.819139	no
380.695	342.298	-0.15338	-0.403272	0.3629	0.81914	no
1.74194	1.48742	-0.227883	-0.402946	0.36225	0.81914	no
3.54054	3.13511	-0.175452	-0.409138	0.3636	0.81914	no
40.3739	37.3727	-0.111438	-0.39988	0.3623	0.81914	no
8.31752	7.53432	-0.142675	-0.401501	0.36325	0.81914	no
22.2611	24.0979	0.114382	0.403197	0.3625	0.81914	no
16.6491	15.413	-0.111296	-0.40254	0.36175	0.81914	no
31.1046	33.8787	0.123251	0.406063	0.3634	0.81914	no
4.26144	3.70341	-0.202489	-0.407114	0.36135	0.81914	no
27.0742	29.1653	0.107339	0.402201	0.36295	0.81914	no
19.9451	18.4966	-0.10877	-0.407548	0.36245	0.81914	no
13.7115	14.8877	0.11874	0.403407	0.3627	0.81914	no
1.35009	1.82216	0.432598	0.4021	0.3627	0.81914	no
112.037	132.232	0.239099	0.398579	0.36205	0.81914	no
1.64352	1.92577	0.228652	0.397268	0.36355	0.81914	no
317.349	343.752	0.1153	0.399227	0.3617	0.81914	no
1.84027	2.38315	0.372951	0.42633	0.36355	0.81914	no
46.4534	50.017	0.106634	0.397324	0.36305	0.81914	no
1.77932	2.07521	0.221933	0.399283	0.3632	0.81914	no
11.1539	12.3721	0.149533	0.406696	0.3615	0.81914	no
18.9163	20.8412	0.139806	0.407943	0.3616	0.81914	no
10.4498	9.58555	-0.124539	-0.407103	0.36295	0.81914	no
13.0619	11.9917	-0.123333	-0.403544	0.36165	0.81914	no
12.6786	11.3808	-0.15579	-0.408421	0.36305	0.81914	no

10.4703	11.5694	0.144006	0.404915	0.3622	0.81914	no
5.73809	5.11975	-0.164497	-0.40449	0.36325	0.81914	no
398.027	429.186	0.108737	0.399345	0.36335	0.81914	no
2.6293	2.93045	0.156447	0.410602	0.3616	0.81914	no
54.6245	59.8665	0.132202	0.40261	0.36325	0.81914	no
167.115	182.281	0.125328	0.405756	0.36105	0.81914	no
1.56316	1.32776	-0.235466	-0.401139	0.3629	0.81914	no
8.63932	9.34759	0.113678	0.409901	0.3627	0.81914	no
4.04299	4.73255	0.227198	0.397514	0.363	0.81914	no
3.0413	3.32001	0.126498	0.402817	0.36315	0.81914	no
10.8952	12.24	0.167908	0.40835	0.3627	0.81914	no
5.52088	4.61621	-0.25819	-0.40813	0.36335	0.81914	no
2.11762	1.84023	-0.202559	-0.392382	0.3627	0.81914	no
2.90383	3.33007	0.197596	0.390175	0.3633	0.81914	no
168.969	182.203	0.108785	0.405159	0.36465	0.819255	no
2.88595	2.35627	-0.292541	-0.405632	0.36415	0.819255	no
5.07595	4.58854	-0.14564	-0.411566	0.36395	0.819255	no
1.61739	1.34335	-0.267832	-0.402269	0.3648	0.819255	no
4.51421	5.54902	0.29776	0.404146	0.3642	0.819255	no
17.1189	18.7591	0.132002	0.401055	0.3638	0.819255	no
3.68168	2.9865	-0.301907	-0.398535	0.36455	0.819255	no
2.52595	2.88667	0.192582	0.399113	0.3641	0.819255	no
9.07272	10.3943	0.196181	0.395674	0.36455	0.819255	no
3.60773	3.10122	-0.218256	-0.409543	0.3645	0.819255	no
1.61209	2.08056	0.36804	0.401146	0.36485	0.819255	no
5.89396	5.29254	-0.155276	-0.404057	0.36475	0.819255	no
7.00213	7.95398	0.183883	0.402128	0.3639	0.819255	no
6.24517	5.57221	-0.164491	-0.404049	0.3641	0.819255	no
14.0533	13.0444	-0.107476	-0.402652	0.3643	0.819255	no
1.64267	1.29166	-0.346815	-0.387644	0.36445	0.819255	no
11.1014	10.1977	-0.122499	-0.403252	0.36465	0.819255	no
16.9922	19.4027	0.191391	0.396182	0.3653	0.819316	no
39.3772	36.4939	-0.109707	-0.403169	0.36505	0.819316	no
7.35022	6.44568	-0.189455	-0.39408	0.365	0.819316	no
31.0663	33.589	0.11264	0.399527	0.36525	0.819316	no
1.96321	1.7063	-0.202341	-0.404484	0.36515	0.819316	no
2.75553	2.32855	-0.242894	-0.401913	0.3652	0.819316	no
147.951	159.799	0.111141	0.401012	0.3654	0.819382	no
2.43891	2.03219	-0.263197	-0.399495	0.3656	0.819672	no
7.61213	7.00204	-0.120526	-0.408239	0.3659	0.820186	no
5.82751	5.1385	-0.181533	-0.400117	0.36605	0.820219	no
5.68069	5.09015	-0.158357	-0.40453	0.3661	0.820219	no
8.78009	10.1991	0.216139	0.398986	0.36655	0.820219	no
13.6092	15.26	0.165175	0.399831	0.36615	0.820219	no
1.85317	2.14518	0.211104	0.401662	0.36655	0.820219	no
3.22172	3.65364	0.181506	0.405405	0.36645	0.820219	no
6.28141	6.89634	0.134743	0.399713	0.36635	0.820219	no

29.1123	26.906	-0.113701	-0.399591	0.36655	0.820219	no
7.71578	8.47521	0.135436	0.399377	0.3664	0.820219	no
1.75137	1.39206	-0.331268	-0.405895	0.3667	0.820397	no
8.18994	8.90639	0.120987	0.396937	0.367	0.820436	no
2.02441	1.11126	-0.865306	-0.440785	0.367	0.820436	no
10.9944	12.4043	0.174074	0.400364	0.36695	0.820436	no
120.369	129.888	0.109808	0.400192	0.3669	0.820436	no
25.8243	28.0704	0.120321	0.397183	0.36875	0.82046	no
42.667	46.5146	0.124564	0.392342	0.36755	0.82046	no
17.4452	15.88	-0.135625	-0.401715	0.3671	0.82046	no
23.8358	25.6827	0.107666	0.400523	0.3692	0.82046	no
6.39942	5.56122	-0.202539	-0.396798	0.36795	0.82046	no
171.033	186.725	0.126641	0.398419	0.36755	0.82046	no
2.23426	1.91722	-0.220782	-0.397539	0.3674	0.82046	no
24.9176	22.911	-0.121128	-0.409957	0.36875	0.82046	no
46.7979	43.223	-0.114646	-0.395899	0.36855	0.82046	no
1.6098	1.40351	-0.197839	-0.398224	0.3688	0.82046	no
1.66698	1.32408	-0.33225	-0.389343	0.36845	0.82046	no
6.19925	6.87861	0.150024	0.403099	0.36895	0.82046	no
12.9638	14.0806	0.119222	0.400423	0.36835	0.82046	no
184.934	171.257	-0.110845	-0.403882	0.36765	0.82046	no
1.65292	1.94897	0.23769	0.391199	0.3682	0.82046	no
8.29002	9.103	0.134966	0.398523	0.3672	0.82046	no
22.4194	24.1511	0.107345	0.401398	0.3692	0.82046	no
39.4019	36.5867	-0.106945	-0.401792	0.3686	0.82046	no
8.17835	9.21065	0.171493	0.396905	0.3682	0.82046	no
40.0583	43.9178	0.132705	0.400475	0.36905	0.82046	no
11.703	10.8054	-0.115125	-0.400989	0.3675	0.82046	no
4.72315	4.24679	-0.153377	-0.40108	0.36905	0.82046	no
4.62832	5.34422	0.207491	0.40315	0.3688	0.82046	no
19.9931	21.8079	0.125348	0.401965	0.3691	0.82046	no
3.61094	4.1671	0.206667	0.397832	0.36725	0.82046	no
42.8803	46.183	0.107049	0.400129	0.3676	0.82046	no
3.63471	3.1814	-0.192179	-0.394473	0.3691	0.82046	no
20.024	18.6072	-0.10587	-0.396466	0.369	0.82046	no
3.96204	4.34142	0.131923	0.399924	0.3692	0.82046	no
14.0475	13.0132	-0.11034	-0.395714	0.3686	0.82046	no
6.59588	5.92581	-0.154552	-0.400237	0.36945	0.820859	no
74.9765	80.7186	0.106461	0.398846	0.3701	0.82136	no
8.91085	8.21095	-0.118015	-0.401838	0.36995	0.82136	no
14.7939	16.1062	0.122612	0.394271	0.3699	0.82136	no
5.01892	4.17074	-0.267074	-0.401653	0.37005	0.82136	no
2.16712	2.48102	0.195156	0.380707	0.37	0.82136	no
6.69021	5.97965	-0.16199	-0.401989	0.36975	0.82136	no
30.0667	32.6242	0.117777	0.400689	0.3702	0.821425	no
55.9789	60.247	0.106008	0.397666	0.3707	0.82188	no
54.3708	50.4723	-0.10734	-0.398352	0.37085	0.82188	no

7.29027	6.68213	-0.125666	-0.400143	0.3709	0.82188	no
15.4419	14.2089	-0.120049	-0.398056	0.3709	0.82188	no
9.37252	10.1617	0.116636	0.399647	0.37065	0.82188	no
30.1071	27.9617	-0.106648	-0.399145	0.37055	0.82188	no
14.0512	13.0108	-0.110983	-0.39603	0.371	0.821899	no
15.1733	16.3495	0.107712	0.397735	0.37105	0.821899	no
14.2033	16.2137	0.190991	0.40068	0.3713	0.821982	no
30.6459	33.1048	0.111345	0.401733	0.3713	0.821982	no
1.53237	1.28792	-0.250722	-0.395575	0.37125	0.821982	no
15.6757	14.4677	-0.115688	-0.395522	0.3714	0.822047	no
46.6091	50.5023	0.115736	0.394067	0.3715	0.822112	no
10.3124	9.33413	-0.143799	-0.395769	0.3717	0.822141	no
1.5309	1.24134	-0.30248	-0.398342	0.37185	0.822141	no
7.6058	8.63436	0.182989	0.399379	0.37175	0.822141	no
6.40116	5.73402	-0.158787	-0.400681	0.3721	0.822141	no
19.7301	21.5063	0.124362	0.392443	0.3721	0.822141	no
32.8798	35.7453	0.120549	0.392836	0.37215	0.822141	no
24.5597	27.0992	0.141958	0.39759	0.3719	0.822141	no
8.15945	7.20722	-0.179029	-0.401874	0.3721	0.822141	no
2.73531	2.40396	-0.186296	-0.398791	0.37195	0.822141	no
1.63131	0.568037	-1.52198	-0.59688	0.3723	0.82216	no
1.78772	1.49574	-0.257256	-0.390985	0.3723	0.82216	no
21.4149	23.2783	0.120374	0.394605	0.3725	0.822445	no
11.3229	10.2816	-0.139175	-0.394873	0.3726	0.822463	no
1.6037	1.3932	-0.202995	-0.38981	0.37265	0.822463	no
15.148	16.4847	0.121998	0.391407	0.3728	0.822482	no
10.3504	11.643	0.169774	0.396941	0.3728	0.822482	no
1.71347	2.00323	0.225406	0.391502	0.3731	0.822785	no
8.40177	9.23338	0.136166	0.392579	0.37305	0.822785	no
41.6922	45.576	0.128496	0.393283	0.37315	0.822785	no
4.34137	3.86986	-0.165869	-0.39715	0.3738	0.823281	no
1.61829	1.35468	-0.256518	-0.396197	0.3737	0.823281	no
16.5679	17.8661	0.108833	0.396524	0.3738	0.823281	no
16.0212	17.3202	0.112479	0.395417	0.37355	0.823281	no
10.8628	12.214	0.169142	0.396174	0.3738	0.823281	no
8.37994	7.77804	-0.107534	-0.395	0.37355	0.823281	no
43.7828	49.9723	0.190765	0.395374	0.37395	0.823455	no
5.31988	4.72208	-0.171971	-0.398952	0.3741	0.823474	no
19.2197	17.8188	-0.109183	-0.395088	0.37405	0.823474	no
2.38409	1.50673	-0.662018	-0.409503	0.37425	0.823492	no
54.8947	51.0507	-0.104736	-0.393401	0.3742	0.823492	no
33.5229	36.3886	0.118341	0.395948	0.37455	0.823528	no
3.64748	2.85499	-0.353411	-0.395758	0.37455	0.823528	no
1.45152	1.17951	-0.299377	-0.396044	0.37435	0.823528	no
14.9181	16.8632	0.176811	0.401284	0.37455	0.823528	no
36.9072	39.7864	0.108372	0.396609	0.37485	0.82372	no
5.63015	6.36845	0.17777	0.395907	0.3748	0.82372	no

39.3772	43.8968	0.156753	0.402014	0.3748	0.82372	no
8.88015	8.18667	-0.117308	-0.391362	0.37545	0.824882	no
9.24713	8.49424	-0.122521	-0.396208	0.3758	0.825495	no
16.2665	17.6108	0.114562	0.389551	0.3761	0.825796	no
8.96041	9.6707	0.110055	0.395622	0.37615	0.825796	no
4.02767	3.64646	-0.14345	-0.389775	0.3761	0.825796	no
57.3643	61.7469	0.106214	0.393161	0.37625	0.825859	no
1.50577	1.26589	-0.250347	-0.386405	0.3764	0.825876	no
14.3339	15.5051	0.113313	0.397535	0.3764	0.825876	no
4.94385	5.49615	0.152784	0.396138	0.3768	0.826598	no
10.0673	11.6293	0.208077	0.395032	0.37695	0.826771	no
4.19136	3.79665	-0.142694	-0.390893	0.37795	0.826935	no
51.1769	55.5847	0.119196	0.391273	0.37775	0.826935	no
52.5311	48.4258	-0.117395	-0.386949	0.37795	0.826935	no
63.8129	70.9764	0.153492	0.389539	0.37755	0.826935	no
6.19436	5.7129	-0.116732	-0.387598	0.37765	0.826935	no
9.34527	8.47439	-0.141126	-0.388443	0.37765	0.826935	no
11.1504	10.2514	-0.121264	-0.385306	0.3776	0.826935	no
6.16138	5.60669	-0.136104	-0.39691	0.3779	0.826935	no
19.1084	20.7483	0.118782	0.392217	0.37785	0.826935	no
187.407	203.854	0.121364	0.39489	0.37735	0.826935	no
1.33639	1.65912	0.312077	0.390176	0.3775	0.826935	no
2.91577	3.21837	0.142452	0.395302	0.3772	0.826935	no
6.8288	6.31402	-0.113072	-0.395569	0.37775	0.826935	no
5.06792	5.92827	0.226217	0.392398	0.3781	0.827107	no
3.037	3.53203	0.217851	0.386277	0.37885	0.828328	no
2.66876	3.16469	0.245895	0.39607	0.37885	0.828328	no
3.54881	2.76654	-0.359254	-0.385019	0.37915	0.828328	no
21.4598	23.4772	0.129622	0.390212	0.37915	0.828328	no
3.17738	3.79052	0.254558	0.39469	0.3793	0.828328	no
23.8005	25.8552	0.119461	0.386378	0.37925	0.828328	no
6.52611	7.41182	0.183604	0.395688	0.3793	0.828328	no
11.569	12.5393	0.116189	0.39769	0.37945	0.828345	no
37.4004	40.1858	0.103631	0.388979	0.37945	0.828345	no
30.6549	28.5034	-0.104982	-0.394534	0.3796	0.828516	no
28.7676	32.341	0.168921	0.390585	0.3811	0.828817	no
1.75925	2.13337	0.278175	0.394432	0.3812	0.828817	no
1.52163	1.3381	-0.185428	-0.384024	0.3803	0.828817	no
3.44955	3.90652	0.179475	0.391657	0.38145	0.828817	no
71.8389	66.8503	-0.103831	-0.385842	0.3801	0.828817	no
49.7529	53.8164	0.113265	0.391662	0.3813	0.828817	no
47.3924	51.0072	0.106044	0.393349	0.38145	0.828817	no
10.3527	8.46501	-0.29042	-0.406364	0.3814	0.828817	no
43.3674	46.5925	0.103485	0.3881	0.38135	0.828817	no
1.44597	1.21263	-0.253902	-0.387921	0.38115	0.828817	no
1.19466	1.45789	0.287288	0.374406	0.3804	0.828817	no
25.4203	27.391	0.107721	0.391396	0.3801	0.828817	no

12.644	13.9518	0.142	0.393964	0.3799	0.828817	no
2.13688	2.54735	0.253489	0.389198	0.3807	0.828817	no
3.5304	4.08418	0.210215	0.389579	0.3804	0.828817	no
21.0052	22.738	0.114355	0.382947	0.38095	0.828817	no
2.74229	3.01594	0.137226	0.387915	0.3813	0.828817	no
50.5403	54.7125	0.114434	0.387169	0.37985	0.828817	no
11.5276	10.5377	-0.129538	-0.38901	0.38135	0.828817	no
2.14973	1.86562	-0.204502	-0.378123	0.38065	0.828817	no
16.8061	15.5829	-0.109018	-0.387935	0.3812	0.828817	no
2.53549	2.21876	-0.192511	-0.384562	0.38095	0.828817	no
4.77377	4.25313	-0.166606	-0.386731	0.38155	0.828833	no
5.32927	4.75529	-0.164405	-0.385703	0.3816	0.828833	no
4.22872	4.84818	0.197224	0.38756	0.38195	0.829438	no
13.0151	11.9741	-0.120276	-0.386832	0.3822	0.829485	no
35.4858	38.2744	0.109136	0.390593	0.38235	0.829485	no
78.2715	84.264	0.106429	0.390219	0.38215	0.829485	no
2.23632	1.9489	-0.198467	-0.387782	0.3822	0.829485	no
3.66683	3.11695	-0.2344	-0.389338	0.3824	0.829485	no
6.60368	6.08948	-0.116952	-0.388915	0.3824	0.829485	no
10.7597	9.89402	-0.121009	-0.387564	0.3826	0.829609	no
12.1494	11.2409	-0.112116	-0.389597	0.3826	0.829609	no
105.209	113.306	0.10697	0.387668	0.38275	0.82978	no
17.4295	18.7385	0.10447	0.384302	0.38295	0.830058	no
31.5793	33.9795	0.105686	0.384522	0.3831	0.830074	no
19.7293	21.2902	0.109854	0.389202	0.38305	0.830074	no
13.8574	12.6793	-0.128173	-0.389798	0.3832	0.830136	no
10.2548	11.0607	0.109146	0.386512	0.3837	0.830599	no
3.05199	3.44043	0.172839	0.385838	0.38365	0.830599	no
33.7604	36.2937	0.104389	0.39222	0.3835	0.830599	no
1.61994	1.9842	0.292617	0.38438	0.38365	0.830599	no
57.4164	61.8003	0.106151	0.387603	0.3838	0.830661	no
16.002	17.4891	0.128205	0.386055	0.38395	0.830831	no
2.73209	3.09448	0.179692	0.381662	0.38455	0.831046	no
12.2723	13.3002	0.11605	0.388561	0.3843	0.831046	no
15.0291	16.4514	0.130458	0.38385	0.3842	0.831046	no
17.5253	18.9109	0.109779	0.387223	0.38455	0.831046	no
29.3408	31.7769	0.115069	0.379827	0.3845	0.831046	no
12.6075	10.94	-0.204681	-0.389638	0.38425	0.831046	no
10.3934	9.59282	-0.11564	-0.382174	0.38495	0.831091	no
15.309	14.139	-0.114704	-0.383586	0.3849	0.831091	no
4.04116	3.44888	-0.22864	-0.384698	0.3848	0.831091	no
62.9561	58.579	-0.103963	-0.389404	0.38465	0.831091	no
6.46307	7.284	0.17251	0.391777	0.38495	0.831091	no
6.38981	5.87929	-0.12013	-0.379797	0.385	0.831091	no
8.9335	9.67862	0.115576	0.381188	0.38515	0.831106	no
2.59939	2.25814	-0.203033	-0.386552	0.3851	0.831106	no
1.62484	1.46716	-0.147276	-0.389356	0.38525	0.831167	no

12.1759	13.9557	0.19683	0.38728	0.38535	0.831182	no
27.3324	24.7502	-0.14317	-0.387884	0.3854	0.831182	no
4.89704	5.47834	0.161828	0.387868	0.3855	0.831243	no
12.0116	13.0618	0.120917	0.382319	0.3857	0.83152	no
21.02	22.7638	0.114978	0.389304	0.3859	0.831643	no
3.71822	3.28092	-0.180514	-0.383918	0.38585	0.831643	no
4.3251	4.89	0.1771	0.386086	0.3863	0.831733	no
26.1712	24.3581	-0.10358	-0.38608	0.38615	0.831733	no
13.7447	15.1451	0.139979	0.38749	0.3863	0.831733	no
4.30703	4.79067	0.153535	0.384659	0.38605	0.831733	no
2.66651	2.33444	-0.191877	-0.376843	0.38625	0.831733	no
52.9331	56.9427	0.105343	0.387255	0.38645	0.831748	no
5.61119	4.82088	-0.21901	-0.382932	0.3864	0.831748	no
1.81147	1.38446	-0.387834	-0.388048	0.3866	0.831763	no
34.9731	32.2093	-0.118771	-0.387483	0.38655	0.831763	no
30.638	32.9486	0.104895	0.388463	0.38675	0.831931	no
13.8943	16.0009	0.203662	0.38228	0.38685	0.831946	no
32.4558	29.8737	-0.119598	-0.38765	0.38725	0.832545	no
13.7603	15.0007	0.124517	0.385606	0.3878	0.832566	no
10.1596	11.2275	0.144198	0.389866	0.38745	0.832566	no
4.83663	4.35659	-0.1508	-0.384291	0.3875	0.832566	no
18.8594	17.5446	-0.104254	-0.383673	0.38815	0.832566	no
11.7896	10.9678	-0.104241	-0.380775	0.38855	0.832566	no
102.054	95.0239	-0.10297	-0.384471	0.38795	0.832566	no
22.3653	24.2625	0.117463	0.380171	0.3885	0.832566	no
1.34254	1.58807	0.242304	0.383019	0.38845	0.832566	no
33.7875	31.413	-0.10513	-0.384759	0.388	0.832566	no
2.62553	3.03292	0.208099	0.389036	0.38845	0.832566	no
5.30685	4.79142	-0.147403	-0.384585	0.38835	0.832566	no
9.93994	9.17622	-0.115338	-0.383698	0.38795	0.832566	no
558.512	615.688	0.140611	0.385586	0.3875	0.832566	no
13.9868	12.8177	-0.125932	-0.383576	0.3885	0.832566	no
39.6178	36.8386	-0.104931	-0.385536	0.3877	0.832566	no
18.2764	16.9999	-0.104457	-0.382153	0.38765	0.832566	no
7.4235	6.84804	-0.116408	-0.383033	0.3888	0.832948	no
20.795	22.4162	0.1083	0.38508	0.3891	0.83313	no
17.6622	15.6707	-0.172603	-0.379705	0.38905	0.83313	no
22.8022	24.7732	0.119609	0.38296	0.3891	0.83313	no
10.4683	11.6305	0.151895	0.382714	0.38955	0.833693	no
2.05302	1.78648	-0.200628	-0.373714	0.38965	0.833693	no
35.5958	38.6708	0.119538	0.381165	0.38965	0.833693	no
1.59523	1.41064	-0.177409	-0.38261	0.3896	0.833693	no
2.70202	3.11173	0.203679	0.371069	0.3902	0.834296	no
250.787	269.141	0.101895	0.382196	0.39045	0.834296	no
50.7209	47.2259	-0.103004	-0.385339	0.39015	0.834296	no
23.2683	25.4214	0.12768	0.383783	0.3905	0.834296	no
26.7669	28.7553	0.103381	0.38352	0.39065	0.834296	no

90.3515	97.3342	0.107399	0.375743	0.3903	0.834296	no
9.47869	8.79424	-0.108128	-0.385258	0.39035	0.834296	no
2.18011	1.8848	-0.209987	-0.375935	0.3906	0.834296	no
24.7835	26.6727	0.105983	0.38317	0.3901	0.834296	no
12.9297	14.1055	0.125571	0.38325	0.3908	0.83431	no
65.5959	70.4195	0.102369	0.381197	0.3908	0.83431	no
11.5338	13.3379	0.209657	0.376441	0.3909	0.83437	no
2.59212	2.9156	0.169659	0.378755	0.3911	0.834644	no
25.0948	26.9225	0.101426	0.380909	0.39145	0.83493	no
7.60991	6.76048	-0.170754	-0.380459	0.39145	0.83493	no
7874.48	8708.12	0.145176	0.379925	0.3914	0.83493	no
58.0431	54.087	-0.101844	-0.380989	0.39175	0.835417	no
32.4099	30.1799	-0.102845	-0.379215	0.3919	0.835583	no
1.41727	1.62924	0.201082	0.376144	0.392	0.835643	no
9.58005	10.3121	0.106229	0.381149	0.39215	0.83581	no
5.50251	5.00977	-0.135344	-0.385624	0.3923	0.835823	no
9.14512	7.78654	-0.232019	-0.380366	0.39225	0.835823	no
10.02	11.3737	0.182822	0.372869	0.3925	0.835849	no
5.1493	5.771	0.164443	0.380136	0.3925	0.835849	no
43.8193	47.0829	0.103638	0.381476	0.3926	0.835849	no
13.6422	15.1178	0.148174	0.37952	0.3926	0.835849	no
4.2368	3.68869	-0.199868	-0.372217	0.39275	0.835921	no
19.3065	17.919	-0.107596	-0.373825	0.39285	0.835921	no
4.08372	3.56858	-0.194535	-0.378794	0.3928	0.835921	no
24.9115	26.8281	0.106938	0.378739	0.39295	0.835981	no
19.0199	17.2654	-0.139625	-0.381153	0.39305	0.836041	no
10.2721	11.9725	0.220989	0.378526	0.39315	0.8361	no
4.15941	3.72357	-0.159693	-0.382542	0.3936	0.836445	no
45.0413	48.3629	0.102649	0.3846	0.39345	0.836445	no
11.3059	10.4124	-0.118776	-0.37143	0.3936	0.836445	no
17.5142	15.92	-0.137685	-0.379729	0.3935	0.836445	no
14.5479	15.7567	0.115157	0.380362	0.3938	0.836471	no
20.723	22.3424	0.108553	0.379228	0.3939	0.836471	no
35.1031	37.7636	0.105396	0.38572	0.3937	0.836471	no
55.7818	60.11	0.107811	0.377731	0.39385	0.836471	no
20.2189	18.7126	-0.111693	-0.38108	0.394	0.83653	no
6.23538	5.36687	-0.216396	-0.37889	0.39445	0.837179	no
282.234	303.598	0.105273	0.379011	0.39445	0.837179	no
45.2963	42.2074	-0.101896	-0.376798	0.3947	0.837251	no
22.0965	20.5878	-0.102029	-0.381813	0.3947	0.837251	no
13.1138	14.2115	0.115975	0.373074	0.39465	0.837251	no
11.958	13.1171	0.133472	0.383358	0.39485	0.837264	no
3.76522	3.3863	-0.153026	-0.376504	0.39485	0.837264	no
8.49447	7.85948	-0.11209	-0.376871	0.3951	0.837641	no
13.7298	12.7569	-0.106041	-0.375797	0.39525	0.837653	no
10.7982	9.95989	-0.116587	-0.377715	0.3952	0.837653	no
3.0777	2.59175	-0.247924	-0.375879	0.39615	0.837704	no

2.63444	2.35949	-0.159023	-0.372793	0.3955	0.837704	no
2.79127	2.54742	-0.131881	-0.378449	0.3965	0.837704	no
8.55179	7.84542	-0.124375	-0.377858	0.396	0.837704	no
2.66243	3.14633	0.240925	0.372315	0.3965	0.837704	no
13.4078	14.5683	0.119756	0.375296	0.3963	0.837704	no
13.6648	12.7377	-0.101363	-0.376979	0.39625	0.837704	no
56.7887	60.8598	0.0998837	0.374046	0.3962	0.837704	no
5.04404	4.39964	-0.197195	-0.376736	0.39615	0.837704	no
10.0486	8.95715	-0.165886	-0.374577	0.39535	0.837704	no
8.26919	11.4482	0.469305	0.404807	0.3962	0.837704	no
2.69319	3.12969	0.216702	0.376759	0.3958	0.837704	no
7.83554	7.10501	-0.141196	-0.377872	0.39585	0.837704	no
1.1296	1.40582	0.315597	0.377827	0.3958	0.837704	no
13.0188	12.0991	-0.105691	-0.383739	0.39645	0.837704	no
4.48796	5.1763	0.205861	0.374391	0.39595	0.837704	no
37.0128	34.5249	-0.100388	-0.373774	0.3962	0.837704	no
19.0076	20.4609	0.106288	0.372798	0.3967	0.837928	no
1.24021	1.50292	0.277182	0.382149	0.39675	0.837928	no
10.9994	10.1347	-0.118122	-0.375287	0.39685	0.837987	no
4.57932	5.04033	0.138383	0.375449	0.39705	0.838257	no
1.77162	2.17793	0.297886	0.3739	0.39715	0.838316	no
64.8265	70.149	0.113839	0.366322	0.39735	0.838585	no
33.0055	35.4734	0.104032	0.377985	0.3978	0.839136	no
40.959	43.9491	0.101654	0.380293	0.39785	0.839136	no
1.46467	1.69304	0.20904	0.374866	0.3979	0.839136	no
4.51678	3.89568	-0.213418	-0.383189	0.3977	0.839136	no
57.1839	62.657	0.131867	0.379225	0.398	0.839195	no
11.1711	10.2696	-0.121402	-0.374276	0.3983	0.83937	no
3.02867	2.65322	-0.190937	-0.370398	0.3983	0.83937	no
64.7614	60.4404	-0.0996215	-0.371999	0.3982	0.83937	no
2.10592	2.36826	0.169374	0.377957	0.39845	0.839534	no
3.06485	2.65227	-0.208591	-0.374028	0.3987	0.839909	no
9.46856	8.54029	-0.14886	-0.376032	0.39885	0.840072	no
12.0771	11.042	-0.129271	-0.372319	0.3991	0.840294	no
39.6658	36.9812	-0.101105	-0.369393	0.39905	0.840294	no
3.3793	3.92648	0.216514	0.370845	0.39935	0.840668	no
1.9106	1.65125	-0.210467	-0.379062	0.39945	0.840726	no
8.09107	9.03293	0.158863	0.374325	0.39975	0.841205	no
37.0591	39.7429	0.100869	0.37611	0.39985	0.841264	no
2.64831	2.25245	-0.233577	-0.374377	0.4003	0.841905	no
15.2497	14.1697	-0.105973	-0.37531	0.4011	0.842179	no
14.544	16.2315	0.158378	0.373481	0.4012	0.842179	no
14.6623	15.7983	0.107653	0.372918	0.401	0.842179	no
15.6264	14.5377	-0.104191	-0.36638	0.40125	0.842179	no
5.84083	6.50529	0.15544	0.377476	0.40105	0.842179	no
10.0911	10.8973	0.110891	0.368579	0.4013	0.842179	no
10.1313	9.38855	-0.109847	-0.370743	0.40105	0.842179	no

17.8963	19.3124	0.109869	0.373403	0.4006	0.842179	no
3.49695	3.00802	-0.217285	-0.372093	0.40125	0.842179	no
10.2383	11.0883	0.115068	0.365513	0.4007	0.842179	no
18.2757	19.6669	0.105841	0.37428	0.4013	0.842179	no
4.60359	5.08838	0.14445	0.373551	0.40085	0.842179	no
7.5455	6.95957	-0.116618	-0.370365	0.4014	0.842237	no
22.0442	24.4142	0.147323	0.376362	0.40155	0.842247	no
3.82913	4.88079	0.3501	0.37258	0.40155	0.842247	no
11.8689	12.7599	0.104424	0.369723	0.40175	0.842315	no
16.9491	15.7152	-0.109053	-0.372159	0.4018	0.842315	no
12.0446	13.0163	0.111942	0.370027	0.4017	0.842315	no
19.0968	21.1351	0.146315	0.374801	0.402	0.842583	no
3.3233	3.77959	0.185611	0.36849	0.40225	0.84286	no
4.88567	3.85275	-0.342667	-0.371436	0.40235	0.84286	no
100.98	109.564	0.117703	0.371904	0.4023	0.84286	no
9.84446	11.2904	0.197709	0.371118	0.4026	0.843232	no
9.46426	10.5629	0.158444	0.373707	0.4027	0.843289	no
8.44322	7.50986	-0.169007	-0.37193	0.4029	0.843471	no
3.78985	4.3042	0.183606	0.378155	0.40305	0.843471	no
28.2859	30.7927	0.122507	0.371808	0.4031	0.843471	no
8.34518	9.53701	0.192595	0.366404	0.40315	0.843471	no
10.1246	11.3329	0.162652	0.370495	0.40295	0.843471	no
27.6228	25.2366	-0.130338	-0.373034	0.40345	0.843549	no
3.81296	3.46512	-0.13801	-0.372006	0.40335	0.843549	no
80.1389	99.4975	0.312158	0.369268	0.4035	0.843549	no
3.75703	4.28398	0.189359	0.365475	0.40355	0.843549	no
13.5534	14.8766	0.134386	0.372887	0.4034	0.843549	no
14.9622	16.3625	0.129071	0.372116	0.40375	0.843815	no
22.0412	20.4415	-0.108706	-0.374064	0.4039	0.843977	no
24.706	26.6794	0.110863	0.364485	0.4042	0.844404	no
11.2905	12.2147	0.11351	0.367785	0.4046	0.84468	no
1114.9	937.296	-0.250331	-0.36609	0.40455	0.84468	no
8.57057	7.98558	-0.101995	-0.365125	0.40475	0.844689	no
156.937	146.59	-0.0983983	-0.368262	0.40475	0.844689	no
8.6343	7.23499	-0.255089	-0.374971	0.40495	0.844908	no
5.60395	6.15292	0.134827	0.371529	0.405	0.844908	no
11.8899	13.6262	0.196641	0.364718	0.4052	0.845173	no
108.604	101.321	-0.100155	-0.36212	0.40575	0.845514	no
1.84363	1.51241	-0.285698	-0.37389	0.40575	0.845514	no
4.16414	3.81179	-0.12755	-0.366577	0.40555	0.845514	no
3.46518	3.89923	0.17026	0.371644	0.4058	0.845514	no
45.8371	49.122	0.0998542	0.371677	0.40545	0.845514	no
25.6622	27.7714	0.113956	0.36986	0.4059	0.84557	no
5.80275	6.3658	0.133607	0.371826	0.40615	0.845693	no
1.98666	2.27742	0.19705	0.370258	0.40625	0.845693	no
2.90146	3.30315	0.187062	0.36666	0.4061	0.845693	no
18.5423	17.2869	-0.101142	-0.367582	0.4062	0.845693	no

14.5396	13.1106	-0.149253	-0.375182	0.40635	0.84575	no
16.8936	18.3597	0.120065	0.365465	0.40675	0.845824	no
22.4042	23.9875	0.0985163	0.367799	0.40655	0.845824	no
14.2666	15.275	0.0985347	0.365143	0.40675	0.845825	no
13.0221	14.0287	0.10742	0.366604	0.40685	0.845881	no
6.59063	5.61347	-0.231525	-0.364225	0.40735	0.846116	no
29.0216	31.1649	0.102793	0.366152	0.4073	0.846116	no
1.70688	2.08616	0.289487	0.369284	0.40705	0.846116	no
2.58282	2.30323	-0.165293	-0.365789	0.40715	0.846116	no
7.68796	8.7464	0.186088	0.362192	0.4074	0.846116	no
10.3928	9.60881	-0.113149	-0.369093	0.4072	0.846116	no
5.36809	5.90387	0.137252	0.368925	0.4079	0.846247	no
6.57959	6.00549	-0.131715	-0.363999	0.4079	0.846247	no
11.0938	12.4182	0.162696	0.369995	0.40765	0.846247	no
40.0223	42.9994	0.103511	0.365789	0.4079	0.846247	no
19.2431	17.9504	-0.100326	-0.368573	0.4083	0.846774	no
66.6586	71.3871	0.0988736	0.370539	0.4083	0.846774	no
19.0906	17.8187	-0.0994667	-0.370495	0.4085	0.847038	no
118.069	126.689	0.10166	0.36452	0.4086	0.847046	no
6.39335	5.88259	-0.120121	-0.368569	0.40865	0.847047	no
37.568	35.0772	-0.09897	-0.366732	0.40885	0.84731	no
1.59499	1.88841	0.243624	0.36949	0.409	0.847318	no
31.9038	26.6582	-0.259149	-0.36938	0.40895	0.847318	no
17.6574	16.4766	-0.0998535	-0.369516	0.40925	0.847685	no
23.9518	25.8276	0.108783	0.367446	0.40945	0.847948	no
21.6397	23.275	0.105101	0.361059	0.4096	0.848108	no
24.3695	22.7716	-0.0978407	-0.365931	0.40975	0.848267	no
6.93846	7.4919	0.110715	0.367045	0.40995	0.84853	no
19.6421	18.3008	-0.102044	-0.368274	0.41005	0.848586	no
8.30653	7.52842	-0.141898	-0.37039	0.41015	0.848642	no
18.5193	19.8662	0.10129	0.37052	0.41045	0.849111	no
20.2305	18.864	-0.1009	-0.366663	0.4111	0.849151	no
7.96798	7.40911	-0.104914	-0.363519	0.41115	0.849151	no
304.986	282.629	-0.109834	-0.362058	0.41095	0.849151	no
32.7402	30.0398	-0.124188	-0.36393	0.4106	0.849151	no
2.26451	2.01192	-0.170627	-0.362008	0.411	0.849151	no
3.41031	4.00077	0.230372	0.372128	0.4109	0.849151	no
4.92113	5.7531	0.225351	0.359054	0.41085	0.849151	no
22.4086	20.8965	-0.100789	-0.368293	0.411	0.849151	no
6.45359	7.03478	0.124402	0.36277	0.4111	0.849151	no
34.6588	37.1149	0.0987761	0.364779	0.41135	0.849309	no
5.6919	6.23112	0.130582	0.360668	0.41145	0.849365	no
10.1652	11.2115	0.141343	0.360779	0.4117	0.849531	no
5.2481	4.83184	-0.119224	-0.362178	0.41175	0.849531	no
6.7545	7.39626	0.130947	0.368827	0.41165	0.849531	no
4.64382	5.15809	0.151525	0.363708	0.41205	0.849801	no
9.67775	8.95149	-0.112543	-0.365971	0.4121	0.849801	no

21.3753	19.9519	-0.0994164	-0.358742	0.4121	0.849801	no
11.4652	10.6733	-0.103252	-0.363974	0.4124	0.850269	no
1.28378	1.50911	0.233307	0.365786	0.41275	0.850489	no
40.7143	37.1197	-0.13335	-0.360812	0.4128	0.850489	no
9.84998	11.6626	0.243696	0.371394	0.4128	0.85049	no
6.23217	5.37542	-0.213358	-0.365822	0.413	0.850751	no
22.6936	24.4292	0.106326	0.367501	0.41315	0.850758	no
9.06259	8.33489	-0.120761	-0.365363	0.41315	0.850758	no
21.1184	23.0336	0.125236	0.364564	0.4142	0.850819	no
5.76678	5.21616	-0.144776	-0.364994	0.41545	0.850819	no
9.36506	10.2582	0.131413	0.363758	0.4154	0.850819	no
1.37661	2.55342	0.89131	0.410618	0.4139	0.850819	no
24.3655	26.0892	0.0986133	0.367574	0.4148	0.850819	no
9.01328	8.14355	-0.146395	-0.363969	0.4141	0.850819	no
51.3671	48.0104	-0.0974957	-0.360511	0.41355	0.850819	no
44.0925	48.3069	0.131696	0.359671	0.4138	0.850819	no
126.111	117.818	-0.098142	-0.367584	0.4143	0.850819	no
17.4269	18.6946	0.101309	0.35877	0.41535	0.850819	no
73.6595	78.815	0.0975987	0.360698	0.41495	0.850819	no
8.69714	9.5583	0.136213	0.366532	0.4145	0.850819	no
1.69758	1.94594	0.196993	0.354483	0.41525	0.850819	no
65.2584	70.1161	0.103583	0.363104	0.41505	0.850819	no
1.37813	1.77408	0.364352	0.352884	0.41525	0.850819	no
5.46286	6.06914	0.151835	0.365568	0.41535	0.850819	no
7.94305	7.31444	-0.118947	-0.366118	0.41455	0.850819	no
34.7098	37.5659	0.114081	0.363016	0.41485	0.850819	no
18.5827	20.7945	0.162244	0.359953	0.4151	0.850819	no
3.38345	3.79049	0.163888	0.364469	0.41495	0.850819	no
153.104	163.708	0.096609	0.362503	0.4134	0.850819	no
16.6827	15.3101	-0.123868	-0.362203	0.4144	0.850819	no
3.01488	2.58514	-0.221858	-0.364423	0.41435	0.850819	no
7.65136	8.26448	0.111208	0.358626	0.4148	0.850819	no
5.57152	5.14586	-0.11466	-0.359965	0.4147	0.850819	no
44.7074	47.8243	0.09723	0.363631	0.4151	0.850819	no
11.0014	10.1825	-0.111588	-0.359427	0.4147	0.850819	no
6.22037	6.75331	0.118593	0.360867	0.4149	0.850819	no
23.5175	21.9545	-0.0992146	-0.364386	0.41365	0.850819	no
23.1843	25.2717	0.124377	0.3654	0.4133	0.850819	no
10.2255	9.52567	-0.102276	-0.364228	0.41415	0.850819	no
6.59824	7.23224	0.132361	0.360059	0.4157	0.851086	no
4.15039	3.61447	-0.199463	-0.352532	0.41575	0.851086	no
51.9667	48.4185	-0.102029	-0.36646	0.4158	0.851086	no
1.46147	1.78063	0.284968	0.361872	0.41595	0.851243	no
77.8589	85.9896	0.143299	0.367349	0.41615	0.851352	no
8.6162	7.96694	-0.113026	-0.359245	0.4161	0.851352	no
20.1781	21.6515	0.101677	0.357505	0.41635	0.851462	no
1.58407	1.80615	0.189288	0.358874	0.4163	0.851462	no

17.268	15.9197	-0.117283	-0.359152	0.4169	0.851537	no
38.3382	41.5037	0.114455	0.361427	0.41685	0.851537	no
28.8217	26.7916	-0.105373	-0.361659	0.4168	0.851537	no
14.7363	13.783	-0.0964877	-0.358368	0.41665	0.851537	no
16.022	17.4719	0.124976	0.362023	0.41685	0.851537	no
6.21825	6.91922	0.154101	0.364889	0.4165	0.851537	no
6.09343	6.71634	0.140421	0.35955	0.41665	0.851537	no
2.58295	2.29578	-0.170034	-0.365462	0.417	0.851592	no
666.986	727.103	0.124504	0.361769	0.4174	0.852164	no
13.5458	14.8532	0.132932	0.357258	0.4175	0.852164	no
92.529	99.1808	0.100155	0.355804	0.41745	0.852164	no
4.76483	5.3694	0.172336	0.355152	0.4176	0.852218	no
3.5274	3.09616	-0.188124	-0.353328	0.41775	0.852225	no
1.14996	1.40932	0.293415	0.365088	0.41785	0.852279	no
21.6078	23.8128	0.140183	0.360538	0.418	0.852286	no
13.2646	14.4288	0.121375	0.356859	0.41795	0.852286	no
8.21865	9.81393	0.25593	0.363337	0.4181	0.852299	no
3.85525	4.29351	0.155334	0.358643	0.4182	0.852299	no
1.83637	1.446	-0.344787	-0.364152	0.41825	0.852299	no
4.02976	5.48813	0.445621	0.38242	0.4183	0.852299	no
1.81115	2.16297	0.256106	0.35819	0.41845	0.852455	no
2.35895	2.12855	-0.148276	-0.361927	0.41855	0.852509	no
230.885	262.356	0.184351	0.359935	0.41895	0.852583	no
13.8547	12.6417	-0.132182	-0.355256	0.41875	0.852583	no
9.2012	10.3914	0.175492	0.353532	0.4187	0.852583	no
18.0804	19.7508	0.127485	0.359158	0.41895	0.852583	no
72.2856	67.3994	-0.100973	-0.353476	0.41905	0.852583	no
4.14506	3.78372	-0.131588	-0.363781	0.419	0.852583	no
32.2951	34.7978	0.107678	0.357669	0.4191	0.852583	no
3.50047	2.92076	-0.261205	-0.36987	0.41925	0.852651	no
19.9711	26.2595	0.394925	0.389639	0.4195	0.852651	no
29.4887	31.5311	0.0966121	0.357738	0.4195	0.852651	no
29.2836	31.6372	0.111531	0.360316	0.41945	0.852651	no
1.81415	2.23213	0.299127	0.35593	0.41935	0.852651	no
3.20394	3.52114	0.136194	0.36276	0.4196	0.852705	no
24.6704	23.0636	-0.097163	-0.358882	0.41995	0.852832	no
7.78989	7.1881	-0.115991	-0.361356	0.4202	0.852832	no
92.9202	99.3487	0.0965098	0.359843	0.42005	0.852832	no
5.27217	6.17566	0.228196	0.349858	0.42025	0.852832	no
25.8222	24.1614	-0.0959078	-0.358589	0.42025	0.852832	no
6.24175	5.50731	-0.180604	-0.358006	0.4199	0.852832	no
3.34583	3.75608	0.166865	0.358395	0.4202	0.852832	no
34.0941	36.6808	0.105503	0.353482	0.42035	0.852886	no
13.0343	12.1846	-0.0972521	-0.356406	0.42045	0.85294	no
4.03101	3.64515	-0.145162	-0.359248	0.42055	0.852994	no
30.2616	27.9269	-0.115832	-0.358338	0.42095	0.853507	no
35.7219	38.2707	0.0994297	0.36157	0.4209	0.853507	no

6.67317	6.20145	-0.105767	-0.356725	0.42115	0.853764	no
148.242	158.947	0.100594	0.35585	0.4214	0.854122	no
18.7896	20.2294	0.106517	0.350925	0.4215	0.854175	no
15.9958	17.2979	0.112907	0.351583	0.4216	0.854229	no
2.20297	1.93097	-0.190122	-0.354651	0.4218	0.854485	no
17.2396	18.7533	0.121417	0.359131	0.42195	0.854491	no
9.48949	8.86314	-0.0985121	-0.360267	0.42195	0.854491	no
4.56168	5.33919	0.227056	0.350788	0.42345	0.854698	no
18.9321	17.6766	-0.0989947	-0.359975	0.42245	0.854698	no
33.2397	31.1131	-0.0953873	-0.357865	0.4233	0.854698	no
26.4024	28.4427	0.107391	0.353544	0.4233	0.854698	no
7.1566	8.13409	0.184705	0.353972	0.4225	0.854698	no
3.45356	3.83161	0.149868	0.353437	0.42335	0.854698	no
17.7974	16.5429	-0.105453	-0.357614	0.4229	0.854698	no
8.88125	9.55327	0.105231	0.351429	0.4232	0.854698	no
5.44136	5.86965	0.109308	0.361203	0.42295	0.854698	no
18.0116	19.541	0.117573	0.35287	0.42345	0.854698	no
6.26278	7.21156	0.203509	0.360326	0.42215	0.854698	no
17.5359	18.7645	0.0976972	0.357629	0.4225	0.854698	no
6.06321	6.51931	0.104636	0.356996	0.42345	0.854698	no
10.3672	9.61073	-0.109307	-0.351167	0.4229	0.854698	no
24.2116	25.8961	0.0970337	0.356211	0.4232	0.854698	no
29.6307	32.1786	0.119011	0.354843	0.4231	0.854698	no
15.5546	17.622	0.180035	0.351174	0.4237	0.855054	no
34.56	39.3032	0.185542	0.358109	0.42385	0.855208	no
30.3707	28.4216	-0.0956898	-0.358789	0.42395	0.855262	no
3.36611	3.01332	-0.159729	-0.353901	0.42405	0.855315	no
12.4832	11.6003	-0.105822	-0.353045	0.42415	0.855368	no
22.318	20.6785	-0.110073	-0.348367	0.42475	0.855471	no
100.153	107.209	0.0982309	0.357678	0.4252	0.855471	no
1.36491	1.61351	0.241402	0.359725	0.42535	0.855471	no
6.4559	7.06096	0.129248	0.363156	0.4254	0.855471	no
12.0224	11.1905	-0.103449	-0.357448	0.4245	0.855471	no
48.7586	45.5683	-0.0976262	-0.358299	0.4256	0.855471	no
5.19611	5.74388	0.144593	0.358276	0.42545	0.855471	no
4.38154	4.75579	0.118248	0.354463	0.42485	0.855471	no
4.92059	5.5705	0.178976	0.346348	0.42465	0.855471	no
3.79297	4.34546	0.196182	0.356775	0.4248	0.855471	no
3.14941	2.80727	-0.165913	-0.3564	0.4245	0.855471	no
11.2644	10.5373	-0.0962648	-0.355244	0.4254	0.855471	no
39.8009	37.1281	-0.100289	-0.349655	0.4252	0.855471	no
30.2701	28.3462	-0.0947395	-0.354191	0.42445	0.855471	no
24.2311	22.5959	-0.100801	-0.353375	0.42515	0.855471	no
5.79324	5.25984	-0.139352	-0.351642	0.42555	0.855471	no
9.32333	10.1655	0.124759	0.356071	0.4256	0.855471	no
2.85344	2.54605	-0.164443	-0.350263	0.42555	0.855471	no
5.36058	4.94517	-0.116368	-0.355361	0.4248	0.855471	no

10.4162	9.22355	-0.17544	-0.35168	0.42605	0.855588	no
4.5837	5.11576	0.158435	0.35314	0.4261	0.855588	no
7.06483	6.30505	-0.164146	-0.353359	0.426	0.855588	no
4.88877	4.34369	-0.170551	-0.346579	0.42595	0.855588	no
8.9179	8.3023	-0.103193	-0.350331	0.4261	0.855588	no
39.565	37.0318	-0.0954625	-0.349648	0.42595	0.855588	no
53.5123	57.1235	0.0942125	0.353064	0.4262	0.855593	no
15.3845	16.789	0.126041	0.352552	0.42625	0.855593	no
31.9999	29.9314	-0.0964071	-0.353833	0.42805	0.855905	no
1.25727	1.47054	0.226054	0.351735	0.42655	0.855905	no
5.32236	4.84412	-0.135832	-0.354011	0.4281	0.855905	no
5.57308	4.9929	-0.158596	-0.354947	0.42675	0.855905	no
3.9754	3.32256	-0.258802	-0.359371	0.42705	0.855905	no
12.0662	11.2641	-0.0992329	-0.357655	0.4266	0.855905	no
7.02704	6.25783	-0.167255	-0.357315	0.4276	0.855905	no
2.63484	1.94053	-0.441265	-0.349739	0.42685	0.855905	no
1.29138	1.60657	0.315074	0.380656	0.428	0.855905	no
23.5254	25.2559	0.102403	0.349718	0.427	0.855905	no
8.36401	7.48553	-0.160092	-0.347716	0.42805	0.855905	no
74.139	84.9997	0.197225	0.356433	0.4279	0.855905	no
21.2724	19.9484	-0.0927139	-0.344459	0.42755	0.855905	no
4.6216	5.05106	0.128195	0.351344	0.4278	0.855905	no
1.81701	2.05045	0.174373	0.345197	0.4268	0.855905	no
13.8109	12.8584	-0.103093	-0.351129	0.4275	0.855905	no
92.5832	99.3228	0.101375	0.346284	0.428	0.855905	no
6.23822	5.46209	-0.191681	-0.350944	0.4271	0.855905	no
7.10801	7.81435	0.136681	0.351099	0.4281	0.855905	no
18.9646	20.5844	0.118245	0.353052	0.427	0.855905	no
57.1174	60.9959	0.0947816	0.355184	0.42725	0.855905	no
16.9854	15.9175	-0.0936823	-0.352563	0.4273	0.855905	no
6.638	7.25517	0.12826	0.355128	0.4278	0.855905	no
2.45426	3.0019	0.29059	0.350369	0.4282	0.855958	no
52.9021	56.4751	0.0942879	0.351295	0.4283	0.856011	no
3.55575	4.01369	0.174773	0.350316	0.4284	0.856063	no
9.11765	9.80843	0.105359	0.345605	0.4287	0.856221	no
9.42043	8.7306	-0.109712	-0.349705	0.4286	0.856221	no
147.214	157.163	0.0943442	0.349482	0.4287	0.856221	no
5.81345	6.98131	0.264103	0.350609	0.42885	0.856226	no
141.112	131.837	-0.0980868	-0.348771	0.42885	0.856226	no
24.126	26.0287	0.109512	0.350983	0.4291	0.856578	no
4.92064	5.35178	0.121174	0.351308	0.4294	0.85703	no
26.6733	24.991	-0.0939875	-0.350098	0.42965	0.857381	no
4.67552	5.32824	0.188532	0.341482	0.43015	0.857842	no
7.85689	7.22669	-0.120623	-0.354602	0.43015	0.857842	no
14.8855	13.8487	-0.104158	-0.346769	0.43025	0.857842	no
6.1597	6.84602	0.152404	0.353055	0.43025	0.857842	no
19.7034	21.405	0.119499	0.349215	0.43045	0.858093	no

33.3515	31.186	-0.0968528	-0.343613	0.43055	0.858145	no
16.4656	15.3962	-0.0968786	-0.351745	0.43065	0.858198	no
102.906	109.79	0.0934244	0.348289	0.43125	0.859246	no
14.6653	13.6011	-0.108682	-0.345267	0.4316	0.859501	no
46.0681	43.0894	-0.0964344	-0.350666	0.4316	0.859501	no
3.83316	3.48633	-0.136824	-0.352776	0.4316	0.859501	no
1.58032	1.22664	-0.365506	-0.347866	0.43175	0.859505	no
6.81233	7.51979	0.142543	0.353377	0.43175	0.859505	no
37.4508	40.059	0.0971299	0.346788	0.4324	0.860209	no
8.30944	7.19554	-0.207647	-0.344868	0.43235	0.860209	no
32.87	30.8125	-0.0932583	-0.350445	0.4322	0.860209	no
71.494	79.7039	0.156829	0.349077	0.43235	0.860209	no
13.028	12.1699	-0.0982934	-0.352897	0.43275	0.860368	no
27.9519	29.8334	0.0939838	0.350942	0.4328	0.860368	no
6.4374	6.97475	0.115662	0.346477	0.43265	0.860368	no
29.8406	32.6011	0.127644	0.347411	0.43285	0.860368	no
57.4883	61.3241	0.0931846	0.346097	0.43265	0.860368	no
64.625	69.6322	0.107662	0.343436	0.4331	0.860423	no
2.13007	2.41252	0.179636	0.34483	0.4331	0.860423	no
33.4351	36.0887	0.110183	0.352584	0.43305	0.860423	no
4.1947	3.60335	-0.219228	-0.346126	0.4333	0.860674	no
12.0772	12.9085	0.0960312	0.346354	0.4336	0.860927	no
2.29285	2.05204	-0.16008	-0.348415	0.43365	0.860927	no
28.1841	26.4108	-0.0937557	-0.349981	0.43415	0.861625	no
2.2438	2.55883	0.189541	0.346263	0.43415	0.861625	no
29.09	27.2672	-0.0933582	-0.348933	0.43425	0.861677	no
595.272	747.666	0.328846	0.34566	0.4349	0.861795	no
8.64076	7.83524	-0.141182	-0.351128	0.4347	0.861795	no
62.7328	66.9548	0.0939675	0.349589	0.4348	0.861795	no
103.933	113.27	0.124118	0.34765	0.4345	0.861795	no
10.1385	11.2692	0.152541	0.346631	0.4345	0.861795	no
10.9335	10.2458	-0.0937161	-0.352314	0.43485	0.861795	no
2.85712	2.45258	-0.220261	-0.34694	0.43505	0.861795	no
28.7088	26.922	-0.0927077	-0.348659	0.43495	0.861795	no
15.2547	14.0744	-0.11618	-0.343509	0.4352	0.861795	no
23.9021	25.6916	0.104156	0.341862	0.4352	0.861795	no
13.8954	14.8594	0.0967737	0.348513	0.43475	0.861795	no
24.5223	26.1658	0.0935886	0.351914	0.43515	0.861795	no
2.01188	1.80714	-0.154838	-0.344338	0.4355	0.861949	no
23.3194	21.8548	-0.0935832	-0.346065	0.4354	0.861949	no
26.1199	27.8763	0.0938936	0.346749	0.43545	0.861949	no
2.60845	2.20349	-0.243404	-0.345832	0.43565	0.861952	no
19.3251	20.9462	0.11621	0.344114	0.43565	0.861952	no
14.3154	15.6242	0.126213	0.344218	0.43575	0.862003	no
1.93326	1.70775	-0.178937	-0.340507	0.43585	0.862006	no
14.001	13.0289	-0.103813	-0.346658	0.4359	0.862006	no
21.394	22.8344	0.094002	0.350011	0.43615	0.862354	no

6.46538	7.15427	0.146069	0.346857	0.4363	0.862504	no
7.90965	8.63332	0.126301	0.34765	0.4364	0.862554	no
3.76464	3.41424	-0.140946	-0.350111	0.4366	0.862656	no
11.3057	10.3823	-0.122934	-0.34763	0.4367	0.862707	no
135.164	144.25	0.093862	0.349523	0.437	0.862713	no
2.06161	1.81165	-0.186468	-0.351569	0.437	0.862713	no
13.1923	14.835	0.169307	0.338255	0.43695	0.862713	no
30.6531	33.1545	0.113174	0.349012	0.43695	0.862713	no
17.5899	16.4636	-0.0954677	-0.343348	0.43715	0.862863	no
4.56731	5.08969	0.156232	0.350237	0.4373	0.863012	no
25.9235	28.1154	0.117103	0.346058	0.4375	0.86326	no
7.15812	7.68149	0.101806	0.344788	0.43785	0.863804	no
41.9239	44.7504	0.0941257	0.343898	0.4381	0.863857	no
5.58084	5.12418	-0.12316	-0.341866	0.43805	0.863857	no
10.7629	10.0098	-0.104656	-0.343111	0.438	0.863857	no
13.9431	15.1028	0.115268	0.344712	0.43855	0.863865	no
2.43697	2.13349	-0.191872	-0.339085	0.4383	0.863865	no
30.9939	33.4612	0.110508	0.347488	0.4385	0.863865	no
21.2523	19.5767	-0.118476	-0.341508	0.43835	0.863865	no
6.13466	7.28776	0.248491	0.340179	0.43845	0.863865	no
16.5648	14.9241	-0.150471	-0.340598	0.4387	0.863966	no
21.2647	23.009	0.113737	0.346987	0.43875	0.863966	no
3.38041	3.10426	-0.122949	-0.345387	0.43885	0.864016	no
12.8767	14.169	0.137978	0.348208	0.43905	0.864263	no
52.2687	49.1014	-0.0901824	-0.338981	0.4392	0.864412	no
23.2621	25.0776	0.108419	0.338997	0.43965	0.864712	no
18.4229	19.8989	0.111191	0.342724	0.4396	0.864712	no
16.5784	17.7305	0.0969278	0.341397	0.43945	0.864712	no
9.34908	8.64561	-0.112856	-0.345355	0.43955	0.864712	no
26.5944	28.8552	0.117707	0.339002	0.4398	0.864861	no
5.51844	5.12179	-0.107611	-0.336691	0.4399	0.864911	no
184.685	198.195	0.101854	0.339367	0.44135	0.865814	no
35.1975	38.7002	0.136869	0.340319	0.4418	0.865814	no
10.0557	9.37738	-0.100758	-0.344106	0.4413	0.865814	no
18.402	17.2696	-0.0916246	-0.337282	0.44065	0.865814	no
21.5981	23.2491	0.10627	0.338843	0.4415	0.865814	no
16.1131	17.4287	0.113232	0.343671	0.4412	0.865814	no
5.17625	4.50997	-0.198788	-0.339057	0.44155	0.865814	no
1519.22	1655.56	0.123981	0.339607	0.4407	0.865814	no
2.40442	1.98632	-0.275592	-0.340241	0.4412	0.865814	no
3.9321	5.27003	0.42251	0.344037	0.4417	0.865814	no
38.264	35.895	-0.0922046	-0.345007	0.4409	0.865814	no
7.68327	6.89728	-0.155693	-0.346074	0.44095	0.865814	no
11.9784	11.2455	-0.091082	-0.338642	0.4418	0.865814	no
8.32504	9.05383	0.121071	0.344802	0.44185	0.865814	no
3.68359	3.22312	-0.192655	-0.343654	0.44095	0.865814	no
25.9424	27.8624	0.103006	0.342769	0.4411	0.865814	no

15.7697	16.8405	0.0947783	0.337888	0.44095	0.865814	no
3.01359	3.35309	0.154009	0.336917	0.4417	0.865814	no
2.6168	2.96923	0.182283	0.340737	0.44145	0.865814	no
7.25139	8.02378	0.146026	0.349702	0.44195	0.865816	no
13.0871	12.0804	-0.115472	-0.34087	0.442	0.865816	no
2.10638	1.81884	-0.211748	-0.334905	0.4422	0.866062	no
3.47338	3.83848	0.144194	0.346716	0.4424	0.866162	no
5.45506	5.93691	0.122119	0.341679	0.44235	0.866162	no
68.9805	64.5304	-0.0962082	-0.349038	0.4426	0.866407	no
4.17062	3.72489	-0.163067	-0.334841	0.44275	0.866555	no
6.41261	7.19883	0.16685	0.336867	0.443	0.866898	no
3.69047	4.08363	0.146048	0.341809	0.4431	0.866948	no
8.76868	9.52962	0.120058	0.334946	0.44355	0.867586	no
10.2435	11.1506	0.122402	0.343931	0.44365	0.867586	no
25.2513	23.6273	-0.0959034	-0.341071	0.44365	0.867586	no
35.866	33.7033	-0.0897266	-0.334265	0.44385	0.867782	no
74.6023	69.9496	-0.0929034	-0.339986	0.4439	0.867782	no
69.9962	74.5831	0.0915724	0.341586	0.4442	0.868223	no
11.7619	10.869	-0.113907	-0.342548	0.4444	0.868467	no
11.9849	12.9578	0.112612	0.336037	0.4445	0.868517	no
12.0099	13.0053	0.11487	0.342021	0.4448	0.868957	no
46.1327	49.1815	0.0923264	0.34458	0.44505	0.869299	no
82.9018	88.3581	0.091959	0.338662	0.44515	0.869348	no
26.5836	28.4469	0.0977344	0.337512	0.44525	0.869397	no
13.6749	12.5179	-0.127543	-0.336838	0.4455	0.869739	no
2.1843	2.52247	0.207664	0.337193	0.44585	0.869984	no
276.398	294.136	0.0897342	0.33427	0.4457	0.869984	no
8.93718	8.30655	-0.10557	-0.337501	0.44625	0.87018	no
1.50583	1.28835	-0.225034	-0.336734	0.44615	0.87018	no
54.5145	58.0727	0.0912201	0.340982	0.4462	0.87018	no
3.38711	3.06148	-0.145824	-0.33689	0.4465	0.870229	no
18.1581	20.0916	0.145978	0.336679	0.44645	0.870229	no
12.1805	13.0516	0.0996566	0.333637	0.44755	0.870391	no
55.401	59.1545	0.0945763	0.334588	0.4526	0.870391	no
33.3113	31.1453	-0.0970002	-0.335334	0.4472	0.870391	no
208.557	222.519	0.0934861	0.339459	0.44775	0.870391	no
40.4425	43.2028	0.0952533	0.339062	0.4487	0.870391	no
4.15583	4.66209	0.16584	0.333729	0.4498	0.870391	no
1.5825	1.37989	-0.197644	-0.33802	0.44685	0.870391	no
14.8034	13.8573	-0.0952814	-0.334134	0.45355	0.870391	no
5.86208	5.38498	-0.122472	-0.335484	0.45235	0.870391	no
39.7367	37.3333	-0.090012	-0.336474	0.45335	0.870391	no
1.16874	1.45103	0.312129	0.334252	0.45015	0.870391	no
2.17073	1.89662	-0.194754	-0.333651	0.44805	0.870391	no
6.64592	7.38474	0.152079	0.334308	0.4494	0.870391	no
4.67835	5.02729	0.103781	0.339092	0.4471	0.870391	no
2.78447	2.37821	-0.227526	-0.33071	0.4502	0.870391	no

6.44511	7.12429	0.144542	0.334043	0.45215	0.870391	no
6.59522	7.26353	0.139251	0.333808	0.45385	0.870391	no
10.8498	9.99954	-0.11774	-0.339419	0.4521	0.870391	no
2.60562	2.94821	0.178216	0.336598	0.44705	0.870391	no
2.12548	2.38032	0.163362	0.327374	0.45085	0.870391	no
1.04932	1.42231	0.438775	0.335359	0.45395	0.870391	no
43.0539	40.4359	-0.0905069	-0.339067	0.44785	0.870391	no
5.87076	6.76503	0.204549	0.329412	0.45385	0.870391	no
13.953	15.0762	0.111697	0.334925	0.45345	0.870391	no
9.0555	10.3555	0.193535	0.335359	0.45195	0.870391	no
7.20493	7.84672	0.123106	0.336961	0.44815	0.870391	no
68.0658	72.587	0.0927826	0.338411	0.4495	0.870391	no
46.4195	49.3806	0.0892116	0.332492	0.45255	0.870391	no
151.861	141.615	-0.100779	-0.337303	0.45285	0.870391	no
5.01344	5.38493	0.103127	0.330066	0.454	0.870391	no
12.1444	11.1392	-0.124642	-0.341627	0.44705	0.870391	no
1.92122	1.554	-0.306035	-0.333006	0.44695	0.870391	no
7.42127	8.15449	0.135929	0.336271	0.4522	0.870391	no
2.12251	1.76062	-0.269693	-0.340768	0.4533	0.870391	no
12.0619	13.0186	0.110124	0.329655	0.44935	0.870391	no
2.71721	3.03889	0.161419	0.329536	0.4536	0.870391	no
54.5205	58.056	0.0906462	0.33517	0.4512	0.870391	no
2.43956	2.10926	-0.209879	-0.330592	0.45015	0.870391	no
30.4208	32.3555	0.0889541	0.332323	0.454	0.870391	no
52.9556	49.6633	-0.0926026	-0.336625	0.45205	0.870391	no
7.98848	7.2852	-0.132953	-0.335163	0.45335	0.870391	no
49.2369	46.2806	-0.0893321	-0.329456	0.45045	0.870391	no
11.5724	12.4778	0.108676	0.333579	0.4506	0.870391	no
12.1228	11.34	-0.0963105	-0.331832	0.45375	0.870391	no
5.53882	5.1624	-0.101537	-0.33577	0.44985	0.870391	no
22.1768	20.8207	-0.0910302	-0.341341	0.4511	0.870391	no
4.40975	4.96559	0.17127	0.328396	0.45315	0.870391	no
7.18201	6.21322	-0.209047	-0.333403	0.4496	0.870391	no
24.0619	26.0465	0.11434	0.330486	0.45355	0.870391	no
42.8461	47.3076	0.142906	0.341381	0.4497	0.870391	no
310.604	332.901	0.100019	0.33378	0.4497	0.870391	no
3.61689	3.04562	-0.248014	-0.337164	0.44915	0.870391	no
5.74928	6.24094	0.118383	0.332022	0.45375	0.870391	no
4.76467	5.26777	0.144815	0.336743	0.449	0.870391	no
3.16435	3.61061	0.190335	0.341528	0.44945	0.870391	no
20.5119	19.2672	-0.0903147	-0.333622	0.45	0.870391	no
1.42513	1.26079	-0.176767	-0.331481	0.4527	0.870391	no
24.9712	23.4496	-0.0907055	-0.336677	0.44805	0.870391	no
16.3483	15.0473	-0.119638	-0.336504	0.4536	0.870391	no
72.2555	76.8833	0.0895635	0.336013	0.45335	0.870391	no
8.81702	9.83414	0.157508	0.335029	0.4531	0.870391	no
12.1816	11.3352	-0.103893	-0.330488	0.45315	0.870391	no

606.514	566.433	-0.0986345	-0.336459	0.4533	0.870391	no
5.56768	5.11737	-0.121675	-0.336021	0.45365	0.870391	no
4.44861	3.99508	-0.155131	-0.337702	0.44995	0.870391	no
15.4534	16.6292	0.105797	0.329856	0.4539	0.870391	no
3.34734	3.74699	0.162717	0.33425	0.45105	0.870391	no
20.2635	18.2733	-0.149141	-0.337831	0.44775	0.870391	no
5.80039	5.34435	-0.118137	-0.341401	0.44745	0.870391	no
10.5192	11.307	0.104192	0.34049	0.4474	0.870391	no
5.89855	5.3614	-0.137749	-0.341374	0.4484	0.870391	no
1.1811	1.44062	0.286562	0.339657	0.4531	0.870391	no
273.263	290.858	0.0900212	0.336618	0.4525	0.870391	no
2.30578	1.91612	-0.267064	-0.336137	0.4516	0.870391	no
131.289	140.441	0.0972157	0.33738	0.44945	0.870391	no
50.544	46.5766	-0.117934	-0.340078	0.44815	0.870391	no
2.14082	1.81272	-0.240013	-0.338666	0.44885	0.870391	no
43.8269	41.1638	-0.0904385	-0.331941	0.45295	0.870391	no
10.6326	9.94121	-0.096996	-0.337453	0.4509	0.870391	no
14.8245	16.9192	0.190687	0.330541	0.4509	0.870391	no
19.4772	21.2097	0.122934	0.338278	0.44835	0.870391	no
13.4658	12.4894	-0.108596	-0.334256	0.44865	0.870391	no
8.69249	9.4046	0.113597	0.339634	0.4502	0.870391	no
1.95855	2.30945	0.237764	0.338488	0.452	0.870391	no
2.80599	3.08042	0.134619	0.33762	0.4523	0.870391	no
32.5944	34.6773	0.0893703	0.334916	0.4519	0.870391	no
9.94632	9.07115	-0.132877	-0.340288	0.4492	0.870391	no
11.1556	10.0702	-0.147675	-0.339747	0.4505	0.870391	no
60.5724	56.9314	-0.0894356	-0.333028	0.45155	0.870391	no
22.5377	24.0955	0.096423	0.33545	0.45185	0.870391	no
47.6762	50.838	0.0926377	0.344874	0.45265	0.870391	no
6.05594	5.66419	-0.0964798	-0.336861	0.4505	0.870391	no
1.72486	1.51052	-0.191436	-0.332413	0.4535	0.870391	no
1.91335	1.63998	-0.222423	-0.332962	0.4509	0.870391	no
4.80645	4.26563	-0.172214	-0.338116	0.4473	0.870391	no
58.6958	62.6274	0.0935377	0.338201	0.4541	0.870439	no
1.78027	2.02951	0.189032	0.333042	0.45425	0.87044	no
8.04791	9.43277	0.229067	0.344404	0.4542	0.87044	no
24.198	27.3099	0.174534	0.335987	0.45435	0.870488	no
11.7314	12.7024	0.11473	0.330243	0.45445	0.870536	no
4.52521	5.52034	0.286772	0.321633	0.45455	0.870584	no
21.943	23.361	0.0903438	0.336713	0.4548	0.870919	no
13.0627	14.0285	0.102905	0.327542	0.45505	0.871254	no
11.7448	11.0002	-0.0944988	-0.328794	0.45535	0.871541	no
1.85922	1.67687	-0.148931	-0.333778	0.45535	0.871541	no
14.6188	15.9546	0.126149	0.336838	0.45555	0.871781	no
5.97223	4.75014	-0.3303	-0.329412	0.4558	0.872019	no
4.01946	4.29254	0.0948298	0.330098	0.4559	0.872019	no
58.0426	54.4279	-0.0927669	-0.328306	0.4559	0.872019	no

13.3966	14.4744	0.111637	0.329483	0.456	0.872019	no
79.4161	84.6852	0.0926782	0.332048	0.45605	0.872019	no
32.7192	30.6619	-0.0936901	-0.330154	0.45725	0.87216	no
15.4454	14.5181	-0.0893285	-0.327835	0.4573	0.87216	no
8.21929	7.35096	-0.161081	-0.32811	0.4572	0.87216	no
53.4418	56.8448	0.089058	0.333513	0.4571	0.87216	no
58.6313	62.6744	0.0962074	0.327139	0.4566	0.87216	no
1.93716	2.17786	0.168968	0.327868	0.45655	0.87216	no
2.52618	2.88579	0.192009	0.333877	0.457	0.87216	no
22.439	25.0171	0.15691	0.336902	0.4566	0.87216	no
2.7159	2.21825	-0.292008	-0.33442	0.45645	0.87216	no
32.852	30.8821	-0.0892101	-0.329983	0.4574	0.87216	no
34.5035	36.8006	0.0929858	0.330688	0.4571	0.87216	no
6.46391	7.13402	0.14231	0.334021	0.45705	0.87216	no
2.0881	1.73529	-0.267017	-0.330623	0.45735	0.87216	no
2.01835	2.24982	0.156628	0.329864	0.4563	0.87216	no
2.83946	2.50959	-0.178164	-0.332847	0.4569	0.87216	no
6.69119	7.14334	0.0943358	0.328887	0.45665	0.87216	no
12.7162	11.9202	-0.0932587	-0.328898	0.45665	0.87216	no
34.5599	32.4827	-0.0894265	-0.334625	0.4575	0.872208	no
12.692	13.6462	0.104578	0.324447	0.4577	0.872446	no
3.08478	2.5968	-0.248432	-0.329603	0.45855	0.873588	no
18.4416	20.071	0.122149	0.330498	0.4584	0.873588	no
12.9249	12.0525	-0.100819	-0.328907	0.4586	0.873588	no
8.26535	8.90701	0.107865	0.326776	0.4585	0.873588	no
4.40174	5.03065	0.192671	0.334996	0.4593	0.873919	no
8.37889	9.08851	0.117286	0.330295	0.45905	0.873919	no
3.27899	3.67439	0.16425	0.329976	0.45885	0.873919	no
13.2898	12.29	-0.112826	-0.329	0.45895	0.873919	no
6.54644	7.16412	0.130079	0.330717	0.45915	0.873919	no
2.20987	2.42652	0.134923	0.329909	0.45925	0.873919	no
48.7574	51.8304	0.0881763	0.328172	0.4599	0.87458	no
15.2946	16.5236	0.111504	0.326242	0.4598	0.87458	no
2.07422	2.29656	0.146908	0.327136	0.46025	0.87458	no
130.554	121.241	-0.106772	-0.33199	0.46005	0.87458	no
28.1846	25.7284	-0.131546	-0.329207	0.4602	0.87458	no
8.61805	7.65375	-0.171194	-0.335225	0.4601	0.87458	no
3.4693	3.95431	0.188781	0.332209	0.45985	0.87458	no
13.5308	12.3182	-0.135458	-0.328707	0.46025	0.87458	no
5.81753	6.35923	0.128446	0.329797	0.4606	0.874911	no
7.48176	8.51142	0.186021	0.325903	0.46065	0.874911	no
17.9284	19.362	0.110981	0.329679	0.4605	0.874911	no
5.89824	6.44657	0.128247	0.32633	0.4608	0.875053	no
2.26234	2.5449	0.169791	0.322508	0.46115	0.875431	no
28.3295	30.8721	0.123995	0.329109	0.4611	0.875431	no
1.52	1.30166	-0.223721	-0.333668	0.46165	0.875711	no
11.5458	10.8466	-0.0901338	-0.320652	0.4614	0.875711	no

16.2483	17.3634	0.09576	0.325913	0.4619	0.875711	no
9.2209	8.44717	-0.126439	-0.329278	0.46145	0.875711	no
22.2585	23.8288	0.0983475	0.325536	0.4619	0.875711	no
5.58594	5.10329	-0.130371	-0.334735	0.46175	0.875711	no
11.6203	10.6535	-0.125323	-0.327977	0.4619	0.875711	no
1.61317	1.82029	0.174267	0.321677	0.4616	0.875711	no
1.30615	1.51075	0.209947	0.327727	0.46205	0.875852	no
4.43725	3.98335	-0.155684	-0.327496	0.4623	0.875897	no
3.33174	3.99129	0.260577	0.328598	0.4623	0.875897	no
12.7689	13.575	0.088325	0.322539	0.46225	0.875897	no
22.5021	24.5368	0.124892	0.331334	0.46245	0.876037	no
1.6195	1.40726	-0.202667	-0.330652	0.4626	0.876037	no
45.0192	50.0416	0.152589	0.321558	0.46255	0.876037	no
28.1001	29.976	0.0932341	0.325217	0.4628	0.87613	no
9.92229	10.894	0.134782	0.33012	0.46275	0.87613	no
1.27659	1.44624	0.180003	0.327875	0.4647	0.876156	no
20.1086	21.3676	0.0876121	0.324644	0.46385	0.876156	no
7.01821	6.45832	-0.119945	-0.327859	0.46485	0.876156	no
4.52498	4.00273	-0.176925	-0.321924	0.46455	0.876156	no
3.24541	2.8857	-0.169479	-0.318148	0.4645	0.876156	no
1.9163	2.2047	0.202256	0.32119	0.4637	0.876156	no
21.0693	19.7597	-0.0925781	-0.332701	0.4648	0.876156	no
2.60846	3.2975	0.338178	0.328623	0.4641	0.876156	no
5.23478	6.43331	0.297433	0.324086	0.4646	0.876156	no
22.9234	24.4545	0.0932802	0.319088	0.46415	0.876156	no
163.504	175.132	0.0991152	0.330304	0.4635	0.876156	no
71.6631	76.113	0.0869126	0.324598	0.46325	0.876156	no
6.68801	6.29005	-0.0885056	-0.325551	0.46485	0.876156	no
36.735	39.1288	0.0910729	0.32168	0.4634	0.876156	no
7.08874	7.92078	0.160112	0.325856	0.46395	0.876156	no
1.34216	1.56479	0.221414	0.325121	0.46345	0.876156	no
1.38966	1.5963	0.200005	0.326206	0.46345	0.876156	no
2.41925	2.81193	0.217003	0.325248	0.4631	0.876156	no
6.50924	5.86493	-0.150374	-0.329231	0.4631	0.876156	no
9.23986	8.66328	-0.0929577	-0.323026	0.46325	0.876156	no
5.98434	6.46876	0.112296	0.326127	0.4644	0.876156	no
69.8019	74.2411	0.0889525	0.324097	0.4641	0.876156	no
12.6489	11.8667	-0.0920912	-0.326005	0.4648	0.876156	no
2.39704	2.10208	-0.189434	-0.31904	0.46405	0.876156	no
1.56244	1.30199	-0.263088	-0.326568	0.46385	0.876156	no
2.6702	2.26149	-0.239676	-0.327808	0.4633	0.876156	no
5.29226	4.63955	-0.189898	-0.326619	0.46495	0.876203	no
180.674	196.182	0.118806	0.325476	0.4652	0.876389	no
20.6242	21.9144	0.0875399	0.324752	0.46515	0.876389	no
17.5022	19.5342	0.158465	0.32212	0.46545	0.876434	no
58.5983	62.2332	0.0868257	0.32399	0.4654	0.876434	no
1.54876	1.98273	0.356369	0.320096	0.4653	0.876434	no

7.57064	8.08254	0.0943932	0.319789	0.46605	0.876756	no
8.02126	8.65595	0.109863	0.321013	0.4661	0.876756	no
15.8531	17.076	0.107214	0.321912	0.46625	0.876756	no
1162.56	1281.95	0.141043	0.321525	0.4661	0.876756	no
19.9817	21.3268	0.0939862	0.322753	0.4662	0.876756	no
12.8231	11.964	-0.10005	-0.325696	0.46625	0.876756	no
11.9381	12.7793	0.0982319	0.320407	0.4661	0.876756	no
34.7276	36.9518	0.0895615	0.320819	0.4663	0.876756	no
14.1059	13.1878	-0.0970969	-0.319657	0.4662	0.876756	no
10.6231	13.2802	0.322074	0.317779	0.46655	0.876894	no
3.58235	3.17779	-0.172886	-0.326245	0.4666	0.876894	no
7.45021	6.67137	-0.159299	-0.290243	0.46655	0.876894	no
4.54389	4.2075	-0.110967	-0.322002	0.4667	0.87694	no
53.7271	57.2346	0.0912381	0.324064	0.46695	0.877126	no
2.87629	2.56398	-0.165822	-0.324326	0.4669	0.877126	no
17.7376	16.692	-0.0876475	-0.320202	0.4674	0.87726	no
13.801	14.6583	0.086949	0.321338	0.46725	0.87726	no
18.824	17.0073	-0.146416	-0.325141	0.46755	0.87726	no
351.965	377.317	0.100348	0.321413	0.46725	0.87726	no
17.2162	18.3374	0.0910205	0.324911	0.46755	0.87726	no
18.5357	19.7895	0.0944234	0.322886	0.4674	0.87726	no
72.6494	77.0339	0.0845422	0.317124	0.46785	0.877398	no
90.9869	97.1345	0.0943255	0.320175	0.46785	0.877398	no
18.4049	17.2636	-0.0923539	-0.322121	0.46825	0.877534	no
4.36455	3.95454	-0.142322	-0.326748	0.4683	0.877534	no
47.8885	50.8499	0.0865634	0.323641	0.4683	0.877534	no
12.1345	12.9688	0.0959219	0.324093	0.4681	0.877534	no
15.9105	14.9052	-0.0941598	-0.320515	0.4683	0.877534	no
7.14534	6.59552	-0.115516	-0.322572	0.4685	0.877767	no
27.4823	30.1262	0.132515	0.329069	0.46865	0.877906	no
10.6545	9.95748	-0.0976142	-0.322852	0.4689	0.878179	no
20.9848	18.8794	-0.152531	-0.316889	0.4694	0.878179	no
3.18949	3.82934	0.26377	0.315865	0.46905	0.878179	no
3.04766	2.74562	-0.150574	-0.317738	0.46935	0.878179	no
8.76983	9.38951	0.098502	0.322485	0.46935	0.878179	no
7.94671	7.3214	-0.118239	-0.327593	0.46935	0.878179	no
3.15716	2.90285	-0.121155	-0.324843	0.4692	0.878179	no
3727.05	3390.71	-0.136445	-0.316655	0.46895	0.878179	no
72.1743	76.7097	0.0879239	0.316472	0.46965	0.878363	no
3.9967	4.64456	0.216732	0.323294	0.46965	0.878363	no
3.33953	3.73677	0.162146	0.311774	0.4698	0.878455	no
18.5186	17.4136	-0.0887638	-0.318712	0.46985	0.878455	no
1.73904	1.56235	-0.154579	-0.322634	0.46995	0.8785	no
8.64317	9.4631	0.130754	0.320598	0.47005	0.878546	no
8.11626	8.7745	0.112503	0.326074	0.47025	0.878637	no
52.1025	48.9055	-0.091355	-0.322123	0.4705	0.878963	no
16.4539	17.5325	0.0916086	0.314429	0.4707	0.879054	no

10.5595	11.3163	0.0998615	0.319763	0.47065	0.879054	no
36.4146	34.2178	-0.0897708	-0.320797	0.4708	0.879097	no
3.26783	3.02796	-0.10999	-0.322738	0.47095	0.879097	no
24.3779	25.8802	0.0862758	0.322676	0.47095	0.879097	no
16.5123	15.3211	-0.108021	-0.321675	0.4714	0.879747	no
2.13482	1.80684	-0.240645	-0.318291	0.47145	0.879747	no
91.8308	86.378	-0.0883154	-0.321868	0.47155	0.879793	no
6.8487	7.41957	0.115505	0.324318	0.472	0.88016	no
14.7242	13.6701	-0.107167	-0.317932	0.47205	0.88016	no
2.5541	2.94546	0.205675	0.316765	0.472	0.88016	no
7.02961	6.59989	-0.0910014	-0.319474	0.47195	0.88016	no
9.07438	8.53544	-0.0883331	-0.321109	0.4723	0.880485	no
98.3027	105.382	0.100318	0.31514	0.47255	0.880668	no
2.32251	1.78708	-0.378082	-0.307672	0.47255	0.880668	no
12.1867	11.3842	-0.0982738	-0.318227	0.47265	0.880713	no
5.96967	6.55065	0.133987	0.320148	0.4729	0.880849	no
17.5013	18.6768	0.0937794	0.318869	0.4728	0.880849	no
161.085	171.563	0.0909194	0.31356	0.47295	0.880849	no
32.7397	34.7391	0.085517	0.32026	0.47335	0.881115	no
1.69994	1.41215	-0.267586	-0.319081	0.4737	0.881115	no
5.53494	4.97355	-0.154292	-0.316354	0.47355	0.881115	no
43.687	41.1864	-0.0850353	-0.319665	0.47345	0.881115	no
10.3929	11.1727	0.10438	0.315516	0.4736	0.881115	no
1.66419	1.43874	-0.210018	-0.317078	0.47365	0.881115	no
1.93755	2.19123	0.177506	0.315828	0.47365	0.881115	no
8.32044	9.18554	0.142706	0.318992	0.47325	0.881115	no
3.32586	3.84674	0.20991	0.314629	0.47395	0.881298	no
30.602	33.5042	0.130717	0.323576	0.47395	0.881298	no
12.8236	13.6956	0.0949015	0.319501	0.47425	0.881715	no
5.01092	4.6057	-0.121657	-0.319594	0.4744	0.881756	no
29.2031	30.9549	0.0840501	0.314934	0.4745	0.881756	no
36.262	38.4534	0.0846523	0.31612	0.4745	0.881756	no
2.82102	2.54061	-0.151042	-0.315218	0.4747	0.881987	no
30.2109	32.7169	0.114965	0.321396	0.47515	0.882541	no
2.75854	2.48702	-0.149486	-0.315926	0.4751	0.882541	no
19.1324	17.9376	-0.0930317	-0.315979	0.47535	0.882771	no
8.9203	9.51815	0.0935885	0.314941	0.47575	0.883324	no
24.7836	23.2231	-0.0938243	-0.305156	0.4758	0.883324	no
14.936	16.3578	0.131185	0.319818	0.47615	0.883829	no
5.07885	4.6903	-0.114821	-0.316065	0.4763	0.883829	no
10.3177	11.1733	0.114939	0.319974	0.4763	0.883829	no
27.8972	30.1485	0.111964	0.314648	0.4764	0.883873	no
17.6512	18.8915	0.0979717	0.313118	0.4765	0.883918	no
4.53433	4.96345	0.130454	0.318348	0.4766	0.883962	no
1.67049	1.3548	-0.30219	-0.309395	0.47675	0.884099	no
4.67724	5.072	0.116899	0.3191	0.47715	0.88418	no
2.55035	2.8413	0.155856	0.309046	0.47715	0.88418	no

7.72884	8.37861	0.11646	0.316722	0.47695	0.88418	no
10.039	10.7132	0.0937807	0.318691	0.477	0.88418	no
3.46376	3.11673	-0.152308	-0.315327	0.4772	0.88418	no
2.87159	2.59392	-0.146717	-0.314642	0.4777	0.884824	no
9.39436	10.2924	0.131712	0.316201	0.47775	0.884824	no
53.6632	56.9852	0.086653	0.313414	0.4794	0.884917	no
11.8378	13.5136	0.19101	0.31666	0.47935	0.884917	no
20.1156	21.3285	0.0844682	0.3165	0.4793	0.884917	no
1.29539	1.53053	0.240646	0.315048	0.47885	0.884917	no
3.29711	3.64997	0.146685	0.311471	0.47855	0.884917	no
5.12145	5.5911	0.12658	0.313562	0.4787	0.884917	no
1.83777	2.04075	0.151144	0.313601	0.47925	0.884917	no
3.26728	3.55797	0.122961	0.313685	0.4781	0.884917	no
25.5791	27.4407	0.101351	0.315926	0.4791	0.884917	no
6.68145	7.55258	0.176808	0.318792	0.47895	0.884917	no
21.6447	22.9906	0.0870275	0.315876	0.47915	0.884917	no
2.22725	2.56888	0.205875	0.31468	0.479	0.884917	no
19.5392	20.7784	0.0887104	0.321731	0.4785	0.884917	no
34.854	37.0273	0.0872667	0.315316	0.4791	0.884917	no
9.73792	9.04801	-0.106014	-0.315695	0.4794	0.884917	no
7.04164	6.53958	-0.106712	-0.316513	0.4779	0.884917	no
2.10715	1.75683	-0.262319	-0.31488	0.47815	0.884917	no
5.18186	4.71294	-0.136845	-0.317361	0.47885	0.884917	no
8.83075	8.1392	-0.117647	-0.316661	0.4788	0.884917	no
19.3564	20.666	0.0944496	0.313965	0.4792	0.884917	no
2.5521	2.2804	-0.162397	-0.312986	0.47825	0.884917	no
24.8899	23.4281	-0.0873195	-0.31575	0.47955	0.885054	no
51.6927	48.7533	-0.0844587	-0.315992	0.4799	0.885559	no
1.71072	1.98349	0.213434	0.317047	0.48035	0.885686	no
10.1051	11.0791	0.132759	0.31863	0.48005	0.885686	no
14.8436	13.9453	-0.0900583	-0.316534	0.48015	0.885686	no
31.7122	33.9085	0.0966068	0.311641	0.48035	0.885686	no
50.4928	53.5722	0.085405	0.31383	0.4802	0.885686	no
21.9547	23.2419	0.0821943	0.307269	0.4806	0.885725	no
15.2476	16.4763	0.11181	0.314772	0.4805	0.885725	no
9.47412	8.78304	-0.109271	-0.316497	0.48055	0.885725	no
10.232	10.9367	0.0960865	0.308245	0.481	0.885755	no
2.37938	2.15468	-0.143109	-0.313659	0.4811	0.885755	no
3.44059	3.12238	-0.140009	-0.317433	0.481	0.885755	no
10.7682	10.0506	-0.0995017	-0.314844	0.48115	0.885755	no
74.9549	79.6765	0.0881305	0.319132	0.481	0.885755	no
4.51641	4.20154	-0.10426	-0.3179	0.4811	0.885755	no
51.5887	54.7416	0.0855808	0.316008	0.48135	0.885838	no
10.1952	9.31817	-0.129773	-0.314087	0.4815	0.885838	no
20.3688	21.7576	0.0951623	0.314318	0.4815	0.885838	no
8.38862	7.87012	-0.0920466	-0.314154	0.4815	0.885838	no
8.59333	9.32596	0.118034	0.312875	0.48185	0.886061	no

16.9067	15.8761	-0.0907448	-0.313897	0.48185	0.886061	no
24.0635	22.638	-0.0881017	-0.318581	0.48215	0.886472	no
8.12245	7.47132	-0.120552	-0.318807	0.48235	0.886559	no
3.88487	4.23398	0.124146	0.313556	0.4823	0.886559	no
41.1563	38.761	-0.0865082	-0.313558	0.4826	0.886879	no
12.8814	13.6474	0.0833315	0.308554	0.48295	0.886961	no
51.3878	54.4341	0.0830833	0.310995	0.4828	0.886961	no
5.96056	6.47711	0.119903	0.307399	0.4828	0.886961	no
12.3593	11.4061	-0.115795	-0.314548	0.48335	0.887491	no
15.0277	14.138	-0.0880464	-0.310006	0.4837	0.887491	no
16.0723	17.2866	0.105077	0.309772	0.48365	0.887491	no
11.5264	12.9912	0.172594	0.312176	0.48355	0.887491	no
3.71309	4.15542	0.162373	0.307661	0.48385	0.887491	no
92.2328	97.7266	0.0834719	0.313297	0.48355	0.887491	no
3.0123	2.74659	-0.133221	-0.307118	0.48385	0.887491	no
2.79726	2.54171	-0.138215	-0.31323	0.4837	0.887491	no
8.04617	8.68394	0.110047	0.315202	0.48395	0.887535	no
3.6392	3.34489	-0.121659	-0.313166	0.4842	0.887616	no
15.5908	16.6197	0.0922016	0.306826	0.4843	0.887616	no
8.47639	9.00923	0.0879544	0.310859	0.48415	0.887616	no
3.19896	2.80383	-0.190206	-0.305589	0.48425	0.887616	no
9.56016	9.00799	-0.0858288	-0.307608	0.48455	0.887654	no
5.44784	4.93061	-0.143918	-0.308364	0.48445	0.887654	no
76.4601	71.4309	-0.0981598	-0.307599	0.48455	0.887654	no
1.43555	1.662	0.211316	0.314022	0.48475	0.88774	no
8.20274	7.52786	-0.123866	-0.30807	0.48475	0.88774	no
2.98234	3.31571	0.152873	0.307538	0.4849	0.887875	no
191.762	180.735	-0.0854379	-0.303869	0.4851	0.888048	no
7.46936	7.03568	-0.0862955	-0.306505	0.4852	0.888048	no
84.999	79.991	-0.087609	-0.310285	0.48525	0.888048	no
6.13609	6.75557	0.138757	0.31529	0.4853	0.888048	no
2.57408	2.85351	0.148677	0.310258	0.48555	0.888086	no
8.8272	9.38807	0.0888731	0.308606	0.4855	0.888086	no
3.4738	3.12276	-0.153691	-0.304486	0.48545	0.888086	no
6.44775	5.91694	-0.123945	-0.306878	0.48585	0.888258	no
260.517	276.157	0.0841116	0.315026	0.48595	0.888258	no
12.6081	13.5064	0.0992958	0.314791	0.48595	0.888258	no
9.9365	9.31544	-0.0931136	-0.304228	0.48585	0.888258	no
7.07302	8.11526	0.198311	0.310195	0.4873	0.88835	no
1.97643	2.28168	0.2072	0.309652	0.4873	0.88835	no
162.896	172.563	0.0831783	0.307335	0.48705	0.88835	no
2.46751	2.1928	-0.170279	-0.303408	0.4865	0.88835	no
7.10847	7.93231	0.158202	0.313012	0.4873	0.88835	no
1.79512	2.09441	0.222466	0.314105	0.48675	0.88835	no
4.59188	5.00444	0.124124	0.313346	0.48725	0.88835	no
2.70075	2.98433	0.144047	0.307065	0.48725	0.88835	no
10.8568	10.2479	-0.0832697	-0.312658	0.4868	0.88835	no

26.5205	28.1177	0.0843685	0.306568	0.4871	0.88835	no
7.07937	6.6219	-0.0963748	-0.306262	0.4863	0.88835	no
2.75592	2.47922	-0.152645	-0.308802	0.48695	0.88835	no
21.2944	22.5975	0.0856902	0.312676	0.48725	0.88835	no
4.16153	3.74116	-0.153631	-0.305928	0.4862	0.88835	no
2.00252	2.2215	0.149721	0.31028	0.487	0.88835	no
26.8546	25.3382	-0.0838534	-0.312692	0.48725	0.88835	no
73.2447	78.6712	0.103113	0.305535	0.48755	0.888382	no
13.9515	14.8652	0.0915228	0.302009	0.4877	0.888382	no
2.81947	2.51145	-0.166904	-0.306208	0.48765	0.888382	no
2.42926	2.15788	-0.170907	-0.305275	0.4877	0.888382	no
7.69133	7.1045	-0.1145	-0.308347	0.4874	0.888382	no
2.04407	1.83642	-0.154553	-0.301257	0.488	0.888789	no
4.16366	4.60965	0.146806	0.302654	0.48815	0.888923	no
5.27494	4.90069	-0.106168	-0.309283	0.48835	0.88896	no
5.97261	5.30143	-0.17198	-0.304083	0.48825	0.88896	no
4.45103	4.09743	-0.119418	-0.305812	0.4884	0.88896	no
7.39599	6.54045	-0.177353	-0.302817	0.48855	0.889045	no
12.2191	10.9493	-0.158294	-0.299326	0.4886	0.889045	no
9.53164	8.81934	-0.112054	-0.310093	0.4889	0.889173	no
9.6932	10.473	0.111634	0.3031	0.4889	0.889174	no
5.60331	5.13754	-0.125202	-0.308028	0.4888	0.889174	no
8.80159	9.63182	0.130044	0.305945	0.489	0.889216	no
16.8736	15.896	-0.0861058	-0.313644	0.48955	0.88975	no
2.24198	1.91014	-0.231099	-0.307203	0.4896	0.88975	no
26.8663	28.7912	0.0998286	0.312444	0.4896	0.88975	no
3.56152	3.88405	0.125069	0.307159	0.4897	0.889793	no
206.374	193.245	-0.0948281	-0.307719	0.4905	0.889855	no
14.201	15.0551	0.0842508	0.302537	0.49005	0.889855	no
42.4497	35.5508	-0.255873	-0.310062	0.4904	0.889855	no
39.3684	37.1892	-0.0821537	-0.308323	0.4905	0.889855	no
75.3638	70.7704	-0.0907256	-0.309732	0.4904	0.889855	no
6.82564	6.399	-0.0931184	-0.308455	0.49025	0.889855	no
17.3713	18.9302	0.12398	0.306035	0.49015	0.889855	no
31.5122	29.7743	-0.0818457	-0.306939	0.4902	0.889855	no
14.6286	13.6978	-0.094854	-0.304414	0.49045	0.889855	no
20.5352	22.1966	0.112243	0.303958	0.49135	0.890001	no
2.66645	2.26057	-0.238237	-0.30933	0.49085	0.890001	no
44.5835	47.3461	0.0867361	0.303992	0.49135	0.890001	no
34.9629	37.1326	0.0868596	0.305243	0.4908	0.890001	no
18.776	17.6587	-0.0885146	-0.307872	0.49075	0.890001	no
168.086	179.106	0.0916158	0.309902	0.4912	0.890001	no
559.227	596.591	0.0933078	0.300237	0.491	0.890001	no
406.627	431.62	0.0860567	0.310282	0.4911	0.890001	no
7.33829	7.9344	0.112677	0.307011	0.4915	0.890001	no
4.41147	4.90073	0.151737	0.310602	0.4913	0.890001	no
13.6745	14.598	0.0942803	0.298459	0.4915	0.890001	no

3.56652	3.18454	-0.163432	-0.301583	0.49255	0.890141	no
4.82892	5.37337	0.154127	0.305753	0.4925	0.890141	no
31.4333	29.605	-0.0864537	-0.303704	0.49255	0.890141	no
4.83525	5.35724	0.147898	0.306491	0.4917	0.890141	no
2.28093	2.52372	0.145935	0.292862	0.49185	0.890141	no
24.4447	25.855	0.0809257	0.302167	0.49265	0.890141	no
1.32898	1.49505	0.16987	0.304546	0.49185	0.890141	no
43.41	46.314	0.0934223	0.307411	0.49235	0.890141	no
11.4729	10.7632	-0.0921172	-0.3048	0.49245	0.890141	no
7.17237	6.68295	-0.101965	-0.305789	0.49235	0.890141	no
22.4294	23.9396	0.0940112	0.29768	0.4926	0.890141	no
6.27355	6.86577	0.130139	0.308062	0.4924	0.890141	no
2.36829	2.09418	-0.177461	-0.308084	0.49225	0.890141	no
89.0716	94.7061	0.0884906	0.302017	0.49275	0.890183	no
81.7415	87.2165	0.0935326	0.299788	0.49315	0.890346	no
16.1781	17.8397	0.141057	0.305118	0.4933	0.890346	no
20.992	19.8486	-0.0808018	-0.302402	0.49315	0.890346	no
31.7486	29.9783	-0.0827763	-0.308077	0.4931	0.890346	no
4.84478	5.39875	0.156196	0.304722	0.4932	0.890346	no
12.5834	13.7277	0.125574	0.299172	0.49325	0.890346	no
4.6805	4.31959	-0.115769	-0.306253	0.49345	0.890473	no
2.5114	2.2751	-0.142561	-0.305542	0.4936	0.890473	no
13.1235	13.8768	0.0805207	0.299279	0.4939	0.890629	no
1.95519	2.23426	0.192488	0.30568	0.49425	0.890629	no
18.0876	19.1303	0.0808617	0.299877	0.4942	0.890629	no
2.52666	2.25409	-0.164688	-0.303206	0.4941	0.890629	no
1.93583	1.72839	-0.163524	-0.304398	0.49405	0.890629	no
15.1174	14.193	-0.0910255	-0.300937	0.49425	0.890629	no
12.2964	11.5877	-0.0856469	-0.302868	0.4943	0.890629	no
121.189	128.348	0.0827991	0.301215	0.494	0.890629	no
4.07069	4.45622	0.130548	0.306602	0.49465	0.890797	no
14.0672	15.0115	0.0937367	0.307572	0.4947	0.890797	no
3.30654	2.86543	-0.206575	-0.303868	0.4948	0.890839	no
5.14376	4.72729	-0.12181	-0.308739	0.49515	0.890917	no
1.93317	1.7466	-0.146417	-0.299222	0.49515	0.890917	no
1.93715	2.25083	0.21652	0.30047	0.4954	0.891229	no
42.0621	39.7875	-0.0802045	-0.299948	0.4955	0.891271	no
7.00449	6.43282	-0.122828	-0.308837	0.4956	0.891313	no
4.13693	3.82793	-0.111996	-0.306135	0.4963	0.891558	no
1.15381	1.51674	0.394573	0.288483	0.496	0.891558	no
2.82906	3.17193	0.165038	0.300038	0.49595	0.891558	no
22.2001	20.9741	-0.0819577	-0.303752	0.49625	0.891558	no
3.54785	3.19666	-0.150377	-0.295707	0.49605	0.891558	no
9.24005	9.86442	0.0943336	0.302008	0.4962	0.891558	no
6.24203	5.73039	-0.12338	-0.305849	0.49635	0.891558	no
39.3399	36.1498	-0.122005	-0.304453	0.4967	0.891574	no
3.93206	3.66248	-0.102463	-0.299637	0.4969	0.891574	no

15.0948	16.0195	0.0857756	0.300132	0.49705	0.891574	no
13.3917	14.7179	0.136224	0.300334	0.4965	0.891574	no
5.97104	6.35687	0.0903345	0.303446	0.49675	0.891574	no
11.6371	12.3506	0.085839	0.305687	0.49665	0.891574	no
6.11556	6.56061	0.101345	0.29941	0.4969	0.891574	no
10.8583	11.5279	0.0863339	0.301681	0.4966	0.891574	no
17.2281	15.6145	-0.141876	-0.304702	0.49705	0.891574	no
1.57784	1.77149	0.16701	0.299246	0.4973	0.891687	no
1.87622	1.63745	-0.196375	-0.2992	0.4975	0.891687	no
16.2833	17.5207	0.105665	0.305613	0.4976	0.891687	no
2.81793	2.53186	-0.154437	-0.300026	0.49765	0.891687	no
19.5387	17.8183	-0.132973	-0.296403	0.4976	0.891687	no
2.36332	1.79674	-0.395438	-0.308644	0.49735	0.891687	no
2.22015	2.81012	0.339975	0.295053	0.49745	0.891687	no
548.489	618.426	0.173138	0.292108	0.49805	0.892266	no
29.2267	30.9281	0.0816349	0.298409	0.49825	0.892487	no
2.51601	2.22645	-0.176391	-0.295227	0.4985	0.892749	no
4.77708	5.7309	0.262632	0.30144	0.49855	0.892749	no
7.33435	7.81452	0.0914888	0.296518	0.4989	0.892825	no
9.89449	11.2381	0.183696	0.29857	0.49885	0.892825	no
3.66931	3.96808	0.11293	0.301101	0.49875	0.892825	no
8.91903	9.59978	0.106114	0.301501	0.4989	0.892825	no
7.54807	8.31856	0.140225	0.296374	0.49915	0.893135	no
2.79787	3.1084	0.151844	0.294347	0.49935	0.893218	no
5.76763	6.30479	0.128471	0.298369	0.4993	0.893218	no
11.287	12.0924	0.0994358	0.302759	0.49975	0.893795	no
13.6144	14.5022	0.0911387	0.295691	0.5	0.893802	no
3.90303	3.61368	-0.111127	-0.30325	0.5006	0.893802	no
15.2264	14.3463	-0.0858903	-0.294477	0.5004	0.893802	no
12.7591	13.5617	0.088016	0.300129	0.5005	0.893802	no
2.46997	2.09833	-0.235254	-0.298857	0.50005	0.893802	no
2.60734	2.33627	-0.158372	-0.300886	0.5004	0.893802	no
5.27207	5.72521	0.118961	0.303026	0.5003	0.893802	no
11.5484	12.2616	0.0864502	0.298691	0.50025	0.893802	no
2.53263	2.83085	0.160594	0.287346	0.5002	0.893802	no
923.278	768.541	-0.264643	-0.309514	0.5003	0.893802	no
17.1539	16.196	-0.0828991	-0.304939	0.50055	0.893802	no
1.31607	1.54029	0.226963	0.303784	0.50085	0.893878	no
33.1335	31.3314	-0.0806815	-0.301587	0.50095	0.893878	no
10.4148	9.71822	-0.0998701	-0.299051	0.50085	0.893878	no
194.814	183.486	-0.0864281	-0.302089	0.50115	0.89396	no
6.31545	7.47864	0.24389	0.286509	0.5011	0.89396	no
15.8591	16.7621	0.0798882	0.298966	0.50125	0.894001	no
7.59992	8.35239	0.136205	0.29963	0.50145	0.89422	no
2.3344	2.65102	0.183498	0.294636	0.50175	0.894296	no
14.1812	15.2617	0.105936	0.300124	0.5018	0.894296	no
65.8222	69.5867	0.080237	0.298836	0.50175	0.894296	no

8.15955	9.49467	0.218628	0.300403	0.5019	0.894337	no
2.89774	3.35405	0.210977	0.295396	0.502	0.894378	no
8.24115	7.74379	-0.0898049	-0.292529	0.50215	0.894508	no
2.22463	1.99394	-0.157939	-0.294547	0.5028	0.895117	no
14.3555	13.4116	-0.0981203	-0.296215	0.5028	0.895117	no
26.103	24.7068	-0.0793062	-0.295415	0.503	0.895335	no
1.85387	1.63468	-0.18153	-0.293717	0.5033	0.895499	no
1.53531	1.79275	0.223646	0.299406	0.50325	0.895499	no
12.2251	13.0548	0.0947297	0.298612	0.50335	0.895499	no
29.3072	27.6201	-0.0855359	-0.295112	0.5034	0.895499	no
6.88372	7.53441	0.130307	0.298492	0.5039	0.895873	no
10.577	11.2864	0.0936575	0.296496	0.5038	0.895873	no
118.047	127.424	0.110273	0.29463	0.5041	0.895873	no
20.239	21.3946	0.0801103	0.294171	0.50395	0.895873	no
9.10106	9.7584	0.10061	0.297998	0.50395	0.895873	no
9.05672	9.84151	0.119892	0.302372	0.50415	0.895873	no
1.77283	1.56994	-0.175346	-0.292533	0.5041	0.895873	no
21.7421	23.4763	0.110715	0.299866	0.50425	0.895913	no
13.6645	14.8272	0.11781	0.29689	0.50565	0.89593	no
5.70047	5.32192	-0.0991348	-0.291691	0.5066	0.89593	no
8.56369	7.93822	-0.109417	-0.298889	0.50455	0.89593	no
14.4203	13.566	-0.0881022	-0.296753	0.5066	0.89593	no
14.0767	14.9849	0.0901924	0.29401	0.5049	0.89593	no
16.03	15.0384	-0.0921266	-0.294409	0.50585	0.89593	no
3.25773	3.71676	0.190177	0.297577	0.5061	0.89593	no
3.73551	3.30004	-0.178822	-0.298047	0.50655	0.89593	no
4.37146	3.97748	-0.136261	-0.298418	0.50655	0.89593	no
17.412	18.4295	0.0819337	0.298679	0.50645	0.89593	no
1.41988	1.25109	-0.182577	-0.300627	0.5057	0.89593	no
1.71864	1.91779	0.158177	0.292561	0.5065	0.89593	no
23.4447	24.8219	0.0823501	0.303739	0.50665	0.89593	no
32.3035	30.5996	-0.0781777	-0.291917	0.50625	0.89593	no
16.9328	15.891	-0.0916165	-0.292065	0.50625	0.89593	no
17.3676	16.432	-0.07989	-0.299031	0.5049	0.89593	no
3.3409	3.73173	0.159609	0.292425	0.50465	0.89593	no
3.2753	3.81951	0.22176	0.294612	0.5062	0.89593	no
3.71533	4.09068	0.138852	0.29471	0.5063	0.89593	no
16.3672	17.7935	0.120546	0.297174	0.5061	0.89593	no
43.2871	40.862	-0.083176	-0.294902	0.5066	0.89593	no
5.63637	5.05272	-0.157705	-0.29902	0.50545	0.89593	no
3.93535	4.40437	0.162443	0.295209	0.50635	0.89593	no
4.04019	3.69803	-0.127665	-0.295876	0.50495	0.89593	no
5.12531	4.65136	-0.139989	-0.298122	0.5063	0.89593	no
9.46019	10.1727	0.104765	0.295389	0.50565	0.89593	no
49.9757	53.583	0.100549	0.295338	0.50585	0.89593	no
11.276	12.1619	0.109108	0.295668	0.5063	0.89593	no
27.4749	25.5261	-0.106139	-0.299032	0.5046	0.89593	no

9.67181	9.11056	-0.0862461	-0.296427	0.50445	0.89593	no
3.05794	3.51768	0.202065	0.295354	0.50675	0.89597	no
17.8503	18.9208	0.0840224	0.297305	0.5069	0.896099	no
4.59047	4.96945	0.114444	0.296562	0.5073	0.89619	no
4.43732	4.7787	0.106929	0.294062	0.50745	0.89619	no
7.39005	8.62302	0.222609	0.294023	0.5077	0.89619	no
3.4946	3.0563	-0.193341	-0.29559	0.5078	0.89619	no
2.5492	2.01933	-0.336168	-0.284122	0.50775	0.89619	no
2.11524	2.26883	0.101124	0.295156	0.50745	0.89619	no
5.89283	6.48006	0.137046	0.297162	0.50715	0.89619	no
8.73437	7.92361	-0.140545	-0.29473	0.50755	0.89619	no
11.5299	12.6012	0.128183	0.300095	0.50775	0.89619	no
13.0303	14.1199	0.115853	0.29816	0.5072	0.89619	no
1.32352	1.44589	0.127576	0.295194	0.50755	0.89619	no
5.72817	6.57268	0.198408	0.295825	0.5079	0.896231	no
2.31286	2.61014	0.17445	0.295354	0.5081	0.896447	no
22.4274	23.6972	0.0794561	0.298337	0.50835	0.89648	no
443.492	474.949	0.0988651	0.297726	0.5082	0.89648	no
8.6978	8.04673	-0.112249	-0.297906	0.50835	0.89648	no
44.2462	47.9967	0.117382	0.296947	0.50875	0.896505	no
16.6095	15.7056	-0.0807317	-0.292963	0.5087	0.896505	no
11.8018	11.0726	-0.0920225	-0.293211	0.50855	0.896505	no
1.85095	1.47624	-0.326336	-0.291381	0.5085	0.896505	no
175.788	166.402	-0.0791671	-0.29619	0.50875	0.896505	no
69.6712	73.5445	0.0780559	0.289836	0.5091	0.896786	no
1.58713	1.81097	0.190338	0.294605	0.50945	0.896786	no
34.2518	36.7173	0.100281	0.294248	0.50945	0.896786	no
3.84287	4.20308	0.129261	0.293153	0.5093	0.896786	no
19.6409	18.5052	-0.0859261	-0.292779	0.5093	0.896786	no
1.71167	1.9616	0.196626	0.285829	0.5094	0.896786	no
3.81201	4.15681	0.124925	0.299189	0.509	0.896786	no
5.32553	5.71707	0.10235	0.29436	0.51065	0.896893	no
11.298	11.9919	0.0859918	0.288852	0.51085	0.896893	no
264.878	246.085	-0.106175	-0.288742	0.5097	0.896893	no
6.20443	5.79878	-0.0975512	-0.291457	0.5106	0.896893	no
5.12295	4.76372	-0.104888	-0.295616	0.5104	0.896893	no
112.486	118.897	0.0799702	0.295183	0.5099	0.896893	no
1.69924	1.96554	0.210039	0.291802	0.5109	0.896893	no
6.28933	5.78625	-0.120277	-0.298692	0.50975	0.896893	no
1.70373	2.07392	0.283664	0.294737	0.5101	0.896893	no
5.97907	6.77344	0.179967	0.291174	0.51065	0.896893	no
10.102	9.42455	-0.100146	-0.289576	0.51085	0.896893	no
34.3099	32.5048	-0.077973	-0.291766	0.5107	0.896893	no
8.63775	9.18378	0.0884324	0.296225	0.51075	0.896893	no
55.8131	52.8736	-0.0780544	-0.292653	0.51005	0.896893	no
111.202	104.348	-0.0917926	-0.295008	0.51005	0.896893	no
3.20455	3.52949	0.139341	0.290784	0.51055	0.896893	no

25.2011	26.7192	0.0843908	0.292919	0.5103	0.896893	no
2.28159	2.48342	0.12229	0.290442	0.511	0.896933	no
1.44823	1.25904	-0.201958	-0.285735	0.51145	0.89726	no
4.15493	3.79348	-0.131304	-0.288554	0.51155	0.89726	no
31.2773	33.0966	0.0815667	0.295113	0.5115	0.89726	no
28.3193	29.8594	0.0764012	0.285525	0.51165	0.89726	no
1.52764	1.26512	-0.272034	-0.292973	0.51165	0.89726	no
5.93344	5.62442	-0.0771656	-0.290256	0.5129	0.897493	no
4.26732	4.93297	0.209128	0.289793	0.5124	0.897493	no
86.6104	82.0458	-0.0781101	-0.291536	0.51275	0.897493	no
7.82378	8.46756	0.114081	0.29919	0.5134	0.897493	no
6.61536	7.04801	0.0913958	0.290981	0.51295	0.897493	no
5.39881	6.2376	0.208348	0.29225	0.51225	0.897493	no
9.5378	8.86837	-0.104987	-0.291207	0.5139	0.897493	no
16.916	15.8571	-0.0932633	-0.289236	0.5126	0.897493	no
17.211	16.2913	-0.0792289	-0.295373	0.51325	0.897493	no
24.0454	25.3822	0.0780574	0.289205	0.51345	0.897493	no
2.04463	1.2706	-0.686336	-0.257264	0.51335	0.897493	no
5.45622	6.05724	0.150757	0.291926	0.51395	0.897493	no
26.2367	24.8573	-0.0779123	-0.287276	0.51325	0.897493	no
2.65008	2.27438	-0.220561	-0.289651	0.51385	0.897493	no
3.56198	3.27505	-0.121166	-0.286784	0.51365	0.897493	no
71.013	74.9826	0.0784716	0.288888	0.51255	0.897493	no
67.2983	71.3273	0.0838838	0.292406	0.5123	0.897493	no
2.08348	1.88345	-0.145618	-0.28533	0.51405	0.897493	no
8.11039	7.29713	-0.152442	-0.288609	0.5137	0.897493	no
2.07032	1.85977	-0.154731	-0.28435	0.5141	0.897493	no
45.1741	47.7162	0.0789828	0.291425	0.5121	0.897493	no
17.5356	16.4718	-0.0902842	-0.286557	0.5133	0.897493	no
106.226	112.138	0.0781348	0.287008	0.5131	0.897493	no
4.24227	3.92866	-0.110797	-0.292945	0.5138	0.897493	no
1.73515	2.36518	0.44689	0.297012	0.51255	0.897493	no
3.1167	2.88719	-0.110357	-0.290073	0.5141	0.897493	no
4.84161	4.21714	-0.19922	-0.284248	0.51285	0.897493	no
28.2349	26.5528	-0.0886135	-0.289348	0.51315	0.897493	no
6.67799	7.21654	0.111893	0.29296	0.5141	0.897493	no
10.532	11.1492	0.0821585	0.288726	0.51475	0.897596	no
5.57757	6.0642	0.120679	0.288644	0.51505	0.897596	no
16.2498	17.7038	0.123634	0.291414	0.51505	0.897596	no
22.6686	23.9895	0.0817106	0.290497	0.51555	0.897596	no
32.5883	34.3912	0.0776822	0.289178	0.5144	0.897596	no
9.77175	10.5609	0.112038	0.289034	0.5143	0.897596	no
2.36919	2.08752	-0.182605	-0.293697	0.51535	0.897596	no
3.02223	3.40824	0.173413	0.292344	0.51465	0.897596	no
57.9151	54.1524	-0.0969166	-0.290495	0.5154	0.897596	no
41.7002	44.0378	0.0786887	0.288409	0.5151	0.897596	no
2.86551	2.5874	-0.147286	-0.287635	0.51535	0.897596	no

46.9047	49.5395	0.0788473	0.289619	0.5155	0.897596	no
6.73005	6.21954	-0.113808	-0.292427	0.5148	0.897596	no
52.1459	55.0459	0.0780816	0.289012	0.51475	0.897596	no
10.7558	9.7466	-0.142147	-0.291237	0.51545	0.897596	no
25.9862	24.4955	-0.0852314	-0.286459	0.51475	0.897596	no
21.6864	23.0332	0.0869265	0.288844	0.51575	0.89781	no
18.2321	17.2201	-0.0823883	-0.291054	0.51615	0.897968	no
31.439	33.6113	0.0963879	0.285756	0.51615	0.897968	no
5.55918	5.93441	0.0942315	0.285991	0.5161	0.897968	no
75.3927	79.5579	0.0775814	0.288644	0.51605	0.897968	no
1.76318	2.02833	0.202117	0.283607	0.51645	0.898284	no
4.20125	3.60551	-0.220616	-0.296416	0.51695	0.898284	no
29.255	30.9205	0.0798787	0.289049	0.51675	0.898284	no
4.03675	3.76861	-0.0991643	-0.286304	0.51685	0.898284	no
11.012	11.7158	0.0893777	0.290866	0.51695	0.898284	no
61.1775	64.5409	0.0772139	0.289513	0.5169	0.898284	no
42.5319	40.2895	-0.0781418	-0.29314	0.5166	0.898284	no
59.0421	62.3899	0.0795683	0.289964	0.51665	0.898284	no
6.66518	6.30225	-0.080778	-0.289415	0.5172	0.898584	no
9.81265	10.503	0.0980925	0.287054	0.5173	0.898623	no
20.3529	21.5166	0.0802128	0.285898	0.5174	0.898662	no
4.89521	4.60811	-0.0871954	-0.29046	0.51765	0.898962	no
21.5996	22.7881	0.0772738	0.287266	0.51785	0.898993	no
8.7649	9.37971	0.0978056	0.290749	0.51775	0.898993	no
2.22181	2.03585	-0.126104	-0.2842	0.5179	0.898993	no
23.2786	24.7658	0.08934	0.283416	0.518	0.899033	no
7.05683	8.94251	0.341661	0.292529	0.51845	0.899276	no
58.2648	61.5851	0.0799581	0.286666	0.51845	0.899276	no
12.0887	12.7805	0.0802836	0.285448	0.51845	0.899276	no
2.68507	2.50552	-0.0998499	-0.288981	0.51835	0.899276	no
25.5591	26.9423	0.076037	0.283519	0.51885	0.899282	no
312.24	331.399	0.0859174	0.284678	0.51915	0.899282	no
1.52993	1.27429	-0.263771	-0.288026	0.5188	0.899282	no
4.40836	4.15853	-0.0841677	-0.283377	0.51875	0.899282	no
73.0969	77.1692	0.0782156	0.287104	0.51915	0.899282	no
12.6881	13.6801	0.108602	0.291956	0.5187	0.899282	no
12.9316	12.2284	-0.0806594	-0.28872	0.519	0.899282	no
3.56507	3.21221	-0.150364	-0.289382	0.5188	0.899282	no
11.171	12.6457	0.178885	0.285074	0.51905	0.899282	no
24.1278	22.0845	-0.127665	-0.290252	0.51945	0.899478	no
85.4	90.3718	0.0816361	0.288462	0.51965	0.899478	no
11.1675	11.8835	0.0896584	0.287737	0.51965	0.899478	no
24.1292	22.8607	-0.0779094	-0.288592	0.51955	0.899478	no
15.0432	16.0127	0.0901074	0.282824	0.5194	0.899478	no
1.67354	1.84464	0.140436	0.280648	0.51975	0.899517	no
26.2504	27.6601	0.0754682	0.283132	0.52	0.899547	no
50.0819	52.7739	0.0755351	0.283443	0.52	0.899547	no

174.584	184.428	0.0791361	0.289207	0.52	0.899547	no
10.3108	9.7601	-0.0791838	-0.289793	0.5204	0.899971	no
1.88698	1.72854	-0.126525	-0.283758	0.5205	0.90001	no
5.21842	4.76215	-0.132	-0.28346	0.5207	0.900222	no
2.10649	1.79286	-0.232578	-0.289216	0.5215	0.900399	no
3.28817	3.0608	-0.103375	-0.28837	0.52145	0.900399	no
5.99875	6.41476	0.0967336	0.283213	0.5212	0.900399	no
7.90984	7.33704	-0.108452	-0.289187	0.521	0.900399	no
7.63076	7.1885	-0.0861362	-0.2784	0.5213	0.900399	no
27.3546	25.9058	-0.0785126	-0.283096	0.52125	0.900399	no
9.56389	9.01005	-0.0860633	-0.284901	0.5214	0.900399	no
125.02	132.457	0.0833643	0.284915	0.521	0.900399	no
18.0393	19.3524	0.101366	0.282144	0.52145	0.900399	no
14.8512	15.8156	0.0907743	0.283677	0.52185	0.900861	no
3.96299	3.23166	-0.294311	-0.281364	0.52195	0.900861	no
35.179	37.1933	0.0803297	0.284704	0.522	0.900861	no
56.1505	59.2981	0.0786863	0.288892	0.5224	0.901284	no
40.6065	38.4941	-0.0770712	-0.281465	0.5224	0.901284	no
33.8245	35.6421	0.0755147	0.282081	0.52255	0.901408	no
6.22349	5.7972	-0.102369	-0.282784	0.52265	0.901447	no
2.07226	2.28917	0.143622	0.276548	0.523	0.901717	no
24.2343	22.9468	-0.0787589	-0.286766	0.5229	0.901717	no
2.18905	2.56992	0.231415	0.283956	0.52335	0.901717	no
4.3961	4.9015	0.157001	0.281362	0.52305	0.901717	no
20.2059	18.6501	-0.115594	-0.281377	0.5233	0.901717	no
84.0018	79.6996	-0.0758468	-0.27965	0.52325	0.901717	no
4.27108	3.96615	-0.106861	-0.283462	0.52325	0.901717	no
13.3929	14.9441	0.158112	0.282757	0.5239	0.901949	no
120.367	128.694	0.0965108	0.283335	0.52365	0.901949	no
2.36722	2.04056	-0.214234	-0.266948	0.5239	0.901949	no
15.926	16.9863	0.0929842	0.282467	0.52395	0.901949	no
7.17442	6.71737	-0.0949655	-0.282818	0.52375	0.901949	no
5.65273	6.17027	0.126386	0.280072	0.52385	0.901949	no
11.9754	11.2321	-0.0924471	-0.288693	0.5244	0.902141	no
74.6326	70.58	-0.080546	-0.284545	0.52415	0.902141	no
12.6703	11.693	-0.115799	-0.286444	0.52435	0.902141	no
41.9526	44.3419	0.0799113	0.280201	0.52445	0.902141	no
22.5713	21.2856	-0.0846115	-0.278871	0.52425	0.902141	no
2.43552	2.83307	0.218136	0.283839	0.5253	0.90243	no
2.2389	1.99112	-0.16921	-0.288079	0.52545	0.90243	no
33.0099	34.8855	0.079727	0.286511	0.5255	0.90243	no
6.76468	6.23954	-0.116582	-0.279759	0.5252	0.90243	no
2.35662	2.13922	-0.139632	-0.280007	0.5253	0.90243	no
3.82457	3.26387	-0.228717	-0.26796	0.5255	0.90243	no
331.18	314.068	-0.0765356	-0.285461	0.5253	0.90243	no
6.33097	5.90419	-0.100687	-0.27709	0.52505	0.90243	no
2.59305	2.33203	-0.153059	-0.279214	0.5252	0.90243	no

2.12762	1.95408	-0.12275	-0.28436	0.5249	0.90243	no
5.90148	5.51104	-0.0987511	-0.287035	0.52575	0.902592	no
9.83413	11.313	0.20211	0.284569	0.5258	0.902592	no
2.71072	3.00314	0.147797	0.272991	0.52605	0.902879	no
34.3624	36.2569	0.0774265	0.283648	0.5262	0.902879	no
88.8213	84.1778	-0.077465	-0.283349	0.52645	0.902908	no
37.7665	39.9377	0.0806434	0.278546	0.52635	0.902908	no
6.10781	6.57759	0.106904	0.2764	0.5264	0.902908	no
130.545	138.213	0.0823525	0.282706	0.52665	0.903117	no
4.48644	4.80837	0.0999747	0.28064	0.5268	0.903241	no
12.2832	13.0085	0.0827785	0.28259	0.5271	0.903575	no
15.3702	16.2054	0.0763405	0.280611	0.5273	0.903698	no
11.1946	10.3795	-0.109065	-0.279142	0.52745	0.903822	no
14.4391	13.5463	-0.092082	-0.275944	0.528	0.90445	no
31.0685	32.9836	0.086294	0.27684	0.52805	0.90445	no
7.54315	8.04849	0.0935499	0.278893	0.5284	0.904821	no
3.60469	4.06669	0.173982	0.27973	0.5285	0.904821	no
7.44107	7.01675	-0.0847069	-0.277107	0.52845	0.904821	no
2.14304	2.44529	0.190348	0.278717	0.52885	0.905286	no
4.36479	3.96994	-0.136796	-0.274624	0.529	0.90541	no
8.90834	9.44512	0.0844115	0.276271	0.52915	0.905533	no
12.067	12.7674	0.0814075	0.272716	0.53255	0.905766	no
12.883	12.1156	-0.0886015	-0.282202	0.53165	0.905766	no
4.55642	4.23211	-0.106525	-0.282552	0.53035	0.905766	no
7.84938	8.28041	0.0771249	0.27971	0.53075	0.905766	no
3.09174	2.85287	-0.116004	-0.278898	0.5315	0.905766	no
6.91848	7.77917	0.16916	0.27301	0.5318	0.905766	no
23.7902	25.1266	0.0788495	0.284031	0.53225	0.905766	no
36.2744	38.398	0.0820795	0.277473	0.53035	0.905766	no
2.75596	2.37298	-0.215857	-0.281004	0.53005	0.905766	no
96.7114	104.089	0.106064	0.278766	0.53185	0.905766	no
69.8821	66.3046	-0.0758146	-0.281332	0.53085	0.905766	no
43.2671	40.9149	-0.0806434	-0.278904	0.5315	0.905766	no
6.00255	5.42552	-0.145815	-0.270873	0.53205	0.905766	no
1394.18	1516.63	0.121453	0.270904	0.5327	0.905766	no
3.34616	3.06603	-0.126134	-0.281853	0.5315	0.905766	no
7.06285	6.63075	-0.0910778	-0.276697	0.53295	0.905766	no
35.3318	33.532	-0.0754305	-0.277788	0.53155	0.905766	no
23.6247	24.8909	0.0753212	0.278381	0.531	0.905766	no
3.09685	2.68458	-0.206102	-0.280805	0.53095	0.905766	no
28.6181	27.1822	-0.0742643	-0.277823	0.5322	0.905766	no
3.90729	3.44809	-0.18037	-0.282663	0.5329	0.905766	no
12.1884	11.402	-0.096221	-0.282373	0.5302	0.905766	no
11.1409	11.759	0.0779009	0.279125	0.5321	0.905766	no
8.2588	8.83443	0.0972051	0.276893	0.5328	0.905766	no
10.0591	10.8968	0.115409	0.280203	0.5329	0.905766	no
20.9437	22.9366	0.131135	0.273585	0.53095	0.905766	no

51.5451	48.8686	-0.0769281	-0.281045	0.53115	0.905766	no
2.12027	2.35488	0.151405	0.28134	0.53145	0.905766	no
35.0833	36.9594	0.0751577	0.281177	0.5316	0.905766	no
46.373	49.0483	0.0809183	0.276069	0.53235	0.905766	no
8.32759	9.15174	0.136147	0.274556	0.53005	0.905766	no
242.478	225.048	-0.107619	-0.277028	0.5296	0.905766	no
9.24942	9.87346	0.0941931	0.280983	0.53265	0.905766	no
21.2182	20.1194	-0.0767177	-0.274705	0.53205	0.905766	no
21.4107	22.6208	0.0793217	0.280599	0.52985	0.905766	no
2.17637	2.52011	0.211563	0.278548	0.5325	0.905766	no
10.1067	9.35074	-0.112155	-0.278101	0.53255	0.905766	no
3.64678	4.04692	0.150201	0.278787	0.53075	0.905766	no
3.94591	4.25228	0.107878	0.280567	0.53045	0.905766	no
12638	13637.6	0.109828	0.278673	0.5321	0.905766	no
17.004	15.9086	-0.0960699	-0.280478	0.53265	0.905766	no
66.3603	63.0226	-0.074451	-0.275215	0.5301	0.905766	no
2.5178	3.01305	0.259062	0.285224	0.5305	0.905766	no
3.59492	3.93385	0.129981	0.276912	0.52985	0.905766	no
15.0405	14.1338	-0.0897066	-0.281673	0.5331	0.905888	no
12.8327	13.8048	0.105351	0.281606	0.5333	0.906095	no
6.63182	6.13836	-0.111551	-0.278804	0.53355	0.906122	no
19.0654	20.1731	0.0814776	0.2785	0.53345	0.906123	no
4.46534	4.82777	0.112587	0.279095	0.5335	0.906123	no
163.204	173.969	0.0921526	0.278363	0.53385	0.906235	no
24.3437	26.0251	0.0963572	0.281946	0.5338	0.906235	no
1.64143	1.35791	-0.273561	-0.277406	0.5337	0.906235	no
6.93603	6.3804	-0.120463	-0.274018	0.53405	0.906442	no
30.4317	28.9125	-0.0738812	-0.273389	0.53505	0.907344	no
18.7422	17.6446	-0.0870626	-0.274829	0.53505	0.907344	no
44.0671	41.8483	-0.0745346	-0.275243	0.53485	0.907344	no
26.4738	25.1226	-0.0755796	-0.279879	0.535	0.907344	no
4.1761	3.76734	-0.14861	-0.279482	0.53475	0.907344	no
26.1703	27.6917	0.0815255	0.280559	0.53505	0.907344	no
2.80849	2.54239	-0.143606	-0.274239	0.53605	0.907693	no
1.89076	2.10565	0.155302	0.280686	0.53585	0.907693	no
2.9548	3.80184	0.363637	0.269392	0.536	0.907693	no
3.32259	3.72065	0.163248	0.277498	0.53635	0.907693	no
20.823	19.7885	-0.0735105	-0.273746	0.5355	0.907693	no
24.0983	22.7947	-0.080235	-0.270863	0.5362	0.907693	no
1.81414	1.40597	-0.367725	-0.25744	0.5363	0.907693	no
14.5919	13.8463	-0.0756676	-0.278177	0.5356	0.907693	no
2.1309	1.81848	-0.228734	-0.276699	0.5362	0.907693	no
13.0831	12.3769	-0.0800455	-0.27634	0.5361	0.907693	no
33.468	35.3174	0.0775967	0.271799	0.53585	0.907693	no
17.6782	18.6629	0.0781971	0.273744	0.5363	0.907693	no
2.00327	1.7646	-0.183017	-0.274786	0.5357	0.907693	no
2.98343	2.70787	-0.139814	-0.265887	0.53645	0.90773	no

8.32601	7.86739	-0.0817401	-0.273385	0.5368	0.90819	no
1.50411	1.74992	0.218381	0.278173	0.53835	0.908252	no
35.7543	33.8356	-0.0795773	-0.275087	0.53795	0.908252	no
30.2622	32.0888	0.0845518	0.274034	0.5371	0.908252	no
28.8562	27.1373	-0.0885994	-0.273483	0.53825	0.908252	no
55.3976	58.3088	0.0738893	0.274469	0.53765	0.908252	no
13.9533	13.2521	-0.0743903	-0.272971	0.5371	0.908252	no
19.1884	20.6271	0.104311	0.272515	0.53755	0.908252	no
10.8211	11.6205	0.102829	0.274738	0.53835	0.908252	no
1.65454	1.45438	-0.186021	-0.276398	0.53795	0.908252	no
125.973	118.974	-0.0824676	-0.276378	0.53795	0.908252	no
1.94596	2.20449	0.179968	0.271641	0.53805	0.908252	no
10.5477	11.1961	0.0860703	0.272079	0.5376	0.908252	no
4.53437	4.98349	0.136254	0.271395	0.53795	0.908252	no
14.4204	16.4971	0.194103	0.281739	0.5375	0.908252	no
14.5929	15.3729	0.0751193	0.273089	0.53695	0.908252	no
2.62065	2.22836	-0.23394	-0.276213	0.5376	0.908252	no
115.391	121.798	0.0779584	0.269148	0.53755	0.908252	no
18.3828	19.3835	0.0764795	0.274682	0.5384	0.908252	no
3.09071	2.86663	-0.108581	-0.272462	0.5381	0.908252	no
21.9567	23.1768	0.0780198	0.276305	0.53855	0.908373	no
9.56455	10.112	0.0802936	0.274344	0.53865	0.90841	no
1.56331	1.36237	-0.19849	-0.276811	0.53875	0.908447	no
198.076	208.254	0.0722877	0.270717	0.53915	0.908989	no
13.4209	14.3948	0.101066	0.274163	0.53935	0.909063	no
12.9752	12.2908	-0.0781695	-0.272185	0.53945	0.909099	no
46.7177	44.3931	-0.0736331	-0.268837	0.5396	0.90922	no
7.86109	8.40447	0.0964273	0.274994	0.5397	0.909257	no
24.6374	26.0041	0.07789	0.272231	0.53985	0.909378	no
25.1443	23.3702	-0.105562	-0.269199	0.53995	0.909414	no
4.17148	4.72322	0.179213	0.276759	0.54025	0.909524	no
5.62981	6.17567	0.133509	0.266076	0.54015	0.909524	no
10.9947	11.9423	0.119264	0.273261	0.54025	0.909524	no
2.18914	1.7239	-0.344688	-0.271819	0.5415	0.91006	no
46.2946	48.7164	0.0735635	0.271286	0.5408	0.91006	no
127.134	133.863	0.0744032	0.268968	0.54175	0.91006	no
17.5975	18.6448	0.0834003	0.266564	0.5409	0.91006	no
2.98602	2.6666	-0.163218	-0.274458	0.54135	0.91006	no
3.09903	2.81553	-0.138409	-0.26364	0.5418	0.91006	no
7.45395	8.10602	0.120989	0.273279	0.54165	0.91006	no
7.93027	8.45011	0.0916016	0.269471	0.54185	0.91006	no
11.2691	11.9877	0.0891894	0.269114	0.5418	0.91006	no
7.1674	6.52987	-0.134395	-0.266924	0.5412	0.91006	no
11.6754	12.3045	0.0757078	0.266095	0.5414	0.91006	no
4.21446	3.94583	-0.0950177	-0.272292	0.5416	0.91006	no
14.1539	14.9432	0.0782917	0.272951	0.54095	0.91006	no
8.57756	9.33969	0.122807	0.27157	0.5419	0.91006	no

18.1914	17.2533	-0.0763838	-0.272422	0.54105	0.91006	no
14.0085	13.066	-0.100494	-0.272274	0.5417	0.91006	no
10.4611	11.2459	0.104374	0.272443	0.54115	0.91006	no
6.66932	6.2349	-0.0971733	-0.268109	0.5423	0.910339	no
47.2198	49.778	0.0761148	0.266194	0.54285	0.910339	no
14.648	15.4785	0.0795695	0.267804	0.54275	0.910339	no
14.0359	13.3221	-0.0752945	-0.270235	0.5424	0.910339	no
11.5702	10.9533	-0.0790455	-0.272437	0.54265	0.910339	no
15.5522	16.5028	0.0855922	0.270764	0.5424	0.910339	no
55.2701	58.093	0.0718663	0.268066	0.54255	0.910339	no
2.3857	2.12682	-0.165716	-0.272719	0.5424	0.910339	no
1.74076	1.97984	0.185662	0.269858	0.54275	0.910339	no
44.8892	47.5977	0.084523	0.270406	0.54295	0.910376	no
12.3499	13.1387	0.0893265	0.270064	0.5432	0.910663	no
18.6906	19.8399	0.0860901	0.264181	0.54335	0.910783	no
13.9299	13.2254	-0.0748797	-0.268303	0.5435	0.910903	no
2.82715	3.30251	0.224213	0.273328	0.5437	0.911012	no
7.07931	6.66568	-0.0868556	-0.272389	0.54375	0.911012	no
1.67174	1.54007	-0.118354	-0.274707	0.5438	0.911012	no
1.3043	1.4793	0.181644	0.265226	0.54405	0.911037	no
26.5805	28.5525	0.103249	0.269025	0.5439	0.911037	no
10.7191	11.4012	0.088995	0.271656	0.544	0.911037	no
7.58506	8.04353	0.0846697	0.266916	0.54415	0.911073	no
12.0671	12.6867	0.0722375	0.268798	0.54495	0.911135	no
2.19	2.04739	-0.0971435	-0.271983	0.54445	0.911135	no
26.5155	27.8791	0.0723439	0.267184	0.54455	0.911135	no
8.63742	9.14909	0.083028	0.26864	0.545	0.911135	no
1.5575	0.988521	-0.655891	-0.296999	0.54505	0.911135	no
3.05126	3.3812	0.148133	0.270154	0.545	0.911135	no
11.745	12.4524	0.0843692	0.267887	0.54435	0.911135	no
77.0196	73.2649	-0.072103	-0.269148	0.5447	0.911135	no
52.7835	55.4909	0.0721647	0.270439	0.54445	0.911135	no
16.721	15.864	-0.0759003	-0.26845	0.5448	0.911135	no
28.264	30.2273	0.0968865	0.270524	0.54495	0.911135	no
5.75627	5.35387	-0.10455	-0.272045	0.5452	0.91116	no
16.2257	17.0608	0.0724019	0.267074	0.5453	0.91116	no
6.98318	7.60359	0.122797	0.272946	0.54525	0.91116	no
2.56123	1.90585	-0.426403	-0.278691	0.54545	0.911232	no
5.78299	5.32631	-0.118679	-0.269333	0.5455	0.911232	no
39.5276	41.7547	0.0790776	0.267781	0.54605	0.911889	no
9.06201	9.58968	0.0816506	0.26631	0.54605	0.911889	no
5.11811	5.48709	0.100431	0.267458	0.5464	0.912342	no
1.78945	2.05995	0.203095	0.269379	0.5466	0.912545	no
10.5842	10.0256	-0.0782258	-0.268134	0.5469	0.912557	no
5.21536	5.82952	0.16061	0.267355	0.547	0.912557	no
5.7146	6.28209	0.136592	0.270914	0.5468	0.912557	no
9.1868	8.53068	-0.106902	-0.264441	0.54685	0.912557	no

7.86124	8.77365	0.158421	0.259081	0.54695	0.912557	no
12.9954	11.995	-0.115571	-0.269373	0.5474	0.913093	no
21.3068	22.4452	0.0750953	0.273374	0.5478	0.913175	no
120.012	113.367	-0.0821775	-0.267788	0.5479	0.913175	no
11.963	13.318	0.154798	0.264461	0.54785	0.913175	no
18.7903	17.8698	-0.0724621	-0.263091	0.54895	0.913175	no
2.54952	2.23267	-0.19146	-0.258764	0.5491	0.913175	no
5.97618	6.41533	0.1023	0.268375	0.5487	0.913175	no
2.19408	1.98997	-0.140872	-0.266877	0.5485	0.913175	no
35.0356	36.9725	0.0776299	0.271355	0.54805	0.913175	no
3.32664	3.67085	0.142049	0.26472	0.54905	0.913175	no
2.53376	3.04901	0.267065	0.26132	0.54905	0.913175	no
1.85796	1.61795	-0.199552	-0.269771	0.5481	0.913175	no
17.7457	18.6587	0.0723813	0.271912	0.5483	0.913175	no
13.5471	14.3	0.0780386	0.26446	0.5484	0.913175	no
4.00957	4.32645	0.109735	0.267035	0.5491	0.913175	no
10.1047	10.9666	0.118097	0.266507	0.549	0.913175	no
10.467	11.4744	0.132571	0.265476	0.5485	0.913175	no
8.54095	7.78148	-0.134351	-0.264235	0.5477	0.913175	no
4.48888	4.20796	-0.0932352	-0.26368	0.54755	0.913175	no
4.29407	4.04839	-0.085	-0.265829	0.54885	0.913175	no
8.36554	7.91072	-0.0806496	-0.269809	0.54805	0.913175	no
9.02087	9.5692	0.0851325	0.263609	0.5492	0.913211	no
87.6152	82.7949	-0.0816392	-0.262856	0.5493	0.913247	no
54.7472	52.0306	-0.0734255	-0.269673	0.5494	0.913282	no
9.41358	8.88101	-0.0840189	-0.262854	0.5496	0.913353	no
16.1932	17.1765	0.0850532	0.268582	0.54955	0.913353	no
12.1279	11.4475	-0.0832961	-0.267722	0.5499	0.913531	no
27.8983	29.4006	0.0756691	0.265966	0.5501	0.913531	no
15.8088	16.6062	0.0709936	0.265474	0.5501	0.913531	no
18.4039	17.3886	-0.0818672	-0.263159	0.55	0.913531	no
7.18769	6.74735	-0.0912065	-0.262965	0.5499	0.913531	no
18.1505	19.1437	0.0768583	0.2692	0.5504	0.913708	no
19.5148	20.8231	0.0936116	0.262709	0.5505	0.913708	no
8.79032	8.10416	-0.117253	-0.265723	0.55055	0.913708	no
2.92556	3.20822	0.133063	0.266636	0.5503	0.913708	no
11.7805	10.897	-0.112467	-0.269279	0.5507	0.913744	no
3.06749	2.82696	-0.117806	-0.263011	0.55145	0.913944	no
128.32	137.87	0.103568	0.264055	0.5513	0.913944	no
6.31703	6.77569	0.10112	0.266751	0.55115	0.913944	no
6.081	6.55105	0.107417	0.26565	0.55105	0.913944	no
90.8477	95.9999	0.0795829	0.262937	0.5513	0.913944	no
6.94827	6.43539	-0.110626	-0.267098	0.55125	0.913944	no
8.31582	7.73057	-0.105284	-0.26565	0.55145	0.913944	no
10.6809	11.5785	0.116416	0.264188	0.551	0.913944	no
5.99285	5.66658	-0.0807643	-0.262467	0.55165	0.914145	no
11.1269	9.73614	-0.192635	-0.260766	0.55195	0.914464	no

18.3502	20.1699	0.13641	0.263441	0.5521	0.914499	no
3.5601	3.32111	-0.100256	-0.263225	0.5522	0.914535	no
30.6076	28.6877	-0.0934601	-0.263342	0.5527	0.91458	no
14.3926	13.6046	-0.0812275	-0.262573	0.5526	0.91458	no
7.88863	7.49507	-0.0738329	-0.263149	0.5527	0.91458	no
23.0523	21.9216	-0.072557	-0.265949	0.5524	0.91458	no
8.42862	9.06749	0.105407	0.261876	0.55235	0.91458	no
1.89765	1.65849	-0.194344	-0.2623	0.5526	0.91458	no
4.82409	4.57917	-0.0751704	-0.261422	0.5528	0.914616	no
15.8966	16.7756	0.0776518	0.264646	0.553	0.914686	no
3.22089	2.928	-0.137543	-0.256913	0.553	0.914686	no
4.25906	3.71109	-0.198692	-0.264874	0.5533	0.914732	no
226.999	244.034	0.104398	0.25919	0.55325	0.914732	no
17.5931	16.7411	-0.0716166	-0.265515	0.5535	0.914732	no
4.03965	3.77157	-0.099065	-0.262759	0.5535	0.914732	no
12.5158	13.2242	0.0794253	0.257058	0.5534	0.914732	no
54.1988	51.42	-0.0759323	-0.264104	0.554	0.915428	no
25.2019	26.7115	0.083927	0.262545	0.5542	0.915498	no
36.3136	34.5297	-0.0726727	-0.271466	0.55435	0.915615	no
32.2065	30.6683	-0.0706061	-0.264318	0.5545	0.915733	no
10.2602	9.67967	-0.0840238	-0.259999	0.55465	0.91585	no
1.53175	1.39736	-0.132475	-0.259675	0.5557	0.915867	no
16.2289	15.4499	-0.0709742	-0.257364	0.55745	0.915867	no
4.65073	5.06512	0.12314	0.262515	0.55665	0.915867	no
1.50289	1.65567	0.139673	0.264707	0.55615	0.915867	no
2.8248	3.12081	0.143774	0.25762	0.55635	0.915867	no
199.632	211.518	0.0834348	0.257954	0.55775	0.915867	no
16.0462	17.1203	0.0934749	0.264523	0.55525	0.915867	no
65.4377	62.2954	-0.0709979	-0.265462	0.55725	0.915867	no
5.85225	5.00607	-0.225313	-0.212765	0.5559	0.915867	no
2.81479	3.08459	0.132052	0.256894	0.55595	0.915867	no
54.0059	51.0254	-0.0819003	-0.261946	0.5564	0.915867	no
38.9011	40.8531	0.0706361	0.263219	0.5558	0.915867	no
21.9862	23.1436	0.0740184	0.256037	0.55545	0.915867	no
111.033	105.475	-0.0740835	-0.262902	0.55655	0.915867	no
22.0274	24.0875	0.128987	0.26081	0.55715	0.915867	no
11.1909	10.5202	-0.0891603	-0.25709	0.55805	0.915867	no
13.5899	14.2528	0.0687088	0.255125	0.5575	0.915867	no
1.71204	2.0523	0.261528	0.265048	0.55775	0.915867	no
17.6629	16.6482	-0.085352	-0.261573	0.5566	0.915867	no
5.25376	4.79143	-0.132895	-0.262467	0.5571	0.915867	no
9.98864	9.46571	-0.0775764	-0.258646	0.55795	0.915867	no
68.5856	71.9935	0.0699596	0.261289	0.55605	0.915867	no
112.943	118.444	0.0686153	0.256449	0.55675	0.915867	no
61.7301	65.6403	0.0886067	0.261457	0.55795	0.915867	no
7.38764	6.9873	-0.0803801	-0.261613	0.55675	0.915867	no
5.03608	5.77932	0.198598	0.265054	0.55705	0.915867	no

36.7303	34.9928	-0.0699103	-0.25858	0.5573	0.915867	no
11.2395	12.2428	0.123348	0.267648	0.55545	0.915867	no
8.00823	7.27825	-0.137892	-0.258316	0.55585	0.915867	no
22.8855	21.798	-0.0702406	-0.261109	0.55535	0.915867	no
3.35681	3.66998	0.128683	0.267706	0.5569	0.915867	no
25.1238	26.4889	0.0763326	0.258215	0.55725	0.915867	no
1.69063	1.85552	0.134261	0.257851	0.557	0.915867	no
63.7299	60.0971	-0.0846741	-0.264009	0.55705	0.915867	no
34.1888	32.494	-0.0733525	-0.259504	0.55625	0.915867	no
12.8333	12.151	-0.0788202	-0.260279	0.55495	0.915867	no
14.849	13.8326	-0.102288	-0.262256	0.55755	0.915867	no
2.52284	2.75559	0.127317	0.260574	0.5571	0.915867	no
3.74543	3.47532	-0.107985	-0.263209	0.55645	0.915867	no
6.05361	5.72163	-0.0813686	-0.254974	0.55685	0.915867	no
6.90649	7.37054	0.0938178	0.258462	0.5585	0.916476	no
6.28341	6.63811	0.0792254	0.262296	0.5586	0.916511	no
5.13539	4.86252	-0.0787713	-0.262809	0.559	0.916779	no
17.8962	18.78	0.0695419	0.257628	0.55895	0.916779	no
8.1587	8.68594	0.0903426	0.261377	0.55945	0.917374	no
27.7083	26.3841	-0.0706464	-0.261481	0.5596	0.917374	no
22.9021	24.0068	0.0679625	0.254713	0.5606	0.91759	no
3.90985	3.42925	-0.189218	-0.260196	0.5601	0.91759	no
1.16569	1.50557	0.369129	0.262072	0.55995	0.91759	no
55.5161	58.3541	0.0719283	0.257443	0.5602	0.91759	no
1.63545	1.9984	0.289157	0.260655	0.56005	0.91759	no
16.4104	15.3605	-0.0953847	-0.262552	0.5606	0.91759	no
14.0425	14.7787	0.0737187	0.258914	0.56015	0.91759	no
2545.27	2328.48	-0.128429	-0.255963	0.5606	0.91759	no
10.7362	11.4227	0.0894112	0.257101	0.56045	0.91759	no
16.9319	17.7855	0.0709607	0.26225	0.5604	0.91759	no
1.83545	1.66285	-0.142479	-0.254103	0.5606	0.91759	no
1.58676	1.37041	-0.211477	-0.224544	0.56075	0.917706	no
87.1001	92.0515	0.0797674	0.260737	0.56135	0.917994	no
20.4446	19.4835	-0.0694645	-0.258263	0.56125	0.917994	no
40.6516	42.7468	0.0725051	0.260071	0.5613	0.917994	no
12.9797	12.335	-0.0735071	-0.254821	0.5614	0.917994	no
35.1548	36.9024	0.0699965	0.262741	0.56115	0.917994	no
10.4749	9.86721	-0.0862176	-0.257566	0.56175	0.918065	no
32.4112	30.7791	-0.0745449	-0.254324	0.56235	0.918065	no
1.57911	1.35957	-0.215966	-0.257043	0.56225	0.918065	no
4.60037	5.09803	0.14819	0.26126	0.5625	0.918065	no
31.963	34.1109	0.0938316	0.259633	0.562	0.918065	no
12.3319	12.9327	0.0686226	0.255811	0.5625	0.918065	no
22.943	24.1059	0.0713282	0.255297	0.56255	0.918065	no
22.8301	21.6507	-0.0765219	-0.254486	0.5619	0.918065	no
1.75152	1.36179	-0.363107	-0.261557	0.56245	0.918065	no
393.933	417.248	0.0829538	0.257771	0.56185	0.918065	no

18.5959	17.1176	-0.11951	-0.258614	0.5624	0.918065	no
33.9754	32.3647	-0.0700677	-0.262242	0.56205	0.918065	no
14.7899	15.5723	0.0743728	0.256853	0.56205	0.918065	no
2.55793	2.79355	0.127122	0.258827	0.5633	0.918184	no
2.74171	3.02336	0.141077	0.257849	0.56295	0.918184	no
3.23805	3.46352	0.0971177	0.259522	0.56325	0.918184	no
135.407	128.282	-0.0779839	-0.258339	0.5636	0.918184	no
4.61424	4.35194	-0.0844341	-0.254736	0.56355	0.918184	no
4.54741	4.87328	0.0998482	0.258176	0.56365	0.918184	no
11.6954	11.0208	-0.085714	-0.254764	0.5635	0.918184	no
95.0672	99.7462	0.0693143	0.25896	0.56365	0.918184	no
1.86827	1.58383	-0.238281	-0.257913	0.56365	0.918184	no
4.05141	4.41463	0.123869	0.255637	0.5636	0.918184	no
3.26081	2.98906	-0.125536	-0.253672	0.5628	0.918184	no
18.5698	17.0931	-0.119542	-0.255838	0.5629	0.918184	no
5.40367	5.00559	-0.110398	-0.255817	0.5628	0.918184	no
15.362	16.2576	0.0817495	0.253441	0.5641	0.918226	no
52.0657	49.6461	-0.0686528	-0.253281	0.5638	0.918226	no
11.0452	9.98133	-0.146122	-0.25307	0.56405	0.918226	no
11.3539	12.0437	0.0851004	0.256738	0.56415	0.918226	no
41.5826	45.0821	0.116574	0.257555	0.56405	0.918226	no
5.77546	6.47769	0.165545	0.257531	0.56415	0.918226	no
35.0528	33.4107	-0.0692225	-0.255408	0.5645	0.918234	no
8.53166	8.98498	0.0746899	0.257313	0.5645	0.918234	no
6.74299	6.23167	-0.113769	-0.25467	0.56425	0.918234	no
3.40624	3.10671	-0.132792	-0.254342	0.5645	0.918234	no
7.95528	8.40303	0.0789974	0.258603	0.56465	0.918268	no
12.9201	13.8421	0.0994398	0.258112	0.56685	0.918408	no
11.3415	11.911	0.0706757	0.255755	0.56605	0.918408	no
22.2939	21.1555	-0.0756149	-0.25563	0.56685	0.918408	no
4.19506	3.87793	-0.113407	-0.257857	0.56675	0.918408	no
8.27055	8.75808	0.0826316	0.250809	0.566	0.918408	no
1.37037	1.59475	0.218764	0.253704	0.56605	0.918408	no
7.37881	7.98884	0.114598	0.256447	0.56665	0.918408	no
4.10567	6.23777	0.603411	0.0884633	0.5664	0.918408	no
4.04541	3.80039	-0.0901414	-0.256703	0.5669	0.918408	no
5.91949	7.06975	0.256186	0.246485	0.56525	0.918408	no
262.954	249.3	-0.0769261	-0.251019	0.56565	0.918408	no
11.9378	12.5217	0.0688892	0.253806	0.5657	0.918408	no
12.4399	13.1927	0.0847679	0.259089	0.56595	0.918408	no
9.67389	9.1582	-0.0790325	-0.250331	0.5653	0.918408	no
2.19106	2.01291	-0.12235	-0.255724	0.5661	0.918408	no
8.60758	9.12981	0.0849771	0.254494	0.56695	0.918408	no
5.71206	6.10771	0.0966205	0.257343	0.5668	0.918408	no
9.1071	11.7164	0.363459	0.244059	0.56495	0.918408	no
5.93724	5.53062	-0.102353	-0.26028	0.5653	0.918408	no
28.8079	30.2136	0.0687309	0.257312	0.56685	0.918408	no

24.527	25.9803	0.0830468	0.256166	0.56645	0.918408	no
22.0009	20.9672	-0.0694307	-0.250895	0.5655	0.918408	no
5.14356	4.72271	-0.123151	-0.256158	0.56695	0.918408	no
26.7052	25.4174	-0.0713041	-0.258451	0.56565	0.918408	no
3.10851	3.49709	0.16993	0.252145	0.5669	0.918408	no
10.594	11.1158	0.0693676	0.253415	0.56505	0.918408	no
2.7931	2.56307	-0.123992	-0.251984	0.56705	0.918442	no
471.468	512.527	0.120467	0.257217	0.5673	0.918497	no
12.4101	11.5626	-0.102048	-0.254116	0.5674	0.918497	no
3.65904	3.20852	-0.189555	-0.257331	0.56725	0.918497	no
40.2683	42.2348	0.0687896	0.256641	0.56735	0.918497	no
3.05775	2.84917	-0.101931	-0.254264	0.5677	0.918855	no
27.4705	28.8786	0.0721211	0.253727	0.5679	0.919003	no
9.17375	8.15438	-0.169935	-0.255384	0.56795	0.919003	no
3.66321	4.03328	0.138845	0.246799	0.56825	0.919361	no
16.9963	17.8164	0.0679843	0.255526	0.5684	0.919475	no
28.9067	27.5902	-0.0672485	-0.252004	0.5685	0.919509	no
19.0626	20.2757	0.0890134	0.257544	0.5686	0.919543	no
6.66255	6.26332	-0.0891479	-0.251985	0.56885	0.919644	no
38.7165	41.6841	0.106551	0.252909	0.56875	0.919644	no
7.77453	7.26965	-0.0968701	-0.250895	0.5689	0.919644	no
7.80223	8.31649	0.0920877	0.255392	0.56935	0.920243	no
1.88613	1.73257	-0.122513	-0.247329	0.56945	0.920277	no
20.4019	19.482	-0.0665617	-0.249774	0.57	0.920478	no
5.50869	6.01005	0.125667	0.248104	0.56985	0.920478	no
10.148	10.7116	0.0779834	0.253134	0.5698	0.920478	no
7.82128	7.34813	-0.0900278	-0.250946	0.5699	0.920478	no
14.2387	13.4983	-0.0770438	-0.253552	0.56985	0.920478	no
16.5487	15.4798	-0.0963302	-0.250915	0.57025	0.920673	no
2.70002	2.51	-0.105282	-0.25195	0.57045	0.920821	no
39.8283	41.8597	0.0717692	0.251261	0.5705	0.920821	no
7.52839	6.94121	-0.117155	-0.255541	0.5706	0.920854	no
10.6142	11.1425	0.0700679	0.252119	0.5711	0.92088	no
7.92772	7.4667	-0.0864354	-0.24758	0.571	0.92088	no
15.4158	16.3647	0.0861785	0.245344	0.57125	0.92088	no
59.7795	62.7624	0.0702493	0.25168	0.5712	0.92088	no
26.6388	27.9668	0.0701842	0.250439	0.57125	0.92088	no
7.19677	7.57635	0.0741529	0.252997	0.57115	0.92088	no
10.6086	11.3728	0.100348	0.251549	0.5707	0.92088	no
9.98698	10.5504	0.0791796	0.258402	0.5712	0.92088	no
21.1513	20.1967	-0.0666292	-0.250342	0.57155	0.921067	no
1.96334	2.18339	0.153256	0.250522	0.57185	0.921067	no
6.33213	6.70069	0.0816193	0.250655	0.572	0.921067	no
2.97464	2.74617	-0.115296	-0.250458	0.57195	0.921067	no
2.66442	2.41059	-0.144434	-0.24559	0.5719	0.921067	no
18.9852	20.0094	0.0757982	0.249699	0.57195	0.921067	no
2.74567	2.51992	-0.123778	-0.257849	0.5716	0.921067	no

11.2148	11.8961	0.0850849	0.252857	0.5722	0.921261	no
27.8255	26.2856	-0.0821332	-0.252788	0.5725	0.921489	no
22.4453	21.4306	-0.0667388	-0.250017	0.5725	0.921489	no
8.14136	7.72296	-0.076116	-0.246916	0.5726	0.921522	no
9.54985	8.39725	-0.185562	-0.253573	0.57275	0.921636	no
25.0537	23.7505	-0.0770656	-0.246017	0.57315	0.922057	no
6.74028	7.24008	0.103198	0.251395	0.57325	0.922057	no
6.03917	6.64764	0.138491	0.255901	0.57325	0.922057	no
20.661	19.7161	-0.0675372	-0.252652	0.5735	0.922076	no
282.332	303.061	0.102216	0.25027	0.5734	0.922076	no
4.17121	3.72107	-0.16475	-0.253304	0.57345	0.922076	no
63.5302	67.4076	0.0854689	0.249119	0.57365	0.92219	no
27.2592	28.5465	0.0665688	0.248022	0.57385	0.922384	no
571.442	544.77	-0.0689618	-0.250538	0.574	0.922497	no
9.47717	8.91835	-0.0876793	-0.249287	0.57435	0.922837	no
9.04052	8.61996	-0.0687253	-0.249318	0.57445	0.922837	no
1.90361	1.72398	-0.142999	-0.251239	0.5744	0.922837	no
2.67622	2.11894	-0.336855	-0.254905	0.57515	0.923068	no
3.70095	3.38775	-0.12757	-0.249385	0.57505	0.923068	no
2.86866	2.66835	-0.104428	-0.24717	0.57515	0.923068	no
10.4814	11.0525	0.0765487	0.244092	0.57515	0.923068	no
7.61452	7.07749	-0.105516	-0.248834	0.57485	0.923068	no
60.9385	63.8597	0.0675534	0.252004	0.5748	0.923068	no
7.29121	7.73111	0.0845168	0.246628	0.5751	0.923068	no
83.2744	87.3916	0.0696224	0.250411	0.576	0.923176	no
2.64787	2.91397	0.138153	0.249714	0.57545	0.923176	no
7.12154	6.54999	-0.120696	-0.250558	0.57625	0.923176	no
16.9306	17.742	0.0675419	0.248945	0.57555	0.923176	no
28.2919	29.6542	0.0678438	0.252035	0.5759	0.923176	no
9.01522	7.91386	-0.187981	-0.25283	0.5755	0.923176	no
19.6487	20.6545	0.0720183	0.250066	0.57585	0.923176	no
127.14	133.23	0.0674953	0.244274	0.5762	0.923176	no
4.66517	5.16679	0.147339	0.249303	0.5762	0.923176	no
1.77881	1.47965	-0.265659	-0.249327	0.5761	0.923176	no
19.2867	18.3807	-0.0694138	-0.247514	0.57615	0.923176	no
45.8133	47.964	0.0661866	0.248162	0.5766	0.923609	no
2.09565	1.90011	-0.141319	-0.244086	0.57685	0.923835	no
4.8711	4.39796	-0.147415	-0.251719	0.5769	0.923835	no
9.63576	10.253	0.0895774	0.250068	0.5776	0.924446	no
3.67498	3.93207	0.0975542	0.25208	0.57745	0.924446	no
2.67162	2.91127	0.12393	0.243053	0.5775	0.924446	no
70.7499	74.0882	0.066517	0.247132	0.57755	0.924446	no
1.75828	1.90874	0.118454	0.245442	0.5778	0.924512	no
160.141	169.108	0.078603	0.243079	0.5778	0.924512	no
2.43376	2.68506	0.141769	0.247852	0.5781	0.924595	no
4.7494	4.35052	-0.126559	-0.240394	0.57805	0.924595	no
7.84402	7.24355	-0.114896	-0.246003	0.5782	0.924595	no

8.65481	9.47434	0.130523	0.249379	0.57825	0.924595	no
1.48225	1.32114	-0.165999	-0.244886	0.5818	0.924894	no
6.16848	6.78512	0.13746	0.245477	0.58145	0.924894	no
1.22983	1.48379	0.27083	0.247335	0.5788	0.924894	no
4.84873	4.50943	-0.104661	-0.246681	0.5807	0.924894	no
16.7507	17.5591	0.0679973	0.245462	0.5801	0.924894	no
2.78203	2.51401	-0.146149	-0.24742	0.5813	0.924894	no
2.04122	2.24206	0.135396	0.245574	0.5814	0.924894	no
36.2421	33.4216	-0.116887	-0.240471	0.5811	0.924894	no
14.0608	14.7405	0.0681108	0.243059	0.5837	0.924894	no
12.1965	11.6322	-0.0683459	-0.244891	0.58005	0.924894	no
31.6396	30.1282	-0.0706177	-0.243575	0.58215	0.924894	no
95.9825	100.415	0.0651264	0.243566	0.5817	0.924894	no
2.77398	2.48982	-0.155916	-0.243637	0.5806	0.924894	no
87.2512	91.6078	0.0702963	0.247044	0.57895	0.924894	no
3.60487	3.36061	-0.101227	-0.246374	0.5815	0.924894	no
2.34657	2.55108	0.120552	0.243098	0.57855	0.924894	no
25.08	23.8639	-0.0717067	-0.242433	0.5797	0.924894	no
6.27716	5.58829	-0.167705	-0.241523	0.5833	0.924894	no
2.85858	2.59676	-0.138583	-0.24286	0.5835	0.924894	no
13.8989	13.2031	-0.0740886	-0.242797	0.58265	0.924894	no
24.8965	26.783	0.105379	0.246554	0.5823	0.924894	no
11.4748	12.088	0.0751055	0.247315	0.5819	0.924894	no
11.5533	12.1597	0.0737971	0.247967	0.58295	0.924894	no
15.5409	14.8166	-0.0688502	-0.241811	0.5809	0.924894	no
13.5733	12.8329	-0.0809256	-0.248972	0.5821	0.924894	no
6.46848	7.14924	0.144364	0.244494	0.58355	0.924894	no
5.1252	5.45014	0.0886844	0.246437	0.5807	0.924894	no
34.0393	32.4369	-0.0695653	-0.24716	0.5826	0.924894	no
4.15081	3.81148	-0.12304	-0.239291	0.5813	0.924894	no
64.9235	61.9651	-0.067285	-0.23905	0.58385	0.924894	no
4.22546	3.90732	-0.11293	-0.245447	0.58205	0.924894	no
54.5851	57.4821	0.0746037	0.244249	0.58375	0.924894	no
20.3003	22.992	0.179628	0.241953	0.5822	0.924894	no
2.20164	1.96592	-0.163375	-0.249933	0.57955	0.924894	no
6.58934	6.94003	0.0748074	0.245274	0.5799	0.924894	no
7.70146	8.23325	0.0963304	0.2425	0.58285	0.924894	no
5.54356	5.93959	0.0995518	0.244988	0.5825	0.924894	no
14.1369	14.9955	0.0850702	0.249536	0.5799	0.924894	no
14.996	14.1555	-0.0832104	-0.245688	0.5808	0.924894	no
1.75185	1.95517	0.158415	0.250027	0.58325	0.924894	no
5.86228	5.53662	-0.0824556	-0.24799	0.57895	0.924894	no
55.2064	57.9718	0.0705146	0.243258	0.58385	0.924894	no
562.827	591.703	0.0721812	0.247125	0.57955	0.924894	no
2.6183	2.31978	-0.174645	-0.244959	0.5821	0.924894	no
6.76766	7.43839	0.136335	0.242996	0.5812	0.924894	no
6.03585	5.6527	-0.0946176	-0.248687	0.5792	0.924894	no

7.26735	6.91193	-0.0723408	-0.250061	0.5812	0.924894	no
13.7891	13.1136	-0.0724603	-0.249141	0.5814	0.924894	no
11.0069	11.6449	0.0812922	0.247893	0.58295	0.924894	no
6.16079	6.51211	0.0800084	0.245086	0.58085	0.924894	no
17.6682	18.5192	0.0678709	0.247737	0.5787	0.924894	no
10.0111	9.53085	-0.0709283	-0.239963	0.5834	0.924894	no
9.80435	9.2296	-0.0871549	-0.24254	0.58225	0.924894	no
35.5428	33.415	-0.0890606	-0.248896	0.58055	0.924894	no
[truncated: 710,265 more chars]
